# The lichens of the Alps – an annotated checklist

**DOI:** 10.3897/mycokeys.31.23568

**Published:** 2018-03-12

**Authors:** Pier Luigi Nimis, Josef Hafellner, Claude Roux, Philippe Clerc, Helmut Mayrhofer, Stefano Martellos, Peter O. Bilovitz

**Affiliations:** 1 Department of Life Sciences, University of Trieste, Via L. Giorgieri 10, 34127 Trieste, Italy; 2 Institute of Plant Sciences, NAWI Graz, University of Graz, Holteigasse 6, 8010 Graz, Austria; 3 Chemin des Vignes-Vieilles, 84120 Mirabeau, France; 4 Conservatoire et Jardin botaniques de la Ville de Genève, 1 chemin de l’Impératrice, 1292 Chambésy/GE, Switzerland

**Keywords:** Europe, biodiversity, lichenised fungi, fungi, taxonomy

## Abstract

This is the first attempt to provide an overview of the lichen diversity of the Alps, one of the biogegraphically most important and emblematic mountain systems worldwide. The checklist includes all lichenised species, plus a set of non- or doubtfully lichenised taxa frequently treated by lichenologists, excluding non-lichenised lichenicolous fungi. Largely based on recent national or regional checklists, it provides a list of all infrageneric taxa (with synonyms) hitherto reported from the Alps, with data on their distribution in eight countries (Austria, France, Germany, Liechtenstein, Monaco, Italy, Slovenia, Switzerland) and in 42 Operational Geographic Units, mostly corresponding to administrative subdivisions within the countries. Data on the main substrates and on the altitudinal distribution are also provided. A short note points to the main ecological requirements of each taxon and/or to open taxonomic problems. Particularly poorly known taxa are flagged and often provided with a short description, to attract the attention of specialists. The total number of infrageneric taxa is 3,163, including 117 non- or doubtfully lichenised taxa. The richness of the lichen biota fairly well corresponds with the percent of the Alpine area occupied by each country: Austria (2,337 taxa), Italy (2,169), France (2,028), Switzerland (1,835), Germany (1,168), Slovenia (890) and Lichtenstein (152), no lichen having ever been reported from Monaco. The number of poorly known taxa is quite high (604, 19.1% of the total), which indicates that, in spite of the Alps being one of the lichenologically most studied mountain systems worldwide, much work is still needed to reach a satisfactory picture of their real lichen diversity. Thirteen new combinations are proposed in the genera *Agonimia*, *Aspicilia*, *Bagliettoa*, *Bellemerea*, *Carbonea*, *Lepra*, *Miriquidica*, *Polysporina*, *Protothelenella*, *Pseudosagedia* and *Thelidium*.

## Introduction

In the history of biogeography, the Alps play a most important role: they are one of the largest continuous natural areas in Europe and probably the most studied mountain system worldwide, to the point that terms such as “alpine” and “subalpine” are widely used for any mountain system in the world.

Situated between the Eurosiberian and the Mediterranean biogeographic regions, the Alps are an interzonal mountain system distributed amongst eight countries over an area of ca. 170,000 km^2^, with a length of ca. 1,200 km and a maximum width of 300 km; they start at sea level and peak at 4,807 m (Mt. Blanc). The Alps are present in eight countries: Austria (28.7% of the overall area of the Alps), Italy (27.2%), France (21.4%), Switzerland (13.2%), Germany (5.8%), Slovenia (3.6%), Liechtenstein (0.08%) and Monaco (0.01%) with a total population of ca. 11.1 million people. The Alps, which include fourteen national parks and many regional protected areas, shelter a large number of natural and semi-natural habitats, with a rich diversity of organisms and landscapes. They are one of the richest biodiversity hotspots in Europe, hosting e.g. 4,450 vascular plant taxa with a density of 2,200 taxa per 10,000 km² (Aeschimann et al. 2011a), the most species-rich areas being in the West and the South, the richest in endemics corresponding to areas that were glacier-free during the Pleistocene, such as the southern part of the Western Alps and the Eastern Alps (Tribsch 2004, Aeschimann et al. 2011b).

The Alps are also the mountain system which was explored with more continuity by botanists, zoologists and mycologists, including lichenologists. It is not easy for present readers to imagine the problems facing the first scholars in studying the lichens of the Alps: neither highways nor rapid trains existed in the Alpine region and any ascension to the Alpine belt had to be made with days of travel through dusty or muddy roads and by hard climbing through paths built by shepherds, with uncomfortable overnight stays in primitive shelters with limited food, finally carrying down the heavy collections to the next village. In spite of these difficulties, the Alps have been intensively studied since the earliest years by important lichenologists such as, to mention only a few, M. Anzi (1812–1881), F. Arnold (1828–1901), F. Baglietto (1826–1916), S. Garovaglio (1805–1882), Ph. Hepp (1797–1867), A. M. Hue (1840–1917), E. Kernstock (1852–1900), A. von Krempelhuber (1813–1882), A. Massalongo (1824–1860), W. Nylander (1822–1899), J. Müller Argoviensis (1828–1896), A. E. Sauter (1800–1881), L. E. Schaerer (1785–1853), G. A. Scopoli (1723–1788), E. Stizenberger (1827–1895) and F. X. von Wulfen (1728–1805). In the second half of the 19^th^ Century, the first attempts of national-regional checklists appeared, such as those of Krempelhuber (1861) for Bavaria, Stizenberger (1882, 1883) for Switzerland and Jatta (1900) for Italy. In the 20^th^ Century, the lichenological exploration of the Alps continued and intensified to the present times, especially from the post World War II period, when important Masters such as Georges Clauzade (1914–2002), Eduard Frey (1888–1974) and Josef Poelt (1924–1995) contributed to a revival of lichenological studies in the Alps by training a new generation of lichenologists, including most authors of the present checklist.

Thus, the Alps are, beyond doubt, one of the lichenologically best investigated parts of the world. Surprisingly, however, no general overview of their lichen diversity was ever attempted, all of the existing checklists having being compiled at the national or regional levels, a situation which also applies to most of the other taxonomic groups, including animals and to most transnational orobiomes worldwide, with the notable exception for lichens of the Carpathian mountains (Bielczyk et al. 2004, Lisická 2005). This lack of a general overview hampered the possibility of comparing the biogeographic traits of such an emblematic area as the Alps with those of other mountains systems worldwide, including not only other European orobiomes (Carpathians, Pyrenees, Scandinavian Mts., Caucasus) but also extra-European ones (Himalayas, Rocky Mountains, tropical high mountains of Africa, New Zealand Alps etc.), to elucidate various patterns of disjunctions and overall distribution, both on the taxonomic level (species, genera) and on that of entire biota. This fact is particularly annoying in the case of lichens, which include many broad-ranging species and relatively few endemics, so that many taxa described from the Alps have been later detected in other parts of the world.

Work for the present checklist started almost 15 years ago, upon a suggestion by P.L. Nimis. The idea was to rapidly produce a catalogue of lichens known from the Alps, by electronically merging the information contained in the checklists of Germany (Grummann 1963, Scholz 2000), Italy (Nimis 1993), Austria (Türk and Poelt 1993, Hafellner and Türk 2001), Slovenia (Suppan et al. 2000) and Switzerland (Clerc 2004, at that time in preparation), plus those included in the still unpublished catalogue of the lichens of France by C. Roux and collaborators. A first general list was produced in 2005, but its completion proved to be much less easy than foreseen, mainly because of the many open taxonomic problems and the necessity for continuous updates due to intense lichenological exploration in most countries. The progress of lichenological activity in several “Alpine” countries was such, that in the last few years, new, updated checklists were published for Switzerland (Clerc and Truong 2012), France (Roux et al. 2014, 2017), Italy (Nimis and Martellos 2003, Nimis 2016), Germany (Wirth et al. 2013), and Austria (Hafellner and Türk 2016).

The present checklist tries to summarise all of this information, providing, for the first time, a complete annotated catalogue of all lichenised fungi hitherto reported from the Alps.

### Delimitation of the Alps

In planning a checklist of the Alps, the authors had to face the question of delimiting the corresponding geographic area. As there is no unique delimitation of the Alps, one was adopted approaching the boundaries proposed by Marazzi (2005), within which Aeschimann et al. (2004) only retained the Alpine phytogeographic unit. However, some differences are identified: 1) contrary to Aeschimann et al. (2004), in the Western Alps, these limits extend to sea level, also encompassing areas with an eu-Mediterranean vegetation and the coastal rocks along the Mediterranean Sea. 2) Monaco is included; however, to the authors’ knowledge, there is no lichen record from this small country (ca. 2 km^2^), which is practically devoid of natural areas. 3) Contrary to Marazzi (2005), the Mt. Salève range was included as for example Führer (1979) did, when he drew the border of the Alps along the Rhine and therefore regarded it as part of the Bornes Alpes. Despite geological similarities with the Jura, Mt. Salève is much closer to the Alps and lichens described from there may well occur in the adjacent, equally mainly calcareous Massif de Bornes. 4) The area of Trnovsky gozd in Slovenia has also been included, this being sometimes considered as part of the Alps (e.g. by Bätzing 2001, 2015), sometimes of the Dinarides (e.g. by Marazzi 2005).

Alpine and pre-Alpine Slovenia were delimited according to the phytogeographic units proposed by Wraber (1969) and Zupančič et al. (1987), because of the lack of suitable administrative subdivisions in the young country when the checklist was starting to be prepared. With the exception of Slovenia, the further subdivision of the Alpine area into Operational Geographic Units corresponds with those of the main administrative units (Bundesländer in Austria, Départements in France, Regierungsbezirke in Germany/Bavaria, Regioni in Italy, Cantons in Switzerland), as this is the way the records are organised in the national lichen checklists.

In several cases, the adopted delimitation of the Alps does not correspond with the limits of the administrative subdivisions; typical is the case of Liguria (Italy), where only a very minor part of the regional territory falls within the Alpine area. In such cases, the authors have tried, as far as possible, to eliminate from the regional lists all species which occur in these regions, but have no record from the Alpine area proper.

**Figure 1.**
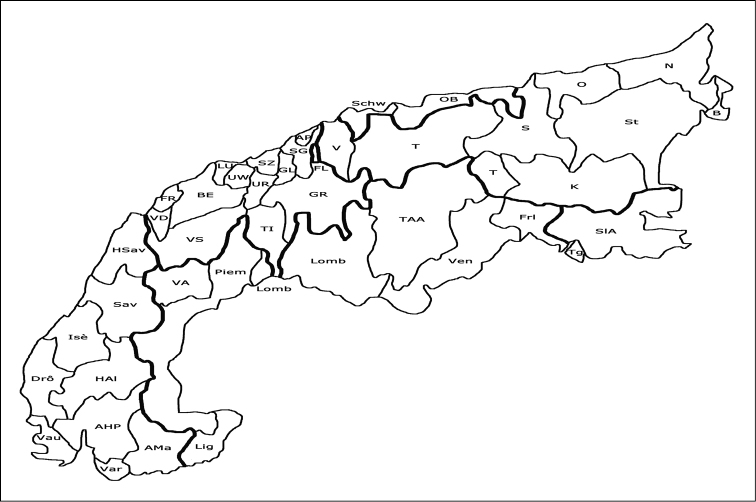
Delimitation of the Alps, with the administrative subdivisions (for abbreviations, see below).

### Structure of the checklist

The list is mainly based on records published in the recent checklists of Slovenia (Suppan et al. 2000, integrated by Mayrhofer 2006), Switzerland (Clerc and Truong 2012), Germany (Grummann 1963, Scholz 2000, Wirth et al. 2013), France (Roux et al. 2014, 2017), Italy (Nimis 2016) and Austria (Hafellner and Türk 2016). The authors refer to these works for a more extensive list of references and/or further details on the data sources. The data concerning Liechtenstein derive from a still unpublished work by Hafellner and Boom (in prep.). In a few cases, floristic and taxonomic treatments published after the national checklists were also taken into consideration. Several non-lichenised species which were and are traditionally treated by lichenologists are included, but non-lichenised lichenicolous fungi are excluded. Particularly dubious records are listed at the end.

### Nomenclature and synonyms

The authors have tried to update nomenclature to the latest standards. However, the authors have preferred to maintain some old, well-established genera such as *Caloplaca* *s.lat.* and *Aspicilia* *s.lat.*, since too many species from the Alps still await a re-assignment to the new genera in which they were split, mainly on the basis of molecular data. Generic concepts of cetrariod macrolichens are the object of a long ongoing controversy amongst different working groups: a recent phylogenetic reconstruction based on sequence data resulted in the recognition of a few genera only, which include morphologically and chemically fairly different groups (e.g. inclusion of *Allocetraria*, *Cetrariella*, *Usnocetraria* and *Vulpicida* in *Cetraria*; inclusion of *Flavocetraria*, *Tuckermannopsis*, *Tuckneraria* and further genera in *Nephromopsis*). As other working groups are expected to have different views, for the time being, the traditionally more or less well-established genera are maintained. The authors’ treatment of morphologically and chemically heterogeneous groups also needs an explanation. For example, the *Xanthoparmelia pulla*-group, the *Lecidea atrobrunnea*-group, the *Sarcogyne regularis*-group and the *Thamnolia vermicularis*-group include morphotypes and chemical strains, which in the past have been treated partly as species, partly as infraspecific taxa. Their taxonomic value is still not well understood. For practical reasons, infraspecific ranks are applied here, so that closely related taxa and strains can be listed together, but the authors are aware of the subjectivity of this decision.

Apart from the basionyms, well-established synonyms used in publications about lichens in the Alps are included, but due to space limitations, an index of all cited names is not included. Such a thesaurus will be included in a forthcoming online version of the checklist.

### Lichenised and non-lichenised species

**L** Lichenised species.

**F** Non- or doubtfully lichenised species usually reported by lichenologists.

### Poorly known taxa

# – This checklist includes quite a high number of very poorly understood taxa, often only known from the type material. The authors have decided to retain most of them, for the following reasons: 1) They could constitute good taxa, as is happening for some of the many species of Verrucariaceae described by M. Servít, 2) They could prove to be the correct name for other taxa described later, 3) In some cases their omission was mainly due to the unavailability of the type material, which was recently discovered and awaits further study (e.g. for some of the taxa described by M. Anzi, see Nimis 2016).

### Substrates (Subs.)

The main types of substrates are abbreviated as follows:

**sil** siliceous rocks and corresponding man-made substrata (e.g. roofing tiles),

**cal** calciferous rocks and corresponding man-made substrata (e.g. concrete, cement, asbestos etc.),

**int** intermediate rocks (such as calciferous schists),

**met** metal-rich siliceous rocks,

**sax** rocks (without more detailed information),

**ter-cal** calciferous soil,

**ter-sil** acidic soil (mostly on siliceous substrata),

**bry** living mosses,

**deb** plant debris,

**cor** bark,

**xyl** lignum,

**fol** living leaves,

**res** resin,

**alg** living algal colonies,

**par** parasitic on other lichens,

**aqu** temporary or permanently submerged.

### Bioclimatic/Altitudinal distribution (Alt.)

1 Mesomediterranean belt (potential vegetation: evergreen broad-leaved forests dominated by *Quercus ilex*),

2 Submediterranean/colline belt (potential vegetation: mixed deciduous forests dominated by *Quercus* and *Carpinus*),

3 Montane belt (potential vegetation: deciduous forests dominated by *Fagus sylvatica* and closed coniferous forests with *Picea abies*),

4 Subalpine belt (potential vegetation: open, taiga-like forests dominated by *Larix decidua* and/or *Pinus cembra* and *Rhododendron*),

5 Alpine (potential vegetation: treeless Alpine grasslands and tundras, to the lower limit of perennial snow and the equilibrium line of glaciers),

6 Nival (as before, above the lower limit of perennial snow and glaciers).

### Regional distribution

For each infrageneric taxon, the authors report the presence in the 7 Alpine countries and in 42 Operational Geographic Units, corresponding to their main subdivisions. Particularly dubious records are flagged with “?”. In the very few cases of records from a country without specification of locality, the abbreviation of that country has been repeated.

*Austria* **(Au)**: **V** – Vorarlberg, **T** – Tirol, **S** – Salzburg, **K** – Kärnten, **St** – Steiermark, **O** – Oberösterreich, **N** – Niederösterreich (incl. Wien), **B** – Burgenland.

*Germany* **(Ge)**: **OB** – Oberbayern, **Schw** – Schwaben.

*Switzerland* **(Sw)**: **AP** – Appenzell, **BE** – Bern, **FR** – Fribourg, **GL** – Glarus, **GR** – Graubünden, **LU** – Luzern, **SG** – St. Gallen, **SZ** – Schwyz, **TI** – Ticino, **UR** – Uri, **UW** – Unterwalden, **VD** – Vaud, **VS** – Valais.

*France* **(Fr)**: in brackets the number designating each Departement in the French administrative system. **AHP** – Alpes-de-Haute-Provence (04), **HAl** – Haute-Alpes (05), **AMa** – Alpes-Maritimes (06), **Drô** – Drôme (26), **Var** – Var (83), **Isè** – Isère (38), **Sav** – Savoie (73), **HSav** – Haute-Savoie (74), **Vau** – Vaucluse (84).

*Italy* **(It)**: **Frl** – Friuli (excluding the Province of Trieste), **Ven** – Veneto, **TAA** – Trentino-Alto Adige, **Lomb** – Lombardia, **Piem** – Piemonte, **VA** – Valle d’Aosta, **Lig** – Liguria (limited to the westernmost part of the region).

*Slovenia* **(Sl)**: **SlA** – Alpine and Pre-Alpine Slovenia, **Tg** – Trnovsky gozd.

*Liechtenstein* **(Li)**.

### Notes

The notes to each taxon briefly describe its main ecology and distribution. For poorly known taxa, a brief description has often been added, in order to help the reader understanding to what the name may refer. For obvious reasons of space, in the notes, the authors have refrained from citing any literature, except the national checklists on which the present catalogue is based. Those are referred to for more detailed literature citations.

### Databasing the checklist

The present checklist will be converted into a freely searchable database within a month from its publication in paper-form. The database will also include a searchable thesaurus of synonyms, which will compensate for the fact that, for reasons of space, this paper-printed version is not provided with an alphabetical index for the thousands of names included in the text.

### The lichen diversity of the Alps: some numbers

The present checklist includes, excluding the dubious records, 3,163 infrageneric taxa, 3,009 of which are certainly lichenised. The number of poorly known taxa is quite high (604, 19% of the total), which indicates that much work is needed to reach a satisfactory picture of the real lichen diversity of the Alpine system.

The number of infrageneric taxa known for the different countries and their subdivisions, only their “Alpine” areas being considered, is as follows:

Austria (2,337 infrageneric taxa): V – Vorarlberg (1,249), T – Tirol (1,704), S – Salzburg (1,495), K – Kärnten (1,525), St – Steiermark (1,670), O – Oberösterreich (1,001), N – Niederösterreich (1,194), B – Burgenland (280).

Italy (2,169): Frl – Friuli (1,022), Ven – Veneto (1,160), TAA – Trentino-Alto Adige (1,562), Lomb – Lombardia (1,298), Piem – Piemonte (1,282), VA – Valle d’Aosta (793), Lig – Liguria (722).

France (2,028): AHP – Alpes-de-Haute-Provence (1,056), HAl – Haute-Alpes (788), AMa – Alpes-Maritimes (1,392), Drô – Drôme (363), Var – Var (841), Isè – Isère (747), Sav – Savoie (858), HSav – Haute-Savoie (1,062), Vau – Vaucluse (848).

Germany (1,168): OB – Oberbayern (942), Schw – Schwaben (630).

Switzerland (1,835): AP – Appenzell (51), BE – Bern (960), FR – Fribourg (147), GL – Glarus (305), GR – Graubünden (1,206), LU – Luzern (609), SG – St. Gallen (238), SZ – Schwyz (873), TI – Ticino (697), UR – Uri (655), UW – Unterwalden (467), VD – Vaud (598), VS – Valais (1,191).

Slovenia (890): SlA – Alpine and Pre-Alpine Slovenia (843), Tg – Trnovsky gozd (346).

Liechtenstein (152).

The number of taxa is well in agreement with the percentage of the Alpine area occupied by the various countries. Comparing the smaller OGUs is quite difficult, considering that they vary considerably in surface areas, geomorphological heterogeneity and degree of conservation of local ecosystems. In general terms, however, the richest areas are located in the Eastern Alps, such as Tyrol (1,704 taxa), Steiermark (1,670), Trentino-Alto Adige (1,562) and Kärnten (1,525), while, even considering their mostly smaller surface areas, several OGUs located in the Western Alps, especially in Switzerland and in France and those in Germany and in Slovenia, would need a more intense lichenological exploration.

### Concluding remarks

Checklists summarise, in a more or less critical way, the hitherto known information on the biodiversity of a given group of organisms in a given area. They can have different nature, scope and contents and they should always be judged considering the situation of floristic and taxonomic research that they reflect. Obviously, not all literature records can be accepted uncritically: the circumscription of taxa may differ amongst authors, recent taxonomic revisions might have shown that a given taxon actually includes several taxa of the corresponding rank, some authors may be more reliable than others etc. The author of a checklist is often forced to make difficult decisions, since in most cases, it is not possible to check directly all identifications cited in literature. Checklists might differ also on account of the degree of exploration of the area they cover. In the case of poorly explored areas, they just summarise the current state of knowledge, but cannot pretend to be exhaustive. For well-explored areas, one could think that they do not only represent a basis for future updates, but also a kind of *prodromus* for a true Flora. This, however, is not the case of the present checklist. The idea that the degree of taxonomic knowledge parallels that of floristic exploration, i.e. that in well-studied areas, most infrageneric taxa are likely to be relatively well-delimited taxonomically, proved to be basically wrong. The authors’ checklist includes many long-forgotten names referring to very poorly understood taxa, often only known from the type collection, which are in need of critical revision. Thus, the total number of taxa accepted in this checklist does not reflect the actual species diversity of the Alps, due to inadequate taxonomic knowledge. Incidentally, further taxonomic research will often reduce rather than increase the number of accepted taxa. The citation of these names in the checklist is, however, important, because it will bring these potentially correct names, often published in long-forgotten papers, to the attention of specialists. For this reason, a number of species were also transferred to genera to which they most likely belong, in order to increase the probability of their inclusion in future critical revisions.

Checklists are never-ending ventures, subject to continuous updating following the developments of current research. It is hoped that the present checklist will prove to be a valuable tool for retrieving and accessing the enormous amount of information on the lichens of the Alps which has accumulated during centuries of research, offering a basis for specimen revision, for the critical re-appraisal of poorly-known taxa and for the further exploration of under-investigated areas, becoming a catalyst for new, more intensive investigations. The best criterion for a checklist to have accomplished its task as a facility to the scientific community, is the speed of its becoming outdated (Nimis 2016), which is what is paradoxically wished for the present one.

### Taxonomic and nomenclatural novelties

***Agonimia bryophilopsis*** (Vain.) Hafellner, comb. nov. MB 824184 – Bas.: *Polyblastia bryophilopsis* Vain., Acta Soc. Fauna Flora Fennica 49(2): 104 (1921).

***Aspicilia niesenensis*** (H. Magn.) Hafellner, comb. nov. MB 824185 – Bas.: *Lecanora niesenensis* H. Magn., Kungl. Svensk Vetensk. Handl. ser 3, 17: 97 (1939).

***Bagliettoa crassiuscula*** (Servít) Hafellner, comb. nov. MB 824186 – Bas.: *Verrucaria crassiuscula* Servít, Studia Botanica Cechoslovaca 9: 78 (1948) as nom. nov. for *Verrucaria crassa* A. Massal. 1852 *non* Eschw. 1833.

***Bellemerea subnivea*** (Müll. Arg.) Hafellner, comb. nov. MB 824187 – Bas.: *Lecanora subnivea* Müll. Arg., Flora (Regensburg) 55: 467 (1872).

***Carbonea viriduloatra*** (B. de Lesd.) Hafellner, comb. nov. MB 824188 – Bas.: *Lecidea viriduloatra* B. de Lesd., Bull. Soc. Bot. France 57: 32 (1910).

***Lepra erumpens*** (Erichsen) Hafellner, comb. nov. MB 824189 – Bas.: *Pertusaria erumpens* Erichsen, Acta Fauna Flora Univ., ser. 2, Bot., 1(17): 1 (1935).

***Miriquidica aeneovirens*** (Müll. Arg.) Hafellner, comb. nov. MB 824190 – Bas.: *Lecidea aeneovirens* Müll. Arg., Flora (Regensburg) 57: 530 (1874).

***Polysporina limborinella*** (Müll. Arg.) Hafellner, comb. nov. MB 824191 – Bas.: *Lecidea limborinella* Müll. Arg., Bull. Trav. Soc. Murithienne Valais 10: 64 (1881).

***Protothelenella anodonta*** (Nyl.) Hafellner, comb. nov. MB 824192 – Bas.: *Odontotrema anodontum* Nyl., Flora 52: 411 (1869).

***Protothelenella viridis*** (Rehm) Hafellner, comb. nov. MB 824193 – Bas.: *Melanomma viridis* Rehm, Hedwigia 21: 119 (1882).

***Pseudosagedia lucens*** (Taylor) Hafellner, comb. nov. MB 824194 – Bas.: *Verrucaria lucens* Taylor, in Mackay, Flora Hibernica 2: 257 (1836).

***Thelidium helveticum*** (Servít) Hafellner, comb. nov. MB 824195 – Bas.: *Involucrothele helvetica* Servít, Rozpravy Československé Akademie Věd 65(3): 15 (1955).

***Thelidium pyrenophorellum*** (Servít) Hafellner, comb. nov. MB 824196 – Bas.: *Involucrothele pyrenophorella* Servít, Rozpravy Československé Akademie Věd 63(7): 22 (1953).

## The taxa

### Lichenised taxa

***Absconditella annexa* (Arnold) Vězda**

Syn.: *Gyalecta annexa* (Arnold) H. Olivier, *Secoliga annexa* Arnold

L – Subs.: ter-sil, bry-sil – Alt.: 4-5 – Note: an ephemeral lichen found on moribund bryophytes and organic soil over siliceous substrata; in the study area so far reported only from the Eastern Alps (Austria, Italy), but certainly more widespread. – **Au**: V, T, K, St. **It**: Frl.

***Absconditella delutula* (Nyl.) Coppins & H. Kilias**

Syn.: *Absconditella modesta* (Hegetschw.) Vězda, *Gyalecta modesta* (Hegetschw.) Zahlbr., *Lecidea delutula* Nyl., *Lecidea modesta* Hegetschw., *Secoliga modesta* (Hegetschw.) Arnold

L – Subs.: sil – Alt.: 3 – Note: a coloniser of small stones and pebbles in moist and shaded situations; so far reported from a few scattered localities in the Alps, but perhaps more widespread. – **Au**: S, St, N. **Sw**: LU. **Fr**: Isè.

***Absconditella lignicola* Vězda & Pišút**

L – Subs.: xyl – Alt.: 2-3 – Note: on moist decaying wood in the shade of forests, mostly on logs and horizontal cut surfaces; perhaps more widespread in the Alps. – **Au**: T, K, St, O, N, B. **Sw**: GR, LU, SZ, VS. **It**: TAA. **Sl**: SlA.

***Absconditella pauxilla* Vězda & Vivant**

L – Subs.: xyl – Alt.: 3-4 – Note: distinguished from *A. lignicola* by the narrower ascospores (< 3 µm); on wood, more rarely on siliceous rocks in forests; in the study area so far only reported from Switzerland. – **Sw**: SZ.

***Absconditella sphagnorum* Vězda & Poelt**

L – Subs.: bry, xyl – Alt.: 3 – Note: on moribund *Sphagnum* in raised bogs, usually in the uppermost part of the cushions, in sunny places; locally abundant in late summer and autumn, especially after dry summers, and probably somehow overlooked due to its ephemeral character. – **Au**: St. **Ge**: OB, Schw.

***Absconditella trivialis* (Willey *ex* Tuck.) Vězda**

Syn.: Gyalecta geoica (Wahlenb.) Ach. f. trivialis Willey *ex* Tuck.

L – Subs.: ter-sil – Alt.: 3 – Note: on clay soil under moist conditions; perhaps more widespread in the Alps, being easy to overlook. – **Au**: St. **Ge**: OB.

***Acarospora admissa* (Nyl.) Kullh.**

Syn.: *Lecanora admissa* Nyl.

L – Subs.: sil – Alt.: 3-5 – Note: similar to *A. nitrophila*, but thallus squamules with wavy-crenulate, mostly black margins; in the study area so far only reported from the Western Alps (France). – **Fr**: AHP, AMa, Sav.

***Acarospora albomarginata* (H. Magn.) Clauzade & Cl. Roux [*nom.illeg.* , *non* B. de Lesd. *nec* (Herre) G. Salisb.**]

Syn.: Acarospora hospitans H. Magn. f. albomarginata H. Magn.

L # – Subs.: sil – Alt.: 3–4 – Note: a poorly known species resembling in habitus, and probably related to *A. hospitans*, differing in having larger thalline squamules with a white margin, apothecia usually 2–5 per areole, surrounded by a prominent thin thalline margin and with a rough to umbonate disc, asci containing more than 100 ellipsoid ascospores (3.5–5 × 2–3 μm); on exposed outcrops and boulders of schists with low content of calcium in sunny sitations; only known from the type locality in the Eastern Alps (Switzerland). – **Sw**: GR.

***Acarospora anomala* H. Magn.**

L # – Subs.: xyl – Alt.: 3–4 – Note: a species of eutrophicated, dry and hard lignum, closely related to other saxicolous species, hitherto reported from Scandinavia and a few scattered localities in the Alps. – **Fr**: Isè. **It**: TAA.

***Acarospora austriaca* H. Magn.**

L # – Subs.: sil – Alt.: 4 – Note: the type, from the Austrian Alps, is perhaps *A. helvetica*, but according to Roux et al. (2014) it is different from *A. complanata*. – **Au**: St.

**Acarospora badiofusca (Nyl.) Th. Fr. subsp. badiofusca**

Syn.: *Lecanora badiofusca* Nyl.

L – Subs.: sil, int – Alt.: 3–5 – Note: an arctic-alpine to boreal-montane, circumpolar species of base-rich or lime-containing siliceous rocks, such as mica-schists and calciferous sandstone, found on faces wetted by rain, including stones near the ground in grasslands; widespread and locally common throughout the Alps. – **Au**: V, T, S, K, St. **Sw**: BE, GR, LU, SZ, UR, VS. **Fr**: AHP, HAl, AMa, Isè, Sav, HSav. **It**: Frl, TAA, Lomb, Piem, VA, Lig.

**Acarospora badiofusca (Nyl.) Th. Fr. subsp. badiorubra Clauzade & Cl. Roux**

L – Subs.: sil – Alt.: 3–5 – Note: non-calcicolous, and more thermophilous than the typical subspecies; certainly more widespread in the Alps. – **Au**: T, K, St. **Fr**: AHP, HAl. **It**: Frl, VA.

***Acarospora bullata* Anzi**

L – Subs.: int – Alt.: 3–5 – Note: closely related to *A. complanata*, but with a clearly effigurated thallus; on steeply inclined faces of base-rich, weakly calciferous siliceous rocks; probably more widespread, but certainly not common in the Alps. – **Au**: K, St. **Sw**: GR, VS. **Fr**: AMa. **It**: Lomb, Piem, VA.

***Acarospora cervina* A. Massal.**

Syn.: Acarospora glaucocarpa (Ach.) Körb. var. cervina (A. Massal.) Cl. Roux

L – Subs.: cal – Alt.: 1–5 – Note: a widespread, probably holarctic species found on the top of exposed, more or less calcareous boulders in natural habitats, especially common in dry-continental areas, but with a wide altitudinal range, sometimes considered as a form of *A. glaucocarpa*. The nomenclature should be studied further: according to Nimis (2016) Massalongo was not describing a species, but proposing a new combination. – **Au**: V, T, S, K, St, O, N. **Ge**: OB, Schw. **Fr**: AHP, HAl, AMa, Drô, Isè, Sav, HSav, Var, Vau. **It**: Frl, Ven, TAA, Lomb, Piem, VA.

***Acarospora chrysocardia* Poelt & M. Steiner**

L – Subs.: sil-par – Alt.: 2–3 – Note: on base-rich siliceous rocks, growing on the thalli of *Diploschistes scruposus* below the subalpine belt; hitherto known only from dry-warm valleys of the Western Alps and Catalonia, and certainly worthy of protection. – **Sw**: VS. **It**: Piem, VA.

***Acarospora cinerascens* J. Steiner**

Syn.: *Acarospora alboatra* H. Magn.

L – Subs.: sil – Alt.: 2–3 – Note: on weathered base-rich siliceous rocks, restricted to dry-warm valleys of the Alps with a continental climate. – **Sw**: VS. **It**: TAA, VA.

***Acarospora complanata* H. Magn**

Syn.: ?*Acarospora crozalsii* B. de Lesd.

L # – Subs.: sil – Alt.: 3–5 – Note: this species was described from France, on basaltic rocks, and has a southern distribution in Europe, extending to North Africa; it has been also reported from North America. It belongs to a difficult complex of closely related taxa, which is in need of revision. Its ecology is poorly understood as well, the species being most frequent on base-rich siliceous rocks. – **Au**: T, S, K, St, N. **Sw**: BE, TI. **Fr**: AMa, Var, Vau. **It**: Piem, Lig.

***Acarospora discreta* (Ach.) Th. Fr.**

Syn.: *Acarospora durietzii* H. Magn., Parmelia squamulosa Ach. var. discreta Ach.

L # – Subs.: sax – Alt.: 3–4 – Note: based on a type from extra-Alpine Europe (Scandinavia), with a few records from from the Swiss Alps. – **Sw**: GR, VS.

***Acarospora freyi* H. Magn.**

Syn.: Acarospora impressula Th. Fr. var. freyi (H. Magn.) Clauzade & Cl. Roux

L – Subs.: sil, met, int – Alt.: 5–6 – Note: probably overlooked and more widespread in the Alps, with optimum near and above treeline, this lichen starts the life-cycle on *Aspicilia candida* and *A. polychroma* on calciferous rocks which are at least partly decalcified on the surface. – **Au**: T, S. **Sw**: BE. **Fr**: AHP, HAl, AMa. **It**: Lomb, Piem, VA.

***Acarospora fuscata* (Schrad.) Arnold.**

Syn.: *Acarospora squamulosa* (Schrad.) Trevis., *Lichen fuscatus* Schrad.

L – Subs.: sil, int – Alt.: 2–5 – Note: a holarctic species of acid siliceous rocks wetted by rain, sometimes growing on other nitrophytic lichens; widespread throughout the Alps. – **Au**: V, T, S, K, St, O, N, B. **Ge**: OB, Schw. **Sw**: BE, GR, LU, SZ, TI, UR, VD, VS. **Fr**: AHP, HAl, AMa, Isè, Sav, HSav, Var, Vau. **It**: Frl, Ven, TAA, Lomb, Piem, VA. **Sl**: SlA.

***Acarospora gallica* H. Magn.**

Syn.: ?*Acarospora hungarica* H. Magn.

L – Subs.: sil, int – Alt.: 1–3 – Note: a probably holarctic species of base-rich, weakly calciferous siliceous substrata, such as calcareous sandstone, brick, and roofing tiles, with several scattered records from the Alps. – **Au**: T, K, N. **Sw**: GR. **Fr**: AMa, Var. **It**: Piem, Lig.

***Acarospora glaucocarpa* (Ach.) Körb.**

Syn.: Acarospora cervina A. Massal. var. conspersa (Th. Fr.) Clauzade & Cl. Roux, Acarospora glaucocarpa (Ach.) Körb. var. conspersa Th. Fr., *Biatora conspersa* Fr. *nom. nud.*, *Parmelia glaucocarpa* Ach.

L – Subs.: cal – Alt.: 1–5 – Note: a widespread, probably holarctic species found on more or less calcareous boulders in natural habitats, sometimes overgrowing other crustose lichens, with a wide altitudinal range but most common in upland areas; closely related to *A. cervina*, perhaps more frequent in less exposed situations; widespread and locally common throughout the Alps. – **Au**: V, T, S, K, St, O, N, B. **Ge**: OB, Schw. **Sw**: BE, GR, LU, SZ, UR, UW, VD, VS. **Fr**: AHP, HAl, AMa, Isè, Sav, HSav, Var, Vau. **It**: Frl, Ven, TAA, Lomb, Piem, Lig. **Sl**: SlA, Tg.

***Acarospora hellbomii* H. Magn.**

Syn.: ?*Acarospora marcii* H. Magn.

L # – Subs.: sil, met – Alt.: 3–4 – Note: the type, from extra-Alpine Europe (Scandinavia), is perhaps identical with *A. peliscypha*; the synonymy with *A. marcii*, which is also based on a type from extra-Alpine Europe (SW Europe), is uncertain. – **Fr**: AMa. **It**: TAA, VA (as *A. marcii*).

***Acarospora helvetica* H. Magn.**

Syn.: *Acarospora intermedia* H. Magn.

L # – Subs.: sil – Alt.: 3-4 – Note: a taxon based on a type from the Alps; according to Roux it is different from *A. complanata*. – **Fr**: AHP, AMa.

***Acarospora heufleriana* Körb.**

Syn.: Acarospora heufleriana Körb. var. massiliensis Harm., *Acarospora massiliensis* (Harm.) H. Magn.

L – Subs.: sil-par – Alt.: 1–2 – Note: on horizontal to gently sloping faces near the ground in open habitats, especially in grasslands, sometimes starting the life-cycle on other crustose lichens; restricted to dry-continental areas, both in the Alps and in the Mediterranean Region. – **Au**: T. **Sw**: VS. **It**: TAA, Piem, VA.

***Acarospora hospitans* H. Magn.**

Syn.: Acarospora impressula Th. Fr. var. hospitans (H. Magn.) Clauzade & Cl. Roux

L – Subs.: sil-par – Alt.: 4–5 – Note: closely related to *A. impressula*; parasitic on several silicicolous species of *Aspicilia*; widespread throughout the siliceous Alps. – **Au**: V, T, S, K, St. **Ge**: OB. **Sw**: BE, VD, VS. **Fr**: AHP, HAl, AMa, Sav, HSav. **It**: TAA, Piem.

***Acarospora hostilis* H. Magn.**

L # – Subs.: sil, sil-par – Alt.: 4–5 – Note: a species resembling in habitus *A. veronensis*, with a thallus consisting of dispersed, red-brown, irregular, flattened areoles (not reacting with K, C, Pd) with incised to sublobate margins and a pale underside, a thin thalline cortex of small cells, immersed, contiguous to fusing apothecia (0.2–0.4 mm in diam.), a more than 100 µm tall hymenium, and asci with more than 100, broadly ellipsoid ascospores (2–3.5 × 1.5 μm); on siliceous boulders, often invading the thallus of other crustose lichens; described from the treeline ecotone in Northern Italy and only known from the Alps; the study of the type material could prove that this a synonym for another species. – **It**: TAA.

***Acarospora imbricatula* H. Magn.**

L – Subs.: sil – Alt.: 2–3 – Note: hitherto known only from dry-continental valleys in the Alps, on south-facing slopes, where it is locally common. – **Sw**: VS. **Fr**: AHP, HAl, AMa. **It**: TAA, VA.

***Acarospora impressula* Th. Fr.**

Syn.: *Acarospora atrata* Hue

L – Subs.: sil, met – Alt.: 2–5 – Note: an arctic-alpine to boreal-montane, probably circumpolar species found on metal-rich rocks and roofing slates, more rarely on weakly calciferous siliceous rocks, usually in upland areas, with optimum above treeline; probably overlooked and more widespread in the Alps. – **Au**: V, T, S, K, St, N. **Sw**: BE, GR, SZ, VS. **Fr**: AHP, HAl, AMa, Sav, HSav. **It**: Frl, TAA, Piem, VA.

***Acarospora insolata* H. Magn.**

L – Subs.: sil, sil-par – Alt.: 2–5 – Note: on inclined surfaces of siliceous rocks wetted by rain, often growing on other crustose lichens (*e.g. Immersaria*, *Rhizocarpon*); certainly more widespread in the Alps. – **Au**: ?V. **Ge**: OB. **Sw**: GR, TI. **It**: Ven, Piem.

***Acarospora laqueata* Stizenb.**

Syn.: ?*Acarospora caesiocinerea* B. de Lesd., *Lecanora laqueata* (Stizenb.) Stizenb.

L – Subs.: cal – Alt.: 1–2 – Note: on hard calcareous rocks, both on vertical faces and at the top of birds’ perching sites in dry-continental areas; very rare in the Alps. – **Sw**: VS. **Fr**: AHP, Var, Vau. **It**: Lomb.

***Acarospora macrospora* (Hepp) A. Massal. *ex* Bagl.**

Syn.: *Acarospora castanea* (DC.) Körb, *Acarospora squamulosa* *sensu* Th. Fr. *non* (Schrad.) Trevis., *Myriospora macrospora* Hepp

L – Subs.: cal – Alt.: 3–5 – Note: on steeply inclined faces of fissured calcareous rocks in upland areas; widespread throughout the Alps. See also note on *A. murorum*. – **Au**: V, T, S, K, St, O, N. **Ge**: OB, Schw. **Sw**: BE, GR, LU, SZ, VD, VS. **Fr**: AHP, AMa, Drô, Isè, Sav, HSav, Var, Vau. **It**: Frl, Ven, TAA, Lomb, Piem. **Sl**: SlA, Tg.

***Acarospora melaplaca* (Nyl.) Arnold**

Syn.: *Lecanora melaplaca* Nyl.

L # – Subs.: sil – Alt.: 3–5 – Note: a species with blackish-brown thallus and effigurate, very thin areolae, whose ecology and distribution need further study. – **Au**: T. **Sw**: GR.

***Acarospora microcarpa* (Nyl.) Wedd.**

Syn.: *Acarospora tersa* (Fr.) J. Steiner, Lecanora schleicheri (Ach.) Nyl. var. microcarpa Nyl., *Lecanora tersa* (Fr.) Nyl.

L – Subs.: sil-par – Alt.: 1–2 – Note: a mainly Mediterranean-Atlantic species with optimum in coastal situations; on basic siliceous rocks wetted by rain, parasitic on several crustose lichens, especially *Diploschistes actinostoma*; extremely rare in the dry-continental valleys of the Alps. – **It**: TAA, Piem.

***Acarospora modenensis* H. Magn.**

Syn.: ?*Acarospora engadinensis* H. Magn.

L – Subs.: sil – Alt.: 1–4 – Note: a mild-temperate species of siliceous rocks, often found on walls; apparently more frequent in the Western and Southern Alps, but probably overlooked and more widespread elsewhere in lowland areas. – **Sw**: GR. **Fr**: HAl, AMa, HSav. **It**: Piem, Lig.

***Acarospora moenium* (Vain.) Räsänen**

Syn.: *Aspicilia excavata* G. Thor & Timdal, *Aspicilia moenium* (Vain.) G. Thor & Timdal, *Endocarpon moenium* Vain.

L – Subs.: cal – Alt.: 2–3 – Note: a mainly temperate, inconspicuous species, usually sterile, the rare case of a sorediate *Acarospora*; certainly more widespread on man-made substrata (mortar, cement, etc.), or more rarely on calciferous schists, on steeply inclined faces. – **Au**: S, St, N, B. **Ge**: OB, Schw. **Sw**: GR, VS. **It**: Frl, TAA, Lomb. **Sl**: SlA.

***Acarospora murorum* A. Massal.**

Syn.: *Acarospora macrospora* (Hepp) A. Massal. *ex* Bagl. subsp. murorum (A. Massal.) Clauzade & Cl. Roux. *Acarospora truncata* (A. Massal.) A. Massal., *Biatorella truncata* A. Massal.

L – Subs.: cal – Alt.: 1–3 – Note: a mild-temperate species found on walls, gravestones, and monuments; related to *A. macrospora*, but with a different ecology and altitudinal distribution; apparently more frequent in the Western and Southern Alps. – **Au**: S. **Sw**: BE, LU. **Fr**: AHP, AMa, Sav, HSav, Var, Vau. **It**: Frl, Ven, TAA, Lomb, Piem, Lig.

**Acarospora nitrophila H. Magn. subsp. nitrophila**

L – Subs.: sil – Alt.: 2–5 – Note: a widespread species found on steeply inclined to overhanging faces of basic siliceous rocks, usually in species-poor communities, mostly near settlements; widespread throughout the Alps. – **Au**: V, T, S, K, St, N. **Ge**: OB, Schw. **Sw**: BE, GR, SZ, TI, UR, VS. **Fr**: HAl, AMa, HSav, Var. **It**: TAA, Lomb, Piem, VA, Lig.

**Acarospora nitrophila H. Magn. subsp. normanii (H. Magn.) Clauzade & Cl. Roux**

Syn.: *Acarospora normanii* H. Magn.

L # – Subs.: sil – Alt.: 3–5 – Note: a rather poorly known taxon, in the study area so far only reported from the Alps of Austria and France. – **Au**: ?V. **Fr**: HAl.

**Acarospora nitrophila H. Magn. subsp. praeruptorum (H. Magn.) Clauzade & Cl. Roux**

Syn.: Acarospora nitrophila H. Magn. var. praeruptorum (H. Magn.) Clauzade & Cl. Roux, *Acarospora praeruptorum* H. Magn.

L # – Subs.: sil – Alt.: 2–5 – Note: in the checklist of Italy (Nimis 2016) this taxon is subsumed under *A. nitrophila*. – **Au**: T, S, St, N. **Fr**: AHP, AMa, Sav, Var.

**Acarospora nodulosa (Dufour) Hue var. nodulosa**

Syn.: *Lecanora nodulosa* (Dufour) Colmeiro, *Parmelia nodulosa* Dufour, *Urceolaria nodulosa* (Dufour) Schaer.

L – Subs.: ter-cal – Alt.: 2 – Note: a xeric subtropical species, parasitic on *Diploschistes* spp., found on weathered gypsum and calcareous substrata in very open habitats. – **Sw**: VS.

**Acarospora nodulosa (Dufour) Hue var. reagens (Zahlbr.) Clauzade & Cl. Roux**

Syn.: *Acarospora reagens* Zahlbr.

L # – Subs.: ter-cal – Alt.: 2 – Note: this taxon, based on a type from Western North America, is sometimes treated as chemical strain without rank, sometimes at species level; often parasitic on *Diploschistes diacapsis*. – **Fr**: Drô.

***Acarospora oligospora* (Nyl.) Arnold**

Syn.: *Acarospora glebosa* (Flot.) Körb., *Lecanora oligospora* Nyl.

L – Subs.: sil, int – Alt.: 1–3 – Note: a holarctic-temperate species found on basic siliceous rocks (*e.g.* calciferous sandstone and schist), usually on pebbles, but also on walls, roofing tiles, etc., below the subalpine belt. – **Au**: T, St. **Ge**: OB. **Fr**: AMa, Sav, HSav. **It**: Ven, TAA, Lomb, Piem.

***Acarospora peliscypha* Th. Fr.**

L – Subs.: sil, met – Alt.: 3–5 – Note: an arctic-alpine to boreal-montane, probably circumpolar species found on siliceous, often iron-rich substrata, on exposed birds’ perching rocks (*e.g.* windy ridges, isolated boulders). See also notes on *A. bullata* and *A. rugulosa*. – **Au**: V, T, S, K, St, N. **Sw**: GR, UR. **Fr**: HAl, HSav. **It**: TAA, Lomb, Piem, VA.

***Acarospora picea* H. Magn.**

L – Subs.: sil – Alt.: 5 – Note: among the species with a brown effigurate thallus, this is the only one with globose ascospores; described from high altitudes in the Sierra Nevada (Spain), with a few records from the Western Alps (France), growing on non – or slightly calciferous siliceous rocks in dry, sunny, moderately eutrophicated situations. – **Fr**: AHP, AMa.

***Acarospora pyrenopsoides* H. Magn.**

L # – Subs.: sil – Alt.: 2–5 – Note: a taxon probably belonging to the *A. nitrophila*-group; the small apothecia, several per areola, and the narrowly ellipsoid ascospores are diagnostic; the ecology is poorly known, but the species usually occurs on steep faces and overhangs of siliceous rocks; known from a few localities in the Alps; the type is from Greenland. – **Au**: T. **Fr**: AMa, HSav.

***Acarospora rosulata* (Th. Fr.) H. Magn.**

Syn.: Acarospora discreta (Ach.) Th. Fr. f. rosulata Th. Fr.

L – Subs.: sil – Alt.: 4–5 – Note: characterised by the rosulate thalli with indistinctly lobate peripheral squamules with a whitish lower surface, this species, described from Norway where it is rare, is known from Western North America, Asia (Mongolia) and the Alps. It grows on sun-exposed siliceous rocks, with optimum in dry, subcontinental areas; being easily overlooked, the species might be more widespread in the Alps. – **Fr**: AHP, AMa, HSav. **It**: Lomb.

***Acarospora rugulosa* Körb.**

L – Subs.: sil, met – Alt.: 3–5 – Note: closely related to *A. peliscypha*. – **Au**: T, S, K. **Fr**: AHP, AMa.

***Acarospora schleicheri* (Ach.) A. Massal.**

Syn.: *Urceolaria schleicheri* Ach.

L – Subs.: ter-cal, ter-par – Alt.: 2 – Note: on consolidated soil and facultatively parasitic on *Diploschistes diacapsis*, in dry habitats; widespread in Eurasia and North America; in the Alps confined to inner-Alpine dry valleys; the type material is from the Alps (Switzerland). – **Sw**: VS.

***Acarospora scotica* Hue**

L – Subs.: sil – Alt.: 1–3 – Note: a probably Mediterranean-Atlantic species of siliceous rocks wetted by rain, reaching the montane (rarely also the subalpine) belt in the Western Alps. – **Fr**: AHP, AMa, Var, Vau. **It**: Lomb, Piem.

***Acarospora similis* H. Magn.**

L # – Subs.: xyl – Alt.: 2–4 – Note: a lignicolous taxon of the *A. veronensis*-group, often found on roofing tiles, based on a type from outside the Alps (Switzerland), with a few records from the Alps. – **Fr**: HSav. **It**: TAA.

***Acarospora sinopica* (Wahlenb.) Körb.**

Syn.: Acarospora smaragdula (Wahlenb.) A. Massal. var. sinopica (Wahlenb.) A. Massal., *Endocarpon sinopicum* Wahlenb., *Polysporinopsis sinopica* (Wahlenb.) Vězda, *Zeora sinopica* (Wahlenb.) Flot.

L – Subs.: met – Alt.: 2–5 – Note: a probably holarctic species of iron-rich rocks and mine-spoil heaps in exposed situations; widespread, but local, throughout the Alps. – **Au**: V, T, S, K, St, B. **Sw**: BE, GR, TI, UR, VD, VS. **Fr**: HAl, AMa, Sav, HSav. **It**: Frl, TAA, Lomb, Piem, VA.

**Acarospora sulphurata (Arnold) Arnold var. sulphurata**

Syn.: Acarospora heufleriana Körb. f. sulphurata Arnold

L – Subs.: sil – Alt.: 2–3 – Note: a species of dry-continental areas, found on basic siliceous rocks, often near the ground, in dry grasslands, both in dry Mediterranean areas and in continental inner-Alpine valleys. – **It**: TAA, Piem, VA.

**Acarospora sulphurata (Arnold) Arnold var. rubescens Buschardt**

L – Subs.: sil – Alt.: 2–3 – Note: perhaps just a chemical strain; hitherto known only from the Alps, where it is restricted to inner-Alpine valleys. – **It**: TAA.

**Acarospora suzai H. Magn. var. tyroliensis H. Magn.**

Syn.: *Acarospora tyroliensis* (H. Magn.) H. Magn.

L # – Subs.: sil – Alt.: 2–5 – Note: a taxon of the *A. nitrophila*-complex, which needs further study. – **Au**: T. **Fr**: AMa.

***Acarospora tenuicorticata* H. Magn.**

L # – Subs.: sil – Alt.: 3–4 – Note: in the study area only reported from a few localities in the Eastern Alps (Austria). – **Au**: S, St.

**Acarospora tongletii (Hue) Hue var. tongletii**

Syn.: *Acarospora variegata* H. Magn., *Lecanora tongletii* Hue

L – Subs.: sil – Alt.: 2–5 – Note: a temperate to southern boreal-montane, probably circumpolar species, most frequent on base-rich sandstone walls; much overlooked, and perhaps more widespread in the Alps. – **Au**: K. **Sw**: GR.

**Acarospora tongletii (Hue) Hue var. paupera (H. Magn.) Clauzade & Cl. Roux**

Syn.: *Acarospora paupera* H. Magn.

L # – Subs.: sax – Alt.: 2–3 – Note: in the study area only reported from the base of the Western Alps (France). – **Fr**: AMa.

***Acarospora umbilicata* Bagl.**

Syn.: *Acarospora cinerea* (Nyl.) Wedd., *Acarospora percaenoides* (Nyl.) Flagey, *Acarospora rufidulocinerea* Hue

L – Subs.: sil – Alt.: 1–3 – Note: a mild-temperate, mainly Mediterranean-Atlantic species found on steeply inclined sunny faces of basic siliceous substrata, on roofing tiles and brick; widespread but rare in the Alps. – **Au**: T, N. **Sw**: VS. **Fr**: AMa, Sav, Var. **It**: TAA, Piem, VA, Lig.

***Acarospora valdobbiensis* Bagl. & Carestia**

Syn.: *Biatorella valdobbiensis* (Bagl. & Carestia) Zahlbr., *Lecanora valdobbiensis* (Bagl. & Carestia) Stizenb., *Sarcogyne valdobbiensis* (Bagl. & Car.) Jatta

L # – Subs.: sil – Alt.: 4–5 – Note: a very poorly known taxon, also reported from Macedonia. The type, which from the description is actually an *Acarospora*, was collected on schist in the alpine belt. – **It**: Piem.

***Acarospora veronensis* A. Massal.**

Syn.: Acarospora fuscata (Schrad.) Arnold. subsp. discreta *sensu* Th. Fr., *Acarospora magnussonii* Samp.

L – Subs.: sil – Alt.: 1–5 – Note: a holarctic early coloniser of base – rich siliceous pebbles, roofing tiles, walls, sometimes also found on soil and lignum, also in small settlements: occasionally overgrowing other crustose lichens, with a wide altitudinal range; widespread throughout the Alps. – **Au**: V, T, S, K, St, O, N. **Ge**: OB. **Sw**: BE, GR, LU, SZ, TI, UR, VS. **Fr**: AHP, HAl, AMa, Sav, HSav, Var, Vau. **It**: Frl, Ven, TAA, Lomb, Piem, VA.

***Acarospora versicolor* Bagl. & Carestia**

Syn.: *Acarospora cineracea* (Nyl.) Hue; incl. *Acarospora miskolensis* H. Magn.

L – Subs.: sil – Alt.: 1–3 – Note: on basic siliceous rocks, also on walls in small Alpine settlements, and on thin soil layers, probably more widespread in the Alps, below the subalpine belt. – **Au**: ?V, T, St, N. **Sw**: BE, GR, VS. **Fr**: AMa, Sav. **It**: TAA, Lomb, Piem.

***Acolium inquinans* (Sm.) A. Massal.**

Syn.: *Acolium neesii* (Flot.) Körb., *Acolium subsimile* (Nyl.) Arnold, *Acolium tympanellum* (Ach.) Gray, *Calicium cembrinum* Ach., *Calicium inquinans* (Sm.) Schaer., *Calicium neesii* Flot., *Calicium tympanellum* Ach., *Cyphelium cembrinum* (Ach.) Ach., *Cyphelium inquinans* (Sm.) Trevis., Cyphelium inquinans (Sm.) Trevis. var. ollare (Ach.) Trevis., *Cyphelium neesii* (Flot.) Trevis., *Cyphelium ollare* Ach., *Cyphelium pileatum* Ach., *Cyphelium subsimile* (Nyl.) Trevis., *Cyphelium tympanellum* (Ach.) Ach., *Lichen inquinans* Sm., *Trachylia inquinans* (Sm.) Rabenh., *Trachylia neesii* (Flot.) Rabenh., *Trachylia subsimilis* Nyl., *Trachylia tympanella* (Ach.) Fr.

L – Subs.: xyl, cor – Alt.: 2–4 – Note: a temperate to southern boreal-montane, circumpolar lichen found on old conifer stumps, more rarely on lignum of broad-leaved deciduous trees (especially *Quercus* and *Castanea*), and on wooden fence-posts, with optimum in upland areas. – **Au**: T, S, K, St, O, N. **Ge**: OB, Schw. **Sw**: BE, GR, LU, SZ, UR, VD, VS. **Fr**: AMa, HSav. **It**: Ven, TAA, Lomb, Piem.

***Acolium karelicum* (Vain.) M. Prieto & Wedin**

Syn.: *Cyphelium karelicum* (Vain.) Räsänen, Cyphelium lucidum (Th. Fr.) Th. Fr. var. karelicum Vain.

L – Subs.: cor, xyl – Alt.: 3–4 – Note: a mainly cool-temperate to southern boreal-montane lichen found on ancient boles of conifers in semi-natural forests, often on basal parts of trunks, mostly on old *Abies*, much more rarely on lignum. – **Au**: V, T, S, K, St, O, N. **Ge**: Ge. **Sw**: BE, SZ, UW, VS. **Fr**: AHP, AMa. **It**: TAA.

***Acolium sessile* (Pers.) Arnold**

Syn.: *Cyphelium sessile* (Pers.) Trevis., *Calicium sessile* Pers.

L – Subs.: xyl-par – Alt.: 3–4 – Note: a species with a grey thallus forming insular patches on the thalli of epiphytic *Pertusaria*-species (mainly *P. coccodes*), found on very old oaks; also known from North America, it is most common in Western Europe; records from the Alps have a fairly different ecology, and therefore need confirmation. – **Sw**: GR, UR. **It**: Lomb, Lig.

***Acrocordia cavata* (Ach.) R.C. Harris**

Syn.: *Arthopyrenia cavata* (Ach.) R.C. Harris, *Verrucaria cavata* Ach.

L – Subs.: cor – Alt.: 1–3 – Note: a mild-temperate, incompletely holarctic species found on smooth bark in humid deciduous forests; widespread but not common in the Alps. – **Au**: V, T, S, K, St, O, N. **Sw**: GL, GR, SZ, UW. **Fr**: Var, Vau. **It**: Frl, TAA.

**Acrocordia conoidea (Fr.) Körb. var. conoidea**

Syn.: *Arthopyrenia conoidea* (Fr.) Zahlbr., *Acrocordia epipolaea* (Borrer) A.L. Sm., *Verrucaria conoidea* Fr.

L – Subs.: cal – Alt.: 1–5 – Note: a mild-temperate species of compact limestone and dolomite, mostly in woodlands, on sheltered faces seldom wetted by rain, with optimum in submediterranean areas; widespread and locally common throughout the Alps. The forma carnea Arnold, with pale perithecia, has been reported from the Julian Pre-Alps. – **Au**: V, K, St, O, N. **Ge**: OB. **Sw**: BE, GR, LU, VD. **Fr**: AHP, AMa, Drô, Isè, HSav, Var, Vau. **It**: Frl, Ven, TAA, Lomb, Piem, VA, Lig. **Sl**: SlA, Tg.

**Acrocordia conoidea (Fr.) Körb. var. glacialis (Bagl. & Carestia) Vězda**

Syn.: *Acrocordia glacialis* Bagl. & Carestia

L # – Subs.: cal – Alt.: 5–6 – Note: a taxon with small spores, only known from the type locality in the Italian Alps where it was found on fissures of marble. – **It**: VA.

**Acrocordia conoidea (Fr.) Körb. var. suzae (Vězda) Vězda**

Syn.: Arthopyrenia conoidea (Fr.) Zahlbr. var. suzae Vězda

L # – Subs.: cal – Alt.: 3–4 – Note: differing from var. conoidea by the sessile perithecia, this taxon is based on a type from the Carpathian Mts.; its taxonomic value is uncertain and its distribution is poorly known. – **Fr**: AMa.

**Acrocordia gemmata (Ach.) A. Massal. var. gemmata**

Syn.: *Acrocordia alba* (Schrad.) B. de Lesd., *Acrocordia sphaeroides* (Wallr.) Arnold, *Arthopyrenia alba* (Schrad.) Zahlbr., *Arthopyrenia gemmata* (Ach.) A. Massal., *Arthopyrenia sphaeroides* (Wallr.) Zahlbr., Arthopyrenia tersa *auct. non* Körb., *Lichen gemmatus* Ach., *Verrucaria gemmata* (Ach.) Ach.

L – Subs.: cor – Alt.: 1–3 – Note: a mild-temperate species found on rough bark of mature broad-leaved trees (both deciduous and evergreen) in open woodlands; widespread throughout the Alps. – **Au**: V, T, S, K, St, O, N, B. **Ge**: OB. **Sw**: BE, FR, LU, SZ, TI, VD. **Fr**: AMa, Isè, HSav, Var, Vau. **It**: Frl, Ven, TAA, Lomb, Piem, Lig. **Sl**: SlA, Tg.

**Acrocordia gemmata (Ach.) A. Massal. var. rhododendri Hinteregger**

L – Subs.: cor – Alt.: 4 – Note: a recently-described variety found on shrubs, mostly in the subalpine belt, to be looked for throughout the Alps. – **Sl**: SlA.

***Acrocordia macrospora* A. Massal.**

Syn.: Acrocordia conoidea (Fr.) Körb. var. macrospora (A. Massal.) B. de Lesd., *Arthopyrenia macrospora* (A. Massal.) J. Steiner

L – Subs.: sil – Alt.: 1–3 – Note: an apparently Mediterranean-Atlantic, mild-temperate species (from Macaronesia to Norway), found on base-rich or weakly calciferous siliceous rocks in sheltered situations; certainly rare in the Alps. – **Au**: V. **Sw**: SZ. **Fr**: Var. **It**: Ven, Lomb. **Sl**: Tg.

***Acrocordia salweyi* (Leight. *ex* Nyl.) A.L. Sm.**

Syn.: *Arthopyrenia salweyi* (Leight. *ex* Nyl.) Zahlbr., *Verrucaria salweyi* (Leight. *ex* Nyl.) Leight. *ex* Cromb.

L – Subs.: ter-sil – Alt.: 1–2 – Note: an apparently Mediterranean-Atlantic, mild-temperate species (from Macaronesia to Norway), found on soft calcareous substrata (mortar, calciferous sandstone) in warm-humid areas; certainly very rare in the Alps. – **Fr**: AMa. **It**: TAA, Lig.

***Acrocordia scotophora* A. Massal.**

L # – Subs.: cor – Alt.: 2 – Note: a species with an effuse, white, farinose thallus and finally semi-immersed perithecia, persistent interascal filaments, cylindrical, 8-spored asci, and 1-septate ascospores with rounded ends, arranged in a single row; on the bark of deciduous trees; only recorded from Northern Italy and in urgent need of critical re-evaluation (frequently considered as a synonym of *Anisomeridium biforme*, but perhaps closely related to, or a synonym of *A. gemmata*). – **It**: Ven.

***Acrocordia subglobosa* (Vězda) Poelt & Vězda**

Syn.: *Arthopyrenia subglobosa* Vězda

L # – Subs.: cal – Alt.: 3 – Note: this species is characterised by sessile ascomata, but contrary to A. conoidea var. suzae, has a basally closed (entire) involucrellum; the type material is from the Sudety Mts., and the distribution is poorly known. – **Fr**: AMa.

***Adelolecia kolaensis* (Nyl.) Hertel & Rambold**

Syn.: *Catillaria tavastiana* H. Magn., *Lecidea conferenda* Nyl., *Lecidea dolosula* (Nyl.) Vain., *Lecidea kolaensis* Nyl., *Lecidea migratoria* Lynge, *Lecidea umbratilis* (Arnold) Th. Fr., *Lecidella umbratilis* Arnold

L – Subs.: sil – Alt.: 4–5 – Note: an arctic-alpine, probably circumpolar species of basic to weakly calciferous siliceous rocks in exposed situations, with optimum above treeline. – **Au**: ?V, T, K, St, N. **Ge**: OB. **Sw**: BE. **Fr**: AMa, Isè, Sav. **It**: TAA, Piem, VA.

***Adelolecia pilati* (Hepp) Hertel & Hafellner**

Syn.: *Biatora pilati* Hepp, *Buellia modicula* (Nyl.) Dalla Torre & Sarnth., Lecidea auriculata Th. Fr. var. hardangeriana Vain., *Lecidea chrysotheicha* Nyl., *Lecidea lyngeana* Zahlbr., *Lecidea modicula* Nyl., *Lecidea pilati* (Hepp) Körb., *Lecidea proludens* Nyl., *Lecidea subauriculata* Lynge *nom.illeg. non* B. de Lesd., *Lecidea tirolica* Vain., *Lecidella botryosa* Hepp *ex* Arnold, *Lecidella proludens* (Nyl.) Arnold

L – Subs.: sil, met – Alt.: 3–6 – Note: an arctic-alpine, circumpolar species of steeply inclined to underhanging surfaces of weathered, metal-rich metamorphic rocks seldom wetted by rain, from the subalpine to the nival belt; widespread in the Alps and also occurring in the high Mediterranean mountains. – **Au**: V, T, S, K, St, O, N. **Sw**: BE, GR, LU, TI, UW, VS. **Fr**: AMa, HSav. **It**: Frl, Ven, TAA, Lomb, Piem, VA, Lig.

***Adelolecia rhododendrina* (Nyl.) Printzen *ex* Hafellner & Türk**

Syn.: *Lecidea rhododendrina* Nyl.

L – Subs.: cor – Alt.: 3–4 – Note: on twigs of subalpine shrubs, especially *Rhododendron ferrugineum*; probably more common and widespread throughout the Alps. – **Au**: V, T, S, K, St. **It**: Frl.

***Agonimia allobata* (Stizenb.) P. James**

Syn.: *Amphoroblastia allobata* (Stizenb.) Servít, *Polyblastia allobata* (Stizenb.) Zschacke, *Verrucaria allobata* Stizenb.

L – Subs.: cor, bry – Alt.: 2–3 – Note: a mild-temperate species with subtropical affinities, found on ancient deciduous trees, in crevices or amongst mosses, in undisturbed forests or in deep gorges; widespread but usually rare in the Alps. – **Au**: S, K, O, N. **Sw**: GR, SZ, TI, UW, VS. **Fr**: AHP, AMa, Var, Vau. **It**: Ven.

***Agonimia borysthenica* Dymytrova, Breuss & S.Y. Kondr.**

L – Subs.: cor – Alt.: 2–3 – Note: a species of the *A. allobata*-group (asci 8-spored) with thallus consisting of distinct subglobose granules recalling those of *A. vouauxii* (with 2-spored asci) and larger ascospores; the type is from the Ukraine, and the distribution is poorly known. – **Sw**: TI.

***Agonimia bryophilopsis* (Vain.) Hafellner**

Syn.: *Polyblastia bryophilopsis* Vain.

L # – Subs.: deb, bry, int – Alt.: 4–5 – Note: a species said to be similar to *Polyblastia nigrata* (a heterotypic synonym of *A. gelatinosa*), but thallus whitish-grey and ascomata subglobose and only basally immersed, the 8-spored asci with non-pigmented muriform ascospores; overgrowing mosses and plant debris on calcareous soil; apparently rare or not recognised in the Alps. – **Au**: T, S, St.

***Agonimia gelatinosa* (Ach.) M. Brand & Diederich**

Syn.: *Endocarpon gelatinosum* (Ach.) Müll. Arg., *Polyblastia caliginosa* Norman, *Polyblastia gelatinosa* (Ach.) Th. Fr., *Polyblastia nigrata* Nyl., *Verrucaria gelatinosa* Ach., *Verrucaria nigrata* Nyl.

L – Subs.: deb, bry – Alt.: 3–5 – Note: a rather inconspicuous species growing on plant debris and mosses in dry calcareous grasslands, with optimum near treeline; widespread in the Alps. – **Au**: ?V, T, S, K, St, O, N. **Ge**: OB, Schw. **Sw**: BE, LU, SZ, VD, VS. **Fr**: AHP, AMa, Vau. **It**: Frl, TAA. **Sl**: SlA.

***Agonimia globulifera* M. Brand & Diederich**

L – Subs.: ter-cal – Alt.: 2–4 – Note: a species growing on soil, plant debris and mosses in dry calcareous grasslands, mostly below the subalpine belt. The sterile, glossy black globules are diagnostic, while ascomata are rare and dull black; the total distribution is poorly known. – **Au**: St, O. **Sw**: LU.

***Agonimia octospora* Coppins & P. James**

L – Subs.: cor – Alt.: 2 – Note: a mild-temperate species with subtropical affinities, found on basal parts of old broad-leaved trees, on bark or amongst mosses in rather open, humid woodlands, with several records from the Western Alps (France). – **Fr**: AHP, AMa, Isè, Var, Vau.

***Agonimia opuntiella* (Buschardt & Poelt) Vězda**

Syn.: *Phaeophyscia opuntiella* (Buschardt & Poelt) Hafellner, *Physcia opuntiella* Buschardt & Poelt

L – Subs.: bry, ter-cal, cor, deb – Alt.: 1–3 – Note: a mild-temperate species found on terricolous mosses and plant debris over calcareous substrata, sometimes amongst mosses on basal parts of old trees; widespread, but only locally rather common in the Alps. – **Au**: T, K, St, N. **Sw**: LU, TI, VS. **Fr**: AMa, Var, Vau. **It**: Frl, TAA, Lomb.

***Agonimia tristicula* (Nyl.) Zahlbr.**

Syn.: *Polyblastia tristicula* (Nyl.) Arnold, *Sporodictyon tristiculum* (Nyl.) Dalla Torre & Sarnth., *Verrucaria tristicula* Nyl.

L – Subs.: bry-cal, deb, ter-cal, cor – Alt.: 1–5 – Note: a probably holarctic species with a wide altitudinal and latitudinal range, found on terricolous mosses, but also, albeit rarely, on basal parts of old trunks in calcareous areas; widespread and often common throughout the Alps. – **Au**: V, T, S, K, St, O, N. **Ge**: OB, Schw. **Sw**: BE, GL, GR, LU, SG, SZ, TI, UR, UW, VS. **Fr**: AHP, AMa, Drô, Sav, HSav, Var, Vau. **It**: Frl, Ven, TAA, Lomb, Piem, Lig. **Sl**: SlA. **Li**.

***Agonimia vouauxii* (B. de Lesd.) M. Brand & P. Diederich**

Syn.: *Polyblastia vouauxii* B. de Lesd.

L – Subs.: bry, deb, ter-cal, cal, bry-cal, xyl – Alt.: 3–5 – Note: a taxon described from maritime Northern France, where it colonises organic waste like paper, leather, etc.; elsewhere it was reported from soil rich in calcium in open vegetation types; the distribution in the Alps is poorly known, and records from high altitudes need verification. – **Au**: V, St, O. **Sw**: BE. **Fr**: AHP.

***Ainoa geochroa* (Körb.) Lumbsch & I. Schmitt**

Syn.: *Biatora geochroa* Körb., *Lecidea geochroa* (Körb.) Lettau, *Trapelia geochroa* (Körb.) Hertel

L – Subs.: ter-sil – Alt.: 3–5 – Note: on fresh acidic mineral soil in open vegetation developing in areas with a long snow cover (*Solorinion croceae*-communities); distribution in the Alps poorly known, perhaps overlooked. – **Au**: V, T, S, K, St. **Sw**: UR, VS.

***Ainoa mooreana* (Carroll) Lumbsch & I. Schmitt**

Syn.: *Biatora brujeriana* (Schaer. *ex* D. Dietr.) Arnold, *Biatora lopadioides* Th. Fr., *Biatora torellii* Anzi, *Lecidea brujeriana* (Schaer. *ex* D. Dietr.) Leight., *Lecidea lopadioides* (Th. Fr.) Grummann, *Lecidea mooreana* Carroll, *Lecidea oblita* Bagl. & Carestia, *Lecidea torellii* (Anzi) Nyl., *Trapelia mooreana* (Carroll) P. James, *Trapelia torellii* (Anzi) Hertel

L – Subs.: sil – Alt.: 3–5 – Note: a circumboreal-montane early coloniser of weathered siliceous rocks, also known from the Southern Hemisphere, mostly found on pebbles, or on large boulders near the soil surface in rather disturbed habitats (*e.g.* on track sides, in clearings of light forests, etc.), with optimum near treeline. – **Au**: V, T, S, K, St. **Sw**: SZ, TI. **Fr**: HSav. **It**: Frl, Ven, TAA, Lomb, Piem, VA. **Sl**: SlA.

***Alectoria nigricans* (Ach.) Nyl.**

Syn.: *Alectoria thulensis* (Th. Fr.) Nyl., Cornicularia ochroleuca (Hoffm.) DC. var. nigricans Ach., *Gowardia nigricans* (Ach.) Halonen, Myllys, Velmala & Hyvärinen

L – Subs.: ter-sil, deb-sil – Alt.: 4–6 – Note: an arctic-alpine, circumpolar species found on ground or on rocks in wind-exposed siliceous ridges in moss-lichen heaths; widespread in the siliceous Alps. – **Au**: V, T, S, K, St, N. **Sw**: BE, GR, SZ, TI, UR, VS. **Fr**: HAl, Isè, Sav, HSav. **It**: Frl, Ven, TAA, Lomb, Piem, VA.

***Alectoria ochroleuca* (Hoffm.) A. Massal.**

Syn.: *Bryopogon ochroleucu*s (Hoffm.) Link, *Cornicularia ochroleuca* (Hoffm.) DC., *Usnea ochroleuca* Hoffm.

L – Subs.: ter-sil, bry, deb, cor – Alt.: 3–6 – Note: an arctic-alpine, circumpolar species found on windy ridges in moss-lichens heaths, more frequent on siliceous substrata, but sometimes also occurring in areas with dolomite; widespread throughout the Alps. – **Au**: V, T, S, K, St, O, N. **Ge**: OB, Schw. **Sw**: BE, GR, TI, UR, VD, VS. **Fr**: AHP, HAl, Isè, Sav, HSav. **It**: Frl, Ven, TAA, Lomb, Piem, VA.

***Alectoria sarmentosa* (Ach.) Ach.**

Syn.: *Alectoria cincinnata* (Fr.) Lynge, *Alectoria luteola* Mont. *ex* De Not., Alectoria ochroleuca (Hoffm.) A. Massal. var. sarmentosa (Ach.) Nyl., Alectoria sarmentosa (Ach.) Ach. subsp. vexillifera (Nyl.) D. Hawksw., *Alectoria vexillifera* (Nyl.) Stizenb., *Lichen sarmentosus* Ach.

L – Subs.: cor, xyl – Alt.: 3–4 – Note: a cool-temperate to boreal-montane, probably circumpolar species found on branches, more rarely on trunks of (mainly) conifers in forests with frequent fog; in the Alps it was probably more common in the past, presently certainly declining, being very sensitive to forest management. – **Au**: T, S, K, St, O, N. **Ge**: OB. **Sw**: BE, GL, GR, LU, SZ, UR, VD, VS. **Fr**: HAl, Isè, Sav, HSav. **It**: Frl, Ven, TAA, Lomb, Piem, VA. **Sl**: SlA.

***Alectoria variegata* (Samp.) Tav.**

Syn.: Alectoria dichotoma var. variegata Samp., Alectoria ochroleuca var. variegata (Samp.) Zahlbr.

L # – Subs.: ter-sil – Alt.: 5 – Note: a species of doubtful taxonomic value, differing from other Alectorias of the *A. ochroleuca*-group mainly in the conspicuous violet-black patches on the thallus surface; described from siliceous boulders in the montane belt in Portugal and hardly recorded from elsewhere, except one terricolous finding in the alpine belt of the Western Alps (France). – **Fr**: HAl.

***Allantoparmelia alpicola* (Th. Fr.) Essl.**

Syn.: *Hypogymnia alpicola* (Th. Fr.) Hav., *Parmelia alpicola* Th. Fr., *Parmelia jinretleni* Gyeln., *Parmelia nigrita* (Flot.) Hillmann

L – Subs.: sil – Alt.: 5–6 – Note: an arctic-alpine, circumpolar species found on hard siliceous rocks, often on quartz, in wind-exposed ridges near or above treeline; probably ranging throughout the siliceous Alps. – **Au**: V, T, S, K, St, N. **Sw**: BE, GR, UR, VS. **Fr**: Isè, HSav. **It**: TAA, Lomb.

***Allocetraria madreporiformis* (Ach.) Kärnefelt & A. Thell**

Syn.: *Dactylina madreporiformis* (Ach.) Tuck., *Dufourea madreporiformis* Ach., *Evernia madreporiformis* (Ach.) Fr.

L – Subs.: ter-cal, deb – Alt.: 4–5 – Note: an arctic-alpine species found in open grasslands and in wind-exposed ridges near and above treeline; widespread throughout the Alps. – **Au**: V, T, S, K, St, O. **Ge**: OB. **Sw**: BE, FR, GR, VD, VS. **It**: Frl, TAA, Lomb, Piem, VA. **Sl**: SlA.

***Alyxoria culmigena* (Lib.) Ertz**

Syn.: *Opegrapha atrorimalis* Nyl., *Opegrapha betulina* Sm. *non* Pers., *Opegrapha culmigena* Lib., *Opegrapha herbarum* Mont., *Opegrapha turneri* Leight., Opegrapha varia Pers. var. herbarum (Mont.) Källsten

L – Subs.: cor, deb – Alt.: 1–3 – Note: usually on bark of broad-leaved trees, but also on coarse debris like stems of larger herbs; mostly at low altitudes in areas with mild winters. – **Au**: O, N. **Fr**: AMa, Isè, Var, Vau. **It**: ?TAA, Piem. **Sl**: SlA.

***Alyxoria mougeotii* (A. Massal.) Ertz, Frisch & G. Thor**

Syn.: *Opegrapha leightonii* Cromb. *ex* Nyl., *Opegrapha mougeotii* A. Massal.

L # – Subs.: cal – Alt.: 1–3 – Note: on steeply inclined surfaces of calcareous or base-rich siliceous substrata (limestone, calcareous sandstone, roofing tiles), in areas with mild winters, below the montane belt; related to *A. varia*. – **Au**: S, O, B. **Sw**: SZ. **Fr**: AHP, AMa, Vau. **It**: Ven, Piem.

***Alyxoria ochrocheila* (Nyl.) Ertz & Tehler**

Syn.: *Opegrapha atricolor* Stirt., *Opegrapha ochrocheila* Nyl., *Opegrapha rubescens* Sandst.

L – Subs.: cor, xyl – Alt.: 2–3 – Note: a Mediterranean-Atlantic species found on on the smooth bark of (mostly) evergreen broad-leaved trees and shrubs, more rarely on lignum; in the Alps it is very rare and restricted to areas with mild winters. The orange pruina on the exciple is diagnostic. – **Sw**: SZ. **Fr**: AMa. **Sl**: Tg.

***Alyxoria varia* (Pers.) Ertz & Tehler**

Syn.: *Alyxoria diaphora* (Ach.) Gray, *Alyxoria notha* (Ach.) Gray, *Opegrapha chlorina* Pers., *Opegrapha cymbiformis* Flörke, *Opegrapha diaphora* (Ach.) Ach., *Opegrapha lichenoides* Pers., *Opegrapha notha* Ach., *Opegrapha pulicaris* *auct.* *p.p. non* Pers. *ex* Fr., *Opegrapha rimalis* Pers., *Opegrapha varia* Pers., Opegrapha varia Pers. var. fagicola A. Massal., *Opegrapha vulvella* Ach.

L – Subs.: cor, xyl – Alt.: 1–4 – Note: a mainly temperate lichen found on old trees in humid but rather open forests, occasionally on basic siliceous rocks in humid and shaded situations. The delimitation of this species is still an open problem: here it is still treated as a collective taxon. – **Au**: V, T, S, K, St, O, N. **Ge**: OB. **Sw**: BE, FR, GL, GR, LU, SG, SZ, TI, UR, UW, VD, VS. **Fr**: AHP, HAl, AMa, Drô, Isè, Sav, HSav, Var, Vau. **It**: Frl, Ven, TAA, Lomb, Piem, VA, Lig. **Sl**: SlA.

***Alyxoria variiformis* (Anzi) Ertz**

Syn.: *Opegrapha variiformis* Anzi

L – Subs.: cal – Alt.: 2 – Note: a mild-temperate to Mediterranean-Atlantic species found on steeply inclined faces of calciferous rocks near the coast, in rather shaded and humid situations, with a few records from the base of the Western Alps (France). – **Fr**: AHP, Var, Vau.

***Amandinea cacuminum* (Th. Fr.) H. Mayrhofer & Sheard**

Syn.: *Rinodina cacuminum* (Th. Fr.) Malme *non* (A. Massal.) Anzi, Rinodina sophodes (Ach.) A. Massal. var. milvina (Wahlenb.) Th. Fr. f. cacuminum Th. Fr.

L – Subs.: sil – Alt.: 5 – Note: on large boulders frequently visited by birds; described from Scandinavia, and also reported from the Eastern Alps (Austria). – **Au**: St.

***Amandinea oleicola* (Nyl.) Giralt & van den Boom**

L – Subs.: cor – Alt.: 1 – Note: a recently resurrected epiphytic species known from Tuscany, Portugal and the Canary Islands, also reported from the base of the Western Alps. – **Fr**: Var.

***Amandinea pelidna* (Ach.) Fryday & Arcadia**

Syn.: *Amandinea lecideina* (H. Mayrhofer & Poelt) Scheid. & H. Mayrhofer, *Biatora pelidna* (Ach.) Rabenh., Lecidea lygaea Ach. f. pelidna (Ach.) Ach., *Lecidea pelidna* Ach., *Rinodina lecideina* H. Mayrhofer & Poelt

L # – Subs.: sil – Alt.: 4–5 – Note: this species differs from *A. punctata* in the median spore wall thickening, the rimose thallus, and perhaps in the frequent presence of pycnidia. The typical form is apparently a coloniser of siliceous boulders and outcrops near the seashore (type from a lowland locality in Ireland), and the identity of records from the Alps, treated as “orophilous ecotype” by Roux et al. (2017), requires further critical study. – **Au**: Au. **Fr**: AHP. **It**: TAA.

***Amandinea punctata* (Hoffm.) Coppins & Scheid.**

Syn.: *Buellia cupreola* Müll. Arg., *Buellia myriocarpa* (DC.) De Not., *Buellia punctata* (Hoffm.) A. Massal., *Buellia punctiformis* (Hoffm.) A. Massal., *Buellia stigmatea* (Schaer.) Körb., *Karschia thallophila* (Ohlert) Rehm, *Lecidea myriocarpa* (DC.) Röhl., *Lecidea punctata* (Hoffm.) Flörke, *Patellaria myriocarpa* DC., *Verrucaria punctata* Hoffm.

L # – Subs.: cor, xyl, sil, deb, bry, ter-sil – Alt.: 1–4 – Note: a very poorly understood taxon; in its present circumscription, an almost cosmopolitan lichen found on a wide variety of substrata, including bark, lignum, siliceous rocks, roofing tiles and brick; heterogeneous, and in need of revision. – **Au**: V, T, S, K, St, O, N, B. **Ge**: OB, Schw. **Sw**: BE, FR, GR, LU, SG, SZ, TI, VD, VS. **Fr**: AHP, HAl, AMa, Drô, Isè, Sav, HSav, Var, Vau. **It**: Frl, Ven, TAA, Lomb, Piem, VA, Lig. **Sl**: SlA, Tg. **Li**.

***Amygdalaria consentiens* (Nyl.) Hertel, Brodo & May. Inoue**

Syn.: *Lecidea consentiens* Nyl.

L – Subs.: sil – Alt.: 5 – Note: a probably circum-arctic species, peculiar in having sunken apothecia and lacking soralia; very rare in the Alps. – **Au**: T. **Ge**: OB, Schw.

***Amygdalaria panaeola* (Ach.) Hertel & Brodo**

Syn.: *Huilia panaeola* (Ach.) Hertel, *Lecidea panaeola* Ach., *Psora panaeola* (Ach.) Anzi

L – Subs.: sil – Alt.: 3–5 – Note: an arctic-alpine to boreal-montane, incompletely circumpolar species of weathered, mineral-rich siliceous rocks close to the ground, in areas with late snow cover, with optimum above treeline. – **Au**: T, St. **Sw**: BE, UR, VS. **It**: TAA, Lomb, Piem, VA.

***Amylora cervinocuprea* (Arnold) Rambold**

Syn.: *Aspicilia cervinocuprea* Arnold, Aspicilia olivacea Bagl. & Carestia f. cervinocuprea (Arnold) Arnold, *Lecanora cervinocuprea* (Arnold) Mig.

L – Subs.: sil – Alt.: 5–6 – Note: on vertical to overhanging faces of gneissic rocks; perhaps more widespread in the Alps, but not common. – **Au**: T, K. **Sw**: GR, VS. **It**: TAA.

***Anaptychia bryorum* Poelt**

Syn.: Anaptychia fusca (Huds.) Vain. var. stippaea *auct.*, Anaptychia stippaea *auct.*

L – Subs.: ter-cal, bry-cal, deb – Alt.: 4–5 – Note: an arctic-alpine to boreal-montane, probably circumpolar species found amongst mosses and moribund plants over base-rich siliceous substrata; widespread almost throughout the Alps. – **Au**: V, T, S, K, St. **Ge**: OB. **Sw**: BE, GL, GR, SZ, UR, VS. **Fr**: HAl, AMa. **It**: Frl, Ven, TAA, Lomb, Piem, VA.

***Anaptychia ciliaris* (L.) Flot.**

Syn.: *Anaptychia melanosticta* (Ach.) Trass, *Borrera ciliaris* (L.) Ach., *Borrera solenaria* Duby, *Hagenia ciliaris* (L.) W. Mann, *Lichen ciliaris* L., *Parmelia ciliaris* (L.) Ach., *Physcia ciliaris* (L.) DC.

L – Subs.: cor, cal, ter-cal – Alt.: 2–4 – Note: a temperate species found on bark of more or less isolated trees, sometimes also on rock and amongst terricolous mosses in open situations; widespread throughout the Alps, but probably declining. See also note on *A. crinalis*. – **Au**: V, T, S, K, St, O, N, B. **Ge**: OB, Schw. **Sw**: BE, GL, GR, LU, SG, SZ, UR, VD, VS. **Fr**: AHP, HAl, AMa, Drô, Isè, Sav, HSav, Var, Vau. **It**: Frl, Ven, TAA, Lomb, Piem, VA, Lig. **Sl**: SlA, Tg. **Li**.

***Anaptychia crinalis* (Schleich.) Vězda *ex* J. Nowak**

Syn.: Anaptychia ciliaris (L.) Körb. var. crinalis (Schleich.) Rabenh., Physcia ciliaris (L.) DC. var. crinalis Schleich.

L # – Subs.: cor – Alt.: 3 – Note: perhaps just a simple forma of *A. ciliaris* (intermediate morphs are common), confined to humid beech forests. – **Au**: T, K, N. **Sw**: BE, GR, VD, VS. **Fr**: Isè, Sav, HSav. **It**: Frl, Ven, TAA, Lomb, Piem.

***Anaptychia runcinata* (With.) J.R. Laundon**

Syn.: *Anaptychia aquila* (Ach.) A. Massal., *Anaptychia fusca* (Huds.) Vain., *Lichen runcinatus* With., *Physcia fusca* (Huds.) A.L. Sm.

L – Subs.: sil – Alt.: 1–3 – Note: a Mediterranean-Atlantic, European species found on hard siliceous boulders, sometimes overgrowing epilithic mosses, with a few records from the base of the Western Alps. – **Fr**: Vau. **It**: Lig.

***Anaptychia ulotricoides* (Vain.) Vain.**

Syn.: *Physcia ulotricoides* Vain.

L – Subs.: sil – Alt.: 4 – Note: a species described from the inner-Asian steppe zone, where it grows on various substrates including bark and rock; in the Alps it is certainly not common, being perhaps restricted to dry valleys. – **Fr**: AHP.

***Anema decipiens* (A. Massal.) Forssell**

Syn.: *Collema decipiens* (A. Massal.) Nyl., *Omphalaria decipiens* A. Massal., *Thyrea decipiens* (A. Massal.) A. Massal.

L – Subs.: cal, int – Alt.: 1–4 – Note: on steeply inclined, sunny surfaces of calcareous rocks (mainly limestone, but also calciferous schists and sandstone) with periodical water seepage after rain, below the alpine belt. – **Au**: V, T, S, O. **Ge**: OB, Schw. **Sw**: GR, VS. **Fr**: AHP, AMa, Var, Vau. **It**: Ven, TAA, Lomb, Piem.

***Anema moedlingense* Zahlbr.**

L – Subs.: cal – Alt.: 1–2 – Note: the suberect, deeply sulcate squamules with a reticulate surface are diagnostic; on sunny calcareous rocks with periodical water seepage; often confused with *A. nummularium*, and certainly more widespread in the Alps. – **Fr**: AMa. **It**: Frl.

***Anema nummularium* (Dufour *ex* Durieu & Mont.) Nyl. *ex* Forssell**

Syn.: *Anema notarisii* (A. Massal.) Forssell, *Anema nummulariellum* Nyl., *Collema nummularium* Dufour *ex* Durieu & Mont., *Omphalaria frustillata* Nyl., *Omphalaria notarisii* A. Massal., *Thyrea frustillata* (Nyl.) Zahlbr.

L – Subs.: cal – Alt.: 2–4 – Note: on steeply inclined surfaces of limestone and dolomite with periodical water seepage after rain, below the subalpine belt. See also note on *A. moedlingense*. – **Au**: K, T, St, N. **Ge**: OB, Schw. **Sw**: SZ, VD, VS. **Fr**: AHP, HAl, AMa, Drô, Var, Vau. **It**: Frl, Ven, TAA, Lomb, Piem, VA, Lig.

***Anema prodigulum* (Nyl.) Henssen**

Syn.: *Omphalaria prodigula* Nyl., *Thyrea prodigula* (Nyl.) Zahlbr.

L – Subs.: cal – Alt.: 1–2 – Note: on sunny seepage tracks of calcareous rocks, mostly below the montane belt. – **Fr**: AHP, AMa. **It**: Frl.

***Anema suffruticosum* P.P. Moreno & Egea**

L – Subs.: cal – Alt.: 1–2 – Note: on sunny seepage tracks of calcareous, more rarely of base-rich siliceous rocks; certainly not common, but probably more widespread in the Alps. – **Fr**: AMa, Var. **It**: TAA.

***Anema tumidulum* Henssen *ex* P.M. Jørg., M. Schultz & Guttová**

L – Subs.: cal – Alt.: 2–3 – Note: this species seems to be fairly common in Central Europe. It grows on steeply inclined, sunny surfaces of calcareous or basic siliceous rocks with periodical water seepage after rain, with optimum in upland areas. – **Au**: ?N. **Sw**: GR, TI, VS. **Fr**: AHP, AMa. **It**: Frl, Lomb, VA.

***Anisomeridium biforme* (Schaer.) R.C. Harris**

Syn.: *Acrocordia biformis* (Schaer.) Arnold, *Acrocordia polycarpa* (Körb.) Körb., *Arthopyrenia biformis* (Schaer.) A. Massal., *Arthopyrenia byssacea* (Taylor) A.L. Sm., *Arthopyrenia conformis* (Nyl.) Müll. Arg., *Arthopyrenia tersa* Körb. *non auct.*, *Ditremis biformis* (Schaer.) R.C. Harris, *Epicymathia thallophila* (Cooke) Sacc., *Leiophloea biformis* (Schaer.) Trevis., *Verrucaria biformis* Schaer., *Verrucaria conformis* Nyl.

L – Subs.: cor, xyl – Alt.: 1–3 – Note: a mild-temperate, probably holarctic species found on deciduous trees in open and humid woodlands, *e.g.* along creeks and rivers on *Fraxinus*, *Populus* and *Salix*, sometimes also on oaks. – **Au**: S, K, O. **Ge**: OB, Schw. **Sw**: BE, FR, GR, LU. **Fr**: AHP, AMa, Isè, Var. **It**: Frl, Ven, Lomb, Piem.

***Anisomeridium carintiacum* (J. Steiner) R.C. Harris**

Syn.: *Arthopyrenia carintiaca* J. Steiner, *Paraphysothele carintiaca* (J. Steiner) Keissl.

L # – Subs.: sil-aqu – Alt.: 2–3 – Note: in the study area only known from the type locality in Austria. – **Au**: K.

***Anisomeridium macrocarpum* (Körb.) V. Wirth**

Syn.: *Acrocordia macrocarpa* Körb., *Arthopyrenia macrocarpa* (Körb.) Zahlbr.

L – Subs.: cor, xyl – Alt.: 2–3 – Note: a mainly Central European lichen also known from Northern Spain, with a poorly developed, mainly endosubstratic thallus; the large 1-septate ascospores (30–45 × 7–10 μm) are diagnostic; on the trunks of broad-leaved deciduous trees in woodlands, mostly near the base of the boles, or on roots. – **Au**: V, T, S, K, O. **Ge**: OB. **Sw**: BE, GL, GR, SZ, VD, VS. **It**: Ven.

***Anisomeridium polypori* (Ellis & Everh.) M.E. Barr**

Syn.: *Anisomeridium juistense* (Erichsen) R.C. Harris, *Anisomeridium nyssaegenum* (Ellis & Everh.) R.C. Harris, *Anisomeridium willeyanum* (R.C. Harris) R.C. Harris, *Apiospora polypori* Ellis & Everh., *Apiosporella polypori* (Ellis & Everh.) Höhnel, *Arthopyrenia willeyana* R.C. Harris, *Didymella polypori* (Ellis & Everh.) Ellis & Everh., *Ditremis nyssaegena* (Ellis & Everh.) R.C. Harris, *Melanopsamma corticola* Ellis & Everh., *Mycosphaerella hepaticarum* (Pat.) Petrak, *Sarcinulella banksiae* B. Sutton & Alcorn, *Stigmatea hepaticarum* Pat., *Thelidium juistense* Erichsen, *Zygonella nyssaegenum* Ellis & Everh.

L – Subs.: cor – Alt.: 1–3 – Note: a mainly mild-temperate, perhaps holarctic species, mainly found on bark along rivers and brooks; overlooked for a long time, but certainly widespread and locally common in the Alps. – **Au**: V, T, S, K, St, O, N. **Ge**: OB, Schw. **Sw**: GL, GR, SZ, TI, UR, UW, VS. **Fr**: AMa, Drô, Isè, Var, Vau. **It**: Frl, Ven, TAA, Lomb, Piem. **Sl**: SlA.

***Anisomeridium ranunculosporum* (Coppins & P. James) Coppins**

Syn.: *Arthopyrenia ranunculospora* Coppins & P. James

L – Subs.: cor – Alt.: 2–3 – Note: this species is peculiar in the shape of the one-septate ascospores with the lower cell much longer than the upper one; thalli with macroconidiomata only are difficult to identify; on smooth bark in old-growth broad-leaved forests; perhaps overlooked but certainly rare in the Alps, more common in extra-Alpine Europe. – **Au**: S.

***Anisomeridium viridescens* (Coppins) R.C. Harris**

Syn.: *Arthopyrenia viridescens* Coppins

L – Subs.: cor – Alt.: 2–3 – Note: the brown, persistent involucrellum reacting K+ green, as well as the strongly branched interascal filaments are diagnostic; usually on smooth bark in woodlands with a long ecological continuity; the total distribution is incompletely known. – **Au**: St.

**Anzina carneonivea (Anzi) Scheid. var. carneonivea**

Syn.: *Caloplaca carneonivea* (Anzi) Jatta, *Diphratora carneonivea* (Anzi) Jatta, *Gyalecta carneonivea* (Anzi) Lettau, *Gyalolechia carneonivea* Anzi, *Lecidea carneonivea* (Anzi) Nyl., *Pertusaria carneonivea* (Anzi) Vain., *Pertusaria infralapponica* Vain., *Pertusaria tauriscorum* Zahlbr., *Secoliga carneonivea* (Anzi) Arnold, *Varicellaria carneonivea* (Anzi) Erichsen

L – Subs.: xyl, deb, ter, bry, cor – Alt.: 3–5 – Note: on acidic substrata such as bark – especially of conifers – wood, plant debris, moribund bryophytes, in the understory of upper montane moist forest, and among shrubs, with optimum in the subalpine belt; widespread, but in some areas of the Alps still overlooked. – **Au**: V, T, S, K, St, O, N. **Ge**: OB. **Sw**: BE, GL, GR, SZ, TI, VD, VS. **Fr**: HSav. **It**: Frl, Ven, TAA, Lomb.

**Anzina carneonivea (Anzi) Scheid. var. tetraspora Scheid.**

L # – Subs.: cor – Alt.: 3–5 – Note: the taxonomic value of this variety is unclear; the ecology is as in the type variety. – **Au**: V, T, S, K, St. **Sw**: GR, TI.

***Aphanopsis coenosa* (Ach.) Coppins & P. James**

Syn.: *Collema coenosum* Ach., *Lecidea humigena* Taylor, *Lecidea praecox* Vězda

L – Subs.: ter-sil – Alt.: 2–3 – Note: on humid, bare, clayey or fine-grained sandy soil on track sides or ditch margins in woodlands; easy to overlook and perhaps more widespread in the Alps, but certainly not common. – **Au**: St. **Sw**: VS. **It**: Lomb.

***Arctoparmelia centrifuga* (L.) Hale**

Syn.: *Lichen centrifugus* L., *Parmelia centrifuga* (L.) Ach., *Xanthoparmelia centrifuga* (L.) Hale

L – Subs.: sil – Alt.: 3–5 – Note: an arctic-alpine species of exposed siliceous rocks; certainly very rare in the Alps. Italian records need confirmation (see Nimis 2016). – **Au**: T, St, ?N. **It**: ?Ven, ?Piem.

***Arctoparmelia incurva* (Pers.) Hale**

Syn.: *Lichen incurvus* Pers., *Parmelia incurva* (Pers.) Fr., *Parmelia multifida* *auct.*, *Xanthoparmelia incurva* (Pers.) Hale

L – Subs.: sil – Alt.: 3–5 – Note: a circumpolar, arctic-alpine to boreal-montane species found on steeply inclined, hard, acid siliceous rocks in cold, wind-exposed mountain summits and boulder fields; rare in the Alps, and distribution insufficiently documented. – **Au**: T, S, K, St. **Sw**: VS. **Fr**: HSav. **It**: Piem.

***Arthonia apatetica* (A. Massal.) Th. Fr.**

Syn.: *Allarthonia apatetica* (A. Massal.) Lettau, *Arthonia exilis* *auct.*, *Arthonia rugulosa* (Kremp.) Almq., *Catillaria apatetica* A. Massal., *Coniangium apateticum* (A. Massal.) A. Massal.

L – Subs.: cor-par, xyl – Alt.: 2–3 – Note: a mainly temperate species found on trunks and young twigs of deciduous trees in sheltered situations, with optimum in the submediterranean belt. See also note on *A. tenellula*. – **Au**: V, T, S, K, St, O. **Ge**: OB. **Sw**: VS. **Fr**: AHP, AMa, HSav, Var, Vau. **It**: Frl, Ven, TAA, Lomb, Piem. **Sl**: SlA.

***Arthonia arthonioides* (Ach.) A.L. Sm.**

Syn.: *Arthonia aspersa* Leight., *Arthonia lecideoides* Th. Fr., *Arthonia trachylioides* Nyl., *Arthonia xylophila* V. Wirth & P. James, *Trachylia arthonioides* (Ach.) Fr., *Lecidea arthonioides* Ach.

L – Subs.: cor – Alt.: 1–2 – Note: a southern species, known from Europe and North America, found on acidic rocks and exposed roots in dry underhangs, also on dry undersides of trees in sheltered, humid situations, such as in forests; from the Alps there are a few scattered records only. – **Au**: O. **It**: Lomb.

***Arthonia atra* (Pers.) A. Schneid.**

Syn.: *Opegrapha atra* Pers., *Opegrapha denigrata* Ach., *Opegrapha fuliginosa* Pers. *ex* Ach., *Opegrapha salicina* A. Massal., *Opegrapha stenocarpa* Ach.

L – Subs.: cor – Alt.: 1–3 – Note: mainly on smooth bark of deciduous trees, widespread and often common throughout the Alps, below the subalpine belt. – **Au**: V, T, S, K, St, O, N, B. **Ge**: OB, Schw. **Sw**: BE, LU, SZ, TI, VD, VS. **Fr**: AHP, AMa, Drô, Isè, Sav, HSav, Var, Vau. **It**: Frl, Ven, TAA, Lomb, Piem, VA, Lig. **Sl**: SlA, Tg.

***Arthonia caesiella* Nyl.**

Syn.: *Arthonia aphthoides* Flagey, *Arthonia aphthosa* Flagey, *Arthonia galactiformis* Flagey

L – Subs.: cor – Alt.: 2 – Note: a species with bluish grey-pruinose ascomata, based on a type from extra-Alpine Southern France. – **Fr**: Vau.

***Arthonia calcicola* Nyl.**

Syn.: *Allarthonia calcicola* (Nyl.) Redinger

L – Subs.: cal – Alt.: 1–2 – Note: an early coloniser of calcareous walls and mortar; overlooked and probably more common, especially in the eu-Mediterranean belt, and also present in the warm-dry valleys of the Alps. – **Au**: K, St. **It**: TAA.

***Arthonia cinereopruinosa* Schaer.**

Syn.: *Arthonia lilacina* (Ach.) Körb., *Arthonia pinicola* (Hepp) A. Massal., *Pyrenotheca stictica* Fr., *Trachylia cinereopruinosa* (Schaer.) A. Massal.

L – Subs.: cor – Alt.: 2–3 – Note: a mild-temperate species found on smooth bark of deciduous trees in dense humid forests. – **Au**: O, N. **Ge**: OB. **Sw**: BE, LU, VD. **Fr**: Vau. **It**: Frl, Ven, Lomb.

***Arthonia didyma* Körb.**

Syn.: *Arthonia aspersella* Leight., *Arthonia atrofuscella* Nyl., *Arthonia pineti* Körb., *Caldesia didyma* (Körb.) Trevis.

L – Subs.: cor – Alt.: 2–3 – Note: a cool-temperate species found on smooth bark in humid areas. – **Au**: V, T, S, K, St, O, N, B. **Ge**: OB, Schw. **Sw**: BE, GL, GR, SZ, TI, UR, UW, VD, VS. **Fr**: AMa, Drô, Isè, Var. **It**: Ven, TAA, Lomb, Piem. **Sl**: SlA, Tg.

***Arthonia dispersa* (Schrad.) Nyl.**

Syn.: *Arthonia epipasta* (Ach.) Körb., *Arthonia minutula* Nyl., *Opegrapha dispersa* Schrad., *Opegrapha epipasta* (Ach.) Ach.

L – Subs.: cor – Alt.: 1–3 – Note: a holarctic species found on smooth, nutrient-rich bark, *e.g.* of *Fraxinus*. It belongs to a difficult complex which still awaits elucidation. – **Au**: S, K, St, O, N, B. **Sw**: BE, GR, TI, VD. **Fr**: Drô, Var, Vau. **It**: Frl, Ven, TAA, Lomb, Piem, VA. **Sl**: SlA.

***Arthonia excipienda* (Nyl.) Leight.**

Syn.: Arthonia astroidea Ach. var. excipienda Nyl., Arthonia dispersa (Schrad.) Nyl. subsp. excipienda (Nyl.) Nyl., Arthonia dispersa (Schrad.) Nyl. var. excipienda (Nyl.) H. Olivier, *Arthonia hibernica* Nyl.

L – Subs.: cor – Alt.: 2–3 – Note: on smooth bark of deciduous trees and shrubs in riparian montane woodlands; probably overlooked or confused with *A. punctiformis*. – **Ge**: OB. **It**: TAA, Lig.

***Arthonia faginea* Müll. Arg.**

Syn.: *Allarthonia faginea* (Müll. Arg.) Redinger

L – Subs.: cor – Alt.: 3 – Note: the ascospores which are 2-septate when fully developed are diagnostic, recalling somewhat *A. radiata*, but with a coccoid photobiont; on smooth bark under suboceanic conditions; distribution insufficiently known. – **Au**: N. **Fr**: HSav.

***Arthonia fuliginosa* (Turner & Borrer) Flot.**

Syn.: *Spiloma fuliginosum* Turner & Borrer

L – Subs.: cor – Alt.: 2–3 – Note: a mild-temperate lichen growing on acid bark, especially of *Abies*, in humid montane forests. – **Au**: V, T, K, O, N. **Ge**: OB, Schw. **It**: Frl, TAA. **Sl**: SlA.

***Arthonia galactites* (DC.) Dufour**

Syn.: *Arthonia marginella* Dufour *ex* Nyl., Arthonia punctiformis Ach. var. galactina Ach., *Opegrapha galactites* (DC.) M. Choisy, *Verrucaria galactites* DC.

L – Subs.: cor – Alt.: 1–3 – Note: a mild-temperate species with optimum on the smooth bark of *Fraxinus ornus*, but also of *Populus* and even *Pistacia*; probably overlooked or/and confused with other species, but certainly not common in the Alps. – **Au**: O. **Sw**: TI. **Fr**: Isè. **It**: Frl, Ven, TAA, Lomb, Piem, Lig. **Sl**: SlA.

***Arthonia granitophila* Th. Fr.**

Syn.: *Melaspilea granitophila* (Th. Fr.) Coppins, *Melaspilea subarenacea* J. Nowak & Kiszka

L – Subs.: sil – Alt.: 2–3 – Note: a melaspileoid species with an indistinct thallus and black, somewhat elongated ascomata, peculiar in *Arthonia* by the carbonised exciple; on shaded siliceous rocks, with optimum in the montane belt; widespread in Europe but not common, with several records from the Eastern Alps only (Austria). – **Au**: V, T, S, K, St.

***Arthonia granosa* B. de Lesd.**

L – Subs.: cor – Alt.: 2 – Note: this species recalls in habitus *A. galactites*, but has much larger ascospores. It is has a Mediterranean-Atlantic distribution in coastal situations with humid maritime winds, generally on *Juniperus*, but also on *Olea* and *Quercus ilex*; in the study area it is rare and restricted to low altitudes in the Western Alps, near the Mediterranean coast, on the smooth bark of broad-leaved trees. – **Fr**: AMa.

***Arthonia incarnata* Th. Fr. *ex* Almq.**

L – Subs.: cor – Alt.: 4 – Note: almost exclusively on bark of young to middle-aged conifers (*Picea*, *Abies*); a typical species of boreal to temperate-montane regions; very rare in the Alps, most records being old. – **Au**: St.

***Arthonia lapidicola* (Taylor) Branth & Rostr.**

Syn.: *Allarthonia fusca* (A. Massal.) Sandst., *Allarthonia lapidicola* (Taylor) Zahlbr., *Arthonia fusca* (A. Massal.) Hepp, *Arthonia koerberi* (J. Lahm *ex* Arnold) Malbr., Arthonia vagans Almq. var. koerberi (J. Lahm *ex* Arnold) Almq., *Catillaria fusca* A. Massal., *Catillaria ooliticola* Walt. Watson, *Coniangium fuscum* (A. Massal.) A. Massal., *Coniangium lapidicola* (Taylor) Arnold, *Coniangium rupestre* Körb., *Lecidea lapidicola* Taylor

L – Subs.: cal, deb – Alt.: 2–5 – Note: a holarctic species of calcareous rocks and mortar, most frequent on pebbles, but also on walls, roofing tiles etc.; widespread throughout the Alps. – **Au**: V, T, S, K, St, O. **Ge**: OB, Schw. **Sw**: BE, GR, SZ, UR, VD. **Fr**: AHP, HAl, AMa, Drô, Isè, Sav, HSav, Var, Vau. **It**: Ven, TAA, Lomb, Piem.

***Arthonia ligniaria* Hellb.**

L – Subs.: cor – Alt.: 2 – Note: in external appearance this species recalls *A. lapidicola*, but the ascospores are much larger; on bark of mature broad-leaved trees; distribution insufficiently known. – **Sw**: LU.

***Arthonia lignariella* Coppins**

L – Subs.: cor-alg – Alt.: 4 – Note: in appearance and ascoma anatomy this species resembles *A. ligniaria*, but the hymenium is lower and the ascospores smaller; mostly on rotting wood of stumps, but also on bark; widespread in Western and Northern Europe, and also reported from the Swiss Alps. – **Sw**: SZ.

***Arthonia mediella* Nyl.**

Syn.: *Arthonia globulosiformis* (Hepp) Arnold, *Arthonia sordaria* Körb.

L – Subs.: cor – Alt.: 2–4 – Note: a cool-temperate to boreal-montane, probably circumpolar species, living as an early coloniser of acid bark, most often of conifers, found both in humid *Abies*-*Fagus* forests and in open *Larix* stands; widespread throughout the Alps. – **Au**: T, S, K, St, O. **Sw**: GL, GR, LU, SZ, TI, UR, VD, VS. **Fr**: HSav. **It**: TAA, Lomb, Piem, Lig. **Sl**: SlA.

***Arthonia patellulata* Nyl.**

Syn.: *Allarthonia patellulata* (Nyl.) Zahlbr., *Arthonia betuleti* Nyl., *Coniangium krempelhuberi* A. Massal.

L – Subs.: cor – Alt.: 3–4 – Note: a cool-temperate to boreal-montane, probably circumpolar species found on smooth bark, mostly of *Populus tremula*; from the Alps there are only scattered records. – **Au**: K. **Ge**: OB. **It**: Ven, Lomb, Piem.

***Arthonia radiata* (Pers.) Ach.**

Syn.: *Arthonia astroidea* Ach., *Arthonia epipastoides* Nyl., *Arthonia montellica* A. Massal., *Arthonia sorbina* Körb., *Arthonia swartziana* Ach., *Arthonia vulgaris* Schaer., *Lichen astroites* Ach., *Opegrapha astroidea* (Ach.) Ach., *Opegrapha radiata* Pers., *Opegrapha swartziana* (Ach.) Hepp

L – Subs.: cor – Alt.: 1–4 – Note: a mainly temperate, incompletely holarctic lichen, the only *Arthonia* found in non-natural habitats such as in settlements, parks, etc., even in moderately polluted situations, exceptionally reaching the subalpine belt; widespread throughout the Alps. – **Au**: V, T, S, K, St, O, N, B. **Ge**: OB, Schw. **Sw**: BE, FR, GL, GR, LU, SG, SZ, TI, UR, UW, VD, VS. **Fr**: AHP, AMa, Drô, Isè, Sav, HSav, Var, Vau. **It**: Frl, Ven, TAA, Lomb, Piem, VA, Lig. **Sl**: SlA, Tg. **Li**.

***Arthonia reniformis* (Pers.) Röhl.**

Syn.: *Arthonia gyrosa* Ach., *Naevia gyrosa* (Ach.) A. Massal., *Opegrapha reniformis* Pers.

L – Subs.: cor – Alt.: 2–3 – Note: a mild-temperate species of smooth bark, especially of *Carpinus*, more rarely of *Fagus* and *Corylus*, in humid deciduous woodlands; from the Alps there are only scattered records. – **Au**: K, St. **Sw**: SZ. **Fr**: Isè. **It**: Ven, TAA.

***Arthonia ruana* A. Massal.**

Syn.: *Arthonia anastomosans* (Ach.) Nyl., *Arthonia beltraminiana* (A. Massal.) Anzi, ?*Arthonia rosacea* Anzi, *Arthoniopsis ruana* (A. Massal.) Trevis., *Arthothelium anastomosans* (Ach.) Arnold, *Arthothelium beltraminianum* A. Massal., *Arthothelium dispersum* *auct.*, *Arthothelium ruanum* (A. Massal.) Körb., *Arthothelium rosaceum* (Anzi) Zahlbr., *Arthothelium ruanideum* (Nyl.) Arnold

L – Subs.: cor – Alt.: 2–3 – Note: a temperate, suboceanic species found on smooth bark of deciduous trees and shrubs (*e.g. Alnus*, *Fagus*, *Fraxinus*, *Corylus*, etc.) in humid forests, often on the basal parts of the trunks; widespread in the Alps, but generally not common. – **Au**: V, T, S, K, St, O, N, B. **Ge**: OB, Schw. **Fr**: AMa. **It**: Frl, Ven, TAA, Lomb, Piem. **Sl**: SlA.

**Arthonia spadicea Leight. var. spadicea**

Syn.: *Arthonia lurida* Ach. *non auct.*, Arthonia lurida Ach. var. spadicea (Leight.) Nyl., *Coniangium spadiceum* (Leight.) Arnold

L – Subs.: cor, xyl – Alt.: 2–3 – Note: a mainly temperate lichen found on smooth bark, more rarely on wood, in humid forests; widespread in the Alps, but generally not common. – **Au**: V, T, S, K, St, O, N. **Ge**: OB, Schw. **Sw**: BE, GL, GR, SZ, TI, UW, VD, VS. **Fr**: AMa, Isè, Var. **It**: Ven, TAA, Lomb, Piem, Lig.

**Arthonia spadicea Leight. var. subspadicea (Nyl.) Redinger**

Syn.: *Arthonia subspadicea* Nyl.

L # – Subs.: cor – Alt.: 3 – Note: the external appearance is like var. spadicea, but the hymenium in section is colourless to slightly yellowish and not reacting with K; the ecology as well resembles that of var. spadicea; the taxonomic value of this variety is in need of evaluation. – **Au**: V, T.

***Arthonia stellaris* Kremp.**

Syn.: *Arthonia armoricana* Leight.

L – Subs.: cor – Alt.: 2–3 – Note: a mild-temperate lichen found on smooth bark, *e.g.* of *Corylus*, and in *Abies*-*Fagus* forests. – **Au**: V, S, O. **Ge**: OB. **Sw**: BE. **Fr**: AMa, Drô, Var.

***Arthonia subastroidea* Anzi**

Syn.: *Arthothelium subastroideum* (Anzi) Rehm

L – Subs.: cor – Alt.: 3–4 – Note: a cool-temperate to boreal-montane early coloniser of bark, *e.g.* of *Pinus cembra* and *Fagus* in the Alps; perhaps non-lichenised. – **It**: Lomb.

***Arthonia tenellula* Nyl.**

Syn.: *Allarthonia tenellula* (Nyl.) B. de Lesd., *Arthonia horaria* Norman

L # – Subs.: xyl – Alt.: 2–3 – Note: differing from *A. apatetica* mainly in the hypophloeodic thallus; the taxonomic value of this species is in need of re-evaluation: it was described from a site near the sea shore in western France; specimens from the Alps are in need of verification, and the distribution is insufficiently known. – **Au**: T, K, St.

***Arthonia trifurcata* (Hepp *ex* Müll. Arg.) Cl. Roux**

Syn.: *Opegrapha trifurcata* Hepp *ex* Müll. Arg.

L – Subs.: cal – Alt.: 2–4 – Note: this species has been often confused with *A. calcarea*, so that its distribution is still poorly known. The thallus is thinner and greenish-white and the apothecia are smaller than in *A. calcarea*, which also has a different ecology, being a littoral species; the arthonioid asci and similarities with *A. atra* were interestingly already noticed by Müller Argoviensis; the type is from the Jura Massif in France. It grows on calcareous rocks in rather sheltered situations below the montane belt, and sometimes it starts the life-cycle on other crustose lichens. – **Au**: T, S, St. **Ge**: OB, Schw. **Fr**: AHP, AMa, Sav, HSav, Var, Vau. **It**: Ven.

***Arthonia viburnea* Müll. Arg.**

L # – Subs.: cor – Alt.: 2–3 – Note: a species with minute, hemispherical to subglobose, black ascomata and 5-septate ascospores (15–18 × 4.5–6 μm); apparently reported only from the type locality in France, where it was found on branches of *Viburnum lantana*. – **Fr**: HSav.

***Arthonia vinosa* Leight.**

Syn.: Arthonia lurida *auct. non* Ach., Coniangium luridum *auct. non* (Ach.) Fr., *Coniangium vinosum* (Leight.) A. Massal., *Coniangium vulgare* Fr.

L – Subs.: cor, xyl – Alt.: 2–3 – Note: a mild-temperate species found near the base of old trees, more rarely on lignum, in very humid and closed-canopied forests; related to *A. spadicea*; widespread in the Alps, but generally not common. – **Au**: T, S, K, St, O, N. **Ge**: OB, Schw. **Sw**: BE, GR, SZ, VD, VS. **Fr**: Isè. **It**: Frl, Ven, TAA. **Sl**: SlA, Tg.

***Arthopyrenia arnoldii* Zahlbr.**

L # – Subs.: cor – Alt.: 3 – Note: a species with a thin, epiphloeodic, continuous, whitish thallus and minute, hemispherical to depressed ascomata (to 0.25 mm in diam) which are finally black and glossy, thin distinct branching interascal filaments, 4 – to 8-spored asci (to 65 × 15 µm), 1-septate, ellipsoid to oblong ascospores with both cells of about equal size, surrounded by a perisporal sheath (14–18 × 5–8 µm), and bacillary pycnoconidia (to 4 µm long); based on a type from Italy where it was found on branches of *Larix*. – **Au**: St. **It**: TAA.

***Arthothelium lirellans* (Almq.) Coppins**

Syn.: *Arthonia lirellans* Almq.

L # – Subs.: cor – Alt.: 2–3 – Note: on smooth bark in woodlands with a long ecological continuity, in sites with an oceanic climate; distribution still insufficiently known, perhaps sometimes mistaken for *Arthonia punctiformis*. – **Au**: K, St.

***Arthothelium spectabile* A. Massal.**

Syn.: *Arthonia difformis* Nyl.

L – Subs.: cor – Alt.: 2–3 – Note: a temperate-suboceanic lichen found on the smooth bark of deciduous trees in ancient forests; from the Alps there are only a few scattered records. – **Au**: St, O, N. **Ge**: Schw. **Fr**: AMa. **It**: Ven, Lomb.

***Arthrorhaphis alpina* (Schaer.) R. Sant.**

Syn.: Arthrorhaphis citrinella (Ach.) Poelt var. alpina (Schaer.) Poelt, *Bacidia alpina* (Schaer.) Vain., Bacidia citrinella (Ach.) Branth & Rostr. subsp. alpina (Schaer.) J.R. Laundon, *Bacidia flavovirescens* (Turner & Borrer *ex* Schaer.) Anzi var. alpina (Schaer.) A.L. Sm., *Lecidea flavovirescens* Turner & Borrer *ex* Schaer. var. alpina Schaer.

L – Subs.: ter-sil, ter-sil-par, ter-cal, ter-cal-par, bry – Alt.: 4–6 – Note: an arctic-alpine, circumpolar species found on weakly calciferous soil rich in humus, first parasymbiotic on *Baeomyces*, later an autonomous lichen; widespread throughout the Alps. – **Au**: V, T, S, K, St, O, N. **Ge**: OB, Schw. **Sw**: BE, GR, SG, ?SZ, TI, UR, VD, VS. **Fr**: HAl, AMa, Sav, HSav. **It**: Frl, TAA, Lomb, Piem, VA.

***Arthrorhaphis citrinella* (Ach.) Poelt**

Syn.: *Arthrorhaphis flavovirescens* (Turner & Borrer *ex* Schaer.) Th. Fr., *Bacidia citrinella* (Ach.) Branth & Rostr., *Bacidia flavovirescens* (Turner & Borrer *ex* Schaer.) Anzi, *Bacidia flavovirescens* (Turner & Borrer *ex* Schaer.) Anzi var. citrinella (Ach.) Vain., *Lecanactis citrinella* (Ach.) H. Olivier, *Lecidea citrinella* (Ach.) Ach., *Lecidea flavovirescens* Turner & Borrer *ex* Schaer., *Lichen citrinellus* Ach., *Lichen flavovirescens* Dicks. *nom.illeg. non* Wulfen, *Mycobacidia flavovirescens* (Turner & Borrer *ex* Schaer.) Rehm, *Raphiospora flavovirescens* (Turner & Borrer *ex* Schaer.) A. Massal., *Scoliciosporum flavovirescens* (Turner & Borrer *ex* Schaer.) Jatta, *Skolekites citrinellus* (Ach.) Norman

L – Subs.: ter-sil, sil-par, bry – Alt.: 3–5 – Note: an arctic-alpine, circumpolar species found on mosses and soil rich in humus in sheltered situations, older thalli are lichenised, younger ones are lichenicolous on *Baeomyces*; widespread throughout the Alps. – **Au**: V, T, S, K, St, N. **Ge**: OB, Schw. **Sw**: BE, GR, LU, SZ, TI, UR, VD, VS. **Fr**: AHP, HAl, AMa, Sav, HSav. **It**: Frl, Ven, TAA, Lomb, Piem, VA. **Sl**: SlA.

***Arthrorhaphis vacillans* Th. Fr. & Almq. *ex* Th. Fr.**

Syn.: *Arthrorhaphis anziana* (Lynge) Poelt, *Bacidia anziana* Lynge, *Bacidia vacillans* (Th. Fr. & Almq. *ex* Th. Fr.) Rostr.

L – Subs.: ter-sil, ter-sil-par, ter-cal, ter-cal-par – Alt.: 4–6 – Note: an arctic-alpine, circumpolar species found in humid soil near and above treeline. It starts the life-cycle as a parasite of *Baeomyces placophyllus*, later becoming autotrophic, and is the most calcium-tolerant among the *Arthrorhaphis*-species, often occurring over calcareous schists and even marmor (Obermayer *in litt.*). – **Au**: T, S, K, St. **Ge**: OB. **Sw**: SG, ?SZ. **It**: TAA.

***Arthrosporum populorum* A. Massal.**

Syn.: *Arthrosporum accline* (Flot.) A. Massal., *Bacidia acclinis* (Flot.) Zahlbr., *Bacidia populorum* (A. Massal.) Trevis., *Bilimbia acclinis* (Flot.) Trevis., *Bilimbia populorum* (A. Massal.) Vain., *Lecidea acclinis* Flot.

L – Subs.: cor – Alt.: 1–3 – Note: a mild-temperate lichen found on smooth bark of deciduous trees and shrubs, especially *Fraxinus*, *Populus* and *Salix*; widespread in the Alps, but probably declining. – **Au**: T, S, K, St, N. **Sw**: GR, VS. **Fr**: AMa, HSav, Var, Vau. **It**: Frl, Ven, TAA, Lomb, Piem, Lig.

***Aspicilia adaequata* (Lettau) Poelt**

Syn.: *Lecanora adequata* Lettau

L # – Subs.: cal, int – Alt.: 4–6 – Note: a species recalling *A. candida*, but marginal lobes less distinct and apothecia with black, non-pruinose discs; the distribution in the Alps is still insufficiently known. – **Au**: V, T, St.

***Aspicilia aquatica* (Fr.) Körb.**

Syn.: *Aspicilia eluta* (Nyl.) Hue, *Aspicilia flageyi* Hue, *Lecanora amphibola* *sensu* Vain., *Lecanora aquatica* (Fr.) Hepp, *Lecanora flageyi* (Hue) Zahlbr., *Lecanora mazarina* (Wahlenb.) H. Magn., *Lecanora rivulorum* H. Magn., Parmelia cinerea (L.) Hepp var. aquatica Fr.

L – Subs.: sil-aqu – Alt.: 2–5 – Note: a probably holarctic species of periodically submerged rocks and boulders along streams; widespread throughout the siliceous Alps. See also note on *A. proluta*. – **Au**: V, T, S, K, St. **Sw**: BE, GR, TI, VS. **Fr**: AHP, HAl, AMa, HSav, Var. **It**: TAA, Lomb, Piem, Lig. **Sl**: SlA.

***Aspicilia bricconensis* Hue**

Syn.: *Lecanora bricconensis* (Hue) Zahlbr.

L # – Subs.: sil – Alt.: 3–5 – Note: a chemically variable species of siliceous rocks, reported from scattered localities in Alps. – **Au**: T, S, St. **Sw**: TI. **Fr**: AHP, AMa. **It**: TAA.

***Aspicilia bunodea* (A. Massal.) Maheu & A. Gillet**

Syn.: *Lecanora bunodea* (A. Massal.) Jatta, *Pachyospora bunodea* A. Massal.

L # – Subs.: sil, int – Alt.: 2–3 – Note: most probably related to *A. contorta* (see Roux et al. 2014). – **It**: Ven.

***Aspicilia cacuminum* (Müll. Arg.) Kernst.**

Syn.: *Lecanora cacuminum* Müll. Arg.

L # – Subs.: cal, int – Alt.: 5–6 – Note: a high-alpine species with subeffigurate thallus margins, whose taxonomic value is in need of re-evaluation; the distribution is still insufficiently known. – **Au**: T. **Sw**: VD.

***Aspicilia caesiocinerea* (Nyl. *ex* Malbr.) Arnold**

Syn.: Aspicilia gibbosa *auct. non* (Ach.) Körb, *Aspicilia rolleana* Hue, *Circinaria caesiocinerea* (Nyl. *ex* Malbr.) A. Nordin, Savić & Tibell, *Lecanora caesiocinerea* Nyl. *ex* Malbr., *Lecanora rolleana* (Hue) Zahlbr.

L – Subs.: sil, int – Alt.: 1–5 – Note: on siliceous rocks wetted by rain, with a wide altitudinal range. Very heterogeneous both morphologically and ecologically, and in need of revision; widespread throughout the Alps. – **Au**: V, T, S, K, St, N, B. **Ge**: OB. **Sw**: BE, GR, SZ, TI, UR, UW, VD, VS. **Fr**: AHP, HAl, AMa, Sav, HSav, Var, Vau. **It**: Frl, Ven, TAA, Lomb, Piem, VA, Lig. **Sl**: SlA. **Li**.

***Aspicilia calcarea* (L.) Bagl.**

Syn.: *Aspicilia lundensis* (Fr.) Uloth, *Circinaria calcarea* (L.) A. Nordin, Savić & Tibell, *Lecanora calcarea* (L.) Sommerf., *Lecanora lundensis* (Fr.) Zahlbr., *Lecidea calcarea* (L.) Schaer., *Lichen calcareus* L., *Pachyospora calcarea* (L.) A. Massal., *Parmelia calcarea* (L.) Michx., *Patellaria calcarea* (L.) Trevis., *Urceolaria calcarea* (L.) Ach., *Verrucaria calcarea* (L.) Humb.

L – Subs.: cal – Alt.: 1–4 – Note: a mainly Mediterranean to mild-temperate species found on limestone and dolomite, sometimes also on other calciferous substrata; absent only from large conurbations, sometimes reaching beyond treeline; widespread and common throughout the Alps. – **Au**: V, T, S, K, St, O, N, B. **Ge**: OB, Schw. **Sw**: GR, LU, SZ, TI, VD, VS. **Fr**: AHP, HAl, AMa, Drô, Isè, Sav, HSav, Var, Vau. **It**: Frl, Ven, TAA, Lomb, Piem, VA, Lig. **Sl**: Tg.

***Aspicilia calcitrapa* Cl. Roux & A. Nordin**

L – Subs.: sil – Alt.: 2 – Note: on siliceous rocks in sunny places at low elevations, where it forms a community together with *Pertusaria chiodectonoides*; most common in the Pyrenees, with a few records from the SW Alps. – **Fr**: AHP, AMa.

***Aspicilia candida* (Anzi) Hue**

Syn.: Aspicilia candida (Anzi) Hue var. flavoreagens Asta & Cl. Roux [invalidly published, ICN Art. 40.1 + 8], Aspicilia polychroma Anzi var. candida Anzi, *Aspicilia rosacea* Hue, *Lecanora candida* (Anzi) Nyl., *Lecanora rosacea* (Hue) Zahlbr.

L – Subs.: cal, int, sil – Alt.: 3–6 – Note: known from Europe and North America, this lichen occurs in the Alps on weakly calciferous rocks, especially calcareous schists, mostly near or above treeline. The species is chemically variable (see *e.g.* Roux et al. 2014); widespread in the Alps, wherever suitable substrata are present. – **Au**: V, T, S, K, St, N. **Ge**: OB, Schw. **Sw**: BE, GR, UW, VS. **Fr**: AHP, HAl, AMa, Isè, Sav, HSav, Vau. **It**: Frl, TAA, Lomb, Piem, VA, Lig.

***Aspicilia capituligera* (Poelt) Poelt**

Syn.: *Lecanora capituligera* Poelt

L – Subs.: sil, sil-aqu, int – Alt.: 5–6 – Note: a lichen with distinctly elongated marginal lobes and cone-shaped papillae producing terminally soredia-like diaspores; optimum in the spray-zone of alpine streams; the distribution is still insufficiently known. – **Au**: T. **Sw**: GR.

***Aspicilia cinerea* (L.) Körb.**

Syn.: *Aspicilia depressa* (Ach.) Anzi, *Lecanora cinerea* (L.) Sommerf., *Lecanora excipularis* H. Magn. *nomen sed non planta*, *Lichen cinereus* L., *Parmelia cinerea* (L.) Hepp, *Sagedia depressa* Ach., *Urceolaria cinerea* (L.) Ach.

L – Subs.: sil, int, cor – Alt.: 2–5 – Note: on acid to basic siliceous rocks wetted by rain. Taken in the broadest sense, this is a holarctic and probably bipolar, extremely variable lichen, widespread from subtropical to arctic areas. Material from the Alps should be also compared with *A. calcitrapa* Cl. Roux & Nordin, with which the species has been frequently confused (see Roux et al. 2014). In the Alps it has been often confused with other *Aspicilia*-species (*A. bricconensis*, *A. prestensis*, *A. spermatomanes*, see Roux et al. 2014). – **Au**: V, T, S, K, St, O, N, B. **Ge**: OB. **Sw**: BE, GR, LU, ?SZ, TI, UR, VD, VS. **Fr**: AHP, HAl, AMa, Isè, Sav, HSav, Vau. **It**: Frl, Ven, TAA, Lomb, Piem, VA, Lig. **Sl**: SlA.

***Aspicilia contorta* (Hoffm.) Körb.**

Syn.: Aspicilia calcarea (L.) Bagl. var. contorta (Hoffm.) Körb., *Circinaria contorta* (Hoffm.) A. Nordin, Savić & Tibell, Lecanora calcarea (L.) Sommerf. var. contorta (Flörke) Hepp, *Lecanora contorta* (Hoffm.) J. Steiner, Pachyospora calcarea A. Massal. var. contorta (Hoffm.) A. Massal., *Parmelia contorta* (Hoffm.) Spreng. *non* Bory, *Verrucaria contorta* Hoffm.

L – Subs.: cal, int – Alt.: 1–5 – Note: less frequent than *A. hoffmanniana*, and generally bound to less disturbed situations, but widespread throughout the Alps. – **Au**: V, T, S, K, St, O, N, B. **Ge**: OB, Schw. **Sw**: BE, GR, LU, SZ, TI, VD, VS. **Fr**: AHP, HAl, AMa, Drô, Isè, Sav, HSav, Var, Vau. **It**: Frl, Ven, TAA, Lomb, Piem, VA, Lig. **Sl**: SlA. **Li**.

***Aspicilia corallophora* (Poelt) Hafellner & Türk**

Syn.: *Lecanora corallophora* Poelt

L – Subs.: sil-aqu – Alt.: 5 – Note: a lichen with a non-effigurate, coarsely areolate thallus and soralia developing from verrucose areoles, the soredia transforming into isidia; on periodically submerged rocks and boulders along Alpine streams; the distribution is still insufficiently known. – **Au**: T.

***Aspicilia coronata* (A. Massal.) B. de Lesd.**

Syn.: Aspicilia calcarea (L.) Bagl. var. coronata (A. Massal.) Körb., *Aspicilia laurensii* B. de Lesd., *Lecanora coronata* (A. Massal.) Jatta, *Lecanora laurensii* (B. de Lesd.) Croz., *Pachyospora coronata* A. Massal.

L – Subs.: cal – Alt.: 2–5 – Note: most common on hard calcareous rocks and sometimes on dolomite, mostly in upland areas; this taxon is probably heterogeneous. – **Au**: V, T, K, St. **Ge**: OB. **Sw**: LU, VS. **Fr**: AHP, HAl, AMa, Drô, Isè, Sav, HSav, Var, Vau. **It**: Ven.

***Aspicilia cupreoglauca* B. de Lesd.**

Syn.: *Lecanora lacunosa* Zschacke *non* Mereschk.

L – Subs.: sil – Alt.: 2 – Note: a mild-temperate to Mediterranean lichen found on base-rich siliceous rocks wetted by rain, mostly on sunny, horizontal surfaces, with a few records from the Western Alps. – **Fr**: AMa, Var, Vau.

***Aspicilia cupreogrisea* (Th. Fr.) Hue**

Syn.: *Circinaria cupreogrisea* (Th. Fr.) A. Nordin, Savić & Tibell, *Lecanora cupreogrisea* Th. Fr.

L – Subs.: sil – Alt.: 2–5 – Note: on siliceous rocks in the mountains; perhaps more widespread in the Alps. – **Au**: ?V, S. **Fr**: AHP, HAl, AMa, HSav, Vau. **It**: VA.

***Aspicilia delimitata* (H. Magn.) *ined.* (provisionally placed here, ICN Art. 36.1b)**

Syn.: *Lecanora delimitata* H. Magn., *Lecidea rustrelensis* B. de Lesd.

L – Subs.: sil – Alt.: 2 – Note: According to Roux et al. (2014, 2017) *A. rustrelensis* is identical to *A. delimitata*. – **Fr**: Vau.

***Aspicilia elmorei* (E.D. Rudolph) *ined.* (provisionally placed here, ICN Art. 36.1b)**

Syn.: Aspicilia desertorum *auct. p.p. non* (Kremp.) Mereschk., Aspicilia esculenta *auct. p.p. non* (Pall.) Flagey, *Circinaria elmorei* (E.D. Rudolph) Owe-Larss., A. Nordin & Sohrabi, *Lecanora elmorei* E.D. Rudolph

L # – Subs.: cal – Alt.: 4–5 – Note: a xeric subtropical lichen of steeply inclined, hard, more or less calciferous rocks and dolomite. The taxonomy of this group is still unsettled: saxicolous crustose forms formerly called *Aspicilia desertorum* belong to the *Circinaria elmorei*-complex, which is presently under revision (Sohrabi, *in litt*.). – **Fr**: AHP, HAl, AMa.

***Aspicilia fimbriata* (H. Magn.) Oxner**

Syn.: *Lecanora fimbriata* H. Magn.

L # – Subs.: sil – Alt.: 4 – Note: a fertile species of a brownish-grey colour with very narrow marginal lobes, reacting K+ red; based on a type from Siberia; the conspecificity of populations from the Alps is uncertain. – **Au**: K, St.

***Aspicilia fumosula* (Müll. Arg.) Hue**

Syn.: *Lecanora fumosula* Müll. Arg.

L # – Subs.: sax – Alt.: 5 – Note: a species recalling in habitus *Immersaria athroocarpa*, but perhaps related to *A. cupreogrisea*, with an areolate, brown to brown-black thallus on a black hypothallus, apothecia (*c.* 0.5 mm in diam.) immersed in the thallus, usually 1 per areole, with a blackish-brown disc, 8-spored asci, and oblong to ellipsoid ascospores (*c.* 10 × 4–5 μm); on siliceous rocks in the high-alpine belt, only known from the type locality in the Western Alps (Switzerland). – **Sw**: VS.

***Aspicilia gibbosa* (Ach.) Körb.**

Syn.: *Circinaria gibbosa* (Ach.) A. Nordin, Savić & Tibell, *Lecanora gibbosa* (Ach.) Nyl., *Lecanora gibbosula* H. Magn., *Urceolaria gibbosa* Ach.

L – Subs.: sil – Alt.: 3–5 – Note: this species is known with certainty only from Northern Europe and records from elsewhere require confirmation; the epithet “*gibbosa*” was frequently used by European authors for *A. caesiocinerea*. – **Au**: V, S, K, St, N. **It**: TAA, Lomb, Piem.

***Aspicilia glomerulans* (Poelt) Poelt**

Syn.: *Lecanora glomerulans* Poelt

L # – Subs.: sil-aqu – Alt.: 5 – Note: a usually sterile species with coarse, partly branched isidia, found on siliceous boulders along streams at high altitudes; the distribution is still insufficiently known. – **Au**: T.

***Aspicilia goettweigensis* (Zahlbr.) Hue**

Syn.: *Lecanora goettweigensis* Zahlbr.

L – Subs.: sil – Alt.: 2–3 – Note: a lowland species of sunny siliceous rocks, belonging to the *A. gibbosa*-group, with a dark grey thallus and a K+ yellow medulla; the original spelling (“*göttweigensis*“) was corrected following ICN 60.6. – **Au**: N. **Sw**: VS.

***Aspicilia grisea* Arnold**

Syn.: *Aspicilia insolata* (H. Magn.) Hav., *Lecanora grisea* (Arnold) Lettau *non* Ach., *Lecanora griseolans* Zahlbr., *Lecanora insolata* H. Magn.

L – Subs.: sil, int, cor – Alt.: 3–6 – Note: a chemically variable species (see Roux et al. 2014), found on siliceous rocks, sometimes also on pebbles; certainly more widespread in the Alps, but very much overlooked. – **Au**: V, T, S, K, St, N. **Sw**: GR, VS. **Fr**: Sav, HSav. **It**: Frl. **Sl**: SlA.

***Aspicilia helvetica* Hue**

Syn.: *Lecanora helvetica* (Hue) Zahlbr.

L # – Subs.: sil – Alt.: 4 – Note: a species with a reddish-white, rather thick, areolate thallus (not showing any reaction), apothecia (0.4–0.8 mm in diam.) immersed in the thallus, usually 1 per areole, hymenium exceeding 150 μm in height, large 8-spored asci, and broadly ellipsoid ascospores (15–24 × 12–16 μm); on siliceous rocks (granite); only known from the type locality in the Eastern Alps (Switzerland). – **Sw**: GR.

***Aspicilia henrici* B. de Lesd.**

L # – Subs.: sil – Alt.: 2–5 – Note: a taxon described from Aosta Valley (Italy). It is similar to *A. valpellinensis*, but K-. – **Sw**: BE, GR, VS. **It**: VA.

***Aspicilia hispida* Mereschk.**

Syn.: *Agrestia cyphellata* J.W. Thomson, *Agrestia hispida* (Mereschk.) Hale & W.L. Culb., *Circinaria hispida* (Mereschk.) A. Nordin, Savić & Tibell, *Lecanora hispida* (Mereschk.) Zahlbr., *Sphaerothallia hispida* (Mereschk.) Follmann & A. Crespo

L – Subs.: ter-cal – Alt.: 4–5 – Note: a species of the steppes of Central Asia, with a disjunct distribution in the most continental parts of the Iberian Peninsula, in the mountains of Greece, and in the Western Alps. – **Fr**: AMa. **It**: Piem.

***Aspicilia hoffmanniana* (S. Ekman & Fröberg *ex* R. Sant.) Cl. Roux & M. Bertrand**

Syn.: *Aspicilia caesioalba* (Le Prévost *ex* Duby) Hue, Aspicilia contorta (Hoffm.) Körb. subsp. hoffmanniana S. Ekman & Fröberg, Aspicilia hoffmannii *auct. non* (Ach.) Flagey, Lecanora calcarea (L.) Sommerf. var. hoffmannii (Ach.) Sommerf., *Lecanora hoffmannii* (Ach.) Müll. Arg.

L – Subs.: cal – Alt.: 1–4 – Note: an early coloniser of a wide variety of calciferous or base-rich substrata, from limestone and dolomite to brick, roofing tiles and mortar walls; widespread throughout the Alps, mostly below treeline. – **Au**: V, T, K, St, O, N, B. **Sw**: SZ. **Fr**: AHP, HAl, AMa, Drô, Isè, Sav, HSav, Var, Vau. **It**: Frl, Ven, TAA, Lomb, Piem, VA, Lig.

***Aspicilia inornata* Arnold**

Syn.: *Lecanora inornata* (Arnold) Zahlbr.

L – Subs.: sil, sil-aqu – Alt.: 3–5 – Note: this species, which has been frequently confused with *A. viridescens*, grows on periodically submerged schistose rocks along brooks, mostly in the mountains. – **Au**: V, T, K. **Sw**: GR. **It**: Ven, TAA, VA.

***Aspicilia intermutans* (Nyl.) Arnold**

Syn.: *Aspicilia ammotropha* Hue, *Aspicilia trachytica* Flagey *non* (A. Massal.) Arnold, *Aspiciliella intermutans* (Nyl.) M. Choisy, *Lecanora ammotropha* (Hue) Zahlbr., *Lecanora intermutans* Nyl.

L – Subs.: sil – Alt.: 1–2 – Note: on more or less base-rich siliceous rocks wetted by rain. This is one of the most frequent silicicolous *Aspicilia* of Mediterranean Europe; most of the records from the Alps, especially those from high altitudes, are likely to refer to *A. cinerea*, *A. prestensis*, *A. spermatomanes*, and *Aspilidea myrinii*. – **Au**: ?V, ?St. **Sw**: ?GR, ?VS. **Fr**: AHP, AMa, Isè, Var, Vau. **It**: Ven, TAA, Lig.

***Aspicilia laevata* (Ach.) Arnold**

Syn.: Aspicilia cinerea (L.) Körb. var. laevata (Ach.) Körb., Aspicilia gibbosa (Ach.) Körb. var. laevata (Ach.) Stein, *Aspicilia lusca* (Nyl.) B. de Lesd., *Aspicilia sylvatica* Arnold, *Aspicilia vitrea* Anzi, *Lecanora distinguenda* Zahlbr., Lecanora gibbosa var. laevata (Ach.) Th. Fr., *Lecanora laevata* (Ach.) Nyl., *Lecanora lusca* Nyl., *Lecanora sylvatica* (Arnold) Sandst., *Sagedia laevata* Ach.

L – Subs.: sil, sil-aqu – Alt.: 2–5 – Note: a mainly boreal-montane, circumpolar species found on periodically submerged siliceous rocks, sometimes also in humid forests; widespread but not common throughout the siliceous Alps. – **Au**: V, T, S, St. **Ge**: OB. **Sw**: BE, GR, VS. **Fr**: HAl, AMa, Isè, HSav. **It**: Frl, TAA, Lomb, Piem. **Sl**: SlA.

***Aspicilia laevatoides* (H. Magn.) Oxner**

Syn.: *Lecanora laevatoides* H. Magn.

L # – Subs.: sil, sil-aqu – Alt.: 2–4 – Note: a species described from South Tyrol, apparently ranging from Southern Scandinavia to the mountains of North Africa, on periodically submerged siliceous rocks along brooks, with a few records from the Southern Alps (Italy). – **It**: TAA, Lomb.

***Aspicilia lignicola* Hue**

Syn.: Aspicilia gibbosa (Ach.) Körb. f. lignicola Anzi, *Lecanora lignicola* (Hue) Zahlbr.

L # – Subs.: xyl – Alt.: 3–4 – Note: a rarely collected species, reported from the Alps and the North African mountains. – **Au**: T. **Fr**: AHP, HAl. **It**: Lomb, Piem.

***Aspicilia lobulata* (Anzi) Hue**

Syn.: *Aspicilia calcarea* (L.) Bagl. α [var.] *concreta* (Schaer.) Körb. [f.] *lobulata* Anzi, Aspicilia verruculosa *auct. non* Kremp., *Lecanora effigurans* Zahlbr., *Lecanoraverruculosa auct. non* (Kremp.) J. Steiner *nec* Bagl. *nec* Jatta

L # – Subs.: cal – Alt.: 4–6 – Note: a species with a bluish-grey, effigurate thallus; differences from *A. candida* are in need of evaluation; the type is on serpentine (but the species also occurs on slightly calcareous schists). – **Ge**: Schw. **Fr**: AHP, HAl, AMa, Sav, HSav. **It**: TAA.

***Aspicilia mashiginensis* (Zahlbr.) Oxner**

Syn.: Aspicilia cinerea (L.) Körb. f. papillata Arnold, Aspicilia mastrucata *auct. eur. austr. non* (Wahlenb.) Ach., *Lecanora mashiginensis* Zahlbr.

L – Subs.: sil, int – Alt.: 4–5 – Note: on basic siliceous rocks, often on weakly calciferous schists, in humid-shaded situations near and above treeline; to be looked further throughout the Alps. – **Au**: T, S, K, St. **Fr**: AHP, HAl, AMa, Isè. **It**: Frl, TAA, Piem.

***Aspicilia mastrucata* (Wahlenb.) Th. Fr.**

Syn.: *Lecanora lyckselensis* H. Magn., *Lecanora mastrucata* (Wahlenb.) Ach., *Lecanora subreagens* H. Magn., *Lichen mastrucatus* Wahlenb., *Sagedia mastrucata* (Wahlenb.) A. Nordin, Savić & Tibell

L # – Subs.: sil, int – Alt.: 4–6 – Note: a species found on siliceous to weakly calciferous rocks, mostly above treeline, belonging to a poorly understood complex, reported from Northern Europe, upland areas of Central Europe and Turkey; it is however dubious that the samples from the Alps belong to *A. mastrucata* in the strict sense. – **Au**: V, T, S, K, St, N. **Ge**: Ge. **Sw**: BE, GR, VS.

***Aspicilia mauritii* Hue**

Syn.: *Lecanora mauritii* (Hue) Zschacke

L # – Subs.: sil – Alt.: 4 – Note: this silicicolous species is known from a few localities in the Western Alps (Switzerland). – **Sw**: GR.

***Aspicilia montana* (H. Magn.) Hav.**

Syn.: *Lecanora montana* H. Magn.

L # – Subs.: sil, met – Alt.: 4 – Note: a species with a thin, rimose, brownish-grey thallus reacting K+ red, mostly developing at the same time roundish, coarsely granular soralia and apothecia with broad, black margins; a taxon based on a type from Northern Sweden, whose ecology and distribution are still insufficiently known. – **Au**: S.

***Aspicilia niesenensis* (H. Magn.) Hafellner**

Syn.: *Lecanora niesenensis* H. Magn.

L # – Subs.: cal – Alt.: 5 – Note: a poorly known species with a spreading, areolate, lead-grey thallus reacting K-, immersed apothecia, usually several per areole, with black discs, hymenium *c.* 100 μm high, paraphyses moniliform in the upper part, 8-spored asci, broadly ellipsoid ascospores (16–21 × 10–12 μm), and filiform, arcuate conidia; on calcareous rocks in the alpine belt; only known from the type locality in the Western Alps (Switzerland). – **Sw**: BE.

***Aspicilia nunatakkorum* (Poelt) Poelt *ex* Hafellner & Türk**

Syn.: Aspicilia mastrucata (Wahlenb.) Th. Fr. f. pseudoradiata Arnold, *Lecanora nunatakkorum* Poelt

L – Subs.: sil – Alt.: 5–6 – Note: a species with a (non-effigurate!) dark-grey, papillate (to almost dwarf-fruticose) thallus partially reacting K+ red, often sterile, but sometimes with solitary, sessile apothecia; on exposed siliceous rocks at high altitudes; the distribution is still insufficiently known, but is probably wider than the few records would suggest. – **Au**: T. **Ge**: Schw.

***Aspicilia obscurata* (Fr. *ex* Nyl.) Arnold**

Syn.: Lecanora cinerea (L.) Sommerf. var. obscurata Fr. *ex* Nyl.

L – Subs.: sil – Alt.: 3–4 – Note: the conspecificity of records from the Eastern Alps (Austria) with the type from Scandinavia is in need of evaluation; ecology and distribution are still insufficiently known. – **Au**: V, T.

***Aspicilia plumbeola* (Müll. Arg.) Hue**

Syn.: *Lecanora plumbeola* Müll. Arg.

L # – Subs.: sil – Alt.: 5 – Note: a species resembling in habitus a juvenile *Miriquidica plumbea*, with a bluish-grey, areolate thallus on a black hypothallus, apothecia (*c.* 0.3 mm in diam.) immersed in the thallus, usually 1 per areole, with a brown-black disc, 8-spored asci, and ellipsoid ascospores (18–20 × 9–10 μm); on siliceous rocks in the high-alpine belt; only known from the type locality in the Western Alps (Switzerland). – **Sw**: VS.

**Aspicilia polychroma Anzi subsp. polychroma**

Syn.: *Lecanora polychroma* (Anzi) Nyl.

L – Subs.: sil – Alt.: 3–5 – Note: a mainly arctic-alpine, perhaps circumpolar, chemically and morphologically variable species with optimum on more or less calciferous siliceous rocks; widespread throughout the Alps, wherever suitable substrata are present. – **Au**: V, T, S, K, St. **Ge**: Schw. **Sw**: GR, VS. **Fr**: AHP, HAl, AMa, Isè, Sav. **It**: Ven, TAA, Lomb, Piem, VA, Lig.

**Aspicilia polychroma Anzi subsp. hypertrophica Cl. Roux**

Syn.: Aspicilia polychroma Anzi subsp. hypertrophica Asta & Cl. Roux [invalidly published], Aspicilia polychroma Anzi var. kalireagens Asta & Cl. Roux [invalidly published]

L – Subs.: int – Alt.: 4–5 – Note: this lichen, differing from the typical subspecies in the well-developed thallus containing variable amounts of atranorin, grows on sunny surfaces of weakly calciferous siliceous rocks, often with *Lecanora albula*; taxon based on a type from the Pyrenees; from the Alps there are only a few scattered records. – **Au**: V. **Ge**: OB. **Fr**: AHP, HAl, AMa.

***Aspicilia prestensis* Cl. Roux & A. Nordin**

L – Subs.: sil – Alt.: 3–5 – Note: a species described from the Pyrenees, with an areolate, non-effigurate, whitish-grey thallus reacting K+ red, often confused with *A. cinerea* (with smaller ascospores and conidia) and *A. spermatomanes* (with glebulose areolae, cortex with a brown pigment, longer conidia); it grows on acidic rocks, with optimum in sunny places, near and above treeline. A similar species, *A. epiglypta* is restricted to coastal areas in Northern Europe, and records from elsewhere may be due to confusion with other species, especially with *A. prestensis*. – **Fr**: AHP, AMa, Sav, HSav. **It**: TAA.

***Aspicilia proluta* (Nyl.) Hue**

Syn.: *Aspicilia submersa* (Lamy) Hue, *Lecanora caesiocinerea* Nyl. *ex* Malbr. f. proluta Nyl., *Lecanora proluta* (Nyl.) Zahlbr., Lecanora subdepressa (Arnold) Nyl. var. submersa Lamy, *Lecanora submersa* (Lamy) Zahlbr.

L # – Subs.: sil, sil-aqu – Alt.: 3–4 – Note: the differences from *A. aquatica* (both species have ascospores of comparable size) are in need of re-evaluation; on periodically or constantly inundated siliceous rocks; a poorly known taxon based on a type from the Pyrenees, whose distribution is insufficiently known. – **Fr**: HSav. **It**: Lig.

***Aspicilia reagens* (Zahlbr.) Cl. Roux & M. Bertrand**

Syn.: Lecanora calcarea f. reagens Zahlbr., Aspicilia calcarea (L.) Bagl. var. reagens (Zahlbr.) Szatala

L – Subs.: cal – Alt.: 2 – Note: this taxon was often regarded just a chemical strain of *A. calcarea* with abundant norstictic acid. It is however more closely related to *A. serenensis* than to *A. calcarea* because of the cortex not filled by crystals, the rounded apothecia with a well-developed thalline margin, the amphithecium containing the *subdepressa*-brown pigment, and the size of conidia. It differs from *A. serenensis* in the chemistry (norstictic and stictic acids), in several morphological traits, and in the altitudinal distribution. To be looked for further in the Alps. – **Fr**: AHP, AMa, Drô, Var, Vau. **It**: Ven.

***Aspicilia rosulata* Körb.**

Syn.: *Aspicilia proserpens* (Nyl.) Hue

L # – Subs.: sil, int – Alt.: 5–6 – Note: a fertile species with a grey to blackish-brown, effigurate thallus and finally sessile apothecia; based on a type from Franz Josef Land, where it grows on periodically inundated boulders; identity and uniformity of records from the Alps are uncertain, because the secondary chemistry is apparently not uniform, and the ecology is different (on exposed, usually steep rock faces). – **Au**: T, S, K. **Fr**: HAl.

***Aspicilia serenensis* Cl. Roux & M. Bertrand**

L – Subs.: cal – Alt.: 2–4 – Note: a recently described species, differing from *A. calcarea* (closely related), *A. farinosa* and *A. subfarinosa* in having a distinctly thicker thallus (with a thick medulla), larger apothecia with the pigment *subdepressa*-brown in the cortex of the thalline margin, slightly longer conidia, and a more orophilous distribution. The species might have been filed under *A. calcarea* in the past, and should be looked for throughout the Alps. – **Fr**: AHP, AMa, Drô, Isè, Var, Vau.

***Aspicilia simoensis* Räsänen**

Syn.: *Lecanora bahusiensis* H. Magn., *Lecanora isidiata* (H. Magn.) H. Magn., *Lecanora simoensis* (Räsänen) Zahlbr., Lecanora simoensis (Räsänen) Zahlbr. var. isidiata H. Magn., *Sagedia simoensis* (Räsänen) A. Nordin, Savić & Tibell

L – Subs.: sil, int – Alt.: 2–5 – Note: a mostly sterile species with an areolate thallus reacting K+ red; based on a type from Finland and described as sorediate from the beginning; populations in the Alps have dense clusters of isidia later breaking down into soredia-like propagules (fitting Lecanora simoensis var. isidiata H. Magn.); most frequent on siliceous boulders visited by birds, widespread in the Alps. – **Au**: V, T, S, K, St. **Ge**: OB. **Sw**: GR, LU, SZ. **Fr**: AHP, AMa, HSav. **It**: Ven, TAA, VA. **Sl**: SlA.

***Aspicilia sophodopsis* (Nyl.) Arnold**

Syn.: *Lecanora sophodopsis* Nyl.

L # – Subs.: sil – Alt.: 4 – Note: a species with a thallus recalling a sterile *Staurothele ambrosiana* in being minutely granulose, olive-brown to greyish-brown, but reacting K+ red; perhaps this could be the correct name for alpine populations of putative “*A. leprosescens*” or even “*A. simoensis*”; on siliceous boulders, in the study area so far only known from the Eastern Alps (Austria). – **Au**: T.

***Aspicilia spermatomanes* Maheu & A. Gillet**

L – Subs.: sil – Alt.: 3–5 – Note: a species described from the Pyrenees and recently re-evaluated, whose distribution is insufficiently known: there are many records from the French Alps, but the species is likely to be widespread throughout the Alps, having often being confused with other species, especially *A. cinerea*. – **Fr**: AHP, AMa, Sav.

***Aspicilia subdepressa* Arnold**

Syn.: *Aspicilia caesiocinerea* (Nyl. *ex* Malbr.) Arnold var. subdepressa (Arnold) Clauzade & Cl. Roux, *Lecanora subdepressa* (Arnold) Nyl., *Pachyospora subdepressa* (Arnold) M. Choisy

L – Subs.: sil – Alt.: 2–4 – Note: a silicicolous species of vertical to inclined rocks in rather dry areas, with optimum in the montane belt, with a few scattered records from the Alps. – **Au**: T, K, N. **Fr**: AMa. **It**: TAA, Piem.

***Aspicilia supertegens* Arnold**

Syn.: *Aspicilia prinii* B. de Lesd., *Lecanora leucostoma* H. Magn., *Lecanora supertegens* (Arnold) Zahlbr.

L – Subs.: sil-aqu – Alt.: 3–5 – Note: a boreal-montane to arctic-alpine, perhaps circumpolar, variable lichen found on lime-free but base-rich rocks, often on mica-schist in humid situations (near brooks, melting snow, etc.). – **Au**: V, T, S, K. **Ge**: Schw. **Sw**: GR, VS. **Fr**: HAl, AMa, Sav. **It**: Frl, TAA, Lomb.

***Aspicilia valpellinensis* B. de Lesd.**

Syn.: Aspicilia cinerea (L.) Körb. var. chiodectonoides Anzi, *Lecanora valpellinensis* (B. de Lesd.) Zahlbr.

L # – Subs.: int – Alt.: 4–5 – Note: on calciferous schists; only known from the Italian Alps (Scandinavian material belongs to *A. supertegens*, see Nimis 2016). – **It**: Lomb, VA.

***Aspicilia verrucigera* Hue**

Syn.: *Lecanora verrucigera* (Hue) Zahlbr.

L # – Subs.: sil – Alt.: 3–4 – Note: a fertile species with a grey, verrucose thallus; taxon based on a type from Finland, whose ecology and distribution are insufficiently understood. – **Fr**: HAl.

**Aspicilia verrucosa (Ach.) Körb. subsp. verrucosa**

Syn.: *Amygdalaria verrucosa* (Ach.) Norman, *Lecanora urceolaria* (Fr.) Wetmore, *Lecanora verrucosa* (Ach.) Laurer, *Megaspora verrucosa* (Ach.) Hafellner & V. Wirth, *Pachyospora verrucosa* (Ach.) A. Massal., *Pertusaria freyi* Erichsen, *Urceolaria verrucosa* Ach.

L – Subs.: bry, deb, ter-cal – Alt.: 2–6 – Note: a circumpolar, arctic-alpine lichen found on mosses and plant debris over calciferous ground in open situations; it descends to lower altitudes in dry-continental areas; widespread and common throughout the Alps. – **Au**: V, T, S, K, St, O, N. **Ge**: OB. **Sw**: BE, GR, LU, SZ, TI, UR, VD, VS. **Fr**: AHP, HAl, AMa, Isè, Sav, HSav, Vau. **It**: Frl, Ven, TAA, Lomb, Piem, VA, Lig. **Sl**: SlA. **Li**.

**Aspicilia verrucosa (Ach.) Körb. subsp. mutabilis (Ach.) Cl. Roux**

Syn.: *Aspicilia mutabilis* (Ach.) Körb., *Lecanora mutabilis* (Ach.) Nyl., Megaspora verrucosa (Ach.) Hafellner & V. Wirth var. mutabilis (Ach.) Nimis & Cl. Roux, *Pachyospora mutabilis* (Ach.) A. Massal., *Patellaria mutabilis* (Ach.) Trevis., *Urceolaria mutabilis* Ach.

L # – Subs.: cor – Alt.: 2–4 – Note: on basal parts of old deciduous trees; doubtfully distinct from the typical subspecies. – **Fr**: AHP, HAl, AMa, Isè, Sav, HSav, Vau. **It**: Ven, Lomb, Piem, VA.

***Aspicilia verruculosa* Kremp.**

L # – Subs.: sil, int – Alt.: 4–5 – Note: on weakly calciferous rocks. A critical taxon, known only from the Southern European mountains, but perhaps just a chemotype of *A. polychroma* (see *e.g.* Roux et al. 2014). – **Au**: V, T, S, K, St, O. **Ge**: OB. **Sw**: BE, GR, UR. **Fr**: AHP, HAl, AMa, Isè, Sav, Vau. **It**: TAA, Lomb, Piem.

***Aspicilia viridescens* (A. Massal.) Hue**

Syn.: *Pachyospora viridescens* A. Massal.

L – Subs.: sil – Alt.: 2–4 – Note: this species was often confused with *A. contorta*, but it occurs on siliceous rocks; it is rather common at low altitudes in the Western Alps. – **Fr**: AHP, HAl, AMa, Isè, Var, Vau. **It**: Ven, TAA.

***Aspicilia zonata* (Ach.) R. Sant.**

Syn.: *Aspicilia waldrastensis* (H. Magn.) Clauzade & Rondon, *Lecanora waldrastensis* H. Magn., *Sagedia zonata* Ach.

L – Subs.: sil – Alt.: 3–4 – Note: a fertile silicicolous species with a grey, areolate thallus, sometimes with concentric marginal zones; the distribution is insufficiently known. The species has been frequently confused with *A. caesiocinerea*, from which it differs in the 8-spored asci, the slightly smaller spores, and the absence of aspicilin. Several records of *A. caesiocinerea* from upland areas could refer to this species. – **Au**: V, T. **Ge**: Schw. **Fr**: AHP, HAl, AMa, Sav. **It**: TAA.

***Aspilidea myrinii* (Fr.) Hafellner**

Syn.: *Aspicilia adunans* (Nyl.) Arnold, Aspicilia cinerea (L.) Körb. var. alpina (Fr.) Körb., *Aspicilia glacialis* (Arnold) Dalla Torre & Sarnth., *Aspicilia myrinii* (Fr.) Stein, *Lecanora adunans* Nyl., *Lecanora myrinii* (Fr.) Tuck., *Parmelia myrinii* Fr.

L – Subs.: sil – Alt.: 3–6 – Note: a mainly arctic-alpine, circumpolar species found on crystalline schists and acid siliceous rocks in upland areas; widespread in the Alps. – **Au**: V, T, S, K, St, N. **Ge**: Schw. **Sw**: GR, VS. **Fr**: AHP, HAl, HSav. **It**: Frl, TAA, Lomb, Piem, VA.

***Atla alpina* Savić & Tibell**

Syn.: Polyblastia theleodes *auct. p.p*.

L – Subs.: cal – Alt.: 4–5 – Note: on calcareous rocks (mesozoic limestone, marble of variable ages) with at least locally increased humidity; widespread and fairly common throughout the Alps, but often filed under “*Polyblastia theleodes*”, and hence under-recorded. – **Au**: T, S, K, St. **Ge**: OB, Schw. **It**: Ven, TAA, Lomb, Piem, VA.

***Atla wheldonii* (Travis) Savić & Tibell**

Syn.: *Polyblastia wheldonii* Travis

L – Subs.: ter-cal – Alt.: 4 – Note: a relatively rare species and the only terricolous one in the genus; optimum on basic sandy soil, from where it may spread over decaying mosses and plant remains, mostly near treeline. – **Sl**: SlA.

***Bacidia absistens* (Nyl.) Arnold**

Syn.: *Bacidia intermissa* (Nyl.) Malme, *Lecidea absistens* Nyl., *Lecidea intermissa* Nyl.

L – Subs.: cor, bry – Alt.: 3 – Note: a mild-temperate to humid subtropical species found on base-rich substrata, in clearings of ancient forests, sometimes on epiphytic bryophytes, with a few scattered records from the Alps. – **Au**: T, S, K, St, O. **Sw**: BE. **It**: TAA. **Sl**: SlA.

***Bacidia arceutina* (Ach.) Arnold**

Syn.: *Bacidia leightoniana* (Larbal. *ex* Leight.) H. Olivier, Biatora luteola (Schrad.) Fr. var. fuscella (Fr.) Th. Fr., *Lecidea arceutina* (Ach.) Gray, *Lecidea leightoniana* Larbal. *ex* Leight., Lecidea luteola (Schrad.) Ach. var. arceutina Ach., Lecidea luteola (Schrad.) Ach. var. fuscella Fr.

L – Subs.: cor, cal, bry – Alt.: 1–4 – Note: a mild-temperate to humid subtropical species found on bark of broad-leaved trees (especially *Acer*, *Fraxinus* and *Populus*) in open woodlands near rivers, very rarely calcicolous or muscicolous; widespread throughout the Alps. – **Au**: V, T, S, K, St, O, N. **Ge**: OB, Schw. **Sw**: BE, FR, UW, VD. **Fr**: AHP, AMa, Isè, Var, Vau. **It**: Frl, TAA, Lomb, Piem. **Sl**: SlA.

***Bacidia auerswaldii* (Hepp *ex* Stizenb.) Mig.**

Syn.: *Bacidia effusa* (Auersw. *ex* Rabenh.) Lettau *non* (Sm.) Trevis, *Bacidia effusella* Zahlbr., *Bilimbia effusa* Auersw. *ex* Rabenh., *Lecidea auerswaldii* Hepp *ex* Stizenb., *Lecidea effusa* (Auersw. *ex* Rabenh.) Stizenb.

L – Subs.: cor – Alt.: 3 – Note: a mild-temperate to humid subtropical, mainly subatlantic species of humid open forests, with a few records from the Eastern Alps only (Austria). – **Au**: S, O, N.

***Bacidia badensis* (Körb.) Zahlbr.**

Syn.: *Bilimbia badensis* Körb.

L # – Subs.: xyl – Alt.: 4 – Note: also reported from Germany, on wood in the subalpine belt, with a few scattered records from the Alps. – **Au**: S. **It**: Lomb.

***Bacidia bagliettoana* (A. Massal. & De Not.) Jatta**

Syn.: Bacidia atrosanguinea var. argillicola (Malbr.) H. Olivier, *Bacidia maceriarum* B. de Lesd., *Bacidia muscorum* (Ach.) Mudd, *Bacidia pezizoidea* *sensu* Anzi, *Lecidea muscorum* Ach. *non* (Th. Fr.) Dalla Torre & Sarnth., *Scoliciosporum bagliettoanum* A. Massal. & De Not.

L – Subs.: ter-cal, bry, deb – Alt.: 2–5 – Note: an arctic-alpine to boreal-montane, circumpolar lichen of moribund bryophytes and plant debris in dry grasslands, or in fissures of calcareous rocks and dolomite, with optimum in upland areas. The holotype of *B. maceriarum*, examined by Roux (unpublished), proved to belong to this species; widespread throughout the Alps. – **Au**: V, T, S, K, St, O, N. **Ge**: OB, Schw. **Sw**: BE, GR, LU, SZ, TI, UR, UW, VD, VS. **Fr**: AHP, HAl, AMa, Drô, Isè, Sav, HSav, Var, Vau. **It**: Frl, Ven, TAA, Lomb, Piem, Lig. **Sl**: SlA, Tg.

***Bacidia biatorina* (Körb.) Vain.**

Syn.: Bacidia acerina *auct. non* (Ach.) Arnold, Raphiospora atrosanguinea Ach. var. biatorina Körb.

L – Subs.: cor – Alt.: 2–3 – Note: a rare, oceanic species growing on trunks of mature trees in old woodlands, mostly in *Lobarion*-communities; probably more widespread in the Alps than the relatively few scattered records suggest. – **Au**: S, K, St. **Ge**: OB. **Sw**: SG. **Sl**: Tg.

***Bacidia caesiomarginata* (Kernst.) Lettau**

Syn.: *Bilimbia caesiomarginata* Kernst.

L – Subs.: cal, int, bry – Alt.: 4–5 – Note: a species with apothecial margins covered with a bluish-grey pruina when young, and 1–3-septate, elongated-oblong ascospores; on limestone and overgrowing bryophytes in shaded situations, *e.g.* within subalpine forests; apparently rather rare. – **Au**: V, T, K, St. **Ge**: Schw.

***Bacidia circumspecta* (Nyl. *ex* Vain.) Malme**

Syn.: *Bacidia bacillifera* (Nyl.) Arnold *p.p.*, *Bacidia quercicola* (Nyl.) Vain., Lecidea bacillifera Nyl. var. circumspecta Nyl. *ex* Vain., *Lecidea circumspecta* (Vain.) Hedl.

L – Subs.: cor – Alt.: 2–3 – Note: a mild-temperate lichen found on old trees in open, humid woodlands, more rarely on primarily acid, but nutrient-enriched bark. – **Au**: T, S, K, St, O, N. **Ge**: OB. **Sw**: BE, GR, LU, SZ, UW, VS. **Fr**: AHP, AMa, Isè, Var. **It**: TAA, Lomb, Piem.

***Bacidia coprodes* (Körb. *ex* Arnold) Lettau**

Syn.: *Bacidia salevensis* (Müll. Arg.) Zahlbr., *Bacidia subtrachona* (Arnold) Lettau, Bacidia trachona *auct. p.p.*, *Bilimbia coprodes* Körb. *ex* Arnold, *Bilimbia subtrachona* Arnold, *Gyalecta salevensis* (Müll. Arg.) H. Olivier, *Patellaria salevensis* Müll. Arg.,

L – Subs.: cal, int, cor – Alt.: 2–4 – Note: on steeply inclined to underhanging faces of calciferous or base-rich siliceous rocks, exceptionally on bark in deep crevices at the base of trunks. – **Au**: S K St O N. **Sw**: LU. **Fr**: HAl, AMa, Sav, HSav, Vau. **It**: Ven, TAA, Lig.

***Bacidia fraxinea* Lönnr.**

Syn.: *Bacidia fallax* (Körb.) Lettau, Bacidia rubella (Hoffm.) A. Massal. var. fallax Körb.

L – Subs.: cor – Alt.: 1–3 – Note: a mild-temperate, probably Mediterranean-Atlantic lichen found on deciduous trees, especially *Acer*, in open, humid woodlands, with a few scattered records from the Alps. – **Au**: N. **Fr**: AMa, Sav, Var, Vau.

***Bacidia friesiana* (Hepp) Körb.**

Syn.: *Biatora friesiana* Hepp

L – Subs.: cor, xyl – Alt.: 1–3 – Note: a mild-temperate lichen, most frequent on *Sambucus*, or near the base of trees with nutrient-rich bark, with optimum in the submediterranean belt. – **Au**: V, T, K, St, O, N. **Fr**: AMa, Var, Vau. **It**: Frl, TAA, Lomb, Piem, Lig. **Sl**: SlA.

***Bacidia fuscoviridis* (Anzi) Lettau**

Syn.: *Biatorina albidocarnea* (Nyl.) A.L. Sm., *Bilimbia albidocarnea* (Nyl.) A.L. Sm., *Bilimbia albocarnea* (Nyl.) A.L. Sm., *Bilimbia fuscoviridis* Anzi, *Lecidea albidocarnea* Nyl., *Toninia albidocarnea* (Nyl.) Guillaumot

L – Subs.: sil, met – Alt.: 2–3 – Note: a mild-temperate lichen found on calciferous and basic siliceous rocks in sheltered and humid situations; rarely collected, being often sterile, with a few scattered records from the Alps. – **Au**: S, K, St. **Ge**: OB. **Sw**: LU. **It**: Frl, Lomb. **Sl**: SlA.

***Bacidia herbarum* (Stizenb.) Arnold**

Syn.: *Bacidia fraterna* Anzi, *Mycobilimbia herbarum* (Stizenb.) Rehm, *Secoliga herbarum* Stizenb.

L – Subs.: bry-cal, deb-cal – Alt.: 3–5 – Note: a cool-temperate to arctic-alpine, probably circumpolar lichen found on plant remains and moribund bryophytes on calciferous ground, more rarely on bark, with optimum in upland areas; widespread throughout the Alps. – **Au**: V, T, S, K, St, O, N. **Ge**: OB, Schw. **Sw**: LU, TI, UR, UW, VD, VS. **Fr**: HAl, AMa, Sav, HSav, Vau. **It**: Frl, TAA, Lomb, Piem.

***Bacidia igniarii* (Nyl.) Oxner**

Syn.: *Bacidia abbrevians* (Nyl.) Th. Fr., *Bilimbia igniarii* (Nyl.) Arnold, *Lecidea igniarii* Nyl.

L # – Subs.: cor – Alt.: 2–3 – Note: a northern species of smooth bark, very rarely found on lignum; on the whole a critical taxon, which needs revision, with a few scattered records from the Alps. – **Ge**: OB. **Sw**: GR. **Fr**: AMa, Vau. **It**: TAA, Lomb, Piem.

***Bacidia illudens* (Nyl.) Lynge**

Syn.: *Lecidea illudens* Nyl.

L # – Subs.: ter-sil, ter-cal, bry – Alt.: 2–3 – Note: a species with apothecial margins mostly covered with a bluish-grey pruina, and 5–7-septate, acicular ascospores; on decaying bryophytes; based on a type from Finland, with a few scattered records from the Alps. – **Au**: St. **Sw**: GR.

***Bacidia incompta* (Borrer) Anzi**

Syn.: *Bacidia atrosanguinea* (Schaer.) Anzi, *Bacidia subinundata* (Nyl.) Blomb. & Forssell, *Bacidia viridula* Erichsen, *Biatora atrosanguinea* (Schaer.) Hepp, *Lecidea atrosanguinea* (Schaer.) Th. Fr. *non* (Hoffm.) Nyl., *Lecidea incompta* Borrer, *Scoliciosporum molle* A. Massal.

L – Subs.: cor, deb, bry – Alt.: 2–3 – Note: a temperate species found on base-rich bark, especially of *Ulmus*, near wounds of the trunk, more rarely on plant debris and terricolous mosses. – **Au**: S, St, O, N. **Sw**: GR, SZ, VS. **Fr**: Sav, HSav. **It**: Ven, TAA, Lomb, Piem. **Sl**: SlA.

***Bacidia laminularis* (Müll. Arg.) Zahlbr.**

Syn.: *Bilimbia nitschkeana* J. Lahm *ex* Rabenh. var. laminularis (Müll. Arg.) H. Olivier, *Patellaria laminularis* Müll. Arg.

L # – Subs.: cor – Alt.: 3 – Note: a species with bluish-black apothecia and 2–4-septate, narrowly ellipsoid ascospores which are only *c.* 10 µm long; on the whole a poorly known taxon, perhaps a *Micarea*. – **Fr**: HSav.

***Bacidia laurocerasi* (Delise *ex* Duby) Zahlbr.**

Syn.: *Bacidia atrogrisea* (Delise) Körb., *Bacidia elevata* Körb., Bacidia endoleuca *auct. non* (Nyl.) J. Kickx f., Bacidia subacerina Vain. subsp. laurocerasi (Delise *ex* Duby) Vain., *Biatora atrogrisea* Delise, *Patellaria laurocerasi* Delise *ex* Duby

L – Subs.: cor – Alt.: 1–3 – Note: a humid subtropical to Mediterranean-Atlantic lichen found on smooth bark of broad-leaved trees in open, humid forests; widespread throughout the Alps but generally rare, and perhaps declining. – **Au**: T, S, K, St, O, N. **Ge**: OB. **Sw**: BE, GL, GR, SZ, UW, VD. **Fr**: AMa, Isè, Var, Vau. **It**: Frl, Ven, TAA, Lomb. **Sl**: SlA.

***Bacidia leptosperma* (Anzi) Lettau**

Syn.: *Bilimbia leptosperma* Anzi

L # – Subs.: bry – Alt.: 4–5 – Note: a species with a whitish, granular thallus delimited by a white prothallus, small, sessile, black, first plane then convex and immarginate apothecia with a brown epithecium and a black hypothecium, coeherent paraphyses, 8-spored asci, and 1–3-septate, fusiform ascospores measuring *c.* 9 × 3 µm; only known from the type material, collected on terricolous mosses over granite above Bormio (Italy). – **It**: Lomb.

***Bacidia notarisiana* (A. Massal.) Zahlbr.**

Syn.: *Bilimbia notarisiana* A. Massal.

L – Subs.: cal – Alt.: 1–2 – Note: on calcareous rocks, sometimes in anthropogenic settings (*e.g.*, cement constructions); currently known only from low or moderate elevations in Northern Italy, but likely to be more widespread in the Mediterranean region. – **It**: Frl, Lig.

***Bacidia piciloides* (Zahlbr.) *ined.* (provisionally placed here, ICN Art. 36.1b)**

Syn.: *Catillaria piciloides* Zahlbr.

L # – Subs.: sil – Alt.: 3 – Note: a species which is perhaps related to *Catillaria picila*, with a thin, greyish, subleprose to pulverulent thallus and sessile, brown-black, lecideine apothecia with a brown-black exciple and hypothecium, a brownish epihymenium, conglutinated, not distinctly capitate paraphyses, 8-spored asci, and hyaline, oblong 1-septate ascospores (16–18 × 5–6 μm); on sandstone in a montane forest, only known from the type locality in the Eastern Alps (Austria). – **Au**: N.

***Bacidia polychroa* (Th. Fr.) Körb.**

Syn.: *Bacidia acerina* (Ach.) Arnold, *Bacidia anceps* Anzi, *Bacidia fuscorubella* (Ach.) Bausch, *Bacidia polysita* (Stirt.) A.L. Sm., *Biatora polychroa* Th. Fr., *Lecidea acerina* (Ach.) Röhl., *Secoliga fuscorubella* (Ach.) Stizenb., *Verrucaria fuscorubella* Hoffm. *nom. inval*.

L – Subs.: cor – Alt.: 1–3 – Note: a mild-temperate to humid subtropical lichen found on broad-leaved trees in open, humid forests, with a few scattered records from the Alps. – **Au**: K, St, N. **Fr**: Var. **It**: TAA, Lomb. **Sl**: SlA.

***Bacidia punica* Llop**

L # – Subs.: cor – Alt.: 1 – Note: a recently-described epiphytic species, widespread but not common in shaded-humid situations, with optimum within eu-Mediterranean vegetation, also reported from the base of the Western Alps. It may, however, prove to be a synonym of *Bacidina phacodes* (see Nimis 2016). – **Fr**: AMa.

***Bacidia rosella* (Pers.) De Not.**

Syn.: *Biatora alabastrina* (Ach.) W. Mann, *Lecidea alabastrina* Ach., *Lichen rosellus* Pers.

L – Subs.: cor – Alt.: 1–3 – Note: a mild-temperate to Mediterranean-Atlantic lichen found on deciduous trees (especially *Acer* and *Fraxinus*, but also on *Quercus ilex*), in humid, open forests and in woodlands along rivers; widespread in the Alps, but generally rare, and perhaps declining. – **Au**: S, K, St, O, N. **Ge**: Schw. **Sw**: BE, UR. **Fr**: AMa, Var. **It**: Ven, Lomb, Piem.

***Bacidia rubella* (Hoffm.) A. Massal.**

Syn.: *Bacidia luteola* *auct.*, *Biatora luteola* *auct.*, *Lecidea luteola* *auct.*, *Lichen luteolus* Schrad. *nom.illeg.*, *Verrucaria rubella* Hoffm.

L – Subs.: cor – Alt.: 1–3 – Note: a temperate lichen found on old trees, especially oaks, still widespread, but probably declining, with optimum in the submediterranean belt; widespread throughout the Alps, but generally not very common. – **Au**: V, T, S, K, St, O, N. **Ge**: OB, Schw. **Sw**: BE, FR, GL, GR, LU, SZ, TI, UW, VD, VS. **Fr**: AHP, AMa, Drô, Isè, Sav, HSav, Var, Vau. **It**: Frl, Ven, TAA, Lomb, Piem, Lig. **Sl**: SlA, Tg. **Li**.

***Bacidia scoliciosporoides* (Bagl. & Carestia) Lettau**

Syn.: *Bilimbia scoliciosporoides* Bagl. & Carestia

L # – Subs.: deb – Alt.: 5 – Note: this long-forgotten and poorly understood species, often wrongly attributed to Baglietto alone, is characterised by a white, subleprose, rugulose-subgranulose, spreading thallus, small, subsessile, plane to convex, black apothecia with a thin to poorly evident proper margin (resembling those of *Lecidella wulfenii*), a yellowish brown epihymenium, a colourless hypothecium, adglutinate paraphyses, 8-spored, clavate asci, and large, fusiform, 3–7-septate ascospores which are 5–6 times as long as wide; known only from the type collection, on *Silene acaulis* in the alpine belt; the type material, most probably in MOD, would be worthy of further study. – **It**: Piem.

***Bacidia sordida* (Anzi) Lettau**

Syn.: *Bilimbia sordida* Anzi

L # – Subs.: cal – Alt.: 3 – Note: a species with a dirty white, thin, farinose, rimose-areolate thallus, small, black apothecia (turning brownish when wet) with a hyaline to pale brownish hypothecium, 8-spored asci, and 1–3-septate, hyaline, straight to slightly curved ascospores measuring 15–20 *× c.* 5 µm; only known from the type collection, on calciferous rocks at 1,350 m, this species could belong to *Lecania*. – **It**: Lomb.

***Bacidia subacerina* Vain.**

Syn.: *Bacidia violacea* (Arnold) Arnold

L – Subs.: cor – Alt.: 3 – Note: a species with brown to blackish apothecia and acicular ascospores; the purple-reddish epihymenium reacting K+ violet and the reddish exciple reacting K+ purple are diagnostic to separate it from *B. laurocerasi*; on acid bark in boreal to temperate-montane forests; in the study area there are only a few scattered records from the Eastern Alps (Austria, Slovenia). – **Au**: S, K, St. **Sl**: SlA.

***Bacidia subincompta* (Nyl.) Arnold**

Syn.: *Bacidia affinis* (Stizenb.) Vain., Bacidia atrosanguinea (Hepp) Anzi var. corticola Th. Fr., *Bacidia hegetschweileri* (Hepp) Vain. *non auct.*, *Bacidia intermediella* Vězda, *Bacidia separabilis* (Nyl.) Arnold, Biatora atrosanguinea Hepp var. hegetschweileri Hepp, *Lecidea hegetschweileri* Hepp, *Lecidea separabilis* Nyl., *Lecidea subincompta* Nyl.

L – Subs.: cor – Alt.: 1–4 – Note: a mainly temperate lichen found on bark of old broad-leaved trees (especially *Fagus* and *Quercus*) in open, humid woodlands; widespread throughout the Alps. – **Au**: V, T, S, K, St, O, N. **Ge**: OB, Schw. **Sw**: BE, GL, GR, LU, SG, SZ, TI, UR, UW, VD, VS. **Fr**: AHP, AMa, Drô, Isè, HSav, Var. **It**: Frl, Ven, TAA, Lomb, Piem, Lig. **Sl**: SlA, Tg. **Li**.

***Bacidia touzalinii* (Harm.) Zahlbr.**

Syn.: *Lecidea touzalinii* Harm.

L # – Subs.: cor – Alt.: not reported – Note: a corticolous species with blackish apothecia lacking a distinct margin, and 3-septate, narrowly ellipsoid ascospores; very poorly understood and perhaps belonging to *Micarea*; only known from the type locality in the Western Alps. – **Fr**: HSav.

***Bacidia trachona* (Ach.) Lettau**

Syn.: *Biatora trachona* (Ach.) Körb., *Bilimbia trachona* (Ach.) Trevis., *Verrucaria trachona* Ach.

L – Subs.: sil – Alt.: 1–4 – Note: the true *B. trachona* does not belong to *Bacidia* and has a more or less suboceanic distribution in Europe, from Portugal to Scandinavia. Several records from the Alps are dubious and could refer to *B. coprodes*. – **Au**: V, T, K. **Ge**: Ge. **Sw**: BE, GR, SZ, TI, VD. **Fr**: AMa, Var, Vau.

***Bacidia vermifera* (Nyl.) Th. Fr.**

Syn.: Bacidia hegetschweileri *auct. non* (Hepp) Vain., *Bacidia rhodopsis* Th. Fr. & Almq., *Bilimbia lecideoides* (Hazsl. *ex* Körb.) Th. Fr., *Lecidea vermifera* Nyl., *Scoliciosporum vermiferum* (Nyl.) Arnold

L – Subs.: cor – Alt.: 3–4 – Note: on the bark of broad-leaved trees in rather humid situations, more rarely on lignum, with scattered records from the Alps. – **Au**: V, T, St, O, N. **Fr**: Isè. **It**: TAA, Lomb, Piem.

***Bacidia viridescens* (A. Massal.) Th. Fr.**

Syn.: Bacidia muscorum (F.H. Wigg.) Mudd var. viridescens (A. Massal.) Arnold, *Heterothecium viridescens* A. Massal.

L # – Subs.: sil, cal – Alt.: 2 – Note: a species with marginate blackish apothecia and 6–8-septate acicular ascospores, pigmentation of hypothecium not indicated; on soil and overgrowing bryophytes; most likely a synonym of *B. bagliettoana*; B. viridescens *sensu* *auct. brit*. is likely to refer to a different species. – **Au**: O. **It**: Ven.

***Bacidia viridifarinosa* Coppins & P. James**

L – Subs.: sil, cor – Alt.: 2 – Note: a suboceanic species growing on shaded, smooth and not too acid siliceous rocks in oceanic humid woodlands, sometimes on smooth bark at the base of old deciduous trees; mostly sterile, with confluent soralia giving raise to yellow-green farinose soredia; the type material is from an old *Tilia* tree; it is not a *Bacidia* and belongs in the Pilocarpaceae; from the Alps there are only a few scattered records. – **Ge**: OB. **Fr**: AMa, Var, Vau.

***Bacidina arnoldiana* (Körb.) V. Wirth & Vězda**

Syn.: *Bacidia arnoldiana* Körb., *Lecidea larbalestieri* Cromb., *Woessia arnoldiana* (Körb.) Sérus. & Diederich

L – Subs.: cal – Alt.: 1–3 – Note: recently, two species were recognised within *B. arnoldiana*: a saxicolous species which corresponds to the type, and a corticolous species named *B. sulphurella*; some records from the Alps are likely to belong to *B. sulphurella*. – **Au**: O, N. **Ge**: OB, Schw. **Sw**: BE, GL, GR, SZ, TI, UR, UW, VS. **Fr**: AMa, Isè, Vau. **It**: Frl., Ven. **Sl**: SlA.

***Bacidina assulata* (Körb.) S. Ekman**

Syn.: *Bacidia anomala* A. Massal., *Bacidia assulata* (Körb.) Vězda, *Bacidia effusa* *auct.*, *Bacidia intermedia* (Hepp *ex* Stizenb.) Arnold, Bacidia rubella (Hoffm.) A. Massal. var. assulata Körb., Bilimbia effusa *auct*.

L – Subs.: cor – Alt.: 2–3 – Note: an epiphytic species belonging to a complex which is still in need of elucidation. – **Au**: V, T, K, St, O, N. **Ge**: OB, Schw. **Fr**: AMa. **It**: TAA, Lomb.

***Bacidina caligans* (Nyl.) Llop & Hladun**

Syn.: *Bacidia caligans* (Nyl.) A.L. Sm., *Lecidea caligans* Nyl., *Woessia caligans* (Nyl.) Sérus. & Diederich

L – Subs.: cal, cor – Alt.: 2 – Note: this species belongs to the *B. arnoldiana*-group, but is sorediate and generally sterile; it has a mild-temperate to Mediterranean distribution and is mainly calcicolous (sometimes occurring also on mosses and mortar, rarely on bark at the base of trunks), in shaded and humid situations at relatively low elevations; hitherto known only from the Western Alps, but, being easily overlooked, it could be more widespread. – **Fr**: HAl, AMa, Isè, Var.

***Bacidina chloroticula* (Nyl.) Vězda & Poelt**

Syn.: *Bacidia chloroticula* (Nyl.) A.L. Sm., *Bacidia lehriana* Erichsen, *Bacidia neglecta* Vězda, *Bacidia paulula* Erichsen, *Bacidia subchlorotica* (Nyl.) Flagey, *Bacidina neglecta* (Vězda) Vězda, *Lecidea chloroticula* Nyl., *Lecidea subchlorotica* Nyl., *Woessia chloroticula* (Nyl.) Sérus. & Diederich

L – Subs.: cor, sil – Alt.: 2–4 – Note: a mainly temperate to southern boreal species found on evergreen leaves and base-rich bark, sometimes on plant debris, calcareous stones, etc., mostly near the ground; certainly overlooked, and perhaps more widespread in the Alps, below the subalpine belt. – **Au**: V, T, St, O. **Ge**: OB, Schw. **Sw**: GR, SZ, VS. **Fr**: AMa, Isè, Var, Vau. **It**: Ven, Lomb. **Li**.

***Bacidina delicata* (Larbal. *ex* Leight.) V. Wirth & Vězda**

Syn.: *Bacidia arceutinella* Zahlbr., *Bacidia delicata* (Larbal. *ex* Leight.) Coppins, *Bilimbia arceutinoides* Anzi, *Lecidea effusa* *auct.* var. delicata Larbal. *ex* Leight., *Woessia delicata* (Larbal. *ex* Leight.) Sérus. & Diederich

L – Subs.: cor, sil – Alt.: 1–3 – Note: a Mediterranean-Atlantic to humid subtropical species found on bark, especially of *Sambucus* and *Salix* and – but only in very humid areas – on roofing tiles and plant debris. – **Au**: S, St, N. **Ge**: OB, Schw. **Fr**: AMa, Vau. **It**: Frl, Ven, Piem. **Sl**: SlA.

***Bacidina egenula* (Nyl.) Vězda**

Syn.: *Bacidia egenula* (Nyl.) Arnold, *Bacidia epiphylla* Wheldon & Travis, *Bacidia genuensis* B. de Lesd., *Bacidia mediterranea* B. de Lesd., *Bacidia sbarbaronis* B. de Lesd., *Lecidea egenula* Nyl.

L – Subs.: sil, xyl – Alt.: 1–4 – Note: a mild-temperate to humid subtropical species, most common on pebbles over moist ground in areas with siliceous substrata; from the Alps there are only a few scattered records. – **Au**: K, St, O. **Fr**: HSav, Var. **It**: Lomb, Lig.

***Bacidina inundata* (Fr.) Vězda**

Syn.: Bacidia arnoldiana Körb. var. inundata (Fr.) Körb., *Bacidia inundata* (Fr.) Körb., *Biatora inundata* Fr., *Lecidea inundata* (Fr.) Nyl., *Lichingoldia gyalectiformis* D. Hawksw. & Poelt, *Woessia inundata* (Fr.) Sérus. & Diederich

L – Subs.: sil-aqu, cal-aqu, xyl – Alt.: 1–4 – Note: apparently this is a holarctic lichen found on periodically inundated or otherwise moist siliceous rocks, more rarely on lignum, in humid-shaded situations, with a wide altitudinal range. – **Au**: T, S, K, St. **Sw**: GR. **Fr**: HAl, AMa. **It**: TAA, Lomb, Piem. **Sl**: SlA.

***Bacidina neosquamulosa* (Aptroot & Herk) S. Ekman**

Syn.: *Bacidia neosquamulosa* Aptroot & Herk

L – Subs.: cor – Alt.: 3 – Note: a species of the *B. arnoldiana*-group resembling *B. sulphurella*, but thallus minutely squamulose-isidiate, with longer, 3 – to 7-septate ascospores measuring 45–60 × 1.5–2 μm, and globose, erumpent dark pycnidia (to 0.13 mm in diam.) containing filiform, distinctly 3 – to 7-septate, curved macroconidia (40–55 × 1.5–2 μm); on trees with subneutral bark in nutrient-rich, dusty situations such as in urban parks and forest edges along secondary dirt roads; widespread in Europe and also recorded from Western North America, with a single record from the Eastern Alps (Austria), but probably more widespread. – **Au**: N.

***Bacidina phacodes* (Körb.) Vězda**

Syn.: *Bacidia albescens* (Stizenb.) Bausch, *Bacidia chlorotica* (Ach.) Sandst., *Bacidia phacodes* Körb., *Lecidea chlorotica* (Ach.) Nyl.

L – Subs.: cor, bry, sil – Alt.: 1–4 – Note: a mild-temperate to humid subtropical lichen found on bark of broad-leaved trees, more rarely on rock or silicicolous mosses, often on dry undersides of thick branches of ancient trees; widespread in the Alps, but not very common. – **Au**: V, T, S, K, St, O, N. **Ge**: OB, Schw. **Sw**: GR, SZ, UW. **Fr**: AMa, Drô, Isè, Var, Vau. **It**: Frl, TAA. **Sl**: SlA.

***Bacidina sulphurella* (Samp.) M. Hauck & V. Wirth**

Syn.: Bacidia arnoldiana Körb. var. corticola Arnold, *Bacidia sulphurella* Samp., *Woessia fusarioides* D. Hawksw., Poelt & Tscherm.-Woess

L – Subs.: cor – Alt.: 2–3 – Note: on bark, especially of *Sambucus*, sometimes invading corticolous mosses, more rarely on twigs, needles and living leaves in very humid sites. Some records of epiphytic *B. arnoldiana* could refer to this species. – **Au**: S, K, St, B. **It**: Frl.

***Bacidina vasakii* (Vězda) Vězda**

Syn.: *Bacidia vasakii* Vězda, *Woessia vasakii* (Vězda) Sérus.

L – Subs.: fol – Alt.: 2 – Note: a mild-temperate to humid pantropical species described from the Caucasus and also known from the Pyrenees, with a granular to subcoralloid thallus, hemispherical, whitish apothecia, and mostly 3-septate, acicular ascospores, found in the understory of forests, mostly on twigs and leaves of *Buxus*; rare and scattered in the Western Alps, at low elevations – **Fr**: AMa, Isè, Var, Vau.

***Bactrospora dryina* (Ach.) A. Massal.**

Syn.: *Arthonia dryina* (Ach.) Jatta, *Lecanactis dryina* (Ach.) Vain., *Lecanactis dryophila* Lettau, *Lecidea dryina* (Ach.) Ach., *Lichen dryinus* Ach., *Melaspilea patersonii* Stirt.

L – Subs.: cor – Alt.: 1–2 – Note: a mild-temperate to Mediterranean-Atlantic species found on bark of old, isolated deciduous trees, especially oaks, on faces which are seldom wetted by rain; rare, and certainly declining. – **Au**: St. **It**: Ven, Piem, Lig.

***Baeomyces carneus* Flörke**

Syn.: *Baeomyces byssoides* (L.) P. Gaertn., G. Mey. & Scherb. var. carneus (Flörke) Hepp, *Baeomyces caprinus* (Th. Fr.) H. Magn., *Baeomyces fuscorufescens* Vain., Baeomyces rufus (Huds.) Rebent. var. carneus (Flörke) Nyl.

L – Subs.: ter-sil, sil – Alt.: 2–6 – Note: a mainly boreal-montane, perhaps circumpolar lichen found on soils high in clay and on weathered siliceous rocks; widespread in the Alps, but generally not common. – **Au**: T, K, St, N, B. **Fr**: Sav, HSav. **It**: TAA, Lomb. **Sl**: SlA.

***Baeomyces placophyllus* Ach.**

Syn.: *Ludovicia placophylla* (Ach.) Trevis.

L – Subs.: ter-sil, ter-cal – Alt.: 3–5 – Note: an arctic-alpine to boreal-montane, probably circumpolar lichen found on sandy-clay soil in open stands (*e.g.* montane-subalpine grasslands), often in moderately disturbed habitats, sometimes reaching the alpine belt; widespread throughout the siliceous Alps. – **Au**: V, T, S, K, St, N. **Ge**: OB, Schw. **Sw**: BE, GL, GR, SZ, UW, VS. **Fr**: AMa, Isè, Sav, HSav. **It**: Frl, Ven, TAA, Lomb, Piem, VA.

**Baeomyces rufus (Huds.) Rebent. var. rufus**

Syn.: *Baeomyces byssoides* (L.) P. Gaertn., G. Mey. & Scherb., Baeomyces rufus (Huds.) Rebent. var. subsquamulosus Nyl., *Baeomyces rupestris* Pers. *ex* Ach., *Biatora byssoides* (L.) Fr., *Lichen fungiformis* Scop., *Lichen rufus* Huds., *Rinodina humilis* H. Magn., *Sphyridium byssoides* (L.) Beltr., *Sphyridium fungiforme* (Scop.) Flot., *Tubercularia rufa* (Huds.) Kuntze

L – Subs.: ter-sil, sil, xyl, bry – Alt.: 2–5 – Note: a holarctic early coloniser of acid soils with high clay content and of weathered siliceous rocks, often found in disturbed sites; mostly sterile in upland areas; widespread throughout the Alps. – **Au**: V, T, S, K, St, O, N, B. **Ge**: OB, Schw. **Sw**: BE, GR, LU, SG, SZ, UR, UW, VD, VS. **Fr**: AHP, HAl, AMa, Isè, Sav, HSav, Var, Vau. **It**: Frl, Ven, TAA, Lomb, Piem, VA. **Sl**: SlA, Tg.

**Baeomyces rufus (Huds.) Rebent. var. callianthus (Lettau) Lettau *ex* Frey**

Syn.: *Baeomyces callianthus* Lettau

L – Subs.: ter-sil, xyl – Alt.: 3 – Note: a taxon of the *B. rufus*-group with thallus composed of minute squamules and pink-coloured, distinctly marginate apothecia reacting K+ yellow, then red; on acid soil, rarely on stumps or encrusting bryophytes; distribution insufficiently known. – **Au**: T, S, St.

***Bagliettoa baldensis* (A. Massal.) Vězda**

Syn.: *Amphoridium baldense* (A. Massal.) A. Massal., *Protobagliettoa exesa* (Servít) Servít, *Protobagliettoa kutakiana* Servít, *Verrucaria baldensis* A. Massal., Verrucaria baldensis A. Massal. var. insculptoides (J. Steiner) Servít, Verrucaria calciseda DC. f. insculptoides J. Steiner, *Verrucaria subconcentrica* (J. Steiner) Servít, Verrucaria subconcentrica (J. Steiner) Servít var. metzleri Servít

L – Subs.: cal – Alt.: 1–4 – Note: a mainly mild-temperate species of compact calcareous rocks in natural, sheltered situations, with optimum in the submediterranean belt; widespread throughout the Alps. – **Au**: V, T, S, K, St, O, N. **Ge**: OB, Schw. **Sw**: BE, GR, LU, SZ, UR, UW, VD. **Fr**: AHP, AMa, Drô, Isè, Sav, HSav, Var, Vau. **It**: Frl, Ven, TAA, Lomb, Piem, VA, Lig. **Sl**: SlA, Tg.

***Bagliettoa caesiella* (Servít) *ined.* (provisionally placed here, ICN Art. 36.1b)**

Syn.: *Amphoridium caesiellum* (Servít) Servít, *Verrucaria caesiella* Servít, Verrucaria calciseda DC. f. caesia Anzi

L # – Subs.: cal – Alt.: 2–4 – Note: a calcicolous species closely related to *B. calciseda*, with a spreading, whitish mainly endolithic thallus, differing in the smaller, densely arranged ascomata (to 0.3 mm in diam.), and the ellipsoid ascospores measuring 18–21 × 10–12 μm; rarely collected, and perhaps more widespread in the Alps. – **Fr**: AMa. **It**: Lomb.

***Bagliettoa calciseda* (DC.) Gueidan & Cl. Roux**

Syn.: *Amphoridium calcisedum* (DC.) Servít, *Verrucaria calciseda* DC., Verrucaria calciseda DC. f. interrupta Anzi, Verrucaria calciseda DC. f. tuberculosa Servít, *Verrucaria hiascens* (Ach.) Hepp *non auct.*, *Verrucaria interrupta* (Anzi *ex* Arnold) J. Steiner *nom.illeg*.

L – Subs.: cal – Alt.: 1–4 – Note: on limestone and dolomite, more rarely on other calciferous rocks, often associated with *Aspicilia calcarea*; widespread throughout the Alps. – **Au**: V, T, S, K, St, O, N. **Ge**: OB, Schw. **Sw**: BE, GR, LU, SZ, UW, VD. **Fr**: AHP, HAl, AMa, Drô, Isè, Sav, HSav, Var, Vau. **It**: Frl, Ven, Lomb. **Sl**: SlA, Tg.

***Bagliettoa cazzae* (Zahlbr.) Vězda & Poelt**

Syn.: *Protobagliettoa alocyza* (Arnold) Servít, *Protobagliettoa cazzae* (Zahlbr.) Servít, *Verrucaria cazzae* Zahlbr., Verrucaria cazzae Zahlbr. var. graeca Servít

L – Subs.: cal – Alt.: 1–2 – Note: a chiefly Mediterranean lichen of steeply inclined to horizontal, hard calcareous rocks, absent from non-natural habitats, most frequent in the Southern and Western Alps at low elevations. – **Fr**: AHP, AMa, Var, Vau. **It**: Lomb.

***Bagliettoa crassiuscula* (Servít) Hafellner**

Syn.: *Amphoridium crassum* (Arnold) Servít, *Bagliettoa crassa* (Arnold) Cl. Roux, *Thelidium crassum* Arnold, Verrucaria calciseda DC. var. crassa (Arnold) Arnold, *Verrucaria crassa* A. Massal. 1852 *non* Eschw. 1833 *nom.illeg.*, *Verrucaria crassiuscula* Servít

L – Subs.: cal – Alt.: 2–3 – Note: a taxon of the *B. calciseda*-group with a thick, rugulose-verrucose thallus, and ellipsoid ascospores (12–18 × 6–7 μm, acc. to Servít even larger); the distribution is still insufficiently known. – **Fr**: AMa, Drô, Vau. **It**: Ven.

***Bagliettoa limborioides* A. Massal.**

Syn.: Bagliettoa sphinctrina *auct. non* (Ach.) Körb., Limboria sphinctrina *auct. non* (Ach.) Dufour, *Protobagliettoa grummannii* (Servít) Servít, *Thrombium limborioides* (A. Massal.) Zschacke, ?*Verrucaria bosniaca* Servít, Verrucaria bosniaca Servít f. albae Servít, *Verrucaria ceracea* J. Steiner, *Verrucaria grummannii* Servít, *Verrucaria limborioides* (A. Massal.) Clauzade & Cl. Roux, Verrucaria sphinctrina *auct. non* Ach., *Verrucaria sphinctrina* *auct.* var. bavarica Servít, *Verrucaria sphinctrina* *auct.* f. gallica Servít, *Verrucaria sphinctrina* *auct.* var. lojkae Servít, *Verrucaria sphinctrina* *auct.* var. tiroliensis Servít

L – Subs.: cal – Alt.: 1–3 – Note: a mild-temperate to Mediterranean lichen found on steeply inclined faces of compact calcareous rocks, and on small boulders; probably more widespread in the Alps, but overlooked, or confused with similar species. – **Au**: T, S, N. **Ge**: OB. **Sw**: ?BE, ?GR. **Fr**: AMa, Vau. **It**: Ven, TAA, Lig.

***Bagliettoa marmorea* (Scop.) Gueidan & Cl. Roux**

Syn.: *Amphoridium marmoreum* (Scop.) Baroni, Amphoridium marmoreum (Scop.) Baroni var. roseum (Kremp.) Syd., *Amphoridium purpurascens* (Hoffm.) A. Massal., *Lichen marmoreus* Scop., *Urceolaria wulfenii* Ach., *Verrucaria marmorea* (Scop.) Arnold, *Verrucaria purpurascens* Hoffm., Verrucaria purpurascens Hoffm. var. rosea A. Massal.

L – Subs.: cal – Alt.: 1–4 – Note: on hard, compact limestone rocks in natural habitats, with optimum below the montane belt; widespread throughout the Alps. – **Au**: T, S, K, St, O, N. **Ge**: OB. **Sw**: BE, UW. **Fr**: AHP, HAl, AMa, Drô, Isè, Sav, HSav, Var, Vau. **It**: Frl, Ven, TAA, Lomb, Lig. **Sl**: SlA, Tg.

***Bagliettoa parmigera* (J. Steiner) Vězda & Poelt**

Syn.: *Amphoridium saxivorum* (Servít) Grummann, *Protobagliettoa obscurata* (Servít) Servít, *Protobagliettoa parmigera* (J. Steiner) Servít, *Verrucaria gyelnikii* Servít, Verrucaria inaequata (Servít) Servít f. helvetica Servít, Verrucaria inaequata (Servít) Servít var. berchtesgadensis Servít, *Verrucaria parmigera* J. Steiner, Verrucaria parmigera J. Steiner f. geographica Servít, *Verrucaria saxivora* Servít, *Verrucaria subrosea* Servít

L – Subs.: cal – Alt.: 1–5 – Note: close to *B. baldensis* with which it is sometimes merged; a mainly mild-temperate lichen found on compact limestone and in exposed situations; widespread throughout the Alps, with optimum below the subalpine belt. – **Au**: V, T, S, K, St, O, N. **Ge**: OB. **Fr**: AHP, HAl, AMa, Drô, Isè, Sav, HSav, Var, Vau. **It**: Frl, Ven, TAA, Lomb, Piem, VA, Lig. **Sl**: SlA.

***Bagliettoa parmigerella* (Zahlbr.) Vězda & Poelt**

Syn.: *Protobagliettoa bagliettoaeformis* (Hazsl.) Servít, *Protobagliettoa erumpens* (Servít) Servít, *Protobagliettoa inaequata* (Servít) Servít, *Protobagliettoa parmigerella* (Zahlbr.) Servít, *Protobagliettoa sphinctrinella* (Zschacke) Servít, Verrucaria bagliettoaeformis (Hazsl.) Servít var. erumpens, *Verrucaria harrimannii* *sensu* Anzi, *Verrucaria inaequata* (Servít) Servít, *Verrucaria parmigerella* Zahlbr., *Verrucaria pinguis* J. Steiner, *Verrucaria sphinctrinella* Zschacke, Verrucaria sphinctrinella Zschacke f. loferensis Servít, Verrucaria sphinctrinella Zschacke f. viridis Servít, Verrucaria steineri Kušan var. inaequata Servít

L – Subs.: cal – Alt.: 1–4 – Note: a mild-temperate lichen found on compact limestone and dolomite in sheltered situations (*e.g.* in forests), with optimum in the submediterranean belt. – **Au**: V, S, St, O, N. **Ge**: OB. **Fr**: AHP, AMa, Drô, Isè, HSav, Var, Vau. **It**: Frl, Ven, TAA, Lig. **Sl**: SlA.

***Bagliettoa steineri* (Kušan) Vězda**

Syn.: *Protobagliettoa steineri* (Kušan) Servít *ex* J. Nowak & Tobol., *Verrucaria steineri* Kušan, Verrucaria steineri Kušan var. mittenwaldensis Servít

L – Subs.: cal – Alt.: 1–4 – Note: a mild-temperate species found on compact calcareous rocks, especially limestone, in natural habitats; frequently confused with *B. baldensis*. – **Au**: V, S, K, St, O, N. **Ge**: OB, Schw. **Sw**: BE, GR, LU, SZ. **Fr**: AHP, AMa, Drô, HSav, Var, Vau. **It**: Frl, Lomb. **Sl**: SlA.

***Bagliettoa suzaeana* (Servít) Gueidan & Cl. Roux**

Syn.: *Verrucaria suzaeana* Servít, Verrucaria suzaeana Servít var. sendtneriana Servít

L – Subs.: cal – Alt.: 1–2 – Note: this recently resurrected species is closely related to *B. parmigera*, differing in the less dense and more irregularly distributed perithecia. The species has not been distinguished in most of the earlier literature, so that its distribution is very poorly known. – **Ge**: OB. **Fr**: AMa, Var, Vau.

***Bellemerea alpina* (Sommerf.) Clauzade & Cl. Roux**

Syn.: *Aspicilia alpina* (Sommerf.) Arnold, *Lecanora alpina* Sommerf.

L – Subs.: sil, met, cor – Alt.: 4–6 – Note: a mainly arctic-alpine, circumpolar lichen of hard siliceous rocks wetted by rain near or above treeline; widespread throughout the siliceous Alps. – **Au**: V, T, S, K, St, N. **Ge**: Schw. **Sw**: BE, GR, SZ, TI, UR, VS. **Fr**: AHP, HAl, AMa, Isè, Sav, HSav. **It**: Frl, Ven, TAA, Lomb, Piem, VA.

***Bellemerea cinereorufescens* (Ach.) Clauzade & Cl. Roux**

Syn.: *Aspicilia cinereorufescens* (Ach.) A Massal., *Lecanora cinereorufescens* (Ach.) Hepp, *Urceolaria cinereorufescens* Ach.

L – Subs.: sil, met – Alt.: 3–6 – Note: a species with a wide range of genotypes resulting in a number of morphs, growing on metal-rich siliceous rocks at high elevations; widespread in the Holarctic region, including the Alps. – **Au**: V, T, S, K, St. **Ge**: Schw. **Sw**: BE, GR, SZ, TI, UR, VS. **Fr**: AHP, HAl, AMa, Isè, Sav, HSav. **It**: Frl, Ven, TAA, Lomb, Piem, VA.

***Bellemerea diamarta* (Ach.) Hafellner & Cl. Roux**

Syn.: *Aspicilia diamarta* (Ach.) Boistel, Lecanora cinereorufescens (Ach.) Hepp var. diamarta (Ach.) Nyl., *Lecanora diamarta* (Ach.) Vain., *Lecanora ferruginata* Harm., *Urceolaria diamarta* Ach.

L – Subs.: sil, met – Alt.: 3–6 – Note: an arctic-alpine, circumpolar lichen, somehow more hygro – and less photophytic than *B. alpina*. – **Au**: V, T, S, K, St. **Sw**: TI, VS. **Fr**: Sav, HSav. **It**: Ven, TAA, Lomb, Piem, VA.

***Bellemerea sanguinea* (Kremp.) Hafellner & Cl. Roux**

Syn.: *Aspicilia sanguinea* Kremp., *Lecanora incarnata* Kremp., *Lecanora sanguinea* (Kremp.) Mig.

L – Subs.: sil, met – Alt.: 3–6 – Note: a taxon of the *B. cinereorufescens*-group with a thin, rimose, grey thallus, aspicilioid, dark-red apothecia and larger ascospores; on schists with a slight content of calcium; the distribution is still poorly known. – **Au**: V, T, S, K. **Ge**: Schw. **Sw**: VS. **Fr**: AHP, HAl, AMa, Isè, Sav, HSav. **It**: TAA, Lomb, Piem.

***Bellemerea subcandida* (Arnold) Hafellner & Cl. Roux**

Syn.: Aspicilia cinereorufescens (Ach.) A. Massal. f. subcandida Arnold, Lecanora sanguinea (Kremp.) Mig. f. subcandida (Arnold) Mig., *Lecanora subcandida* (Arnold) Lettau

L – Subs.: cal, int – Alt.: 4–5 – Note: a probably overlooked and certainly more widespread, characteristic lichen of base-rich, weakly calciferous siliceous rocks; known from the Alps and the Pyrenees; perhaps just a calcicolous morph of *B. cinereorufescens*. – **Au**: V, T, S. **Sw**: VS. **Fr**: AHP, HAl, AMa, Sav, HSav. **It**: Ven, TAA, Piem, Lig.

***Bellemerea subnivea* (Müll. Arg.) Hafellner**

Syn.: *Aspicilia subnivea* (Müll. Arg.) Hue, *Lecanora subnivea* Müll. Arg.

L # – Subs.: cal, int – Alt.: 5 – Note: a species related to *B. cinereorufescens*, but the rimulose to areolate thallus pure white to bluish white (medulla I+ violet, thallus not reacting with K), with an effuse to subarachnoid marginal zone, apothecia (to *c.* 0.4 mm in diam) with dark brown discs and fully immersed in the areoles, 8-spored asci, and broadly ellipsoid, simple ascospores (*c.* 15 × 8–11 μm); on calcareous schist in the high alpine belt; so far only recorded from the Western Alps (Switzerland). – **Sw**: VD.

***Bellemerea subsorediza* (Lynge) R. Sant.**

Syn.: *Aspicilia subsorediza* (Lynge) R. Sant., *Lecidea subsorediza* Lynge

L – Subs.: sil, cor – Alt.: 3–6 – Note: on siliceous rocks in open lichen communities (*e.g.* near glaciers); probably more widespread in the Alps, but overlooked, being most often sterile. – **Au**: V, T, S, K, St. **It**: Frl, TAA.

***Biatora aureolepra* T. Sprib. & Tønsberg**

L – Subs.: cor – Alt.: 3 – Note: a recently-described species, which in Europe is only known from Central Norway and a single locality in the Eastern Alps (Austria). – **Au**: O.

***Biatora beckhausii* (Körb.) Tuck.**

Syn.: *Bacidia beckhausii* Körb., *Bacidia minuscula* Anzi, *Bacidia stenospora* (Hepp) Arnold, *Biatora stenospora* Hepp *Micarea beckhausii* (Körb.) Vězda, *Micarea minuscula* (Anzi) Vězda

L – Subs.: cor, xyl – Alt.: 2–4 – Note: a mainly mild-temperate lichen found on bark of broad-leaved trees (especially *Fraxinus*) in open, humid, mostly montane woodlands; widespread throughout the Alps. – **Au**: V, T, S, K, St, O, N. **Ge**: OB, Schw. **Sw**: BE, GR, SZ, UR, UW, VS. **Fr**: AHP, Sav, HSav. **It**: Frl, TAA, Lomb, Piem. **Sl**: SlA, Tg.

***Biatora brunnea* Anzi**

Syn.: *Lecidea brunnea* (Anzi) Stizenb.

L # – Subs.: sil – Alt.: 5–6 – Note: a species with a thick, well-delimited, verrucose-areolate, brown thallus, dark brown, sessile, presumably lecanorine apothecia becoming convex and immarginate, a yellowish hypothecium, conglutinate paraphyses with a brown cap, 8-spored asci, and simple ellipsoid ascospores with a thin episporium, measuring 13–15 *× c.* 7 µm; only known from the type collection (on mica-schist at high elevation), and well worthy of further study. Wrongly reported from Switzerland by Stizenberger, Lichenes Helvetici (1882–1883): the cited locality is in Italy. – **It**: Lomb.

***Biatora chrysantha* (Zahlbr.) Printzen**

Syn.: *Biatora epixanthoidiza* *auct.*, *Biatora gyrophorica* (Tønsberg) Coppins, *Lecidea chrysantha* Zahlbr., *Lecidea epixanthoidiza* *auct.*, *Lecidea gyrophorica* Tønsberg, *Lecidea incana* Ach. *ex* Sommerf.,

L – Subs.: cor, bry – Alt.: 3–4 – Note: on epiphytic bryophytes in humid forests, more rarely on bark or soil; widespread throughout the Alps. – **Au**: T, S, K, St, O, N. **Ge**: OB, Schw. **Sw**: BE, GL, GR, SZ, TI, UR, UW, VS. **Fr**: AHP, AMa. **It**: Frl, Ven, TAA, Piem, Lig. **Sl**: SlA.

***Biatora efflorescens* (Hedl.) Räsänen**

Syn.: *Biatora epixanthoidiza* (Nyl.) Räsänen, *Lecidea efflorescens* (Hedl.) Erichsen, *Lecidea epixanthoidiza* Nyl., *Lecidea helvola* (Körb. *ex* Hellb.) Th. Fr. f. efflorescens Hedl.

L – Subs.: cor, bry, xyl – Alt.: 2–4 – Note: a probably holarctic lichen found on a wide variety of trees with smooth bark, sometimes overgrowing mosses, rarely on lignum, mostly in upland areas; widespread throughout the Alps. – **Au**: V, T, S, K, St, O, N. **Ge**: OB, Schw. **Sw**: BE, GR, SZ, TI, UR, UW, VS. **Fr**: Isè. **It**: Frl, Ven, TAA, Lomb. **Sl**: SlA, Tg.

***Biatora fallax* Hepp**

Syn.: *Biatorina fallax* (Hepp) Hepp, Biatora vernalis (L) Fr. f. fallax (Hepp) Arnold, *Lecidea fallax* (Hepp) Linds.

L – Subs.: cor – Alt.: 3–4 – Note: a species with a granular-verrucose to microsquamulose thallus reacting Pd+ red; corticolous, on the base of coniferous trees; most records from the Alps are historical. – **Au**: T, S, K. **Ge**: OB, Schw. **Sw**: BE, GR, SZ, TI, UR, VS. **Sl**: SlA.

***Biatora flavopunctata* (Tønsberg) Hinteregger & Printzen**

Syn.: *Lecanora flavopunctata* Tønsberg

L – Subs.: cor – Alt.: 3–4 – Note: perhaps a boreal-montane species found on twigs of subalpine shrubs, especially *Rhododendron ferrugineum*. – **Au**: V, T, S, K, St. **Ge**: OB. **Sw**: BE, GL, GR, SZ, TI, UR, VS. **Fr**: Sav. **It**: Frl, Piem, Lig.

***Biatora fuscovirens* Bagl. & Carestia**

Syn.: *Lecidea fuscovirens* (Bagl. & Carestia) Lettau

L # – Subs.: cor – Alt.: 3 – Note: a species of uncertain affinity, with a spreading, granulose-verrucose, greenish thallus delimited by a brown prothallus, small rounded, isolated, soon immarginate apothecia with a reddish brown disc, adglutinate, rather thick paraphyses, a yellowish epihymenium, and elliptical, subacute ascospores which are *c.* 2 times as long as wide and *c.* ⅓ larger than those of *Trapeliopsis viridescens*, with which it was compared in the protologue; known only from the type collection, on *Castanea*. – **It**: Piem.

***Biatora globulosa* (Flörke) Fr.**

Syn.: *Bacidia globulosa* (Flörke) Hafellner & V. Wirth, *Bacidia pinguicula* (Bagl. & Carestia) Lettau, *Biatora hyalina* Fr., *Biatora minuta* (Schaer.) Hepp, *Biatora sylvana* Körb., *Biatorina globulosa* (Flörke) Körb., *Bilimbia pinguicula* Bagl. & Carestia, *Catillaria globulosa* (Flörke) Th. Fr., *Lecania globulosa* (Flörke) van den Boom & Sérus., *Lecania hyalina* (Fr.) R. Sant., *Lecidea globulosa* Flörke, *Lecidea minuta* (Schaer.) A. Massal., *Lecidea sylvana* (Körb.) Th. Fr.

L – Subs.: cor, xyl – Alt.: 2–4 – Note: a mainly temperate, perhaps holarctic lichen found on acid and rough bark of broad-leaved trees in sheltered situations, often in fissures, and in association with calicioid species; widespread throughout the Alps. – **Au**: V, T, S, K, St, O, N. **Ge**: OB, Schw. **Sw**: BE, FR, GL, GR, LU, SZ, TI, UR, UW, VD, VS. **Fr**: AHP, AMa, Isè, HSav, Var, Vau. **It**: Frl, Ven, TAA, Lomb, Piem, VA, Lig. **Sl**: SlA, Tg.

***Biatora helvola* Körb. *ex* Hellb.**

Syn.: *Lecidea helvola* (Körb. *ex* Hellb.) Hedl., Lecidea vernalis (L.) Ach. subsp. helvola (Körb. *ex* Hellb.) Th. Fr.

L – Subs.: cor, xyl – Alt.: 3–4 – Note: a mainly boreal-montane, circumpolar species found on basal parts of trees in open forests, often with *Parmeliopsis hyperopta*; widespread, to be looked for further in the Western Alps. – **Au**: V, T, S, K, St, O, N. **Ge**: OB, Schw. **Sw**: BE, SZ, UR, UW, VD, VS. **It**: Frl, Ven, TAA. **Sl**: SlA, Tg.

***Biatora hemipolia* (Nyl.) S. Ekman & Printzen**

Syn.: *Bacidia hemipolia* (Nyl.) Malme, Lecidea arceutina (Ach.) Gray f. hemipolia Nyl., *Lecidea hemipolia* (Nyl.) Nyl.

L – Subs.: cor – Alt.: 2–3 – Note: a species of the *B. beckhausii*-*B. globulosa*-group with acicular ascospores; on bark of deciduous trees, with a few scattered records from the Alps. – **Ge**: Ge. **Fr**: Vau.

***Biatora holomicra* Anzi**

Syn.: *Lecidea holomicra* (Anzi) Jatta

L # – Subs.: cor – Alt.: 3–4 – Note: a species with a thin grey thallus, hemispherical, small, black apothecia, a brownish-yellow hypothecium, and small, narrowly elliptical ascospores measuring *c.* 7.5 × 3.5 µm. Most probably a *Micarea*. – **It**: Lomb.

***Biatora meiocarpa* (Nyl.) Arnold**

Syn.: *Lecidea meiocarpa* Nyl.

L – Subs.: cor, xyl – Alt.: 2–4 – Note: this species was often confused with *B. helvola*, differing in the C – thallus (C+ red in *B. helvola*), the thicker paraphysal tips, and the filiform, often curved conidia (bacilliform and straight in *B. helvola*); on smooth bark of deciduous trees; from the Alps there are only a few scattered records, the older ones from Switzerland are dubious. – **Sw**: SZ, VS. **Fr**: AMa.

***Biatora mendax* Anzi**

Syn.: *Biatora propinquata* (Nyl.) Arnold, *Biatora subflavida* (Nyl.) Arnold, *Biatorina mendax* (Anzi) Jatta, *Catillaria mendax* (Anzi) Lettau, *Lecidea mendax* (Anzi) Hue, *Lecidea propinquata* Nyl., *Lecidea subflavida* Nyl.

L – Subs.: cor – Alt.: 3–4 – Note: an epiphytic species found in shaded and humid situations, with optimum in humid beech forests with *Abies alba*. – **Au**: T, S, K, St, N. **Sw**: BE. **It**: Frl, TAA, Lomb. **Sl**: SlA, Tg.

***Biatora ocelliformis* (Nyl.) Arnold**

Syn.: *Biatora atroviridis* (Arnold) Hellb., *Biatorina subglobulosa* (Nyl.) Arnold, *Bilimbia ocelliformis* (Nyl.) Branth & Rostr., *Lecidea admixta* Kullh., *Lecidea atroviridis* (Arnold) Th. Fr., *Lecidea ocelliformis* Nyl., *Lecidea subglobulosa* Nyl., Lecidea turgidula Fr. var. atroviridis Arnold

L – Subs.: cor – Alt.: 2–4 – Note: a boreal-montane species found on the bark of deciduous and coniferous trees in montane to subalpine forests. – **Au**: V, T, S, K, St, O, N. **Ge**: OB. **Sw**: BE, UW, VS. **Fr**: AHP, AMa. **It**: TAA, Piem. **Sl**: SlA, Tg.

***Biatora pontica* Printzen & Tønsberg**

L – Subs.: cor – Alt.: 3 – Note: described from Turkey, and also known from Europe and Eastern North America, this species occurs on acid to subacid bark in shaded and humid situations within old montane forests, mainly on *Fagus* and *Abies*; so far known only from the Eastern Alps (Austria, Italy, Slovenia). – **Au**: T, St. **It**: Frl. **Sl**: SlA.

***Biatora rufidula* (Graewe) S. Ekman & Printzen**

Syn.: *Bacidia rufidula* (Graewe) Zahlbr., *Bilimbia rufidula* Graewe, *Lecidea rufidula* (Graewe) Stizenb.

L – Subs.: cor – Alt.: 4 – Note: a boreal-montane lichen, restricted to *Picea abies* in the subalpine belt of the Alps; probably more widespread in the Alps, but perhaps declining. – **Ge**: OB. **It**: TAA.

***Biatora sphaeroidiza* (Vain.) Printzen & Holien**

Syn.: *Lecidea sphaeroidiza* Vain.

L – Subs.: cor – Alt.: 3–4 – Note: a boreal-montane species which occurs both on conifers and deciduous trees and shrubs (*e.g. Alnus*, *Salix*, *Sorbus*, *Vaccinium*) in rather humid areas, only reported from the Eastern Alps (Slovenia). – **Sl**: SlA.

***Biatora subduplex* (Nyl.) Räsänen *ex* Printzen**

Syn.: Biatora vernalis (L.) Fr. f. subduplex (Nyl.) Arnold, *Catillaria subduplex* (Nyl.) H. Olivier, *Lecidea apochroeiza* Nyl., *Lecidea internectens* Nyl., *Lecidea subduplex* (Nyl.) Nyl., Lecidea vernalis (L.) Ach. f. subduplex Nyl., Lecidea vernalis (L.) Ach. var. subduplex (Nyl.) Vain.

L – Subs.: deb, bry, cor – Alt.: 3–5 – Note: one of the commonest *Biatora*-species in the Alps, especially on plant remains and on basal parts of subalpine shrubs. See also note on *B. vernalis*. – **Au**: V, T, S, K, St, O, N. **Ge**: OB, Schw. **Sw**: BE, GR, SZ, TI, UR, VD, VS. **Fr**: AMa, Sav, HSav. **It**: Frl, Ven, TAA, Lomb, Piem, VA, Lig. **Sl**: SlA, Tg.

***Biatora subgilva* (Arnold) Hinteregger**

Syn.: Biatora vernalis (L.) Fr. f. subgilva (Arnold) Arnold, Biatora vernalis (L.) Fr. var. subgilva Arnold, Lecidea vernalis (L.) Ach. f. subgilva (Arnold) Zahlbr.

L – Subs.: cor – Alt.: 4 – Note: a rare species growing on old, decaying branches and stems of *Rhododendron* in areas with siliceous substrata; in the study area it is so far known only from the Eastern Alps (Austria, Italy). – **Au**: V, T. **It**: Frl.

***Biatora vacciniicola* (Tønsberg) Printzen**

Syn.: *Lecidea vacciniicola* Tønsberg

L – Subs.: cor – Alt.: 3–5 – Note: a species with a sorediate, often sterile, partly endophloeodic thallus and confluent soralia reacting C+ red; on bark of various trees, mostly near the base and on branches of dwarf shrubs, often with *B. subduplex*; from the Alps there are only a few scattered records. – **Au**: T, S. **Sw**: BE, GR, VS.

***Biatora valerii* Anzi**

Syn.: *Lecidea valerii* (Anzi) Jatta

L # – Subs.: sil – Alt.: 3–4 – Note: a species with a pinkish white, orbicular, thick, almost pulvinate, plicate thallus, the individual thalli solitary to confluent, 5–8 mm across, 2–4 mm tall, apothecia and spores as in *Lecanora polytropa*; only known from the type collection, on schist, this taxon would be well worthy of further study. – **It**: Lomb.

***Biatora vernalis* (L.) Fr.**

Syn.: *Bacidia vernalis* (L.) Clauzade & Rondon, Biatora sphaeroides (Dicks.) Hornem. var. vernalis (L.) Rabenh., *Bilimbia vernalis* (L.) Trevis., *Lecidea vernalis* (L.) Ach., *Lichen vernalis* L., *Patellaria vernalis* (L.) Spreng., *Pyrrhospora vernalis* (L.) M. Choisy, *Secoliga vernalis* (L.) Norman

L – Subs.: cor, bry, deb, ter-sil – Alt.: 3–4 – Note: a mostly boreal-montane, circumpolar species, ranging from Northern Scandinavia to the Alps, the Pyrenees and the Balkan mountains, becoming progressively rarer southwards, found on bryophytes, plant debris, soil and bark. Several records could refer to *B. subduplex*; widespread throughout the Alps. – **Au**: V, T, S, K, St, N. **Ge**: OB, Schw. **Sw**: BE, GR, ?SZ, VD, VS. **Fr**: AHP, HAl, AMa, Isè, Sav. **It**: Frl, Ven, TAA, Lomb, Piem, VA. **Sl**: SlA, Tg.

***Biatora veteranorum* Coppins & Sérus.**

Syn.: *Catillaria alba* Coppins & Vězda

L – Subs.: xyl – Alt.: 2–4 – Note: a mild-temperate lichen found on decorticated trunks of old deciduous trees protected from rain, with a few scattered records from the Alps. – **Au**: T. **Sw**: GL, GR, SZ, TI. **It**: TAA.

***Biatorella fossarum* (Dufour *ex*Fr.) Arnold.**

Syn.: *Biatora rousselii* (De Not.) Durieu & Mont., *Biatorella rousselii* De Not., *Lecidea fossarum* Dufour *ex* Fr.

L # – Subs.: ter-int – Alt.: 1–3 – Note: closely related to *B. hemisphaerica*; on slightly calciferous, often strongly decalcified soil in rather disturbed habitats; chiefly southern in Europe, with a few scattered records from the Alps. – **Ge**: OB, Schw. **Fr**: Drô. **It**: Ven, TAA.

***Biatorella germanica* A. Massal. *ex* Körb.**

L – Subs.: cal – Alt.: 3–4 – Note: on sheltered calcareous rocks; perhaps overlooked, but certainly not common, from the Alps there are only a few scattered records. – **Ge**: Schw. **Sw**: VD. **It**: Frl, Piem.

***Biatorella hemisphaerica* Anzi**

Syn.: *Biatorella fossarum* (Dufour *ex* Fr.) Arnold. var. rubicunda (Th. Fr.) Th. Fr.

L – Subs.: ter-cal, bry – Alt.: 2–5 – Note: on calciferous soil and amongst bryophytes, most often in rock fissures; widespread in the Alps, but generally not very common. – **Au**: T, S, St, O, N. **Ge**: OB, Schw. **Sw**: GR, SZ, VD, VS. **It**: Frl, Ven, TAA, Lomb, Lig.

***Biatorella heterospora* Kalb & Vězda**

L – Subs.: cor – Alt.: 3 – Note: a corticolous species, peculiar in having a inspersed epihymenium with olive-blue granules, and ellipsoid ascospores of various shape; apparently rare (for the Alps reported only from Austria), and ecology therefore insufficiently known. – **Au**: K, St.

***Biatorella microhaema* Norman**

Syn.: *Strangospora microhaema* (Norman) R.A. Anderson

L – Subs.: cor, xyl – Alt.: 3–4 – Note: a cool-temperate to boreal-montane, perhaps circumpolar species with minute, blood-red, immarginate apothecia, whose taxonomic position is still unresolved; on base-rich bark and slightly eutrophicated lignum with a few scattered records from the Alps. – **Au**: V, T, St. **Ge**: OB. **It**: TAA.

***Biatorella tiroliensis* H. Magn.**

L # – Subs.: ter-cal – Alt.: 4 – Note: a terricolous species, probably belonging to the *B. germanica*-group, but with fewer ascospores per ascus, only known from the type locality in Austria. – **Au**: T.

***Biatoridium delitescens* (Arnold) Hafellner**

Syn.: *Biatorella delitescens* Arnold, *Strangospora delitescens* (Arnold) Coppins

L – Subs.: cor – Alt.: 3 – Note: a species differing from the more common *B. monasteriense* in the exciple lacking attached thallus granules, and the paraphyses without enlarged tips; on bark in *Xanthorion*-communities; distribution insufficiently known, but apparently rare in the Alps. – **Au**: St. **Ge**: Schw. **Sw**: BE.

***Biatoridium monasteriense* J. Lahm *ex* Körb**

Syn.: *Biatorella elegans* (A. Massal.) Stizenb., *Biatorella monasteriensis* (J. Lahm *ex* Körb.) J. Lahm, *Biatoridium elegans* (A. Massal.) Reinke, *Chiliospora elegans* A. Massal.

L – Subs.: cor, xyl – Alt.: 1–3 – Note: a mild-temperate lichen found on deciduous trees with base-rich bark (*Acer*, *Fraxinus*, *Sambucus*); much overlooked in the past, but locally not infrequent on *Sambucus* along brooks, and widespread throughout the Alps. – **Au**: V, T, S, K, St, O, N. **Ge**: OB, Schw. **Sw**: GL, GR, SZ, VS. **Fr**: AMa, Var, Vau. **It**: Frl, Ven. **Sl**: SlA, Tg.

***Bilimbia accedens* Arnold**

Syn.: *Bacidia accedens* (Arnold) Lettau, *Mycobilimbia accedens* (Arnold) V. Wirth *ex* Hafellner, *Myxobilimbia accedens* (Arnold) Hafellner

L – Subs.: bry, deb – Alt.: 3–5 – Note: on mosses overgrowing soil and rocks, with optimum on calciferous substrata in upland areas. **Au**: T, S, K, St, O, N. **Ge**: OB, Schw. **Fr**: HAl, AMa. **It**: Frl, TAA, Lomb, Piem. **Sl**: SlA.

***Bilimbia lobulata* (Sommerf.) Hafellner & Coppins**

Syn.: *Bacidia sabulosa* (A. Massal.) Lettau, *Biatora regeliana* Hepp, *Bilimbia leucophaea* A.L. Sm., *Bilimbia regeliana* (Hepp) Körb., *Bilimbia sabulosa* A. Massal., *Bilimbia syncomista* (Flörke) Körb., *Catillaria subnegans* (Nyl.) Arnold, *Lecidea claudeliana* Harm., *Lecidea lobulata* Sommerf., *Lecidea subnegans* Nyl., *Lecidea syncomista* (Flörke) Nyl., *Mycobilimbia lobulata* (Sommerf.) Hafellner, *Myxobilimbia lobulata* (Sommerf.) Hafellner, *Toninia claudeliana* (Harm.) H. Olivier, *Toninia lobulata* (Sommerf.) Lynge, *Toninia sabulosa* (A. Massal.) Samp., *Toninia syncomista* (Flörke) Th. Fr.

L – Subs.: ter-cal, bry-cal, deb – Alt.: 2–6 – Note: a cool-temperate to arctic-alpine, circumpolar lichen found on terricolous mosses and bare calciferous soil, from the Alps to the high Mediterranean mountains; widespread and locally common throughout the Alps. – **Au**: V, T, S, K, St, O, N. **Ge**: OB, Schw. **Sw**: BE, FR, GR, LU, SZ, TI, VD, VS. **Fr**: AHP, HAl, AMa, Isè, Sav, HSav, Vau. **It**: Frl, Ven, TAA, Lomb, Piem, VA, Lig. **Sl**: SlA. **Li**.

***Bilimbia microcarpa* (Th. Fr.) Th. Fr.**

Syn.: *Bacidia hypnophila* (Turner *ex* Ach.) Zahlbr. subsp. microcarpa (Th. Fr.) Th. Fr., *Bacidia microcarpa* (Th. Fr.) Lettau, Bacidia obscurata (Sommerf.) Zahlbr. var. microcarpa Th. Fr., *Bilimbia hypnophila* (Turner *ex* Ach.) Th. Fr., subsp. microcarpa (Th. Fr.) Th. Fr., Bilimbia obscurata (Sommerf.) Th. Fr. var. microcarpa Th. Fr., *Lecidea meiobola* Nyl., *Lecidea microcarpa* (Th. Fr.) Vain., *Mycobilimbia microcarpa* (Th. Fr.) Brunnb., *Myxobilimbia microcarpa* (Th. Fr.) Hafellner

L – Subs.: deb, bry – Alt.: 3–5 – Note: an arctic-alpine lichen found on mosses in dry grasslands, sometimes on epilithic bryophytes, with optimum near or above treeline; widespread throughout the Alps. – **Au**: V, T, S, K, St, O, N. **Ge**: OB, Schw. **Sw**: GR, LU, SZ, TI, UR, UW. **Fr**: AHP, HAl, Isè, Sav, Vau. **It**: Frl, Ven, TAA, Lomb, Piem, Lig. **Sl**: SlA.

**Bilimbia sabuletorum (Schreb.) Arnold var. sabuletorum**

Syn.: *Bacidia borborodes* (Körb.) Lettau, *Bacidia descendens* (Stizenb.) Mig., *Bacidia hypnophila* (Turner *ex* Ach.) Zahlbr., *Bacidia propinqua* (Stizenb.) Arnold, *Bacidia sabuletorum* (Schreb.) Lettau, *Biatora propinqua* Stizenb., *Bilimbia borborodes* Körb., *Bilimbia hexamera* De Not., *Bilimbia hypnophila* (Turner *ex* Ach.) Th. Fr., *Lecidea hypnophila* Turner *ex* Ach., *Lecidea sabuletorum* (Schreb.) Ach., *Lichen sabuletorum* Schreb., *Mycobilimbia sabuletorum* (Schreb.) Hafellner, *Myxobilimbia sabuletorum* (Schreb.) Hafellner

L – Subs.: bry, deb, ter-cal, xyl, cor – Alt.: 1–5 – Note: a holarctic, mainly temperate lichen found on mosses overgrowing soil and calciferous rocks, and tree bark, also in urban environments (*e.g.* on walls); widespread and locally common throughout the Alps. – **Au**: V, T, S, K, St, O, N, B. **Ge**: OB, Schw. **Sw**: BE, GL, GR, LU, SG, SZ, UR, UW, VD, VS. **Fr**: AHP, AMa, Drô, Isè, Sav, HSav, Var, Vau. **It**: Frl, Ven, TAA, Lomb, Piem, VA, Lig. **Sl**: SlA, Tg.

**Bilimbia sabuletorum (Schreb.) Arnold var. dolosa (Duby) *ined.* (provisionally placed here, ICN Art. 36.1b)**

Syn.: Bacidia sabuletorum (Schreb.) Lettau var. dolosa (Duby) V. Wirth, Bacidia sabuletorum (Schreb.) Lettau f. dolosa (Duby) Zahlbr., *Bilimbia dolosa* (Duby) Dalla Torre & Sarnth., Bilimbia sabuletorum (Schreb.) Arnold f. dolosa (Duby) Arnold, *Patellaria dolosa* Duby

L # – Subs.: bry, deb – Alt.: 3–5 – Note: closely related to (or identical with) the typical variety; probably widespread, but poorly documented because most often not distinguished from var. sabuletorum. – **Au**: V, T, O, N.

***Blennothallia crispa* (Huds.) Otálora, P.M. Jørg. & Wedin var. crispa**

Syn.: *Blennothallia cheilea* (Ach.) Trevis., *Collema cheileum* (Ach.) Ach., *Collema conchilobum* (Flot.) Körb., *Collema crispum* (Huds.) Weber *ex* F.H. Wigg. var. crispum, *Lichen crispus* Huds.

L – Subs.: cal, ter-cal – Alt.: 1–5 – Note: a mainly mild-temperate lichen found both on calcareous rocks and soil, often in rather disturbed habitats such as walls in villages below the subalpine belt, sometimes reaching beyond treeline; widespread throughout the Alps. – **Au**: V, T, S, K, St, O, N. **Ge**: OB, Schw. **Sw**: BE, SZ, TI, UW, VD, VS. **Fr**: AHP, AMa, Isè, Sav, HSav, Var, Vau. **It**: Frl, Ven, TAA, Lomb, Piem, VA, Lig. **Sl**: SlA.

***Blennothallia crispa* (Huds.) Otálora, P.M. Jørg. & Wedin var. metzleri (Arnold) *ined.* (provisionally placed here, ICN Art. 36.1b)**

Syn.: Collema cheileum (Ach.) Ach. var. metzleri Arnold, *Collema crispum* (Huds.) Weber *ex* F.H. Wigg. var. metzleri (Arnold) Degel., *Collema metzleri* (Arnold) J. Steiner, *Collema monocarpum* Dufour *ex* Schaer., *Collema platycarpum* Durieu & Mont.

L – Subs.: cal, ter-cal – Alt.: 1–3 – Note: a taxon characterised by constantly small thalli and one to few apothecia per thallus; on calcareous rocks, often in somewhat shaded and moist situations; probably widespread, but poorly documented because often not distinguished from the typical variety. – **Au**: K, St. **Sw**: SZ. **Fr**: Vau.

***Botryolepraria lesdainii* (Hue) Canals, Hern.-Mar., Gómez-Bolea & Llimona**

Syn.: *Crocynia lesdainii* Hue, *Lepraria aeruginosa* *sensu* Sm. *non* (Weiss) Sm., *Lepraria lesdainii* (Hue) R.C. Harris

L – Subs.: cal, cor – Alt.: 2–3 – Note: a leprarioid species with a peculiar micromorphology, the thallus being composed of minute, shrub-like clusters with subterminal groups of algal cells; usually on calcareous rocks in full shade; widespread, but not common in the Alps. – **Au**: S, K, St, O. **Sw**: LU. **Fr**: AHP, AMa, Drô, Var, Vau. **It**: Frl, Ven, Lomb. **Sl**: SlA.

***Brianaria bauschiana* (Körb.) S. Ekman & M. Svenss.**

Syn.: *Biatora bauschiana* Körb., *Biatora rusticella* (Nyl.) Walt. Watson, *Biatora semipallens* (Nyl.) Walt. Watson, *Catillaria microspora* Maslowa, *Lecidea bauschiana* (Körb.) Lettau, *Lecidea dilutiuscula* Nyl., *Lecidea infidula* Nyl., *Lecidea rusticella* Nyl., *Lecidea semipallens* Nyl., *Micarea bauschiana* (Körb.) V. Wirth & Vězda

L – Subs.: sil, ter-sil, cor – Alt.: 2–3 – Note: on a wide variety of substrata (rocks, exposed roots, consolidated soil) in shaded-dry situations (*e.g.* in underhangs), but restricted to humid areas. – **Au**: S, K, St, O, N. **Ge**: OB. **Fr**: Var. **It**: Ven, TAA, Lomb, Lig.

***Brianaria lutulata* (Nyl.) S. Ekman & M. Svenss.**

Syn.: *Biatora anthrophila* (Larbal. *ex* Leight.) Walt. Watson, *Biatora paucula* (Nyl.) Walt. Watson, *Lecidea anthrophila* Larbal. *ex* Leight., *Lecidea botryiza* Nyl. *ex* Stirt., *Lecidea laxula* Nyl., *Lecidea lutulata* Nyl., *Lecidea paucula* Nyl., *Lecidea poliodes* Nyl., *Micarea lutulata* (Nyl.) Coppins, *Micarea poliodes* (Nyl.) Vězda, *Micarea umbrosa* Vězda & V. Wirth

L – Subs.: sil, met – Alt.: 2–4 – Note: on siliceous, often metal-rich rocks in dry and sheltered underhangs, in humid natural habitats; widespread, but generally not common in the Alps. – **Au**: ?V, S, K, St. **Sw**: VS. **Fr**: AMa. **It**: Frl, VA.

***Brianaria sylvicola* (Flot. *ex* Körb.) S. Ekman & M. Svenss.**

Syn.: *Biatora smaragdina* Arnold, *Biatora sylvicola* (Flot. *ex* Körb.) Müll. Arg., *Lecidea aggerata* Mudd, *Lecidea hellbomii* J. Lahm, *Lecidea hypocyanea* Vain. *non* Stirt., *Lecidea incincta* Nyl., *Lecidea sylvicola* Flot. *ex* Körb., *Lecidea vainioi* H. Magn., *Micarea sylvicola* (Flot. *ex* Körb.) Vězda & V. Wirth

L – Subs.: sil, xyl – Alt.: 2–4 – Note: on shaded, humid siliceous rocks, in underhangs, *e.g.* in forests; widespread in the Alps. – **Au**: V, T, S, K, St, N. **Sw**: UR, VS. **Fr**: HAl, HSav. **It**: Frl, TAA, Lomb, VA. **Sl**: SlA.

***Brianaria tuberculata* (Sommerf.) S. Ekman & M. Svenss.**

Syn.: *Lecidea botryocarpa* Nyl., *Lecidea latens* Taylor, *Lecidea subinfidula* Nyl., *Lecidea tuberculata* Sommerf., *Micarea tuberculata* (Sommerf.) R.A. Anderson

L – Subs.: sil, cor – Alt.: 2–5 – Note: on siliceous rocks in humid forests, but also on exposed roots, mostly under overhangs, with a few scattered records from the Alps. – **Au**: V, T, K, St, N. **Fr**: AMa. **It**: TAA.

***Brodoa atrofusca* (Schaer.) Goward**

Syn.: *Hypogymnia atrofusca* (Schaer.) Räsänen, Hypogymnia intestiniformis (Vill.) Räsänen var. atrofusca (Schaer.) Poelt, *Parmelia atrofusca* (Schaer.) Cromb., Parmelia ceratophylla Schaer. var. atrofusca Schaer., Parmelia intestiniformis (Vill.) Ach. var. atrofusca (Schaer.) Hasselrot

L – Subs.: sil – Alt.: 3–5 – Note: on wind-exposed siliceous rocks wetted by rain near or above treeline, less bound to a long snow cover than *B. intestiniformis*; widespread throughout the siliceous Alps. – **Au**: V, T, S, K, St. **Sw**: BE, GR, TI, UR, VS. **Fr**: AHP, HAl, AMa, Isè, HSav. **It**: Frl, Ven, TAA, Lomb, Piem, VA.

***Brodoa intestiniformis* (Vill.) Goward**

Syn.: *Hypogymnia encausta* (Sm.) Walt. Watson, *Hypogymnia intestiniformis* (Vill.) Räsänen, *Imbricaria encausta* (Sm.) DC., *Lichen intestiniformis* Vill., *Menegazzia encausta* (Sm.) Navàs, *Parmelia encausta* (Sm.) Ach., *Parmelia intestiniformis* (Vill.) Ach.

L – Subs.: sil – Alt.: 3–5 – Note: an arctic-alpine to boreal-montane, circumpolar lichen found in more sheltered and less wind-exposed situations than *B. atrofusca*, on faces of acid siliceous rocks with a long snow-cover, with optimum above treeline; widespread and locally rather common throughout the Alps. – **Au**: V, T, S, K, St, N. **Sw**: BE, GR, LU, SZ, TI, UR, VD, VS. **Fr**: AHP, HAl, AMa, Isè, Sav, HSav. **It**: Frl, Ven, TAA, Lomb, Piem, VA. **Sl**: SlA.

***Bryobilimbia hypnorum* (Lib.) Fryday, Printzen & S. Ekman**

Syn.: *Biatora atrofusca* Hepp, *Biatora cartilaginea* Lönnr., *Lecidea atrofusca* (Hepp) Mudd, *Lecidea fusca* (Schaer.) Th. Fr., *Lecidea hypnorum* Lib., *Lecidea sanguineoatra* *sensu* Nyl. *non* (Wulfen) Ach., *Lecidea templetonii* Taylor, *Mycobilimbia hypnorum* (Lib.) Kalb & Hafellner

L – Subs.: bry, ter-cal, deb, cor, xyl – Alt.: 2–5 – Note: a cool-temperate to arctic-alpine, probably circumpolar lichen found on mosses, plant debris, soil, bark and lignum, especially in upland areas with calcareous substrata; widespread and common throughout the Alps. – **Au**: V, T, S, K, St, O, N. **Ge**: OB, Schw. **Sw**: BE, GR, LU, SZ, TI, UR, UW, VD, VS. **Fr**: AHP, HAl, AMa, Isè, Sav, HSav, Var. **It**: Frl, Ven, TAA, Lomb, Piem, VA, Lig. **Sl**: SlA, Tg.

***Bryobilimbia sanguineoatra* (Wulfen) Fryday, Printzen & S. Ekman**

Syn.: *Lecidea sanguineoatra* (Wulfen) Ach., *Lichen sanguineoater* Wulfen, *Mycobilimbia sanguineoatra* (Wulfen) Kalb & Hafellner *nom.illeg*.

L – Subs.: cor, bry – Alt.: 2–5 – Note: optimum in open humid forests, on mosses at the base of old boles, sometimes on soil. – **Au**: V, St, O, N. **Ge**: Ge. **Sw**: BE, GL, GR, SZ, TI, UR. **Fr**: AHP, Isè, HSav. **It**: Piem. **Sl**: SlA, Tg.

***Bryodina rhypariza* (Nyl.) Hafellner & Türk**

Syn.: *Bryonora rhypariza* (Nyl.) Poelt, *Lecanora rhypariza* Nyl.

L – Subs.: bry – Alt.: 4–5 – Note: a mainly arctic-alpine species found on mosses (*Andreaea*, *Grimmia*) near or above treeline, often associated with cyanobacteria (*Stigonema*). – **Au**: V, T, S, K, St. **Sw**: BE, GL, GR, UR, VS. **Fr**: HAl. **It**: TAA, Lomb, Piem, VA.

***Bryonora castanea* (Hepp) Poelt**

Syn.: *Biatora castanea* Hepp, *Lecanora castanea* (Hepp) Th. Fr.

L – Subs.: deb, bry, ter-sil, ter-cal – Alt.: 4–6 – Note: a mainly arctic-alpine, circumpolar lichen found on soil, mosses, plant remains and on other lichens in Alpine grasslands, mostly in sites with a long snow cover, on siliceous substrata; widespread in the siliceous Alps. – **Au**: V, T, S, K, St, O, N. **Sw**: GR, UR, TI, VS. **Fr**: AHP, HAl, Sav. **It**: Frl, Ven, TAA, Lomb, Piem, VA. **Sl**: SlA.

***Bryonora corallina* Poelt**

L # – Subs.: bry – Alt.: 5 – Note: thallus consisting of minute, densely aggregated “podetia”; based on a sterile type (ascomata still unknown), hence taxonomic placement uncertain; on decaying tufts of mosses over siliceous rocks; known only from a single locality in the Austrian Alps. – **Au**: K.

***Bryonora curvescens* (Mudd) Poelt**

Syn.: *Biatora curvescens* (Mudd) Th. Fr., *Lecania curvescens* (Mudd) A.L. Sm., Lecanora castanea (Hepp) Th. Fr. f. curvescens (Mudd) Th. Fr., *Lecanora curvescens* (Mudd) Nyl., *Pannaria curvescens* Mudd

L – Subs.: bry, ter-sil – Alt.: 4–6 – Note: an arctic-alpine, circumpolar lichen found on bryophytes (*e.g. Andreaea*, *Grimmia*) in sites with periodic seepage of water, with optimum above treeline on siliceous substrata, with a few scattered records from the Alps. – **Au**: T, S. **Sw**: GR. **It**: TAA.

***Bryonora pruinosa* (Th. Fr.) Holt.-Hartw.**

Syn.: Lecanora castanea (Hepp) Th. Fr. var. pruinosa Th. Fr.

L – Subs.: bry, deb – Alt.: 4–5 – Note: similar to *B. castanea*, but apothecial margin darker than the occasionally pruinose disc, and ascospores less than 16 µm long; on plant debris and moribund lichens; in Europe it has an arctic-alpine distribution, but is apparently rare in the Alps. – **Au**: T. **Sw**: GR, LU, SZ, VS.

***Bryoria bicolor* (Ehrh.) Brodo & D. Hawksw.**

Syn.: *Alectoria bicolor* (Ehrh.) Nyl., *Bryopogon bicolor* (Ehrh.) Elenkin, Bryopogon jubatus (L.) Link var. bicolor (Ehrh.) Rabenh., *Cornicularia bicolor* (Ehrh.) Ach., *Lichen bicolor* Ehrh.

L – Subs.: cor, bry, sil – Alt.: 3–5 – Note: a mainly boreal-montane, circumpolar lichen found on mossy trunks of old, more or less isolated trees in areas with frequent fog, sometimes on mossy rocks; widespread throughout the Alps, but perhaps declining. – **Au**: V, T, S, K, St, O, N. **Ge**: OB, Schw. **Sw**: BE, FR, GL, GR, LU, SZ, UR, UW, VD, VS. **Fr**: HAl, Isè, Sav, HSav. **It**: Frl, Ven, TAA, Lomb, Piem. **Sl**: SlA.

***Bryoria capillaris* (Ach.) Brodo & D. Hawksw.**

Syn.: *Alectoria cana* (Ach.) Leight., *Alectoria capillaris* (Ach.) Cromb., *Alectoria implexa* *auct.*, *Alectoria setacea* (Ach.) Motyka, *Bryopogon canum* (Ach.) M. Choisy, *Bryopogon capillaris* (Ach.) Bystrek, *Bryoria setacea* (Ach.) Brodo & D. Hawksw., Parmelia jubata (L.) Ach. var. capillaris Ach.

L – Subs.: cor, sil – Alt.: 3–4 – Note: a cool-temperate to boreal-montane, circumpolar lichen, with optimum in humid *Fagus*-*Abies* forests, mostly on twigs, but also on boles of isolated trees in areas with frequent fog; widespread and still locally common throughout the Alps, but perhaps declining. – **Au**: V, T, S, K, St, O, N. **Ge**: OB, Schw. **Sw**: BE, GR, LU, SZ, TI, VD, VS. **Fr**: AHP, HAl, AMa, Isè, Sav, HSav, Vau. **It**: Frl, Ven, TAA, Lomb, Piem, VA, Lig. **Sl**: SlA.

***Bryoria carpatica* (Motyka) Bystrek**

Syn.: *Alectoria carpatica* Motyka

L # – Subs.: cor – Alt.: 3 – Note: a species similar and/or related to *B. tortuosa*, with grey to brown, dull, irregularly branched, contorted and flexuous, Pd+ yellow thalli with numerous pseudocyphellae; in moist montane forests *e.g.* along streams; rare in the Central European mountains, including the Eastern Alps (Austria, Switzerland). – **Au**: N. **Sw**: GR.

***Bryoria chalybeiformis* (L.) Brodo & D. Hawksw.**

Syn.: *Alectoria chalybeiformis* (L.) Röhl., Alectoria jubata (L.) Ach. var. chalybeiformis (L.) Ach., *Alectoria prostratosteola* Gyeln., *Bryopogon chalybeiforme* (L.) Link, *Lichen chalybeiformis* L.

L # – Subs.: sil, ter-sil, xyl, deb, bry – Alt.: 3–5 – Note: an arctic-alpine to boreal-montane, circumpolar lichen found on wind-exposed rocks, but also on soil, mosses and plant remains in exposed habitats with frequent fog, with optimum near and above treeline. It could be just a terricolous morph of *B. fuscescens*; widespread throughout the Alps. – **Au**: V, T, S, K, St, N. **Ge**: OB. **Sw**: FR, GR, SG, TI, UR, VS. **Fr**: HAl, Sav, HSav. **It**: Ven, TAA, Lomb, Piem, VA. **Sl**: SlA.

***Bryoria crispa* (Motyka) Bystrek**

Syn.: *Alectoria crispa* Motyka

L # – Subs.: cor – Alt.: 2–3 – Note: a name applied to a lichen with thallus reacting Pd+ red, soralia with spinulose margins, and isidioid soredia, perhaps a morph of *B. fuscescens*; on bark of various trees; distribution poorly documented, because often not distinguished. – **Au**: St, N.

***Bryoria fremontii* (Tuck.) Brodo & D. Hawksw.**

Syn.: *Alectoria fremontii* Tuck.

L – Subs.: cor – Alt.: 3–4 – Note: a cool-temperate to boreal-montane, easily recognizable species found on twigs of conifers in damp forests; there is no recent record from Italy, and no other trusted record from the Alps. – **Sw**: ?Sw. **It**: TAA, Piem.

***Bryoria furcellata* (Fr.) Brodo & D. Hawksw.**

Syn.: *Alectoria nidulifera* Norrl., *Cetraria furcellata* Fr.

L – Subs.: cor, xyl – Alt.: 3–4 – Note: a mainly boreal-montane, circumpolar lichen found on isolated conifers near treeline, sometimes on lignum; apparently rare in the Alps. – **Au**: S, K, N. **Sw**: ?Sw. **It**: Frl, Ven. **Sl**: SlA.

***Bryoria fuscescens* (Gyeln.) Brodo & D. Hawksw.**

Syn.: ?*Alectoria achariana* Gyeln., *Alectoria haynaldii* Gyeln., Alectoria jubata *auct. p.p.*, Alectoria jubata var. lanestris Ach., *Alectoria lanestris* (Ach.) Gyeln., *Bryopogon jubatus* (L.) Link, *Bryopogon lanestris* (Ach.) Gyeln., *Bryoria lanestris* (Ach.) Brodo & D. Hawksw., *Bryoria subcana* (Nyl. *ex* Stizenb.) Brodo & D. Hawksw., *Evernia jubata* (L.) Fr.

L – Subs.: cor, xyl, sil – Alt.: 2–5 – Note: a polymorphic and chemically variable, temperate to boreal-montane, circumpolar species with a broad ecological range; widespread and still locally common throughout the Alps. – **Au**: V, T, S, K, St, O, N, B. **Ge**: OB, Schw. **Sw**: BE, GL, GR, LU, SG, SZ, UR, UW, VD, VS. **Fr**: AHP, HAl, AMa, Drô, Isè, Sav, HSav, Var, Vau. **It**: Frl, Ven, TAA, Lomb, Piem, VA, Lig. **Sl**: SlA.

***Bryoria fuscidula* (Arnold) Bystrek**

Syn.: Alectoria cana (Ach.) Leight. f. fuscidula Arnold, *Alectoria fuscidula* (Arnold) Vain., Alectoria implexa (Hoffm.) Röhl. var. fuscidula (Arnold) Motyka

L # – Subs.: cor – Alt.: 3 – Note: a species resembling *B. implexa* in the pendent, cespitose, brown thalli, but branches with fissural soralia and distinctly blackened apices and thallus K+ yellow soon turning to red; in montane forests, only recorded from the Eastern Alps (Italy). – **It**: TAA.

***Bryoria implexa* (Hoffm.) Brodo & D. Hawksw.**

Syn.: *Alectoria catharinae* Räsänen, *Alectoria implexa* (Hoffm.) Nyl. *non auct.*, *Alectoria osteola* Gyeln., *Alectoria pseudofuscescens* Gyeln., *Alectoria subachariana* Gyeln., *Alectoria vrangiana* Gyeln., *Alectoria zopfii* Asahina, *Bryopogon implexus* (Hoffm.) Elenkin, *Bryoria friabilis* Brodo & D. Hawksw., *Bryoria osteola* (Gyeln.) Brodo & D. Hawksw., *Bryoria pseudofuscescens* (Gyeln.) Brodo & D. Hawksw., *Bryoria vrangiana* (Gyeln.) Brodo & D. Hawksw., *Usnea implexa* Hoffm.

L – Subs.: cor – Alt.: 2–4 – Note: a cool-temperate to boreal-montane, circumpolar, chemically heterogeneous species, most common on branches of coniferous, more rarely of deciduous trees in areas with frequent fog; frequent in the Alps. – **Au**: V, T, S, K, St, O, N. **Ge**: OB. **Sw**: BE, GL, GR, LU, SZ, TI, UR, UW, VD, VS. **Fr**: AHP, HAl, AMa, Isè. **It**: Frl, Ven, TAA, Lomb, Piem, VA. **Sl**: SlA, Tg.

***Bryoria kuemmerleana* (Gyeln.) Brodo & D. Hawksw.**

Syn.: *Alectoria kuemmerleana* Gyeln.

L – Subs.: cor – Alt.: 3 – Note: a species with partly pruinose thalli reacting K+ red, C-, and elongate, fusiform pseudocyphellae; on bark of various trees; distribution poorly documented because often not distinguished. – **Ge**: Schw. **Fr**: AHP, AMa. **It**: TAA.

***Bryoria mirabilis* (Motyka) Bystrek**

Syn.: *Alectoria mirabilis* Motyka, *Alectoria subprolixa* *sensu* Motyka

L # – Subs.: cor – Alt.: 3–4 – Note: a taxon resembling *B. capillaris*, with pendent, olive-brown, regularly branched thalli reacting K+ yellow then red, and Pd+ yellow, the branches evenly coloured, sometimes with tuberculate soralia; in mixed and coniferous forests; widespread in Central Europe but not common (likely to be not always properly distinguished); from the Alps there are a few scattered records. – **Au**: St. **Ge**: Schw. **Sw**: VS. **Fr**: Sav. **It**: TAA.

***Bryoria motykana* (Bystrek) Bystrek**

Syn.: *Alectoria motykana* Bystrek

L # – Subs.: cor – Alt.: 3–4 – Note: a species with pendent, grey to greyish – brown thalli reacting Pd+ yellow but K – (psoromic acid), branches with pseudocyphellae, fissural soralia, and distinctly blackened apices; widespread in Europe from the boreal to the nemoral zone in mixed and coniferous forests, but not common; from the Alps there are so far a few scattered records. – **Au**: T. **Sw**: VS. **It**: TAA, VA.

***Bryoria nadvornikiana* (Gyeln.) Brodo & D. Hawksw.**

Syn.: *Alectoria altaica* (Gyeln.) Räsänen, Alectoria implexa (Hoffm.) Röhl. var. nadvornikiana (Gyeln.) Zahlbr., *Alectoria nadvornikiana* Gyeln., *Alectoria spinulosa* Ahlner *nom. nud*.

L – Subs.: cor – Alt.: 3–4 – Note: a boreal-montane, circumpolar, shade-tolerant species of mixed upper montane to oroboreal forests, mostly on low, dead twigs and branches of conifers; widespread in the Alps, to be looked for further in the Western Alps of France. – **Au**: V, T, S, K, St, O, N. **Ge**: OB, Schw. **Sw**: BE, GL, GR, LU, SZ, UR, UW, VS. **It**: Frl, Ven, TAA, Lomb, Piem. **Sl**: SlA.

***Bryoria positiva* (Gyeln.) Bystrek**

Syn.: *Alectoria positiva* (Gyeln.) Motyka, Bryoria fuscescens (Gyeln.) Brodo & D. Hawksw. var. positiva (Gyeln.) Brodo & D. Hawksw.

L # – Subs.: cor, xyl – Alt.: 3–5 – Note: the differentation from *B. fuscescens*, based on a different chemistry, is not accepted by several authors. – **Au**: V, T, K, St.

***Bryoria simplicior* (Vain.) Brodo & D. Hawksw.**

Syn.: Alectoria nidulifera Norrl. f. simplicior Vain., *Alectoria simplicior* (Vain.) Lynge

L – Subs.: cor – Alt.: 3–4 – Note: a boreal-montane, circumpolar lichen found on isolated conifers; to be looked for further throughout the Alps. – **Au**: T. **Sw**: VS. **It**: TAA, VA.

***Bryoria smithii* (Du Rietz) Brodo & D. Hawksw.**

Syn.: *Alectoria smithii* Du Rietz

L – Subs.: cor, sax – Alt.: 3–4 – Note: a temperate to boreal-montane species found on large, more or less shaded rock walls, more rarely on bark, especially on twigs of conifers in damp montane forests; widespread, but apparently rather rare in the Alps. – **Au**: T, S, St. **Ge**: OB. **Sw**: SZ. **It**: Frl, Ven, Lomb, Piem.

***Bryoria taborensis* (Gyeln.) Hafellner & Obermayer**

Syn.: Alectoria ostrobottnica Gyeln. var. taborensis Gyeln.

L # – Subs.: cor – Alt.: 3 – Note: this name was regionally used for a species resembling *B. implexa*, but with the chemistry of *B. fuscescens*, perhaps referring to *B. vrangiana*; on bark of various trees; distribution poorly documented because often not distinguished. – **Au**: St.

***Bryoria tenuis* (E. Dahl) Brodo & D. Hawksw.**

Syn.: *Alectoria tenuis* E. Dahl

L – Subs.: cor – Alt.: 3 – Note: a species described from Greenland, with erect to decumbent thalli with blackened bases and at least some perpendicular lateral spines, therefore recalling *B. bicolor* (but lacking the third-order perpendicular branches of that species); Austrian specimens – despite their mention in one of the major genus monographs – are in urgent need of critical revision. – **Au**: V, T, S, K.

***Bryostigma muscigenum* (Th. Fr.) Frisch & G. Thor**

Syn.: *Arthonia leucodontis* (Poelt & Döbbeler) Coppins, *Arthonia muscigena* Th. Fr., *Bryostigma leucodontis* Poelt & Döbbeler

L – Subs.: cor, bry – Alt.: 3–4 – Note: a species with a strongly reduced thallus containing a chlorococcoid photobiont, and minute, hemispherical ascomata, sometimes confused with *Arthonia apatetica*; it grows on the bark of deciduous trees, but also on epiphytic bryophytes (*e.g. Leucodon sciuroides*) and on leaves in humid forests; widespread in the Alps, but probably often overlooked – **Au**: T, St. **Ge**: OB. **Sw**: GR, SG, SZ, UW, VS. **It**: TAA, Lomb, VA.

***Buellia abstracta* (Nyl.) H. Olivier**

Syn.: Buellia sequax *auct. non* (Nyl.) Zahlbr., *Lecidea abstracta* Nyl.

L – Subs.: sil – Alt.: 2–3 – Note: a much misunderstood silicicolous species, in the past frequently confused with *B. sequax*; for the study area there is a single record from the Southern Alps (Italy). – **It**: TAA.

***Buellia aethalea* (Ach.) Th. Fr.**

Syn.: *Buellia aethaleoides* (Nyl.) H. Olivier, *Buellia atropallidula* (Nyl.) J. Lahm, *Buellia baltica* Erichsen, *Buellia impressula* (Leight.) A.L. Sm., *Buellia nigerrima* (Nyl.) Arnold, *Buellia ocellata* (Flörke *ex* Flot.) Körb. var. tenella Müll. Arg., *Buellia subatra* Erichsen, *Buellia verruculosa* (Sm.) Mudd, *Gyalecta aethalea* Ach., *Lecanora umbrinofusca* Nyl., *Lecidea aethalea* (Ach.) Nyl., *Lecidea aethaleoides* Nyl., *Lecidea atroalbella* Nyl., *Lecidea nigerrima* Nyl., *Rinodina atropallidula* (Nyl.) Arnold, *Rinodina ocellulata* Bagl. & Carestia, *Rinodina umbrinofusca* (Nyl.) H. Olivier

L – Subs.: sil – Alt.: 1–5 – Note: on horizontal to weakly inclined, exposed, hard, crystalline siliceous rocks wetted by rain, mostly in species-poor stands; widespread in the Alps, but common only in dry areas. – **Au**: V, T, S, K, St, N, B. **Ge**: OB, Schw. **Sw**: GR, UR, VD, VS. **Fr**: AHP, HAl, AMa, Var. **It**: Frl, TAA, Lomb, Piem, VA, Lig.

***Buellia arborea* Coppins & Tønsberg**

L – Subs.: xyl – Alt.: 3–4 – Note: a usually sterile species with bluish-greenish, roundish to elongated, flat to concave soralia, the soredia reacting K – in squash preparations; usually on periodically dry wood of logs and old fences at high elevations; widespread in the Alps and regionally rather common. – **Au**: V, T, K, St, O, N. **Ge**: Schw. **Sw**: GR, LU, SZ, VS. **Fr**: Sav. **It**: Frl.

***Buellia arnoldii* Servít**

Syn.: *Hafellia arnoldii* (Servít) Hafellner & Türk

L – Subs.: cor – Alt.: 3–4 – Note: a mild-temperate species found on thin twigs of conifers in humid stands, with optimum in the subalpine belt; probably overlooked and more widespread in the Alps. – **Au**: T, S, St, N. **Ge**: OB. **Sw**: BE, UW. **It**: TAA.

***Buellia asterella* Poelt & Sulzer**

L – Subs.: ter-cal – Alt.: 2–3 – Note: a mainly western European species growing on calciferous or gypsicolous soil in dry grasslands, presently extinct over much of its former range: the only verifiable extant populations being in the Vågå region of Norway. – **Sw**: GR, VD, VS. **Fr**: AHP.

***Buellia atrocinerella* (Nyl.) Scheid.**

Syn.: *Lecanora atrocinerella* Nyl., *Rinodina atrocinerella* (Nyl.) Boistel

L – Subs.: sil – Alt.: 2–3 – Note: a species with a brownish-grey thallus and elongated marginal areoles reacting K+ yellow, then red; on siliceous rocks in xerothermic sites at low elevations; in the study area so far only known from the Western Alps (France, Italy). – **Fr**: HAl. **It**: Lig.

**Buellia disciformis (Fr.) Mudd f. disciformis**

Syn.: *Buellia major* De Not., *Buellia parasema* De Not., *Hafellia disciformis* (Fr.) Marbach & H. Mayrhofer, *Lecidea disciformis* (Fr.) Nyl., Lecidea parasema (Ach.) Ach. var. disciformis Fr.

L – Subs.: cor – Alt.: 1–4 – Note: a holarctic, humid subtropical to southern boreal-montane lichen found on smooth bark in rather humid woodlands, especially in open montane beech forests; widespread throughout the Alps. – **Au**: V, T, S, K, St, O, N, B. **Ge**: OB, Schw. **Sw**: BE, GL, GR, LU, SG, SZ, TI, UR, UW, VS. **Fr**: AHP, HAl, AMa, Isè, Sav, HSav, Var, Vau. **It**: Frl, Ven, TAA, Lomb, Piem, VA, Lig. **Sl**: SlA, Tg.

**Buellia disciformis (Fr.) Mudd f. microspora (Vain.) Zahlbr.**

Syn.: Hafellia disciformis (Fr.) Marbach & H. Mayrhofer var. microspora (Vain.)

L – Subs.: cor – Alt.: 2–3 – Note: morphology and ecology as in the typical form, but ascospores less than 20 µm long; based on a type from Northern Finland; distribution insufficiently known because not always distinguished. – **Au**: V, T, S, K, St, O, N. **Ge**: OB. **Fr**: AHP, AMa, Vau. **Sl**: SlA.

***Buellia dispersa* (A. Massal.) A. Massal.**

Syn.: Buellia dispersa (A. Massal.) A. Massal. var. cinerascens Bagl. *Buellia duartei* Samp., Buellia italica A. Massal. var. tumida A. Massal., *Buellia squamulata* (Nyl.) Zahlbr., *Buellia tergestina* J. Steiner & Zahlbr., *Buellia tumida* (A. Massal.) Bagl., Catolechia maritima A.Massal. var. dispersa A. Massal., *Lecidea squamulata* Nyl.

L – Subs.: sil, int – Alt.: 1–5 – Note: a xeric subtropical to mild-temperate lichen of base-rich or slightly lime-containing siliceous rocks in warm-dry situations, present both in the Mediterranean area and in dry valleys of the Alps. – **Au**: T. **Sw**: GR, VS. **Fr**: AMa, Var, Vau. **It**: Ven, TAA, Lomb, Piem, VA, Lig.

***Buellia ectolechioides* (Vain.) Erichsen**

Syn.: *Melanaspicilia ectolechioides* Vain.

L – Subs.: int – Alt.: 4 – Note: a species of the *B. aethalea*-group, characterised by small, grey thalli not reacting with K but typically with a I+ blue medulla; on stones and low siliceous rocks; based on a type from Northern Siberia; distribution in the Alps very insufficiently known. – **Au**: K.

***Buellia elegans* Poelt**

Syn.: Buellia epigea (Pers.) Tuck. var. angustata (Müll. Arg.) Zahlbr., Buellia epigea (Pers.) Tuck. var. effigurata (Schaer.) Zahlbr., Buellia epigaea (Pers) Tuck. var. major (Müll. Arg.) Zahlbr., Diploicia epigaea (Pers.) A. Massal. var. angustata Müll. Arg., Diploicia epigaea (Pers.) A. Massal. var. effigurata (Schaer.) Körb., Diploicia epigaea (Pers.) A. Massal. var. major Müll. Arg., Lecidea epigaea (Pers.) Schaer. var. effigurata Schaer.

L – Subs.: ter-cal – Alt.: 4–6 – Note: a widespread steppe-species found on soil deriving from calciferous schists in open grasslands, most frequent in dry-warm valleys in the Alps. – **Au**: V, T, S, K, St. **Ge**: OB. **Sw**: BE, VD, VS. **Fr**: Sav. **It**: Lomb, Piem, VA.

***Buellia epigaea* (Pers.) Tuck.**

Syn.: *Buellia nivea* (Anzi) Zahlbr., *Catolechia epigaea* (Pers.) Anzi, *Diploicia epigaea* (Pers.) A. Massal., *Lecanora epigaea* (Pers.) Ach., *Lecidea epigaea* (Pers.) Schaer., *Lichen epigaeus* Pers., *Parmelia epigaea* (Pers.) Ach., *Psora epigaea* (Pers.) Hoffm., *Rinodina nivea* Anzi

L – Subs.: bry, deb, ter-cal – Alt.: 2–5 – Note: widespread in Europe, from submediterranean regions to Scandinavia, on base-rich mineral soil, weathered gypsum and gypsum soil; widespread in the Alps as well, but generally not common. – **Au**: T, S, K, St, O. **Sw**: GR, LU, SG, UR, UW, VD, VS. **Fr**: AHP, Sav, HSav, Vau. **It**: TAA, Lomb, Piem, VA.

***Buellia erubescens* Arnold**

Syn.: Buellia disciformis (Fr.) Mudd var. saprophila (Ach.) Mudd, *Buellia jorgei* Samp., Buellia parasema De Not. var. saprophila (Ach.) Körb., *Buellia zahlbruckneri* J. Steiner *non* *sensu* T. Schauer, Lecidea parasema (Ach.) Ach. var. saprophila Ach.

L – Subs.: cor, xyl, bry – Alt.: 1–5 – Note: on acid and smooth bark in warm-humid areas; the distribution in the Alps is very poorly known: all earlier records of this species from upland areas of the Alps refer to *Tetramelas chloroleucus* (see Nimis 2016). – **Au**: V, T, S, K, St, O, N. **Ge**: OB, Schw. **Sw**: BE, GL, GR, LU, SZ, TI, UW, VS. **Fr**: Var. **Sl**: SlA.

***Buellia fusca* (Anzi) Kernst.**

Syn.: Buellia spuria (Schaer.) Anzi var. fusca Anzi

L # – Subs.: sil – Alt.: 1–2 – Note: on vertical to underhanging surfaces of siliceous rocks near the ground in warm-dry situations, such as in arid grasslands and in openings of Mediterranean garrigues; known only from the Eastern and Western Alps, and the Pyrenees. Related to *B. tyrolensis*, but chemically different, this taxon needs further study. – **Fr**: AMa. **It**: TAA, Lomb.

***Buellia griseovirens* (Turner & Borrer *ex* Sm.) Almb.**

Syn.: *Aplotomma turgidum* (A. Massal.) Beltr., *Buellia betulina* (Hepp) Th. Fr., *Buellia elenkinii* Tomin, *Buellia griseovirens* (Turner & Borrer *ex* Sm.) Almb. var. superreagens (Servít) Poelt, *Buellia turgida* (A. Massal.) Lettau, *Diplotomma betulinum* (Hepp) Arnold, *Diplotomma superreagens* (Servít) Szatala, *Diplotomma turgidum* A. Massal., *Lecidea betulina* Hepp, *Rhizocarpon betulinum* (Hepp) Zwackh, *Rhizocarpon efflorescens* Th. Fr., *Sporodichium betulinum* (Hepp) Vain., *Variolaria griseovirens* Turner & Borrer *ex* Sm.

L – Subs.: cor, xyl – Alt.: 1–5 – Note: a probably holarctic, temperate to boreal-montane lichen found on smooth bark of deciduous trees and shrubs in rather humid, well-lit situations, more rarely on wood, with optimum in the submediterranean belt; widespread throughout the Alps. – **Au**: V, T, S, K, St, O, N, B. **Ge**: OB, Schw. **Sw**: BE, FR, GL, GR, LU, SZ, TI, UR, UW, VS. **Fr**: AHP, AMa, Drô, Isè, HSav, Var, Vau. **It**: Frl, Ven, TAA, Lomb, Piem, VA, Lig. **Sl**: SlA, Tg. **Li**.

***Buellia henricii* B. de Lesd.**

L # – Subs.: sil – Alt.: mon-salp – Note: a silicicolous species with a very thin, whitish grey, continuous thallus reacting K-, delimited by a black prothallus, forming patches of 2–3 cm in diam., numerous black apothecia (*c.* 0.2 mm in diam.), first immersed in the thallus, then sessile, the disc first concave, then persistently plane, with a thin proper margin, epithecium brown, hymenium colourless, amyloid, hypothecium pale brown, paraphyses coherent, 8-spored asci, and 1-septate, not constricted, brown ascospores measuring 18–20 × 12–13(-15) µm; only known from the type collection at 1,500 m, and probably belonging to *Diplotomma*. – **It**: VA.

***Buellia jugorum* (Arnold) Arnold**

Syn.: Buellia verruculosa (Sm.) Mudd var. jugorum Arnold

L – Subs.: int, sil – Alt.: 4–5 – Note: an arctic-alpine to boreal-montane, circumpolar species found on small siliceous pebbles in wind-exposed ridges, sometimes overgrowing other crustose lichens; probably more widespread in the Alps. – **Au**: V, T, K, St. **Sw**: GR, UR. **Fr**: AHP, HAl. **It**: TAA.

***Buellia leptocline* (Flot.) A. Massal.**

Syn.: *Buellia gevrensis* Th. Fr., *Buellia hypopodioides* (Nyl.) Arnold, Buellia leptocline (Flot.) A. Massal. var. mougeotii (Hepp *ex* Arnold) Th. Fr., *Lecidea hypopodioides* Nyl., *Lecidea leptocline* Flot., *Lecidea mougeotii* Hepp

L – Subs.: sil – Alt.: 3–5 – Note: a mainly boreal-montane species found on steeply inclined to underhanging, hard siliceous rocks. – **Au**: V, S, K, St, N. **Ge**: OB. **Sw**: GR, VS. **Fr**: HAl, HSav, Var. **It**: Frl, Ven, TAA, Lomb, Piem, Lig. **Sl**: SlA.

***Buellia leptoclinoides* (Nyl.) J. Steiner**

Syn.: Buellia disciformis (Fr.) Mudd var. saxicola H. Olivier, *Hafellia leptoclinoides* (Nyl.) Scheid. & H. Mayrhofer, *Lecidea leptoclinoides* Nyl.

L – Subs.: cor, sil – Alt.: 1 – Note: a probably humid subtropical to mild-temperate species found on bark and on coastal siliceous rocks subject to humid, salt-laden winds; in the study area so far only known from the base of the Western Alps, not far from the sea. – **Fr**: Var, Vau.

***Buellia leptolepis* Bagl. & Carestia**

Syn.: *Karschia leptolepis* (Bagl. & Carestia) Arnold, Karschia saxatilis (Schaer.) Rehm f. leptolepis (Bagl. & Carestia) Keissl.

L # – Subs.: sil-par – Alt.: 3–6 – Note: a parasite of crustose Lecanoraceae; hitherto known from the Alps and Scandinavia; perhaps a synonym of *B. ectolechioides*. – **Au**: T. **Sw**: VS. **It**: Piem, VA.

***Buellia longispora* Scheid.**

L – Subs.: sil – Alt.: 2–3 – Note: a species of the *B. dispersa*-group with a white thallus reacting K+ yellow, then red, a I+ blue medulla, sessile apothecia, narrowly ellipsoid ascospores with a rugulate ornamentation (to 30 µm long); on steep rock faces of siliceous rocks, from the Alps known from a few localities below the subalpine belt. – **Fr**: AHP. **It**: TAA.

***Buellia miriquidica* Scheid.**

L – Subs.: sil-par – Alt.: 3–5 – Note: recalling the related *B. uberior*, but thalli containing miriquidic acid and ascospores with a psilate ornamentation; on hard siliceous rocks at high elevations, lichenicolous on *Schaereria fuscocinerea*; widespread in the Alps, but rarer than *B. uberior*. – **Au**: T, S, K, St. **Sw**: BE, GR, UR, VS.

***Buellia ocellata* (Flot.) Körb.**

Syn.: *Buellia arcularum* (Harm.) Lettau, *Buellia frisiaca* Erichsen, Buellia verruculosa *auct. non* (Sm.) Mudd, *Lecanora victoris* Harm., *Lecidea arcularum* Harm., *Lecidea kaleida* Taylor, *Lecidea ocellata* (Flot.) Flörke, Lecidea petraea (Wulfen) Ach. var. ocellata Flot., *Rinodina ocellata* (Flot.) Branth. & Rostr. *non* (Hoffm.) Arnold, *Rinodina victoris* (Harm.) H. Olivier

L – Subs.: sil – Alt.: 1–3 – Note: a temperate, perhaps holarctic species found on small siliceous pebbles, but also on steeply inclined faces near the ground, below the subalpine belt. – **Au**: T, K, St, N. **Sw**: TI, VS. **Fr**: AMa, Drô, HSav. **It**: Ven, TAA, Lomb, Piem. **Sl**: SlA.

***Buellia sanguinolenta* T. Schauer**

Syn.: *Hafellia sanguinolenta* (T. Schauer) Hafellner & Türk

L – Subs.: cor – Alt.: 3 – Note: a species of the *B. disciformis*-group with a whitish thallus reacting K+ yellow then red, and ascospores longer than 25 µm; on bark, mostly of *Abies* in old-growth forests; in the study area known from a few localities in the Eastern Alps. – **Au**: N. **Ge**: OB.

***Buellia sardiniensis* J. Steiner**

Syn.: Buellia leptocline A. Massal. var. minor Bagl., *Buellia lusitanica* J. Steiner

L – Subs.: sil – Alt.: 2 – Note: a species with white thalli exceeding 5 cm in diam. reacting K+ yellow then red (diagnostic against *B. saxorum*) and C+ red, a I+ violet medulla, sessile apothecia, and ascospores of the *Physconia*-type; on hard siliceous rocks in both coastal and inland habitats; in the study area so far known only from the base of the Western Pre-Alps. – **Fr**: Vau.

***Buellia saxorum* A. Massal.**

Syn.: *Buellia superans* (Nyl.) Mong., *Lecidea saxorum* (A. Massal.) Hepp, *Lecidea superans* Nyl.

L – Subs.: sil – Alt.: 1–3 – Note: on steeply inclined surfaces of siliceous rocks, mostly not far from the coast. – **Fr**: Vau. **It**: Ven, Lig.

***Buellia schaereri* De Not.**

Syn.: *Buellia destructans* (Tobler) R. Sant., *Buellia nigritula* (Nyl.) Mudd, *Karschia destructans* Tobler, *Lecidea nigritula* Nyl.

L – Subs.: cor, xyl – Alt.: 2–4 – Note: a mainly cool-temperate to boreal-montane, circumpolar species found on acid bark, especially of conifers, and on wooden poles in upland areas; widespread throughout the Alps. – **Au**: V, T, S, K, St, O, N. **Ge**: OB, Schw. **Sw**: BE, GL, GR, LU, SG, SZ, TI, UR, UW, VD, VS. **Fr**: AMa, HSav. **It**: Frl, Ven, TAA, Lomb, Piem, Lig. **Sl**: SlA.

***Buellia sororia* Th. Fr.**

Syn.: *Buellia sororioides* Erichsen

L # – Subs.: sil, int – Alt.: 3–5 – Note: a species of the *B. aethalea*-group with a non-amyloid medulla and ascospores longer (mean *c.* 16 µm) than in *B. aethalea*, but thallus also K+ yellow, then red; on siliceous rocks inbetween other crustose lichens; described from Sweden and not generally accepted, probably widespread, but poorly documented in countries where it is treated as synonym of *B. aethalea*. – **Au**: T, K, N.

***Buellia spuria* (Schaer.) Anzi**

Syn.: *Buellia italica* A. Massal., *Buellia lactea* (A. Massal.) Körb., Buellia lactea (A. Massal.) Körb. var. olivaceofusca Anzi, *Buellia liguriensis* B. de Lesd., *Buellia olivaceofusca* (Anzi) Zahlbr., *Catolechia lactea* A. Massal., *Lecidea italica* (A. Massal.) Wedd. *non* B. de Lesd., *Lecidea spuria* Schaer.

L – Subs.: sil – Alt.: 1–3 – Note: a mild-temperate to subtropical, chemically variable species, most common on granite, often found on walls. – **Au**: T, N. **Sw**: GR, TI, UR, VS. **Fr**: AHP, HAl, AMa, Var, Vau. **It**: Ven, TAA, Lomb, Piem, VA, Lig.

***Buellia stellulata* (Taylor) Mudd**

Syn.: *Buellia candidula* Arnold, Buellia lactea (A. Massal.) Körb. var. maritima (A. Massal.) Anzi, *Buellia maritima* (A. Massal.) Bagl., *Buellia minutula* (Hepp) Arnold, Buellia subalbula (Nyl.) Müll. Arg. var. adriatica Zahlbr., *Catolechia maritima* A. Massal., *Lecidea candidella* Nyl., *Lecidea microtera* Nyl., *Lecidea stellulata* Taylor

L – Subs.: cal, sil, int – Alt.: 1–3 – Note: a mild-temperate to subtropical lichen found on calciferous and base-rich, hard siliceous rocks (*e.g.* on basalt), both near the coast and in dry-warm valleys of the Alps. – **Au**: T, N. **Sw**: BE, GR, TI. **Fr**: AMa, Sav, Var, Vau. **It**: TAA, Lomb, Piem, VA, Lig.

***Buellia subdisciformis* (Leight.) Jatta.**

Syn.: *Buellia ryssolea* (Leight.) A.L. Sm., *Buellia sejuncta* J. Steiner, *Lecidea ryssolea* Leight., *Lecidea subdisciformis* Leight.

L – Subs.: sil – Alt.: 1–3 – Note: a mild-temperate to humid subtropical species found on siliceous rocks, chiefly Mediterranean-Atlantic in Europe. Austrian and Swiss records from high altitudes are very unlikely. – **Au**: ?K. **Sw**: ?VS. **Fr**: AHP, AMa, Var, Vau.

***Buellia subsquamosa* J. Steiner**

L – Subs.: sil – Alt.: 1–3 – Note: a rarely collected lichen of porous siliceous rocks rich in minerals, both in the Mediterranean area and in dry-continental valleys of the Alps, where it exceptionally reaches the subalpine belt. – **Sw**: ?VS. **Fr**: HAl, Vau. **It**: TAA.

***Buellia tesserata* Körb.**

Syn.: *Buellia cerussata* Llimona & Werner

L – Subs.: sil – Alt.: 2 – Note: this species, based on a type from Norway, is very similar *B. fimbriata* but has a different chemistry (barbatic acid); it grows on siliceous rocks and is apparently rare. – **It**: Lomb.

***Buellia tyrolensis* Körb.**

Syn.: *Buellia buellioides* (Metzler) Buschardt, *Buellia cinereomarginata* B. de Lesd., *Buellia luridula* (Nyl.) Zahlbr., Buellia spuria (Schaer.) Anzi var. fusca Anzi, *Lecidea luridula* Nyl., *Lecidea scotochroa* Nyl., *Rinodina buellioides* Metzler

L – Subs.: sil – Alt.: 1–3 – Note: on siliceous rocks in dry-warm areas, related to *B. fusca*, but chemically different, occurring both in the Mediterranean area and in dry-continental valleys of the Alps. – **Sw**: GR, TI, VS. **Fr**: AMa, Var. **It**: TAA, Lomb, Lig.

***Buellia triseptata* A. Nordin**

Syn.: Buellia lauricassiae *auct. eur. non* (Fée) Müll. Arg., Buellia triphragmia *auct. non* (Nyl.) Arnold

L # – Subs.: xyl – Alt.: 3–4 – Note: mainly lignicolous, more rarely on the bark of conifers in upland areas, this taxon needs further study. – **Au**: V, T, S, St, N. **Sw**: GR. **Fr**: HAl. **It**: TAA, Piem.

***Buellia uberior* Anzi**

Syn.: *Buellia atrocinerea* (Anzi) Zahlbr., *Buellia contermina* Arnold, Buellia lactea (A. Massal.) Körb. var. atrocinerea Anzi, *Buellia nitida* Eitner, *Buellia subbadia* Anzi

L – Subs.: sil-par – Alt.: 3–5 – Note: a mainly arctic-alpine, circumpolar species found on hard, lime-free siliceous rocks, mainly on inclined to subvertical faces wetted by rain, initially parasitic on *Schaereria fuscocinerea*. – **Au**: V, T, S, K, St. **Ge**: Schw. **Sw**: BE, GR, TI, VS. **Fr**: AHP. **It**: Frl, Ven, TAA, Lomb, VA.

***Buellia uberiuscula* (Nyl.) Zahlbr.**

Syn.: *Lecidea uberiuscula* Nyl.

L – Subs.: sil-par – Alt.: 5 – Note: a species of the *B. aethalea*-group with a minute grey thallus reacting K+ yellow then red, and an amyloid medulla; on nutrient-rich surfaces of siliceous rocks, lichenicolous on other crustose lichens (*e.g.*, *Acarospora fuscata*, *Sporastatia testudinea*); widespread, but rare in the Alps. – **Au**: T.

***Buellia vilis* Th. Fr.**

Syn.: *Buellia enteroleucoides* (Nyl.) Arnold, *Buellia modica* (Nyl.) Lettau, Lecidea disciformis Nyl. var. enteroleucoides Nyl., *Lecidea enteroleucoides* (Nyl.) Lamy, *Lecidea modica* Nyl.

L – Subs.: sil – Alt.: 3–5 – Note: a mainly arctic-alpine, probably circumpolar early coloniser of siliceous pebbles in windy situations, and of recently eroded granitic boulders; certainly more widespread in the Alps, but easily overlooked. – **Au**: T, K. **Sw**: UR, VS. **Fr**: AMa, HSav. **It**: Frl, TAA.

***Buellia violaceofusca* G. Thor & Muhr**

L – Subs.: cor – Alt.: 3 – Note: a sterile crustose lichen with a pale grey thallus and maculiform soralia of a dark brownish colour with a violet tinge; on bark of old deciduous trees in shaded montane forests (ecological requirements similar to those of *Caloplaca lucifuga*); from the Alps there are, so far, a few records only. – **Au**: V, S, K.

***Bunodophoron melanocarpum* (Sw.) Wedin**

Syn.: *Lichen melanocarpus* Sw., *Sphaerophorus compressus* Ach., *Sphaerophorus melanocarpus* (Sw.) DC.

L – Subs.: sil, ter-sil, cor – Alt.: 3 – Note: a humid subtropical to mild-temperate species found on mossy bark and rocks in very moist forests; rare and probably declining in the Alps. – **Au**: T, K, N. **Ge**: OB. **Sw**: BE, SZ, UR. **It**: Lomb, Piem.

***Byssoloma leucoblepharum* (Nyl.) Vain.**

Syn.: *Bilimbia leucoblephara* (Nyl.) Arnold, *Calidia rhizophora* Stirt., *Lecidea leucoblephara* Nyl.

L – Subs.: cor, fol – Alt.: 1–2 – Note: a pantropical foliicolous species, sometimes occurring also on bark; extremely rare in the Alps. – **Au**: K. **Fr**: AMa.

***Byssoloma marginatum* (Arnold) Sérus.**

Syn.: *Bacidia marginata* (Arnold) Lettau, *Bacidia micromma* (Nyl. *ex* Stizenb.) Hulting, *Bilimbia marginata* Arnold, *Tapellaria similis* Kalb

L – Subs.: cor – Alt.: 1–3 – Note: a humid subtropical to mild-temperate lichen, growing both on bark and on needles of conifers in warm-humid areas; extremely rare in the Alps. – **Au**: St.

***Byssoloma subdiscordans* (Nyl.) P. James**

Syn.: *Byssoloma rotuliforme* (Müll. Arg.) R. Sant., *Byssoloma subdiscordans* *sensu* Lettau *non* (Nyl.) Vain., *Byssoloma tricholomum* *sensu* Lettau *non* (Mont.) Zahlbr., *Chiodecton subdiscordans* Nyl., *Patellaria rotuliformis* Müll. Arg.

L – Subs.: cor, fol – Alt.: 1–3 – Note: a humid subtropical to tropical species, with isolated outliers in humid parts of the mild-temperate zone; in the Alps it is mainly found in montane, humid forests, on twigs and leaves of conifers, and it might be more widespread, but not common. – **Au**: T, K, St, N. **Ge**: OB. **Fr**: AMa.

***Caeruleum heppii* (Hepp *ex* Arnold) K. Knudsen & Arcadia**

Syn.: *Acarospora heppii* Hepp *ex* Arnold, *Myriospora heppii* Nägeli *ex* Hepp *nom. inval*.

L – Subs.: cal, int – Alt.: 1–3 – Note: an easily overlooked early coloniser of small calcareous pebbles in dry grasslands, which also occurs on concrete and mortar in small settlements and on walls of calciferous sandstone; widespread in the Alps below the subalpine belt, but only locally common. – **Au**: V, T, S, K, St, O, N. **Ge**: OB, Schw. **Sw**: LU, SZ, UW, VS. **Fr**: HSav. **It**: Ven, TAA, Lomb.

***Calicium abietinum* Pers.**

Syn.: *Calicium cervicatulum* Ach., *Calicium curtum* Turner & Borrer *ex* Sm., *Calicium minutum* (Körb.) Arnold, Calicium nigrum *auct. p.p*.

L – Subs.: xyl, cor – Alt.: 2–4 – Note: a temperate to boreal-montane, circumpolar species found on old wood of conifers, but also on bark, especially of *Abies*, much more rarely on deciduous trees (*e.g.* on *Castanea*) and, in humid areas, on wooden poles; widespread throughout the Alps. – **Au**: T, S, K, St, O, N, B. **Ge**: OB, Schw. **Sw**: BE, LU, SZ, TI, UW, VD, VS. **Fr**: AHP, AMa, HSav, Var, Vau. **It**: Ven, TAA, Lomb, Piem. **Sl**: SlA, Tg.

***Calicium adaequatum* Nyl.**

Syn.: *Calicium marianum* (Nádv.) Nádv.

L – Subs.: cor – Alt.: 3–4 – Note: a species peculiar in having apothecia with pale (olivaceous) stalks with an amyloid reaction (as the exciple) in squash preparations; on bark (mostly on twigs) of deciduous trees (*Alnus*, *Acer*) under oceanic conditions; rare, or only seldom collected, as it can be easily overlooked. – **Au**: T, St. **Sw**: BE, GR, SZ.

***Calicium adspersum* Pers.**

Syn.: *Calicium lenticulare* *sensu* Nádv., *Calicium mutabile* Ach., *Calicium roscidum* (Ach.) Flörke

L – Subs.: cor, xyl – Alt.: 2–3 – Note: a holarctic, temperate species found on bark, rarely on lignum of deciduous trees, especially oaks, often in fissures of the bark, more rarely on conifers; widespread in the Alps, but generally not very common. – **Au**: V, T, S, K, St, O, N. **Ge**: OB. **Sw**: BE, GR, LU, SZ, UR, UW, VD, VS. **Fr**: HSav. **It**: Lomb, Piem.

***Calicium corynellum* (Ach.) Ach.**

Syn.: *Caliciella corynella* (Ach.) Vain., Caliciella corynella (Ach.) Vain. var. stipitata Vain., Caliciella corynella (Ach.) Vain. var. subsessile Vain., Calicium chlorinum *auct. non* (Ach.) Schaer., Calicium corynellum (Ach.) Ach. var. paroicum (Ach.) Ach., Calicium corynellum (Ach.) Ach. var. stipitatum (Vain.) Zahlbr., Calicium corynellum (Ach.) Ach. var. subsessile (Vain.) Zahlbr., *Calicium paroicum* Ach., *Chaenotheca paroica* (Ach.) Zwackh., *Cyphelium paroicum* (Ach.) Arnold, *Lichen corynellus* Ach., *Sphinctrina paroica* (Ach.) Trevis., *Strongyleuma paroicum* (Ach.) Vain. *non auct.*

L – Subs.: sil, xyl – Alt.: 2–3 – Note: a temperate, probably holarctic species found beneath overhangs of hard siliceous rocks in humid areas; probably more widespread in the Alps, but never common. – **Au**: S, N. **Sw**: GR. **Fr**: Isè, HSav. **It**: Ven, TAA, Lomb, Piem, VA.

***Calicium denigratum* (Vain.) Tibell**

Syn.: *Calicium curtum* Turner & Borrer *ex* Sm. var. denigratum Vain.

L – Subs.: cor, xyl – Alt.: 3–4 – Note: similar to *C. abietinum* in the endoxylic thallus and the epruinose apothecia, but with more slender stalks, ascospores with a length-width ratio <2, and a coarsely areolate sculpture; on decorticated stumps and snags in montane coniferous forests; widespread also in the Alps, but not very common. – **Au**: T, S, K, O, N. **Ge**: OB. **Sw**: BE, LU, SZ.

***Calicium glaucellum* Ach.**

Syn.: *Calicium discoidale* Ach.

L – Subs.: cor, xyl – Alt.: 2–4 – Note: a temperate to boreal-montane, holarctic species found on lignum and acid bark, especially on decorticated stumps of conifers, but also of broad-leaved trees (*e.g.* on *Castanea*); widespread in the Alps, but not very common. – **Au**: V, T, S, K, St, O, N. **Ge**: OB, Schw. **Sw**: BE, GL, GR, LU, SZ, UW, VD, VS. **Fr**: AHP, AMa. **It**: Frl, Ven, TAA. **Sl**: SlA, Tg.

***Calicium lenticulare* Ach.**

Syn.: *Calicium amylocaule* Lettau, *Calicium atroviride* Körb., Calicium cladoniscum *auct. non* Ach., *Calicium lenticulare* Ach. var. cladoniscum *auct. non* (Ach.) Schaer., Calicium quercinum Pers. var. lenticulare (Ach.) Nyl., *Calicium schaereri* *sensu* Nádv. *non* De Not., *Calicium subquercinu*m Asahina, *Calicium virescens* (Schaer.) Hepp

L – Subs.: xyl, cor – Alt.: 3–4 – Note: a mainly cool-temperate to boreal-montane, circumpolar species found on lignum of decorticated stumps and trunks of conifers; widespread throughout the Alps. – **Au**: V, T, S, K, St, O, N. **Sw**: BE, GR, SZ, UR, UW, VS. **Fr**: HSav. **It**: Frl, Ven, TAA, Lomb, Piem. **Sl**: Tg.

***Calicium lucidum* (Th. Fr.) M. Prieto & Wedin**

Syn.: *Acolium lucidum* (Th. Fr.) Rabenh., *Acolium viridulum* Schaer., *Calicium virellum* Nyl., *Cyphelium lucidum* (Th. Fr.) Th. Fr., *Trachylia lucida* Th. Fr.

L – Subs.: cor, xyl – Alt.: 3–4 – Note: a mainly boreal-montane, circumpolar species found on old conifers in humid, open forests with frequent fog; widespread in the Alps, but only locally common. – **Au**: V, T, S, K, St, N. **Ge**: Schw. **Sw**: BE, GR, SZ, UR, TI, UW. **It**: Ven, TAA, Lomb.

***Calicium montanum* Tibell**

L – Subs.: cor, xyl – Alt.: 2–4 – Note: a recently-described species with a relatively thick, pale grey thallus, short-stalked apothecia with a white pruina, and ascospores with coarse irregular cracks; usually on wood of conifers in the montane belt; probably more widespread in the Alps. – **Au**: V, O. **Ge**: OB, Schw. **Sw**: BE, GL, GR, LU, SZ, TI, UR, UW.

***Calicium notarisii* (Tul.) M. Prieto & Wedin**

Syn.: *Acolium notarisii* Tul., *Cyphelium notarisii* (Tul.) Blomb. & Forssell, Cyphelium tigillare (Ach.) Ach. subsp. notarisii (Tul.) W.A.Weber, *Pseudacolium notarisii* (Tul.) Vain., *Trachylia notarisii* (Tul.) Nyl., Trachylia tigillaris (Ach.) Fr. var. notarisii (Tul.) Th. Fr.

L – Subs.: cor, xyl – Alt.: 3–4 – Note: a mainly cool-temperate to southern boreal-montane lichen found on dry, weathered wood (*e.g.* on fences, wooden poles), but also on acid bark of old trees (especially *Quercus*); perhaps more widespread in the Alps. – **Au**: St, N. **Sw**: SW, VD, VS. **Fr**: AHP, HAl, AMa. **It**: Piem, VA.

***Calicium parvum* Tibell**

L – Subs.: cor – Alt.: 2–4 – Note: a species with a thin, verruculose, grey thallus, minute epruinose apothecia, clavate asci and ascospores with polygonal, broad warts, often accompanied by conspicuous pycnidia; on bark of conifers; widespread also in the Alps, but not very common, and in the past probably confused with other species. – **Au**: T, S, K, St, O. **Ge**: OB. **Sw**: BE, GR, SZ, UW. **Fr**: AHP, AMa, Drô, Vau. **Sl**: SlA.

***Calicium pinastri* Tibell**

L – Subs.: cor, xyl – Alt.: 2–4 – Note: a species with a thin, grey thallus, short-stalked, minute, epruinose apothecia, cylindrical asci, and ascospores with irregular cracks, found on the bark of conifers (most often *Pinus sylvestris*); recently-described and still with a few records from the Alps, but probably more widespread. – **Ge**: OB. **Sw**: LU, SZ, UW. **It**: TAA.

***Calicium pinicola* (Tibell) M. Prieto & Wedin**

Syn.: *Cyphelium pinicola* Tibell

L – Subs.: xyl, cor – Alt.: 3–4 – Note: a mainly temperate to southern boreal-montane lichen found on bark of conifers, especially of *Pinus*, near the base of the trunks; less confined to high altitudes than *C. tigillare*. – **Au**: T, S, K, St. **Ge**: OB, Schw. **Sw**: BE, GR, LU, VS. **Fr**: AHP, HAl, AMa, HSav. **It**: TAA, Lomb, Piem.

***Calicium quercinum* Pers.**

Syn.: *Calicium curtiusculum* Nyl., *Calicium decipiens* A. Massal., Calicium lenticulare Ach. var. bacillare Ach., Calicium lenticulare Ach. var. curtiusculum (Nyl.) Lettau

L – Subs.: xyl, cor – Alt.: 2–3 – Note: a holarctic, temperate species found on lignum and bark of deciduous trees, more rarely of conifers, especially on old oaks and on *Castanea*; widespread in the Alps, but generally rare. – **Au**: V, T, O. **Ge**: OB. **Sw**: BE, GR, VS. **Fr**: HSav. **It**: Lomb, Piem.

***Calicium salicinum* Pers.**

Syn.: Calicium hyperellum (Ach.) Ach. var. salicinum (Pers.) Schaer., *Calicium lichenoides* (L.) Schumach., *Calicium sphaerocephalum* (L.) Ach., Calicium sphaerocephalum (L.) Ach. var. xylonellum (Ach.) Wahlenb., *Calicium trachelinum* (Ach.) Ach., *Calicium xylonellum* Ach.

L – Subs.: xyl, cor – Alt.: 1–4 – Note: a holarctic, temperate species, most frequent on dry parts of the boles of deciduous, acid-barked trees, but also on lignum (fence-posts, decorticated stumps); widespread throughout the Alps. – **Au**: V, T, S, K, St, O, N. **Ge**: OB, Schw. **Sw**: BE, GR, LU, SZ, UW, VD, VS. **Fr**: AHP, AMa, Drô, Isè, Sav, HSav, Var, Vau. **It**: Frl, Ven, TAA, Lomb, Lig. **Sl**: SlA, Tg.

***Calicium tigillare* (Ach.) Pers.**

Syn.: *Acolium tigillare* (Ach.) Gray, *Cyphelium tigillare* (Ach.) Ach., Cyphelium trachylioides *auct. non* (Nyl. *ex* Branth & Rostr.) Erichsen, *Cyphelium viridescens* *auct.*, *Lichen tigillaris* Ach., *Trachylia tigillaris* (Ach.) Fr.

L – Subs.: xyl, cor – Alt.: 3–5 – Note: a mainly boreal-montane, circumpolar lichen found on hard, dry wood, especially of conifers, on wooden fences and fence-posts, often together with *Ramboldia elabens*; widespread throughout the Alps. – **Au**: V, T, S, K, St, O, N. **Ge**: OB, Schw. **Sw**: BE, GR, LU, SZ, TI, UR, UW, VD, VS. **Fr**: AHP, HAl, AMa, Isè, Sav, HSav. **It**: Frl, Ven, TAA, Lomb, Piem, VA. **Sl**: SlA. **Li**.

***Calicium trabinellum* (Ach.) Ach.**

Syn.: Calicium adspersum Pers. var. roscidulum (Nyl.) Harm., Calicium adspersum Pers. var. trabinellum (Ach.) Schaer., *Calicium incrustans* Körb., *Calicium roscidulum* Nyl. *ex* F.Wilson, *Calicium validiusculum* Trevis., Calicium xylonellum Ach. var. trabinellum Ach.

L – Subs.: xyl, cor – Alt.: 3–4 – Note: a holarctic, temperate to boreal-montane species found on hard wood, especially on old, decorticated stumps of conifers, more rarely of deciduous or even evergreen broad-leaved trees (*e.g. Quercus ilex* in montane Mediterranean forests); widespread throughout the Alps. – **Au**: V, T, S, K, St, O, N, B. **Ge**: OB, Schw. **Sw**: BE, GR, LU, SZ, VD, VS. **Fr**: AHP, HAl, AMa, HSav. **It**: Frl, Ven, TAA, Lomb, Piem. **Sl**: SlA.

***Calicium viride* Pers.**

Syn.: *Calicium baliolum* Ach., *Calicium hyperellum* (Ach.) Ach., *Calicium lygodes* Ach., *Calicium peltatum* Ach., *Calicium proboscidale* Ach., Calicium trachelinum (Ach.) Ach. var. epiphloeum Ach.

L – Subs.: cor – Alt.: 2–4 – Note: a holarctic, temperate to boreal-montane lichen found on *Abies* and *Picea*, but also on the rough bark of old oaks in humid areas; widespread and locally rather common throughout the Alps. – **Au**: V, T, S, K, St, O, N, B. **Ge**: OB, Schw. **Sw**: BE, GL, GR, LU, SZ, TI, UR, UW, VD, VS. **Fr**: AHP, HAl, AMa, Isè, Sav, HSav, Vau. **It**: Frl, Ven, TAA, Lomb. **Sl**: SlA.

***Callome multipartita* (Sm.) Otálora, P.M. Jørg. & Wedin**

Syn.: *Collema multipartiens* Nyl., *Collema multipartitum* Sm., *Lathagrium multipartitum* (Sm.) Arnold, *Synechoblastus multipartitus* (Sm.) Körb.

L – Subs.: cal – Alt.: 2–5 – Note: a mainly temperate to southern boreal-montane species found on calcareous rocks in rather sheltered situations; widespread throughout the Alps, but not very common. – **Au**: V, T, S, K, St, O, N. **Ge**: OB, Schw. **Sw**: BE, GR, LU, SZ, TI, UR, UW, VD, VS. **Fr**: AHP, HAl, AMa, Drô, HSav, Var, Vau. **It**: Frl, Ven, TAA, Lomb, Piem, Lig. **Sl**: SlA.

***Caloplaca adriatica* (Zahlbr.) Servít**

Syn.: Caloplaca schaereri (Arnold) Zahlbr. var. adriatica Zahlbr.

L – Subs.: cal – Alt.: 2–3 – Note: a Mediterranean to mild-temperate lichen found on steeply inclined, hard limestone rocks, with a few records from the Southern and Western Alps. – **Fr**: AMa, Drô, Var, Vau. **It**: Ven, TAA.

***Caloplaca aegatica* Giralt, Nimis & Poelt**

Syn.: Caloplaca quercina *auct. non* Flagey

L – Subs.: cor – Alt.: 2 – Note: a species with a greyish thallus, relatively large apothecia with golden-yellow margins and orange-red discs, broadly-ellipsoid ascospores (often less than 8 per ascus), and conspicuous pycnidia with blackish ostiolar regions; on bark of broad-leaved trees in *Xanthorion*-communities; widespread in the Mediterranean region, including Macaronesia, also reported from the Western Alps, at low elevations. – **Fr**: Var.

***Caloplaca albopruinosa* (Arnold) H. Olivier**

Syn.: *Biatorina albopruinosa* Arnold, *Caloplaca agardhiana* *auct.*, *Pyrenodesmia agardhiana* (Ach.) A. Massal., *Blastenia agardhiana* *auct.*, ?Blastenia agardhiana var. cinereovirens (J. Steiner) Szatala, ?Blastenia agardhiana var. minuta (J. Steiner) Szatala, *Callopisma agardhianum* *auct.*, ?Caloplaca agardhiana var. nigricans Jatta

L – Subs.: cal – Alt.: 1–4 – Note: this species differs from *C. alociza* in the hymenium devoid of crystals and the apothecia with a thalline margin. It occurs on hard limestones and dolomite in sunny, exposed sites, mostly in the mountains. Austrian records are lumped together with *C. alociza*. – **Sw**: GR, VD. **Fr**: AHP, HAl, AMa, Drô, Isè, Sav, HSav, Var, Vau. **It**: Frl, Ven, TAA, Lomb, Piem, Lig. **Sl**: SlA, Tg.

***Caloplaca alnetorum* Giralt, Nimis & Poelt**

Syn.: *Athallia alnetorum* (Giralt, Nimis & Poelt) Arup, Frödén & Søchting, Caloplaca flavorubescens *auct. non* (Huds.) J.R. Laundon

L – Subs.: cor – Alt.: 2–4 – Note: a temperate species growing on broad-leaved trees; most common in humid areas in the mountains; widespread in the Alps, but overlooked, or confused with other taxa. – **Au**: T, K, St, O, N. **Fr**: AHP, Sav. **It**: TAA, Ven. **Li**.

***Caloplaca alociza* (A. Massal.) Mig.**

Syn.: *Biatorina alociza* A. Massal., *Blastenia alociza* (A. Massal.) Werner, *Lecaniella alociza* (A. Massal.) Jatta, *Sporoblastia alociza* (A. Massal.) Trevis., *Pyrenodesmia alociza* (A. Massal.) Arnold

L – Subs.: cal – Alt.: 1–5 – Note: this species differs from *C. albopruinosa* in the hymenium inspersed by crystals and the apothecia without a thalline margin; on hard limestones and dolomite, with a wide altitudinal range; widespread and common throughout the Alps. – **Au**: V, T, S, K, St, O, N. **Ge**: OB, Schw. **Sw**: UR, VS. **Fr**: AHP, HAl, AMa, Drô, Sav, HSav, Var, Vau. **It**: Frl, Ven, TAA. **Sl**: SlA.

***Caloplaca ammiospila* (Wahlenb. *ex* Ach.) H. Olivier**

Syn.: *Blastenia ammiospila* (Wahlenb. *ex* Ach.) Arup, Søchting & Frödén, Blastenia ferruginea (Huds.) A. Massal. var. muscicola (Schaer.) A. Massal., *Caloplaca cinnamomea* (Th. Fr.) H. Olivier, *Caloplaca discoidalis* (Vain.) Lynge, Caloplaca ferruginea (Huds.) Th. Fr. var. ammiospila (Wahlenb. *ex* Ach.) Th. Fr., Caloplaca ferruginea (Huds.) Th. Fr. var. cinnamomea Th. Fr., Caloplaca ferruginea (Huds.) Th. Fr. var. muscicola *auct.*, *Caloplaca vacillans* (Th. Fr.) H. Magn., *Lecidea ammiospila* Wahlenb. *ex* Ach.

L – Subs.: deb, bry-cal, ter-cal – Alt.: 3–5 – Note: a mainly arctic-alpine to boreal-montane, bipolar lichen found on terricolous mosses and plant debris, more rarely on decaying, rather soft lignum, or even on the bark of subalpine shrubs and boreal trees, most frequent above or near treeline; widespread throughout the Alps. – **Au**: V, T, S, K, St, O, N. **Ge**: OB, Schw. **Sw**: BE, GR, LU, SZ, UR, VS. **Fr**: AHP, HAl, AMa, Isè, Sav, HSav. **It**: Frl, Ven, TAA, Lomb, Piem, VA. **Sl**: SlA.

***Caloplaca anchon-phoeniceon* Poelt & Clauzade**

L – Subs.: sil-par – Alt.: 5 – Note: a species with minute thalli and one to several sessile apothecia, both bright red; parasitic on silicicolous *Aspicilia*-species at high elevations; widespread in the Alps, but rare. – **Au**: T, S. **Sw**: UR. **Fr**: AHP, HAl, AMa.

***Caloplaca anularis* Clauzade & Poelt**

Syn.: *Caloplaca scrobiculata* *auct.* non. H. Magn.

L – Subs.: cal – Alt.: 4–5 – Note: a species of the Eurasiatic mountains, from the temperate zone southwards, found on steeply inclined, compact limestone and dolomite; perhaps more frequent but undercollected in the Alps because of its preference for sites which are of difficult access. – **Au**: K, St. **Fr**: HAl, AMa. **It**: Frl, Ven, TAA. **Sl**: SlA.

***Caloplaca approximata* (Lynge) H. Magn.**

Syn.: *Amundsenia approximata* (Lynge) Søchting, Arup & Frödén, Caloplaca vitellinula (Nyl.) H. Olivier f. approximata Lynge

L – Subs.: cal, int – Alt.: 3–4 – Note: a species with a strongly reduced, pale yellow thallus, bright orange apothecia, and narrowly ellipsoid ascospores with thin septa, perhaps closely related to *C. cacuminum*; on schists containing various amounts of calcium; based on a type from Novaya Zemlya and widespread from the Arctic to high elevations in the boreal zone; in the Alps so far known only from a single locality. – **Sw**: SZ.

***Caloplaca arcis* (Poelt & Vězda) Arup**

Syn.: Caloplaca citrina (Hoffm.) Th. Fr. var. arcis Poelt & Vězda, *Flavoplaca arcis* (Poelt & Vězda) Arup, Frödén & Søchting

L – Subs.: sil – Alt.: 2–3 – Note: a species of the *C. citrina*-group with thalli developing coarse blastidia in the centre, distinctly lobate at the margins, often with apothecia; on mineral-rich siliceous rocks and elsewhere also on calcareous rocks, also on man-made walls near settlements; widespread, but in the Alps poorly recorded, probably because it was not distinguished in the past. – **Au**: S, St, B. **Fr**: AMa, Var, Vau.

***Caloplaca arcisproxima* Vondrák, Říha, Arup & Søchting**

Syn.: *Flavoplaca arcisproxima* (Vondrák, Říha, Arup & Søchting) Arup, Søchting & Frödén

L – Subs.: sil – Alt.: 1–2 – Note: a species of the *C. citrina*-group with a thallus consisting of subumbilicate, minute squamules with margins divided into tiny lobes, developing marginal soralia; on various rock types close to the sea; in the study area so far known from a single locality in the Western Alps. – **Fr**: AMa.

***Caloplaca arenaria* (Pers.) Müll. Arg.**

Syn.: *Blastenia arenaria* (Pers.) A. Massal., *Blastenia lamprocheila* (DC.) Arnold, *Caloplaca craspedia* (Ach.) Szatala, *Caloplaca ferruginascens* (Nyl.) H. Olivier, *Caloplaca festiva* (Ach.) Zwackh *non auct.*, *Caloplaca lamprocheila* (DC.) Flagey, *Lecanora lamprocheila* (DC.) Nyl., *Lichen arenarius* Pers., *Rufoplaca arenaria* (Pers.) Arup, Søchting & Frödén

L – Subs.: sil, int – Alt.: 1–5 – Note: a holarctic lichen found on calciferous siliceous rocks, including walls, often overgrowing other crustose lichens; on the whole, a heterogeneous taxon in need of revision; widespread throughout the Alps. – **Au**: V, T, S, K, St, N, B. **Ge**: OB. **Sw**: BE, GR, SZ, TI, UR, VD, VS. **Fr**: AHP, HAl, AMa, Sav, HSav, Var, Vau. **It**: Frl, Ven, TAA, Lomb, Piem, VA, Lig.

***Caloplaca areolata* (Zahlbr.) Clauzade**

Syn.: Caloplaca cerina (Hedw.) Th. Fr. var. areolata Zahlbr., Caloplaca spalatensis *auct. non* Zahlbr.

L – Subs.: cal – Alt.: 1–2 – Note: a mild-temperate, characteristic, but much misunderstood species found on the top of calcareous birds’ perching boulders, mostly at low altitudes, with a few records from the Western Alps. – **Fr**: Var, Vau. **It**: Lig.

***Caloplaca arnoldii* (Wedd.) Zahlbr. *ex* Ginzb. subsp. arnoldii**

Syn.: *Calogaya arnoldii* (Wedd.) Arup, Frödén & Søchting, *Caloplaca biatorinoides* (Clauzade & Cl. Roux) Gaya, Caloplaca saxicola (Hoffm.) Nordin subsp. arnoldii (Wedd.) Clauzade & Cl. Roux, Caloplaca saxicola (Hoffm.) Nordin subsp. biatorinoides Clauzade & Cl. Roux, Physcia pusilla A. Massal. f. turgida A. Massal., Physcia pusilla A. Massal. var. lobulata f. minor Arnold

L – Subs.: cal, int – Alt.: 2–5 – Note: a well-distinct taxon of the extremely critical *C. saxicola*-complex; on steeply inclined calciferous rocks (limestone, dolomite, calcareous schists) in open habitats; certainly more widespread in the Alps. – **Sw**: BE, GR, LU, SZ, VS. **Fr**: AHP, AMa, Drô, Sav, HSav, Var, Vau. **It**: Frl, Ven, TAA, Piem.

***Caloplaca arnoldii* (Wedd.) Zahlbr. *ex* Ginzb. subsp. oblitterata (Pers.) Gaya**

Syn.: *Caloplaca discernenda* (Nyl.) Zahlbr., *Caloplaca miniatula* (Nyl.) Zahlbr., *Caloplaca murorum* *auct.* f. miniatula (Nyl.) Ozenda & Clauzade, *Caloplaca murorum* *auct.* var. oblitterata (Pers.) Jatta, *Caloplaca pyraceoides* B. de Lesd., Caloplaca saxicola (Hoffm.) Nordin subsp. oblitterata (Pers.) Clauzade & Cl. Roux, *Lecanora discernenda* Nyl., *Lecanora miniatula* Nyl., *Lichen oblitteratus* Pers.

L – Subs.: sil, int – Alt.: 3–4 – Note: a very polymorphic taxon (see Roux et al. 2014) with a mainly temperate to boreal distribution in Europe, also known from the Southern European mountains, most frequent on base-rich siliceous rocks or on decalcified calcareous rocks, usually in nutrient-poor stands, both on vertical cliffs and overhangs, and on horizontal surfaces of siliceous boulders; especially the southern populations seem to prefer rather shaded conditions. – **Au**: V, K, St. **Ge**: OB, Schw. **Sw**: BE, VS, SZ. **Fr**: AMa. **It**: TAA, Lomb, Piem.

***Caloplaca arnoldiiconfusa* Gaya & Nav.-Ros.**

Syn.: *Calogaya arnoldiiconfusa* (Gaya & Nav.-Ros.) Arup, Frödén & Søchting

L – Subs.: cal, int – Alt.: 3–5 – Note: in the past this species, which is widespread in Central Europe, was confused with *C. arnoldii*, which substitutes in upland areas, on vertical, sun-exposed calcareous and dolomitic rocks. – **Au**: V, T, S, K, St, O, N. **Ge**: OB. **Sw**: GR, SZ. **Fr**: AHP, AMa. **It**: Frl, Ven, TAA, Piem. **Li**.

***Caloplaca asserigena* (Stizenb. *ex* J. Lahm) H. Olivier**

Syn.: *Blastenia asserigena* (Stizenb. *ex* J. Lahm) Zahlbr., *Blastenia assigena* Arnold, *Callopisma asserigenum* Stizenb. *ex* J. Lahm, *Caloplaca assigena* (Arnold) Dalla Torre & Sarnth., *Lecanora asserigena* Stizenb. *nom. nud*.

L # – Subs.: cor, xyl – Alt.: 3–4 – Note: a species with a thin grey thallus and small aggregated apothecia with brown-red to blackish discs, the epihymenium reacting K+ purple; on thin twigs of various trees, more rarely on wood; most records from the Alps are historical. – **Au**: T, K, St, N. **Ge**: OB. **Sw**: UR. **It**: Frl.

***Caloplaca athroocarpa* (Anzi) Jatta**

Syn.: *Blastenia athroocarpa* (Anzi) Arnold, *Callopisma athroocarpon* (Anzi) Bagl. & Carestia, *Gyalolechia athroocarpa* Anzi, *Lecanora ammiospiloides* Nyl.

L # – Subs.: cor, xyl – Alt.: 4 – Note: on eutrophicated wood, more rarely on bark, on basal parts of isolated trees; a poorly understood taxon, which needs further study. – **Ge**: OB. **Sw**: GR, VS. **It**: Ven, TAA, Lomb, Piem.

***Caloplaca atroalba* (Tuck.) Zahlbr.**

Syn.: *Placodium atroalbum* Tuck.

L – Subs.: cal – Alt.: 4–5 – Note: a species with a rimose to areolate, yellowish-brownish thallus, apothecia with zeorine proper margin, a white thalline margin, black disc and amphithecium; on limestone and calcareous sandstone; based on a type from Western North America, the identity of European records is in need of re-evaluation. – **Au**: V, S, K. **Fr**: AHP, AMa.

***Caloplaca atroflava* (Turner) Mong.**

Syn.: Caloplaca atroflava (Turner) Mong. var. submersa (Nyl.) H. Magn., Caloplaca ferruginea (Huds.) Th. Fr. var. obscura Th. Fr., *Caloplaca turneriana* (Ach.) H. Olivier, *Lecidea atroflava* Turner, *Placodium atroflavum* (Turner) A.L. Sm., *Placodium turnerianum* (Ach.) A.L. Sm.

L – Subs.: sil-aqu – Alt.: 2–5 – Note: a rather southern species in Europe, found on base-rich or eutrophicated siliceous rocks, especially basalt, sometimes periodically submerged in Mediterranean creeks and rivulets; rare in the Alps, with a scattered distribution. – **Au**: T. **Fr**: HAl, AMa, Sav, Var. **It**: Lomb, Lig.

***Caloplaca aurantia* (Pers.) Hellb.**

Syn.: *Amphiloma aurantius* (Pers.) Müll. Arg., *Amphiloma callopismum* (Ach.) Körb., *Callopisma vulgaris* De Not., Caloplaca aurantia (Pers.) Hellb. var. intermedia Zahlbr., Caloplaca aurantia (Pers.) Hellb. var. papillata Poelt, *Caloplaca callopisma* (Ach.) Th. Fr., *Gasparrinia aurantia* (Pers.) Syd., *Gasparrinia callopisma* (Ach.) Syd., *Lecanora callopisma* Ach., *Lichen aurantius* Pers., *Placodium aurantium* (Pers.) Vain., *Placodium callopismum* (Ach.) Mérat, *Variospora aurantia* (Pers.) Arup, Frödén & Søchting

L – Subs.: cal – Alt.: 1–3 – Note: a mild-temperate to subtropical species found on a wide variety of calciferous substrata; common in the Mediterranean-submediterranean belts, rarer at higher altitudes, more helio – and thermophytic than the closely related *C. flavescens*; widespread throughout the Alps, at low elevations. – **Au**: V, T, K, N. **Sw**: LU, TI, VS. **Fr**: AHP, HAl, AMa, Drô, Sav, HSav, Var, Vau. **It**: Frl, Ven, TAA, Lomb, Piem, VA, Lig.

***Caloplaca australis* (Arnold) Zahlbr.**

Syn.: *Candelariella australis* (Arnold) Zahlbr., *Fulgensia australis* (Arnold) Poelt, *Gasparrinia australis* (Arnold) Dalla Torre & Sarnth., *Gyalolechia australis* (Arnold) J. Steiner, *Physcia australis* Arnold, *Variospora australis* (Arnold) Arup, Søchting & Frödén

L – Subs.: cal – Alt.: 4–5 – Note: on sun-exposed calciferous rocks, *e.g.* on the top of large, isolated boulders; certainly more widespread in the Alps. – **Au**: V, T, K, St, O. **Ge**: Schw. **Sw**: SZ, VD. **Fr**: AHP, HAl, AMa, Sav, HSav. **It**: Frl, Ven, TAA, Piem, VA. **Sl**: SlA.

***Caloplaca austrocitrina* Vondrák, Říha, Arup & Søchting**

Syn.: *Flavoplaca austrocitrina* (Vondrák, Říha, Arup & Søchting) Arup, Søchting & Frödén

L – Subs.: cal – Alt.: 3–4 – Note: a species of the *C. citrina*-group with an areolate to subsquamulose, usually yellow to greenish-orange thallus and marginal soralia; apothecia not rare; mostly on artificial substrates like concrete or mortar in strongly manured places, *e.g.* in sites visited by dogs; certainly much more common in the Alps and hidden behind records of *C. citrina*. – **Au**: St. **Fr**: AMa. **It**: Frl.

***Caloplaca biatorina* (A. Massal.) J. Steiner**

Syn.: *Berengeria biatorina* (A. Massal.) Trevis., *Calogaya biatorina* (A. Massal.) Arup, Frödén & Søchting, *Caloplaca baumgartneri* Zahlbr., Caloplaca biatorina (A. Massal.) J. Steiner var. baumgartneri (Zahlbr.) Poelt, Caloplaca biatorina (A. Massal.) J. Steiner var. sympecta J. Steiner, *Caloplaca callopiza* (Nyl.) Jatta, *Gasparrinia biatorina* (A. Massal.) Szatala, *Lecanora callopiza* Nyl., Physcia elegans (Link) De Not. var. biatorina A. Massal., *Placodium biatorinum* (A. Massal.) M. Choisy, *Placodium callopizum* (Nyl.) Flagey

L – Subs.: cal, int – Alt.: 3–5 – Note: a holarctic species found in the mountains of Southern Europe; on limestone and dolomite, more rarely on base-rich siliceous rocks, most often at the top of isolated boulders in open, nitrogen-rich situations, mostly above or near treeline; widespread throughout the Alps. – **Au**: V, T, S, K, St, O, N. **Ge**: OB, Schw. **Sw**: BE, GR, LU, SZ, TI, VS. **Fr**: AHP, HAl, AMa, Sav, HSav. **It**: Frl, Ven, TAA, Lomb, Piem, VA.

***Caloplaca bryochrysion* Poelt**

Syn.: *Calogaya bryochrysion* (Poelt) Vondrák

L – Subs.: ter, bry, cal – Alt.: 4–5 – Note: on mosses, soil and plant debris over calcareous substrata, but also directly on calcareous rocks, in sheltered but light-rich situations, with optimum above treeline; very much overlooked, or confused with other sorediate species in the Alps; the relationships with *C. epiphyta* await clarification. – **Au**: V T S K St O N. **It**: Frl, Ven, TAA, Piem.

***Caloplaca cacuminum* Poelt**

Syn.: Callopisma aurantiacum (Lightf.) A. Massal. var. microsporum Arnold, Caloplaca aurantiaca (Lightf.) Th. Fr. var. microspora (Arnold) Dalla Torre & Sarnth.

L – Subs.: cal, int – Alt.: 4–5 – Note: a probably arctic-alpine species found on limestone and dolomite in exposed habitats, often starting the life-cycle on other lichens; perhaps more widespread in the Alps. – **Au**: V, T, S, K, St, O. **Ge**: OB, Schw. **Fr**: AHP, HAl, Sav. **It**: Frl, TAA, Piem, VA.

***Caloplaca caesiorufella* (Nyl.) Zahlbr.**

Syn.: *Caloplaca leptocheila* H. Magn., *Lecanora caesiorufella* Nyl.

L – Subs.: sil – Alt.: 4–5 – Note: a species of the *C. ferruginea*-group with an endosubstratic to thin, whitish-grey thallus and sessile apothecia with both disc and margin of a reddish-brown colour; based on a type from Bering Island offshore from Kamchatka, where it was found on twigs and plant remains, and widespread in the Arctic on driftwood, therefore records from siliceous rocks and conspecifity with *C. leptocheila* in need of re-evaluation. In the Alps usually on stones and low outcrops, probably widespread, but distribution insufficiently known. – **Au**: V, T, S, K, St.

***Caloplaca castellana* (Räsänen) Poelt**

Syn.: *Pachypeltis castellana* (Räsänen) Søchting, Frödén & Arup, *Placodium castellanum* Räsänen

L – Subs.: sil, int – Alt.: 4–5 – Note: a species with a thallus consisting of dispersed, brownish-orange squamules, often overgrowing *Spilonema*, and usually a single reddish apothecium per squamule; on steep rock faces of mineral-rich schists with variable contents of calcium; widespread in the Holarctic region, with scattered records from the Alps. – **Au**: V, T, S, K, St. **Sw**: VS.

***Caloplaca cerina* (Hedw.) Th. Fr.**

Syn.: *Blastenia nivea* B. de Lesd., *Callopisma cerinum* (Hedw.) De Not., Caloplaca cerina (Hedw.) Th. Fr. var. erhartii (Schaer.) Trevis., *Caloplaca gilva* (Hoffm.) Zahlbr., *Caloplaca gilvolutea* (Nyl.) Jatta, *Lecanora cerina* (Hedw.) Ach., *Lecanora gilvolutea* Nyl., *Lichen cerinus* Hedw., *Placodium cerinum* (Hedw.) Nägeli *ex* Hepp, *Placodium gilvum* (Hoffm.) Vain., *Zeora cerina* (Hedw.) Flot.

L – Subs.: cor, xyl – Alt.: 1–5 – Note: a holarctic, subtropical to boreal-montane lichen with optimum on smooth, mineral-rich bark (*e.g.* of *Acer*, *Fraxinus*, *Juglans*) but also on moderately eutrophicated bark of other trees, rare in polluted areas. In the complex of *C. cerina* *s.lat.* morphological differences among taxa are slight, while ecological and distributional differences are often remarkably clear. The treatment of this group is far from being complete, and it is still difficult to handle the nomenclature: at least some of the samples growing on plant debris are now segregated into *C. stillicidiorum*. – **Au**: V, T, S, K, St, O, N, B. **Ge**: OB, Schw. **Sw**: BE, GL, GR, FR, LU, SG, SZ, TI, UR, UW, VS. **Fr**: AHP, HAl, AMa, Drô, Isè, Sav, HSav, Var, Vau. **It**: Frl, Ven, TAA, Lomb, Piem, VA, Lig. **Sl**: SlA, Tg. **Li**.

***Caloplaca cerinella* (Nyl.) Flagey**

Syn.: *Athallia cerinella* (Nyl.) Arup, Frödén & Søchting, *Callopisma cerinellum* (Nyl.) Walt. Watson, *Caloplaca perfida* Malme, *Lecanora cerinella* Nyl., *Placodium cerinellum* (Nyl.) Vain.

L – Subs.: cor – Alt.: 1–3 – Note: a temperate species found on base – or nutrient-rich bark (*e.g.* very common on *Sambucus* or on *Juglans* in open habitats); widespread throughout the Alps. See also note on *C. cerinelloides*. – **Au**: V, T, S, K, St, O, N. **Ge**: OB, Schw. **Sw**: GR, LU, SZ, TI, VD, VS. **Fr**: AHP, AMa, Drô, Isè, HSav, Var, Vau. **It**: Frl, Ven, TAA, Lomb, Piem. **Sl**: SlA.

***Caloplaca cerinelloides* (Erichsen) Poelt**

Syn.: *Athallia cerinelloides* (Erichsen) Arup, Frödén & Søchting, Caloplaca pyracea (Ach.) Zwackh var. cerinelloides Erichsen

L – Subs.: cor, xyl – Alt.: 1–3 – Note: superficially resembling *C. cerinella*, but with a different number of spores per ascus. Also the ecology and distribution are different: *C. cerinelloides* has a more northern distribution and usually occurs on *Populus tremula*, or even on twigs of conifers. – **Au**: V, T, S, K, St, O, N. **Ge**: OB, Schw. **Sw**: BE, GL, GR, LU, SZ, TI, VS. **Fr**: AHP, AMa. **It**: TAA, Lomb, Piem. **Sl**: SlA, Tg.

***Caloplaca cerinoides* (Anzi) Jatta**

Syn.: *Placodium cerinoides* Anzi

L # – Subs.: sil, int – Alt.: 1–2 – Note: a poorly understood species of base-rich siliceous rocks, especially basalt. Earlier Italian records (none from the Alps) most likely refer to *C. thracopontica* Vondrák & Šoun, while *C. cerinoides* could prove to be a synonym of *C. atroflava*. – **Fr**: AHP, HAl, AMa, Sav.

***Caloplaca chalybaea* (Fr.) Müll. Arg.**

Syn.: *Caloplaca alpestris* *sensu* Ozenda & Clauzade, *Caloplaca olivacea* (A. Massal.) Jatta, Caloplaca variabilis (Pers.) Th. Fr. f. chalybaea (Fr.) Clauzade & Cl. Roux, Caloplaca variabilis (Pers.) Th. Fr. f. ochracea (Körb.) Müll. Arg., Caloplaca variabilis (Pers.) Th. Fr. subsp. ocellulata (Ach.) Boistel, Caloplaca variabilis (Pers.) Th. Fr. var. ocellulata (Ach.) Boistel, Caloplaca variabilis (Pers.) Th. Fr. var. ocellulata (Ach.) Boistel f. chalybaea (Fr.) Clauzade & Cl. Roux, *Parmelia chalybaea* Fr., *Placodium chalybaeum* (Fr.) Hepp, *Pyrenodesmia chalybaea* (Fr.) A. Massal., *Pyrenodesmia olivacea* A. Massal.

L – Subs.: cal – Alt.: 1–5 – Note: a mild-temperate species known from Europe and adjoining Africa and Asia, found on hard calciferous rocks (mostly on compact limestone) and dolomite, often, but not exclusively, on steeply inclined faces; widespread throughout the Alps. – **Au**: V, T, S, K, St, O, N. **Ge**: OB, Schw. **Sw**: BE, GR, LU, UW, VD, VS. **Fr**: AHP, HAl, AMa, Drô, Isè, Sav, HSav, Var, Vau. **It**: Frl, Ven, TAA, Lomb, Piem, VA, Lig. **Sl**: SlA, Tg.

***Caloplaca chanousiae* Sambo**

Syn.: *Fulgensia chanousiae* (Sambo) Poelt

L # – Subs.: int – Alt.: 5 – Note: on weakly calciferous schists; reported only from the Western and Southern Alps; a revision of the type material is badly needed. – **Fr**: AHP, HAl, AMa, Sav. **It**: TAA, VA.

***Caloplaca chlorina* (Flot.) H. Olivier**

Syn.: Caloplaca cerina (Hedw.) Th. Fr. var. chlorina (Flot.) Müll. Arg., Placodium cerinum (Hedw.) Hepp var. chlorinum (Flot.) Anzi, Zeora cerina (Hedw.) Flot. var. chlorina Flot.

L – Subs.: sil, int – Alt.: 2–5 – Note: on siliceous, nutrient-enriched rocks, mostly in upland areas; widespread throughout the Alps. – **Au**: T, S, K, St, O, N, B. **Ge**: OB, Schw. **Sw**: BE, GR, LU, TI, UR, UW, VS. **Fr**: AHP, AMa, Isè, Sav, HSav, Var. **It**: Frl, Ven, TAA, Lomb, Piem, VA. **Sl**: SlA.

***Caloplaca chrysodeta* (Vain.) Dombr. *comb. inval.***

Syn.: *Callopisma chrysodetum* (Vain.) Räsänen, *Leproplaca chrysodeta* (Vain.) J.R. Laundon *ex* Ahti, *Placodium chrysodetum* Vain.

L – Subs.: cal, int – Alt.: 2–4 – Note: a temperate to humid subtropical species found on shaded, steeply inclined or underhanging surfaces of calciferous rocks, sometimes also overgrowing epilithic mosses or even occurring on the undersides of inclined old trunks of trees with base-rich bark; widespread throughout the Alps. – **Au**: V, T, S, K, St, O, N, B. **Ge**: OB, Schw. **Sw**: BE, LU, SZ, VS. **Fr**: AHP, HAl, AMa, HSav, Var, Vau. **It**: Frl, Ven, Piem, Lig.

***Caloplaca chrysophthalma* Degel.**

Syn.: *Solitaria chrysophthalma* (Degel.) Arup, Søchting & Frödén

L – Subs.: cor, xyl – Alt.: 2–3 – Note: a rare mild-temperate lichen found on base-rich bark of isolated trees (*e.g. Populus*, *Juglans* and *Fraxinus*). – **Au**: T, St. **Ge**: OB, Schw. **Sw**: GR, LU, VS. **Fr**: AHP, HAl.

***Caloplaca cirrochroa* (Ach.) Th. Fr.**

Syn.: *Amphiloma cirrochroum* (Ach.) Körb., *Gasparrinia cirrochroa* (Ach.) Stein, *Lecanora cirrochroa* Ach., *Lecanora murorum* *auct.* var. cirrochroa (Ach.) Rabenh., *Leproplaca cirrochroa* (Ach.) Arup, Frödén & Søchting, Physcia callopisma (Ach.) A. Massal. var. cirrochroa (Ach.) A. Massal., *Physcia cirrochroa* (Ach.) Arnold, *Placodium cirrochroum* (Ach.) Rabenh.

L – Subs.: cal – Alt.: 1–5 – Note: a mainly temperate, probably holarctic species found on hard limestone and dolomite in rather shaded and sheltered situations, often on faces seldom wetted by rain; widespread throughout the Alps. – **Au**: V, T, S, K, St, O, N, B. **Ge**: OB, Schw. **Sw**: BE, GR, LU, SZ, UR, VD, VS. **Fr**: AHP, AMa, Drô, Isè, Sav, HSav, Var, Vau. **It**: Frl, Ven, TAA, Lomb, Piem, VA, Lig. **Sl**: SlA, Tg.

***Caloplaca citrina* (Hoffm.) Th. Fr.**

Syn.: *Amphiloma citrinum* (Hoffm.) Müll. Arg., *Blastenia citrina* (Hoffm.) B. de Lesd., *Callopisma citrinum* (Hoffm.) A. Massal., *Flavoplaca citrina* (Hoffm.) Arup, Frödén & Søchting, *Lecanora citrina* (Hoffm.) Ach., *Lichen citrinus* (Hoffm.) Ach., *Placodium citrinum* (Hoffm.) Hepp, *Pyrenodesmia citrina* (Hoffm.) Trevis., *Verrucaria citrina* Hoffm.

L – Subs.: cal, xyl – Alt.: 1–5 – Note: *C. citrina* is often claimed to be an almost cosmopolitan lichen. However, after a recent molecular revision of the entire complex, it seems that the species has a rather restricted distribution centered in Central Europe. The species complex, which still needs a thorough revision in the Alps, occurs on a wide variety of substrata, from asbestos-cement, concrete and mortar to basic siliceous rocks or even eutrophicated wood, being very tolerant to, and even favoured by eutrophication (*e.g.* urine-deposits). Several records could refer to other species in the complex. – **Au**: V, T, S, K, St, O, N, B. **Ge**: OB, Schw. **Sw**: BE, GR, LU, SZ, VS. **Fr**: AHP, HAl, AMa, Drô, Isè, Sav, HSav, Var, Vau. **It**: Frl, Ven, TAA, Lomb, Piem, VA, Lig. **Sl**: SlA.

***Caloplaca clauzadeana* (Gaya) Nav.-Ros. & Cl. Roux**

Syn.: *Caloplaca arnoldii* (Wedd.) Zahlbr. *ex* Ginzb. subsp. clauzadeana Gaya

L – Subs.: cal – Alt.: 2–3 – Note: a taxon of the *C. saxicola*-group forming large rosettes of densely pruinose thalli (resulting in a salmon colour), with strongly convex lobes; on vertical to overhanging walls of limestone at low elevations; in the study area so far only known from the southern part of the Western Alps. – **Fr**: AHP, Var, Vau.

***Caloplaca coccinea* (Müll. Arg.) Poelt**

Syn.: *Blastenia arnoldiana* Servít & Čern., *Blastenia coccinea* Müll. Arg., *Caloplaca arnoldiana* (Servít & Čern.) Servít & Poelt, Caloplaca aurantiaca (Lightf.) Th. Fr. f. nubigena (Arnold) Dalla Torre & Sarnth., *Caloplaca flammea* (Anzi) Jatta, *Placodium flammeum* Anzi

L – Subs.: cal – Alt.: 3–6 – Note: on steeply inclined faces of limestones and dolomite in upland areas; known from the mountains of Southern Europe and ranging throughout the Alps. – **Au**: V, T, S, K, St, O, N. **Ge**: OB, Schw. **Sw**: VD, VS. **Fr**: AHP, HAl, AMa, Isè, Sav, HSav, Vau. **It**: Frl, Ven, TAA, Lomb. **Sl**: SlA.

***Caloplaca conciliascens* (Nyl.) Zahlbr.**

Syn.: *Lecanora conciliascens* Nyl.

L – Subs.: sil, int – Alt.: 2–4 – Note: related to *C. exsecuta*, known from the *locus classicus* in Tyrol, from a single locality in the French Maritime Alps and in Central Switzerland, and from dry-warm sites in Piemonte. – **Au**: T. **Sw**: SZ. **Fr**: AMa. **It**: Piem.

***Caloplaca concinerascens* (Nyl.) H. Olivier**

Syn.: *Lecanora concinerascens* Nyl.

L # – Subs.: cal – Alt.: 2–4 – Note: this species (with a grey-brown epithecium reacting K+ weakly pale violet) belongs to the *Pyrenodesmia*-complex. It has been probably confused with *C. conversa* (with a brown epithecium reacting K+ purple), and its distribution is therefore poorly known. It was hitherto found on sun-exposed surfaces of weakly to strongly calciferous rocks in the French Southern Alps, the Mediterranean region and the warmest parts of the Massif Central. – **Fr**: AHP, AMa, Vau.

***Caloplaca conglomerata* (Bagl.) Jatta**

Syn.: *Callopisma conglomeratum* Bagl., *Caloplaca amabilis* Zahlbr., *Caloplaca peludella* (Nyl.) Hasse, *Lecanora peludella* Nyl.

L – Subs.: sil – Alt.: 1–2 – Note: a Mediterranean to xeric subtropical species of base-rich siliceous rocks, often growing with species of *Peltula*, and starting the life-cycle on other crustose lichens; mostly Mediterranean, but also found, although very rarely, in dry-continental valleys of the Alps. – **Fr**: AMa, Var, Vau. **It**: TAA, Lomb, Lig.

***Caloplaca conversa* (Kremp.) Jatta**

Syn.: *Callopisma conversum* Kremp., *Callopisma fallax* Bagl., Caloplaca conversa (Kremp.) Jatta var. fallax (Bagl.) Wunder, *Caloplaca fallax* (Bagl.) Jatta, *Caloplaca oreadum* (Stizenb.) Jatta, *Placodium conversum* (Kremp.) Anzi

L – Subs.: cal, int, sil – Alt.: 1–4 – Note: a mild-temperate to subtropical-montane lichen found on calciferous or basic siliceous rocks (especially basalt) in sunny sites with short periods of water seepage, often on colonies of cyanobacteria. According to Vondrák (see Nimis 2016) the species, which is related to *C. conglomerata*, is heterogeneous, and more species are involved, some of them fully lacking anthraquinones. See also note on *C. concinerascens*. – **Au**: V, T, S, K. **Ge**: OB, Schw. **Sw**: LU, SZ, TI, VS. **Fr**: AMa, Var. **It**: Frl, TAA, Lomb, Piem, VA.

***Caloplaca coralliza* Arup & Åkelius**

Syn.: *Blastenia coralliza* (Arup & Åkelius) Arup, Søchting & Frödén

L – Subs.: cor – Alt.: 3–4 – Note: a recently described species, which seems to be less common than the similar *C. herbidella* in the Alps. – **Au**: K. **Ge**: Ge. **Sw**: GR, UW.

***Caloplaca coronata* (Kremp. *ex* Körb.) J. Steiner**

Syn.: Callopisma aurantiacum (Lightf.) A. Massal. var. coronatum Kremp. *ex* Körb., Caloplaca aurantiaca (Lightf.) Th. Fr. var. coronata (Kremp. *ex* Körb.) Jatta, *Flavoplaca coronata* (Kremp. *ex* Korb.) Arup, Frödén & Søchting

L – Subs.: cal, cal-par – Alt.: 1–4 – Note: a mild-temperate to subtropical, mainly Mediterranean lichen found on the top of sun-exposed calcareous boulders, especially in small depressions of the rock, often starting the life-cycle on other crustose lichens; much overlooked in the past, and certainly more common; exceptionally reaching the subalpine belt on south-facing rocks in dry-continental valleys of the Alps. – **Au**: T, K, St, N, B. **Ge**: OB, Schw. **Sw**: LU, VS. **Fr**: AHP, AMa, Drô, HSav, Var, Vau. **It**: Frl, Ven, TAA, Lomb, Piem, Lig.

***Caloplaca cravensis* (Clauzade & Wunder) Cl. Roux**

Syn.: Caloplaca concinerascens (Nyl.) H. Olivier subsp. cravensis (Clauzade & Wunder) Clauzade & Cl. Roux, Caloplaca conversa (Kremp.) Jatta var. cravensis Clauzade & Wunder

L – Subs.: sil – Alt.: 2 – Note: a taxon of the *C. conversa*-aggregate, with thallus and apothecia reacting C+ red (gyrophoric acid); on pebbles and small boulders of hard siliceous rocks at low elevations; in the study area so far only known from the Western Alps. – **Fr**: Var, Vau.

**Caloplaca crenularia (With.) J.R. Laundon var. crenularia**

Syn.: *Blastenia crenularia* (With.) Arup, Søchting & Frödén, Blastenia ferruginea (Huds.) A. Massal. var. plumbea A. Massal., *Caloplaca caesiorufa* (Ach.) Flagey, *Caloplaca festiva auct. non* (Ach.) Zwackh, *Caloplaca sbarbaronis* B. de Lesd., *Lichen crenularius* With.

L – Subs.: sil, int – Alt.: 1–5 – Note: a temperate to subtropical species found on horizontal to weakly inclined faces of a wide variety of siliceous rocks, very heterogeneous, and in need of revision. According to Vondrák (see Nimis 2016), records of *C. crenularia* from (sub-)alpine habitats belong to a still undescribed species (Blastenia psychrophila *ined.*), which is known from Veneto and Piemonte in Italy. – **Au**: V, K, St. **Sw**: VS. **Fr**: AHP, HAl, AMa, Sav, HSav, Var, Vau. **It**: Frl, Ven, TAA, Lomb, Piem, VA, Lig. **Sl**: SlA.

**Caloplaca crenularia (With.) J.R. Laundon var. contigua (A. Massal.) *ined.* (provisionally placed here, ICN Art. 36.1b)**

Syn.: Caloplaca festiva (Ach.) Zwackh var. contigua (A. Massal.) H. Olivier, *Caloplaca squamulosa* *sensu* Ozenda & Clauzade *non* (Wedd.) B. de Lesd.

L # – Subs.: sil – Alt.: 2–3 – Note: a taxon belonging to a polymorphic complex, hitherto reported only from the Western Alps. See also note on the typical variety. – **Fr**: AHP, Var, Vau.

**Caloplaca crenularia (With.) J.R. Laundon var. depauperata (H. Magn.) Calat. & Barreno**

Syn.: Caloplaca festiva (Ach.) Zwackh var. depauperata H. Magn.

L # – Subs.: sil – Alt.: 2–3 – Note: a taxon belonging to a polymorphic complex, differing in the very poorly developed thallus, known from the French Southern Alps (at low elevations), the Mediterranean region, the Pyrenees, and Corsica. – **Fr**: AMa, Vau.

***Caloplaca crenulatella* (Nyl.) H. Olivier**

Syn.: Caloplaca lactea (A. Massal.) Zahlbr. f. aestimabilis (Arnold) Lettau, Caloplaca lactea (A. Massal.) Zahlbr. f. ecrustacea (Harm.) Zahlbr., *Lecanora crenulatella* Nyl., *Placodium crenulatellum* (Nyl.) A.L. Sm., *Xanthocarpia crenulatella* (Nyl.) Frödén, Arup & Søchting

L – Subs.: cal – Alt.: 1–4 – Note: a mild-temperate to subtropical species, often found on calcareous walls, perhaps parasitic of *Verrucaria nigrescens* when young. Very much misunderstood in the past (see also note on *C. lactea*): recently, this taxon has been considered to be paraphyletic and to contain at least four lineages. Some records from siliceous rocks could refer to *C. prinii* B. de Lesd. – **Au**: V, T, S, K, St, O, N. **Ge**: OB, Schw. **Sw**: GR, SZ, TI, VS. **Fr**: AHP, HAl, AMa, Drô, Isè, Sav, HSav, Var, Vau. **It**: Frl, Ven, TAA, Lomb, Piem, VA, Lig.

***Caloplaca dalmatica* (A. Massal.) H. Olivier *s.lat.***

Syn.: *Acarospora velana* A. Massal., Callopisma aurantiacum (Lightf.) A. Massal. var. placidium A. Massal., Callopisma aurantiacum (Lightf.) A. Massal. var. velanum A. Massal., *Callopisma dalmaticum* A. Massal., Caloplaca aurantiaca (Lightf.) Th. Fr. var. placidia (A. Massal.) Dalla Torre & Sarnth., Caloplaca aurantiaca (Lightf.) Th. Fr. var. velana (A. Massal.) Flagey, *Caloplaca dolomiticola* (Hue) Zahlbr., *Caloplaca placidia* (A. Massal.) J. Steiner, *Caloplaca schaereri* (Arnold) Zahlbr., *Caloplaca velana* (A. Massal.) Du Rietz, Caloplaca velana (A. Massal.) Du Rietz var. dalmatica (A. Massal.) Clauzade & Cl. Roux, Caloplaca velana (A. Massal.) Du Rietz var. dolomiticola (Hue) Clauzade & Cl. Roux, Caloplaca velana (A. Massal.) Du Rietz var. placidia (A. Massal.) Clauzade & Cl. Roux, Caloplaca velana (A. Massal.) Du Rietz var. schaereri (Arnold) Clauzade & Cl. Roux, *Lecanora dolomiticola* Hue, *Lecidea schaereri* Flörke *ex* Arnold *nom.illeg.*, *Lecidea velana* (A. Massal.) Hue, Placodium aurantiacum (Lightf.) Anzi var. velanum (A. Massal.) Anzi, *Variospora velana* (A. Massal.) Arup, Søchting & Frödén

L – Subs.: cal – Alt.: 1–5 – Note: extremely polymorphic and in need of revision: according to a broad species concept, *C. dalmatica* is the oldest name; on a wide variety of calcareous substrata in exposed, rather eutrophicated situations; widespread throughout the Alps. – **Au**: V, T, S, K, St, O, N, B. **Ge**: OB. **Sw**: BE, GR, SZ, TI, UR, VD, VS. **Fr**: AHP, HAl, AMa, Drô, Isè, Sav, HSav, Var, Vau. **It**: Frl, Ven, TAA, Lomb, Piem, VA, Lig. **Sl**: SlA.

***Caloplaca decipiens* (Arnold) Blomb. & Forssell**

Syn.: *Amphiloma decipiens* (Arnold) Bagl., *Calogaya decipiens* (Arnold) Arup, Frödén & Søchting, *Gasparrinia decipiens* (Arnold) Syd., *Lecanora decipiens* (Arnold) Nyl., *Physcia decipiens* Arnold, *Placodium decipiens* (Arnold) Leight.

L – Subs.: cal – Alt.: 2–4 – Note: a temperate, somehow subcontinental species found on calciferous substrata, especially on mortar walls; not common everywhere in the Alps, perhaps because of its subcontinental character, but abundant, and locally extremely abundant in some dry valleys of the Alps, mostly on walls in small villages, much rarer in natural habitats. – **Au**: V, T, S, K, St, O, N, B. **Ge**: OB, Schw. **Sw**: BE, GR, SZ, VD. **Fr**: AHP, HAl, AMa, Drô, Sav, HSav, Var, Vau. **It**: Frl. Ven, TAA, Piem, Lig.

***Caloplaca demissa* (Körb.) Arup & Grube**

Syn.: *Imbricaria demissa* Flot. *nom. inval.*, *Lecanora demissa* (Körb.) Zahlbr., *Lecanora incusa* (Flot.) Vain., *Placodium demissum* Körb.

L – Subs.: sil, int – Alt.: 1–4 – Note: a mild-temperate to xeric subtropical species found on south-facing, steeply inclined to underhanging surfaces of basic siliceous rocks, both in dry-warm valleys of the Alps (in the submediterranean belt) and in the Mediterranean belt; much less bound to water seepage than *Peltula euploca* and ecologically related species. This species, always sterile, was earlier positioned in the Lecanoraceae, in the genera *Lecanora* and *Placolecanora*, because of its general appearance, and has a quite remote position in the Teloschistaceae, with no supported sister lineage. It was segregated in the genus *Olegblumia*, but with a wrong basionym, which makes that name illegitimate. – **Au**: T, K, St, B. **Sw**: BE, GR, VS. **Fr**: AMa, Sav, Var. **It**: Ven, TAA, Lomb, Piem, VA. **Sl**: SlA.

***Caloplaca dichroa* Arup**

Syn.: *Flavoplaca dichroa* (Arup) Arup, Frödén & Søchting

L – Subs.: cal – Alt.: 2–3 – Note: a species recalling *C. citrina* in developing areoles dissolving more or less completely into blastidia and granules, usually fertile and then the thick-walled ascospores are diagnostic, often occurring in a yellow and an orange colour form; on limestone or more rarely on similar anthropogenic substrates, widespread in the Alps, but distribution still insufficiently documented. – **Au**: T, St. **Sw**: LU, SZ.

***Caloplaca diphyodes* (Nyl.) Jatta**

Syn.: *Callopisma diphyodes* (Nyl.) Bagl. & Carestia, Callopisma variabile (Pers.) Trevis. var. lecideina Müll. Arg., Caloplaca diphyodes (Nyl.) Jatta var. helygeoides (Vain.) H. Olivier, *Caloplaca helygeoides* (Vain.) Dalla Torre & Sarnth., *Caloplaca lecideina* (Müll. Arg.) Clauzade & Rondon *comb. inval.*, Caloplaca variabilis (Pers.) Th. Fr. subsp. diphyodes (Nyl.) Clauzade & Cl. Roux, Caloplaca variabilis (Pers.) Th. Fr. var. lecideina (Müll. Arg.) H. Olivier, *Lecanora diphyodes* Nyl.

L – Subs.: sil, int – Alt.: 4–6 – Note: known both from the Arctic and the mountains of the temperate zone, this lichen occurs on siliceous rocks in sheltered situations, often along creeks. The species has been much misunderstood, mainly due to the synonymisation with *C. lecideina* (Müll. Arg.) Clauzade & Rondon, a calcicolous species. The type of *C. diphyodes*, from Central France, is clearly silicicolous, and the species is more or less aquatic (Roux et al. 2014). – **Au**: V, T, S, K. **Sw**: SZ, TI. **Fr**: AHP, HAl, AMa, Drô, Sav, Vau. **It**: Frl, TAA, Piem.

***Caloplaca emilii* Vondrák, Khodos., Cl. Roux & V. Wirth**

Syn.: Caloplaca areolata *auct. p.p. non* (Zahlbr.) Clauzade

L – Subs.: cal – Alt.: 2 – Note: a species of the *C. xerica*-group (a secondary species of *C. areolata*) with a grey thallus, areoles with simple, globose blastidia produced along the edges, apothecia rather common, with discs in shades of orange-brown, and a grey thalline margin; on horizontal to slightly inclined rock faces of limestone outcrops in habitats with steppe-like conditions; widespread in the Mediterranean region, with several records from the Western Alps. – **Fr**: AHP, AMa, Drô, Var, Vau.

***Caloplaca epierodens* Cl. Roux & M. Bertrand**

Syn.: *Variospora epierodens* (Cl. Roux & M. Bertrand) Cl. Roux & M. Bertrand

L – Subs.: cal-par – Alt.: 4–5 – Note: a species of the *C. dalmatica*-group living as a parasite upon *C. erodens*, forming salmon-red thalli up to 1.5 cm in diam., with verruculose areoles separated by wide cracks, and broadly ellipsoid ascospores; on calcareous rocks with its host; so far only recorded from the Western Alps in France but likely to have a broader distribution. – **Fr**: AHP, AMa.

***Caloplaca epithallina* Lynge**

L – Subs.: sil-par – Alt.: 4–6 – Note: a rather continental species found on well-lit surfaces of siliceous rocks, *e.g.* on isolated boulders in grasslands, growing on other crustose and even foliose lichens (common hosts in the Alps are *Dimelaena oreina*, *Psorinia conglomerata*, *Rhizoplaca* spp., *Umbilicaria* spp.); most frequent in dry-continental valleys of the Alps. – **Au**: T, S, K, St. **Sw**: GR, VS. **Fr**: HAl, AMa, Sav. **It**: TAA.

***Caloplaca erodens* Tretiach, Pinna & Grube**

Syn.: *Pyrenodesmia erodens* (Tretiach, Pinna & Grube) Søchting, Arup & Frödén

L – Subs.: cal – Alt.: 3–4 – Note: a recently-described, usually sterile species, destructive to its substrate resulting in concave depressions with whitish marginal rims; on sunny rock walls and boulders of limestone, also on rock heads visited by birds; widespread in the Alps, but often overlooked. – **Au**: K, St, O. **Ge**: OB. **Fr**: AHP, AMa, HSav, Vau. **It**: Frl, Ven, TAA, Piem, VA.

***Caloplaca erythrocarpa* (Pers.) Zwackh**

Syn.: *Blastenia arenaria* *sensu* A. Massal., *Blastenia lallavei* (Clemente *ex* Ach.) A. Massal., Callopisma arenarium *auct. p.p. non* (Pers.) Müll. Arg., Caloplaca arenaria *auct. p.p. non* (Pers.) Müll. Arg., Caloplaca erythrocarpa (Pers.) Zwackh f. diffractoareolata B. de Lesd., *Caloplaca lallavei* (Clemente *ex* Ach.) Flagey, *Kuettlingeria lallavei* (Clemente *ex* Ach.) Trevis., *Lecidea lallavei* Clemente *ex* Ach., *Patellaria erythrocarpa* Pers., *Placodium lallavei* (Clemente *ex* Ach.) Anzi

L – Subs.: cal, int – Alt.: 1–3 – Note: a mainly mild-temperate to subtropical species found on limestone, dolomite, calciferous sandstone, much more rarely mortar and brick, on horizontal to weakly inclined faces wetted by rain, often starting the life-cycle on calcicolous *Aspicilia*-species; optimum in natural habitats; most frequent in the Southern and Western Alps, at low elevations. – **Sw**: ?VS. **Fr**: AHP, AMa, Drô, Isè, Var, Vau. **It**: Frl, Ven, TAA, Lomb, Piem. **Sl**: SlA, Tg.

***Caloplaca exsecuta* (Nyl.) Dalla Torre & Sarnth.**

Syn.: *Blastenia exsecuta* (Nyl.) Servít, *Lecanora exsecuta* Nyl.

L – Subs.: sil, int – Alt.: 4–5 – Note: a mainly boreal-montane, probably circumpolar, very variable lichen of basic siliceous rocks in humid, wind-protected situations; certainly much more widespread in the Alps. – **Au**: T, S, K, St. **Ge**: Schw. **Fr**: AHP, HAl, AMa, HSav. **It**: Frl, TAA, Piem, VA.

***Caloplaca ferrarii* (Bagl.) Jatta**

Syn.: *Callopisma ferrarii* Bagl., *Xanthocarpia ferrarii* (Bagl.) Frödén, Arup & Søchting

L – Subs.: cal – Alt.: 1–2 – Note: a temperate early coloniser of mortar walls, gypsum outcrops and other calciferous, often man-made, soft substrata at relatively low elevations; perhaps more widespread in lowland areas of the Alps. Recently, this taxon was shown to be paraphyletic and to contain at least two lineages. – **Au**: ?V. **Sw**: ?Sw. **Fr**: AHP, Drô, Var, Vau. **It**: Frl, TAA, Lomb, Piem, VA, Lig.

***Caloplaca ferruginea* (Huds.) Th. Fr.**

Syn.: *Biatora ferruginea* (Huds.) Fr., *Blastenia ferruginea* (Huds.) A. Massal., *Callopisma ferrugineum* (Huds.) Trevis., *Caloplaca aurantiaca* (Lightf.) Th. Fr. *non auct.*, *Gasparrinia ferruginea* (Huds.) Tornab., *Lecanora ferruginea* (Huds.) Link, *Lichen ferrugineus* Huds., *Placodium ferrugineum* (Huds.) Hepp

L – Subs.: cor – Alt.: 1–4 – Note: a mild-temperate species, with optimum on oaks in the submediterranean belt, absent from heavily disturbed areas. According to Vondrák (see Nimis 2016) three widespread species looking like “*C. ferruginea*” are known from Europe. Two of them are probably absent from the Alps, most of the records being from oceanic Europe and Macaronesia. The species common in the Alps has a mainly southern distribution in Europe, reaching Southern England and Central Europe (no recent records from Germany), and does not belong to *C. ferruginea* in the strict sense. – **Au**:  V, T, S, K, St, O, N. **Ge**: Ge. **Sw**: BE, GR, LU, SZ, TI, UR, VD, VS. **Fr**: AHP, HAl, AMa, Drô, Isè, Sav, HSav, Var, Vau. **It**: Frl, Ven, TAA, Lomb, Piem, VA. **Sl**: SlA, Tg.

***Caloplaca festivella* (Nyl.) Kieff.**

Syn.: Lecanora ferruginea (Huds.) Link var. festivella Nyl.

L – Subs.: sil – Alt.: 3–5 – Note: a rare, mainly oromediterranean species mainly found on schists, in underhangs, with a few records from the Western Alps (France, Italy). – **Fr**: Sav. **It**: Piem, Lig.

**Caloplaca flavescens (Huds.) J.R. Laundon var. flavescens**

Syn.: *Amphiloma heppianum* Müll. Arg., *Caloplaca heppiana* (Müll. Arg.) Zahlbr., *Caloplaca sympagaea* (Ach.) Sandst., *Gasparrinia heppiana* (Müll. Arg.) Verseghy, *Lecanora heppiana* (Müll. Arg.) Hue, *Lecanora sympagaea* Ach., *Lichen flavescens* Huds., *Physcia heppiana* (Müll. Arg.) Arnold, Placodium callopismum (Ach.) Mérat var. plicatum (Wedd.) Leight., *Placodium flavescens* (Huds.) A.L. Sm., *Placodium heppianum* (Müll. Arg.) Puget, *Placodium sympageum* (Ach.) Bremme, *Variospora flavescens* (Huds.) Arup, Frödén & Søchting

L – Subs.: cal, int – Alt.: 1–3 – Note: a mainly temperate species found on limestone, dolomite, calciferous sandstone, sometimes on brick, mortar and roofing tiles, and walls, monuments etc., somehow less helio – and xerophytic than the closely related *C. aurantia*; sometimes ascending to above treeline; widespread throughout the Alps. – **Au**: S, K, St, O, N, B. **Ge**: OB, Schw. **Sw**: VD, VS. **Fr**: AHP, HAl, AMa, Drô, Isè, Sav, HSav, Var, Vau. **It**: Frl, Ven, TAA, Lomb, Piem, VA, Lig. **Sl**: SlA, Tg.

**Caloplaca flavescens (Huds.) J.R. Laundon var. brevilobata (Nyl.) *ined.* (provisionally placed here, ICN Art. 36.1b)**

Syn.: *Caloplaca brevilobata* (Nyl.) Zahlbr., Caloplaca heppiana (Müll. Arg.) Zahlbr. var. brevilobata (Nyl.) A.E.Wade, *Lecanora brevilobata* Nyl.

L # – Subs.: sil – Alt.: 2–4 – Note: a taxon belonging to a polymorphic complex, hitherto reported only from the Western Alps (France). – **Fr**: Sav, Var.

***Caloplaca flavocitrina* (Nyl.) H. Olivier**

Syn.: Caloplaca citrina (Hoffm.) Th. Fr. var. flavocitrina (Nyl.) Walt. Watson, *Flavoplaca flavocitrina* (Nyl.) Arup, Frödén & Søchting, *Lecanora flavocitrina* Nyl.

L – Subs.: cal, sil – Alt.: 2–3 – Note: a species of the *C. citrina*-group mainly occurring on limestone, concrete and mortar, with a yellow to orange-yellow, areolate thallus, the areoles with marginal soralia, but often fertile; several records of this common, widespread and ecologically wide-ranging species might be filed under *C. citrina*. The entire group needs a thorough revision in the Alps. – **Au**: O. **Ge**: OB, Schw. **Sw**: SZ. **Fr**: AHP, AMa, Drô, HSav, Var, Vau. **It**: TAA. **Sl**: SlA.

***Caloplaca fulvolutea* (Nyl.) Jatta**

Syn.: *Callopisma fulvoluteum* (Nyl.) Arnold, Caloplaca jungermanniae (Vahl) Th. Fr. var. fuscolutoides Räsänen, *Lecanora fulvolutea* Nyl.

L – Subs.: bry – Alt.: 4–5 – Note: a taxon close to *C. jungermanniae* with intensely yellow apothecia showing persistently protruding margins; muscicolous, almost exclusively overgrowing *Grimmia* on acidic rocks; based on a type from Northern Finland, with a few records from the Alps. The species was also reported from Italy (outside the Alps), but these records refer to *C. chelyae* Pérez-Vargas. – **Fr**: HAl, HSav.

***Caloplaca furfuracea* H. Magn.**

Syn.: *Blastenia furfuracea* (H. Magn.) Arup, Søchting & Frödén

L – Subs.: xyl – Alt.: 3–4 – Note: all European collections are from Northern Sweden, apart from some specimens found in Wallis, Switzerland, and in France (Alps of Haute-Provence). Several specimens identified as *C. furfuracea* from other regions in Europe (Norway, Austria, Turkey and Croatia) were found to belong to *C. herbidella*. – **Sw**: VS. **Fr**: AHP.

***Caloplaca fuscoatroides* J. Steiner**

L – Subs.: sil, int – Alt.: 1 – Note: a widespread Mediterranean lichen found on basic siliceous rocks wetted by rain, also reported from the base of the Western Alps; the species is a member of the *C. xerica*-group, which is close to *Pyrenodesmia* *s.lat.*. – **Fr**: Vau.

***Caloplaca fuscorufa* H. Magn.**

L – Subs.: int – Alt.: 5 – Note: a species of the *C. ferruginea*-group with a whitish-grey, rimose to areolate thallus and sessile, brownish-red apothecia recalling those of *C. crenularia*, but with larger ascospores; forms with blackening margins may be also confused with *C. exsecuta*; on stones and low outcrops of calciferous schists, often near streams; the type is from central Sweden and the species is widespread in Northern Europe; from the Alps there is so far a single record, which needs re-confirmation. – **Sw**: SZ.

***Caloplaca glaucescens* (Bagl. & Carestia) Jatta**

Syn.: *Candelariella glaucescens* (Bagl. & Carestia) Lettau, *Gyalolechia glaucescens* Bagl. & Carestia

L # – Subs.: sil – Alt.: 2–3 – Note: a species of uncertain affinity, with a grey, granulose-verrucose thallus, small, sessile apothecia with a plane, reddish disc and a paler thalline margin, articulated paraphyses with a yellow cap, 8-spored asci, and oblong-ellipsoid, 1-septate ascospores (the septum not pervious) measuring 12–15 × 4–5 µm; known only from the type collection, on granite, at *c.* 3,000 m; the type material would be worthy of further study. – **It**: Piem.

***Caloplaca glomerata* Arup**

Syn.: *Variospora glomerata* (Arup) Arup, Søchting & Frödén

L – Subs.: cal – Alt.: 2 – Note: a species of the *C. dalmatica*-group with an areolate thallus, usually richly fertile, with several crowded apothecia per areole, and ascospores with lumina recalling the *Mischoblastia*-type; on limestone in *Aspicilia calcarea*-communities, often invading thalli of species of the *C. variabilis*-group; in the Alps so far only known from a single locality, but probably not distinguished in the past. – **Fr**: Vau.

***Caloplaca granulosa* (Müll. Arg.) J. Steiner**

Syn.: *Amphiloma granulosum* Müll. Arg., *Flavoplaca granulosa* (Müll. Arg.) Arup, Frödén & Søchting, *Placodium granulosum* (Müll. Arg.) Hepp

L – Subs.: cal, sil – Alt.: 1–3 – Note: a mild-temperate lichen found on compact limestone rocks, more rarely on dolomite, especially on weakly inclined faces with periodical seepage of nitrogen-rich solutions. – **Au**: V, K, St, N, B. **Fr**: AHP, HAl, AMa, Drô, HSav, Var, Vau. **It**: Frl, Ven, TAA.

***Caloplaca grimmiae* (Nyl.) H. Olivier**

Syn.: Caloplaca congrediens *auct. non* (Nyl.) Zahlbr., *Caloplaca consociata* J. Steiner, *Lecanora grimmiae* Nyl.

L – Subs.: sil-par, cal-par – Alt.: 1–5 – Note: a holarctic, temperate to boreal-montane lichen, whose ecology and distribution are narrower than those of its host (*Candelariella vitellina* *s.lat.*); it occurs throughout the Alps, and even in the Mediterranean belt, sometimes on walls, but, contrary to the host, it is absent from urban areas; widespread throughout the Alps. – **Au**: V, T, S, K, St, B. **Ge**: OB, Schw. **Sw**: BE, UR, VS. **Fr**: AHP, AMa, HSav, Var, Vau. **It**: Frl, Ven, TAA, Lomb, Piem, VA, Lig.

***Caloplaca haematites* (Chaub.) Zwackh**

Syn.: *Callopisma haematites* (Chaub.) A. Massal., *Lecanora haematites* Chaub., *Placodium haematites* (Chaub.) Anzi

L – Subs.: cor – Alt.: 1–2 – Note: a mainly Mediterranean lichen found on smooth bark, especially common on *Ficus carica*. The species, despite its similarity with *C. cerina*, according to Vondrák (see Nimis 2016), does not belong to *Caloplaca* *s.str.* and is related to *Pyrenodesmia* *s.lat.* Apparently more frequent in the Southern and Western Alps. The basionym’s authorship is often cited “Chaub. *ex* St.-Amans”, but in the introduction to the Flore Agenaise Saint-Amans clearly states, referring to Chaubard, that “*les lichens lui appartiennent en entier*”. See also note on *C. congrediens*. – **Sw**: VS. **Fr**: Sav, HSav, Var, Vau. **It**: Ven, TAA, Lomb, Piem, Lig.

***Caloplaca havaasii* H. Magn.**

Syn.: *Flavoplaca havaasii* (H. Magn.) Arup, Frödén & Søchting

L – Subs.: sil, int – Alt.: 4–5 – Note: a species with spreading thalli consisting of scattered, verruciform to squamulose, orange-red areolae, apothecia usually one per areolae, with persistent margins and finally sessile; on steep faces of siliceous schists at high elevations; rare in the Alps. – **Au**: T, S, K, St.

***Caloplaca herbidella* (Nyl. *ex* Hue) H. Magn.**

Syn.: *Blastenia herbidella* (Nyl. *ex* Hue) Servít, Lecidea caesiorufa (Wibel) Ach. f. herbidella Nyl. *ex* Hue

L – Subs.: cor, xyl – Alt.: 2–4 – Note: a species found on bark, especially on basal parts of trunks, more rarely on lignum. Populations from the Mediterranean zone (*e.g.* on *Olea* and *Juniperus*) could prove to belong to *C. coralliza*. According to Vondrák (see Nimis 2016) this species has a broadly Mediterranean distribution and is absent from America, with the easternmost limit in the Caucasus and an isolated population in the Urals, while there are some other (mostly undescribed) species which are acidophilous, growing typically on conifers and lignum, some of which may also occur in the Alps. – **Au**: V, T, S, K, St, O, N, B. **Ge**: OB, Schw. **Sw**: BE, GL, GR, LU, SG, SZ, TI, UR, UW, VD, VS. **Fr**: AHP, HAl, AMa, Drô, Isè, HSav, Var. **It**: Frl, Ven, TAA, Lomb, Piem, VA, Lig. **Sl**: SlA, Tg. **Li**.

***Caloplaca heufleriana* (A. Massal.) Zahlbr.**

Syn.: *Pyrenodesmia heufleriana* A. Massal.

L # – Subs.: sil – Alt.: ? – Note: a very poorly known species with a whitish-grey, areolate thallus, apothecia immersed at first, later sessile, with a black (brown when wet) disc, 8-spored asci, and bilocular, ellipsoid ascospores (*c.* 18 × 8 μm); on schistose rocks, described from Switzerland without a precise locality, and only known from the type (*ad saxa micacea Helvetiae*). – **Sw**: Sw.

***Caloplaca holocarpa* (Hoffm) A.E. Wade**

Syn.: *Athallia holocarpa* (Hoffm.) Arup, Frödén & Søchting, Callopisma aurantiacum (Lightf.) A. Massal. var. holocarpum (Hoffm.) A. Massal., Caloplaca aurantiaca (Lightf.) Th. Fr. var. holocarpa (Hoffm.) Th. Fr., Caloplaca pyracea (Ach.) Zwackh var. holocarpa (Hoffm.) Th. Fr., Placodium aurantiacum (Lightf.) Anzi var. holocarpum (Hoffm.) Anzi, Placodium pyraceum (Ach.) Anzi var. holocarpum (Hoffm.) Anzi, *Verrucaria holocarpa* Hoffm.

L # – Subs.: xyl, sil – Alt.: 1–5 – Note: this is a silicicolous, rarely lignicolous species of more or less eutrophicated habitats, mostly found on the top of isolated boulders. The epithet *holocarpa*, however, has been widely used for different lichens occurring both on bark and on calcareous rocks. Many records from the Alps could refer to other species. – **Au**: T, S, K, St, O, N, B. **Ge**: OB, Schw. **Sw**: BE, FR, GL, GR, LU, SG, SZ, TI, UR, UW, VD, VS. **Fr**: AHP, AMa, Isè, Sav, HSav, Vau. **It**: Frl, Ven, TAA, Lomb, Piem, VA, Lig. **Sl**: SlA, Tg. **Li**.

***Caloplaca hungarica* H. Magn.**

Syn.: *Blastenia hungarica* (H. Magn.) Arup, Søchting & Frödén, *Caloplaca depauperata* H. Magn. *non* (Müll. Arg.) Zahlbr., ?Caloplaca ferruginea (Huds.) Th. Fr. f. corticola Anzi, Caloplaca ferruginea (Huds.) Th. Fr. var. hungarica (H. Magn.) Clauzade & Cl. Roux, *Caloplaca subathallina* H. Magn.

L # – Subs.: cor, xyl – Alt.: 3–4 – Note: a temperate to boreal-montane lichen found on twigs of acid-barked trees, incl. oaks and *Larix*, perhaps overlooked, or confused with *C. ferruginea* in the past, but not common everywhere in the Alps, perhaps because of its subcontinental character. According to Vondrák (see Nimis 2016) the species, as currently understood, is not homogeneous: morphologically identical populations growing in Mediterranean habitats at low altitudes belong to “Blastenia xerothermica *ined.*”, which has a strictly Mediterranean distribution and is quite common in Italy and SE France. Some samples from lowland areas in the Southern Alps might belong to this still undescribed taxon, which is listed below. – **Au**: V, T, S, K, St, O. **Ge**: OB, Schw. **Sw**: GR, SZ, TI, VS. **Fr**: AHP, HAl, AMa, Isè, Sav, Vau. **It**: Frl, Ven, TAA, Lomb, Piem, VA, Lig. **Sl**: SlA.

**Caloplaca hungarica*auct. p.p. non* H. Magn**,

Syn.: Caloplaca ferruginea *auct. p.p. non* (Huds.) Th. Fr.

L # – Subs.: cor – Alt.: 1–2 – Note: a taxon of the *C. ferruginea*-group with an unresolved taxonomy and nomenclature, similar and probably related to *C. hungarica* H. Magn., characterised by pycnidia with black ostioles; on branches of various broad-leaved and coniferous trees in open habitats; widespread in the Mediterranean region, not rare at low elevations in the Western Alps (France), and likely to occur also in adjacent Italy. See also note to *C. hungarica* H. Magn. – **Fr**: AHP, Var, Vau.

***Caloplaca inconnexa* (Nyl.) Zahlbr.**

Syn.: Blastenia arenaria (Pers.) A. Massal. var. parasitica Jatta, Caloplaca percrocata (Arnold) J. Steiner var. parasitica Jatta, Caloplaca tenuatula (Nyl.) Zahlbr. subsp. inconnexa (Nyl.) Clauzade & Cl. Roux, *Lecanora inconnexa* Nyl.

L – Subs.: cal-par – Alt.: 1–4 – Note: a mild-temperate species found on the top of isolated calcareous boulders and rock outcrops, on calcareous rocks wetted by rain in sunny situations, especially common on *Acarospora cervina* and *Aspicilia calcarea*. The species, as currently understood, certainly belongs to *Athallia* (see Nimis 2016), but the type material is likely to belong in *Variospora*, so that the nomenclature of this lichen might change in the near future. The relationships with *C. necator* still remain to be clarified. – **Au**: ?V, ?T, N. **Fr**: AHP, HAl, AMa, Drô, Isè, Sav, HSav, Var, Vau. **It**: Frl, Ven, TAA, Lomb, Piem, VA, Lig. **Sl**: SlA.

***Caloplaca insularis* Poelt**

L – Subs.: cal-par, int-par – Alt.: 5–6 – Note: on calcareous schists, parasitic on *Aspicilia candida* and *A. polychroma*; certainly more widespread in the Alps. – **Au**: ?V, T, S, St. **Fr**: AHP, HAl, AMa. **It**: TAA. Piem, Lig.

***Caloplaca interfulgens* (Nyl.) J. Steiner**

Syn.: *Lecanora interfulgens* Nyl., *Xanthocarpia interfulgens* (Nyl.) Frödén, Arup & Søchting

L – Subs.: cal – Alt.: 2–4 – Note: a species of the *C. crenulatella-lactea*-group with a well-developed thallus consisting of yellow areoles becoming squamulose towards the margin; on limestone, mostly in at least seasonally dry habitats; in the study area so far only known from the Western Alps, in a xerothermic station. – **Fr**: AMa.

***Caloplaca interna* Poelt & Nimis**

L – Subs.: sil-par – Alt.: 1–2 – Note: on south-facing, vertical surfaces of basic siliceous rocks which are, albeit seldom, wetted by water seepage after rain, often found near *Peltula*-stands, but somehow less bound to periodical seepage of liquid water; the host (*Aspicilia* spec.) is mostly sterile; also found in dry-continental valleys of the Alps. – **It**: Piem.

***Caloplaca isidiigera* Vězda**

Syn.: Caloplaca cerina (Hedw.) Th. Fr. var. cyanopolia (Nyl.) H. Olivier, Placodium cerinum (Hedw.) Hepp f. cyanopolium (Nyl.) A.L. Sm.

L – Subs.: cal, int – Alt.: 3–4 – Note: on nutrient-enriched calcareous rocks, mostly in upland areas. This species was frequently confused with *C. emilii* (on exposed calcareous rocks in the Mediterranean and submediterranean belts) and *C. chlorina* (mainly on siliceous rocks); widespread in the Alps. – **Au**: V, T, S, K, St, O, N. **Ge**: OB. **Sw**: SZ. **Fr**: AHP, AMa, Sav, HSav. **It**: Frl, TAA, Lomb, Piem. **Li**.

***Caloplaca italica* B. de Lesd.**

L # – Subs.: cal – Alt.: 5–6 – Note: a calcicolous species characterised by the absence of a visible thallus, bright orange, immersed apothecia (0.2–0.3 mm in diam.) with a thin orange margin, and ascospores measuring 9–13 × 6–6.5 µm; known only from the type locality at *c.* 3,000 m. – **It**: Piem.

***Caloplaca jungermanniae* (Vahl) Th. Fr.**

Syn.: *Blastenia fuscolutea* (Ach.) A. Massal., *Bryoplaca jungermanniae* (Vahl) Søchting, Frödén & Arup, *Caloplaca fuscolutea* (Ach.) Th. Fr., *Lichen jungermanniae* Vahl, *Placodium fuscoluteum* (Ach.) Hepp

L – Subs.: deb, ter-cal – Alt.: 4–5 – Note: an arctic-alpine, circumpolar species of terricolous bryophytes and plant debris on calciferous substrata, but less common in areas with pure limestone; widespread in the Alps. – **Au**: V, T, S, K, St, N. **Ge**: OB. **Sw**: GR, VS. **Fr**: AHP, HAl, AMa, Sav. **It**: Ven, TAA, Lomb, Piem. **Sl**: SlA.

***Caloplaca lactea* (A. Massal.) Zahlbr.**

Syn.: *Blastenia lactea* (A. Massal.) Trevis., Callopisma luteoalbum (Turner) A. Massal. var. lacteum A. Massal., *Gyalolechia lactea* (A. Massal.) A. Massal., Placodium pyraceum (Ach.) Anzi var. lacteum (A. Massal.) A.L. Sm., *Xanthocarpia lactea* (A. Massal.) A. Massal.

L – Subs.: cal – Alt.: 1–6 – Note: a mainly Mediterranean to temperate species, an early coloniser of small calcareous pebbles in open habitats (*e.g.* stony ground in dry grasslands); in the Alps it is more frequent in dry-warm areas. – **Au**: V, T, S, K, St, O, N, B. **Ge**: OB, Schw. **Sw**: GR, LU, SZ, VD, VS. **Fr**: AHP, HAl, AMa, Drô, Isè, Sav, Var, Vau. **It**: Frl, Ven, TAA, Lomb, Piem, VA, Lig. **Sl**: SlA, Tg.

***Caloplaca lacteoides* Nav.-Ros. & Hladun**

L – Subs.: cal – Alt.: 2–3 – Note: a species of the *C. crenulatella-lactea*-group with an endolithic thallus, finally sessile apothecia which are less than 0.5 mm in diam. and of a egg-yolk yellow colour, narrowly ellipsoid ascospores longer than 16 µm, with a thin (less than 3 µm) septum; usually on calcareous pebbles and low outcrops in *Aspicilia contorta*-communities, but also on concrete; mainly Mediterranean, with some records from the Western Alps only. – **Fr**: AHP, AMa, Var, Vau.

***Caloplaca ligustica* B. de Lesd.**

Syn.: *Caloplaca pyrithromoides* (Nyl.) C.W. Dodge, *Chrysomma pyrithromoides* (Nyl.) M. Choisy & Werner

L – Subs.: sil – Alt.: 1–2 – Note: on schists in open habitats, on dry surfaces with a short water flow after rain; poorly understood, but probably a good species known from Italy, France, and the Iberian Peninsula. The type specimen (PRA-V 03097), from the base of the Western Alps (Spotorno) was morphologically appraised by Vondrák (see Nimis 2016) and belongs to *Rufoplaca*. – **It**: Lig.

***Caloplaca limonia* Nimis & Poelt**

Syn.: *Flavoplaca limonia* (Nimis & Poelt) Arup, Frödén & Søchting

L – Subs.: sil, cal – Alt.: 1–2 – Note: a species of the *C. citrina*-group with an areolate, pale yellow thallus, the areoles and the apothecial margins developing blastidia on the entire surface; on calcareous rocks or on base-rich, hard siliceous cliffs in dry and sun-exposed to shaded and damp situations, but also on twigs of maritime shrubs or on soil, below the montane belt. The species, described from the calcareous cliffs along the coast of the Island of Marettimo, is also known from inland localities, and is certainly more widespread; earlier records might be under *C. citrina* *s.lat.* – **Fr**: AHP, AMa, Var. **It**: Ven.

***Caloplaca livida* (Hepp) Jatta**

Syn.: *Blastenia livida* (Hepp) Lettau, *Callopisma lividum* (Hepp) Körb., *Caloplaca convexa* (Schaer.) Zahlbr., *Placodium lividum* Hepp

L – Subs.: bry, deb – Alt.: 4–5 – Note: an arctic-alpine species found on plant debris and bryophytes overgrowing acid siliceous rocks, with optimum near and above treeline. – **Au**: T. **Sw**: BE, GR, LU, UW. **Fr**: HAl, Sav. **It**: TAA, Lomb, Piem, VA.

***Caloplaca lobulata* (Flörke) Hellb.**

Syn.: *Calogaya lobulata* (Flörke) Arup, Frödén & Søchting, *Caloplaca boulyi* (Zahlbr.) M. Steiner & Poelt, *Lecanora lobulata* Flörke, Parmelia parietina (L.) Ach. var. lobulata (Flörke) Fr., *Xanthoria boulyi* Zahlbr., *Xanthoria lobulata* (Flörke) B. de Lesd., Xanthoria parietina (L.) Th. Fr. var. lobulata (Flörke) Rabenh., Xanthoria parietina (L.) Th. Fr. var. turgida (Schaer.) Arnold

L – Subs.: cor, xyl – Alt.: 1–3 – Note: a mild-temperate, subcontinental species, most frequent in the inner-Alpine dry valleys. – **Au**: T, K, St, O, N. **Sw**: GR, LU, VD, VS. **Fr**: Sav, HSav. **It**: Ven, TAA, Lomb, Piem.

***Caloplaca lucifuga* G. Thor**

L – Subs.: cor, xyl – Alt.: 2–3 – Note: a temperate species found on ancient, more or less isolated deciduous trees, especially oaks or *Castanea*, often in crevices of rough bark and on faces seldom wetted by rain; rare in the Alps. – **Au**: St. **Ge**: OB. **Sw**: GR, TI. **It**: Piem, Lig.

***Caloplaca luctuosa* (Anzi) Jatta**

Syn.: *Biatorina luctuosa* Anzi

L # – Subs.: sil – Alt.: 2–3 – Note: a species with a granulose, olive-coloured thallus delimited by a black prothallus, small, zeorine, black, sessile apothecia with a plane to convex disc, a greenish brown epihymenium and a pale hypothecium, 8-spored asci, and 1-septate, ellipsoid ascospores with a thick epispore, measuring *c.* 14 × 8 µm; this is probably a species of *Lecania* (see Nimis 1993: 190). – **It**: Lomb.

***Caloplaca luteoalba* (Ach.) Th. Fr.**

Syn.: *Biatorina luteoalba* (Ach.) Körb., *Callopisma luteoalbum* (Ach.) A. Massal., Caloplaca luteoalba (Ach.) Th. Fr. var. persooniana (Ach.) H. Olivier, *Candelariella luteoalba* (Ach.) Lettau, *Cerothallia luteoalba* (Ach.) Arup, Frödén & Søchting, *Gyalecta persooniana* Ach., *Gyalolechia luteoalba* (Ach.) Arnold, *Lecanora ulmicola* (DC.) Hue, *Lecidea cinereofusca* (Weber) Ach. v. *luteoalba* Ach. *Placodium luteoalbum* (Ach.) Anzi

L – Subs.: cor – Alt.: 2 – Note: a mild-temperate lichen found on dust-covered bark, and in the wound tracks of injured, old trunks of deciduous trees, especially *Ulmus*; more frequent in the past, now strongly declining and perhaps extinct in several parts of the Alps. – **Fr**: Var. **It**: Ven, Lomb, Piem. **Sl**: SlA.

***Caloplaca macrocarpa* (Anzi) Zahlbr.**

Syn.: ?*Caloplaca alpigena* Poelt *ined.*, Placodium aurantiacum (Lightf.) Anzi var. macrocarpon Anzi, *Variospora macrocarpa* (Anzi) Arup, Søchting & Frödén

L # – Subs.: cal – Alt.: 3–5 – Note: a species of the *C. dalmatica*-group with a thallus of dispersed areoles in shades of orange, partly covered by sessile, relatively large (*c.* 1 mm in diam.), orange-red apothecia with persistent, paler margins; on subhorizontal rock faces and rocky heads at high elevations; widespread in the Alps but regionally undercollected. According to Vondrák (see Nimis 2016), however, at least the material called *C. macrocarpa* by Poelt and Hafellner (GZU) belongs to a different species. – **Au**: V, T, S, K, St, O, N. **Ge**: OB. **It**: Lomb.

***Caloplaca magni-filii* Poelt**

L – Subs.: sil-par – Alt.: 4–5 – Note: a species of the *C. ferruginea*-group with a strongly reduced thallus and small rusty-red apothecia with soon excluded margins; exclusively parasitic on thalli of the silicicolous *Miriquidica nigroleprosa*, usually from the high montane to the lower alpine belt; widespread in the European parts of the Holarctic, including the Alps, but rare. – **Au**: T, K, St. **Sw**: UR. **Fr**: HSav.

**Caloplaca marmorata*auct. non* (Bagl.) Jatta**

Syn.: Callopisma marmoratum *auct. non* Bagl., Caloplaca lactea (A. Massal.) Zahlbr. f. fulva (Harm.) Zahlbr., Caloplaca lactea (A. Massal.) Zahlbr. f. rubra (B. de Lesd.) Zahlbr., Gyalolechia lactea (A. Massal.) A. Massal. f. rubra B. de Lesd., Lecanora lactea (A. Massal.) Leight. f. fulva Harm., *Xanthocarpia marmorata* (*auct. non* Bagl.) Frödén, Arup & Søchting

L – Subs.: cal – Alt.: 2 – Note: a species of the *C. crenulatella-lactea*-group, with an endolithic thallus and finally sessile, rusty-red apothecia which are less than 0,5 mm in diam.; usually on limestone pebbles; widespread in the Mediterranean region, with some records from the Western Alps, at low elevations. According to Nimis (2016), the type of *Callopisma marmoratum* Bagl. (MOD-TSB) clearly belongs to the lichen which is usually called “*Caloplaca subochracea*” (see note on that species), a fact that will have quite heavy nomenclatural consequences to be dealt with in future studies. – **Fr**: AHP, AMa, Drô, Var, Vau.

***Caloplaca microphyllina* (Tuck.) Hasse**

Syn.: *Placodium microphyllinum* Tuck.

L – Subs.: xyl, cor – Alt.: 2–3 – Note: a mainly xeric subtropical to mild-temperate lichen of continental areas, found on basal parts of trunks, rarely on eutrophicated lignum, described from North America and also reported from dry valleys of the Alps. – **Au**: K, St. **It**: TAA.

***Caloplaca monacensis* (Leder.) Lettau**

Syn.: Callopisma cerinum (Hedw.) De Not. var. cyanoleprum (DC.) A. Massal., Caloplaca cerina (Hedw.) var. cyanolepra (DC.) J. J.Kickx, Patellaria cerina (Hedw.) Hoffm. var. cyanolepra DC., *Pyrenodesmia monacensis* Leder.

L – Subs.: cor – Alt.: 2–3 – Note: a species of the *C. cerina*-group with an almost completely granulose thallus and apothecia of the *C. cerina*-type, usually with whitish-pruinose margins; on bark of deciduous trees along roads or in rural landscapes; widespread in Europe with scattered records from the Alps, but not common. – **Au**: V, S, K, St, N, B. **Ge**: OB. **Sw**: SZ. **Fr**: Sav. **It**: Frl, Ven, TAA. **Sl**: SlA, Tg.

***Caloplaca nana* (Gaya) Nav.-Ros. & Cl. Roux**

Syn.: *Caloplaca arnoldii* (Wedd.) Zahlbr. *ex* Ginzb. subsp. nana Gaya

L # – Subs.: cal, int – Alt.: 3 – Note: this species has the same ecology as *C. arnoldii*; from the Alps there are only a few scattered records. – **Au**: N. **Sw**: SZ.

***Caloplaca navasiana* Nav.-Ros. & Cl. Roux**

Syn.: *Flavoplaca navasiana* (Nav.-Ros. & Cl. Roux) Arup, Søchting & Frödén

L – Subs.: cal – Alt.: 1 – Note: a species with an endolithic to whitish, strongly reduced thallus, sessile, orange apothecia of biatorine appearance due to a strongly developed parathecium, and ascospores with internal thickenings measuring *c.* half of the total length; on limestone close to the seashore, widespread along the coasts of the Mediterranean Sea, including the base of the Western Alps. – **Fr**: AMa.

***Caloplaca necator* Poelt & Clauzade**

Syn.: Caloplaca inconnexa (Nyl.) Zahlbr. var. nesodes Poelt & Nimis, *Athallia nesodes* (Poelt & Nimis) Halıcı & Vondrák *comb. inval*.

L – Subs.: sil – Alt.: 1–2 – Note: a mainly Mediterranean species found on siliceous rocks, starting the life-cycle as a parasite of *Aspicilia*-species, especially *A. viridescens*. According to Roux et al. (2014) the smaller size of spores distinguishing *C. necator* from C. inconnexa var. nesodes is due to the fact that the holotype material of the former is poorly developed: other specimens (isotype) collected at the type locality show the same spore size range as the latter taxon. – **Fr**: Var, Vau. **It**: Lig.

***Caloplaca nideri* J. Steiner**

L – Subs.: cal – Alt.: 2 – Note: a species with an orange thallus of *c.* 2 cm diam. (recalling *C. flavescens*), the lobes *c.* 1 mm wide, more or less concolorous apothecia, and ellipsoid ascospores with rather thin septa; a mainly eastern Mediterranean, calcicolous species based on a type from Greece; in the Alps so far only known from a single lowland locality close to the eastern border. – **Au**: N.

***Caloplaca nivalis* (Körb.) Th. Fr.**

Syn.: *Bacidia livida* (Bagl. & Carestia) Lettau, *Bilimbia livida* Bagl. & Carestia, *Candelariella nivalis* (Körb.) Lettau, *Gyalolechia nivalis* (Körb) A. Massal., *Placodium nivale* (Körb.) Tuck., *Zeora nivalis* Körb.

L – Subs.: bry, deb, sil, ter – Alt.: 4–5 – Note: a cool-temperate to boreal-montane, circumpolar species found on silicicolous mosses (mainly *Andreaea* and *Grimmia*); widespread in the Alps. See also note on *C. tornoensis*. – **Au**: V, T, S, K, St, N. **Sw**: BE, UR, VS. **Fr**: AHP, Sav, HSav. **It**: TAA, Lomb, Piem, VA.

**Caloplaca nubigena (Kremp.) Dalla Torre & Sarnth. var. nubigena**

Syn.: Callopisma ochraceum (Schaer.) A. Massal. var. nubigenum Kremp.

L – Subs.: cal, int – Alt.: 4–6 – Note: on calcareous rocks in upland areas, often near summits. Similar to *C. coccinea*, but differing in morphology, thallus colour, altitudinal distribution (not restricted to above treeline) and in its parasitism on *Clauzadea immersa*; widespread in the Alps. – **Au**: V, T, S, K, St, O, N. **Ge**: OB. **Sw**: BE, VD. **Fr**: AHP, HAl, AMa, Drô, Isè, Sav, Var, Vau. **It**: Frl, TAA. **Sl**: SlA.

**Caloplaca nubigena (Kremp.) Dalla Torre & Sarnth. var. keissleri (Servít) Clauzade & Cl. Roux**

Syn.: *Blastenia keissleri* Servít, *Caloplaca keissleri* (Servít) Poelt

L – Subs.: cal – Alt.: 3–5 – Note: a taxon with an endolithic thallus indicated by usually whitish patches and sunken orange apothecia with slightly concave discs and thin parathecial margins (somehow recalling *Protoblastenia incrustans*); on boulders and outcrops of limestone; fairly common at higher elevations in the eastern Mediterranean region; apparently more frequent in the Western Alps. – **Au**: V, T. **Fr**: AHP, AMa, Drô, Var, Vau.

**Caloplaca oasis (A. Massal.) Szatala f. oasis**

Syn.: Callopisma aurantiacum (Lightf.) A. Massal. var. oasis A. Massal., Caloplaca aurantiaca (Lightf.) Th. Fr. f. oasis (A. Massal.) Th. Fr., *Flavoplaca oasis* (A. Massal.) Arup, Frödén & Søchting

L – Subs.: cal-par – Alt.: 1–4 – Note: a mild-temperate lichen found on hard, compact limestones in sheltered sites with plenty of diffuse light, such as in open deciduous forests; parasitic on endolithic lichens, especially *Bagliettoa*-species without an involucrellum (mainly *B. calciseda*, sometimes *B. marmorea*). – **Au**: T, St. **Ge**: OB, Schw. **Sw**: VS. **Fr**: AHP, AMa, Drô, Isè, Sav, HSav, Var, Vau. **It**: Ven.

***Caloplaca oasis* (A. Massal.) Szatala f. lithophila*auct.***

Syn.: Caloplaca holocarpa *auct. ital. p.p.*, *Caloplaca lithophila auct. non* H. Magn., Caloplaca luteoalba (Ach.) Th. Fr. var. saxicola (Hepp) H. Olivier

L – Subs.: cal – Alt.: 2–5 – Note: this lichen, which is quite common on limestone and mortar, has been much misunderstood. In our opinion, it differs from *C. oasis*, because of the very different ecology and life-cycle (it is not parasitic on *Bagliettoa*-species). – **Au**: V, T, K, St, N. **It**: Frl, Ven TAA, Lomb, Piem, VA, Lig. **Sl**: SlA.

***Caloplaca obliterans* (Nyl.) Blomb. & Forssell**

Syn.: Caloplaca cirrochroa (Ach.) Th. Fr. var. obliterans (Nyl.) Servít, *Gasparrinia obliterans* (Nyl.) Dalla Torre & Sarnth., *Lecanora obliterans* (Nyl.) Lamy, *Leproplaca obliterans* (Nyl.) Arup, Frödén & Søchting, *Physcia obliterans* (Nyl.) Arnold, Placodium cirrochroum (Ach.) Rabenh. var. obliterans (Nyl.) A.L. Sm., *Placodium obliterans* Nyl.

L – Subs.: sil, int – Alt.: 3–5 – Note: a cool-temperate to boreal-montane, circumpolar species found in underhangs of basic siliceous rocks, especially calcareous schists, mostly in upland areas. – **Au**: T, S, K, St. **Ge**: OB, Schw. **Sw**: GR, SZ, VS. **Fr**: AHP, AMa, HSav. **It**: Ven, TAA, Lomb, Piem, VA, Lig.

***Caloplaca obscurella* (J. Lahm *ex* Körb.) Th. Fr.**

Syn.: *Blastenia obscurella* J. Lahm *ex* Körb., *Callopisma obscurellum* (J. Lahm *ex* Körb.) J. Lahm, *Caloplaca refellens* (Nyl.) H. Olivier, *Lecanora obscurella* (J. Lahm *ex* Körb.) Lamy, *Lecanora refellens* Nyl., *Placodium refellens* (Nyl.) A.L. Sm.

L – Subs.: cor – Alt.: 1–3 – Note: a temperate, perhaps holarctic species found on isolated deciduous trees, not uncommon in orchards. The basionym is often cited as “J. Lahm *ex* Körb.”, but Körber explicitly attributes the species to Lahm (“*Lahm in litt. ad Kbr*.”). See also note *C. sarcopisioides*. – **Au**: S, K, St, O. **Ge**: Ge. **Sw**: LU, SZ. **Fr**: AHP, AMa, Var, Vau. **It**: Frl, Ven, TAA, Lomb, Piem, VA. **Sl**: SlA, Tg.

***Caloplaca ochracea* (Schaer.) Th. Fr.**

Syn.: Biatora aurantiaca (Lightf.) Fr. var. ochracea (Schaer.) Rabenh., *Blastenia ochracea* (Schaer.) Trevis., *Callopisma ochraceum* (Schaer.) A. Massal., *Callopisma tetrastichum* (Nyl.) Walt. Watson, Caloplaca aurantiaca (Lightf.) Th. Fr. var. ochracea (Schaer.) H. Olivier, *Caloplaca tetrasticha* (Nyl.) H. Olivier, *Gyalolechia ochracea* (Schaer.) Syd., Lecanora aurantiaca (Lightf.) Flot. var. ochracea (Schaer.) Nyl., *Lecanora ochracea* (Schaer.) Nyl., *Lecidea ochracea* Schaer., *Placodium ochraceum* (Schaer.) Anzi, *Placodium tetrastichum* (Nyl.) H. Olivier, *Xanthocarpia ochracea* (Schaer.) A. Massal. & De Not.

L – Subs.: cal – Alt.: 1–3 – Note: a warm-temperate species found on hard, compact limestones in more or less sheltered situations. – **Au**: K, N. **Sw**: LU, SZ, UW, VD, VS. **Fr**: AHP, HAl, AMa, Drô, Isè, Sav, HSav, Var, Vau. **It**: Frl, Ven, TAA, Lomb, Piem, Lig. **Sl**: SlA, Tg.

***Caloplaca paulii* Poelt**

Syn.: *Variospora paulii* (Poelt) Arup, Søchting & Frödén

L – Subs.: sil – Alt.: 4–5 – Note: a species with a thick, areolate, dull orange thallus with indistinctly lobate marginal areolae, relatively large apothecia with zeorine margins (the thalline rim soon excluded), and narrowly fusiform ascospores with thin septa; on limestone and marl slates at high altitudes; also known from various Eurasian orobiomes and from Greenland, but rather rare. – **Au**: V, T, S, K, St. **Fr**: AHP, HAl, AMa, Sav.

***Caloplaca percrocata* (Arnold) J. Steiner**

Syn.: *Blastenia percrocata* Arnold, *Callopisma percrocatum* (Arnold) Jatta

L – Subs.: sil, int – Alt.: 4–5 – Note: on base-rich and lime-containing siliceous rocks. – **Au**: V, T. **Ge**: Schw. **Sw**: GR, SZ, TI. **Fr**: AHP, HAl, AMa, HSav. **It**: Ven, TAA, Piem, Lig.

***Caloplaca pollinii* (A. Massal.) Jatta**

Syn.: *Blastenia pollinii* A. Massal., Callopisma ferrugineum (Huds.) Trevis. var. pollinii (A. Massal.) Bagl., *Callopisma pollinii* (A. Massal.) Trevis., *Caloplaca phaeocarpella* (Nyl.) Zahlbr., *Lecanora nigricans* (Tuck. *ex* Nyl.) Nyl., *Lecanora phaeocarpella* Nyl., *Lecidea gibberosa* Pollini *non* Ach., *Placodium phaeocarpellum* (Nyl.) A.L. Sm., *Placodium pollinii* (A. Massal.) A.L. Sm., *Huneckia pollinii* (A. Massal.) S.Y. Kondr., Elix, Kärnefelt, A. Thell, J. Kim, A.S. Kondratyuk & Hur

L – Subs.: cor, xyl – Alt.: 1–2 – Note: a warm-temperate species, mostly found on the smooth bark of trees such as *Alnus* along rivers; much more common in the past, presently extinct over much of its former range. – **Au**: N. **Ge**: OB. **Sw**: TI, UR. **Fr**: AHP, AMa, Var, Vau. **It**: Frl, Ven, Lomb, Piem, Lig. **Sl**: SlA, Tg.

***Caloplaca polycarpa* (A. Massal.) Zahlbr.**

Syn.: Callopisma aurantiacum (Lightf.) A. Massal. var. polycarpum A. Massal., *Callopisma polycarpum* (A. Massal.) A. Massal., Caloplaca inconnexa (Nyl.) Zahlbr. var. verrucariarum Clauzade & Cl. Roux [invalidly published, ICN Art. 40.1 + 8], Caloplaca polycarpa (A. Massal.) Zahlbr. subsp. verrucariarum Cl. Roux, *Caloplaca tenuatula* (Nyl.) Zahlbr., Caloplaca tenuatula (Nyl.) Zahlbr. subsp. verrucariarum (Clauzade & Cl. Roux) Clauzade & Cl. Roux *comb. inval.*, *Flavoplaca polycarpa* (A. Massal.) Arup, Frödén & Søchting, *Lecanora tenuatula* Nyl.

L – Subs.: cal-par – Alt.: 1–3 – Note: a mainly warm-temperate species found on compact limestone, more rarely dolomite, in sheltered situations, with optimum in open woodlands, growing on the thalli of *Bagliettoa*-species with an involucrellum, especially *B. parmigera* and *B. parmigerella*. The species is morphologically variable but, pending further study, it is still treated here in a very broad sense (for a different arrangement see Roux et al. 2014). In the past, it might have been confused with *C. oasis*. – **Au**: T, St, N. **Sw**: GR, UR, VS. **Fr**: AHP, AMa, Drô, HSav, Var, Vau. **It**: Frl, Ven, TAA, Lomb, Piem, VA, Lig.

***Caloplaca prinii* B. de Lesd.**

Syn.: *Caloplaca clauzadei* B. de Lesd., *Caloplaca diffusa* Vondrák & Llimona

L # – Subs.: sil – Alt.: 2–3 – Note: a taxon of the *C. crenulatella-lactea*-group with a thallus consisting of minute orange granules which also develop on the outside of the apothecial margins, and narrowly ellipsoid ascospores with thin septa; perhaps lichenicolous, overgrowing *Aspicilia*-species, on basic siliceous pebbles; in the strict sense only known from the type locality in the Western Alps at a low elevation, not far from the Mediterranean coast. – **Fr**: AHP, AMa, Var.

***Caloplaca proteus* Poelt**

Syn.: Caloplaca cirrochroa (Ach.) Th. Fr. subsp. fulva (Körb.) Clauzade & Cl. Roux, *Leproplaca proteus* (Poelt) Arup, Frödén & Søchting, Physcia murorum *auct.* var. placibilis Kremp., Placodium pusillum (A.Massal) Anzi var. miniatum *sensu* Anzi

L – Subs.: cal – Alt.: 3–5 – Note: on steeply inclined to underhanging surfaces of compact, more or less calcareous rocks in rather sheltered situations, most frequent in warm-dry sites in the Alps. – **Au**: V, T, S, K, St, O, N. **Ge**: OB. **Sw**: GR. **Fr**: AMa, HSav, Var. **It**: Ven, TAA, Lomb, Piem, VA. **Sl**: SlA.

***Caloplaca pseudofulgensia* Gaya & Nav.-Ros.**

L – Subs.: cal – Alt.: 2–4 – Note: a calcicolous and nitrophilous species, nearly always found growing with *C. pusilla*. – **Au**: N. **Sw**: LU, SZ. **Fr**: Vau.

***Caloplaca pulchrevirens* (Anzi) Jatta**

Syn.: *Placodium pulchrevirens* Anzi

L # – Subs.: cor – Alt.: 2–3 – Note: this species should be characterised by the bright green thallus and the small, urceolate, immersed apothecia, but the colour of the thallus could have been due to deposits of substances containing copper, used in agriculture against plant pathogens (see Nimis 1993: 190); the type material badly needs revision. – **It**: Lomb.

***Caloplaca pusilla* (A. Massal.) Zahlbr.**

Syn.: *Callopisma pusillum* (A. Massal.) Trevis., *Caloplaca murorum* *auct.* var. pulvinata (A. Massal.) Jatta, *Caloplaca murorum* *auct.* f. pulvinata (A. Massal.) Ozenda & Clauzade, Caloplaca saxicola (Hoffm.) Nordin subsp. pulvinata (A. Massal.) Clauzade & Cl. Roux, *Gasparrinia pusilla* (A. Massal.) Tornab., *Physcia murorum* *auct.* var. pulvinata A. Massal., *Physcia murorum* *auct.* var. pulvinata A. Massal. f. euphora A. Massal., *Physcia pusilla* A. Massal., Physcia pusilla A. Massal. var. turgida A. Masssal., *Placodium pusillum* (A. Massal.) Anzi, *Calogaya pusilla* (A. Massal.) Arup, Frödén & Søchting

L – Subs.: cal – Alt.: 1–4 – Note: a common and widespread, coniophilous and ornithocoprophilous species growing mainly on walls and horizontal surfaces of calcareous rocks, frequently confused with *C. saxicola*. See also note on *C. saxicola*. – **Au**: T, N. **Ge**: OB, Schw. **Sw**: LU, SZ. **Fr**: AHP, HAl, AMa, Drô, Isè, Sav, HSav, Var, Vau. **It**: Frl, Ven, TAA, Lomb, Piem, VA, Lig.

***Caloplaca pyracea* (Ach.) Zwackh**

Syn.: *Athallia pyracea* (Ach.) Arup, Frödén & Søchting, *Lecanora pyracea* (Ach.) Nyl., Parmelia cerina (Hedw.) Ach. var. pyracea Ach., *Placodium pyraceum* (Ach.) Anzi

L – Subs.: cor, cal, sil, xyl – Alt.: 2–6 – Note: a temperate to boreal-montane, holarctic lichen found on nutrient-rich or eutrophicated bark of isolated trees (mainly *Acer*, *Fraxinus* and *Juglans*), with a wide altitudinal range; widespread throughout the Alps. – **Au**: V, T, S, K, St, O, N, B. **Ge**: OB, Schw. **Sw**: SZ. **Fr**: AHP, HAl, AMa, Isè, Sav, HSav, Var, Vau. **It**: Frl, Ven, TAA, Lomb, Piem, VA, Lig.

***Caloplaca raesaenenii* Bredkina**

Syn.: *Caloplaca thuringiaca* Søchting & Stordeur, *Placodium geophilum* Räsänen *non* Th. Fr.

L – Subs.: deb, xyl – Alt.: 2–3 – Note: a submediterranean-Turanic to west-Pontic species of the *C. holocarpa*-group, with an endosubstratic to greenish-grey, crustose thallus and bright orange apothecia; on plant remains and dead branches of half-shrubs under more or less continental conditions; from the Alps there are so far a few records only. – **Sw**: VS. **It**: TAA.

***Caloplaca rouxii* Gaya, Nav.-Ros. & Llimona**

Syn.: *Caloplaca murorum* *auct.* var. miniata (Hoffm.) Ozenda & Clauzade, Caloplaca saxicola (Hoffm.) Nordin subsp. miniata (Hoffm.) Clauzade & Cl. Roux, *Lichen miniatus* Hoffm. *non* L.

L – Subs.: cal – Alt.: 4–5 – Note: a species with an Alpine distribution, growing mainly on the top of calcareous boulders in sunny and nutrient-enriched sites, often with *C. biatorina* and *Xanthoria elegans*; probably much more widespread in the Alps. – **Au**: K, St. **Fr**: AHP, HAl, AMa, Sav.

***Caloplaca rubelliana* (Ach.) Lojka**

Syn.: *Callopisma rubellianum* (Ach.) A. Massal., *Lecanora rubelliana* Ach.

L – Subs.: sil – Alt.: 1–3 – Note: a warm-temperate to subtropical, widespread lichen found on hard, basic siliceous rocks (especially basalt), often with *Acarospora sulphurata*; apparently more frequent in the Southern and Western Alps. – **Au**: T, K. **Sw**: UR, VS. **Fr**: HAl, AMa, Var, Vau. **It**: Ven, TAA, Lomb, Piem, VA.

***Caloplaca rubroaurantiaca* B. de Lesd.**

L # – Subs.: sil – Alt.: 1–4 – Note: a member of the difficult *C. arenaria*-group, characterised by smaller spores with a thinner septum and the orange apothecia, with some scattered records from the Alps. – **Sw**: VS. **Fr**: AHP, HAl, AMa. **It**: Piem, VA, Lig.

***Caloplaca sarcopisioides* (Körb.) Zahlbr.**

Syn.: *Callopisma sarcopisioides* Körb.

L # – Subs.: cor – Alt.: 3 – Note: closely related to (or identical with) *C. obscurella*, this name is applied to richly fruiting populations with endophloeodic to thin scurfy thallus lacking the crateriform soralia diagnostic for *C. obscurella*; based on a type from Croatia; on eutrophic bark of *e.g.* roadside trees at low elevations; distribution poorly documented because often not distinguished from *C. obscurella*. – **Au**: O.

***Caloplaca saxicola* (Hoffm.) Nordin**

Syn.: *Amphiloma murorum* Körb. *nom.illeg.*, *Callopisma murorum* De Not. *nom.illeg.*, *Callopisma steropeum* (Ach.) Körb., *Caloplaca murorum* Th. Fr. *nom.illeg.*, Caloplaca tegularis *auct. non* (Ehrh.) Zahlbr., *Gasparrinia murorum* Tornab. *nom. lleg.*, *Physcia murorum* A. Massal. *nom.illeg.*, *Placodium murorum* DC. *nom.illeg.*, *Psora saxicola* Hoffm., *Xanthoria murorum* Th. Fr. *nom.illeg*.

L # – Subs.: cal, int, sil – Alt.: 1–5 – Note: a mainly cool-temperate, much misunderstood and still problematic species occurring both on calcareous and basic siliceous rocks, also in urban areas and on man-made substrata. Many records could refer to *C. pusilla*. – **Au**: V, T, S, K, St, O, N, B. **Ge**: OB, Schw. **Sw**: BE, GR, SZ, UR, UW, VD, VS. **Fr**: AHP, HAl, AMa, Vau. **It**: Ven, Frl. **Sl**: SlA, Tg.

***Caloplaca saxifragarum* Poelt**

Syn.: *Athallia saxifragarum* (Poelt) Arup, Frödén & Søchting, Callopisma luteoalbum (Ach.) A. Massal. var. microcarpum (Anzi) Anzi, Callopisma pyraceum (Ach.) Stein var. microcarpum (Anzi) Arnold, Caloplaca pyracea (Ach.) Zwackh var. microcarpa (Anzi) Dalla Torre & Sarnth., *Caloplaca schoeferi* Poelt, Placodium luteoalbum (Ach.) Hepp var. microcarpum Anzi

L – Subs.: bry, deb – Alt.: 4–6 – Note: a circumpolar, arctic-alpine lichen found on plant debris (especially on dead leaves of *Saxifraga*, *Dryas* and *Carex firma*), and on moribund bryophytes in open habitats over calcareous or dolomitic substrata, most common above treeline; widespread throughout the Alps. – **Au**: V, T, S, K, St, O, N. **Ge**: OB, Schw. **Sw**: GR, UR, VS. **Fr**: AHP, HAl, Isè, AMa, Sav, HSav. **It**: Frl, Ven, TAA, Lomb, Piem, VA, Lig. **Sl**: SlA.

***Caloplaca schistidii* (Anzi) Zahlbr.**

Syn.: *Calogaya schistidii* (Anzi) Arup, Frödén & Søchting, *Candelariella schistidii* (Anzi) Lettau, *Fulgensia schistidii* (Anzi) Poelt, *Gyalolechia schistidii* Anzi, Lecidea luteoalba (Ach.) Ach. var. muscicola *sensu* Schaer.

L – Subs.: bry-cal, bry-ter – Alt.: 2–5 – Note: on pulvinate epilithic mosses (mainly *Grimmia anomodon* and *Schistidium apocarpum*) over calcareous substrata; widespread throughout the Alps. – **Au**: V, T, S, K, St, O, N. **Ge**: OB, Schw. **Sw**: BE, GR, UR, UW, VD, VS. **Fr**: AHP, HAl, AMa, Drô, Isè, Sav, HSav, Var, Vau. **It**: Ven, TAA, Lomb, Piem, VA. **Sl**: SlA.

***Caloplaca scotoplaca* (Nyl.) H. Magn.**

Syn.: Caloplaca caesiorufa *auct. non* (Ach.) Flagey, *Lecanora scotoplaca* Nyl., *Rufoplaca scotoplaca* (Nyl.) Arup, Søchting & Frödén

L – Subs.: sil – Alt.: 2–3 – Note: a species of the *C. arenaria*-group with a dark grey to blackish, minutely areolate thallus, and usually numerous, small, rusty-brown apothecia with concolorous margins and broadly ellipsoid ascospores with septal thickening measuring ⅓-½ of the total length; on siliceous rocks in slightly manured places; overall distribution holarctic, in the Alps insufficiently documented, but evidently not common. – **Au**: K. **Fr**: AHP. **It**: TAA, Lomb, Piem.

***Caloplaca sinapisperma* (DC.) Maheu & A. Gillet**

Syn.: *Blastenia leucoraea* (Ach.) Th. Fr., *Blastenia sinapisperma* (DC.) A. Massal., *Bryoplaca sinapisperma* (DC.) Søchting, Frödén & Arup, *Caloplaca leucoraea* (Ach.) Branth, Lecidea ferruginea (Huds.) Chevall. var. sinapisperma (DC.) Schaer., Lecidea fuscolutea Ach. var. leucoraea Ach., *Lichen sinapispermus* (DC.) DC., *Patellaria sinapisperma* DC., *Placodium sinapispermum* (DC.) Hepp

L – Subs.: bry, deb, ter-cal – Alt.: 3–5 – Note: a holarctic lichen ranging from the Arctic zone to the high southern mountains, found on mosses and plant debris on calcareous or base-rich siliceous substrata, with optimum near or above treeline, sometimes reaching the montane belt in open habitats; widespread throughout the Alps. – **Au**: V, T, S, K, St, O, N. **Ge**: OB, Schw. **Sw**: BE, FR, GR, SZ, TI, UR, UW, VD, VS. **Fr**: AHP, HAl, AMa, Isè, Sav, HSav, Vau. **It**: Frl, Ven, TAA, Lomb, Piem, VA. **Sl**: SlA. **Li**.

***Caloplaca soralifera* Vondrák & Hrouzek**

L – Subs.: cal – Alt.: 2–3 – Note: a species of the *C. xerica*-group with a grey, but often whitish-pruinose, areolate thallus, the areolae with marginal soralia, apothecia (if present) with orange to brown disc, a zeorine exciple with an orange parathecium and a grey thalline margin, ascospores ellipsoid, with septal thickening *c.* 1/3 to 1/2 as long as the total length; on concrete, mortar or siliceous pebbles in manured places; hitherto mainly reported from Eastern Europe but probably more widespread, at least in the continental inner Alpine valleys. – **Au**: St. **It**: TAA.

***Caloplaca sorocarpa* (Vain.) Zahlbr.**

Syn.: *Placodium sorocarpum* Vain.

L – Subs.: cor – Alt.: 3–5 – Note: a usually sterile lichen with a grey thallus and concolorous, circular, often raised soralia; easily overlooked, it is one of the most common sorediate crusts growing on the branches of *Rhododendron*; still undercollected in some parts of the Alps. – **Au**: V, T, S, K, St. **Sw**: GR, SZ, VS. **It**: Frl, TAA.

***Caloplaca spotornonis* B. de Lesd.**

L # – Subs.: sil, int – Alt.: 1–2 – Note: a species with a thin, glaucous grey thallus of flat, angulose, to 0.9 mm wide areoles forming a crust of 1–2 cm in diam., numerous contiguous apothecia arising from the areoles (0.3–0.5 mm in diam.), the disc plane, orange, the margin thin and concolourous, 8-spored asci, and oblong-ellipsoid, polar-diblastic spores with a rather thin septum, measuring 10–14(-15) × 3.5–4 µm; known only from the type collection, on schist near Spotorno. – **It**: Lig.

***Caloplaca squamulata* (Nyl.) H. Olivier**

Syn.: Caloplaca variabilis (Pers.) Th. Fr. var. squamulata (Nyl.) Boistel, *Lecanora squamulata* Nyl., *Pannaria squamulata* (Nyl.) Hue, *Psoroma squamulatum* (Nyl.) Hue

L – Subs.: cor – Alt.: 2 – Note: a species with a whitish-grey, minutely squamulose thallus and *Lecanora*-like apothecia, perhaps a taxon of the *C. obscurella*-group; on bark of deciduous trees at low elevations; extremely rare, in the study area so far only known from a single locality of the Eastern Alps. – **Au**: O.

***Caloplaca stillicidiorum* (Vahl) Lynge *s.lat.***

Syn.: Callopisma cerinum (Hedw.) De Not. var. muscorum A. Massal., Caloplaca cerina (Hedw.) Th. Fr. f. chloroleuca Sm., Caloplaca cerina (Hedw.) Th. Fr. var. chloroleuca (Sm.) Th. Fr., Caloplaca cerina (Hedw.) Th. Fr. var. muscorum (A. Massal.) Jatta, Caloplaca cerina (Hedw.) Th. Fr. var. stillicidiorum (Vahl) Th. Fr., *Caloplaca muscorum* (A. Massal.) M. Choisy & Werner, *Lichen stillicidiorum* Vahl, *Placodium cerinum* (Hedw.) Nägeli *ex* Hepp var. stillicidiorum (Vahl) Hepp

L – Subs.: bry, deb, cor, ter-cal – Alt.: 3–5 – Note: a mainly arctic-alpine, circumpolar lichen found on mosses and plant debris in tundra-like habitats, especially in areas with calcareous or basic siliceous rocks. This is the only taxon of the *C. cerina*-group hosting the parasite *Stigmidium cerinae*, which suggests that it could be an independent species. The *Caloplaca cerina*-like lichens growing on the ground on various substrata such as bryophytes, plant debris, bark of exposed roots and chamaephytes form four monophyletic groups and six ecotypes, which cannot be always distinguished on the basis of morphological characters. – **Au**: V, T, S, K, St, O, N. **Ge**: OB, Schw. **Sw**: SZ. **Fr**: AHP, HAl, AMa, Drô, Isè, Sav, HSav, Vau. **It**: Frl, Ven, TAA, Lomb, Piem, VA, Lig. **Sl**: SlA. **Li**.

***Caloplaca subalpina* Vondrák, Šoun & Palice**

L – Subs.: cal – Alt.: 4 – Note: mostly on vertical, sheltered, but well-lit rocks beneath overhangs; in the Alps only known from Austria, on base-rich schist. – **Au**: St.

***Caloplaca submergenda* (Nyl.) H. Olivier**

Syn.: *Caloplaca nigrozonata* (Lamy) Zahlbr., *Lecanora nigrozonata* Lamy, *Lecanora submergenda* Nyl., Lecanora submergenda Nyl. var. nigrozonata (Lamy) H. Olivier

L # – Subs.: sil-aqu, sil – Alt.: 2–3 – Note: a species (of the *C. cerina*-group?) with a thin, rimose to areolate, grey thallus and sessile apothecia with reddish-yellow discs and zeorine margins with an entire thalline rim, recalling *C. diphyodes* or *C. aractina*, but ascospores distinctly smaller; on siliceous, periodically submerged rocks and boulders along streams and headwaters of rivers; not common, in the study area so far reported from a few localities in the Western Alps. – **Fr**: AMa, Var.

**Caloplaca subochracea*auct. non* (Wedd.) Clauzade & Cl. Roux var. subochracea**

L – Subs.: cal – Alt.: 1 – Note: a group of closely related taxa with a rimose to areolate thallus of various colours and rusty-brown apothecia; on limestone boulders and outcrops at sites not far from the Mediterranean sea, usually in rather shaded situations; widespread but not common, in the study area only known from low elevations at the base of the Western Alps. There are open issues with the epithet of this lichen, since the type material by Weddell corresponds to a species which is not the same as that called *Caloplaca subochracea* by most recent authors (see Roux et al. 2014). Furthermore, according to Nimis (2016) the type of *Callopisma marmoratum* Bagl. (MOD-TSB) clearly belongs to this lichen and not to that which is usually called “*Caloplaca marmorata*” by most authors. – **Fr**: AMa.

**Caloplaca subochracea*auct. non* (Wedd.) Clauzade & Cl. Roux var. luteococcinea Cl. Roux**

Syn.: Caloplaca aurantiaca (Lightf.) Th. Fr. var. africana *sensu* Ozenda & Clauzade *non* Flagey, *Caloplaca subochracea* *auct. non* (Wedd.) Clauzade & Cl. Roux var. luteococcinea Clauzade & Cl. Roux [invalidly published, ICN Art. 40.1 + 8]

L – Subs.: cal – Alt.: 2 – Note: a strain with a thallus of a bright yellow colour, found in Mediterranean lowlands close to the sea, including the base of the Western Alps (France); distribution as the typical variety, but rarer. – **Fr**: AMa.

***Caloplaca subpallida* H. Magn.**

Syn.: Caloplaca oxfordensis *auct. non* Fink *ex* J. Hedrick, *Rufoplaca subpallida* (H. Magn.) Arup, Søchting & Frödén

L # – Subs.: sil – Alt.: 2–4 – Note: a cool-temperate to arctic-alpine lichen found on mineral-rich siliceous rocks, sometimes parasitic on other lichens (*Aspicilia*, *Rhizocarpon*, *Xanthoparmelia*). The species has been often considered as a synonym of *C. oxfordensis* J. Hedrick, a taxon described from North America, but molecular data indicate that the two species are different. – **Au**: T, S, K, St. **Sw**: TI, VS. **Fr**: AHP, AMa, Var. **It**: Frl, Ven, TAA, Piem, VA.

***Caloplaca subsoluta* (Nyl.) Zahlbr.**

Syn.: Callopisma aurantiacum (Lightf.) A. Massal. var. irrubescens Arnold, Caloplaca aurantia (Pers.) Hellb. var. irrubescens (Arnold) Jatta, *Caloplaca irrubescens* (Arnold) Zahlbr., Lecanora murorum (Ach.) Ach. subsp. subsoluta Nyl., *Lecanora subsoluta* (Nyl.) Nyl., Placodium aurantiacum (Lightf.) Anzi subsp. irrubescens (Arnold) A.L. Sm., *Squamulea subsoluta* (Nyl.) Arup, Søchting & Frödén

L – Subs.: sil – Alt.: 1–3 – Note: a mild-temperate to subtropical, widespread lichen found on steeply inclined, sunny surfaces of basic siliceous rocks, often with *Peltula euploca*, but less bound to seepage tracks. – **Au**: ?V, T, K, St. **Sw**: TI, VS. **Fr**: HAl, AMa, Sav, Var. **It**: Frl, Ven, TAA, Lomb, Piem, VA.

***Caloplaca substerilis* Vondrák, Palice & van den Boom**

L – Subs.: cor – Alt.: 3 – Note: a recently-described species with a pale grey to white, partly endophloeodal, partly diffuse, minutely squamulose thallus, with soralia in cracks of the bark or marginally on squamules, apothecia rare; on nutrient-rich bark in continental parts of Europe, with a few records from the Eastern Alps. – **Au**: K, St.

***Caloplaca teicholyta* (Ach.) J. Steiner**

Syn.: *Blastenia teicholyta* (Ach.) Bausch, *Blastenia visianica* A. Massal., *Callopisma visianicum* (A. Massal.) Trevis., Caloplaca arenaria *auct. p.p. non* (Pers.) Müll. Arg., Caloplaca erythrocarpa *auct. p.p. non* (Pers.) Zwackh, *Caloplaca visianica* (A. Massal.) Jatta, *Kuettlingeria teicholyta* (Ach.) Trevis., *Kuettlingeria visianica* (A. Massal.) Trevis., *Lecanora teicholyta* Ach., *Placodium teicholytum* (Ach.) DC.

L – Subs.: cal, int – Alt.: 1–3 – Note: a warm-temperate early coloniser of calciferous substrata (but very rare on pure limestone), often found on sandstone and mortar, mostly on man-made substrata (walls, monuments, roofing tiles, brick walls), common also in settlements; widespread throughout the Alps, at low elevations. – **Au**: T, K, St, O, N, B. **Sw**: BE, LU, SZ, VS. **Fr**: AHP, HAl, AMa, Drô, Sav, HSav, Var, Vau. **It**: Frl, Ven, TAA, Lomb, Piem, VA, Lig.

***Caloplaca tenuata* (Nyl.) Zahlbr.**

Syn.: *Lecanora tenuata* (Nyl.) Nyl., *Placodium tenuatum* Nyl.

L – Subs.: cal – Alt.: 2 – Note: an often misunderstood taxon with the appearance of a minute, lobate *Caloplaca* of a yellow-orange colour and ascospores whose septum is *c.* ¼ of the total spore length; on very sunny surfaces of limestone in the Mediterranean region, with some records from the Western Alps only, but certainly more widespread. – **Fr**: AHP, AMa, Var, Vau.

***Caloplaca tetraspora* (Nyl.) H. Olivier**

Syn.: *Blastenia tetraspora* (Nyl.) Rehm, *Bryoplaca tetraspora* (Nyl.) Søchting, Frödén & Arup, *Caloplaca oligospora* Th. Fr., *Lecanora tetraspora* Nyl.

L – Subs.: ter-cal, deb – Alt.: 4–5 – Note: a boreal-montane to arctic-alpine, circumpolar species found on bryophytes and plant debris in areas with base-rich or somehow calciferous siliceous substrata; certainly more widespread in the Alps. – **Au**: V, T, S, K, St, N. **Ge**: OB, Schw. **Sw**: GR, SZ, VS. **Fr**: HAl, HSav. **It**: Frl, TAA, Piem.

***Caloplaca tiroliensis* Zahlbr.**

Syn.: Callopisma cerinum (Hedw.) De Not. f. flavum (Anzi) Dalla Torre & Sarnth., Caloplaca cerina (Hedw.) Th. Fr. f. flava (Anzi) Jatta, Caloplaca cerina (Hedw.) Th. Fr. var. flava (Anzi) Dalla Torre & Sarnth., *Caloplaca friesii* H. Magn., Caloplaca jungermanniae (Vahl) Th. Fr. var. subolivacea (Th. Fr.) Th. Fr., *Caloplaca subolivacea* (Th. Fr.) Lynge, *Parvoplaca tiroliensis* (Zahlbr.) Arup, Søchting & Frödén, *Placodium cerinum* (Hedw.) Nägeli *ex* Hepp var. flavum Anzi

L – Subs.: bry, deb, ter-cal – Alt.: 3–6 – Note: a holartic, arctic-alpine species, mainly found on mosses and plant debris, often on leaves of *Saxifraga*, in *Carex firma*-stands over calcareous substrata; common in the Alps near and above treeline, up to the nival belt. – **Au**: V, T, S, K, St, O, N. **Ge**: OB, Schw. **Sw**: GR, LU, SZ, UR, VD, VS. **Fr**: AHP, HAl, AMa, Isè, Sav, HSav. **It**: Frl, Ven, TAA, Lomb, Piem, VA. **Li**.

***Caloplaca tominii* Savicz**

Syn.: *Xanthocarpia tominii* (Savicz) Frödén, Arup & Søchting

L – Subs.: ter-cal – Alt.: 4 – Note: on calciferous soils in dry grasslands of continental valleys of the Alps; the total distribution is incompletely and fragmentarily circumboreal in the Northern Hemisphere. – **Au**: T.

***Caloplaca tornoensis* H. Magn.**

L – Subs.: bry – Alt.: 4–5 – Note: a species recalling *C. nivalis*, but with ellipsoid to fusiform ascospores, with wider septal thickenings; usually muscicolous (*Andreaea*, *Grimmia*) over siliceous rocks; widespread in the Arctic region; in the Alps so far only known from a single locality, but perhaps sometimes misidentified as *C. nivalis*. – **Au**: S.

***Caloplaca tremniacensis* (A. Massal.) Jatta**

Syn.: *Callopisma tremniacense* A. Massal., *Candelariella tremniacensis* (A. Massal.) Lettau

L # – Subs.: cal – Alt.: 2–3 – Note: a species with a dark grey, areolate thallus, the areoles first concave and contiguous, then separate and becoming verrucose, yellow apothecia arising from the center of the areoles, first immersed, then sessile, with a flat disc and a thin concolour margin, a yellowish epiphymenium, clavate paraphyses, 8-spored asci, and polar-diblastic ascospores that are *c.* 2 times as long as wide; an interesting taxon, known only from the Eastern Pre-Alps, whose type material well deserves further study (see Nimis 1993: 191). – **It**: Ven.

***Caloplaca tristiuscula* H. Magn.**

Syn.: *Rufoplaca tristiuscula* (H. Magn.) Arup, Søchting & Frödén

L # – Subs.: sil, int – Alt.: 4–5 – Note: a silicicolous species of the *C. arenaria*-group with a dark grey to blackish-grey, verrucose thallus and apothecia with rusty-yellow discs and margins with an outer thalline cover, based on a type from Sweden, with a few records from the Eastern Alps. – **Au**: St. **It**: TAA.

***Caloplaca turkuensis* (Vain.) Zahlbr.**

Syn.: *Caloplaca jemtlandica* H. Magn., *Placodium turkuense* Vain.

L – Subs.: cor – Alt.: 3 – Note: mainly on bark of broad-leaved trees and rarely of conifers, from the lowlands to the mountains in deciduous and mixed forests, or on wayside trees. – **Au**: V, S, K, St, N. **Sw**: SZ.

***Caloplaca variabilis* (Pers.) Th. Fr.**

Syn.: *Blastenia rhinodinoides* (J. Steiner) Szatala, *Callopisma variabile* (Pers.) Trevis., *Caloplaca alpestris* (Ach.) Ozenda & Clauzade, *Caloplaca fulva* (Anzi) J. Steiner, *Caloplaca intercedens* (Trevis.) J. Steiner, *Caloplaca paepalostoma* (Anzi) Jatta, *Caloplaca rhinodinoides* J. Steiner, Caloplaca variabilis (Pers.) Th. Fr. f. fulva (Anzi) Clauzade & Cl. Roux, Caloplaca variabilis (Pers.) Th. Fr. f. paepalostoma (Anzi) Clauzade & Cl. Roux, Caloplaca variabilis (Pers.) Th. Fr. var. ochracea Müll. Arg., *Lecanora variabilis* (Pers.) Ach., *Lichen variabilis* Pers., *Placodium fulvum* Anzi, *Placodium paepalostomum* Anzi, *Placodium variabile* (Pers.) Hepp, *Pyrenodesmia intercedens* Trevis., *Pyrenodesmia variabilis* (Pers.) A. Massal., *Rinodina articulata* Bagl., *Thelotrema intercedens* (Trevis.) Nyl.

L – Subs.: cal, int – Alt.: 1–5 – Note: a probably holarctic, subtropical to boreal-montane, very polymorphic lichen found on a wide variety of calciferous substrata wetted by rain; widespread throughout the Alps. – **Au**: V, T, S, K, St, O, N, B. **Ge**: OB, Schw. **Sw**: BE, GR, LU, SZ, TI, UW, VD, VS. **Fr**: AHP, HAl, AMa, Drô, Isè, Sav, HSav, Var, Vau. **It**: Frl, Ven, TAA, Lomb, Piem, Lig. **Sl**: SlA.

***Caloplaca viridirufa* (Ach.) Zahlbr.**

Syn.: *Caloplaca aractina* (Fr.) Häyrén, Caloplaca cerina (Hedw.) Th. Fr. var. aractina (Fr.) Th. Fr., Caloplaca fuscoatra *auct. non* (Decouillè) Zahlbr., *Lecidea viridirufa* Ach., *Parmelia aractina* Fr., Placodium fuscoatrum *auct. non* (Nyl.) A.L. Sm.

L – Subs.: sil – Alt.: 1–5 – Note: according to Vondrák (see Nimis 2016) *C. viridirufa* is not homogeneous, and includes several taxa with different ecology and distribution. – **Au**: T, St, N. **Sw**: VS. **Fr**: AMa, Sav, Var.

***Caloplaca vitellinaria* Szatala**

Syn.: Caloplaca holocarpa (Hoffm.) Wade var. vitellinaria (Szatala) Clauzade & Cl. Roux

L – Subs.: sil-par, cal-par, xyl-par – Alt.: 3–5 – Note: a rather rare, subcontinental species, perhaps belonging to the *C. pyracea-holocarpa*-group, without a visible thallus, but with agglomerated apothecia with orange-red discs and somewhat paler margins (instead of brown as in *C. grimmiae*); lichenicolous on *Candelariella vitellina* which itself can grow on a wide range of substrates; for the study area there are only a few records from the Eastern Alps (Austria). – **Au**: K, St.

***Caloplaca vitellinula* (Nyl.) H. Olivier**

Syn.: *Athallia vitellinula* (Nyl.) Arup, Frödén & Søchting, *Lecanora vitellinula* Nyl.

L # – Subs.: sil, int, cor – Alt.: 2–4 – Note: a species of the *C. pyracea-holocarpa*-group, with a thin yellowish thallus and apothecia of a yolk-yellow colour; the species is based on a corticolous type from northern Fennoscandia, but the name was also used for saxicolous populations of similar appearance, so that the distribution is very poorly known. – **Au**: T, K, St, O, N. **Fr**: AHP, HAl, AMa, Isè. **It**: Ven, TAA, VA.

***Caloplaca xantholyta* (Nyl.) Jatta**

Syn.: *Lecanora xantholyta* Nyl., *Lepraria xantholyta* (Nyl.) Lettau, *Leproplaca xantholyta* (Nyl.) Hue, *Placodium xantholytum* (Nyl.) Nyl.

L – Subs.: cal, sil, ter-cal – Alt.: 1–4 – Note: a mild-temperate lichen of steeply inclined to underhanging surfaces of limestone and other calcareous rocks in humid, often shaded situations. – **Au**: V, T, S, K, St, O, N. **Ge**: OB, Schw. **Sw**: SZ, VD, VS. **Fr**: AHP, HAl, AMa, Drô, Sav, HSav, Var, Vau. **It**: Frl, Ven, TAA, Lomb, Piem.

***Caloplaca xerica* Poelt & Vězda**

L – Subs.: sil, int – Alt.: 2–3 – Note: on weathered surfaces of basic siliceous rocks, restricted to dry-continental valleys of the Alps and perhaps more widespread in Eurasia; the variety *venostana* Poelt differs in the blackish apothecial discs. – **Au**: T. **Sw**: VS. **It**: TAA, Piem, VA.

***Calvitimela aglaea* (Sommerf.) Hafellner**

Syn.: *Lecidea aglaea* Sommerf., *Lecidea brunneri* Nyl., *Lecidea crombei* Nyl., *Lecidea relanderi* Räsänen, *Lecidella aglaea* (Sommerf.) Körb., *Oedemocarpus aglaeus* (Sommerf.) Trevis., *Tephromela aglaea* (Sommerf.) Hertel & Rambold

L – Subs.: sil – Alt.: 3–6 – Note: an arctic-alpine, circumpolar species found on inclined faces of hard siliceous rocks in upland areas. – **Au**: V, T, S, K, St, N. **Sw**: BE, GR, UR, VS. **Fr**: Sav, HSav. **It**: Frl, TAA, Lomb, Piem, VA.

***Calvitimela armeniaca* (DC.) Hafellner**

Syn.: *Lecidea armeniaca* (DC.) Fr., *Lecidea nigrita* Schaer., *Lecidea viridiatra* Ach., *Lecidella armeniaca* (DC.) Bagl., *Oedemocarpus armeniacus* (DC.) Trevis., *Psora armeniaca* (DC.) A. Massal., *Rhizocarpon armeniacum* DC., *Tephromela armeniaca* (DC.) Hertel & Rambold

L – Subs.: sil – Alt.: 3–6 – Note: an arctic-alpine, circumpolar species found on hard siliceous rocks in wind-exposed situations; when young, it is a facultative parasite of *Sporastatia testudinea*; widespread throughout the siliceous Alps. – **Au**: V, T, S, K, St, N. **Sw**: BE, GR, TI, UR, UW, VS. **Fr**: AHP, HAl, AMa, Isè, Sav, HSav. **It**: Frl, Ven, TAA, Lomb, Piem, VA, Lig.

***Candelaria concolor* (Dicks.) Stein**

Syn.: *Blasteniospora concolor* (Dicks.) Trevis., *Caloplaca concolor* (Dicks.) Jatta, *Caloplaca laciniosa* (Nyl.) H. Olivier, *Candelaria laciniosa* (Nyl.) Kieff., *Candelaria vulgaris* A. Massal., *Lecanora concolor* (Dicks.) Lamy *nom.illeg.*, *Lecanora laciniosa* Nyl., *Lichen concolor* Dicks., *Physcia concolor* (Dicks.) Bagl. & Carestia, *Xanthoria concolor* (Dicks.) Th. Fr.

L – Subs.: cor, xyl – Alt.: 1–3 – Note: a mild-temperate, probably holarctic species found on bark, more rarely on calciferous substrata, mostly on isolated trees in agricultural areas, on wayside trees, etc.; widespread throughout the Alps. – **Au**: V, T, S, K, St, O, N, B. **Ge**: OB, Schw. **Sw**: BE, FR, GL, GR, LU, SG, SZ, TI, UR, UW, VD, VS. **Fr**: AHP, HAl, AMa, Drô, Isè, Sav, HSav, Var, Vau. **It**: Frl, Ven, TAA, Lomb, Piem, VA, Lig. **Sl**: SlA, Tg. **Li**.

***Candelaria coudercii* Harm.**

L # – Subs.: cor – Alt.: 4 – Note: a species with a thallus consisting of more or less disciform, entire squamules, some becoming microlobulate, but not larger than 1 mm in diam., most probably a species of *Candelariella*; only known from the type locality in the Western Alps, growing on the bark of *Pinus*. – **Fr**: HSav.

***Candelaria pacifica* M. Westb. & Arup**

L – Subs.: cor – Alt.: 3–4 – Note: a species with minute lobes lacking a lower cortex and rhizines; on eutrophicated bark; the extra-Alpine spreading is apparently recent, in the Alps there are so far only a few records, but perhaps the species was not distinguished from *C. concolor* in the past. – **Au**: O. **Sw**: GR, VS. **It**: Frl, Ven, Lomb.

***Candelariella aggregata* M. Westb.**

L – Subs.: cal, ter – Alt.: 2–4 – Note: a species resembling *C. aurella*, but with a granular thallus and usually aggregated apothecia lacking a hypothecial stipe of elongated strongly gelatinised cells, based on a type from a high elevation locality in Western North America, on plant debris and cushions of bryophytes; so far there are only a few records from the Alps, but perhaps the species was not distinguished from *C. aurella* in the past. – **Sw**: GR, VS. **Fr**: AHP, Sav.

***Candelariella antennaria* Räsänen**

L – Subs.: cor, xyl – Alt.: 2–3 – Note: a species resembling *C. aurella*, but with a grey thallus and apothecia lacking a hypothecial stipe of elongated strongly gelatinised cells, based on a type from Argentina, but also widespread in North America; usually corticolous on the bark of broad-leaved trees, but also on *Juniperus* and wood, with a few records from the Alps. – **Au**: T. **Sw**: GR, VS.

**Candelariella aurella (Hoffm.) Zahlbr. subsp. aurella**

Syn.: *Caloplaca epixantha* (Ach.) Flagey, *Caloplaca subsimilis* (Th. Fr.) Th. Fr., *Candelariella cerinella* (Flörke) Zahlbr., *Candelariella dispersa* (Räsänen) Hakul., *Candelariella epixantha* (Nyl.) Sandst., *Candelariella heidelbergensis* (Nyl.) Poelt, *Candelariella litoralis* Hakul., Candelariella vitellina (Hoffm.) Müll. Arg. var. aurella (Hoffm.) A.L. Sm., *Gyalolechia aurella* (Hoffm.) Körb., *Lecanora epixantha* (Ach.) Nyl., *Lecanora heidelbergensis* Nyl., Lecanora vitellina (Hoffm.) Ach. var. aurella (Hoffm.) Ach., *Lecidea epixantha* Ach., Parmelia murorum (Hoffm.) Ach. var. aurella (Hoffm.) Ach., *Verrucaria aurella* Hoffm.

L – Subs.: cal, int, bry, deb, xyl – Alt.: 1–6 – Note: a holarctic, subtropical to arctic-alpine, almost cosmopolitan species found on a wide variety of calciferous substrata, from limestone and dolomite to mortar, asbestos-cement and concrete, exceptionally on eutrophicated and dusty lignum and bark, sometimes starting the life-cycle on other crustose lichens; widespread and common throughout the Alps. – **Au**: V, T, S, K, St, O, N, B. **Ge**: OB, Schw. **Sw**: BE, FR, GR, LU, SZ, TI, UR, UW, VD, VS. **Fr**: AHP, HAl, AMa, Drô, Isè, Sav, HSav, Var, Vau. **It**: Frl, Ven, TAA, Lomb, Piem, VA, Lig. **Sl**: SlA. **Li**.

**Candelariella aurella (Hoffm.) Zahlbr. subsp. glebulosa (Asta, Clauzade & Cl. Roux) Cl. Roux *comb. inval.***

Syn.: Candelariella oleaginescens Rondon var. glebulosa Asta, Clauzade & Cl. Roux [invalidly published, ICN Art. 40.1 + 8]

L – Subs.: cal, int – Alt.: 3–5 – Note: a taxon with a glebulose, grey thallus, mostly growing on large, weakly to strongly calciferous boulders in moderately to strongly eutrophicated situations, restricted to high elevations, with a few records from the Alps. – **Au**: ?V, ?T. **Fr**: AHP, AMa, Drô, Sav.

***Candelariella boleana* Etayo, Palice & T. Sprib.**

L – Subs.: cor – Alt.: 2–3 – Note: a species with a granulose yellow thallus, lecanorine apothecia, and asci containing 16–32 spherical spores; on bark of various trees, widespread in Southern and Central Europe, but rare, with a single record from the Alps. – **Sw**: UW.

***Candelariella carnica* Poelt**

L # – Subs.: ter-cal – Alt.: 5 – Note: a species with a squamulose, yellow thallus, only known in the sterile state; on decalcified soil over limestone in the alpine belt; so far only known from the type locality in the Eastern Alps. – **Au**: K.

***Candelariella commutata* Otte & M. Westb.**

Syn.: *Candelariella aurella* (Hoffm.) Zahlbr. var. *unilocularis* auct. non. (Elenkin) Zahlbr.

L – Subs.: ter-cal, deb, cal, bry-cal – Alt.: 4–5 – Note: an arctic-alpine species, widespread in the mountains of the southern holarctic zone; it is found on epilithic mosses on limestone and dolomite, a characteristic element of calcareous mountains, with optimum above treeline. – **Au**: V, T, S, K, St, O. **Ge**: OB, Schw. **Sw**: BE, FR, GR, VD, VS. **It**: Frl, Ven, TAA, Lomb, Piem.

***Candelariella coralliza* (Nyl.) H. Magn.**

Syn.: *Candelariella pulvinata* (Malbr.) Zahlbr., Candelariella vitellina (Hoffm.) Müll. Arg. var. pulvinata (Malbr.) Mereschk., *Lecanora coralliza* Nyl., Lecanora vitellina (Hoffm.) Ach. var. pulvinata Malbr.

L – Subs.: sil, xyl – Alt.: 2–5 – Note: a mainly boreal-montane to arctic-alpine, circumpolar species found on siliceous rocks, more rarely on lignum or even dust-covered bark in open habitats, most frequent in alpine to subalpine pastures, on isolated boulders used as birds’ perches; certainly widespread throughout the Alps, but overlooked, or subsumed under *C. vitellina* by several authors. – **Au**: V, T, S, K, St, O, N. **Ge**: OB. **Sw**: LU, UR, VS. **Fr**: AHP, AMa, Isè, Sav, HSav. **It**: Frl, TAA, Piem, VA. **Sl**: SlA.

***Candelariella efflorescens* R.C. Harris & W.R. Buck**

L # – Subs.: cor – Alt.: 2–4 – Note: a species with a finely granulose-sorediate thallus; European material is mostly sterile, and only fertile specimens (with 32-spored asci) can be assigned with certainty to this species; for similar specimens with 8-spored asci *C. xanthostigmoides* (Müll. Arg.) R.W. Rogers might be the correct name (see below); the species is also hard to distinguish from some morphs of *C. reflexa*; usually on eutrophicated bark of broad-leaved trees, widespread and common in the Alps at low elevations. – **Au**: V, T, S, K, St, O, N, B. **Ge**: OB. **Sw**: BE, GR, LU, VS. **It**: Frl, Ven, TAA, Lomb.

***Candelariella faginea* Nimis, Poelt & Puntillo**

L – Subs.: cor – Alt.: 3 – Note: a species with a thallus composed of minute roundish squamules, later forming granulose blastidia, and asci with a variable number of ascospores (8 to 32), found on bark of deciduous trees (*e.g. Fagus sylvatica*) in more or less closed forests; widespread but not common in montane forests of Mediterranean orobiomes, with a single record from the Western Alps (Italy). – **It**: Piem.

***Candelariella granuliformis* M. Westb.**

L – Subs.: ter – Alt.: 4–5 – Note: a rarely fertile species of the *C. vitellina*-group with a thallus composed of granules breaking down into minute blastidia, based on a type from Northern Canada; on soil or encrusting bryophytes or plant debris; the total distribution is arctic-alpine, and in the Alps it is so far known from a few localities. – **Sw**: GR, VS.

***Candelariella kuusamoensis* Räsänen**

L – Subs.: xyl, ter – Alt.: 3–4 – Note: a boreal-montane, poorly understood lichen found on the top of poles and wooden fences, on plant debris and soil, more rarely on rocks; certainly more widespread in the Alps. – **Au**: S, K, St, O. **Ge**: Ge. **It**: Frl, Lig. **Sl**: SlA.

***Candelariella lutella* (Vain.) Räsänen**

L – Syn.: Lecanora xanthostigma (Ach.) Röhl. var. lutella Vain.

Subs.: cor – Alt.: 2–4 – Note: a cool-temperate, perhaps holarctic lichen of smooth bark, especially of *Alnus*; regionally overlooked, or confused with similar species, but certainly not common in the Alps. – **Au**: V, T, K, St, N. **Sw**: GR. **Fr**: AHP, HAl, AMa, Drô, Isè, Var, Vau. **It**: Frl, Ven, TAA, Piem.

***Candelariella medians* (Nyl.) A.L. Sm.**

Syn.: *Caloplaca granulata* (Schaer.) Lindau, *Caloplaca medians* (Nyl.) Flagey, *Candelaria medians* (Nyl.) Flagey, *Candelariella granulata* (Schaer.) Zahlbr., *Gasparrinia medians* (Nyl.) Syd., *Lecanora granulata* (Schaer.) Vain., *Lecanora medians* (Nyl.) Nyl., Parmelia parietina (L.) Ach. var. granulata Schaer., *Placodium medians* Nyl., *Xanthoria medians* (Nyl.) Zwackh

L – Subs.: cal – Alt.: 1–3 – Note: a mild-temperate lichen found on man-made calcareous substrata (churches, other monuments, top of statues in parks and of gravestones), especially above the Mediterranean belt, but also on the top of isolated calcareous boulders in natural situations; most frequent in the Southern and Western Alps. – **Au**: N. **Fr**: AHP, AMa, Drô, HSav, Var, Vau. **It**: Frl, Ven, TAA, Lomb, Piem, Lig.

***Candelariella plumbea* Poelt & Vězda**

L – Subs.: cal, int – Alt.: 3–5 – Note: an arctic-alpine lichen found on dolomitic rocks wetted by rain in exposed habitats, often developing along small cracks; probably more widespread in the Alps. – **Au**: V, K, St, N. **It**: Frl, Ven, Piem, VA. **Sl**: SlA.

***Candelariella reflexa* (Nyl.) Lettau**

Syn.: *Caloplaca reflexa* (Nyl.) Flagey, *Lecanora reflexa* (Nyl.) Nyl., Lecanora vitellina (Hoffm.) Ach. var. reflexa Nyl.

L – Subs.: cor – Alt.: 1–3 – Note: a mild-temperate, holarctic species with a minutely squamulose thallus developing laminal soralia; specimens with poorly developed squamules and marginal soralia may belong to either *C. efflorescens* or *C. xanthostigmoidea*; on isolated trees, especially along waysides and in agricultural areas; widespread throughout the Alps. – **Au**: V, T, S, K, St, O, N, B. **Ge**: OB, Schw. **Sw**: BE, FR, GL, GR, LU, SG, SZ, TI, UR, UW, VD, VS. **Fr**: AHP, AMa, Isè, HSav, Var, Vau. **It**: Frl, Ven, TAA, Lomb, Piem, VA, Lig. **Sl**: SlA, Tg. **Li**.

***Candelariella subdeflexa* (Nyl.) Lettau**

Syn.: *Lecanora subdeflexa* Nyl.

L – Subs.: cor – Alt.: 2–3 – Note: a mild-temperate, perhaps holarctic lichen found on isolated trees, especially *Fraxinus*, *Populus* and *Juglans*, often near the base of the trunks; widespread in the Alps, but not common, and probably declining. – **Au**: S, K, St. **Ge**: OB. **Sw**: BE, GR, LU, TI, VS. **Fr**: AHP, AMa, Sav. **It**: TAA, Lomb, Piem, VA. **Sl**: SlA.

***Candelariella superdistans* (Nyl.) Malme**

Syn.: *Lecanora superdistans* Nyl.

L – Subs.: cor-par – Alt.: 3 – Note: a cool-temperate lichen parasitic on *Lecanora populicola*; very much overlooked in the past, but never common in the Alps. – **Sw**: ?GR. **It**: Frl.

***Candelariella unisepta* (Stizenb.) Zahlbr.**

Syn.: *Lecanora unisepta* Stizenb.

L # – Subs.: bry – Alt.: 4–5 – Note: a species of unclear generic placement, with a grey, granular to verrucose thallus, densely crowded, marginate apothecia with a yellow-brownish to blackish-brown discs (not reacting with K), unpigmented in longitudinal section apart from the brown epihymenium, 8-spored asci, and ellipsoid, 1-septate, hyaline ascospores (18–22 × 6–8 μm); encrusting bryophytes over calcareous rocks; only known from low-alpine localities in the Western Alps (Switzerland). – **Sw**: LU, UW.

***Candelariella viae-lacteae* G. Thor & V. Wirth**

L – Subs.: cor – Alt.: 2–4 – Note: a species with a granular, grey thallus and apothecia containing 8-spored asci; on bark of various trees including conifers; rather widespread in Europe, but apparently rare, with only a few records from the Alps. – **Au**: S. **Sw**: GR, VS.

***Candelariella vitellina* (Hoffm.) Müll. Arg.**

Syn.: *Callopisma vitellinum* (Hoffm.) Bagl., *Caloplaca vitellina* (Hoffm.) Th. Fr., *Candelaria vitellina* (Hoffm.) A. Massal., Candelariella vitellina (Hoffm.) Müll. Arg. var. corrusca (Ach.) Ozenda & Clauzade, *Gyalolechia vitellina* (Hoffm.) Anzi, *Lecanora vitellina* (Hoffm.) Ach., *Lichen vitellinus* Ehrh., *Verrucaria vitellina* (Hoffm.) Hoffm., *Xanthoria vitellina* (Hoffm.) Th. Fr., Zeora vitellina (Hoffm.) Flot. var. corruscans (Ach.) Flot.

L – Subs.: sil, int, bry, xyl, cor – Alt.: 1–6 – Note: a holarctic, almost cosmopolitan lichen with a wide ecological range, found on a wide variety of siliceous rocks, on roofing tiles, brick, and sometimes bryophytes, lignum and acid bark; widespread and common throughout the Alps. – **Au**: V, T, S, K, St, O, N, B. **Ge**: OB, Schw. **Sw**: BE, GR, LU, SZ, TI, UR, VD, VS. **Fr**: AHP, HAl, AMa, Drô, Isè, Sav, HSav, Var, Vau. **It**: Frl, Ven, TAA, Lomb, Piem, VA, Lig. **Sl**: SlA, Tg. **Li**.

**Candelariella vitellina (Hoffm.) Müll. Arg. f. flavovirella (Nyl.) Alb. Hend.**

Syn.: *Candelariella flavovirella* (Nyl.) Lettau, *Lecanora flavovirella* Nyl.

L # – Subs.: xyl – Alt.: 3 – Note: a relatively rare form of *C. vitellina* (with moderately polyspored asci), characterised by a granulose, yellow-green thallus, whose unusual colour is due to the total or partial lack of calycin; on various substrates, including rocks (type on sandstone) and eutrophicated wood; here and there throughout Europe at low elevations, with a few records from the Eastern Alps (Austria). – **Au**: St.

***Candelariella xanthostigma* (Ach.) Lettau**

Syn.: Callopisma vitellinum (Hoffm.) Mudd var. xanthostigmum (Ach.) Bagl., *Caloplaca xanthostigma* (Ach.) H. Olivier, *Candelaria xanthostigma* (Ach.) Kieff., Candelariella vitellina (Hoffm.) Müll. Arg. var. xanthostigma (Ach.) Elenkin, Lecanora citrina (Hoffm.) Ach. var. xanthostigma Ach., Lecanora vitellina (Hoffm.) Ach. var. xanthostigma (Ach.) Nyl.

L – Subs.: cor, xyl – Alt.: 1–4 – Note: a mild – to cool-temperate, perhaps holarctic species found on bark of more or less isolated trees, especially oaks, but also on conifers, much more rarely on lignum; widespread throughout the Alps. – **Au**: V, T, S, K, St, O, N, B. **Ge**: OB, Schw. **Sw**: BE, FR, GL, GR, LU, SG, SZ, TI, UR, UW, VD, VS. **Fr**: AHP, HAl, AMa, Drô, Isè, Sav, HSav, Var, Vau. **It**: Frl, Ven, TAA, Lomb, Piem, VA, Lig. **Sl**: SlA, Tg. **Li**.

***Candelariella xanthostigmoides* (Müll. Arg.) R.W. Rogers**

Syn.: *Lecanora xanthostigmoides* Müll. Arg.

L – Subs.: cor – Alt.: 2–3 – Note: a species with a finely granulose thallus (as in *C. efflorescens*), but with 8-spored asci, based on a type from Australia (identity of European samples therefore in need of verification); sterile specimens are usually named *C. efflorescens*. – **Sw**: SZ.

***Carbonea assimilis* (Hampe *ex* Körb.) Hafellner & Hertel**

Syn.: *Lecidea assimilis* (Hampe *ex* Körb.) Th. Fr., *Lecidella assimilis* Hampe *ex* Körb., *Psora assimilis* (Hampe *ex* Körb.) Zahlbr.

L – Subs.: sil-par – Alt.: 3–5 – Note: a species with a thallus of minute pale brown glossy areoles which are diagnostic, and *Carbonea*-apothecia; on exposed, inclined faces of siliceous rocks, parasitic on other crustose lichens (*e.g.* species of *Aspicilia*, *Lecanora*, *Lecidea*, *Tephromela*); widespread but not common, perhaps regionally still overlooked. – **Au**: S, K, St. **Sw**: UR.

***Carbonea atronivea* (Arnold) Hertel**

Syn.: *Lecidea atronivea* Arnold

L – Subs.: cal, sil, int – Alt.: 4–6 – Note: an arctic-alpine, circumpolar species found on lime-containing siliceous rocks (*e.g.* calciferous schists), starting the life-cycle on *Lecidella* species; certainly overlooked and more widespread in the Alps. – **Au**: V, T, S, K, St. **Ge**: OB, Schw. **Sw**: BE, GR, VS. **Fr**: AHP, HAl, AMa, Isè, HSav. **It**: Ven, TAA, Piem, VA.

***Carbonea distans* (Kremp.) Hafellner & Obermayer**

Syn.: *Biatora mosigiicola* Eitner, *Lecanora mosigiicola* (Eitner) Hertel & Rambold, *Lecidea distans* Kremp., *Lecidea mosigiicola* (Eitner) Zahlbr., *Lecidea straminea* Anzi, *Lecidella distans* (Kremp.) Körb.

L – Subs.: sil-par – Alt.: 4–6 – Note: an arctic-alpine obligate parasite of *Orphniospora mosigii*, found on steeply inclined, exposed faces of hard siliceous rocks in upland areas. – **Au**: V, T, S, K, St. **Ge**: Schw. **Sw**: GR, TI, UR, VS. **Fr**: AHP, HAl, AMa, Sav, HSav. **It**: Frl, TAA, Lomb, Piem.

***Carbonea latypizodes* (Nyl.) Knoph & Rambold**

Syn.: *Lecidea lacteola* Nyl., Lecidea latypea Ach. var. latypizodes (Nyl.) H. Olivier, *Lecidea latypizodes* Nyl., *Lecidella lacteola* (Nyl.) Hertel & Leuckert

L – Subs.: sil – Alt.: 2–4 – Note: an early coloniser of small pebbles in dusty situations, especially near the ground, with a wide altitudinal range and scattered records from the Alps. – **Au**: St. **It**: TAA, Lomb, VA.

***Carbonea nivaria* (Arnold) Rambold**

Syn.: *Lecidea nivaria* (Arnold) Dalla Torre & Sarnth., *Lecidella nivaria* Arnold

L – Subs.: sil – Alt.: 4–6 – Note: this silicicolous species seems to prefer climatically rough ridges at high altitudes; in the study area it is only known from the Austrian Alps. – **Au**: T.

***Carbonea viriduloatra* (B. de Lesd.) Hafellner**

Syn.: *Lecidea viriduloatra* B. de Lesd.

L – Subs.: sil – Alt.: 4 – Note: a species with a thin, green-black, areolate thallus composed of minute, plane areoles and small black apothecia which are concave at first. later plane and with persistent margins, in section with a brown-violet hypothecium and an emerald-green epihymenium, ascospores small and broadly ellipsoid; based on a type from the Pyrenees, on granite at high elevation, with a single record from the Eastern Alps (Austria), which needs confirmation. – **Au**: S.

***Carbonea vorticosa* (Flörke) Hertel**

L – Syn.: *Lecidea asperella* Stirt., *Lecidea guettingeri* Müll. Arg., *Lecidea kuendigiana* Müll. Arg., *Lecidea pullulans* Th. Fr., Lecidea sabuletorum (Schreb.) Ach. var. vorticosa Flörke, *Lecidea sublatypea* Leight. *ex* Cromb., *Lecidea vorticosa* (Flörke) Körb.

Subs.: sil, int – Alt.: 3–6 – Note: an arctic-alpine, circumpolar species found on steeply inclined faces of lime-poor sandstone, schists and gneiss, rarely on dolomite, in upland areas; widespread throughout the Alps. – **Au**: V, T, S, K, St, O, N. **Ge**: OB. **Sw**: BE, GR, LU, SZ, TI, UR, VS. **Fr**: AHP, HAl, AMa, Sav, HSav. **It**: Frl, Ven, TAA, Lomb, Piem, VA.

***Carbonicola anthracophila* (Nyl.) Bendiksby & Timdal**

Syn.: *Biatora anthracophila* (Nyl.) Hafellner, *Hypocenomyce anthracophila* (Nyl.) P. James & Gotth. Schneid., *Lecidea anthracophila* Nyl., *Lecidea cladonioides* Th. Fr., Lecidea cladonioides Th. Fr. var. albocervina (Räsänen) Zahlbr., *Psora cladonioides* (Th. Fr.) Elenkin, Psora cladonioides (Th. Fr.) Elenkin var. albocervina Räsänen

L – Subs.: xyl – Alt.: 3–4 – Note: a circumboreal-montane lichen found on charred wood, mostly in upland areas; rare in the Alps, with a few scattered records. – **Au**: T, St. **It**: Piem.

***Carbonicola myrmecina* (Ach.) Bendiksby & Timdal**

Syn.: *Hypocenomyce castaneocinerea* (Räsänen) Timdal, *Lecidea myrmecina* (Ach.) Fr., Lecidea scalaris (Ach.) Ach. var. myrmecina Ach., Psora cladonioides (Th. Fr.) Elenkin var. castaneocinerea Räsänen, *Psora myrmecina* (Ach.) Boistel, Psora ostreata Hofm. var. myrmecina (Ach.) Th. Fr.

L – Subs.: cor, xyl – Alt.: 2–4 – Note: a species of the former *Hypocenomyce anthracophila*-group, with a thallus consisting of ascending, usually proliferating squamules reacting Pd-, the concolorous margins being sorediate, and with compound, dark-brown apothecia (but rarely fertile); lignicolous, usually on charred wood; rather common in the boreal zone, but rare in the Alps, having so far been reported only from the Western Alps (France). – **Fr**: Var.

***Catapyrenium cinereum* (Pers.) Körb.**

Syn.: *Dermatocarpon cinereum* (Pers.) Th. Fr., *Dermatocarpon hepaticum* (Ach.) Th. Fr. *non auct.*, *Dermatocarpon tephroides* (Ach.) W. Mann, *Endocarpon cinereum* Pers., *Endocarpon hepaticum* Ach. *non auct.*, *Endopyrenium cinereum* (Pers.) Oxner, *Involucrocarpon cinereum* (Pers.) Servít, *Lichen tephroides* Ach., *Sagedia cinerea* (Pers.) Fr., *Verrucaria polythecia* Ach., *Verrucaria tephroides* (Ach.) Wallr.

L – Subs.: ter-cal – Alt.: 3–6 – Note: a boreal-montane to arctic-alpine, circumpolar species occurring also in more southern mountains on siliceous, base-rich soil with mica, or amongst terricolous bryophytes, usually near or above treeline; widespread throughout the Alps. – **Au**: V, T, S, K, St, O, N. **Ge**: OB, Schw. **Sw**: BE, GR, LU, SZ, TI, UR, UW, VD, VS. **Fr**: AHP, HAl, AMa, Isè, Sav, HSav, Vau. **It**: Frl, Ven, TAA, Lomb, Piem, VA, Lig. **Sl**: SlA, Tg.

***Catapyrenium daedaleum* (Kremp.) Stein**

Syn.: *Dermatocarpon cartilagineum* (Nyl.) Zahlbr., *Dermatocarpon daedaleum* (Kremp.) Th. Fr., *Endocarpon daedaleum* Kremp., *Endopyrenium daedaleum* (Kremp.) Körb., *Placidiopsis daedalea* (Kremp.) Creveld, *Placidium daedaleum* (Kremp.) Kremp., *Placocarpus daedaleus* (Kremp.) Trevis., *Verrucaria daedalea* (Kremp.) Nyl.

L – Subs.: ter-cal, deb, bry – Alt.: 3–5 – Note: a boreal-montane to arctic-alpine, circumpolar species found on plant debris, mosses and bare, humus-rich soil on calciferous ground near or above treeline; widespread throughout the Alps. – **Au**: V, T, S, K, St, O, N. **Ge**: OB. **Sw**: GR, SZ, VD, VS. **Fr**: AHP, HAl, AMa, Sav, HSav. **It**: Frl, Ven, TAA, Lomb, Piem, VA. **Sl**: SlA.

***Catapyrenium psoromoides* (Borrer) R. Sant.**

Syn.: Dermatocarpon daedaleum (Kremp.) Th. Fr. var. corticola H. Magn., *Dermatocarpon psoromoides* (Borrer) Dalla Torre & Sarnth., *Endocarpon psoromia* (Nyl.) Boistel, *Endocarpon psoromoides* (Borrer) Hook., *Guignardia psoromoides* (Borrer) Keissl., *Laestadia psoromoides* (Borrer) Vouaux, Placidium cartilagineum (Nyl.) Arnold var. muscicolum Arnold, *Placocarpus psoromoides* (Borrer) Trevis., *Verrucaria psoromia* Nyl., *Verrucaria psoromoides* Borrer

L – Subs.: cor – Alt.: 1–2 – Note: a mild-temperate, probably holarctic lichen found on the base of old trees, especially on rough bark in parklands and open woodlands, occasionally on epiphytic bryophytes, very rarely on epilithic mosses; apparently more frequent in the Western and Southern Alps. – **Ge**: Ge. **Fr**: AHP, AMa, Drô, Var, Vau. **It**: Frl, TAA, Lomb.

***Catillaria atomarioides* (Müll. Arg.) H. Kilias**

Syn.: Catillaria lenticularis (Ach.) Th. Fr. var. atomarioides (Müll. Arg.) Erichsen, *Catillaria microcarpa* R. Sant., *Lecidea atomarioides* Müll. Arg.

L – Subs.: sil – Alt.: 2–4 – Note: an inconspicuous lichen found on steeply inclined surfaces of hard siliceous rocks in humid situations, mostly in upland areas; very much overlooked and certainly more widespread in the Alps. – **Au**: T, S, K, St. **Sw**: SZ, VS. **Fr**: AMa, HSav, Vau.

***Catillaria chalybeia* (Borrer) A. Massal.**

Syn.: *Biatora deplanatula* Müll. Arg., *Biatorina baliola* (Nyl.) Hellb., *Biatorina chalybeia* (Borrer) Mudd, Biatorina lenticularis (Ach.) Körb. var. chalybeia (Borrer) Anzi, Biatorina lenticularis (Ach.) Körb. var. chloropoliza (Nyl.) A.L. Sm., *Biatorina nubila* Norman, *Buellia chalybeia* (Borrer) Bagl., Catillaria chalybeia (Borrer) A. Massal. var. chloropoliza (Nyl.) H. Kilias, *Catillaria chloroscotina* (Nyl.) Arnold, *Catillaria doliocarpa* (Müll. Arg.) Arnold, Catillaria lenticularis (Ach.) Th. Fr. var. nubila (Norman) Arnold, Catillaria lenticularis (Ach.) Th. Fr. var. vulgaris (Körb.) Th. Fr., Catillaria nigroclavata (Nyl.) J. Steiner var. baliola (Nyl.) Zahlbr., *Lecidea baliola* Nyl., *Lecidea chalybeia* Borrer, *Lecidea deplanatula* (Müll. Arg.) Müll. Arg., *Lecidea spodoplaca* Nyl., *Microlecia chalybeia* (Borrer) M. Choisy, *Patellaria doliocarpa* Müll. Arg.

L – Subs.: sil, int – Alt.: 1–5 – Note: a holarctic, subtropical to arctic, facultatively lichenised species found on a wide range of siliceous substrata, including roofing tiles, brick, and even gypsum, in sheltered situations and on periodically inundated rocks, common both in natural and urban areas, especially on walls, and widespread throughout the Alps. – **Au**: V, T, S, K, St, O, N, B. **Ge**: OB, Schw. **Sw**: BE, GR, SZ, TI, VD, VS. **Fr**: AHP, HAl, AMa, Sav, HSav, Var, Vau. **It**: Frl, Ven, TAA, Lomb, Piem, VA, Lig. **Sl**: SlA.

***Catillaria cohabitans* (Jatta) Lettau**

Syn.: *Biatorina cohabitans* Jatta

L # – Subs.: cor – Alt.: 3 – Note: a species with a very thin, inconspicuous thallus, living together with (containing?) *Trentepohlia* algae, with very small, orange apothecia, and ellipsoid, 1-septate, hyaline ascospores (5–6 × 2 μm), perhaps a species of Coenogonium (Dimerella); on bark (*e.g. Fagus*); only recorded from the Southern Alps (Italy). – **It**: TAA, Lomb.

***Catillaria contristans* (Nyl.) Zahlbr.**

Syn.: *Bacidia dufourii* (Ach. *ex* Nyl.) Lettau, *Biatora hypocyanea* (Stirt.) Zahlbr., *Biatorina contristans* (Nyl.) Arnold, *Bilimbia dufourii* Ach. *ex* Nyl., *Catillaria dufourii* (Ach *ex* Nyl.) Vain., *Lecidea contristans* Nyl., *Lecidea dufourii* Ach. *ex* Nyl., *Lecidea hypocyanea* Stirt.

L – Subs.: ter-sil, bry-sil – Alt.: 3–5 – Note: on dead bryophytes (*Andreaea*, *Grimmia*) and soil rich in humus over acid siliceous rocks in upland areas; apparently rare in the Alps, but perhaps overlooked. – **Au**: T, S, K, St. **Sw**: VS. **It**: Lomb.

***Catillaria detractula* (Nyl.) H. Olivier**

Syn.: *Lecania detractula* (Nyl.) Arnold, *Lecanora detractula* Nyl.

L – Subs.: cal – Alt.: 2–5 – Note: a temperate species of calcareous rocks in open situations; ecology and distribution need further study. – **Au**: V, St, N. **Fr**: Var.

***Catillaria endodesmia* (Müll. Arg.) Zahlbr.**

Syn.: *Lecidea endodesmia* (Müll. Arg.) Stizenb., *Patellaria endodesmia* Müll. Arg.

L # – Subs.: cal – Alt.: 4 – Note: a calcicolous species of unclear relationship, resembling *C. lenticularis*, but with branched and anastomosing paraphyses; only known from the type locality. – **Sw**: VS.

***Catillaria erysiboides* (Nyl.) Th. Fr.**

Syn.: *Biatorina erysiboides* (Nyl.) Arnold, *Lecidea erysiboides* Nyl.

L – Subs.: xyl, cor – Alt.: 2–4 – Note: on hard lignum, *e.g.* on horizontal faces of old stumps; widespread but not common in the Alps. – **Au**: S, K, N. **Sw**: GR. **Fr**: Var. **It**: Frl, Ven, TAA.

***Catillaria haematophaea* (Anzi) Lettau**

Syn.: *Biatorina haematophaea* Anzi

L # – Subs.: sil – Alt.: 3 – Note: a species with a granulose-verrucose, olivaceous brown thallus, the verrucae arising singly from a bluish-black hypothallus, later coalescing into a more or less continuous crust, apothecia small, sessile, the disc plane, reddish black, the margin thick, epithecium brownish, hymenium of conglutinate paraphyses, I+ intensely and persistently blue, hypothecium pale, asci 8-spored, ascospores hyaline, not well-developed in the type material; on shaded granitic rocks; the taxonomic position of this species awaits clarification (see Nimis 1993: 206). – **It**: Lomb.

***Catillaria ignita* (Anzi) Zahlbr.**

Syn.: *Biatorina ignita* Anzi

L # – Subs.: sil – Alt.: 3 – Note: a species with an inconspicuous thallus, small, red to rusty-red, solitary to crowded and then angular apothecia with a flat disc and a prominent margin, and hyaline, 1-septate, narrowly oblong ascospores measuring *c.* 14.7 × 3–4 µm; only known from the type collection, on micaceous schists, this species could prove to be a *Caloplaca* *s.lat.* (see Nimis 1993: 206). – **It**: Lomb.

***Catillaria lenticularis* (Ach.) Th. Fr.**

Syn.: *Biatora lenticularis* (Ach.) Fr., *Biatorina heppii* A. Massal., *Biatorina lenticularis* (Ach.) Körb., Biatorina lenticularis (Ach.) Körb. var. erubescens Flot., *Biatorina lojkana* J. Lahm, *Biatorina pulicaris* A. Massal., *Catillaria dolosa auct.*, Catillaria lenticularis (Ach.) Th. Fr. var. erubescens (Flot.) Th. Fr., *Catillaria lojkana* (J. Lahm) Zahlbr., *Catillaria rhyparophaea* (Nyl.) Zahlbr., *Catillaria umbrinella* Zahlbr., *Lecania actaea* (Nyl.) B. de Lesd., *Lecanora actaea* Nyl., *Lecidea gagei* Hook., *Lecidea lenticularis* Ach., *Lecidea rhyparophaea* Nyl., *Lecidea umbrinella* Nyl. *nom.illeg.*, *Microlecia lenticularis* (Ach.) M. Choisy

L – Subs.: cal, int – Alt.: 1–6 – Note: a mainly mild-temperate lichen found on limestone, more rarely on dolomite, sometimes on nutrient-enriched, base-rich siliceous rocks, with optimum in open woodlands but present also inside conurbations, with a wide altitudinal range; widespread throughout the Alps. – **Au**: V, T, S, K, St, O, N. **Ge**: OB, Schw. **Sw**: BE, GR, LU, SZ, UR, UW, VD, VS. **Fr**: AHP, HAl, AMa, Drô, Isè, Sav, HSav, Var, Vau. **It**: Frl, Ven, TAA, Lomb, Piem, VA, Lig. **Sl**: SlA, Tg.

***Catillaria mediterranea* Hafellner**

Syn.: *Pleoscutula pleiospora* (Vouaux) Vouaux, *Scutula pleiospora* Vouaux

L – Subs.: fol-par, cor-par – Alt.: 2–3 – Note: a species of the *C. nigroclavata*-group with a simply organised, thin thallus, and 16-spored asci; on macrolichens (*e.g. Ramalina bourgeana*, *Anaptychia ciliaris*); widespread in Macaronesia and in the Mediterranean region, including the base of the Western Alps, where it is rare. – **Fr**: Var.

***Catillaria melanophaea* (Anzi) Lettau**

Syn.: *Biatorina melanophaea* Anzi, *Lecania melanophaea* (Anzi) Zahlbr.

L # – Subs.: sil – Alt.: 5 – Note: a species with a brownish, rather thick, rugulose, rimose-areolate thallus, small, adnate apothecia with a rather convex, dark brown, scabrid disc and a thin, finally disappearing margin which has the same colour as the thallus, a brownish epithecium, a pale hypothecium, coherent paraphyses, 8-spored asci and 1-septate, hyaline, straight to curved ascospores measuring 15.4–17.2 × c. 8.6 µm; the taxonomic position of this species, known only from the type collection on mica-schists, needs clarification. – **It**: Lomb.

***Catillaria minuta* (A. Massal.) Lettau**

Syn.: *Biatorina arnoldii* Kremp., Biatorina arnoldii Kremp. var. luteella (Nyl.) A.L. Sm., *Biatorina minuta* A. Massal., *Catillaria arnoldii* (Kremp.) Th. Fr.

L – Subs.: cal – Alt.: 1–5 – Note: a mainly mild-temperate species found on steeply inclined or underhanging faces of compact limestones in sheltered situations, *e.g.* in narrow gorges along creeks. – **Au**: V, T, S, K, St, O, N. **Ge**: OB, Schw. **Sw**: SZ. **Fr**: AMa, HSav, Vau. **It**: Frl, Ven, Lomb.

***Catillaria modesta* (Müll. Arg.) Coppins**

Syn.: *Lecidea modesta* Müll. Arg.

L # – Subs.: cal – Alt.: 4 – Note: a species with an unresolved taxonomy, growing on calcareous schists; in the Alps only known from the type locality, but also reported from Great Britain. – **Sw**: VD.

***Catillaria nigroclavata* (Nyl.) J. Steiner**

Syn.: *Biatorina nigroclavata* (Nyl.) Arnold, *Catillaria ilicis* (A. Massal.) A. Massal., *Lecidea ilicis* A. Massal., *Lecidea nigroclavata* Nyl., *Microlecia nigroclavata* (Nyl.) M. Choisy

L – Subs.: cor, xyl – Alt.: 1–3 – Note: a mainly mild-temperate, holarctic species found on twigs, very rarely on trunks of isolated deciduous trees, also in rather disturbed habitats, *e.g.* in parklands, and on wayside trees; widespread throughout the Alps. – **Au**: V, T, S, K, St, O, N. **Ge**: OB, Schw. **Sw**: BE, FR, GL, GR, LU, SG, SZ, TI, UR, UW, VS. **Fr**: AHP, AMa, Drô, Isè, Var, Vau. **It**: Frl, Ven, TAA, Lomb, Piem, VA, Lig. **Sl**: SlA, Tg. **Li**.

***Catillaria picila* (A. Massal.) Coppins**

Syn.: *Biatora picila* A. Massal., *Biatorina picila* (A. Massal.) Zahlbr., *Catillaria anomaloides auct. non* (A. Massal.) Lettau, *Lecidea anomaliza* Nyl., *Lecidea picila* (A. Massal.) Nyl.

L – Subs.: cal, int – Alt.: 2–5 – Note: a temperate species found on compact calcareous rocks, especially limestone but also on calciferous schists, often together with *C. minuta*. – **Au**: V, T, S, K, St, O, N. **Ge**: Ge. **Sw**: BE, SZ, VD. **It**: Ven, TAA, Lomb, Piem.

***Catillaria rugulosa* (Hepp) Lettau**

Syn.: *Biatora rugulosa* Hepp

L # – Subs.: cor – Alt.: 3 – Note: perhaps related to *Lecania* (-*cyrtella*-group); on bark of broad-leaved trees (*e.g. Fagus*); in the study area only known from the Western Alps, but without recent records. – **Fr**: HSav.

***Catillaria scotinodes* (Nyl.) Coppins**

Syn.: *Catillaria confusior* (Nyl.) Zahlbr., *Lecidea confusior* Nyl., *Lecidea scotinodes* Nyl.

L # – Subs.: int – Alt.: 5 – Note: a species growing on calciferous schists which perhaps belongs to *Toninia*, with ascomata as in the “*Catillaria athallina*-group” and mainly non-septate ascospores; rare, in the Alps so far known from a single locality only. – **Sw**: SZ.

***Catillaria stenocarpa* B. de Lesd.**

L # – Subs.: sil – Alt.: 1 – Note: a taxon reported from the base of the Western Alps near Spotorno (Italy) and from Spain, which, according to the original description, differs from *C. lenticularis* in the growth on siliceous substrata and in the narrower spores, measuring 8–8.5 × 0.5–2 µm (Roux et al. 2014: 282). – **It**: Lig.

***Catinaria atropurpurea* (Schaer.) Vězda & Poelt**

Syn.: *Biatora atropurpurea* (Schaer.) Hepp, *Biatorina atropurpurea* (Schaer.) A. Massal., *Catillaria adpressa* (Hepp) Schuler, *Catillaria atropurpurea* (Schaer.) Th. Fr., Lecidea sphaeroides (Dicks.) Röhl. var. atropurpurea Schaer.

L – Subs.: cor, xyl – Alt.: 1–3 – Note: a mild-temperate to subtropical lichen found on trunks of old broad-leaved trees, often on parts which are seldom wetted by rain, or on undersides of thick branches; widespread in the Alps, but generally not common. – **Au**: V, T, S, K, St, O, N. **Ge**: OB, Schw. **Sw**: BE, GR, LU, SZ, UW, VS. **Fr**: AHP, AMa, HSav, Var, Vau. **It**: Ven, TAA, Lomb. **Sl**: SlA.

***Catinaria neuschildii* (Körb.) P. James**

Syn.: *Biatorina neuschildii* Körb., *Biatorina subpulicaris* Anzi, Catillaria atropurpurea (Schaer.) Th. Fr. subsp. neuschildii (Körb.) Th. Fr., *Catillaria neuschildii* (Körb.) Th. Fr., *Catillaria subpulicaris* (Anzi) Lettau

L – Subs.: xyl, cor – Alt.: 2–3 – Note: a temperate lichen found on trunks of old, mostly broad-leaved trees, often on faces which are seldom wetted by rain, such as undersides of thick branches; on the whole a poorly known taxon related to *C. atropurpurea*, which requires further study. – **Au**: St. **It**: Frl, Lomb. **Sl**: SlA.

***Catolechia wahlenbergii* (Flot. *ex* Ach.) Körb.**

Syn.: *Buellia pulchella* (Schaer.) Tuck., *Buellia wahlenbergii* (Flot. *ex* Ach.) Sheard, *Catolechia galbula* (DC.) Anzi, *Catolechia pulchella* (Schaer.) A. Massal., *Lecidea galbula* (DC.) Nyl., *Lecidea pulchella* Schaer., *Lecidea wahlenbergii* Flot. *ex* Ach., *Psora galbula* DC., *Toninia galbula* (DC.) Boistel

L – Subs.: ter-sil, sil, deb – Alt.: 4–5 – Note: an arctic-alpine, probably circumpolar lichen found on acid soil rich in humus and over bryophytes in fissures of vertical to overhanging siliceous rocks in cold, perennially humid situations; widespread in the Alps, but only locally common. – **Au**: V, T, S, K, St, N. **Ge**: Schw. **Sw**: GR, TI, VD, VS. **Fr**: Isè, HSav. **It**: Frl, TAA, Lomb, Piem, VA.

**Cephalophysis leucospila (Anzi) H. Kilias & Scheid. var. leucospila**

Syn.: *Lecidea leucospila* Anzi, *Lecidea mashiginii* Lynge, *Lecidea subtumidula* Nyl., *Lecidea ultima* Th. Fr.

L – Subs.: cal, int – Alt.: 4–6 – Note: an arctic-alpine, circumpolar species found on limestone and dolomite in exposed, steeply inclined faces; widespread throughout the Alps. – **Au**: V, T, S, K, St, O. **Ge**: OB, Schw. **Sw**: BE, GR, VS. **Fr**: AHP, HAl, AMa, Isè, Sav, HSav, Vau. **It**: Frl, Ven, TAA, Lomb.

**Cephalophysis leucospila (Anzi) H. Kilias & Scheid. var. caelivicina (Poelt & Hertel) H. Kilias & Scheid.**

Syn.: Lecidea ultima Th. Fr. var. caelivicina Poelt & Hertel

L – Subs.: cal, int – Alt.: 5–6 – Note: differing from the typical variety in the distinctly epilithic thallus, this lichen is found on exposed cliffs of limestone and schists rich in calcium; rare and restricted to very high mountains, perhaps not always distinguished in the Alps. – **Au**: V, T, S, K, O. **Sw**: GR.

***Cetraria aculeata* (Schreb.) Fr.**

Syn.: Cetraria aculeata (Schreb.) Fr. var. campestris Schaer., Cetraria aculeata (Schreb.) Fr. var. sorediata Du Rietz, Cetraria aculeata (Schreb.) Fr. var. spadicea (Roth.) Ach., *Cetraria bohemica* Anders, *Cetraria tenuissima* (L.) Vain., Cetraria tenuissima (L.) Vain. var. campestris (Schaer.) Erichsen, *Coelocaulon aculeatum* (Schreb.) Link, *Coelocaulon bohemicum* (Anders) Clauzade & Cl. Roux *comb. inval.*, *Cornicularia aculeata* (Schreb.) Ach., Cornicularia aculeata (Schreb.) Ach. var. acanthella (Ach.) Ach., Cornicularia aculeata (Schreb.) Ach. var. campestris (Schreb.) Rabenh., Cornicularia aculeata (Schreb.) Ach. var. sorediata (Du Rietz) Du Rietz, *Cornicularia bohemica* (Anders) Zahlbr., *Cornicularia spadicea* (Roth.) Ach., *Cornicularia tenuissima* (L.) Zahlbr., *Lichen aculeatus* Schreb.

L – Subs.: ter-sil – Alt.: 2–4 – Note: on mainly siliceous, often sandy mineral soil in clearings of *Calluna*-heathlands, with optimum in wind-exposed situations; widespread throughout the Alps. – **Au**: T, S, K, St. **Ge**: OB, Schw. **Sw**: BE, GR, LU, TI, UR, VD, VS. **Fr**: AHP, HAl, AMa, Drô, Isè, Sav, HSav, Var, Vau. **It**: Frl, Ven, TAA, Lomb, Piem, VA, Lig.

***Cetraria ericetorum* Opiz**

Syn.: *Cetraria crispa* (Ach.) Nyl., Cetraria islandica (L.) Ach. var. crispa Ach., Cetraria islandica (L.) Ach. var. subtubulosa Fr. *ex* Nyl., Cetraria islandica (L.) Ach. var. tenuifolia (Retz.) Vain., *Cetraria subtubulosa* (Fr. *ex* Nyl.) Zopf, *Cetraria tenuifolia* (Retz.) R. Howe

L – Subs.: ter-sil, ter-cal – Alt.: 3–5 – Note: an arctic-alpine, circumpolar species, with optimum on wind-exposed ridges on siliceous substrata; widespread and common throughout the Alps. – **Au**: V, T, S, K, St, O, N. **Ge**: OB, Schw. **Sw**: BE, GR, SZ, TI, UR, UW, VD, VS. **Fr**: AHP, HAl, AMa, Isè, Sav, HSav. **It**: Frl, Ven, TAA, Lomb, Piem, VA. **Sl**: SlA.

**Cetraria islandica (L.) Ach. subsp. islandica**

Syn.: Cetraria islandica (L.) Ach. var. platyna (Ach.) Ach., *Lichen islandicus* L., *Physcia islandica* (L.) Michx.

L – Subs.: ter-sil, ter-cal, xyl, cor – Alt.: 2–6 – Note: an arctic-alpine to boreal-montane, circumpolar lichen found on mineral and organic soil, amongst thick moss carpets, exceptionally on bark or lignum near the ground, with optimum near treeline; common and widespread throughout the Alps. – **Au**: V, T, S, K, St, O, N, B. **Ge**: OB, Schw. **Sw**: BE, GR, LU, SG, SZ, TI, UR, VD, VS. **Fr**: AHP, HAl, AMa, Drô, Isè, Sav, HSav, Var, Vau. **It**: Frl, Ven, TAA, Lomb, Piem, VA, Lig. **Sl**: SlA, Tg. **Li**.

**Cetraria islandica (L.) Ach. subsp. crispiformis (Räsänen) Kärnefelt**

Syn.: Cetraria islandica (L.) Ach. var. crispiformis Räsänen

L – Subs.: ter-sil – Alt.: 4–5 – Note: a taxon with narrower lobes and few, very small, laminal pseudocyphellae, found in the understory of subalpine coniferous forests and alpine heaths; it has an arctic-alpine distribution and in Europe is common in Scandinavia and in the British Isles, while from continental Europe there are only a few records; rare in the Alps, but perhaps overlooked or not distinguished from the typical variety. – **Au**: T, S, St. **Sw**: VS.

***Cetraria muricata* (Ach.) Eckfeldt**

Syn.: Cetraria aculeata (Schreb.) Fr. f. hispida Cromb., Cetraria aculeata (Schreb.) Fr. var. alpina Schaer., *Cetraria stuppea* Zopf, Coelocaulon aculeatum (Schreb.) Link subsp. hispidum (Cromb.) D. Hawksw., *Coelocaulon muricatum* (Ach.) J.R. Laundon, Cornicularia aculeata (Schreb.) Ach. var. alpina (Schaer.) Rabenh., Cornicularia aculeata (Schreb.) Ach. var. muricata (Ach.) Ach., *Cornicularia muricata* (Ach.) Ach., Cornicularia tenuissima (L.) Zahlbr. var. alpina (Schaer.) Zahlbr., Cornicularia tenuissima (L.) Zahlbr. var. hispida (Cromb.) Keissl., *Cornicularia tenuissima* (L.) Zahlbr. var. muricata (Ach.) Dalla Torre & Sarnth., *Lichen muricatus* Ach.

L – Subs.: ter-sil, ter-cal – Alt.: 2–5 – Note: optimum on siliceous soil in wind-exposed siliceous ridges above treeline, but also found on decalcified soils on calcareous substrata; widespread throughout the Alps. – **Au**: V, T, S, K, St, N. **Ge**: OB, Schw. **Sw**: GR, SZ, UR, VS. **Fr**: AHP, HAl, AMa, Isè, Sav, HSav, Var, Vau. **It**: Frl, Ven, TAA, Lomb, Piem, VA, Lig. **Sl**: SlA.

***Cetraria obtusata* (Schaer.) van den Boom & Sipman**

Syn.: Cetraria aculeata (Schreb.) Fr. var. obtusata Schaer.

L – Subs.: ter-sil – Alt.: 4–6 – Note: ecologically similar to *C. ericetorum*, but much rarer, and perhaps more bound to dry-continental situations. – **Au**: V, T, K. **Sw**: BE, GR, SZ, TI, UR, VS. **Fr**: AHP, HAl. **It**: TAA, Lomb, VA.

***Cetraria sepincola* (Ehrh.) Ach.**

Syn.: *Cetraria scutata* (Wulfen) Poetsch *non auct.*, *Lichen sepincola* Ehrh., *Platysma sepincola* (Ehrh.) Hoffm., *Tuckermannopsis sepincola* (Ehrh.) Hale

L – Subs.: cor, xyl – Alt.: 3–4 – Note: a subarctic-subalpine, circumpolar species found on small twigs of shrubs and trees, especially *Betula*, *Alnus viridis*, *Rhododendron ferrugineum*, mostly near the ground, and in areas with siliceous substrata; widespread throughout the Alps, but only locally common. – **Au**: V, T, S, K, St, O, N. **Sw**: BE, GR, LU, VD, VS. **Fr**: HSav. **It**: Frl, Ven, TAA, Lomb, Piem, Lig. **Sl**: SlA.

***Cetrariella commixta* (Nyl.) A. Thell & Kärnefelt**

Syn.: *Cetraria commixta* (Nyl.) Th. Fr., *Melanelia commixta* (Nyl.) A. Thell, *Parmelia commixta* (Nyl.) Th. Fr., *Platysma commixtum* Nyl.

L – Subs.: sil – Alt.: 3–6 – Note: a circumpolar, arctic-alpine lichen found on hard siliceous rocks wetted by rain in upland areas; somehow more bound to cold-humid sites than the superficially similar *Melanelia hepatizon*; widespread in the Alps. – **Au**: V, T, S, K, St, N. **Sw**: BE, GR, UR, VS. **Fr**: HAl, AMa, Isè, Sav, HSav. **It**: TAA, Lomb, Piem, VA.

***Cetrariella delisei* (Bory *ex* Schaer.) Kärnefelt & A. Thell**

Syn.: *Cetraria delisei* (Bory *ex* Schaer.) Nyl., *Cetraria hiascens* (Fr.) Th. Fr., Cetraria islandica (L.) Ach. var. delisei Bory *ex* Schaer.

L – Subs.: ter-sil – Alt.: 5 – Note: an arctic-alpine species of acid soil in rather humid situations; very rare in the Alps, being only known from Austria. – **Au**: T, S, K.

***Cetrariella sorediella* (Lettau) V.J. Rico & A. Thell**

Syn.: Cetraria commixta (Nyl.) Th. Fr. f. sorediella Lettau, Melanelia commixta (Nyl.) A. Thell var. sorediella (Lettau) Hafellner & Türk, *Melanelia sorediella* (Lettau) V.J. Rico, van den Boom & Barrasa

L – Subs.: sil – Alt.: 3–5 – Note: a species resembling *C. commixta*, but only known in the sterile state, with soralia-like areas on the surface and along the margins, intermixed with often crowded, detachable isidia-like structures with a pycnidium enclosed in the apex; on siliceous rocks, mostly on boulders in streams; more common in the mountains of SW Europe, rare elsewhere; obviously rare in the Alps. – **Au**: T, S, K, N.

***Cetrelia cetrarioides* (Delise *ex* Duby) W.L. Culb. & C.F. Culb.**

Syn.: *Parmelia cetrarioides* (Delise *ex* Duby) Nyl., Parmelia perlata (Huds.) Ach. var. cetrarioides Delise *ex* Duby

L – Subs.: cor, sil, xyl – Alt.: 2–4 – Note: a species with the perlatolic acid syndrome plus traces of imbricaric acid, found on the bark of broad-leaved trees and on epiphytic mosses, more rarely on silicicolous mosses in humid, old, mostly montane forests. – **Au**: V, T, S, K, St, O, N, B. **Ge**: OB, Schw. **Sw**: SZ. **Fr**: HSav. **It**: Frl, Ven, TAA. **Sl**: SlA, Tg. **Li**.

***Cetrelia chicitae* (W.L. Culb.) W.L. Culb. & C.F. Culb.**

Syn.: *Cetraria chicitae* W.L. Culb.

L – Subs.: cor – Alt.: 3 – Note: a species with the alectoronic and α-collatolic acids syndrome, the rarest *Cetrelia* in the Eastern Alps. – **Au**: V, S, K, St, O, N. **It**: Frl. **Sl**: SlA.

***Cetrelia monachorum* (Zahlbr.) W.L. Culb. & C.F. Culb.**

Syn.: *Parmelia monachorum* Zahlbr.

L – Subs.: cor, bry-sil – Alt.: 3 – Note: a species with the imbricaric acid syndrome (major) and perlatolic acid (minor), found on the bark of broad-leaved trees, more rarely on silicicolous mosses in humid, old, mostly montane forests; probably the most common species of *Cetrelia* in the Alps; several records listed under *C. cetrarioides* could refer to this taxon. – **Au**: V, T, S, K, St, O, N, B. **Ge**: OB, Schw. **It**: Frl, TAA. **Sl**: SlA.

***Cetrelia olivetorum* (Nyl.) W.L. Culb. & C.F. Culb.**

Syn.: *Parmelia cetrarioides* (Delise *ex* Duby) Nyl. var. rubescens (Th. Fr.) Du Rietz, *Parmelia olivetorum* Nyl., *Parmelia rubescens* (Th. Fr.) Vain. *nom.illeg.*, *Pseudoparmelia aradensis* Gyeln.

L – Subs.: cor, bry-sil – Alt.: 2–3 – Note: a species with the olivetoric acid syndrome, found on bark of broad-leaved trees and on epiphytic, more rarely silicicolous mosses in humid, old forests, locally still abundant in montane *Abies*-*Fagus* forests, especially in the Eastern Alps. – **Au**: V, T, S, K, St, O, N, B. **Ge**: OB, Schw. **Sw**: BE, GL, GR, LU, SG, SZ, TI, UR, UW, VD, VS. **It**: Frl, Ven, TAA, Lomb, Piem, Lig. **Sl**: SlA, Tg.

***Chaenotheca brachypoda* (Ach.) Tibell**

Syn.: *Chaenotheca sulphurea* (Retz.) Middelb. & Mattsson, *Coniocybe brachypoda* Ach., *Coniocybe griseola* Ach., *Coniocybe sulphurea* (Retz.) Nyl.

L – Subs.: xyl, cor – Alt.: 2–4 – Note: on decorticated stumps of deciduous trees, more rarely on bark and siliceous rocks, in old humid forests, on faces slightly protected from rain. – **Au**: T, S, K, St. **Ge**: OB, Schw. **Sw**: BE, GR, SZ. **It**: TAA, Piem.

***Chaenotheca brunneola* (Ach.) Müll. Arg.**

Syn.: *Calicium brunneolum* Ach., *Calicium flexipes* Ach., Calicium melanophaeum Ach. var. brunneolum (Ach.) Schaer., *Cyphelium brunneolum* (Ach.) De Not., *Phacotium brunneolum* (Ach.) Trevis.

L – Subs.: xyl – Alt.: 2–4 – Note: on relatively soft-decomposed lignum of old coniferous stumps in humid woodlands, more rarely on wood of deciduous trees, very rarely corticolous; widespread throughout the Alps. – **Au**: V, T, S, K, St, O, N. **Ge**: OB, Schw. **Sw**: BE, GL, GR, LU, SZ, UW, VD, VS. **Fr**: AHP, AMa, Drô, HSav. **It**: Frl, Ven, TAA, Lomb, Piem, Lig. **Sl**: SlA.

***Chaenotheca chlorella* (Ach.) Müll. Arg.**

Syn.: *Calicium carthusiae* Harm., *Calicium chlorellum* Ach., *Chaenotheca carthusiae* (Harm.) Lettau, *Chaenotheca suzai* Nádv.

L – Subs.: cor, xyl – Alt.: 2–4 – Note: optimum on old oaks inside forests, in fissures of the bark, sometimes on decorticated trunks, also of conifers, especially on dry undersides and inside hollow trunks. – **Au**: S. **Sw**: BE, VS. **Fr**: HSav. **It**: Frl, Ven, TAA, Lomb, Lig.

***Chaenotheca chrysocephala* (Ach.) Th. Fr.**

Syn.: *Calicium chrysocephalum* Ach., Calicium chrysocephalum Ach. var. filare Ach., *Cyphelium chrysocephalum* (Ach.) Chevall., *Phacotium chrysocephalum* (Ach.) Trevis.

L – Subs.: cor, xyl – Alt.: 2–4 – Note: a boreal-montane, circumpolar species found on the acid bark of both broad-leaved trees and conifers, more rarely on hard lignum, with optimum on *Larix* near treeline; widespread throughout the Alps. – **Au**: V, T, S, K, St, O, N, B. **Ge**: OB, Schw. **Sw**: BE, GL, GR, LU, SG, SZ, TI, UR, UW, VD, VS. **Fr**: AHP, HAl, AMa, Drô, Isè, Sav, HSav, Vau. **It**: Frl, Ven, TAA, Lomb, Piem, VA, Lig. **Sl**: SlA.

***Chaenotheca cinerea* (Pers.) Tibell**

Syn.: *Calicium cinereum* Pers., *Calicium schaereri* De Not. *non* *sensu* Nádv., *Chaenotheca albida* (Körb.) Zahlbr., *Chaenotheca schaereri* (De Not.) Zahlbr.

L – Subs.: cor – Alt.: 2–3 – Note: a mild-temperate species found on the nutrient-rich bark of several trees (*e.g. Acer*, *Fraxinus*, *Populus*, *Ulmus*), in deep fissures of the bark seldom wetted by rain, with optimum at low elevations; recent records from the Alps are rare. – **Au**: St, N. **Sw**: BE. **It**: Ven, Lomb.

***Chaenotheca ferruginea* (Turner *ex* Sm.) Mig.**

Syn.: *Calicium ferrugineum* Turner *ex* Sm., *Calicium melanophaeum* Ach., Calicium roscidum (Ach.) Flörke var. pinastri Ach., *Chaenotheca melanophaea* (Ach.) Zwackh, *Cyphelium ferrugineum* (Turner *ex* Sm.) Ach., *Cyphelium melanophaeum* (Ach.) A. Massal., *Phacotium ferrugineum* (Turner *ex* Sm.) Gray, *Phacotium melanophaeum* (Ach.) Trevis.

L – Subs.: cor, xyl – Alt.: 2–4 – Note: a cool-temperate to boreal-montane, circumpolar species found on acidic bark, especially of very old oaks, *Castanea* and conifers, on faces protected from rain, sometimes on decorticated stumps and even charred wood; reported as tolerant of air pollution, and expanding in Northern Europe; widespread throughout the Alps. – **Au**: V, T, S, K, St, O, N, B. **Ge**: OB, Schw. **Sw**: BE, GL, GR, LU, SG, SZ, TI, UR, UW, VS. **Fr**: AHP, AMa, Isè, HSav, Vau. **It**: Frl, Ven, TAA, Lomb, Piem, Lig. **Sl**: SlA, Tg.

***Chaenotheca furfuracea* (L.) Tibell**

Syn.: *Calicium capitellatum* Ach., *Calicium furfuraceum* (L.) Pers., *Coniocybe furfuracea* (L.) Ach., *Mucor furfuraceus* L.

L – Subs.: cor, xyl, deb, sil – Alt.: 1–4 – Note: a holarctic lichen found beneath overhanging faces protected from rain, especially in forests, often on exposed roots, but rather indifferent to the substrata (also found on siliceous rocks and lignum); widespread throughout the Alps. – **Au**: V, T, S, K, St, O, N, B. **Ge**: OB, Schw. **Sw**: BE, GL, GR, LU, SG, SZ, TI, UR, UW, VS. **Fr**: AHP, HAl, AMa, Isè, Sav, HSav, Vau. **It**: Frl, Ven, TAA, Lomb, Piem, VA, Lig. **Sl**: SlA, Tg.

***Chaenotheca gracilenta* (Ach.) Mattsson & Middelb.**

Syn.: *Calicium gracilentum* Ach., *Coniocybe gracilenta* (Ach.) Ach., *Cybebe gracilenta* (Ach.) Tibell

L – Subs.: xyl, cor, ter – Alt.: 3–4 – Note: a circumboreal-montane species found on rotting wood and decaying bark on faces protected from rain, such as hollows of old stumps in ancient, humid forests; widespread throughout the Alps. – **Au**: V, T, S, K, St, O, N. **Ge**: OB. **Sw**: BE, GL, GR, SZ, UW, VD, VS. **Fr**: HAl, AMa, Isè, Vau. **It**: Frl, Ven, TAA, Lomb, Piem. **Sl**: SlA.

***Chaenotheca hispidula* (Ach.) Zahlbr.**

Syn.: *Calicium aciculare* (Gray) Fr., *Calicium hispidulum* (Ach.) Ach., Calicium trachelinum (Ach.) Ach. var. hispidulum Ach., *Chaenotheca acicularis* (Gray) Zwackh, Chaenotheca chlorella (Ach.) Müll. Arg. var. hispidula (Ach.) Vain., *Chaenotheca chlorelloides* (Anzi) Zahlbr., *Cyphelium aciculare* (Gray) Arnold, *Cyphelium chlorelloides* Anzi, *Phacotium aciculare* (Gray) Trevis., *Phacotium hispidulum* (Ach.) Trevis.

L – Subs.: cor – Alt.: 1–4 – Note: a cool-temperate, probably holarctic lichen found in dry hollows and undersides, and on the bases of ancient trees, especially oaks, in humid deciduous forests. – **Au**: V, T, S, St, O. **Ge**: OB, Schw. **Sw**: GL, GR, SZ, UW, VS. **Fr**: AHP. **It**: Ven, TAA, Lomb, Piem.

***Chaenotheca laevigata* Nádv.**

L – Subs.: cor – Alt.: 3 – Note: a cool-temperate to southern boreal-montane lichen found in bark fissures of acid-barked deciduous and coniferous trees in humid forests, more rarely on lignum; probably overlooked and more widespread, but certainly never common in the Alps. – **Au**: V, S, St, N. **Sw**: SZ. **Fr**: AMa. **It**: TAA.

***Chaenotheca phaeocephala* (Turner) Th. Fr.**

Syn.: *Calicium phaeocephalum* (Turner) Fr., *Calicium saepiculare* Ach., *Chaenotheca chlorella auct. non* (Ach.) Müll. Arg., *Cyphelium phaeocephalum* (Turner) Körb., *Lichen phaeocephalus* Turner

L – Subs.: cor, xyl – Alt.: 2–4 – Note: a cool-temperate, holarctic lichen found on old oaks in open woodlands, in bark fissures seldom wetted by rain. – **Au**: V, T, S, St, O, N. **Ge**: OB, Schw. **Sw**: BE, GL, GR, LU, SZ, UR, UW, VS. **Fr**: HAl, AMa, Vau. **It**: Ven, TAA.

***Chaenotheca servitii* Nádv.**

L – Subs.: xyl – Alt.: 3 – Note: a very rare species with a poorly developed endosubstratic or thin, smooth greenish thallus with *Stichococcus*, apothecia with long, slender stalks, in the upper part with a reddish to yellow pruina which covers also the outside of the capitulum, which is broadly obovoid to lenticular with a well developed exciple, asci cylindrical with rather small, spherical, mostly smooth, uniseriate ascospores (*c.* 4 µm in diam.); on lignum of deciduous trees, for the study area only recorded from the Eastern Alps (Austria). – **Au**: N.

***Chaenotheca sphaerocephala* Nádv.**

L – Subs.: cor – Alt.: 3–4 – Note: a species with a minutely granular, whitish thallus and apothecia with globose, epruinose capitula virtually lacking an exciple, based on a lignicolous type from Chile; ecology still poorly known; recently reported on bark from montane forests in the Central Alps. – **Sw**: LU, SZ.

***Chaenotheca stemonea* (Ach.) Müll. Arg.**

Syn.: *Calicium physarellum* Ach., *Calicium stemoneum* (Ach.) Ach., Calicium trichiale Ach. var. stemoneum Ach., *Chaenotheca aeruginosa auct. non* (Turner *ex* Sm.) A.L. Sm., *Cyphelium stemoneum* (Ach.) De Not., *Phacotium physarellum* (Ach.) Trevis.

L – Subs.: cor, xyl – Alt.: 2–4 – Note: a cool-temperate to boreal-montane, circumpolar lichen found in rain-protected hollows of conifer trunks inside forests, especially near the ground, both on bark and on lignum, sometimes on acid-barked deciduous trees, *e.g. Betula*, *Quercus*; widespread throughout the Alps. – **Au**: V, T, S, K, St, O, N, B. **Ge**: OB, Schw. **Sw**: BE, GL, GR, LU, SG, SZ, TI, UR, UW, VS. **Fr**: AMa, HSav. **It**: Frl, Ven, TAA, Lomb, Piem, Lig. **Sl**: Tg.

***Chaenotheca subroscida* (Eitner) Zahlbr.**

Syn.: *Cyphelium subroscidum* Eitner

L – Subs.: cor – Alt.: 3–4 – Note: a species with a minutely granular, grey thallus and apothecia with a greenish-yellow pruina on the exciple and the upper part of the stalks (closely related to *C. phaeocephala*); usually on bark of coniferous trees (*Picea*, *Abies*) in rather moist montane forests; widespread in the Alps, but rare. – **Au**: V, T, St, N. **Sw**: BE, SZ, UR, VD. **It**: Ven, TAA. **Sl**: SlA.

***Chaenotheca trichialis* (Ach.) Th. Fr.**

Syn.: *Calicium cinereum auct.*, *Calicium elassosporum* Nyl., *Calicium trichiale* Ach., *Chaenotheca aeruginosa* (Turner *ex* Sm.) A.L. Sm. *non auct.*, Chaenotheca brunneola (Ach.) Müll. Arg. var. elassospora (Nyl.) A.L. Sm., *Chaenotheca elassospora* (Nyl.) Zahlbr., *Cyphelium trichiale* (Ach.) De Not., *Phacotium trichiale* (Ach.) Trevis.

L – Subs.: cor, xyl – Alt.: 1–4 – Note: a holarctic species found on acid-barked deciduous trees, conifers and lignum, in forests and woodlands; widespread throughout the Alps. – **Au**: V, T, S, K, St, O, N. **Ge**: OB, Schw. **Sw**: BE, GL, GR, LU, SG, SZ, TI, UR, UW, VD, VS. **Fr**: AHP, AMa, Isè, HSav, Vau. **It**: Frl, Ven, TAA, Lomb, Piem. **Sl**: SlA.

***Chaenotheca xyloxena* Nádv.**

Syn.: *Chaenotheca nudiuscula* (Schaer.) Nádv.

L – Subs.: xyl – Alt.: 3–4 – Note: a cool-temperate to circumboreal-montane lichen found on hard and dry lignum, especially of conifers, in humid forests, more rarely on bark and on lignum of deciduous trees. – **Au**: V, T, S, K, St, O, N. **Ge**: OB, Schw. **Sw**: BE, LU, SZ, VS. **Fr**: AMa, Vau. **It**: Frl, Ven, TAA, Lomb. **Sl**: SlA.

***Cheiromycina flabelliformis* B. Sutton**

L – Subs.: cor, xyl – Alt.: 2–3 – Note: a species with a crustose, epiphloeodic thallus and cushion-shaped sporodochia in which multicellular conidia develop, branching three-dimensionally, originating from conspicuous globose cells; on bark of mostly broad-leaved trees in moist forests; widespread in the Holarctic region; from the Alps there are only a few records at low to mid-elevations, but elsewhere the species reaches the treeline ecotone. – **Au**: St, N. **Ge**: OB. **Sw**: SZ. **It**: TAA.

***Cheiromycina petri* D. Hawksw. & Poelt**

L – Subs.: cor – Alt.: 3 – Note: thallus and sporodochia as in *C. flabelliformis*, but basal cells of conidia not differentiated, branching of conidia two-dimensional, and branches shorter; on bark of trees; widespread in the Holarctic region, with a few records only from the Eastern Alps. – **Au**: St.

***Chrysothrix caesia* (Flot.) Ertz & Tehler**

Syn.: *Allarthonia caesia* (Flot.) Zahlbr., *Arthonia caesia* (Flot.) Körb., *Coniangium caesium* Flot.

L – Subs.: cor – Alt.: 2–3 – Note: a mild-temperate species also known from North America, found on the smooth bark of deciduous trees, especially *Carpinus*; often sterile and overlooked in the Alps, being easily confused with species of *Lepraria*. – **Au**: K, St. **It**: Frl. **Sl**: SlA, Tg.

***Chrysothrix candelaris* (L.) J.R. Laundon**

Syn.: *Bilimbia fulgens* Hampe *ex* A. Massal., *Byssus candelaris* L., *Crocynia flava* (Schreb.) Hue, *Lepraria candelaris* (L.) Fr., *Lepraria citrina auct. p.p.*, *Lepraria flava* (Schreb.) Sm.

L – Subs.: cor, xyl, sil – Alt.: 1–4 – Note: a cool-temperate to circumboreal-montane lichen found on dry, shaded parts of the trunks of deciduous and coniferous trees, on faces protected from rain, sometimes on lignum, with a wide altitudinal range; widespread and common throughout the Alps. – **Au**: V, T, S, K, St, O, N. **Ge**: OB, Schw. **Sw**: BE, GL, GR, LU, SG, SZ, TI, UR, UW, VS. **Fr**: AHP, AMa, Isè, Sav, HSav, Var, Vau. **It**: Frl, Ven, TAA, Lomb, Piem, VA, Lig. **Sl**: SlA.

***Chrysothrix chlorina* (Ach.) J.R. Laundon**

Syn.: *Calicium chlorinum* (Ach.) Schaer. *non auct. p.p.*, *Crocynia chlorina* (Ach.) Hue, *Lepra chlorina* (Ach.) DC., *Lepraria chlorina* (Ach.) Ach. *ex* Sm., *Lichen chlorinus* Ach., *Pulveraria chlorina* (Ach.) Ach.

L – Subs.: sil – Alt.: 1–5 – Note: a widespread lichen found in underhangs and crevices of siliceous rocks in shaded, humid situations; limited to areas with high air humidity, but with a wide altitudinal range. – **Au**: V, T, S, K, St, N, B. **Sw**: BE, GR, LU, SZ, TI, UR, VS. **Fr**: AMa, Isè, HSav, Var, Vau. **It**: Frl, Ven, Lomb, Piem, VA. **Sl**: SlA.

***Cladonia acuminata* (Ach.) Norrl.**

Syn.: Cenomyce pityrea (Flörke) Ach. f. acuminata Ach., Cladonia acuminata (Ach.) Norrl. subsp. foliata (Arnold) Vain., Cladonia acuminata (Ach.) Norrl. var. norrlinii (Vain.) Lynge, *Cladonia foliata* (Arnold) Kernst., *Cladonia norrlinii* Vain.

L – Subs.: bry, ter-cal – Alt.: 2–4 – Note: a cool-temperate to circumboreal-montane lichen found on calciferous soil rich in humus in open situations; widespread but not common in the Alps. – **Au**: T, K, O. **Ge**: Schw. **Sw**: BE, GR, SZ, TI, UW, VS. **It**: Frl, TAA, Lomb, Piem, VA.

***Cladonia amaurocraea* (Flörke) Schaer.**

Syn.: *Capitularia amaurocraea* Flörke, *Cladonia destricta* (Nyl.) Ohlert

L – Subs.: ter-sil, ter-cal, bry – Alt.: 3–5 – Note: a circumpolar, boreal-subarctic-subalpine lichen found on soil and bryophytes in open habitats, mostly in sites with a long snow cover; widespread throughout the Alps, but only locally common. – **Au**: , T, S, K, St, O, N. **Ge**: OB, Schw. **Sw**: BE, GR, SZ, UR, VS. **Fr**: Sav, HSav. **It**: Frl, Ven, TAA, Lomb, Piem, VA.

**Cladonia arbuscula (Wallr.) Flot. subsp. arbuscula**

Syn.: *Cladina arbuscula* (Wallr.) Hale & W.L. Culb., *Cladina sylvatica* (Ach.) Cromb., *Cladonia sylvatica* (Ach.) Rabenh., *Patellaria arbuscula* (Wallr.) Wallr., Patellaria foliacea Wallr. var. arbuscula Wallr.

L – Subs.: ter-cal, ter-sil, bry, xyl – Alt.: 2–6 – Note: a circumpolar, boreal-subarctic-subalpine lichen growing in lichen-rich tundra-like vegetation on mineral soil in exposed habitats; sometimes found on lignum. Several records, especially from Italy, could refer to subsp. squarrosa, which is more widespread in the Alps. – **Ge**: OB. **Sw**: BE, GR, LU, SZ, TI, UR, VS. **Fr**: HSav. **It**: Frl, Ven, TAA, Lomb, Piem, VA. **Sl**: SlA, Tg.

**Cladonia arbuscula (Wallr.) Flot. subsp. squarrosa (Wallr.) Ruoss**

Syn.: Cladina arbuscula (Wallr.) Hale & W.L. Culb. subsp. squarrosa (Wallr.) Burgaz, *Cladonia squarrosa* (Wallr.) Flot., Patellaria coccinea Wallr. var. squarrosa Wallr., *Patellaria squarrosa* (Wallr.) Wallr.

L – Subs.: ter-sil, ter-cal, bry, xyl, cor – Alt.: 2–5 – Note: this is the most widespread subspecies of the *C. arbuscula*-complex in the Alps; several records listed under C. arbuscula subsp. arbuscula could refer to this taxon. In the recent Nordic Lichen Flora, however, the psoromic acid strain is regarded as a taxonomically unimportant chemotype. – **Au**: V, T, S, K, St, O, N, B. **Ge**: OB, Schw. **Sw**: AP, BE, FR, GL, GR, LU, SG, SZ, TI, UR, UW, VD, VS. **Fr**: AHP, HAl, AMa, Drô, Isè, Sav, HSav, Vau. **It**: Frl, Ven, TAA, Piem. **Sl**: SlA.

***Cladonia bacilliformis* (Nyl.) Sarnth.**

Syn.: Cladonia carneola (Fr.) Fr. var. bacilliformis Nyl.

L – Subs.: xyl, ter – Alt.: 3–4 – Note: a species with a yellowish green squamulose primary thallus and unbranched, acuminate podetia with a sorediate surface, later sometimes with narrow scyphi, apothecia ochraceous but rarely developed; usually on rotten wood in forests dominated by conifers, but also on soil layers over siliceous rocks; not common in the Alps, mainly in the montane belt. – **Au**: T, S, K. **Sw**: GR, LU, SZ, VS. **Fr**: HSav. **It**: TAA.

***Cladonia bellidiflora* (Ach.) Schaer.**

Syn.: *Lichen bellidiflorus* Ach.

L – Subs.: ter-sil, deb, xyl – Alt.: 3–5 – Note: a cool-temperate to boreal-montane, circumpolar lichen found on acid soil and mossy rocks in wind-protected and humid situations (*e.g.* in sites with a long snow cover), most frequent near or above treeline; widespread throughout the Alps. – **Au**: V, T, S, K, St, O, N. **Ge**: OB, Schw. **Sw**: BE, GR, LU, SZ, TI, UR, UW, VD, VS. **Fr**: Sav, HSav. **It**: Frl, Ven, TAA, Lomb, Piem, VA, Lig. **Sl**: SlA.

***Cladonia borealis* S. Stenroos**

L – Subs.: bry, ter-sil, sil – Alt.: 2–4 – Note: on mineral siliceous soil in open habitats; related to *C. coccifera*, and with a similar ecology; certainly more widespread in the Alps. – **Au**: T, S, K, St, O. **Sw**: GR, SZ, VS. **Fr**: AHP, Sav. **It**: Frl, Ven, TAA, Lomb.

***Cladonia botrytes* (K.G. Hagen) Willd.**

Syn.: *Lichen botrytes* K.G. Hagen

L – Subs.: xyl, ter – Alt.: 3–4 – Note: a circumpolar, boreal-montane lichen found on decaying wood, mostly on horizontal faces of stumps and fallen trunks, especially of conifers, more rarely on decaying bark; widespread in the Alps, but generally not common. – **Au**: T, S, K, St, O, N, B. **Ge**: OB. **Sw**: GR, VD, VS. **It**: Frl, Ven, TAA, Lomb. **Sl**: SlA.

***Cladonia caespiticia* (Pers.) Flörke**

Syn.: *Baeomyces caespiticius* Pers., *Cladonia agariciformis* (Wulfen) Arnold

L – Subs.: ter-sil, xyl, cor, bry – Alt.: 2–5 – Note: a cool-temperate to southern boreal-montane, circumpolar lichen found on mineral, generally sandy-clay soil, occasionally on rotting wood and on bases of ancient trunks, in sheltered situations; widespread throughout the Alps. – **Au**: V, T, S, K, St, O, N, B. **Ge**: OB, Schw. **Sw**: BE, GR, LU, SZ, TI, UR, VS. **Fr**: AMa, Sav, HSav, Var, Vau. **It**: Ven, TAA, Lomb, Piem, VA, Lig. **Sl**: SlA.

***Cladonia cariosa* (Ach.) Spreng.**

Syn.: *Cladonia locarnensis* Frey *nom.illeg.*, *Cladonia pityrodes* Nyl., *Cladonia symphycarpodes* Nyl., *Lichen cariosus* Ach.

L – Subs.: ter-sil, ter-cal – Alt.: 2–5 – Note: a boreal-montane to subarctic-subalpine, circumpolar lichen found on disturbed mineral, often sandy soil over calcareous or base-rich substrata, with a wide altitudinal range; widespread throughout the Alps. – **Au**: V, T, S, K, St, O, N. **Ge**: OB, Schw. **Sw**: BE, GR, TI, UR, VD, VS. **Fr**: HAl, AMa, Isè, Sav, HSav. **It**: Ven, TAA, Lomb, Piem, Lig. **Sl**: SlA.

***Cladonia carneola* (Fr.) Fr.**

Syn.: *Cenomyce carneola* Fr., *Cladonia carneopallida* (Ach.) Nyl.

L – Subs.: cor, bry, xyl, ter – Alt.: 3–5 – Note: a circumpolar, mainly boreal-montane to subarctic lichen found on rotting wood and soil rich in humus in open montane to subalpine woodlands, sometimes reaching the alpine belt; widespread throughout the Alps. – **Au**: V, T, S, K, St, O, N. **Ge**: OB, Schw. **Sw**: BE, GR, LU, SZ, UR, VD, VS. **Fr**: AHP, HAl, Isè, HSav. **It**: Frl, Ven, TAA, Lomb, Piem, VA. **Sl**: SlA.

***Cladonia cenotea* (Ach.) Schaer.**

Syn.: *Baeomyces cenoteus* Ach., *Cladonia brachiata* (Fr.) Hampe, *Cladonia uncinata* Hoffm.

L – Subs.: xyl, cor, ter-sil – Alt.: 2–4 – Note: a temperate to boreal-montane, circumpolar species found on rotting wood, mainly on old stumps, and on soil rich in humus, with a wide altitudinal range; widespread and common throughout the Alps. – **Au**: V, T, S, K, St, O, N, B. **Ge**: OB, Schw. **Sw**: BE, GR, LU, SZ, TI, UR, UW, VD, VS. **Fr**: HAl, Isè, Sav, HSav, Var, Vau. **It**: Frl, Ven, TAA, Lomb, Piem, VA. **Sl**: SlA.

**Cladonia cervicornis (Ach.) Flot. subsp. cervicornis**

Syn.: Cladonia verticillata (Hoffm.) Schaer. var. cervicornis (Ach.) Flörke, *Lichen cervicornis* Ach.

L – Subs.: ter-sil – Alt.: 1–5 – Note: a temperate to southern boreal-montane lichen found on mineral siliceous soil in open grasslands and garrigues; several records from upland areas could refer to subsp. verticillata or even to *C. macrophyllodes*. – **Au**: V, T, S, K, St, N. **Sw**: BE, GR, TI, UR, VD, VS. **Fr**: HAl, AMa, Isè, Sav, HSav, Var, Vau. **It**: Ven, TAA, Lomb, Piem, VA, Lig.

**Cladonia cervicornis (Ach.) Flot. subsp. verticillata (Hoffm.) Ahti**

Syn.: Cladonia cervicornis (Ach.) Flot. var. verticillata (Hofffm.) Flot., Cladonia pyxidata (L.) Hoffm. var. verticillata Hoffm., *Cladonia verticillata* (Hoffm.) Schaer., Cladonia verticillata (Hoffm.) Schaer. var. evoluta (Th. Fr.) Stein

L – Subs.: ter-sil, sil – Alt.: 2–5 – Note: a boreal-montane to subarctic-subalpine, circumpolar lichen found on acid soil in open habitats; more frequent in upland areas than the typical subspecies. – **Au**: V, T, S, K, St, N, B. **Sw**: ?BE, ?GR, ?UR. **Fr**: HAl, AMa, Isè, Sav, HSav, Var, Vau. **It**: Ven, TAA, Lomb, Piem, VA. **Sl**: SlA.

***Cladonia chlorophaea* (Flörke *ex* Sommerf.) Spreng.**

Syn.: *Cenomyce chlorophaea* Flörke *ex* Sommerf., Cladonia pyxidata (L.) Hoffm. var. chlorophaea (Flörke *ex* Sommerf.) Flörke, Cladonia pyxidata (L.) Hoffm. subsp. chlorophaea (Flörke *ex* Sommerf.) V. Wirth

L – Subs.: ter-cal, ter-sil, xyl, cor – Alt.: 1–5 – Note: closely related to the non-sorediate *C. pyxidata* and rich in ecotypes, several morphologically similar taxa with differing secondary chemistry are treated separately, specimens with unknown chemistry are usually listed here; on acid soil, rotten wood or mossy rocks; widespread on all continents; common throughout the Alps from the lowlands to the alpine belt. – **Au**: V, T, S, K, St, O, N, B. **Ge**: OB, Schw. **Sw**: BE, GL, GR, LU, SZ, TI, UR, VD, VS. **Fr**: AHP, HAl, AMa, Drô, Isè, Sav, HSav, Var, Vau. **It**: Frl, Ven, TAA, Lomb, Piem, VA. **Sl**: SlA, Tg.

***Cladonia ciliata* Stirt.**

Syn.: *Cladina ciliata* (Stirt.) Trass, Cladina ciliata (Stirt.) Trass var. tenuis (Flörke) Ahti & M.J. Lai, *Cladina leucophaea* (Abbayes) Hale & W.L. Culb., *Cladina tenuis* (Flörke) B. de Lesd., Cladonia ciliata Stirt. f. flavicans (Flörke) Ahti & DePriest, Cladonia ciliata Stirt. var. tenuis (Flörke) Ahti, *Cladonia laxiuscula* (Delise) Sandst., *Cladonia leucophaea* Abbayes, Cladonia rangiferina (L.) F.H. Wigg. f. flavicans Flörke, Cladonia rangiferina (L.) F.H. Wigg. var. tenuis Flörke, *Cladonia tenuis* (Flörke) Harm., Cladonia tenuis (Flörke) Harm. var. leucophaea (Abbayes) Ahti

L – Subs.: ter-sil, bry – Alt.: 2–3 – Note: a rare temperate species found on mosses in shrublands, especially in undisturbed maquis vegetation, restricted to humid areas. The species occurs in two chemotypes which rarely grow together, the colour varying from dark brown to straw-yellow in f. flavicans (Flörke) Ahti & DePriest. – **Au**: T, S, O, N. **Ge**: OB. **Sw**: BE, LU, SZ, VD. **Fr**: AHP, AMa, Isè, Sav, Var, Vau. **It**: Ven, TAA, Lig. **Sl**: SlA.

***Cladonia coccifera* (L.) Willd.**

Syn.: *Capitularia asotea* (Ach.) Flörke, Cladonia coccifera (L.) Willd. var. asotea Ach., Cladonia coccifera (L.) Willd. var. stemmatina (Ach.) Vain., *Cladonia cornucopioides* F. Wilson, *Lichen cocciferus* L.

L – Subs.: ter-sil, bry, deb – Alt.: 3–6 – Note: a cool-temperate to arctic-alpine, circumpolar lichen found on soil in open situations, such as in dry tundra-like habitats, more rarely on wood; widespread throughout the Alps. – **Au**: V, T, S, K, St, O, N. **Ge**: OB, Schw. **Sw**: BE, GL, GR, LU, SZ, TI, UR, VS. **Fr**: HAl, AMa, Isè, Sav, HSav, Vau. **It**: Frl, Ven, TAA, Lomb, Piem, VA, Lig. **Sl**: SlA.

***Cladonia coniocraea* (Flörke) Spreng.**

Syn.: *Cenomyce coniocraea Flörke*, *Cladonia apolepta* (Ach.) H.M.M. Hansen & M. Lund, Cladonia fimbriata (L.) Fr. var. coniocraea (Flörke) Nyl., *Cladonia pycnotheliza* Nyl.

L – Subs.: cor, xyl, deb, ter-sil – Alt.: 1–4 – Note: a widespread, holarctic species found on a wide variety of organic substrata, including bark, and then mostly on basal parts of boles, but mostly on soil rich in humus and on rotten wood, with a wide altitudinal range; widespread and common throughout the Alps. See also note on *C. ochrochlora*. – **Au**: V, T, S, K, St, O, N, B. **Ge**: OB, Schw. **Sw**: BE, FR, GL, GR, LU, SG, SZ, TI, UR, UW, VS. **Fr**: AHP, HAl, AMa, Isè, Sav, HSav, Var, Vau. **It**: Frl, Ven, TAA, Lomb, Piem, VA, Lig. **Sl**: SlA, Tg. **Li**.

***Cladonia convoluta* (Lam.) Anders**

Syn.: *Cladonia endiviifolia* (Dicks.) Fr., Cladonia foliacea (Huds.) Willd. subsp. convoluta (Lam.) Clauzade & Cl. Roux, Cladonia foliacea (Huds.) Willd. subsp. endiviifolia (Dicks.) Boistel, Cladonia foliacea (Huds.) Willd. var. convoluta (Lam.) Vain., Cladonia foliacea (Huds.) Willd. var. endiviifolia (Dicks.) Schaer., *Lichen convolutus* Lam.

L # – Subs.: ter-cal – Alt.: 1–3 – Note: a mild-temperate lichen found on calcareous mineral soil in dry grasslands, or in intradunal depressions, also occurring in dry-continental valleys of the Alps. A recent revision of the *C. foliacea-C. convoluta*-complex showed that neither morphological characters nor phylogenetic analyses gave evidence to delimit two taxa; however, since there are some ecological and distributional differences, we prefer to provisionally treat here the calcicolous forms separately. – **Au**: N. **Fr**: AHP, AMa, Drô, Isè, Sav, HSav, Var, Vau. **It**: Frl, Ven, TAA, Lomb, Piem, VA, Lig. **Sl**: SlA.

***Cladonia cornuta* (L.) Hoffm.**

Syn.: Cladonia radiata (Schreb.) Ach. var. cornuta (L.) M. Choisy, *Lichen cornutus* L.

L – Subs.: ter-sil, xyl, deb – Alt.: 2–5 – Note: a boreal-montane to subarctic-subalpine, circumpolar species found on mineral and organic soil, but also on wood, with optimum in subalpine areas with siliceous substrata; widespread throughout the Alps. – **Au**: V, T, S, K, St, O, N. **Ge**: OB, Schw. **Sw**: BE, GR, SZ, TI, VD, VS. **Fr**: AMa, Isè, Sav, HSav. **It**: Ven, TAA, Lomb, Piem, VA. **Sl**: SlA.

**Cladonia crispata (Ach.) Flot. var. crispata**

Syn.: Baeomyces turbinatus Ach. var. crispatus Ach., Cladonia crispata (Ach.) Flot. var. dilacerata (Schaer.) Malbr., Cladonia crispata (Ach.) Flot. var. divulsa (Delise) Arnold, Cladonia crispata (Ach.) Flot. var. elegans (Delise) Vain., Cladonia crispata (Ach.) Flot. var. infundibulifera (Schaer.) Vain., Cladonia crispata (Ach.) Flot. var. subracemosa Vain.

L – Subs.: ter-sil, ter-cal, bry, xyl – Alt.: 3–5 – Note: a boreal-montane to subarctic-subalpine, circumpolar species found on soil, more rarely on lignum, in open habitats, in areas near treeline with siliceous substrata; widespread throughout the Alps. – **Au**: V, T, S, K, St, O, N. **Ge**: OB, Schw. **Sw**: BE, GR, LU, SZ, TI, UR, VD, VS. **Fr**: AHP, AMa, Isè, Sav, HSav. **It**: Frl, Ven, TAA, Lomb, Piem, VA. **Sl**: SlA.

**Cladonia crispata (Ach.) Flot. var. cetrariiformis (Delise) Vain.**

Syn.: Cladonia crispata (Ach.) Flot. var. gracilescens (Rabenh.) Vain.

L – Subs.: ter-sil, bry – Alt.: 4–5 – Note: differing from the type variety in the pointed tips of the very narrow, tube-like funnels, confusable with *C. subfurcata* with glossy esquamulose podetia with melanotic dying bases; on acid soil; common in Atlantic Europe; in the Alps only known from the treeline ecotone upwards, but not common, and not always distinguished from the typical variety. – **Au**: T, S, K, St. **Fr**: Sav, HSav, Var.

***Cladonia cryptochlorophaea* Asahina**

L – Subs.: ter-sil, bry, sil – Alt.: 2–5 – Note: a mainly cool-temperate, perhaps holarctic lichen found on soil rich in humus, on peat, etc., probably with a western distribution in Europe. Perhaps better treated as a chemical strain of *C. grayi*. – **Au**: T, K, St, N. **Sw**: BE, LU, VS. **Fr**: AMa, Var. **It**: Ven. **Sl**: Tg.

***Cladonia cyanipes* (Sommerf.) Nyl.**

Syn.: Cenomyce carneopallida (Ach.) Nyl. var. cyanipes Sommerf.

L – Subs.: bry, sil, ter-sil, xyl – Alt.: 3–5 – Note: a mainly boreal-montane, perhaps circumpolar species found in open heaths and forest glades amongst bryophytes and on organic soil, much more rarely on wood, in areas with siliceous substrata near and above treeline; widespread in the Alps, but generally rare. – **Au**: T, S, K, St, O, N. **Ge**: OB. **Sw**: GR. **Fr**: HSav. **It**: TAA, Piem. **Sl**: SlA.

***Cladonia decorticata* (Flörke) Spreng.**

Syn.: *Capitularia decorticata* Flörke

L – Subs.: ter-sil, deb, xyl – Alt.: 2–4 – Note: an arctic-alpine to boreal-montane, circumpolar species found on mineral, more rarely on organic soil and rotting wood in open habitats, restricted to siliceous areas. – **Au**: T, S, K, St, N. **Sw**: BE, GR, SZ, TI, VS. **Fr**: Isè. **It**: TAA, Lomb, Piem, VA.

***Cladonia deformis* (L.) Hoffm.**

Syn.: *Cladonia crenulata* (Ach.) Flörke, *Lichen deformis* L.

L – Subs.: xyl, cor, deb, ter-sil – Alt.: 3–5 – Note: a mainly boreal-montane, circumpolar species found on rotting wood and organic soil; some records could refer to *C. sulphurina*; widespread throughout the Alps. – **Au**: V, T, S, K, St, O, N. **Ge**: OB, Schw. **Sw**: BE, GR, LU, SZ, TI, UR, UW, VD, VS. **Fr**: HAl, AMa, Isè, Sav, HSav. **It**: Frl, Ven, TAA, Lomb, Piem, VA. **Sl**: SlA.

***Cladonia digitata* (L.) Hoffm.**

Syn.: Cladonia digitata (L.) Hoffm. var. brachytes (Ach.) Vain., Cladonia digitata (L.) Hoffm. var. ceruchoides Vain., *Lichen digitatus* L.

L – Subs.: xyl, cor, bry – Alt.: 3–5 – Note: a cool-temperate to boreal-montane, circumpolar species found on strongly weathered lignum, mosses, at the base of trunks, sometimes on soil rich in humus; widespread throughout the Alps. – **Au**: V, T, S, K, St, O, N, B. **Ge**: OB, Schw. **Sw**: BE, GL, GR, LU, SG, SZ, TI, UR, UW, VD, VS. **Fr**: AHP, HAl, AMa, Isè, Sav, HSav. **It**: Frl, Ven, TAA, Lomb, Piem, VA, Lig. **Sl**: SlA, Tg.

***Cladonia diversa* Asperges *ex* S. Stenroos**

L – Subs.: ter – Alt.: 4–5 – Note: related to *C. coccifera*, and with a similar ecology, but perhaps more bound to humid habitats; probably more widespread in the Alps. – **It**: Frl, Ven, TAA.

***Cladonia ecmocyna* Leight.**

Syn.: Cladonia elongata (Wulfen) Hoffm. var. ecmocyna (Leight.) Räsänen, Cladonia gracilis (L.) Willd. var. ecmocyna (Leight.) Kernst.

L – Subs.: ter-sil – Alt.: 3–5 – Note: a mainly boreal-montane to subarctic-subalpine, circumpolar lichen found on organic soil and amongst bryophytes in cool depressions with a late snow cover. – **Au**: V, T, S, K, St, O. **Sw**: GR, UR, VS. **Fr**: Isè, HSav. **It**: Frl, Ven, TAA, Lomb, VA.

***Cladonia fimbriata* (L.) Fr.**

Syn.: Cladonia fimbriata (L.) Fr. var. dendroides (Flörke) Müll. Arg., Cladonia fimbriata (L.) Fr. var. longipes (Flörke) Rabenh., Cladonia fimbriata (L.) Fr. var. major (K.G. Hagen) H. Magn., Cladonia fimbriata (L.) Fr. var. minor (K.G. Hagen) H. Magn., Cladonia fimbriata (L.) Fr. var. prolifera (Retz.) A. Massal., Cladonia fimbriata (L.) Fr. var. tenuipes (Delise) H. Olivier, *Cladonia major* (K.G. Hagen) Sandst., *Cladonia minor* (K.G. Hagen) Szatala, *Lichen fimbriatus* L.

L – Subs.: ter-sil, bry, deb, cor, xyl – Alt.: 1–5 – Note: a temperate to arctic-alpine, holarctic species found on rotten wood, soil, at the base of trunks, with a wide ecological range and a correspondingly wide altitudinal range; widespread and common throughout the Alps. – **Au**: V, T, S, K, St, O, N, B. **Ge**: OB, Schw. **Sw**: BE, GL, GR, LU, SG, SZ, TI, UR, UW, VD, VS. **Fr**: AHP, HAl, AMa, Drô, Isè, Sav, HSav, Var, Vau. **It**: Frl, Ven, TAA, Lomb, Piem, VA, Lig. **Sl**: SlA, Tg.

***Cladonia firma* (Nyl.) Nyl.**

Syn.: Cladonia alcicornis (Lightf.) Fr. var. firma Nyl., Cladonia foliacea (Huds.) Willd. var. firma (Nyl.) Vain., *Cladonia nylanderi* Cout.

L – Subs.: ter-int – Alt.: 1–2 – Note: a mild-temperate lichen found on mineral, often base-rich soil in open Mediterranean grasslands, with several records from the base of the Western Alps and from dry-continental valleys. – **Fr**: AMa, Var, Vau. **It**: TAA, Lig.

***Cladonia floerkeana* (Fr.) Flörke**

Syn.: *Cenomyce floerkeana* Fr., *Cladonia berghsonii* Asperges, Cladonia floerkeana (Fr.) Flörke var. brebissonii (Delise) Vain., Cladonia floerkeana (Fr.) Flörke var. carcata (Ach.) Vain., Cladonia floerkeana (Fr.) Flörke var. chloroides (Flörke) Vain., Cladonia macilenta Hoffm. subsp. floerkeana (Fr.) V. Wirth, Cladonia macilenta Hoffm. var. carcata (Ach.) Nyl., Cladonia macilenta Hoffm. var. corticata Vain.

L – Subs.: ter-sil, bry, xyl, deb – Alt.: 2–4 – Note: a circumboreal-montane species found on organic soil and peat, but also on sand, more rarely on lignum, with optimum in the subalpine belt; widespread throughout the Alps but only locally common. – **Au**: V, T, S, K, St, O, N. **Ge**: OB. **Sw**: BE, GR, LU, VD, VS. **Fr**: Isè, Sav, HSav. **It**: Frl, TAA, Lomb, Piem, VA. **Sl**: SlA.

***Cladonia foliacea* (Huds.) Willd.**

Syn.: *Cladonia alcicornis* (Lightf.) Fr., Cladonia foliacea (Huds.) Willd. var. alcicornis (Lightf.) Schaer., Cladonia foliacea (Huds.) Willd. var. damicornis (Ach.) Th. Fr., *Lichen foliaceus* Huds.

L – Subs.: ter-sil – Alt.: 1–3 – Note: a mild-temperate lichen, an ecological vicariant of *C. convoluta* on more or less acid, but often base-rich ground. See also note on *C. convoluta*. – **Au**: K, St, B. **Sw**: GR, LU, TI, VD, VS. **Fr**: AHP, HAl, AMa, Drô, Isè, Sav, HSav, Var, Vau. **It**: Ven, TAA, Lomb, Piem, VA.

***Cladonia furcata* (Huds.) Schrad.**

Syn.: *Cenomyce furcata* (Huds.) Ach., *Cladonia corymbosa* (Ach.) Krohn, Cladonia furcata (Huds.) Schrad. var. corymbosa (Ach.) Nyl., Cladonia furcata (Huds.) Schrad. var. palamaea (Ach.) Nyl., Cladonia furcata (Huds.) Schrad. var. pinnata (Flörke) Vain., Cladonia furcata (Huds.) Schrad. var. racemosa (Hoffm.) Flörke, Cladonia furcata (Huds.) Schrad. var. subulata Flörke, *Cladonia racemosa* Hoffm., *Lichen furcatus* Huds.

L – Subs.: ter-cal, ter-sil, bry, xyl – Alt.: 1–5 – Note: a holarctic, temperate to boreal-montane lichen found on soil, amongst mosses, sometimes on bark and lignum, in areas with calcareous or siliceous base-rich rocks, with a wide altitudinal range; widespread and common throughout the Alps. – **Au**: V, T, S, K, St, O, N, B. **Ge**: OB, Schw. **Sw**: BE, GR, LU, SG, SZ, TI, UR, UW, VD, VS. **Fr**: AHP, HAl, AMa, Drô, Isè, Sav, HSav, Var, Vau. **It**: Frl, Ven, TAA, Lomb, Piem, VA, Lig. **Sl**: SlA, Tg.

***Cladonia glauca* Flörke**

Syn.: Cladonia cenotea (Ach.) Schaer. var. glauca (Flörke) Leight.

L – Subs.: ter-sil, xyl – Alt.: 2–4 – Note: a cool-temperate to boreal-montane, perhaps circumpolar lichen found on acid soil in open habitats; widespread throughout the Alps, but generally rare. – **Au**: V, T, S, K, St, O. **Sw**: GR, LU, VD. **Fr**: AHP, HAl, AMa, Var, Vau. **It**: Ven, TAA, Piem, VA. **Sl**: SlA.

**Cladonia gracilis (L.) Willd. subsp. gracilis**

Syn.: *Cladonia chordalis* (Flörke) Nyl., Cladonia gracilis (L.) Willd. var. aspera Flörke, Cladonia gracilis (L.) Willd. var. chordalis (Flörke) Schaer., *Lichen gracilis* L.

L – Subs.: ter-cal, ter-sil, deb – Alt.: 2–4 – Note: a circumpolar, cool-temperate to southern arctic lichen found on acid soil, more rarely on decaying wood in upland areas. According to Ahti (see Nimis 2016) its presence in the Alps is dubious, and most records could refer to *C. macroceras*. – **Au**: V, T, S, K, St, O. **Ge**: OB, Schw. **Sw**: BE, GR, LU, SZ, TI, UR, VD, VS. **Fr**: AHP, HAl, AMa, Drô, Isè, Sav, HSav, Vau. **It**: Frl, Ven, TAA, Lomb, Piem, VA. **Sl**: SlA.

**Cladonia gracilis (L.) Willd. subsp. turbinata (Ach.) Ahti**

Syn.: *Cladonia gracilis* (L.) Willd. var. * auct.*, *Cladonia pachyscypha* Sandst. *ex* Zahlbr., *Lichen turbinatus* Ach.

L – Subs.: ter – Alt.: 4 – Note: a taxon with short podetia, all with scyphi (subulate podetia lacking); on the forest floor, but also on rotten wood; common in the middle boreal zone, in the Alps in coniferous forests of the upper montane belt (not above treeline); there are several scattered records, but infraspecific taxa of the *C. gracilis*-group were not always distinguished. According to Ahti (see Nimis 2016), however, the presence of *C. gracilis* in the Alps is dubious, and most records could prove to refer to *C. macroceras* – **Sw**: GR, LU, SZ. **Fr**: Sav. **Sl**: SlA.

***Cladonia grayi* G. Merr. *ex* Sandst.**

Syn.: Cladonia pyxidata (L.) Hoffm. subsp. grayi (G. Merr *ex* Sandst.) V. Wirth

L – Subs.: xyl, deb, bry, ter-cal – Alt.: 2–4 – Note: a holarctic, rather northern representative of the *C. pyxidata*-*chlorophaea*-complex, found on soil rich in humus, peat, and rotting wood. – **Au**: V, S, K, St, O. **Sw**: BE, LU, UR, VS. **Fr**: Isè, Sav, HSav. **It**: Frl, Ven, TAA, Piem, VA. **Sl**: SlA.

***Cladonia humilis* (With.) J.R. Laundon**

Syn.: *Cladonia conistea* (“Delise”) Asahina, *Cladonia conoidea* Ahti, *Lichen humilis* With.

L – Subs.: ter-sil, xyl, sil – Alt.: 1–3 – Note: a mild-temperate, widespread species found on disturbed, often sandy soil, more rarely on lignum and mossy trees; apparently more frequent in the Southern and Western Alps. – **Au**: T. **Sw**: VS. **Fr**: AHP, HAl, HSav, Var. **It**: Ven, Lomb, Piem, Lig. **Sl**: SlA.

***Cladonia incrassata* Flörke**

Syn.: Cladonia coccifera (L.) Willd. var. incrassata (Flörke) Laurer

L – Subs.: bry, deb – Alt.: 2–3 – Note: a cool-temperate to boreal-montane species with a fragmented circumpolar range, found on peaty and humus-rich soil and on strongly weathered lignum; with a few scattered records from the Alps, where it is evidently rare. – **Au**: S, N. **Sw**: UW. **Fr**: Isè, HSav. **It**: Lomb, Piem. **Sl**: SlA.

***Cladonia luteoalba* Wheldon & A. Wilson**

L – Subs.: deb, sil – Alt.: 4 – Note: a species with a conspicuous primary thallus consisting of large squamules which are revolute when dry, with a yellow underside, podetia rarely present, small, unbranched, ecorticate, with subulate tips; on soil layers over siliceous rocks and plant debris in open habitats; widespread in Europe but not common, with a few records from the Eastern Alps. – **Au**: T, S.

***Cladonia macilenta* Hoffm.**

Syn.: *Cladonia bacillaris* (Ach.) Genth, Cladonia macilenta Hoffm. var. clavata (Ach.) H. Olivier, Cladonia macilenta Hoffm. var. scabrosa (Mudd) Cromb., Cladonia macilenta Hoffm. var. squamigera Vain.

L – Subs.: xyl, deb, cor, ter-sil – Alt.: 2–4 – Note: a cool-temperate to boreal-montane, circumpolar lichen found on different organic substrata such as rotting wood, bark (mostly on basal parts of trunks), and more rarely on soil rich in humus; widespread throughout the Alps. – **Au**: V, T, S, K, St, O, N, B. **Ge**: OB, Schw. **Sw**: BE, GR, LU, SG, SZ, TI, UR, UW, VD, VS. **Fr**: AHP, AMa, Isè, HSav, Var, Vau. **It**: Frl, Ven, TAA, Lomb, Piem, VA. **Sl**: SlA, Tg.

***Cladonia macroceras* (Delise) Hav.**

Syn.: Cenomyce gracilis (L.) Dufour var. macroceras Delise, *Cladoniaelongata auct. p.p.*, Cladonia gracilis (L.) Willd. var. macroceras (Delise) Flot.

L – Subs.: ter-sil, ter-cal, bry, deb – Alt.: 2–5 – Note: a subarctic-subalpine, circumpolar lichen, one of the most abundant species in *Rhododendron* heaths throughout the Alps, mostly deeply immersed amongst mosses; widespread and common throughout the Alps. See also note on *C. gracilis*. – **Au**: V, T, S, K, St, O, N. **Ge**: OB, Schw. **Sw**: BE, GR, LU, SZ, TI, UR, UW, VD, VS. **Fr**: AHP, HAl, AMa, Isè, Sav, HSav. **It**: Frl, Ven, TAA, Lomb, Piem, VA. **Sl**: SlA, Tg.

***Cladonia macrophylla* (Schaer.) Stenh.**

Syn.: *Cladonia alpicola* (Flot.) Vain., Cladonia ventricosa (Lightf.) J.F. Gmel. var. macrophylla Schaer.

L – Subs.: ter-sil, bry, deb – Alt.: 3–5 – Note: a northern-Alpine species found on organic soil and weathered siliceous rocks; widespread in the Alps but generally not common. – **Au**: T, S, K, St, O, N. **Ge**: OB, Schw. **Sw**: BE, GR, VS. **Fr**: Isè, HSav. **It**: TAA, Lomb, Piem, VA. **Sl**: SlA.

***Cladonia macrophyllodes* Nyl.**

Syn.: Cladonia lepidota Nyl. var. macrophyllodes (Nyl.) Du Rietz

L – Subs.: ter-sil, bry – Alt.: 4–6 – Note: an arctic-alpine, circumpolar species found on soil in open sites with a long snow cover, optimum in the alpine belt of the siliceous Alps. – **Au**: V, T, S, K, St, O, N. **Ge**: OB, Schw. **Sw**: BE, GR, TI, UR, VS. **Fr**: HAl, Isè, Sav, HSav. **It**: Frl, Ven, TAA, Lomb, Piem, VA. **Sl**: SlA.

***Cladonia magyarica* Vain.**

L – Subs.: ter-cal – Alt.: 2 – Note: a species recalling *C. pyxidata*, with a primary thallus of erect squamules showing the white underside when dry, and podetia, including the inner side of scyphi, covered with elongate squamules; a lowland species of sandy soil in continental parts of Europe; in the Alps only known from the easternmost foothills. – **Au**: N.

***Cladonia mediterranea* P.A. Duvign. & Abbayes**

Syn.: *Cladina mediterranea* (P.A. Duvign. & Abbayes) Follmann & Hern.-Padr., *Cladonia macaronesica* Ahti

L – Subs.: ter, bry – Alt.: 1 – Note: a Mediterranean-Macaronesian lichen found in Mediterranean maquis vegetation amongst pleurocarpous mosses in sheltered situations with plenty of diffuse light, with a few records from the base of the Western Alps. – **Fr**: AMa, Var, Vau.

***Cladonia merochlorophaea* Asahina**

L – Subs.: sil, ter-sil, bry – Alt.: 2–5 – Note: a mainly cool-temperate, probably circumpolar lichen found on humus-rich soil. – **Au**: T, S, St, O. **Ge**: OB, Schw. **Sw**: LU, SZ, VS. **Fr**: AMa, HSav. **It**: Frl, TAA, Lomb, Piem, VA. **Sl**: Tg.

***Cladonia mitis* Sandst.**

Syn.: *Cladina mitis* (Sandst.) Hustich, Cladonia arbuscula (Wallr.) Flot. subsp. mitis (Sandst.) Ruoss, *Cladonia subsylvatica* Stirt.

L – Subs.: ter-sil, ter-cal, bry, xyl, cor – Alt.: 3–5 – Note: a typical member of subalpine-alpine tundras, perhaps more common at higher altitudes than *C. arbuscula*; widespread throughout the Alps. – **Au**: V, T, S, K, St, O, N. **Ge**: OB, Schw. **Sw**: AP, BE, GL, GR, LU, SG, SZ, TI, UR, UW, VD, VS. **Fr**: AHP, HAl, AMa, Drô, Isè, Sav, HSav, Var, Vau. **It**: Frl, Ven, TAA, Lomb, Piem, VA, Lig.

***Cladonia monomorpha* Aptroot, Sipman & Herk**

Syn.: Cladonia pyxidata (L.) Hoffm. var. baccifera Räsänen

L # – Subs.: ter-sil – Alt.: 2–5 – Note: only recently recognised as a distinct species in the *C. pyxidata*-group, and perhaps more widespread, this species should be characterised by squamules with narrowly recurved margins, by the presence of discoid, bullate plates on the podetial surface, and by long and sometimes branched proliferations of the scyphus margins supporting the apothecial discs. The species was described from the Netherlands, where it occurs in acid inland sand dunes with the highest terrestrial lichen diversity, and it appears to be widespread in Europe on siliceous rocks and acid sand. According to Ahti (see Nimis 2016) preliminary DNA data from the type locality show that it does not differ from “normal” *C. pyxidata*, except that *C. pyxidata* is not uniform at all. However, the type of *C. pyxidata*, which comes from Italy, is morphologically different. – **Au**: V, S, St, N. **Sw**: SZ. **It**: Lomb.

***Cladonia norvegica* Tønsberg & Holien**

L – Subs.: xyl, cor – Alt.: 3–4 – Note: a cool-temperate to boreal-montane lichen found on decaying trunks and stumps in moist-shaded habitats such as ancient, undisturbed woodlands; when epiphytic, on basal parts of conifers; probably more widespread in the Alps. – **Au**: S, K, St, O, N. **Ge**: OB, Schw. **Sw**: SZ, UW. **It**: Frl. **Sl**: SlA.

***Cladonia novochlorophaea* (Sipman) Brodo & Ahti**

Syn.: Cladonia merochlorophaea Asahina var. novochlorophaea Sipman

L – Subs.: ter-sil – Alt.: 2–5 – Note: a species of the *C. grayi*-group, with a primary thallus of non-coalescent small squamules and usually dark brown, scyphose podetia with a corticate, verruculose surface lacking sorediate granules, but later with corticate schizidia; confirmation by analysis of secondary chemistry is necessary for a correct identification; on acid soil over siliceous rocks and in arctic-alpine heaths; widespread but not common, in the Alps, so far with a few records only. – **Fr**: AMa.

***Cladonia ochrochlora* Flörke**

Syn.: Cladonia fimbriata (L.) Fr. var. ochrochlora (Flörke) Schaer., Cladonia furcata (Huds.) Schrad. var. notabilis Müll. Arg., *Cladonia lepidula* Kremp., Cladonia ochrochlora Flörke var. pycnotheliza (Nyl.) Harm., Cladonia ochrochlora Flörke var. spadicea Müll. Arg., *Cladonia pergracilis* Kremp.

L – Subs.: cor, xyl, deb, ter-sil – Alt.: 2–4 – Note: very similar to *C. coniocraea*, but with podetia corticated in the lower third and at the bottom of the often present scyphi; a mainly temperate species found on rotten wood and at the base of tree trunks in both deciduous and coniferous forests; in the Alps it ranges from the lowlands to the montane belt, and is rather common. – **Au**: V, T, S, K, St, O, N, B. **Ge**: OB, Schw. **Sw**: BE, GR, LU, SZ, UR, VD, VS. **Fr**: AHP, AMa, Isè, HSav. **It**: Frl, Ven, TAA, Lomb, Piem, VA. **Sl**: Tg.

***Cladonia parasitica* (Hoffm.) Hoffm.**

Syn.: *Cladonia delicata auct.*, *Lichen parasiticus* Hoffm.

L – Subs.: xyl, cor – Alt.: 2–3 – Note: a mainly temperate, probably holarctic species, normally lignicolous, on stumps, sometimes on basal parts of old trunks; widespread in the Alps. – **Au**: S, K, St, O, N. **Ge**: OB, Schw. **Sw**: BE, TI, UR, VD. **Fr**: AMa, Isè, Sav, HSav, Var. **It**: Frl, Ven, TAA, Lomb, Piem, VA, Lig. **Sl**: SlA, Tg.

***Cladonia peziziformis* (With.) J.R. Laundon**

Syn.: *Cladonia capitata* (Michx.) Spreng., Cladonia cariosa (Ach.) Spreng. var. leptophylla (Ach.) Hepp, *Cladonia leptophylla* (Ach.) Flörke, *Cladonia leptophylloides* Harm., *Lichen peziziformis* With.

L – Subs.: ter-sil – Alt.: 2–3 – Note: a mainly temperate lichen found on soil in open woodlands (oak, pine), in areas with siliceous substrata, with a few scattered records from the Alps. – **Au**: St. **Sw**: VD, VS. **Fr**: Isè. **It**: Lig.

***Cladonia phyllophora* Hoffm.**

Syn.: *Baeomyces degenerans* Flörke, *Cladonia degenerans* (Flörke) Spreng., *Cladonia lepidota* (Ach.) Nyl. *non auct.*, *Cladonia trachyna* (Ach.) Nyl.

L – Subs.: ter-sil, bry, deb – Alt.: 2–5 – Note: a cool-temperate to boreal-montane, probably holarctic lichen found on acid mineral soil; widespread throughout the Alps. – **Au**: V, T, S, K, St, O, N. **Ge**: OB. **Sw**: BE, GR, SZ, TI, UR, VD, VS. **Fr**: HAl, AMa, HSav. **It**: Frl, Ven, TAA, Lomb, Piem, VA. **Sl**: SlA.

***Cladonia pleurota* (Flörke) Schaer.**

Syn.: *Capitularia pleurota* Flörke, Cladonia coccifera (L.) Willd. subsp. pleurota (Flörke) Vain., Cladonia coccifera (L.) Willd. var. pleurota (Flörke) Schaer., *Cladonia frondescens* Nyl.

L – Subs.: xyl, deb, ter-sil – Alt.: 3–5 – Note: an arctic-alpine to boreal-montane, circumpolar lichen found on soil, rotting wood, more rarely on basal parts of trunks in open habitats, with optimum near or above treeline; widespread and common throughout the Alps. – **Au**: V, T, S, K, St, O, N. **Ge**: OB, Schw. **Sw**: BE, GR, LU, SG, SZ, TI, UR, VD, VS. **Fr**: AHP, AMa, Isè, Sav, HSav. **It**: Frl, Ven, TAA, Lomb, Piem, VA. **Sl**: SlA.

***Cladonia pocillum* (Ach.) Grognot**

Syn.: *Baeomyces pocillum* Ach., Cladonia pyxidata (L.) Hoffm. subsp. pocillum (Ach.) Vain., Cladonia pyxidata (L.) Hoffm. var. pocillum (Ach.) Schaer.

L – Subs.: ter-cal, bry, deb – Alt.: 1–5 – Note: a widespread holarctic species found on calciferous soil and amongst bryophytes in dry, open grasslands. The species, in its current circumscription, is heterogeneous; widespread and common throughout the Alps. – **Au**: V, T, S, K, St, O, N. **Ge**: OB, Schw. **Sw**: BE, GR, LU, SZ, TI, UR, VS. **Fr**: AHP, HAl, AMa, Drô, Isè, Sav, HSav, Var, Vau. **It**: Frl, Ven, TAA, Lomb, Piem, VA, Lig. **Sl**: SlA, Tg. **Li**.

***Cladonia polycarpoides* Nyl.**

Syn.: *Cladonia subcariosa auct*.

L – Subs.: ter-cal, ter-sil – Alt.: 2–3 – Note: a mainly temperate lichen found on calcareous mineral soil in open grasslands and on soil pockets on large isolated boulders. – **Au**: K, N. **Sw**: BE, GR, TI, VD. **Fr**: Sav, HSav. **It**: Frl, TAA, Lomb, Piem, VA, Lig.

***Cladonia polydactyla* (Flörke) Spreng.**

Syn.: *Cenomyce polydactyla* Flörke, *Cladonia bouillenei* P.A. Duvign., Cladonia brebissonii (Delise) Parrique subsp. monguillonii (Harm.) Choisy, Cladonia digitata (L.) Hoffm. var. deminuta Mong., *Cladonia flabelliformis* Vain., *Cladonia monguillonii* Harm.

L – Subs.: xyl, cor, ter – Alt.: 2–4 – Note: a cool-temperate to boreal-montane, circumpolar lichen found on organic soil and rotting wood in forests, more rarely on bark, in the basal parts of old trunks; widespread throughout the Alps. – **Au**: V, T, S, K, St, O, N. **Ge**: OB, Schw. **Sw**: BE, GR, SZ, VD, VS. **Fr**: AMa, Isè, Sav, HSav, Var. **It**: Frl, Ven, TAA, Lomb, Piem, VA. **Sl**: SlA, Tg.

***Cladonia portentosa* (Dufour) Coem.**

Syn.: *Cenomyce portentosa* Dufour, *Cladina impexa* (Harm.) B. de Lesd., *Cladina portentosa* (Dufour) Follmann, *Cladonia condensata* (Sandst.) Zahlbr., *Cladonia impexa* Harm., *Cladonia laxiuscula auct.*, *Cladonia spumosa* (Flörke) Zahlbr.

L – Subs.: ter-sil, bry, deb – Alt.: 2–4 – Note: a mainly western species in Europe, found on acid soil in open situations, such as in *Calluna*-heaths; most of the records from the Alps (especially the Central and Eastern Alps) require re-confirmation. – **Au**: V, T, S, K, O. **Ge**: OB. **Sw**: BE, GR, LU, SZ, UR, UW, VD. **Fr**: AHP, HAl, AMa, Isè, Sav, HSav, Var, Vau. **It**: TAA, Lomb, Piem, VA, Lig. **Sl**: SlA.

***Cladonia pseudopityrea* Vain.**

L – Subs.: xyl, ter – Alt.: 2 – Note: a Mediterranean to Mediterranean-montane species found on lignum *e.g.* of *Olea*, *Abies*, *Pinus*, *Fagus*, but also on soil rich in humus in forests, especially along creeks; with a few records from the Alps of Switzerland. – **Sw**: UR, TI.

***Cladonia pyxidata* (L.) Hoffm.**

Syn.: *Cladonia neglecta* (Flörke) Spreng., *Lichen pyxidatus* L.

L # – Subs.: ter-cal, ter-sil, bry, deb, xyl, cor – Alt.: 1–6 – Note: a widespread, very polymorphic, holarctic species with a wide altitudinal-latitudinal range, which is common throughout the Alps. In its present circumscription, however, the species appears to be heterogeneous. See also note on *C. monomorpha*. – **Au**: V, T, S, K, St, O, N, B. **Ge**: OB, Schw. **Sw**: BE, GL, GR, LU, SG, SZ, TI, UR, UW, VD, VS. **Fr**: AHP, HAl, AMa, Drô, Isè, Sav, HSav, Var, Vau. **It**: Frl, Ven, TAA, Lomb, Piem, VA, Lig. **Sl**: SlA, Tg. **Li**.

***Cladonia ramulosa* (With.) J.R. Laundon**

Syn.: *Baeomyces anomaeus* Ach., *Capitularia pityrea* Flörke, *Cenomyce pityrea* (Flörke) Ach., *Cladonia anomaea* (Ach.) Ahti & P. James, *Cladonia lamarckii* Nyl., *Cladonia pityrea* (Flörke) Fr., *Lichen ramulosus* With.

L – Subs.: bry, sil, cor, xyl, ter-sil – Alt.: 1–3 – Note: a mainly temperate to southern boreal-montane lichen found on epilithic bryophytes, rotting wood and organic soil. – **Au**: T, S, K, St, O, N. **Sw**: BE, GR, SZ, TI, VD. **Fr**: AHP, HAl, AMa, Isè, Sav, HSav, Var, Vau. **It**: Frl, Ven, TAA, Lomb, Piem, VA. **Sl**: SlA.

***Cladonia rangiferina* (L.) F.H. Wigg.**

Syn.: *Cladina alpestris* (L.) Nyl. *non auct.*, *Cladina rangiferina* (L.) Nyl., *Cladonia alpestris* (L.) Rabenh. *non auct.*, *Cladonia gigantea* (Bory) Abbayes, *Cladonia vicaria* R. Sant., *Lichen rangiferinus* L., *Patellaria rangiferina* (L.) Wallr.

L – Subs.: ter-sil, ter-cal, xyl – Alt.: 2–5 – Note: a circumpolar arctic-alpine lichen, one of the most abundant elements of lichen-rich tundra-like vegetation on mineral soil in exposed habitats, with optimum near and above treeline; widespread throughout the Alps. – **Au**: V, T, S, K, St, O, N, B. **Ge**: OB, Schw. **Sw**: AP, BE, GL, GR, LU, SG, SZ, TI, UR, UW, VD, VS. **Fr**: AHP, HAl, AMa, Isè, Sav, HSav. **It**: Frl, Ven, TAA, Lomb, Piem, VA. **Sl**: SlA, Tg.

***Cladonia rangiformis* Hoffm.**

Syn.: *Cladonia aberrans auct.*, Cladonia furcata (Huds.) Schrad. subsp. rangiformis (Hoffm.) Boistel, *Cladonia muricata auct.*, *Cladonia pungens* (Ach.) Gray, Cladonia rangiformis Hoffm. var. muricata (Delise) Arnold, Cladonia rangiformis Hoffm. var. pungens (Ach.) Vain.

L – Subs.: ter-cal, deb – Alt.: 1–3 – Note: a mainly temperate species found on calciferous or base-rich siliceous soil in open habitats, with optimum in dry grasslands; widespread throughout the Alps. – **Au**: V, S, K, St, O, N, B. **Ge**: OB, Schw. **Sw**: GR, LU, SZ, TI, VS. **Fr**: AHP, HAl, AMa, Drô, Isè, Sav, HSav, Var, Vau. **It**: Frl, Ven, TAA, Lomb, Piem, Lig. **Sl**: SlA.

***Cladonia rei* Schaer.**

Syn.: Cladonia fimbriata (L.) Fr. var. nemoxyna (Ach.) Coem., *Cladonia nemoxyna* (Ach.) Arnold

L – Subs.: ter-sil, deb – Alt.: 2–3 – Note: a mainly temperate, probably holarctic species found on mineral clay and base-rich soil, mostly in slightly disturbed habitats such as track sides and clearings of open forests and heaths. – **Au**: V, T, K, St, O. **Sw**: BE, GR. **Fr**: HSav. **It**: Frl, Ven, TAA, Lomb, Piem. **Sl**: SlA.

***Cladonia scabriuscula* (Delise) Nyl.**

Syn.: *Cenomyce scabriuscula* Delise, Cladonia furcata (Huds.) Schrad. var. adspersa (Flörke) F. Wilson, Cladonia furcata (Huds.) Schrad. var. pungens Ach. *non* Fr., Cladonia furcata (Huds.) Schrad. var. recurva A.L. Sm., Cladonia furcata (Huds.) Schrad. var. scabriuscula (Delise) Coem., *Cladonia pungens* (Ach.) Gray *non auct.*, *Cladonia surrecta* (Flörke) Sandst.

L – Subs.: ter-sil, deb – Alt.: 1–3 – Note: a mainly temperate, widespread but rare lichen found on soil and amongst mosses in humid-sheltered situations, such as in open woodlands. – **Au**: N. **Fr**: AHP, Isè. **It**: Frl, TAA, Piem.

**Cladonia squamosa Hoffm. var. squamosa**

Syn.: *Cenomyce sparassa* (Ach.) Ach., Cladonia squamosa Hoffm. var. denticollis (Hoffm.) Flörke, Cladonia squamosa Hoffm. var. levicorticata Sandst., Cladonia squamosa Hoffm. var. muricella (Delise) Vain., Cladonia squamosa Hoffm. var. phyllocoma (Rabenh.) Vain., Cladonia squamosa Hoffm. var. polychonia Flörke

L – Subs.: ter-sil, xyl, deb, bry, cor – Alt.: 1–5 – Note: a holarctic lichen found on organic substrata in sheltered situations, rarely on bark, in the basal parts of trunks; a very polymorphic taxon, widespread throughout the Alps. – **Au**: V, T, S, K, St, O, N, B. **Ge**: OB, Schw. **Sw**: BE, GL, GR, LU, SZ, TI, UR, UW, VD, VS. **Fr**: AMa, Drô, Isè, Sav, HSav, Var, Vau. **It**: Frl, Ven, TAA, Lomb, Piem, VA. **Sl**: SlA, Tg.

**Cladonia squamosa Hoffm. var. subsquamosa (Nyl. *ex* Leight.) Vain.**

Syn.: Cladonia delicata (Ehrh.) Flörke var. subsquamosa Nyl. *ex* Leight., Cladonia squamosa Hoffm. var. allosquamosa Hennipman, *Cladonia subsquamosa* (Nyl. *ex* Leight.) Cromb.

L – Subs.: cor, xyl – Alt.: 2–4 – Note: more hygrophytic than the typical variety, and more bound to higher altitudes. – **Au**: V, T, S, K, St, O, N. **Ge**: OB. **Fr**: AHP, AMa, Isè, Sav, HSav, Var. **It**: Frl, Ven, Lomb, Piem, VA. **Sl**: SlA.

***Cladonia stellaris* (Opiz) Pouzar & Vězda**

Syn.: *Cenomyce stellaris* Opiz, *Cladina alpestris auct. non* (L.) Nyl., *Cladina stellaris* (Opiz) Brodo, *Cladonia aberrans* (Abbayes) Stuckenb., *Cladonia alpestris auct. non* (L.) Rabenh.

L – Subs.: ter-sil, bry, deb – Alt.: 3–5 – Note: a circumpolar, subarctic-subalpine species found in wind-protected sites with a long snow cover; widespread throughout the Alps, but generally rare. – **Au**: V, T, S, K, St, O, N. **Ge**: OB. **Sw**: BE, GR, LU, SG, SZ, TI, UR, VD, VS. **Fr**: Isè, Sav, HSav. **It**: Ven, TAA, Lomb, Piem, VA. **Sl**: SlA.

***Cladonia straminea* (Sommerf.) Flörke**

Syn.: *Cenomyce straminea* Sommerf., *Cladonia metacorallifera* Asahina

L – Subs.: bry, sil – Alt.: 4 – Note: a species of the *C. coccifera*-group, better known under its synonym *C. metacorallifera*, with dark green podetia covered by minute squamules, and narrow scyphi; mostly on siliceous substrata in places with little snow in winter; widespread in the Northern Hemisphere, with a few records from the Eastern Alps, somewhat below treeline. – **Au**: T, S, St. **Sl**: SlA.

***Cladonia strepsilis* (Ach.) Grognot**

Syn.: *Baeomyces strepsilis* Ach.

L – Subs.: ter-sil, ter-cal, deb – Alt.: 3–5 – Note: a cool-temperate to boreal-montane lichen found on humus-rich soil overlaying siliceous rocks and amongst bryophytes in humid depressions periodically filled by water, in open situations. – **Au**: V, T, S, K, St, N. **Sw**: VS, TI. **Fr**: HSav. **It**: Frl, TAA, Lomb, Piem, Lig.

***Cladonia stygia* (Fr.) Ruoss**

Syn.: *Cladina stygia* (Fr.) Ahti, Cladonia rangiferina (L.) F.H. Wigg. f. stygia Fr.

L – Subs.: bry – Alt.: 2–4 – Note: a species recalling a robust *C. rangiferina*, but the dying bases of podetia strongly blackening, and the pycnidial slime red; in bogs and similar moist habitats; widespread in the Holarctic region, most common in the northern boreal zone; in the Alps rare, and restricted to raised bogs from the valley bottoms to the subalpine belt. – **Au**: V, T, S, K, St, O. **Ge**: OB, Schw. **Sw**: BE, GR, LU, UW, VD. **Fr**: HSav. **It**: VA. **Sl**: SlA.

***Cladonia subcervicornis* (Vain.) Kernst.**

Syn.: Cladonia verticillata (Hoffm.) Schaer. var. subcervicornis Vain.

L – Subs.: ter-sil – Alt.: 1–3 – Note: on siliceous rocks and on soil rich in humus in open habitats; very rare in the Alps. – **Sw**: ?TI, ?VS. **Fr**: AMa, Isè, Vau. **It**: Ven, Piem.

***Cladonia subfurcata* (Nyl.) Arnold**

Syn.: Cladonia degenerans (Flörke) Spreng. f. subfurcata Nyl.

L – Subs.: ter-sil – Alt.: 4 – Note: similar to C. crispata var. cetrariiformis, but with glossy esquamulose podetia with melanotic dying bases; in bogs and mountain heaths; the centre of distribution is in the Arctic and northern boreal zone, with a single record from the Eastern Alps. – **Au**: T.

***Cladonia sublacunosa* Vain.**

L # – Subs.: ter-sil – Alt.: 4–5 – Note: a species of the *C. uncialis*-group, with podetia lacking a cortical layer in the upper parts, which are distinctly arachnoid; on stony siliceous soil between siliceous boulders; only known from two high-elevation localities in the Eastern Alps; its taxonomic status is uncertain. – **Au**: T. **Ge**: OB.

***Cladonia subrangiformis* Sandst.**

Syn.: Cladonia furcata (Huds.) Schrad. subsp. subrangiformis (Sandst.) Abbayes *nom.illeg.*, Cladonia furcata (Huds.) Schrad. var. subrangiformis (Sandst.) Hennipman *nom.illeg*.

L # – Subs.: ter-cal, ter-sil, bry – Alt.: 1–3 – Note: a mild-temperate lichen found on mineral calciferous soil, often amongst bryophytes. This taxon has so far no valid name at subspecific rank, and recent molecular data do not support its separation from *C. furcata*, so that it could be better treated at the level of forma. – **Au**: T, S, K, St, O. **Sw**: GR, VS. **Fr**: HAl, AMa, Sav. **It**: Ven, TAA, Lomb, Piem, VA. **Sl**: SlA.

***Cladonia subulata* (L.) F.H. Wigg.**

Syn.: *Cladonia cornutoradiata* (Vain.) Zopf, Cladonia fimbriata (L.) Fr. var. cornutoradiata Vain., Cladonia fimbriata (L.) Fr. var. radiata (Schreb.) Cromb., Cladonia fimbriata (L.) Fr. var. subcornuta Nyl *ex* Cromb., Cladonia fimbriata (L.) Fr. var. subulata (L.) Vain., *Cladonia radiata* (Schreb.) Ach., Cladonia subulata (L.) F.H. Wigg. var. radiata (Schreb.) Ozenda & Clauzade, *Lichen subulatus* L.

L – Subs.: ter-sil, deb, bry – Alt.: 2–4 – Note: a cool-temperate to subarctic lichen found on mineral soil on track sides and in clearings of open forests and heaths, more rarely on rotting wood, in areas with siliceous substrata; several records from the Alps require re-confirmation. – **Au**: V, T, S, K, St, O, N, B. **Ge**: OB, Schw. **Sw**: BE, GL, GR, LU, SZ, TI, UR, VD, VS. **Fr**: AHP, AMa, Isè, Sav, HSav, Var, Vau. **It**: Frl, Ven, TAA, Lomb, Piem. **Sl**: SlA.

***Cladonia sulphurina* (Michx.) Fr.**

Syn.: Cladonia deformis (L.) Hoffm. var. gonecha (Ach.) Arnold, *Cladonia gonecha* (Ach.) Asahina, *Scyphophorus sulphurinus* Michx.

L – Subs.: xyl, bry, deb, ter-sil – Alt.: 3–5 – Note: a circumboreal-subarctic lichen found on organic substrata in cold-shaded situations, most common on rotting wood, *e.g.* on stumps and decaying fallen trunks; widespread throughout the Alps. – **Au**: V, T, S, K, St, O, N. **Ge**: OB. **Sw**: GR, LU, SZ, TI, UR, VS. **Fr**: HAl, Isè, Sav, HSav. **It**: Frl, Ven, TAA, Lomb, Piem, VA. **Sl**: SlA.

***Cladonia symphycarpa* (Flörke) Fr.**

Syn.: *Capitularia symphycarpa* Flörke, *Cladonia dahliana* Kristinsson, *Cladonia hungarica* (Vain.) Szatala, *Cladonia symphycarpia auct*.

L – Subs.: ter-cal, bry – Alt.: 1–5 – Note: a holarctic species found on calcareous ground in dry grasslands, or on the top of exposed calcareous boulders; widespread throughout the Alps. – **Au**: V, T, S, K, St, O, N. **Ge**: OB, Schw. **Sw**: BE, GR, LU, SZ, TI, UR, VD, VS. **Fr**: AHP, HAl, AMa, Drô, Isè, Sav, HSav, Var, Vau. **It**: Frl, Ven, TAA, Lomb, Piem, VA. **Sl**: SlA. **Li**.

***Cladonia trassii* Ahti**

Syn.: *Cladonia lepidota auct*.

L – Subs.: ter – Alt.: 4–5 – Note: an arctic-alpine species also recorded from Tierra del Fuego, resembling the strictly arctic *C. stricta*, but with centrally proliferating scyphi and constantly containing atranorin; on acid soil near or above treeline; all Alpine records of this species are under *C. stricta* or *C. lepidota*. According to Ahti (see Nimis 2016) records from the Alps most likely refer to *C. trassii*. – **Au**: T, S, K. **Sw**: VS. **It**: Lomb, Piem.

***Cladonia turgida* Hoffm.**

L – Subs.: ter-sil – Alt.: 3–4 – Note: a mainly boreal-montane, circumpolar species found on acid soil in open habitats, with optimum near treeline; rare in the Alps. – **Au**: O, N. **Sw**: GR, VD, VS. **Fr**: Sav. **It**: Frl, Lomb, Piem, VA.

***Cladonia uliginosa* (Ahti) Ahti**

Syn.: *Cladonia gracilescens auct.*, *Cladonia lepidota var. gracilescens auct.*, Cladonia stricta (Nyl.) Nyl. var. uliginosa Ahti

L – Subs.: ter-sil, bry – Alt.: 4–6 – Note: a species with an evanescent primary thallus, scyphose podetia repeatedly proliferating from the centre, and a strongly melanotic medulla high up in living podetia; on wet, acid soil or on soil layers over siliceous rocks; the distribution is mainly subarctic-continental, and the records from the Alps need verification. – **Au**: K. **Sw**: BE, GR.

***Cladonia umbricola* Tønsberg & Ahti**

L – Subs.: ter-sil, xyl, cor – Alt.: 3–4 – Note: a species with red apothecia and pycnidia, resembling *C. polydactyla*, but podetia more greenish grey and smaller (to 2 cm tall), scyphi narrower (rarely more than 2 mm wide), covered by farinose soredia, and marginal proliferations lacking or short, with squamatic acid and therefore UV+ white; on decaying wood and at the base of trunks of conifers as well as on acidic soil in humid-shaded situations; widespread in Western Europe and North America but not common, in the study area so far only known from the Western Alps. – **Fr**: HSav.

**Cladonia uncialis (L.) F.H. Wigg. subsp. uncialis**

Syn.: *Cladonia stellata* Schaer., Cladonia uncialis (L.) F.H. Wigg. var. obtusata (Ach.) Räsänen, *Lichen uncialis* L.

L – Subs.: ter-cal, ter-sil, bry – Alt.: 2–5 – Note: an arctic-alpine to northern boreal-montane, circumpolar species found on soil and amongst mosses in very open habitats with a long snow cover, near or above treeline; widespread throughout the Alps. – **Au**: V, T, S, K, St, O, N. **Ge**: OB, Schw. **Sw**: BE, GR, LU, SZ, TI, UR, UW, VD, VS. **Fr**: HAl, Isè, Sav, HSav. **It**: Frl, Ven, TAA, Lomb, Piem, VA.

**Cladonia uncialis (L.) F.H. Wigg. subsp. biuncialis (Hoffm.) M. Choisy**

Syn.: *Cladonia biuncialis* Hoffm., Cladonia uncialis (L.) F.H. Wigg. var. dicraea (Ach.) Räsänen, Cladonia uncialis (L.) F.H. Wigg. var. turgescens (Delise) Fr., Cladonia uncialis (L.) F.H. Wigg. subsp. dicraea (Ach.) D. Hawksw.

L – Subs.: ter-sil, bry – Alt.: 3–5 – Note: a taxon with a mainly anisotomic-dichotomous branching and dichotomous terminal pointed branchlets, containing squamatic acid, genetically distinct from the typical subspecies; over acid rocky soils and in coastal heaths and bogs outside the Alps; in the Alps mainly in alpine heaths. – **Au**: V, T, S, K, St. **Ge**: Schw. **Sw**: SZ. **Fr**: HSav. **It**: Frl, Ven.

***Clauzadea chondrodes* (A. Massal.) Hafellner & Türk**

Syn.: *Biatora chondrodes* A. Massal., *Biatora cyclisca* A. Massal., *Clauzadea cyclisca* (A. Massal.) V. Wirth, *Lecidea chondrodes* (A. Massal.) Malbr., *Lecidea cyclisca* (A. Massal.) Malbr., *Lecidea savonensis* B. de Lesd., *Protoblastenia chondrodes* (A. Massal.) Zahlbr., *Protoblastenia cyclisca* (A. Massal.) Szatala

L – Subs.: cal – Alt.: 2–3 – Note: a mainly temperate lichen found on limestone and dolomite, on surfaces with short periods of water seepage after rain, often with colonies of cyanobacteria, but avoiding very dry situations. – **Au**: V, T, O, N. **Ge**: OB. **Fr**: AHP, AMa, Sav, HSav, Var, Vau. **It**: Frl, Ven, TAA, Lomb, Lig. **Sl**: Tg.

***Clauzadea immersa* (Hoffm.) Hafellner & Bellem.**

Syn.: *Biatora immersa* (Hoffm.) P. Syd., *Hymenelia immersa* (Hoffm.) Körb., *Lecidea calcivora* (Schaer.) A. Massal., *Lecidea immersa* (Hoffm.) Ach., Lecidea immersa (Hoffm.) Ach. var. calcivora Schaer., *Lecidella immersa* (Hoffm.) Körb., *Lichen immersus* Weber, *Protoblastenia immersa* (Hoffm.) J. Steiner, *Verrucaria immersa* Hoffm.

L – Subs.: cal – Alt.: 1–5 – Note: a temperate to southern boreal-montane lichen found on a wide variety of calciferous rocks, especially limestone, with a wide altitudinal range; widespread throughout the Alps. – **Au**: V, T, S, K, St, O, N. **Ge**: OB, Schw. **Sw**: BE, GR, LU, SZ, VD. **Fr**: AHP, HAl, AMa, Drô, Isè, Sav, HSav, Var, Vau. **It**: Frl, Ven, TAA, Lomb, Piem, VA, Lig. **Sl**: SlA, Tg.

***Clauzadea metzleri* (Körb.) Clauzade & Cl. Roux *ex* D. Hawksw.**

Syn.: *Biatora metzleri* Körb., *Biatora oolithina* (Nyl.) Arnold, *Lecidea metzleri* (Körb.) Th. Fr., *Lecidea oolithina* Nyl., *Protoblastenia metzleri* (Körb.) J. Steiner

L – Subs.: cal – Alt.: 1–4 – Note: a mainly temperate, holarctic early coloniser of small calcareous pebbles in dry grasslands; widespread throughout the Alps. – **Au**: V, T, S, O. **Ge**: OB, Schw. **Sw**: LU, SZ, VD. **Fr**: AHP, AMa, Isè, Sav, HSav, Var, Vau. **It**: Frl, Ven, TAA, Lomb.

***Clauzadea monticola* (Ach. *ex* Schaer.) Hafellner & Bellem.**

Syn.: *Biatora fuscorubens* Nyl., *Biatora monticola* (Ach. *ex* Schaer.) Hepp, *Biatora ochracea* Hepp *nom.illeg.*, *Lecidea caementicola* Erichsen, *Lecidea fuscorubens* (Nyl.) Nyl., *Lecidea monticola* Ach. *ex* Schaer., *Lecidea ochracea* (Arnold) Zwackh, *Lecidea rubigineoatra* Vain., *Lecidea subacervata* Müll. Arg., *Lecidea sympathetica* Taylor *ex* Leight., *Lecidella fuscorubens* (Nyl.) Stein, *Lecidella ochracea* Arnold, *Protoblastenia fuscorubens* (Nyl.) Räsänen, *Protoblastenia monticola* (Ach. *ex* Schaer.) J. Steiner, *Protoblastenia ochracea* (Arnold) Zahlbr., *Sarcogyne calcomaura* Norman

L – Subs.: cal – Alt.: 1–5 – Note: a holarctic pioneer species of calciferous rocks (limestone, dolomite, sandstone, calciferous schists), also found on man-made substrata (*e.g.* on mortar walls), and even on gypsum, with optimum below the montane belt; widespread throughout the Alps. – **Au**: V, T, S, K, St, O, N. **Ge**: OB, Schw. **Sw**: BE, GR, LU, SZ, TI, VD, VS. **Fr**: AHP, HAl, AMa, Drô, Isè, Sav, HSav, Var, Vau. **It**: Frl, Ven, TAA, Lomb, Piem, Lig. **Sl**: SlA, Tg. **Li**.

***Clauzadeana macula* (Taylor) Coppins & Rambold**

Syn.: *Aspicilia morioides* Blomb. *ex* Arnold, *Clauzadeana instratula* (Nyl.) Cl. Roux, *Lecanora morioides* (Blomb. *ex* Arnold) Blomb., *Lecidea instratula* Nyl., *Lecidea macula* Taylor, *Lecidea perustula* Nyl., *Lecidea pissodes* Stirt., *Psora pissodes* (Stirt.) Walt. Watson

L – Subs.: sil, sil-par – Alt.: 3–5 – Note: on hard, steeply inclined crystalline siliceous rocks; probably overlooked in the Alps, but certainly not common. – **Au**: V, T, K, St. **It**: TAA, Piem.

***Clavascidium imitans* (Breuss) M. Prieto**

Syn.: *Catapyrenium imitans* Breuss, *Placidium imitans* (Breuss) Breuss

L – Subs.: ter-cal – Alt.: 3 – Note: similar to *C. lacinulatum*, but pycnoconidia rod-shaped; on soil in steppe-like vegetation; based on a type from Mongolia and apparently widespread in Asia, with a single record from the montane belt of the Western Alps (France), on gypsaceous soil. – **Fr**: Sav.

**Clavascidium lacinulatum (Ach.) M. Prieto var. lacinulatum**

Syn.: *Catapyrenium lacinulatum* (Ach.) Breuss, Endocarpon hepaticum Ach. var. lacinulatum Ach., *Placidiopsis grappae* Beltr., *Placidium lacinulatum* (Ach.) Breuss, Placidium rufescens (Ach.) A. Massal. var. trapeziforme A. Massal.

L – Subs.: ter-cal – Alt.: 1–5 – Note: a mainly Mediterranean-Atlantic to mild-temperate terricolous species found on loess and calciferous ground, most frequent in dry grasslands at relatively low elevations; apparently more frequent in the Western and Southern Alps. See also Nimis (1993: 545). – **Sw**: GR, VS. **Fr**: AMa, Var, Vau. **It**: Ven, TAA.

**Clavascidium lacinulatum (Ach.) M. Prieto var. atrans (Breuss) M. Prieto**

Syn.: Placidium lacinulatum (Ach.) Breuss var. atrans Breuss

L # – Subs.: ter-cal – Alt.: 3 – Note: a variety with dark perithecial walls, based on a type from Eastern North America, where it grows on soil over ophiolitic rocks; in the Alps only known from a single locality over calcareous soil; on the whole an insufficiently known taxon in need of critical re-evaluation. – **Sw**: VS.

***Clavascidium umbrinum* (Breuss) Breuss**

Syn.: *Catapyrenium umbrinum* Breuss

L – Subs.: ter-cal – Alt.: 1–2 – Note: on calciferous, clayey soil; only known from Dalmatia, France and Piemonte, this species is worthy of further study. – **It**: Piem.

***Cliostomum corrugatum* (Ach.) Fr.**

Syn.: *Biatora ehrhartiana* (Ach.) W. Mann, *Biatorina ehrhartiana* (Ach.) Mudd, *Biatorina graniformis* (K.G. Hagen) A.L. Sm., *Buellia cliostomoides* A. Massal., *Catillaria ehrhartiana* (Ach.) Th. Fr., *Catillaria graniformis* (K.G. Hagen) Vain., *Cliostomum graniforme* (K.G. Hagen) Coppins, *Lecanora ehrhartiana* (Ach.) Fr., *Lecidea corrugata* Ach., *Lecidea ehrhartiana* (Ach.) Ach., *Limboria corrugata* (Ach.) Ach., *Rhytisma corrugatum* (Ach.) Fr.

L – Subs.: cor, xyl – Alt.: 2–3 – Note: a mainly cool-temperate species found on old oaks, but also on *Abies* in humid stands, more rarely on lignum (decorticated trunks, wooden poles); widespread throughout the Alps, but generally rare. – **Au**: T, S, St, N. **Ge**: OB. **Sw**: BE, GR, SZ, TI, UR, UW, VS. **Fr**: AMa. **It**: Ven, TAA, Lomb. **Sl**: SlA.

***Cliostomum flavidulum* Hafellner & Kalb**

Syn.: *Lecanora navarrensis* Etayo

L – Subs.: cor – Alt.: 3 – Note: a species with a pale yellowish, sorediate thallus, occasionally fertile with biatorine, often pruinose apothecia, and 1-septate ascospores; on both broad-leaved and coniferous trees in forests and open woodlands in areas with an oceanic climate; widespread in Western Europe, from the Alps there are so far a few records along the northern rim. – **Sw**: LU, UW.

***Cliostomum griffithii* (Sm.) Coppins**

Syn.: *Bacidia imitatrix* Malme, *Biatora mixta* Fr., *Biatorina griffithii* (Sm.) A. Massal., *Biatorina mixta* (Fr.) Hellb., *Biatorina tricolor auct.*, *Catillaria griffithii* (Sm.) Malme, *Catillaria tricolor auct. non* (With.) Th. Fr., *Lichen griffithii* Sm.

L – Subs.: cor – Alt.: 1–3 – Note: a mild-temperate species with a fragmented holarctic range, found on bark of old isolated trees in open, humid woodlands, rarely on lignum; widespread in the Alps, but generally rare. – **Au**: V, T, O, N. **Fr**: AMa, Isè, Var. **It**: Frl, Ven, Lomb.

***Cliostomum haematommatis* (Keissl.) D. Hawksw., Earl.-Benn. & Coppins**

Syn.: *Lichenophoma haematommatis* Keissl.

L – Subs.: cor – Alt.: 3 – Note: a species with a whitish grey to bluish grey, sorediate to leprose thallus, ascomata unknown, but conspicuous, black, semi-immersed pycnidia with elongate cylindrical conidiogenous cells and narrowly ellipsoid conidia usually present; on bark of trees in areas with a humid climate; so far only known from the Eastern Alps (Austria). – **Au**: St.

***Cliostomum leprosum* (Räsänen) Holien & Tønsberg**

Syn.: *Catillaria leprosa* Räsänen

L # – Subs.: cor – Alt.: 3 – Note: a species with a whitish, abundantly sorediate thallus, biatorine apothecia (occasionally present) with yellowish discs, and conspicuous black pycnidia (always present); the difference from *C. haematommatis* is in need of reevaluation; on bark of conifers in old-growth forests; widespread in the Holarctic region; in the Alps so far only a few records along the northern rim. – **Sw**: LU, UW.

***Cliostomum pallens* (Kullh.) S. Ekman**

Syn.: *Bilimbia pallens* Kullh.

L – Subs.: cor – Alt.: 3–4 – Note: a species with an esorediate thallus, pale yellow, minute biatorine apothecia, 3-septate, bacilliform ascospores, and inconspicuous pycnidia; on bark of broad-leaved and coniferous trees, widespread in the Holarctic region especially in the boreal zone, with a few records from the Alps. – **Au**: K. **Sw**: GR, TI.

***Coenogonium luteum* (Dicks.) Kalb & Lücking**

Syn.: *Biatorina lutea* (Dicks.) Körb., *Dimerella lutea* (Dicks.) Trevis., *Gyalecta lutea* (Dicks.) Hornem., *Lecidea lutea* (Dicks.) Taylor, *Lichen luteus* Dicks., *Microphiale lutea* (Dicks.) Zahlbr.

L – Subs.: cor, bry – Alt.: 1–3 – Note: a mild-temperate to humid subtropical lichen found on bark and epiphytic liverworts in semi-natural, old, humid forests; widespread but rare throughout the Alps. – **Au**: V, S, K, St, O, N. **Ge**: OB, Schw. **Sw**: BE, SZ, TI, UR, UW. **Fr**: AMa, Var. **It**: Ven, Lomb, Piem. **Sl**: SlA, Tg.

***Coenogonium pineti* (Ach.) Lücking & Lumbsch**

Syn.: *Belonium piceae* Henn., *Biatora pineti* (Ach.) Fr., *Biatorina diluta* (Pers.) Th. Fr., *Biatorina pineti* (Ach.) A. Massal., *Biatorinopsis diluta* (Pers.) Müll. Arg., *Dimerella diluta* (Pers.) Trevis., *Dimerella pineti* (Ach.) Vězda, *Gyalecta alnicola* B. de Lesd., *Gyalecta diluta* (Pers.) Blomb. & Forssell, *Gyalecta pineti* (Ach.) Tuck., *Gyalecta rosea* (Eitner) Zahlbr., *Lecidea pineti* Ach., *Microphiale diluta* (Pers.) Zahlbr., *Peziza diluta* Pers.

L – Subs.: cor, xyl, deb, bry – Alt.: 1–3 – Note: a probably holarctic lichen, most common on acid bark, both of conifers and of broad-leaved trees, below the subalpine belt; widespread throughout the Alps, especially in non-heavily disturbed semi-natural areas with a humid climate. – **Au**: V, T, S, K, St, O, N, B. **Ge**: OB, Schw. **Sw**: BE, FR, GL, GR, LU, SZ, TI, UR, UW, VS. **Fr**: AHP, HAl, AMa, Isè, HSav, Var, Vau. **It**: Frl, Ven, TAA, Lomb, Piem, Lig. **Sl**: SlA, Tg.

***Coenogonium tavaresianum* (Vězda) Lücking, Aptroot & Sipman**

Syn.: *Dimerella tavaresiana* Vězda

L – Subs.: cor – Alt.: 1–2 – Note: a mild-temperate lichen found on acid bark of conifers and broad-leaved trees in open, humid and warm woodlands, reported only from the base of the Western Alps. – **Fr**: AMa.

***Collema flaccidum* (Ach.) Ach.**

Syn.: *Collema rupestre* (Sw.) Rabenh., *Lathagrium rupestre* (Sw.) A. Massal., *Lichen flaccidus* Ach., *Lichen rupestris* Sw. *nom.illeg.*, *Parmelia flaccida* (Ach.) Ach., *Synechoblastus flaccidus* (Ach.) Körb., *Synechoblastus rupestris* (Sw.) Trevis.

L – Subs.: cor, bry, sil, int – Alt.: 1–3 – Note: a mainly temperate to southern boreal-montane lichen with a fragmented holarctic range, found on bark, epilithic mosses, base-rich siliceous and slightly calciferous rocks in sheltered, humid situations; widespread throughout the Alps. – **Au**: V, T, S, K, St, O, N, B. **Ge**: OB, Schw. **Sw**: BE, FR, GL, GR, LU, SG, SZ, TI, UR, UW, VD, VS. **Fr**: AHP, HAl, AMa, Drô, Isè, Sav, HSav, Var, Vau. **It**: Frl, Ven, TAA, Lomb, Piem, VA, Lig. **Sl**: SlA, Tg. **Li**.

***Collema furfuraceum* (Arnold) Du Rietz**

Syn.: Collema nigrescens (Huds.) DC. var. furfuraceum (Arnold) H. Olivier, Synechoblastus nigrescens (Huds.) Trevis. var. furfuraceum Arnold

L – Subs.: cor – Alt.: 1–3 – Note: a mainly temperate, probably holarctic lichen found on bark of broad-leaved trees and on epiphytic mosses, more common in the past, presently confined to semi-natural, open lowland forests. – **Au**: T, K, O. **Ge**: OB. **Sw**: GR, TI, VS. **Fr**: AHP, HAl, AMa, Drô, Isè, Sav, HSav, Var, Vau. **It**: Ven, TAA, Lomb, Lig. **Sl**: SlA.

***Collema glebulentum* (Nyl. *ex* Cromb.) Degel.**

Syn.: *Collema coralliferum* Degel., *Collema furvellum* Räsänen, *Leptogium glebulentum* Nyl. *ex* Cromb.

L – Subs.: sil, ter-sil – Alt.: 3–5 – Note: on basic siliceous rocks in humid situations; perhaps somehow more widespread throughout the Alps, but often confused with other species. – **Au**: V, T, S, St. **Sw**: BE, VS. **It**: Lomb.

***Collema nigrescens* (Huds.) DC.**

Syn.: *Collema vespertilio* (Lightf.) Hoffm., *Lathagrium nigrescens* (Huds.) Gray, *Lichen nigrescens* Huds., *Lichen verspertilio* Lightf., *Synechoblastus nigrescens* (Huds.) Trevis., *Synechoblastus vespertilio* (Lightf.) Hepp

L – Subs.: cor – Alt.: 1–3 – Note: a mainly temperate species found on more or less isolated trees (depending on air humidity); more common in the past, presently absent from urban areas but locally still frequent in humid, semi-natural habitats, more wide-ranging in altitude and latitude than the closely related *C. subnigrescens*; widespread in the Alps, but only locally common. – **Au**: V, T, S, K, St, O, N. **Ge**: OB, Schw. **Sw**: BE, GL, GR, SZ, TI, UR, VD, VS. **Fr**: AHP, HAl, AMa, Isè, Sav, HSav, Var, Vau. **It**: Frl, Ven, TAA, Lomb, Piem, VA, Lig. **Sl**: SlA, Tg.

***Collema ryssoleum* (Tuck.) A. Schneid.**

Syn.: *Collema meridionale* Hue, Collema nigrescens (Huds.) DC. subsp. ryssoleum Tuck.

L – Subs.: sil – Alt.: 1–2 – Note: a mild-temperate lichen found on steeply inclined seepage tracks of basic siliceous rocks; a mainly western species in Europe, also reported from the base of the Western Alps. – **Fr**: AMa, Var. **It**: Lig.

***Collema subflaccidum* Degel.**

Syn.: *Collema subfurvum auct*.

L – Subs.: cor – Alt.: 1–3 – Note: a mainly temperate, incompletely holarctic species found on more or less isolated broad-leaved trees in humid-rainy areas; more common in the past, presently absent from urban areas and most common along the Southern and Western Alps. – **Au**: St, N. **Sw**: TI. **Fr**: AHP, AMa, Drô, Isè, Sav, HSav, Var, Vau. **It**: Frl, Ven, TAA, Lomb, Piem, Lig. **Sl**: SlA.

***Collema subnigrescens* Degel.**

Syn.: Collema nigrescens (Huds.) DC. var. caesium (Clemente) Colmeiro, Collema nigrescens (Huds.) DC. var. subnigrescens (Degel.) Pišút, Collema subnigrescens Degel. var. caesium (Clemente) Degel., Parmelia nigrescens (Huds.) Ach. var. caesia Clemente, Synechoblastus nigrescens (Huds.) Trevis. f. caesium (Clemente) Hue

L – Subs.: cor – Alt.: 1–3 – Note: a mild-temperate lichen found on the bark of more or less isolated broad-leaved trees; more thermophytic than the closely related *C. nigrescens*; apparently more frequent in the Southern and Western Alps. – **Sw**: TI, UW. **Fr**: AHP, AMa, Isè, HSav, Var, Vau. **It**: Frl, Ven, Piem.

***Collemopsidium algovicum* (Servít) ined. (provisionally placed here, ICN Art. 36.1b)**

Syn.: *Arthopyrenia algovica* (Servít) Riedl, *Paraphysothele algovica* Servít, *Thelidium algovicum* (Servít) P. Scholz *comb. inval*.

L # – Subs.: int-aqu – Alt.: 3 – Note: a poorly known species with a thin, olive-green, subgranular thallus forming patches to 1.5 cm in diam., ascomata to *c.* 0.25 mm in diam., a hemispherical involucrellum spreading and covered by a thin thalline layer, the perithecial wall dark (to *c.* 150 µm in diam.), persisting and poorly ramified interascal filaments, and 1-septate ascospores (to *c.* 20 µm long) with a somewhat wider upper cell; on temporarily submerged calcareous schists, only known from the type locality in the Eastern Alps (Germany). – **Ge**: Schw.

***Collemopsidium angermannicum* (Degel.) A. Nordin**

Syn.: *Arthopyrenia angermannica* Degel., *Arthopyrenia strontianensis* Swinscow, *Pyrenocollema strontianense* (Swinscow) R.C. Harris

L – Subs.: sil – Alt.: 2–3 – Note: a species with a thin, episubstratic, smooth to rimose, olive-brown to dark brown thallus with a cyanobacterial photobiont (developing globose cells), scattered, black perithecioid ascomata, fissitunicate asci, 1-septate ascospores, and richly branched hamathecial elements; on often submerged siliceous rocks along streams and margins of lakes; widespread in the Holarctic region, but not common; also recorded from the Western Alps in sites with a untypical ecology, and therefore in need of confirmation. – **Fr**: AMa, Var.

***Collemopsidium argilospilum* (Nyl.) Coppins & Aptroot**

Syn.: *Pyrenocollema argilospilum* (Nyl.) Coppins, *Verrucaria argilospila* Nyl.

L – Subs.: ter-sil – Alt.: 3 – Note: a species with an olive-black, thin thallus becoming somewhat gelatinous when wet (with a cyanobacterial photobiont), based on a type from Finland; on moist sandy or clayey soil in inland habitats; a rare taxon with a few scattered records in Europe, including a single record from the Alps, along the edge of a forest road; ecology otherwise poorly known. – **Au**: K.

***Collemopsidium caesium* (Nyl.) Coppins & Aptroot**

Syn.: *Arthopyrenia caesia* (Nyl.) Zahlbr., *Arthopyrenia nylanderi* (Hepp) Riedl, *Leiophloea caesia* (Nyl.) Trevis., *Leiophloea nylanderi* (Hepp) Trevis., *Pseudarthopyrenia caesia* (Nyl.) Keissl., *Pyrenocollema caesium* (Nyl.) R.C. Harris, *Pyrenocollema tichothecioides* (Arnold) R.C. Harris, *Sagedia nylanderi* Hepp, *Verrucaria caesia* Nyl.

L – Subs.: cal – Alt.: 3–5 – Note: a species with a partly endolithic, whitish to bluish grey thallus (forming dark brown, thin patches on non-calcareous rocks only), semi-immersed perithecioid ascomata containing anastomosing interascal filaments, 8-spored asci, and 1-septate ascospores with an attenuated lower end; the generic placement and the synonymy of *Verrucaria caesia* (type from the western Mediterranean near the sea, ascospores exceeding 25 µm in length) and *Arthopyrenia tichothecioides* (type from the Northern Alps in the montane belt, ascospores shorter than 25 µm) are in need of re-evaluation, as is the ecology (perhaps a lichenicolous species); on boulders and steeply inclined to vertical surfaces of permanently damp or moist limestones or base-rich siliceous rocks; widespread in the Holarctic region, but not common; in the Alps ranging from mid – to high elevations. – **Au**: T, S, St, O. **Ge**: OB. **Fr**: AHP, AMa, Sav, HSav, Var. **It**: TAA.

***Collemopsidium minutulum* (Bornet) ined. (provisionally placed here, ICN Art. 36.1b)**

Syn.: *Arnoldia minutula* Bornet, *Lempholemma minutulum* (Bornet) Zahlbr., *Pyrenocollema minutulum* (Bornet) Puym.

L # – Subs.: ter – Alt.: 2 – Note: a species with a thallus of black globules (to 0.5 mm in diam.), a *Nostoc*-like photobiont, immersed ascomata, 8-spored asci embedded in an amyloid hymenial gel, and ellipsoid, simple ascospores; on soil *e.g.* along secondary dirt roads; easy to be overlooked and distribution therefore poorly documented, with a few records from the Western Alps. – **Sw**: VS. **Fr**: AMa.

***Coniocarpon cinnabarinum* DC.**

Syn.: *Arthonia cinnabarina* (DC.) Wallr., *Arthonia gregaria* (Weigel) Körb. *non* Fée, *Arthonia tumidula* (Ach.) Ach., *Coniocarpon gregarium* (Weigel) Schaer., *Sphaeria gregaria* Weigel, *Spiloma tumidulum* Ach.

L – Subs.: cor – Alt.: 1–3 – Note: a mild-temperate, perhaps holarctic species found on *Fraxinus*, but also on trees with harder and more acid bark, such as *Carpinus*, *Fagus* and even *Quercus ilex*, in open, humid woodlands, *e.g.* along rivers; widespread in the Alps, but much more frequent in the past. – **Au**: V, T, S, K, St, O, N. **Ge**: OB, Schw. **Sw**: BE, TI, VD. **Fr**: AMa, Drô, Isè, Sav, HSav, Var, Vau. **It**: Frl, Ven, TAA, Lomb, Piem. **Sl**: SlA.

***Coniocarpon elegans* (Ach.) Duby**

Syn.: *Arthonia elegans* (Ach.) Almq., *Arthonia ochracea* Dufour, *Coniocarpon ochraceum* (Dufour) Fr., *Spiloma elegans* Ach.

L – Subs.: cor – Alt.: 2–3 – Note: a mild-temperate lichen found on smooth bark, *e.g.* of *Corylus*, in humid woodlands, such as along rivers, often with *Pseudoschismatomma rufescens*. – **Au**: S, N. **It**: Ven, Lomb, Piem. **Sl**: SlA, Tg.

***Cornicularia normoerica* (Gunnerus) Du Rietz**

Syn.: *Alectoria tristis* (Weber) Th. Fr., *Cetraria normoerica* (Gunnerus) Lynge, *Cetraria tristis* (Weber) Fr., *Cornicularia tristis* (Weber) Ach., *Imbricaria tristis* (Weber) Anzi, *Lichen normoericus* Gunnerus, *Parmelia tristis* (Weber) Spreng., *Platysma triste* (Weber) Nyl.

L – Subs.: sil – Alt.: 3–6 – Note: a circumpolar, arctic-alpine lichen found on hard, wind-exposed siliceous rocks, with optimum above treeline; widespread throughout the siliceous Alps. – **Au**: V, T, S, K, St, N. **Ge**: Schw. **Sw**: BE, GR, LU, TI, UR, VS. **Fr**: AHP, HAl, AMa, Isè, Sav, HSav. **It**: Frl, Ven, TAA, Lomb, Piem, VA, Lig.

***Crespoa crozalsiana* (Harm.) Lendemer & B.P. Hodk.**

Syn.: ?*Canoparmelia carneopruinata* (Zahlbr.) Elix & Hale, *Canoparmelia crozalsiana* (B. de Lesd. *ex* Harm.) Elix & Hale, ?*Parmelia carneopruinata* Zahlbr., *Parmelia crozalsiana* B. de Lesd. *ex* Harm., ?*Parmelia sbarbaronis* B. de Lesd., ?*Parmotrema carneopruinatum* (Zahlbr.) D. Hawksw., *Parmotrema crozalsianum* (B. de Lesd. *ex* Harm.) D. Hawksw., ?*Pseudoparmelia carneopruinata* (Zahlbr.) Hale, *Pseudoparmelia crozalsiana* (B. de Lesd. *ex* Harm.) Hale

L – Subs.: cor – Alt.: 1–2 – Note: a mild-temperate species with subtropical affinities, also reported from North America, locally abundant only – and strangely – at the base of the Western Alps (especially in *Olea*-plantations); *C. carnopruinata*, known from Liguria, is doubtfully distinct. – **Fr**: Var. **It**: Lig.

***Cresponea premnea* (Ach.) Egea & Torrente**

Syn.: Cresponea premnea (Ach.) Egea & Torrente var. saxicola (Leight.) Egea & Torrente, *Lecanactis plocina* (Ach.) A. Massal., *Lecanactis premnea* (Ach.) Arnold, Lecanactis premnea (Ach.) Arnold var. saxicola (Leight.) H. Olivier, *Lecidea premnea* Ach., Lecidea premnea Ach. var. saxicola Leight.

L – Subs.: sil, cor – Alt.: 1–3 – Note: a mild-temperate lichen found on siliceous rocks and on the bark of old deciduous trees (mainly oaks) in rain-protected faces, in very open, humid, park-like woodlands. – **Au**: S. **Fr**: Var. **It**: Ven, TAA, Lomb, Piem.

***Cryptodiscus gloeocapsa* (Nitschke *ex* Arnold) Baloch, Gilenstam & Wedin**

Syn.: *Bryophagus gloeocapsa* Nitschke *ex* Arnold, *Gloeolecta bryophaga* (Körb. *ex* Arnold) Vězda, *Gloeolecta gloeocapsa* (Nitschke *ex* Arnold) Lettau, *Gyalecta gloeocapsa* (Nitschke *ex* Arnold) Zahlbr.

L – Subs.: ter-sil, bry, deb – Alt.: 2–5 – Note: encrusting leafy hepatics or bryophytes, usually over sandy to clayey soils, mostly in shaded situations, *e.g.* along forest roads; widespread in the Holarctic region, in the Alps probably still regionally overlooked. – **Au**: V, T, S, K, St, N. **Sw**: SZ.

***Cryptolechia carneolutea* (Turner) A. Massal.**

Syn.: *Gyalecta carneolutea* (Turner) H. Olivier, *Gyalectina carneolutea* (Turner) Vězda, *Pachyphiale carneolutea* (Turner) Samp., *Parmelia carneolutea* Turner, *Pertusaria carneolutea* (Turner) Anzi, *Pertusaria protuberans* Th. Fr. *nom.illeg*.

L – Subs.: cor – Alt.: 1–2 – Note: a mild-temperate lichen with subtropical affinities found on nutrient-rich bark in very humid situations; perhaps extinct in the Insubrian district of Italy. – **It**: Lomb.

***Cryptothele rhodosticta* (Taylor) Henssen**

Syn.: *Pyrenopsis rhodosticta* (Taylor) Müll. Arg., *Verrucaria rhodosticta* Taylor

L – Subs.: int – Alt.: 3–5 – Note: a species with a crustose, areolate, dark reddish brown thallus and immersed perithecioid ascomata with an amyloid hymenial gel and cylindrical, thin-walled asci with attenuated apices containing simple, ellipsoid ascospores; on periodically submerged siliceous rocks near lakes and streams; rare in (North)Western Europe; records from the Alps have an atypical ecology and therefore need verification. – **Au**: ?V, T. **It**: ?TAA, ?Lomb, ?VA.

***Cypheliopsis mediterranea* (B. de Lesd.) Nádv.**

Syn.: *Cyphelium mediterraneum* B. de Lesd.

L # – Subs.: sil – Alt.: 2 – Note: a cyphelioid species with a grey, areolate thallus and immersed apothecia containing cylindrical asci giving rise to a mazaedium mainly consisting of dark brown, non-septate, spherical ascospores; on siliceous rocks not far from the Mediterranean Sea; only known from the type locality at the base of the Western Alps. – **Fr**: Var.

***Cystocoleus ebeneus* (Dillwyn) Thwaites**

Syn.: *Coenogonium ebeneum* (Dillwyn) A.L. Sm., *Coenogonium germanicum* Glück, *Coenogonium nigrum auct. non* (Huds.) Zahlbr., *Conferva ebenea* Dillwyn, *Cystocoleus niger auct. non* (Huds.) Har.

L – Subs.: sil, bry – Alt.: 2–5 – Note: a cool-temperate to boreal-montane, probably holarctic lichen found on vertical to underhanging surfaces of siliceous rocks protected from rain in very humid situations, more rarely on soil. The species often grows with *Racodium rupestre*, forming black, felt-like patches over extensive areas of rock; the most commonly associated lichens are species of *Lepraria*; widespread throughout the Alps. – **Au**: V, T, S, K, St. **Ge**: OB, Schw. **Sw**: GR, SZ, UW, VS. **Fr**: HAl, AMa, Isè. **It**: Frl, Ven, TAA, Lomb, Piem, VA, Lig. **Sl**: SlA.

***Dactylina ramulosa* (Hook.) Tuck.**

Syn.: *Dufourea muricata* Laurer, *Dufourea ramulosa* Hook.

L – Subs.: ter-cal, deb – Alt.: 4–6 – Note: an arctic-alpine, circumpolar species found on soil developing from calcareous schists, mostly above treeline; widespread in the Alps, but generally rare. – **Au**: V, T, S, K, St, O. **Ge**: OB. **Sw**: GR, UR, VS. **It**: TAA, Lomb, VA.

***Dendriscosticta wrightii* (Tuck.) B. Moncada & Lücking**

Syn.: *Sticta wrightii* Tuck.

L – Subs.: cor – Alt.: 3 – Note: this is the type species of a recent generic segregation of *Sticta* belonging to the Lobariaceae, close to *Ricasolia* (*Lobaria amplissima*-group), with a large thallus, whitish-grey on the lower side, bearing cyphelloid structures lacking an overarching rim, usually fertile; on bark of deciduous trees (especially *Acer pseudoplatanus*) along the northern slopes of the Alps under oceanic climatic conditions, extremely rare or regionally extinct: all records are historical. – **Au**: S. **Ge**: OB.

***Dendrographa decolorans* (Turner & Borrer *ex* Sm.) Ertz & Tehler**

Syn.: *Arthonia decolorans* (Turner & Borrer *ex* Sm.) Erichsen, *Lepraria decolorans* (Turner & Borrer *ex* Sm.) Almb., *Opegrapha albocincta* Nyl., *Opegrapha pitardii* B. de Lesd., *Schismatomma albocinctum* (Nyl.) Zahlbr., *Schismatomma decolorans* (Turner & Borrer *ex* Sm.) Clauzade & Vězda, *Schismatomma pitardii* (B. de Lesd.) Torrente & Egea, *Spiloma decolorans* Turner & Borrer *ex* Sm.

L – Subs.: cor – Alt.: 1–2 – Note: a mild-temperate, mostly western species found on ancient trees, with a few records from the Southern and Western Alps. – **Fr**: AMa, Var. **It**: Frl, Lig.

***Dendrographa latebrarum* (Ach.) Ertz & Tehler**

Syn.: *Crocynia albissima* B. de Lesd., *Crocynia fragilissima* Hue, *Crocynia hueana* B. de Lesd., *Crocynia latebrarum* (Ach.) Vain., *Lecanactis latebrarum* (Ach.) Arnold, *Lepraria latebrarum* (Ach.) Sm., *Lichen latebrarum* Ach.

L – Subs.: sil, cor – Alt.: 2–4 – Note: a mainly temperate species found beneath underhangs and in crevices of siliceous rocks which are seldom wetted by rain, much more rarely on old trunks of *Quercus*; widespread in the Alps, but generally not very common. – **Au**: V, T, S, St. **Sw**: GR, SZ, UR, VS. **Fr**: AMa, Isè, Sav, HSav, Var, Vau. **It**: Frl, TAA, Lomb.

***Dermatocarpon arnoldianum* Degel.**

L # – Subs.: sil-aqu – Alt.: 3–5 – Note: a rather poorly understood, but characteristic, perhaps holarctic species found on calciferous or base-rich siliceous rocks in periodically wet places, or near the ground. – **Au**: T, S, K, St. **Ge**: Ge. **Sw**: BE, GR, UR, VS. **It**: Frl, Ven, Lomb, Piem.

***Dermatocarpon complicatum* (Lightf.) W. Mann**

Syn. *Dermatocarpon decipiens auct. non* (A. Massal.) Dalla Torre & Sarnth., Dermatocarpon miniatum var. complicatum (Lightf.) Th. Fr.

L – Subs.: sil-aqu – Alt.: 2–5 – Note: a species of acid siliceous rocks, growing on periodically inundated surfaces. It is well distinct from *D. intestiniforme*, but it was frequently confused with it. *D. complicatum* seems to include most high-elevation records of the lichen hitherto called *D. decipiens* or D. miniatum var. decipiens by European authors, differing from *D. luridum* in the pruinose thallus and the non-amyloid medulla. From the original description by Massalongo, the true *D. decipiens* seems to be a synonym of *D. intestiniforme*. – **Au**: V, T, S, K, St. **Fr**: AHP, HAl, AMa, Isè, Sav, HSav, Var. **It**: Ven, TAA, Lomb, Piem.

***Dermatocarpon intestiniforme* (Körb.) Hasse**

Syn.: *Dermatocarpon polyphyllum* (Wulfen) Dalla Torre & Sarnth., *Endocarpon intestiniforme* Körb.

L – Subs.: cal, int – Alt.: 3–5 – Note: a mainly boreal-montane to arctic-alpine, circumpolar lichen found on sunny rock surfaces in periodically wet places; widespread throughout the Alps. Several records could refer to *D. complicatum* (see note on that species) – **Au**: V, T, S, K, St, O, N. **Ge**: OB. **Sw**: BE, GR, LU, SZ, UR, VS. **Fr**: AHP, HAl, AMa, Drô, Isè, Sav, HSav, Vau. **It**: Frl, Ven, TAA, Piem, VA. **Sl**: SlA.

***Dermatocarpon leptophyllodes* (Nyl.) Zahlbr.**

Syn.: *Dermatocarpon diffractum* (Th. Fr.) Blomb. & Forssell, *Dermatocarpon lorenzianum* Anders, Dermatocarpon miniatum (L.) W. Mann var. diffractum Th. Fr., *Endocarpon leptophyllodes* Nyl.

L – Subs.: sil-aqu – Alt.: 3–5 – Note: a temperate to southern boreal-montane species found on periodically inundated surfaces of basic siliceous rocks. The species is not easily recognised as belonging to *Dermatocarpon*, the thallus consisting of tightly arranged squamiform lobes (but with the pseudoparenchymatic lower cortex which is typical for the genus); from the Alps there are only a few scattered records, but perhaps the speies has been overlooked. – **Fr**: Isè, Sav. **It**: VA.

***Dermatocarpon leptophyllum* (Ach.) K.G.W. Lång**

Syn.: *Lichen leptophyllus* Ach.

L – Subs.: cal – Alt.: 3–4 – Note: a species of the *D. miniatum*-group with umbilicate thalli usually provided with blackish grey, concave lobes, and subglobose, uniseriate ascospores in cylindrical asci, found on horizontal or depressed rock faces of calcareous rocks in seasonally wet places; from the Alps there are only a few scattered records, but the species might have been not always distinguished from *D. miniatum*. – **Au**: V, T, S, K, St, N. **Sw**: VS. **Fr**: AHP, HAl. **It**: Ven, TAA, Lomb.

***Dermatocarpon luridum* (With.) J.R. Laundon**

Syn.: *Biatora lurida* (With.) Fr., *Dermatocarpon aquaticum* Herre, *Dermatocarpon fluviatile* (Weber) Th. Fr., *Dermatocarpon weberi* (Ach.) W. Mann, *Endocarpon aquaticum* Chevall. *nom.illeg.*, *Endocarpon fluviatile* (Weber) DC., *Lichen luridus* With., *Schaereria lurida* (With.) Gyeln.

L – Subs.: sil-aqu, sil – Alt.: 2–5 – Note: a cool-temperate to subarctic-subalpine, probably circumpolar species found on periodically inundated siliceous rocks near creeks and brooks, or on steeply inclined, shaded faces with frequent water seepage. – **Au**: V, T, S, K, St, N. **Ge**: Schw. **Sw**: BE, GR, TI, UR, UW, VS. **Fr**: AHP, HAl, AMa, Sav, HSav. **It**: Lomb, Piem, VA, Lig.

***Dermatocarpon meiophyllizum* Vain.**

Syn.: Dermatocarpon bachmannii Anders var. inundatum Klem., *Dermatocarpon meiophyllum* Vain.

L – Subs.: sil-aqu – Alt.: 2–5 – Note: on periodically inundated siliceous rocks, especially in the splash zone of lake shores or along creeks, in seepage tracks on slightly sloping faces; from the Alps there are several scattered records. – **Au**: T, S, N. **Fr**: Sav. **It**: Ven, TAA, Lomb.

**Dermatocarpon miniatum (L.) W. Mann var. miniatum**

Syn.: Dermatocarpon miniatum (L.) W. Mann var. aetneum (Tornab.) Zahlbr., Dermatocarpon miniatum (L.) W. Mann var. imbricatum (A. Massal.) Dalla Torre & Sarnth., Dermatocarpon miniatum (L.) W. Mann var. umbilicatum (Schaer.) Vain., *Endocarpon miniatum* (L.) P. Gaertn., G. Mey. & Scherb., *Endocarpon miniatum* (L.) P. Gaertn., G. Mey. & Scherb. var. aetneum Tornab., *Endocarpon miniatum* (L.) P. Gaertn., G. Mey. & Scherb. var. imbricatum A. Massal., *Lichen miniatus* L.

L – Subs.: cal, int – Alt.: 1–5 – Note: on more or less calciferous and on basic siliceous rocks, from calcareous schists to limestone and dolomite, especially on steeply inclined to underhanging surfaces, and in rain-tracks, with a wide altitudinal range; widespread throughout the Alps. – **Au**: V, T, S, K, St, O, N. **Ge**: OB, Schw. **Sw**: BE, GR, LU, SG, SZ, TI, UR, UW, VD, VS. **Fr**: AHP, HAl, AMa, Drô, Isè, Sav, HSav, Var, Vau. **It**: Frl, Ven, TAA, Lomb, Piem, VA, Lig. **Sl**: SlA, Tg. **Li**.

**Dermatocarpon miniatum (L.) W. Mann var. cirsodes (Ach.) Zahlbr.**

Syn.: *Dermatocarpon caesium* Räsänen, *Endocarpon miniatum* (L.) P. Gaertn., G. Mey. & Scherb. var. cirsodes Ach.

L – Subs.: cal, int – Alt.: 2–3 – Note: a morph with thick thalli and a distinctly papillose lower surface; on calcareous or basic siliceous rocks, usually in long-time dry localities; distribution insufficiently known because regionally not distinguished. – **Au**: S, K, St, N. **Fr**: AHP, HAl, AMa.

***Dermatocarpon moulinsii* (Mont.) Zahlbr.**

Syn.: *Endocarpon miniatum* (L.) P. Gaertn., G. Mey. & Scherb. var. exasperatum A. Massal., *Endocarpon moulinsii* Mont.

L – Subs.: sil – Alt.: 3–5 – Note: a silicicolous, holarctic species of periodically wetted rocks, with several records from the Southern Alps only (Italy). – **It**: Frl, Ven, TAA, Piem, Lig.

***Dermatocarpon rivulorum* (Arnold) Dalla Torre & Sarnth.**

Syn.: *Endocarpon rivulorum* Arnold

L – Substr.: sil, sil-aqu – Alt.: 3–5 – Note: a widespread, cool-temperate to arctic-alpine, circumpolar species found on periodically submerged siliceous rocks, in seepage tracks or along small streams, often completely inundated during summer, also occurring in melt-water seepages below snow-beds and along lakeshores. – **Au**: V, T, S, K, St. **Ge**: Ge. **Sw**: BE, GR, TI, UR, VS. **Fr**: HAl, AMa, Sav, HSav. **It**: Frl, TAA, Lomb, Piem, VA.

***Dibaeis baeomyces* (L. f.) Rambold & Hertel**

Syn.: *Baeomyces roseus* Pers., *Dibaeis rosea* (Pers.) Clemente, *Lichen baeomyces* L. f.

L – Subs.: ter-sil – Alt.: 2–5 – Note: on humid, disturbed clay soil, often in *Calluna*-heaths; widespread throughout the Alps. – **Au**: V, T, S, K, St, O, N, B. **Ge**: OB, Schw. **Sw**: BE, GR, LU, SG, SZ, TI, UR, VD, VS. **Fr**: Isè, Sav, HSav. **It**: Frl, Ven, TAA, Lomb, Piem, VA. **Sl**: SlA, Tg.

***Dimelaena lichenicola* K. Knudsen, Sheard, Kocourk. & H. Mayrhofer**

L – Subs.: sil-par – Alt.: 2–3 – Note: a recently-described lichenicolous lichen growing on *D. oreina*; the record from the Italian Alps is the only one in Europe. – **It**: TAA.

***Dimelaena oreina* (Ach.) Norman**

Syn.: *Beltraminia oreina* (Ach.) Trevis., *Dimelaena griseoviridis* (H. Magn.) Vězda, *Lecanora mougeotioides* Nyl., *Lecanora oreina* (Ach.) Ach., Lecanora straminea Ach. var. oreina Ach., *Rinodina altissima* H. Magn., *Rinodina hueana* Vain. *non* (Harm.) Mig., *Rinodina mougeotioides* (Nyl.) Mong., *Rinodina oreina* (Ach.) A. Massal., Rinodina oreina (Ach.) A. Massal. var. mougeotioides (Nyl.) Zahlbr.

L – Subs.: sil – Alt.: 1–5 – Note: a widespread, holarctic species found on hard siliceous rocks, including quartz, in sunny-dry situations, often on steeply inclined faces, common only in dry-continental areas, from some parts of the Mediterranean coast to dry valleys of the Alps; the species is chemically variable. – **Au**: V, T, S, K, St, N. **Sw**: BE, GR, UR, VD, VS. **Fr**: AHP, HAl, AMa, HSav, Var, Vau. **It**: Frl, Ven, TAA, Lomb, Piem, VA, Lig.

***Diploicia canescens* (Dicks.) A. Massal.**

Syn.: *Buellia canescens* (Dicks.) De Not., *Catolechia canescens* (Dicks.) Anzi, *Diplotomma canescens* (Dicks.) Flot., *Lecidea canescens* (Dicks.) Ach., *Lichen canescens* Dicks., *Placodium canescens* (Dicks.) DC.; incl: Diploicia canescens (Dicks.) A. Massal. var. euthallina (Servít) ined. (provisionally placed here, ICN Art. 36.1b)

L – Subs.: cal, cor – Alt.: 1–2 – Note: a rather western and southern lichen in Europe, found on a wide variety of substrata including base-rich or eutrophicated bark, calciferous sandstone, and limestone, sometimes also found in underhangs of calcareous rocks protected from rain; rare in the Alps, somehow more frequent in the Western and Southern Alps. – **Au**: N, B. **Sw**: ?VS. **Fr**: AMa, Isè, HSav, Var, Vau. **It**: Ven, TAA, Lomb, Lig. **Sl**: SlA.

***Diploschistes actinostoma* (Ach.) Zahlbr.**

Syn.: *Acrorixis actinostoma* (Ach.) Trevis., *Diploschistes sbarbaronis* B. de Lesd., *Limboria actinostoma* (Ach.) A. Massal., *Urceolaria actinostoma* Pers *ex* Ach. *nom. inval.*, *Urceolaria actinostoma* (Ach.) Schaer., *Verrucaria actinostoma* Ach.

L – Subs.: sil – Alt.: 1–3 – Note: a mild-temperate lichen found on basic siliceous substrata, including roofing tiles, more rarely on porous, weakly calciferous rocks, exceptionally also on superficially decalcified limestones; most frequent and abundant south of the Alps. The specific epithet is usually misspelled as “*actinostomus*”, but it is a name, not an adjective, meaning “a mouth with rays”. – **Sw**: VS. **Fr**: AHP, AMa, Var, Vau. **It**: Ven, TAA, Lomb, Piem, VA, Lig.

***Diploschistes albescens* Lettau**

L # – Subs.: cal, ter-cal – Alt.: 2–5 – Note: this species, frequently considered as a synonym of *D. diacapsis*, differs in the tetrasporous asci and the thallus reacting strongly K+ yellow, then rapidly violet-red; it grows on gypsaceous or very porous calcareous rocks and soil in dry, sunny situations; so far, it has only been distinguished in the Western Alps (Haute-Vésubie, France) – **Fr**: AMa.

***Diploschistes candidissimus* (Kremp.) Zahlbr.**

Syn.: Acrorixis actinostoma (Ach.) Trevis. var. tectorum (A. Massal.) Trevis., Diploschistes actinostoma (Ach.) Zahlbr. var. farinosus (Anzi) Zahlbr., *Diploschistes calcareus* (Müll. Arg.) J. Steiner, *Diploschistes farinosus* (Anzi) Vězda, Limboria actinostoma (Ach.) A. Massal. var. farinosa Anzi, Limboria actinostoma (Ach.) A. Massal. var. tectorum A. Massal., *Limboria candidissima* Kremp., Urceolaria actinostoma (Ach.) Schaer. var. tectorum (A. Massal.) Jatta

L – Subs.: cal – Alt.: 2–3 – Note: a species with a whitish grey, pruinose thallus, entirely immersed apothecia with punctiform discs, 4 – to – 8-spored asci, and medium-sized muriform ascospores; on calcareous rocks; widespread in the Mediterranean region and other parts of the world with a similar climate (*e.g.* Southern Australia); in the Alps only known from some southern localities, such as dry valleys. – **Fr**: AHP, Var. **It**: Ven, TAA, Piem.

***Diploschistes diacapsis* (Ach.) Lumbsch**

Syn.: *Diploschistes albissimus* (Ach.) Dalla Torre & Sarnth., *Diploschistes gypsaceus auct. p.p.*, *Diploschistes steppicus* Reichert, *Urceolaria diacapsis* Ach., Urceolaria scruposa (Schreb.) Ach. var. diacapsis (Ach.) Schaer.

L – Subs.: ter-cal – Alt.: 1–3 – Note: a widespread species of arid grasslands found on calciferous or base-rich soil in open, dry situations. – **Sw**: ?BE, ?FR, ?GR, ?TI, ?VS. **Fr**: HAl, AMa, Drô, Vau. **It**: TAA, Lomb, Piem, Lig.

***Diploschistes euganeus* (A. Massal.) J. Steiner**

Syn.: *Diploschistes clausus* (Flot.) Zahlbr., *Limboria euganea* A. Massal., *Urceolaria euganea* (A. Massal.) Jatta

L – Subs.: sil – Alt.: 1–2 – Note: a mild-temperate lichen found on basic siliceous rocks, more rarely on brick and roofing tiles, in warm-humid areas, sometimes starting the life-cycle on *Ochrolechia parella*; most frequent in the Southern Alps, at low elevations. – **Sw**: TI. **It**: Ven, TAA, Lomb.

***Diploschistes gypsaceus* (Ach.) Zahlbr.**

Syn.: *Diploschistes cretaceus* (Ach.) Lettau, *Diploschistes ochrophanes* Lettau, Diploschistes scruposus (Schreb.) Norman subsp. cretaceus (Ach.) Clauzade & Cl. Roux, Diploschistes scruposus (Schreb.) Norman subsp. gypsaceus (Ach.) Clauzade & Cl. Roux, Diploschistes scruposus (Schreb.) Norman subsp. ochrophanes (Lettau) Clauzade & Cl. Roux, Diploschistes scruposus (Schreb.) Norman var. cretaceus (Ach.) Müll. Arg., *Gyalecta cretacea* Ach., Lecanora scruposa (Schreb.) Nyl. var. gypsacea (Ach.) Sommerf., *Urceolaria cretacea* (Ach.) Balb., *Urceolaria gypsacea* Ach., Urceolaria scruposa (Schreb.) Ach. var. cretacea (Ach.) Schaer., Urceolaria scruposa (Schreb.) Ach. var. gypsacea (Ach.) Körb.

L – Subs.: cal, int – Alt.: 1–5 – Note: a temperate to southern boreal-montane lichen found in rock fissures, on vertical or underhanging surfaces of calcareous rocks, often in woodlands, with a wide altitudinal range; widespread throughout the Alps. – **Au**: V, T, S, K, St, O, N. **Ge**: OB, Schw. **Sw**: BE, GR, LU, SZ, VS. **Fr**: AHP, HAl, AMa, Drô, Isè, Sav, HSav, Var, Vau. **It**: Frl, Ven, TAA, Lomb, Piem. **Sl**: SlA.

***Diploschistes muscorum* (Scop.) R. Sant.**

Syn.: *Diploschistes bryophiloides* (Nyl.) Zahlbr., *Diploschistes bryophilus* (Ehrh. *ex* Ach.) Zahlbr., *Diploschistes lichenicola* (Mont. & Fr.) Vain., Diploschistes scruposus (Schreb.) Norman subsp. muscorum (Scop.) Clauzade & Cl. Roux, Diploschistes scruposus (Schreb.) Norman var. arenarius (Ach.) Müll. Arg., Diploschistes scruposus (Schreb.) Norman var. bryophilus (Ehrh. *ex* Ach.) Müll. Arg., Diploschistes scruposus (Schreb.) Norman var. parasiticus (Sommerf.) Zahlbr., *Lichen impressus* Sw., *Lichen muscorum* Scop., *Melittiosporum lichenicola* (Mont. & Fr.) Massee, *Stictis lichenicola* Mont. & Fr., *Urceolaria bryophila* (Ehrh.) Funck, *Urceolaria bryophiloides* Nyl., Urceolaria scruposa (Schreb.) Ach. var. arenaria Schaer.

L – Subs.: par, bry, ter-cal – Alt.: 1–5 – Note: a holarctic lichen, often – but apparently not always – parasitic on *Cladonia* squamules (especially *C. pocillum* and *C. symphycarpa*, sometimes also on the podetia of *C. rangiformis*), generally on mosses and plant debris in dry grasslands on limestone, with a wide altitudinal range. Not always distinguished from *D. diacapsis* in the older literature and related to *D. scruposus*; widespread throughout the Alps. – **Au**: V, T, S, K, St, O, N, B. **Ge**: OB, Schw. **Sw**: BE, GL, GR, SZ, TI, UW, VS. **Fr**: AHP, AMa, Drô, Isè, Sav, HSav, Var, Vau. **It**: Frl, Ven, TAA, Lomb, Piem, VA, Lig. **Sl**: SlA, Tg.

***Diploschistes neutrophilus* (Clauzade & Cl. Roux) Fern.-Brime & Llimona**

Syn.: Diploschistes diacapsis (Ach.) Lumbsch subsp. neutrophilus (Clauzade & Cl. Roux) Clauzade & Cl. Roux, Diploschistes gypsaceus (Ach.) Zahlbr. subsp. neutrophilus Clauzade & Cl. Roux

L # – Subs.: ter-sil – Alt.: 2 – Note: this taxon was originally segregated from *D. gypsaceus* on account of its different ecology (it grows on neutral sandy to clay soil) and the amyloid reaction of the medulla, a character which is not always evident; it grows on subneutral sandy soils in the western Mediterranean region, including the base of the SW Alps. – **Fr**: Vau.

***Diploschistes scruposus* (Schreb.) Norman**

Syn.: Diploschistes diacapsis (Ach.) Lumbsch subsp. interpediens (Nyl.) Cl. Roux, *Diploschistes interpediens* (Nyl.) Zahlbr., Diploschistes scruposus (Schreb.) Norman subsp. interpediens (Nyl.) Clauzade & Cl. Roux, Diploschistes scruposus (Schreb.) Norman subsp. iridatus (A. Massal.) Clauzade & Cl. Roux, Diploschistes scruposus (Schreb.) Norman subsp. violarius (Nyl.) Clauzade & Cl. Roux, *Diploschistes scruposus* (Schreb.) Norman var. clauzadei B. de Lesd., *Diploschistes violarius* (Nyl.) Zahlbr., *Lichen scruposus* Schreb., *Urceolaria scruposa* (Schreb.) Ach., *Urceolaria violaria* (Nyl.) Nyl.

L – Subs.: sil, ter-sil – Alt.: 1–5 – Note: a widespread holarctic lichen found on siliceous rocks, more rarely on soil, with a wide altitudinal range. Formerly frequently confused with similar species. The species, in its present circumscription, seems to be heterogeneous; widespread throughout the Alps. – **Au**: V, T, S, K, St, O, N, B. **Ge**: OB, Schw. **Sw**: BE, FR, GL, GR, LU, SZ, TI, VS. **Fr**: AHP, HAl, AMa, Drô, Isè, Sav, HSav, Var, Vau. **It**: Frl, Ven, TAA, Lomb, Piem, VA, Lig. **Sl**: SlA. **Li**.

***Diplotomma alboatrum* (Hoffm.) Flot.**

Syn.: *Abacina alboatra* (Hoffm.) Norman, *Buellia alboatra* (Hoffm.) Th. Fr., Buellia alboatra (Hoffm.) Th. Fr. var. ambigua (Ach.) Th. Fr., Buellia alboatra (Hoffm.) Th. Fr. var. subochracea Zahlbr., Buellia alboatra (Hoffm.) Th. Fr. var. vulgata Th. Fr., *Buellia ambigua* (Ach.) Malme, *Buellia atromaculata* Sandst., *Buellia epipolia* (Ach.) Mong. *non auct.*, *Buellia lainea* (Ach.) Clauzade & Ozenda, *Buellia subochracea* (Zahlbr.) J. Steiner, Diplotomma alboatrum (Hoffm.) Flot. var. epipolium (Ach.) A. Massal., *Diplotomma ambiguum* (Ach.) Flagey, *Diplotomma atromaculatum* (Sandst.) Szatala, *Diplotomma epipolium* (Ach.) Arnold *non auct.*, Diplotomma epipolium (Ach.) Arnold var. ambiguum (Ach.) Arnold, *Diplotomma heppianum* (Müll. Arg.) Arnold, *Diplotomma laineum* (Ach.) J. Nowak & Tobol., *Diplotomma subochraceum* (Zahlbr.) Szatala, *Diplotomma tegulare* Körb., *Lecanora lainea* Ach., *Lecidea alboatra* (Hoffm.) Chevall., Lecidea alboatra (Hoffm.) Chevall. var. ambigua (Ach.) Harm., *Lecidea ambigua* Ach., *Lecidea heppiana* Müll. Arg., *Lecidea soreumidia* Stirt., *Lichen alboater* Hoffm., *Rhizocarpon alboatrum* (Hoffm.) Anzi, *Rhizocarpon ambiguum* (Ach.) Zahlbr., *Rhizocarpon heppianum* (Müll. Arg.) Müll. Arg., *Rhizocarpon soreumidium* (Stirt.) A.L. Sm.

L – Subs.: cor, xyl, int – Alt.: 1–5 – Note: a mild-temperate to southern boreal-montane lichen found on bark, lignum and base-rich or slightly calciferous rocks, brick, roofing tiles etc., mostly below the subalpine belt; widespread throughout the Alps. – **Au**: S, K, St, N. **Ge**: OB, Schw. **Sw**: BE, GR, LU, SZ, TI, VD, VS. **Fr**: AHP, HAl, Isè, Sav, HSav, Var, Vau. **It**: Frl, Ven, TAA, Lomb, Piem, VA, Lig. **Sl**: SlA.

***Diplotomma chlorophaeum* (Hepp *ex* Leight.) Kr.P. Singh & S.R. Singh**

Syn.: *Buellia chlorophaea* (Hepp *ex* Leight.) Lettau, *Buellia porphyrica* (Arnold) Mong., *Diplotomma porphyricum* Arnold, *Lecidea chlorophaea* Hepp *ex* Leight., *Lecidea porphyrica* (Arnold) Stizenb., *Rhizocarpon chlorophaeum* (Hepp *ex* Leight.) Müll. Arg.

L – Subs.: sil, int – Alt.: 1–5 – Note: a temperate, perhaps holarctic early coloniser of basic siliceous rocks and roofing tiles; overlooked, and certainly more widespread in the Alps. – **Au**: T, St. **Ge**: Ge. **Fr**: AHP, Var, Vau. **It**: TAA, Lomb, Piem, Lig.

***Diplotomma hedinii* (H. Magn.) P. Clerc & Cl. Roux**

Syn.: *Buellia epipolia auct.*, *Buellia hedinii* H. Magn., *Diplotomma epipolium auct. non* (Ach.) Arnold

L – Subs.: cal, sil – Alt.: 2–5 – Note: a mainly temperate species of exposed calcareous rocks; widespread throughout the Alps. – **Au**: V, T, S, K, St, O, N, B. **Sw**: BE, GR, LU, SZ, VD, VS. **Fr**: AHP, HAl, AMa, Drô, Isè, Sav, HSav, Var, Vau. **It**: Frl, Ven, TAA, Lomb, Piem, VA, Lig.

***Diplotomma lutosum* A. Massal.**

Syn.: *Buellia dispersa* (Kremp.) Lindau, *Buellia subdispersa* Mig., Diplotomma alboatrum (Hoffm.) Flot. var. dispersum Kremp., *Diplotomma dispersum* (Kremp.) Arnold, *Diplotomma subdispersum* (Mig.) Etayo & Breuss

L – Subs.: cal, int – Alt.: 3–5 – Note: an apparently widespread but rare, or at least rarely distinguished, mostly silicicolous species, characterised by 4-celled spores with transversal septa only, and by the I+ blue reaction of the medulla. – **Au**: V, T, S, K, St, O, N. **Ge**: Schw. **Sw**: BE, GR, LU. **Fr**: AHP, HAl, AMa. **It**: Frl, Lomb, Piem.

***Diplotomma murorum* (A. Massal.) Coppins**

Syn.: Buellia epipolia var. murorum (A. Massal.) Zahlbr., Diplotomma alboatrum var. murorum A. Massal.

L # – Subs.: sil-par – Alt.: 2. – Note: a mild-temperate lichen starting the life-cycle on species of the *Caloplaca teicholyta*-complex, the peculiar biology of which deserves further study. – **It**: Ven.

***Diplotomma nivale* (Bagl. & Carestia) Hafellner**

Syn.: *Buellia margaritacea* (“Sommerf.”) Lynge, *Buellia nivalis* (Bagl. & Carestia) Hertel, *Diplotomma margaritaceum auct. non* (Ach.) Szatala, *Leciographa nivalis* Bagl. & Carestia, *Polyschistes nivalis* (Bagl. & Carestia) Keissl., *Tryblidaria nivalis* (Bagl. & Carestia) Rehm

L – Subs.: cal-par, sil-par – Alt.: 3–5 – Note: mainly on *Caloplaca* species on steeply inclined to vertical faces of more or less calciferous rocks in upland areas. – **Au**: V, T, S, K, St, O, N. **Ge**: OB. **Fr**: AHP, AMa, HSav. **It**: Ven, TAA, Piem, Lig.

***Diplotomma pharcidium* (Ach.) M. Choisy**

Syn.: Buellia alboatra (Hoffm.) Th. Fr. var. athroa (Ach.) Th. Fr., Buellia alboatra (Hoffm.) Th. Fr. var. zabotica (Körb.) Th. Fr., *Buellia pharcidia* (Ach.) Malme, *Buellia zabotica* (Körb.) Räsänen, *Diplotomma athroum* (Ach.) Stein, *Diplotomma zaboticum* Körb., *Lecanora pharcidia* Ach., Lecidea parasema (Ach.) Ach. var. athroa Ach.

L – Subs.: cor, xyl – Alt.: 2–3 – Note: a corticolous species of the *D. alboatrum*-group with relatively large apothecia with a prominent, thick margin, and additionally a thalline veil; usually on smooth bark of deciduous trees (*Populus*, *Fraxinus*); an inland species at low elevations; from the Alps there are only some scattered records, but perhaps it was not always distinguished from similar species. – **Au**: N. **Sw**: GR.

***Diplotomma scheideggerianum* (Bricaud & Cl. Roux) Nimis**

Syn.: *Buellia scheideggeriana* Bricaud & Cl. Roux

L – Subs.: cal-par – Alt.: 2–3 – Note: a mild-temperate lichen which seems to have a narrower ecological range than that of its hosts (*Caloplaca chrysodeta*, *C. xantholyta*), being slightly more hygro – and less photophytic; from the Alps there are several scattered records. – **Au**: St. **Sw**: GR. **Fr**: AHP, Var, Vau. **It**: Frl.

***Diplotomma venustum* (Körb.) Körb.**

Syn.: Buellia alboatra (Hoffm.) Th. Fr. var. venusta (Körb.) Th. Fr., *Buellia suevica* Bertsch, *Buellia venusta* (Körb.) Lettau, Diplotomma alboatrum (Hoffm.) Flot. var. venustum Körb., Diplotomma epipolium (Ach.) Arnold var. reagens J. Steiner

L – Subs.: cal, cal-par – Alt.: 1–5 – Note: this mild-temperate to Mediterranean lichen, at least when young, is a constant parasite on *Protoparmeliopsis versicolor*, reaching above treeline south of the Alps; widespread throughout the Alps. – **Au**: V, T, K, St, O, N. **Ge**: OB, Schw. **Sw**: BE, GR, SZ, VS. **Fr**: AHP, HAl, AMa, Drô, Sav, HSav, Var, Vau. **It**: Frl, Ven, Lomb, Piem, Lig.

***Dirina ceratoniae* (Ach.) Fr.**

Syn.: *Dirina repanda* Fr. *non auct.*, *Lecania ceratoniae* (Ach.) Stizenb., *Lecanora ceratoniae* Ach., Lecanora repanda Duby f. corticola Harm., *Parmelia ceratoniae* (Ach.) Spreng.

L – Subs.: cor – Alt.: 1 – Note: *D. ceratoniae* and *D. massiliensis* have been extensively studied from the molecular point of view, which showed that *D. ceratoniae* is not only corticolous, but quite frequently saxicolous as well. Mostly, the saxicolous specimens can be morphologically distinguished from the strictly saxicolous *D. massiliensis*. However, there are cases where saxicolous specimens of the two species are virtually indistinguishable without DNA data. In the study area the species is known only from the base of the Western Alps, not far from the coast. – **Fr**: AMa.

***Dirina massiliensis* Durieu & Mont.**

Syn.: *Biatora praerimata* (Nyl.) Walt. Watson, *Bilimbia stenhammari* (Fr. *ex* Stenh.) Boistel, *Dirina cyclosora* Poelt & Nimis, *Dirina patronii* Bagl., *Dirina repanda auct. non* Fr., *Dirina stenhammari* (Fr. *ex* Stenh.) Poelt & Follmann, *Dirinopsis massiliensis* De Not., *Lecanactis opponens* (Nyl.) H. Olivier, *Lecanactis stenhammari* (Fr. *ex* Stenh.) Arnold, *Lecanora repanda* Fr. *ex* Duby, *Lecidea conspurcata* (Sm.) Ach., *Lecidea opponens* Nyl., *Lecidea stenhammari* Fr. *ex* Stenh., *Variolaria conspurcata* (Sm.) Turner & Borrer

L – Subs.: cal, sil, int – Alt.: 1–3 – Note: on steeply inclined or underhanging surfaces of basic siliceous or calcareous rocks, very variable according to the type of substrata (thallus colour depends on the quantity of calcium oxalates, and on the density of epilichenic cyanobacteria); in the study area the sexual form is restricted to coastal situations at the base of the SW Alps, while the sterile form is more widespread. – **Au**: T, S, K, St, O, N. **Ge**: OB, Schw. **Sw**: BE, LU, GR. **Fr**: AHP, AMa, Drô, Sav, HSav, Var, Vau. **It**: Ven, TAA, Piem, Lig.

***Eiglera flavida* (Hepp) Hafellner**

Syn.: *Aspicilia argillacea* Anzi, *Aspicilia flavida* (Hepp) Rehm, Aspicilia flavida (Hepp) Rehm f. detrita Arnold, *Aspicilia micrantha* Körb., *Aspicilia ochracea* (Schaer.) A.Massal., *Lecanora flavida* Hepp, Lecanora flavida Hepp f. detrita (Arnold) Mig., *Lecidea contraria* Malme

L – Subs.: cal, int – Alt.: 3–5 – Note: a cool-temperate to arctic-alpine lichen found on base-rich or weakly calciferous rocks, not rarely on pebbles and small stones near the ground in cold sites; widespread throughout the Alps, but easy to overlook. – **Au**: V, T, S, K, St, N. **Ge**: OB, Schw. **Sw**: BE, GR, SZ, VS. **Fr**: AHP, HAl, AMa, Isè, Sav, HSav. **It**: Frl, Ven, TAA, Lomb, Piem, VA. **Li**.

***Eiglera homalomorpha* (Nyl.) Clauzade & Cl. Roux *ex* Hafellner & Türk**

Syn.: *Aspicilia homalomorpha* (Nyl.) Hue, *Hymenelia homalomorpha* (Nyl.) Poelt & Vězda, *Lecanora homalomorpha* Nyl., *Lecidea cavatula* Nyl.

L – Subs.: cal, int – Alt.: 3–5 – Note: mainly on limestone and dolomite near the ground, such as on basal parts of steep cliffs; widespread throughout the Alps, where it is locally common. – **Au**: V, T, S, K, St, O, N. **Ge**: OB. **Sw**: BE, LU, SZ, VS. **Fr**: AHP, HAl, AMa, Drô, Sav, HSav, Vau. **It**: Frl.

***Elixia flexella* (Ach.) Lumbsch**

Syn.: *Lecidea flexella* (Ach.) Hedl., *Leptographa flexella* (Ach.) M. Choisy, *Limboria flexella* Ach., *Lithographa flexella* (Ach.) Zahlbr., *Placographa flexella* (Ach.) Th. Fr., *Ptychographa flexella* (Ach.) Coppins, *Xylographa flexella* (Ach.) Nyl.

L – Subs.: xyl – Alt.: 3–4 – Note: on lignum, especially on vertical sides of stumps, with optimum in the subalpine belt; certainly more widespread throughout the Alps. – **Au**: V, T, S, K, St, O, N. **Ge**: OB. **Sw**: UR, VS. **It**: TAA, Lomb. **Sl**: SlA.

***Encephalographa elisae* A. Massal.**

Syn.: Encephalographa cerebrin a (DC.) A. Massal. var. elisae (A. Massal.) Anzi, *Encephalographa rubiformis* A. Massal., *Melaspilea elisae* (A. Massal.) Redinger, *Melaspilea rubiformis* (A. Massal.) Redinger

L – Subs.: cal – Alt.: 1–2 – Note: a mild-temperate lichen found on compact calcareous rocks in sheltered, microclimatically stable situations, often in underhangs; apparently more frequent in the Western and Southern Alps. – **Fr**: AHP, AMa, Drô, Isè, Vau. **It**: Frl, Ven, Lomb, Piem.

***Enchylium bachmanianum* (Fink) Otálora, P.M. Jørg. & Wedin var. millegranum (Degel.) ined. (provisionally placed here, ICN Art. 36.1b)**

Syn.: Collema bachmanianum (Fink) Degel. var. millegranum Degel.

L – Subs.: ter-sil, ter-cal – Alt.: 3–5 – Note: this variety differs from the typical on in the presence of granuliform isidia on both thallus and apothecial margins; when sterile, it is difficult to distinguish from the equally terricolous E. tenax var. vulgare, as well as from the usually calcicolous *Lathagrium fuscovirens*; the distribution is arctic-alpine, the species being extremely rare in the Central European mountains, with a few records from the Alps. The specimen from Tyrol has been revised as belonging to var. millegranum; the exsiccata on which the record from Italy (Lombardia) is based has been revised by Degelius as +/ – var. millegranum. The presence of the typical variety in the Alps is therefore dubious. – **Au**: T, St. **It**: Lomb.

***Enchylium coccophorum* (Tuck.) Otálora, P.M. Jørg. & Wedin**

Syn.: *Collema coccophorum* Tuck., *Collema crenatum* (Müll. Arg.) Zahlbr., *Collema harmandii* Samp.

L – Subs.: cal – Alt.: 1–3 – Note: on calciferous soil in dry grasslands; this almost cosmopolitan species of dry areas – which can be easily mistaken for *E. tenax* – might be more widespread in the Alps. – **Au**: St. **Fr**: AHP, Vau.

***Enchylium conglomeratum* (Hoffm.) Otálora, P.M. Jørg. & Wedin**

Syn.: *Collema conglomeratum* Hoffm., *Collema fasciculare* (L.) Weber *ex* F.H. Wigg. var. conglomeratum (Hoffm.) Ach., *Collema verruculosum* Hepp *ex* Müll. Arg. *non* *sensu* Arnold, *Synechoblastus conglomeratus* (Hoffm.) Körb.

L – Subs.: cor – Alt.: 1–3 – Note: a mainly temperate species with a fragmented holarctic range, found on nutrient-rich bark, especially of *Juglans*; formerly more widespread, presently restricted to the vicinity of small settlements in mountain valleys, where it is locally abundant. – **Au**: T, K, St, O, N. **Sw**: BE, GR, UW, VS. **Fr**: AHP, AMa, Sav, HSav, Var, Vau. **It**: Frl, Ven, TAA, Lomb, Piem.

***Enchylium expansum* (Degel.) P.M. Jørg.**

Syn.: Collema tenax (Sw.) Ach. var. expansum Degel.

L – Subs.: ter-cal – Alt.: ?5 – Note: known almost exclusively from arctic-alpine localities, at temperate latitudes this species appears to be restricted to high altitudes, *e.g.* at *c.* 2,700 m in the single locality known for the Alps. – **Au**: T.

***Enchylium ligerinum* (Hy) Otálora, P.M. Jørg. & Wedin**

Syn.: *Collema ligerinum* (Hy) Harm., Collema pulposum (Bernh.) Ach. var. ligerinum Hy, *Collema verruculosum* *sensu* Arnold

L – Subs.: cor – Alt.: 2–3 – Note: a mild-temperate species found on base-rich bark, especially of *Juglans* and *Populus*; more widespread in the past, but locally still common near small settlements in montane valleys of the Alps. – **Au**: T, K, St. **Sw**: BE, GR, TI, UW, VS. **Fr**: AHP, AMa, Drô, Sav, Var, Vau. **It**: Frl, Ven, TAA, Lomb, Piem, VA, Lig. **Li**.

***Enchylium limosum* (Ach.) Otálora, P.M. Jørg. & Wedin**

Syn.: *Collema forissii* Szatala, *Collema glaucescens* Hoffm., *Collema limosum* (Ach.) Ach., *Collema viscosum* A. Massal., *Lichen limosus* Ach., *Parmelia limosa* (Ach.) Ach.

L – Subs.: ter-cal – Alt.: 1–3 – Note: a holarctic, temperate to boreal-montane, short-lived species of mineral, clay soil in disturbed habitats; certainly overlooked, but never common in the Alps. – **Au**: V, T, S, St. **Sw**: TI, VS. **Fr**: AMa, Sav. **It**: Ven, Lomb, Piem.

***Enchylium polycarpon* (Hoffm.) Otálora, P.M. Jørg. & Wedin subsp. polycarpon**

Syn.: *Collema orbiculare* (Schaer.) Tonglet, *Collema polycarpon* Hoffm., *Collema stygium* Rabenh., *Collemodium polycarpoides* Nyl., *Leptogium polycarpoides* (Nyl.) Harm., *Lathagrium orbiculare* (Schaer.) Arnold, *Synechoblastus orbicularis* (Schaer.) Dalla Torre & Sarnth., *Synechoblastus polycarpus* (Hoffm.) Dalla Torre & Sarnth.

L – Subs.: cal – Alt.: 1–5 – Note: a holarctic species found on exposed, hard, calciferous rocks and dolomite; widespread throughout the Alps. – **Au**: V, T, S, K, St, O, N. **Ge**: OB, Schw. **Sw**: BE, GL, GR, SZ, TI, UR, UW, VD, VS. **Fr**: AHP, HAl, AMa, Isè, Sav, HSav, Var, Vau. **It**: Frl, Ven, TAA, Lomb, Piem, VA, Lig. **Sl**: Tg.

***Enchylium polycarpon* (Hoffm.) Otálora, P.M. Jørg. & Wedin subsp. corcyrense (Arnold) ined. (provisionally placed here, ICN Art. 36.1b)**

Syn.: Collema polycarpon Hoffm. subsp. corcyrense (Arnold) Pišút, Collema polycarpon Hoffm. var. corcyrense (Arnold) Harm., *Collema ragusanum* Zahlbr., *Collema salevense* (Müll. Arg.) Zahlbr., *Lathagrium akralense* Flagey, *Lathagrium flaccidulum* Flagey, Lathagrium orbiculare (Schaer.) Arnold f. corcyrense Arnold, *Lathagrium salevense* (Müll. Arg.) M. Choisy, *Synechoblastus salevensis* Müll. Arg.

L – Subs.: cal – Alt.: 1–4 – Note: more thermophytic and more southern than the typical subspecies, this taxon is worthy of further study. – **Au**: T, N. **Fr**: AMa, HSav, Var. **It**: Lomb, Piem, Lig.

***Enchylium tenax* (Sw.) Gray**

Syn.: *Collema ceranoides* Borrer, *Collema concinnum* Flot., *Collema crustaceum* Kremp., *Collema intestiniforme* Rabenh., *Collema palmatum auct.*, *Collema pulposulum* Nyl., *Collema pulposum* (Bernh.) Ach., Collema pulposum (Bernh.) Ach. var. corallinum A. Massal., Collema pulposum (Bernh.) Ach. var. tenax (Sw.) Nyl., Collema pulposum (Bernh.) Ach. var. vulgare (Schaer.) Schaer., *Collema subcorallinum* Degel., *Collema subpulposum* Nyl., *Collema substellatum* H. Magn., *Collema tenax* (Sw.) Ach., Collema tenax (Sw.) Ach. var. ceranoides (Borrer) Degel., Collema tenax (Sw.) Ach. var. corallinum (A. Massal.) Degel., Collema tenax (Sw.) Ach. var. crustaceum (Kremp.) Degel., Collema tenax (Sw.) Ach. var. substellatum (H. Magn.) Degel., Collema tenax (Sw.) Ach. var. vulgare (Schaer.) Degel., *Collema trachselii* Schaer., *Lichen pulposus* Bernh., *Lichen tenax* Sw., *Parmelia pulposa* (Bernh.) Ach.

L – Subs.: ter-cal, bry – Alt.: 1–6 – Note: an extremely polymorphic and ecologically wide-ranging species, certainly the most common of the genus in the Alps; it is a widespread holarctic, almost cosmopolitan lichen found on calciferous or base-rich siliceous soil in open habitats (*e.g.* in dry grasslands), on consolidating sand dunes and on terricolous bryophytes, more rarely directly on rock, often found also in disturbed habitats such as track sides in urban settlements. – **Au**: V, T, S, K, St, O, N, B. **Ge**: OB, Schw. **Sw**: BE, GL, GR, LU, SG, SZ, TI, UR, UW, VD, VS. **Fr**: AHP, HAl, AMa, Drô, Isè, Sav, HSav, Var, Vau. **It**: Frl, Ven, TAA, Lomb, Piem, VA, Lig. **Sl**: SlA, Tg. **Li**.

***Endocarpon adscendens* (Anzi) Müll. Arg.**

Syn.: Dermatocarpon pusillum (Hedw.) Anzi var. adscendens Anzi, *Endocarpon evirescens* (Nyl.) Nyl., *Endocarpon pallidum auct*.

L – Subs.: ter-cal, ter-sil, bry – Alt.: 2–4 – Note: a mainly temperate, perhaps holarctic lichen found on terricolous mosses, often near and on cyanobacterial colonies, with optimum in upland areas with base-rich siliceous rocks; widespread throughout the Alps. – **Au**: V, T, S, K, St, O, N. **Ge**: OB. **Sw**: BE, GR, SZ, TI, UR, VD, VS. **Fr**: HAl, AMa, Isè, HSav. **It**: Frl, TAA, Lomb, Piem. **Sl**: SlA.

***Endocarpon adsurgens* Vain.**

L – Subs.: ter-cal – Alt.: 3–5 – Note: a species recalling *E. adscendens* in the polyphyllous thallus of adscending squamules (to 2 mm across) with a dull brown upper side and a blackish lower side, attached by a few rhizines, the hymenial algal cells globose; on soil layers over calcareous rocks, based on a type from Finland, but widespread in Europe; from the Alps there are some scattered records, but the species is rather rare. – **Au**: V, T, K, St. **Sw**: GR, VS. **Fr**: AMa. **It**: TAA.

***Endocarpon latzelianum* Servít**

L – Subs.: cal, sil – Alt.: 1–2 – Note: a species with adpressed to imbricate, small squamules (to 0.6 mm across) with crenulate to sublobate margins, relatively small ascospores (less than 40 µm long), and globose to broadly ellipsoid hymenial algal cells; based on a type from Croatia and showing a southern distribution in Europe; in the Alps it is very rare, at low elevations. – **Sw**: LU.

***Endocarpon loscosii* Müll. Arg.**

L – Subs.: ter-cal – Alt.: 3 – Note: a species with a monophyllus, plane, totally adnate, olivaceous thallus, relatively small ascospores (less than 40 µm long), and globose to elongate hymenial algal cells; on clay to sandy soil; based on a type from Spain and with a southern distribution in Europe and a single record from an inner dry valley of the Western Alps. – **Sw**: VS.

***Endocarpon pallidulum* (Nyl.) Nyl.**

Syn.: *Verrucaria pallidula* Nyl.

L – Subs.: ter-cal – Alt.: 2 – Note: a species with a pale brownish thallus consisting of roundish squamules which are less than 1 mm across, and with relatively small ascospores (less than 40 µm long); based on a type from the Andes (Peru), where it was growing on sandy soil, with two records from the Central and Western Alps, which urgently need confirmation. – **Sw**: LU. **Fr**: AMa.

***Endocarpon pallidum* Ach.**

Syn.: *Dermatocarpon pallidum* (Ach.) Mudd, *Endopyrenium pallidum* (Ach.) Boistel, *Verrucaria pallida* (Ach.) Nyl.

L – Subs.: ter-cal – Alt.: 1–2 – Note: a mainly southern lichen found in open, dry, calcareous grasslands; the epithet “*pallidum*” was often used in the past to designate *E. adscendens*; most records are from the Southern Alps. – **Ge**: OB. **Fr**: Var. **It**: Frl, TAA, Lomb, Piem.

***Endocarpon psorodeum* (Nyl.) Blomb. & Forssell**

Syn.: *Dermatocarpon psorodeum* (Nyl.) Vain., *Verrucaria psorodea* Nyl.

L – Subs.: sil, int, cal – Alt.: 2–3 – Note: on mineral-rich basic siliceous rocks with some water seepage, often associated to colonies of cyanobacteria; probably more widespread throughout the Alps, especially in dry-continenral valleys. – **Au**: K, O, N. **Sw**: SZ. **Fr**: Isè. **It**: Piem.

***Endocarpon pusillum* Hedw.**

Syn.: *Dermatocarpon glomeruliferum* A. Massal., *Dermatocarpon pusillum* (Hedw.) Anzi, *Dermatocarpon sorediatum* (Borrer) Arnold, *Endocarpon garovaglii* (Mont.) Schaer., *Endocarpon glomeruliferum* (A. Massal.) Trevis., Endocarpon pusillum Hedw. var. garovaglii (Mont.) Willey, *Endocarpon schaereri* Körb., *Endocarpon sorediatum* (Borrer) Hook., *Endocarpon subscabridulum* (Nyl.) Nyl., *Endocarpon trapeziforme* (J. Koenig) Trevis. *non auct.*, *Endopyrenium pusillum* (Hedw.) Körb., *Lichen trapeziformis* J. Koenig, *Verrucaria garovaglii* Mont., *Verrucaria sorediata* Borrer, *Verrucaria subscabridula* Nyl.

L # – Subs.: cal, ter-cal – Alt.: 1–5 – Note: on calcareous soil, most often in fissures of calcareous rocks. *E. pusillum* in the sense of most European authors is heterogeneous, and perhaps could be subdivided into several species; widespread throughout the Alps. – **Au**: V, T, S, K, St, O, N, B. **Ge**: OB, Schw. **Sw**: FR, GR, LU, SZ, TI, VS. **Fr**: AHP, AMa, Isè, Sav, HSav, Var, Vau. **It**: Frl, Ven, TAA, Lomb, Piem, VA, Lig. **Sl**: SlA.

***Endocarpon schisticola* (B. de Lesd.) Servít**

Syn.: *Endopyrenium schisticola* B. de Lesd.

L # – Subs.: sil – Alt.: 2 – Note: a species with an epilithic thallus consisting of adpressed, roundish squamules (1–3 mm in diam.), several immersed ascomata per squamule (to 0.25 mm in diam.), an involucrellum adpressed to the perithecial wall in the upper third, 2-spored asci, and colourless, oblong, muriform ascospores with 7–13 transversal and up to 3 longitudinal septa (to 45 µm long); on schist; only known from the Western Alps (Italy). – **It**: Lig.

***Endohyalina insularis* (Arnold) Giralt, van den Boom & Elix**

Syn.: Buellia saxatilis Schaer. f. insularis Arnold, *Rinodina insularis* (Arnold) Hafellner

L – Subs.: sil-par – Alt.: 3–4 – Note: a widespread, but apparently rare silicicolous species described from South Tyrol, with a srongly reduced thallus and an obligately lichenicolous growth on species of the *Lecanora rupicola*-group; in the Alps it is not common. – **Au**: T, K, St. **Fr**: AMa, Var. **It**: TAA. **Sl**: SlA.

***Endohyalina interjecta* (Müll. Arg.) Giralt**

Syn.: *Buellia interjecta* Müll. Arg., *Lecidea interjecta* (Müll. Arg.) Stizenb., *Rinodina interjecta* (Müll. Arg.) H. Mayrhofer, Scheid. & Sheard

L # – Subs.: sil – Alt.: 3 – Note: a buelliod lichen with a brown, areolate thallus and *Dirinaria*-type ascospores, found on granite boulders; only known from the type locality in the Western Alps. – **Fr**: HSav.

***Enterographa crassa* (DC.) Fée**

Syn.: *Chiodecton crassum* (DC.) Zahlbr., *Chiodecton venosum* (Pers.) Zahlbr., *Enterographa venosa* (Pers.) A. Massal., *Leucodecton crassum* (DC.) A. Massal., *Opegrapha crassa* DC, *Sagedia crassa* (DC.) A. Massal., *Stigmatidium crassum* (DC.) Duby

L – Subs.: cor – Alt.: 1–2 – Note: a mild-temperate to humid subtropical lichen found on smooth bark in riparian, open, humid-warm woodlands below the montane belt; extremely rare in the Alps (Insubrian District, Western Alps). – **Fr**: AMa, Var. **It**: Ven, Lomb.

***Enterographa elaborata* (Leight.) Coppins & P. James**

Syn.: *Enterographa jorgei* Vězda & Vivant, *Enterographa venosa* *sensu* A. L. Sm., *Opegrapha venosa* Sm. *nom.illeg. non* Pers., *Platygramma elaborata* Leight., *Stigmatidium venosum* (Sm.) Nyl.

L – Subs.: cor – Alt.: 1–2 – Note: a species with a pale grey thallus reacting C+ red and Pd+ yellow, and long lirelliform ascomata; on bark of broad-leaved trees in forests and woodlands under maritime influence; distributed in suitable habitats not too far from the coast in Western Europe, further south from Macaronesia eastwards to the Black Sea area, with a few records from the Western Alps at low elevations. – **Fr**: AMa. **It**: Lig.

***Enterographa hutchinsiae* (Leight.) A. Massal.**

Syn.: *Chiodecton hutchinsiae* (Leight.) Zahlbr., *Enterographa germanica* (A. Massal.) A. Massal., *Opegrapha hutchinsiae* (Leight.) Körb., *Platygramma hutchinsiae* Leight., *Stigmatidium germanicum* A. Massal., *Stigmatidium hutchinsiae* (Leight.) Nyl.

L – Subs.: sil – Alt.: 2–3 – Note: a mild-temperate to humid subtropical lichen found on vertical to underhanging surfaces of hard siliceous rocks, rarely on smooth bark, at relatively low elevations; extremely rare in the Alps. – **Au**: St, N.

***Enterographa zonata* (Körb.) Källsten**

Syn.: *Lecanactis zonata* (Körb.) A. Massal., *Opegrapha horistica* (Leight.) Stein, *Opegrapha zonata* Körb., *Verrucaria horistica* Leight.

L – Subs.: sil – Alt.: 2–4 – Note: a temperate to southern boreal-montane, perhaps circumpolar lichen found on vertical to underhanging surfaces of hard siliceous rocks in deep gorges or mature forests, very rarely on bark, with optimum in the montane belt; widespread throughout the Alps. – **Au**: V, T, S, K, St, N. **Sw**: BE, SG, TI, UR, UW, VS. **Fr**: AHP, AMa, HSav, Var, Vau. **It**: Frl, TAA, Lomb, Piem, Lig. **Sl**: SlA.

***Eopyrenula grandicula* Coppins**

L – Subs.: cor – Alt.: 3 – Note: a species with 3-dystoseptate, large macroconidia (*c.* 15–20 µm long) which are diagnostic, found in *Graphidion* communities on the smooth bark of deciduous trees within old-growth forests, in areas with high precipitations; widespread in Western Europe but not common, with a single locality in the Eastern Alps. – **Au**: S.

***Eopyrenula leucoplaca* (Wallr.) R.C. Harris**

Syn.: *Leptosphaeria leucoplaca* (Wallr.) Vain., *Porina chiomela* (Norman) Zahlbr., *Pyrenula alba* A. Massal., *Pyrenula farrea auct. non* (Ach.) Branth & Rostr., *Pyrenula leucoplaca* (Wallr.) Körb., *Pyrenula quercus* A. Massal., *Pyrenula schaereri* A. Massal., *Sagedia chiomela* Norman, *Spermatodium leucoplacum* (Wallr.) Trevis., *Verrucaria farrea auct. non* (Ach.) Branth & Rostr., *Verrucaria leucoplaca* Wallr.

L – Subs.: cor – Alt.: 2–3 – Note: a temperate species found on the (mostly) smooth bark of deciduous trees in open, humid forests; widespread throughout the Alps, but not common, probably more frequent in the past. – **Au**: S, K, St. **Ge**: OB. **Sw**: BE, LU. **Fr**: AHP, AMa, HSav, Var, Vau. **It**: Ven, TAA.

***Ephebe hispidula* (Ach.) Horw.**

Syn.: *Cornicularia hispidula* Ach., *Ephebe spinulosa* Th. Fr.

L – Subs.: sil – Alt.: 2 – Note: a species forming small cushions consisting of rough filaments due to the presence of small branchlets, with 16-spored asci; on boulders along streams and small rivers; most records are from Northern Europe, with a single record from the Central Alps, which needs confirmation. – **Sw**: TI.

***Ephebe lanata* (L.) Vain.**

Syn.: *Cornicularia lanata* (L.) DC., *Ephebe intricata* Lamy, *Ephebe lapponica* Nyl., *Ephebe pubescens auth. p.p.*, *Ephebeia cantabrica* Nyl., *Ephebeia martindalei* Nyl., *Lichen lanatus* L., *Parmelia lanata* (L.) Wallr.

L – Subs.: sil, sil-aqu – Alt.: 3–5 – Note: an arctic-alpine to boreal-montane, circumpolar lichen with outliers in the cool-temperate zone, found on steeply inclined, periodically wetted or inundated siliceous rocks, on seepage tracks, etc., with optimum above treeline; widespread throughout the siliceous Alps. – **Au**: V, T, S, K, St, O, N. **Sw**: BE, GR, TI, VD, VS. **Fr**: AHP, HAl, AMa, Sav, HSav, Var. **It**: Ven, TAA, Lomb, Piem, VA.

***Ephebe multispora* (E. Dahl) Henssen**

Syn.: *Spilonematopsis multispora* E. Dahl

L – Subs.: sil-aqu – Alt.: ?4 – Note: the smallest of the *Ephebe* species, with thread-like thalli recalling a *Stigonema*-cushion, terminal pycnoascocarps (to 0.1 mm in diam.) with a spinulose margin, 16-spored asci, and simple, subspherical ascospores (4–6 × 3–5 μm); based on a type from Greenland, on humid rocks; very rare, with a single record from the Eastern Alps (Austria). – **Au**: T.

***Ephebe perspinulosa* Nyl.**

Syn.: *Ephebe papillata* H. Magn., *Ephebe trachytera* (Nyl. *ex* Vain.) Henssen *nom.illeg.*, *Ephebeia perspinulosa* (Nyl.) Räsänen

L – Subs.: sil-aqu – Alt.: 4–5 – Note: on periodically wetted siliceous rocks above and near treeline; perhaps more widespread in the Alps. – **It**: Piem.

***Epilichen glauconigellus* (Nyl.) Hafellner**

Syn.: Buellia scabrosa (Ach.) A. Massal. var. cinereascens Th. Fr., *Lecidea glauconigella* Nyl.

L – Subs.: ter-sil, ter-par – Alt.: 3–5 – Note: a species with an indistinct thallus and crowded but dispersed, marginate apothecia; parasitic on species of *Baeomyces* (mostly *B. rufus*); widespread in Northern Europe, very rare in the Central European mountains, with a few records from the Eastern Alps. – **Au**: T, S, St.

***Epilichen scabrosus* (Ach.) Clem.**

Syn.: *Buellia scabrosa* (Ach.) A. Massal., *Karschia scabrosa* (Ach.) Rehm, *Lecidea scabrosa* Ach., *Skolekites scabrosus* (Ach.) Norman

L – Subs.: ter-sil, ter-sil-par – Alt.: 3–5 – Note: optimum in cold-humid situations in upland areas, at first a parasite on *Baeomyces*-species, becoming autotrophic when old; widespread throughout the siliceous Alps. – **Au**: V, T, S, K, St, N. **Ge**: Schw. **Sw**: BE, GR, TI, UR, VD, VS. **Fr**: AHP, HAl, AMa, HSav, Vau. **It**: Frl, Ven, TAA, Lomb, Piem, VA.

***Epiphloea byssina* (Hoffm.) Henssen & P.M. Jørg**

Syn.: *Collema byssinum* Hoffm., Collema cheileum (Ach.) Ach. var. byssinum (Hoffm.) Körb., *Leptogium amphineum* Ach. *ex* Nyl., *Leptogium anomalum* (Nyl.) Harm., *Leptogium byssinum* (Hoffm.) Zwackh *ex* Nyl., *Polychidium byssinum* (Hoffm.) Trevis.

L – Subs.: ter-cal – Alt.: 2–5 – Note: an inconspicuous, perhaps overlooked, ephemeral lichen of calciferous clay soil. – **Au**: T. **It**: TAA.

***Epiphloea terrena* (Nyl.) Trevis.**

Syn.: *Amphidium terrenum* (Nyl.) Nyl., *Leptogium crozalsianum* Harm., *Leptogium terrenum* Nyl.

L – Subs.: ter-sil – Alt.: 1 – Note: on bare siliceous soil in Mediterranean grasslands and garrigues, in the Alps with a single station in Liguria, near Spotorno. – **It**: Lig.

***Euopsis granatina* (Sommerf.) Nyl.**

Syn.: *Lecanora granatina* Sommerf., *Pyrenopsis granatina* (Sommerf.) Nyl.

L – Subs.: sil – Alt.: 4–6 – Note: a species with an areolate thallus, the granulose areoles being dark brown and spotted pale brown due to the presence of two photobionts, the minute lecanorine apothecia recalling small garnets; on periodically wet siliceous boulders and outcrops in sunny places; widespread in the Holarctic region but altogether rare, also recorded from Papua New Guinea; for the Alps only reported from two distant localities. – **Sw**: VS. **It**: TAA.

***Euopsis pulvinata* (Schaer.) Vain.**

Syn.: *Blennothallia haemalea* (Sommerf.) Trevis., *Collema haemaleum* Sommerf., *Lecidea pulvinata* Schaer., *Pannaria haemalea* (Sommerf.) A. Massal., *Pyrenopsis haemalea* (Sommerf.) Norrl., *Pyrenopsis macrocarpa* E. Dahl, *Pyrenopsis pulvinata* (Schaer.) Th. Fr.

L – Subs.: sil – Alt.: 3–4 – Note: a cool-temperate to boreal-montane, perhaps circumpolar lichen found on siliceous rocks, especially in seepage tracks on small pebbles in wet places, sometimes even on soil; much overlooked, and probably more widespread in the Alps. – **Au**: V, T, S, St. **Sw**: BE, UR, VS. **It**: TAA.

***Evernia divaricata* (L.) Ach.**

Syn.: *Letharia divaricata* (L.) Hue, *Lichen divaricatus* L.; incl. *Evernia perfragilis* Llano

L – Subs.: cor, xyl, deb, ter – Alt.: 3–5 – Note: a cool-temperate to southern boreal-montane, circumpolar lichen found on twigs of coniferous and deciduous trees in semi-natural, humid, montane to subalpine forests; albeit very rarely, it also occurs on soil on windy ridges with frequent fog; widespread throughout the Alps. – **Au**: V, T, S, K, St, O, N. **Ge**: OB, Schw. **Sw**: BE, FR, GL, GR, LU, SZ, TI, UR, UW, VD, VS. **Fr**: AHP, HAl, AMa, Isè, Sav, HSav, Vau. **It**: Frl, Ven, TAA, Lomb, Piem, VA, Lig. **Sl**: SlA, Tg. **Li**.

***Evernia illyrica* (Zahlbr.) Du Rietz**

Syn.: Evernia divaricata (L.) Ach. subsp. illyrica Zahlbr., *Letharia illyrica* (Zahlbr.) Harm.

L – Subs.: cor – Alt.: 3 – Note: a Mediterranean-montane species found in humid beech-fir forests; extremely rare in the Alps, being known from a few localities in Slovenia. – **Sl**: SlA, Tg.

***Evernia mesomorpha* Nyl.**

Syn.: Evernia prunastri (L.) Ach. var. thamnodes Flot., *Evernia thamnodes* (Flot.) Arnold, *Letharia mesomorpha* (Nyl.) Du Rietz

L – Subs.: cor, xyl, sil – Alt.: 3–5 – Note: a boreal-montane, circumpolar lichen found on bark (often on twigs) of conifers, sometimes on lignum (*e.g.* on wooden poles, and decorticated branches), with optimum in the subalpine belt; widespread throughout the Alps, but only locally common. – **Au**: V, T, S, K, St, O. **Ge**: OB, Schw. **Sw**: BE, GR, TI, VS. **Fr**: HAl, AMa, HSav. **It**: Frl, Ven, TAA, Lomb, Piem, VA.

***Evernia prunastri* (L.) Ach.**

Syn.: *Evernia arenaria auct. non* (Retz.) Fr., *Evernia herinii* P.A. Duvign., *Letharia arenaria auct.*, *Lichen prunastri* L., *Parmelia prunastri* (L.) Ach.

L – Subs.: cor, xyl – Alt.: 1–4 – Note: a widespread holarctic lichen, rare only in disturbed situations and in dry habitats; common throughout the Alps. – **Au**: V, T, S, K, St, O, N, B. **Ge**: OB, Schw. **Sw**: BE, FR, GL, GR, LU, SG, SZ, TI, UR, UW, VD, VS. **Fr**: AHP, HAl, AMa, Drô, Isè, Sav, HSav, Var, Vau. **It**: Frl, Ven, TAA, Lomb, Piem, VA, Lig. **Sl**: SlA, Tg. **Li**.

***Farnoldia dissipabilis* (Nyl.) Hertel**

Syn.: *Lecidea dissipabilis* Nyl., Lecidea jurana Schaer. var. sublutescens (Nyl.) Hertel, *Lecidea obstans* Nyl., *Lecidea sublutescens* Nyl., *Melanolecia dissipabilis* (Nyl.) Hertel, Tremolecia jurana (Schaer.) Hertel var. sublutescens (Nyl.) Hertel

L – Subs.: cal – Alt.: 4–6 – Note: on calciferous rocks, especially in rock fissures and on steeply inclined to slightly overhanging surfaces near or above treeline; very closely related to *F. jurana*, this taxon, known from the Central European mountains (Alps, Carpathians), awaits further study – **Au**: V, T, K, St, O. **Ge**: OB, Schw. **Sw**: GR, VS. **Fr**: AHP, Sav. **It**: Ven, TAA, Piem.

**Farnoldia hypocrita (A. Massal.) Fröberg var. hypocrita**

Syn.: *Biatora emergens* Müll. Arg., *Haplocarpon lithospersum* (Zahlbr.) M. Choisy, *Lecidea emergens* Flot. *nom.illeg.*, *Lecidea hypocrita* A. Massal., Lecidea jurana Schaer. var. emergens (Müll. Arg.) Boistel, *Lecidea lithospersa* Zahlbr., *Lecidea lithygra* *sensu* Fr.

L – Subs.: cal – Alt.: 3–6 – Note: a taxon with a partially to entirely endolithic thallus and large, epruinose apothecia with a broad margin, the hymenial surface becoming uneven to subgyrose; on sunny surfaces of limestone and dolomite; widespread in Europe and Greenland, and rather common in the Alps. – **Au**: V, T, S, K, St, O, N. **Ge**: OB, Schw. **Sw**: BE, GR, LU, SZ, UW, VD. **Fr**: AMa, Isè, Sav. **It**: Frl, Ven, TAA, Lomb, Piem. **Sl**: Tg. **Li**.

**Farnoldia hypocrita (A. Massal.) Fröberg var. ligans (Nyl.) Hafellner & Türk**

Syn.: Lecidea hypocrita A. Massal. var. ligans (Nyl.) Hertel, *Lecidea ligans* Nyl.

L – Subs.: cal – Alt.: 3–5 – Note: a variety with smaller apothecia with thin, flexuose margins and often blue-grey pruinose discs; on sunny surfaces of limestone and dolomite; a rare taxon of the Central European mountains; the distribution in the Alps is incompletely known because this variety was not always distinguished. – **Au**: T, St, O. **Fr**: Sav, HSav. **It**: TAA.

**Farnoldia jurana (Schaer.) Hertel subsp. jurana**

Syn.: *Biatora annularis* Müll. Arg., *Haplocarpon juranum* (Schaer.) M. Choisy, *Lecidea albosuffusa* Th. Fr., *Lecidea annularis* (Müll. Arg.) Müll. Arg., *Lecidea calcigena* Flörke *ex* Körb., *Lecidea inferior* Nyl., *Lecidea jurana* Schaer., *Lecidea petrosa* Arnold, Lecidea petrosa Arnold var. glaucocarpa Arnold, *Lecidea subvorticosa* Nyl., *Melanolecia jurana* (Schaer.) Hertel, *Tremolecia jurana* (Schaer.) Hertel

L – Subs.: cal – Alt.: 3–6 – Note: a cool-temperate to arctic-alpine, circumpolar species found on limestone and dolomite, more rarely on other calciferous rocks (*e.g.* sandstone and schist); one of the most common calcicolous species above and near treeline throughout the Alps. – **Au**: V, T, S, K, St, O, N. **Ge**: OB, Schw. **Sw**: BE, GR, LU, SZ, UR, VD, VS. **Fr**: AHP, HAl, AMa, Isè, Sav, HSav, Vau. **It**: Frl, Ven, TAA, Lomb, Piem, VA. **Sl**: SlA, Tg. **Li**.

**Farnoldia jurana (Schaer.) Hertel subsp. bicincta (Hertel) Clauzade & Cl. Roux *ex* Hafellner & Türk**

Syn.: Lecidea jurana Schaer. var. bicincta Hertel, Melanolecia jurana (Schaer.) Hertel subsp. bicincta (Hertel) Clauzade & Cl. Roux, Melanolecia jurana (Schaer.) Hertel var. bicincta (Hertel) Hertel

L – Subs.: cal – Alt.: 4–5 – Note: on exposed calcareous rocks near and above treeline, often associated with *Hymenelia coerulea*; probably more widespread in the Alps. – **Au**: V, T, S, K, St, O, N. **Ge**: OB. **Fr**: Sav, HSav. **It**: Frl, Ven, TAA, Piem.

**Farnoldia jurana (Schaer.) Hertel subsp. caerulea (Kremp.) M. Brand**

Syn.: *Lecidea caerulea* Kremp.

L # – Subs.: cal – Alt.: 3–4 – Note: a name applied to a small-spored morph of the *F. jurana* aggregate; on limestone and dolomite; distribution incompletely known, because it was not always distinguished. – **Au**: S, O. **Ge**: OB.

**Farnoldia jurana (Schaer.) Hertel subsp. muverani (Müll. Arg.) Hafellner & Türk**

Syn.: *Biatora muverani* Müll. Arg., Lecidea jurana Schaer. var. muverani (Müll. Arg.) Hertel, *Lecidea muverani* (Müll. Arg.) Müll. Arg., Melanolecia jurana (Schaer.) Hertel var. muverani (Müll. Arg.) Hertel

L – Subs.: cal – Alt.: 3–5 – Note: a taxon with non-umbonate apothecia, the hypothecium about twice as high as the hymenium, the exciple relatively thin, reacting slowly K+ crimson to violet; on limestone and calcareous schists; a rare taxon of the Central European mountains; the distribution in the Alps is incompletely known because this variety was not always distinguished. – **Au**: V, T, S, St, O. **Fr**: HSav.

***Farnoldia micropsis* (A. Massal.) Hertel**

Syn.: *Lecidea macrospora* Lynge, *Lecidea micropsis* A. Massal., *Lecidea nivalis* Anzi, *Lecidea rhaetica* Hepp *ex* Th. Fr., *Lecidea valpellinensis* B. de Lesd, *Lecidella micropsis* (A. Massal.) Körb., *Lecidella rhaetica* (Hepp *ex* Th. Fr.) Körb., *Melanolecia micropsis* (A. Massal.) Hertel, *Tremolecia nivalis* (Anzi) Hertel

L – Subs.: cal, int – Alt.: 4–6 – Note: an arctic-alpine, circumpolar species, with optimum on calciferous sandstone and schists, rarer on limestone and dolomite, mostly on inclined faces; widespread throughout the Alps. – **Au**: V, T, S, K, St, O, N. **Ge**: OB, Schw. **Sw**: BE, GR, TI, UR, VD, VS. **Fr**: AHP, HAl, AMa, Sav, HSav. **It**: Frl, Ven, TAA, Lomb, Piem, VA, Lig. **Sl**: SlA.

***Farnoldia muscigena* (Vězda) Hafellner & Tretiach**

Syn.: Lecidea jurana Schaer. var. muscigena Vězda, *Melanolecia muscigena* (Vězda) Hertel

L – Subs.: bry – Alt.: 4–5 – Note: known from the Central European mountains (Tatra, Alps), this lichen is found on moribund bryophytes, crustose lichens and plant debris over calcareous substrata; probably more widespread in the Alps – **Au**: S, St. **It**: Frl, Piem.

***Farnoldia similigena* (Nyl.) Hertel**

Syn.: *Lecidea similigena* Nyl., *Lecidea subrhaetica* Arnold *ex* Lettau, *Melanolecia similigena* (Nyl.) Hertel, *Tremolecia similigena* (Nyl.) Hertel

L – Subs.: int – Alt.: 4–6 – Note: a rare arctic-alpine species found on inclined to vertical faces of calciferous siliceous rocks (*e.g.* calcareous sandstone and schist). – **Au**: V, T. **Ge**: Schw. **Sw**: GR. **Fr**: HAl, Sav. **It**: TAA.

***Felipes leucopellaeus* (Ach.) Frisch & G. Thor**

Syn.: *Arthonia leucopellaea* (Ach.) Almq., *Arthonia marmorata* Nyl., *Arthonia melaleuca* *sensu* Malme, *Arthonia schaereri* A. Massal., *Melaspilea associata* Norman, *Trachylia leucopellaea* (Ach.) Eitner, Spiloma melaleucum Ach. var. leucopellaeum Ach.

L – Subs.: cor – Alt.: 2–3 – Note: a species with lobate ascomata bordered by byssoid hyphae, and phragmospored, microcephalic ascospores; on bark of conifers in old-growth forests under suboceanic climatic conditions; widespread in the temperate to boreal zones, in the Alps lacking only in areas with a more continental climate. – **Au**: V, T, S, K, St, O, N. **Ge**: OB, Schw. **Sw**: BE, GL, LU, SG, SZ, UR, UW. **It**: Frl. **Sl**: SlA.

***Fellhanera bouteillei* (Desm.) Vězda**

Syn.: *Biatora bouteillei* (Desm.) A. Massal., *Biatorina bouteillei* (Desm.) Arnold, *Biatorina littorella* (Nyl.) A.L. Sm., *Catillaria bouteillei* (Desm.) Zahlbr., *Catillaria littorella* (Nyl.) Zahlbr., *Catillaria rubicola* (P. Crouan & H. Crouan) H. Olivier, *Lecanora bouteillei* (Desm.) Harm., *Lecidea bouteillei* (Desm.) Nyl., *Lecidea littorella* Nyl., *Parmelia bouteillei* Desm.

L – Subs.: cor, fol – Alt.: 1–3 – Note: a temperate to southern boreal-montane species found on leaves and twigs of conifers (especially *Abies* in the Alps), but also on evergreen Mediterranean trees and shrubs in very humid situations. – **Au**: S, K, St, O. **Ge**: OB, Schw. **Sw**: VS. **Fr**: AHP, AMa, Drô, Isè, HSav, Var, Vau. **It**: Ven, TAA, Piem, VA, Lig. **Sl**: SlA.

***Fellhanera gyrophorica* Sérus., Coppins, Diederich & Scheid.**

L – Subs.: cor – Alt.: 2–3 – Note: a species with a farinose to granular, greenish thallus and sessile, pale to brownish pycnidia reacting C+ red (apothecia rare); on bark of deciduous and coniferous trees or overgrowing bryophytes in lowland to lower montane forests with a suboceanic climate; so far only known from Europe; in the Alps the distribution is still insufficiently documented, as the species was overlooked or undercollected, being almost always sterile. – **Au**: O. **Sw**: BE.

***Fellhanera subtilis* (Vězda) Diederich & Sérus.**

Syn.: *Arthonia subtilis* (Vězda) Vězda, *Bacidia subtilis* Vězda

L – Subs.: cor, bry – Alt.: 2–4 – Note: on twigs of small shrubs (*Vaccinium*, *Calluna*), more rarely on mosses (*e.g. Polytrichum*) and on branches of *Picea* in cold sites, on north-facing slopes or in deep gorges, usually in upland areas; perhaps more widespread in the Alps. – **Au**: V, T, S, K, St, O, N. **Ge**: OB. **Sw**: GR, SZ, VS. **It**: Frl. **Sl**: SlA, Tg.

***Fellhanera viridisorediata* Aptroot, M. Brand & Spier**

L – Subs.: cor – Alt.: 2–3 – Note: a usually epiphytic species with a granular, greenish grey, farinose-sorediate thallus containing roccellic acid, the soralia initially crateriform, later pustular and eventually fusing, the sessile apothecia rarely present, with dark brown discs and paler persistent margins, a paraplectenchymatic, hyaline exciple and a brownish hypothecium, 8-spored asci, and mainly 1-septate ascospores (14–17 × 3–5 μm) with a thin perispore; widespread in Europe, but most common in its western parts, with a single record from the Eastern Alps (Germany). – **Ge**: Schw.

***Fellhaneropsis myrtillicola* (Erichsen) Sérus. & Coppins**

Syn.: *Bacidia buxi* Vězda & Vivant, *Bacidia gorgonea* Vězda & Poelt, *Bacidia myriocarpa* Erichsen, *Bacidia myrtillicola* Erichsen, *Bacidia nitschkeana* (J. Lahm *ex* Rabenh.) Zahlbr. var. perpusilloides Erichsen, *Fellhanera buxi* (Vězda & Vivant) Vězda, *Fellhanera myrtillicola* (Erichsen) Hafellner

L – Subs.: cor, fol – Alt.: 2–3 – Note: a species with an inconspicuous thallus, small (less than 0.2 mm in diam.), pale brown to bluish-grey, soon virtually immarginate apothecia, mostly 3-septate ascospores which are longer than 18 µm, and filiform, curved macroconidia (*Fellhanera subtilis* has ascospores shorter than 16 µm and pyriform-clavate conidia); usually on twigs and leaves (including needles of *e.g. Abies*) in the understory of forests, but also in semi-natural habitats; widespread in Western and Central Europe and Macaronesia; the occurrence in the Alps still insufficiently documented. – **Au**: T, K, St. **Ge**: OB. **Fr**: AMa, Isè, Vau. **It**: Frl.

***Fellhaneropsis vezdae* (Coppins & P. James) Sérus. & Coppins**

Syn.: *Bacidia vezdae* Coppins & P. James, *Fellhanera vezdae* (Coppins & P. James) V. Wirth

L – Subs.: cor – Alt.: 2–3 – Note: a species with apothecia in various shades of brown, frequently becoming tuberculate, and mostly 5–7-septate ascospores which are longer than 30 µm; on bark of broad-leaved (mainly *Quercus*) and coniferous (*e.g. Abies*) trees in very humid forests, especially on basal parts of trunks, sometimes foliicolous; widespread in Western and Central Europe and Macaronesia, with a few records from the Alps. – **Au**: S, St. **Sw**: BE, SG.

***Flavocetraria cucullata* (Bellardi) Kärnefelt & A. Thell**

Syn.: *Allocetraria cucullata* (Bellardi) Randlane & Saag, *Cetraria cucullata* (Bellardi) Ach., *Lichen cucullatus* Bellardi, *Nephromopsis cucullata* (Bellardi) Divakar, A. Crespo & Lumbsch

L – Subs.: ter-sil, ter-cal, deb – Alt.: 3–5 – Note: a circumpolar, arctic-alpine lichen, a typical element of tundra-like vegetation in open, dry habitats, mostly above treeline, most frequent on basic siliceous substrata, in wind-exposed ridges; widespread throughout the Alps. – **Au**: V, T, S, K, St, O, N. **Ge**: OB, Schw. **Sw**: BE, GR, LU, SG, SZ, TI, UR, VD, VS. **Fr**: AHP, HAl, AMa, Isè, Sav, HSav. **It**: Frl, Ven, TAA, Lomb, Piem, VA. **Sl**: SlA. **Li**.

***Flavocetraria nivalis* (L.) Kärnefelt & A. Thell**

Syn.: *Allocetraria nivalis* (L.) Randlane & Saag, *Cetraria nivalis* (L.) Ach., *Lichen nivalis* L., *Nephromopsis nivalis* (L.) Divakar, A. Crespo & Lumbsch

L – Subs.: ter-sil, ter-cal, deb – Alt.: 3–6 – Note: a circumpolar, arctic-alpine lichen, a typical element of tundra-like vegetation of open, dry habitats above treeline; widespread throughout the Alps. – **Au**: V, T, S, K, St, O, N. **Ge**: OB, Schw. **Sw**: BE, GR, SG, SZ, TI, UR, VS. **Fr**: AHP, HAl, AMa, Isè, Sav, HSav. **It**: Frl, Ven, TAA, Lomb, Piem, VA, Lig. **Sl**: SlA.

***Flavoparmelia caperata* (L.) Hale**

Syn.: *Imbricaria caperata* (L.) DC., *Lichen caperatus* L., *Parmelia caperata* (L.) Ach., *Parmelia cylisphora* (Ach.) Vain., *Parmelia herreana* Zahbr., *Parmelia negativa* Gyeln., *Parmelia subglauca* Nyl., *Pseudoparmelia caperata* (L.) Hale

L – Subs.: cor, sil – Alt.: 1–3 – Note: a mild-temperate lichen found on isolated deciduous, more rarely evergreen trees, only exceptionally on rocks (*e.g.* on north-exposed faces of basic siliceous rocks in dry-continental valleys of the Alps); common and abundant in the submediterranean belt throughout the Alps, rarer elsewhere. – **Au**: V, T, S, K, St, O, N, B. **Ge**: OB, Schw. **Sw**: BE, GL, GR, LU, SG, SZ, TI, UR, UW, VD, VS. **Fr**: AHP, HAl, AMa, Drô, Isè, Sav, HSav, Var, Vau. **It**: Frl, Ven, TAA, Lomb, Piem, VA, Lig. **Sl**: SlA, Tg. **Li**.

***Flavoparmelia soredians* (Nyl.) Hale**

Syn.: Parmelia caperata (L.) Ach. var. soredians (Nyl.) Hillmann, *Parmelia soredians* Nyl., *Pseudoparmelia soredians* (Nyl.) Hale

L – Subs.: cor – Alt.: 1–2 – Note: a mild-temperate lichen found on broad-leaved, more rarely coniferous trees, with optimum in areas with a warm-humid climate, usually below the montane belt; apparently more frequent in the Western and Southern Alps, at low elevations. – **Fr**: AHP, AMa, Isè, Var, Vau. **It**: Frl, Ven, TAA, Lomb, Piem, Lig.

***Flavopunctelia flaventior* (Stirt.) Hale**

Syn.: *Parmelia andreana* Müll. Arg., *Parmelia flaventior* Stirt., *Parmelia kernstockii* Lynge & Zahlbr., *Parmelia lobarina* Zahlbr., *Parmelia variata* Hue, *Punctelia flaventior* (Stirt.) Krog

L – Subs.: cor, xyl – Alt.: 2–3 – Note: a species of rather continental areas, found on more or less isolated deciduous trees; most frequent in dry valleys of the Alps. – **Au**: V, T, S, K, St, O, N, B. **Ge**: OB. **Sw**: BE, GR, LU, SG, SZ, TI, VD, VS. **Fr**: Isè. **It**: Frl, Ven, TAA, Lig. **Sl**: SlA. **Li**.

***Flavopunctelia soredica* (Nyl.) Hale**

Syn.: *Parmelia manshurica* Asahina, *Parmelia soredica* Nyl., *Parmelia ulophyllodes* (Vain.) Savicz, *Punctelia soredica* (Nyl.) Krog

L – Subs.: cor – Alt.: 3–4 – Note: a mainly epiphytic species, restricted to valleys of the Alps with a continental climate. – **Sw**: ?BE. **It**: TAA, Lomb.

***Frigidopyrenia bryospila* (Nyl.) Grube**

Syn.: *Arthopyrenia bryopsila* (Nyl.) Arnold, *Collemopsidium bryospilum* (Nyl.) Coppins, *Didymella bryospila* (Nyl.) H. Magn., *Verrucaria bryospila* Nyl.

L – Subs.: bry, deb – Alt.: 5 – Note: a circum-arctic-alpine species found on soil and plant debris, with a squamulose, olive to brownish-black thallus, a chroococcoid photobiont, ascomata with a peridium pigmented mainly in the intercellular spaces, cylindrical fissitunicate asci, and 1-septate ascospores; known from two localites only in the Alps. – **Au**: S. **Sw**: BE.

***Fritzea lamprophora* (Körb.) Stein**

Syn.: *Lecidea lamprophora* (Körb.) Zahlbr., *Psora lamprophora* Körb.

L # – Subs.: sil – Alt.: 5 – Note: a species with a brown, glossy thallus consisting of flat squamules with free margins, laminal, red-brown, immarginate apothecia, and simple (!) ascospores; ecology poorly known, type from Southern Poland, on basalt in a gorge, with a single historical record from the Eastern Alps (Austria). – **Au**: N.

***Frutidella caesioatra* (Schaer.) Kalb**

Syn.: *Lecidea arctica* Sommerf., *Lecidea caesioatra* Schaer., *Lecidella arctica* (Sommerf.) Körb., *Lecidella caesioatra* (Schaer.) Kalb

L – Subs.: sil, ter-sil, bry-sil – Alt.: 4–6 – Note: an arctic-alpine lichen found on silicicolous mosses, especially *Andreaea* and *Grimmia*, in places with a long snow cover, more rarely directly on rock. – **Au**: V, T, S, K, St. **Ge**: Ge. **Sw**: BE, GR, UR. **Fr**: HSav. **It**: Ven, TAA, Lomb, Piem, VA.

***Frutidella furfuracea* (Anzi) M. Westb. & M. Svenss.**

Syn.: *Biatora amaurospoda* Anzi, *Biatora furfuracea* Anzi, *Biatora pullata* Norman, *Frutidella pullata* (Norman) Schmull, *Lecidea amaurospoda* (Anzi) Vain., *Lecidea anziana* Zahlbr., *Lecidea furfuracea* (Anzi) Jatta, *Lecidea ostrogothensis* Nyl., *Lecidea perobscurans* Nyl., *Lecidea pullata* (Norman) Th. Fr.

L – Subs.: cor, xyl – Alt.: 2–4 – Note: on bark, on basal parts of (mainly) coniferous trees, more rarely on lignum, often associated with *Parmeliopsis ambigua*, with optimum in the subalpine belt; widespread throughout the Alps. – **Au**: V, T, S, K, St, O, N. **Ge**: OB. **Sw**: BE, GL, GR, LU, SG, SZ, TI, UR, UW, VD, VS. **It**: Frl, Ven, TAA, Lomb, Piem. **Sl**: SlA.

***Fuscidea arboricola* Coppins & Tønsberg**

L – Subs.: cor – Alt.: 3 – Note: a species with a greenish to brownish, areolate thallus (similar to *F. praeruptorum*) being UV – and reacting Pd+ red, usually surrounded by a brownish prothallus, the more central areolae breaking up apically to form green soralia, mostly remaining sterile; on bark of broad-leaved trees in various forest types, from the lowlands to the montane belt; widespread in Europe and Eastern North America; there are several scattered records from the Alps, where the species was certainly undercollected. – **Au**: T, K. **Sw**: LU. **Sl**: SlA.

***Fuscidea austera* (Nyl.) P. James**

Syn.: *Fuscidea aggregata* (Flot.) V. Wirth & Vězda, *Fuscidea aggregatilis* (Grummann) V. Wirth & Vězda, *Fuscidea taeniarum* (Malme) V. Wirth & Vězda, *Lecanora austera* Nyl., *Lecidea aggregata* (Flot.) H. Magn., *Lecidea aggregatilis* Grummann

L – Subs.: sil – Alt.: 3–5 – Note: on steeply inclined to underhanging surfaces of hard siliceous rocks in upland areas. – **Au**: T, S, K, St. **Sw**: GL. **Fr**: AMa. **It**: TAA.

***Fuscidea cyathoides* (Ach.) V. Wirth & Vězda**

Syn.: *Biatora rivulosa* (Ach.) Fr., *Fuscidea subrivulosa* (Vain.) P. James, *Lecidea cyathoides* (Ach.) Ach., *Lecidea rivulosa* Ach., *Lichen cyathoides* Ach.

L – Subs.: sil – Alt.: 2–4 – Note: a cool-temperate to southern boreal-montane, perhaps circumpolar lichen found on siliceous rocks in humid areas; widespread throughout the Alps. – **Au**: T, S, K, St, N. **Ge**: Ge. **Sw**: BE, GR, SZ, TI, VD, VS. **Fr**: AMa, Var, Vau. **It**: Frl, Ven, TAA, Lomb, Piem. **Sl**: SlA.

***Fuscidea gothoburgensis* (H. Magn.) V. Wirth & Vězda**

Syn.: *Fuscidea maculosa* (H. Magn.) Poelt, *Lecidea gothoburgensis* H. Magn.

L – Subs.: sil – Alt.: 3–6 – Note: apparently a rare lichen on steep surfaces of very hard siliceous rocks in shaded, cool habitats. – **Au**: T, S, K, St. **Sw**: GR, VS. **Fr**: HSav.

***Fuscidea kochiana* (Hepp) V. Wirth & Vězda**

Syn.: *Biatora indigula* (Nyl.) Walt. Watson, *Biatora kochiana* (Hepp) Rabenh., Biatora rivulosa (Ach.) Fr. var. kochiana (Hepp) Fr., *Lecanora mammillifera* Stirt., *Lecidea coriacella* Nyl., *Lecidea interludens* Nyl., *Lecidea kochiana* Hepp, *Lecidea morosa* Dufour, Lecidea rivulosa Ach. var. kochiana (Hepp) Schaer.

L – Subs.: sil – Alt.: 3–6 – Note: on steeply inclined surfaces of hard siliceous rocks in moderately shaded, humid situations, with optimum near or above treeline; widespread throughout the Alps. – **Au**: V, T, S, K, St, N. **Ge**: Schw. **Sw**: BE, GR, LU, TI, UR, UW, VS. **Fr**: HAl, AMa, Sav, HSav. **It**: Frl, Ven, TAA, Lomb, Piem, VA.

***Fuscidea lightfootii* (Sm.) Coppins & P. James**

Syn.: *Biatora lightfootii* (Sm.) Hepp, *Biatorina lightfootii* (Sm.) Körb., *Catillaria lightfootii* (Sm.) H. Olivier, *Lecidea lightfootii* (Sm.) Ach., *Lichen lightfootii* Sm.

L – Subs.: cor – Alt.: 2–3 – Note: a species with a grey-green to brownish-green, areolate thallus (similar to *F. arboricola*), being bluish-white under UV-light and reacting C-, often sorediate and at the same time bearing dark grey-brown to blackish apothecia with centrally constricted ascospores; on small twigs of various trees and shrubs, often near bogs or streams; widespread in the Holarctic region, with a western tendency in Europe and a few scattered records from the Alps. – **Au**: T, O. **Ge**: Ge. **Sw**: BE, SZ. **Fr**: Var.

***Fuscidea lygaea* (W. Mann) V. Wirth & Vězda**

Syn.: *Biatora lygaea* W. Mann, *Catillaria massalongoi* Körb. *non auct.*, *Fuscidea periplaca* (Nyl.) V. Wirth & Vězda, *Fuscidea tenebrica* (Nyl.) V. Wirth & Vězda, Lecidea kochiana Hepp var. lygaea (W. Mann) Leight., *Lecidea lygaea* Ach. *nom.illeg.*, *Lecidea obscurata* (Ach.) Schaer., *Lecidea periplaca* Nyl., *Lecidea tenebrica* Nyl., *Rhizocarpon massalongii* (Körb.) Malme *non auct*.

L – Subs.: sil – Alt.: 2–5 – Note: on steeply inclined to underhanging surfaces of hard siliceous rocks in upland areas; widespread throughout the Alps. – **Au**: V, T, S, K, St, N. **Sw**: BE, GR, UR, VS. **Fr**: AMa, Isè, Sav, Var. **It**: TAA, Lomb, Piem, VA.

***Fuscidea mollis* (Wahlenb.) V. Wirth & Vězda**

Syn.: *Biatora mollis* (Wahlenb.) Arnold, *Lecidea mollis* (Wahlenb.) Nyl., Lecidea rivulosa Ach. var. mollis Wahlenb.

L – Subs.: sil – Alt.: 3–6 – Note: a mainly western species with isolated outposts in the Central European mountains, found on steeply inclined, sheltered surfaces of siliceous rocks. – **Au**: V, T, S, K, St. **Sw**: UR. **It**: TAA, Piem, VA.

***Fuscidea praeruptorum* (Du Rietz & H. Magn.) V. Wirth & Vězda**

Syn.: *Lecidea praeruptorum* Du Rietz & H. Magn.

L – Subs.: sil, cor – Alt.: 3 – Note: a species with a grey-green, areolate thallus (similar to *F. arboricola*) being white under UV-light and reacting C+ red, with a brown prothallus usually visible between the areolae and at the margins, the more central areolae with brownish-grey to greyish-green, often concave soralia, mostly remaining sterile; usually saxicolous on shaded rocks under overhangs, but also switching to the bark of various trees; widespread in the Holarctic region, with a boreal to temperate-montane pattern and a few scattered records from the Alps. – **Au**: K. **Sw**: TI. **Fr**: Isè, Var.

***Fuscidea pusilla* Tønsberg**

L – Subs.: cor – Alt.: 3–4 – Note: a species with a minute, greenish, areolate thallus being bluish-white under UV-light and reacting C-, surrounded by a brown prothallus, often occurring in colonies, the more central areolae breaking up apically to form green soralia (the species is only known in the sterile state); on bark of broad-leaved trees in various forest types, from the lowlands to the montane belt; widespread in the Holarctic region, with several scattered records from the Alps, where it was certainly undercollected. – **Au**: T, K, St, O. **Sw**: SZ, TI, UR, UW. **It**: Lomb. **Sl**: SlA.

***Fuscidea recensa* (Stirt.) Hertel, V. Wirth & Vězda**

Syn.: *Fuscidea curvula* (H. Magn.) Hertel, *Lecidea arcuatula* (Arnold) Hue, *Lecidea curvula* H. Magn., *Lecidea recensa* Stirt.

L – Subs.: sil – Alt.: 2–4 – Note: on hard siliceous rocks in humid, sheltered sites, usually below the subalpine belt; overlooked, being often sterile, and probably more widespread in the Alps. – **Au**: St, N. **It**: TAA, VA.

***Fuscidea stiriaca* (A. Massal.) Hafellner**

Syn.: *Biatora stiriaca* A. Massal., *Biatorinella fagicola* (Zschacke) Deschâtres & Werner, Biatorinella rivulosa (Ach.) Deschâtres & Werner var. corticola (Fr.) Werner, Fuscidea cyathoides (Ach.) V. Wirth & Vězda var. corticola (Fr.) Kalb., *Fuscidea fagicola* (Zschacke) Hafellner & Türk, Lecidea cyathoides (Ach.) Ach. var. corticola (Fr.) H. Magn., *Lecidea fagicola* Zschacke, Lecidea rivulosa Ach. var. corticola (Fr.) Jatta, *Lecidea stiriaca* (A. Massal.) Jatta

L # – Subs.: cor – Alt.: 2–3 – Note: a cool-temperate to southern boreal-montane lichen found on bark (mainly of *Fagus*), not always distinguished from *F. cyathoides*. – **Au**: K, St. **Fr**: AHP, Var, Vau. **It**: Frl, Ven, TAA, Lomb, Piem, Lig. **Sl**: SlA, Tg.

***Fuscopannaria confusa* (P.M. Jørg.) P.M. Jørg.**

Syn.: *Pannaria confusa* P.M. Jørg.

L – Subs.: cor, sil – Alt.: 5 – Note: a species resembling *F. mediterranea*, but squamules with a plane surface and less than 2 mm long, with marginal, coarsely granular soralia; on branches of various trees and shrubs near the ground, but also on rocks in very humid places, like in the spray zone of waterfalls; a rare European species with a boreal to temperate-high montane distribution pattern; in the Alps known from two localities only. – **Au**: T. **Sw**: VS.

***Fuscopannaria ignobilis* (Anzi) P.M. Jørg.**

Syn.: *Pannaria ignobilis* Anzi, *Pannaria romanoana* Hue, *Pannaria servitiana* Gyeln.

L – Subs.: cor – Alt.: 1–2 – Note: a Mediterranean-Atlantic species usually found in cracks of the bark of ancient trees, near the base of the boles, with scattered records from the base of the Western Alps (France, Italy), and from Slovenia. – **Fr**: AMa, Var, Vau. **It**: Lig. **Sl**: Tg.

***Fuscopannaria leucosticta* (Tuck. *ex* E. Michener) P.M. Jørg.**

Syn.: *Pannaria craspedia* Körb., *Pannaria leucosticta* (Tuck. *ex* E. Michener) Nyl., *Parmelia leucosticta* Tuck. *ex* E. Michener

L – Subs.: cor – Alt.: 2–3 – Note: on mossy trunks of broad-leaved trees; there are no recent records from the Alps of this declining species. – **Sw**: ?GR. **It**: Frl, Ven, Lomb, Piem.

***Fuscopannaria mediterranea* (Tav.) P.M. Jørg.**

Syn.: *Pannaria mediterranea* Tav.

L – Subs.: cor – Alt.: 1–2 – Note: a mild-temperate to Mediterranean species found on bark of ancient broad-leaved trees in semi-natural, rather undisturbed, humid woodlands, more rarely on siliceous, mossy rocks, with some records from the base of the Western Alps (France). – **Fr**: AHP, AMa, Var, Vau.

***Fuscopannaria nebulosa* (Hoffm.) ined. (provisionally placed here, ICN Art. 36.1b)**

Syn.: *Moelleropsis nebulosa* (Hoffm.) Gyeln., Pannaria brunnea (Sw.) A. Massal. var. coronata (Hoffm.) A. Massal., *Pannaria nebulosa* (Hoffm.) Nyl., *Patellaria nebulosa* Hoffm., *Trachyderma nebulosum* (Hoffm.) Trevis.

L – Subs.: ter-sil – Alt.: 2–4 – Note: a mild-temperate early coloniser of clay-sandy soil, especially earth banks along unpaved roads, with optimum in humid areas with siliceous substrata; widespread throughout the siliceous Alps. – **Au**: T, S, K, St, N. **Sw**: GR, VD, VS. **Fr**: HAl, AMa, HSav, Var, Vau. **It**: Ven, TAA, Lomb, Piem, VA, Lig. **Sl**: SlA.

***Fuscopannaria praetermissa* (Nyl.) P.M. Jørg.**

Syn.: Lecidea carnosa (Dicks.) Sommerf. var. lepidiota Sommerf., Massalongia carnosa (Dicks.) Körb. var. lepidiota (Sommerf.) Körb., *Pannaria lepidiota* (Sommerf.) Th. Fr., *Pannaria praetermissa* Nyl., *Parmeliella lepidiota* (Sommerf.) Vain., *Parmeliella praetermissa* (Nyl.) P. James, *Toninia caeruleonigricans* (Lightf.) Th. Fr. *non auct.*, *Trachyderma praetermissum* (Nyl.) Trevis.

L – Subs.: ter-cal, bry, deb – Alt.: 3–5 – Note: an arctic-alpine to boreal-montane, circumpolar lichen found on calciferous soil, mosses and plant debris; widespread throughout the Alps. – **Au**: V, T, S, K, St, O, N. **Ge**: OB, Schw. **Sw**: BE, GR, LU, SZ, TI, UR, VD, VS. **Fr**: AHP, HAl, AMa, Sav, HSav. **It**: Frl, Ven, TAA, Lomb, Piem. **Sl**: SlA.

***Gabura fascicularis* (L.) P.M. Jørg.**

Syn.: *Arctomia fascicularis* (L.) Otálora & Wedin, *Collema aggregatum auct.*, *Collema ascaridosporum* (A. Massal.) Degel., *Collema fasciculare* (L.) Weber *ex* F.H. Wigg., *Lathagrium ascaridosporum* A. Massal., *Lichen fascicularis* L., *Synechoblastus aggregatus auct.*, *Synechoblastus ascaridosporus* (A. Massal.) Zwackh, *Synechoblastus fascicularis* (L.) A.L. Sm.

L – Subs.: cor – Alt.: 1–3 – Note: a mild-temperate lichen with a fragmented holarctic range, found on old broad-leaved trees, often on mosses, in open, humid stands; somehow more frequent in the past, presently very much declining. – **Au**: V, T, S, K, St, O, N. **Ge**: OB. **Sw**: GR, VD. **Fr**: AHP, AMa, Isè, Sav, Var, Vau. **It**: Ven, TAA, Lomb, Piem, Lig. **Sl**: Tg.

***Gloeoheppia turgida* (Ach.) Gyeln.**

Syn.: *Acarospora endocarpea* (Fr.) Flagey, *Endocarpon turgidum* Ach., *Heppia endocarpea* (Fr.) Hue, *Heppia turgida* (Ach.) Nyl., *Lecanora endocarpea* (Fr.) Nyl.

L – Subs.: ter-cal – Alt.: 1 – Note: a mainly Mediterranean lichen found on calciferous soil in dry grasslands, occasionally on weathered basic siliceous rocks; in the study area it is known with certainty only from the base of the Western Alps (France). – **Sw**: ?Sw. **Fr**: AMa.

***Glypholecia scabra* (Pers.) Müll. Arg.**

Syn.: *Acarospora scabra* (Pers.) Th. Fr., *Glypholecia candidissima* Nyl., *Glypholecia grumulosa* (Schaer.) Zahlbr., *Glypholecia rhagadiosa* Nyl. *nom.illeg.*, *Urceolaria scabra* Pers.

L – Subs.: cal, int – Alt.: 3–4 – Note: an incompletely holarctic species found on exposed surfaces of calciferous and base-rich siliceous rocks, mainly in dry-continental areas. – **Au**: T. **Sw**: GR, VS. **Fr**: AHP, HAl, AMa, Isè, Sav. **It**: Piem, VA, Lig.

***Gomphillus calycioides* (Delise *ex* Duby) Nyl.**

Syn.: *Baeomyces calycioides* Delise *ex* Duby, *Baeopodium calycioides* (Delise *ex* Duby) Trevis., *Berengeria calycioides* (Delise *ex* Duby) A. Massal., *Mycetodium calycioides* (Delise *ex* Duby) A. Massal.

L – Subs.: bry, cor – Alt.: 1–2 – Note: a mild-temperate to tropical species found on bryophytes, mostly on basal parts of old trunks in mature warm-humid forests at low elevations. The regions from which it was reported, mostly in the Insubrian district of Italy, are presently affected by air pollution, so that the species might be extinct. – **Sw**: TI. **It**: Ven, Lomb, Piem.

***Graphis betulina* (Pers.) Ach.**

Syn.: *Graphis juglandis* Garov. *ex* A. Massal., Graphis scripta (L.) Ach. var. betulina (Pers.) Arnold, *Opegrapha betulina* Pers.

L # – Subs.: cor – Alt.: 3 – Note: a taxon of the *G. scripta*-group with apothecia surrounded by a conspicuous, often raised white thalline margins; on bark of broad-leaved trees in various forest types; widespread in the Holarctic region, but for a long time not distinguished and distributional data therefore likely to be incomplete; also known from several localities in the Alps, but less common than *G. scripta* or *G. pulverulenta*. See also note on *G. scripta*. – **Au**: K, St, O. **Sw**: ?SZ. **Fr**: AMa, Drô. **It**: Ven, Lomb, Lig.

***Graphis elegans* (Borrer *ex* Sm.) Ach.**

Syn.: *Aulacographa elegans* (Borrer *ex* Sm.) Leight., *Graphis neglecta* Erichsen, *Graphis petrina* Nyl., *Graphis ramificans* Nyl., *Graphis sulcata* (Pers.) A. Massal., *Opegrapha elegans* Borrer *ex* Sm., *Phaeographis ramificans* (Nyl.) Lettau

L – Subs.: cor – Alt.: 1–3 – Note: a mild-temperate to humid subtropical species found on smooth bark, mainly of *Ilex* in warm-humid woodlands; very rare, and probably declining in the Alps. – **Au**: V, O. **Sw**: GR, UR. **Fr**: Isè. **It**: Frl, TAA, Lomb, Piem.

***Graphis inustuloides* Lücking**

Syn.: *Graphina anguina auct. eur. non* (Mont.) Müll. Arg., *Graphis inustula* Nyl. *non* Stirt., *Thalloloma anguinum auct. non* (Mont.) Trevis., *Ustalia anguina auct. eur. non* Mont.

L # – Subs.: cor – Alt.: 1–2 – Note: a mild-temperate to tropical species found on smooth bark, now perhaps extinct from the only known station in Italy (Insubrian District). – **It**: Lomb.

***Graphis macrocarpa* (Pers.) Röhl.**

Syn.: Graphis scipta (L.) Ach. var. macrocarpa (Pers.) Ach., *Opegrapha macrocarpa* Pers.

L # – Subs.: cor – Alt.: 2–3 – Note: a taxon of the *G. scripta*-group, characterised by apothecia with rounded ends and widely exposed, epruinose, brown discs, found on the bark of broad-leaved trees in different forest types; for a long time not distinguished, and distributional data therefore likely incomplete; also known from several localities in the Alps, but less common than *G. scripta* or *G. pulverulenta*. See also note on *G. scripta*. – **Au**: K, St. **Sw**: SZ. **Li**.

***Graphis pulverulenta* (Pers.) Ach.**

Syn.: *Graphis abietina* (Schaer.) Malbr., *Graphis cerasi* (Pers.) Ach., *Graphis diffracta* Turner, *Graphis litterella* (Ach.) Röhl., Graphis scripta (L.) Ach. var. abietina (Schaer.) Rabenh., Graphis scripta (L.) Ach. var. cerasi (Pers.) Ach., Graphis scripta (L.) Ach. var. pulverulenta (Pers.) Ach., Graphis scripta (L.) Ach. var. serpentina (Ach.) G. Mey., *Graphis serpentina* (Ach.) Ach., *Graphis subtilis* (Pers.) Röhl., *Opegrapha abietina* (Schaer.) Malbr., *Opegrapha cerasi* Pers., *Opegrapha pulverulenta* Pers., *Opegrapha serpentina* (Ach.) Schrad.

L # – Subs.: cor – Alt.: 2–3 – Note: a taxon of the *G. scripta*-group, characterised by apothecia with mostly acute ends and widely exposed, white – to grey-pruinose discs, found on the bark of broad-leaved trees in different forest types; for a long time not distinguished and distributional data therefore likely to be incomplete, but common in the Alps. See also note on *G. scripta*. – **Au**: S, St, O, N, B. **Sw**: BE, SZ, TI, UR. **Fr**: AMa, Drô, Sav, HSav, Var. **It**: Frl, TAA, Lomb, Piem. **Sl**: SlA.

***Graphis scripta* (L.) Ach.**

Syn.: *Graphis hebraica* (Hoffm.) Röhl., *Graphis limitata* (Pers.) Röhl., *Graphis microcarpa* (Ach.) Röhl., Graphis scripta (L.) Ach. var. limitata (Pers.) Ach., Graphis scripta (L.) Ach. var. spathea Ach., *Graphis spathea* (Ach.) Röhl., *Lichen scriptus* L., *Opegrapha limitata* Pers.

L # – Subs.: cor – Alt.: 1–3 – Note: a widespread temperate to southern boreal-montane lichen found on smooth bark, mostly in deciduous forests, in humid areas also on twigs and branches, but normally on trunks, in drier areas restricted to the base of the boles. In the narrow sense, this taxon is characterised by apothecia with more or less hidden discs and a thin (<= 0.1 mm) to absent thalline margin, while several species have been segregated based on morphological characters only (*G. betulina*, *G. macrocarpa*, *G. pulverulenta*). A recent study based on both molecular and morphological characters showed that, although between six and seven putative species are nested within the *G. scripta*-complex, these do not fully correspond to the taxa that were distinguished based on apothecium morphology. Pending further studies, we treat here *G. scripta* in a broad sense, while the fewer recent records of the morphologically defined “species” are provisionally treated as separate entities. – **Au**: V, T, S, K, St, O, N, B. **Ge**: OB, Schw. **Sw**: BE, FR, GL, GR, LU, SG, SZ, TI, UR, UW, VD, VS. **Fr**: AMa, Drô, Isè, Sav, HSav, Var, Vau. **It**: Frl, Ven, TAA, Lomb, Piem, VA, Lig. **Sl**: SlA, Tg.

***Gregorella humida* (Kullh.) Lumbsch**

Syn.: *Biatora humida* Kullh., *Lecidea humida* (Kullh.) Th. Fr., *Leprocollema europaeum* H. Magn., *Moelleropsis humida* (Kullh.) Coppins & P.M. Jørg.

L – Subs.: ter-cal, ter-sil – Alt.: 2–3 – Note: an inconspicuous lichen with a blackish-grey thallus composed of goniocysts containing a nostociform photobiont, and convex, virtually immarginate, colourless to medium brown (when dry) apothecia with cylindrical to subclavate asci recalling those of *Trapelia*, containing simple, relatively large ascospores; pioneer on soil and debris *e.g.* on dump heaps and margins of white roads; widespread in the boreal to temperate zones of Europe, but rare (perhaps overlooked), in the Alps, being only known from a single locality. – **Au**: St.

***Gyalecta arbuti* (Bagl.) Baloch & Lücking**

Syn.: *Bacidia arbuti* (Bagl.) Jatta, *Bacidiopsis arbuti* Bagl., *Pachyphiale arbuti* (Bagl.) Arnold

L – Subs.: cor – Alt.: 1 – Note: a species of Mediterranean, rather humid forests, frequently confused with *G. carneola*, with a few records from the base of the Western Alps. – **Fr**: AMa, Var, Vau.

***Gyalecta bilimbioides* Anzi**

L # – Subs.: cal – Alt.: 3 – Note: known only from the type collection, on dolomite, this species, characterised by the small urceolate apothecia with a reddish disc and a black margin, and by 2–6-celled ascospores, well deserves further study. – **It**: Ven.

***Gyalecta carneola* (Ach.) Hellb.**

Syn.: *Bacidia carneola* (Ach.) De Not., *Bacidia cornea* (With.) A. Massal., *Biatora carneola* (Ach.) Fr., *Gyalecta cornea* (With.) Tuck., *Gyalecta interserta* (Nyl.) H. Olivier, *Lecidea carneola* Ach., *Lecidea interserta* Nyl., *Pachyphiale carneola* (Ach.) Arnold, *Pachyphiale cornea* (With.) Poetsch

L – Subs.: cor – Alt.: 1–3 – Note: a mild-temperate species found in old, humid forests; widespread, but rare and very much declining in the Alps. – **Au**: S, O, N. **Ge**: Ge. **Sw**: GR, SZ, UR, VD, VS. **Fr**: Drô, Var. **It**: Frl, Ven, Lomb, Piem. **Sl**: SlA.

***Gyalecta derivata* (Nyl.) H. Olivier**

Syn.: *Gyalecta biformis* (Körb.) H. Olivier, *Gyalecta croatica* Schuler & Zahlbr., Gyalecta truncigena (Ach.) Hepp var. biformis (Körb.) Vězda, Gyalecta truncigena (Ach.) Hepp var. croatica (Schuler & Zahlbr.) Vězda, Gyalecta truncigena (Ach.) Hepp var. derivata (Nyl.) Boistel, *Lecidea derivata* Nyl.

L – Subs.: cor – Alt.: 2–3 – Note: a species of the *G. truncigena*-group found on broad-leaved trees (especially *Acer* and *Fraxinus*) in humid areas, with elongate-fusiform ascospores which occasionally have 1–2 straight longitudinal septa, widespread in Europe and also known from North Africa, but rather rare; in the Alps known from several scattered localities, but not always distinguished from *G. truncigena*. – **Au**: S, K, St, N. **Ge**: OB. **Sw**: SZ. **Fr**: AHP, AMa, Var.

***Gyalecta erythrozona* Lettau**

L – Subs.: int – Alt.: 4–5 – Note: a species of the *G. leucaspis*-group characterised by entire (rather than radially incised) apothecial margins, and elongate-fusiform (rather than acicular) ascospores; it grows on schists containing some calcium on moist, shaded, steep rock faces or under overhangs; widespread in the Holarctic region, in the Central European orobiomes it mostly occurs near or above treeline; in the Alps it is known from several scattered localities, and is evidently rare. – **Au**: V, T, S, K, St. **Ge**: Schw. **Sw**: TI. **Fr**: HAl, AMa, Sav. **It**: Frl.

***Gyalecta fagicola* (Arnold) Kremp.**

Syn.: *Bacidia fagicola* Arnold, *Gyalecta corticola* (Lönnr.) A.L. Sm., *Lecidea congruella* Nyl., *Pachyphiale corticola* Lönnr., *Pachyphiale fagicola* (Arnold) Zwackh

L – Subs.: cor – Alt.: 2–3 – Note: optimum in open deciduous forests, but in humid areas also found on isolated, old trees; widespread throughout the Alps, but generally not common. – **Au**: V, T, S, K, St, O, N. **Ge**: OB, Schw. **Sw**: GR, LU, SZ, VS. **Fr**: Var, Vau. **It**: Frl, TAA. **Sl**: SlA, Tg. **Li**.

***Gyalecta flotowii* Körb.**

Syn.: Gyalecta truncigena (Ach.) Hepp var. querceti (Nyl.) Boistel, *Lecidea querceti* Nyl.

L – Subs.: cor, bry – Alt.: 1–3 – Note: a mild-temperate lichen found on broad-leaved trees in clearings of ancient, undisturbed forests, especially in deep fissures of the bark, often on *Acer* and *Fraxinus*. – **Au**: T, S, St, O, N. **Ge**: OB. **Sw**: UW. **Fr**: AMa, Var, Vau. **It**: Lig. **Sl**: SlA.

***Gyalecta foveolaris* (Ach.) Schaer.**

Syn.: *Gyalecta wahlenbergiana* Ach., *Petractis foveolaris* (Ach.) A. Massal., *Secoliga foveolaris* (Ach.) A. Massal., *Urceolaria foveolaris* Ach.

L – Subs.: ter-cal, bry-cal, deb – Alt.: 3–5 – Note: a circumpolar, arctic-alpine lichen found on calciferous soil, occasionally also on rocks, in humid and shaded situations near and above treeline; widespread throughout the Alps. – **Au**: V, T, S, K, St, O, N. **Ge**: OB, Schw. **Sw**: UR, VD, VS. **Fr**: HAl, AMa, Isè, Sav. **It**: TAA, Lomb, Piem, Lig. **Sl**: SlA.

***Gyalecta friesii* Flot. *ex* Körb.**

Syn.: *Gyalecta denudata* Th. Fr., *Petractis friesii* (Flot. *ex* Körb.) A. Massal., *Secoliga friesii* (Flot. *ex* Körb.) A. Massal.

L – Subs.: bry, deb – Alt.: 3–5 – Note: a circumboreal-montane species growing on bryophytes and plant debris, more rarely on bark of conifers and on siliceous rocks, with optimum near or above treeline; apparently very rare in the Alps. – **Au**: S. **Ge**: Ge. **It**: Ven.

***Gyalecta geoica* (Wahlenb. *ex* Ach.) Ach.**

Syn.: *Lichen geoicus* Wahlenb. *ex* Ach., *Secoliga geoica* (Wahlenb. *ex* Ach.) Körb.

L – Subs.: ter-cal, bry – Alt.: 2–5 – Note: a cool-temperate to arctic-alpine, circumpolar species found on soil, bryophytes and plant debris over calcareous or base-rich siliceous substrata, often in rock fissures in sheltered situations, mostly in upland areas; widespread throughout the Alps. – **Au**: V, T, S, K, St, O, N. **Ge**: OB, Schw. **Sw**: BE, GR, SZ, TI, VD, VS. **Fr**: AHP, HSav, Vau. **It**: Frl, Ven, TAA, Lomb, Piem. **Sl**: Tg.

***Gyalecta herculina* (Rehm) Baloch, Lumbsch & Wedin**

Syn.: *Belonia herculina* (Rehm) Keissl., *Segestrella herculina* Rehm

L – Subs.: cor – Alt.: 3 – Note: a species with perithecioid, yellow-brown ascomata, the acicular to vermiform ascospores being discharged through a narrow pore; on bark of broad-leaved trees (*e.g. Fagus*), usually near the base of trunks; rare in SE Europe, with a single record from the Eastern Alps. – **Sl**: SlA.

***Gyalecta hypoleuca* (Ach.) Zahlbr.**

Syn.: *Gyalecta exanthemoides* (A. Massal.) Zahlbr., *Gyalecta gyalectoides* (A. Massal.) Lindau, *Gyalecta thelotremoides* (Nyl.) Kremp., *Lecidea thelotremoides* Nyl., *Petractis hypoleuca* (Ach.) Vězda, *Secoliga gyalectoides* (A. Massal.) A. Massal., *Thelotrema gyalectoides* A. Massal., Thelotrema gyalectoides A. Massal. var. exanthemoides A. Massal., *Urceolaria hypoleuca* Ach., *Volvaria gyalectoides* (A. Massal.) Trevis.

L – Subs.: cal – Alt.: 2–5 – Note: a cool-temperate species found on steeply inclined to underhanging faces of dolomitic rocks and limestones in rather sheltered situations, mostly in woodlands; widespread throughout the Alps. – **Au**: V, T, S, K, St, O, N. **Ge**: OB. **Sw**: BE, GL, GR, LU, SZ, UW, VD. **Fr**: AHP, AMa, Drô, Sav, HSav, Var, Vau. **It**: Frl, Ven, TAA, Lomb. **Sl**: SlA.

***Gyalecta incarnata* (Th. Fr. & Graewe) Baloch & Lücking**

Syn.: *Belonia incarnata* Th. Fr. & Graewe, *Belonia russula* Körb. *ex* Nyl. var. terrigena (Eitner) Keissl., *Belonia terrigena* Eitner, *Gongylia incarnata* (Th. Fr. & Graewe) Zahlbr., *Gongylia macrospora* Suza *ex* Servít

L – Subs.: ter-sil, bry – Alt.: 3–5 – Note: a mainly arctic-alpine species found on soil rich in humus, often in rather disturbed habitats, such as on mountain track sides, mostly above treeline; easy to overlook and probably more widespread in the Alps, but rare. – **Au**: V, T, S, K, St. **Ge**: Schw. **It**: Frl, TAA.

**Gyalecta jenensis (Batsch) Zahlbr. var. jenensis**

Syn.: *Gyalecta cupularis* (Hedw.) Schaer., Gyalecta jenensis (Batsch) Zahlbr. var. montenegrina Servít, *Lecanora cupularis* (Hedw.) Duby, *Lecidea cupularis* (Hedw.) Ach., *Peziza jenensis* Batsch

L – Subs.: cal, bry – Alt.: 2–5 – Note: a holarctic species found on limestone, dolomite and other types of calciferous rocks, occasionally over bryophytes, in shaded situations such as in deep rock fissures and underhangs, with a wide altitudinal range; widespread and locally common throughout the Alps. – **Au**: V, T, S, K, St, O, N, B. **Ge**: OB, Schw. **Sw**: BE, GR, LU, SZ, TI, UW, VS. **Fr**: AHP, AMa, Drô, Isè, Sav, HSav, Var, Vau. **It**: Frl, Ven, TAA, Lomb, Piem, VA, Lig. **Sl**: SlA, Tg.

**Gyalecta jenensis (Batsch) Zahlbr. var. macrospora Vězda**

L # – Subs.: cal – Alt.: 5 – Note: this variety, characterised by ascospores longer than 30 µm (shorter than 25 µm in the typical one), is based on a type from maritime Western Europe, on siliceous rocks; the identity of records from calcareous rocks in the alpine belt of the Austrian Alps needs confirmation. – **Au**: ?K. **Fr**: AMa, Var.

***Gyalecta kukriensis* (Räsänen) Räsänen**

Syn.: Gyalecta cupularis (Hedw.) Schaer. var. kukriensis Räsänen, Gyalecta jenensis (Batsch) Zahlbr. var. deminuta Norman *ex* Lettau, Gyalecta jenensis (Batsch) Zahlbr. var. kukriensis (Räsänen) Zahlbr.

L – Subs.: int, sil – Alt.: 4–5 – Note: on calciferous schists near or above treeline; also known from the Carpathians and Scandinavia, for the Alps reported from two distant localities only. – **Au**: S. **It**: Piem.

***Gyalecta leucaspis* (Kremp.) Kremp.**

Syn.: *Gyalecta acicularis* Anzi, *Secoliga leucaspis* Kremp. *ex* A. Massal. *nom.illeg.*, *Thelotrema leucaspis* Kremp.

L – Subs.: cal – Alt.: 2–5 – Note: on shaded, steeply inclined faces of dolomitic rocks, mostly below the alpine belt; certainly less common than *G. jenensis*, but widespread throughout the Alps. – **Au**: V, T, S, K, St, O, N. **Ge**: OB. **Sw**: GR, VD. **Fr**: AHP, AMa, Drô, Isè, HSav, Var, Vau. **It**: Frl, Ven, TAA, Lomb, Piem. **Sl**: Tg.

***Gyalecta liguriensis* (Vězda) Vězda**

Syn.: Gyalecta truncigena (Ach.) Hepp var. liguriensis Vězda

L – Subs.: cor – Alt.: 1–2 – Note: on bark of ancient trees in humid, sheltered situations; in the study area it is known only from the base of the Western Alps. – **Fr**: AMa, Var, Vau.

***Gyalecta nidarosiensis* (Kindt) Baloch & Lücking**

Syn.: *Belonia caudata* (Vězda & Vivant) P.M. Jørg. & Vězda, *Belonia nidarosiensis* (Kindt) P.M. Jørg. & Vězda, *Clathroporina calcarea* Walt. Watson, *Clathroporina caudata* Vězda & Vivant, *Microglaena nidarosiensis* Kindt

L – Subs.: sil – Alt.: 2–3 – Note: a species with perithecioid, pale to pink ascomata and muriform ascospores with attenuated ends; on steep faces of calcareous cliffs, in overhangs or in sheltered places on stone walls; most common in Western Europe, for the Alps reported from a few localities only. – **Au**: T. **Fr**: AHP, AMa.

***Gyalecta nigritella* Cl. Roux & M. Bertrand**

L # – Subs.: cal – Alt.: 2 – Note: a species resembling the Mediterranean *G. thelotremella* Bagl., but apothecia with black discs and margins, and ascospores smaller and submuriform; on dolomitic boulders; only known from the type locality in the Western Alps (at 1,265 m). – **Fr**: AMa.

***Gyalecta ophiospora* (Lettau) Baloch & Lücking**

Syn.: *Pachyphiale ophiospora* Lettau

L – Subs.: cor – Alt.: 2–3 – Note: a species of the *G. cornea*-group (asci polyspored and ascospores acicular), with spiral-shaped ascospores in twisted arrangement, found on bark of broad-leaved trees in montane forests; widespread in temperate Europe, including the Alps, but rare. – **Au**: V, S, O, N. **Sw**: FR, SZ.

***Gyalecta peziza* (Mont.) Anzi**

Syn.: *Biatora peziza* Mont., *Secoliga peziza* (Mont.) Arnold

L – Subs.: ter-cal, bry-cal – Alt.: 3–5 – Note: a mainly arctic-alpine, circumpolar species found on slightly calciferous soil rich in humus, and on terricolous bryophytes near or above treeline; widespread throughout the Alps, but generally rare. – **Au**: T, S, K. **Sw**: GR, VS. **Fr**: HAl. **It**: TAA, Lomb, Piem.

***Gyalecta rosea* (Schaer.) ined. (provisionally placed here, ICN Art. 36.1b)**

Syn.: *Lecidea rosea* Schaer.

L # – Subs.: cal – Alt.: 3 – Note: a species with a thin, white, rough thallus and urceolate, immersed apothecia with rose to flesh-coloured discs crowned by a thalline margin; the microscopic characters are unknown and the generic placement is in need of re-evaluation; this could possibly be the oldest name for one of the calcicolous *Gyalecta*-species; on calcareous rock at high elevation, only known from the type locality in the Western Alps (Switzerland). – **Sw**: BE.

***Gyalecta russula* (Körb. *ex* Nyl.) Baloch, Lumbsch & Wedin**

Syn.: *Belonia fennica* Vain., *Belonia russula* Körb. *ex* Nyl., *Beloniella cinerea* Norman, *Gyalecta bacidiospora* (Eitner) Zahlbr., *Secoliga bacidiospora* Eitner

L – Subs.: sil, ter-sil, bry – Alt.: 3–5 – Note: a mainly arctic-alpine, probably circumpolar species found on base-rich soil, often on bryophytes, and on steeply inclined or underhanging surfaces of basic siliceous rocks, with optimum above treeline; perhaps more widespread in the Alps, but generally not common. – **Au**: T, S, St. **Ge**: OB. **Fr**: AMa. **It**: Frl, TAA.

***Gyalecta sbarbari* Vězda**

L – Subs.: cor, cal – Alt.: 1–3 – Note: a rare species resembling *G. truncigena*, but with longer, fusiform ascospores (26–30 × 6–7 μm), with mostly 9–11 transversal septa and 1 incomplete longitudinal septum; the type was on calcareous rocks in a humid-shaded site near the coast of Liguria, but the typical substrate seems to be bark; records from Austria need critical re-evaluation. – **Au**: K, O. **It**: Lig.

***Gyalecta subclausa* Anzi**

Syn.: *Gyalecta chlorobaea* Nyl., *Gyalecta elegantula* Müll. Arg., *Gyalecta rosellovirens* Nyl.

L – Subs.: cal – Alt.: 3–5 – Note: an inconspicuous, perhaps overlooked species found on vertical faces of calcareous rocks in humid, damp and shaded situations in upland areas. – **Au**: ?V, St. **Fr**: AHP, AMa, HSav, Var, Vau. **It**: Frl, Lomb. **Sl**: Tg.

***Gyalecta sudetica* Vězda**

L – Subs.: int – Alt.: 5 – Note: a species of the *G. leucaspis*-group, but apothecial margins entire rather than radially incised as in *G. erythrozona*, differing from the latter in the submuriform ascospores with attenuated ends, recalling those of *G. kukriensis*; on calcareous schists in the montane belt of Central European orobiomes, with a few records from the Eastern Alps (Austria). – **Au**: K, St.

***Gyalecta truncigena* (Ach.) Hepp**

Syn.: *Gyalecta abstrusa* (Wallr.) A. Massal., Gyalecta wahlenbergiana Ach. var. truncigena Ach.

L – Subs.: cor – Alt.: 1–3 – Note: a temperate lichen found on mature trees, mostly *Acer* and *Fraxinus*, but also on the slightly nutrient-enriched bark of more acid-barked trees, such as oaks, in mild-humid areas; more common in the past, presently localised in clearings of ancient, open, humid forests; widespread throughout the Alps, but generally not very common. – **Au**: V, T, S, K, St, O, N. **Ge**: OB. **Sw**: BE, FR, GR, TI, UR. **Fr**: AMa, Isè, HSav, Var, Vau. **It**: Ven, TAA, Lomb. **Sl**: SlA, Tg.

***Gyalecta ulmi* (Sw.) Zahlbr.**

Syn.: *Gyalecta rubra* (Hoffm.) A. Massal., *Haematomma rubrum* (Hoffm.) H. Olivier, *Lecania rubra* (Hoffm.) Müll. Arg., *Lecanora rubra* (Hoffm.) Ach., *Lepadolemma rubrum* (Hoffm.) Trevis., *Lichen ulmi* Sw., *Phialopsis rubra* (Hoffm.) Körb., *Phialopsis ulmi* (Sw.) Arnold

L – Subs.: cor, bry-cal – Alt.: 2–3 – Note: a warm-temperate lichen found on mature trees (especially near the base of *Ulmus*), but also on mosses on steeply inclined faces of calciferous rocks; apparently widespread throughout the Alps but rare, and probably more frequent in the past. – **Au**: V, T, S, K, St, O, N. **Ge**: OB. **Sw**: BE, GR, SG, SZ, UW, VD, VS. **Fr**: AHP, AMa, Isè, Sav, HSav, Var. **It**: Ven, Lomb, Piem. **Sl**: SlA.

***Gyalectidium setiferum* Vězda & Sérus.**

L – Subs.: fol – Alt.: 2 – Note: a species forming whitish-grey to greenish finely verrucose thalli provided with scattered sterile setae and hyphophores, only known in the sterile state; on leaves of evergreen shrubs and juvenile trees (*e.g. Buxus*, *Abies*) in the understory of very humid forests; widespread in Europe from Brittany in Western France to the Western Caucasus, with a few records from the Western Alps (France). – **Fr**: AMa.

***Gyalidea asteriscus* (Anzi) Aptroot & Lücking**

Syn.: *Solorinella asteriscus* Anzi

L – Subs.: ter-cal – Alt.: 2–4 – Note: a typical lichen of steppe grasslands on loess, whose distribution extends widely into Central Asia, found on loess and (in the Alps) on soil deriving from calcareous schists; restricted to strongly continental valleys in the Alps. – **Au**: T, S, K. **Sw**: GR, TI, VS. **Fr**: Sav. **It**: TAA, Lomb, Piem, VA.

***Gyalidea diaphana* (Körb *ex* Nyl.) Vězda**

Syn.: *Bacidia bayeri* (E. Senft) Servít, *Biatora diaphana* Körb. *ex* Nyl., *Biatorina diaphana* (Körb. *ex* Nyl.) Körb., *Catillaria bayeri* E. Senft, *Catillaria diaphana* (Körb. *ex* Nyl.) Lettau

L – Subs.: sil-aqu – Alt.: 3–5 – Note: a species recalling *G. fritzei*, but with 2-celled ascospores; on long-time inundated siliceous boulders in streams; widespread in the Arctic zone of Europe and in the alpine belt of European orobiomes, with a few records from the Eastern Alps, but perhaps overlooked elsewhere. – **Au**: St, O, N.

***Gyalidea fritzei* (Stein) Vězda**

Syn.: *Gyalecta fritzei* Stein

L – Subs.: sil – Alt.: 3–5 – Note: a species with a thin smooth thallus, sessile apothecia with concave discs and brown to blackish margins, and muriform ascospores, found on siliceous rocks in humid-shaded habitats, such as along creeks, with optimum above treeline; for the Alps it is known from several scattered localities. – **Au**: T, S, St. **Sw**: TI. **It**: Lomb.

***Gyalidea fruticola* M. Svenss. & G. Thor**

L – Subs.: cor – Alt.: 4 – Note: a species with small, whitish apothecia with minute discs, and submuriform, oblong ascospores; corticolous on basal branches of shrubs (*e.g. Lonicera*) in various forest types; apparently rather common in Scandinavia, with a single record from the Eastern Alps (Italy). – **It**: TAA.

***Gyalidea lecideopsis* (A. Massal.) Lettau *ex* Vězda var. lecideopsis**

Syn.: *Gyalecta albocrenata* Arnold, *Gyalecta hyalina* Hepp, *Gyalecta lecideopsis* A. Massal., *Gyalecta stigmatoides* (Nyl.) Boistel, *Gyalidea albocrenata* (Arnold) Lettau, *Gyalidea lecideopsis* (A. Massal.) Lettau *ex* Vězda var. stigmatoides (Nyl.) Vězda, *Lecidea stigmatoides* Nyl.

L – Subs.: cal, int – Alt.: 2–5 – Note: a northern-montane species found on limestone, dolomite, calciferous schists, on porous, damp faces; easily overlooked, but certainly rare in the Alps. – **Au**: V, T, S, St, O, N. **Ge**: OB, Schw. **Sw**: GR, SZ, UR. **Fr**: HSav. **It**: Ven, TAA, Piem.

***Gyalidea lecideopsis* (A. Massal.) Lettau *ex* Vězda var. convarians (Nyl.) Vězda**

Syn.: *Gyalecta convarians* Nyl., *Gyalidea lecideopsis* (A. Massal.) Lettau *ex* Vězda var. eucarpa (Servít) Vězda, *Lopadium cacuminum* H. Magn.

L – Subs.: cal – Alt.: 3 – Note: a taxon with reduced spore numbers per ascus and muriform ascospores which are longer than 30 µm, found on limestone and dolomite in moist places; widespread in Europe and also known from Asia and Arctic North America, but much rarer than the typical variety. – **Au**: V, T. **Ge**: OB. **It**: TAA.

***Gyalidea roseola* (Arnold) Lettau**

Syn.: *Gyalecta roseola* Arnold

L – Subs.: sil – Alt.: 3–4 – Note: on periodically wetted faces of siliceous rocks (especially crystalline schists) near creeks and waterfalls in upland areas; an overall rare species, known from a few localities in NW Europe and from the Alps. – **Au**: T, S. **It**: TAA.

***Gyalidea scutellaris* (Bagl. & Carestia) Lettau**

Syn.: *Gyalecta arctica* Malme, *Gyalecta pseudogeoica* Anzi, *Gyalecta scutellaris* Bagl. & Carestia

L – Subs.: sil, ter-sil – Alt.: 4–5 – Note: an arctic-alpine species found on humid, acid substrata, such as moribund bryophytes and soil rich in humus, with optimum above treeline. – **Au**: T, S. **Sw**: ?Sw. **It**: Ven, Lomb, Piem.

***Gyalidea subscutellaris* (Vězda) Vězda**

Syn.: *Gyalecta subscutellaris* Vězda

L – Subs.: deb – Alt.: 5 – Note: similar to *G. scutellaris*, but apothecia markedly smaller (less than 0.4 mm diam.) and with smaller muriform ascospores (≤ 20 µm); on acid to subneutral debris; rare in the European orobiomes, with a single record from the Eastern Alps (Austria). – **Au**: K.

***Gyalideopsis helvetica* van den Boom & Vězda**

L – Subs.: cor, xyl – Alt.: 3–4 – Note: a species with a smooth, greyish-green, glossy thallus and scattered, excavate soralia, apothecia (when present) reddish-brown, with submuriform, fusiform ascospores in mostly 4-spored asci; it grows on fallen, decorticated tree trunks; widespread in the Holarctic region but altogether rare, with a few scattered records from the Alps. – **Au**: K, St. **Sw**: SZ, VS. **It**: TAA.

***Gyalideopsis modesta* Vězda & Poelt**

L – Subs.: sil – Alt.: 2 – Note: a species recalling a *Gyalidea*, but interascal filaments branched and anastomosing, with less than 15 µm long, submuriform ascospores; on pebbles of siliceous schists in places with alternately moist and dry conditions, such as on talus of secondary roads; only known from the Eastern Alps, rare. – **Au**: St.

***Gyalideopsis piceicola* (Nyl.) Vězda & Poelt**

Syn.: *Gyalecta piceicola* (Nyl.) Arnold, *Gyalidea piceicola* (Nyl.) Lettau, *Lecidea piceicola* Nyl.

L – Subs.: cor, xyl – Alt.: 3–4 – Note: a species with a smooth, greyish-green, glossy thallus, minute, reddish – to blackish-brown apothecia, and 4-spored asci with submuriform, fusiform ascospores; on twigs of conifers (mostly *Picea*) in montane forests with frequent fog, usually in the lowermost canopy; widespread in the Holarctic region; in the Alps only known from scattered localities, but perhaps overlooked because of its very special ecology. – **Au**: T, S, K, St. **Sw**: BE, SZ.

***Gyalideopsis tuerkii* Vězda**

L – Subs.: ter-int – Alt.: 4 – Note: a species with a smooth, greyish-green thallus, relatively large, reddish-brown apotheci, and submuriform, fusiform ascospores; on debris of calcareous schists; so far only known from the Eastern Alps, near treeline. – **Au**: T.

***Gyalolechia aurea* (Schaer.) A. Massal.**

Syn.: *Caloplaca aurea* (Schaer.) Th. Fr., *Lecidea aurea* Schaer., *Thalloidima aureum* (Schaer.) Müll. Arg.

L – Subs.: ter-cal, cal – Alt.: 3–5 – Note: a species of the mountains of Central and Southern Europe, found on plant debris and mosses in fissures and cracks of calcareous rocks and dolomite, with optimum above treeline; widespread but not always common in the Alps. – **Au**: V, T, S, K, St, O, N. **Ge**: OB, Schw. **Sw**: BE, LU, UR, UW, VD, VS. **Fr**: AHP, HAl, HSav. **It**: Frl, Ven, TAA, Lomb, VA. **Sl**: SlA. **Li**.

**Gyalolechia bracteata (Hoffm.) A. Massal. subsp. bracteata**

Syn.: *Caloplaca bracteata* (Hoffm.) Jatta, Caloplaca bracteata (Hoffm.) Jatta f. alpina (Th. Fr.) Zahlbr., *Fulgensia bracteata* (Hoffm.) Räsänen, Fulgensia bracteata (Hoffm.) Räsänen var. alpina (Th. Fr.) Räsänen, *Lecanora bracteata* (Hoffm.) Ach., *Placodium bracteatum* (Hoffm.) Nyl., Placodium fulgens (Sw.) DC. var. alpinum Th. Fr., *Psora bracteata* Hoffm.

L – Subs.: ter-cal – Alt.: 2–6 – Note: frequently fertile, with non-septate ascospores; in the study area on soil over calcareous schists, usually on wind-exposed ridges. – **Au**: V, T, S, K, St, O, N. **Ge**: OB. **Sw**: GR, UR, VD, VS. **Fr**: AHP, HAl, AMa, Isè, Sav, HSav. **It**: Frl, Ven, TAA, Lomb, Piem, VA.

**Gyalolechia bracteata (Hoffm.) A. Massal. subsp. deformis (Erichsen) ined. (provisionally placed here, ICN Art. 36.1b)**

Syn.: Caloplaca bracteata (Hoffm.) Jatta var. deformis Erichsen, Fulgensia bracteata (Hoffm.) Räsänen subsp. deformis (Erichsen) Poelt

L – Subs.: ter-cal, bry – Alt.: 2–6 – Note: a usually sterile morph spreading by schizidia developing from the central squamules and exposing the white medulla after they have split off; on soil in fissures; in the Alps most common in the limestone mountain chains, in the extra-Alpine foreland mostly over gypsum soils. – **Au**: T, K, S, St, N. **Sw**: LU, SZ, VS.

***Gyalolechia delphinensis* (Poelt) Søchting, Frödén & Arup**

Syn.: *Fulgensia delphinensis* Poelt

L – Subs.: ter-cal – Alt.: 4–5 – Note: a speciess recalling *G. bracteata* in habitus, but with 1-septate asacospores; on gypsum soil in the Western Alps. – **Fr**: HAl, Sav.

***Gyalolechia desertorum* (Tomin) Søchting, Frödén & Arup**

Syn.: *Caloplaca desertorum* Tomin, *Caloplaca geoica* H. Magn., *Fulgensia desertorum* (Tomin) Poelt, *Placodium desertorum* Tomin

L – Subs.: cal, int, ter-cal – Alt.: 1–3 – Note: a species found in open grasslands on more or less calciferous soil, to be looked for further in dry-continental valleys of the Alps. – **Au**: T. **Sw**: VS. **Fr**: HAl.

***Gyalolechia epiphyta* (Lynge) Vondrák**

Syn.: *Caloplaca epiphyta* Lynge, *Caloplaca laricina* Rondon, *Caloplaca xanthostigmoidea auct. non* (Räsänen) Zahlbr., *Gyalolechia xanthostigmoidea auct. non* (Räsänen) Søchting, Frödén & Arup

L – Subs.: bry-cal, deb-cal, ter-cal, xyl – Alt.: 3–5 – Note: this lichen is widespread in the arctic and temperate-montane to alpine zones of the Northern Hemisphere, mainly in continental regions, being usually epiphytic or epixylic (often on *Juniperus*), but it also occurs on soil and mosses in rock crevices over calcareous rocks (*e.g.* on boulders visited by birds) in arctic-alpine habitats or in steppes; the relationships with *Caloplaca bryochrysion* await clarification. The epiphytic *Caloplaca xanthostigmoidea* is based on a type from Eastern North America and is apparently a different species. – **Au**: V, T, S, K, St. **Ge**: OB, Schw. **Sw**: GR, VS. **Fr**: AHP, HAl, AMa, Isè, Sav. **It**: Frl, Ven, TAA, Lomb, Piem. **Sl**: SlA.

***Gyalolechia flavorubescens* (Huds.) Søchting, Frödén & Arup**

Syn.: *Caloplaca aurantiaca auct. non* (Lightf.) Th. Fr., *Caloplaca flavorubescens* (Huds.) J.R. Laundon, Caloplaca flavovirescens (Wulfen) Dalla Torre & Sarnth. var. salicina (J.F.Gmel.) Dalla Torre & Sarnth., *Caloplaca salicina* (J.F. Gmel.) Szatala, *Caloplaca suberythrella* (Nyl.) Clauzade & Rondon, *Lichen flavorubescens* Huds.

L – Subs.: cor – Alt.: 1–3 – Note: a mainly temperate lichen, most common on old, more or less isolated deciduous trees, especially oaks; a member of a difficult and variable group; widespread throughout the Alps. – **Au**: T, S, K, St. **Sw**: BE, GR, LU, TI, UR, VD, VS. **Fr**: AHP, HAl, AMa, Isè, Sav, HSav, Var, Vau. **It**: Frl, Ven, TAA, Lomb, Piem, VA, Lig. **Sl**: Tg.

***Gyalolechia flavovirescens* (Wulfen) Søchting, Frödén & Arup**

Syn.: Callopisma aurantiacum (Lightf.) A. Massal. var. flavovirescens (Wulfen) A. Massal., Caloplaca aurantiaca (Lightf.) Th. Fr. var. flavovirescens (Wulfen) Th. Fr., Caloplaca aurantiaca (Lightf.) Th. Fr. var. inalpina (Ach.) H. Magn., *Caloplaca erythrella* (Ach.) Kieff., Caloplaca flavorubescens (Huds.) J.R. Laundon subsp. flavovirescens (Wulfen) Clauzade & Cl. Roux, *Caloplaca flavovirescens* (Wulfen) Dalla Torre & Sarnth., *Lecanora erythrella* (Ach.) Ach., *Lichen flavovirescens* Wulfen, Placodium aurantiacum (Lightf.) Anzi var. flavovirescens (Wulfen) Hepp, Placodium aurantiacum (Lightf.) Anzi var. inalpinum (Ach.) H. Magn.

L – Subs.: cal, int – Alt.: 1–5 – Note: a mainly temperate species growing on weakly calcareous sandstone and calciferous schists, on boulders and walls, with optimum at relatively low elevations; widespread throughout the Alps. – **Au**: V, T, S, K, St, O, N, B. **Ge**: OB, Schw. **Sw**: BE, GR, LU, SZ, VD, VS. **Fr**: AHP, HAl, AMa, Drô, Isè, Sav, HSav, Var, Vau. **It**: Frl, Ven, TAA, Lomb, Piem, VA, Lig. **Sl**: Tg.

***Gyalolechia fulgens* (Sw.) Søchting, Frödén & Arup**

Syn.: *Caloplaca fulgens* (Sw.) Körb., *Fulgensia fulgens* (Sw.) Elenkin, *Lecanora fulgens* (Sw.) Ach., *Lichen fulgens* Sw., *Placodium fulgens* (Sw.) DC., *Psoroma fulgens* (Sw.) A. Massal., *Squamaria fulgens* (Sw.) Hook.

L – Subs.: cal, ter-cal – Alt.: 1–4 – Note: a subtropical to temperate lichen found on calcareous rocks and thin layers of soil, often in rock fissures, usually below the subalpine belt. – **Au**: T, St, N. **Sw**: GR, TI, VD, VS. **Fr**: AHP, HAl, AMa, Drô, Isè, Sav, HSav, Var, Vau. **It**: Ven, TAA, Lomb, Piem, VA.

***Gyalolechia fulgida* (Nyl.) Søchting, Frödén & Arup**

Syn.: *Caloplaca fulgida* (Nyl.) J. Steiner, *Fulgensia fulgida* (Nyl.) Szatala, *Lecanora fulgida* (Nyl.) Hue, *Placodium fulgidum* Nyl.

L – Subs.: cal, ter-cal – Alt.: 1–2 – Note: a mainly Mediterranean lichen found on rock, especially in fissures, more rarely on soil in dry grasslands, with several records from the base of the Western Alps (France, Italy). – **Fr**: AHP, AMa, Drô, Isè, Var, Vau. **It**: Lig.

***Gyalolechia klementii* (Kalb) Søchting, Frödén & Arup**

Syn.: *Fulgensia klementii* Kalb

L – Subs.: bry-cal – Alt.: 3 – Note: a species recalling *G. pruinosa*, but the effigurate marginal lobes lemon-yellow, and ascospores simple, with pointed ends; based on a type from Spain and also recorded from Greece; on soil or encrusting calcicolous bryophytes, with a few lowland records from the Western Alps. – **Sw**: LU. **Fr**: AMa.

**Gyalolechia pruinosa Körb. var. pruinosa**

Syn.: *Caloplaca pruinosa* (Körb.) Zahlbr., *Fulgensia pruinosa* (Körb.) Poelt

L – Subs.: cal, ter-cal – Alt.: 3–5 – Note: on steeply inclined to underhanging faces of calcareous rocks, mostly in fissures, sometimes on epilithic bryophytes, with optimum above treeline. – **Au**: V, T, K, St, O. **Ge**: OB, Schw. **Sw**: GR, SZ. **Fr**: AHP, HAl. **It**: TAA, Lomb. **Sl**: SlA.

**Gyalolechia pruinosa Körb. var. fissiseda (Poelt) ined. (provisionally placed here, ICN Art. 36.1b)**

Syn.: Caloplaca aurea (Schaer.) Th. Fr. var. fissiseda Poelt, Fulgensia pruinosa (Körb.) Poelt var. fissiseda Poelt

L – Subs.: ter-cal, cal – Alt.: 5 – Note: on the top of birds’ perching boulders; for the study area only reported from Austria, but perhaps not distinguished elsewhere. – **Au**: V, T, S, St.

***Gyalolechia subbracteata* (Nyl.) Søchting, Frödén & Arup**

Syn.: *Caloplaca subbracteata* (Nyl.) Lettau, Fulgensia fulgens (Sw.) Elenkin f. subbracteata (Nyl.) Nimis, *Fulgensia sorediosa* Klem., *Fulgensia subbracteata* (Nyl.) Poelt, *Lecanora subbracteata* Nyl.

L # – Subs.: ter-cal – Alt.: 1–3 – Note: on calciferous ground, in clearings of grasslands and shrublands, with optimum in the Mediterranean belt. A critical taxon, characterised by schizidia, which, however, also occur in other related species. According to Roux et al. (2014), it cannot be separated from *G. fulgens*, the schizidia being just a re-generation form from damages to the thallus, but molecular data show that the two taxa are distinct (see Nimis 2016). – **Sw**: VS. **It**: Ven, TAA, Piem, Lig.

***Gypsoplaca macrophylla* (Zahlbr.) Timdal**

Syn.: *Lecidea macrophylla* Zahlbr.

L – Subs.: int – Alt.: 4 – Note: a species with an olive-brown, squamiform thallus bearing laminal, undelimited, reddish-brown ascomata; on soil layers over calcareous schists under periodically xeric conditions leading to steppe vegetation; widespread in the Holarctic region, but with a single locality in the Eastern Alps. – **Au**: T.

***Gyrographa gyrocarpa* (Flot.) Ertz & Tehler**

Syn.: *Graphis gyrocarpa* (Flot.) Spreng., *Opegrapha gyrocarpa* Flot., Opegrapha saxicola Ach. var. gyrocarpa (Flot.) Stizenb.

L – Subs.: sil – Alt.: 2–4 – Note: on steeply inclined to underhanging surfaces of siliceous rocks, often in forests, in cold-humid situations, more rarely on subacid bark; widespread throughout the siliceous Alps. – **Au**: V, T, S, K, St, N. **Sw**: UR, VS. **Fr**: AHP, AMa, HSav, Var, Vau. **It**: Frl, Ven, TAA, Lomb, Piem, Lig. **Sl**: SlA.

**Haematomma ochroleucum (Neck.) J.R. Laundon var. ochroleucum**

Syn.: *Haematomma coccineum* (Dicks.) Körb., *Haematomma leiphaemum* (Ach.) Zopf, *Haematomma vulgare* A. Massal., *Lecanora haematomma* Ach. *nom.illeg.*, *Lepra leiphaema* (Ach.) Mérat, *Lichen ochroleucus* Neck.

L – Subs.: cor, sil – Alt.: 1–4 – Note: a mild-temperate species found on steeply inclined to underhanging, somehow rain-protected surfaces of siliceous rocks, but also on bark in humid forests; widespread throughout the Alps. – **Au**: V, T, S, K, St, O, N, B. **Ge**: OB, Schw. **Sw**: BE, GL, GR, LU, SG, SZ, TI, UR, UW, VD, VS. **Fr**: AMa, Isè, HSav, Vau. **It**: Frl, Ven, TAA, Lomb, Piem. **Sl**: SlA.

**Haematomma ochroleucum (Neck.) J.R. Laundon var. porphyrium (Pers.) J.R. Laundon**

Syn.: Haematomma coccineum (Dicks.) Körb. var. porphyrium (Pers.) Th. Fr, *Haematomma porphyrium* (Pers.) Zopf, Lichen haematomma Ehrh. var. porphyrius Pers.

L – Subs.: sil – Alt.: 1–5 – Note: a mild-temperate taxon, much rarer on bark than the typical variety, and perhaps slightly less photophytic. – **Au**: V, T, S, K, St, N. **Sw**: UR. **Fr**: AHP. **It**: Ven, TAA, Lomb, Piem.

***Halecania alpivaga* (Th. Fr.) M. Mayrhofer**

Syn.: *Lecania alpivaga* Th. Fr., *Lecania thallophila* H. Magn.

L – Subs.: sil, int, int-par – Alt.: 3–5 – Note: a cool-temperate to arctic-alpine species found on weakly calciferous and basic siliceous rocks in humid situations, frequently parasitic on various species of *Placynthium* (but also observed on *Collema*); probably more widespread in the Alps. – **Au**: T, S, K, St, N. **Sw**: GR, SZ. **Fr**: AHP. **It**: TAA, Lomb. **Sl**: SlA.

***Halecania elaeiza* (Nyl.) M. Mayrhofer**

Syn.: *Lecanora elaeiza* Nyl.

L – Subs.: cal – Alt.: 3–4 – Note: a species with relatively small ascospores (< 15 µm long) found on calcareous rocks in upland areas; altogether rare in Eastern and Central Europe, including the Alps. – **Au**: T, N. **It**: TAA.

***Halecania lecanorina* (Anzi) M. Mayrhofer & Poelt**

Syn.: *Diphratora disparata* Jatta *nom.illeg.*, *Gyalolechia lecanorina* (Anzi) Anzi, *Lecania disparata* Lettau *nom. illeg*, *Lecania lecanorina* (Anzi) Zahlbr., *Lecaniella disparata* Jatta *nom.illeg.*, *Lecanora disparata* Nyl. *nom.illeg.*, *Thalloidima disparatum* Arnold *nom.illeg.*, *Thalloidima lecanorinum* Anzi, *Toninia lecanorina* (Anzi) H. Olivier

L – Subs.: bry, ter-cal, deb – Alt.: 4–5 – Note: on thin layers of soil, on mosses and plant debris over calcareous substrata, with optimum near treeline; perhaps more widespread in the Alps, but certainly not common. – **Au**: T, S, K, St, O, N. **Ge**: OB. **Sw**: GR, SG. **It**: Lomb, Piem, VA.

***Halecania pannarica* M. Brand & van den Boom**

L – Subs.: sil-par – Alt.: 3–4 – Note: a species resembling *H. giraltiae*, with a thallus composed of small squamules (Pd+ orange), laminal, bluish-black soralia (small apothecia occasionally present); on boulders and outcrops of various types of schists, often parasitic on *Aspicilia*-species (rarely on *Acarospora* and *Rhizocarpon*); so far known from a few localities in the Western Alps. – **Sw**: UR, VS. **Fr**: AMa.

***Halecania spodomela* (Nyl.) M. Mayrhofer**

Syn.: *Lecania spodomela* (Nyl.) A.L. Sm., *Lecanora spodomela* Nyl., *Lecidea nigrificans* Nyl.

L – Subs.: sil-par – Alt.: 2–3 – Note: a silicicolous species resembling *H. alpivaga*, but with a strongly reduced thallus and smaller apothecia, usually parasitic on *Placynthium*; in Western Europe it is mainly coastal, and the central and eastern European historical records are in need of critical re-evaluation; in the study area it is known from a single locality in the Western Alps. – **It**: Piem.

***Halecania viridescens* Coppins & P. James**

L – Subs.: cor – Alt.: 2–3 – Note: a species recalling *Rinodina efflorescens* in appearance, but the pale green to greenish-brown areoles are very fragile, dissolving into pale vivid-green soralia with farinose soredia (Pd+ red), apothecia occasionally present, with relatively thin ascospores; on slightly eutrophic bark of deciduous trees (mainly *Salix*) often near rivers and lakes; most frequent in Western Europe, with a few records from the Eastern Alps, but perhaps not always recognised elsewhere. – **Au**: K, O. **Ge**: OB.

***Harpidium rutilans* Körb.**

Syn.: *Acarospora rutilans* (Körb.) Hue, *Zeora rutilans* Flot. *nom. nud*.

L – Subs.: sil – Alt.: 1–4 – Note: on steeply inclined surfaces of siliceous rocks; both in the Mediterranean region and in dry-warm valleys of the Alps, perhaps overlooked, but certainly not common. – **Au**: T. **Sw**: GR. **Fr**: Var. **It**: TAA.

***Helocarpon crassipes* Th. Fr.**

Syn.: *Lecidea crassipes* (Th. Fr.) Nyl., *Micarea crassipes* (Th. Fr.) Coppins

L – Subs.: bry, deb – Alt.: 4 – Note: thallus composed of small granules dispersed in a pale hypothallus, encrusting bryophytes in arctic-alpine environments; widespread in Fennoscandia, presence in the Central European mountains uncertain, and records from the Alps in need of verification. – **Ge**: OB. **Sw**: GR.

***Helocarpon pulverulum* (Th. Fr.) Türk & Hafellner**

Syn.: *Helocarpon crassipes auct. eur. merid. non* Th. Fr., Lecidea crassipes (Th. Fr.) Nyl. f. pulverula Th. Fr., *Micarea crassipes auct. eur. merid. non* (Th. Fr.) Coppins

L # – Subs.: bry, deb – Alt.: 4–5 – Note: thallus composed of densely arranged pulverulent granules, taxonomic value in need of re-evaluation; on bryophytes and plant debris on the ground and amongst rocks, in areas with siliceous substrata, with optimum near treeline; widespread in the Holarctic region, and fairly common in the Eastern Alps. – **Au**: V, T, S, K, St, O, N. **Ge**: OB. **It**: Frl, Ven, TAA.

***Henrica melaspora* (Taylor) Savić & Tibell**

Syn.: *Anthracothecium melasporum* (Taylor) Müll. Arg., *Polyblastia melaspora* (Taylor) Zahlbr., ?*Polyblastia plotocarpa* Norman *ex* Zschacke, *Polyblastia scotinospora* (Nyl.) Hellb., *Polyblastia subinumbrata* (Nyl.) A.L. Sm., *Verrucaria melaspora* Taylor

L – Subs.: sil, int – Alt.: 3–6 – Note: on siliceous to somewhat calcareous, wet rocks in open situations, often on slate, usually along rivers or by lakeshores or on pebbles at least intermittently flushed with running water, near or above treeline. – **Au**: T, S, K, St. **Sw**: VS. **Fr**: AMa. **It**: TAA, Piem.

***Henrica theleodes* (Sommerf.) Savić, Tibell & Nav.-Ros.**

Syn.: *Henrica ramulosa* B. de Lesd., *Polyblastia theleodes* (Sommerf.) Th. Fr., *Verrucaria theleodes* Sommerf.

L – Subs.: int, sil – Alt.: 4–6 – Note: in niches and fissures of humid calcareous schists and granodiorite, usually along rivers or by lakeshores, often occurring together with cyanobacterial lichens, usually above treeline; known from Scandinavia, Iceland, Greenland, the Alps and the Pyrenees, and from Colorado in North America. – **Au**: T, S, St. **Fr**: AMa, Sav, HSav. **It**: TAA, Lomb, Piem, VA.

***Henrica vallorcinensis* (Croz.) ined. (provisionally placed here, ICN Art. 36.1b)**

Syn.: *Polyblastia vallorcinensis* (Croz.) Zschacke, *Verrucaria vallorcinensis* Croz.

L # – Subs.: cal, sil – Alt.: 4–5 – Note: a species with a thin, grey to brown thallus, protruding hemispherical ascomata with a descending involucrellum, and finally brown, muriform ascospores; perhaps close to or even identical with *H. melaspora*; on humid faces of siliceous rocks along river banks; only known from the type locality in the Western Alps (France), and from Switzerland, near the border with France (Haute Savoie). – **Sw**: VS. **Fr**: HSav.

***Heppia adglutinata* (Kremp.) A. Massal.**

Syn.: *Heppia urceolata* (Schaer.) Nägeli, *Heppia virescens* (Mont.) Nyl., *Lecanora adglutinata* Kremp., *Nylanderopsis salevensis* Gyeln., *Solorina virescens* Mont.

L – Subs.: ter-cal – Alt.: 2–5 – Note: a cool-temperate to boreal-montane, circumpolar, ephemeral lichen of disturbed calciferous soil in dry, open grasslands; some records of *H. lutosa* might belong here. – **Au**: V. **Ge**: OB. **Sw**: GR, SZ, TI, VS. **Fr**: AHP, HAl, AMa, HSav, Vau. **It**: Piem, Lig.

***Heppia lutosa* (Ach.) Nyl.**

Syn.: *Collema lutosum* Ach., *Collema sanguinolentum* (Kremp.) Stizenb., *Heppia atlantica* Gyeln.

L – Subs.: ter-cal – Alt.: 1–5 – Note: a mainly Mediterranean-Atlantic lichen found on more or less calciferous soil in dry grasslands below the montane belt; it was often confused, in the older literature, with *H. adglutinata*, which is bound to upland areas; widespread throughout the Alps. – **Au**: V, T, S, K, St, N. **Sw**: GR, LU, SZ, TI, UR, VD, VS. **Fr**: AHP, AMa, Var, Vau. **It**: Frl, Ven, TAA, Lomb, Piem, VA, Lig. **Sl**: Tg. **Li**.

***Hertelidea botryosa* (Fr.) Printzen & Kantvilas**

Syn.: *Biatora botryosa* Fr., *Lecidea botryosa* (Fr.) Th. Fr.

L – Subs.: cor, xyl – Alt.: 3–4 – Note: a probably circumboreal-montane to cool-temperate lichen found on lignum, often on burnt trunks of conifers and *Quercus*, more rarely on acid bark, usually in upland areas but below treeline; with several scattered records from the Alps. – **Au**: N. **Fr**: HSav. **It**: Frl, Lomb, VA.

***Heterodermia obscurata* (Nyl.) Trevis.**

Syn.: *Anaptychia hypoleuca auct. non* (Muhl.) A. Massal., *Anaptychia obscurata* (Nyl.) Vain., *Anaptychia sorediifera* (Müll. Arg.) Du Rietz & Lynge, *Heterodermia hypoleuca auct. non* (Muhl.) Trevis., *Physcia obscurata* Nyl., *Pseudophyscia speciosa auct. non* (Wulfen) Müll. Arg.

L – Subs.: cor – Alt.: 1–3 – Note: a mild-temperate species found on more or less isolated trees, occasionally on epilithic mosses. See also note to *Polyblastidium subneglectum*. – **Au**: V, T, S, O. **Ge**: Ge. **Sw**: UW. **It**: Ven, TAA, Lig.

***Heterodermia speciosa* (Wulfen) Trevis.**

Syn.: *Anaptychia speciosa* (Wulfen) A. Massal., *Lichen speciosus* Wulfen, *Parmelia speciosa* (Wulfen) Ach., *Physcia speciosa* (Wulfen) Nyl., *Pseudophyscia speciosa* (Wulfen) Müll. Arg.

L – Subs.: cor, bry-sil – Alt.: 1–4 – Note: a temperate species found on bark, epiphytic bryophytes, sometimes on mossy rocks in humid, mostly montane woodlands; widespread throughout the Alps, but generally rather rare. – **Au**: V, T, S, K, St, O, N. **Ge**: OB, Schw. **Sw**: BE, GL, GR, SG, SZ, TI, UR, UW, VS. **Fr**: AHP, AMa, Sav, HSav, Vau. **It**: Frl, Ven, TAA, Lomb, Piem, VA, Lig. **Sl**: SlA, Tg.

***Heteroplacidium compactum* (A. Massal.) Gueidan & Cl. Roux**

Syn.: *Catapyrenium compactum* (A. Massal.) R. Sant., *Dermatocarpon compactum* (A. Massal.) Blomb. & Forssell, *Dermatocarpon crassulum* (Müll. Arg.) Zahlbr., *Endopyrenium crassulum* Müll. Arg., *Placidium compactum* A. Massal., *Rhodocarpon compactum* (A. Massal.) Lönnr., *Verrucaria compacta* (A. Massal.) Jatta

L – Subs.: cal, sil – Alt.: 1–5 – Note: on more or less calcareous rocks, sometimes on other crustose lichens, but not parasitic, usually in upland areas but below treeline. This name probably includes several taxa related to *H. fusculum*, whose taxonomic status is in need of clarification. – **Au**: V, T, S, K, St, O, N. **Sw**: GR, SZ. **Fr**: AHP, AMa, HSav. **It**: TAA, Piem, Lig.

***Heteroplacidium contumescens* (Nyl.) Breuss**

Syn.: *Catapyrenium contumescens* (Nyl.) Breuss, *Dermatocarpon contumescens* (Nyl.) Zahlbr., *Endocarpon contumescens* Nyl.

L – Subs.: cal, ter – Alt.: 1 – Note: on steeply inclined to underhanging surfaces of base-rich or calciferous rocks, sometimes on soil in rock fissures, mostly in warm-dry situations, *e.g.* in grasslands and garrigues; in the study area only known from the Western Alps (France). – **Fr**: Vau.

***Heteroplacidium divisum* (Zahlbr.) Breuss**

Syn.: *Catapyrenium divisum* (Zahlbr.) Breuss, *Dermatocarpon divisum* Zahlbr.

L – Subs.: ter – Alt.: 1 – Note: a Mediterranean species recalling *H. imbricatum* but with much thinner and strongly divided squamules, found on base-rich soil over siliceous substrata in dry sites; hitherto known only from Southern Europe (Italy, SE Spain, Balkan Peninsula). – **It**: Ven.

***Heteroplacidium fusculum* (Nyl.) Gueidan & Cl. Roux**

Syn.: *Dermatocarpon insulare* (A. Massal.) Mig., *Dermatocarpon nantianum* (H. Olivier) Zahlbr., *Endocarpon insulare* (A. Massal.) A. Massal., *Endocarpon nantianum* H. Olivier, *Endopyrenium insulare* (A. Massal.) Dalla Torre & Sarnth., *Placidium insulare* A. Massal., *Verrucaria fuscula* Nyl., *Verrucaria insularis* (A. Massal.) Jatta, Verrucaria insularis (A. Massal.) Jatta var. major Zehetl.

L – Subs.: cal, cal-par – Alt.: 1–5 – Note: a calcicolous species with a crustose-areolate, dark brown thallus but otherwise with a *Heteroplacidium* anatomy, growing on taxa of the *Aspicilia calcarea*-group, but finally often becoming independent; widespread and fairly common in the Mediterranean region, with some outposts in Central Europe; apparently more frequent in the Southern and Western Alps, mostly in the lowlands. – **Ge**: OB. **Fr**: AHP, HAl, AMa, Drô, HSav, Var, Vau. **It**: Frl, Ven, TAA, Lomb, Piem, Lig.

***Heteroplacidium imbricatum* (Nyl.) Breuss**

Syn.: *Catapyrenium imbricatum* (Nyl.) Clauzade & Cl. Roux, *Dermatocarpon imbricatum* (Nyl.) Zahlbr., *Endocarpon imbricatum* Nyl., *Endopyrenium imbricatum* (Nyl.) Boistel

L – Subs.: ter-cal – Alt.: 1–2 – Note: in fissures of hard calcareous rocks and amongst mosses, especially limestone, in rather sheltered situations, at low elevations; apparently more frequent in the Southern and Western Alps. – **Fr**: AHP, AMa, Drô, Var, Vau. **It**: Frl, Ven, Lig.

***Heteroplacidium zamenhofianum* (Clauzade & Cl. Roux) Cl. Roux**

Syn.: *Dermatocarpon compactum* *sensu* Clauzade & Rondon, *Verrucaria zamenhofiana* Clauzade & Cl. Roux

L – Subs.: cal-par – Alt.: 4–5 – Note: a species with a crustose, dark brown thallus with incised to sublobate areoles, growing on taxa of the *Staurothele areolata*-group on slightly inclined to subhorizontal surfaces of calcareous rocks in upland areas; widespread in Europe and North America, as well as in the Alps; it is easy to overlook because the thalli of host and parasite are concolorous. – **Au**: V, T. **Fr**: AHP, HAl, AMa, Isè, Sav, HSav. **It**: Frl, Ven, TAA, Piem, VA, Lig.

***Hydropunctaria amphibia* (Clemente) Cl. Roux**

Syn.: *Verrucaria amphibia* Clemente, *Verrucaria symbalana* Nyl.

L – Subs.: sil-aqu, cal-aqu – Alt.: 1 – Note: a species with a green-brown to brown-black, cracked thallus provided with abundant black ridges, flat-topped, crenate perithecia, and narrowly ellipsoid ascospores (l/w ratio ≥ 2.5); on both siliceous and calcareous seashore rocks; common and widespread along the coasts of the northern Atlantic Ocean, rare in the Mediterranean region, with a few records from the base of the Western Alps (France, Italy). – **Fr**: AMa. **It**: Lig.

***Hydropunctaria maura* (Wahlenb.) C. Keller, Gueidan & Thüs**

Syn.: *Involucrothele magnussonii* Servít, *Verrucaria haeyrenii* Erichsen, *Verrucaria malmei* Servít, *Verrucaria maura* Wahlenb., *Verrucaria trachinodes* Norman, *Verrucaria zschackeana* Erichsen

L – Subs.: sax – Alt.: 1 – Note: a species with a dull brown to black, cracked thallus provided with abundant black dots, immersed to protruding perithecia, and ellipsoid ascospores (l/w ratio ≤ 2.0); on both siliceous and calcareous seashore rocks, forming a conspicuous belt at the upper edge of the littoral zone; widespread and common, subcosmopolitan, including the Mediterranean region at the base of the Western Alps, along the coast. – **It**: Lig.

***Hydropunctaria rheitrophila* (Zschacke) C. Keller, Gueidan & Thüs**

Syn.: *Verrucaria cinereolutescens* Zschacke, *Verrucaria kernstockii* Zschacke, *Verrucaria minutipuncta* Erichsen, *Verrucaria rheitrophila* Zschacke, *Verrucaria scotinodes* Zschacke

L – Subs.: sil-aqu, cal-aqu – Alt.: 2–5 – Note: a species with an olive-green to orange-brown thallus, in longitudinal section usually with carbonised dots or/and columns, and perithecia with a basally open involucrellum; on both siliceous and calcareous rocks in cold, fast-running streams or in permanently submerged to frequently wetted places; widespread in the Holarctic region and also known from the Southern Hemisphere; also widespread in the Alps, but not common. – **Au**: T, S, K, St, N. **Sw**: GR. **Fr**: AHP, AMa, Var. **It**: Ven, TAA. **Sl**: SlA.

***Hydropunctaria scabra* (Vězda) C. Keller, Gueidan & Thüs**

Syn.: *Verrucaria scabra* Vězda

L – Subs.: sil-aqu – Alt.: 4–5 – Note: a species differing from *H. rheitrophila* by an involucrellum enclosing entirely the perithecium and reaching the thallus base, where it fuses with the carbonised basal layer; on siliceous rocks in the amphibious zone of streams, often near rapids and waterfalls, but also on lake shores; widespread in Europe but only locally abundant; with a few records from the Eastern Alps, but perhaps overlooked or misidentified as *H. rheitrophila* elsewhere. – **Au**: T, S, K. **Fr**: AMa.

***Hymenelia aigneri* (Zahlbr.) Hafellner & Türk**

Syn.: *Ionaspis aigneri* Zahlbr.

L # – Subs.: cal – Alt.: 3–5 – Note: a species with a peach-flower coloured to pure red thallus containing a trentepohlioid photobiont, black apothecia, and hymenium bright red in the upper part, reacting N+ purple; on calcareous stones *e.g.* on screes in the montane belt; distribution insufficiently known; in the study area only reported from two localities in the Eastern Alps. – **Au**: ?V, N.

***Hymenelia carnosula* (Arnold) Lutzoni**

Syn.: *Aspicilia carnosula* Arnold, *Ionaspis carnosula* (Arnold) Arnold

L – Subs.: cal – Alt.: 3–4 – Note: a species of the *H. epulotica*-group with a white thallus containing a trentepohlioid photobiont, pale flesh-coloured apothecia, and subglobose ascospores ≤ 10 µm in diam.; on limestone below or near treeline; distribution insufficiently known; in the study area only reported from the Eastern Alps. – **Au**: ?V, K, St, N. **Ge**: OB, Schw.

***Hymenelia coerulea* A. Massal.**

Syn.: *Aspicilia coerulea* (A. Massal.) Lindau, *Hymenelia hiascens* A. Massal., *Lecanora cantiana* (Garov.) Zahlbr., *Lecanora coerulea* (A. Massal.) Jatta, *Lecanora pseudocoerulea* Zahlbr., *Manzonia cantiana* Garov.

L – Subs.: cal – Alt.: 2–6 – Note: on steeply inclined surfaces of hard calciferous rocks, including moderately dolomitic, hard limestone; certainly widespread and locally abundant throughout the Alps, with optimum in the montane and subalpine belts. – **Au**: V, T, S, K, St, O, N. **Ge**: OB, Schw. **Sw**: BE, GR, LU, SZ, UW, VD, VS. **Fr**: AHP, AMa, Drô, Isè, Sav, HSav, Var, Vau. **It**: Frl, Ven, TAA, Lomb. **Sl**: SlA, Tg.

***Hymenelia cyanocarpa* (Anzi) Lutzoni**

Syn.: *Aspicilia cyanocarpa* Anzi, *Ionaspis cyanocarpa* (Anzi) Jatta

L – Subs.: sil-aqu – Alt.: 3–5 – Note: on periodically inundated, hard siliceous rocks, with optimum above treeline; probably more widespread in the Alps. – **Au**: S, St. **Fr**: Sav, HSav. **It**: TAA, Lomb.

***Hymenelia epulotica* (Ach.) Lutzoni**

Syn.: *Aspicilia epulotica* (Ach.) Anzi, *Gyalecta epulotica* Ach., *Ionaspis epulotica* (Ach.) Blomb. & Forssell, Ionaspis epulotica (Ach.) Blomb. & Forssell var. patellula (Arnold) H. Magn., *Pinacisca epulotica* (Ach.) Trevis

L – Subs.: cal – Alt.: 1–5 – Note: an arctic-alpine to cool-temperate, circumpolar species found on hard, compact calciferous rocks such as limestone, dolomite, calcareous schists, in sheltered-humid situations; widespread throughout the Alps. – **Au**: V, T, S, K, St, O, N. **Ge**: OB, Schw. **Sw**: BE, GR, LU, SZ, UR, UW, VD, VS. **Fr**: AHP, HAl, AMa, Drô, Isè, Sav, HSav, Var, Vau. **It**: Frl, Ven, TAA, Lomb, Piem. **Sl**: SlA. **Li**.

***Hymenelia haematina* (Körb.) Lutzoni**

Syn.: *Aspicilia haematina* Körb., *Ionaspis haematina* (Körb.) Th. Fr.

L # – Subs.: met – Alt.: 4 – Note: a silicicolous species with a brown-red thallus, black, urceolate, immarginate apothecia, and subglobose ascospores of *c.* 10 µm in diam.; ecology and distribution are insufficiently known; in the study area only reported from a few localities of the Eastern Alps (Austria). – **Au**: ?T, K.

***Hymenelia heteromorpha* (Kremp.) Lutzoni**

Syn.: Aspicilia cinereorufescens (Ach.) A. Massal. var. heteromorpha Kremp., *Ionaspis annularis* H. Magn., *Ionaspis heteromorpha* (Kremp.) Arnold, *Ionaspis ochracella* (Nyl.) H. Magn., *Ionaspis reducta* H. Magn., Ionaspis rhodopsis (Sommerf.) Blomb. & Forssell var. melanopsis (Sommerf.) Zahlbr., *Ionaspis schismatopis* (Nyl.) Hue

L – Subs.: cal, int – Alt.: 3–5 – Note: a species with an epilithic, rimose, whitish thallus, small apothecia with black discs, and subglobose ascospores of *c.* 10 µm in diam.; on limestone and dolomite, widespread in Europe from the temperate to the arctic zone; widespread also in the Alps, but not common. – **Au**: V, T, S, K, St, O. **Ge**: OB, Schw. **Sw**: LU, SZ. **Fr**: HAl, Isè, Sav, Vau. **It**: Ven, TAA, Lomb, Piem.

***Hymenelia melanocarpa* (Kremp.) Arnold**

Syn.: Hymenelia prevostii (Duby) Kremp. var. melanocarpa Kremp., *Ionaspis cyrtaspis auct.*, *Ionaspis melanocarpa* (Kremp.) Arnold

L – Subs.: cal – Alt.: 3–5 – Note: a mainly arctic-alpine, circumpolar species, most frequent on hard, compact calciferous rocks in upland areas; widespread throughout the Alps. – **Au**: V, T, S, K, St, O, N. **Ge**: OB, Schw. **Sw**: BE, GR, SZ, UW, VD, VS. **Fr**: AHP, AMa, Isè, Sav, HSav, Vau. **It**: Frl, Ven, TAA, Piem, Lig. **Sl**: SlA, Tg.

***Hymenelia prevostii* (Duby) Kremp.**

Syn.: *Aspicilia prevostii* (Duby) Anzi, *Ionaspis prevostii* (Duby) Arnold, *Lecanora prevostii* (Duby) Th. Fr., *Lecidea prevostii* (Duby) Schaer., *Urceolaria prevostii* Duby

L # – Subs.: cal – Alt.: 2–5 – Note: on hard calcareous rocks, especially compact limestone. According to Roux et al. (2014), this is just a phycotype of *H. epulotica* with trebouxioid algae. – **Au**: V, T, S, K, St, O, N. **Ge**: OB. **Sw**: SZ. **Fr**: AHP, HAl, AMa, Drô, Sav, HSav, Var, Vau. **It**: Frl, Ven, TAA, Lomb, Piem, Lig.

***Hymenelia rhodopis* (Sommerf.) Lutzoni**

Syn.: *Ionaspis rhodopis* (Sommerf.) Blomb. & Forssell, Lecanora acharii (Ach.) Sommerf. var. rhodopis Sommerf.

L – Subs.: cal – Alt.: 3–5 – Note: a species with an epilithic, rimose, whitish thallus, small apothecia with pale pink discs, and broadly ellipsoid ascospores measuring 17–20 × 10–12 μm; common on calcareous stones; widespread in the Holarctic region, but not always distinguished from *H. epulotica* (with an endolithic thallus). – **Au**: V, T, S, St, N. **Sw**: SZ.

***Hymenelia similis* (A. Massal.) M. Choisy**

Syn.: *Aspicilia isabellina* Jatta, *Aspicilia similis* (A. Massal.) Anzi, *Ionaspis similis* (A. Massal.) Jatta, *Lecanora carneopallens* Nyl., *Lecanora similis* (A. Massal.) Nyl., *Pinacisca similis* A. Massal.

L – Subs.: cal – Alt.: 1–5 – Note: on shaded and steeply inclined surfaces of calciferous rocks, especially limestone and dolomite, descending to low altitudes in humid, coastal areas; widespread throughout the Alps. – **Au**: V, T, S, K, N. **Ge**: Ge. **Sw**: BE. **Fr**: AHP, AMa, Drô, HSav, Var, Vau. **It**: Ven, TAA, Lomb, Piem. **Sl**: Tg.

***Hyperphyscia adglutinata* (Flörke) H. Mayrhofer & Poelt**

Syn.: *Lecanora adglutinata* Flörke, *Physcia adglutinata* (Flörke) Nyl., *Physcia elaeina auct. non* (Wahlenb.) A.L. Sm., *Physciopsis adglutinata* (Flörke) M. Choisy

L – Subs.: cor – Alt.: 1–3 – Note: a widespread mild-temperate species found on isolated, mostly deciduous trees with nutrient-rich or – enriched bark, also in areas with intensive agriculture; widespread throughout the Alps, mainly at low elevations. – **Au**: V, T, S, K, St, O, N, B. **Ge**: OB. **Sw**: BE, FR, GR, LU, SG, TI, VD, VS. **Fr**: AHP, AMa, Drô, Isè, Sav, HSav, Var, Vau. **It**: Frl, Ven, TAA, Lomb, Piem, VA, Lig. **Sl**: SlA. **Li**.

***Hypocenomyce scalaris* (Ach.) M. Choisy**

Syn.: *Biatora ostreata* (Hoffm.) Th. Fr., *Lecidea ostreata* (Hoffm.) Schaer., *Lecidea scalaris* (Ach.) Ach., *Lichen scalaris* Ach., *Psora ostreata* Hoffm., *Psora scalaris* (Ach.) Hook.

L – Subs.: cor, xyl, sil – Alt.: 2–4 – Note: a temperate to boreal-montane, circumpolar lichen found on acid bark, especially of conifers, but also on *Castanea* and on lignum, including charred wood; widespread and locally common throughout the Alps. – **Au**: V, T, S, K, St, O, N, B. **Ge**: OB, Schw. **Sw**: GR, LU, SG, TI, UR, VD, VS. **Fr**: AHP, HAl, AMa, Isè, Sav, HSav, Var, Vau. **It**: Frl, Ven, TAA, Lomb, Piem, VA, Lig. **Sl**: SlA.

***Hypogymnia austerodes* (Nyl.) Räsänen**

Syn.: *Parmelia austerodes* Nyl., *Parmelia obscurascens* (Bitter) Zahlbr., *Parmelia obscurata* (Ach.) Bitter *non auct.*, Parmelia obscurata (Ach.) Bitter var. isidiata (Lynge) H. Magn.

L – Subs.: cor, xyl, sil – Alt.: 3–4 – Note: a mainly boreal-montane, circumpolar species found on acid bark, especially of conifers, and on lignum, occasionally on siliceous rocks; perhaps most frequent in the climatically most continental parts of the Alps, with optimum near treeline. – **Au**: V, T, S, K, St, O. **Ge**: OB, Schw. **Sw**: BE, GR, LU, SZ, UR, VS. **Fr**: AHP, HAl, AMa, Drô, Isè, HSav. **It**: Ven, TAA, Lomb, Piem, VA.

***Hypogymnia bitteri* (Lynge) Ahti**

Syn.: *Hypogymnia obscurata auct.*, *Parmelia bitteri* Lynge, *Parmelia obscurata auct. et* *sensu* Bitter

L – Subs.: cor, xyl, ter-sil – Alt.: 3–6 – Note: a cool-temperate to boreal-montane, circumpolar lichen found on acid bark, especially of conifers, occasionally on lignum and on siliceous rocks, with optimum near treeline; widespread throughout the Alps. – **Au**: V, T, S, K, St, O, N. **Ge**: OB, Schw. **Sw**: BE, GL, GR, LU, SG, SZ, TI, UR, UW, VS. **Fr**: HAl, AMa, Isè, HSav. **It**: Frl, Ven, TAA, Lomb, Piem, VA. **Sl**: SlA. **Li**.

***Hypogymnia farinacea* Zopf**

Syn.: *Hypogymnia bitteriana* (Zahlbr.) Räsänen, *Parmelia bitteriana* Zahlbr., *Parmelia farinacea* Bitter

L – Subs.: cor, xyl – Alt.: 2–4 – Note: a cool-temperate to boreal-montane lichen, widespread throughout the Alps. – **Au**: V, T, S, K, St, O, N, B. **Ge**: OB, Schw. **Sw**: BE, GL, GR, LU, SG, SZ, TI, UR, UW, VD, VS. **Fr**: AHP, HAl, AMa, Drô, Isè, Sav, HSav, Var, Vau. **It**: Frl, Ven, TAA, Lomb, Piem, VA. **Sl**: SlA, Tg. **Li**.

***Hypogymnia physodes* (L.) Nyl.**

Syn.: *Imbricaria physodes* (L.) DC., *Lichen physodes* L., *Parmelia physodes* (L.) Ach., Parmelia physodes (L.) Ach. var. labrosa Ach., Parmelia physodes (L.) Ach. var. platyphylla Ach.

L – Subs.: cor, xyl, deb, sil – Alt.: 1–5 – Note: a widespread holarctic lichen, common throughout the Alps. – **Au**: V, T, S, K, St, O, N, B. **Ge**: OB, Schw. **Sw**: BE, FR, GL, GR, LU, SG, SZ, TI, UR, UW, VD, VS. **Fr**: AHP, HAl, AMa, Drô, Isè, Sav, HSav, Var, Vau. **It**: Frl, Ven, TAA, Lomb, Piem, VA, Lig. **Sl**: SlA, Tg. **Li**.

***Hypogymnia tubulosa* (Schaer.) Hav.**

Syn.: Hypogymnia physodes (L.) Nyl. var. tubulosa (Schaer.) Walt. Watson, Parmelia physodes (L.) Ach. var. tubulosa Schaer., *Parmelia tubulosa* (Schaer.) Bitter

L – Subs.: cor, xyl – Alt.: 2–4 – Note: a mainly temperate, holarctic species, certainly much rarer than *H. physodes*, and bound to more natural and humid situations; widespread throughout the Alps. – **Au**: V, T, S, K, St, O, N, B. **Ge**: OB, Schw. **Sw**: BE, GL, GR, LU, SG, SZ, TI, UR, UW, VD, VS. **Fr**: AHP, HAl, AMa, Drô, Isè, Sav, HSav, Var, Vau. **It**: Frl, Ven, TAA, Lomb, Piem, VA, Lig. **Sl**: SlA, Tg. **Li**.

***Hypogymnia vittata* (Ach.) Parrique**

Syn.: Imbricaria physodes (L.) DC. var. vittata (Ach.) Körb., Parmelia physodes (L.) Ach. var. vittata Ach., *Parmelia vittata* (Ach.) Röhl.

L – Subs.: cor, sil, bry, ter-sil – Alt.: 2–5 – Note: a circumboreal-montane lichen found on bark, often on basal parts of trunks, on acid soil and overgrowing moribund bryophytes; widespread throughout the Alps, but generally not common. – **Au**: V, T, S, K, St, O, N. **Ge**: OB, Schw. **Sw**: BE, GL, GR, LU, SZ, TI, UR, UW, VD, VS. **Fr**: HAl, AMa, Isè, Sav, HSav. **It**: Frl, Ven, TAA, Lomb, Piem.

***Hypotrachyna afrorevoluta* (Krog & Swinscow) Krog & Swinscow**

Syn.: *Parmelia afrorevoluta* Krog & Swinscow, *Parmelinopsis afrorevoluta* (Krog & Swinscow) Elix & Hale

L – Subs.: cor – Alt.: 2–3 – Note: a species differing from *H. revoluta* in having lobes with a glossy lower surface, often simple, glossy rhizines, and coarse soredia developing from pustules, based on a type from Eastern Africa. It grows on bark of broad-leaved trees in areas with a more or less oceanic climate; widespread in both Hemispheres, and perhaps spreading in Europe in recent years. The species is very similar to *H. revoluta*, and some records of the latter could refer to it; widespread also in the Alps, especially on the northern side. – **Au**: V, T, S, St, O, N. **Ge**: Ge. **Sw**: AP, BE, FR, GL, GR, LU, SG, SZ, TI, UW, VD. **Fr**: Isè, HSav, Var. **It**: Lig.

***Hypotrachyna britannica* (D. Hawksw. & P. James) P. James**

Syn.: Hypotrachyna revoluta (Flörke) Hale var. britannica (D. Hawksw. & P. James) P. Scholz, *Parmelia britannica* D. Hawksw. & P. James, Parmelia revoluta Flörke var. britannica (D. Hawksw. & P. James) V. Wirth, *Parmelinopsis britannica* (D. Hawksw. & P. James) Elix

L – Subs.: sil, cor – Alt.: 2–3 – Note: a species resembling *H. afrorevoluta* in the coarse soredia developing from pustules, but soredia blue-black and easily eroding, lower surface dull and therefore similar to *H. revoluta*; on both rocks and bark, usually near the coast; widespread in Western Europe, with a few records from the Swiss Alps. – **Sw**: GR, TI, ?VS.

***Hypotrachyna laevigata* (Sm.) Hale**

Syn.: *Lichen laevigatus* Sm., *Parmelia laevigata* (Sm.) Ach.

L – Subs.: cor – Alt.: 1–3 – Note: a humid subtropical to mild-temperate lichen found in ancient, very humid forests, on mossy trunks and rocks, very much declining. – **Au**: V, T, S, St, O, N. **Ge**: OB, Schw. **Sw**: BE, LU, SZ, UW, ?VS. **It**: Frl, Ven, TAA, Lomb, Piem, VA. **Sl**: SlA, Tg.

***Hypotrachyna minarum* (Vain.) Krog & Swinscow**

Syn.: *Parmelia minarum* Vain., *Parmelinopsis minarum* (Vain.) Elix & Hale

L – Subs.: cor – Alt.: 2–3 – Note: *H. horrescens* is an Atlantic species, and its records from Switzerland, Italy and Slovenia most probably refer to this species. – **Sw**: TI. **It**: TAA, Lig. **Sl**: SlA.

***Hypotrachyna revoluta* (Flörke) Hale**

Syn.: *Imbricaria revoluta* (Flörke) Flot., *Parmelia revoluta* Flörke

L – Subs.: cor, sil – Alt.: 1–3 – Note: a mild-temperate lichen found on deciduous trees, exceptionally on mossy siliceous rocks in humid areas, very much declining, and absent from urban areas. – **Au**: V, T, S, K, St, O, N. **Ge**: OB, Schw. **Sw**: BE, GL, ?GR, LU, SG, SZ, TI, UR, UW, VD, VS. **Fr**: AMa, Isè, HSav, Var. **It**: Frl, Ven, TAA, Lomb, Piem, VA. **Sl**: SlA. **Li**.

***Hypotrachyna sinuosa* (Sm.) Hale**

Syn.: *Imbricaria sinuosa* (Sm.) Körb., *Lichen sinuosus* Sm., *Parmelia despreauxii* Delise *ex* Duby, *Parmelia sinuosa* (Sm.) Ach.

L – Subs.: cor – Alt.: 2–3 – Note: a widespread, but rare mild-temperate species found on bark and epiphytic mosses in open, humid and cold forests. – **Au**: V, T, S, K, St, O, N. **Ge**: OB, Schw. **Sw**: BE, FR, GL, GR, LU, SZ, UW, VD. **It**: Frl, Ven, TAA, Lomb, Piem.

***Hypotrachyna taylorensis* (M.E. Mitch.) Hale**

Syn.: Parmelia revoluta Flörke var. rugosa Cromb., *Parmelia rugosa* Taylor *non* Fr., *Parmelia taylorensis* M.E. Mitch.

L – Subs.: cor – Alt.: 3 – Note: a rare mild-temperate species found on mossy trunks in ancient, undisturbed, moist forests. – **Au**: V, T, S, St, O, N. **Ge**: OB, Schw. **Sw**: LU, SZ, TI, UW. **It**: Lomb, Piem.

***Icmadophila ericetorum* (L.) Zahlbr.**

Syn.: *Baeomyces aeruginosus* (Scop.) DC., *Baeomyces icmadophilus* (L. f.) Bory, *Biatora icmadophila* (L. f.) Fr., *Icmadophila aeruginosa* (Scop.) Trevis., *Icmadophila elveloides* (Weber) Hedl., *Lecidea icmadophila* (L. f.) Ach., *Lichen ericetorum* L., *Patellaria aeruginosa* (Scop.) Spreng.

L – Subs.: xyl, bry, ter-sil – Alt.: 2–5 – Note: a cool-temperate to boreal-montane, circumpolar species found on decaying wood and moribund bryophytes, usually in upland areas; common throughout the Alps. – **Au**: V, T, S, K, St, O, N. **Ge**: OB, Schw. **Sw**: BE, GR, LU, SZ, TI, UR, UW, VD, VS. **Fr**: AHP, AMa, Isè, Sav, HSav. **It**: Frl, Ven, TAA, Lomb, Piem, VA. **Sl**: SlA, Tg.

***Immersaria athroocarpa* (Ach.) Rambold & Pietschm.**

Syn.: *Amygdalaria athroocarpa* (Ach.) Clauzade & Cl. Roux, *Lecidea athroocarpa* (Ach.) Ach., *Lecidella athroocarpa* (Ach.) Arnold, *Lichen athroocarpus* Ach., *Porpidia athroocarpa* (Ach.) Hertel & Rambold

L – Subs.: sil, sil-par, met – Alt.: 3–5 – Note: a cool-temperate to arctic-alpine, chemically variable, circumpolar species found on siliceous, often iron-rich and weathered rocks in exposed situations, starting the life-cycle on species of *Aspicilia* *s.lat.* – **Au**: V, T, S, K, St, N. **Sw**: TI. **Fr**: AMa, Var. **It**: TAA, Lomb, Piem, VA, Lig. **Sl**: SlA.

***Immersaria cupreoatra* (Nyl.) Calat. & Rambold**

Syn.: *Aspicilia cupreoatra* (Nyl.) Arnold, *Aspicilia olivacea* Bagl. & Carestia, *Bellemerea cupreoatra* (Nyl.) Clauzade & Cl. Roux, *Lecanora cupreoatra* Nyl.

L – Subs.: sil-par – Alt.: 3–5 – Note: an arctic-alpine lichen of siliceous rocks starting the life-cycle on *Buellia*-species, most frequent above treeline; with several scattered records from the Alps. – **Au**: T, S. **Sw**: GR. **It**: Piem, VA, Lig.

***Imshaugia aleurites* (Ach.) S.L.F. Mey.**

Syn.: *Cetraria aleurites* (Ach.) Th. Fr., *Imbricaria aleurites* (Ach.) Lam. & DC., *Lichen aleurites* Ach., *Parmelia aleurites* (Ach.) Ach., *Parmelia diffusa* (Hoffm.) Sandst. *non auct.*, *Parmeliopsis aleurites* (Ach.) Nyl., *Parmeliopsis pallescens* (Hoffm.) Zahlbr., *Parmeliopsis placorodia* *sensu* Jatta *non auct*.

L – Subs.: cor, xyl – Alt.: 2–4 – Note: a circumboreal-montane to cool-temperate species found on acid bark, mostly of conifers and on decorticated stumps, with optimum near treeline; widespread throughout the Alps. – **Au**: V, T, S, K, St, O, N, B. **Ge**: OB, Schw. **Sw**: BE, GL, GR, LU, SG, SZ, TI, UR, UW, VD, VS. **Fr**: AHP, HAl, Isè, Sav, HSav. **It**: Frl, Ven, TAA, Lomb, Piem, VA, Lig.

***Ingvariella bispora* (Bagl.) Guderley & Lumbsch**

Syn.: *Diploschistes bisporus* (Bagl.) J. Steiner, Diploschistes bisporus (Bagl.) J. Steiner var. ochraceus (Anzi) Poelt *comb. inval.*, *Diploschistes ochraceus* (Anzi) J. Steiner, *Diploschistes scruposulus* (Nyl.) J. Steiner, *Rhizocarpon clauzadei* B. de Lesd., *Urceolaria bispora* Bagl., *Urceolaria ferruginea* Harm., *Urceolaria ochracea* Anzi, *Urceolaria scruposula* Nyl.

L – Subs.: sil – Alt.: 1–3 – Note: on base-rich siliceous substrata, mostly on horizontal surfaces; mainly in dry-continental valleys of the Alps. – **Au**: T. **Fr**: AMa, Var. **It**: TAA, Lomb, VA, Lig.

***Inoderma byssaceum* (Weigel) Gray**

Syn.: *Arthonia biformis* (Flörke) Schaer., *Arthonia byssacea* (Weigel) Almq., *Lecidea biformis* Flörke, *Pyrenothea biformis* (Flörke) A. Massal., *Pyrenothea byssacea* (Weigel) A. Massal., *Sphaeria byssacea* Weigel

L – Subs.: cor – Alt.: 2–3 – Note: a mild-temperate species found on very old deciduous trees with acid bark (usually oaks) in open woodlands, often near rivers; most of the few records from the Alps are old. – **Au**: S. **It**: Ven, TAA.

***Involucropyrenium pusillum* Breuss & Türk**

L – Subs.: bry-cal – Alt.: 3 – Note: a species recalling *I. squamulosum*, but with a basally closed, entire involucrellum, smaller perithecia and smaller ascospores; on bryophytes overgrowing boulders of limestone; only known from the type locality in the Eastern Alps. – **Au**: O.

***Involucropyrenium romeanum* (B. de Lesd.) Breuss**

Syn.: *Involucropyrenium squamulosum* (M. Brand & van den Boom) Breuss, *Verrucaria romeana* B. de Lesd., *Verrucaria squamulosa* M. Brand & van den Boom

L – Subs.: cal – Alt.: 3 – Note: a species recalling *Verrucaria macrostoma*, but thallus squamulose, perithecia at the edge or between squamules, with involucrellum reaching the base but basally open, and ascospores to *c.* 30 µm long; on calcareous rocks, including man-made substrates; rather common in Western Europe with a single record from the Eastern Alps, but perhaps overlooked or misidentified elsewhere. – **Au**: S. **Fr**: HSav.

***Involucropyrenium terrigenum* (Zschacke) Breuss**

Syn.: *Catapyrenium terrigenum* (Zschacke) Breuss, *Verrucaria terrigena* Zschacke

L – Subs.: ter-sil – Alt.: 4–5 – Note: a species with squamules closely attached to the soil, fusing to form a grey crust, perithecia with an entire involucrellum, and broadly ellipsoid to subglobose ascospores; on acidic soil, ecology otherwise poorly known; very rare and so far only reported from two localities in the Alps. – **Au**: T. **Fr**: AHP.

***Involucropyrenium tremniacense* (A. Massal.) Breuss**

Syn.: *Catapyrenium tremniacense* A. Massal., *Dermatocarpon tremniacense* (A. Massal.) J. Steiner, *Involucrocarpon tremniacense* (A. Massal.) Servít, *Verrucaria tremniacensis* (A. Massal.) Nyl.

L – Subs.: ter-cal – Alt.: 2–4 – Note: a widespread terricolous species of open grasslands over more or less calcareous substrata; apparently most frequent in the Western and Southern Alps. – **Au**: K. **Sw**: UR. **Fr**: HAl, Isè, HSav, Vau. **It**: Ven, Piem, VA.

***Involucropyrenium waltheri* (Kremp.) Breuss**

Syn.: *Catapyrenium waltheri* (Kremp.) Körb., *Dermatocarpon waltheri* (Kremp.) Blomb. & Forssell, *Involucrocarpon waltheri* (Kremp.) Servít, *Verrucaria waltheri* Kremp.

L – Subs.: ter-cal, ter-sil – Alt.: 4–5 – Note: on humus-rich soil in alpine grasslands; probably more widespread in the Alps. – **Au**: V, T, S, K, St, O, N. **Ge**: OB. **Sw**: GR, SZ, VS. **It**: TAA, Piem, Lig.

***Ionaspis ceracea* (Arnold) Hafellner & Türk**

Syn.: *Aspicilia ceracea* Arnold, *Hymenelia ceracea* (Arnold) M. Choisy, *Lecanora ceracea* (Arnold) Stizenb.

L – Subs.: sil – Alt.: 2–5 – Note: on siliceous pebbles and stones near the soil; widespread in the Alps, but often overlooked, or confused with small *Acarospora*-species. – **Au**: V, T, S, K, St, O, N. **Sw**: BE, GR, SZ, VS. **Fr**: AMa, Sav, HSav. **It**: TAA, Lomb, Piem.

***Ionaspis delibuta* (Ach.) Hue**

Syn.: *Lecidea delibuta* Ach.

L # – Subs.: ?cal – Alt.: ? – Note: a species resembling in habitus *Hymenelia epulotica* and perhaps belonging to that genus, with a thin, continuous, grey to ochraceous thallus and small, immersed, later somewhat elevated apothecia with blackish discs covered by a white pruina; on calcareous(?) rocks, other ecological requirements not documented, growing together with *Opegrapha rupestris* (*fide* Schaerer); reported from Switzerland without a precise locality, and only known from the type. – **Sw**: Sw.

***Ionaspis lacustris* (With.) Lutzoni**

Syn.: *Aspicilia fulvomellea* (A.L. Sm.) Walt. Watson, *Aspicilia lacustris* (With.) Th. Fr., *Hymenelia lacustris* (With.) M. Choisy, *Lecanora fulvomellea* A.L. Sm., *Lecanora lacustris* (With.) Nyl., *Lichen lacustris* With.

L – Subs.: sil-aqu – Alt.: 2–5 – Note: a cool-temperate to arctic-alpine, circumpolar species found on siliceous rocks, periodically submerged in mountain creeks. – **Au**: V, T, S, K, St, N. **Sw**: BE, GR, SZ, VS. **Fr**: AMa, HSav. **It**: TAA, Lomb, Piem, VA.

***Ionaspis obtecta* (Vain.) R. Sant.**

Syn.: *Aspicilia obtecta* (Vain.) Hav., *Hymenelia obtecta* (Vain.) Poelt & Vězda, *Lecanora obtecta* Vain.

L – Subs.: sil, sil-aqu, met – Alt.: 3–4 – Note: a species recalling *I. lacustris*, but with a rimose, ochraceous-brownish thallus, an amyloid medulla, and immersed, but somewhat protruding apothecia with blackish-brown margins; on exposed siliceous, occasionally metal-rich rocks; widespread in Europe but altogether rare, with a few scattered records from the Alps. – **Au**: S, St. **Fr**: HSav. **It**: TAA.

***Ionaspis odora* (Ach. *ex* Schaer.) Stein**

Syn.: *Aspicilia odora* (Ach. *ex* Schaer.) A. Massal., *Gyalecta odora* Ach. *ex* Schaer, *Ionaspis chrysophana auct. non* (Körb.) Stein

L – Subs.: sil-aqu – Alt.: 3–5 – Note: a cool-temperate to arctic-alpine, circumpolar species found on hard siliceous rocks, amphibious along mountain creeks. – **Au**: V, T, S, K, St. **Sw**: BE, GR, UR, VS. **Fr**: AMa, HSav. **It**: TAA, Lomb, Piem, VA. **Sl**: SlA.

***Ionaspis spitzbergensis* H. Magn. (not validly published ICN 36.1.)**

L – Subs.: sil – Alt.: 5 – Note: an arctic-alpine species with an effuse, minutely areolate to subgranular thallus, and minute black apothecia with both margin and epihymenium bluish to blackish green, reacting N+ red violet, the ascospores not exceeding 10 µm in length; on siliceous stones and low outcrops, with a single record from the Eastern Alps – **Au**: S.

***Ionaspis suaveolens* (Fr.) Th. Fr. *ex* Stein**

Syn.: *Aspicilia chrysophana* Körb., *Gyalecta suaveolens* Fr., *Ionaspis chrysophana* (Körb.) Stein

L – Subs.: sil, sil-aqu – Alt.: 3–5 – Note: on hard, compact, siliceous rocks in moist and rather shaded situations, mostly in upland areas. – **Au**: V, T, S, K, St. **Ge**: OB, Schw. **Sw**: BE, GR, UR, VS. **Fr**: HSav. **It**: TAA, Lomb, Piem.

***Jamesiella anastomosans* (P. James & Vězda) Lücking, Sérus. & Vězda**

Syn.: *Gyalideopsis anastomosans* P. James & Vězda

L – Subs.: cor, xyl – Alt.: 2–3 – Note: a species with filmy glaucous thalli provided with peculiar spine-like, isidiiform hyphophores, frequently sterile in Central Europe, rather regularly fertile in Western Europe, with biatorine, red-brown apothecia and muriform ascospores; on bark and stumps in moist forests; widespread in the Northern Hemisphere and also known from New Zealand; widespread also in the Alps, but probably still undercollected. – **Au**: K, S, St, O, N. **Ge**: OB, Schw. **Sw**: GL, GR, SZ, TI, UW, VS. **Fr**: Var. **It**: TAA.

***Japewia subaurifera* Muhr & Tønsberg**

L – Subs.: cor – Alt.: 3–4 – Note: a usually sterile species with a greenish to brown thallus, with soralia at first punctiform and convex, but later often confluent, outer soredia brownish, inner soredia yellowish green, apothecia brown and biatorine, usually less convex than those of *J. tornoensis*; on bark of both broad-leaved and coniferous trees in moist habitats; widespread in the Holarctic region, with a few scattered records from the Alps. – **Au**: T. **Sw**: GR, VS. **Sl**: SlA.

***Japewia tornoensis* (Nyl.) Tønsberg**

Syn.: *Biatora breadalbanensis* (Stirt.) Walt. Watson, *Biatora tornoensis* (Nyl.) Th. Fr., *Lecidea breadalbanensis* Stirt., *Lecidea frigidella* Nyl., *Lecidea tornoensis* Nyl., *Mycoblastus tornoensis* (Nyl.) R.A. Anderson

L – Subs.: cor, xyl, deb – Alt.: 3–5 – Note: a circumboreal-montane species found on twigs of shrubs, on terricolous mosses and plant debris in upland areas, usually over siliceous substrata. – **Au**: V, T, S, K, St, O, N. **Ge**: OB. **Sw**: BE, GL, GR, TI, UR, VS. **It**: Frl, Ven, TAA, VA.

***Koerberia biformis* A. Massal.**

L – Subs.: cor – Alt.: 1–2 – Note: a mild-temperate species found on rough bark, mostly of old deciduous trees, especially *Castanea* and *Quercus*, in humid areas; apparently more frequent in the Western and Southern Alps, but never common. – **Fr**: AHP, AMa, Drô, Var, Vau. **It**: Frl, Ven, Lomb.

***Koerberiella wimmeriana* (Körb.) Stein**

Syn.: *Aspicilia acceptanda* (Nyl.) Arnold *ex* Hue, *Aspicilia leucophyma* (Leight.) Hue, *Aspicilia littoralis* (Vain.) Hue, *Lecanora acceptanda* Nyl., *Lecanora creatina* Norman *ex* Th. Fr., *Lecanora leucophyma* Leight., *Lecanora littoralis* (Vain.) Zahlbr., *Lecanora wimmeriana* (Körb.) Poetsch, *Lecanorella josiae* Frey, *Lecidea ceratina* (Norman *ex* Th. Fr.) Stizenb., *Perspicinora leucophyma* (Leight.) Riedl, *Pertusaria littoralis* Vain., *Zeora wimmeriana* Körb.

L – Subs.: sil, sil-aqu – Alt.: 4–5 – Note: an arctic-alpine, circumpolar species found on periodically wetted siliceous rocks; widespread, but regionally often overlooked in the Alps, being often sterile. – **Au**: V, T, S, K, St. **Ge**: Ge. **Sw**: BE, GR, TI, VS. **Fr**: HAl, AMa, HSav. **It**: TAA, Lomb, Piem.

***Lambiella furvella* (Nyl. *ex* Mudd) M. Westb. & Resl**

Syn.: *Lecidea furvella* Nyl. *ex* Mudd, *Lecidea furvula* Nyl., *Lecidea orphnaeilla* Stirt., *Lecidea spongiosula* Nyl., *Rimularia furvella* (Nyl. *ex* Mudd) Hertel & Rambold

L – Subs.: sil-par – Alt.: 3–5 – Note: a species with a brown, rimose to areolate thallus, the areoles with minute granular isidia easily and soon breaking down to form confluent sorediate crusts, usually sterile; sometimes confused with *Miriquidica intrudens*, but lacking the paler margin of the areoles and the black marginal soralia; on exposed siliceous rocks, where it acts as a non-specialised parasitic lichen (frequent hosts are species of *Rhizocarpon*, *Aspicilia*, *Lecidea*, *Lecanora* and others); widespread in the Northern Hemisphere; widespread in the Alps, but probably overlooked in some regions. – **Au**: V, ?T, S, K, St, N. **Sw**: BE, UR, VD. **Fr**: HSav. **Sl**: SlA.

***Lambiella gyrizans* (Nyl.) M. Westb. & Resl**

Syn.: *Lecidea fuscocinerea auct. non* Nyl., *Lecidea gyrizans* Nyl., *Rimularia gyrizans* (Nyl.) Hertel & Rambold

L – Subs.: sil – Alt.: 5 – Note: a fertile species with an areolate thallus in various shades of brownish-grey to dark brown with convex areoles, roundish to angular, lecideoid apothecia with umbonate to gyrose discs, and ellipsoid ascospores exceeding 10 µm in length; on siliceous boulders and outcrops in the boreal zone and in the montane to subalpine belt in temperate orobiomes, rarely higher; widespread in the Northern Hemisphere but not common, with a single record from the Eastern Alps (Austria). – **Au**: St.

***Lambiella insularis* (Nyl.) T. Sprib.**

Syn.: *Biatora intumescens* (Flörke *ex* Flot.) Hepp, *Lecidea insularis* Nyl., *Lecidea intumescens* (Flörke *ex* Flot.) Nyl., *Lecidea petraea auct.* var. intumescens Flörke, *Lecidella intumescens* (Flörke *ex* Flot.) Arnold, *Nesolechia intumescens* (Flörke *ex* Flot.) Sacc., *Rimularia insularis* (Nyl.) Rambold & Hertel, *Toninia intumescens* (Flörke *ex* Flot.) Boistel

L – Subs.: sil-par, int-par – Alt.: 1–6 – Note: a widespread holarctic lichen invading the thalli of the *Lecanora rupicola*-group; contrary to the host, it is absent from disturbed habitats; widespread throughout the Alps. – **Au**: V, T, S, K, St, N. **Sw**: BE, GR, TI, VS. **Fr**: AHP, HAl, AMa, Sav, HSav, Var, Vau. **It**: Frl, TAA, Lomb, Piem, VA, Lig.

***Lasallia pustulata* (L.) Mérat**

Syn.: *Gyrophora pustulata* (L.) Ach., *Lichen pustulatus* L., *Macrodictya pustulata* (L.) A. Massal., *Umbilicaria pustulata* (L.) Hoffm.

L – Subs.: sil – Alt.: 1–4 – Note: a temperate to boreal-montane, circumpolar species found on periodically wetted, but rapidly drying surfaces of basic siliceous rocks, usually in seepage tracks, with a wide altitudinal range, but usually absent above treeline; widespread throughout the Alps but only locally common. – **Au**: T, K, St. **Sw**: BE, GR, TI, UR, VD, VS. **Fr**: AMa, Isè, Sav, HSav, Var, Vau. **It**: Ven, TAA, Lomb, Piem, VA, Lig.

***Lathagrium auriforme* (With.) Otálora, P.M. Jørg. & Wedin**

Syn.: *Collema auriculatum* Hoffm., *Collema auriforme* (With.) Coppins & J.R. Laundon, *Collema granosum auct.*, *Riccia auriformis* With.

L – Subs.: cal, bry – Alt.: 1–4 – Note: a temperate to southern boreal-montane, holarctic lichen found on calcicolous mosses, rarely directly on rock in sheltered situations, *e.g.* in woodlands or on shaded walls; rare within large settlements and in areas with intensive agriculture, otherwise widespread throughout the Alps. – **Au**: V, T, S, K, St, O, N. **Ge**: OB, Schw. **Sw**: AP, BE, FR, GL, GR, LU, SG, SZ, TI, UR, UW, VS. **Fr**: AHP, HAl, AMa, Drô, Isè, Sav, HSav, Var, Vau. **It**: Frl, Ven, TAA, Lomb, Piem, VA, Lig. **Sl**: SlA, Tg.

***Lathagrium cristatum* (L.) Otálora, P.M. Jørg. & Wedin var. cristatum**

Syn.: *Collema cristatum* (L.) Weber *ex* F.H. Wigg., *Collema granuliferum* Nyl., *Collema hypergenum* Nyl., *Collema melaenum* (Ach.) Ach., *Lichen cristatus* L.

L – Subs.: cal, bry-cal, ter-cal – Alt.: 1–5 – Note: a holarctic lichen found on exposed limestone and dolomite with some seepage of water after rain (an ecological feature which is very evident in dry Mediterranean areas, where the species is confined to rain-tracks), widespread throughout the Alps. – **Au**: V, T, S, K, St, O, N. **Ge**: OB, Schw. **Sw**:  BE, FR, GL, GR, LU, SG, SZ, TI, UW, VD, VS. **Fr**: AHP, HAl, AMa, Drô, Isè, Sav, HSav, Var, Vau. **It**: Frl, Ven, TAA, Lomb, Piem, VA, Lig. **Sl**: SlA, Tg.

***Lathagrium cristatum* (L.) Otálora, P.M. Jørg. & Wedin var. marginale (Huds.) ined. (provisionally placed here, ICN Art. 36.1b)**

Syn.: *Collema cristatum* (L.) Weber *ex* F.H. Wigg. var. marginale (Huds.) Degel., *Collema marginale* (Huds.) Hoffm., *Collema multifidum* (Scop.) Rabenh., *Lichen marginalis* Huds., *Lichen multifidus* Scop.

L # – Subs.: cal – Alt.: 3–5 – Note: a morph with adnate, distinctly and repeatedly furcate lobes that are more or less linear and concave, mostly fertile, but occasionally isidiate (isidia globular to clavate); on limestone boulders and outcrops from mid – to high elevations; widespread in the Northern Hemisphere, but real distribution in the Alps difficult to evaluate, because it was not always distinguished from the typical variety. – **Au**: V, T, S, K, St, N. **Ge**: OB. **Fr**: AHP, AMa, Isè, Sav, HSav, Var. **Sl**: SlA, Tg.

***Lathagrium dichotomum* (With.) Otálora, P.M. Jørg. & Wedin**

Syn.: *Collema dichotomum* (With.) Coppins & J.R. Laundon, *Collema fluviale* (Huds.) Ach., *Collema fluviatile* (Huds.) Steud., *Tremella dichotoma* With.

L – Subs.: sil-aqu – Alt.: 3 – Note: a species with a bright to dark green thallus usually not exceeding 3 cm in diam., with adnate to partly ascending, repeatedly branched, strap-like lobes, the apothecia usually sparse or lacking; on permanently inundated siliceous boulders in rivers and large creeks with not too fast running water; widespread in Europe but rare, with a single record from the Eastern Alps, which needs confirmation. – **Ge**: OB.

***Lathagrium fuscovirens* (With.) Otálora, P.M. Jørg. & Wedin**

Syn.: *Collema furvum* (Ach.) DC., *Collema fuscovirens* (With.) J.R. Laundon, *Collema stillicidiorum* Harm., *Collema subgranosum* Harm., *Collema tuniforme* (Ach.) Ach., *Collema verruciforme* (Ach.) Nyl., *Lichen furvus* Ach., *Lichen fuscovirens* With., *Lichen tuniformis* Ach., *Parmelia furva* (Ach.) Ach.

L – Subs.: cal, bry – Alt.: 1–5 – Note: a widespread holarctic lichen found on calciferous rocks, more rarely on epilithic mosses, in moderately sheltered sites with some water seepage after rain, with a wide altitudinal range; widespread throughout the Alps. – **Au**: V, T, S, K, St, O, N, B. **Ge**: OB, Schw. **Sw**: BE, FR, GR, LU, SG, SZ, TI, UR, UW, VD, VS. **Fr**: AHP, HAl, AMa, Isè, Sav, HSav, Var. **It**: Frl, Ven, TAA, Lomb, Piem, VA, Lig. **Sl**: SlA. **Li**.

***Lathagrium undulatum* (Laurer *ex* Flot.) Poetsch var. undulatum**

Syn.: *Collema laureri* Flot., *Collema undulatum* Laurer *ex* Flot., *Lathagrium laureri* (Flot.) Arnold, *Synechoblastus laureri* (Flot.) Körb., Synechoblastus laureri (Flot.) Körb. var. microphyllinus Anzi

L – Subs.: cal – Alt.: 2–5 – Note: a cool-temperate to arctic-alpine, probably circumpolar lichen found on calciferous rocks with some water seepage after rain, mostly in upland areas; widespread throughout the Alps. – **Au**: V, T, S, K, St, O, N. **Ge**: OB, Schw. **Sw**: BE, GL, GR, LU, SZ, TI, UR, UW, VD, VS. **Fr**: AHP, HAl, AMa, Drô, Sav, HSav, Var, Vau. **It**: Frl, Ven, TAA, Lomb, Piem, VA, Lig. **Sl**: SlA.

***Lathagrium undulatum* (Laurer *ex* Flot.) Poetsch var. granulosum (Degel.) ined. (provisionally placed here, ICN Art. 36.1b)**

Syn.: *Collema undulatum* Laurer *ex* Flot. var. granulosum Degel.

L # – Subs.: cal – Alt.: 3–5 – Note: a usually sterile morph with more or less globular isidia, not always distinguished from the typical variety; on calcareous rocks and soil layers; widespread in the Northern Hemisphere, but real distribution in the Alps difficult to evaluate. – **Au**: V, T, S, K, St, O, N. **Ge**: OB. **Fr**: AHP, HAl, AMa, Drô, Isè, Sav, HSav, Var, Vau. **Sl**: SlA.

***Lecanactis abietina* (Ach.) Körb.**

Syn.: *Cyphelium incrustans* Ach., Lecanactis illecebrosa (Dufour) Fr. var. megaspora G. Merr., *Lecanactis megaspora* (G. Merr.) Brodo, *Lecidea abietina* (Ach.) Ach., *Lichen abietinus* Ach., *Pyrenotea leucocephala* (Ach.) Fr., *Pyrenula leucocephala* Ach., *Schismatomma abietinum* (Ach.) A. Massal. *non* (Humb.) Almq.

L – Subs.: cor – Alt.: 2–4 – Note: a cool-temperate lichen, mostly found in mixed forests with *Abies*, on dry undersides of trunks and old branches, in crevices of the bark, much more rarely on old *Quercus*; widespread throughout the Alps, but generally not very common. – **Au**: V, T, S, K, St, O, N. **Ge**: OB, Schw. **Sw**: BE, GL, LU, SG, SZ, UW, VD, VS. **Fr**: AMa, Isè, HSav, Var. **It**: Frl, TAA, Piem. **Sl**: SlA.

***Lecania atrynoides* M. Knowles**

Syn.: *Lecania macrocarpa* B. de Lesd.

L – Subs.: sil – Alt.: 1–2 – Note: a species with a granular to rimose-areolate, greyish to brownish thallus, and non-pruinose apothecia with red-brown to blackish discs and thin, later excluded thalline margins; on nutrient-enriched siliceous rocks in the supralittoral zone, often in crevices and overhangs; widespread in SW Europe and Macaronesia, but not common, with a few records from the Western Alps (Italy). – **It**: Piem, Lig.

***Lecania coeruleorubella* (Mudd) M. Mayrhofer**

Syn.: Lecania coerulescens Mudd var. coeruleorubella Mudd, Lecania nylanderiana A. Massal. var. coeruleorubella (Mudd) Zahlbr.

L – Subs.: cal – Alt.: 3 – Note: a species with an areolate thallus in various shades of brown, the areolae coarsely sorediate to blastidiate, the sessile apothecia with red-brown to black discs and granular to occasionally sorediate margins, usually partly pruinose, most ascospores 3-septate; on mortar and limestone on shaded walls; widespread in Europe, with a few, mostly historical records from the Eastern Alps (Austria). – **Au**: T, St, N.

***Lecania croatica* (Zahlbr.) Kotlov**

Syn.: *Catillaria croatica* Zahlbr.

L – Subs.: cor – Alt.: 2–3 – Note: a species with an almost immersed to rimose-areolate and then pale grey to tan thallus with discrete punctiform soralia, without chemical substances, often sterile; apothecia entirely ochraceous to pale brown, with simple to 1-septate ascospores; on bark of deciduous trees in different forest types; widespread in the Northern Hemisphere and also in the Alps, but not common; sterile material, however, may not always have been identified correctly. – **Au**: K, St, O. **Ge**: OB, Schw. **Sw**: SZ. **Sl**: SlA.

***Lecania cuprea* (A. Massal.) van den Boom & Coppins**

Syn.: Bacidia albidocarnea (Nyl.) Zahlbr. var. alborubella (Nyl.) Zahlbr., Bacidia albidocarnea (Nyl.) Zahlbr. var. albovirella (Nyl.) Zahlbr., *Bacidia albovirella* (Nyl.) H. Olivier, *Bacidia cuprea* (A. Massal.) Lettau, *Bacidia cupreorosella* (Nyl. *ex* Stizenb.) A. Schneid., *Bacidia prasinoides* (Nyl.) Nyl., *Biatora cupreorosella* (Nyl. *ex* Stizenb.) Tuck., *Bilimbia cuprea* A. Massal., *Catillaria umbraticula* (Nyl.) P. James, *Lecidea albovirella* Nyl., *Lecidea cupreorosella* Nyl. *ex* Stizenb.

L – Subs.: int – Alt.: 1–3 – Note: a mainly temperate species found on underhanging or vertical, base-rich or somehow calciferous rocks in woodlands and gorges, sometimes overgrowing epilithic mosses; widespread in the Alps. – **Au**: T, K, St, O, N. **Sw**: SZ, TI, UW. **Fr**: AHP, HAl, AMa, Drô, Isè, Var, Vau. **It**: Frl, Ven, Piem, Lig.

***Lecania cyrtella* (Ach.) Th. Fr.**

Syn.: *Biatora anomala* Fr., *Biatora cyrtella* (Ach.) W. Mann, ?*Biatora microcyrtella* Anzi, *Biatorina cyrtella* (Ach.) A. Massal., *Biatorina heterobaphia* Anzi, *Bilimbia anomala* (Fr.) Mudd, *Catillaria heterobaphia* (Anzi) Lettau, *Lecanora cyrtella* (Ach.) Röhl., *Lecidea austriaca* Zahlbr., *Lecidea cyrtella* Ach., *Lecidea subalpina* Zahlbr. *non* Stizenb., *Patellaria cyrtella* (Ach.) Müll. Arg., *Sporoblastia cyrtella* (Ach.) Trevis.

L – Subs.: cor, xyl – Alt.: 1–5 – Note: a holarctic lichen found on the base-rich bark of isolated trees, *e.g.* on *Populus*, *Juglans*, *Fraxinus, Sambucus*, mostly in *Xanthorion*-communities; some earlier records could refer to *L. cyrtellina* and *L. sambucina*; widespread throughout the Alps. – **Au**: V, T, S, K, St, O, N, B. **Ge**: OB, Schw. **Sw**: BE, FR, GL, GR, LU, SZ, TI, UW, VD, VS. **Fr**: AHP, AMa, Isè, Sav, HSav, Var, Vau. **It**: Frl, Ven, TAA, Lomb, Piem, VA, Lig. **Sl**: SlA, Tg. **Li**.

***Lecania cyrtellina* (Nyl.) Sandst.**

Syn.: *Lecanora cyrtellina* Nyl., *Lecidea cyrtellina* (Nyl.) Lettau

L – Subs.: cor – Alt.: 2–5 – Note: a species recalling *L. cyrtella*, but most ascospores only up to 3 µm wide, occasionally accompanied by macroconidiomata containing simple to 1-septate, falcate macroconidia; on base-rich bark of broad-leaved trees in the shade of old-growth forests; widespread in Europe, NW Africa and North America, as well as in the Alps, but not common, regionally perhaps overlooked. – **Au**: T, S, K, St. **Sw**: BE, GR, SZ, TI, VS. **Fr**: AHP, AMa, Drô, Isè, Var, Vau. **Sl**: SlA.

***Lecania dubitans* (Nyl.) A.L. Sm.**

Syn.: *Lecania dimera* (Nyl.) Th. Fr., *Lecanora dimera* Nyl., *Lecidea dubitans* Nyl.

L – Subs.: cor – Alt.: 2–4 – Note: a species recalling *L. cyrtella*, but with strongly curved ascospores; mostly on bark of broad-leaved trees; widespread in Europe but altogether rare; from the Alps there are some scattered records, mostly in the montane belt. – **Au**: St, N. **Sw**: BE, GR, VD, VS. **Fr**: Isè.

***Lecania erysibe* (Ach.) Mudd**

Syn.: Lecidea luteola (Schrad.) Ach. var. erysibe (Ach.) Ach., *Lichen erysibe* Ach.

L – Subs.: cal, int – Alt.: 1–3 – Note: a mainly temperate lichen mostly found on calcareous substrata, often on mortar, concrete and brick walls; in the past often confused with other species. – **Au**: V, T, S, K, St, N. **Sw**: GR, VS. **Fr**: AMa, Vau. **It**: Frl, Ven, TAA, Lomb, Piem, VA, Lig.

***Lecania flavescens* Lynge**

L – Subs.: sil – Alt.: 3 – Note: a species with a yellowish-grey, verrucose to rimose-areolate thallus, finally convex apothecia with black, epruinose discs, an intensely violet epihymenium and 8-spored asci containing 1-septate ascospores; based on a type from Novaya Zemlya, where it was found on pure chalk; extremely rare, for the few records from Central and Southern Europe the colour of the epihymenium is given as red-brown, and the ascospores are reported as wider; of these, one record is from the Alps on schistose rocks. – **Sw**: GR.

***Lecania fraudulenta* (Hepp) ined. (provisionally placed here, ICN Art. 36.1b)**

Syn.: *Biatora fraudulenta* Hepp, *Biatorina fraudulenta* (Hepp) Arnold, *Catillaria subfraudulenta* Zahlbr., *Lecidea fraudulenta* (Hepp) Stizenb.

L # – Subs.: cor – Alt.: 3 – Note: a species which is perhaps related to *L. cyrtella*, with hyaline, ellipsoid to ovoid, mostly simple (some 1-septate intermixed) ascospores (8–12 μm long), other characters not mentioned in the protologue; on bark of deciduous trees; only known from the type locality in the Western Alps (Switzerland). – **Sw**: UR.

***Lecania fuscella* (Schaer.) A. Massal.**

Syn.: *Lecania syringea* (Ach.) Th. Fr., Lecanora hagenii (Ach.) Ach. var. syringea (Ach.) Ach., Parmelia pallida (Pers.) Wallr. var. fuscella Schaer.

L – Subs.: cor – Alt.: 1–3 – Note: a mild-temperate species found on base-rich bark of well-lit trees, especially *Populus*, *Juglans* and *Ulmus*; widespread in the Alps, mostly at low elevations. – **Au**: V, T, S, K, St, O, N. **Ge**: OB. **Sw**: BE, FR, LU, SZ, VD. **Fr**: AHP, AMa, HSav, Var. **It**: Ven, TAA, Lomb, Piem, VA, Lig.

***Lecania hutchinsiae* (Nyl.) A.L. Sm.**

Syn.: *Lecanora hutchinsiae* Nyl.

L – Subs.: sil – Alt.: 3 – Note: a species similar to the calcicolous *L. sylvestris*, with a thin, continuous to rimose-areolate thallus coloured in shades of grey, sessile apothecia which are convex from the beginning, with brown discs and soon excluded margins; on siliceous rocks and boulders, often in shaded habitats near the coast; widespread in Western Europe; the only record from the Alps needs confirmation. – **Sw**: SZ.

***Lecania inundata* (Hepp *ex* Körb.) M. Mayrhofer**

Syn.: *Biatorina inundata* Hepp *ex* Körb., Biatorina proteiformis A. Massal. var. compacta A. Massal., *Catillaria italica* B. de Lesd., Lecania erysibe (Ach.) Mudd var. inundata (Hepp *ex* Körb.) Zahlbr., *Lecania porracea* (Stizenb.) Flagey, *Lecania sbarbaronis* B. de Lesd., *Lecanora sbarbaronis* (B. de Lesd.) Zahlbr.

L – Subs.: cal – Alt.: 1–4 – Note: a mild-temperate calcicolous species, often found on man-made substrata; in the past confused with *L. erysibe* or *L. turicensis*, and perhaps more widespread in the Alps. – **Au**: T, S, K, St, N. **Sw**: BE, GR, VS. **Fr**: AMa. **It**: Frl, Ven, TAA, Piem, VA.

***Lecania koerberiana* J. Lahm**

Syn.: *Lecania opuntiae* Bagl.

L – Subs.: cor – Alt.: 2–3 – Note: a mild-temperate and altogether rare species found on nutrient-rich or – enriched bark; closely related to *L. fuscella*, and in the past often confused with *L. naegelii*, to which some of the records may refer. – **Au**: T, K, O. **Fr**: AHP, AMa, Isè, Sav, Var, Vau. **It**: TAA.

***Lecania naegelii* (Hepp) Diederich & van den Boom**

Syn.: *Bacidia abscondita* Erichsen, *Bacidia naegelii* (Hepp) Zahlbr., *Biatora naegelii* Hepp, *Bilimbia naegelii* (Hepp) Kremp., *Bilimbia vallis-tellinae* Anzi

L – Subs.: cor – Alt.: 1–4 – Note: a mainly mild-temperate species with optimum in the submediterranean belt, but also present within eu-Mediterranean vegetation in humid, coastal sites; widespread throughout the Alps, below treeline. – **Au**: V, T, S, K, St, O, N, B. **Ge**: OB. **Sw**: BE, FR, GL, GR, LU, SG, SZ, TI, UR, UW, VD, VS. **Fr**: AHP, AMa, Drô, Isè, Sav, HSav, Var, Vau. **It**: Frl, Ven, TAA, Lomb, Piem, Lig. **Sl**: SlA, Tg. **Li**.

***Lecania nylanderiana* A. Massal.**

Syn.: *Lecania athroocarpa* (Nyl.) Trevis., *Lecania cooperta* (Ach.) Poetsch

L – Subs.: cal – Alt.: 2–4 – Note: a temperate species found on vertical to underhanging surfaces of calcareous rocks; several records from the Alps need confirmation. – **Au**: T, K, St, O, N. **Ge**: OB, Schw. **Sw**: BE, UR, VD, VS. **Fr**: AHP, Sav, Vau. **It**: Frl, Ven, TAA, Lomb, Piem, VA, Lig.

***Lecania olivacella* (Nyl.) Zahlbr.**

Syn.: *Lecania subalbens* (Nyl.) Hazsl., *Lecanora olivacella* Nyl., *Lecanora subalbens* Nyl.

L – Subs.: cal, sil – Alt.: 1–5 – Note: on calcareous and basic siliceous rocks; a widespread, but rare species, with a wide altitudinal range, to be looked for further in the Alps. – **Au**: T, St. **Fr**: AMa, Var. **It**: Piem, Lig.

***Lecania polycycla* (Anzi) Lettau**

Syn.: *Callopisma genevense* Müll. Arg., *Lecania amblyospora* (Harm.) Zahlbr., *Lecania genevensis* (Müll. Arg.) Lettau, *Lecanora amblyospora* Harm., *Rinodina polycycla* Anzi

L – Subs.: cal – Alt.: 1–3 – Note: a mainly temperate species of calcareous rocks, sometimes also occurring on concrete walls. – **Au**: T, St, O, N. **Sw**: VS. **Fr**: AHP, AMa, HSav, Var. **It**: Ven, Piem.

***Lecania pusilla* Tretiach**

L – Subs.: bry-cal – Alt.: 2 – Note: a very inconspicuous lichen found on calcicolous bryophytes inside deciduous woods; so far reported only from the Eastern Alps (Italy). – **It**: Frl.

***Lecania rabenhorstii* (Hepp) Arnold**

Syn.: *Biatorina ceramonea* A. Massal., *Biatorina rabenhorstii* (Hepp) A. Massal., *Lecania alborubra* B. de Lesd., *Lecania algarbiensis* Cout., Lecania erysibe (Ach.) Mudd var. ceramonea (A. Massal.) Zahlbr., Lecania erysibe (Ach.) Mudd var. rabenhorstii (Hepp) Mudd, *Lecaniella rabenhorstii* (Hepp) Jatta, *Patellaria rabenhorstii* Hepp

L – Subs.: cal, int – Alt.: 1–3 – Note: closely related to *L. inundata*; on more or less calciferous substrata, including concrete, tiles, cement etc., also in non-natural situations such as on walls in villages; apparently more frequent in the Southern and Western Alps. – **Au**: St, B. **Sw**: SZ. **Fr**: AHP, AMa, Drô, Isè, Sav, HSav, Var, Vau. **It**: Frl, Ven, TAA, Lomb, Piem, VA.

***Lecania sambucina* (Körb.) Zahlbr.**

Syn.: *Biatorina sambucina* Körb., Lecania cyrtella (Ach.) Th. Fr. subsp. sambucina (Körb.) Arnold

L – Subs.: cor, xyl – Alt.: 2–3 – Note: a lichen recalling *L. cyrtella*, but with 16-spored asci, found in lichen-rich communities on old deciduous trees with a rough, base-rich bark, such as *Sambucus*, *Populus*, and *Salix*; probably widespread throughout Europe, including the Alps, but very likely overlooked or mistaken for *L. cyrtella*, with which it has been often synonymised. – **Au**: O, N.

***Lecania suavis* (Müll. Arg.) Mig.**

Syn.: *Callopisma suave* Müll. Arg., *Lecania tavaresiana* Clauzade & Vězda

L – Subs.: cal – Alt.: 1–4 – Note: on steeply inclined to underhanging surfaces of calcareous rocks, often near small cracks, but also on walls of mortar, usually below the montane belt; widespread throughout the Alps. – **Au**: V, T, S, K, St, O, N. **Ge**: OB, Schw. **Sw**: BE, GR, SZ, VD, VS. **Fr**: HAl, Vau. **It**: TAA, Lomb, Piem, Lig. **Sl**: SlA.

***Lecania subfuscula* (Nyl.) S. Ekman**

Syn.: *Bacidia circumpallens* (Nyl.) Arnold, *Bacidia subfuscula* (Nyl.) Th. Fr., *Biatora siberiensis* Willey, *Lecidea circumpallens* Nyl., *Lecidea subfuscula* Nyl.

L – Subs.: ter-cal – Alt.: 2 – Note: a species with a whitish, rather thick, granular thallus and apothecia with brown to blackish discs and margins often remaining pale, and narrowly fusiform to bacilliform, mostly 3-septate ascospores; mostly on soil and plant debris, rarely directly on rock, in nutrient-rich habitats; based on a type from Iceland and widespread in the Northern Hemisphere, but in the Alps reported from a few localities at low elevations. – **Sw**: UW. **It**: Ven.

**Lecania sylvestris (Arnold) Arnold var. sylvestris**

Syn.: *Biatora holomelaena* (Flörke) Hepp, *Biatora sylvestris* Arnold, *Biatorina sylvestris* (Arnold) Körb., *Catillaria sylvestris* (Arnold) Lettau

L – Subs.: cal – Alt.: 1–4 – Note: a mild-temperate lichen of calcareous substrata, including mortar walls; probably more widespread in the Alps, but never common; closely related to *L. hutchinsiae*. – **Au**: St, O. **Sw**: SZ. **Fr**: HSav. **It**: Ven, Lomb.

**Lecania sylvestris (Arnold) Arnold var. umbratica (Arnold) M. Mayrhofer**

Syn.: Biatorina proteiformis A. Massal. f. umbratica Arnold, Lecania erysibe (Ach.) Mudd f. umbratica (Arnold) Zahlbr.

L – Subs.: cal – Alt.: 1–4 – Note: a mainly temperate lichen of calcareous rocks; probably more widespread in the Alps. – **Au**: V, St. **It**: Frl.

***Lecania turicensis* (Hepp) Müll. Arg.**

Syn.: *Biatora turicensis* Hepp, *Biatorina albariella* (Nyl.) Arnold, *Biatorina proteiformis* A. Massal., Biatorina proteiformis A. Massal. var. dispersa A. Massal., Biatorina proteiformis A. Massal. var. lecideina A. Massal., Biatorina rabenhorstii (Hepp) A. Massal. var. turicensis (Hepp) Anzi, Biatorina turicensis (Hepp) A. Massal. var. farinosa A. Massal., *Lecania albariella* (Nyl.) Müll. Arg., Lecania erysibe (Ach.) Mudd f. dispersa (A. Massal.) Zahlbr., Lecania erysibe (Ach.) Mudd f. lecideina (A. Massal.) Maheu & A. Gillet, Lecania erysibe (Ach.) Mudd var. proteiformis (A. Massal.) Boistel, *Lecania farinosa* (A. Massal.) B. de Lesd., *Lecania phaeoleucodes* (Nyl.) Zahlbr., *Lecania proteiformis* (A. Massal.) Arnold, *Lecania subcaesia* (Nyl.) B. de Lesd., Lecaniella rabenhorstii (Hepp) Jatta var. turicensis (Hepp) Jatta, *Lecanora proteiformis* (A. Massal.) Nyl.

L – Subs.: cal, int, sil – Alt.: 1–5 – Note: on calcareous rocks, mortar, basic siliceous rocks, brick and roofing tiles, often on man-made substrata, usually below the subalpine belt; widespread throughout the Alps. – **Au**: V, T, S, K, St, O, N, B. **Ge**: OB. **Sw**: BE, SZ, TI, VS. **Fr**: AHP, HAl, AMa, Sav, HSav, Var, Vau. **It**: Frl, Ven, TAA, Lomb, Piem, Lig.

***Lecanographa abscondita* (Th. Fr.) Egea & Torrente**

Syn.: *Lecanactis abscondita* (Th. Fr.) Lojka, *Opegrapha abscondita* Th. Fr.

L – Subs.: sil – Alt.: 3–5 – Note: similar to *Psoronactis dilleniana*, but thallus C+ red and ascospores thicker at the upper end and provided with a thick perisporal sheath; on siliceous rocks under overhangs and in other sheltered places; widespread in the Northern Hemisphere, with a few records from the Eastern Alps. – **Au**: T, S, St.

***Lecanographa amylacea* (Ehrh. *ex* Pers.) Egea & Torrente**

Syn.: *Lecanactis amylacea* (Ehrh. *ex* Pers.) Arnold, *Lecanactis illecebrosa* (Dufour) Fr., *Lecidea farinosa* (Ach.) Nyl. *non* H. Magn., *Lichen amylaceus* Ehrh. *ex* Pers., *Opegrapha illecebrosa* Dufour, *Schismatomma illecebrosum* (Dufour) A. Massal.

L – Subs.: cor – Alt.: 1–2 – Note: a mild-temperate, mainly western lichen found on well-lit, old deciduous trees, especially oaks, on faces seldom wetted by rain; very rare in the Alps. – **Sw**: GR. **It**: Ven, TAA.

***Lecanographa lyncea* (Sm.) Egea & Torrente**

Syn.: *Lecanactis emersa* (Müll. Arg.) Stizenb., *Lecanactis lyncea* (Sm.) Fr., *Lecanactis plocina* (Ach.) A. Massal. *non auct.*, *Lecanactis stictica* Durieu & Mont., *Lecanactis vestita* (Müll. Arg.) Stizenb., *Lichen lynceus* Sm., *Opegrapha emersa* Müll. Arg., *Opegrapha lyncea* (Sm.) Borrer *ex* Hook., *Opegrapha stictica* (Durieu & Mont.) Nyl.

L – Subs.: cor, xyl – Alt.: 1–2 – Note: a mild-temperate, mainly western lichen found on the rough, acid bark of very old isolated trees, especially oaks; very rare, and so far reported only from the Western and Southern Alps. – **Fr**: AMa, Var. **It**: Ven, TAA, Lomb.

***Lecanora aitema* (Ach.) Hepp**

Syn.: Lecanora aitema (Ach.) Hepp var. saepincola (Ach.) Hedl., *Lecanora saepincola* (Ach.) Arnold, Lecanora symmicta (Ach.) Ach. var. aitema (Ach.) Th. Fr., Lecanora symmicta (Ach.) Ach. var. saepincola (Ach.) Nyl., Lecanora symmictera Nyl. var. aitema (Ach.) Nyl., *Lecidea aitema* Ach., *Lecidea saepincola* Ach.

L – Subs.: cor, xyl – Alt.: 3–4 – Note: this species differs from *L. symmicta*, of which it is treated as a synonym by several authors, in the dark green to black apothecial discs and the early excluded apothecial margins; on twigs of *Calluna* and other shrubs, more rarely on lignum and bark of coniferous trees and oaks; most records from the Alps are ancient. – **Au**: S, O. **Ge**: OB. **Sw**: LU, SZ. **Fr**: HSav. **It**: Frl, TAA, Lomb, Piem.

***Lecanora albella* (Pers.) Ach.**

Syn.: Lecanora albella (Pers.) Ach. var. cinerella Flörke, Lecanora albella (Pers.) Ach. var. sordidescens Th. Fr., *Lecanora pallida* (Schreb.) Rabenh. *non* Chévall., *Lecanora scrupulosa auct.*, *Lecanora subalbella* Nyl., *Lichen albellus* Pers., *Lichen pallidus* Schreb., *Patellaria pallida* (Schreb.) Trevis.

L – Subs.: cor, xyl – Alt.: 2–4 – Note: a mainly temperate, perhaps holarctic lichen found on smooth bark, especially of *Fagus*, but also of *Abies* in deciduous woodlands; widespread throughout the Alps. – **Au**: V, T, S, K, St, O, N, B. **Ge**: OB, Schw. **Sw**: BE, GL, GR, LU, SG, SZ, TI, UR, UW, VD, VS. **Fr**: Isè, Sav, HSav. **It**: Frl, Ven, TAA, Lomb, Piem, VA, Lig. **Sl**: SlA, Tg.

**Lecanora albellula (Nyl.) Th. Fr. var. albellula**

Syn.: *Lecanora cembricola* Nyl., *Lecanora effusella* Hedl., *Lecanora glaucella* (Flot.) Nyl., Lecanora mughicola Nyl. var. cembricola (Nyl.) Dalla Torre & Sarnth., *Lecanora ochromma* Nyl., *Lecanora ochrostomoides* Nyl., *Lecanora piniperda* Körb. *nom.illeg.*, Lecanora piniperda Körb. var. glaucella (Flot.) Körb., *Lecidea albellula* Nyl.

L – Subs.: xyl, cor – Alt.: 2–4 – Note: a probably circumboreal-montane species of the *L. saligna*-group, better known under its synonym *L. piniperda*, with often densely crowded apothecia that are usually less than 0.5 mm in diam., found on hard lignum and acid bark, usually in upland areas, with a mainly western distribution in Europe; widespread throughout the Alps. – **Au**: V, T, K, St, O, N. **Ge**: OB. **Sw**: GR, SZ, VS. **Fr**: AHP, HAl, AMa, Drô, HSav, Var, Vau. **It**: Frl, Ven, TAA, Lomb, Piem, VA, Lig. **Sl**: SlA.

**Lecanora albellula (Nyl.) Th. Fr. var. macroconidiata M. Brand & van den Boom**

L – Subs.: xyl – Alt.: 3 – Note: a variety with reddish-brown to blackish apothecial discs, and sessile pycnidia often containing allantoid, up to 3-septate macroconidia; on wood or acid bark, widespread in Western Europe but not common; in the Alps so far only known from Switzerland. – **Sw**: SZ.

**Lecanora albula (Nyl.) Hue var. albula**

Syn.: *Squamaria albula* Nyl.

L # – Subs.: sil, int – Alt.: 4–6 – Note: a species based on a type from the French Alps (Dauphiné), characterised by a relatively thick, whitish thallus with subeffigurate margins and apothecia of the *L. polytropa*-type, perhaps heterogenous in its current circumscription; on very sunny surfaces of rocks which are very poor in calcium, in warm-dry situations. – **Au**: V, T, S, K, St. **Ge**: ?B. **Sw**: BE. **Fr**: AHP, HAl, AMa, Vau.

**Lecanora albula (Nyl.) Hue var. vocontia Clauzade & Cl. Roux**

L – Subs.: int – Alt.: 4 – Note: a variety differing in the thallus with a Pd+ yellow to red reaction; on horizontal to inclined rock faces of rocks which usually have a low content in calcium; in the study area so far known from a few localities in the Western Alps (France). – **Fr**: AHP, HAl, Vau.

**Lecanora allophana (Ach.) Nyl. f. allophana**

Syn.: *Lecanora subfusca* (L.) Ach. nom. rej., Lecanora subfusca (L.) Ach. f. allophana Ach.

L – Subs.: cor – Alt.: 2–3 – Note: a mainly temperate lichen found on isolated deciduous trees with base-rich bark, especially *Juglans*, *Acer* and *Fraxinus*, often along roads; most frequent in slightly continental areas; widespread throughout the Alps. – **Au**: V, T, S, K, St, O, N, B. **Ge**: OB, Schw. **Sw**: AP, BE, FR, GL, GR, LU, SG, SZ, TI, UR, UW, VD, VS. **Fr**: AHP, AMa, Drô, Isè, Sav, HSav, Var, Vau. **It**: Frl, Ven, TAA, Lomb, Piem, VA, Lig. **Sl**: SlA, Tg. **Li**.

**Lecanora allophana (Ach.) Nyl. f. sorediata Vain.**

L – Subs.: cor – Alt.: 2–4 – Note: this taxon was sometimes considered as a synonym of *L. impudens*, which, however has a different chemistry; its distribution in the Alps is difficult to assess, since it was not always distinguished. – **Au**: T. **Ge**: Schw. **Sw**: AP, BE, FR, GL, GR, LU, SG, SZ, TI, UR, VD, VS. **It**: Frl.

***Lecanora alpigena* (Ach.) Cl. Roux**

Syn.: Lecanora polytropa (Hoffm.) Rabenh. var. alpigena (Ach.) Rabenh., Lecanora varia (Hoffm.) Ach. var. alpigena Ach.

L – Subs.: sil, int – Alt.: 4–5 – Note: on siliceous or slightly calciferous rocks in upland areas, with optimum above treeline; probably more widespread in the Alps. – **Au**: V, T, S, K, St, N. **Fr**: AHP, HAl, AMa, Sav, HSav. **It**: Frl, Ven, TAA, Lomb, Piem, VA, Lig.

***Lecanora anopta* Nyl.**

Syn.: *Lecidea anopta* (Nyl.) Lettau

L – Subs.: xyl, cor – Alt.: 3–5 – Note: a species related to *L. saligna*, from which it differs in the reddish-brown to blackish apothecia with evanescent thalline margins, recalling those of *L. cadubriae* (but that species is K+ yellow to orange-red); on decorticated trunks, more rarely on the bark of conifers; probably more widespread in the Alps. – **Au**: T, S, K, St, O. **Ge**: OB. **Sw**: BE, GR, SZ, VD, VS. **It**: Ven, TAA. **Sl**: SlA.

***Lecanora argentata* (Ach.) Malme**

Syn.: *Lecanora subfusca auct.*, *Lecanora subfuscata* H. Magn., *Lecanora subrugosa* Nyl., Parmelia subfusca (L.) Ach. var. argentata Ach.

L – Subs.: cor, xyl – Alt.: 2–4 – Note: a widespread, temperate to southern boreal-montane lichen with optimum on smooth bark, especially of *Fagus*. The synonymisation of *L. subrugosa* with this species is supported by molecular data; widespread and common throughout the Alps. – **Au**: V, T, S, K, St, O, N, B. **Ge**: OB, Schw. **Sw**: AP, BE, FR, GL, GR, LU, SG, SZ, TI, UR, UW, VD, VS. **Fr**: AHP, AMa, Drô, Isè, Sav, HSav, Var, Vau. **It**: Frl, Ven, TAA, Lomb, Piem, VA, Lig. **Sl**: SlA, Tg. **Li**.

***Lecanora argopholis* (Ach.) Ach.**

Syn.: *Lecanora blyttii* (Fr.) Schaer., *Lecanora frustulosa auct. non* (Dicks.) Ach., *Lecanora oregana* Tuck., *Lecanora subventosa* (Nyl.) Nyl., *Lecanora thiodes* Spreng., Parmelia atra (Huds.) Ach. var. argopholis Ach., *Schistoplaca argopholis* (Ach.) Brusse

L – Subs.: sil, int – Alt.: 3–5 – Note: a holarctic lichen found on base-rich, sometimes weakly calciferous siliceous rocks, occasionally on detritus, bryophytes and other lichens (*e.g.* on *Psora globifera*). – **Au**: V, T, S, K, St, N. **Sw**: GR, VS. **Fr**: AHP, HAl, AMa, Isè, Vau. **It**: Ven, TAA, Lomb, Piem, VA.

***Lecanora atromarginata* (H. Magn.) Hertel & Rambold**

Syn.: *Lecidea atromarginata* H. Magn.

L – Subs.: sil – Alt.: 4–6 – Note: a holarctic species found on calciferous sandstone and basalt, with optimum above treeline, up to the nival belt; closely related to *L. marginata*, differing mainly in chemistry (usnic and stictic acids); from the Alps there are several scattered records. – **Au**: T. **Ge**: Schw. **Sw**: GR, TI, VS. **Fr**: HAl. **It**: Ven, TAA.

***Lecanora barkmaniana* Aptroot & Herk**

L – Subs.: cor – Alt.: 2–3 – Note: a species of the *L. subfusca*-group with a grey, rimose to angular-verrucose thallus bearing punctiform but soon confluent soralia, the soredia granular and greenish-grey, apothecia rare; corticolous, usually on roadside trees in sites with strong eutrophication; most common in Western Europe and widespread in the Alps, but rarely collected. – **Ge**: OB. **Sw**: GL, GR, SZ, TI, UW, VS. **Fr**: Isè. **Sl**: SlA.

**Lecanora bicincta Ramond var. bicincta**

Syn.: Lecanora rupicola (L.) Zahlbr. var. bicincta (Ramond) Clauzade & Cl. Roux, Lecanora sordida (Pers.) Th. Fr. var. bicincta (Ramond) Th. Fr.

L # – Subs.: sil, int – Alt.: 3–5 – Note: a holarctic lichen found on vertical to underhanging surfaces of hard siliceous rocks. According to Roux et al. (2014) this is just a morphotype of *L. rupicola*, transitional forms being common; widespread throughout the Alps. – **Au**: V, T, S, K, St, N. **Ge**: Schw. **Sw**: GR, TI, UR, VS. **Fr**: AHP, HAl, AMa, Isè, HSav. **It**: Frl, Lomb, Piem, VA, Lig.

**Lecanora bicincta Ramond var. sorediata (Flot.) Leuckert & Poelt**

Syn.: Zeora glaucoma (Hoffm.) Flot. var. sorediata Flot.

L # – Subs.: sil – Alt.: 3–5 – Note: probably more widespread, at least in the Alps, but overlooked, and certainly not common. See also note on var. bicincta. – **Au**: T, St. **Sw**: GR, TI. **Fr**: AMa. **It**: Frl, Lomb, Piem, Lig.

***Lecanora bicinctoidea* Blaha & Grube**

L # – Subs.: sil – Alt.: 4–5 – Note: a species of the *L. swartzii*-group but recalling *L. bicincta* in the areolate thallus and the blackish inner parathecial rim; on vertical to overhanging faces of siliceous cliffs; in the study area so far only reported from the Eastern Alps. – **Au**: St.

***Lecanora biformis* (Ramond) Clauzade & Cl. Roux**

Syn.: *Lecidea biformis* (Ramond) Ramond, *Lichen biformis* Ramond

L – Subs.: sil – Alt.: 3–4 – Note: a species recalling *L. sulphurea*, but with a thinner thallus and smaller, aspicilioid apothecia; on siliceous rocks in upland areas; in the study area so far known from a few localities in the Western Alps (France), but perhaps not recognised elsewhere. – **Fr**: AMa, Isè, Sav.

***Lecanora boligera* (Norman *ex* Th. Fr.) Hedl.**

Syn.: *Biatora nylanderi auct. non* Anzi, Lecidea fuscescens Sommerf. f. boligera Norman *ex* Th. Fr.

L – Subs.: cor – Alt.: 4–5 – Note: a circumboreal-montane species found on twigs of *Rhododendron* and other shrubs in open, often windy situations, especially on small branches, sometimes on plant debris and lignum, with optimum near treeline. – **Au**: V, T, S, K, St. **Sw**: GR, VS. **It**: Frl. **Sl**: SlA.

***Lecanora cadubriae* (A. Massal.) Hedl.**

Syn.: *Biatora admixta* Th. Fr., *Biatora cadubriae* A. Massal., *Lecidea cadubriae* (A. Massal.) Th. Fr., *Lecidea magnussoniana* Hertel, *Lecidea ramulicola* H. Magn. *non* (H. Magn.) Hillm., *Lecidea subinsequens* Nyl.

L – Subs.: cor, xyl, bry – Alt.: 3–5 – Note: a circumboreal-montane species found on the bark of conifers, especially near the base of the trunks, more rarely on lignum of decorticated trunks, with optimum in the upper montane and subalpine belts; widespread throughout the Alps. – **Au**: V, T, S, K, St, O, N. **Ge**: OB. **Sw**: BE, GR, SG, SZ, TI, UR, UW, VD, VS. **Fr**: AHP, HAl, AMa. **It**: Frl, Ven, TAA, Lomb, Piem, VA. **Sl**: SlA.

***Lecanora caesiosora* Poelt**

Syn.: Lecanora cenisia Ach. var. soredians Suza, *Lecanora soralifera* H. Magn. *non* (Suza) Räsänen

L – Subs.: sil – Alt.: 3–5 – Note: on hard siliceous rocks in upland areas; very much overlooked, but certainly not common in the Alps. – **Au**: V, T, S, K, St, N. **Sw**: SZ. **Fr**: AMa. **It**: Piem. **Sl**: SlA.

***Lecanora campestris* (Schaer.) Hue**

Syn.: Lecanora atra (Huds.) Ach. var. expansa Ach., Lecanora campestris (Schaer.) Hue var. expansa (Ach.) Erichsen, *Lecanora genuensis* B. de Lesd., *Lecanora maceriaecola* B. de Lesd., *Lecanora ossicola* Erichsen, Lecanora subfusca (L.) Ach. var. campestris (Schaer.) Rabenh., *Lecanora subglabrata* Werner, *Lecanora viridans* Maheu & Werner, Parmelia subfusca (L.) Ach. var. campestris Schaer.

L – Subs.: sil, int – Alt.: 1–4 – Note: a holarctic lichen mostly found on basic siliceous rocks (rarely also on superficially decalcified limestone), especially hard sandstone, often on small stones or on surfaces not far from the ground; widespread throughout the Alps, mostly below treeline. – **Au**: V, T, S, K, St, O, N, B. **Ge**: OB, Schw. **Sw**: BE, GR, LU, SZ, TI, VD, VS. **Fr**: AHP, HAl, AMa, Isè, Sav, HSav, Var, Vau. **It**: Frl, Ven, TAA, Lomb, Piem, VA, Lig. **Sl**: SlA.

***Lecanora carpinea* (L.) Vain.**

Syn.: Lecanora albella (Pers.) Ach. var. angulosa (Schreb.) Flot., *Lecanora angulosa* (Schreb.) Ach., *Lecanora erikssonii* H. Magn., Lecanora pallida (Schreb.) Rabenh. var. angulosa (Schreb.) Rabenh., *Lichen carpineus* L.

L – Subs.: cor – Alt.: 1–4 – Note: a mainly temperate early coloniser of smooth bark, with a wide altitudinal range; common throughout the Alps below the subalpine belt. The species has several morphotypes, and may consist of several species which still need to be delimited. – **Au**: V, T, S, K, St, O, N, B. **Ge**: OB, Schw. **Sw**: AP, BE, FR, GL, GR, LU, SG, SZ, TI, UR, UW, VD, VS. **Fr**: AHP, HAl, AMa, Drô, Isè, Sav, HSav, Var, Vau. **It**: Frl, Ven, TAA, Lomb, Piem, VA, Lig. **Sl**: SlA, Tg. **Li**.

***Lecanora cateilea* (Ach.) A. Massal.**

Syn.: Lecanora subfusca var. cateilea Ach. –

L – Subs.: cor – Alt.: 2–3 – Note: this is a member of the *L. albella*-group, which includes species with a pruinose disc and an ecorticate apothecial margin. It is distinguished by the polysporous asci and the Pd+ yellow apothecial margin (psoromic acid), and has a mainly northern distribution in Europe, growing on bark in rather shaded and humid situations – **It**: Ven.

***Lecanora cavicola* Creveld**

L – Subs.: sil – Alt.: 4–6 – Note: a species found on acid siliceous rocks near or above treeline; perhaps more widespread, but not common in the Alps, where it reaches the nival belt. – **Au**: T, S, K, St. **It**: Piem.

***Lecanora cenisia* Ach.**

Syn.: *Lecanora atrynea* (Ach.) Nyl., Lecanora atrynea (Ach.) Nyl. var. melacarpa Nyl., Lecanora cenisia Ach. var. atrynea (Ach.) H. Magn., Lecanora cenisia Ach. var. melacarpa (Nyl.) Boistel, *Lecanora transcendens* (Nyl.) Arnold, *Parmelia cenisia* (Ach.) Fr., *Zeora cenisia* (Ach.) Flot.

L – Subs.: sil, cor, xyl – Alt.: 3–6 – Note: a circumpolar, cool-temperate to arctic-alpine lichen of siliceous rocks, more rarely also found on hard lignum; widespread and common throughout the Alps. – **Au**: V, T, K, S, St, N. **Ge**: OB, Schw. **Sw**: BE, GR, TI, UR, VD, VS. **Fr**: AHP, HAl, AMa, Isè, Sav, HSav, Var, Vau. **It**: Frl, Ven, TAA, Lomb, Piem, VA, Lig. **Sl**: SlA.

**Lecanora chlarotera Nyl. subsp. chlarotera**

Syn.: Lecanora chlarotera Nyl. f. rugosella (Zahlbr.) Poelt, *Lecanora crassula* H. Magn., *Lecanora rugosa* (Nyl.) Nyl. *non* Ach., *Lecanora rugosella* Zahlbr.

L – Subs.: cor, xyl, sil – Alt.: 1–4 – Note: this is certainly one of the the most common epiphytic *Lecanora* at low elevations throughout the Alps. – **Au**: V, T, S, K, St, O, N, B. **Ge**: OB, Schw. **Sw**: AP, BE, FR, GL, GR, LU, SG, SZ, TI, UR, UW, VD, VS. **Fr**: AHP, HAl, AMa, Drô, Isè, Sav, HSav, Var, Vau. **It**: Frl, Ven, TAA, Lomb, Piem, VA, Lig. **Sl**: SlA, Tg. **Li**.

**Lecanora chlarotera Nyl. subsp. meridionalis (H. Magn.) Clauzade & Cl. Roux**

Syn.: Lecanora chlarotera Nyl. f. meridionalis (H. Magn.) Ozenda & Clauzade, *Lecanora meridionalis* H. Magn.

L # – Subs.: cor – Alt.: 1–2 – Note: a very controversial taxon, perhaps just a form of *L. chlarotera* with darker apothecial discs. – **Fr**: AHP, AMa, Var, Vau. **It**: Frl, Ven, Lomb, Piem, Lig.

***Lecanora chloroleprosa* (Vain.) H. Magn.**

Syn.: Lecanora chlorophaeodes Nyl. subsp. chloroleprosa Vain.

L – Subs.: sil – Alt.: 4 – Note: a species with a yellowish blue-green thallus composed of granules and subspherical areoles partly transforming in equally coloured soredia, based on a type from Northern Karelia; on siliceous rocks in windy situations; very rare in the Alps, apparently more common in Scandinavia. – **Au**: K.

***Lecanora cinereofusca* H. Magn.**

Syn.: *Lecanora degelii* T. Schauer & Brodo

L – Subs.: cor – Alt.: 3 – Note: a rare species found on smooth bark of old deciduous trees, especially *Fagus*, in humid montane forests. For specimens on *Abies* with large, thick-walled ascospores see *L. insignis*. – **Au**: V, T, S, K, St, O, N. **Ge**: OB, Schw. **Sw**: BE, SZ, UW. **Fr**: Vau. **It**: Frl, Lig.

***Lecanora circumborealis* Brodo & Vitik.**

Syn.: *Lecanora coilocarpa auct. non* (Ach.) Nyl.

L – Subs.: cor, xyl – Alt.: 2–5 – Note: a circumboreal-montane lichen found on acid bark, often on twigs, sometimes on lignum, mostly in upland areas, with optimum in the subalpine belt; widespread throughout the Alps. – **Au**: V, T, S, K, St, O, N. **Ge**: OB, Schw. **Sw**: BE, GL, GR, LU, SZ, TI, UR, VS. **Fr**: Isè, Sav, HSav, Vau. **It**: Frl, Ven, TAA, Lomb, Piem, VA. **Sl**: SlA, Tg.

***Lecanora compallens* Herk & Aptroot**

L – Subs.: cor – Alt.: 2–3 – Note: a sorediate species recalling *L. expallens* (with which it often grows together) except the thallus colour, which is whitish-grey due to the lack of usnic acid (which is only detectable in the granular bluish-green soredia); on a wide variety of roadside trees, but avoiding those with very acid bark; rarely collected in the Alps at low elevations, more common in Western Europe. – **Sw**: UW. **Fr**: AMa, Drô, Vau.

***Lecanora concolor* Ramond**

Syn.: *Placodium concolor* (Ramond) Körb., *Squamaria concolor* (Ramond) Nyl.

L – Subs.: sil – Alt.: 4–6 – Note: on vertical to underhanging surfaces of hard siliceous rocks, with optimum above treeline; widespread throughout the Alps, where it reaches the nival belt. – **Au**: V, T, S, K. **Sw**: BE, GR, UR, VS. **Fr**: AHP, HAl, AMa, Isè, Sav, HSav. **It**: TAA, Lomb, Piem, VA.

***Lecanora conizaeoides* Nyl. *ex* Cromb.**

Syn.: *Lecanora conizaea auct. non* (Ach.) Nyl., Lecanora conizaea f. variola Arnold, Lecanora farinaria Borrer var. conizaeoides (Nyl. *ex* Cromb.) A.L. Sm., *Lecanora pityrea* Erichsen, *Lecanora pseudovaria* Degel. *nom. nud*.

L – Subs.: cor – Alt.: 2–3 – Note: a species with a sorediate thallus and apothecia usually present as well, reacting Pd+ red (fumarprotocetraric acid); on acid bark; in cities with severe air pollution it was often the only lichen that survived, more common in second half of the XX century in times of acid air-pollution, apparently becoming rarer and regionally even disappearing due nitrogen-rich pollution. – **Au**: V, T, S, K, St, O, N. **Ge**: OB, Schw. **Sw**: BE, GR, LU, SG, TI. **Fr**: Isè, Var, Vau. **It**: Lomb, Piem VA. **Sl**: SlA, Tg.

***Lecanora daunasii* Houmeau & Cl. Roux**

L – Subs.: sil – Alt.: 4–5 – Note: a species related to *L. sulphurea*, differing in the smaller apothecia with orange-brownish discs, recalling *Protoblastenia rupestris* in colour; on steep faces of siliceous rock in shaded situations; in the study area so far only reported from the Western Alps (France). – **Fr**: AHP.

***Lecanora diaboli* Frey & Poelt**

Syn.: Lecanora concolor Ramond f. elata (Arnold) Mig., Placodium concolor (Ramond) Körb. f. elatum Arnold

L – Subs.: cal, int – Alt.: 3–6 – Note: a species with large, thick thalli forming rosettes, otherwise recalling *L. concolor*; on steep rock faces of marly limestone or schists and even gneiss at high elevations; widespread in the Alps, but rare. – **Au**: T. **Sw**: GR. **Fr**: AHP, HAl, AMa.

***Lecanora dispersoareolata* (Schaer.) Lamy**

Syn.: Lecanora muralis (Schreb.) Rabenh. var. dispersoareolata Schaer., *Placodium dispersoareolatum* (Schaer.) Körb., *Squamaria dispersoareolata* (Schaer.) Anzi

L – Subs.: int, sil – Alt.: 3–6 – Note: on exposed, weakly calcareous or basic siliceous rocks in upland areas; widespread throughout the Alps. – **Au**: V, T, S, K, St. **Ge**: Schw. **Sw**: BE, GR, UR, UW, VS. **Fr**: AHP, HAl, AMa, Isè, Sav, HSav. **It**: Frl, Ven, TAA, Lomb, Piem, VA. **Sl**: SlA.

***Lecanora eminens* Asta, Clauzade & Cl. Roux**

Syn.: *Lecanora prominens* Asta, Clauzade & Cl. Roux *non* Clauzade & Vězda

L – Subs.: cal, int – Alt.: 4–5 – Note: a species related to *L. albula* with which it often grows together, with a chalky-white thallus consisting of dispersed areoles bordered by a black hypothallus, the apothecia provided with a blue-green disc, a persisting thalline margin reacting Pd+ yellow (psoromic acid), and minute ascospores; on schists poor in calcium carbonate, usually on south-exposed steep faces in the alpine belt; in the study area so far known from a few localities of the Western Alps (France). – **Fr**: HAl, AMa.

***Lecanora epanora* (Ach.) Ach.**

Syn.: *Lichen epanorus* Ach., *Parmelia epanora* (Ach.) Ach., *Patellaria epanora* (Ach.) Trevis.

L – Subs.: met – Alt.: 2–5 – Note: a holarctic early coloniser of steeply inclined to underhanging surfaces of metal-rich metamorphic rocks, mostly in upland areas. – **Au**: T, S, K, St. **Sw**: BE, GR, UR, VS. **Fr**: AMa, HSav. **It**: Ven, TAA, Lomb, Piem, VA.

**Lecanora epibryon (Ach.) Ach. var. epibryon**

Syn.: Lecanora subfusca (L.) Ach. var. hypnorum (Wulfen) Schaer., *Lichen epibryon* Ach.

L – Subs.: bry, deb – Alt.: 3–5 – Note: a circumpolar, arctic-alpine species found on mosses and plant debris in open calcareous grasslands and alpine tundras, often on ridges in *Carex firma* stands; common throughout the Alps near and above treeline. – **Au**: V, T, S, K, St, O, N. **Ge**: OB. **Sw**: BE, FR, GR, LU, SG, SZ, TI, VD, VS. **Fr**: AHP, HAl, AMa, Isè, Sav, HSav. **It**: Frl, Ven, TAA, Lomb, Piem, VA, Lig. **Sl**: SlA. **Li**.

**Lecanora epibryon (Ach.) Ach. var. bryopsora Doppelb. & Poelt**

Syn.: *Lecanora bryopsora* (Doppelb. & Poelt) Hafellner & Türk

L # – Subs.: bry, deb – Alt.: 3–5 – Note: on mosses and plant debris on calcareous substrata; certainly more widespread in the Alps, but difficult to recognise, being often sterile. Molecular data suggest that this is just a sorediate morph of *L. epibryon*. – **Au**: V, T, S, K, St. **Fr**: AHP, Sav. **It**: Piem.

***Lecanora eurycarpa* Poelt, Leuckert & Cl. Roux**

Syn.: *Myriolecis eurycarpa* (Poelt, Leuckert & Cl. Roux) Hafellner & Türk

L – Subs.: cal – Alt.: 3–5 – Note: a species with a poorly developed thallus and dispersed, large apothecia with whitish margins turning to bluish or blackish towards the edge of the hymenium, the discs brown, epruinose; usually on steep rock faces of various types of schist including those rich in iron, mostly at high elevations; widespread in the Alps but rare. The species, which contains usnic acid, does not belong into *Myriolecis* (see Roux et al. 2017) – **Au**: T, S, St. **Sw**: VS. **Fr**: AHP, AMa, HSav.

***Lecanora expallens* Ach.**

Syn.: *Lecanora conizaea* (Ach.) Nyl., Lecanora expallens Ach. var. conizaea Ach., *Lecanora foehrensis* Erichsen, *Lecidea soraliata* Vain.

L – Subs.: cor, xyl – Alt.: 1–4 – Note: a mainly temperate species found on acid, generally rough bark, especially abundant on *Quercus* in open woodlands, sometimes on lignum; widespread and often common throughout the Alps. – **Au**: T, S, K, St, O, N. **Ge**: OB, Schw. **Sw**: BE, LU, SZ, UW, VS. **Fr**: AHP, AMa, Drô, Isè, HSav, Var. **It**: Frl, Ven, TAA, Lomb, Piem, VA, Lig. **Sl**: SlA, Tg.

***Lecanora expersa* Nyl.**

Syn.: Lecanora coilocarpa (Ach.) Nyl. var. sorediata Räsänen, *Lecanora elisa* Nyl., *Lecanora raesaenenii* Gyeln.

L – Subs.: cor, xyl – Alt.: 3–4 – Note: a sorediate species of the *L. subfusca*-group with the chemistry of *L. circumborealis*, characterised by roundish, usually not confluent soralia; usually on wood, occasionally on bark, including branches of subalpine shrubs (*Rhododendron*) at high elevations; widespread in the Alps but regionally undercollected, being often sterile. – **Au**: V, T, S, K, St, O. **Ge**: OB. **Sw**: BE, GL, GR, LU, SZ, TI, UW, VD, VS. **Fr**: HAl. **It**: Ven. **Sl**: SlA.

***Lecanora farinaria* Borrer**

L – Subs.: cor – Alt.: 3–4 – Note: a taxon of the *L. subfusca*-group with a sorediate thallus, resembling *L. barkmaniana*, but with mounds of yellowish-white soredia, a different chemistry, and apothecia, when present, of the *pulicaris*-type; usually on wood but occasionally also on bark; rare in the Alps, more common in NW Europe. – **Au**: T, S. **Sw**: TI, VS.

***Lecanora flageyana* Müll. Arg.**

L # – Subs.: cor – Alt.: 2 – Note: a species with a thin, greyish thallus and minute apothecia which are aspicilioid at first, later recalling those of *Myriolecis persimilis*; on the bark of deciduous trees at low elevations; in the study area so far known from a single locality in the Western Alps (France). – **Fr**: HSav.

***Lecanora flahaultiana* Hue**

L # – Subs.: cal – Alt.: 5–6 – Note: a calcicolous species with a whitish, pulvinate, fragile thallus, sessile apothecia, and minute, broadly ellipsoid to subspherical ascospores; in the study area so far known only from the Western Alps (France). – **Fr**: AHP, Sav.

***Lecanora flavoleprosa* Tønsberg**

L – Subs.: xyl, cor – Alt.: 2–4 – Note: a species morphologically resembling *L. expallens*, with a greyish to yellowish thallus and soon confluent, pale yellow to yellow-green soralia (containing usnic acid, an unnamed xanthone and terpenoids including zeorin), apothecia occasionally present, of a dark aeruginose colour; on bark of various trees in all forest belts; distribution still incompletely documented. Earlier records from BE, LU, UW and VD in Switzerland refer to *Lecanora compallens* or *L. strobilina*. – **Au**: S, K, N. **Sw**: FR.

***Lecanora formosa* (Bagl. & Carestia) Knoph & Leuckert**

Syn.: *Lecidea contorta* Bagl. & Carestia, *Lecidea formosa* Bagl. & Carestia, *Lecidea lacticolor* Arnold, *Lecidea mesotropiza* Nyl., *Lecidea nansenii* Lynge, *Lecidea subdita* Nyl., *Lecidella bullata auct. non* Körb.

L – Subs.: sil, int – Alt.: 3–6 – Note: on slightly underhanging surfaces of siliceous rocks, especially crystalline schist, in humid and cold situations, with optimum in the alpine and nival belts, often starting the life-cycle on other crustose lichens; widespread in the Alps, but generally not common. – **Au**: T, S, K, St. **Sw**: GR, SZ, VS. **Fr**: AHP, HAl, Sav. **It**: Frl, TAA, Piem, VA.

***Lecanora freyi* Poelt**

L – Subs.: cal, int – Alt.: 3–5 – Note: a rosulate species with minute lobes recalling small thalli of *Lecanora valesiaca*, and apothecia with brownish-greenish to dark green discs; on steep rock faces of calcareous schists at high elevations; widespread in the Alps, but altogether rare. – **Au**: V, T, S, K, St. **Ge**: OB, Schw. **Sw**: GR. **Fr**: AHP.

***Lecanora frustulosa* (Dicks.) Ach.**

Syn.: Lecanora frustulosa (Dicks.) Ach. var. ludwigii (Spreng.) Th. Fr., *Lecanora hydrophila* Sommerf., *Lecanora ludwigii* (Spreng.) Ach., *Lecidea boissoniana* Croz., *Lichen frustulosus* Dicks., *Patellaria frustulosa* (Dicks.) Trevis., *Toninia boissoniana* (Croz.) Zahlbr.

L – Subs.: sil, int – Alt.: 2–5 – Note: on steeply inclined surfaces of weakly calciferous siliceous rocks, often in otherwise dry seepage tracks, mostly in upland areas. – **Au**: V, T, S, K, St. **Sw**: BE, GR, TI, VS. **Fr**: AHP, HAl, AMa, Isè, Sav, HSav. **It**: Ven, TAA, Lomb, Piem, VA.

***Lecanora fuscescens* (Sommerf.) Nyl.**

Syn.: *Biatora fuscescens* (Sommerf.) Fr., *Biatorella fuscescens* (Sommerf.) Boistel, *Lecidea fuscescens* Sommerf.

L – Subs.: xyl, cor, deb – Alt.: 3–5 – Note: a probably circumboreal-montane species found on twigs of shrubs, especially *Rhododendron ferrugineum* in the subalpine belt, sometimes on lignum. – **Au**: V, T, S, K, St. **Ge**: OB. **Sw**: BE, GR, UR, VS. **Fr**: HSav. **It**: Ven, TAA, Lomb, Piem.

***Lecanora gangaleoides* Nyl.**

Syn.: Lecanora cenisia Ach. var. gangaleoides (Nyl.) Harm.

L – Subs.: int – Alt.: 1–5 – Note: a mild-temperate lichen found on base-rich, but lime-poor siliceous rocks in sheltered situations, often in underhangs; most frequent in the Western Alps. – **Au**: ?V. **Fr**: AHP, HAl, AMa, Sav, Var, Vau. **It**: Ven, Lig.

***Lecanora gisleri* (Anzi *ex* Arnold) Arnold**

Syn.: *Biatora gisleri* Anzi *ex* Arnold, *Lecidea gisleri* (Anzi *ex* Arnold) Stizenb.

L – Subs.: cor, xyl – Alt.: 4–5 – Note: a probably circumboreal-montane lichen found on twigs of shrubs, especially *Rhododendron ferrugineum* in the subalpine belt. – **Au**: V, T, S, K, St. **Ge**: Schw. **Sw**: VS. **It**: Frl, TAA, Lomb, Piem.

***Lecanora gisleriana* Müll. Arg.**

Syn.: *Lecanora gisleri* Poelt & Ullrich *non* (Anzi *ex* Arnold) Arnold

L – Subs.: met-par – Alt.: 3–5 – Note: on iron rich siliceous rocks near and above treeline, parasitic on the thalli of *L. epanora*, *L. handelii* and *L. subaurea*; widespread but localised in the Alps. – **Au**: T, S, K. **Sw**: UR. **Fr**: HSav. **It**: VA.

***Lecanora glabrata* (Ach.) Nyl.**

Syn.: Lecanora allophana (Ach.) Nyl. var. glabrata (Ach.) J. Steiner, Lecanora subfusca (L.) Ach. var. glabrata Ach.

L – Subs.: cor – Alt.: 2–4 – Note: a mainly temperate species found on smooth bark of deciduous trees; several old records from the Alps require confirmation. – **Au**: T, K, St, N, B. **Ge**: OB. **Sw**: GR, TI. **Fr**: HAl, AMa, Drô, Isè, Sav, HSav, Vau. **It**: Frl, Ven, TAA, Lomb, Piem, VA, Lig. **Sl**: SlA, Tg.

***Lecanora glaucolutescens* Nyl.**

L – Subs.: sil – Alt.: 3–5 – Note: a species with a granular to leprose, bluish-greenish to yellowish thallus reacting C+ orange and concolorous apothecia, based on a type from Portugal, on quartzitic rocks near the sea; apparently rare, the records from mid – to high elevations (Austria) are dubious. – **Au**: ?K. **Fr**: Isè.

***Lecanora handelii* J. Steiner**

L – Subs.: met, sil – Alt.: 3–5 – Note: on steeply inclined to underhanging surfaces of metalliferous rocks in upland areas; widespread in the Alps, wherever suitable substrata are present. – **Au**: ?V, T, S, K, St. **Sw**: GR, UR, VS. **Fr**: HSav. **It**: Lomb, Piem, VA.

***Lecanora horiza* (Ach.) Linds.**

Syn.: *Lecanora laevis* Poelt, *Lecanora parisiensis* Nyl., Lecanora subfusca (L.) Ach. f. horiza Ach.

L – Subs.: cor – Alt.: 1–3 – Note: a mainly Mediterranean species found on smooth bark of isolated broad-leaved trees; much rarer in the Alps than in the Mediterranean mountains. – **Au**: S, K, St, N. **Sw**: GL, GR, SG, TI, UW, VS. **Fr**: AHP, AMa, Isè, HSav, Var, Vau. **It**: Lig. **Sl**: SlA, Tg.

***Lecanora hybocarpa* (Tuck.) Brodo**

Syn.: *Parmelia hybocarpa* Tuck.

L – Subs.: cor – Alt.: 2–3 – Note: on the bark of more or less isolated trees in lowland areas; apparently common in North America, this species has been recently reported also from Europe, and is also known from the Western Alps. – **Sw**: ?SZ. **Fr**: Isè.

***Lecanora hypopta* (Ach.) Vain.**

Syn.: *Biatora hypopta* (Ach.) Räsänen, Lecanora subintricata (Nyl.) Th. Fr. var. convexula Arnold, *Lecidea hypopta* Ach.

L – Subs.: xyl – Alt.: 3–4 – Note: on hard lignum, especially on decorticated stumps, more rarely on the bark of conifers in upland areas; certainly more widespread in the Alps. – **Au**: V, T, S, K, St, O, N. **Ge**: OB. **It**: TAA, Piem.

***Lecanora hypoptella* (Nyl.) Grummann**

Syn.: *Lecanora symmictiza* (Nyl.) Hedl., *Lecidea symmictiza* Nyl., *Lecidea hypoptella* Nyl.

L – Subs.: xyl, cor – Alt.: 3–4 – Note: a mainly boreal-montane lichen found on lignum and acid bark in upland areas; it belongs to a poorly known group and is widespread in Scandinavia, being also known from the British Isles, France, Central Europe, and the mountains of the Iberian Peninsula; perhaps more widespread in the Alps. – **It**: Ven, TAA.

***Lecanora hypoptoides* (Nyl.) Nyl.**

Syn.: *Lecidea hypoptoides* Nyl.

L – Subs.: xyl – Alt.: 3–4 – Note: a boreal-montane lichen found on hard lignum, especially on decorticated stumps, more rarely on the acid bark of conifers and *Castanea*, mostly in upland areas. – **Au**: T, S, K, St, N. **Sw**: BE, GR, SZ, VS. **Fr**: AHP, AMa. **It**: Ven, TAA, Lomb, Piem. **Sl**: SlA.

***Lecanora impudens* Degel.**

Syn.: *Lecanora chloropolia auct. non* (Erichsen) Almb., *Lecanora maculata* (Erichsen) Almb., *Pertusaria farinacea* H. Magn., *Pertusaria maculata* Erichsen *non* H. Magn.

L – Subs.: cor – Alt.: 2–4 – Note: a temperate species found on base-rich bark, especially on isolated *Fraxinus* in humid riparian woodlands; widespread throughout the Alps. – **Au**: V, T, S, K, St, O, N, B. **Ge**: OB, Schw. **Sw**: BE, GR, LU, SZ, VD, VS. **Fr**: HAl, AMa, Isè, Var. **It**: Frl, TAA. **Sl**: SlA. **Li**.

***Lecanora insignis* Degel.**

L – Subs.: cor – Alt.: 3 – Note: a rare species of the *L. subfusca*-group, peculiar in having large, thick-walled ascospores; apparently restricted to the bark of coniferous trees in humid montane forests; in the study area so far known from the Eastern Alps only. – **Au**: O, N. **Ge**: OB, Schw.

***Lecanora intricata* (Ach.) Ach.**

Syn.: Biatora polytropa (Hoffm.) Fr. var. intricata (Ach.) Fr., *Lecanora mutabilis* Sommerf., Lecanora polytropa (Hoffm.) Rabenh. var. intricata (Ach.) Schaer., Lecanora varia (Hoffm.) Ach. var. intricata (Ach.) Th. Fr., *Parmelia intricata* Ach.

L – Subs.: sil – Alt.: 3–6 – Note: a circumpolar, arctic-alpine, ecologically wide-ranging silicicolous species; common in the Alps with optimum above treeline. – **Au**: V, T, S, K, St, O, N. **Ge**: Schw. **Sw**: BE, GR, LU, SZ, TI, UR, VD, VS. **Fr**: AHP, HAl, AMa, Isè, HSav. **It**: Frl, Ven, TAA, Lomb, Piem, VA, Lig. **Sl**: SlA.

***Lecanora intumescens* (Rebent.) Rabenh.**

Syn.: Lecanora subfusca (L.) Ach. var. intumescens (Rebent.) Flot., Ochrolechia parella (L.) A. Massal. var. tumidula (Pers.) Arnold *non auct.*, *Ochrolechia tumidula* (Pers.) Arnold *non auct.*, *Parmelia intumescens* Rebent., *Patellaria intumescens* (Rebent.) Trevis.

L – Subs.: cor – Alt.: 2–4 – Note: a cool-temperate species found on smooth subacid bark, with optimum in humid beech forests; widespread and locally common throughout the Alps. – **Au**: V, T, S, K, St, O, N, B. **Ge**: OB. **Sw**: BE, GL, GR, LU, SG, SZ, TI, UR, UW, VS. **Fr**: AHP, HAl, AMa, Drô, Isè, Sav, HSav, Var, Vau. **It**: Frl, Ven, TAA, Lomb, Piem, Lig. **Sl**: SlA, Tg. **Li**.

***Lecanora jamesii* J.R. Laundon**

L – Subs.: cor – Alt.: 2–4 – Note: a sorediate species with a whitish-grey thallus and circular, convex, farinose, pale yellow soralia, usually sterile; on bark of deciduous trees in rather humid situations; in the Alps it is rare, being more common in Western Europe. – **Au**: T, N. **Sw**: VS.

***Lecanora latro* Poelt**

L – Subs.: sil-par – Alt.: 4–5 – Note: a silicicolous species with usually small and scattered individual thalli developing on those of its obligate host, *Miriquidica nigroleprosa*, with minute yellowish areolae, lecanorine apothecia with blackish discs, and subspherical ascospores; there are scattered reports from the Alps, where the species is much rarer than its host. – **Au**: T, S, St. **Sw**: UR.

***Lecanora lecideoides* (Nyl.) Harm.**

Syn.: Lecanora rubrofusca B. de Lesd. var. nigra B. de Lesd., *Lecanora sbarbaroana* (Klem.) H. Magn. *ex* Sbarbaro, Lecanora subfusca (L.) Ach. var. lecideoides Nyl.

L # – Subs.: sil – Alt.: 1 – Note: a very poorly known species of siliceous rocks, reported from a few localities in Western and Southern Europe, including the base of the Western Alps. – **Fr**: Var. **It**: Lig.

***Lecanora leptacina* Sommerf.**

Syn.: Lecanora intricata (Ach.) Ach. var. leptacina (Sommerf.) Stizenb.

L – Subs.: bry, ter – Alt.: 5–6 – Note: a probably circumpolar, arctic-alpine lichen found on mosses (*Andreaea*, *Grimmia*) and plant debris in sites with a long snow cover above treeline, in areas with siliceous substrata; perhaps more widespread in the Alps, but not common. – **Au**: T, K. **Sw**: BE, GR, VS. **Fr**: AHP. **It**: Lomb, Piem.

***Lecanora leptacinella* Nyl.**

L – Subs.: bry, cor – Alt.: 4–5 – Note: an arctic-alpine species with thalli consisting of some scattered, yellowish-white areoles soon giving raise to lecanorine apothecia with brown to blackish discs; muscicolous on moribund mosses (*Polytrichum*, *Rhacomitrium*), rarely on other bryophytes or on branches of subalpine shrubs (*Rhododendron*), often together with *Japewia tornoensis* and/or *Lecidea polytrichinella*; in the Alps it might be more widespread, having been perhaps overlooked in some regions. – **Au**: V, T, S, K, St. **Sw**: GR.

***Lecanora leptyrodes* (Nyl.) Degel.**

Syn.: Lecanora angulosa (Schreb.) Ach. var. leptyrodes Nyl., *Lecanora nemoralis* Makar. *non auct.*, *Lecanora pycnocarpa* H. Magn.

L – Subs.: cor, xyl – Alt.: 2–4 – Note: a mainly cool-temperate early coloniser found on the smooth bark of young trunks and branches (mainly of *Fagus* and *Betula*) which, however, is able to persist on ancient trees, with optimum in beech forests; widespread and locally common throughout the Alps. – **Au**: V, S, K, O, N. **Ge**: OB. **Sw**: BE, FR, GL, GR, LU, SG, SZ, TI, UW, VD, VS. **Fr**: AHP, AMa, Drô, Vau. **It**: Frl, Ven, TAA, Lomb, Piem, VA, Lig. **Sl**: SlA, Tg.

***Lecanora leuckertiana* Zedda**

Syn.: *Lepraria leuckertiana* (Zedda) L. Saag

L – Subs.: cor – Alt.: 3 – Note: on old trees in humid, but well-lit situations, with optimum in humid Mediterranean forests. The species, which contains usnic acid and is always sterile, does not belong to *Lepraria*. – **Au**: K. **It**: TAA. **Sl**: SlA.

***Lecanora leucoderma* (Anzi) Stizenb.**

Syn.: *Zeora leucoderma* Anzi

L # – Subs.: cal – Alt.: 3 – Note: a species with a thin, white, smooth, cartilaginous thallus, adnate to sessile, small, apothecia with a reddish disc and a very thin margin separated from the disc, and small, ovoid, hyaline, simple ascospores; the type material, collected on shaded calcareous rocks in the Rhaetic Alps (Italy), at 1,900 m, is badly preserved (see Nimis 2016). The species might even belong to *Protoblastenia*. – **It**: Lomb.

***Lecanora lojkaeana* Szatala**

Syn.: *Squamaria ferruginea* Szatala

L – Subs.: sil – Alt.: 3–5 – Note: a rarely collected species known from the Alps, the Central European mountains, and Scandinavia, found beneath overhanging surfaces of hard siliceous rocks in upland areas; perhaps overlooked and more widespread in the Alps, being almost always sterile. – **Au**: V, T, S, K, St. **Sw**: GR, VS. **Fr**: HSav. **It**: TAA.

***Lecanora luteovernalis* Brodo**

L – Subs.: deb – Alt.: 5 – Note: a species of the *L. symmicta*-group with a verrucose to granulose, yellowish to yellowish-green thallus growing on plant remains, and apothecia with yellowish-green to black discs soon becoming convex and margins excluded, based on a type from Arctic Canada; in the Alps so far recorded from a single locality in Switzerland. – **Sw**: SZ.

***Lecanora magnussoniana* Hafellner & Türk**

Syn.: *Squamarina magnussonii* Frey & Poelt

L – Subs.: int – Alt.: 4–5 – Note: a species with small, ochraceous yellow, indistinctly rosulate thalli, sessile apothecia with thick thalline margins and brownish discs, and asci of *Lecanora*-type; on calcareous rocks under overhangs; known from a few scattered localities in the Alps. – **Au**: S, St. **Sw**: GR.

***Lecanora margacea* Poelt**

L – Subs.: int – Alt.: 5 – Note: a species with a thallus of whitish, roundish to later indistinctly lobate squamules, and sessile apothecia with lecanorine margins and brown discs; on calcareous schists and marly limestones; widespread in Europe but rare, with a few records from the Eastern Alps. – **Au**: V, T, S, K.

***Lecanora marginata* (Schaer.) Hertel & Rambold**

Syn.: *Biatora elata* (Schaer.) Hepp, *Lecanora eliminata* (Arnold) Nyl., Lecanora marginata (Schaer.) Hertel & Rambold subsp. elata (Schaer.) Clauzade & Cl. Roux, *Lecidea elata* Schaer., Lecidea elata Schaer. var. formata Maheu & A. Gillet, Lecidea elata Schaer. var. marginata (Schaer.) A. Massal., Lecidea elata Schaer. var. subfarinosa H. Magn., *Lecidea eliminata* (Arnold) Arnold, *Lecidea marginata* Schaer., Lecidea marginata Schaer. subsp. elata (Schaer.) Clauzade & Cl. Roux, Lecidea marginata Schaer. var. elata (Schaer.) Anzi, Lecidea marginata Schaer. var. subfarinosa (H. Magn.) J. Nowak & Tobol., *Lecidea shlidenii* Räsänen, *Lecidea sulphurella* Th. Fr., *Lecidella elata* (Schaer.) Körb., *Lecidella marginata* (Schaer.) Körb.

L – Subs.: cal, int – Alt.: 3–6 – Note: a circumpolar, arctic-alpine lichen found on limestone, dolomite, and more or less calciferous siliceous rocks; widespread throughout the Alps. – **Au**: V, T, S, K, St. **Ge**: Schw. **Sw**: BE, GL, GR, LU, TI, UR, UW, VD, VS. **Fr**: AHP, HAl, AMa, Isè, Sav, HSav. **It**: Frl, Ven, TAA, Lomb, Piem, VA.

***Lecanora minutissima* A. Massal.**

L # – cal – Alt.: 2 – Note: a species with a more or less farinose, whitish thallus, very small, gyalectiform, sessile apothecia with a yellowish disc and a persistent margin, 8-spored asci, and ovoid-fusiform, simple ascospores measuring *c.* 9 × 3.6 µm, also reported from the Czech Republic and Romania; this taxon, which perhaps belongs to *Myriolecis*, well deserves further study. The type (VER) was collected “*ad saxa jurassica Prov. Veronensis (Grozzana)”.* – **It**: Ven.

***Lecanora mughicola* Nyl.**

Syn.: Lecanora varia (Hoffm.) Ach. var. alpina Anzi *ex* Arnold, Lecanora varia (Hoffm.) Ach. var. melanocarpa Anzi *ex* Arnold

L – Subs.: xyl – Alt.: 3–4 – Note: a circumboreal-montane lichen found on hard lignum, mostly of conifers, in upland areas, with optimum in the subalpine belt; widespread throughout the Alps. – **Au**: V, T, S, K, St, O, N. **Ge**: OB, Schw. **Sw**: BE, GR, LU, SZ, TI, VS. **Fr**: AHP, HAl, AMa, Isè, Vau. **It**: Frl, Ven, TAA, Lomb, Piem, VA. **Sl**: SlA. **Li**.

***Lecanora mugosphagneti* Poelt & Vězda**

L – Subs.: cor – Alt.: 3 – Note: a species of the *L. albella*-group with a whitish thallus bearing confluent soralia, apothecia very rare, as in *L. albella*; on bark of *Pinus mugo* in bogs; in the study area so far known from a few localities in the Eastern Alps. – **Au**: V. **Sw**: LU.

***Lecanora nohedensis* Cl. Roux & M. Barbero**

L – Subs.: sil-par – Alt.: 2–3 – Note: a species with a strongly reduced thallus of scattered areoles, finally sessile apothecia with lecanorine margins and brownish-green discs, and broadly ellipsoid ascospores; parasitic on *Placopyrenium breussii* (which is itself a parasite on *Aspicilia calcitrapa*) on inclined to subvertical, well-lit faces of siliceous schists; in the study area so far known only from the base of the Western Alps. – **Fr**: AMa.

***Lecanora norvegica* Tønsberg**

L – Subs.: xyl – Alt.: 4 – Note: a species recalling *Loxospora elatina*, but with a very different secondary chemistry (atranorin, protocetraric acid), so far only known in the sterile state, characterised by an episubstratic pale grey thallus with a marginal part consisting of minute areolae, and a central continuous part with minute tubercles which become sorediate; on trunks of coniferous trees in sites with a continental climate; in the study area so far known only from the Western Alps (Switzerland). – **Sw**: VS.

***Lecanora orbicularis* (Schaer.) Vain.**

Syn.: Lecanora concolor Ramond var. angustata (Arnold) Jatta, Lecanora polytropa (Hoffm.) Rabenh. var. orbicularis Schaer.

L – Subs.: sil – Alt.: 4–6 – Note: an arctic-alpine lichen growing on hard siliceous rocks, often on steeply inclined to underhanging faces, with optimum above treeline; widespread in the siliceous Alps. – **Au**: V, T, S, K, St. **Sw**: BE, GR, VS. **Fr**: AHP, HAl, AMa, Sav. **It**: TAA, Lomb, Piem, VA.

***Lecanora orosthea* (Ach.) Ach.**

Syn.: *Biatora orosthea* (Ach.) W. Mann, Lecanora sulphurea (Hoffm.) Ach. var. orosthea (Ach.) Flagey, *Lecidea orosthea* (Ach.) Ach., *Lichen orostheus* Ach., *Zeora orosthea* (Ach.) Flot.

L – Subs.: sil – Alt.: 2–5 – Note: on vertical or underhanging surfaces of siliceous rocks protected from rain in upland areas; certainly more widespread in the Alps. – **Au**: ?V, ?T, K. **Sw**: BE, VS. **Fr**: HSav, Var, Vau. **It**: TAA, Lomb.

***Lecanora pallidesulphurea* Schaer.**

L # – Subs.: sil – Alt.: 4–5 – Note: a silicicolous species with a pale yellow, pulverulent, rimose to areolate thallus, and innate to sessile, marginate apothecia with black convex discs; only known from the type locality in the Western Alps (Switzerland), on granite. – **Sw**: VS.

***Lecanora pallidiformis* (Anzi) Bagl.**

Syn.: *Lecidea pallidiformis* Anzi (“*pallidaeformis*”)

L # – Subs.: sil – Alt.: 4–5 – Note: a species with a verrucose-granulose, white thallus, immaginate, subglobose, black apothecia with a greenish pruina, a reddish epithecium, a yellowish hypothecium, and simple, ellipsoid, hyaline ascospores measuring 10–12 × 4–5 µm; the type, which well deserves further study, was collected on mica-schists in the Alps near Sondrio (Italy). – **It**: Lomb.

***Lecanora pannonica* Szatala**

L – Subs.: sil – Alt.: 2–4 – Note: differing from *L. caesiosora* in the thallus of bullate areolae, with granular, bluish-grey to dark grey soredia in soralia usually located at the edge of the areoles, as well as in the presence of gangaleoidin; on hard siliceous rocks, in the Alps also on man-made substrata, especially on vertical faces, very much overlooked, or confused with other species. – **Au**: S, St. **It**: TAA, Piem.

***Lecanora phaeostigma* (Körb.) Almb.**

Syn.: *Biatora flavella* Blomb., *Biatora phaeostigma* Körb., *Lecanora obscurella* (Sommerf.) Hedl., *Lecidea nitida* Sommerf., *Lecidea obscurella* (Sommerf.) Nyl.

L – Subs.: cor, xyl – Alt.: 2–4 – Note: this species, which is related to *L. cadubriae*, seems to be widespread in Northern and Central Europe; it grows on the bark of conifers, especially near the base of the trunks, more rarely on lignum of decorticated trunks, with optimum in the upper montane and subalpine belts. – **Au**: V, T, S, K, St, O, N. **Ge**: OB. **Sw**: FR, GR, LU, TI, UW, VS. **Fr**: Vau. **It**: Ven.

***Lecanora polycarpella* Zahlbr.**

Syn.: *Lecanora polycarpa* Anzi *nom.illeg*.

L # – Subs.: cal – Alt.: 2–3 – Note: a very poorly known calcicolous species characterised by a spreading, thin, grey, minutely squamulose-areolate thallus, the areolae with a white margin merging with the concolorous prothallus, very numerous, minute, sessile apothecia with a brownish-black, plane to convex disc and a very thin, white-pulverulent thalline margin, 8-spored asci, and hyaline, ovoid, simple ascospores measuring 4.5–6 × 9–10 µm; reported only from the type locality and from the Eastern Alps (Veneto). – **It**: Ven, Lomb.

***Lecanora polytropa* (Hoffm.) Rabenh.**

Syn.: *Biatora polytropa* (Hoffm.) Fr., *Lecanora illusoria* (Ach.) Leight., Lecanora varia (Hoffm.) Ach. var. illusoria Ach., *Verrucaria polytropa* Hoffm.

L – Subs.: sil, int, xyl, cor – Alt.: 2–6 – Note: a cool-temperate to arctic-alpine, circumpolar, ecologically wide-ranging lichen found on siliceous rocks wetted by rain, with a wide altitudinal range, but most frequent near and above treeline; widespread and very common throughout the Alps. – **Au**: V, T, S, K, St, O, N, B. **Ge**: OB, Schw. **Sw**: BE, GR, LU, SZ, TI, UR, UW, VD, VS. **Fr**: AHP, HAl, AMa, Isè, Sav, HSav, Var, Vau. **It**: Frl, Ven, TAA, Lomb, Piem, VA, Lig. **Sl**: SlA. **Li**.

***Lecanora populicola* (DC.) Duby**

Syn.: *Lecanora distans* (Ach.) Ach., Lecanora subfusca (L.) Ach. var. distans (Ach.) D. Dietr., *Parmelia distans* (Ach.) Mart., *Patellaria populicola* DC.

L – Subs.: cor – Alt.: 2–3 – Note: a cool-temperate to circumboreal-montane lichen found especially on *Populus tremula* and *Alnus* in the montane belt; apparently more frequent in the Southern and Western Alps. – **Sw**: GR, VS. **Fr**: AHP, HAl, AMa, HSav, Var. **It**: Ven, TAA, Lomb, Piem, Lig.

***Lecanora praesistens* Nyl.**

Syn.: *Lecanora pleiospora* J. Steiner, Lecanora pleiospora J. Steiner f. diluta J. Steiner

L – Subs.: cor – Alt.: 3–4 – Note: a polyspored epiphytic species of the *L. subfusca*-group with epihymenial crystals dissolving in K (*pulicaris*-type); widespread in the Alps, but rarely reported. – **Au**: S, St. **Sw**: BE, GL, GR, LU, SG, SZ, UR, VD, VS. **Fr**: Isè, Sav, Vau.

***Lecanora printzenii* Pérez-Ortega, Vivas & Hafellner**

L – Subs.: sil-par – Alt.: 5 – Note: lichenicolous on various species of *Umbilicaria*, causing serious damages in the areas of the lichen where it grows; so far only reported from the Eastern Alps (Austria). – **Au**: St.

***Lecanora protecta* Bagl. & Carestia**

L # – Subs.: sil – Alt.: 5 – Note: thallus ash-grey, K+ intensely brownish red, warty, on a pale prothallus, apothecia of various sizes, frequent or scattered, with a brown, flat disk, and a rather thick, wavy-wrinkled, indented, permanent margin, spores 8 per ascus, simple, ellipsoidal-spindle-shaped, 12–14 µm long. This species, which probably belongs to *Protoparmelia*, is known only from the type collection. – **It**: Piem.

***Lecanora pseudistera* Nyl.**

Syn.: *Lecanora atrofusca* Maheu & Werner, *Lecanora clauzadei* B. de Lesd., *Lecanora ripartii* *sensu* Poelt *non* Lamy, *Lecanora rubrofusca* B. de Lesd.

L – Subs.: ter-sil, sil, int – Alt.: 1–2 – Note: a mild-temperate to Mediterranean species found on calciferous sandstone and other base-rich siliceous rocks in warm-dry areas, mostly at low elevations; apparently more frequent in the Western and Southern Alps. – **Sw**: TI, VS. **Fr**: AMa, Var, Vau. **It**: TAA, Lomb, Piem, VA, Lig.

***Lecanora pseudosarcopidoides* M. Brand & van den Boom**

L – Subs.: xyl – Alt.: 3–4 – Note: a recently-described lignicolous species found on rotting trunks and wood of conifers, mainly in the subalpine belt, in species-poor stands, almost always associated with *Parmeliopsis ambigua*. It is superficially similar to *L. saligna*, differing in the form of the conidia and in other minor morphological characters, and is certainly much more widespread in the Alps. – **Au**: T, K. **Sw**: SZ, VS. **Fr**: AHP, HAl, AMa, Sav. **It**: TAA.

***Lecanora pulicaris* (Pers.) Ach.**

Syn.: *Lecanora chlarona* (Ach.) Nyl., *Lecanora coilocarpa* (Ach.) Nyl. *non auct.*, *Lecanora detrita* (Hoffm.) Ach., *Lecanora pinastri* (Schaer.) H. Magn., Lecanora subfusca (L.) Ach. var. detrita (Hoffm.) A. Massal., Lecanora subfusca (L.) Ach. var. pinastri Schaer., *Patellaria pulicari*s Pers.

L – Subs.: cor, xyl – Alt.: 2–4 – Note: a cool-temperate to boreal-montane, circumpolar lichen found on bark of conifers, more rarely of broad-leaved trees, and on lignum, both on twigs and trunks; widespread throughout the Alps. – **Au**: V, T, S, K, St, O, N, B. **Ge**: OB, Schw. **Sw**: BE, GL, GR, LU, SG, SZ, TI, UR, UW, VS. **Fr**: AHP, HAl, AMa, Drô, Isè, Sav, HSav, Var, Vau. **It**: Frl, Ven, TAA, Lomb, Piem, VA, Lig. **Sl**: SlA, Tg. **Li**.

***Lecanora quercicola* Coppins & P. James**

L – Subs.: cor – Alt.: 3 – Note: a species of the *L. saligna*-group with a granular to finely warted thallus and lecanorine apothecia with brown discs covered by a white to yellowish pruina, frequently with conidiomata containing broadly fusiform, curved macroconidia; on the bark of deciduous trees in parks and open woodlands; most common in Western Europe, with a few records from the Eastern Alps (Austria) and the Insubrian district (Italy). – **Au**: St. **It**: Lomb.

***Lecanora reagens* Norman**

L – Subs.: int, sil – Alt.: 2–5 – Note: a species found under overhangs of hard, mineral-rich siliceous rocks (gneiss, schists), mostly in fissures, occasionally on epilithic mosses, with optimum in upland areas. – **Au**: ?V, T, S, K, St, N. **Sw**: GR. **It**: TAA, Piem.

***Lecanora rouxii* S. Ekman & Tønsberg**

Syn.: *Lepraria flavescens* Cl. Roux & Tønsberg, *Lepraria flavescens* Clauzade & Cl. Roux *nom. inval*.

L – Subs.: cal – Alt.: 1–3 – Note: on vertical to underhanging surfaces of weathered or fissured calcareous rocks seldom wetted by rain, often in woodlands, mostly in natural habitats; certainly more widespread in the Alps. Closely related to *Lecanora swartzii*. – **Au**: ?V, T, S, K, St, O. **Ge**: OB. **Sw**: ?BE, SZ. **Fr**: AHP, AMa, Drô, Isè, Var, Vau. **It**: Ven.

***Lecanora rubicunda* Bagl.**

Syn.: *Lecanora augustinii* Erichsen, *Lecanora circumrubens* Samp., *Lecanora ochraceorosea* Werner, *Lecanora olivieri* Zahlbr., Lecanora subfusca (L.) Ach. var. sylvestris Nyl. *ex* Stizenb., *Lecanora sylvestris* (Nyl.) J. Steiner

L – Subs.: cor – Alt.: 1 – Note: a mainly Mediterranean lichen found on smooth bark in open maquis and garrigue vegetation; confused with *L. chlarotera* in the past, but certainly not common, even in the Mediterranean region, including a few records from the base of the Western Alps. – **Fr**: AMa.

**Lecanora rupicola (L.) Zahlbr. subsp. rupicola var. rupicola**

Syn.: *Lecanora glaucoma* (Hoffm.) Ach., *Lecanora rimosa* (Retz.) Röhl., *Lecanora sordida* (Pers.) Th. Fr., *Lecanora stenhammari* (Körb.) Jatta, *Lichen rupicola* L., *Parmelia sordida* (Pers.) Fr.

L – Subs.: sil, int, xyl – Alt.: 1–6 – Note: a widespread, holarctic silicicolous lichen; the other varieties have been not always distinguished, so their distribution is poorly known. – **Au**: V, T, S, K, St, O, N, B. **Ge**: OB. **Sw**: BE, GR, LU, TI, VD, VS. **Fr**: AHP, HAl, AMa, Isè, Sav, HSav, Var, Vau. **It**: Frl, Ven, TAA, Lomb, Piem, VA, Lig. **Sl**: SlA.

**Lecanora rupicola (L.) Zahlbr. subsp. rupicola var. efflorens Leuckert & Poelt**

Syn.: Lecanora rupicola (L.) Zahlbr. f. sorediata (Flot.) Zahlbr.

L – Subs.: sil – Alt.: 3–5 – Note: a name applied to sorediate morphs of *L. rupicola* *s.str.*, whose taxonomic value is uncertain; on inclined rock faces of siliceous rocks, in somehow more shaded-humid situations; probably more widespread in in the Alps. – **Au**: ?V. **Fr**: AHP, AMa. **It**: Frl, Piem.

**Lecanora rupicola (L.) Zahlbr. subsp. rupicola var. glaucescens (Sw.) Poelt & Vězda *comb. inval.***

L – Subs.: sil – Alt.: 4 – Note: a name applied to morphs with apothecia soon becoming convex, whose taxonomic status is uncertain, found under overhangs of siliceous rocks; distribution poorly known. – **Au**: St. **Fr**: AHP, AMa.

**Lecanora rupicola (L.) Zahlbr. subsp. subplanata (Nyl.) Leuckert & Poelt**

Syn.: Lecanora rupicola (L.) Zahlbr. var. subplanata (Nyl.) Clauzade & Cl. Roux, *Lecanora subradiosa* Nyl. *non auct.*, *Lecanora subplanata* Nyl.

L – Subs.: sil – Alt.: 1–5 – Note: a taxon resembling in habitus subsp. rupicola, but containing xanthones in the medulla. – **Au**: V, T, S, K, St. **Sw**: BE, GR, TI, UR, VS. **Fr**: AHP, AMa, HSav, Var. **It**: TAA, Lomb, Piem, VA. **Sl**: SlA.

**Lecanora rupicola (L.) Zahlbr. subsp. sulphurata (Ach.) Leuckert & Poelt**

Syn.: *Lecanora flavescens* (Bagl.) Bagl., Lecanora glaucoma (Hoffm.) Ach. var. sulphurata Ach., Lecanora rupicola (L.) Zahlbr. var. sulphurata (Ach.) Clauzade & Cl. Roux, Lecanora sordida (Pers.) Th. Fr. var. flavescens Bagl., *Lecanora sulphurata* (Ach.) Nyl.

L – Subs.: sil – Alt.: 1–5 – Note: a taxon containing xanthones in the cortex and therefore of a distinctly yellowish colour; in the Alps it is common only in dry situations, such as on south-exposed faces. – **Fr**: AMa, Var. **It**: Piem, VA, Lig.

***Lecanora salicicola* H. Magn.**

Syn.: *Lecanora migdina* *sensu* Poelt & Vězda, Lecanora pulicaris (Pers.) Ach. subsp. rhododendri (Harm.) Clauzade & Cl. Roux, *Lecanora rhododendri* (Harm.) Motyka, Lecanora subfuscata H. Magn. var. rhododendri (Harm.) Poelt

L – Subs.: cor – Alt.: 3–5 – Note: a probably circumboreal-montane lichen found on dead or decaying twigs of shrubs, especially *Rhododendron ferrugineum* in the subalpine belt; certainly widespread throughout the Alps. – **Au**: V, T, S, K, St. **Ge**: Schw. **Sw**: BE, GR, TI, VS. **Fr**: HAl, Isè. **It**: Ven, TAA, Lomb, Piem, VA.

***Lecanora saligna* (Schrad.) Zahlbr.**

Syn.: *Biatora effusa* (Hoffm.) A. Massal., *Lecanora effusa* (Hoffm.) Ach., Lecanora effusa (Hoffm.) Ach. var. sarcopis (Ach.) Th. Fr., Lecanora saligna (Schrad.) Zahlbr. var. sarcopis (Ach.) Tomin, *Lecanora sarcopis* (Ach.) Ach., *Lecanoropsis saligna* (Schrad.) M. Choisy, *Lichen salignus* Schrad., *Zeora effusa* (Hoffm.) Anzi

L – Subs.: xyl, cor – Alt.: 2–4 – Note: a holarctic, temperate to boreal-montane lichen found on hard, undecomposed wood or on bark of conifers; widespread throughout the Alps. – **Au**: V, T, S, K, St, O, N, B. **Ge**: OB, Schw. **Sw**: BE, GL, GR, LU, SG, SZ, TI, UR, UW, VD, VS. **Fr**: AHP, HAl, AMa, Isè, Sav, Var, Vau. **It**: Frl, Ven, TAA, Lomb, Piem, VA, Lig. **Sl**: SlA.

***Lecanora sarcopidoides* (A. Massal.) A.L. Sm.**

Syn.: *Biatora pumilionis* (Rehm) Oxner, *Biatora sarcopidoides* A. Massal., *Lecanora metaboliza* Nyl., *Lecanora metaboloides* Nyl., Lecanora piniperda Körb. subsp. sarcopidoides (A. Massal.) Hedl., *Lecanora pumilionis* (Rehm) Arnold, Lecanora sarcopidoides (A. Massal.) A.L. Sm. var. hypnophaga Poelt, *Lecidea sarcopidoides* (A. Massal.) Ohlert

L # – Subs.: cor, xyl – Alt.: 2–4 – Note: a taxon of the *L. symmicta*-complex, found on acid bark, most often of conifers, and on lignum, usually in upland areas; widespread throughout the Alps. – **Au**: V, T, S, K, St, N. **Ge**: OB, Schw. **Sw**: BE, GR. **Fr**: AHP, HAl, AMa, Sav, HSav. **It**: Frl, Ven, TAA, Lomb, Piem. **Sl**: SlA.

***Lecanora silvae-nigrae* V. Wirth**

L – Subs.: sil – Alt.: 3–6 – Note: a European orophyte found on inclined to underhanging surfaces of siliceous, often iron-rich rocks, mostly near and above treeline. In G there is also a sample collected by E. Frey in Switzerland (GR) at 3,125 m, on mosses. – **Au**: V, T, S, K, St. **Sw**: GR, TI, VS. **Fr**: AHP, HAl, AMa. **It**: Frl, Piem, VA.

***Lecanora soralifera* (Suza) Räsänen *non* H. Magn.**

Syn.: *Lecanora efflorescens* (Cromb.) Lettau, Lecanora intricata (Ach.) Ach. var. soralifera Suza, Lecanora polytropa (Hoffm.) Rabenh. var. efflorescens Cromb.

L – Subs.: sil, met – Alt.: 2–5 – Note: on iron-rich rocks, including pebbles; certainly more widespread in the Alps. – **Au**: V, T, K, St. **Sw**: GR. **Fr**: AMa. **It**: Frl, Piem.

***Lecanora sororia* Bagl. & Carestia**

L # – Subs.: sil – Alt.: 3–4 – Note: a species of uncertain affinity, with a verrucose-areolate, whitish-grey, K+ red thallus of minute, bullate areolae on a dark grey to black hypothallus, sessile apothecia with a brownish red, grey-pruinose disc and a thin thalline margin, thread-like, conglutinate paraphyses, a pale yellow epiphymenium, 8-spored asci, and uniseriate, ellipsoid ascospores measuring 20–30 × 10–12 µm; only known from the type collection, on gneiss; the type material would be well worthy of further study. – **It**: Piem.

***Lecanora stenotropa* Nyl.**

Syn.: Lecanora polytropa (Hoffm.) Rabenh. var. stenotropa (Nyl.) A.L. Sm.

L – Subs.: sil – Alt.: 2–5 – Note: this silicicolous species, which is closely related to *L. polytropa*, is apparently quite widespread and locally common along the Southern Alps (Roux et al. 2014). Several other records might be hidden under *L. polytropa*. – **Au**: ?T. **Fr**: AHP, AMa. **It**: Piem.

***Lecanora strobilina* (Spreng.) Kieff.**

Syn.: *Lecanora conizaea auct. non* (Ach.) Nyl., *Parmelia strobilina* Spreng.

L – Subs.: cor, xyl – Alt.: 1–4 – Note: a mainly temperate species found on acid bark and lignum, mostly in open woodlands; previously confused with other species and hence distribution rather poorly known. – **Au**: K, St. **Sw**: GL, GR, SZ, TI, UR, UW, VS. **Fr**: AHP, AMa, Drô, Var, Vau. **It**: Frl, TAA, Lig. **Sl**: SlA.

***Lecanora subaurea* Zahlbr.**

Syn.: *Lecanora aurea* Eitner *non* (Schaer.) Schaer., *Lecanora hercynica* Poelt & Ullrich

L – Subs.: met – Alt.: 2–5 – Note: a species with a conspicuous, grey-green to yellow-green, areolate thallus (due to the presence of rhizocarpic acid), with marginal soralia on the areoles; on metal-rich rocks (Fe, Cu), usually in well-lit habitats; widespread in Europe, including the Alps, except in some countries where suitable rocks are rare. – **Au**: V, T, S, K, St. **Sw**: GR, TI, VS. **Fr**: HSav. **It**: Frl, Lomb, Piem.

***Lecanora subcarnea* (Lilj.) Ach.**

Syn.: *Lecanora ochrinaeta* Ach., *Lecanora pallescens* A. Massal., Lecanora sordida (Pers.) Th. Fr. var. subcarnea (Lilj.) Th. Fr., *Lecanora trevisanii* A. Massal., *Lichen subcarneus* Lilj., *Zeora subcarnea* (Lilj.) Arnold

L – Subs.: sil – Alt.: 1–5 – Note: a mild-temperate to Mediterranean species found on steeply inclined to underhanging surfaces of siliceous rocks, mostly below the upper montane belt; widespread in the Alps, but generally not very common. – **Au**: V, T, K, N. **Ge**: Schw. **Sw**: GR. **Fr**: AMa, Isè, Sav, HSav, Var, Vau. **It**: Ven, TAA, Lomb, Piem, VA.

***Lecanora subcarpinea* Szatala**

L – Subs.: cor – Alt.: 1–4 – Note: a taxon of the *L. carpinea*-group with thallus and apothecial margins reacting Pd+ intensely yellow to orange, found on smooth bark of well-lit deciduous trees, including branches and twigs, mostly at low altitudes; not always distinguished, and hence distribution insufficiently known, but apparently widespread in the Alps. – **Au**: T, K, St. **Ge**: OB, Schw. **Sw**: GR, LU, SG, SZ, TI. **Fr**: AHP, HAl, AMa, Drô, Isè, Var, Vau. **It**: Frl, VA. **Sl**: SlA, Tg.

***Lecanora subintricata* (Nyl.) Th. Fr.**

Syn.: Lecanora varia (Hoffm.) Ach. var. subintricata Nyl., *Lecanoropsis subintricata* (Nyl.) M. Choisy

L – Subs.: xyl, cor – Alt.: 2–4 – Note: a circumboreal-montane lichen found on lignum and, more rarely, on the bark of conifers, mostly in upland areas, widespread throughout the Alps. – **Au**: V, T, S, K, St, O, N. **Ge**: OB, Schw. **Sw**: BE, GL, GR, LU, SZ, TI, UW, VD, VS. **Fr**: AHP, HAl, AMa, HSav, Vau. **It**: Frl, TAA, Lomb, Piem, VA, Lig. **Sl**: SlA.

***Lecanora subravida* Nyl.**

Syn.: Lecanora varia (Hoffm.) Ach. var. subravida (Nyl.) Nyl.

L # – Subs.: cor, xyl – Alt.: 3–4 – Note: this name is mostly used for a lignicolous taxon, perhaps of the *L. saligna*-group, but there are nomenclatural problems which we are unable to resolve at the moment; the species. which might be heterogeneous, has scattered records from the Alps. – **Ge**: Schw. **Sw**: SZ. **Fr**: HSav, Vau. **Li**.

***Lecanora sulphurea* (Hoffm.) Ach.**

Syn.: Lecanora polytropa (Hoffm.) Rabenh. var. sulphurea (Hoffm.) Schaer., *Lecidea sulphurea* (Hoffm.) Wahlenb., *Lichen sulphureus* Hoffm., *Zeora sulphurea* (Hoffm.) Flot.

L – Subs.: sil, sil-par – Alt.: 1–6 – Note: a widespread and locally common lichen which often starts the life-cycle on *Tephromela atra*, with a wide altitudinal range; in the Alps it is usually silicicolous, but in the Mediterranean mountains calcicolous forms are frequent. – **Au**: V, T, S, K, St, N. **Ge**: Schw. **Sw**: BE, GR, UR, VD, VS. **Fr**: AMa, Isè, HSav, Var, Vau. **It**: Frl, Ven, TAA, Lomb, Piem, VA, Lig.

**Lecanora swartzii (Ach.) Ach. subsp. swartzii**

Syn.: Lecanora glaucoma (Hoffm.) Ach. var. swartzii (Ach.) Nyl., Lecanora sordida (Pers.) Th. Fr. var. swartzii (Ach.) Rabenh., *Lecanora subradiosa auct. non* Nyl., *Lichen swartzii* Ach., Zeora sordida (Pers.) Körb. var. swartzii (Ach.) Körb.

L – Subs.: sil – Alt.: 3–5 – Note: an arctic-alpine lichen found on steeply inclined to underhanging surfaces of siliceous rocks, with optimum near and above treeline; most frequent in the Alps, but also occurring in the high Mediterranean mountains. – **Au**: V, T, S, K, St, N. **Sw**: GR, VS. **Fr**: AHP, HAl, AMa, Isè, HSav, Var, Vau. **It**: Ven, TAA, Lomb, Piem, VA, Lig.

**Lecanora swartzii (Ach.) Ach. subsp. caulescens (J. Steiner) Leuckert & Poelt**

Syn.: Lecanora subradiosa Nyl. var. caulescens J. Steiner

L – Subs.: sil – Alt.: 5 – Note: a lichen forming minute, podetia-like structures growing in dense cushions of *c.* 1–2 cm in diam. and up to 1 cm high, otherwise like subsp.swartzii; under overhangs of siliceous rocks, from the montane to the lower alpine belt; the distribution is poorly known, and in the study area is so far limited to the Eastern Alps (Austria). – **Au**: St.

**Lecanora swartzii (Ach.) Ach. subsp. nylanderi (Räsänen) Leuckert & Poelt**

Syn.: Lecanora subradiosa Nyl. var. nylanderi Räsänen

L – Subs.: sil – Alt.: 4–5 – Note: a lichen containing xanthones missing in subsp. swartzii, found under overhangs of siliceous rocks at high elevations; widespread in the Alps and rather common in areas with suitable habitats. – **Au**: T, S, K, St.

**Lecanora symmicta (Ach.) Ach. var. symmicta**

Syn.: *Biatora maculiformis* (Hoffm.) Beltr., *Biatora symmicta* (Ach.) A. Massal., Lecanora symmicta (Ach.) Ach. var. symmictera (Nyl.) Zahlbr., *Lecanora symmictera* Nyl., *Lecanora trabalis* (Ach.) Nyl., Lecanora varia (Hoffm.) Ach. var. maculiformis (Hoffm.) Rabenh., Lecanora varia (Hoffm.) Ach. var. symmicta Ach., *Lecidea symmicta* (Ach.) Ach., *Zeora maculiformis* (Hoffm.) Trevis.

L – Subs.: cor, xyl, cal, ter-sil – Alt.: 1–5 – Note: a holarctic, boreal-montane to temperate lichen found on acid bark, often on twigs of shrubs, with a wide altitudinal range; widespread and common throughout the Alps. – **Au**: V, T, S, K, St, O, N, B. **Ge**: OB, Schw. **Sw**: GL, GR, LU, SZ, TI, UR, UW, VS. **Fr**: AHP, HAl, AMa, Isè, Sav, HSav, Var, Vau. **It**: Frl, Ven, TAA, Lomb, Piem, VA, Lig. **Sl**: SlA, Tg.

***Lecanora symmicta* (Ach.) Ach. var. *sorediosa auct. non* Westman**

L # – Subs.: cor – Alt.: 3–5 – Note: in Central Europe this name is used for perhaps heterogenous sorediate lichens of the *L. symmicta*-group (but different from *L. orae-frigidae* R. Sant., a lignicolous maritime-arctic species not occurring in the Alps); on branches of shrubs (*Rhododendron*) and on plant debris and moribund mosses at high elevations; in the study area so far known only from the Eastern Alps (Austria), but probably overlooked or confused with other taxa elsewhere. – **Au**: V, T, S, K, St.

***Lecanora thysanophora* R.C. Harris**

L – Subs.: cor – Alt.: 2–3 – Note: an epiphytic species which is widespread and common in Eastern North America, superficially very similar to *Haematomma ochroleucum*, but with a different chemistry; probably much more widespread in the Alps, but confused with other species in the past. – **Au**: T, S, K, St, O, N. **Ge**: OB. **Sl**: SlA.

***Lecanora tolypodes* Poelt & Vězda**

L – Subs.: sil-par – Alt.: 5 – Note: a species of the *L. polytropa*-group growing as a parasite on *Aspicilia*-species, developing minute fertile thalli on steep faces of siliceous rocks; so far only known from the alpine belt in Switzerland. – **Sw**: GR.

***Lecanora torquata* (Fr.) Körb.**

Syn.: *Parmelia torquata* Fr.

L # – Subs.: sil – Alt.: 3–5 – Note: an often misunderstood species, probably a *Protoparmelia*. – **Sw**: BE, GR, TI, UR, VS. **It**: Ven, Lomb, Piem.

***Lecanora umbrosa* Degel.**

Syn.: *Lecanora neglecta* (Räsänen) Räsänen, *Lecanora sorediifera* (Th. Fr.) Räsänen *non* Fée, Lecanora subfusca (L.) Ach. var. sorediifera Th. Fr.

L – Subs.: sil, int – Alt.: 3–5 – Note: a cool-temperate to circumboreal-montane lichen found on steeply inclined to underhanging surfaces of weakly calciferous or base-rich, weathered siliceous rocks near or above treeline; widespread in the Alps but regionally overlooked, being often sterile. – **Au**: V, T, S, K, St, B. **Ge**: ?Ge. **Sw**: GR, SG, SZ, VS. **Fr**: AHP, HAl, AMa, Sav, HSav. **It**: Frl, Ven, Piem.

***Lecanora valesiaca* (Müll. Arg.) Stizenb.**

Syn.: *Placodium valesiacum* Müll. Arg., *Squamaria valesiaca* (Müll. Arg.) H. Olivier

L – Subs.: int, sil – Alt.: 1–4 – Note: on base-rich rocks (gneiss, porphyr, schists, etc.) containing some calcium, in warm-dry situations. – **Au**: T, S. **Sw**: GR, VS. **Fr**: Sav. **It**: TAA, Piem, VA, Lig.

***Lecanora varia* (Hoffm.) Ach.**

Syn.: *Lecanora subvaria* Nyl., Lecanora varia (Hoffm.) Ach. var. abbrevians Hedl., Lecanora varia (Hoffm.) Ach. var. denudata Bagl., Lecanora varia (Hoffm.) Ach. var. subvaria (Nyl.) H. Olivier, *Patellaria varia* Hoffm.

L – Subs.: xyl, cor – Alt.: 2–6 – Note: a cool-temperate to circumboreal-montane lichen found on hard lignum, more rarely on smooth, hard, acid bark, especially of conifers, in upland areas; widespread throughout the Alps. – **Au**: V, T, S, K, St, O, N, B. **Ge**: OB, Schw. **Sw**: BE, GR, LU, SZ, TI, VS. **Fr**: AHP, HAl, AMa, Isè, Sav, HSav, Vau. **It**: Frl, Ven, TAA, Lomb, Piem, VA, Lig. **Sl**: SlA, Tg. **Li**.

***Lecanora variolascens* Nyl.**

Syn.: ?*Lecanora bavarica* Poelt, Lecanora subfusca (L.) Ach. var. variolosa Körb.

L – Subs.: cor – Alt.: 2–3 – Note: mostly found at the base of trunks of isolated deciduous trees; so far only reported from a few localities in the Alps, but perhaps overlooked, being mostly sterile. – **Au**: St, O, N. **Fr**: AHP. **Ge**: OB, Schw. **It**: TAA.

***Lecanora vinetorum* Poelt & Huneck**

L # – Subs.: cor, xyl – Alt.: 2–4 – Note: a species of the *L. varia*-complex, characterised by the presence of vinetorin. – **Sw**: TI. **It**: TAA.

***Lecanora viridiatra* (Stenh.) Nyl. *ex* Zahlbr.**

Syn.: *Biatora viridiatra* Stenh., *Lecidea luteoatra* Nyl., *Lecidea viridiatra* (Stenh.) Schaer. *non* Ach. *nec* (Wulfen) Lamy, *Psora viridiatra* (Stenh.) Anzi

L – Subs.: sil – Alt.: 4–5 – Note: a species of steeply inclined to vertical surfaces of very hard siliceous rocks rich in quartz, in seepage tracks, near or above treeline. – **Au**: V, T, S, K, St. **Fr**: HAl, AMa, HSav. **It**: Piem, VA.

***Lecidea aemulans* (Arnold) Britzelm.**

Syn.: *Lecidella aemulans* Arnold

L – Subs.: cal – Alt.: 4–5 – Note: a calcicolous species of unclear relationship, as the paraphyses are given as conglutinated and the ascospores are relatively slim, provisionally placed here in *Lecidea* *s.lat.*; reported from the Alps, the Tatra Mnts., and Spitsbergen, with optimum near or above treeline. – **Ge**: OB. **Sw**: GL. **It**: TAA.

***Lecidea albofuscescens* Nyl.**

Syn.: *Biatora albofuscescens* (Nyl.) Arnold

L – Subs.: cor – Alt.: 3–4 – Note: a probably circumboreal species found on conifers, especially *Picea*, rarely on acid-barked deciduous trees such as *Betula* and *Sorbus aucuparia* in upland areas. It does not belong to *Lecidea* *s.str.* – **Au**: K, N. **Ge**: Ge. **It**: Ven, TAA. **Sl**: SlA.

***Lecidea albohyalina* (Nyl.) Th. Fr.**

Syn.: *Biatora albohyalina* (Nyl.) Bagl. & Carestia, Lecidea anomala Ach. f. albohyalina Nyl.

L – Subs.: cor, xyl – Alt.: 3–4 – Note: on smooth, acid bark. The species does not belong to *Lecidea* *s.str.* – **Au**: K, St. **Fr**: AHP. **It**: Ven, TAA, Piem.

***Lecidea albolivida* Lettau**

L # – Subs.: cor – Alt.: 3 – Note: a species with a thin, spreading, granular, whitish thallus, sessile apothecia with olive discs and darker margins, a dark brown hypothecium, 8-spored asci, and narrowly ellipsoid ascospores; on bark of coniferous trees; so far only recorded from the Eastern Alps in Germany, where it is probably extinct. – **Ge**: OB.

***Lecidea alpestris* Sommerf.**

L # – Subs.: ter-sil, bry – Alt.: 4–5 – Note: a circumpolar, arctic-alpine lichen found on naked soil, mosses and plant debris over siliceous substrata, more rarely on bark, on basal parts of conifers in the subalpine belt. Systematic position and delimitation of this species are still not clear. – **Au**: T. **Sw**: BE, TI. **It**: Frl, Ven, TAA, Lomb, Piem.

***Lecidea amabilis* Müll. Arg.**

L # – Subs.: sax – Alt.: 5 – Note: a poorly known species of unclear relationships, with a reddish to orange-white thallus forming isles on a black hypothecium, which may fuse in later stages, immersed, immarginate, black-brown (pale brown to reddish-brown when moist) apothecia with convex discs, a hyaline hypothecium, a brownish epihymenium, 8-spored asci, and oblong to ellipsoid ascospores (10–15 × 5–7 μm); on siliceous rocks in the high-alpine belt; only known from the type locality in the Western Alps (Switzerland). – **Sw**: VS.

***Lecidea andersonii* Filson**

Syn.: *Lecidea pseudopromiscens* Hertel & Rambold

L – Subs.: sil – Alt.: 5–6 – Note: a bipolar species, widespread in continental Antarctica and known from Iceland, with only a few records from the Alps, where it was probably overlooked. – **Sw**: VS.

***Lecidea antiqua* B. de Lesd.**

L # – Subs.: sil – Alt.: 3 – Note: a lecideoid species of unclear relationship (?Teloschistaceae), with a grey, areolate to verrucose thallus (K-!), orange apothecia to *c.* 0.5 mm in diam. (K+ ?red), finally sessile and persistently flat, with a thin margin slightly paler than the disc, a hyaline hypothecium, a granular, yellow epihymenium, hardly conglutinated paraphyses, 8-spored asci, and simple ascospores (to *c.* 20 µm long); only known from the type locality in the Western Alps (Italy), on schist. – **It**: VA.

***Lecidea areolata* Schaer.**

L # – Subs.: sil – Alt.: 5 – Note: a silicicolous species which probably belongs to the *Lecanora marginata*-group. – **Sw**: ?VS. **Fr**: HSav.

**Lecidea atrobrunnea (DC.) Schaer. subsp. atrobrunnea**

Syn.: *Lecidea funckii* Flot., *Lecidea protecta* H. Magn., *Lecidella atrobrunnea* (DC.) Körb., *Psora atrobrunnea* (DC.) A. Massal., *Sporastatia funckii* (Flot.) Dalla Torre & Sarnth.

L – Subs.: sil, cal, int – Alt.: 3–6 – Note: a bipolar, arctic-alpine to boreal-montane species of acid siliceous rocks in exposed situations, starting the life-cycle on species of *Bellemerea* or on *L. silacea*, with optimum near and above treeline. The species is chemically variable, and several chemotypes were distinguished; widespread throughout the Alps. – **Au**: V, T, S, K, St. **Ge**: ?Ge. **Sw**: BE, GR, SZ, TI, UR, VS. **Fr**: AHP, HAl, AMa, Isè, Sav, HSav. **It**: Ven, TAA, Lomb, Piem, VA, Lig.

**Lecidea atrobrunnea (DC.) Schaer. subsp. porphyrilica Hertel & Leuckert**

L – Subs.: sil – Alt.: 5–6 – Note: a rare chemical strain of the *L. atrobrunnea*-complex containing only porphyrilic acid; on siliceous rocks at high elevations; distribution insufficiently known. – **Sw**: GR, UR, VS.

**Lecidea atrobrunnea (DC.) Schaer. subsp. saxosa Hertel & Leuckert**

Syn.: *Lecidea gneissicola* Zahlbr., *Lecidea saxosa* R.S. Anderson *nom. inval.*, *Lecidea syncarpa* Zahlbr.

L – Subs.: sil – Alt.: 4–6 – Note: a rather common chemical strain of the *L. atrobrunnea*-complex containing the substances of the norstictic acid syndrome; widespread in the mountains of the Northern Hemisphere. – **Au**: T, St, N. **Sw**: VS. **Fr**: AHP, HAl, AMa.

**Lecidea atrobrunnea (DC.) Schaer. subsp. stictica Hertel & Leuckert**

L – Subs.: sil – Alt.: 4–6 – Note: a holarctic silicicolous taxon with optimum near and above treeline; widespread but rarely collected in the Alps. – **Au**: T, K. **Sw**: VS. **Fr**: AHP, HAl, AMa. **It**: Ven, Lomb.

**Lecidea atrobrunnea (DC.) Schaer. var. chamaelepis Hertel ined.**

L – Subs.: sil – Alt.: 5 – Note: a name used for a chemical strain with 2’-O-methylperlatolic acid, differing from *L. atrobunnea* *s.str.* also in the longer ascospores; on siliceous rocks in the alpine belt; distribution insufficiently known. – **Au**: T.

**Lecidea auriculata Th. Fr. subsp. auriculata**

Syn.: *Lecidea confoederans* Nyl.

L – Subs.: sil, int – Alt.: 3–6 – Note: a much misunderstood circumpolar, arctic-alpine species found on siliceous rocks in wind-exposed, sunny situations, in the high-alpine belt of humid mountains; much rarer in the Alps than the closely related *L. promiscens*. – **Au**: V, T, K, St, N. **Sw**: BE, GR, TI, UR, VD, VS. **Fr**: AHP, HAl, AMa, Sav, HSav. **It**: TAA, Piem, VA.

**Lecidea auriculata Th. Fr. subsp. brachyspora Th. Fr.**

Syn.: *Lecidea brachyspora* (Th. Fr.) Nyl.

L – Subs.: int – Alt.: 3–5 – Note: ecologically similar to the typical subspecies, from which it differs in the shorter spores, this taxon is also known from the Himalayas; the only certain records for the Alps are from Salzburg and Hautes-Alpes, all other records need confirmation. – **Au**: S. **Fr**: AHP. **It**: Ven, TAA, Piem.

***Lecidea bagliettoana* Zahlbr.**

Syn.: *Lecidea interjecta* Bagl. & Carestia *non* Nyl. *nec* (Müll. Arg.) Stizenb.

L # – Subs.: sil – Alt.: 4–5. Note: a species described from the Italian Alps, differing from *L. auriculata* in the very narrow, sublinear ascospores, also reported from Bulgaria. – **It**: Piem.

***Lecidea berengeriana* (A. Massal.) Nyl.**

Syn.: *Biatora berengeriana* A. Massal., *Biatora cupreiformis* (Nyl.) Arnold, *Biatora poetschiana* Körb, *Lecidea cupreiformis* (Nyl.) Nyl., *Lecidea miscella* Sommerf. *non* Ach., *Lecidea strasseri* Zahlbr., *Mycobilimbia berengeriana* (A. Massal.) Hafellner & V. Wirth

L – Subs.: ter-cal, bry-cal, deb – Alt.: 2–5 – Note: a circumpolar, arctic-alpine to boreal-montane lichen found on mosses and plant debris over calcareous substrata. The species does not belong to *Lecidea* nor to *Mycobilimbia* and is closely related to *Romjularia*; widespread throughout the Alps. – **Au**: V, T, S, K, St, O, N. **Ge**: OB, Schw. **Sw**: BE, GR, LU, SZ, TI, UR, UW, VD, VS. **Fr**: AHP, HAl, AMa, Isè, Sav, HSav, Var. **It**: Frl, Ven, TAA, Lomb, Piem. **Sl**: SlA. **Li**.

***Lecidea betulicola* (Kullh.) H. Magn.**

Syn.: *Biatora betulicola* Kullh., *Lecidea epiphaea* Nyl., *Lecidea lignaria* (Körb.) Nyl., *Lecidea plusiospora* Th. Fr.; incl. Lecidea betulicola (Kullh.) H. Magn. f. endamylea (Hedl.) Hinteregger

L – Subs.: cor, xyl – Alt.: 3–5 – Note: a boreal-montane species found on acid bark and lignum in upland areas. The generic position is still not clear. – **Au**: T, S, K, St, O. **Sw**: SZ, VD. **Fr**: Isè. **It**: TAA. **Sl**: SlA.

***Lecidea cervinicola* B. de Lesd.**

Syn.: Lecidea promiscua Nyl. var. cervinicola (B. de Lesd.) Clauzade & Cl. Roux

L – Subs.: sil – Alt.: 4–6 – Note: on low siliceous boulders, pebbles and flat stones scattered over the ground in alpine heaths, sometimes also on large rock faces. – **Au**: V, T, S, K. **Sw**: BE, GR, TI, VS. **Fr**: AHP, HAl, AMa, Isè. **It**: TAA, Lomb, Piem, VA.

***Lecidea commaculans* Nyl.**

Syn.: *Lecidea intercalanda* Arnold

L – Subs.: sil – Alt.: 4–5 – Note: an arctic-alpine species of humid siliceous rocks near and above treeline; probably more widespread in the Alps. – **Au**: ?V, S. **It**: TAA, Piem.

***Lecidea confluens* (Weber) Ach.**

Syn.: *Lecidea leptoceramia* Anzi, *Lecidea leucitica* (Schaer.) Arnold, *Lecidea vapulata* Anzi, *Lichen confluens* Weber, *Verrucaria confluens* (Weber) Weber ex. F.H. Wigg.; incl. Lecidea confluens (Weber) Ach. var. leucitica Schaer.

L – Subs.: sil – Alt.: 3–6 – Note: a boreal-montane to arctic-alpine, circumpolar species found on low siliceous stones and boulders with a long snow cover, with optimum near treeline, but reaching the nival belt; widespread throughout the Alps. – **Au**: V, T, S, K, St, N. **Ge**: OB, Schw. **Sw**: BE, GR, LU, SZ, TI, UR, VD, VS. **Fr**: AHP, HAl, AMa, Isè, Sav, HSav. **It**: Frl, Ven, TAA, Lomb, Piem, VA. **Sl**: SlA.

***Lecidea confluentula* Müll. Arg.**

Syn.: *Lecidea matildae* H. Magn., *Lecidea rimiseda* Nyl.

L # – Subs.: sil – Alt.: 3–5 – Note: known from NW Europe and the French Alps, this silicicolous species is related to *L. fuscoatra*, but is most frequent above treeline. – **Fr**: HAl, HSav. **It**: TAA.

***Lecidea confluescens* Nyl.**

Syn.: *Lecidea venustula* Arnold

L – Subs.: int, sil – Alt.: 4–6 – Note: on inclined to vertical faces of calciferous rocks, especially lime-containing schists; related to *L. lapicida*, but calcicolous; locally not rare in the Alps, and probably much more widespread, but overlooked. – **Au**: V, T, S. **Sw**: SZ. **Fr**: AHP, HAl, AMa, Sav. **It**: TAA, Piem, Lig.

***Lecidea decolor* Arnold**

L # – Subs.: sil – Alt.: 3–5 – Note: a species with a thin, rimose, whitish to greyish thallus (K-reaction negative, but medulla I+ violet), minute, black, non-pruinose apothecia, a brown hypothecium. a olive-green epihymenium, 8-spored asci, and oblong ascospores (12–15 × 5–6 μm); on siliceous rocks (*e.g.* mica-schist) at high elevations; only recorded from a few localities in the Alps. According to Hertel (*in litt.*) it belongs to the *L. lapicida*-group. – **Au**: T. **Sw**: VS.

***Lecidea diducens* Nyl.**

Syn.: Lecidea auriculata Th. Fr. var. diducens (Nyl.) Th. Fr., *Lecidea sarcogyniza* Nyl.

L – Subs.: sil – Alt.: 4–6 – Note: a circum – and bipolar, arctic-alpine silicicolous lichen, ecologically similar to, and closely related to *L. auriculata*. – **Au**: T. **Sw**: TI, VS. **It**: TAA, Lomb, Piem, VA, Lig.

***Lecidea dodecamera* Müll. Arg.**

L # – Subs.: cor – Alt.: 3 – Note: a species with a thin leprose thallus, sessile apothecia with biatorine margins, a hyaline hypothecium and 12–16-spored asci; on bark of coniferous trees in montane forests; in the study area so far known only from the Western Alps (France). – **Fr**: HSav.

***Lecidea ecrustacea* (Anzi *ex* Arnold) Arnold**

Syn.: *Lecidea complicata* H. Magn., *Lecidea lactea* Flörke *ex* Schaer. var. ecrustacea (Arnold) Clauzade & Cl. Roux, *Lecidella polycarpa* (Flörke *ex* Sommerf.) Körb. var. ecrustacea Anzi *ex* Arnold, *Lecidea pseudopilati* (Vain.) Vain.

L # – Subs.: sil – Alt.: 4–6 – Note: a silicicolous taxon with optimum above treeline, doubtfully distinct from L. lapicida var. pantherina; reported from several localities in the Alps. – **Au**: ?V, T, S, St. **Sw**: GR, UR, VS. **It**: TAA, Lomb, Piem, VA.

***Lecidea enclitica* Nyl.**

L # – Subs.: xyl – Alt.: 3–4 – Note: a lignicolous species described from Northern Finland, in the protologue compared to *Biatora globulosa*, but ascospores broader; so far only known from Switzerland in the Alps. – **Sw**: BE, GR.

***Lecidea erythrophaea* Flörke *ex* Sommerf.**

Syn.: *Biatora erythrophaea* (Flörke *ex* Sommerf.) Fr., *Lecidea hyalinella* (Körb.) Jatta, *Lecidea tenebricosa auct. non* Ach.

L – Subs.: cor – Alt.: 3–4 – Note: a mainly boreal-montane, probably circumpolar species of acid bark, especially of conifers, in humid-cold situations, *e.g.* in *Sphagnum* bogs. Closely related to *L. rhododendri* and certainly not a *Lecidea* *s.str.* – **Au**: V, T, S, K. **Ge**: OB. **Sw**: BE, GR, SZ, TI, VS. **It**: Frl, TAA, Lomb.

***Lecidea exigua* Chaub.**

Syn.: *Biatora decandollei* Hepp, *Biatora exigua* (Chaub.) Fr., *Biatora geographica* A. Massal., *Lecidea decandollei* (Hepp) Jatta

L – Subs.: cor – Alt.: 1–3 – Note: a mild-temperate to Mediterranean lichen found in very open woodlands, on smooth bark, especially on branches of deciduous trees; very rare in the Alps. – **Au**: St. **Fr**: Isè. **It**: Ven, Lomb, Piem.

***Lecidea fissuriseda* Poelt**

Syn.: *Mycobilimbia fissuriseda* (Poelt) Poelt & Hafellner

L – Subs.: cal, int – Alt.: 4–5 – Note: a probably circumpolar, arctic-alpine lichen found in thin fissures of calciferous rocks (calcareous schist, dolomite, much more rarely pure limestone) near and especially above treeline; certainly more widespread in the Alps. It does not belong in *Lecidea* *s.str.* nor in *Mycobilimbia*, being related to *Clauzadea* in the Porpidiaceae. – **Au**: T, S, K, St, N. **Ge**: OB. **Sw**: BE, GR. **It**: TAA, Piem, VA.

***Lecidea fuliginosa* Taylor**

Syn.: *Biatora conglomerata* A. Massal., *Biatora fuliginosa* (Taylor) Fr., *Lecidea confusa* Nyl., *Psora confusa* (Nyl.) Maheu & A. Gillet, *Psora conglomerata* (A. Massal.) Körb., *Psora fuliginosa* (Taylor) Stein, *Psora koerberi* A. Massal., *Toninia confusa* (Nyl.) Boistel

L – Subs.: sil – Alt.: 2–4 – Note: in small fissures of hard siliceous rocks in open, but wind-protected situations, mostly in upland areas. Most probably not a *Lecidea* *s.str.* – **Au**: T, S, K, St. **Sw**: GR, VS. **Fr**: Var. **It**: TAA, Lomb, Piem, VA.

***Lecidea fuscoatra* (L.) Ach.**

Syn.: *Lecidea algeriensis* Zahlbr., *Lecidea fumosa* (Hoffm.) Ach., *Lecidea prostratula* Stirt., *Lecidea psoroides* Bagl. & Carestia, *Lecidea sardoa* Bagl., *Lecidea trabicola* Erichsen, *Lichen fuscoater* L., *Patellaria fumosa* (Hoffm.) Hoffm., *Psora fumosa* (Hoffm.) A. Massal., *Psora prostratula* (Stirt.) Walt. Watson

L – Subs.: sil – Alt.: 1–5 – Note: a mainly temperate, widespread, extremely variable lichen found on rock faces wetted by rain on a wide variety of substrata, from base-rich siliceous rocks to brick and roofing tiles. *L. grisella* was not always distinguished from this species in the past; widespread throughout the Alps. – **Au**: V, T, S, K, St, O, N, B. **Ge**: OB, Schw. **Sw**: BE, GR, LU, SZ, TI, UR, VD, VS. **Fr**: AHP, HAl, AMa, Isè, Sav, HSav, Var, Vau. **It**: Frl, Ven, TAA, Lomb, Piem, VA, Lig. **Sl**: SlA.

***Lecidea fuscoatrata* Nyl.**

Syn.: Psora subfumosa (Arnold) Arnold var. fuscoatrata (Nyl.) Arnold

L # – Subs.: sil – Alt.: 3–5 – Note: a taxon resembling *L. fuscoatra*, but thallus C – and ascospores less than 10 µm long, probably belonging to the *L. atrobrunnea*-group and close to or even identical with *L. subfumosa*; on siliceous rocks in the alpine belt; the distribution is insufficiently known. – **Au**: T. **Fr**: Sav. **It**: TAA.

***Lecidea globulispora* Nyl.**

Syn.: *Biatora antiloga* (Stirt.) Walt. Watson, *Lecidea antiloga* Stirt., *Lecidea infralapponica* Vain., *Lecidella antiloga* (Stirt.) M. Choisy

L – Subs.: xyl – Alt.: 3–4 – Note: a mainly cool-temperate to boreal-montane lichen also known from the Southern Hemisphere found on hard, exposed lignum, more rarely on conifer bark; to be looked for further in the Alps. The generic position of this species, related to the North American *L. paddensis* (Tuck.) Zahlbr., is not clear. – **Sw**: VS. **Fr**: Vau.

**Lecidea goniophila Flörke var. gracilis (Arnold) ined. (provisionally placed here, ICN Art. 36.1b)**

Syn.: Lecidella goniophila (Flörke) Körb. var. gracilis Arnold

L # – Subs.: cal – Alt.: 4–5 – Note: a taxon recalling *Lecidella stigmatea*, but paraphyses more conglutinated and ascospores narrower and often with pointed ends (to be compared with *L. aemulans*); on boulders of dolomite, so far known only from the Eastern Alps (Austria). – **Au**: T.

***Lecidea grisella* Flörke**

Syn.: *Biatora livescens* (Leight.) Walt. Watson, Lecidea fumosa (Hoffm.) Ach. var. grisella (Flörke) Müll. Arg., Lecidea fuscoatra (L.) Ach. var. grisella (Flörke) Nyl., Lecidea grisella Flörke f. mosigii (Ach.) Zahlbr., *Lecidea livescens* Leight., *Lecidea segregula* Nyl.

L # – Subs.: sil – Alt.: 2–4 – Note: this taxon was subsumed for a long time into *L. fuscoatra*, but appears to be a well-distinct species, mainly distinguished by the rimose instead of areolate thallus. It grows on base-rich siliceous rocks, often on man-made substrata, *e.g.* on roofing tiles, and seems to be most frequent at lower elevations than *L. fuscoatra*. Some authors, however (*e.g.* Roux et al. 2014) still prefer to treat this taxon as a variety of the extremely polymorphic *L. fuscoatra*. – **Au**: V, T, S, K, St, B. **Ge**: OB, Schw. **Sw**: GR, SZ, TI, UR. **Fr**: AMa, Sav, HSav, Var, Vau. **It**: Frl, Ven, TAA, Piem, Lig.

***Lecidea grummannii* Hertel**

L – Subs.: sil-par – Alt.: 5 – Note: a rare species of the *L. atrobrunnea*-complex with pale brownish, glossy areoles and sunken, black, glossy apothecia producing broadly ellipsoid to subglobular ascospores; parasitic on silicicolous brown *Acarospora*-species, on which it develops small insular thalli; only known from the Southern Alps in Austria. – **Au**: K.

***Lecidea haerjedalica* H. Magn.**

L – Subs.: sil, met – Alt.: 4–5 – Note: an arctic-alpine, silicicolous species of wind-exposed, snow-free sites, most frequent on crystalline schists near or above treeline; from the Alps there are several scattered records. – **Au**: S, K. **Sw**: VS. **Fr**: AHP, AMa. **It**: TAA, Lig.

***Lecidea huxariensis* (Beckh. *ex* J. Lahm) Zahlbr.**

Syn.: *Biatora huxariensis* Beckh. *ex* J. Lahm

L – Subs.: xyl – Alt.: 3 – Note: a rare species of unclear relationship, with minute blackish apothecia and asci with 8–12 small ascospores; on wood, including fences; in the study area so far only known from the Eastern Alps (Austria). – **Au**: N.

***Lecidea ileiformis*Fr.**

L – Subs.: ter-cal – Alt.: 3 – Note: a rare species with a thick whitish thallus, large, black, finally convex, virtually immarginate apothecia, and *c.* 10 µm long ascospores, based on a type from Dovrefjell in Southern Norway; on soil in upland areas; distribution in the Alps poorly documented. – **Sw**: VS. **Fr**: HSav.

***Lecidea infirmata* Arnold**

Syn.: Lecidea paupercula Th. Fr. f. infirmata (Arnold) Lettau

L # – Subs.: sil – Alt.: 5 – Note: known only from the type collection and from a single record from Austria, this silicicolous species is related to *L. atrobrunnea*. – **Au**: K. **It**: TAA.

***Lecidea inflata* Anzi**

L # – Subs.: ter-sil – Alt.: 4–5 – Note: a species with a thick, cartilaginous, rugose-plicate to bullate, yellowish white thallus forming small pillows, large, adnate, confluent, black apothecia which are first flat and thinly marginate, then convex, a brownish epithecium, a pale hypothecium, coherent paraphyses, 8-spored asci, and simple, hyaline, ellipsoid ascospores measuring *c.* 18.9 × 13 µm; only known from the type collection and from a few localities in the mountains of Central Europe, this taxon deserves further study. – **It**: Lomb.

***Lecidea inturgescens* Nyl.**

L # – Subs.: sil – Alt.: 2–3 – Note: a taxon growing on siliceous rocks, with optimum in the montane belt. Closely related to *L. fuscoatra*. – **Fr**: HSav. **It**: Lomb.

***Lecidea isidiosa* Anzi**

L # – Subs.: bry-sil – Alt.: 5 – Note: this species of uncertain affinity, characterised by a whitish, leprose-isidiose thallus, small, brown, convex apothecia with a dark hypothecium and lax paraphyses, and simple spores measuring 13–17 × 3–5 µm, is known only from the type collection above Bormio (Italy). – **It**: Lomb.

***Lecidea italica* B. de Lesd. *nom.illeg. non* Wedd.**

L # – Subs.: sil – Alt.: 5 – Note: a species with a white, thin thallus of scattered to contiguous, angular areoles reacting K-, on a black hypothallus, a I+ blue medulla, numerous, black, sometimes thinly pruinose, sessile apothecia (2–2.5 mm in diam.), the disc urceolate to concave, the margin prominent and wavy, an olivaceous epithecium reacting K-, a strongly amyloid hymenium of coherent paraphyses, a colourless hypothecium, 8-spored asci, and ellipsoid to oblong, hyaline, simple ascospores measuring 12–17 × 6–7(-8) μm; only known from the type collection in Valpelline, at 2,900 m. – **It**: VA.

***Lecidea laboriosa* Müll. Arg.**

Syn.: *Lecidea leptoboloides* Nyl., *Lecidea lithophilopsis* Nyl.

L # – Subs.: sil – Alt.: 4–5 – Note: a still insufficiently known species of *L. plana*-group with narrower ascospores; on siliceous rocks, usually at high elevations; distribution poorly documented. – **Au**: V. **Sw**: VS.

***Lecidea labulata* (Hepp *ex* Metzler) Zahlbr.**

Syn.: *Biatora labulata* Hepp *ex* Metzler

L # – Subs.: cal – Alt.: 2–3 – Note: a calcicolous species of unclear relationship, with a thallus consisting of whitish grey, small squamules dispersed on a grey prothallus, sessile, globose, immarginate, black apothecia with an emerald-green hymenium, 8-spored asci, and simple, ellipsoid ascospores measuring 7–9 × 3–5 μm; only known from two localities in Austria and Switzerland. – **Au**: S. **Sw**: GR.

**Lecidea lapicida (Ach.) Ach. var. lapicida**

Syn.: *Biatora ochromela* (Ach.) Hepp, *Lecidea contiguella* Nyl., *Lecidea declinans* Nyl., *Lecidea declinascens* Nyl., *Lecidea dendroclinis* Nyl., *Lecidea hoelii* Lynge, *Lecidea lactea* Flörke *ex* Schaer. var. ochromela (Ach.) Arnold, *Lecidea ochromela* (Ach.) Anzi, *Lecidea scotoplaca* H. Magn., *Lecidea subinvoluta* Müll. Arg., *Lecidea subplanata* Vain., *Lecidea vestrogothica* H. Magn., *Lecidella lapicida* (Ach.) Körb., *Lecidella ochromela* (Ach.) Arnold, *Lichen lapicida* Ach.

L – Subs.: sil, met – Alt.: 3–6 – Note: a circumpolar, arctic-alpine to boreal-montane and cool-temperate species with a broad ecological range, found on hard, acid siliceous rocks, mostly in exposed, windy situations in upland areas; widespread throughout the Alps. – **Au**: V, T, S, K, St, N. **Ge**: OB, Schw. **Sw**: BE, GR, LU, SZ, TI, UR, VD, VS. **Fr**: AHP, HAl, AMa, Isè, Sav, HSav. **It**: Frl, Ven, TAA, Lomb, Piem, VA, Lig. **Sl**: SlA. **Li**.

**Lecidea lapicida (Ach.) Ach. var. pantherina (Hoffm.) Ach.**

Syn.: *Lecidea cyanea* (Ach.) Th. Fr., *Lecidea lactea* Flörke *ex* Schaer., Lecidea lapicida (Ach.) Ach. var. lactea (Flörke *ex* Schaer.) V. Wirth, *Lecidea pantherina* (Hoffm.) Th. Fr., *Lecidea peralbida* (Th. Fr.) H. Olivier, *Lecidea polycarpa* Flörke, *Lecidea theiodes* Sommerf., *Lecidea variegata* Fr., *Lecidella lactea* (Flörke *ex* Schaer.) Arnold, *Lecidella pantherina* (Hoffm.) Stein, Verrucaria contigua Hoffm. var. pantherina Hoffm.

L # – Subs.: sil – Alt.: 3–6 – Note: doubtfully distinct from var. lapicida and perhaps just a chemical strain of the latter, with a similar distribution and ecology; widespread and common throughout the Alps. – **Au**: V, T, S, K, St, N. **Ge**: OB. **Sw**: BE, GR, TI, VS. **Fr**: AHP, HAl, AMa, Isè, Sav, HSav, Var. **It**: Frl, Ven, TAA, Lomb, Piem, VA, Lig. **Sl**: SlA.

**Lecidea lapicida (Ach.) Ach. var. spilotica (Nyl.) Clauzade & Cl. Roux**

Syn.: *Lecidea spilotica* Nyl.

L # – Subs.: sil – Alt.: 3–5 – Note: a not generally recognised taxon, very close to L. lapicida var. pantherina, based on type from the Pyrenees on schist, accepted only in the French lichenological literature. – **Fr**: AHP, AMa, Isè, Sav.

***Lecidea lenticella* (Arnold) Stizenb.**

Syn.: *Biatora lenticella* Arnold

L – Subs.: cal – Alt.: 4–5 – Note: a species with a thin whitish thallus recalling *Catillaria lenticularis*, but apart from the non-septate ascospores, with conglutinated paraphyses lacking clavate tips and pigment caps; on limestone from the subalpine to the alpine belt. – **Au**: T. **Ge**: OB. **Sw**: VS.

***Lecidea leprarioides* Tønsberg**

Syn.: Lecidea turgidula Fr. var. pulveracea Th. Fr.

L – Subs.: xyl, cor – Alt.: 3–4 – Note: a rather rare, mainly boreal species of acid bark in upland areas, related to *L. turgidula*. – **Au**: St, N. **Ge**: Ge. **Sw**: BE, GR, LU, SG, UR, VD, VS. **It**: TAA.

***Lecidea leprosolimbata* (Arnold) Lettau *ex* Poelt**

Syn.: Lecidea atrobrunnea (DC.) Schaer. f. leprosolimbata (Arnold) Lettau, Psora atrobrunnea (DC.) A. Massal. var. leprosolimbata Arnold

L – Subs.: int-par, sil-par – Alt.: 3–6 – Note: on sunny, inclined, hard, weakly calciferous siliceous rocks, starting the life-cycle on the thalli of *Bellemerea subcandida*; certainly more widespread in the Alps, and locally even common. – **Au**: V, T, S, K, St. **Sw**: BE, GR, VS. **Fr**: AHP, HAl, Sav, HSav. **It**: TAA, Piem, VA, Lig.

**Lecidea leucothallina Arnold var. leucothallina**

Syn.: *Lecidea kujalae* Räsänen

L – Subs.: sil, met – Alt.: 4–5 – Note: an arctic-alpine species found on boulders and siliceous pebbles, especially on crystalline schist near the ground, in sites with a long snow cover, with optimum above treeline; known only from the Alps and rarely collected. – **Au**: T. **Sw**: GR. **It**: TAA, Lomb, VA.

**Lecidea leucothallina Arnold var. discrepans Rambold & Hertel**

Syn.: Lecidea leucothallina Arnold var. subdiscrepans Rambold & Hertel *ex errore*

L – Subs.: sil – Alt.: 6 – Note: on hard siliceous rocks, often in sites with a long snow cover; in the Alps hitherto only known from a single collection in Tyrol. – **Au**: T.

***Lecidea lithophila* (Ach.) Ach.**

Syn.: *Lecidea farinosa* H. Magn., *Lecidea heteromorpha* H. Magn., Lecidea lapicida (Ach.) Ach. f. lecanactis (A. Massal.) Arnold, Lecidea lapicida (Ach.) Ach. var. lithophila Ach., *Lecidea lithophiliza* Nyl., *Lecidea pruinosa auct.*, *Lecidella lithophila* (Ach.) Arnold, *Lecidella pruinosa* Körb.

L – Subs.: sil, met – Alt.: 2–6 – Note: an ecologically wide-ranging, pioneer species found on vertical to slightly inclined surfaces of acid siliceous rocks close to the ground, also on iron-rich substrata, with optimum above the montane belt; widespread throughout the siliceous Alps. – **Au**: V, T, S, K, St, O, N. **Ge**: OB, Schw. **Sw**: GR, TI, UR, VS. **Fr**: HAl, AMa, Isè, Sav, HSav. **It**: Ven, TAA, Lomb, Piem, VA, Lig. **Sl**: SlA.

***Lecidea lygaeoides* (Anzi) Jatta**

Syn.: *Biatora lygaeoides* Anzi

L # – Subs.: sil – Alt.: 3 – Note: a species with a brownish thallus of minute scattered granules, delimited by a well-developed bluish-black prothallus, small black apothecia with a flat disc and a thin proper margin, a brownish epi – and hypothecium, 8-spored asci, and simple, ellipsoid, hyaline ascospores measuring 13–14 × 6.8–8.6 µm; see also Nimis (1993: 393). – **It**: Lomb.

***Lecidea lyngei* Degel.**

Syn.: *Lecidea arnoldii* Lynge *non* (Kremp.) Nyl.

L # – Subs.: sil – Alt.: 5 – Note: a silicicolous species described (as *Lecidea arnoldii* Lynge) from Novaya Zemlya, perhaps belonging to *Miriquidica*, differing from *M. garovaglii* in the cortex reacting K+ red. Lynge argued in the protologue that “Psora aenea f. corrugata Arnold” might be a heterotypic synonym, which is the reason why the species turns up in a lichen checklist of the Alps. – **Au**: T.

***Lecidea magnussonii* Lynge**

L # – Subs.: bry – Alt.: 4 – Note: a species very closely related to *Schaereria cinereorufa*, with a thick, verrucose, brownish-grey thallus, black apothecia containing easily separating paraphyses and narrowly cylindrical asci with uniseriate, globose ascospores; overgrowing silicicolous mosses in the subalpine to alpine belts; for the study area there is a single record from the Eastern Alps (Austria). – **Au**: S.

***Lecidea malmeana* Zahlbr.**

Syn.: Lecidea enalliza Nyl. var. subplana Malme, *Lecidea microsporella* Malme *non* Lettau

L – Subs.: cor – Alt.: 3 – Note: a species with a thin, whitish thallus and small, blackish apothecia containing 16-spored asci with narrowly ellipsoid ascospores, based on a type from Sweden; on bark of coniferous trees in montane forests, with a single record from the Western Alps (Switzerland). – **Sw**: BE.

***Lecidea miscella* Ach. *non* Sommerf.**

L # – Subs.: bry – Alt.: 4–5 – Note: a species with a thick, granular-verrucose, whitish thallus, small, black, convex, immarginate apothecia with a brown hypothecium, conglutinated paraphyses, and ellipsoid to oblong ascospores, based on a type from Sweden; on soil and plant remains at high elevations, with a few records only from the Alps. – **Au**: S. **It**: TAA.

***Lecidea montanvertiana* Croz.**

Syn.: *Biatora montanvertiana* (Croz.) M. Choisy

L # – Subs.: bry – Alt.: 4 – Note: a species overgrowing bryophytes, with a white, granular thallus reacting K+ yellow, small biatorine, finally convex, greenish-black apothecia with a hyaline hypothecium, conglutinated paraphyses, and ellipsoid ascospores which are mostly longer than 15 µm; in the study area only known from a single station in the Western Alps (France), at 1,910 m. – **Fr**: HSav.

***Lecidea moritzii* B. de Lesd.**

Syn.: *Lecidea cacuminum* B. de Lesd. *non* Vain. *nec* H.Magn.

L # – Subs.: cal – Alt.: 5 – Note: a species with a chalky white, rimose to areolate, relatively thick thallus (2–4 mm in diam.), the older areoles becoming somewhat lobate with rounded lobes and surrounded by a hypothalline black margin, black marginate apothecia (to 2 mm in diam.), an emerald green epithecium and hypothecium (?), 8-spored asci, and hyaline, oblong to subglobose, simple ascospores (5–7 × 2–4 μm); on (presumably) calcareous rocks in the high-alpine belt; only known from the type locality in the Eastern Alps (Switzerland). – **Sw**: GR.

***Lecidea nivosa* Müll. Arg.**

Syn.: *Biatora nivea* Müll. Arg., *Lecidea nivea* (Müll. Arg.) Mig. *nom. inval. non* P. Crouan & H. Crouan

L # – Subs.: cal, int – Alt.: 6 – Note: a species resembling in habitus *Carbonea atronivea* but probably belonging to the *L. lapicida*-group, with a white, rather thin, rimose to areolate, rough thallus (no reaction with K, medulla I+ violet), small, black, *non* pruinose, marginate, first immersed, later adpressed apothecia (*c.* 0.5 mm in diam.), a brown hypothecium, a brown epihymenium with a greenish tinge, 8-spored asci, and hyaline, simple, ellipsoid ascospores (6–9 × 3–4.5 μm); on calcareous schists in the nival belt; only known from the type locality in the Western Alps (Switzerland). – **Sw**: VD.

***Lecidea nylanderi* (Anzi) Th. Fr.**

Syn.: *Biatora nylanderi* Anzi, *Lecidea fuscescens* Nyl. *non* Sommerf.

L – Subs.: cor – Alt.: 2–4 – Note: a probably circumboreal-montane lichen found on the bark old conifers inside forests, much more rarely on lignum, usually in upland areas. The species, related to *Myochroidea leprosula*, does not belong to *Lecidea* *s.str.* – **Au**: T, S, K, St, O, N. **Ge**: OB. **Sw**: GL, GR, LU, TI, UR, UW, VS. **Fr**: AHP, Isè, Vau. **It**: Ven, TAA, Lomb.

***Lecidea obluridata* Nyl.**

L – Subs.: sil – Alt.: 4–5 – Note: a silicicolous species resembling *L. fuscoatra*, but thallus containing confluentic acid, apothecia adnate, flat, with thin margins, hypothecium brown, and ascospores oblong, *c.* 10 µm long; based on a type from low elevation in the Pyrenees; the distribution in the Alps is poorly known. – **Au**: V, T, S. **Sw**: GR. **Fr**: AMa, Isè, HSav. **It**: Piem.

***Lecidea paratropoides* Müll. Arg.**

L – Subs.: sil – Alt.: 2–5 – Note: on siliceous rocks near the ground in dry areas, with optimum near and above treeline; a member of the *L. auriculata*-group, known from Central Asia, the dry valleys of the Alps, the mountains of Sicily and the Pyrenees, mostly in continental areas. – **Au**: T, S. **Sw**: GR, VS. **It**: TAA, VA.

***Lecidea paupercula* Th. Fr.**

Syn.: *Lecidea atroocarpoides* Vain., *Lecidea kittilensis* Vain.

L # – Subs.: sil – Alt.: 3–5 – Note: a species close to *L. praenubila*, not generally accepted as distinct, characterised by the thin areoles with a red-brown centre and paler margin and the dark brown hypothecium, based on a type from Northern Norway; on siliceous rocks at high elevations; the identity of Alpine records is uncertain. – **Au**: T, K. **Fr**: AHP, AMa, Sav.

***Lecidea percutiens* Poelt**

L – Subs.: sil-par – Alt.: 2 – Note: a species of unclear relationships, only known in the sterile state, forming small, greenish, verrucose thalli with isidiate-sorediate papillae on the thalli of *Diploschistes scruposus*, mostly on sandstone at low elevations; so far only known from the Western Alps (France). – **Fr**: Vau.

***Lecidea personata* (Körb.) Jatta**

Syn.: *Lecidella personata* Körb.

L # – Subs.: sil – Alt.: 3–5 – Note: a very poorly known species reported from several scattered localities in eastern Central Europe. The Italian material, collected on granite near Bormio, was distributed by Anzi (Lich. Lang. 570). – **It**: Lomb.

***Lecidea pertecta* Hertel ined.**

L # – Subs.: sil – Alt.: 5 – Note: a species resembling *L. paratropoides*, but with a different chemistry (stictic acid syndrome); on siliceous rocks in the alpine belt; so far only known from the Eastern Alps (Austria). – **Au**: Au.

***Lecidea plana* (J. Lahm) Nyl.**

Syn.: *Catillaria eximia* Malme, *Catillaria stromatoides* H. Magn., *Lecidea enteromorpha* (Flot.) Vain., *Lecidea latypea* Ach., *Lecidella plana* J. Lahm

L – Subs.: sil, met – Alt.: 2–5 – Note: a circumpolar, arctic-alpine to boreal-montane lichen found on acid siliceous rocks, often on iron-rich substrata, on low boulders wetted by rain in humid areas, with optimum near and above treeline. – **Au**: V, T, S, K, St, B. **Sw**: TI, VS. **Fr**: HAl, AMa, Isè, HSav. **It**: Ven, TAA, Lomb, Piem, VA. **Sl**: SlA.

***Lecidea plebeja* Nyl.**

Syn.: *Lecidea enalliza* Nyl.

L – Subs.: cor, xyl – Alt.: 3–4 – Note: a boreal to temperate-montane species with an indistinct to minutely verrucose thallus and usually small, black apothecia with thin margins, a brown hypothecium, and small, ovoid ascospores, based on a type from Finland; on wood and bark of conifers (*Picea*); from the Alps there are a few records only. – **Au**: T, S. **Sl**: SlA.

***Lecidea polycarpoides* (Müll. Arg.) Müll. Arg.**

Syn.: *Biatora polycarpoides* Müll. Arg.

L # – Subs.: sil, ?int – Alt.: 4–5 – Note: a species probably related to (or identical with) L. lapicida var. pantherina, with a thin, rimose to areolate, grey to smoke-grey thallus (reacting K+ yellow then red, medulla I+ violet, *fide* Hertel), black, non-pruinose, marginate, first immersed, later adpressed apothecia (*c.* 0.3–0.5 mm in diam.), a brownish hypothecium, a bluish-black epihymenium, 8-spored asci, and oblong to ellipsoid, simple, hyaline ascospores (12–15 × 5–7 μm); on siliceous (somehow calciferous?) rocks; only known from the type locality in the Western Alps (Switzerland). – **Sw**: VD.

***Lecidea polygonia* Flot. *ex* Nyl.**

L # – Subs.: sax – Alt.: 4 – Note: a taxon with an unresolved nomenclature of unclear application, compared by Nylander with *Immersaria athroocarpa*, from which it should differ in the smaller ascospores; on siliceous rocks, with some scattered records in Europe, including the Alps. A “*L. polygonia* Müll. Arg.” as cited in the Swiss checklist does not exist. This epithet goes back to L. fumosa var. polygonia introduced as a *nom. nud.* by Flotow, Lich. exs. no. 139 (1830), and was also cited by Nylander (Flora 64: 186, 1881) when he published a description under the name “*L. polygonia* (Flot.)”. If this is the protologue, the taxon has to be ascribed to Nylander as *L. polygonia* Flot. *ex* Nyl. A further complication is that Körber (Systema: 253, 1855) citing also the exsiccatum of Flotow, apparently published a taxon “*L. fumosa* α [var.] *nitida* [f.] *polygonia* Flot.” that has to be ascribed to Körber. This name, however, was not cited by Nylander, and therefore cannot be the protologue of Nylander’s species. – **Sw**: UR.

***Lecidea polytrichinella* Hertel, Obermayer & Poelt**

L – Subs.: bry – Alt.: 4–5 – Note: a tiny species of unclear relationships, with a thallus of minute, whitish areoles, small hemispherical, immarginate, brown to blackish apothecia, asci recalling those of *Biatora*, and small ascospores; encrusting leaflets of moribund *Polytrichum*, often together with *Lecanora leptacinella*; overall distribution arctic to temperate-alpine; widespread in the Alps but rarely collected. – **Au**: S, K, St. **Sl**: SlA.

***Lecidea praenubila* Nyl.**

Syn.: *Lecidea aeneola* (Arnold) Vain., *Lecidea atrocervina* Vain., *Lecidea helsingforsiensi*s Nyl.

L – Subs.: sil – Alt.: 4–6 – Note: an arctic-alpine to boreal-montane, perhaps circumpolar silicicolous species found on horizontal surfaces or on pebbles; much rarer in the alpine belt of the Alps than in Northern Europe. At least in Austria, there is no certain record: in the Alps the species might have been confused with *L. subfumosa*. – **Au**: ?T. **Sw**: GR, VS. **Fr**: AHP. **It**: TAA, VA.

***Lecidea privati* Müll. Arg.**

L # – Subs.: sax – Alt.: 5 – Note: a silicicolous species of unclear relationships, with a thallus of reddish-brown to greenish-brown, densely arranged, small areoles on a black prothallus, marginate, plane, black apothecia with a pigmented hypothecium and an olive-blackish epihymenium, 8-spored asci, and oblong to ellipsoid ascospores (9–10 × 4–5 µm); only known from the type locality in the Western Alps (Switzerland). – **Sw**: VS.

***Lecidea promiscens* Nyl.**

Syn.: Lecidea promiscua Nyl. var. promiscens (Nyl.) Clauzade & Cl. Roux, *Lecidea strepsodea* Nyl.

L – Subs.: sil – Alt.: 2–6 – Note: a circum – and bipolar, arctic-alpine to boreal-montane lichen found on boulders close to the ground and on siliceous pebbles in alpine heaths; widespread in the Alps. – **Au**: V, T, S, K, St. **Ge**: ?OB. **Sw**: BE, GR, TI, UR, VS. **Fr**: AHP, HAl, AMa, Isè, Sav, HSav. **It**: Frl, TAA, Piem, VA.

***Lecidea promiscua* Nyl.**

Syn.: *Lecidea dilabens* Th. Fr., *Lecidea gregalis* Arnold, *Lecidea speciosa* Müll. Arg.

L # – Subs.: sil – Alt.: 3–6 – Note: a member of the difficult *L. auriculata*-complex, closely related to *L. promiscens* and with a similar ecology; apparently common in the alpine belt of the Alps, but overlooked. – **Au**: T, K, St, N. **Sw**: SZ, VS. **Fr**: AHP, AMa, Isè. **It**: Frl, TAA.

***Lecidea proxima* Anzi**

L # – Subs.: cor – Alt.: 4 – Note: from the description this species appears to be related to *Ramboldia elabens*, differing in the intensely greenish apothecial disc, the pale hypothecium and the larger spores; the type material, collected on dry twigs of *Larix*, deserves further study. – **Sw**: GR. **It**: Lomb.

***Lecidea pseudoplana* Hertel ined.**

L # – Subs.: sil – Alt.: 3–4 – Note: a silicicoloous species resembling *L. paratropoides*, but with a different chemistry (planaic acid); in the study area so far only known from the Eastern Alps (Austria). – **Au**: Au.

***Lecidea ramulosa* Th. Fr.**

L – Subs.: cal, deb – Alt.: 5 – Note: a species of unclear relationships with a thick, whitish to bluish-grey thallus of ramified and unequally thick particles, and adnate, black apothecia which are at first plane and marginate, later convex and immarginate, based on a type from Northern Norway where it appears to be more common; on mosses and soil in alpine environments, rarely reported from the Alps. – **Sw**: GR.

***Lecidea rapax* Hertel**

L – Subs.: sil-par – Alt.: 4–6 – Note: a permanently lichenicolous species of the *L. atrobrunnea*-group, which invades thalli of the species *Bellemerea alpina* and *B. cinereorufescens*; closely related to *L. leprosolimbata*, and so far known only from the Alps. Roux et al. (2014) consider this species as a silicicolous ecotype of *L. leprosolimbata*. – **Au**: T, S, K, St. **Sw**: GR, TI, UR, VS. **Fr**: AHP, AMa. **It**: Frl, TAA, Piem, VA.

***Lecidea rhagadiella* (Nyl.) Th. Fr.**

Syn.: *Lecanora rhagadiella* Nyl.

L – Subs.: sil – Alt.: 4 – Note: a species of unclear relationships with an unusual combination of characters: thallus rather thick, rimose, whitish, with amyloid hyphae, urceolate to almost plane apothecia sunken in the thallus, a hyaline hypothecium, and rather large, broadly ellipsoid ascospores, based on a type from Northern Finland, with a few records from the Western Alps (France), on hard siliceous rocks at high elevations. – **Fr**: HAl, AMa.

***Lecidea rhododendri* (Hepp) Zahlbr.**

Syn.: *Biatora rhododendri* (Hepp) Arnold, Biatora sylvana Körb. var. rhododendri Hepp

L – Subs.: cor – Alt.: 3–5 – Note: on twigs of *Rhododendron* and other subalpine shrubs. The species does not belong to *Biatora* *s.str.* and is closely related to *L. erythrophaea*; it does not belong to *Lecidea* *s.str.* either. – **Au**: V, T, S, K, St, O, N. **Ge**: OB, Schw. **Sw**: GR, LU, UR. **It**: Frl, Ven, TAA. **Sl**: SlA.

***Lecidea sarcogynoides* Körb.**

Syn.: *Lecidea squamata* Flagey

L – Subs.: sil – Alt.: 1–3 – Note: a constantly cryptothalline species with a red-violet hymenium, found on exposed, steeply inclined faces of non-calcareous, mineral-rich rocks in lichen-poor communities of dry-warm sites in the lower altitudinal belts, records from high elevations being unlikely; widespread in the Alps. – **Au**: ?T, ?S, ?K, St, B. **Sw**: UR, VS. **Fr**: HSav, Vau. **It**: Ven, TAA, Lomb, Piem, VA, Lig.

***Lecidea sauteri* Körb.**

L – Subs.: sil, int – Alt.: 4–5 – Note: a species of the *L. auriculata*-group with a very thick thallus (with the confluentinic acid syndrome), and ascospores broader than in *L. auriculata*, found on steep faces of siliceous cliffs in the subalpine and alpine belts; in the study area so far known only from the Eastern Alps (Austria). – **Au**: S.

***Lecidea scabridisca* V. Wirth**

L – Subs.: sil – Alt.: 4(?-5) – Note: a species with a greyish-brown to brown, areolate thallus, a medulla reacting K+ red, black apothecia with umbonate to gyrose discs, and a brown hypothecium; on siliceous rocks, mostly in boulder screes of the subalpine belt; in the study area so far known only from the Eastern Alps (Austria). – **Au**: ?T, St.

***Lecidea siderolithica* Müll. Arg.**

Syn.: *Lecidea nigrogrisea* Nyl.

L # – Subs.: sil, met – Alt.: 2–3 – Note: a species of the *L. fuscoatra*-group developing small thin crustose thalli reacting C+ red (gyrophoric acid), with smaller ascospores than in *L. fuscoatra*; on siliceous rocks at mid-elevations; in the study area so far known only from the Western Alps (France). – **Fr**: HAl, HSav.

***Lecidea silacea* (Hoffm.) Ach.**

Syn.: *Lecidea subsilacea* Nyl., *Lecidella silacea* (Hoffm.) Körb., *Patellaria silacea* Hoffm., *Psora tabacina* Ramond *ex* DC. *non auct.*, *Toninia tabacina* (Ramond *ex* DC.) Flagey *non auct*.

L – Subs.: met, sil – Alt.: 3–6 – Note: a probably circumpolar, arctic-alpine to boreal-montane lichen found on iron-containing rocks in humid, sheltered situations, mostly in upland areas. – **Au**: V, T, S, K, St. **Sw**: BE, GR, TI, VS. **Fr**: HAl, AMa, Isè, Sav, HSav. **It**: Frl, Ven, TAA, Lomb, Piem, VA, Lig.

***Lecidea speirodes* Nyl.**

Syn.: Lecidea contigua (Hoffm.) Fr. var. subcretacea Arnold, *Lecidea decorosa* Arnold, *Lecidea subcretacea* (Arnold) P. Syd., *Lecidea subumbonata* *sensu* Arnold *et* Lettau *non* Nyl.

L – Subs.: int, sil – Alt.: 4–6 – Note: a lichen known from the central and southern European mountains (Alps, Pyrenees, Cordillera Cantabrica in Spain, Tatra) found on steeply inclined, superficially decalcified calciferous rocks or on lime-containing siliceous rocks. – **Au**: V, T. **Ge**: Schw. **Sw**: BE, GR, SZ. **Fr**: AHP, HAl, AMa, Sav, HSav, Vau. **It**: TAA, Piem, VA, Lig.

***Lecidea sphaerella* Hedl.**

L # – Subs.: xyl – Alt.: 3 – Note: a species of unclear relationship, with a very thin, greenish-white to greyish thallus, brownish to blackish-red (pale in the shade), hemispherical to subglobose apothecia, a reddish to brownish hypothecium (and therefore unlikely to represent a *Lecania*), with some intermixed 1-septate ascospores; on bark of various trees and on wood; overall distribution boreal to temperate-montane, with a few uncertain records from the Eastern Alps (Austria). – **Au**: ?St, ?N.

***Lecidea sphaerospora* Bagl. & Carestia**

L # – Subs.: sil – Alt.: 4–5 – Note: a species with a thick, grey-white, verrucose thallus reacting K-, sessile, black, often confluent apothecia with plane to finally convex and immarginate disc, a dark greenish epithecium, a colourless hypothecium, 8-spored asci, and subellipsoid to spherical ascospores measuring 7–9 µm in diam.; a long-forgotten taxon of uncertain affinities, only known from the type collection, which would deserve further study. – **It**: Piem.

***Lecidea spotornonis* B. de Lesd.**

L # – Subs.: sil – Alt.: 2 – Note: a silicicolous species reported only from the type collection above Spotorno. Thallus thin, blackish, bearing very small (0.1–0.2 mm in diam.), black, rounded apothecia with a concave disc and a thick proper margin; epithecium reddish, paraphyses free, simple; hymenium *c.* 45 µm tall, ascospores hyaline, simple, measuring 10–14 × 4–6 µm. – **It**: Lig.

***Lecidea spuriiformis* Anzi**

L # – Subst.: sil – Alt.: 4–5 – Note: a species with a well-developed, rimose-areolate, white thallus delimited by a black prothallus, small, black apothecia with a prominent proper margin, and elliptical, subacute ascospores measuring *c.* 18 × 8–10 µm; the type, which well deserves further study, was collected on mica-schists on Mt. Spluga, in Swiss territory. – **It**: TAA. **Sw**: GR.

***Lecidea steineri* Hertel**

L – Subs.: sil – Alt.: 4–5 – Note: a species of the *L. auriculata*-group recalling *L. promiscens*, but with an unpigmented to pale emerald hypothecium, and a cryptothalline thallus with a distinctly amyloid medulla, but lacking secondary compounds; on siliceous rocks, with optimum in the alpine belt; in the study area so far known with certainty only from the Western Alps (France). – **Au**: ?T. **Fr**: HAl, AMa.

***Lecidea stratura* K. Knudsen & Lendemer**

L – Subs.: sil – Alt.: 4 – Note: a recently-described silicicolous species of the *L. tessellata*-group based on a type from California, with a relatively thin, greyish thallus consisting of areoles with a rough surface, an amyloid medulla, apothecia often one per areole in a more or less central position, and a pigmented hypothecium; in the study area so far known only from the Eastern Alps (Austria). – **Au**: St.

***Lecidea subconfluens* Anzi**

L # – Subs.: sil – Alt.: 4–5 – Note: there is much confusion over the description of this species; to our knowledge, the first mention at species level provided with a description is in Anzi (Symbola Lichenum Rariorum etc. 1864: 18) which pre-dates that of another taxon with the same epithet described by Th. Fries made by H. Olivier (1881); the species, which differs from *L. confluens* in the thallus with pale areoles, the presence of a prothallus, and the faintly grey-pruinose apothecia, has thin paraphyses with a brownish tip and narrowly ellipsoid, obtuse ascospores with a thin episporium, measuring 12–15 × 5–7 µm. – **It**: Lomb, Piem.

***Lecidea subcongrua* Vain. *non* Nyl. *nom.illeg.***

Syn.: *Lecidella subcongrua* (“Vain.”) R. Sant. ined.

L # – Subs.: sil – Alt.: 4 – Note: an arctic to temperate-alpine species of unclear relationships, perhaps belonging to *Lecidella*, with a usually verrucose, whitish thallus reacting K+ yellow, black, glossy, plane apothecia of medium size, initially with a thin margin, later convex and immarginate, with a pale hypothecium and a bluish-green epihymenium, tightly coherent paraphyses, and medium-sized, ellipsoid ascospores; on hard siliceous rocks in wind-exposed situations, with several records from the Eastern Alps (Austria), but not distinguished elsewhere. – **Au**: ?V, S, St. **It**: TAA.

***Lecidea subfumosa* (Arnold) Zwackh**

Syn.: Psora atrobrunnea (DC.) A. Massal. var. subfumosa Arnold, *Psora subfumosa* (Arnold) Arnold

L # – Subs.: sil – Alt.: 5 – Note: an arctic-alpine, silicicolous species of the alpine belt, closely related to *L. atrobrunnea*. – **Au**: T. **It**: TAA.

***Lecidea subtrullissata* Müll. Arg.**

L # – Subs.: sil – Alt.: 3 – Note: a species of unclear relationships recalling a *Porpidia* in habitus, with a thin, whitish, rimose to areolate thallus, large sessile apothecia, the discs with a bluish-grey pruina when young, a dark hypothecium, a c. 70 µm high hymenium with sparsely branched paraphyses, and very small, ellipsoid ascospores; on iron-rich siliceous sandstone; in the study area so far only known from the Western Alps (France). – **Fr**: HSav.

***Lecidea sudetica* Körb.**

Syn.: *Lecidea alboflava* (Körb.) Arnold, *Lecidea virescens* Müll. Arg., *Lecidella alboflava* Körb.

L # – Subs.: sil – Alt.: 2–5 – Note: a silicicolous species reported from several localities in the Alps and in Central Europe, with optimum in upland areas; with a few scattered records from the Alps. – **Au**: St. **Sw**: BE, GR, VS. **It**: Lomb.

***Lecidea swartzioidea* Nyl.**

Syn.: *Lecidea arnoldiana* Dalla Torre & Sarnth., *Lecidea gneissacea* Zahlbr., *Lecidea jemtlandensis* H. Magn., Lecidea lapicida (Ach.) Ach. var. swartzioidea (Nyl.) Nyl., *Lecidea lithophiloides* Müll. Arg. *non* Nyl., *Lecidea metamorpha* Anzi, Lecidea swartzioidea Nyl. var. lithophiloides (Müll. Arg.) Clauzade & Cl. Roux, *Lecidea vogesiaca* Schaer.

L – Subs.: sil – Alt.: 3–6 – Note: a circumpolar, arctic-alpine to boreal-montane lichen of siliceous rocks, most common near and above treeline; closely related to *L. lapicida* and doubtfully worthy of being separated from it as a distinct species. – **Au**: V, T, S, K, St, N. **Sw**: GR, TI, VS. **Fr**: AHP, HAl, AMa, Isè, Sav, HSav. **It**: Frl, TAA, Lomb, Piem, Lig.

**Lecidea tessellata Flörke var. tessellata**

Syn.: *Lecidea cyanea* *sensu* Th. Fr., *Lecidea homalodes* Nyl., *Lecidea magna* Lynge, *Lecidea occidentalis* Lynge, *Lecidea spilota* Fr., *Lecidella spilota* (Fr.) Körb.

L – Subs.: sil, sil-par, cal – Alt.: 3–6 – Note: a cool-temperate to arctic-alpine, circumpolar species found on hard, often mineral-rich siliceous rocks in upland areas, which sometimes starts the life-cycle as a parasite of other crustose lichens, especially *Aspicilia*-species; widespread and common in the Alps. – **Au**: V, T, S, K, St, N. **Sw**: BE, GR, LU, SZ, UR, VD, VS. **Fr**: AHP, HAl, AMa, Sav, HSav. **It**: Frl, Ven, TAA, Lomb, Piem, VA, Lig.

**Lecidea tessellata Flörke var. caesia (Anzi) Arnold**

Syn.: *Biatora casimirii* Müll. Arg., *Lecidea azurea* Kremp., *Lecidea casimirii* (Müll. Arg.) Müll. Arg., *Lecidea injuncta* Nyl., Lecidea spilota Fr. var. caesia Anzi, *Lecidella azurea* (Kremp.) Körb.

L – Subs.: sil, sil-par, int-par – Alt.: 4–6 – Note: on calciferous siliceous rocks, dolomite, and superficially decalcified, hard limestones near and above treeline, starting the life-cycle on other crustose lichens. – **Au**: V, T, S, K, St, N. **Ge**: OB, Schw. **Sw**: BE, GR, VD, VS. **Fr**: AHP, HAl, AMa, Sav, HSav. **It**: Frl, TAA, Lomb, Piem, Lig.

***Lecidea titubans* Bagl. & Carestia**

L # – Subs.: sil – Alt.: 4–5 – Note: a species of uncertain affinity, with a whitish, areolate thallus developing on a conspicuous black hypothallus, plane, black, often deformed and confluent apothecia with a plane disc and a thin proper margin, a greenish-brown epithecium, a thin hymenium of coherent paraphyses, a colourless hypothecium, 8-spored asci, and elliptical, hyaline, simple ascospores which are *c.* 2 times as long as wide; only known from the type collection, on schists, which deserves further study. – **It**: Piem.

***Lecidea turgidula*Fr.**

Syn.: *Biatora turgidula* (Fr.) Nyl., *Lecidella turgidula* (Fr.) Körb., *Oedemocarpus turgidulus* (Fr.) Trevis.

L – Subs.: cor, xyl – Alt.: 2–5 – Note: on hard lignum, more rarely on bark of conifers; the systematic position of this species is not clear: it certainly does not belong to *Lecidea* *s.str.* – **Au**: V, T, S, K, St, O, N. **Ge**: OB. **Sw**: GR, LU, SZ, VD, VS. **Fr**: Isè, HSav, Var, Vau. **It**: Ven, TAA, Lomb, Piem. **Sl**: SlA.

***Lecidea umbonata* (Hepp) Mudd**

Syn.: *Biatora umbonata* Hepp, *Lecidea acosmeta* Lettau, *Lecidea exornans* (Arnold) Nyl., *Lecidea omphaliza* Lettau, *Lecidea subumbonata* Nyl. *non* *sensu* Arnold, *Lecidea umboniza* Nádv., *Lecidella exornans* (Arnold) Arnold, *Lecidella umbonata* (Hepp) Körb.

L – Subs.: int – Alt.: 3–6 – Note: a circumpolar, mainly arctic-alpine, variable species found on calciferous siliceous rocks, especially schist, in cool and humid situations; widespread throughout the Alps. – **Au**: V, T, S, K, St, N. **Ge**: Schw. **Sw**: BE, GR, LU, SZ, TI, UR, UW, VD, VS. **Fr**: AHP, HAl, AMa, Isè, Sav, HSav. **It**: Frl, Ven, TAA, Piem, Lig.

***Lecidea variegatula* Nyl.**

L – Subs.: sil, met – Alt.: 2–3 – Note: a species developing small thalli consisting of minute, glossy, yellowish-brown areoles with a non-amyloid medulla lacking lichen compounds, non-pruinose apothecia with a thin margin, hymenium less than 40 µm high, and minute ascospores; on low siliceous outcrops and pebbles at low elevations; widespread in Europe but rarely collected, with a single record from the Eastern Alps (Austria). – **Au**: St.

***Lecidea verruca* Poelt**

L – Subs.: sil-par – Alt.: 4–5 – Note: an arctic-alpine, bipolar silicicolous species of the *L. tessellata*-group with a peculiar ecology, always growing on *Aspicilia*-species near or above treeline. – **Au**: T, S, K, St, N. **Sw**: TI, UR. **It**: TAA, Piem.

***Lecidea vicinalis* Müll. Arg.**

L # – Subs.: sax – Alt.: 4 – Note: a species resembling in habitus a small-fruiting form of *L. paratropoides*, with a kryptothalline thallus, black, sessile, distinctly marginate apothecia in irregular dense groups, a red-brown hypothecium, 8-spored asci, and minute, ellipsoid ascospores (6–8 × 4–6 μm); on siliceous rocks (*e.g.* granite) at high elevations; only known from the type locality in the Western Alps (Switzerland). – **Sw**: VS.

***Lecidella albida* Hafellner**

Syn.: *Lecidea alba* Schleich. *ex* Schaer. *non* (Ach.) Flörke, *Lecidella alba* (Schleich.) Hertel *nom.illeg*.

L – Subs.: cor – Alt.: 2–3 – Note: a mainly Central European species growing on the smooth bark of more or less isolated individuals of *Fagus*, *Fraxinus* and *Acer*, more rarely of conifers, in non-eutrophicated, rather humid situations; from the Alps there are a few scattered records. – **Au**: St. **Sw**: SZ, UW. **Fr**: HSav. **It**: TAA. **Sl**: SlA.

***Lecidella anomaloides* (A. Massal.) Hertel & H. Kilias**

Syn.: *Biatora pungens* Körb., *Biatorina anomaloides* (A. Massal.) Jatta, *Catillaria anomaloides* (A. Massal.) Lettau, *Lecidea anomaloides* A. Massal., Lecidea elaeochroma (Ach.) Ach. var. pungens (Körb.) Th. Fr., *Lecidea goniophila auct. non* Flörke, *Lecidea pilularis* (Ach.) Fr., *Lecidea pungens* (Körb.) Nyl., *Lecidella goniophila auct.*, *Lecidella pilularis* (Ach.) Stein, *Lecidella pungens* (Körb.) Körb.

L – Subs.: sil, int – Alt.: 2–6 – Note: on steeply inclined to slightly underhanging, hard, base-rich or weakly calciferous siliceous rocks, with a wide altitudinal range; widespread throughout the Alps. – **Au**: V, T, S, K, St, N. **Ge**: Schw. **Sw**: BE, GR, LU, SG, UR, UW, VD, VS. **Fr**: HAl, AMa, Isè, Sav, HSav, Var. **It**: Ven, TAA, Lomb, Piem, VA. **Sl**: SlA.

**Lecidella asema (Nyl.) Knoph & Hertel var. asema**

Syn.: *Lecidea asema* Nyl., *Lecidea distrata* Arnold *non* Nyl., *Lecidea distratula* Zahlbr., *Lecidea latypea auct. p.p. non* Ach., *Lecidea polyantha* Taylor *ex* Leight., *Lecidea subincongrua* Nyl., *Lecidella subincongrua* (Nyl.) Hertel & Leuckert

L – Subs.: sil, int – Alt.: 1–4 – Note: a widespread, chemically variable species of basic siliceous rocks, on faces wetted by rain, with a wide altitudinal range, but rarely occurring above treeline. – **Au**: V, T, S, K, St, N, B. **Sw**: BE, GR, TI, VS. **Fr**: HAl, AMa, Var. **It**: Frl, TAA, Lomb, Piem, VA.

**Lecidella asema (Nyl.) Knoph & Hertel var. elaeochromoides (Nyl.) Nimis & Tretiach**

Syn.: *Lecidea catalinaria* Stizenb., *Lecidea elaeochromoides* (Nyl.) Flagey, Lecidea parasema (Ach.) Ach. var. elaeochromoides Nyl., Lecidea subincongrua Nyl. var. elaeochromoides (Nyl.) Poelt, *Lecidella elaeochromoides* (Nyl.) Knoph & Hertel, Lecidella subincongrua (Nyl.) Hertel & Leuckert var. elaeochromoides (Nyl.) Hertel & Leuckert

L # – Subs.: sil – Alt.: 1–5 – Note: the chemistry of *L. asema* is quite complex; this variety with a yellow thallus is common in the Mediterranean region, and so easily recognizable, that we still prefer to distinguish it from *L. asema* *s.str.*, at least at varietal level; in the Alps it is most frequent in the dry-warm valleys of the Alps. – **Au**: T, S, St. **Ge**: Schw. **Fr**: Var, Vau. **It**: TAA, Piem.

***Lecidella carpathica* Körb.**

Syn.: *Blastenia rejecta* Th. Fr., *Lecidea baskalensis* Szatala, *Lecidea carpathica* (Körb.) Szatala, *Lecidea continuior* Nyl., *Lecidea diffractula* H. Magn., *Lecidea durietzii* H. Magn., *Lecidea fennica* Räsänen, *Lecidea kotiluotensis* Vain, *Lecidea latypea auct. p.p. non* Ach., *Lecidea latypiza auct. non* Nyl., *Lecidea latypizella* Nádv., *Lecidea loudiana* Zahlbr., *Lecidea pertingens* Nyl., *Lecidea subsmaragdula* H. Magn., *Lecidea suprasedens* Zahlbr., Lecidella carpathica Körb. var. latypizella (Nádv.) Hertel, *Nesolechia vainioana* Räsänen

L – Subs.: sil, int, cal – Alt.: 2–6 – Note: a widespread holarctic lichen with a broad altitudinal and latitudinal range, found on base-rich rocks wetted by rain in exposed situations, often starting the life-cycle on other crustose lichens; widespread and common throughout the Alps. – **Au**: V, T, S, K, St, O, N, B. **Ge**: OB, Schw. **Sw**: BE, GR, SZ, TI, UR, VS. **Fr**: AHP, HAl, AMa, Isè, Sav, HSav, Var, Vau. **It**: Frl, Ven, TAA, Lomb, Piem, VA, Lig. **Sl**: SlA.

***Lecidella effugiens* (Nilson) Knoph & Hertel**

Syn.: *Lecidea effugiens* Nilson, *Lecidea incongruella* Vain., *Lecidella albidocinerella* (Vain.) Poelt & Vězda, *Lecidella incongruella* (Vain.) Hertel & Leuckert

L – Subs.: sil – Alt.: 3–6 – Note: a member of the *L. asema*-complex; on more or less calcareous or base-rich siliceous rocks, with optimum near or above treeline; probably more widespread in the Alps. – **Au**: T, S, K, St. **It**: TAA.

**Lecidella elaeochroma (Ach.) M. Choisy f. elaeochroma**

Syn.: *Biatora ambigua* A. Massal., *Biatora tabescens* Körb., *Lecidea achristotera* Nyl., *Lecidea elaeochroma* (Ach.) Ach., *Lecidea flavens* (Nyl.) Nyl., *Lecidea limitata auct. non* Scop., *Lecidea olivacea* (Hoffm.) A. Massal., Lecidea parasema (Ach.) Ach. var. elaeochroma Ach., Lecidea parasema (Ach.) Ach. var. flavens Nyl., Lecidea parasema (Ach.) Ach. var. olivacea (Hoffm.) Mong., Lecidea parasema (Ach.) Ach. var. rugulosa Ach., *Lecidella achristotera* (Nyl.) Hertel & Leuckert, *Lecidella ambigua* (A. Massal.) Körb., Lecidella elaeochroma (Ach.) M. Choisy var. flavicans (Ach.) Hertel, *Lecidella olivacea* (Hoffm.) Hazsl.

L – Subs.: cor, xyl – Alt.: 1–5 – Note: this is one of the commonest epiphytic lichens of the Alps below the subalpine belt, with an extraordinarily wide ecological and altitudinal range. The value of *L. achristotera* (here treated as a synonym) is questioned by some authors, because in *Lecidella* the presence of a hymenial inspersion might not have the same importance as in other genera. Some records could refer to *L. euphorea*. – **Au**: V, T, S, K, St, O, N, B. **Ge**: OB, Schw. **Sw**: BE, FR, GL, GR, LU, SG, SZ, TI, UR, UW, VD, VS. **Fr**: AHP, HAl, AMa, Drô, Isè, Sav, HSav, Var, Vau. **It**: Frl, Ven, TAA, Lomb, Piem, VA, Lig. **Sl**: SlA, Tg. **Li**.

**Lecidella elaeochroma (Ach.) M. Choisy f. soralifera (Erichsen) D. Hawksw.**

Syn.: Lecidea limitata (Scop.) Gray var. soralifera (Erichsen) J.R. Laundon, Lecidea olivacea (Hoffm.) A. Massal. var. soralifera (Erichsen) Erichsen, Lecidella elaeochroma (Ach.) M. Choisy var. soralifera (Erichsen) Hertel

L # – Subs.: cor – Alt.: 2–3 – Note: this sorediate-fruiting lichen, in our opinion, is just an occasionally sorediate form of *L. elaeochroma.* It is more widespread than the few records would suggest, but never common, generally occurring together with fruiting specimens in humid-warm areas. The “occasional” appearance of asexually reproducing forms along south-to-north gradients, however, well deserves the attention of lichenologists. – **Au**: T, S, K, St, O, N. **Sw**: BE, GR. **Fr**: AHP, AMa. **It**: Ven, TAA, Lig.

***Lecidella euphorea* (Flörke) Hertel**

Syn.: *Biatorina dolosa* (Ach.) A.L. Sm., *Lecidea achrista* (Sommerf.) Britzelm., *Lecidea dolosa* Ach., *Lecidea euphorea* (Flörke) Nyl., *Lecidea glomerulosa* (DC.) Steud., Lecidea parasema (Ach.) Ach. var. euphorea (Flörke) Arnold, Lecidea sabuletorum Flörke var. euphorea Flörke, *Lecidella dolosa* (Ach.) Stein, *Lecidella glomerulosa* (DC.) M. Choisy

L # – Subs.: cor, xyl – Alt.: 2–4 – Note: the value of this taxon is questioned by some authors, who treat it as chemical strain (lacking xanthones) of *L. elaeochroma*; most frequent on bark of deciduous trees in montane to subalpine forests, also on low shrubs (*e.g. Rhododendron*) in the treeline ecotone. – **Au**: V, T, S, K, St, O, N, B. **Ge**: OB, Schw. **Fr**: AHP, HAl, AMa, Drô, Isè, HSav, Var, Vau. **It**: Frl, Ven, TAA, Lomb, Piem, VA, Lig.

***Lecidella flavosorediata* (Vězda) Hertel & Leuckert**

Syn.: *Lecidea flavosorediata* Vězda, Lecidella elaeochroma (Ach.) M. Choisy var. flavosorediata (Vězda) Clauzade & Cl. Roux

L – Subs.: cor – Alt.: 2–4 – Note: this epiphytic species, which deserves further study, seems to be most frequent in the mountains of Southern Europe, including the Alps. – **Au**: S, K, St, O, N. **Ge**: OB, Schw. **Sw**: BE, GL, GR, LU, SZ, TI, UW, VS. **Fr**: AHP, Drô, Isè, Vau. **It**: Ven, TAA, Lig. **Sl**: SlA, Tg.

***Lecidella granulosula* (Nyl.) Knoph & Leuckert**

Syn.: *Lecidea chodatii* Samp., *Lecidea goniophiloides* B. de Lesd., *Lecidea granulosula* Nyl., *Lecidella chodatii* (Samp.) Knoph & Leuckert, Lecidella viridans (Flot.) Körb. var. chodatii (Samp.) Hertel & Leuckert

L – Subs.: sil – Alt.: 2–3 – Note: on basic siliceous rocks; probably restricted to dry-warm valleys of the Alps. – **It**: TAA, Lomb.

***Lecidella laureri* (Hepp) Körb.**

Syn.: *Biatora laureri* Hepp, Lecidea euphorea (Flörke) Nyl. var. laureri (Hepp) Vain., *Lecidea laureri* (Hepp) Anzi

L – Subs.: cor, xyl – Alt.: 2–4 – Note: on eutrophicated lignum and base-rich bark. – **Au**: V, T, S, K, St, O, N. **Sw**: GR, SZ, UR, VS. **It**: Ven, TAA, Lomb, Piem. **Sl**: SlA, Tg.

***Lecidella patavina* (A. Massal.) Knoph & Leuckert**

Syn.: *Buellia sordida* (A. Massal.) Jatta, *Catillaria sordida* A. Massal., *Lecidea acrocyanea* (Th. Fr.) H. Magn., *Lecidea alaiensis* Vain., *Lecidea araratica* Müll. Arg., *Lecidea endolithea* Lynge, *Lecidea epipolioides* (J. Steiner) Szatala, *Lecidea inamoena* Müll. Arg., *Lecidea patavina* A. Massal., *Lecidea piemontensis* B. de Lesd., *Lecidea portensis* Nádv., *Lecidea rolleana* H. Magn., Lecidea rolleana H. Magn. var. portensis (Nádv.) Hertel, *Lecidea spitsbergensis* Lynge, *Lecidella alaiensis* (Vain.) Hertel, Lecidella alaiensis (Vain.) Hertel var. spitzbergensis (Lynge) Clauzade & Cl. Roux, *Lecidella endolithea* (Lynge) Hertel & Leuckert, *Lecidella inamoena* (Müll. Arg.) Hertel, *Lecidella spitsbergensis* (Lynge) Hertel & Leuckert

L – Subs.: cal – Alt.: 2–6 – Note: a circumpolar, cool-temperate to arctic-alpine, nitrophilous lichen, one of the most common calcicolous lichens of upland areas throughout the Alps; related to *L. stigmatea*. – **Au**: V, T, S, K, St, O, N. **Ge**: OB, Schw. **Sw**: BE, GL, GR, LU, SZ, TI, UR, VS. **Fr**: AHP, HAl, AMa, Drô, Isè, Sav, HSav, Var, Vau. **It**: Frl, Ven, TAA, Lomb, Piem, VA, Lig.

***Lecidella pulveracea* (Schaer.) P. Syd.**

Syn.: *Biatora pulveracea* (Schaer.) Stein, Lecidea enteroleuca Ach. var. pulveracea Schaer., *Lecidea pulveracea* (Schaer.) Th. Fr.

L – Subs.: xyl, cor – Alt.: 2–3 – Note: a mainly temperate lichen found especially on *Fraxinus*, sometimes on nutrient-enriched lignum. – **Au**: V, T, S, K, St. **Ge**: OB. **Sw**: BE, FR, LU, SZ, UW, VD. **Fr**: AHP, HAl, Isè, HSav, Var, Vau. **It**: Frl, Ven, TAA. **Sl**: SlA.

***Lecidella scabra* (Taylor) Hertel & Leuckert**

Syn.: *Lecidea alienata* Nyl., *Lecidea enterochlora* Taylor, Lecidea parasema (Ach.) Ach. var. prasinula Wedd., *Lecidea prasinula* (Wedd.) B. de Lesd., *Lecidea protrusa* Fr., *Lecidea scabra* Taylor, *Lecidella dirumpens* (Hertel & Poelt) Hertel & Poelt, *Lecidella prasinula* (Wedd.) Hertel, *Lithographa larbalestieri* Leight.

L – Subs.: sil – Alt.: 1–4 – Note: a mainly temperate to Mediterranean lichen found on basic siliceous substrata wetted by rain in species-poor stands; the species is chemically heterogeneous. – **Au**: V, T, S, St, N. **Sw**: GL, VS. **Fr**: Var. **It**: TAA, Lomb, Piem, Lig.

***Lecidella stigmatea* (Ach.) Hertel & Leuckert**

Syn.: *Bacidia arthoniza* (Nyl.) Zahlbr., *Bacidia biseptata* H. Magn., *Bacidia ostrogothica* Malme, *Biatora arctoides* Hellb., *Lecidea arthoniza* Nyl., *Lecidea caesiocinerea* H. Magn., *Lecidea cinnamomea* Flörke *ex* Hellb., *Lecidea diasemoides* Nyl., *Lecidea enteroleuca auct. p.p.*, *Lecidea glabra* (Kremp.) Hellb., *Lecidea imitatrix* Zahlbr., *Lecidea incongrua* (Nyl.) Nyl., *Lecidea micacea* (Körb.) H. Olivier, *Lecidea parasema auct.* subsp. incongrua Nyl., *Lecidea prominula* Borrer, *Lecidea restricta* Stirt., *Lecidea stigmatea* Ach., *Lecidea subsequens* Nyl., *Lecidea vulgata* Zahlbr., *Lecidella aequata* (Flörke) Kremp., *Lecidella glabra* Kremp., *Lecidella incongrua* (Nyl.) Arnold, *Lecidella micacea* Körb., *Lecidella vulgata* (Zahlbr.) M. Choisy; incl. Lecidella stigmatea (Ach.) Hertel & Leuckert f. egena (Kremp.) Clauzade & Cl. Roux

L – Subs.: cal, sil, int – Alt.: 1–6 – Note: a variable and ecologically wide-ranging lichen, often found in disturbed habitats, especially on sandstone walls, sometimes starting the life-cycle on other crustose lichens; widespread throughout the siliceous Alps. – **Au**: V, T, S, K, St, O, N, B. **Ge**: OB, Schw. **Sw**: BE, GR, LU, SZ, TI, UR, VD, VS. **Fr**: AHP, HAl, AMa, Drô, Isè, Sav, HSav, Var, Vau. **It**: Frl, Ven, TAA, Lomb, Piem, VA, Lig. **Sl**: SlA, Tg.

***Lecidella subviridis* Tønsberg**

L – Subs.: cor – Alt.: 2–3 – Note: a species with a sorediate to leprose, grey-green thallus with a peculiar secondary chemistry (atranorin, thiophanic acid, arthothelin), apothecia rare; on the bark of trees and dwarf shrubs in forests under oceanic influence, and at low elevations; the distribution is insufficiently known. – **Au**: S, O. **Sl**: SlA, Tg.

***Lecidella umbrosa* (Bagl. *ex* A. Massal.) Hertel**

Syn.: *Biatora umbrosa* Bagl. *ex* A. Massal., *Lecidea umbrosa* (Bagl. *ex* A. Massal.) Müll. Arg.

L # – Subs.: sax – Alt.: 1–3 – Note: a species of base-rich siliceous rocks, related to *L. anomaloides*, which needs further study. – **It**: TAA.

***Lecidella viridans* (Flot.) Körb.**

Syn.: *Biatora viridans* (Flot.) Hepp, *Lecidea elaeochromiza* (Nyl.) H. Olivier, Lecidea sabuletorum (Schreb.) Ach. var. viridans Flot., *Lecidea viridans* (Flot.) Lamy, *Lecidella elaeochromiza* (Nyl.) M. Choisy

L – Subs.: sil, int – Alt.: 1–3 – Note: on base-rich or slightly calciferous siliceous rocks, especially on steeply inclined faces, in dry-warm areas. – **Au**: T, K, St. **Ge**: Schw. **Sw**: ?GR. **Fr**: AMa, Sav, HSav. **It**: Frl, Ven, TAA, Lomb, Piem.

***Lecidella wulfenii* (Hepp) Körb.**

Syn.: *Biatora wulfenii* Hepp, Lecidea elaeochroma (Ach.) Ach. var. muscorum Th. Fr., Lecidea glomerulosa (DC.) Steud. var. muscorum (Th. Fr.) Vain., *Lecidea heppii* R.A. Anderson & W.A. Weber, *Lecidea muscorum* (Th. Fr.) Dalla Torre & Sarnth., *Lecidea wulfeniana* Grummann, *Lecidea wulfenii* (Hepp) Arnold *non* Ach., *Lecidella heppii* R.A. Anderson

L – Subs.: deb, bry – Alt.: 3–5 – Note: a circumpolar, arctic-alpine lichen found on moribund bryophytes and plant remains in exposed habitats near and above treeline. – **Au**: V, T, S, K, St, O, N. **Ge**: OB, Schw. **Sw**: BE, GR, SZ, TI, UR, VD, VS. **Fr**: AHP, HAl, AMa, Isè, Sav, HSav. **It**: Frl, Ven, TAA, Lomb, Piem, VA, Lig. **Sl**: SlA.

***Lecidella xylophila* (Th. Fr.) Knoph & Leuckert**

Syn.: *Lecidea xylophila* Th. Fr.

L # – Subs.: xyl – Alt.: 4 – Note: a species growing on lignum near treeline, which needs further study. – **Sw**: GR. **It**: TAA.

***Lecidoma demissum* (Rutstr.) Gotth. Schneid. & Hertel**

Syn.: *Biatora atrorufa* (Dicks.) Fr., *Biatora demissa* (Rutstr.) Fr., *Lecidea atrorufa* (Dicks.) Ach., *Lecidea demissa* (Rutstr.) Ach., *Lepidoma demissum* (Rutstr.) M. Choisy, *Lichen demissus* Rutstr., *Psora atrorufa* (Dicks.) Hook., *Psora demissa* (Rutstr.) Stein

L – Subs.: ter-sil, ter-cal – Alt.: 4–6 – Note: a circumpolar, arctic-alpine lichen found on soil, rarely on siliceous rocks, in clearings of alpine grasslands with a long snow cover. – **Au**: V, T, S, K, St, O, N. **Ge**: OB, Schw. **Sw**: BE, GR, LU, SG, SZ, TI, UR, UW, VD, VS. **Fr**: AHP, HAl, AMa, Isè, Sav, HSav. **It**: Frl, Ven, TAA, Lomb, Piem, VA. **Sl**: SlA.

***Leimonis erratica* (Körb.) R.C. Harris & Lendemer**

Syn.: *Lecidea demarginata* Nyl., *Lecidea dispansa* Nyl., *Lecidea erratica* Körb., *Lecidea expansa* Nyl., *Lecidea tephrizans* Leight., *Micarea erratica* (Körb.) Hertel, Rambold & Pietschm.

L – Subs.: sil – Alt.: 2–3, ?5 – Note: a species with an endolithic to verrucose, usually rimose thallus and black, sessile, lecideoid apothecia with a persistent thin margin and a dark hypothecium, asci of *Byssoloma*-type, and small, narrowly ellipsoid ascospores; on siliceous pebbles in open habitats (ecologically similar to *Porpidia crustulata*); widespread in the Holarctic region but also recorded from the Southern Hemisphere, with a few records from the Eastern Alps (Austria), but distribution in the Alps likely to be incompletely documented. – **Au**: ?V, T, K, St.

***Lemmopsis arnoldiana* (Hepp) Zahlbr.**

Syn.: *Physma arnoldianum* Hepp, *Psorotichia arnoldiana* (Hepp) Körb., *Pyrenocarpus arnoldianus* (Hepp) Trevis.

L – Subs.: cal – Alt.: 1–3 – Note: on calcareous pebbles in shaded woodland floors and crevices in rocky querries, sometimes on mortar; perhaps overlooked in the Alps, but certainly not common. – **Au**: St, N. **Ge**: OB. **Sw**: ?LU. **Fr**: Sav, HSav. **It**: Lig. **Sl**: SlA.

***Lempholemma botryosum* (A. Massal.) Zahlbr.**

Syn.: *Arnoldia botryosa* A. Massal., *Omphalaria botryosa* (A. Massal.) Nyl., *Physma botryosum* (A. Massal.) Zahlbr., *Plectopsora botryosa* (A. Massal.) A. Massal.

L – Subs.: cal – Alt.: 1–4 – Note: on steeply inclined surfaces of hard calciferous rocks with some water seepage after rain, often in sites with cyanobacterial colonies. – **Au**: V, T, S, St, O, N. **Ge**: OB. **Sw**: BE, SZ. **Fr**: HSav. **It**: Frl, TAA, Lomb. **Sl**: SlA.

***Lempholemma chalazanum* (Ach.) B. de Lesd.**

Syn.: *Collema chalazanum* Ach., *Lempholemma franconicum* (A. Massal.) Zahlbr., *Physma chalazanum* (Ach.) Arnold, *Physma franconicum* A. Massal.

L – Subs.: cal, ter-cal, bry-cal – Alt.: 1–3 – Note: a mainly temperate lichen found on soil in open dry grasslands, sometimes overgrowing bryophytes and plant debris, but also on walls; probably overlooked and perhaps more widespread in the Alps. – **Au**: V, T, K, St, N. **Ge**: OB, Schw. **Sw**: GR, VD, VS. **Fr**: AMa, Sav, Var. **It**: Ven, Piem.

***Lempholemma cladodes* (Tuck.) Zahlbr.**

Syn.: *Collema cladodes* Tuck.

L – Subs.: cal – Alt.: 4 – Note: a species with dwarf, fruticose, black thalli forming cushions, the thallus branches with apical swellings forming hormocystangia, or with terminal globose pycnoascocarpia; on calcareous rocks in cool situations; widespread in the Northern Hemisphere (Scandinavia, North America), but not common; rarely collected in the Alps, being only known from Switzerland. – **Sw**: SZ.

***Lempholemma condensatum* (Arnold) Zahlbr.**

Syn.: Plectopsora botryosa (A. Massal.) A. Massal. var. condensata Arnold

L # – Subs.: cal – Alt.: 2–4 – Note: a species close to or perhaps conspecific with *L. intricatum*, with thalli forming dense black cushions; on boulders and cliffs of dolomite and calcareous rocks, ecology otherwise poorly known; reported from scattered localities in the Eastern Alps, but distribution insufficiently documented. – **Au**: T, O, N. **Ge**: OB, Schw. **Sw**: SZ. **It**: TAA.

***Lempholemma elveloideum* (Ach.) Zahlbr.**

Syn.: *Arnoldia cyathodes* A. Massal., *Collema cyathodes* (A. Massal.) Nyl., *Collema elveloideum* Ach., *Omphalaria helveloidea* (Ach.) A. Massal. *nom.illeg., Physma cyathodes* (A. Massal.) Jatta, *Plectopsora cyathodes* (A. Massal.) A. Massal., *Plectopsora elveloidea* (Ach.) Zanfr.

L – Subs.: cal – Alt.: 1–3 – Note: a mainly temperate lichen found on steeply inclined faces of calcareous rocks, in seepage tracks, often with other cyanobacterial lichens. – **Au**: S. **Sw**: VS. **Fr**: Var. **It**: Ven, Lomb, Piem.

***Lempholemma intricatum* (Arnold) Zahlbr.**

Syn.: *Leciophysma fennicum* Räsänen, *Lempholemma fennicum* (Räsänen) Degel., *Omphalaria intricata* Arnold, *Synalissa intricata* (Arnold) Nyl.

L – Subs.: sil, cal – Alt.: 2–5 – Note: on steeply inclined calcareous or basic siliceous rocks in seepage tracks, mostly in humid areas; perhaps more widespread in the Alps. – **Au**: V, S, K, St, O, N. **Ge**: OB. **Sw**: SZ, VS. **It**: Frl.

***Lempholemma isidioides* (Nyl. *ex* Arnold) H. Magn.**

Syn.: *Collema isidiodes* Nyl. *ex* Arnold

L – Subs.: sil – Alt.: 2–3 – Note: a species with a squamulose thallus forming small rosettes, with cylindrical, isidioid lobes in the centre; on irrigated faces of various types of rocks; widespread in Northern Europe, further south most frequent in the mountains; known from scattered localities throughout the Alps. – **Ge**: OB. **Sw**: SZ, VS. **Fr**: AHP, AMa.

***Lempholemma muelleri* (Hepp *ex* Müll. Arg.) Zahlbr.**

Syn.: *Physma muelleri* Hepp *ex* Müll. Arg.

L # – Subs.: cal, sil – Alt.: kol-mon – Note: in the protologue the name is given as “*P. muelleri* Hepp *in litt.*”. As there is no indication that the diagnosis has been provided by Hepp too, the name has to be attributed to the author of the publication (ICN Art. 46.5). A species with a habitus somewhat intermediate between those of *Lathagrium cristatum* and *Scytinium lichenoides*: black lobes with lobulate margins, richly fertile along the margins and on the tips, with punctiform and urceolate apothecia; on boulders along streams and rivers (at the type locality together with *Lichinella heppii*). – **Fr**: HSav.

***Lempholemma polyanthes* (Bernh.) Malme**

Syn.: *Collema chalazanellum* Nyl., *Collema myriococcum* (Ach.) Ach., *Lempholemma chalazanellum* (Nyl.) Zahlbr., *Lempholemma chalazanodes* (Nyl.) Zahlbr., *Lempholemma compactum* (Wallr.) Körb., *Lempholemma fasciculare* (Wulfen) Zahlbr., *Lempholemma myriococcum* (Ach.) Th. Fr., *Lichen polyanthes* Bernh., *Physma chalazanellum* (Nyl.) Erichsen, *Physma chalazanodes* (Nyl.) Arnold, *Physma compactum* (Wallr.) A. Massal., *Physma myriococcum* (Ach.) Körb., *Physma polyanthes* (Bernh.) Arnold

L – Subs.: ter-cal, bry, cal – Alt.: 2–5 – Note: a cool-temperate to arctic-alpine, circumpolar lichen found on terricolous or epilithic bryophytes, over soil or on plant debris, sometimes on walls; much overlooked or confused with *Collema*-species, and probably more widespread in the Alps. – **Au**: T, S, K, St, O, N. **Ge**: OB. **Sw**: GR, VS. **Fr**: AHP, AMa, HSav, Var. **It**: Frl, Ven, TAA, Lomb, Piem. **Sl**: SlA.

***Lempholemma radiatum* (Sommerf.) Henssen**

Syn.: *Thyrea radiata* (Sommerf.) Zahlbr.

L – Subs.: sil, cal – Alt.: 4–5 – Note: a species with a thick thallus forming rosettes, recalling a *Collema*, with strap-like lobes provided with clusters of mostly globose isidia; on long-time moist, mostly calcareous rocks, often amongst bryophytes; known from cool to cold parts of the Holarctic region, with a few records from the Eastern Alps (Austria). – **Au**: T, S, O.

**Lepra albescens (Huds.) Hafellner var. albescens**

Syn.: *Lichen albescens* Huds., *Marfloraea albescens* (Huds.) S.Y. Kondr., Lőkös & Hur, Pertusaria albescens (Huds.) M. Choisy & Werner var. albescens, Pertusaria communis DC. var. discoidea (Pers.) Garov., Pertusaria communis DC. var. variolosa (Flot.) Schaer., *Pertusaria deschatresii* Werner, *Pertusaria discoidea* (Pers.) Malme, *Pertusaria globulifera* (Turner) A. Massal., *Pertusaria leprarioides* Erichsen *non auct.*, *Pertusaria orbiculata* (Schreb.) Zahlbr., *Pertusaria scutellata* Hue, *Variolaria discoidea* Pers.

L – Subs.: cor, bry, sil – Alt.: 1–4 – Note: a mainly temperate epiphytic lichen, most common in deciduous open woodlands of the submediterranean and montane belts; widespread throughout the Alps. – **Au**: V, T, S, K, St, O, N, B. **Ge**: OB, Schw. **Sw**: AP, BE, FR, GL, GR, LU, SG, SZ, TI, UR, UW, VD, VS. **Fr**: AHP, HAl, AMa, Drô, Isè, Sav, HSav, Var, Vau. **It**: Frl, Ven, TAA, Lomb, Piem, VA, Lig. **Sl**: SlA, Tg. **Li**.

***Lepra albescens* var. *corallina sensu auct.* (provisionally placed here, ICN Art. 36.1b)**

Syn.: *Pertusaria albescens* (Huds.) M. Choisy & Werner var. *corallina auct. non* (Zahlbr.) J.R. Laundon, *Pertusaria henrici* *sensu* Erichsen

L # – Subs.: cor – Alt.: 2–3 – Note: a strain with the secondary chemistry of *L. albescens* but with a different morphology, the spreading thallus being widely covered by isidioid structures, usually lacking conspicuous orbicular soralia; the nomenclature is unresolved; here and there in Europe including the Alps but older records need chemical reinvestigation. – **Au**: V, T, K, St, O, N. **Ge**: OB. **Fr**: AHP, AMa, HSav, Var, Vau. **Sl**: SlA.

***Lepra amara* (Ach.) Hafellner**

Syn.: *Marfloraea amara* (Ach.) S.Y. Kondr., Lőkös & Hur, *Pertusaria amara* (Ach.) Nyl., *Pertusaria faginea auct.*, *Pertusaria pulvinata* Erichsen, *Pertusaria slesvicensis* Erichsen, *Variolaria amara* Ach.

L – Subs.: cor, xyl, sil – Alt.: 1–5 – Note: a widespread holarctic lichen, certainly the most common epiphytic species of the genus throughout the Alps, with a wide ecological range; it often behaves as an aggressive competitor, being able to overgrow other crustose lichens and sometimes even bryophytes. – **Au**: V, T, S, K, St, O, N, B. **Ge**: OB. **Sw**: AP, BE, FR, GL, GR, LU, SG, SZ, TI, UR, UW, VD, VS. **Fr**: AHP, HAl, AMa, Isè, HSav, Var, Vau. **It**: Frl, Ven, TAA, Lomb, Piem, VA, Lig. **Sl**: SlA, Tg. **Li**.

***Lepra aspergilla* (Ach.) Hafellner**

Syn.: *Lichen aspergillus* Ach., *Marfloraea aspergilla* (Ach.) S.Y. Kondr., Lőkös & Hur, *Pertusaria aspergilla* (Ach.) J.R. Laundon, *Pertusaria dealbata auct.*, *Pertusaria dealbescens auct.*, *Pertusaria leucosora auct*.

L – Subs.: sil – Alt.: 2–5 – Note: on steeply inclined surfaces of siliceous rocks, with optimum in the montane belt. – **Au**: ?V, T, S, St. **Sw**: TI, VS. **It**: TAA, Lomb, Piem, VA, Lig. **Sl**: SlA.

***Lepra borealis* (Erichsen) I. Schmitt, Hodkinson & Lumbsch**

Syn.: *Pertusaria borealis* Erichsen

L – Subs.: cor – Alt.: 3 – Note: a mostly sterile species, morphologically resembling the *globulifera*-form of *L. albescens*, but with a different secondary chemistry (fumarprotocetraric and protocetraric acids in medulla and soralia) and therefore reacting Pd+ orange-red; based on a type from Alaska, but also known from NW Europe on the bark of deciduous trees, more rarely of conifers; in the Alps there are so far only a few records from forests under suboceanic climatic conditions. – **Au**: N. **Sw**: GR, UW.

***Lepra corallina* (L.) Zahlbr.**

Syn.: *Lichen corallinus* L., *Marfloraea corallina* (L.) S.Y. Kondr., Lőkös & Hur, *Pertusaria corallina* (L.) Arnold, *Pertusaria subdubia* Nyl., *Variolaria corallina* (L.) Ach.

L – Subs.: sil – Alt.: 2–5 – Note: a cool-temperate to boreal-montane lichen found on steeply inclined surfaces of siliceous rocks in rainy areas, where it is sometimes very abundant; widespread throughout the Alps. – **Au**: V, T, S, K, St, O. **Ge**: Schw. **Sw**: BE, GR, LU, TI, UR, VS. **Fr**: HAl, AMa, Isè, Sav, HSav, Vau. **It**: Frl, TAA, Lomb, Piem, VA. **Sl**: SlA.

***Lepra dactylina* (Ach.) Hafellner**

Syn.: *Lichen dactylinus* Ach., *Ochrolechia dactylina* (Ach.) S.Y. Kondr., Lökös & Hur, *Pertusaria dactylina* (Ach.) Nyl.

L – Subs.: sil, ter-sil – Alt.: 5 – Note: a species with a more or less white thallus and tall isidia, medulla Pd+ yellow turning to orange-red (fumarprotocertaric acid), verrucae in which the lecanorate ascomata show flesh-coloured to dark-brown immersed discs, 1-spored asci, and huge ascospores (100–250 × 50–110 μm) with an unzoned wall; mostly on detritus and mosses, rarely directly on soil or siliceous rock in tundra vegetation; circumpolar in the Arctic, scattered further south in the mountains, *e.g.* in Scotland, with a few records from Switzerland and Austria, some of which require confirmation. – **Au**: T, ?St. **Sw**: ?Sw.

***Lepra erumpens* (Erichsen) Hafellner**

Syn.: *Pertusaria erumpens* Erichsen

L – Subs.: sil – Alt.: 2 – Note: a silicicolous species resembling *L. aspergilla*, with a grey, rimose thallus with minute soralia, with fumarprotocetraric and protocetraric acid in medulla and soralia, and therefore reacting Pd+ orange-red; rare in Europe, but perhaps not always distinguished. – **Au**: St. **It**: Lig.

***Lepra excludens* (Nyl.) Hafellner**

Syn.: *Marfloraea excludens* (Nyl.) S.Y. Kondr., Lőkös & Hur, *Pertusaria excludens* Nyl.

L – Subs.: sil – Alt.: 1–5 – Note: a mainly temperate species of sheltered siliceous rocks. – **Au**: ?V, T, S, K, St, N. **Sw**: UR. **Fr**: AMa, HSav, Vau. **It**: Frl, VA, Lig.

***Lepra leucosora* (Nyl.) Hafellner**

Syn.: *Pertusaria digrediens* Nyl., *Pertusaria leucosora* Nyl. *non auct*.

L – Subs.: sil – Alt.: 2–3 – Note: on siliceous rocks in upland areas. According to Roux et al. (2017), *Pertusaria digrediens* is a synonym of *L. leucosora*. – **Fr**: AMa, Isè, Sav, Var, Vau. **It**: TAA, Piem, VA, Lig. **Sl**: SlA.

***Lepra melanochlora* (DC.) Hafellner**

Syn.: *Isidium melanochlorum* DC., *Pertusaria melanochlora* (DC.) Nyl.

L # – Subs.: sil – Alt.: 1–2 – Note: a probably Mediterranean-Atlantic, often misunderstood taxon (*e.g.* confused with *L. mammosa*), found on compact siliceous rocks; the record from Switzerland has neither locality nor collector, and the presence of the species there is more than dubious. In the study area the species is known with certainty only from the French Southern Pre-Alps (Haute-Vésubie). – **Sw**: ?Sw. **Fr**: AMa.

***Lepra monogona* (Nyl.) Hafellner**

Syn.: Pertusaria ceuthocarpa (Sm.) Sm. var. variolosa Mudd, *Pertusaria monogona* Nyl.

L – Subs.: sil – Alt.: 2 – Note: on steeply inclined to vertical surfaces of more or less basic siliceous rocks, often near the coast; in the study area so far known only from the base of the Western Alps (France). – **Fr**: AMa.

***Lepra multipuncta* (Turner) Hafellner**

Syn.: *Pertusaria leptospora* Nitschke *ex* Nyl., *Pertusaria multipuncta* (Turner) Nyl., *Pertusaria sorediata* C. Knight, *Variolaria multipuncta* Turner

L – Subs.: cor – Alt.: 2–3 – Note: a mainly temperate species found on smooth bark of deciduous trees (especially *Carpinus* and *Fagus*) in open, humid deciduous woodlands. – **Au**: V, T, S, K, St, O, N. **Ge**: OB. **Sw**: BE, SG, SZ, TI, VD. **Fr**: HSav. **It**: Frl, Ven, TAA, Lomb, Piem, VA. **Sl**: SlA.

***Lepra ophthalmiza* (Nyl.) Hafellner**

Syn.: *Marfloraea ophthalmiza* (Nyl.) S.Y. Kondr., Lőkös & Hur, *Pertusaria multipuncta auct. non* (Turner) Nyl., Pertusaria multipuncta (Turner) Nyl. var. ophthalmiza Nyl., *Pertusaria ophthalmiza* (Nyl.) Nyl.

L – Subs.: cor – Alt.: 3–4 – Note: a cool-temperate to southern boreal lichen with optimum on the bark of coniferous trees (*Abies*, *Picea*), both on boles and twigs in humid-cold situations (*e.g.* in gorges, dolinas), but also occurring on *Fagus*; certainly more widespread in the Alps; in the past the species might have been confused with *L. multipuncta*, which has a different chemistry. – **Au**: V, T, S, K, St, N. **Ge**: OB, Schw. **Sw**: BE, SZ, UR, UW, VD, VS. **It**: Frl, TAA. **Sl**: SlA.

***Lepra panyrga* (Ach.) Hafellner**

Syn.: *Marfloraea panyrga* (Ach.) S.Y. Kondr., Lőkös & Hur, *Pertusaria panyrga* (Ach.) A. Massal., *Urceolaria panyrga* Ach.

L – Subs.: bry, deb – Alt.: 4 – Note: an arctic-alpine species with a grey thallus (all spot tests negative), with papillate to columnar isidia, occasionally bearing *Lecanora*-like apothecia with sunken dark discs often covered by a grey pruina, and with single-spored asci; encrusting plant debris and terricolous bryophytes at high elevations; in the Alps it is apparently very rare. – **Au**: T.

***Lepra pseudolactea* (Erichsen) Hafellner**

Syn.: *Pertusaria pseudolactea* Erichsen

L – Subs.: sil – Alt.: 5 – Note: a sorediate species recalling *Varicellaria lactea* in habitus, but thallus C-, Pd+ orange-red (fumarprotocetraric and succinprotocetraric acids), apothecia unknown; on siliceous rocks in the alpine belt; so far only recorded from the Eastern Alps (Austria). – **Au**: T.

***Lepra schaereri* (Hafellner) Hafellner**

Syn.: *Pertusaria isidioides* (Schaer.) Arnold *non* (Borrer) Hook. f., *Pertusaria schaereri* Hafellner

L – Subs.: sil – Alt.: 4–6 – Note: on base – or mineral-rich rocks in rainy areas near and above treeline, up to the nival belt. – **Au**: V, T, S, K, St. **Sw**: BE, GR, VS. **Fr**: HAl, HSav. **It**: Frl, TAA, VA.

***Lepra stalactiza* (Nyl.) Hafellner**

Syn.: *Pertusaria stalactiza* Nyl.

L – Subs.: sil – Alt.: 2–4 – Note: a species with a whitish-grey thallus reacting Pd+ orange-red (fumarprotocetraric acid), provided with often persistently hemispherical papillae (which are scarce in fertile forms), *Lecanora*-like apothecia, and single-spored asci; on siliceous rocks in the European orobiomes; very rare in the Alps, below the subalpine belt. – **Au**: S. **Fr**: HAl.

***Lepra trachythallina* (Erichsen) Lendemer & R.C. Harris**

Syn.: *Pertusaria laevigata* (Nyl.) Arnold *non* (Th. Fr.) Anzi, *Pertusaria trachythallina* Erichsen

L – Subs.: cor – Alt.: 2–3 – Note: a cool-temperate, perhaps circumpolar lichen found on smooth bark of deciduous trees, especially *Fagus*, in humid montane forests. – **Au**: T, S, St, O, N. **Sw**: UW. **It**: TAA. **Sl**: SlA.

***Lepra waghornei* (Hulting) Lendemer & R.C. Harris**

Syn.: *Pertusaria waghornei* Hulting

L – Subs.: cor – Alt.: 3 – Note: a suboceanic epiphytic lichen of humid montane forests; apparently very rare in the Alps. – **Au**: T, O. **Ge**: OB, Schw.

***Lepraria alpina* (B. de Lesd.) Tretiach & Baruffo**

Syn.: *Crocynia alpina* B. de Lesd., *Crocynia antarctica* Hue, *Crocynia caerulescens* Hue, *Crocynia candidissima* Hue, *Crocynia henrici* B. de Lesd., *Crocynia minima* Hue, *Crocynia neglecta* Hue, *Lepraria angardiana* Øvstedal, *Lepraria cacuminum* (“A. Massal.”) Loht., *Lepraria caerulescens* (Hue) Botnen & Øvstedal, *Leproloma angardianum* (Øvstedal) J.R. Laundon, *Leproloma cacuminum* (“A. Massal.”) J.R. Laundon

L – Subs.: bry-sil, bry-cal, ter-sil, ter-cal – Alt.: 3–6 – Note: on epilithic mosses and soil in alpine grasslands, both on siliceous and on calcareous substrata, in sites with a long snow cover, up to the nival belt in the Alps. – **Au**: V, T, S, K, St, O. **Sw**: GR, UR, VS. **Fr**: AHP, HAl, AMa. **It**: Frl, Ven, TAA, Lomb, Piem, VA.

***Lepraria borealis* Loht. & Tønsberg**

L – Subs.: bry-sil, ter-sil – Alt.: 2–4 – Note: a circumboreal species growing on siliceous rocks and over epilithic mosses, with optimum in the oroboreal to alpine belts, with scattered records from the Alps. – **Au**: St. **Sw**: VS. **Fr**: AMa, HSav. **It**: Frl, Piem, VA.

***Lepraria caesioalba* (B. de Lesd.) J.R. Laundon**

Syn.: *Crocynia caesioalba* B. de Lesd., *Lepraria zonata* Brodo, *Leproloma caesioalbum* (B. de Lesd.) M. Choisy

L – Subs.: bry, sil – Alt.: 2–5 – Note: on bryophytes, more rarely on siliceous rocks wetted by rain, especially on basal parts of siliceous boulders with a long snow cover; widespread throughout the Alps. – **Au**: V, T, S, K, St. **Sw**: GR, SZ, TI, UR, VS. **Fr**: AHP, AMa, Isè, HSav, Var, Vau. **It**: Frl, Ven, TAA, Lomb, Piem. **Sl**: SlA.

***Lepraria crassissima* (Hue) Lettau**

Syn.: *Crocynia crassissima* Hue

L – Subs.: sil, cal, bry-cal – Alt.: 3–4 – Note: an often misunderstood species occurring on vertical to underhanging surfaces of siliceous, more rarely calciferous rocks and on epilithic mosses in mountain areas. – **Au**: St, N. **Ge**: OB, Schw. **Sw**: SZ. **Fr**: AHP, AMa, Drô, Vau. **It**: Frl.

***Lepraria diffusa* (J.R. Laundon) Kukwa**

Syn.: *Leproloma diffusum* J.R. Laundon

L – Subs.: cal, bry, ter-cal – Alt.: 1–5 – Note: in niches and fissures of calcareous or dolomitic boulders, but also on soil, mosses and plant debris in dry grasslands. – **Au**: T, S, K, St, O, N. **Ge**: Ge. **Sw**: LU, SZ. **It**: Frl, Ven, TAA, Piem, Lig. **Sl**: SlA.

***Lepraria eburnea* J.R. Laundon**

Syn.: *Lepraria frigida* J.R. Laundon

L – Subs.: cor, bry, deb, ter-sil, sil – Alt.: 2–5 – Note: on old trunks in underhangs protected from rain, but also on walls in urban habitats; widespread throughout the Alps. – **Au**: V, T, S, K, St, O, N. **Ge**: OB. **Sw**: BE, GL, GR, LU, SZ, TI, UR, UW, VS. **Fr**: AHP, AMa, Sav. **It**: Frl, Ven, TAA. Lig. **Sl**: SlA, Tg.

***Lepraria elobata* Tønsberg**

L – Subs.: cor, xyl – Alt.: 2–4 – Note: a mainly montane species requiring humid conditions; it prefers acid bark not colonised by bryophytes, especially at the base of old trunks, but it rarely occurs also on soil, lignum and epiphytic mosses; widespread throughout the Alps. – **Au**: T, St, N. **Sw**: AP, BE, FR, GL, GR, LU, SG, SZ, TI, UR, UW, VD, VS. **Fr**: AMa, Drô. **It**: Frl, Ven, TAA, Piem Lig. **Sl**: SlA, Tg.

***Lepraria finkii* (B. de Lesd.) R.C. Harris**

Syn.: *Crocynia aliciae* Hue, *Crocynia finkii* B. de Lesd., *Crocynia lobificans auct. non* (Nyl.) Hue, *Crocynia mollissima* B. de Lesd., *Crocynia sciatropha* Hue, *Lepraria aeruginosa auct. p.p.*, *Lepraria lobificans auct. non* Nyl., *Leproloma lobificans auct. non* (Nyl.) Boistel

L – Subs.: cor, bry, xyl, sax, ter – Alt.: 1–4 – Note: one of the most common species of the genus, found in the lower parts of trunks, but also on rocks, lignum, soil and mosses, also occurring in rather polluted areas and on faces wetted by rain. This species was mostly called *L. lobificans*, but the type of that species proved to be identical with *L. santosii*; widespread throughout the Alps. – **Au**: V, T, S, K, St, O, N. **Ge**: OB. **Sw**: AP, BE, FR, GL, GR, LU, SG, SZ, TI, UR, UW, VD, VS. **Fr**: AHP, HAl, AMa, Isè, Var, Vau. **It**: Frl, Ven, TAA, Lomb, Piem, Lig. **Sl**: SlA, Tg.

***Lepraria granulata* Slavíková**

L – Subs.: bry-sil, sil – Alt.: 4 – Note: a species of the *L. neglecta*-group with a whitish-grey granular thallus similar to that of *L. borealis*, both with atranorin but with different fatty acids; overgrowing mosses mostly on siliceous boulders, rarely in shaded fissures; not common at high elevations in the European orobiomes, with a few records from the Eastern Alps (Austria). – **Au**: K, St.

***Lepraria incana* (L.) Ach.**

Syn.: *Byssus incana* L., *Crocynia maritima* B. de Lesd., *Lepra sulphurea* (Schltdl.) Ehrh., *Lepraria aeruginosa auct.*, *Lepraria glaucella* (Flörke) Ach., *Lepraria sulphurea* Schltdl., *Patellaria incana* (L.) Spreng.

L – Subs.: cor, bry, deb, ter-cal, ter-sil, xyl – Alt.: 1–5 – Note: on acid bark of coniferous and deciduous trees in sites protected from rain, sometimes on siliceous rocks, soil and lignum; widespread throughout the Alps. – **Au**: V, T, S, K, St, O, N. **Ge**: OB. **Sw**: BE, FR, GL, GR, LU, SG, SZ, TI, UW, VD, VS. **Fr**: AHP, HAl, AMa, Drô, Isè, Sav, HSav, Var, Vau. **It**: Frl, Ven, TAA, Lomb, Piem, Lig. **Sl**: SlA, Tg. **Li**.

***Lepraria isidiata* (Llimona) Llimona & A. Crespo**

Syn.: Lepraria crassissima (Hue) Lettau var. isidiata Llimona

L – Subs.: ter-sil – Alt.: 2 – Note: on calciferous soil and on mosses in sheltered situations, but in sunny and arid habitats, also on gypsum; in the study area so far only reported from the base of the Western Alps. – **Fr**: AMa.

***Lepraria jackii* Tønsberg**

Syn.: *Lepraria toensbergiana* Slav.-Bay. & Kukwa

L – Subs.: cor, xyl, sil – Alt.: 1–4 – Note: on the acid to subneutral bark of conifers and other trees, especially on basal parts of trunks in woodlands, but also on siliceous rocks and wood. – **Au**: V, S, K, St, O, N. **Sw**: BE, GL, GR, LU, SG, SZ, TI, UR, UW, VD, VS. **It**: Frl, Ven, TAA, Piem. **Sl**: SlA.

***Lepraria membranacea* (Dicks.) Vain.**

Syn.: *Amphiloma lanuginosum* (Ach.) Nyl., *Crocynia lanuginosa* (Ach.) Hue, *Crocynia membranacea* (Dicks.) Zahlbr., *Leproloma lanuginosum* (Ach.) Nyl. *ex* Cromb., *Leproloma membranaceum* (Dicks.) Vain., *Lichen membranaceus* Dicks., *Pannaria lanuginosa* (Ach.) Körb., *Psoroma lanuginosum* (Ach.) Müll. Arg.

L – Subs.: sil, ter-sil, bry-sil, cor – Alt.: 1–5 – Note: on steeply inclined to weakly underhanging surfaces of siliceous rocks, sometimes on epilithic bryophytes, much more rarely on bark, often forming monospecific stands; widespread throughout the Alps. – **Au**: V, T, S, K, St, O, N. **Sw**: BE, GL, GR, LU, SZ, TI, VD, VS. **Fr**: AHP, HAl, AMa, Isè, Sav, Vau. **It**: Frl, TAA, Lomb, Piem, VA, Lig.

***Lepraria neglecta* (Nyl.) Erichsen**

Syn.: *Crocynia neglecta* (Nyl.) Hue, *Lecidea neglecta* Nyl., *Lecidella neglecta* (Nyl.) Stein

L – Subs.: bry, sil, ter-sil, bry-sil – Alt.: 2–6 – Note: a mainly arctic-alpine lichen found on moss cushions and stony siliceous ground, mostly in snow-beds near or above treeline; widespread throughout the Alps. – **Au**: V, T, S, K, St, O, N. **Ge**: OB. **Sw**: BE, GR, LU, TI, UR, VD, VS. **Fr**: AHP, HAl, AMa, Isè, Sav, HSav, Vau. **It**: TAA, Lomb, Piem, VA.

***Lepraria nivalis* J.R. Laundon**

Syn.: *Crocynia murorum* B. de Lesd., *Lepraria crassissima auct. non* (Hue) Lettau

L – Subs.: cal, cor – Alt.: 1–5 – Note: on lime-rich rocks, on mosses, but also on bark, on steeply inclined or underhanging faces protected from rain; widespread throughout the Alps. – **Au**: V, T, S, K, St, O, N. **Ge**: OB, Schw. **Sw**: BE, LU, SZ, UR, UW, VS. **Fr**: AHP, HAl, AMa, Drô, Isè, Sav, Var, Vau. **It**: Frl, TAA, Lig. **Sl**: SlA.

***Lepraria nylanderiana* Kümmerl. & Leuckert**

L – Subs.: sil, cor, ter-sil – Alt.: 1–3 – Note: on base-rich siliceous rocks and soil, including brick walls, but also on bark; the species can be considered as a good indicator of long ecological continuity, since it always occurs in old and well-preserved forests; in the Alps it is evidently rare. – **Au**: K. **Fr**: AMa. **It**: Piem.

***Lepraria obtusatica* Tønsberg**

L – Subs.: ter, cor – Alt.: 3–5 – Note: a species with a thallus which is greenish when young, turning grey-yellowish with age, with minute soredia and containing obtusatic acid as the major substance; on bark, rarely also on other substrates in shaded situations under suboceanic climates; not common throughout its distributional range, with a few records from the Alps. – **Sw**: GL, LU, UW. **Fr**: AHP, AMa.

***Lepraria rigidula* (B. de Lesd.) Tønsberg**

Syn.: *Crocynia rigidula* B. de Lesd.

L – Subs.: cor, bry, sil, xyl, ter – Alt.: 1–5 – Note: an ecologically wide-ranging species, certainly widespread and locally common, also in the Alps. It seems to prefer acidic substrata and is mainly epiphytic. – **Au**: V, T, S, K, St, O. **Ge**: OB, Schw. **Sw**: AP, BE, FR, GL, GR, LU, SG, SZ, TI, UR, UW, VD, VS. **Fr**: AMa. **It**: Frl, TAA, Lomb, Piem, Lig. **Sl**: SlA, Tg.

***Lepraria sylvicola* Orange**

L – Subs.: xyl – Alt.: 2 – Note: a species which is morphologically similar to *L. jackii*, with a bluish white to bluish grey thallus containing atranorin, roccellic and toensbergianic acids as major substances, with thalline granules of medium size, based on a type from Scotland; on the not too acidic bark of deciduous trees, rarely also on other substrates (wood, basic siliceous rock) in moist forests and woodlands; more common in Western Europe, especially in oak forests, with a few records from the Eastern Alps (Austria). – **Au**: St.

***Lepraria umbricola* Tønsberg**

L – Subs.: bry, cor – Alt.: 2–3 – Note: a warm-temperate species found on sheltered siliceous rocks and mosses, sometimes on the basal parts of old trunks and on shaded sandy soil, with a few records from the Eastern Alps (Austria). – **Au**: St, N.

***Lepraria vouauxii* (Hue) R.C. Harris**

Syn.: *Crocynia arctica* Lynge, *Crocynia vouauxii* Hue, *Lepraria arctica* (Lynge) Wetmore, *Leproloma vouauxii* (Hue) J.R. Laundon

L – Subs.: cor, ter-sil, sax – Alt.: 1–5 – Note: on isolated deciduous trees, in positions which are seldom wetted by rain, sometimes on brick walls, with a wide ecological and altitudinal range; widespread throughout the Alps. – **Au**: V, T, S, K, St, O, N. **Ge**: OB, Schw. **Sw**: BE, FR, GL, GR, LU, SG, SZ, TI, VD, VS. **Fr**: AMa, Var, Vau. **It**: Frl, Ven, TAA, lomb, Piem, Lig. **Sl**: SlA, Tg.

***Leprocaulon quisquiliare* (Leers) M. Choisy**

Syn.: *Leprocaulon microscopicum* (Vill.) Gams, *Leprocaulon nanum* (Ach.) Nyl. *ex* Lamy, *Lichen microscopicus* Vill., *Lichen quisquiliaris* Leers, *Stereocaulon microscopicum* (Vill.) Frey, *Stereocaulon nanum* (Ach.) Ach., *Stereocaulon quisquiliare* (Leers) Hoffm.

L – Subs.: ter-sil, sil, bry-sil, cor – Alt.: 1–5 – Note: a mainly mild-temperate to Mediterranean lichen found on basic siliceous rocks covered by a thin film of soil, sometimes even on subacid bark, exceptionally reaching the montane belt in the Western Alps. – **Au**: V, T, S, K, St, N. **Sw**: BE, GR, TI, UR, VS. **Fr**: AMa, Isè, HSav, Var, Vau. **It**: Ven, TAA, Lomb, Piem, VA, Lig.

***Leptochidium albociliatum* (Desm.) M. Choisy**

Syn.: *Collema albociliatum* (Desm.) Nyl., *Leptogium albociliatum* Desm., *Polychidium albociliatum* (Desm.) Zahlbr.

L – Subs.: ter-sil, bry-sil, sil – Alt.: 2–5 – Note: a cool-temperate to arctic-alpine lichen found amongst bryophytes on rocks or on soil in open shrublands and grasslands on basic siliceous substrata. – **Sw**: GR, TI, VS. **Fr**: AMa, Var, Vau. **It**: TAA, Lomb, Piem.

***Leptogium brebissonii* Mont.**

Syn.: *Leptogium chloromelum auct. non* (Ach.) Nyl., *Leptogium ruginosum* (Schaer.) Nyl., Parmelia membranacea Pers. var. ruginosa Schaer. *Synechoblastus ruginosus* (Schaer.) Hepp

L – Subs.: cor – Alt.: 1–2 – Note: a mild-temperate to humid subtropical lichen, most frequent in open, humid woodlands, especially in old, coastal plantations of *Olea* at the base of the Western Alps. – **Fr**: AMa, Var, Vau. **It**: Lig. **Sl**: Tg.

***Leptogium burnetiae* C.W. Dodge**

Syn.: Leptogium menziesii (Sm.) Mont. f. fuliginosum Müll. Arg.

L – Subs.: cor – Alt.: 2–3 – Note: a mild-temperate species found on the often mossy bark of isolated trees, especially *Fraxinus*, with scattered records from the Alps. – **Au**: T. **Sw**: SZ. **Fr**: AHP, AMa, Var. **It**: Lomb, Lig.

***Leptogium coralloideum* (Meyen & Flot.) Vain.**

Syn.: *Leptogium corrugatomontuosum* Couderc, Leptogium diaphanum (Sw.) Mont. var. coralloideum Meyen & Flot.

L – Subs.: cor – Alt.: 1–2 – Note: a Mediterranean-Atlantic species found on bark of broad-leaved trees, with a few records from the base of the Western Alps. – **Fr**: AMa, Var. **It**: Lig.

***Leptogium corticola* (Taylor) Tuck.**

Syn.: *Collema corticola* Taylor, *Leptogium cimiciodorum* A. Massal.

L – Subs.: cor, bry – Alt.: 1–3 – Note: a mild-temperate to humid subtropical species found in ancient, humid forests; most of the records are old: presently extinct in large parts of its former range. – **Au**: St. **It**: Ven, Lomb, Piem.

***Leptogium cyanescens* (Ach.) Körb.**

Syn.: *Collema cyanescens* (Ach.) Rabenh., Collema tremelloides Ach. f. cyanescens Ach. *Leptogium caesium* (Ach.) Vain., *Leptogium tremelloides auct*.

L – Subs.: bry, sil, ter, cor – Alt.: 1–3 – Note: a mild-temperate to humid subtropical lichen found in humid, old, open forests, occasionally on rocks and epilithic mosses; most of the records are old. – **Au**: V, T, K, St, O, N. **Sw**: BE, GL, GR, TI, VD, VS. **Fr**: AHP, AMa, Isè, Var, Vau. **It**: Frl, Ven, TAA, Lomb, Piem, VA. **Sl**: SlA, Tg.

***Leptogium furfuraceum* (Harm.) Sierk**

Syn.: Leptogium hildenbrandii (Garov.) Nyl. f. furfuraceum Harm.

L – Subs.: cor – Alt.: 3 – Note: a mild-temperate to humid subtropical species of open woodlands in warm-humid areas; rare in the Alps. – **Au**: T. **Fr**: AHP, AMa, Var.

***Leptogium hibernicum* M.E. Mitch. *ex* P.M. Jørg.**

L – Subs.: cor – Alt.: 2 – Note: a species with a thallus swelling considerably when moistened, the broad lobes showing a wrinkled upper surface provided with nodular isidia and marginal lobules, the lower surface with short hairs, looking almost like a pruina; on the subneutral bark of deciduous trees, also overgrowing bryophytes; a rare species in Western Europe, with a few records from the Western Alps (France). – **Fr**: AMa, Var.

***Leptogium hildenbrandii* (Garov.) Nyl.**

Syn.: *Collema hildenbrandii* Garov., Leptogium saturninum (Dicks.) Nyl. var. complicatum Anzi

L – Subs.: cor – Alt.: 2–3 – Note: on isolated trees with base-rich bark, especially *Juglans, Fraxinus* and *Populus* in humid valleys with a rather continental climate; widespread throughout the Alps, but much more common in the past, presently declining. – **Au**: T, K. **Ge**: OB. **Sw**: BE, GR, TI, VD, VS. **Fr**: AHP, AMa, Drô, Isè, Sav, HSav, Var, Vau. **It**: Frl, Ven, TAA, Lomb, Piem, VA.

***Leptogium rivulare* (Ach.) Mont.**

Syn.: *Leptogium crenatellum* Tuck., *Leptogium sernanderi* Du Rietz, *Lichen rivularis* Ach.

L – Subs.: sil, cor – Alt.: 2–4 – Note: a species resembling a small *L. cyanescens*, but lacking isidia, with a blue-grey thallus usually bearing laminal apothecia; on rocks or roots of deciduous trees along rivers; a rare holarctic species, with a few records from the Western Alps (France). – **Fr**: HAl, AMa.

***Leptogium saturninum* (Dicks.) Nyl.**

Syn.: *Collema myochroum* (Ehrh. *ex* Bernh.) Rabenh., *Collema saturninum* (Dicks.) DC., *Leptogium myochroum* (Ehrh. *ex* Bernh.) Nyl., *Lichen saturninus* Dicks., *Mallotium saturninum* (Dicks.) Gray, *Mallotium tomentosum* (Hoffm.) Körb.

L – Subs.: cor, bry-cor – Alt.: 2–4 – Note: a cool-temperate to boreal-montane, circumpolar lichen found on bark, rarely on mossy rocks, only locally common, especially in upland areas; widespread throughout the Alps, but generally not very common. – **Au**: V, T, S, K, St, O, N. **Ge**: OB, Schw. **Sw**: BE, GL, GR, LU, SZ, TI, UR, UW, VD, VS. **Fr**: AHP, HAl, AMa, Isè, Sav, HSav, Var, Vau. **It**: Frl, Ven, TAA, Lomb, Piem, VA. **Sl**: SlA, Tg.

***Letharia vulpina* (L.) Hue**

Syn.: *Chlorea vulpina* (L.) Nyl., *Evernia vulpina* (L.) Ach., *Lichen vulpinus* L., *Parmelia vulpina* (L.) Ach., *Rhytidocaulon vulpinum* (L.) Elenkin

L – Subs.: cor, xyl – Alt.: 3–4 – Note: a circumboreal-montane lichen growing on the bark of coniferous trees, mostly on *Larix* and *Pinus cembra*, more rarely on lignum, near treeline; widespread throughout the Alps, but common and locally abundant only in areas with a continental climate. – **Au**: V, T, S, K, St, O, N. **Ge**: OB. **Sw**: AP, BE, GR, LU, TI, UW, VD, VS. **Fr**: AHP, HAl, AMa, Isè, Sav, HSav, Var. **It**: Frl, Ven, TAA, Lomb, Piem, VA, Lig. **Sl**: SlA.

***Lichenomphalia alpina* (Britzelm.) Redhead, Lutzoni, Moncalvo & Vilgalys**

Syn.: *Agaricus alpinus* Britzelm., *Botrydina luteovitellina* (Pilát & Nannf.) Redhead & Kuyper, *Botrydina vulgaris* Bréb. *p.p.*, *Gerronema luteovitellinum* (Pilát & Nannf.) Singer, *Gerronema alpinum* (Britzelm.) Bresinsky & Stangl, *Omphalia flava* (Cooke) F.H. Møller, *Omphalia luteovitellina* Pilát & Nannf., *Omphalina alpina* (Britzelm.) Bresinsky & Stangl, *Omphalina flava* (Cooke) M. Lange, *Omphalina luteovitellina* (Pilát & Nannf.) M. Lange, *Phytoconis luteovitellina* (Pilát & Nannf.) Redhead & Kuyper

L – Subs.: bry, ter-sil, xyl – Alt.: 4–5 – Note: a basidiolichen of acid organic soil, most common around treeline; perhaps more widespread in the Alps, but regionally overlooked by lichenologists. – **Au**: V, T, S, K, St. **Ge**: Schw. **Sw**: GR. **Fr**: HSav. **It**: TAA.

***Lichenomphalia hudsoniana* (H.S. Jenn.) Redhead, Lutzoni, Moncalvo & Vilgalys**

Syn.: *Coriscium viride* (Ach.) Vain., *Gerronema hudsonianum* (H.S. Jenn.) Singer, *Gerronema luteolilacinum* (J. Favre) Singer, *Hygrophorus hudsoniuanus* H.S. Jenn., *Normandina laetevirens* (Borrer) Nyl., *Normandina viridis* (Ach.) Nyl., *Omphalia hudsoniana* H.S. Jenn., *Omphalia luteolilacina* J. Favre, *Omphalina coriscium* Gams, *Omphalina hudsoniana* (H.S. Jenn.) H.E. Bigelow, *Omphalina luteolilacina* (J. Favre) D.M. Hend., *Phytoconis viridis* (Ach.) Redhead & Kuyper, *Verrucaria laetevirens* Borrer

L – Subs.: bry, deb, ter-bry – Alt.: 2–5 – Note: a circumboreal-montane basidiolichen of wet mosses and peaty soil, more rarely found also on rotting wood in siliceous areas, with optimum near and above treeline. – **Au**: V, T, S, K, St, O, N. **Ge**: OB. **Sw**: GL, GR, SZ. **It**: Frl, TAA, Lomb, Piem, VA.

***Lichenomphalia umbellifera* (L. : Fr.) Redhead, Lutzoni, Moncalvo & Vilgalys**

Syn.: *Agaricus chrysoleucus* Pers., *Agaricus pseudoandrosaceus* Bull., *Agaricus umbelliferus* L. : Fr., *Botrydina vulgaris* Bréb. *p.p.*, *Gerronema ericetorum* (Pers.) Singer, *Omphalia umbellifera* (L. : Fr.) P. Kumm., *Omphalina ericetorum* (Pers.) M. Lange, *Omphalina fulvopallens* P.D. Orton, *Omphalina pseudandrosacea* (Bull.) M.M. Moser *non auct.*, *Omphalina umbellifera* (L.) Quél., *Phytoconis ericetorum* (Pers.) Redhead & Kuyper

L – Subs.: xyl, ter-sil, deb, bry – Alt.: 2–5 – Note: on acid organic soil and rotting wood, ecologically similar to *L. hudsoniana*, but rarer above treeline; widespread throughout the Alps. – **Au**: V, T, S, K, St, O, N. **Ge**: OB. **Sw**: GR, LU, SZ, UW, VD. **Fr**: AMa, Isè, Sav, HSav, Var, Vau. **It**: Frl, Ven, TAA, Lomb, Piem, Lig. **Sl**: SlA.

***Lichenomphalia velutina* (Quél.) Redhead, Lutzoni, Moncalvo & Vilgalys**

Syn.: *Botrydina velutina* (Quél.) Redhead & Kuyper, *Botrydina vulgaris* Bréb. *p.p.*, *Lichenomphalia grisella* (P. Karst.) Redhead, Lutzoni, Moncalvo & Vilgalys, *Omphalia grisella* P. Karst., *Omphalia velutina* Quél., *Omphalina grisella* (P. Karst.) M.M. Moser, *Omphalina pseudandrosacea auct. non* (Bull.) M.M. Moser, *Omphalina velutina* (Quél.) Quél., *Phytoconis pararustica* (Clémençon) P. Roux & P.-A. Moreau, *Phytoconis velutina* (Quél.) Redhead & Kuyper

L – Subs.: ter-sil, bry – Alt.: 2–5 – Note: on acid soil, often in clearings of *Pinus*-stands; known only from Europe (French, Swiss, Austrian and Italian Alps), and North America. – **Au**: S, K, St. **Sw**: GR, UR, VS. **Fr**: Sav, Var. **It**: Ven, TAA, Lomb.

***Lichinella cribellifera* (Nyl.) P.P. Moreno & Egea**

Syn.: *Gonohymenia cribellifera* (Nyl.) Henssen, *Omphalaria cribellifera* Nyl., *Rechingeria cribellifera* (Nyl.) Servít, *Thyrea cribellifera* (Nyl.) Zahlbr.

L – Subs.: sil – Alt.: 2 – Note: on steeply inclined faces of siliceous rocks, especially in seepage tracks, usually below the montane belt; in the study area only reported from the Western Alps (France). – **Fr**: AMa, Var.

***Lichinella heppii* (Müll. Arg.) P. Clerc & Cl. Roux**

Syn.: *Gonohymenia heppii* (Müll. Arg.) Henssen, *Omphalaria heppii* Müll. Arg., *Thyrea heppii* (Müll. Arg.) Lettau

L # – Subs.: cal, sil – Alt.: 2–3 – Note: a critical taxon, purported to be most frequent in humid stands by rivers. The type is from France: “*sandstone boulders along the Arve near Mornex*”. – **Sw**: VS. **Fr**: HSav.

***Lichinella iodopulchra* (Couderc *ex* Croz.) P.P. Moreno & Egea**

Syn.: *Gonohymenia iodopulchra* (Couderc *ex* Croz.) Henssen, Parmelia stygia Schaer. var. pulvinata Schaer., *Omphalaria iodopulchra* Couderc *ex* Croz., *Omphalaria pulvinata auct. non* (Schaer.) Nyl., *Thyrea pulvinata* (Schaer.) A. Massal.

L – Subs.: cal – Alt.: 2 – Note: on steeply inclined to vertical seepage tracks of more or less calcareous or basic siliceous rocks; most common in the Mediterranean belt, with a few scattered records from the Southern and Western Alps. – **Fr**: Var. **It**: Lomb.

***Lichinella octosporella* (Lettau) ined. (provisionally placed here, ICN Art. 36.1b)**

Syn.: *Gonohymenia octosporella* Lettau

L – Subs.: ?cal, int – Alt.: 4 – Note: a species with black thin squamules, flat apothecia, 8-spored asci, and globose ascospores; so far only reported from the base of the Western Alps (France), and from Bavaria. – **Ge**: Schw. **Fr**: AMa.

***Lichinella schleicheri* (Hepp) ined. (provisionally placed here, ICN Art. 36.1b)**

Syn.: *Gonohymenia schleicheri* (Hepp) Henssen, Omphalaria pulvinata (Schaer.) Nyl. var. schleicheri Hepp

L – Subs.: cal, sil – Alt.: 2–3 – Note: a species of the *L. iodopulchra*-group with small foliose black thalli with flat, roundish lobes containing thallinocarps; on periodically wet rock faces, mostly of limestone and calcareous schists; widespread in Europe but probably undercollected; with only a few records from the Alps. – **Au**: K. **Sw**: TI.

***Lichinella stipatula* Nyl.**

L – Subs.: sil, int, cal – Alt.: 1–4 – Note: a holarctic lichen found on steeply inclined, sun-exposed seepage tracks of slightly calciferous or basic siliceous rocks, often overgrowing other lichens; certainly more widespread in the Alps, especially in dry-warm areas. – **Au**: T, St. **Sw**: VS. **Fr**: AMa, Var, Vau. **It**: TAA, Lomb, Piem, VA, Lig.

***Lithographa tesserata* (DC.) Nyl.**

Syn.: *Graphis petraea* (Ach.) Wallr., *Haplographa tumida* Anzi, *Lithographa petraea* (Ach.) Nyl., Lithographa tesserata (DC.) Nyl. var. petraea (Ach.) Redinger, *Lithographa tumida* (Anzi) Ozenda & Clauzade, *Opegrapha petraea* Ach., *Opegrapha tesserata* DC., *Placographa nivalis* Th. Fr., *Placographa tesserata* (DC.) Th. Fr.

L – Subs.: cal, sil – Alt.: 3–5 – Note: on sheltered base-rich siliceous rocks in humid upland areas. – **Au**: T, S, St, N. **Ge**: Schw. **Sw**: UR. **It**: TAA, Lomb.

***Lithothelium triseptatum* (Nyl.) Aptroot**

Syn.: Acrocordia conoidea (Fr.) Körb. var. triseptata (Nyl.) Boistel, *Acrocordia triseptata* (Nyl.) Vězda, *Porina acrocordioides* (Zahlbr.) Zahlbr., *Porina lilacina* Zschacke, *Spermatodium triseptatum* (Nyl.) Trevis., Verrucaria conoidea Fr. var. triseptata Nyl.

L – Subs.: cal – Alt.: 2 – Note: a subtropical species of sheltered, warm-humid, shaded surfaces of calcareous rocks, usually not far from the sea; mainly Mediterranean in Europe, with a few records from the base of the Western Alps (France). – **Fr**: Var, Vau.

***Lobaria linita* (Ach.) Rabenh.**

Syn.: *Sticta linita* Ach.

L – Subs.: sil, bry, ter-sil – Alt.: 3–5 – Note: a circumpolar, arctic-alpine species found on bryophytes and acid soil rich in humus over siliceous substrata near and above treeline; widespread throughout the Alps, but generally rare. – **Au**: V, T, S, K, St, ?O, N. **Ge**: Schw. **Sw**: BE, GL, GR, SZ, TI, UR, VD, VS. **Fr**: HAl, AMa, Isè, Sav, HSav. **It**: Frl, Ven, TAA, Lomb, Piem, VA. **Sl**: SlA.

***Lobaria pulmonaria* (L.) Hoffm.**

Syn.: *Lichen pulmonarius* L., *Lobaria pulmonacea* (Ach.) Shirley, *Sticta pulmonacea* (Ach.) Ach., *Sticta pulmonaria* (L.) Biroli

L – Subs.: cor, bry, sax – Alt.: 1–3 – Note: a mainly temperate, holarctic species found on bark, epiphytic and epilithic mosses, in humid forests, presently with optimum in the montane belt; widespread throughout the Alps, but probably declining. – **Au**: V, T, S, K, St, O, N. **Ge**: OB, Schw. **Sw**: BE, FR, GL, GR, LU, SG, SZ, TI, UR, UW, VD, VS. **Fr**: AHP, HAl, AMa, Drô, Isè, Sav, HSav, Var, Vau. **It**: Frl, Ven, TAA, Lomb, Piem, VA, Lig. **Sl**: SlA, Tg.

***Lobarina scrobiculata* (Scop.) Nyl. *ex* Cromb.**

Syn.: *Lichen scrobiculatus* Scop., *Lobaria scrobiculata* (Scop.) DC., *Lobaria verrucosa* (Huds.) Hoffm., *Lobarina verrucosa* (Huds.) Gyeln. *ex* Räsänen, *Parmelia scrobiculata* (Scop.) Ach., *Sticta scrobiculata* (Scop.) Ach., *Stictina scrobiculata* (Scop.) Nyl.

L – Subs.: cor, sil, bry, bry-sil, ter – Alt.: 2–4 – Note: a mild-temperate, suboceanic species found on old deciduous trees and on mossy rocks in humid open forests; formerly more frequent throughout the Alps, and presently extinct in several parts of its former range. – **Au**: V, T, S, K, St, O, N. **Ge**: OB. **Sw**: BE, GL, GR, LU, SG, UR, UW, VD, VS. **Fr**: AHP, AMa, Sav, HSav, Var. **It**: Frl, Ven, TAA, Lomb, Piem, VA. **Sl**: SlA, Tg.

***Lobothallia alphoplaca* (Wahlenb.) Hafellner**

Syn.: *Acarospora polycarpa* Th. Fr., *Aspicilia alphoplaca* (Wahlenb.) Poelt & Leucker, *Lecanora alphoplaca* (Wahlenb.) Ach., Lecanora alphoplaca (Wahlenb.) Ach. var. inflata Ach., *Lecanora inflata* (Ach.) Jatta, Lecanora melanaspis (Ach.) Ach. var. alphoplaca (Wahlenb.) Th. Fr., *Parmelia alphoplaca* Wahlenb., *Placodium alphoplacum* (Wahlenb.) Link, *Placodium inflatum* (Ach.) A. Massal., *Squamaria alphoplaca* (Wahlenb.) Duby

L – Subs.: sil, ter-cal – Alt.: 2–5 – Note: a widespread species with an apparently disjunct distribution in mountain areas of the Northern Hemisphere, found on compact siliceous rocks wetted by rain in upland areas. – **Au**: T, S, K, St, N. **Sw**: BE, GR, LU, TI, VS. **Fr**: AHP, HAl, AMa, Isè, Sav, HSav. **It**: Frl, TAA, Lomb, Piem, VA.

***Lobothallia cernohorskyana* (Clauzade & Vězda) A. Nordin, Cl. Roux & Sohrabi**

Syn.: *Aspicilia cernohorskyana* (Clauzade & Vězda) Cl. Roux, *Lecanora cernohorskyana* Clauzade & Vězda

L – Subs.: cal – Alt.: 2 – Note: a mainly Mediterranean species growing on soft, porous calcareous marls in sunny situations, with a few records from the base of the Western Alps (France). – **Fr**: Drô, Var, Vau.

***Lobothallia chadefaudiana* (Cl. Roux) A. Nordin, Cl. Roux & Sohrabi**

Syn.: Aspicilia cernohorskyana (Clauzade & Vězda) Cl. Roux var. macedonica Vězda, *Aspicilia chadefaudiana* Cl. Roux

L – Subs.: cal – Alt.: 2–4 – Note: similar to *L. cernohorskyana* in the greenish-brown colour of the epihymenium and the minute ascospores, but thalli areolate-squamulose and thick from the beginning, and apothecia persistently immersed; on various types of calciferous rocks (limestone, dolomite); in the Alps reported from the lowlands to the subalpine ecotone. – **Au**: T, K, St. **Fr**: AHP, AMa, Sav, Var.

***Lobothallia cheresina* (Müll. Arg.) A. Nordin, Cl. Roux & Sohrabi**

Syn.: Aspicilia calcarea (L.) Körb. var. microspora Arnold, *Aspicilia cheresina* (Müll. Arg.) Hue, Aspicilia cheresina (Müll. Arg.) Hue var. microspora (Arnold) Clauzade & Cl. Roux, Aspicilia cheresina (Müll. Arg.) Hue var. justii (Servít) Clauzade & Cl. Roux, *Aspicilia microspora* (Arnold) Hue, *Aspicilia subcaesiocinerea* Werner, *Lecanora cheresina* Müll. Arg., *Lecanora justii* Servít

L – Subs.: cal-par – Alt.: 1–4 – Note: a Southern European and Mediterranean-montane, very characteristic but often overlooked species described from Egypt; it is found on calcareous rocks, starting the life-cycle on *Aspicilia calcarea* and related species. The species is chemically variable, which led to the description of several varieties whose taxonomic value should be re-assessed on the basis of molecular data. – **Fr**: AHP, HAl, AMa, Drô, Isè, Var, Vau. **It**: Lig.

***Lobothallia controversa* Cl. Roux & A. Nordin**

Syn.: *Aspicilia calcarea* (L.) Mudd var. *farinosa auct. non* (Flörke) Hazsl., *Aspicilia farinosa auct. non* (Flörke) Arnold, *Lecanora farinosa* (*auct. non* Flörke) Nyl., *Pachyospora calcarea* A. Massal. var. *farinosa auct. non* (Flörke) A. Massal., *Pachyospora farinosa auct. non* (Flörke) A. Massal., *Urceolaria calcarea auct. non* (L.) Ach. var. farinosa Flörke

L – Subs.: cal – Alt.: 2–5 – Note: a mainly southern species in Europe, found on hard rocks (pure limestone or dolomite), with optimum in the submediterranean and montane belts. The nomenclature has a complicated history, because Flörke used the epithet “*farinosa*” for *A. calcarea*: some old records from the Alps could refer to that species. – **Au**: T, St, N. **Sw**: VS. **Fr**: AHP, HAl, AMa, Drô, Isè, Sav, HSav, Var, Vau. **It**: Frl, Ven, TAA, Lomb, Lig. **Sl**: SlA.

***Lobothallia melanaspis* (Ach.) Hafellner**

Syn.: *Aspicilia melanaspis* (Ach.) Poelt & Leuckert, *Lecanora melanaspis* (Ach.) Ach., *Parmelia melanaspis* Ach.

L – Subs.: sil-aqu – Alt.: 3–5 – Note: a mainly boreal-montane species found on acid to slightly calciferous rocks in the inundation zone on shores of lakes, brooks, and streams, mostly in upland areas; to be looked for throughout the Alps. – **Au**: T, S, K, St. **Sw**: GR, VS. **Fr**: AHP, AMa. **It**: TAA, Lomb, Piem.

***Lobothallia parasitica* (B. de Lesd.) ined. (provisionally placed here, ICN Art. 36.1b)**

Syn.: *Aspicilia parasitica* B. de Lesd., *Lecanora parasitica* (B. de Lesd.) Zahlbr.

L # – Subs.: sil-par – Alt.: 1–3 – Note: a mainly Mediterranean lichen found on steeply inclined, sunny faces of siliceous rocks at relatively low elevations, differing from *L. radiosa* in the parasitic habit and in the presence of stictic acid; apparently more frequent in the Southern and Western Alps. – **Fr**: AMa. **It**: Frl, Lomb, Piem.

***Lobothallia praeradiosa* (Nyl.) Hafellner**

Syn.: *Aspicilia praeradiosa* (Nyl.) Poelt & Leukert, *Lecanora praeradiosa* Nyl.

L – Subs.: cal, int – Alt.: 2–4 – Note: on basic siliceous rocks, especially calciferous schists; mostly restricted to dry-warm valleys of the Alps. – **Au**: T, St, N, B. **Sw**: VS. **Fr**: Sav. **It**: TAA, VA.

***Lobothallia radiosa* (Hoffm.) Hafellner**

Syn.: *Aspicilia radiosa* (Hoffm.) Poelt & Leuckert, *Aspicilia subcircinata* (Nyl.) Coppins, *Lecanora circinata* (Pers.) Ach., *Lecanora radiosa* (Hoffm.) Schaer., *Lecanora subcircinata* Nyl., *Lichen radiosus* Hoffm., *Placodium circinatum* (Pers.) Gray, *Placodium radiosum* (Hoffm.) DC., *Placodium subcircinatum* (Nyl.) Arnold, *Psoroma circinatum* (Pers.) Flagey, *Squamaria circinata* (Pers.) Anzi, *Squamaria subcircinata* (Nyl.) H. Olivier

L – Subs.: sil, cal – Alt.: 1–5 – Note: a widespread holarctic lichen with a very wide altitudinal and latitudinal range, and with correspondingly broad ecological requirements, found on a wide variety of substrata, including basic siliceous rocks, limestone, dolomite, more rarely brick, roofing tiles and mortar; common throughout the Alps. The species, in its present circumscription, is chemically variable: the forms with norstictic acid, corresponding to *Aspicilia subcircinata*, may represent just a chemotype. – **Au**: V, T, S, K, St, N, B. **Ge**: Schw. **Sw**: BE, GR, LU, SZ, UW, VD, VS. **Fr**: AHP, HAl, AMa, Drô, Isè, Sav, HSav, Var, Vau. **It**: Frl, Ven, TAA, Lomb, Piem, VA, Lig. **Sl**: SlA.

***Lobothallia recedens* (Taylor) A. Nordin, Savić & Tibell**

Syn.: *Aspicilia bohemica* Körb., *Aspicilia recedens* (Taylor) Arnold, *Lecanora bohemica* (Körb.) H. Magn., *Lecanora griseola* Th. Fr., *Lecanora recedens* (Taylor) Nyl., *Lecanora subcinerea* Nyl., *Lecidea recedens* Taylor

L – Subs.: sil – Alt.: 2–4 – Note: a lichen ranging from the boreal zone to the Mediterranean mountains, found on periodically wetted but rapidly drying siliceous rocks in upland areas. It is ecologically similar to *Lasallia pustulata*, and is probably more widespread in the Alps. – **Au**: V, T, S, K, N. **Ge**: Ge. **Sw**: GR. **Fr**: AMa. **It**: Ven, TAA, Piem, VA.

***Lopadium disciforme* (Flot.) Kullh.**

Syn.: Heterothecium pezizoideum (Ach.) Stizenb. var. disciforme Flot., Lopadium pezizoideum (Ach.) Körb. var. disciforme (Flot.) Körb., Sporopodium pezizoideum (Ach.) Vain. var. disciforme (Flot.) Vain.

L – Subs.: cor – Alt.: 2–3 – Note: on bark and epiphytic bryophytes on old *Picea* and other conifers, rarely on deciduous trees, especially *Quercus*, in cold-humid forests; perhaps overlooked in the Alps, but certainly not common. – **Au**: T, S, K, St, O, N. **Ge**: OB, Schw. **Sw**: UW. **It**: Frl, Ven, TAA. **Sl**: SlA.

***Lopadium pezizoideum* (Ach.) Körb.**

Syn.: *Lecidea pezizoidea* Ach., *Lopadium muscicola* (Sommerf.) Körb., Lopadium pezizoideum (Ach.) Körb. var. muscicola (Sommerf.) Th. Fr., Sporopodium pezizoideum (Ach.) Vain. var. muscicola (Sommerf.) Vain.

L – Subs.: deb, bry, ter-sil – Alt.: 3–5 – Note: a circumboreal-montane lichen found on bryophytes and plant debris over siliceous rocks, with optimum above treeline. – **Au**: T, S, K, St. **Ge**: OB, Schw. **Sw**: BE, GR, UR, UW, VD, VS. **Fr**: HSav. **It**: Frl, Ven, TAA, Lomb, Piem.

***Loxospora cismonica* (Beltr.) Hafellner**

Syn.: *Haematomma cismonicum* Beltr.

L – Subs.: cor – Alt.: 2–3 – Note: a cool-temperate, suboceanic lichen found on mature trees (mostly *Abies*) in humid, old forests, mostly in the montane belt. – **Au**: V, T, S, K, St, O, N. **Ge**: OB, Schw. **Sw**: BE, GR, SZ, UW, VD, VS. **It**: Frl, Ven. **Sl**: SlA.

***Loxospora elatina* (Ach.) A. Massal.**

Syn.: *Haematomma elatinum* (Ach.) A. Massal., ?*Lecanora chloropolia* (Erichsen) Almb., *Lecanora elatina* Ach., ?*Pertusaria chloropolia* Erichsen

L – Subs.: cor – Alt.: 2–4 – Note: an epiphytic species found on *Abies* and *Picea*, more rarely on deciduous trees (*e.g. Betula*); certainly overlooked in the Alps, being most often sterile, but never common. – **Au**: V, T, S, K, St, O, N. **Ge**: OB, Schw. **Sw**: BE, GL, GR, LU, SG, SZ, TI, UR, UW, VD, VS. **Fr**: Isè. **It**: Frl, Ven, TAA, Lomb. **Sl**: SlA, Tg.

***Maronea constans* (Nyl.) Hepp**

Syn.: *Acarospora constans* (Nyl.) H. Olivier, *Lecanora constans* Nyl., *Maronea berica* A. Massal.

L – Subs.: cor – Alt.: 2–3 – Note: a mild-temperate lichen found on smooth bark, especially on twigs of deciduous, more rarely coniferous trees; probably more frequent in the past. – **Au**: V, T, S, K, St, O. **Ge**: Ge. **Fr**: AHP, AMa, Var, Vau. **It**: Ven, Lomb. **Sl**: SlA.

***Maronella laricina* M. Steiner**

L – Subs.: cor – Alt.: 3 – Note: a species with an inconspicuous thallus, abundant and minute, rusty-brown, lecanorine apothecia reacting K+ purple, and polyspored asci with an amyloid tholus; on eutrophicated bark of solitary trees (*e.g. Larix*) next to manured meadows or along roadsides; apparently a rare species, so far only reported from Austria and Spain, with a few records from the the Eastern Alps. – **Au**: T.

***Massalongia carnosa* (Dicks.) Körb.**

Syn.: *Biatora carnosa* (Dicks.) Rabenh., *Lecanora muscorum* Ach. *nom. superfl.*, *Lichen carnosus* Dicks., *Pannaria muscorum* Delise *ex* Duby, *Pannularia muscorum* Nyl. *ex* Lamy *nom.illeg.*

L – Subs.: bry, sil, ter, bry-sil – Alt.: 3–5 – Note: a circumpolar, arctic-alpine to boreal-montane lichen found on bryophytes and soil rich in humus, on steeply inclined or underhanging faces near the ground, with optimum above or near treeline in areas with siliceous substrata; widespread throughout the Alps. – **Au**: V, T, S, K, St. **Ge**: OB. **Sw**: GR, LU, TI, UR, UW, VS. **Fr**: AHP, AMa, Isè, HSav. **It**: Frl, TAA, Lomb, Piem, VA, Lig.

***Megalaria grossa* (Pers. *ex* Nyl.) Hafellner**

Syn.: *Biatorina premnea* (Fr.) A.L. Sm., *Buellia premnea* (Fr.) Kickx, *Catillaria grossa* (Pers. *ex* Nyl.) Körb., *Catillaria leucoplaca auct.*, *Catillaria premnea* (Fr.) Körb., *Catinaria grossa* (Pers. *ex* Nyl.) Vain., *Catinaria leucoplaca auct.*, *Lecidea grossa* Pers. *ex* Nyl., *Lecidea premnea* Fr.

L – Subs.: cor – Alt.: 2–3 – Note: a mild-temperate to humid subtropical lichen found on base-rich bark of deciduous trees, especially of *Acer* and *Fraxinus*; very rare and certainly declining in the Alps. – **Au**: O. **Ge**: OB. **Fr**: AHP, AMa, Var, Vau. **It**: Ven, Lomb. **Sl**: SlA, Tg.

***Megalaria laureri* (Hepp *ex* Th. Fr.) Hafellner**

Syn.: *Catillaria intermixta auct. non* (Nyl.) Arnold *ex* Głow., *Catillaria laureri* Hepp *ex* Th. Fr., *Catinaria intermixta auct. non* (Nyl.) P. James, *Catinaria laureri* (Hepp *ex* Th. Fr.) Degel., *Gyalecta livida* (Mudd) Zahlbr., *Phialopsis livida* Mudd

L – Subs.: cor – Alt.: 2–3 – Note: a mild-temperate lichen found on the bark of *Quercus* and *Fagus*, more rarely *Abies* in humid forests; very rare and certainly declining in the Alps. – **Au**: S, K, St. **Fr**: AMa, Var. **It**: Frl, Ven, Piem. **Sl**: SlA, Tg.

***Megalaria pulverea* (Borrer) Hafellner & E. Schreiner**

Syn.: *Biatorina pulverea* (Borrer) Mudd, *Catillaria incana* (Delise *ex* Nyl.) H. Olivier, *Catillaria pulverea* (Borrer) Lettau, *Catillochroma pulverea* (Borrer) Kalb, *Catinaria pulverea* (Borrer) Vězda & Poelt, *Lecidea pulverea* Borrer, *Pertusaria miniescens* Erichsen

L – Subs.: cor – Alt.: 2–3 – Note: a cool-temperate lichen found on bark and mossy trunks of deciduous trees and *Abies* in old, humid, montane woodlands. The ascus of *Biatora*-type and other anatomical characters do not support the inclusion of this species into *Megalaria* *s.str.* – **Au**: V, T, S, K, St, O, N. **Ge**: OB. **Sw**: BE, GL, GR, SZ, UR, UW, VD, VS. **It**: TAA. **Sl**: SlA.

***Megalospora pachycarpa* (Delise *ex* Duby) H. Olivier**

Syn.: *Biatora pachycarpa* (Delise *ex* Duby) Fr., *Bilimbia pachycarpa* (Delise *ex* Duby) Boistel, *Bombyliospora incana* A.L. Sm., *Bombyliospora pachycarpa* (Delise *ex* Duby) A. Massal., *Megalospora tuberculosa auct. p.p. non* (Fée) Sipman, *Patellaria pachycarpa* Delise *ex* Duby

L # – Subs.: cor, bry – Alt.: 2–3 – Note: we treat *M. tuberculosa* as circumscribed by Sipman, *i.e.* as an aggregate of taxa, one of which, *M. pachycarpa*, occurs in Europe and Macaronesia and has a peculiar combination of characters (thallus thick, sorediate but without tubercules, soralia often coalescing, containing usnic acid and zeorin, apothecia brown and with an orange-brown epihymenium). It grows on bark of deciduous trees in humid montane forests under oceanic climatic conditions; in the Alps it is therefore most frequent in the outer mountain ranges. – **Au**: V, T, S, St, O, N. **Ge**: OB, Schw. **Sw**: UW. **It**: Frl. **Sl**: Tg.

***Melanelia agnata* (Nyl.) A. Thell**

Syn.: *Cetraria agnata* (Nyl.) Kristinsson, *Platysma agnatum* Nyl.

L – Subs.: ter-sil, sil – Alt.: 4–5 – Note: a rather neglected, arctic-alpine, circumpolar species growing on acid siliceous rocks near and above treeline. The type, collected by Arnold, is from the surroundings of Brenner Pass in South Tyrol. – **Au**: T. **Sw**: GR, UR. **Fr**: AMa. **It**: TAA.

***Melanelia hepatizon* (Ach.) A. Thell**

Syn.: *Cetraria hepatizon* (Ach.) Vain., *Cetraria polyschiza* (Nyl.) Jatta, *Lichen hepatizon* Ach., *Parmelia baumgartneri* Zahlbr., *Platysma hepatizon* (Ach.) Vain., *Platysma polyschizum* Nyl., *Tuckermannopsis hepatizon* (Ach.) Kurok.

L – Subs.: sil – Alt.: 3–6 – Note: a circumpolar, arctic-alpine lichen found on hard siliceous rocks wetted by rain, with optimum above treeline; somehow less bound to cold-humid sites than the similar but unrelated *Cetrariella commixta*; widespread throughout the siliceous Alps. – **Au**: V, T, S, K, St, N. **Ge**: Ge. **Sw**: BE, GR, TI, UR, VS. **Fr**: HAl, Isè, Sav, HSav. **It**: Frl, Ven, TAA, Lomb, Piem, VA.

***Melanelia stygia* (L.) Essl.**

Syn.: *Cetraria stygia* (L.) Schaer., *Cornicularia stygia* (L.) Nyl., *Imbricaria stygia* (L.) DC., *Lichen fahlunensis* L., *Lichen stygius* L., *Parmelia reagens* (Servít) Gyeln., *Parmelia stygia* (L.) Ach.

L – Subs.: sil – Alt.: 3–6 – Note: a circumpolar, arctic-alpine lichen found on siliceous rocks in open habitats, with optimum near and above treeline; widespread throughout the Alps, where it reaches the nival belt. – **Au**: V, T, S, K, St, N. **Sw**: BE, GR, TI, UR, VD, VS. **Fr**: AHP, HAl, AMa, Isè, Sav, HSav. **It**: Frl, Ven, TAA, Lomb, Piem, VA.

***Melanelixia fuliginosa* (Fr. *ex* Duby) O. Blanco, A. Crespo, Divakar, Essl., D. Hawksw. & Lumbsch**

Syn.: *Melanelia fuliginosa* (Fr. *ex* Duby) Essl., *Parmelia fuliginosa* (Fr. *ex* Duby) Nyl. *non* (Ach.) Schaer., Parmelia glabratula (Lamy) Nyl. subsp. fuliginosa (Fr. *ex* Duby) J.R. Laundon, Parmelia glabratula (Lamy) Nyl. var. fuliginosa (Fr. *ex* Duby) Grummann, Parmelia olivacea (L.) Ach. var. fuliginosa Fr. *ex* Duby

L – Subs.: sil, cor – Alt.: 2–4 – Note: a mainly silicicolous species; widespread throughout the Alps. See also note on *M. glabratula*. – **Au**: V, T, S, K, St, O, N, B. **Ge**: OB. **Sw**: BE, GR, SG, VD, VS. **Fr**: HAl, AMa, Isè, Sav, HSav, Var. **It**: Ven, TAA, Lomb, Piem, VA.

***Melanelixia glabra* (Schaer.) O. Blanco, A. Crespo, Divakar, Essl., D. Hawksw. & Lumbsch**

Syn.: *Melanelia glabra* (Schaer.) Essl., *Parmelia glabra* (Schaer.) Nyl., Parmelia olivacea (L.) Ach. var. corticola Th. Fr. f. glabra Schaer.

L – Subs.: cor, xyl – Alt.: 1–4 – Note: a mild-temperate lichen found on more or less isolated, mostly deciduous trees, with optimum in the submediterranean belt, but common also in the beech belt; ecologically similar to *Pleurosticta acetabulum*; widespread throughout the Alps. – **Au**: V, T, S, K, St, O, N. **Ge**: OB, Schw. **Sw**: AP, BE, FR, GL, GR, LU, SG, SZ, TI, UR, UW, VD, VS. **Fr**: AHP, HAl, AMa, Drô, Isè, Sav, HSav, Var, Vau. **It**: Frl, Ven, TAA, Lomb, Piem, VA, Lig. **Sl**: SlA, Tg. **Li**.

***Melanelixia glabratula* (Lamy) Sandler & Arup**

Syn.: *Melanelia fuliginosa* (Fr. *ex* Duby) Essl. subsp. glabratula (Lamy) Coppins, *Melanelia glabratula* (Lamy) Essl., *Parmelia fuliginosa* (Fr. *ex* Duby) Nyl. subsp. glabratula Lamy, *Parmelia fuliginosa* (Fr. *ex* Duby) Nyl. var. laetevirens (Flot. *ex* Körb.) Nyl., *Parmelia glabratula* (Lamy) Nyl., *Parmelia laetevirens* (Flot. *ex* Körb.) F. Rosend.

L – Subs.: cor, sil, int, xyl – Alt.: 1–4 – Note: a mainly temperate, ecologically wide-ranging species occurring both on wayside trees and in open forests (*e.g.* on *Fagus*). The greatest majority of the epiphytic records of *M. fuliginosa* refer to this species; widespread throughout the Alps. – **Au**: V, T, S, K, St, O, N, B. **Ge**: OB. **Sw**: AP, BE, FR, GL, GR, LU, SG, SZ, TI, UR, UW, VD, VS. **Fr**: AHP, HAl, AMa, Drô, Isè, Sav, HSav, Var, Vau. **It**: Frl, Ven, TAA, Lomb, Piem, VA, Lig. **Sl**: SlA, Tg. **Li**.

***Melanelixia subargentifera* (Nyl.) O. Blanco, A. Crespo, Divakar, Essl., D. Hawksw. & Lumbsch**

Syn.: *Melanelia subargentifera* (Nyl.) Essl., *Parmelia conspurcata* (Schaer.) Vain., *Parmelia sorediomanes* (Nyl.) Gyeln., *Parmelia subargentifera* Nyl.

L – Subs.: cor, bry, sil – Alt.: 2–4 – Note: a temperate lichen of areas with a continental climate found on the bark of isolated deciduous trees; widespread throughout the Alps, but most common in dry-warm valleys. – **Au**: V, T, S, K, St, O, N, B. **Ge**: OB. **Sw**: AP, BE, FR, GL, GR, LU, SG, SZ, TI, UR, UW, VD, VS. **Fr**: AHP, HAl, AMa, Drô, Isè, HSav, Var, Vau. **It**: Frl, Ven, TAA, Lomb, Piem, VA, Lig. **Sl**: SlA, Tg. **Li**.

***Melanelixia subaurifera* (Nyl.) O. Blanco, A. Crespo, Divakar, Essl., D. Hawksw. & Lumbsch**

Syn.: *Melanelia subaurifera* (Nyl.) Essl., *Parmelia protaurifera* Gyeln., *Parmelia subaurifera* Nyl.

L – Subs.: cor, sil – Alt.: 1–4 – Note: a mainly temperate, pioneer species of smooth bark, *e.g.* on twigs of shrubs and trees, but also on boles of oaks in open woodlands and parklands; common throughout the Alps, with optimum in the submediterranean belt. – **Au**: V, T, S, K, St, O, N, B. **Ge**: OB. **Sw**: AP, BE, FR, GL, GR, LU, SG, SZ, TI, UR, UW, VD, VS. **Fr**: AHP, HAl, AMa, Drô, Isè, Sav, HSav, Var, Vau. **It**: Frl, Ven, TAA, Lomb, Piem, VA, Lig. **Sl**: SlA, Tg.

***Melanohalea elegantula* (Zahlbr.) O. Blanco, A. Crespo, Divakar, Essl., D. Hawksw. & Lumbsch**

Syn.: *Collema exasperatum* Ach., *Melanelia elegantula* (Zahlbr.) Essl., *Melanelia incolorata* (Parrique) Essl., Parmelia aspidota (Ach.) Poetsch var. elegantula Zahlbr., *Parmelia elegantula* (Zahlbr.) Szatala, Parmelia exasperatula Nyl. var. elegantula (Zahlbr.) Zahlbr., *Parmelia incolorata* (Parrique) Lettau

L – Subs.: cor, sil – Alt.: 2–4 – Note: a mild-temperate lichen found on old trees (*e.g.* oaks, *Castanea*), more rarely on siliceous rocks, with optimum in the montane belt; widespread throughout the Alps. – **Au**: V, T, S, K, St, O, N, B. **Ge**: OB. **Sw**: BE, GR, LU, SG, SZ, TI, VS. **Fr**: AHP, HAl, AMa, Isè, Sav, Vau. **It**: Frl, Ven, TAA, Lomb, Piem, VA, Lig. **Sl**: SlA.

***Melanohalea exasperata* (De Not.) O. Blanco, A. Crespo, Divakar, Essl., D. Hawksw. & Lumbsch**

Syn.: *Imbricaria aspera* (A. Massal.) Körb., *Imbricaria aspidota* (Ach.) Arnold, *Melanelia exasperata* (De Not.) Essl., *Parmelia aspera* A. Massal., *Parmelia aspidota* (Ach.) Poetsch, Parmelia aspidota (Ach.) Poetsch var. exasperata (De Not.) Syd., *Parmelia exasperata* De Not.

L – Subs.: cor – Alt.: 1–3 – Note: a mainly temperate to Mediterranean early coloniser of smooth bark, most common on twigs of shrubs and deciduous trees (*e.g. Prunus*, *Quercus*) below the subalpine belt; widespread throughout the Alps. – **Au**: V, T, S, K, St, O, N. **Ge**: OB. **Sw**: BE, GL, GR, LU, SZ, TI, UR, VD, VS. **Fr**: AHP, HAl, AMa, Drô, Isè, Sav, HSav, Var, Vau. **It**: Frl, Ven, TAA, Lomb, Piem, Lig. **Sl**: SlA, Tg. **Li**.

***Melanohalea exasperatula* (Nyl.) O. Blanco, A. Crespo, Divakar, Essl., D. Hawksw. & Lumbsch**

Syn.: *Melanelia exasperatula* (Nyl.) Essl., Parmelia aspidota (Ach.) Röhl. var. exasperatula (Nyl.) Syd., *Parmelia exasperatula* Nyl., *Parmelia papulosa* (Anzi) Vain.

L – Subs.: cor, xyl, sil – Alt.: 2–5 – Note: a cool-temperate to boreal-montane, circumpolar lichen found on isolated trees, especially on twigs and sometimes even on conifer needles, in *Xanthorion*-communities; especially common on twigs of *Larix* throughout the Alps. – **Au**: V, T, S, K, St, O, N, B. **Ge**: OB, Schw. **Sw**: AP, BE, FR, GL, GR, LU, SG, SZ, TI, UR, UW, VD, VS. **Fr**: AHP, HAl, AMa, Drô, Isè, Sav, HSav, Var, Vau. **It**: Frl, Ven, TAA, Lomb, Piem, VA, Lig. **Sl**: SlA, Tg. **Li**.

***Melanohalea infumata sensu auct. medioeur* . *non* (Nyl.) O. Blanco, A. Crespo, Divakar, Essl., D. Hawksw. & Lumbsch**

Syn.: *Melanelia infumata auct. non* (Nyl.) Essl., *Parmelia elegantula* (Zahlbr.) Szatala subsp. *infumata auct. non* (Nyl.) Clauzade & Cl. Roux, *Parmelia infumata auct. non* Nyl.

L # – Subs.: sil, xyl – Alt.: 4–6 – Note: *M. infumata* *s.str.*, a circumarctic-boreal species, is likely to be absent from the Alps, where it has been frequently confused with a saxicolous strain of the *M. elegantula*-aggregate, which deserves further study. – **Au**: ?V, T, S, K, N. **Sw**: GR, VS.

***Melanohalea laciniatula* (Flagey *ex* H. Olivier) O. Blanco, A. Crespo, Divakar, Essl., D. Hawksw. & Lumbsch**

Syn.: *Melanelia laciniatula* (Flagey *ex* H. Olivier) Essl., Parmelia exasperatula Nyl. var. laciniatula Flagey *ex* H. Olivier, *Parmelia laciniatula* (Flagey *ex* H. Olivier) Zahlbr., *Parmelia laevigatula* (Nyl.) Parrique

L – Subs.: cor, sax, xyl – Alt.: 2–3 – Note: a mainly Mediterranean-montane species found on the smooth bark of old deciduous trees, especially *Fagus*, in open, humid, mostly montane forests; most abundant in the Mediterranean mountains; widespread, but perhaps declining in the Alps. – **Au**: V, St, O, N. **Sw**: GR, VD. **Fr**: AHP, AMa, Vau. **It**: Frl, Ven, Piem, Lig. **Sl**: SlA, Tg.

***Melanohalea septentrionalis* (Lynge) O. Blanco, A. Crespo, Divakar, Essl., D. Hawksw. & Lumbsch**

Syn.: *Melanelia septentrionalis* (Lynge) Essl., Parmelia olivacea (L.) Ach. var. septentrionalis Lynge, *Parmelia septentrionalis* (Lynge) Ahti

L – Subs.: cor – Alt.: 3–5 – Note: a species resembling *M. olivacea*, but thalli smaller, with marginal apothecia; on the bark of various trees (mostly *Alnus*, *Salix* and *Betula*), usually on the branches; widespread in the Holarctic region, more common in Scandinavia, in Central Europe it occurs in the montane to alpine belts, mostly in raised bogs, rarely on dwarf shrubs above treeline, with a few records from the Eastern Alps (Austria). – **Au**: T, S, K, St.

***Melanolecia transitoria* (Arnold) Hertel**

Syn.: *Lecidea henricii* Zahlbr., *Lecidea subcaerulescens* Arnold, *Lecidea transitoria* Arnold, Lecidea transitoria Arnold var. subcaerulescens (Arnold) Arnold, *Tremolecia transitoria* (Arnold) Hertel

L – Subs.: cal – Alt.: 4–6 – Note: a circumpolar, arctic-alpine lichen found on inclined to underhanging surfaces of calcareous rocks above treeline. – **Au**: ?V, T, K, St, O. **Ge**: OB. **Sw**: GR. **Fr**: AHP, HAl, AMa, Sav, Vau. **It**: Ven, TAA, Piem.

***Melaspilea enteroleuca* (Ach.) Ertz & Diederich**

Syn.: *Abrothallus ricasolii* A. Massal., *Buellia ricasolii* (A. Massal.) A. Massal., *Catillaria ricasolii* (A. Massal.) A. Massal., *Lecidea enteroleuca* Ach. *non* Nyl. *nec auct.*, *Lecidea sparsa* Dufour, *Melaspilea arthonioides auct. eur*. *non* (A. Massal.) Nyl., *Melaspilea urceolata auct. eur. non* (Fr.) Almb. *ex* Ertz & Diederich

L – Subs.: cor – Alt.: 1–2 – Note: a mild-temperate species found on hard bark of deciduous trees (*Quercus*, *Morus*, etc.); more widespread in the past, presently declining. We have placed here all records of *M. urceolata* and *M. arthonioides*, which are two different, American species. – **Fr**: AHP, AMa, HSav, Var, Vau. **It**: Frl, Ven, TAA, Lomb, Lig.

***Melaspilea poetarum* (De Not. & Bagl.) Nyl.**

Syn.: *Opegrapha poetarum* De Not. & Bagl.

L – Subs.: cor – Alt.: 1–2 – Note: a mild-temperate species found on more or less smooth bark, especially of *Fraxinus ornus*, with a single record from a very rainy area of the Eastern Alps (Italy). – **It**: Frl.

***Melaspilea tyroliensis* Szatala**

L # – Subs.: sil – Alt.: 4–5 – Note: thallus thin, with white maculiform soralia, apothecia lirelliform, sessile, simple to trifurcate, to 1 mm long and 0.1–0.2 mm wide, with an entire excipulum. a 70–80 µm tall non-inspersed hymenium reacting I+ red, and 1-septate, first hyaline, then brownish ascospores clearly constricted in the middle (13–15 × 6–8 µm); known only from the type collection (Mt. Margola), on syenite – **It**: TAA.

***Menegazzia subsimilis* (H. Magn.) R. Sant.**

Syn.: *Menegazzia dissecta* (Rass.) Hafellner, Menegazzia pertusa (Schaer.) Stein var. dissecta (Rass.) Rass., Menegazzia terebrata (Hoffm.) A. Massal. var. dissecta (Rass.) Poelt, *Parmelia subsimilis* H. Magn.

L – Subs.: cor – Alt.: 2–3 – Note: on bark in humid beech-fir forests, often with *M. terebrata*; the material of *M. terebrata* from the Alps should be checked in search of this species. – **Au**: S, St, O, N. **Ge**: OB, Schw. **Sw**: BE, TI. **It**: Frl.

***Menegazzia terebrata* (Hoffm.) A. Massal.**

Syn.: *Imbricaria terebrata* (Hoffm.) Körb., *Lobaria terebrata* Hoffm., *Menegazzia pertusa* (Schaer.) Stein, *Parmelia diatrypa* (Ach.) Ach., *Parmelia pertusa* Schaer., *Parmelia terebrata* (Hoffm.) Mart., *Physcia diatrypa* (Ach.) Gray

L – Subs.: cor, bry-sil – Alt.: 2–4 – Note: on bark in humid beech-fir forests, exceptionally reaching the submediterranean belt; widespread throughout the Alps, but only locally common. See also note on *M. subsimilis.* – **Au**: V, T, S, K, St, O, N. **Ge**: OB. **Sw**: BE, GL, GR, LU, SG, SZ, TI, UR, UW, VD. **Fr**: HSav. **It**: Frl, Ven, TAA, Lomb, Piem. **Sl**: SlA, Tg.

***Metamelanea umbonata* Henssen**

L – Subs.: cal, int – Alt.: 3–4 – Note: in seepage tracks on steeply inclined, damp rock faces. – **Au**: S, O, N. **Sw**: SZ, UW.

***Micarea adnata* Coppins**

L – Subs.: xyl, cor – Alt.: 2–3 – Note: on rather decomposed lignum, such as on old oak stumps and associated decaying bryophyte mats, more rarely on loose bark of deciduous trees in areas with high rainfall, mostly in woodlands; certainly more widespread in the Alps. – **Au**: V, T, S, K, St, O, N. **Ge**: OB. **Sw**: BE, GR, UW, VD. **It**: Piem. **Sl**: SlA.

***Micarea anterior* (Nyl.) Hedl.**

Syn.: *Lecidea anterior* Nyl.

L – Subs.: xyl – Alt.: 3–4 – Note: a species recalling a discoloured *M. misella*, with an endosubstratic (mostly endoxylic) thallus, brown apothecia (rarely present), their pigment not reacting with K or C, oblong to ovoid, usually simple to 1-septate ascospores, sessile to stalked pycnidia (often present) with a pale to brownish base and a brown wall, and oblong mesoconidia; on decorticated wood in coniferous forests; widespread in Europe but rather rare, with a single record from the Western Alps (Switzerland). – **Sw**: SZ.

***Micarea assimilata* (Nyl.) Coppins**

Syn.: *Lecidea assimilata* Nyl., *Lecidella assimilata* (Nyl.) Arnold

L – Subs.: deb, bry, ter-sil – Alt.: 4–5 – Note: a frequently misidentified species resembling *M. incrassata*, but with a white to brownish thallus of convex to verrucose areolae, often with inconspicuous brown cephalodia inbetween the areolae, numerous, convex, immarginate, black, rather large apothecia (0.3–0.8 mm in diam.) with a green to olivaceous epihymenium and a purple-brown hypothecium, a reflexed exciple of radiating hyphae, and mainly simple, oblong-ellipsoid to fusiform ascospores (12–16 × 3–5 μm); on plant debris and bryophytes; distribution mainly arctic to boreal montane in Europe with a southern outpost in Scotland; all records from the Alps need critical re-evaluation. – **Au**: ?V, ?T, ?S, ?K, ?N. **Ge**: ?OB. **Sw**: ?GR. **Fr**: ?HAl.

***Micarea botryoides* (Nyl.) Coppins**

Syn.: Lecidea apochroeella Nyl. var. botryoides Nyl., *Lecidea botryoides* (Nyl.) Zahlbr.

L – Subs.: bry, sil, ter-sil, xyl – Alt.: 2–4 – Note: on a wide variety of substrata including soil, bryophytes, moribund plants, siliceous rocks, and conifer bark, mostly on vertical or underhanging faces; certainly much overlooked, but never common in the Alps. – **Au**: ?V, S, K, St. **Sw**: GL, GR, VS. **It**: TAA. **Sl**: SlA.

**Micarea cinerea (Schaer.) Hedl. f. cinerea**

Syn.: *Bacidia cinerea* (Schaer.) Trevis., *Biatora delicatula* Körb., *Bilimbia cinerea* (Schaer.) Körb., *Bilimbia delicatula* (Körb.) Körb., *Lecidea cinerea* Schaer., Lecidea sphaeroides (Dicks.) Röhl. var. albella Schaer.

L – Subs.: cor, xyl – Alt.: 2–4 – Note: a cool-temperate to probably circumboreal-montane species found on bark of deciduous and coniferous trees and on epiphytic bryophytes in humid, montane to subalpine forests, more rarely on lignum of fallen, decorticated trunks; widespread throughout the Alps. – **Au**: V, T, S, K, St, O, N. **Ge**: OB. **Sw**: BE, GR, LU, SG, SZ, TI, UW. **Fr**: HSav. **It**: Frl, TAA, Lomb, Piem, Lig. **Sl**: SlA.

**Micarea cinerea (Schaer.) Hedl. f. tenuispora (D. Hawksw. & Poelt) Fryday**

Syn.: *Hastifera tenuispora* D. Hawksw. & Poelt

L – Subs.: ter-sil-par – Alt.: 5 – Note: this is the anamorph of *M. cinerea*. – **Au**: K, St.

***Micarea contexta* Hedl.**

Syn.: *Catillaria contexta* (Hedl.) Zahlbr.

L – Subs.: xyl – Alt.: 3–4 – Note: mostly on wood in sheltered situations, such as in montane to subalpine woodlands; perhaps more widespread in the Alps. – **Au**: Au. **Sw**: SZ. **It**: Frl.

***Micarea coppinsii* Tønsberg**

L – Subs.: cor – Alt.: 2–3 – Note: a species resembling *M. peliocarpa*, but the areolate thallus with flat to capitate, green soralia, and often somewhat stipitate apothecia; on bark of various trees and on branches of dwarf shrubs in sites with an oceanic climate, therefore most common in Western Europe, with a few records from the Eastern and Central Alps. – **Au**: T, K, O, N. **Ge**: OB. **Sw**: BE, LU, SZ.

***Micarea cyanescens* Poelt & Döbbeler**

L – Subs.: bry – Alt.: 3–4 – Note: a species with an inconspicuous thallus and minute apothecia which are whitish when dry, translucent and gelatinous when moist, reacting intensely blue with iodine under the dissecting microscope, with narrowly ellipsoid, one-septate ascospores; on moribund mats of *Campylium halleri* overgrowing calcareous rocks; rare, but perhaps overlooked; known from a few localities from the montane to the subalpine belt in the Eastern Alps. – **Au**: T, St. **Ge**: OB.

***Micarea deminuta* Coppins**

L – Subs.: xyl – Alt.: 3 – Note: a species resembling *M. contexta*, with a thin, effuse whitish to greenish-grey thallus and minute, black, convex to subglobose apothecia (0.1–0.2 mm in diam.) lacking an exciple, with a brownish epihymenium and a dark red-brown hypothecium, simple, ellipsoid to oblong-ellipsoid ascospores (7–11 × 3.5–5 μm), and inconspicuous pycnidia containing bacilliform conidia; mostly on logs and rotting stumps; widespread in Europe, but not common, with a few records from the Eastern Alps (Austria). – **Au**: K.

***Micarea denigrata* (Fr.) Hedl.**

Syn.: *Biatora aniptiza* (Stirt.) Walt. Watson, *Biatora denigrata* Fr., *Biatorina praeviridans* (Nyl.) Boistel, *Biatorina sinothea auct.*, *Catillaria denigrata* (Fr.) Vain., *Catillaria hemipoliella* (Nyl.) Blomb. & Forssell, *Catillaria praeviridans* (Nyl.) Zahlbr., *Catillaria spodiza* (Nyl.) Zahlbr., *Catillaria synothea auct. non* Ach., *Lecidea aniptiza* Stirt., *Lecidea denigrata* (Fr.) Nyl., *Lecidea discretula* Nyl., *Lecidea hemipoliella* Nyl., *Lecidea parissima* Nyl., *Lecidea praeviridans* Nyl., *Lecidea spodiza* Nyl., *Lecidea synothea auct.*, *Micarea andesitica* Vězda, *Micarea hemipoliella* (Nyl.) Vězda

L – Subs.: xyl, cor – Alt.: 2–4 – Note: a cool-temperate to circumboreal-montane, very polymorphic species, most common on wooden poles in the mountains, on fallen trunks and stumps of coniferous and broad-leaved trees, rarer on the bark of conifers; widespread throughout the Alps. – **Au**: V, T, S, K, St, O, N, B. **Ge**: OB. **Sw**: BE, GR, LU, SZ, TI, UR, VD, VS. **Fr**: AMa, Isè, HSav, Var. **It**: Ven, TAA, Lomb, Piem, Lig. **Sl**: SlA.

***Micarea elachista* (Körb.) Coppins & R. Sant.**

Syn.: *Bacidia sororians* (Nyl.) H. Olivier, *Biatora elachista* Körb., *Biatorina glomerella* (Nyl.) Arnold, *Catillaria elachista* (Körb.) Vain., *Catillaria glomerella* (Nyl.) Th. Fr., *Lecidea poliococca* Nyl., *Lecidea sororians* Nyl., *Micarea glomerella* (Nyl.) Hedl.

L – Subs.: xyl, cor – Alt.: 2–3 – Note: a cool-temperate to circumboreal-montane species with optimum on lignum, more rarely on acid bark, in *Castanea*-forests, often with *Chaenotheca ferruginea*; certainly more widespread in the Alps, but much overlooked. – **Au**: T, S, St, O. **Ge**: OB. **Sw**: GR, SZ. **It**: TAA, Lomb.

***Micarea eximia* Hedl.**

Syn.: *Catillaria malmeana* Zahlbr.

L – Subs.: xyl – Alt.: 3–4 – Note: a species with an endoxylic thallus and black, subglobose to tuberculate apothecia with a blue-green epihymenium reacting N+ red, and oblong to ellipsoid, simple to 1-septate ascospores; pycnidia usually present, black, containing short bacilliform mesoconidia; on decorticated wood in coniferous forests; widespread in Europe, but altogether rare, and only recorded from the Western Alps (Switzerland). – **Sw**: SZ.

***Micarea globulosella* (Nyl.) Coppins**

Syn.: *Bacidia globulosella* (Nyl.) Zahlbr., *Lecidea globulosella* Nyl., *Micarea bacidiella* *sensu* Vězda & V. Wirth

L – Subs.: cor, xyl – Alt.: 2–4 – Note: a temperate to probably circumboreal-montane species found on bark of conifers and oaks in humid forests, more rarely on lignum; certainly more widespread in the Alps. – **Au**: V, S. **Ge**: Schw. **Sw**: BE, SZ. **Fr**: Var. **It**: Frl, Piem.

***Micarea hedlundii* Coppins**

L – Subs.: xyl, cor – Alt.: 3–4 – Note: a rather rare species growing on wood and rotting roots of conifers in montane to subalpine forests, ranging from the Alps to Northern Europe. – **Au**: T, S, K, St, O. **Ge**: OB. **Sw**: BE, SZ, UW. **It**: Ven, TAA. **Sl**: SlA.

***Micarea hylocomii* Poelt & Döbbeler**

L – Subs.: bry – Alt.: 3–4 – Note: a species with an inconspicuous thallus, minute, blackish apothecia, and narrowly ellipsoid to rod-shaped, 1-septate, often slightly curved ascospores; on bleached leaflets of moribund to dead *Hylocomium splendens* in montane coniferous forests; overall distribution boreal-montane; in the Alps known from a few localities only. – **Au**: T. **Sw**: GR.

***Micarea incrassata* Hedl.**

L – Subs.: bry, ter-sil – Alt.: 4–5 – Note: a widespread circumboreal species also known from the Southern Hemisphere, growing on acid soil in mountain heaths; apparently rare in the Alps, but perhaps overlooked. – **Au**: T, S, K. **Ge**: Ge. **It**: TAA.

***Micarea leprosula* (Th. Fr.) Coppins & A. Fletcher**

Syn.: *Bacidia leprosula* (Th. Fr.) Lettau, *Bilimbia leprosula* (Th. Fr.) H. Olivier, Bilimbia milliari a (Fr.) Körb. var. leprosula Th. Fr.

L – Subs.: bry, sil, bry-sil, deb – Alt.: 3–5 – Note: a circumboreal species with a thallus as in *M. submilliaria*, consisting of bluish-grey, convex to subglobose areoles easily breaking down to form yellowish-green sorediate patches, but reacting C+ and Pd+ red (argopsin, gyrophoric acid), apothecia often lacking, ascospores mostly 3-septate, less than 30 µm long; on twigs of shrubs; certainly more widespread in the Alps, but overlooked. – **Au**: V, T, S, K, St, O, N. **Ge**: OB. **Sw**: BE, LU, SZ.

**Micarea lignaria (Ach.) Hedl. var. lignaria**

Syn.: *Bacidia gomphillacea* (Nyl.) Zahlbr., *Bacidia granulans* *sensu* H. Magn., *Bacidia lignaria* (Ach.) Lettau, *Bacidia meizospora* (Nyl.) Zahlbr., *Bacidia milliaria* (Fr.) Sandst., *Bilimbia lignaria* (Ach.) A. Massal., *Bilimbia meizospora* (Nyl.) H. Olivier, *Bilimbia milliaria* (Fr.) Th. Fr., *Lecidea geomaea* Taylor, *Lecidea lignaria* Ach., *Lecidea meizospora* Nyl., *Lecidea milliaria* Fr., *Micarea gomphillacea* (Nyl.) Vězda

L – Subs.: cor, xyl, bry, deb – Alt.: 2–5 – Note: a temperate to boreal-montane species found on a wide variety of substrata such as plant remains, bark, and lignum, in humid situations; widespread throughout the Alps. – **Au**: V, T, S, K, St, O, N. **Ge**: OB, Schw. **Sw**: BE, GR, LU, SZ, TI, UR, UW, VD, VS. **Fr**: AHP, HAl, HSav. **It**: Frl, Ven, TAA, Lomb, Piem, Lig. **Sl**: SlA, Tg.

**Micarea lignaria (Ach.) Hedl. var. endoleuca (Leight.) Coppins**

Syn.: Lecidea milliaria Fr. var. endoleuca Leight.

L – Subs.: bry, xyl, cor – Alt.: 2–3 – Note: although sometimes sympatric with the typical variety, this taxon is restricted to very humid areas at lower altitudes; from the Alps there are only a few scattered records. – **Au**: N. **Ge**: Schw. **Sw**: SZ. **It**: TAA.

***Micarea lithinella* (Nyl.) Hedl.**

Syn.: *Lecidea lithinella* Nyl.

L – Subs.: sil – Alt.: 2–3 – Note: on compact siliceous rocks in rather sheltered situations; from the Alps there are a few scattered records. – **Au**: ?V, St, N. **Sw**: LU, SZ. **It**: TAA.

***Micarea lynceola* (Th. Fr.) Palice**

Syn.: *Lecidea lynceola* Th. Fr., *Micarea excipulata* Coppins

L – Subs.: sil – Alt.: 3 – Note: a species resembling *M. polycarpella* in the presence of a large-celled photobiont and the occurrence of a greenish pigment in apothecia and pycnidia, but the black apothecia are plane to subconvex, with a distinct exciple, and the ascospores are simple, ellipsoid to ovoid; usually on loose siliceous pebbles in pioneer communities; widespread in Europe but not common, perhaps often overlooked; in the study area so far only recorded from the Eastern Alps (Austria). – **Au**: K.

***Micarea melaena* (Nyl.) Hedl.**

Syn.: *Bacidia melaena* (Nyl.) Zahlbr., *Biatora stizenbergeri* Hepp, *Bilimbia melaena* (Nyl.) Arnold, *Catillaria constristans* *sensu* H. Magn., *Lecidea ilyophora* Stirt., *Lecidea melaena* Nyl.

L – Subs.: xyl, cor, ter-sil, deb – Alt.: 3–5 – Note: a cool-temperate to circumboreal-montane species found on decomposed lignum of old stumps, but also on plant debris, siliceous rocks and soil rich in humus, mostly in upland areas; widespread throughout the Alps. – **Au**: V, T, S, K, St, O, N. **Ge**: OB. **Sw**: BE, GR, LU, SZ, TI, UR, UW, VS. **Fr**: Sav, HSav. **It**: Ven, TAA, Lomb, Piem. **Sl**: SlA, Tg.

***Micarea melaeniza* Hedl.**

Syn.: *Lecidea melaeniza* (Hedl.) H. Magn.

L – Subs.: cor – Alt.: 3 – Note: a species with an indistinct thallus, subglobose to tuberculate, blackish apothecia with a dark red-brown hypothecium reacting K+ purple-black, and simple, mostly ovoid ascospores; on conifers in boreal to temperate-montane forests; a rare species, in the study area so far only recorded from a single locality in the Eastern Alps (Austria). – **Au**: S.

***Micarea micrococca* (Körb.) Gams *ex* Coppins**

Syn.: *Biatora micrococca* Körb., *Catillaria micrococca* (Körb.) Th. Fr., *Lecidea micrococca* (Körb.) Cromb.

L – Subs.: cor, xyl – Alt.: 3–4 – Note: a member of the *M. prasina*-complex, but with a different secondary chemistry and with pale to grey apothecia which are less variable in colour; on various substrates (bark, wood, debris) in shaded situations; very common in forest plantations of Western Europe; from the Alps there are only a few scattered records; earlier records of *M. prasina* may partly belong here. – **Au**: S, N. **Ge**: OB. **Sw**: SZ. **It**: Ven.

***Micarea minima* Poelt & Döbbeler**

L – Subs.: bry – Alt.: 3 – Note: a species with an indistinct thallus, extremely tiny apothecia which are whitish when dry and translucent when moist, and minute, narrowly ellipsoid to rod-shaped, simple or 1-septate ascospores; on leaflets of moribund to dead *Polytrichum*; apparently widespread in Europe from the lowlands to the montane belt, with a single record from the Eastern Alps (Austria). – **Au**: K.

***Micarea misella* (Nyl.) Hedl.**

Syn.: *Biatora misella* (Nyl.) H.G. Falk, Lecidea anomala Ach. f. misella Nyl., *Lecidea asserculorum* Ach. *nom. nud.*, *Lecidea globularis* (Nyl.) Lamy, *Lecidea melanochroza* Leight. *ex* Cromb., *Lecidea misella* (Nyl.) Nyl., *Micarea globularis* (Nyl.) Hedl.

L – Subs.: xyl, bry, cor, deb – Alt.: 2–4 – Note: a cool-temperate to circumboreal-montane species found on lignum, more rarely on acid bark; widespread throughout the Alps. – **Au**: V, T, S, K, St, O, N. **Ge**: OB. **Sw**: BE, LU, SZ, UW, VS. **Fr**: AHP, HAl, AMa, Isè, HSav, Var, Vau. **It**: Frl, TAA, Lomb. **Sl**: SlA.

***Micarea nigella* Coppins**

L – Subs.: xyl, cor – Alt.: 3 – Note: a species with an endoxylic thallus, black apothecia, stalked pycnidia, and a purple-brown pigment reacting K+ green in epihymenium, hypothecium and pycnidial wall; on wood, mostly in boreal-montane coniferous forests; widespread in Europe, with a single record from the Western Alps (Switzerland). – **Sw**: UW.

***Micarea nitschkeana* (J. Lahm *ex* Rabenh.) Harm.**

Syn.: *Bacidia nitschkeana* (J. Lahm *ex* Rabenh.) Zahlbr., *Bacidia spododes* (Nyl.) Zahlbr., *Bilimbia nitschkeana* J. Lahm *ex* Rabenh., *Bilimbia sophodes* (Nyl.) Arnold, *Lecidea nitschkeana* (J. Lahm *ex* Rabenh.) Stizenb., *Lecidea spododes* Nyl.

L – Subs.: cor, deb, xyl – Alt.: 2–4 – Note: on twigs and small branches of conifers and, more rarely of acid-barked deciduous trees and small shrubs, occasionally also on lignum. – **Au**: T, S, K, St. **Ge**: OB. **Sw**: BE, TI, UW. **Fr**: Var, Vau. **It**: TAA, Lomb. **Sl**: SlA.

***Micarea peliocarpa* (Anzi) Coppins & R. Sant.**

Syn.: *Bacidia albidolivens* (Nyl.) Zahlbr., *Bacidia hemipolioides* (Nyl.) Zahlbr., *Bacidia peliocarpa* (Anzi) Lettau, *Bacidia trisepta* (Nägeli) Zahlbr., *Bacidia triseptatuloides* (Harm.) Zahlbr., *Bacidia violacea* (P. Crouan & H. Crouan *ex* Nyl.) Arnold, *Bilimbia albicans* Arnold, *Bilimbia hemipolioides* (Nyl.) A.L. Sm., *Bilimbia peliocarpa* Anzi, *Bilimbia trisepta* (Nägeli) Hellb., *Bilimbia violacea* (P. Crouan & H. Crouan *ex* Nyl.) Th. Fr. *non* (Arnold) Arnold, *Lecidea albidolivens* Nyl., *Lecidea dufourii* Ach. *ex* Nyl., *Lecidea fraterculans* Nyl., *Lecidea hemipolioides* Nyl., *Lecidea trisepta* Nägeli, *Lecidea triseptatula* Nyl., *Lecidea triseptatuloides* Harm., *Lecidea violacea* P. Crouan & H. Crouan *ex* Nyl. *nom.illeg. non* A. Massal., *Micarea trisepta* (Nägeli) Wetmore, *Micarea violacea* (P. Crouan & H. Crouan *ex* Nyl.) Hedl., *Toninia hemipolioides* (Nyl.) Guillaumot

L – Subs.: cor, xyl, bry, sax, ter-sil – Alt.: 2–4 – Note: a temperate to boreal-montane, ecologically wide-ranging species found on the acid bark of deciduous (especially old oaks and *Fagus*) and coniferous trees, lignum, peaty soil, moribund bryophytes, and small siliceous pebbles; widespread throughout the Alps. – **Au**: V, T, S, K, St, O, N. **Ge**: OB. **Sw**: BE, GL, GR, LU, SG, SZ, TI, UR, UW, VS. **Fr**: AMa, Isè, HSav, Var, Vau. **It**: Frl, Ven, TAA, Lomb, VA. **Sl**: SlA.

***Micarea prasina*Fr.**

Syn.: *Bacidia subviridescens* (Nyl.) Zahlbr., *Biatora prasina* (Fr.) Trevis., *Biatorina prasina* (Fr.) Stein, *Bilimbia subviridescens* (Nyl.) H. Olivier, *Catillaria prasina* (Fr.) Th. Fr., *Catillaria prasiniza* (Nyl.) B. de Lesd., *Catillaria sordidescens* (Nyl.) Zahlbr., *Lecidea abdita* Erichsen, *Lecidea declivitatum* Erichsen, *Lecidea prasinella* Müll. Arg., *Lecidea prasiniza* Nyl., *Lecidea sordidescens* Nyl., *Lecidea subviridescens* Nyl., *Micarea polytrichi* Poelt & Döbbeler, *Micarea subviridescens* (Nyl.) Hedl.

L # – Subs.: cor, xyl, bry, sax, ter – Alt.: 2–4 – Note: a temperate to boreal-montane, morphologically and chemically variable species found on basal parts of old, acid-barked trees in montane forests, and on a wide range of other substrata; in its present circumscription this is one of the most common species of the genus in the Alps; however, this taxon represents a complex assemblage of species, yet to be properly disentangled. – **Au**: V, T, S, K, St, O, N, B. **Ge**: OB. **Sw**: BE, FR, GL, GR, LU, SG, SZ, TI, UW, VS. **Fr**: AHP, AMa, Drô, Isè, HSav, Var, Vau. **It**: Frl, Ven, TAA, Lomb, Piem, VA, Lig. **Sl**: SlA.

***Micarea rhabdogena* (Norman) Hedl.**

Syn.: *Biatora rhabdogena* Norman, *Lecidea rhabdogena* (Norman) Th. Fr.

L – Subs.: xyl – Alt.: 4 – Note: a species resembling *M. elachista*, but thallus endoxylic, with black, hemispherical to tuberculate apothecia, a brown epihymenium not reacting with K or N, a pale hypothecium, mostly simple, ellipsoid to oblong ascospores, and black pycnidia with an olivaceous wall reacting K+ violet, containing micro – or mesoconidia; on wood of conifers in boreal-temperate-montane/subalpine areas, with a single record from the Eastern Alps (Austria). – **Au**: K.

***Micarea submilliaria* (Nyl.) Coppins**

Syn.: *Bacidia subleprosula* Vězda, *Bilimbia submilliaria* (Nyl.) Arnold, *Lecidea granulans* Vain., *Lecidea submilliaria* Nyl., *Micarea granulans* (Vain.) Timdal, *Micarea subleprosula* (Vězda) Vězda

L – Subs.: bry – Alt.: 4–5 – Note: a species with a thallus as in *M. leprosula*, of bluish-grey, convex to subglobose areoles which easily break down to form yellowish-green sorediate patches, but reacting C+ red, Pd+ yellow (alectorialic acid), apothecia often lacking, black with a bluish pruina, ascospores 3 – to 7-septate, more than 35 µm long; overgrowing mosses and plant remnants on siliceous rocks in alpine heaths; widespread but rare, perhaps overlooked, being often sterile, with a few records from the Eastern Alps. – **Au**: S, K. **Ge**: OB.

***Micarea ternaria* (Nyl.) Vězda**

Syn.: *Bacidia ternaria* (Nyl.) Lettau, Lecidea sabuletorum f. ternaria Nyl., *Lecidea ternaria* (Nyl.) Nyl.

L – Subs.: deb – Alt.: 3–4 – Note: an arctic-alpine species growing on plant remains and siliceous rocks; from the Alps there are a few scattered records only. – **Au**: K, St. **Fr**: Sav. **It**: TAA.

***Micarea turfosa* (A. Massal.) Du Rietz**

Syn.: *Biatora turfosa* A. Massal., *Lecidea turfosa* (A. Massal.) Jatta, *Lecidea verrucula* (Norman) Th. Fr., *Lecidella verrucula* (Norman) Stein, *Micarea verrucula* (Norman) Hedl., *Oedemocarpus turfosus* (A. Massal.) Trevis.

L – Subs.: ter-sil, deb – Alt.: 3–5 – Note: a circumboreal-montane species found on peaty soil and terricolous bryophytes in upland areas. – **Au**: T, S, K, St. **Ge**: OB. **Sw**: LU, SZ, UW. **It**: Ven, TAA.

***Micarea viridileprosa* Coppins & van den Boom**

L – Subs.: cor, xyl, deb, sil, ter-sil – Alt.: 2 – Note: a recently described, mostly sterile species found on a wide variety of acid substrata in humid lowland areas; in the study area so far only reported from the base of the Western Alps (France). – **Fr**: AMa.

***Miriquidica aeneovirens* (Müll. Arg.) Hafellner**

Syn.: *Lecidea aeneovirens* Müll. Arg.

L # – Subs.: sil – Alt.: 4–5 – Note: a species resembling *M. garovaglii*, but with a thick, densely areolate, olive-brown thallus reacting K-, surrounded by a black hypothallus, and apothecia with brown-black, plane discs (colour not changing when moist) and thin, little prominent, somewhat glossy margins, an unpigmented hypothecium, a brown to olive-brown epihymenium, 8-spored asci, and ovoid to ellipsoid ascospores (10–14 × 7–8 μm); on siliceous rocks (mica-schist, gneiss) in the lower alpine belt; only known from the type locality in the Western Alps (Switzerland). – **Sw**: VS.

***Miriquidica atrofulva* (Sommerf.) A.J. Schwab & Rambold**

Syn.: *Lecidea atriuscula* H. Magn., *Lecidea atrofulva* Sommerf.

L – Subs.: met, sil – Alt.: 3–5 – Note: a circum – and bipolar lichen of metal-rich rocks, with optimum near and above treeline; mostly sterile and therefore perhaps overlooked in the Alps, but never common. – **Au**: V, T, S, K, St. **It**: TAA.

***Miriquidica complanata* (Körb.) Hertel & Rambold**

Syn.: *Aspicilia complanata* (Körb.) Stein, *Aspicilia microlepis* Körb., *Aspicilia superiuscula* (Nyl.) Hue, *Lecanora complanata* Körb., *Lecanora coracodes* Nyl., *Lecanora kultalensis* Vain., *Lecanora microlepis* (Körb.) Lettau, *Lecanora superiuscula* Nyl., *Lecanora tenebricans* Nyl.; incl. Miriquidica complanata (Körb.) Hertel & Rambold f. sorediata Owe-Larsson & Rambold

L – Subs.: sil – Alt.: 3–5 – Note: on moist siliceous rocks in upland areas, starting the life-cycle on yellow *Rhizocarpon*-species, with optimum above treeline. – **Au**: V, T, S, K, St. **Fr**: HSav. **It**: TAA, Piem, VA.

***Miriquidica deusta* (Stenh.) Hertel & Rambold**

Syn.: *Lecanora deusta* (Stenh.) Nyl., *Lecidea deusta* (Stenh.) Nyl., *Lecidea deustata* Zahlbr., Lecidea fuscoatra (L.) Ach. var. deusta Stenh., ?*Lecidea secernens* H. Magn.

L – Subs.: sil – Alt.: 1–5 – Note: a much misunderstood and overlooked species (being mostly sterile), with a probably western and southern distribution in Europe, found on exposed surfaces of base-rich siliceous rocks, with a wide altitudinal range. Records from Austria are in need of critical revision. – **Au**: ?V, ?T, ?S, ?K, ?St. **Fr**: Vau. **It**: Frl, TAA.

***Miriquidica disjecta* (Nyl.) Hertel & Rambold**

Syn.: *Lecidea disjecta* Nyl.

L – Subs.: sil – Alt.: 3–5 – Note: a species with a whitish thallus containing miriquidic acid, and black, sessile apothecia with a biatorine, brownish exciple, and an unpigmented hypothecium; on siliceous boulders, ecology and overall distribution poorly known; in the study area so far only recorded from the Eastern Alps in Italy (type material, on porphyric rocks near Paneveggio). – **It**: TAA.

***Miriquidica garovaglii* (Schaer.) Hertel & Rambold**

Syn.: *Lecidea aenea* (Fr.) Nyl., Lecidea aenea (Fr.) Nyl. var. garovaglii (Schaer.) Jatta, Lecidea atrobrunnea (DC.) Schaer. var. garovaglii (Schaer.) Jatta, *Lecidea garovaglii* Schaer., *Lecidea glacialis* Lynge, *Parmelia aenea* Fr., *Parmelia garovaglii* (Schaer.) Fr., *Psora garovaglii* (Schaer.) Anzi

L – Subs.: sil – Alt.: 3–6 – Note: a circumpolar, arctic-alpine species found on mineral-rich rocks wetted by rain in wind-exposed situations, such as on peaks and windy ridges, usually near or above treeline; widespread throughout the siliceous Alps. – **Au**: V, T, S, K, St. **Ge**: OB, Schw. **Sw**: BE, GR, TI, UR, VS. **Fr**: AHP, HAl, AMa, Isè, Sav, HSav. **It**: Frl, TAA, Lomb, Piem, VA.

***Miriquidica instrata* (Nyl.) Hertel & Rambold**

Syn.: *Biatora instrata* (Nyl.) Arnold, *Lecidea instrata* Nyl., *Lecidea subobscura* H. Magn.

L – Subs.: sil, sil-par – Alt.: 3–5 – Note: perhaps this is the primary species to *M. invadens*. It has minute areolae which are brown with a paler margin, and bear immersed, brown apothecia; on inclined faces of siliceous rocks, initially a parasite on other crustose lichens, such as species of *Aspicilia*, *Lecidea*, *Lecanora* and *Rhizocarpon*; widespread in the Holarctic region but rare, including in the Alps, where it occurs from the montane to the alpine belt. – **Au**: T, K, St. **Sw**: VS. **Fr**: Sav. **It**: TAA.

***Miriquidica intrudens* (H. Magn.) Hertel & Rambold**

Syn.: *Lecanora intrudens* H. Magn.

L – Subs.: sil-par, int – Alt.: 3–6 – Note: a probably circumpolar, arctic-alpine, silicicolous species which was largely overlooked in the past, starting the life-cycle on yellow *Rhizocarpon*-species, but also on *Aspicilia*-, *Lecanora* – and *Lecidea*-species; certainly more widespread near and above treeline in the Alps; perhaps confused with *Protoparmelia leproloma*, from which it differs in important morphological and chemical characters. – **Au**: V, T, S, K, St. **Sw**: GR, VS. **It**: Frl, TAA, Piem. **Sl**: SlA.

***Miriquidica invadens* Hafellner, Obermayer & Tretiach**

L – Subs.: sil-par – Alt.: 4–6 – Note: an obligate parasite on *Sporastatia polyspora*; widespread in the Alps, with optimum above treeline, and also known from the mountains of the Iberian and Balkan Peninsulas. – **Au**: T, K, St, N. **Sw**: BE, GR. **Fr**: AHP, HAl, AMa. **It**: Frl, TAA, Lomb, Piem.

***Miriquidica leucophaea* (Rabenh.) Hertel & Rambold**

Syn.: *Biatora consanguinea* Anzi, *Biatora leucophaea* Rabenh., *Lecidea aggregatula* Nyl., *Lecidea confertula* Stirt., *Lecidea discolorella* Nyl., *Lecidea karaensis* Lynge, *Lecidea leucophaea* (Rabenh.) Nyl., *Lecidea mesotropa* Nyl., *Lecidea nodulosa* (Körb.) H. Olivier, *Lecidea sporotea* Stirt., *Lecidella nodulosa* Körb., *Psora confertula* (Stirt.) Stirt.

L – Subs.: sil, met – Alt.: 3–5 – Note: a polymorphic species of metal-rich rocks, starting the life-cycle on yellow *Rhizocarpon*-species, more hygrophytic than *M. griseoatra*, being most frequent in sheltered situations, such as on faces with a late snow cover in upland areas. – **Au**: V, T, S, K, St, B. **Sw**: BE, GR, UR, VD, VS. **Fr**: Sav, HSav. **It**: TAA, Lomb, Piem. **Sl**: SlA.

***Miriquidica lulensis* (Hellb.) Hertel & Rambold**

Syn.: *Lecidea lulensis* Hellb.

L – Subs.: met, sil – Alt.: 4–6 – Note: on horizontally or weakly inclined faces of siliceous and often iron-rich rocks; with a few scattered records from the Alps. – **Au**: St. **Sw**: GR. **Fr**: AHP, Vau.

**Miriquidica nigroleprosa (Vain.) Hertel & Rambold var. nigroleprosa**

Syn.: *Lecanora nigroleprosa* Vain., *Lecidea nigrolepros*a (Vain.) H. Magn.

L – Subs.: sil – Alt.: 3–6 – Note: on hard siliceous rocks (*e.g.* granite) in exposed situations such as on windy ridges, starting the life-cycle on yellow *Rhizocarpon*-species; most often sterile, and therefore largely overlooked in the Alps. – **Au**: V, T, S, K, St, N. **Sw**: BE, GR, UR, VS. **Fr**: HSav. **It**: Frl.

**Miriquidica nigroleprosa (Vain.) Hertel & Rambold var. liljenstroemii (Du Rietz) Owe – Larss. & Rambold**

Syn.: *Lecidea liljenstroemii* Du Rietz, *Miriquidica liljenstroemii* (Du Rietz) R. Sant.

L – Subs.: sil – Alt.: 3–5 – Note: this variety differs from var. nigroleprosa in the whitish to pale grey, dull thallus composed of thick, contiguous areoles with convex soralia, and in the secondary chemistry (psoromic additional to miriquidic acid, and therefore medulla Pd+ yellow); on siliceous rocks, in the Alps often in *Rhizocarpon alpicola* associations; widespread in Europe but often not distinguished; in the Alps mainly from the subalpine to the lower alpine belt. – **Au**: T, K, St.

***Miriquidica obnubila* (Th. Fr. & Hellb.) Hertel & Rambold**

Syn.: *Lecidea obnubila* Th. Fr. & Hellb.

L # – Subs.: sil – Alt.: 4 – Note: an apparently rare species based on a type from northern Scandinavia, with a grey, areolate, spreading thallus containing miriquidic acid, delimited by a distinct black hypothallus, sessile, black apothecia with flat discs and persistently prominent margins; the more or less hyaline hypothecium and the olivaceous blue-green to purplish blue epihymenial layer are diagnostic; the only record from the Alps (Western Alps, Switzerland) is in need of reevaluation. *M. obnubila* *sensu* Hertel & Rambold, with a distinctly pigmented hypothecium, is a different species. – **Sw**: VS.

***Miriquidica plumbea* (Garov.) Hafellner, Obermayer & Tretiach**

Syn.: *Lecidea plumbea* Garov. in A. Massal., *Miriquidica limitata* Hertel & Rambold

L – Subs.: sil, met, int – Alt.: 4–6 – Note: an alpine to subnival species, confined to steeply inclined or overhanging surfaces of hard siliceous rocks, often with a high iron content. The basionym is often attributed to Massalongo, but he explicitly attributes the description of the new species to Garovaglio, who also sent him a specimen. – **Au**: T, K. **Sw**: ?GR, ?VS. **Fr**: AMa. **It**: Frl, Lomb, Piem.

***Miriquidica pulvinatula* (Arnold) Hertel & Rambold**

Syn.: *Lecidea circumnigrata* H. Magn., *Lecidea pulvinatula* (Arnold) Dalla Torre & Sarnth., *Lecidea wolfiana* Müll. Arg., *Lecidella pulvinatula* Arnold

L – Subs.: sil, met – Alt.: 4–6 – Note: on iron-rich crystalline rocks near and above treeline; with very few scattered records from the Alps; closely related to *M. leucophaea*. – **Au**: T. **Sw**: VS. **It**: TAA.

***Miriquidica subplumbea* (Anzi) Cl. Roux**

Syn.: *Lecidea inserena* Nyl., *Lecidea subplumbea* Anzi, *Lecidella subplumbea* (Anzi) Arnold, *Miriquidica griseoatra sensu auct. non* (Flot.) Hertel & Rambold

L – Subs.: sil – Alt.: 4–6 – Note: a probably circumpolar, arctic-alpine species found on wind-exposed, acidic siliceous rocks. We have placed here all previous records of *M. griseoatra*. – **Au**: T, S, K, St, N. **Sw**: BE, GR, UR, VS. **Fr**: AHP, AMa, Sav. **It**: Frl, TAA, Lomb, Piem, VA.

***Monerolechia badia* (Fr.) Kalb**

Syn.: *Buellia badia* (Fr.) A. Massal., *Buellia bayrhofferi* (Schaer.) H. Olivier, *Buellia conioptiza* (Nyl.) B. de Lesd., *Buellia duebenii* (Fr.) Hellb., *Buellia pernigrans* (Nyl.) Sandst., *Buellia schisticola* H. Magn., *Catolechia badia* (Fr.) Kremp., *Karschia bayrhofferi* (Schaer.) Rehm, *Lecidea badia* Fr., *Lecidea bayrhofferi* Schaer., *Lecidea conioptiza* Nyl., *Lecidea pernigrans* Nyl., *Monerolechia bayrhofferi* (Schaer.) Trevis., *Rhizocarpon badium* (Fr.) Sambo

L – Subs.: sil-par – Alt.: 1–4 – Note: a holarctic, subtropical to boreal-montane lichen found on steeply inclined, base-rich siliceous rocks, which starts the life-cycle on other lichens, later becoming autonomous; widespread throughout the siliceous Alps. – **Au**: ?V, T, S, K, St, O, B. **Sw**: BE. **Fr**: HAl, AMa, Isè, Sav, HSav, Var, Vau. **It**: Ven, TAA, Lomb, Piem, VA, Lig.

***Montanelia disjuncta* (Erichsen) Divakar, A. Crespo, Wedin & Essl.**

Syn.: *Melanelia disjuncta* (Erichsen) Essl., *Parmelia disjuncta* Erichsen, *Parmelia granulosa* Lynge *nom.illeg.*, *Parmelia granulosula* Oxner, Parmelia sorediata (Ach.) Röhl. var. coralloidea Lynge

L – Subs.: sil – Alt.: 2–5 – Note: a widespread lichen of dry-cool areas found on steeply inclined surfaces of siliceous rocks in upland areas. – **Au**: V, T, S, K, St, O. **Sw**: GR, LU, SZ, TI, UR, VD, VS. **Fr**: AMa. **It**: Ven, TAA, Lomb, Piem, VA. **Sl**: SlA.

***Montanelia panniformis* (Nyl.) Divakar, A. Crespo, Wedin & Essl.**

Syn.: *Melanelia panniformis* (Nyl.) Essl., *Parmelia crustificans* Hilitzer, *Parmelia pannariiformis* (Nyl. *ex* Lamy) Vain., *Parmelia panniformis* (Nyl.) Vain., Parmelia prolixa (Ach.) Röhl. f. panniformis Nyl.

L – Subs.: sil – Alt.: 2–5 – Note: a mainly northern species in Europe found on steeply inclined surfaces of siliceous rocks in upland areas; widespread throughout the Alps, but generally not common. – **Au**: V, T, S, K, St. **Sw**: GR, TI, UR, VS. **Fr**: Sav, HSav. **It**: TAA, Lomb, Piem.

***Montanelia sorediata* (Ach.) Divakar, A. Crespo, Wedin & Essl.**

Syn.: *Imbricaria sorediata* (Ach.) Arnold, *Imbricaria sprengelii* (Flörke) Körb., *Melanelia sorediata* (Ach.) Goward & Ahti, *Melanelia sorediosa* (Almb.) Essl., *Parmelia sorediata* (Ach.) Th. Fr., *Parmelia sorediifera* R. Sant., *Parmelia sorediosa* Almb., *Parmelia sprengelii* Flörke, Parmelia stygia (L.) Ach. var. sorediata Ach.

L – Subs.: sil, cor – Alt.: 2–4 – Note: on vertical seepage tracks of siliceous rocks, mostly in upland areas; widespread throughout the siliceous Alps. – **Au**: V, T, S, K, St, N. **Sw**: BE, GR, UR, VD, VS. **Fr**: AHP, HAl, AMa, Sav, HSav. **It**: Ven, TAA, Piem.

***Montanelia tominii* (Oxner) Divakar, A. Crespo, Wedin & Essl.**

Syn.: *Melanelia substygia* (Räsänen) Essl., *Melanelia tominii* (Oxner) Essl., *Parmelia saximontana* R.A. Anderson & W.A. Weber, *Parmelia substygia* Räsänen, *Parmelia tominii* Oxner

L – Subs.: sil – Alt.: 4–5 – Note: an arctic-alpine to boreal-montane, perhaps circumpolar lichen of exposed siliceous rocks, with several scattered records from the Alps. – **Au**: T, K. **Sw**: GR, VS. **Fr**: AMa. **It**: Ven, TAA, VA.

***Multiclavula corynoides* (Peck) R.H. Petersen**

Syn.: *Clavaria corynoides* Peck, Clavaria mucida Pers. var. rosea Sacc.

L – Subs.: ter-sil – Alt.: 4–5 – Note: a terricolous species with yellowish, straw-coloured to pinkish carpophores which are often subspathulate or laterally compressed toward the apex, and 4–6-sterigmate basidia; widespread in the Holarctic region, but most common in the boreal zone; in the Alps at higher elevations, with a still insufficiently known distribution. – **Au**: T, St. **Ge**: Schw. **It**: TAA.

***Multiclavula mucida* (Pers.) R.H. Petersen**

Syn.: *Clavaria mucida* Pers., *Lentaria mucida* (Pers.) Corner; incl. Lentaria mucida (Pers.) Corner var. hexaspora Geitler

L – Subs.: xyl – Alt.: 2–3 – Note: a species with white to cream-coloured, later grey carpophores, and 4–6-sterigmate basidia; on moist rotten logs in mixed forests; regarded as subcosmopolitan, in Central Europe up to the montane belt; probably still overlooked in parts of the Alps, but certainly rare. – **Au**: V, T, S, K, St, O. **Ge**: OB, Schw. **Sw**: UW. **It**: Ven.

***Multiclavula vernalis* (Schwein.) R.H. Petersen**

Syn.: *Clavaria vernalis* Schwein., *Clavulinopsis vernalis* (Schwein.) Corner

L – Subs.: ter-sil – Alt.: 4–5 – Note: on humic to sandy, acid soil in humid situations; perhaps overlooked by lichenologists, but certainly very rare in the Alps. – **Au**: T, S, St. **Fr**: Sav. **It**: Lomb.

***Mycobilimbia carneoalbida* (Müll. Arg.) S. Ekman & Printzen**

Syn.: *Bacidia carneoalbida* (Müll. Arg.) Coppins, *Bacidia sphaeroides auct.* non. (Dicks.) Zahlbr., *Biatora carneoalbida* (Müll. Arg.) Coppins, *Mycobilimbia sphaeroides* D.D. Awasthi, *Patellaria carneoalbida* Müll. Arg.

L – Subs.: cor, bry – Alt.: 3–4 – Note: on bark, mosses and plant debris, more rarely directly on rock in upland areas; widespread throughout the Alps. – **Au**: V, T, S, K, St, O, N. **Ge**: OB. **Sw**: BE, GR, SZ, UR, VD, VS. **Fr**: HAl, Sav, HSav. **It**: Ven, TAA, Lomb, Piem, Lig. **Sl**: SlA, Tg.

***Mycobilimbia epixanthoides* (Nyl.) Vitik., Ahti, Kuusinen, Lommi & T. Ulvinen *ex* Hafellner & Türk**

Syn.: *Biatora epixanthoides* (Nyl.) Diederich, *Lecidea epixanthoides* Nyl.

L – Subs.: bry, cor – Alt.: 2–3 – Note: on mossy trunks of deciduous trees, more rarely on siliceous rocks; widespread throughout the Alps, but regionally still overlooked. – **Au**: V, T, S, K, St, O, N. **Ge**: OB. **Sw**: GL, GR, SZ, TI, UW, VS. **Fr**: AHP, AMa, Var. **It**: Frl, TAA. **Sl**: SlA, Tg.

***Mycobilimbia olivacea* Aragón, Sarrión & Hafellner**

L # – Subs.: cor – Alt.: 3 – Note: on bark, mainly of conifers, at the base of trunks. The Italian material differs from the description in the paler thallus and in not having biseriate asci. The species is likely to be related to *Lecidea berengeriana*. – **It**: TAA.

***Mycobilimbia pilularis* (Körb.) Hafellner & Türk**

Syn.: *Bacidia sphaeroides* (Dicks.) Zahlbr., *Biatora pilularis* (Körb.) Hepp, *Biatorina pilularis* Körb., *Catillaria sphaeroides* (A. Massal.) Schuler

L – Subs.: cor, bry, deb, ter – Alt.: 2–4 – Note: on mosses growing on the bark of old deciduous trees, especially near the base of trunks in old, humid forests; widespread throughout the Alps. – **Au**: T, S, K, St, O, N. **Ge**: OB. **Sw**: BE, GR, UW, VD. **Fr**: HAl, AMa, Isè, HSav. **It**: Frl, Ven, TAA, Lomb, Piem.

***Mycobilimbia tetramera* (De Not.) Vitik., Ahti, Kuusinen, Lommi & T. Ulvinen *ex* Hafellner & Türk**

Syn.: *Bacidia fusca* (A. Massal.) Du Rietz, *Bacidia indurata* Zahlbr., *Bacidia obscurata* (Sommerf.) Zahlbr., *Bacidia tetramera* (De Not.) Coppins, *Biatora tetramera* (De Not.) Coppins, *Bilimbia fusca* A. Massal., *Bilimbia obscurata* (Sommerf.) Th. Fr., *Bilimbia tetramera* De Not., *Bilimbia triplicans* (Nyl.) Elenkin, *Lecidea triplicans* (Nyl.) Nyl., *Mycobilimbia fusca* (A. Massal.) Hafellner & V. Wirth, *Mycobilimbia obscurata* (Sommerf.) Rehm

L – Subs.: bry, deb, cor, ter – Alt.: 2–5 – Note: on mosses and plant debris on calcareous substrata, sometimes on bark, especially on basal parts of old trunks in open forests, and on other lichens (*e.g. Peltigera*); widespread throughout the Alps. – **Au**: V, T, S, K, St, O, N. **Ge**: OB. **Sw**: BE, GR, SZ, TI, UR, UW, VD, VS. **Fr**: AHP, HAl, AMa, Isè, Sav, HSav, Var, Vau. **It**: Frl, Ven, TAA, Lomb, Piem, VA, Lig. **Sl**: SlA.

***Mycoblastus affinis* (Schaer.) T. Schauer**

Syn.: *Lecidea affinis* Schaer., *Mycoblastus alpinus* (Fr.) Th. Fr. *ex* Hellb., *Mycoblastus melinus* (Kremp. *ex* Nyl.) Hellb., Mycoblastus sanguinarius (L.) Norman var. alpinus (Fr.) Stein

L – Subs.: cor, xyl, sax – Alt.: 3–4 – Note: an incompletely circumboreal-montane species found on old conifers, especially *Abies* and *Picea*, in open, humid, montane to subalpine woodlands, more rarely on lignum or siliceous rocks; perhaps more widespread in the Alps, but generally not common. – **Au**: V, T, S, K, St, O, N. **Ge**: OB. **Sw**: BE, GL, GR, LU, SZ, TI, UR, UW, VD, VS. **It**: Ven, TAA. **Sl**: SlA, Tg.

***Mycoblastus caesius* (Coppins & P. James) Tønsberg**

Syn.: *Haematomma caesium* Coppins & P. James

L – Subs.: cor – Alt.: 3 – Note: a species with a bluish-grey thallus bearing irregular soralia, containing perlatolic acid, always sterile in Europe, but apothecia known from Western North American material; on the acidic smooth bark of deciduous trees, rarely on conifers; widespread in Europe and more common in the West, with only a few records from the montane belt of the Alps. – **Au**: T. **Sw**: BE, UW.

***Mycoblastus melinodes* (Vain.) ined. (provisionally placed here, ICN Art. 36.1b)**

Syn.: Lecidea sanguinaria (L.) Ach. var. melinodes Vain., Mycoblastus sanguinarius (L.) Norman var. melinodes (Vain.) Zahlbr.

L # – Subs.: cor – Alt.: 4 – Note: a not generally accepted taxon with a sorediate thallus and apothecia with a pale hypothecium, based on a type from Siberia, where it was found encrusting bryophytes and plant debris; ecology and distribution are poorly known, with a single record from the Eastern Alps (Austria). – **Au**: S.

***Mycoblastus sanguinarius* (L.) Norman**

Syn.: *Lecidea didymospora* Stirt., *Lecidea sanguinaria* (L.) Ach., *Lichen sanguinarius* L., *Megalospora sanguinaria* (L.) A. Massal., *Oedemocarpus sanguinarius* (L.) Trevis.

L – Subs.: cor, xyl – Alt.: 3–4 – Note: a circumboreal-montane species found on lignum and bark of conifers (especially *Larix*), mostly in the subalpine belt; widespread throughout the Alps, but not always common. – **Au**: S, K, St, O, N. **Ge**: Ge. **Sw**: BE, GL, GR, LU, SZ, VD, VS. **Fr**: Isè. **It**: Ven, TAA, Piem. **Sl**: SlA, Tg.

***Mycoporum elabens* Flot. *ex* Nyl.**

Syn.: *Arthothelium flotovianum* Körb., *Dermatina elabens* (Flot. *ex* Nyl.) Zahlbr.

L – Subs.: cor – Alt.: 3 – Note: a species with a whitish granular thallus, roundish, black oligolocular stromata, fissitunicate asci, and muriform ascospores with 1 longitudinal septum; a rare lichen found on the bark of conifers (*Pinus*, *Abies*); in Europe, sterile material was probably overlooked, and most records are historical; in the study area so far only recorded from a few localities of the Eastern Alps. – **Au**: T, S, St, O. **Ge**: OB.

***Mycoporum fuscocinereum* (Körb.) Nyl.**

Syn.: *Dermatina fuscocinerea* (Körb.) Zahlbr.

L # – Subs.: cor – Alt.: 2–3 – Note: a species with a thallus recalling that of *Phlyctis argena*, with roundish, black, oligolocular stromata and fissitunicate asci containing muriform ascospores with several longitudinal septa; taxonomic status and placement are in need of re-evaluation; there are only few records from Europe, including those from the Eastern Alps (Austria). – **Au**: K, O.

***Myochroidea leprosula* (Arnold) Printzen, T. Sprib. & Tønsberg**

Syn.: *Biatora leprosula* Arnold, *Lecidea leprosula* (Arnold) Harm.

L – Subs.: cor – Alt.: 3–4 – Note: on twigs of subalpine shrubs; certainly more widespread in the Alps. – **Au**: V, T, S, K, St. **Sw**: VS. **It**: Frl.

***Myochroidea porphyrospoda* (Anzi) Printzen, T. Sprib. & Tønsberg**

Syn.: *Biatora porphyrospoda* Anzi, *Lecidea porphyrospoda* (Anzi) Th. Fr.

L – Subs.: cor – Alt.: 3–4 – Note: a mainly boreal-montane, probably circumpolar lichen found especially on basal parts of trunks, on bark, sometimes on lignum, mostly in upland areas; probably more widespread in the Alps. Earlier records from Switzerland (LU, UW) refer to *Protoparmelia hypotremella*. – **Au**: V, T, S, St. **Ge**: OB. **Sw**: GR, TI, VS. **It**: Lomb. **Sl**: SlA.

***Myochroidea rufofusca* (Anzi) Printzen, T. Sprib. & Tønsberg**

Syn.: *Biatora porphyroplaca* Hinteregger & Poelt, *Biatora rufofusca* Anzi, *Lecidea rufofusca* (Anzi) Nyl.

L – Subs.: cor, xyl, deb, bry – Alt.: 4–6 – Note: on terricolous mosses and plant debris on siliceous substrata, in the Alps regularly also on bark and wood of *Rhododendron* in the understory of subalpine coniferous forests, with optimum near treeline, in Northern Europe also on other phorophytes (*Salix*, *Betula*). – **Au**: V, T, S, St. **Sw**: GR, TI, UR, VS. **It**: Ven, TAA, Lomb.

**Myriolecis agardhiana (Ach.) Śliwa, Zhao Xin & Lumbsch subsp. agardhiana var. agardhiana**

Syn.: *Lecanora agardhiana* Ach., *Lecanora agardhianoides* A. Massal., *Lecanora latzelii* Zahlbr.

L – Subs.: cal – Alt.: 1–5 – Note: a widespread holarctic lichen found on horizontal to weakly inclined surfaces of hard limestone and dolomite, with a wide altitudinal range, but with optimum below the subalpine belt; the distinction from other related taxa still needs further study. – **Au**: V, T, S, K, St, O, N. **Ge**: OB, Schw. **Sw**: BE, GR, LU, SG, SZ, TI, UW, VD, VS. **Fr**: AHP, HAl, AMa, Drô, Isè, Sav, HSav, Var, Vau. **It**: Frl, Ven, TAA, Lomb, Piem, Lig. **Sl**: SlA, Tg.

**Myriolecis agardhiana (Ach.) Śliwa, Zhao Xin & Lumbsch subsp. sapaudica (Cl. Roux) Nimis & Cl. Roux var. sapaudica**

Syn.: Lecanora agardhiana Ach. subsp. sapaudica Cl. Roux, Lecanora agardhiana Ach. subsp. sapaudica Clauzade & Cl. Roux [invalidly published, ICN Art. 40.1 + 8]

L – Subs.: cal – Alt.: 3–5 – Note: restricted to areas near or above treeline; certainly more widespread in the Alps. – **Au**: V, T, S, K, St, O, N. **Ge**: OB. **Fr**: AHP, HAl, AMa, Drô, Sav, Vau. **It**: Ven, Piem.

**Myriolecis agardhiana (Ach.) Śliwa, Zhao Xin & Lumbsch subsp. sapaudica (Cl. Roux) Nimis & Cl. Roux var. lecidella (Poelt) ined. (provisionally placed here, ICN Art. 36.1b)**

Syn.: Lecanora agardhiana Ach. subsp. sapaudica Cl. Roux var. lecidella (Poelt) Leuckert & Poelt, *Lecanora lecidella* Poelt

L – Subs.: cal – Alt.: 3–5 – Note: similar to var. sapaudica, but with smaller apothecia which are immersed for a long time. – **Au**: T, K, St, O. **Ge**: OB. **Fr**: AHP, AMa, Drô.

***Myriolecis albescens* (Hoffm.) Śliwa, Zhao Xin & Lumbsch**

Syn.: *Lecanora albescens* (Hoffm.) Branth & Rostr., *Lecanora galactina* Ach., *Lecanora urbana* Nyl., *Patellaria albescens* (Hoffm.) Trevis., *Placodium albescens* (Hoffm.) DC., *Psora albescens* Hoffm., *Squamaria albescens* (Hoffm.) Anzi

L – Subs.: cal – Alt.: 1–5 – Note: a holarctic lichen found on a wide variety of calciferous or base-rich substrata including mortar, brick, roofing tiles, and walls, also in large urban areas; widespread throughout the Alps. – **Au**: V, T, S, K, St, O, N, B. **Ge**: OB, Schw. **Sw**: BE, GR, LU, SZ, VS. **Fr**: AHP, HAl, AMa, Drô, Isè, Sav, HSav, Var, Vau. **It**: Frl, Ven, TAA, Lomb, Piem, VA, Lig. **Sl**: Tg.

***Myriolecis antiqua* (J.R. Laundon) Śliwa, Zhao Xin & Lumbsch**

Syn.: *Lecanora antiqua* J.R. Laundon, *Lecanora conferta auct. non* (Duby *ex* Fr.) Grognot

L – Subs.: cal, int – Alt.: 2–5 – Note: on steeply inclined surfaces of basic siliceous rocks (especially basalt), sometimes on calciferous rocks. The species has been often recorded as *Lecanora conferta*, a northern species which seems to be absent in the Alps. – **Au**: T, K, B. **Sw**: SZ, VS. **It**: TAA, Piem.

***Myriolecis behringii* (Nyl.) Hafellner & Türk**

Syn.: *Lecanora behringii* Nyl., *Lecanora turbinata* Poelt & Leuckert

L – Subs.: cal – Alt.: 6 – Note: a (circum-) arctic-alpine species with an endosubstratic thallus, or with whitish, subsquamulose granules around the apothecia, which are turbinate to substipitate and have brown, non-pruinose discs, ascospores narrowly-ellipsoid; on limestone, in the Arctic also on bones, with a single record from the nival belt in the Eastern Alps (Austria). – **Au**: St, O.

***Myriolecis crenulata* (Hook.) Śliwa, Zhao Xin & Lumbsch**

Syn.: *Lecanora caesioalba* Körb., *Lecanora crenulata* Hook., *Lecanora exomila* Stirt., Lecanora hagenii (Ach.) Ach. var. crenulata (Hook.) Ach., Patellaria subfusca (L.) Wibel var. crenulata (Hook.) Trevis.

L – Subs.: cal, sil – Alt.: 2–5 – Note: a holarctic lichen found on steeply inclined faces or in underhangs of hard calciferous rocks, most frequent in upland areas; widespread throughout the Alps. – **Au**: V, T, S, K, St, O, N. **Ge**: OB, Schw. **Sw**: BE, GR, LU, SZ, VD, VS. **Fr**: AHP, HAl, AMa, Drô, Isè, Sav, HSav, Var, Vau. **It**: Frl, Ven, TAA, Lomb, Piem, VA, Lig. **Li**.

***Myriolecis dispersa* (Pers.) Śliwa, Zhao Xin & Lumbsch**

Syn.: *Lecanora dispersa* (Pers.) Flörke, Lecanora subluta Nyl. var. perspersa Nyl., *Lecanora umbrina auct. non* (Ach.) A. Massal., *Lichen dispersus* Pers., *Patellaria caesioalba* (Le Prévost *ex* Duby) Trevis. var. dispersa (Pers.) Trevis.

L – Subs.: cal, cal-par, ter-cal – Alt.: 1–5 – Note: most frequent in urban areas (*e.g.* on monuments, mortar walls, asbestos-cement) up to the montane belt; records from natural habitats and from upland areas may refer to other species, especially to *M. semipallida.* – **Au**: V, T, S, K, St, O, N, B. **Ge**: OB, Schw. **Sw**: BE, GR, LU, SZ, TI, UR, UW, VD, VS. **Fr**: AHP, HAl, AMa, Drô, Isè, Sav, HSav, Var, Vau. **It**: Frl, Ven, TAA, Lomb, Piem, VA, Lig. **Sl**: SlA.

***Myriolecis hagenii* (Ach.) Śliwa, Zhao Xin & Lumbsch**

Syn.: *Lecanora bormiensis* Nyl., *Lecanora hagenii* (Ach.) Ach., Lecanora hagenii (Ach.) Ach. f. saxifragae Anzi, Lecanora hagenii (Ach.) Ach. var. bormiensis (Nyl.) Dalla Torre & Sarnth., Lecanora hagenii (Ach.) Ach. var. fallax Hepp, *Lichen hagenii* Ach.

L – Subs.: cal, cor, xyl, bry, deb – Alt.: 1–5 – Note: a holarctic lichen belonging to a very difficult complex. It is common on isolated trees with base-rich bark, and on calciferous substrata, including walls of mortar; widespread throughout the Alps. – **Au**: V, T, S, K, St, O, N, B. **Ge**: OB, Schw. **Sw**: BE, FR, GL, GR, LU, SG, SZ, TI, UR, VD, VS. **Fr**: AHP, HAl, AMa, Drô, Sav, HSav, Var, Vau. **It**: Frl, Ven, TAA, Lomb, Piem, VA, Lig. **Sl**: SlA, Tg. **Li**.

***Myriolecis invadens* (H. Magn.) Śliwa, Zhao Xin & Lumbsch**

Syn.: *Lecanora invadens* H. Magn., *Lecanora meolansii* B. de Lesd.

L – Subs.: cal-par – Alt.: 1–5 – Note: on calciferous rocks in upland areas, often starting the life-cycle on other crustose lichens. The species is closely related to *M. semipallida*. – **Au**: ?V, St. **Sw**: VS. **Fr**: AHP, HAl, AMa, Sav, HSav, Var, Vau. **It**: Ven.

***Myriolecis juniperina* (Śliwa) Śliwa, Zhao Xin & Lumbsch**

Syn.: *Lecanora juniperina* Śliwa

L – Subs.: cor – Alt.: 3 – Note: a species with an endosubstratic thallus and densely grouped apothecia with pale discs which are usually covered by a whitish-grey pruina, the epihymenial granules insoluble in K and N, based on type from SW USA; on bark and wood (*Juniperus*, *Quercus*), with a single record from the montane belt of the Western Alps (France). – **Fr**: Sav.

***Myriolecis perpruinosa* (Fröberg) Śliwa, Zhao Xin & Lumbsch**

Syn.: *Lecanora perpruinosa* Fröberg

L – Subs.: cal – Alt.: 3–5 – Note: on calciferous rocks, often starting the life-cycle on other crustose lichens; widespread throughout the Alps. – **Au**: V, T, S, K, St, O, N. **Ge**: OB. **Sw**: GR, VS. **Fr**: AHP, AMa. **It**: Frl, TAA, Lomb, Piem, VA, Lig. **Sl**: SlA.

***Myriolecis persimilis* (Th. Fr.) Śliwa, Zhao Xin & Lumbsch**

Syn.: Lecanora hagenii (Ach.) Ach. subsp. persimilis Th. Fr., *Lecanora persimilis* (Th. Fr.) Arnold, *Lecanora umbrina sensuauct. medioeurop. non* (Ach.) A. Massal.

L # – Subs.: cor, xyl – Alt.: 2–3 – Note: a mild-temperate to Mediterranean lichen which is easily overlooked, most frequent on branches of *Fraxinus*, *Populus* and *Sambucus*. Very closely related to *M. hagenii*. – **Au**: V, T, S, K, St, O, N. **Ge**: OB, Schw. **Sw**: BE, FR, GL, GR, LU, SG, SZ, TI, UW, VS. **Fr**: HAl, AMa, Isè, Sav, HSav, Var. **Sl**: SlA, Tg.

***Myriolecis prominens* (Clauzade & Vězda) Cl. Roux & Nimis**

Syn.: *Lecanora prominens* Clauzade & Vězda

L – Subs.: cal – Alt.: 2 – Note: a calcicolous species; probably more widespread, at least in the Southern Alps – **Fr**: Var, Vau.

***Myriolecis pruinosa* (Chaub.) Śliwa, Zhao Xin & Lumbsch**

Syn.: *Lecanora adriatica* Zahlbr., *Lecanora cretacea* (Müll. Arg.) Stizenb., *Lecanora lagostana* Zahlbr., *Lecanora pruinifera* Nyl., *Lecanora pruinosa* Chaub., *Lecanora sulphurascens* Nyl., *Lecanora teichotea* Nyl., *Placodium cretaceum* Müll. Arg., *Placodium teichoteum* (Nyl.) Boistel, *Squamaria pruinosa* (Chaub.) Duby, *Squamaria sulphurascens* (Nyl.) H. Olivier

L – Subs.: cal – Alt.: 1–4 – Note: a mainly temperate species found on limestone, dolomite, mortar, brick and, more rarely, basic siliceous rocks; apparently more frequent in the Southern and Western Alps. – **Au**: St. **Fr**: AHP, HAl, AMa, Drô, HSav, Var, Vau. **It**: Frl, Ven, Lig. **Sl**: Tg.

***Myriolecis reuteri* (Schaer.) Śliwa, Zhao Xin & Lumbsch**

Syn.: *Lecanora reuteri* Schaer., *Patellaria reuteri* (Schaer.) Trevis., *Placodium reuteri* (Schaer.) A. Massal.

L – Subs.: cal – Alt.: 3–5 – Note: in underhangs or on steeply inclined surfaces of calcareous rocks in upland areas; widespread throughout the Alps. – **Au**: S, K, St, O, N. **Ge**: OB. **Sw**: BE, GR, LU, UW, VD, VS. **Fr**: AHP, AMa, Drô, HSav, Var, Vau. **It**: Frl, Ven, TAA.

***Myriolecis roridula* ined.**

Syn.: *Lecanora roridula* Poelt, Leuckert & Cl. Roux ined.

L # – Subs.: cal, int – Alt.: 2–3 – Note: a so far undescribed taxon recalling *M. dispersa*, but apothecia with reddish-brown discs often partly covered by a coarse pruina; on schists, volcanic rocks and limestone and similar anthropogenic substrates at low elevations; so far only known from the Eastern Alps (Austria). – **Au**: St, N.

***Myriolecis sambuci* (Pers.) Clem.**

Syn.: *Lecanora sambuci* (Pers.) Nyl., *Lecanora sambucioides* H. Magn., *Lichen sambuci* Pers.

L – Subs.: cor – Alt.: 1–4 – Note: a mainly mild-temperate species found on base-rich bark, especially on *Sambucus* and *Populus*; widespread throughout the Alps. – **Au**: V, T, S, K, St, O, N. **Ge**: OB, Schw. **Sw**: BE, GL, GR, LU, SZ, TI, UW. **Fr**: AHP, AMa, Isè, Sav, HSav, Var, Vau. **It**: Ven, TAA, Piem. **Sl**: SlA, Tg.

***Myriolecis semipallida* (H. Magn.) Śliwa, Zhao Xin & Lumbsch**

Syn.: *Lecanora flotoviana auct. non* Spreng., *Lecanora semipallida* H. Magn., *Lecanora xanthostoma* Cl. Roux *ex* Fröberg, *Lecanora xanthostoma* Wedd. *ex* Cl. Roux *nom. inval*.

L – Subs.: cal, int, par – Alt.: 2–6 – Note: a calcicolous species found on the top of exposed boulders, in sites often visited by birds; widespread throughout the Alps, absent from large settlements and very rare on man-made substrata. – **Au**: V, T, S, K, St, O, N. **Ge**: OB, Schw. **Sw**: GR, LU, SZ, VS. **Fr**: AHP, HAl, AMa, Drô, HSav, Var, Vau. **It**: Frl, Ven, TAA, Lomb, Piem, VA, Lig. **Sl**: SlA.

***Myriolecis torrida* (Vain.) Śliwa, Zhao Xin & Lumbsch**

Syn.: *Lecanora torrida* Vain.

L – Subs.: int – Alt.: 4–6 – Note: a species intermediate between *M. dispersa* and *M. albescens*, with a dispersed, areolate thallus and scattered apothecia with dark brown, epruinose discs, the epihymenial granules insoluble in K and N; on more or less calcareous rocks; widespread and known from both Hemispheres, with a few records from the Eastern Alps (Austria), but probably still overlooked elsewhere. – **Au**: K, St.

***Myriolecis wetmorei* (Śliwa) Śliwa, Zhao Xin & Lumbsch**

Syn.: *Lecanora wetmorei* Śliwa

L – Subs.: cor – Alt.: 3–4 – Note: a species described from Western North America, recently found also in Iran and the Caucasus, and also reported from the Eastern Alps (Italy). – **It**: TAA.

**Myriolecis zosterae (Ach.) Śliwa, Zhao Xin & Lumbsch subsp. palanderi (Vain.) ined. (provisionally placed here, ICN Art. 36.1b)**

Syn.: *Lecanora coerulescens* (Baumg.) Arnold, Lecanora hagenii (Ach.) Ach. f. saxifragae Anzi, Lecanora hagenii (Ach.) Ach. var. fallax Hepp, Lecanora hagenii (Ach.) Ach. var. hagenii f. coerulescens (Baumg.) Hazsl., *Myriolecis zosterae* *sensu* Nimis, Myriolecis hagenii (Ach.) Śliwa, Zhao Xin & Lumbsch var. fallax (Hepp) Hafellner

L – Subs.: deb, bry – Alt.: 4–6 – Note: a circumpolar, arctic-alpine lichen found on plant debris and mosses over calciferous substrata, from the Oromediterranean belt to the Arctic zone. This lichen does not belong to *Myriospora hagenii*, due to the larger apothecia which are restricted at the base, but fully corresponds to the description of Lecanora zosterae var. palanderi, a taxon which is quite different from the nominal variety (see Roux et al. 2017); widespread and common throughout the Alps. – **Au**: V, T, S, K, St, O, N. **Ge**: OB. **Fr**: AHP, HAl, Sav, HSav. **It**: Frl, Ven, TAA, Lomb, Piem, VA, Lig. **Sl**: SlA.

***Myriospora rufescens* (Turner *ex* Ach.) Hepp *ex* Uloth**

Syn.: *Acarospora rufescens* (Turner *ex* Ach.) Kremp., Acarospora smaragdula (Wahlenb.) A. Massal. var. rufescens (Turner *ex* Ach.) Clauzade & Cl. Roux, *Lecanora rufescens* (Turner *ex* Ach.) Nyl., *Sagedia rufescens* Turner *ex* Ach., *Silobia rufescens* (Turner *ex* Ach.) M. Westb. & Wedin, *Trimmatothelopsis rufescens* (Turner *ex* Ach.) Cl. Roux & Nav.-Ros.

L – Subs.: sil – Alt.: 2–5 – Note: according to Roux this is a good species, with an Atlantic distribution in Europe. The records from Austria are most probably erroneous. – **Au**: ?St, ?N. **Sw**: GR, UR, VS.

***Myriospora scabrida* (Hedl. *ex* H. Magn.) K. Knudsen & Arcadia**

Syn.: *Acarospora scabrida* Hedl. *ex* H. Magn., *Silobia scabrida* (Hedl. *ex* H. Magn.) M. Westb., *Trimmatothelopsis scabrida* (Hedl. *ex* H. Magn.) Cl. Roux & Nav.-Ros.

L – Subs.: sil – Alt.: 3–5 – Note: a species with an epilithic thallus consisting of pale grey to pale brown squamules, and large apothecia with brown, somewhat raised discs; usually on acidic schistose rocks; widespread in Europe and also known from both Americas; so far only recorded from a few localities in the Alps, perhaps still overlooked elsewhere. – **Au**: V, T, S, St. **Fr**: HSav.

***Myriospora smaragdula* (Wahlenb. *ex* Ach.) Nägeli *ex* Uloth**

Syn.: *Acarospora amphibola* Wedd., *Acarospora flavorubens* Bagl. & Carestia, *Acarospora isotorquensis* Alstrup, *Acarospora lesdainii* Harm. *ex* A.L. Sm., *Acarospora murina* Sandst., *Acarospora smaragdula* (Wahlenb. *ex* Ach.) A. Massal., *Acarospora smaragdula* (Wahlenb. *ex* Ach.) A. Massal. subsp. lesdainii (Harm. *ex* A.L. Sm.) Clauzade & Cl. Roux, *Acarospora undata* Clauzade, Cl. Roux & V. Wirth, *Silobia smaragdula* (Wahlenb. *ex* Ach.) M. Westb. & Wedin, *Trimmatothelopsis smaragdula* (Wahlenb. *ex* Ach.) Cl. Roux & Nav.-Ros.

L – Subs.: sil, int, met – Alt.: 3–5 – Note: a cool-temperate to boreal-montane, perhaps circumpolar, variable species of steeply inclined to underhanging surfaces of base – and often metal-rich, sometimes weakly calciferous siliceous rocks, mostly in upland areas; widespread throughout the Alps. – **Au**: V, T, S, K, St. **Sw**: BE, SZ, UR, VS. **Fr**: AHP, HAl, Sav, HSav. **It**: Frl, TAA, Ven, Lomb, Piem, VA, Lig.

***Myriospora tangerina* (M. Westb. & Wedin) K. Knudsen & Arcadia**

Syn.: *Silobia tangerina* M. Westb. & Wedin

L – Subs.: sil – Alt.: 5 – Note: a species characterised by a pale, orange-ochraceous, subsquamulose thallus and small, punctiform apothecia; described from Sweden, it is also known from Norway, the Czech Republic and Russia (Novaya Zemlja), and in Scandinavia it has a distinctly northern distribution, with a single record from the base of the Western Alps (France). – **Fr**: AMa.

***Naetrocymbe saxicola* (A. Massal.) R.C. Harris**

Syn.: *Arthopyrenia saxicola* A. Massal., *Leiophloea saxicola* (A. Massal.) Riedl, *Naetrocymbe massalongiana* (Hepp) R.C. Harris, *Pyrenocollema saxicola* (A. Massal.) Coppins, *Sagedia massalongiana* Hepp

L – Subs.: cal, int – Alt.: 2–4 – Note: an early coloniser of hard, steeply inclined surfaces of calcareous rocks, on surfaces which rapidly dry out after rain, often with *Hymenelia coerulea*. This species is clearly lichenised, with *Trentepohlia*. – **Au**: S, K, St, O, N. **Ge**: Ge. **Sw**: BE, LU, SZ, UW. **Fr**: AHP, AMa, Drô, Sav, HSav, Var, Vau. **It**: Frl, Ven, TAA, Lomb, Piem. **Sl**: SlA.

***Neocatapyrenium radicescens* (Nyl.) Breuss**

Syn.: *Catapyrenium radicescens* (Nyl.) Breuss, *Dermatocarpon pachylepis* (Anzi) Zahlbr., *Endocarpon pachylepis* Anzi, *Verrucaria radicescens* Nyl.

L – Subs.: ter-sil – Alt.: 4–5 – Note: on more or less fissured siliceous rocks near or above treeline; hitherto known only from Southern France, Italy and Switzerland. – **Sw**: GR. **Fr**: HAl, AMa, Sav. **It**: Lomb.

***Nephroma bellum* (Spreng.) Tuck.**

Syn.: *Nephroma laevigatum auct. non* Ach., *Peltigera bella* Spreng.

L – Subs.: cor, bry, sil, xyl – Alt.: 1–4 – Note: a holarctic *Lobarion*-species of bark, epiphytic bryophytes and mossy rocks in humid forests; widespread throughout the Alps but not very common, and perhaps declining. – **Au**: V, T, S, K, St, O, N. **Ge**: OB. **Sw**: BE, GL, GR, SG, SZ, UR, UW, VD, VS. **Fr**: AHP, Isè, Sav, HSav, Vau. **It**: Frl, Ven, TAA, Lomb, Piem, VA.

***Nephroma expallidum* (Nyl.) Nyl.**

Syn.: *Nephromium expallidum* Nyl.

L – Subs.: ter-sil, ter-cal – Alt.: 4–6 – Note: an arctic-alpine species found on soil and amongst bryophytes over siliceous substrata, near or above treeline; exceptionally reaching the nival belt in the Alps. – **Au**: V, T, S, St. **Ge**: OB. **Sw**: GR, TI, VS. **It**: Ven, TAA, Piem.

***Nephroma helveticum* Ach.**

L – Subs.: cor – Alt.: 3–4 – Note: a cool-temperate to southern boreal-montane, circumpolar lichen found on bark, exceptionally on siliceous rocks in humid, but somehow subcontinental upland areas; probably more widespread in the Alps, but never common, and strongly declining. – **Au**: V, T. **It**: Ven, TAA, Lomb, Piem. **Sl**: Tg.

***Nephroma laevigatum* Ach.**

Syn.: *Nephroma lusitanicum* Schaer., *Nephromium laevigatum* (Ach.) Nyl., *Nephromium lusitanicum* (Schaer.) Nyl.

L – Subs.: cor – Alt.: 1–4 – Note: a mild-temperate to humid subtropical lichen found on bark, epiphytic bryophytes and mossy rocks in humid, open forests; apparently more frequent in the Southern and Western Alps. – **Sw**: GR, TI, UR, VS. **Fr**: AHP, AMa, Isè, Sav, HSav, Var, Vau. **It**: Frl, Ven, TAA, Lomb, Piem, VA. **Sl**: SlA.

***Nephroma parile* (Ach.) Ach.**

Syn.: *Lichen parilis* Ach., Nephroma laevigatum Ach. f. sorediatum Schaer., Nephromium laevigatum (Ach.) Nyl. var. parile (Ach.) Nyl.

L – Subs.: cor, bry, sil – Alt.: 2–5 – Note: a cool-temperate to circumboreal-montane lichen found on bark, epiphytic mosses, basic siliceous rocks and soil in humid and sheltered situations, mostly in upland areas; widespread throughout the Alps. – **Au**: V, T, S, K, St, O, N. **Ge**: OB. **Sw**: BE, GL, GR, LU, SZ, TI, UR, UW, VD, VS. **Fr**: AHP, HAl, AMa, Isè, Sav, HSav, Var, Vau. **It**: Frl, Ven, TAA, Lomb, Piem, VA, Lig. **Sl**: SlA, Tg.

***Nephroma resupinatum* (L.) Ach.**

Syn.: *Lichen resupinatus* L., *Nephroma filarszkyanum* Gyeln., *Nephroma papyraceum* (Hoffm.) De Not., *Nephroma rameum* (Schaer.) A. Massal., *Nephroma tomentosum* (Hoffm.) Flot., *Nephromium resupinatum* (L.) Arnold, *Nephromium tomentosum* (Hoffm.) Nyl.

L – Subs.: cor, bry, sil – Alt.: 2–4 – Note: a mainly temperate, holarctic lichen found on mossy trunks, rocks, more rarely on soil, in cool and sheltered habitats, with optimum in humid beech forests; widespread throughout the Alps, but generally not common. – **Au**: V, T, S, K, St, O, N. **Ge**: OB, Schw. **Sw**: BE, GL, GR, SG, SZ, TI, UR, UW, VD, VS. **Fr**: AHP, HAl, AMa, Drô, Isè, Sav, HSav, Vau. **It**: Frl, Ven, TAA, Lomb, Piem, VA, Lig. **Sl**: SlA, Tg.

***Nephroma tangeriense* (Maheu & A. Gillet) Zahlbr.**

Syn.: *Nephromium tangeriense* Maheu & A. Gillet

L – Subs.: sax, cor – Alt.: 1–2 – Note: a Mediterranean-Atlantic species found on rocks, more rarely on bark, in exposed situations but in humid areas, usually at low elevations, with a few records from the Western Alps (France, Italy). – **Fr**: AHP, AMa, Var, Vau. **It**: Piem.

***Nephromopsis laureri* (Kremp.) Kurok.**

Syn.: *Cetraria complicata* Laurer, *Cetraria laureri* Kremp., *Platysma laureri* (Kremp.) Nyl., *Tuckneraria laureri* (Kremp.) Randlane & A. Thell

L – Subs.: cor, xyl – Alt.: 2–4 – Note: on acid-barked coniferous and deciduous trees in cold-humid montane woodlands, mostly in mixed *Fagus*-*Abies* forests, but also on *Larix* in humid subalpine stands; widespread throughout the Alps, but generally rare. – **Au**: V, T, S, K, St, O, N. **Ge**: OB. **Sw**: BE, GR, LU, SZ, TI, UR, UW. **It**: Frl, Ven, TAA, Lomb. **Sl**: SlA, Tg.

***Nevesia sampaiana* (Tav.) P.M. Jørg., L. Lindblom, Wedin & S. Ekman**

Syn.: *Fuscopannaria sampaiana* (Tav.) P.M. Jørg., Pannaria craspedia Körb. var. isidiata Harm., *Pannaria sampaiana* Tav.

L – Subs.: cor – Alt.: 1–2 – Note: a mild-temperate species found on bark of ancient deciduous trees in humid woodlands; certainly very rare and endangered in the Alps. – **Sl**: Tg.

***Normandina acroglypta* (Norman) Aptroot**

Syn.: *Arthopyrenia chlorococca* (Leight.) A.L. Sm., *Lauderlindsaya acroglypta* (Norman) R. Sant., *Lauderlindsaya chlorococca* (Leight.) Diederich & Sérus., *Lauderlindsaya erichsenii* (Keissl.) Diederich & Sérus., *Normandina erichsenii* (Keissl.) Aptroot, *Polyblastia armericola* Walt. Watson, *Sphaerulina chlorococca* (Leight.) R. Sant., *Thelidium acroglyptum* Norman, *Thelidium chlorococcum* (Leight.) Keissl., *Thelidium erichsenii* Keissl., *Verrucaria chlorococca* Leight.

L – Subs.: cor, bry, ?par – Alt.: 2–3 – Note: a mild-temperate lichen, most often found on trees with subacid to base-rich bark, often on mosses; the biology of this species is disputed. – **Au**: S, K, St, O, N. **Ge**: OB. **Sw**: GR, TI, UW. **It**: Frl, Ven. **Sl**: SlA.

***Normandina pulchella* (Borrer) Nyl.**

Syn.: *Lauderlindsaya borreri* (Tul.) J.C. David & D. Hawksw., *Lenormandia jungermanniae* Nyl., *Lenormandia pulchella* (Borrer) A. Massal., *Normandina jungermanniae* (Nyl.) Nyl., *Sphaeria borreri* Tul., *Verrucaria pulchella* Borrer

L – Subs.: bry, cor – Alt.: 1–3 – Note: a mild-temperate lichen most often found on epiphytic *Frullania* and other liverworts in rather humid areas; widespread throughout the Alps. – **Au**: V, T, S, K, St, O, N, B. **Ge**: OB, Schw. **Sw**: BE, FR, GL, GR, LU, SG, SZ, TI, UR, UW, VD, VS. **Fr**: AMa, Isè, Sav, HSav, Var, Vau. **It**: Frl, Ven, TAA, Lomb, Piem, VA, Lig. **Sl**: SlA, Tg. **Li**.

***Ocellomma picconianum* (Bagl.) Ertz & Tehler**

Syn.: *Lecanactis saltelii* B. de Lesd., *Lecania picconiana* Bagl., *Schismatomma picconianum* (Bagl.) J. Steiner

L – Subs.: cor – Alt.: 2 – Note: a Mediterranean-Atlantic species, most abundant on evergreen trees in Tyrrhenian Italy, especially on *Quercus ilex* in humid Mediterranean woodlands, with a few records from the base of the Western Alps (France, Italy). – **Fr**: AMa, Var, Vau. **It**: Lig.

***Ochrolechia alboflavescens* (Wulfen) Zahlbr.**

Syn.: Lecanora tartarea (L.) Ach. var. alboflavescens (Wulfen) Flot., *Lichen alboflavescens* Wulfen, *Ochrolechia papillata* (Räsänen) Verseghy, Ochrolechia tartarea (L.) A. Massal. var. alboflavescens (Wulfen) A. Massal., *Pertusaria decipiens* Erichsen (*fide* Almborn)

L – Subs.: cor, xyl – Alt.: 3–4 – Note: a boreal-montane species found on bark of conifers, more rarely of acid-barked deciduous trees, usually in upland areas; widespread throughout the Alps. – **Au**: V, T, S, K, St, O, N. **Ge**: OB, Schw. **Sw**: BE, GL, GR, LU, SZ, TI, UR, UW, VS. **Fr**: AHP, HAl, AMa, Isè, Sav, HSav, Vau. **It**: Frl, Ven, TAA, Lomb, Piem, VA. **Sl**: SlA.

***Ochrolechia androgyna* (Hoffm.) Arnold**

Syn.: *Lecanora subtartarea* Nyl., *Lichen androgynus* Hoffm., *Ochrolechia subtartarea* (Nyl.) A. Massal., Ochrolechia tartarea (L.) A. Massal. var. arborea (DC.) Körb.

L – Subs.: cor, sil, ter-bry – Alt.: 2–5 – Note: on bark and on steeply inclined faces of siliceous rocks in humid montane forests, sometimes also on soil and bryophytes; some records might refer to *O. bahusiensis* H. Magn. or to *O. mahluensis* Räsänen. – **Au**: V, T, S, K, St, O, N, B. **Ge**: OB, Schw. **Sw**: BE, GL, GR, LU, SZ, TI, UR, UW, VD, VS. **Fr**: AHP, HAl, AMa, Isè, Sav, HSav. **It**: Frl, Ven, TAA, Lomb, Piem, VA. **Sl**: SlA, Tg.

***Ochrolechia arborea* (Kreyer) Almb.**

Syn.: *Ochrolechia sordidogrisea* (Erichsen) E. Schreiner & Hafellner, *Pertusaria arborea* (Kreyer) Zahlbr., *Pertusaria myriosora* Erichsen, *Pertusaria sordidogrisea* Erichsen, Variolaria lactea Wahlenb. var. arborea Kreyer

L – Subs.: cor – Alt.: 2–4 – Note: a mainly temperate lichen found on isolated deciduous trees with mineral-rich bark. The species is more frequent in Fennoscandia and Central-Eastern Europe, being rare in the western and southern parts of the continent; widespread throughout the Alps. – **Au**: V, T, S, K, St, O, N, B. **Ge**: OB. **Sw**: BE, GL, GR, LU, SZ, TI, UR, UW, VS. **Fr**: AHP, AMa, Isè. **It**: Frl, Ven, TAA, Lomb, Piem, Lig. **Sl**: SlA, Tg.

***Ochrolechia bahusiensis* H. Magn.**

Syn.: Ochrolechia subviridis (Høeg) Erichsen f. pulverulenta Erichsen, Ochrolechia subviridis (Høeg) Erichsen var. lignaria (Erichsen) Erichsen

L – Subs.: cor, xyl – Alt.: 2–4 – Note: a species morphologically resembling *O. arborea*, but with a different secondary chemistry (murolic acid syndrome, gyrophoric acid syndrome), and therefore soralia UV-; mostly on the bark of deciduous trees in various forest types; widespread in Europe, but in Central Europe not consistently distinguished, and distribution therefore incompletely documented. – **Au**: S, St.

***Ochrolechia balcanica* Verseghy**

L – Subs.: cor – Alt.: 2–3 – Note: this conspicuous lichen probably belongs, together with such species as *Parmelia submontana* and *Physconia venusta*, to an ancient pre-glacial Mediterranean-montane element; it is common and often abundant in the Mediterranean mountains, with a few records from the Western Alps. – **Fr**: Var. **It**: Lig.

***Ochrolechia crozalsiana* Clauzade & Vězda**

Syn.: *Ochrolechia erichsenii* Hafellner & Türk, *Pertusaria tumidula* Erichsen

L – Subs.: sil – Alt.: 1–5 – Note: a rare Mediterranean species known from France, Italy and perhaps the Iberian Peninsula; it is related to *O. tartarea*, but is morphologically and chemically different. – **Au**: V, ?T, K, St. **Sw**: GR, VS. **Fr**: Isè, Var. **It**: Lig.

***Ochrolechia frigida* (Sw.) Lynge**

Syn.: *Lichen frigidus* Sw., *Ochrolechia elisabethae-kolae* Verseghy, *Ochrolechia gonatodes* (Ach.) Räsänen, *Ochrolechia lapuensis* (Vain.) Räsänen, *Ochrolechia pterulina* (Nyl.) G.E. Howard, Ochrolechia tartarea (L.) A. Massal. var. telephoroides (Th. Fr.) Arnold

L – Subs.: deb – Alt.: 5–6 – Note: an arctic-alpine, circumpolar species found on mosses, plant debris and soil above treeline; widespread throughout the Alps. – **Au**: S, K, St. **Ge**: OB, Schw. **Fr**: AHP, HAl, AMa, Isè, Sav, HSav. **It**: Ven, Lomb, Piem, VA. **Sl**: SlA.

***Ochrolechia inaequatula auct. non* (Nyl.) Zahlbr.**

L # – Subs.: bry, sil, deb, ter – Alt.: 3–6 – Note: this name is used here for sorediate morphs of an *Ochrolechia* with swollen areoles and a secondary chemistry identical to that of *O. frigida* (gyrophoric acid syndrome); *O. inaequatula* *s.str.* is based on a type from western Alaska; encrusting plant debris and bryophytes, mostly over calcareous to intermediate soils; common in the Alps, but subsumed under *O. frigida* in some countries (*e.g.* in Italy). – **Au**: V, T, S, K, St, N. **Ge**: OB. **Sw**: SZ, VS. **Fr**: HAl, Isè, Sav, HSav, Vau.

***Ochrolechia mahluensis* Räsänen**

Syn.: Ochrolechia androgyna (Hoffm.) Arnold var. pergranulosa Räsänen

L – Subs.: cor – Alt.: 3–4 – Note: a species resembling *O. androgyna*, but with a thinner, sorediate thallus containing the gyrophoric acid syndrome in soralia and apothecia (thalline cortex therefore C-); usually on the acidic bark of coniferous and deciduous trees in open forests; widespread in Europe, in the Central European mountains mostly in the montane and subalpine belts, in the Alps not consistently distinguished, and distribution therefore poorly known. – **Au**: St.

***Ochrolechia microstictoides* Räsänen**

Syn.: *Pertusaria leprarioidesauct. p.p. non* Erichsen, *Pertusaria silvatica* H. Magn.

L – Subs.: cor, xyl – Alt.: 2–4 – Note: a cool-temperate to boreal-montane lichen mostly found on conifers in open, humid forests. – **Au**: V, T, S, K, St, N. **Ge**: Schw. **Sw**: GR, SZ, TI, UW, VS. **Fr**: AHP, Isè. **It**: Ven, TAA. **Sl**: SlA, Tg.

***Ochrolechia pallescens* (L.) A. Massal.**

Syn.: *Lichen pallescens* L., Ochrolechia parella (L.) A. Massal. subsp. pallescens (L.) Clauzade & Cl. Roux, *Ochrolechia parella* (L.) A. Massal. var. *tumidula auct. non* (Pers.) Arnold, *Parmelia pallescens* (L.) Fr.

L – Subs.: cor – Alt.: 1–3 – Note: a mainly temperate species found on deciduous trees in humid areas. The relationships with *O. parella* remain to be clarified: the two species are similar, but have a different ecology and distribution, and they hardly can be treated as forms of one and the same species. – **Au**: V, T, S, K, St, O, N. **Ge**: OB, Schw. **Sw**: BE, GL, GR, LU, SZ, TI, UW, VD, VS. **Fr**: AHP, AMa, Isè, Sav, HSav, Var, Vau. **It**: Frl, Ven, TAA, Lomb, Piem, VA, Lig. **Sl**: SlA, Tg.

***Ochrolechia parella* (L.) A. Massal.**

Syn.: *Lecanora parella* (L.) Ach., *Lichen parellus* L., Ochrolechia pallescens (L.) A. Massal. var. parella (L.) Körb., Ochrolechia parella (L.) A. Massal. var. albissima Zschacke, *Parmelia parella* (L.) Ach.

L – Subs.: sil – Alt.: 1–3 – Note: closely related to *O. pallescens*, but silicicolous; widespread in the Alps, with optimum below the montane belt. – **Au**: V, T, S, K, St, N. **Sw**: BE, GR, VS. **Fr**: HAl, AMa, Isè, Sav, HSav, Var, Vau. **It**: Ven, TAA, Lomb, VA, Lig. **Sl**: SlA.

***Ochrolechia subviridis* (Høeg) Erichsen**

Syn.: *Ochrolechia gallica* Verseghy, *Ochrolechia yasudae auct. non* Vain., *Pertusaria subviridis* Høeg

L – Subs.: cor – Alt.: 2–3 – Note: a mild-temperate lichen found on old, isolated deciduous trees in humid areas. – **Au**: T, ?St. **Ge**: OB. **Sw**: GR, SZ, VD, VS. **Fr**: AHP, AMa, Isè, HSav, Var, Vau. **It**: Lig. **Sl**: SlA, Tg.

***Ochrolechia szatalaensis* Verseghy**

Syn.: *Ochrolechia pseudotartarea* (Vain.) Verseghy, Ochrolechia szatalaensis Verseghy var. macrospora Verseghy, *Ochrolechia tenuissima* Verseghy

L – Subs.: cor – Alt.: 3–4 – Note: a cool-temperate to boreal-montane species with optimum on twigs in humid and cold sites; widespread throughout the Alps. – **Au**: V, T, S, K, St, O, N. **Ge**: OB, Schw. **Sw**: BE, FR, GL, GR, SG, SZ, TI, UR, VD. **Fr**: AMa, Isè. **It**: Frl, Ven, TAA, Lomb, Lig. **Sl**: SlA, Tg.

***Ochrolechia tartarea* (L.) A. Massal.**

Syn.: *Lecanora tartarea* (L.) Ach., *Lichen tartareus* L., Ochrolechia androgyna (Hoffm.) Arnold var. saxorum (Oeder) Verseghy *nonauct.*, *Parmelia tartarea* (L.) Ach., *Pertusaria gyrocheila* Nyl.

L – Subs.: sil, bry – Alt.: 3–5 – Note: on siliceous rocks and on thin soil layers in humid situations, mostly in upland areas. In the past the species was frequently confused with other taxa. – **Au**: V, T, S, ?K, ?St. **Sw**: BE, GR, VD, VS. **Fr**: AMa, Isè, Sav, HSav. **It**: Ven, TAA, Lomb, Piem, VA, Lig.

***Ochrolechia tiroliensis* (Erichsen) Hafellner & Türk**

Syn.: *Pertusaria tiroliensis* Erichsen

L # – Subs.: bry – Alt.: 3 – Note: a species encrusting plant debris and bryophytes, with a sterile thallus containing the gyrophoric acid syndrome; not consistently distinguished and distribution therefore poorly known. – **Au**: V, T, K, St.

***Ochrolechia trochophora* (Vain.) Oshio**

Syn.: Ochrolechia pallescens (L.) A. Massal. var. krempelhuberi Verseghy, *Pertusaria trochophora* Vain.

L – Subs.: cor – Alt.: 3 – Note: a species with a pustulate thallus containing the gyrophoric acid syndrome in the cortex but not in medulla, usually fertile; on bark of deciduous trees; widespread in the Holarctic region, but rare in Europe, with a single historical record from the Eastern Alps (Germany). – **Ge**: OB.

***Ochrolechia turneri* (Sm.) Hasselrot**

Syn.: Lecanora parella (L.) Ach. var. turneri (Sm.) Nyl., *Lecanora turneri* (Sm.) Ach., *Lichen turneri* Sm., Ochrolechia alboflavescens (Wulfen) Zahlbr. var. turneri (Sm.) Verseghy, Ochrolechia pallescens (L.) A. Massal. var. turneri (Sm.) Körb., Ochrolechia parella (L.) A. Massal. var. turneri (Sm.) Arnold, *Pertusaria henrici* Harm. *non* *sensu* Erichsen, *Pertusaria leprarioidesauct. p.p. non* Erichsen

L – Subs.: cor – Alt.: 2–4 – Note: on bark of isolated (mostly) deciduous trees in open, humid, montane to subalpine woodlands; widespread throughout the Alps. – **Au**: V, T, S, K, St, O, N. **Ge**: OB. **Sw**: BE, GR, LU, SZ, TI, UW, VS. **Fr**: AHP, HAl, AMa, Isè, Var, Vau. **It**: Ven, TAA, Lig. **Sl**: SlA, Tg.

***Ochrolechia upsaliensis* (L.) A. Massal.**

Syn.: Lecanora parella (L.) Ach. var. upsaliensis (L.) Ach., *Lichen upsaliensis* L.

L – Subs.: deb, bry, ter-cal – Alt.: 3–6 – Note: an arctic-alpine species of calciferous soil and plant debris, with optimum above treeline; widespread throughout the Alps. – **Au**: V, T, S, K, St, O, N. **Ge**: OB, Schw. **Sw**: BE, FR, GR, LU, SG, SZ, VD, VS. **Fr**: HAl, AMa, Isè, Sav, HSav, Vau. **It**: Frl, Ven, TAA, Lomb, Piem. **Sl**: SlA. **Li**.

***Ochrolechia xanthostoma* (Sommerf.) K. Schmitz & Lumbsch**

Syn.: *Aspicilia poriniformis* (Nyl.) Arnold, *Pertusaria poriniformis* (Nyl.) Clauzade & Cl. Roux, *Pertusaria xanthostoma* (Sommerf.) Fr., *Porina xanthostoma* Sommerf.

L – Subs.: cor, deb, sil – Alt.: 4–5 – Note: on bark, but also on plant debris, more rarely on siliceous rocks in upland areas. The Italian records are rather dubious (see Nimis 2016). – **It**: Lomb, Piem.

***Opegrapha celtidicola* (Jatta) Jatta**

Syn.: Lecanactis lyncea (Sm.) Fr. var. celtidicola Jatta, *Opegrapha betulinoides* B. de Lesd., *Opegrapha thallincola* B. de Lesd., *Opegrapha xylographoides* J. Steiner

L – Subs.: cor, xyl – Alt.: 1–2 – Note: a Mediterranean-Atlantic lichen found on old trees, near the base of the trunks, at relatively low elevations, with a few records from the base of the Western Alps. – **Fr**: AMa. **It**: Lig.

***Opegrapha conferta* Anzi**

Syn.: *Opegrapha confluens* (Ach.) Stizenb. *non auct*.

L – Subs.: sil – Alt.: 1–3 – Note: a mild-temperate to Mediterranean lichen of shaded siliceous rocks. – **Au**: St. **It**: Lomb.

***Opegrapha corticola* Coppins & P. James**

L – Subs.: cor – Alt.: 2 – Note: a western European, mild-temperate species growing on the trunks of ancient trees, especially *Quercus ilex*; easy to overlook, being always sterile, but certainly extremely rare; with a few records from the Western Alps. – **Fr**: AMa, Var, Vau.

***Opegrapha dolomitica* (Arnold) Clauzade & Cl. Roux *ex* Torrente & Egea subsp. dolomitica**

Syn.: Opegrapha rupestris Pers. var. dolomitica Arnold, *Opegrapha saxicola auct. non* Ach.

L – Subs.: cal – Alt.: 1–5 – Note: on vertical or underhanging surfaces of dolomitic rocks and (more rarely) of more or less porous limestone, with a wide altitudinal range; widespread throughout the Alps. – **Au**: V, T, S, K, St, O, N. **Ge**: OB. **Sw**: BE, GR, LU, SZ, UR. **Fr**: AHP, AMa, Drô, Isè, HSav, Vau. **It**: Frl, Ven, TAA, Lomb, Lig. **Sl**: SlA, Tg.

***Opegrapha dolomitica* (Arnold) Clauzade & Cl. Roux *ex* Torrente & Egea subsp. omninocalcicola Cl. Roux**

L # – Subs.: cal – Alt.: 2–3 – Note: a taxon characterised by scarcely umbonate, never gyrose ascomata, whose taxonomic value is in need of evaluation; on compact calcareous rocks, with an ecology resembling that of *Naetrocymbe saxicola*; so far only known from the Western Alps at low elevations, but not consistently distinguished elsewhere in the Alps. – **Fr**: AHP, AMa, Isè, Sav, HSav, Var, Vau.

***Opegrapha lithyrga* Ach.**

Syn.: *Opegrapha lithyrgodes* Nyl.

L – Subs.: sil – Alt.: 2–4 – Note: on vertical to underhanging surfaces of hard siliceous rocks in deep gorges or in mature forests. Closely related to *O. vulgata*. – **Au**: ?V, T, S, K, St, N, B. **Sw**: SZ, UR, VD. **It**: TAA, Lomb, Piem, Lig.

***Opegrapha multipuncta* Coppins & P. James**

L – Subs.: cor – Alt.: 2 – Note: a mild-temperate lichen motly found on fruit-trees in orchards; certainly more widespread in the Alps, but overlooked, being always sterile. – **Fr**: Vau. **It**: Frl.

***Opegrapha niveoatra* (Borrer) J.R. Laundon**

Syn.: *Opegrapha amphotera* Nyl., *Opegrapha dubia auct.*, *Opegrapha subsiderella* (Nyl.) Arnold, Opegrapha vulgata (Ach.) Ach. var. subsiderella Nyl., *Verrucaria niveoatra* Borrer

L – Subs.: cor – Alt.: 1–3 – Note: a mild-temperate lichen found on old trees in open woodlands; closely related to *O. vulgata*, but differing in the shorter spermogonia; widespread throughout the Alps, but generally not very common. – **Au**: V, T, S, K, St, O, N. **Ge**: OB. **Sw**: BE, FR, GL, GR, LU, SZ, TI, UR, UW, VD, VS. **Fr**: AHP, AMa, Var, Vau. **It**: Ven, TAA. **Sl**: SlA, Tg.

***Opegrapha subparallela* Müll. Arg.**

L # – Subs.: xyl – Alt.: 3 – Note: a lignicolous species recalling a black *Xylographa parallela*, perhaps related to *O. ochrocheila*, with an endosubstratic thallus, non-pruinose ascomata, and finally 3-septate ascospores; rare throughout Europe, with a few scattered records from the Alps. – **Au**: S, B. **Fr**: HSav.

***Opegrapha vermicellifera* (Kunze) J.R. Laundon**

Syn.: *Opegrapha fuscella* (Fr.) Almb., *Opegrapha hapaleoides* Nyl., *Opegrapha leptospora* Werner & M. Choisy, *Opegrapha mehdiensis* Werner, *Pyrenothea vermicellifera* Kunze

L – Subs.: cor – Alt.: 2–3 – Note: a mild-temperate lichen found on old trees in humid areas, especially near large rivers, on faces seldom wetted by rain; widespread throughout the Alps. – **Au**: V, T, S, K, St, O, N, B. **Ge**: OB. **Sw**: BE, GL, GR, SG, UW, VS. **Fr**: AMa, Drô, Isè, HSav, Var, Vau. **It**: Lomb, Piem. **Sl**: SlA, Tg.

***Opegrapha vulgata* (Ach.) Ach.**

Syn.: *Hysterina vulgata* (Ach.) Gray, *Lichen vulgatus* Ach., *Opegrapha cinerea* Chevall., *Opegrapha devulgata* Nyl.

L – Subs.: cor, xyl – Alt.: 1–3 – Note: a widespread, but not common temperate species with optimum in humid forests, especially on *Abies*, but also on broad-leaved trees. – **Au**: V, T, S, K, St, O, N. **Ge**: OB. **Sw**: BE, GL, GR, SG, SZ, UR, UW, VD, VS. **Fr**: AHP, AMa, Isè, HSav, Var, Vau. **It**: Frl, Ven, TAA, Lomb, Piem. **Sl**: SlA, Tg.

***Ophioparma lapponica* (Räsänen) Hafellner & R.W. Rogers**

Syn.: *Haematomma lapponicum* Räsänen, Haematomma ventosum (L.) A. Massal. var. lapponicum (Räsänen) Lynge

L – Subs.: sil – Alt.: 3–6 – Note: a species with narrowly ellipsoid (not acicular!) ascospores which are simple to 1-septate at maturity; on siliceous rocks in windy situations; widespread and often common in the Arctic, very rarely reported from the orobiomes of the temperate zone, including the Alps; the few Alpine records (France) urgently need verification. – **Fr**: HSav.

**Ophioparma ventosa (L.) Norman var. ventosa**

Syn.: *Haematomma ventosum* (L.) A. Massal., *Lecanora ventosa* (L.) Ach., *Lepadolemma ventosum* (L.) Trevis., *Lichen ventosus* L., *Zeora ventosa* (L.) Flot.

L – Subs.: sil – Alt.: 3–6 – Note: an arctic-alpine circumpolar lichen found on steeply inclined surfaces of siliceous rocks in wind-exposed situations, with optimum above treeline; widespread throughout the siliceous Alps. – **Au**: V, T, S, K, St, N. **Ge**: Ge. **Sw**: BE, GR, LU, TI, UR, VD, VS. **Fr**: HAl, AMa, Isè, Sav, HSav. **It**: Frl, Ven, TAA, Lomb, Piem, VA.

**Ophioparma ventosa (L.) Norman var. cuprigena (Poelt) Hafellner & R.W. Rogers**

Syn.: Haematomma ventosum (L.) A. Massal. var. cuprigenum Poelt

L – Subs.: met – Alt.: 4 – Note: a variety with a thin thallus lacking thamnolic acid in the medulla (and therefore reacting K-), and apothecia recalling those of a *Caloplaca*, with blood red discs and upper portions of the thalline margin; on copper-rich schists; rare in Northern and Central Europe, with a single record from the Eastern Alps (Austria). – **Au**: S.

***Orphniospora moriopsis* (A. Massal.) D. Hawksw.**

Syn.: *Buellia atrata* (Sm.) Anzi, *Buellia coracina* Körb., *Buellia moriopsis* (A. Massal.) Th. Fr., *Buellia subtenebrosa* Malme, *Catolechia moriopsis* A. Massal., *Lecanora coracina* (Hoffm.) Hepp, *Orphniospora atrata* (Sm.) Poelt, Sporastatia testudinea (Ach.) A. Massal. var. coracina (Hoffm.) Bagl. & Carestia

L – Subs.: sil – Alt.: 4–6 – Note: an arctic-alpine circumpolar lichen found on inclined surfaces of hard siliceous rocks in cold habitats near or above treeline. – **Au**: V, T, S. **Ge**: Schw. **Sw**: GR, VS. **Fr**: AMa, HSav. **It**: Frl, TAA, Lomb, Piem.

***Orphniospora mosigii* (Körb.) Hertel & Rambold**

Syn.: *Aspicilia obscurissima* (Nyl.) Maheu & A. Gillet, *Lecidea mosigii* (Körb.) Anzi, *Lecidea obscurissima* (Nyl.) Nyl., *Lecidella mosigii* Körb.

L – Subs.: sil – Alt.: 4–6 – Note: on steeply inclined surfaces of wind-exposed, hard siliceous rocks in upland areas; widespread throughout the siliceous Alps. – **Au**: V, T, S, K, St. **Ge**: Schw. **Sw**: BE, GR, TI, UR, VS. **Fr**: AHP, HAl, AMa, Isè, Sav, HSav. **It**: Frl, Ven, TAA, Lomb, Piem, VA.

***Pachnolepia pruinata* (Pers.) Frisch & G. Thor**

Syn.: *Arthonia impolita* (Hoffm.) Borrer, *Arthonia pruinata* (Pers.) A.L. Sm., *Arthonia pruinosa* Ach., *Pachnolepia impolita* (Hoffm.) A. Massal., *Patellaria pruinata* Pers.

L – Subs.: xyl, cor – Alt.: 1–3 – Note: a mild-temperate lichen found on isolated, old deciduous trees, especially oaks, in parts of the boles seldom wetted by rain; rare in the Alps, and restricted to areas with a rather humid climate. – **Au**: S. **Ge**: OB. **It**: Ven, Lomb.

***Palicella filamentosa* (Stirt.) Rodr. Flakus & Printzen**

Syn.: *Lecanora filamentosa* (Stirt.) Elix & Palice, *Lecanora ramulicola* (H. Magn.) Printzen & P.F. May, *Lecidea filamentosa* Stirt., *Lecidea hercynica* M. Hauck & Schmull, *Lecidea ramulicola* (H. Magn.) Hillm. *non* H. Magn. (1952) *nec* H. Magn. (1953), Lecidea saepincola Ach. var. ramulicola H. Magn.

L – Subs.: xyl, cor – Alt.: 3 – Note: a species with an endosubstratic to rimose-areolate thallus reacting K+ yellow (atranorin), and ochre to blackish-grey apothecia with persisting biatorine margins; mainly on wood, but also on bark in montane coniferous forests; certainly more widespread in the Alps, but the distribution is poorly known due to frequent misidentifications. – **Au**: S, St. **Ge**: OB.

***Pannaria conoplea* (Ach.) Bory**

Syn.: *Pannaria caeruleobadia* A. Massal. *nom.illeg.*, *Pannaria lanuginosaauct.*, *Pannaria pityrea* *sensu* Degel. *non* DC., *Parmelia conoplea* Ach., *Trachyderma caeruleobadium* (Mudd) Trevis.

L – Subs.: cor, xyl, bry-sil – Alt.: 1–4 – Note: a *Lobarion*-species most common on mossy bark in open, humid forests, sometimes on mossy siliceous rocks; widespread throughout the Alps, but generally not common. – **Au**: V, T, S, K, St, O, N. **Ge**: OB. **Sw**: BE, GL, GR, SZ, TI, UR, UW, VD, VS. **Fr**: AHP, HAl, AMa, Isè, Sav, HSav, Var, Vau. **It**: Frl, Ven, TAA, Lomb, Piem, VA. **Sl**: SlA, Tg.

***Pannaria hookeri* (Borrer *ex* Sm.) Nyl.**

Syn.: *Lichen hookeri* Borrer *ex* Sm., *Pannaria glacialis* Anzi, *Pannaria leucolepis* (Wahlenb.) Nyl.

L – Subs.: int, sil, ter-cal – Alt.: 4–6 – Note: on slightly calciferous soil (mostly deriving from metamorphic rocks) in sites with periodical water seepage, sometimes also directly on rock, with optimum near treeline; widespread, but generally not common in the Alps. – **Au**: V, T, S, K, St. **Sw**: BE, GR, VS. **Fr**: HSav. **It**: Frl, TAA, Lomb, Piem, VA.

***Pannaria rubiginosa* (Ach.) Bory**

Syn.: *Lichen affinis* Dicks., *Lichen rubiginosus* Ach., *Parmelia rubiginosa* (Ach.) Ach.

L – Subs.: cor – Alt.: 1–3 – Note: restricted to rainy-humid areas, mostly on old mossy trunks in forests; strongly declining throughout the Alps. – **Au**: V, T, S, St, O, N. **Ge**: OB. **Sw**: BE, FR, GR, UR, VD, VS. **Fr**: Isè, Sav, HSav, Var. **It**: Frl, Ven, TAA, Lomb. **Sl**: SlA.

***Parabagliettoa cyanea* (A. Massal.) Gueidan & Cl. Roux**

Syn.: *Involucrothele limitata* (Nyl.) Servít, *Thelidium limitatum* (Nyl.) Servít, *Verrucaria cyanea* A. Massal., *Verrucaria decussata* Garov., *Verrucaria dufourii* DC. v. *limitata* Nyl., *Verrucaria limitata* Kremp. *ex* A. Massal. *nom.illeg*.

L – Subs.: cal – Alt.: 1–3 – Note: on steeply inclined surfaces of compact limestone and dolomite in sheltered situations, with optimum in the submediterranean belt; the species differs from *P. dufourii* in the smaller, less prominent perithecia, and in the thalli typically forming mosaics, with conspecific thalli separated by dark lines; the lines produced by one thallus typically do not merge completely with that of its neighbour, so that the lines appear as double. – **Au**: T, O, N. **Ge**: OB. **Sw**: GR, ?SZ, UR, UW. **Fr**: AHP, AMa, Sav, HSav, Var, Vau. **It**: Frl, Ven, TAA, Lomb, Piem. **Sl**: SlA.

***Parabagliettoa disjuncta* (Arnold) Krzewicka**

Syn.: *Verrucaria disjuncta* Arnold, Verrucaria tristis (A. Massal.) Kremp. f. depauperata (A. Massal.) Arnold

L – Subs.: cal – Alt.: 3–6 – Note: a formerly very poorly understood species growing on inclined to vertical surfaces of calcareous rocks; probably more widespread in the Alps. – **Au**: St, O. **Ge**: Ge. **Sw**: SZ. **Fr**: AHP, HAl, AMa, Isè, Sav, HSav, Vau. **It**: Ven.

***Parabagliettoa dufourii* (DC.) Gueidan & Cl. Roux**

Syn.: ?*Involucrothele concinna* (Borrer) Servít, *Involucrothele dufourii* (DC.) Servít, *Thelidium dufourii* (DC.) Servít, Thelidium dufourii (DC.) Servít f. rehmii Servít, Thelidium dufourii (DC.) Servít var. alpinum Servít, Thelidium dufourii (DC.) Servít var. lilacinofuscum Kremp. *ex* Servít, Thelidium dufourii (DC.) Servít var. mojstranense Servít, ?*Verrucaria concinna* Borrer, *Verrucaria dufourii* DC., *Verrucaria malhamensis* Nyl.

L – Subs.: cal, int – Alt.: 1–6 – Note: on steeply inclined surfaces of hard calcareous rocks, mainly limestone, in rather shaded situations, with a wide altitudinal range, reaching the eu-Mediterranean belt in particularly humid and shaded stands; widespread throughout the Alps. – **Au**: V, T, S, K, St, O, N. **Ge**: OB, Schw. **Sw**: BE, GR, SZ, VD. **Fr**: AHP, AMa, Drô, Isè, Sav, HSav, Var, Vau. **It**: Frl, Ven, TAA, Lomb, Piem, Lig. **Sl**: SlA, Tg.

***Paracollema italicum* (B. de Lesd.) Otálora, P.M. Jørg. & Wedin**

Syn.: *Collema italicum* B. de Lesd.

L – Subs.: cor – Alt.: 1 – Note: a mild-temperate, Mediterranean-Atlantic species of humid sites, on trees such as *Olea* and *Quercus ilex*, with a few records from the base of the Western Alps. – **Fr**: AMa, Var. **It**: Lig.

***Parainoa subconcolor* (Anzi) Resl & T. Sprib.**

Syn.: *Biatora subconcolor* Anzi, *Lecidea subconcolor* (Anzi) Jatta, *Trapelia subconcolor* (Anzi) Hertel, *Trapeliopsis subconcolor* (Anzi) Hertel

L – Subs.: sil – Alt.: 3–4 – Note: a rarely collected, subtropical species found on basic siliceous rocks in sheltered situations, mostly in upland areas; in the study area so far only reported from the Southern Alps (Italy). – **It**: TAA, Lomb.

***Paralecanographa grumulosa* (Dufour) Ertz & Tehler**

Syn.: *Chiodecton spilocarpon* Nyl., *Lecanactis doerfleri* Zahlbr., *Lecanactis grumulosa* (Dufour) Fr., Lecanactis grumulosa (Dufour) Fr. var. monstrosa (Bagl.) Egea & Torrente, *Lecanactis monstrosa* Bagl., *Lecanactis nothiza* (Nyl.) P. James, *Lecanactis pictonica* (Nyl.) H. Olivier, *Lecanographa grumulosa* (Dufour) Egea & Torrente, *Lecidea pictonica* Nyl., ?*Opegrapha cavernicola* Llimona & Werner, *Opegrapha diaphoroides* Nyl. *nonauct.*, *Opegrapha dirinaria* (Nyl.) Nyl., *Opegrapha grumulosa* Dufour, Opegrapha grumulosa Dufour var. platycarpa Nyl., *Opegrapha platycarpa* (Nyl.) Nyl.; incl. Lecanographa grumulosa (Dufour) Egea & Torrente var. monstrosa (Bagl.) Egea & Torrente

L – Subs.: sil – Alt.: 1–4 – Note: on steeply inclined to underhanging surfaces of more or less calcareous cliffs subject to humid winds in rather shaded situations. – **Au**: T, K. **Fr**: AHP, AMa, Var, Vau. **It**: Ven, Lomb, Lig.

***Parmelia barrenoae* Divakar, M.C. Molina & A. Crespo**

L – Subs.: cor, sil – Alt.: 2–3 – Note: mostly epiphytic, more rarely on siliceous rocks; the distribution in the Alps is very poorly known because it was almost never distinguished from *P. sulcata*. – **Fr**: AHP, HAl.

***Parmelia ernstiae* Feuerer & A. Thell**

L – Subs.: cor – Alt.: 2–3 – Note: this mainly epiphytic species differs from *P. saxatilis* in the strongly pruinose thallus and isidia, and in molecular characters; it is likely to be more widespread in the Alps, but in the past it was often confused with *P. saxatilis*. – **Au**: St, O. **Ge**: Schw. **Sw**: SZ. **Sl**: SlA.

**Parmelia omphalodes (L.) Ach. subsp. omphalodes**

Syn.: Imbricaria saxatilis (L.) Körb. var. omphalodes (L.) Körb., *Lichen omphalodes* L., Parmelia saxatilis (L.) Ach. var. omphalodes (L.) Fr.

L – Subs.: sil, cor, bry – Alt.: 2–6 – Note: an arctic-alpine, circumpolar lichen found on rocks, epilithic bryophytes, more rarely on soil, mostly near or above treeline; widespread throughout the Alps; several records could refer to the other subspecies. – **Au**:  V, T, S, K, St, N. **Ge**: Ge. **Sw**: BE, GR, LU, SZ, TI, UR, VD, VS. **Fr**: HAl, AMa, Isè, Sav, HSav. **It**: Frl, Ven, TAA, Lomb, Piem, VA, Lig. **Sl**: SlA.

**Parmelia omphalodes (L.) Ach. subsp. pinnatifida (Kurok.) Skult**

Syn.: Parmelia omphalodes (L.) Ach. var. panniformis Ach., *Parmelia pinnatifida* Kurok.

L # – Subs.: sil – Alt.: 3–5 – Note: a name applied to morphs with thalli growing in several layers, with narrow, richly branched, mostly concave lobes, the medulla reacting K+ red and Pd+ orange (salazinic acid plus an otherwise complex secondary chemistry); mostly on siliceous rocks; more common in Northern Europe at high elevations; in the Alps not consistently distinguished, so that the distribution is poorly known. – **Au**: ?V, T, S, K. **Fr**: Sav, HSav.

***Parmelia saxatilis* (L.) Ach.**

Syn.: *Imbricaria saxatilis* (L.) Körb., *Lichen saxatilis* L.

L – Subs.: sil, cor, xyl, deb, bry-sil, ter – Alt.: 1–5 – Note: a often collected, mainly saxicolous lichen occurring in large parts of the world, which for centuries has been regarded as a well-delimited species. Recently, however, it has been found that some morphologically deviating specimens may be regarded as distinct species, such as *P. ernstiae, P. serrana* and *P. squarrosa*; widespread throughout the Alps. – **Au**: V, T, S, K, St, O, N, B. **Ge**: OB, Schw. **Sw**: AP, BE, FR, GL, GR, LU, SG, SZ, TI, UR, UW, VD, VS. **Fr**: AHP, HAl, AMa, Drô, Isè, Sav, HSav, Var, Vau. **It**: Frl, Ven, TAA, Lomb, Piem, VA, Lig. **Sl**: SlA, Tg. **Li**.

***Parmelia serrana* A. Crespo, M.C. Molina & D. Hawksw.**

L – Subs.: sil, cor – Alt.: 3–4 – Note: this recently described species, which is morphologically very similar to *P. saxatilis*, seems to be widespread in Southern Europe, especially in areas with a subcontinental climate; its distribution in the Alps is poorly known, as in the past it was not distinguished from *P. saxatilis*. – **Au**: T, St. **Sw**: SZ. **It**: Frl.

***Parmelia squarrosa* Hale**

L – Subs.: sil – Alt.: 4–5 – Note: this species, which is fairly common in North America and in Japan, also occurs, albeit very rarely, in the Alps. – **Au**: S. **Sw**: GR, VS. **It**: Frl.

***Parmelia submontana* Hale**

Syn.: *Parmelia bohemica* Nádv. *non* Gyeln., *Parmelia contorta* Bory *non* (Hoffm.) Spreng., Parmelia saxatilis (L.) Ach. var. contorta (Bory) Zahlbr.

L – Subs.: cor – Alt.: 2–3 – Note: on the trunks of old trees (mainly *Fagus* and *Abies*) in humid montane forests; widespread throughout the Alps. – **Au**: V, T, S, K, St, O, N. **Ge**: OB, Schw. **Sw**: BE, GL, GR, LU, SG, SZ, TI, UR, UW. **Fr**: AHP, AMa, Drô, Isè, HSav, Var, Vau. **It**: Frl, Ven, TAA, VA, Lig. **Sl**: SlA, Tg. **Li**.

***Parmelia sulcata* Taylor**

Syn.: Parmelia saxatilis (L.) Ach. var. sulcata (Taylor) Nyl., Parmelia sulcata Taylor var. laevis Nyl.

L – Subs.: cor, xyl, sil, deb, bry, ter-cal – Alt.: 1–5 – Note: on acid or subacid bark, exceptionally also on wood; this is certainly the most common and wide-ranging *Parmelia* in the Alps, also present near large urban settlements, rare only in the eu-Mediterranean belt. See also note on *P. barrenoae*. – **Au**: V, T, S, K, St, O, N, B. **Ge**: OB, Schw. **Sw**: AP, BE, FR, GL, GR, LU, SG, SZ, TI, UR, UW, VD, VS. **Fr**: AHP, HAl, AMa, Drô, Isè, Sav, HSav, Var, Vau. **It**: Frl, Ven, TAA, Lomb, Piem, VA, Lig. **Sl**: SlA, Tg. **Li**.

***Parmeliella testacea* P.M. Jørg.**

L – Subs.: cor – Alt.: 1–3 – Note: a mild-temperate lichen found on bark of ancient deciduous trees in mature forests, mostly in old plantations of *Castanea*; apparently restricted to the Southern and Western Alps. – **Fr**: AMa, Var. **It**: Lomb, Lig.

***Parmeliella triptophylla* (Ach.) Müll. Arg.**

Syn.: *Lecanora triptophylla* (Ach.) Link, Lecidea microphylla (Lilj.) Ach. var. schraderi Schaer., *Lecidea triptophylla* Ach., *Lecothecium corallinoides* (Hoffm.) Körb., *Pannaria lasiella* Stirt., *Pannaria triptophylla* (Ach.) A. Massal., *Parmeliella corallinoides* (Hoffm.) Zahlbr.

L – Subs.: cor, sil, bry, ter-bry – Alt.: 1–5 – Note: a widespread, cool-temperate to boreal-montane lichen found on old trees and upon epiphytic bryophytes in humid forests, sometimes also on mossy siliceous rocks, with a wide altitudinal range; widespread throughout the Alps. – **Au**: V, T, S, K, St, O, N. **Ge**: OB, Schw. **Sw**: BE, FR, GL, GR, SG, SZ, UR, UW, VD, VS. **Fr**: AHP, AMa, Drô, Isè, Sav, HSav, Vau. **It**: Frl, Ven, TAA, Lomb, Piem, VA, Lig. **Sl**: SlA, Tg. **Li**.

***Parmelina atricha* (Nyl.) P. Clerc**

Syn.: *Parmelia atricha* Nyl., Parmelia quercina (Willd.) Vain. var. convoluta (Schaer.) Zahlbr.

L – Subs.: sil – Alt.: 2–3 – Note: a saxicolous species described from the Eastern Pyrenees, so far also known from Southern France, Northern Italy and Southern Switzerland, with a mainly submediterranean-montane distribution. It grows on siliceous rocks in more or less exposed, dry areas, with optimum in the montane belt. – **Sw**: GR, VS. **Fr**: AMa. **It**: TAA, Lomb.

***Parmelina carporrhizans* (Taylor) Poelt & Vězda**

Syn.: *Parmelia carporrhizans* Taylor, Parmelia quercina (Willd.) Vain. var. carporrhizans (Taylor) V. Wirth, Parmelia tiliacea (Hoffm.) Ach. subsp. carporrhizans (Taylor) Nyl., Parmelia tiliacea (Hoffm.) Ach. var. carporrhizans (Taylor) Flagey

L – Subs.: cor – Alt.: 1–3 – Note: a mild-temperate to Mediterranean lichen found on isolated, mostly broad-leaved trees, more photo – and thermo-, and less hygrophytic than the closely related *P. pastillifera* and *P. tiliacea*. The species was not always distinguished from *P. quercina* in the old literature. – **Au**: V. **Ge**: OB, Schw. **Sw**: GL, GR, SZ, TI, UR, UW, VS. **Fr**: AHP, HAl, AMa, Drô, Isè, Sav, Var, Vau. **It**: Frl, TAA, Piem. **Sl**: SlA.

***Parmelina pastillifera* (Harm.) Hale**

Syn.: *Parmelia pastillifera* (Harm.) R. Schub. & Klem., Parmelia scortea (Ach.) Ach. var. pastillifera Harm., Parmelia tiliacea (Hoffm.) Ach. var. pastillifera (Harm.) Grummann

L – Subs.: cor, xyl – Alt.: 2–4 – Note: a temperate lichen found on deciduous trees with subacid to subneutral bark, more frequent in rainy upland areas than *P. tiliacea*; widespread and locally common throughout the Alps. – **Au**: V, T, S, K, St, O, N. **Ge**: OB, Schw. **Sw**: BE, FR, GL, GR, LU, SG, SZ, TI, UR, UW, VD, VS. **Fr**: AHP, AMa, Drô, Sav, HSav, Var, Vau. **It**: Frl, Ven, TAA, Lomb, Piem, VA. **Sl**: SlA, Tg. **Li**.

***Parmelina quercina* (Willd.) Hale**

Syn.: *Lichen quercinus* Willd., *Parmelia quercina* (Willd.) Vain.

L – Subs.: cor – Alt.: 2–4 – Note: a mild-temperate lichen found on isolated, mostly broad-leaved trees. See also note on *P. carporrhizans*. – **Au**: V, T, S, K, St, O, N. **Ge**: Schw. **Sw**: BE, GL, GR, LU, SG, SZ, TI, UR, UW, VS. **Fr**: AHP, AMa, Drô, Isè, Sav, HSav, Vau. **It**: Frl, Ven, TAA, Lomb, Piem, VA. **Sl**: SlA. **Li**.

***Parmelina tiliacea* (Hoffm.) Hale**

Syn.: *Imbricaria tiliacea* (Hoffm.) Flot., *Lichen tiliaceus* Hoffm., *Parmelia scortea* (Ach.) Ach., *Parmelia tiliacea* (Hoffm.) Ach., Parmelia tiliacea (Hoffm.) Ach. var. scortea (Ach.) Duby

L – Subs.: cor, xyl, sil, bry-sil – Alt.: 1–4 – Note: a mainly mild-temperate lichen mostly found on broad-leaved trees, sometimes on mossy rocks; widespread and often common throughout the Alps, rare only in somehow continental areas. – **Au**: V, T, S, K, St, O, N, B. **Ge**: OB, Schw. **Sw**: AP, BE, FR, GL, GR, LU, SG, SZ, TI, UR, UW, VD, VS. **Fr**: AHP, HAl, AMa, Drô, Isè, Sav, HSav, Var, Vau. **It**: Frl, Ven, TAA, Lomb, Piem, VA, Lig. **Sl**: SlA, Tg. **Li**.

***Parmeliopsis ambigu* a (Hoffm.) Nyl.**

Syn.: *Foraminella ambigu*a (Hoffm.) S.L.F. Mey., *Lichen ambiguus* Wulfen *nom.illeg.*, *Parmelia ambigua* (Hoffm.) Ach., *Parmelia diffusa auct. non* (Hoffm.) Sandst., *Parmelia subsoredians* Nyl., *Parmeliopsis diffusa auct.*, *Parmeliopsis subsoredians* (Nyl.) Nyl., *Squamaria ambigua* Hoffm.

L – Subs.: cor, xyl, sax – Alt.: 2–5 – Note: a mainly boreal-montane, circumpolar lichen found on basal parts of trunks, especially of conifers, with a long snow cover; widespread and common in the Alps, with optimum in the subalpine belt. – **Au**: V, T, S, K, St, O, N, B. **Ge**: OB, Schw. **Sw**: AP, BE, FR, GL, GR, LU, SG, SZ, TI, UR, UW, VD, VS. **Fr**: AHP, HAl, AMa, Isè, Sav, HSav, Var, Vau. **It**: Frl, Ven, TAA, Lomb, Piem, VA, Lig. **Sl**: SlA, Tg. **Li**.

***Parmeliopsis hyperopta* (Ach.) Arnold**

Syn.: *Foraminella hyperopta* (Ach.) S.L.F. Mey., Imbricaria ambigua (Hoffm.) DC. var. albescens (Wahlenb.) Flot., *Imbricaria hyperopta* (Ach.) Körb., *Parmelia ambigua* (Hoffm.) Ach. var. albescens (Wahlenb.) Schaer., Parmelia diffusa (Weber) Rebent. var. albescens (Wahlenb.) Rabenh., *Parmelia hyperopta* Ach.

L – Subs.: cor, xyl, bry, sil – Alt.: 2–5 – Note: a mainly boreal-montane, circumpolar lichen found on basal parts of trunks, especially of conifers, with a long snow cover; ecology and distribution resemble those of *P. ambigua*, but this lichen is slightly less photo – and more hygrophytic; widespread throughout the Alps. – **Au**: V, T, S, K, St, O, N, B. **Ge**: OB, Schw. **Sw**: AP, BE, FR, GL, GR, LU, SZ, TI, UR, UW, VD, VS. **Fr**: AHP, HAl, AMa, Isè, Sav, HSav, Var, Vau. **It**: Frl, Ven, TAA, Lomb, Piem, VA, Lig. **Sl**: SlA, Tg. **Li**.

***Parmotrema arnoldii* (Du Rietz) Hale**

Syn.: *Parmelia arnoldii* Du Rietz, *Parmelia subarnoldii* Abbayes

L – Subs.: cor, xyl – Alt.: 2–4 – Note: a cool-temperate to tropical lichen restricted to humid beech forests; it frequently grows in the upper branches of the tree canopy in forests with frequent fog, and therefore it often goes unnoticed. – **Au**: V, T, S, K, St, O, N. **Ge**: OB. **Sw**: BE, GL, GR, LU, SZ, UR, UW. **It**: Frl, Ven. **Sl**: SlA.

***Parmotrema crinitum* (Ach.) M. Choisy**

Syn.: *Parmelia ciliata* Nyl., *Parmelia crinita* Ach., *Parmelia excrescens* (Arnold) Hav., Parmelia excrescens (Arnold) Hav. var. pilosella (Hue) Lynge, *Parmelia pilosella* Hue, *Parmelia proboscidea* Taylor

L – Subs.: cor, sil – Alt.: 2–3 – Note: a cool-temperate lichen found on bark in open humid montane forests, rarely on epilithic bryophytes, exceptionally descending to the submediterranean belt in very humid areas; widespread throughout the Alps, but generally not common. – **Au**: V, T, S, K, St, O, N. **Ge**: OB, Schw. **Sw**: BE, GL, GR, LU, SG, SZ, TI, UR, UW. **Fr**: AMa, Var. **It**: Frl, Ven, TAA, Lomb, Piem, VA. **Sl**: SlA.

***Parmotrema perlatum* (Huds.) M. Choisy**

Syn.: *Imbricaria perlata* (Huds.) Körb., *Lichen chinensis* Osbeck, *Lichen perlatus* Huds., *Parmelia coniocarpa* Laurer, *Parmelia perlata* Ach. *nom.illeg.*, *Parmelia trichotera* Hue, *Parmotrema chinense* (Osbeck) Hale & Ahti, *Parmotrema trichoterum* (Hue) M. Choisy

L – Subs.: cor, xyl, sil – Alt.: 1–3 – Note: a mainly mild-temperate lichen found on bark and mossy siliceous rocks, on isolated trees only in humid areas, otherwise in light woodlands and restricted to the mossy base of trunks, exceptionally reaching the dry-continental valleys of the Alps in sheltered situations; widespread throughout the Alps. – **Au**: V, T, S, K, St, O, N, B. **Ge**: OB. **Sw**: BE, GL, GR, LU, SG, SZ, TI, UR, UW, VD, VS. **Fr**: AHP, AMa, Drô, Isè, Sav, HSav, Var, Vau. **It**: Frl, Ven, TAA, Lomb, Piem, VA, Lig. **Sl**: SlA, Tg.

***Parmotrema reticulatum* (Taylor) M. Choisy**

Syn.: *Parmelia ciliata* (DC.) Nyl., *Parmelia concors* Kremp., *Parmelia pseudoreticulata* Tav., *Parmelia reticulata* Taylor, *Parmotrema pseudoreticulatum* (Tav.) Hale, *Parmotrema pseudovirens* (Gyeln.) Elix, *Rimelia reticulata* (Taylor) Hale & A. Flechter

L – Subs.: cor – Alt.: 1–3 – Note: a Mediterranean-Atlantic to mild-temperate lichen found on bark, rarely on mossy siliceous rocks; very rare and declining in the Alps. – **Sw**: GR, TI, UR. **Fr**: AMa, HSav, Var. **It**: Frl, Lomb, Piem. **Sl**: SlA.

***Parmotrema stuppeum* (Taylor) Hale**

Syn.: *Parmelia claudelii* (Harm.) Vain., *Parmelia maxima* Hue, *Parmelia stuppea* Taylor, Parmelia trichotera Hue var. claudelii (Harm.) Du Rietz

L – Subs.: cor, bry – Alt.: 2–3 – Note: a mainly mild-temperate species found in open woodlands with frequent fog, mostly on ancient trees, but also on epilithic bryophytes; rather rare, and certainly declining in the Alps. – **Au**: S, St, O, N. **Ge**: OB, Schw. **Sw**: GL, GR, LU, TI, UR, UW. **It**: Lig.

***Paulia glomerata* Henssen & Tretiach**

L – Subs.: cal – Alt.: 2–4 – Note: on steeply inclined surfaces of calcareous rocks in rainy areas, mostly starting its life-cycle in fissures of the rocks; hitherto known only from the Eastern Alps, to be looked for elsewhere. – **Au**: O. **It**: Frl. **Sl**: SlA.

***Paulia salevensis* (Müll. Arg.) M. Schultz**

Syn.: *Peccania salevensis* (Müll. Arg.) Forssell, *Synalissa salevensis* Müll. Arg.

L # – Subs.: cal – Alt.: 4 – Note: a species with an irregularly fruticose thallus bearing cylindrical, pruinose lobes, terminal apothecia lacking a proper exciple, and prototunicate asci containing rather thick-walled ascospores; on steep faces of calcareous rocks; known with certainty only from the type locality in the Western Alps (France), other records need confirmation. – **Au**: K. **Fr**: HSav.

***Peccania cernohorskyi* (Servít) Czeika & Guttová**

Syn.: *Anema cernohorskyi* (Servít) Henssen, *Thyrea cernohorskyi* Servít

L – Subs.: cal, int – Alt.: 3 – Note: a fertile species with minute subfruticose to peltate thalli covered by verrucose outgrowths, hymenia of an orange-brown colour in the upper part, and filiform conidia; on thin layers of soil or directly on basic siliceous rocks; rare in Central Europe, additional records from Scandinavia on calcareous rocks could not be confirmed; the few records from the Western Alps (France) are from calcareous rocks, and therefore need confirmation. – **Fr**: AHP, AMa.

***Peccania coralloides* (A. Massal.) Arnold**

Syn.: *Corinophorus coralloides* A. Massal., *Omphalaria coralloides* (A. Massal.) Hepp

L – Subs.: cal – Alt.: 1–5 – Note: on steeply inclined, usually south-exposed seepage tracks of calciferous rocks, with a wide altitudinal range; widespread throughout the Alps. – **Au**: ?V, T, S, K, St, N. **Sw**: BE, GR, VD, VS. **Fr**: AHP, HAl, AMa, Sav, HSav, Var, Vau. **It**: Frl, Ven, TAA, Lomb, Piem, Lig.

***Pectenia atlantica* (Degel.) P.M. Jørg., L. Lindblom, Wedin & S. Ekman**

Syn.: *Degelia atlantica* (Degel.) P.M. Jørg. & P. James, *Parmeliella atlantica* Degel.

L – Subs.: cor – Alt.: 1–3 – Note: a mild-temperate lichen, mainly western in Europe, found in moist-warm stands. The species has been frequently confused with isidiate forms of *P. plumbea* (see Roux et al. 2017), and the record from Slovenia needs re-confirmation. – **Sl**: ?Tg.

***Pectenia plumbea* (Lightf.) P.M. Jørg., L. Lindblom, Wedin & S. Ekman**

Syn.: *Coccocarpia plumbea* (Lightf.) Nyl., *Degelia plumbea* (Lightf.) P.M. Jørg. & P. James, *Lichen plumbeus* Lightf., *Pannaria plumbea* (Lightf.) Bory, *Parmeliella plumbea* (Lightf.) Vain.

L – Subs.: cor – Alt.: 1–3 – Note: a mild-temperate lichen with oceanic affinities found on base-rich, often mossy bark of old trees, more rarely on mossy rocks in rainy-humid areas, mostly in *Lobarion*-communities; apparently more frequent in the Southern and Western Alps. – **Sw**: ?GR. **Fr**: HAl, AMa, Sav, Var, Vau. **It**: Frl, Ven, TAA. **Sl**: SlA, Tg.

***Peltigera aphthosa* (L.) Willd.**

Syn.: *Lichen aphthosus* L., *Peltidea aphthosa* (L.) Ach.

L – Subs.: ter-sil, ter-cal, bry – Alt.: 3–6 – Note: a mainly boreal-montane, circumpolar acidophytic vicariant of *P. leucophlebia*, found on terricolous mosses and soil rich in humus, mostly in forests, but also above treeline; it was not always distinguished from *P. leucophlebia* in the older literature; widespread throughout the Alps. – **Au**: V, T, S, K, St, O, N. **Ge**: OB. **Sw**: BE, FR, GR, LU, SG, SZ, UR, VD, VS. **Fr**: AHP, HAl, AMa, Isè, Sav, HSav. **It**: Frl, Ven, TAA, Lomb, Piem, VA. **Sl**: SlA, Tg.

***Peltigera canina* (L.) Willd.**

Syn.: *Lichen caninus* L., *Peltidea canina* (L.) Ach., *Peltidea leucorrhiza* Flörke *nom.illeg.*, *Peltigera suomensis* Gyeln.

L – Subs.: bry, deb, ter-cal, ter-sil – Alt.: 1–5 – Note: a widespread holarctic species found on terricolous mosses and soil in open forests and heathlands, sometimes on bark in the basal parts of old trees; certainly rarer than *P. praetextata*, but widespread and locally common in the Alps, with a wide altitudinal range; it was often confused with *P. membranacea* and *P. praetextata* in the older literature. – **Au**: V, T, S, K, St, O, N, B. **Ge**: OB, Schw. **Sw**: BE, GL, GR, LU, SG, SZ, TI, UR, UW, VD, VS. **Fr**: AHP, HAl, AMa, Isè, Sav, HSav, Var, Vau. **It**: Frl, Ven, TAA, Lomb, Piem, VA, Lig. **Sl**: SlA, Tg.

***Peltigera collina* (Ach.) Schrad.**

Syn.: *Lichen collinus* Ach., *Peltidea collina* (Ach.) Röhl., *Peltigera limbata* Delise *ex* Hepp, *Peltigera molesta* Delise *ex* Duby, *Peltigera perfida* Gyeln., *Peltigera propagulifera* (Flot. *ex* Körb.) Stein, *Peltigera scutata* (Dicks.) Duby, *Peltigera sibirica* Gyeln., *Peltigera subscutata* Gyeln.

L – Subs.: cor, bry, sil, bry-sil – Alt.: 2–4 – Note: a typical *Lobarion*-species found on the mossy bark of old deciduous trees in humid, open forests, sometimes on epilithic mosses, with optimum in the montane belt; widespread throughout the Alps, but generally not very common, and perhaps declining. – **Au**: V, T, S, K, St, O, N. **Ge**: OB, Schw. **Sw**: BE, FR, GL, GR, SG, SZ, TI, UR, UW, VD, VS. **Fr**: AHP, AMa, Isè, Sav, HSav, Var, Vau. **It**: Frl, Ven, TAA, Lomb, Piem, VA, Lig. **Sl**: SlA, Tg.

***Peltigera degenii* Gyeln.**

Syn.: *Peltigera nitens* (Anders) Gyeln., *Peltigera praetextata* (Flörke *ex* Sommerf.) Zopf var. nitens (Anders) Szatala, *Peltigera virescens* (J. Steiner) Gyeln.

L – Subs.: ter-sil, ter-cal, bry, deb – Alt.: 2–4 – Note: a temperate to southern boreal species found on terricolous bryophytes, on soil rich in humus and on mossy rocks in forests, sometimes on bark in the basal parts of trunks, with optimum in the montane belt; widespread throughout the Alps. – **Au**: V, T, S, K, St, O, N. **Ge**: Schw. **Sw**: BE, FR, GL, GR, LU, SG, SZ, UR, UW, VD, VS. **Fr**: HAl, Isè, Sav, HSav, Vau. **It**: Frl, Ven, TAA, Lomb, Piem, Lig. **Sl**: SlA, Tg.

***Peltigera didactyla* (With.) J.R. Laundon**

Syn.: *Lichen didactylus* With., *Lichen spurius* Ach., *Peltidea erumpens* Taylor, *Peltigera erumpens* (Taylor) Lange, *Peltigera hazslinszky* Gyeln., *Peltigera leptodermaauct.*, *Peltigera monophylla* Opiz, *Peltigera pellucida* (Weber) Gyeln., *Peltigera pusilla* (Fr.) Körb., *Peltigera sorediata* (H. Olivier) Fink, *Peltigera spuria* (Ach.) DC.

L – Subs.: ter-sil, ter-cal, bry-cal, bry-sil – Alt.: 2–6 – Note: a cool-temperate to boreal-montane, ephemeral lichen of disturbed mineral soil, most common near and above treeline; widespread throughout the Alps. – **Au**: V, T, S, K, St, O, N. **Ge**: OB. **Sw**: BE, FR, GR, SG, SZ, TI, UR, VD, VS. **Fr**: AHP, HAl, AMa, Drô, Isè, Sav, HSav, Var, Vau. **It**: Frl, Ven, TAA, Lomb, Piem, VA. **Sl**: SlA.

***Peltigera elisabethae* Gyeln.**

Syn.: *Peltigera mauritzii* Gyeln., *Peltigera microphylla* (Anders) Gyeln.

L – Subs.: ter-cal, bry, deb – Alt.: 3–5 – Note: on terricolous bryophytes and soil rich in humus, with optimum in montane to subalpine forests; widespread throughout the Alps. – **Au**: V, T, S, K, St, O, N. **Ge**: OB, Schw. **Sw**: BE, FR, GL, GR, LU, SG, SZ, TI, UR, UW, VD, VS. **Fr**: AHP, AMa, Isè, Sav, HSav, Vau. **It**: Frl, Ven, TAA, Lomb, Piem, VA, Lig. **Sl**: SlA. **Li**.

***Peltigera extenuata* (Nyl. *ex* Vain.) Lojka**

Syn.: Peltigera canina (L.) Willd. var. extenuata Nyl. *ex* Vain., Peltigera didactyla (With.) J.R. Laundon var. extenuata (Nyl. *ex* Vain.) Goffinet & Hastings

L – Subs.: bry, sil, ter – Alt.: 2–4 – Note: this species has more or less the same ecology as *P. didactyla*, but seems to be most frequent in the montane belt, and differs in the richly branched rhizines and the C+ red medulla. – **Au**: T, O. **Sw**: GR, SZ, UR. **It**: VA.

***Peltigera horizontalis* (Huds.) Baumg.**

Syn.: *Antilyssa horizontalis* (Huds.) M. Choisy, *Lichen horizontalis* Huds., *Peltidea horizontalis* (Huds.) Ach., *Peltigera zopfii* Gyeln.

L – Subs.: bry, ter-cal, ter-sil, cor – Alt.: 1–4 – Note: on mosses (also epiphytic and epilithic) and humus-rich soil in the openings of humid forests, with a wide altitudinal range, but with optimum in the montane belt; widespread throughout the Alps. – **Au**: V, T, S, K, St, O, N, B. **Ge**: OB, Schw. **Sw**: AP, BE, FR, GL, GR, LU, SG, SZ, TI, UR, UW, VD, VS. **Fr**: AHP, HAl, AMa, Isè, Sav, HSav, Var, Vau. **It**: Frl, Ven, TAA, Lomb, Piem, VA, Lig. **Sl**: SlA, Tg.

***Peltigera hymenina* (Ach.) Delise**

Syn.: *Peltidea hymenina* Ach., *Peltigera lactucifolia auct. non* (With.) J.R. Laundon, Peltigera polydactylon (Neck.) Hoffm. var. crassoides Gyeln.

L – Subs.: bry, ter-sil, sil – Alt.: 2–4 – Note: on mineral soil in open, but never fully sun-exposed habitats, often associated with mosses, with optimum in the montane belt; widespread throughout the Alps. – **Au**: V, S, K, O. **Ge**: Ge. **Sw**: BE, GR, SZ, UR, VD, VS. **Fr**: AMa, Isè, Sav, HSav, Vau. **It**: Frl, TAA, Piem, Lig. **Sl**: SlA.

***Peltigera kristinssonii* Vitik.**

Syn.: *Peltigera occidentalis* *sensu* Kristinsson

L – Subs.: ter-sil, bry – Alt.: 3–5 – Note: a slightly calciphilous species; probably more widespread in the Alps, with optimum near or above treeline. – **Au**: T, S, K, St. **Ge**: OB. **Sw**: SZ, UR, VS. **Fr**: AHP, HAl, AMa, HSav. **It**: Lomb, Piem, VA.

***Peltigera lepidophora* (Vain.) Bitter**

Syn.: Peltigera canina (L.) Willd. var. lepidophora Vain.

L – Subs.: ter-cal, ter-sil – Alt.: 3–6 – Note: a mainly boreal-montane, circumpolar pioneer species of base-rich mineral soil, most frequent in upland areas, with optimum near treeline; widespread throughout the Alps, but only locally common. – **Au**: V, T, S, K, St, O, N. **Ge**: OB, Schw. **Sw**: BE, GR, SG, SZ, TI, VD, VS. **Fr**: AHP, HAl, Isè, HSav, Vau. **It**: Frl, Ven, TAA, Lomb, Piem, VA.

***Peltigera leucophlebia* (Nyl.) Gyeln.**

Syn.: Peltigera aphthosa (L.) Willd. var. leucophlebia Nyl., Peltigera aphthosa (L.) Willd. var. variolosa A. Massal., *Peltigera variolosa* (A. Massal.) Gyeln., *Peltigera vrangiana* Gyeln.

L – Subs.: ter-cal, bry, ter-sil – Alt.: 2–5 – Note: this is the vicariant of *P. aphthosa* on more or less calcareous substrata in upland areas, most common in the beech belt; widespread and rather common throughout the Alps. – **Au**: V, T, S, K, St, O, N, B. **Ge**: OB, Schw. **Sw**: BE, FR, GL, GR, LU, SG, SZ, TI, UR, UW, VD, VS. **Fr**: AHP, HAl, AMa, Drô, Isè, Sav, HSav, Var, Vau. **It**: Frl, Ven, TAA, Lomb, Piem, VA, Lig. **Sl**: SlA.

***Peltigera malacea* (Ach.) Funck**

Syn.: Peltidea canina (L.) Ach. var. malacea (Ach.) Wahlenb., *Peltidea malacea* Ach., Peltigera canina (L.) Willd. var. malacea (Ach.) Branth & Rostr.

L – Subs.: ter-sil, bry – Alt.: 3–5 – Note: a circumpolar, arctic-alpine lichen found in grasslands and shrublands near and above treeline, often amongst mosses, on siliceous substrata; widespread throughout the Alps. – **Au**: V, T, S, K, St, O. **Ge**: OB. **Sw**: BE, GR, TI, UR, VS. **Fr**: AHP, HAl, AMa, Isè, HSav, Var, Vau. **It**: Frl, Ven, TAA, Lomb, Piem, VA. **Sl**: SlA.

***Peltigera membranacea* (Ach.) Nyl.**

Syn.: Peltidea canina (L.) Ach. var. membranacea Ach., Peltigera canina (L.) Willd. var. membranacea (Ach.) Körb.

L – Subs.: bry, ter-cal, ter-sil – Alt.: 2–4 – Note: on mossy rocks and at the base of boles in old woodlands, usually on base-rich substrata, with optimum in the montane belt; widespread throughout the Alps. – **Au**: V, T, S, K, St, O, N. **Ge**: OB. **Sw**: BE, GR, LU, SG, SZ, TI, UR, UW, VD, VS. **Fr**: AHP, HAl, AMa, Drô, Isè, Sav, HSav, Var, Vau. **It**: Frl, Ven, TAA, Lomb, Piem. **Sl**: SlA.

***Peltigera monticola* Vitik.**

L – Subs.: ter-cal, bry, xyl – Alt.: 3–5 – Note: a recently-described and still rarely collected taxon related to *P. rufescens* and *P. ponojensis*, found on soil and amongst mosses over calcareous substrata, mostly in upland areas. – **Au**: T, S, K, St, O. **Sw**: BE, GR, LU, SZ, TI, UR, VD, VS. **Fr**: Sav. **It**: Frl, Ven. **Sl**: SlA.

***Peltigera neckeri* Hepp *ex* Müll. Arg.**

Syn.: *Peltigera polydactyloides auct*.

L – Subs.: ter-cal, ter-sil, bry, cor, xyl – Alt.: 1–5 – Note: on soil and on terricolous, epiphytic and silicicolous mosses, with a wide altitudinal range, but most frequent in humid-warm beech forests; widespread throughout the Alps. – **Au**: V, T, S, K, St, O, N. **Ge**: OB. **Sw**: BE, GL, GR, LU, SZ, TI, UR, VS. **Fr**: AHP, AMa, Isè, HSav, Var, Vau. **It**: Frl, Ven, TAA, Lomb, Piem, Lig. **Sl**: SlA.

***Peltigera neopolydactyla* (Gyeln.) Gyeln.**

Syn.: *Peltigera occidentalis* (E.Dahl) Kristinsson *non* *sensu* Kristinsson, Peltigera polydactylon (Neck.) Hoffm. var. neopolydactyla Gyeln.

L – Subs.: bry, cor, ter-sil – Alt.: 2–4 – Note: a forest floor species occurring amongst and over mosses, more rarely on rock or on bark, on basal parts of old trees, with optimum in the montane belt; widespread throughout the Alps, but not distinguished from similar species in the older literature. – **Au**: V, T, S, K, St, N. **Ge**: OB. **Sw**: BE, GR, SZ, VD, VS. **Fr**: Isè, HSav. **It**: Frl, Ven, TAA, Lomb, VA.

***Peltigera polydactylon* (Neck.) Hoffm.**

Syn.: Lichen caninus L. var. polydactylon (Neck.) Lightf., *Lichen polydactylus* Neck., *Peltidea polydactyla* (Neck.) Ach., Peltigera canina (L.) Willd. var. polydactyla (Neck.) Branth. & Rostr., Peltigera rufescens (Weiss) Humb. var. polydactyla (Neck.) Torss.

L – Subs.: bry, cor, ter-cal, ter-sil – Alt.: 2–5 – Note: an ecologically wide-ranging species of both mineral and organic, often base-rich soil, and on basal parts of mossy trunks and stumps in open forests, with optimum in the montane belt; widespread throughout the Alps. – **Au**: V, T, S, K, St, O, N. **Ge**: OB, Schw. **Sw**: BE, GL, GR, LU, SG, SZ, TI, UR, UW, VD, VS. **Fr**: AHP, HAl, AMa, Isè, Sav, HSav, Vau. **It**: Frl, Ven, TAA, Lomb, Piem, VA. **Sl**: SlA, Tg.

***Peltigera ponojensis* Gyeln.**

Syn.: *Peltigera plittii* Gyeln.

L – Subs.: bry, ter-cal, ter-sil – Alt.: 2–5 – Note: on subneutral to slightly basic soil in grasslands and heathlands, mostly in upland areas; often confused with *P. rufescens* in the past, and probably more widespread in the Alps. – **Au**: V, T, S, K, St, O. **Ge**: OB. **Sw**: GR, LU, SZ, VD, VS. **Fr**: AHP, HAl, AMa, HSav, Vau. **It**: Frl, Ven, TAA, Piem, VA.

***Peltigera praetextata* (Flörke *ex* Sommerf.) Zopf**

Syn.: Peltidea ulorrhiza Flörke var. praetextata Flörke *ex* Sommerf., Peltigera canina (L.) Willd. subsp. praetextata (Flörke *ex* Sommerf.) Ozenda & Clauzade, Peltigera canina (L.) Willd. var. tectorum Delise, Peltigera rufescens (Weiss) Humb. var. praetextata (Flörke *ex* Sommerf.) Th. Fr., *Peltigera subcanina* Gyeln.

L – Subs.: cor, xyl, bry, bry-cor, deb, ter-cal, ter-sil – Alt.: 1–5 – Note: a holarctic, ecologically wide-ranging species found both in open woodlands and in grasslands (but only in humid areas), on mosses, mineral or organic soil, lignum (on stumps) and bark (on basal parts of old trees); one of the most common species of the genus in the Alps, with a wide altitudinal range. – **Au**: V, T, S, K, St, O, N, B. **Ge**: OB, Schw. **Sw**: AP, BE, FR, GL, GR, LU, SG, SZ, TI, UR, UW, VD, VS. **Fr**: AHP, HAl, AMa, Isè, Sav, HSav, Var, Vau. **It**: Frl, Ven, TAA, Lomb, Piem, VA, Lig. **Sl**: SlA, Tg.

***Peltigera rufescen* s (Weiss) Humb.**

Syn.: Lichen caninus L. var. rufescens Weiss, *Lichen rufescens* (Weiss) Neck., *Peltidea ulorrhiza* Flörke, Peltigera canina (L.) Willd. var. crispa Kickx, Peltigera canina (L.) Willd. var. rufescens (Weiss) Mudd

L – Subs.: ter-cal, bry-cal, cal – Alt.: 1–6 – Note: a widespread holarctic lichen, most common in dry grasslands, especially in upland areas, but also in the Mediterranean belt, where it is generally rare due to intensive grazing and trampling; widespread and common throughout the Alps. – **Au**: V, T, S, K, St, O, N. **Ge**: OB, Schw. **Sw**: BE, GL, GR, LU, SZ, TI, UR, UW, VD, VS. **Fr**: AHP, HAl, AMa, Drô, Isè, Sav, HSav, Var, Vau. **It**: Frl, Ven, TAA, Lomb, Piem, VA, Lig. **Sl**: SlA. **Li**.

***Peltigera scabrosa* Th. Fr.**

Syn.: *Peltigera genuina* Gyeln., *Peltigera pulverulenta auct. non* (Taylor) Nyl., *Peltigera sancti-stephani* Gyeln.

L – Subs.: bry, ter-sil – Alt.: 3–5 – Note: a circumpolar, mainly arctic-alpine lichen found on mossy soil and rocks near and above treeline; quite rare in the Alps. – **Au**: T, S, K, St. **Sw**: ?TI. **Fr**: HSav. **It**: Lomb.

***Peltigera venosa* (L.) Hoffm.**

Syn.: *Lichen venosus* L., *Peltidea venosa* (L.) Ach.

L – Subs.: ter-sil, ter-cal – Alt.: 2–5 – Note: an arctic-alpine to boreal-montane, circumpolar lichen found on soil rich in humus in cold-humid sites near and above treeline; widespread throughout the siliceous Alps. – **Au**: V, T, S, K, St, O, N. **Ge**: OB, Schw. **Sw**: BE, GR, LU, SZ, TI, UR, VD, VS. **Fr**: AHP, HAl, AMa, Isè, Sav, HSav, Var. **It**: Frl, Ven, TAA, Lomb, Piem, VA, Lig. **Sl**: SlA.

***Peltula euploca* (Ach.) Poelt**

Syn.: *Endocarpon guepinii* Delise, *Endocarpon maravignae* Tornab., *Heppia euploca* (Ach.) Vain., *Heppia guepinii* (Delise) Nyl., *Heppia ruinicola* Nyl., *Heppia tenebrata* Nyl., *Lichen euplocus* Ach., ?*Omphalaria veronensis* A. Massal., *Peltula guepinii* (Delise) Gyeln., *Peltula laciniata* (Bagl. & Carestia) Poelt *comb. inval.*, *Peltula ruinicola* (Nyl.) Gyeln., ?*Thyrea veronensis* (A. Massal.) A. Massal.

L – Subs.: sil – Alt.: 1–3 – Note: a widespread species of warm-dry areas found on steeply inclined seepage tracks of basic siliceous rocks, with optimum below the montane belt; in the Alps most frequent in the inner xerothermic valleys. – **Au**: T, K. **Sw**: LU, TI, UW, VS. **Fr**: AMa, Var. **It**: Ven, TAA, Lomb, Piem, VA, Lig.

***Peltula obscurans* (Nyl.) Gyeln.**

Syn.: *Acarospora collemacea* Wedd., *Acarospora subglebosa* (Müll. Arg.) Hue, *Endocarpiscon obscurans* Nyl., *Heppia acarosporoides* Müll. Arg., *Heppia collemacea* (Wedd.) Boistel, *Heppia deserticola* Zahlbr., *Heppia obscurans* (Nyl.) Nyl., *Peltula subglebosa* (Müll. Arg.) Filson, *Solorinaria collemacea* (Wedd.) Gyeln.

L – Subs.: sil – Alt.: 1–2 – Note: on steeply inclined seepage tracks of basic siliceous rocks in lowland areas; a southern species, often found together with *P. euploca*, but much less frequent, with a few records from the Western Alps and the inner xerothermic valleys. – **Fr**: AMa, Var. **It**: TAA.

***Peltula patellata* (Bagl.) Swinscow & Krog**

Syn.: *Acarospora patellata* Bagl., *Heppia polyspora* Tuck., *Peltula polyspora* (Tuck.) Wetmore.

L – Subs.: ter-sil – Alt.: 1–2 – Note: on soil in dry grasslands over siliceous substrata; very rare in the dry valleys of the Alps. – **Sw**: VS. **It**: Lig.

***Peltula placodizans* (Zahlbr.) Wetmore**

Syn.: *Endocarpiscum placodizans* (Zahlbr.) Fink, *Heppia placodizans* Zahlbr.

L – Subs.: sil – Alt.: 1–2 – Note: on steeply inclined seepage tracks of basic siliceous rocks, both in the Mediterranean belt and in warm-dry alpine valleys of the Alps, where it is extremely rare. – **It**: TAA.

***Pertusaria alpina* Hepp *ex* Ahles**

Syn.: *Pertusaria laevigata* (Th. Fr.) Anzi *non* (Nyl.) Arnold, Pertusaria leioplaca DC. var. laevigata Th. Fr.

L – Subs.: cor – Alt.: 2–4 – Note: a mainly temperate species found on the smooth bark of deciduous trees, especially on twigs and branches. – **Au**: V, T, S, K, St, O, N. **Ge**: OB. **Sw**: BE, GL, GR, SZ, UR, UW, VS. **It**: Frl, TAA, Lomb. **Sl**: SlA.

***Pertusaria amarescens* Nyl.**

Syn.: *Pertusaria affinis* Erichsen, *Pertusaria coudercii* Harm., Pertusaria flavicans Lamy var. coudercii (Harm.) Erichsen

L # – Subs.: sil, cal – Alt.: 1–5 – Note: an often misunderstood species, or confused with *P. flavicans* or *Lepra aspergilla*. The species, however, seems to be quite common in the SW Alps (France). – **Au**: ?V. **Fr**: AHP, HAl, AMa, Sav, HSav. HAl, AMa. **It**: Ven, Lig.

***Pertusaria bryontha* (Ach.) Nyl.**

Syn.: Parmelia subfusca (L.) Ach. var. bryontha Ach., *Pertusaria inquinans* (Ach.) Th. Fr., *Pertusaria macrospora* Hepp

L – Subs.: deb, bry, ter-cal, ter-sil – Alt.: 4–6 – Note: an arctic-alpine, circumpolar lichen found on mosses and plant debris, mostly over calcareous substrata, with optimum near and above treeline, up to the nival belt. – **Au**: V, T, S, K, St, O. **Ge**: OB, Schw. **Sw**: BE, GR, SG, UR, UW, VS. **It**: Frl, TAA, Lomb.

***Pertusaria carneopallida* (Nyl.) Nyl**

Syn.: *Lecanora carneopallida* Nyl., *Pertusaria leptocarpa* Anzi

L – Subs.: cor – Alt.: 3–5 – Note: a cool-temperate to boreal-montane lichen found on smooth-barked hardwoods in upland areas; apparently not common in the Alps. – **Au**: T, K. **Ge**: OB, Schw. **It**: Frl, Ven, TAA, Lomb.

***Pertusaria chiodectonoides* Bagl.**

Syn.: *Pertusaria inquinata* (Ach.) Th. Fr., *Pertusaria nolens* Nyl.

L – Subs.: sil – Alt.: 1–3 – Note: a mild-temperate to Mediterranean species of basic siliceous rocks, whose total distribution is very poorly known. – **Au**: T, S, K, St, B. **Sw**: GR. **Fr**: AHP, AMa, Isè, HSav. **It**: TAA, VA, Lig. **Sl**: SlA.

***Pertusaria coccodes* (Ach.) Nyl.**

Syn.: *Lichen coccodes* Ach., *Pertusaria phymatodes* (Ach.) Erichsen

L – Subs.: cor, xyl, bry, sil – Alt.: 1–4 – Note: a mild-temperate lichen, mostly found on old oaks or beech trees, with optimum in open woodlands, much more rarely on siliceous rocks; widespread throughout the Alps, but only locally common. – **Au**: V, T, S, K, St, O, N, B. **Ge**: OB. **Sw**: BE, GL, GR, LU, SZ, TI, UR, UW, VD, VS. **Fr**: AHP, HAl, AMa, Drô, Isè, Sav, HSav, Var, Vau. **It**: Frl, Ven, Lomb. **Sl**: SlA, Tg.

***Pertusaria constricta* Erichsen**

L – Subs.: cor – Alt.: 2–3 – Note: on smooth bark, especially of *Quercus* and *Fagus*; probably more widespread in the Alps. – **Au**: V, T, S, K, St, O, N. **Ge**: OB, Schw. **Sw**: GL, SG, SZ, UR, UW, VS. **Fr**: Var. **It**: Lomb, Piem. **Sl**: SlA.

***Pertusaria coronata* (Ach.) Th. Fr.**

Syn.: Pertusaria coronata (Ach.) Th. Fr. var. isidiifera (Erichsen) Almb., Pertusaria coronata (Ach.) Th. Fr. var. soralifera Erichsen, *Pertusaria isidiifera* Erichsen, *Porina coronata* Ach.

L – Subs.: cor – Alt.: 1–4 – Note: a mild-temperate lichen found on bark of deciduous trees, mostly below the subalpine belt, easily mistaken for the chemically different *P. coccodes*; widespread throughout the Alps. – **Au**: V, T, S, K, O, N. **Ge**: OB. **Sw**: AP, BE, FR, GL, GR, LU, SG, SZ, TI, UR, UW, VD, VS. **Fr**: HAl, AMa, Vau. **It**: Frl, Ven, TAA, VA, Lig. **Sl**: SlA, Tg.

***Pertusaria cyparissi* Körb.**

L – Subs.: cor – Alt.: 2 – Note: a mainly Mediterranean epiphytic species related to *P. hymenea*, but with longer asci and larger ascospores, with a few records from the base of the Western Alps (France). – **Fr**: Var.

***Pertusaria flavicans* Lamy**

Syn.: Pertusaria flavicans Lamy var. schistosa Erichsen

L – Subs.: sil, cal, int – Alt.: 1–5 – Note: on lime-free but mineral-rich siliceous rocks, mostly on sheltered, steeply inclined surfaces; chemically variable and in need of further study; widespread throughout the Alps. – **Au**: V, T, S, K, St, B. **Ge**: Schw. **Sw**: BE, GR, LU, SZ, TI, VS. **Fr**: AHP, HAl, AMa, Isè, Sav, HSav, Vau. **It**: Frl, TAA, Lomb, Lig.

***Pertusaria flavida* (DC.) J.R. Laundon**

Syn.: *Pertusaria lutescens* (Hoffm.) Lamy *nom.illeg.*, *Pertusaria sorediana* Nyl., Pertusaria wulfenii DC. var. lutescens (Hoffm.) Th. Fr., Pertusaria wulfenii DC. var. variolosa Fr., *Variolaria flavida* DC.

L – Subs.: cor – Alt.: 1–3 – Note: a mild-temperate to Mediterranean species with optimum in open oak forests, mostly on old trees; widespread but only locally common in the Alps, below the subalpine belt. – **Au**: T, S, K, St. **Ge**: Ge. **Sw**: BE, GR, SG. **Fr**: AHP, AMa, HSav, Var, Vau. **It**: Frl, Ven, Lomb, Piem. **Sl**: SlA, Tg.

***Pertusaria geminipara* (Th. Fr.) C. Knight *ex* Brodo**

Syn.: *Lecanora geminipara* Th. Fr., *Ochrolechia geminipara* (Th. Fr.) Vain.

L – Subs.: deb, bry, ter-cal, ter-sil – Alt.: 3–6 – Note: an arctic-alpine, circumpolar lichen found on mosses, plant debris and soil over acid substrata near and above treeline. – **Au**: V, T, S, K, St, O, N. **Ge**: OB. **Sw**: GR, UR, VS. **It**: Frl, Ven, TAA, Lomb, Piem, VA.

***Pertusaria glomerata* (Ach.) Schaer.**

Syn.: *Porina glomerata* Ach.

L – Subs.: deb, bry, ter-cal, xyl – Alt.: 4–5 – Note: an arctic-alpine lichen found on more or less calciferous soil rich in humus and on plant debris in sites with a long snow cover; widespread throughout the Alps. – **Au**: V, T, S, K, St, O, N. **Ge**: OB, Schw. **Sw**: BE, GR, LU, SZ, TI, UW, VD, VS. **Fr**: HAl, Sav, HSav. **It**: Frl, Ven, TAA, Lomb, Piem. **Sl**: SlA.

***Pertusaria hymenea* (Ach.) Schaer.**

Syn.: *Lichen hymeneus* Ach., *Pertusaria lecanorodes* Erichsen, *Pertusaria sublecanorodes* Werner, *Pertusaria wulfenii* DC., *Porina rugosa* Ach.

L – Subs.: cor – Alt.: 1–3 – Note: a mainly mild-temperate lichen with optimum on old oaks in open stands; apparently more frequent in the Western and Southern Alps. – **Au**: T, K, O. **Sw**: BE, VD. **Fr**: AHP, HAl, AMa, Sav, Var. **It**: Ven, TAA, Lomb, Piem, Lig. **Sl**: SlA, Tg.

***Pertusaria leioplaca* DC.**

Syn.: *Pertusaria creatomma* (Norman) Zahlbr., *Pertusaria leucostoma auct. sensu* A. Massal., *Pertusaria massalongiana* Beltr., *Pertusaria plena* Anzi, *Porina leioplaca* Ach. *nom.illeg.*, *Porina leucostoma* (Bernh.) Ach., *Sphaeria leucostoma* Bernh.

L – Subs.: cor – Alt.: 1–4 – Note: a holarctic, mainly temperate early coloniser of smooth bark found on a wide variety of (mostly) broad-leaved trees; most common in the montane belt; widespread and common throughout the Alps. – **Au**: V, T, S, K, St, O, N, B. **Ge**: OB. **Sw**: BE, GL, GR, LU, SG, SZ, TI, UR, UW, VD, VS. **Fr**: AMa, Drô, Isè, Sav, HSav, Var. **It**: Frl, Ven, TAA, Lomb, Piem, Lig. **Sl**: SlA, Tg.

***Pertusaria octomela* (Norman) Erichsen**

Syn.: Pertusaria glomerata (Ach.) Schaer. var. octomela Norman

L – Subs.: cor – Alt.: 3 – Note: similar to *P. glomerata* and in the typical strain also with the same secondary chemistry (norstictic acid), but asci 8-spored and ascospores smaller; a second strain contains stictic acid and xanthones; on soil and plant debris, more common in the subarctic to boreal-subalpine zone, with a single record from the montane belt of the Eastern Alps (Austria). – **Au**: T.

***Pertusaria oculata* (Dicks.) Th. Fr.**

Syn.: *Lecanidium oculatum* (Dicks.) A. Massal., *Lecanora oculata* (Dicks.) Ach., *Lichen oculatus* Dicks.

L – Subs.: deb, bry, ter-cal, ter-sil – Alt.: 3–5 – Note: a circumpolar, arctic-alpine lichen found on soil and plant remains on siliceous substrata, mostly above treeline. – **Au**: V, T, S, K, St. **Sw**: TI, VS. **Fr**: HSav. **It**: Frl, TAA, Lomb, Piem, VA, Lig.

**Pertusaria pertusa (L.) Tuck. var. pertusa**

Syn.: *Lichen pertusus* L., *Pertusaria chioneoides* Erichsen, *Pertusaria colliculosa* Körb., *Pertusaria communis* DC., *Pertusaria leioterella* Erichsen, *Variolaria communis* (DC.) Ach.

L – Subs.: cor, sil – Alt.: 1–5 – Note: a mainly temperate lichen with optimum on smooth bark in the deciduous forest belts and in natural habitats, most abundant in the montane belt; widespread and common throughout the Alps. – **Au**: V, T, S, K, St, O, N, B. **Ge**: OB. **Sw**: BE, GL, GR, SG, SZ, TI, UR, UW, VD, VS. **Fr**: AMa, Isè, HSav, Var, Vau. **It**: Frl, Ven, TAA, Lomb, Piem, VA, Lig. **Sl**: SlA, Tg.

**Pertusaria pertusa (L.) Tuck. var. rupestris (DC.) Dalla Torre & Sarnth.**

Syn.: Pertusaria communis DC. var. rupestris DC., *Pertusaria rupestris* (DC.) Schaer.

L – Subs.: sil – Alt.: 1–5 – Note: a mainly temperate lichen found on base-rich siliceous rocks near the coast and in humid mountain areas. – **Au**: T, S, K, St, N, B. **Fr**: HAl, AMa, HSav, Vau. **It**: TAA, Lomb, Piem, VA.

***Pertusaria pseudocorallina* (Lilj.) Arnold**

Syn.: *Isidium microstictum* (Sm.) Turner & Borrer, *Lichen pseudocorallinus* Lilj., *Pertusaria ceuthocarpa* (Sm.) Turner & Borrer ex Fr., *Pertusaria ceuthocarpoides* Zahlbr., Pertusaria ceuthocarpoides Zahlbr. var. microstictica (Sm.) Zahlbr., *Pertusaria concreta* Nyl., *Pertusaria microstictica* (Sm.) Erichsen, *Pertusaria westringii* (Ach.) Hepp

L – Subs.: sil – Alt.: 1–6 – Note: a mild-temperate species found on steeply inclined surfaces of siliceous rocks wetted by rain. – **Au**: V, T, S, K, St. **Sw**: BE, GR, UR, VD, VS. **Fr**: AMa, Sav, Var, Vau. **It**: Ven, TAA, Lomb, Piem, VA, Lig.

***Pertusaria pulvereosulphurata* Harm.**

L # – Subs.: cor – Alt.: 2–3 – Note: the type material recalls a yellowish morph of *Loxospora elatina*, but the chemistry is different (atranorin and unknown substances); likely to belong to a genus other than *Pertusaria*; on bark of deciduous trees in Western Europe; most records from the Alps are in need of re-investigation. – **Sw**: ?BE, ?GL, ?GR, ?SZ, ?VD. **Fr**: Vau.

***Pertusaria pupillaris* (Nyl.) Th. Fr.**

Syn.: *Lecanora pupillaris* Nyl.

L – Subs.: xyl, cor – Alt.: 2–4 – Note: a temperate to southern boreal-montane, perhaps holarctic lichen found on hard lignum and smooth bark; overlooked, being almost always sterile, and perhaps more widespread, albeit never common, in upland areas. – **Au**: T, S, K, St, O, N. **Ge**: OB. **Sw**: GL, GR, SZ, TI, UR, VS. **Fr**: Isè. **It**: TAA. **Sl**: SlA.

***Pertusaria pustulata* (Ach.) Duby**

Syn.: *Lichen melaleucus* Turner & Borrer, *Pertusaria melaleuca* (Turner & Borrer) Duby, Pertusaria wulfenii DC. var. glabrata Anzi, *Porina pustulata* Ach.

L – Subs.: cor – Alt.: 1–3 – Note: a mainly temperate species found on deciduous trees with smooth bark, especially *Carpinus* and *Fagus*, more rarely on deciduous oaks in moist woodlands. – **Au**: V, St. **Sw**: BE. **Fr**: AMa, Isè, Sav, Var. **It**: Frl, Ven, TAA, Lomb, Piem. **Sl**: SlA.

***Pertusaria rupicola* (Fr.) Harm.**

Syn.: Pertusaria rupicola (Fr.) Harm. var. coralloidea (Anzi) Croz., *Pertusaria sulphurea* A. Massal. *non* Schaer., Pertusaria wulfenii DC. var. rupicola Fr.

L – Subs.: sil – Alt.: 1–3 – Note: a mild-temperate lichen of siliceous rocks, most frequent near the coast, but also occurring in humid mountain areas. Isidiate and fruiting specimens are often found together, and intermediate specimens are frequent, the isidiate morphs (those with isidioid papillae bearing pycnidia) are better treated at the rank of *forma.* – **Sw**: GR. **Fr**: AMa, Var. **It**: TAA, Piem, Lig

***Pertusaria saximontana* Wetmore**

Syn.: *Pertusaria christae* Dibben & Poelt

L – Subs.: ter – Alt.: 5 – Note: a species with a grey thallus containing 2’-O-methylperlatolic acid, lecanorate fruiting bodies with a blackish epihymenium reacting K+ violet, and 2-spored asci, based on a type from Wyoming; typically on wood and on bark of conifers in Western North America; the only record from the Alps was terricolous, and based on the recently established synonym *P. christae*. – **Au**: S.

***Pertusaria sommerfeltii* (Sommerf.) Fr.**

Syn.: *Endocarpon sommerfeltii* Sommerf., *Pertusaria angusticollis* Anzi, *Pertusaria melastoma* Nyl.

L – Subs.: cor – Alt.: 3–5 – Note: a circumpolar, subarctic-subalpine to boreal-montane lichen found on smooth bark of subalpine shrubs. – **Au**: V, T, S, St, O. **Ge**: OB, Schw. **Sw**: GR, LU, SG, UR, UW. **Fr**: HAl, HSav. **It**: Frl, Ven, TAA, Lomb, VA.

***Pertusaria stenhammari* Hellb.**

L – Subs.: cor – Alt.: 3–4 – Note: a very rarely collected, apparently panboreal-montane species found on the bark of conifers, with optimum in the upper montane and subalpine belts. Normally fertile, but the var. *elatina* Erichsen, described from the Alps, is sorediate. – **Au**: T. **It**: TAA. **Sl**: SlA.

***Pertusaria trochiscea* Norman**

L – Subs.: bry, deb – Alt.: 4 – Note: a species resembling *P. glomerata*, but with a different secondary chemistry (coronaton), thallus reacting K-, and 4-spored asci; on bryophytes and plant debris, with scattered records in Northern Europe from the subarctic to the boreal-alpine zone, rarely recorded elsewhere, with a few records from the Eastern Alps (Austria). – **Au**: K, St.

***Petractis clausa* (Hoffm.) Kremp.**

Syn.: *Gyalecta clausa* (Hoffm.) A. Massal., *Gyalecta exanthematica* (Sm.) Fr., *Lecidea exanthematica* (Sm.) Nyl., *Lichen clausus* Hoffm., *Petractis exanthematica* (Sm.) Fr., *Thelotrema clausum* (Hoffm.) Schaer., *Thelotrema exanthematicum* (Sm.) Ach., *Urceolaria exanthematica* (Sm.) Ach.

L – Subs.: cal – Alt.: 1–5 – Note: a temperate species found on compact calcareous rocks in humid-shaded situations, such as in gorges and woodlands, with optimum in the submediterranean belt; widespread throughout the Alps. – **Au**: V, T, S, K, St, O, N. **Ge**: OB. **Sw**: BE, GR, LU, SZ, UR, UW, VD, VS. **Fr**: AHP, HAl, AMa, Drô, Isè, Sav, HSav, Var, Vau. **It**: Frl, Ven, TAA, Lomb, Piem, Lig. **Sl**: SlA, Tg.

***Phaeographis dendritica* (Ach.) Müll. Arg.**

Syn.: *Graphis dendritica* (Ach.) Ach., *Opegrapha dendritica* Ach.

L – Subs.: cor – Alt.: 1–3 – Note: a mild-temperate to humid subtropical species found on smooth bark of deciduous and evergreen trees in very humid, open woodlands; strongly declining and presently extinct in several regions (*e.g.* most records from Austria and Italy are historical). – **Au**: S. **Fr**: Isè, Sav. **It**: Ven, Lomb, Lig. **Sl**: SlA.

***Phaeophyscia cernohorskyi* (Nádv.) Essl.**

Syn.: *Phaeophyscia strigosa* (Poelt & Buschardt) N.S. Golubk., *Physcia cernohorskyi* Nádv., Physcia hirsuta Mereschk. var. echinella Poelt, *Physcia strigosa* Poelt & Buschardt

L – Subs.: cal, sil, bry – Alt.: 1–3 – Note: a widespread, often misunderstood species, chiefly epilithic in the northern part of its range, but found on a wide variety of substrata in the southern part, with optimum in dry-warm areas. The relationships with *Ph. hirsuta* await further study: the latter species is rather frequent in the submediterranean belt, whereas *Ph. cernohorskyi* is common and abundant only in dry-warm areas, such as in the dry valleys of the Alps. – **Au**: T, K, St, N. **Sw**: VS. **Fr**: AMa. **It**: TAA, Lomb, Piem, VA, Lig.

***Phaeophyscia ciliata* (Hoffm.) Moberg**

Syn.: *Lichen ciliatus* Hoffm., *Lichen ulothrix* Ach, *Physcia ciliata* (Hoffm.) Du Rietz, *Physcia concrustans* Nyl., *Physcia norrlinii* Vain., *Physcia obscura auct. non* (Ehrh. *ex* Humb.) Fürnr., *Physcia ulothrix* (Ach.) Nyl.

L – Subs.: cor, xyl – Alt.: 2–4 – Note: a temperate to southern boreal lichen, most frequent on *Fraxinus* and *Juglans* in montane valleys, much less common than the closely related *Ph. orbicularis*, being absent from heavily disturbed areas and from eu-Mediterranean vegetation, and with narrower ecological requirements. – **Au**: V, T, S, K, St, O, N. **Ge**: OB. **Sw**: BE, GL, GR, LU, SZ, TI, UR, VS. **Fr**: AHP, AMa, Drô, Sav, HSav, Var, Vau. **It**: Frl, Ven, TAA, Lomb, Piem, VA. **Sl**: SlA, Tg. **Li**.

***Phaeophyscia constipata* (Norrl. & Nyl.) Moberg**

Syn.: *Physcia constipata* Norrl. & Nyl., Physcia pulverulenta (Wahlenb.) Fürnrh. var. tenuis (Körb.) Th. Fr.

L – Subs.: ter-cal, bry-cal – Alt.: 2–5 – Note: a mainly circumboreal-montane species found on mosses and plant debris on basic siliceous substrata, sometimes on soil, in upland areas, with optimum in dry-warm situations; widespread but not common in the Alps. – **Au**: T, S, K. **Sw**: BE, GR, UR, VS. **It**: TAA, Lomb, Piem, VA.

***Phaeophyscia endococcina* (Körb.) Moberg**

Syn.: *Parmelia endococcina* Körb., Parmelia obscura (Ehrh.) Fr. var. endococcina (Körb.) Anzi, *Physcia endochroidea* Nyl., *Physcia endococcina* (Körb.) Th. Fr., *Physcia lithotodes* Nyl.

L – Subs.: sil, xyl – Alt.: 3–5 – Note: a cool-temperate to circumboreal-montane lichen described from Italy, found near creeks and brooks, but also along seepage tracks in warm-dry valleys of the Alps; specimens without the red pigment in the medulla are relatively frequent in the Alps, mostly at low elevations. – **Au**: V, T, S, K, St. **Ge**: Ge. **Sw**: BE, GL, GR, SZ, TI, UR, VD, VS. **Fr**: AHP, HAl, AMa, Sav, HSav. **It**: Frl, Ven, TAA, Lomb, Piem, VA.

***Phaeophyscia endophoenicea* (Harm.) Moberg**

Syn.: *Physcia endophoenicea* (Harm.) Sántha, *Physcia labrata* *sensu* Frey, *Physcia obscura* (Ehrh.) Hampe *ex* Fürnr. var. endophoenicea Harm., *Physcia ocellata* Erichsen

L – Subs.: cor – Alt.: 1–4 – Note: a mild-temperate lichen found on epiphytic bryophytes and bark in open, humid woodlands; specimens without the red pigment in the medulla, which are not rare, can be easily confused with other species. – **Au**: V, T, S, K, St, O, N. **Ge**: OB, Schw. **Sw**: BE, GL, GR, LU, SZ, TI, UR, VD, VS. **Fr**: AMa, Isè, HSav, Var, Vau. **It**: Frl, Ven, TAA, Lomb, Piem, Lig. **Sl**: SlA, Tg.

***Phaeophyscia hirsuta* (Mereschk.) Essl.**

Syn.: *Physcia hirsuta* Mereschk., *Physcia labrata* Mereschk., Physcia labrata Mereschk. var. olivacea Mereschk.

L – Subs.: cor, sil – Alt.: 1–3 – Note: a mainly temperate lichen found on isolated trees, more rarely on rock; widespread throughout the Alps, but only locally common. – **Au**: V, T, S, K, St, O, N. **Ge**: Schw. **Sw**: BE, GL, GR, TI, VS. **Fr**: AHP, HAl, AMa, Drô, Var, Vau. **It**: Frl, Ven, TAA, Lomb, Piem. **Sl**: SlA, Tg. **Li**.

***Phaeophyscia hispidula* (Ach.) Essl.**

Syn.: *Parmelia hispidula* Ach., *Physcia hispidula* (Ach.) Frey, *Physcia setosa* (Ach.) Nyl.

L – Subs.: cal, sil, cor, ter – Alt.: 2–4 – Note: a rare, mainly circumboreal-montane species, mostly found on terricolous or saxicolous bryophytes in upland areas. – **Au**: T, St. **Sw**: GL, GR. **Fr**: Var. **It**: TAA, Lomb, Piem.

***Phaeophyscia insignis* (Mereschk.) Moberg**

Syn.: *Physcia insignis* Mereschk., *Physcia ticinensis* (Mereschk.) Frey, Physcia virella (Ach.) Flagey var. gracilis Mereschk.

L – Subs.: bry, cal, cor, bry-cor – Alt.: 2–3 – Note: a mild-temperate species found on isolated trees with base-rich, soft bark; certainly more widespread albeit never common in the Alps, perhaps often confused with stout specimens of *Hyperphyscia adglutinata*. – **Au**: T, K, St. **Sw**: GR, TI. **It**: Frl, Lomb, Piem.

***Phaeophyscia kairamoi* (Vain.) Moberg**

Syn.: *Phaeophyscia nadvornikii* (Frey & Poelt) N.S. Golubk., Physcia cernohorskyi Nádv. var. erosa Nádv., *Physcia kairamoi* Vain., *Physcia karakorina* Poelt, *Physcia nadvornikii* Frey & Poelt

L – Subs.: cor, bry, cal, sil – Alt.: 2–5 – Note: on base-rich bark, more rarely on calciferous schistose rocks. – **Au**: V, T, S, K, St, O. **Sw**: GR, SG. **It**: Frl, TAA.

***Phaeophyscia nigricans* (Flörke) Moberg**

Syn.: *Lecanora nigricans* Flörke, *Physcia leptothallina* (Vain.) Zahlbr., *Physcia nigricans* (Flörke) Stizenb., Physcia nigricans (Flörke) Stizenb. var. sciastrella (Nyl.) Lynge, Physcia nigricans (Flörke) Stizenb. var. tremulicola (Nyl.) Lynge, *Physcia sciastrella* (Nyl.) Harm., *Physcia tremulicola* Nyl., *Physcia tribacella* Nyl.

L – Subs.: cal, cor, xyl, sil – Alt.: 2–4 – Note: a mainly temperate, perhaps holarctic lichen found on a wide variety of substrata, not infrequent, but often overlooked, on isolated trees in lowland areas, reaching however the subalpine belt; the species has been often misunderstood and confused with dark-coloured specimens of *Ph. orbicularis*. – **Au**: V, T, S, K, St, O, N, B. **Ge**: OB, Schw. **Sw**: GR, LU, SZ, TI, VS. **Fr**: AHP, AMa. **It**: Frl, Ven, TAA, Lomb, Piem, VA, Lig. **Sl**: SlA. **Li**.

***Phaeophyscia orbicularis* (Neck.) Moberg**

Syn.: *Lichen orbicularis* Neck., *Parmelia cycloselis* Ach. *nom.illeg.*, Parmelia obscura (Ehrh.) Fr. var. cycloselis Schaer. *nom.illeg.*, Parmelia obscura (Ehrh.) Fr. var. orbicularis (Neck.) Eschw., *Parmelia pulverulenta* Ach. *nonauct.*, *Physcia cycloselis* (Durieu & Mont.) Vain. *ex* Räsänen, *Physcia hueiana* (Harm.) Räsänen, *Physcia obscura* (Ehrh. *ex* Humb.) Fürnr. *nonauct.*, *Physcia obscura* (Ehrh. *ex* Humb.) Fürnr. var. hueiana (Harm.) H. Olivier, *Physcia obscura* (Ehrh. *ex* Humb.) Fürnr. var. virella (Ach.) Nyl., *Physcia orbicularis* (Neck.) Poetsch, Physcia orbicularis (Neck.) Poetsch var. virella (Ach.) A.L. Sm., *Physcia pulverulenta* (Wahlenb.) Fürnrh. *nonauct.*, *Physcia virella* (Ach.) Flagey, Physcia virella (Ach.) Flagey var. hueiana (Harm.) Sántha

L – Subs.: cor, xyl, cal, sil – Alt.: 1–5 – Note: a holarctic, very polymorphic, ecologically wide-ranging and common species also occurring within settlements on a wide variety of substrata; widespread and common throughout the Alps. – **Au**: V, T, S, K, St, O, N, B. **Ge**: OB, Schw. **Sw**: AP, BE, FR, GL, GR, LU, SG, SZ, TI, UR, UW, VD, VS. **Fr**: AHP, HAl, AMa, Drô, Isè, Sav, HSav, Var, Vau. **It**: Frl, Ven, TAA, Lomb, Piem, VA, Lig. **Sl**: SlA, Tg. **Li**.

***Phaeophyscia poeltii* (Frey) Nimis**

Syn.: *Physcia poeltii* Frey

L – Subs.: cor – Alt.: 1–3 – Note: a temperate species found on isolated deciduous trees with nutrient-rich bark, especially in montane valleys. – **Sw**: BE, GR, SG, TI, VS. **Fr**: AHP, HAl, AMa, Var. **It**: Frl, Ven, TAA, Lomb, Piem, VA.

***Phaeophyscia pusilloides* (Zahlbr.) Essl.**

Syn.: *Physcia pusilla* Mereschk., *Physcia pusilloides* Zahlbr., *Physcia suzai* Nádv.

L – Subs.: cor – Alt.: 2–3 – Note: a temperate species found on isolated deciduous trees with nutrient-rich bark, especially *Juglans* and *Fraxinus* in montane valleys, absent from urban areas, somehow less frequent in semi-natural stands; often confused, in the past, with other species. – **Au**: V, T, S, K, St, O, N. **Ge**: OB, Schw. **Sw**: BE, GR, LU, TI, VD. **Fr**: Var. **It**: Frl, Ven, Lomb, Piem, Lig. **Sl**: SlA.

***Phaeophyscia rubropulchra* (Degel.) Moberg**

Syn.: Physcia orbicularis (Neck.) Poetsch f. rubropulchra Degel.

L – Subs.: cor – Alt.: 2–3 – Note: a mainly mild-temperate epiphytic species, known from Eastern North America and West Asia, with a few relict stations in Europe. – **It**: Frl.

***Phaeophyscia sciastra* (Ach.) Moberg**

Syn.: Hagenia obscura (Ehrh.) De Not. var. sciastra (Ach.) Bagl. & Carestia, *Parmelia sciastra* Ach., *Physcia lithoteaauct.*, *Physcia sciastra* (Ach.) Du Rietz

L – Subs.: cal, sil, bry, xyl, cor – Alt.: 1–5 – Note: a holarctic lichen with a wide altitudinal and longitudinal range, found on the top of exposed calciferous boulders, sometimes on siliceous rocks or even on eutrophicated lignum, epilithic mosses, etc., with a wide altitudinal range; widespread throughout the Alps. – **Au**: V, T, S, K, St, O, N. **Ge**: Ge. **Sw**: BE, GR, LU, SZ, TI, UR, VD, VS. **Fr**: AHP, HAl, AMa, Drô, Sav, HSav. **It**: Frl, Ven, TAA, Lomb, Piem, VA, Lig. **Sl**: SlA.

***Phaeophyscia stiriaca* (Poelt) Clauzade & Cl. Roux *ex* Hafellner & Türk**

Syn.: *Physcia stiriaca* Poelt

L – Subs.: cor – Alt.: 2–3 – Note: a rare species resembling *Ph. endophoenicea* in the presence of red crystals in the lower medulla, but with a pale to tan lower surface and labriform soralia on ascending lobes, developing coarse soredia; on bark of deciduous trees in lowland forests, with a few scattered records throughout Central Europe. – **Au**: V, St. **Ge**: OB.

***Phaeorrhiza nimbosa* (Fr.) H. Mayrhofer & Poelt**

Syn.: *Parmelia nimbosa* Fr., *Rinodina nimbosa* (Fr.) Tr.Fr.

L – Subs.: ter-cal, ter-sil, deb, bry – Alt.: 4–6 – Note: a circumpolar, arctic-alpine species found on naked earth, dead mosses and plant debris on more or less calciferous ground, often in wind-exposed situations, with optimum above treeline; common in the Alps, where it reaches the nival belt. – **Au**: V, T, S, K, St, O, N. **Ge**: OB, Schw. **Sw**:  BE, GR, SZ, TI, UW, VD, VS. **Fr**: AHP, HAl, AMa, Isè, Sav, HSav. **It**: Frl, Ven, TAA, Lomb, Piem, VA, Lig. **Sl**: SlA. **Li**.

**Phaeorrhiza sareptana (Tomin) H. Mayrhofer & Poelt var. sphaerocarpa (Th. Fr.) H. Mayrhofer & Poelt**

Syn.: *Buellia dovrensis* H. Magn., *Buellia hypoleuca* H. Magn., Rinodina nimbosa (Fr.) Th. Fr. var. sphaerocarpa Th. Fr.

L – Subs.: ter-sil, deb – Alt.: 4–6 – Note: a rather rare species found on naked earth, dead mosses and plant debris in dry grasslands near and above treeline. – **Au**: T. **Fr**: AHP, HAl. **It**: Ven, TAA.

***Phlyctis agelaea* (Ach.) Flot.**

Syn.: *Lichen agelaeus* Ach., *Thelotrema variolariodes auct.* var. agelaeum (Ach.) Ach.

L – Subs.: cor – Alt.: 1–3 – Note: a mild-temperate to Mediterranean lichen found on acid-barked trees (especially *Quercus ilex*) in slightly sheltered but not very shaded situations. – **Au**: T, K, St, O, N, B. **Ge**: Ge. **Sw**: BE, GL, TI, UW, VD, VS. **Fr**: AMa, Isè, HSav, Var, Vau. **It**: Frl, Ven, Lomb, Piem, Lig. **Sl**: SlA, Tg.

***Phlyctis argena* (Spreng.) Flot.**

Syn.: *Parmelia argena* Spreng., *Pertusaria reducta* Stirt., *Phlyctis erythrosora* Erichsen, *Urceolaria variolarioides* Pers.

L – Subs.: cor, sil – Alt.: 1–3 – Note: a subtropical to southern boreal-montane, holartic lichen, an aggressive coloniser of smooth bark (*e.g.* of *Carpinus*) in sheltered situations (*e.g.* in forests), with optimum in the deciduous forest belts; widespread and common throughout the Alps. – **Au**: V, T, S, K, St, O, N, B. **Ge**: OB, Schw. **Sw**: AP, BE, FR, GL, GR, LU, SG, SZ, TI, UR, UW, VD, VS. **Fr**: AHP, AMa, Drô, Isè, HSav, Var, Vau. **It**: Frl, Ven, TAA, Lomb, Piem. **Sl**: SlA, Tg. **Li**.

***Phylliscum demangeonii* (Moug. & Mont.) Nyl.**

Syn.: *Collema demangeonii* Moug. & Mont., *Phylliscum endocarpoides* Nyl., *Phylliscum silesiacum* Stein

L – Subs.: sil – Alt.: 3–4 – Note: a cool-temperate to boreal-montane, probably circumpolar lichen found on steeply inclined seepage tracks of siliceous rocks, mostly in upland areas; probably more widespread in the Alps. – **Au**: S. **It**: TAA, Piem.

***Physcia adscendens* H. Olivier**

Syn.: Parmelia stellaris (L.) Ach. var. adscendens Fr., *Physcia ascendens* Bitter

L – Subs.: cor, xyl, cal, sil – Alt.: 1–5 – Note: a widespread holarctic lichen, one of the most common species of the genus throughout the Alps, mostly on isolated trees, but also on walls and eutrophicated calciferous rocks. – **Au**: V, T, S, K, St, O, N, B. **Ge**: OB, Schw. **Sw**: AP, BE, FR, GL, GR, LU, SG, SZ, TI, UR, UW, VD, VS. **Fr**: AHP, HAl, AMa, Drô, Isè, Sav, HSav, Var, Vau. **It**: Frl, Ven, TAA, Lomb, Piem, VA, Lig. **Sl**: SlA, Tg. **Li**.

***Physcia aipolia* (Ehrh. *ex* Humb.) Fürnr.**

Syn.: *Lichen aipolius* Ehrh. *ex* Humb., *Parmelia aipolia* (Ehrh. *ex* Humb.) Ach., Parmelia stellaris (L.) Ach. var. aipolia (Humb.) Hazsl., *Physcia aipolia* (Ehrh. *ex* Humb.) Fürnr. var. acrita (Ach.) Hue, *Physcia aipolia* (Ehrh. *ex* Humb.) Fürnr. var. anthelina (Ach.) Vain., *Physcia aipolia* (Ehrh. *ex* Humb.) Fürnr. var. cercidia (Ach.) Nyl., Physcia stellaris (L.) Nyl. var. aipolia (Ehrh. *ex* Humb.) Th. Fr., Physcia stellaris (L.) Nyl. var. angustata Nyl.

L – Subs.: cor, xyl – Alt.: 1–4 – Note: a mainly temperate species, altitudinally intermediate between *Ph. biziana* and *Ph. stellaris*, most frequent at low elevations only in humid areas. Molecular data suggest that the *Physcia aipolia*-*Ph. caesia*-complex includes several entities which, differing also in morphology and/or chemistry, can be treated as distinct species. – **Au**: V, T, S, K, St, O, N, B. **Ge**: OB, Schw. **Sw**: AP, BE, FR, GL, GR, LU, SG, SZ, TI, UR, UW, VD, VS. **Fr**: AHP, HAl, AMa, Drô, Isè, Sav, HSav, Var, Vau. **It**: Frl, Ven, TAA, Lomb, Piem, VA, Lig. **Sl**: SlA, Tg. **Li**.

***Physcia aipolioides* (Nádv.) Breuss & Türk**

Syn.: Physcia biziana (A. Massal.) Zahlbr. var. aipolioides Nádv.

L – Subs.: cor – Alt.: 2 – Note: a species resembling *Ph. biziana*, but thalli larger, with thicker, slightly pruinose lobes, lacking lobules in the centre, and underside with yellowish areas; widespread and fairly common on bark of roadside trees in the eastern part of Central Europe and in inland localities of the Balkan Peninsula, with a few records from the eastern foothills of the Eastern Alps. – **Au**: N, B.

***Physcia albinea* (Ach.) Nyl.**

Syn.: *Lichen alboniger* Schleich., *Parmelia albinea* Ach., *Physcia albonigra* (Schleich.) Dalla Torre & Sarnth., Physcia stellaris (L.) Nyl. subsp. albinea (Ach.) Clauzade & Cl. Roux

L # – Subs.: sil – Alt.: 3–5 – Note: on basic siliceous rocks, certainly rare and doubtfully distinct from *Ph. stellaris*. – **Au**: T, S, St. **Sw**: GR, TI, UR, VS. **Fr**: AHP, HAl, AMa, HSav, Var. **It**: TAA, Lomb, Piem, VA, Lig.

**Physcia biziana (A. Massal.) Zahlbr. var. biziana**

Syn.: *Physcia ragusana* Zahlbr., *Squamaria biziana* A. Massal.

L – Subs.: cor – Alt.: 1–2 – Note: a Mediterranean to mild-temperate species found on isolated trees at low altitudes; most common at low elevations in the Western and Southern Alps. – **Au**: S, K, St, N. **Fr**: AHP, HAl, AMa, Drô, Isè, Var, Vau. **It**: Frl, Ven, Lomb, Piem, VA, Lig.

**Physcia biziana (A. Massal.) Zahlbr. var. leptophylla Vězda**

Syn.: *Physcia rondoniana* Clauzade & Vězda

L – Subs.: cor, sil – Alt.: 1–2 – Note: an interesting taxon well worth of further study; in the study area so far known only from the base of the Western Alps. – **Fr**: Var. **It**: Lig.

**Physcia caesia (Hoffm.) Fürnr. var. caesia**

Syn.: *Hagenia caesia* (Hoffm.) Bagl. & Carestia, *Lichen caesius* Hoffm., *Parmelia caesia* (Hoffm.) Ach., Physcia caesia (Hoffm.) Fürnr. var. ventosa (Lynge) Frey, *Physcia ventosa* (Lynge) Sántha

L – Subs.: cal, int, sil, xyl, cor – Alt.: 2–6 – Note: a cool-temperate to arctic-alpine, circumpolar species, common only in upland areas, mostly in natural habitats (*e.g.* on the top of calcareous boulders); it exceptionally grows also on bark and lignum impregnated with calcareous dust; widespread and common throughout the Alps. – **Au**: V, T, S, K, St, O, N, B. **Ge**: OB, Schw. **Sw**: AP, BE, GR, LU, SZ, TI, UR, UW, VD, VS. **Fr**: AHP, HAl, AMa, Drô, Isè, Sav, HSav, Var. **It**: Frl, Ven, TAA, Lomb, Piem, VA, Lig. **Sl**: SlA, Tg. **Li**.

**Physcia caesia (Hoffm.) Fürnr. var. caesiella (B. de Lesd.) Clauzade & Cl. Roux**

Syn.: *Physcia caesiella* (B. de Lesd.) Suza, *Physcia subalbinea* Nyl., Physcia tribacoides Nyl. var. caesiella B. de Lesd., *Physcia wainioi* Räsänen

L – Subs.: sil, cal, bry, deb – Alt.: 2–5 – Note: ecologically similar to the typical variety, but most common in dry-warm valleys of the Alps. – **Au**: V, T, S, K, St, B. **Ge**: Schw. **Fr**: AHP, HAl, AMa. **It**: TAA, Lomb, Piem, VA, Lig.

**Physcia caesia (Hoffm.) Fürnr. var. rhaetica Frey**

L – Subs.: int, sil – Alt.: 4–5 – Note: a morph with an orange medulla, most frequent in continental Alpine areas, which is worth of further study. – **Au**: V, T, K. **It**: Piem.

***Physcia clementei* (Turner) Lynge**

Syn.: *Lichen clementei* Turner, *Physcia astroidea auct*.

L – Subs.: cor – Alt.: 1–3 – Note: a Mediterranean to mild-temperate, mainly western species growing on more or less isolated trees; rare throughout the Alps, most frequent in areas with a humid-rainy climate. – **Sw**: TI, UW. **Fr**: AHP, AMa, Var, Vau. **It**: Frl, Ven, Lomb, Piem.

**Physcia dimidiata (Arnold) Nyl. var. dimidiata**

Syn.: Parmelia albinea Ach. var. dimidiata (Arnold) Jatta, Parmelia pulverulenta Ach. var. dimidiata Arnold

L – Subs.: sil, cal, cor – Alt.: 1–4 – Note: a Mediterranean to mild-temperate, probably holarctic lichen found on steeply inclined surfaces of basic siliceous rocks and calciferous sandstone, on old walls, more rarely on the basal parts of old trees, mostly below the subalpine belt. – **Au**: T, S, K, St, N, B. **Sw**: BE, GR, LU, TI, VD, VS. **Fr**: AHP. **It**: TAA, Lomb, Piem, VA.

**Physcia dimidiata (Arnold) Nyl. var. ornata (Nádv.) Moberg**

Syn.: Physcia dimidiata (Arnold) Nyl. f. ornata Nádv.

L – Subs.: cal – Alt.: 2–4 – Note: a variety with narrow lobes and crenate tips; usually on rocks with high content of calcium, often under overhangs; apparently this is the most common variety in Eastern and Central Europe, but it was not generally distinguished, so that the distribution in the Alps is insufficiently known. – **Au**: T, N.

***Physcia dubia* (Hoffm.) Lettau**

Syn.: *Lobaria dubia* Hoffm., Physcia caesia (Hoffm.) Fürnr. var. dubia (Hoffm.) Th. Fr., Physcia dubia (Hoffm.) Lettau var. teretiuscula (Ach.) Clauzade & Cl. Roux, *Physcia intermedia* Vain., *Physcia lyngei* Nádv., *Physcia teretiuscula* (Ach.) Lynge, *Physcia wahlenbergii* Lynge

L – Subs.: cal, sil, int, cor, xyl – Alt.: 1–6 – Note: a widespread holarctic species with a broad latitudinal and altitudinal range, found on base-rich substrata, both in natural situations and on walls in villages, with a wide altitudinal range; widespread throughout the Alps. – **Au**: V, T, S, K, St, O, N, B. **Ge**: OB. **Sw**: BE, GL, GR, LU, SG, SZ, TI, UR, UW, VS. **Fr**: AHP, HAl, AMa, Drô, Isè, Sav, HSav, Var, Vau. **It**: Frl, Ven, TAA, Lomb, Piem, VA, Lig. **Sl**: SlA, Tg. **Li**.

***Physcia leptalea* (Ach.) DC.**

Syn.: *Lichen leptaleus* Ach., *Lichen semipinnatus* J.F. Gmel., *Physcia semipinnata* (J.F. Gmel.) Moberg, *Physcia subteres* (Harm.) Lettau

L – Subs.: cor – Alt.: 1–4 – Note: a Mediterranean to mild-temperate lichen, most common on twigs of shrubs below the montane belt. – **Au**: V, S, K. **Sw**: GR, SZ, TI, VD, VS. **Fr**: AHP, HAl, AMa, Drô, Isè, Sav, HSav, Var, Vau. **It**: Frl, Ven, TAA, Lomb, Piem, Lig. **Sl**: SlA.

***Physcia magnussonii* Frey**

Syn.: *Physcia aipolia* (Ehrh. *ex* Humb.) Fürnr. var. subincisa (Th. Fr.) Lynge, Physcia caesia (Hoffm.) Fürnr. var. albinea Anzi, Physcia stellaris (L.) Nyl. var. subincisa Th. Fr.

L – Subs.: sil – Alt.: 2–4 – Note: on steeply inclined surfaces of base-rich siliceous rocks, often starting its development in fissures of the rock. – **Au**: T, S, K. **Sw**: GR, VS. **Fr**: HAl, AMa, Isè, Sav. **It**: TAA, Lomb, Piem, VA.

***Physcia phaea* (Tuck.) J.W. Thomson**

Syn.: *Parmelia phaea* Tuck., *Physcia aipolia* (Ehrh. *ex* Humb.) Fürnr. subsp. phaea (Tuck.) Clauzade & Cl. Roux, *Physcia melops* Dufour *ex* Nyl.

L – Subs.: int – Alt.: 4–6 – Note: an arctic-alpine to boreal-montane, circumpolar lichen related to *Ph. aipolia*, found on siliceous rocks slightly manured by birds, with optimum above treeline, up to the nival belt in the Alps, where it is generally not common. – **Au**: T, S, K. **Sw**: GL, GR, UR. **Fr**: AHP, AMa. **It**: TAA, VA.

***Physcia stellaris* (L.) Nyl.**

Syn.: *Hagenia stellaris* (L.) De Not., *Lichen stellaris* L., *Parmelia stellaris* (L.) Ach., *Physcia aipolia* (Ehrh. *ex* Humb.) Fürnr. var. ambigua (Ehrh.) H. Olivier

L – Subs.: cor, xyl, sil – Alt.: 2–4 – Note: a (cool-) temperate to southern boreal-montane, circumpolar lichen of isolated trees. *Ph. biziana*, *Ph. aipolia* and *Ph. stellaris*, although often overlapping in their altitudinal distributions, are altitudinal vicariants in the Alps, *Ph. stellaris* has the optimum in and above the beech-belt, and is the most “continental” of the three species; widespread and common throughout the Alps. – **Au**: V, T, S, K, St, O, N, B. **Ge**: OB, Schw. **Sw**: AP, BE, GL, GR, LU, SG, SZ, TI, UW, VD, VS. **Fr**: AHP, HAl, AMa, Drô, Isè, Sav, HSav, Var, Vau. **It**: Frl, Ven, TAA, Lomb, Piem, VA, Lig. **Sl**: SlA, Tg. **Li**.

***Physcia tenella* (Scop.) DC.**

Syn.: *Borrera tenella* (Scop.) Ach., *Hagenia tenella* (Scop.) De Not., *Lichen tenellus* Scop., Parmelia stellaris (L.) Ach. var. tenella (Scop.) Spreng., *Parmelia tenella* (Scop.) Ach., *Physcia adscendens* (Fr.) H. Olivier var. tenella (Scop.) H. Olivier, Physcia stellaris (L.) Nyl. var. tenella (Scop.) Nyl., *Physcia subobscura* (Nyl.) Nyl.

L – Subs.: cor, xyl, cal – Alt.: 1–4 – Note: a mainly temperate species. Its separation from *Ph. adscendens* is not always clear: very characteristic specimens, hardly referrable to *Ph. adscendens*, are most common in the submediterranean belt in semi-natural situations. Some records might be due to confusion with young or poorly developed specimens of *Ph. adscendens*. – **Au**: V, T, S, K, St, O, N. **Ge**: OB, Schw. **Sw**: BE, FR, GL, GR, LU, SG, SZ, TI, UR, UW, VD, VS. **Fr**: AHP, HAl, AMa, Drô, Isè, Sav, HSav, Var, Vau. **It**: Frl, Ven, TAA, Lomb, Piem, VA, Lig. **Sl**: SlA, Tg.

***Physcia tribacia* (Ach.) Nyl.**

Syn.: *Lecanora tribacia* Ach., *Physcia erosa* (Borrer) Leight.

L – Subs.: int, cal cor, xyl – Alt.: 1–3 – Note: a widespread Mediterranean to xeric subtropical lichen found on basic siliceous rocks in sunny situations, often with *Peltula euploca* and ecologically related species, but less bound to periodical seepage of water; widespread in the Alps, being most common in the dry Alpine valleys. – **Au**: V, T, S, K, St. **Sw**: BE, GR, SZ, TI, VD, VS. **Fr**: AMa, Sav, Var. **It**: Ven, TAA, Lomb, Piem, VA.

***Physcia vitii* Nádv.**

L # – Subs.: sil, bry, cor – Alt.: 2–3 – Note: the circumscription of this species is not clear: it resembles a very stout *Ph. adscendens* without fibrils, and seems to be most common in heavily polluted areas. – **Au**: T. **Ge**: Schw. **Sw**: BE, GL, GR, TI. **It**: Frl, Ven, Lomb, Piem, Lig. **Li**.

***Physciella chloantha* (Ach.) Essl.**

Syn.: *Parmelia chloantha* Ach., *Phaeophyscia chloantha* (Ach.) Moberg, *Physcia luganensis* Mereschk., *Physcia obscura* (Ehrh.) Hampe *ex* Fürnr. var. chloantha (Ach.) Rabenh., *Physcia pragensis* Nádv.

L – Subs.: cor, cal – Alt.: 1–3 – Note: a mild-temperate, typically submediterranean species occurring on a wide range of substrata (mostly on bark of isolated trees, but also on limestone in open woodlands), but never common in heavily disturbed habitats. – **Au**: V, S, K, St, O, N. **Ge**: OB, Schw. **Sw**: BE, FR, GL, GR, LU, SG, TI, UW, VD, VS. **Fr**: AMa, Sav, HSav, Var, Vau. **It**: Frl, Ven, TAA, Lomb, Piem, VA. **Sl**: SlA, Tg.

***Physconia detersa* (Nyl.) Poelt**

Syn.: *Hagenia detersa* (Nyl.) Bagl., *Parmelia pulverulenta auct.* var. detersa Nyl., *Physcia detersa* (Nyl.) Nyl., *Physcia detersella* Nádv.

L – Subs.: cor, sil – Alt.: 2–4 – Note: on mossy rocks along steep slopes, and on trunks of deciduous trees in the montane belt. – **Au**: T, K, St, O. **Ge**: OB, Schw. **Sw**: BE, GR, VS. **Fr**: Isè. **It**: Ven, TAA, Lomb, Piem, VA, Lig. **Sl**: SlA.

***Physconia distorta* (With.) J.R. Laundon**

Syn.: *Lichen distortus* With., *Physcia pulverulenta auct. non* (Wahlenb.) Fürnrh., Physconia distorta (With.) J.R. Laundon f. subvenusta (Nyl.) J.Nowak, *Physconia pulverulacea* Moberg, *Physconia pulverulenta* (Wahlenb.) Poelt

L – Subs.: cor, xyl – Alt.: 1–3 – Note: a Mediterranean to temperate lichen of isolated trees, rare only in truly Mediterranean vegetation and in polluted areas, most frequent below the montane belt; widespread throughout the Alps. – **Au**: V, T, S, K, St, O, N, B. **Ge**: OB, Schw. **Sw**: AP, BE, FR, GL, GR, LU, SG, SZ, TI, UR, UW, VD, VS. **Fr**: AHP, HAl, AMa, Drô, Isè, Sav, HSav, Var, Vau. **It**: Frl, Ven, TAA, Lomb, Piem, VA, Lig. **Sl**: SlA, Tg. **Li**.

***Physconia enteroxantha* (Nyl.) Poelt**

Syn.: *Physcia enteroxantha* Nyl., *Physcia enteroxanthella* (Harm.) H. Olivier, *Physcia subdetersa* Nyl.

L – Subs.: cor – Alt.: 1–4 – Note: a Mediterranean to temperate species found on isolated trees, sometimes on mossy rocks; widespread throughout the Alps. – **Au**: T, S, K, St, O, N, B. **Ge**: OB, Schw. **Sw**: GL, GR, LU, SZ, VS. **Fr**: AHP, HAl, AMa, Var, Vau. **It**: Frl, Ven, TAA, Lomb, Piem, VA. **Sl**: SlA, Tg. **Li**.

**Physconia grisea (Lam.) Poelt subsp. grisea**

Syn.: *Hagenia pulverulenta auct.* var. pityrea (Ach.) Bagl. & Carestia, *Lichen griseus* Lam., *Parmelia farrea* Ach., *Parmelia pityrea* (Ach.) Ach., *Parmelia pulverulenta auct.* var. grisea (Lam.) Spreng., *Physcia farrea* (Ach.) Vain., *Physcia grisea* (Lam.) Zahlbr., Physcia grisea (Lam.) Zahlbr. var. pityrea (Ach.) Flagey, *Physcia pityrea* (Ach.) Nyl., *Physconia farrea* (Ach.) Poelt *non* *sensu* Poelt

L – Subs.: cor, sil, ter-sil – Alt.: 1–4 – Note: a mainly mild-temperate, perhaps holarctic lichen found both on bark (often on basal parts of isolated trees) and on calciferous rocks (especially calcareous sandstone, *e.g.* on walls); widespread throughout the Alps, with optimum below the montane belt, locally common also in urban areas. – **Au**: V, T, S, K, St, O, N, B. **Ge**: OB. **Sw**: BE, GL, GR, LU, SG, SZ, TI, UW, VD, VS. **Fr**: AHP, HAl, AMa, Drô, Isè, Sav, HSav, Var, Vau. **It**: Frl, Ven, TAA, Lomb, Piem, VA, Lig. **Sl**: SlA. **Li**.

**Physconia grisea (Lam.) Poelt subsp. lilacina (Arnold) Poelt**

Syn.: *Parmelia pulverulenta auct.* *f. lilacina* Arnold, Physcia grisea (Lam.) Zahlbr. var. lilacina (Arnold) Nádv., *Physcia lilacina* (Arnold) Poelt

L – Subs.: sax, xyl – Alt.: 1–3 – Note: on rock, more rarely on eutrophicated bark or lignum; mostly restricted to dry-warm sites in the Mediterranean belt and in dry-warm valleys of the Alps. – **Fr**: AHP, HAl, AMa, Sav, Vau. **It**: VA, Lig. **Sl**: SlA.

**Physconia muscigena (Ach.) Poelt var. muscigena**

Syn.: *Hagenia pulverulenta auct.* var. muscigena (Ach.) Bagl. & Carestia, *Parmelia muscigena* Ach., *Physcia muscigena* (Ach.) Nyl., Physcia pulverulenta (Wahlenb.) Fürnr. var. muscigena (Ach.) Nyl.

L – Subs.: ter-cal, ter-sil, bry, deb – Alt.: 4–6 – Note: an arctic-alpine, circumpolar lichen found on mosses and plant debris in open situations, such as in grasslands and on mosses growing on isolated calcareous boulders; widespread and common throughout the Alps. – **Au**: V, T, S, K, St, O, N. **Ge**: OB, Schw. **Sw**: BE, FR, GR, LU, SZ, TI, UR, UW, VD, VS. **Fr**: AHP, HAl, AMa, Isè, Sav, HSav, Vau. **It**: Frl, Ven, TAA, Lomb, Piem, VA, Lig. **Sl**: SlA. **Li**.

**Physconia muscigena (Ach.) Poelt var. bayeri (Nádv.) Poelt**

Syn.: *Physcia bayeri* Nádv.

L – Subs.: ter-cal, ter-sil, bry, deb – Alt.: 4–5 – Note: a variety differing from typical *Ph. muscigena* in the yellowish medulla, the colour intensifying with K; ecology comparable to that of the type variety, but also found on mossy faces of both calcareous and volcanic rocks, and perhaps more frequent at lower elevations; not generally distinguished, and therefore distribution in the Alps insufficiently known. – **Au**: T, K, St. **It**: VA.

***Physconia perisidiosa* (Erichsen) Moberg**

Syn.: *Physcia perisidiosa* Erichsen, *Physconia farrea auct. et* *sensu* Poelt *non* (Ach.) Poelt

L – Subs.: cor – Alt.: 1–3 – Note: a Mediterranean to mild-temperate lichen with a fragmented holarctic distribution, found both on bark and on epiphytic mosses; most common in submediterranean areas with a warm-suboceanic climate, but rare in disturbed habitats; widespread throughout the Alps below the subalpine belt. – **Au**: V, T, S, K, St, O, N, B. **Ge**: OB. **Sw**: BE, GL, GR, LU, SG, SZ, UW, VS. **Fr**: AHP, AMa, Drô, Isè, HSav, Var, Vau. **It**: Frl, Ven, TAA, Lomb, Piem, VA, Lig. **Sl**: SlA, Tg. **Li**.

***Physconia petraea* (Poelt) Vězda & Poelt**

Syn.: Physconia muscigena (Ach.) Poelt var. petraea Poelt

L – Subs.: sil, ter-sil, bry-sil – Alt.: 1–4 – Note: on base-rich siliceous rocks and epilithic mosses in dry-warm situations, and in need of further study. – **Au**: T, S, St. **Sw**: VS. **Fr**: AMa. **It**: TAA, Lomb, Piem, VA, Lig.

***Physconia servitii* (Nádv.) Poelt**

Syn.: *Physcia servitii* Nádv.

L – Subs.: cor – Alt.: 1–3 – Note: a Mediterranean-Atlantic to mild-temperate, mainly western lichen found on old trees in open woodlands; apparently restricted to the Western and Southern Alps. – **Fr**: AHP, AMa, Var, Vau. **It**: TAA, Lig.

***Physconia subpulverulenta* (Szatala) Poelt**

Syn.: *Physcia subpulverulenta* Szatala

L – Subs.: cor, bry-cor – Alt.: 2–3 – Note: a mainly Mediterranean-Atlantic lichen of isolated trees, in the study area so far known from the base of the Western Alps only. – **Fr**: Var.

***Physconia thorstenii* A. Crespo & Divakar**

L – Subs.: cor – Alt.: 2–3 – Note: this recently-described corticolous species grows on the nutrient-rich or moderately eutrophicated rough bark of a wide range of both deciduous and evergreen trees. Common in the Central Iberian Peninsula, it is also known from the southern Euro-Asiatic region (Italy, Austria, France, Greece, Cyprus, Saudi Arabia, Afghanistan, Pakistan, Tadzhikistan), and from North Africa (Morocco). It might have been confused with *Ph. distorta*, and might be more frequent. – **It**: TAA.

***Physconia venusta* (Ach.) Poelt**

Syn.: *Anaptychia subaquila* (Nyl.) Kurok., *Parmelia venusta* Ach., Physcia pulverulenta (Wahlenb.) Fürnr. var. venusta (Ach.) Nyl., *Physcia subaquila* Nyl., *Physcia venusta* (Ach.) Nyl.; incl. *Physcia amoena* (Zahlbr.) Nádv.

L – Subs.: cor – Alt.: 2–3 – Note: one of the few lichens whose distribution is centered on the Mediterranean mountains, and one of the most abundant and typical lichens of the Central and South Italian humid beech forests, which is rare in the Alps. The forms called *subaquila* are worthy of further study: they differ in the black lower surface and the saxicolous growth, and could represent a good species. – **Sw**: ?UR, ?VS. **Fr**: AHP, AMa, Var, Vau. **It**: Ven, TAA, Lomb, Lig. **Sl**: Tg.

***Piccolia ochrophora* (Nyl.) Hafellner**

Syn.: *Biatorella ochrophora* (Nyl.) Arnold, *Lecidea ochrophora* Nyl., *Strangospora ochrophora* (Nyl.) R.A. Anderson

L – Subs.: cor – Alt.: 2–3 – Note: a mild-temperate species found on *Populus*, but also on *Sambucus* and other trees with base-rich bark in rather shaded and humid situations; overlooked, but certainly rare and declining in the Alps. – **Au**: V, T, K, St, O, N, B. **Ge**: OB. **Sw**: BE, SG, SZ. **Fr**: Var, Vau. **Sl**: SlA.

***Pilophorus cereolus* (Ach.) Th. Fr**

Syn.: *Lichen cereolus* Ach., Pilophorus robustus Th. Fr. var. cereolus (Ach.) Th. Fr., *Stereocaulon cereolinum* Ach.

L – Subs.: sil – Alt.: 3–4 – Note: an arctic-alpine, probably incompletely circumpolar lichen found on siliceous rocks in moist-wet situations near treeline; all records from the Alps are historical. – **Au**: T. **Sw**: UR. **It**: TAA, Piem.

***Placidiopsis cinerascens* (Nyl.) Breuss**

Subs.: ter – Alt.: 1–2

Syn.: *Catapyrenium circinatum* (Bagl.) Jatta, *Dermatocarpon cinerascens* (Nyl.) Zahlbr., *Endocarpon cinerascens* Nyl., *Endocarpon circinatum* (Bagl.) Lojka, *Placidiopsis circinata* Bagl., *Placidiopsis pisana* Bagl., *Placidium cinerascens* (Nyl.) Arnold, *Placocarpus cinerascens* (Nyl.) Trevis., *Verrucaria cinerascens* (Nyl.) Nyl.

L – Subs.: ter – Alt.: 1–2 – Note: on clayey, somewhat calciferous but often superficially decalcified soil in grasslands and garrigues below the montane belt; apparently more common in the Western and Southern Alps. – **Fr**: AHP, AMa, Var, Vau. **It**: Ven, Piem, VA.

***Placidiopsis crassa* (Anzi) Clauzade & Cl. Roux**

Subs.: sil – Alt.: 2

Syn.: *Dermatocarpon crassum* (Anzi) Zahlbr., *Endocarpon crassum* Anzi

L – Subs.: sil – Alt.: 2 – Note: a rare species found on periodically flooded siliceous rocks below the montane belt. – **Fr**: Var. **It**: Lomb.

***Placidiopsis custnani* (A. Massal.) Körb.**

Subs.: ter-cal – Alt.: 1–3

Syn.: *Catapyrenium custnani* (A. Massal.) Jatta, *Dermatocarpon cartilagineum* (Nyl.) Zahlbr., *Dermatocarpon crenulatum* (Nyl.) Mig., *Endocarpidium custnani* (A. Massal.) Müll. Arg., Endocarpon cinereum Pers. var. cartilagineum Nyl., *Endocarpon custnani* (A. Massal.) Hepp, *Endopyrenium cartilagineum* (Nyl.) P. Syd., *Paraplacidiopsis crenulata* (Nyl.) Servít, *Placidiopsis cartilaginea* (Nyl.) Vain., *Placidiopsis crenulata* (Nyl.) Zschacke, *Placidium custnani* A. Massal., Verrucaria cinerascens (Nyl.) Nyl. var. crenulata Nyl., *Verrucaria crenulata* (Nyl.) Nyl.

L – Subs.: ter-cal, bry-cal – Alt.: 1–5 – Note: an often overlooked terricolous lichen found on calciferous soil and calcicolous mosses, which seems to be most common in the submediterranean belt; apparently more frequent in the Western and Southern Alps. – **Au**: K. **Ge**: Schw. **Sw**: VS. **Fr**: AHP, AMa, HSav, Var, Vau. **It**: Ven, TAA, Lomb, Piem, VA.

***Placidiopsis dermatocarpoides* Anzi**

Syn.: *Catapyrenium dermatocarpoides* (Anzi) Jatta, *Verrucaria dermatocarpoides* (Anzi) Stizenb.

L # – Subs.: sil – Alt.: 4 – Note: a species characterised by a combination of hyaline rhizohyphae and dark brown pigmented ascomatal walls; known only from the type collection in Italy, on soil in fissures of serpentine. – **It**: Lomb.

***Placidiopsis oreades* Breuss**

L – Subs.: ter-cal – Alt.: 4–5 – Note: a species resembling *P. cinerascens* in habitus and in the presence of hyaline rhizohyphae, but lacking a distinct epinecral layer, with a paraplectenchymatic lower cortex becoming brownish with age; on marly soil and in crevices, with scattered records from the mountains of Central Europe and Inner Asia, apparently rare in the Alps. – **Au**: K. **Ge**: OB. **Sw**: BE.

***Placidiopsis pseudocinerea* Breuss**

Syn.: *Placidiopsis cervinaauct. scand*. *non* (Nyl.) Vain.

L – Subs.: ter-cal, bry – Alt.: 4–5 – Note: an arctic-alpine, circumpolar lichen found on soil and on moribund bryophytes on siliceous, base-rich or slightly calciferous soil (*e.g.* on calcareous schist), with optimum near and above treeline; it can be easily confused with *Catapyrenium cinereum* and is certainly much more widespread in the Alps. – **Au**: T, S, K, St. **Sw**: GR, VD, VS. **Fr**: AHP, Sav, HSav. **It**: TAA, Piem, VA.

***Placidiopsis tiroliensis* Breuss**

L – Subs.: ter-cal – Alt.: 4–5 – Note: a species resembling *P. cinerascens*, but with brown rhizohyphae, distinguished from the ecologically similar and much more common *P. pseudocinerea* by the broadly ellipsoid to subspherical ascospores; in crevices of calcareous rocks; so far there are only a few records from the Alps. – **Au**: V, T, St, K. **Sw**: VD. **Fr**: HSav.

***Placidium adami-borosi* Szatala**

Syn.: *Catapyrenium adami-borosi* (Szatala) Breuss

L – Subs.: ter – Alt.: 3 – Note: a mainly Mediterranean (-montane) lichen found on soil derived from metamorphic, base-rich rocks in dry grasslands, morphologically and anatomically similar to *P. lachneum*, but with a different ecology and distribution; from the Alps there is a single record (Switzerland). – **Sw**: TI.

***Placidium boccanum* (Servít) Breuss**

Syn.: *Catapyrenium boccanum* (Servít) Breuss, *Dermatocarpon boccanum* Servít

L – Subs.: ter-cal, cal – Alt.: 1–2 – Note: a Mediterranean to mild-temperate lichen growing on calciferous clayey soil, often also found on walls, including those of mortar; very rare in the Alps. – **Fr**: AMa. **It**: Ven.

***Placidium imbecillum* (Breuss) Breuss**

Syn.: *Catapyrenium imbecillum* Breuss

L – Subs.: ter-cal, bry – Alt.: 4–5 – Note: a terricolous species known from the Alps and from several isolated stations in Southern Europe, with optimum near and above treeline. – **Au**: V, T, K, St, O. **Sw**: GR, TI, UW. **Sl**: SlA.

**Placidium lachneum (Ach.) B. de Lesd. var. lachneum**

Syn.: *Catapyrenium lachneum* (Ach.) R. Sant., *Dermatocarpon lachneum* (Ach.) A.L. Sm., *Endopyrenium lachneum* (Ach.) Hav., *Lichen lachneus* Ach.

L – Subs.: ter-cal, ter-sil, cal – Alt.: 3–6 – Note: a mainly boreal-montane to arctic-alpine, circumpolar lichen found on terricolous bryophytes and on more or less organic calciferous soil in upland areas; widespread throughout the Alps. – **Au**: V, T, S, K, St, O, N. **Ge**: OB. **Sw**: BE, GR, LU, SZ, VS. **Fr**: AHP, HAl, AMa, Isè, Sav, HSav. **It**: Frl, Ven, TAA, Lomb, Piem, VA. **Sl**: SlA.

**Placidium lachneum (Ach.) B. de Lesd. var. oleosum (Breuss) Breuss**

Syn.: Catapyrenium lachneum (Ach.) R. Sant. var. oleosum Breuss

L – Subs.: ter-cal, ter-sil, deb – Alt.: 3–6 – Note: a variety without or with a few pycnidia only, and cells of the lower cortex with many oil droplets; on soil over a wide range of rocks; overall distribution arctic-alpine; widespread in the Alps, mainly from the alpine to nival belt, but not always distinguished. – **Au**: T, S, K, St, O. **Ge**: OB. **Sw**: GR, VD, VS. **Fr**: HAl, AMa.

***Placidium michelii* A. Massal.**

Syn.: *Catapyrenium michelii* (A. Massal.) R. Sant., *Dermatocarpon michelii* (A. Massal.) Zwackh, *Endocarpon michelii* (A. Massal.) Bausch, *Endopyrenium michelii* (A. Massal.) Körb.

L – Subs.: ter-cal, deb – Alt.: 2–3 – Note: a mainly temperate lichen found on mineral, especially sandy soil in open grasslands. – **Sw**: BE, GR, TI. **Fr**: AMa, Isè. **It**: Ven, Lomb, Piem.

***Placidium norvegicum* (Breuss) Breuss**

Syn.: *Catapyrenium norvegicum* Breuss

L – Subs.: ter-cal, bry – Alt.: 4–6 – Note: a species with a thallus usually forming rosettes with incised-lobate margins, large ascospores, and rod-shaped conidia developing inside laminal pycnidia; on mossy soil; overall distribution arctic-alpine, with a few records from the Alps. – **Au**: T, S, St, N. **Sw**: GR, VS.

***Placidium pilosellum* (Breuss) Breuss**

Syn.: *Catapyrenium bullatescens* P.M. McCarthy, *Catapyrenium pilosellum* Breuss

L – Subs.: bry, ter-cal – Alt.: 1–4 – Note: a Mediterranean to mild-temperate lichen found on more or less calciferous soil rich in humus, often growing amongst bryophytes; widespread throughout the Alps, below the subalpine belt. – **Au**: T, St, O, N. **Sw**: BE, GR, TI, UR, VS. **Fr**: AHP, HAl, AMa, Var, Vau. **It**: Frl, Ven, TAA, Piem, Lig. **Sl**: SlA.

***Placidium rufescens* (Ach.) A. Massal.**

Syn.: Catapyrenium lachneum (Ach.) R. Sant. subsp. rufescens (Ach.) Clauzade & Cl. Roux, *Catapyrenium rufescens* (Ach.) Breuss, *Dermatocarpon rufescens* (Ach.) Th. Fr., *Dermatocarpon rufopallens* (Nyl.) Zahlbr., *Dermatocarpon terrigenum* Tomin, *Endocarpon rufescens* Ach., *Endocarpon rufopallens* Nyl., *Endopyrenium rufescens* (Ach.) Körb., *Endopyrenium rufopallens* (Nyl.) Müll. Arg.

L – Subs.: cal, int, sil, ter-cal, bry – Alt.: 1–5 – Note: a Mediterranean to (mainly) mild-temperate, holarctic lichen found on vertical seepage tracks of calcareous rocks, almost always with colonies of cyanobacteria, more rarely on plant debris, calciferous soil, terricolous or epilithic bryophytes; widespread throughout the Alps, reaching the alpine belt in sunny, warm sites. – **Au**: V, T, S, K, St, O, N. **Ge**: OB. **Sw**: BE, GR, LU, SZ, TI, UR, VD, VS. **Fr**: AHP, HAl, AMa, Isè, Sav, HSav, Var, Vau. **It**: Frl, Ven, TAA, Lomb, Piem, VA, Lig.

***Placidium savonicum* (Servít) ined. (provisionally placed here, ICN Art. 36.1b)**

Syn.: *Involucrocarpon savonicum* Servít

L # – Subs.: cal – Alt.: 2 – Note: a species with a verrucose to squamulose, brown thallus consisting of areoles and squamules (to 0.5 mm in diam.) with free, slightly elevated margins, lower cortex not developed, ascomata not protruding, visible only by the dots of the ostioles (*c.* 0.1 mm in diam.), a thin, entire involucrellum, and oblong ascospores (to *c.* 22 µm long); on calcareous rocks; only known from the type locality in Italy. – **It**: Lig.

***Placidium squamulosum* (Ach.) Breuss**

Syn.: *Catapyrenium squamulosum* (Ach.) Breuss, *Dermatocarpon trapeziformeauct. p.p. non Lichen trapeziformis* J. König, *Endocarpon squamulosum* Ach., *Endocarpon exiguum* Nyl.

L – Subs.: ter-cal, bry – Alt.: 1–5 – Note: a widespread holarctic lichen found on calciferous soil, often amongst bryophytes in open dry grasslands, with a rather wide altitudinal range; widespread throughout the Alps. – **Au**: V, T, S, K, St, O, N. **Ge**: OB. **Sw**: BE, GR, SZ, TI, VD, VS. **Fr**: AHP, HAl, AMa, Isè, Sav, HSav, Var, Vau. **It**: Frl, Ven, TAA, Lomb, Piem, Lig. **Sl**: SlA.

***Placidium tenellum* (Breuss) Breuss**

Syn.: *Catapyrenium tenellum* Breuss

L – Subs.: ter-cal – Alt.: 2 – Note: a widespread but rare species of dry, very open grasslands and garrigues on calcareous substrata, extending eastward to Mongolia, with a single record from low elevations in the Western Alps (France). – **Fr**: Vau.

***Placidium velebiticum* (Zahlbr. *ex* Zschacke) Breuss**

Syn.: *Catapyrenium velebiticum* (Zahlbr. *ex* Zschacke) Breuss & Etayo, *Dermatocarpon velebiticum* Zahlbr. *ex* Zschacke

L – Subs.: ter-cal – Alt.: 3 – Note: a species resembling *P. rufescens* in *e.g.* the marginal pycnidia, but squamules thinner (hardly exceeding 400 µm) and ascospores narrower; overgrowing calcicolous bryophytes in temporarily moist sites; widespread in Southern and Central Europe but altogether rare, with a few records from the Eastern Alps (Austria). – **Au**: O, N. **Sl**: SlA.

***Placocarpus melanophthalmosus* Cl. Roux & Gueidan**

L – Subs.: sil – Alt.: 5 – Note: a recently-described species resembling *P. schaereri*, but parasitic on *Rhizoplaca melanophthalma*, with smaller squamules, a thinner medulla, and smaller ascospores with a thinner halo. – **Fr**: AHP.

***Placocarpus schaereri* (Fr.) Breuss**

Syn.: *Catapyrenium schaereri* (Fr.) R. Sant., *Dermatocarpon monstrosum* (Schaer.) Vain., *Dermatocarpon saxorum* (Chaillet) Trevis., *Endocarpon miniatum* (L.) P. Gaertn., G. Mey. & Scherb. var. monstrosum Schaer., *Endocarpon monstrosum* (Schaer.) A. Massal., *Endopyrenium monstrosum* (Schaer.) Hazsl., *Parmelia schaereri* Fr., *Placidium monstrosum* (Schaer.) A. Massal., *Verrucaria schaereri* (Fr.) Nyl.

L – Subs.: cal-par, cal – Alt.: 1–4 – Note: a Mediterranean to mild-temperate lichen found on exposed calcareous boulders, with optimum in the submediterranean belt; when young, it is a constant parasite on *Protoparmeliopsis versicolor*. – **Au**: K, St, N. **Sw**: VS. **Fr**: AHP, HAl, AMa, Drô, Isè, Sav, HSav, Var, Vau. **It**: Frl, Ven, TAA, Lomb, Piem, VA, Lig.

***Placolecis opaca* (Dufour) Hafellner**

Syn.: *Astroplaca opaca* (Dufour) Bagl., *Biatora opaca* (Dufour) Jatta, *Lecidea endochrysoides* Hue, *Lecidea opaca* Dufour, *Psora opaca* (Dufour) A. Massal.

L – Subs.: cal – Alt.: 1–2 – Note: a calcicolous lichen found in the Mediterranean and (more rarely) submediterranean belts, on both shaded and sunny surfaces of compact calcareous rocks; its distribution extends widely into Central Asia; apparently most frequent in the Southern and Western Alps, at low elevations, but generally rare; the records from Switzerland are dubious. – **Sw**: ?GR. **Fr**: AMa, Var, Vau. **It**: Frl, Ven, TAA, Lomb, Piem, Lig.

***Placopsis gelida* (L.) Linds.**

Syn.: *Lecanora gelida* (L.) Ach., Lecanora gelida (L.) Ach. f. neglecta Degel., *Lichen gelidus* L., *Placodium gelidum* (L.) Gray, *Squamaria gelida* (L.) Hook.

L – Subs.: sil – Alt.: 3–5 – Note: a boreal-montane to arctic-alpine, incompletely circumpolar lichen found on small siliceous pebbles and on basal parts of large boulders, mostly in moist situations of the upper montane and subalpine belts; rare in the Alps. – **Au**: T, S, St. **Fr**: HSav. **It**: Piem.

***Placopsis lambii* Hertel & V. Wirth**

L – Subs.: sil – Alt.: 2–4 – Note: a species resembling juvenile thalli of *P. gelida* without cephalodia, usually sorediate, rarely with apothecia and normally poorly developed; ecologically similar to *Lecanora subaurea*, found on low outcrops and small boulders of metal-rich siliceous rocks in sites with an oceanic climate; in the study area known from a few localities, all below treeline in the Eastern Alps (Austria). – **Au**: V, S, O.

***Placopyrenium breussii* Cl. Roux & Gueidan**

L – Subs.: sil-par – Alt.: 2–4 – Note: a species resembling *P. formosum* in the grey, areolate, non-lobate thallus, but ascospores with a thin perispore; lichenicolous on thalli of *Aspicilia calcitrapa* on basic to subneutral siliceous rocks in dry-warm sites; recently described from the Pyrenees and so far only known from the western Mediterranean region, with records from the base of the Western Alps (France). – **Fr**: AMa.

***Placopyrenium canellum* (Nyl.) Gueidan & Cl. Roux**

Syn.: *Verrucaria aspiciliae* Zehetl. *non* (J. Lahm) Stizenb. *nec* Vain., *Verrucaria aspiciliicola* R. Sant., *Verrucaria canella* Nyl.

L – Subs.: cal-par – Alt.: 1–5 – Note: a very common saxicolous species which starts the life-cycle on species of the *Aspicilia calcarea*-complex; certainly more widespread in the Alps, but formerly misunderstood. – **Au**: St, N. **Fr**: AHP, AMa, Drô, Var, Vau. **It**: Frl, Ven, Piem, Lig.

***Placopyrenium cinereoatratum* (Degel.) Orange**

Syn.: *Verrucaria cinereoatrata* Degel.

L – Subs.: sil-aqu(-par) – Alt.: 2–5 – Note: a species resembling *P. fuscellum*, with a crustose to subsquamulose, irregularly areolate, pruinose thallus; a facultative parasite of *Staurothele fissa* on siliceous rocks along streams and lakes; widespread in Western and Northern Europe but rare, with two isolated records from the Alps, but perhaps not recognised, and therefore overlooked elsewhere. – **Au**: T. **Fr**: AMa.

***Placopyrenium formosum* Orange**

L – Subs.: sil-aqu(-par) – Alt.: 2–3 – Note: a species resembling *P. canellum* in the grey, areolate, non-lobate thallus, but with smaller ascospores; a facultative parasite of *Aspicilia aquatica* on siliceous rocks along streams; widespread in Western and Northern Europe but rare, with records only from the Western Alps (France). – **Fr**: AMa.

***Placopyrenium fuscellum* (Turner) Gueidan & Cl. Roux**

Syn.: *Lichen fuscellus* Turner, *Lithocia fuscella* (Turner) A. Massal., *Verrucaria fuscella* (Turner) Winch, *Verrucaria glaucelloides* Hepp, *Verrucaria glaucin*a * auct. non* Ach., *Verrucaria glebulosa* Nyl., *Verrucaria griseoatra* (Kremp.) Servít

L – Subs.: cal, int – Alt.: 1–5 – Note: on steeply inclined calciferous rocks (mainly limestone and dolomite), often on *Verrucaria nigrescens*; a polymorphic taxon in need of revision. – **Au**: ?V, T, S, K, St, O, N. **Ge**: Ge. **Sw**: AP, BE, GR, VS. **Fr**: AHP, HAl, AMa, Drô, Isè, Sav, HSav, Var, Vau. **It**: Frl, Ven, TAA, Lomb, Piem, VA. **Sl**: SlA.

***Placopyrenium gorzegnoense* (Servít) ined. (provisionally placed here, ICN Art. 36.1b)**

Syn.: *Dermatocarpon gorzegnoense* Servít

L # – Subs.: cal – Alt.: 2 – Note: a species with a spreading, verrucose-areolate to squamulose thallus, the squamules (to 1 mm in diam.) subdivided into whitish pruinose verruculae (to 0.2 mm in diam.), with a thick, brown, paraplectenchymatic basal layer, ascomata 1–3 per squamule only slightly protruding, involucrellum lacking, ascospores oblong to ellipsoid (to *c.* 20 µm long); on a calcareous rock, only known from the type locality in the Western Alps (Italy). – **It**: Piem.

***Placopyrenium tatrense* (Vězda) Breuss**

Syn.: *Placidiopsis tatrensis* Vězda

L – Subs.: sil, cal – Alt.: 3–4 – Note: widespread but uncommon in the Pyrenees, the Alps, Carpathians and the Balkan Mountains and Crimea, mainly in the upper montane to the alpine belt. – **Au**: S. **Sw**: GR, VS. **Fr**: AMa.

**Placopyrenium trachyticum (Hazsl.) Breuss var. trachyticum**

Syn.: *Catapyrenium trachyticum* (Hazsl.) R. Sant., *Dermatocarpon trachyticum* (Hazsl.) Vain., *Endopyrenium trachyticum* Hazsl., *Placidiopsis trachytica* (Hazsl.) Servít

L – Subs.: cal, sil – Alt.: 1–4 – Note: on base-rich siliceous rocks. This species, frequently confused with *P. fuscellum*, extends more widely in the submediterranean belt than *P. bucekii*. – **Au**: K, N. **Fr**: AMa, Var, Vau. **It**: Piem, VA.

**Placopyrenium trachyticum (Hazsl.) Breuss var. subtrachyticum (B. de Lesd.) Breuss**

Syn.: *Catapyrenium subtrachyticum* B. de Lesd., *Dermatocarpon subtrachyticum* (B. de Lesd.) Zahlbr., Dermatocarpon trachyticum (Hazsl.) Vain. var subtrachyticum (B. de Lesd.) Servít, *Placidiopsis subtrachytica* (B. de Lesd.) Zschacke

L – Subs.: cal-par – Alt.: 2 – Note: a variety differing from typical *P. trachyticum* in the presence of intermixed 1-septate ascospores; on various types of rocks; rare throughout Europe at low elevations, with a few records from the Western Alps (France). – **Fr**: AMa.

***Placynthiella dasaea* (Stirt.) Tønsberg**

Syn.: *Lecidea dasaea* Stirt.

L – Subs.: cor, xyl, ter-sil – Alt.: 2–4 – Note: on acid soil, lignum and bark in upland areas; probably more widespread in the Alps, but overlooked. – **Au**: T, O. **Sw**: GL, GR, TI, UR, VS. **It**: Ven, TAA, Lomb. **Sl**: SlA.

***Placynthiella hyporhoda* (Th. Fr.) Coppins & P. James**

Syn.: *Lecidea hyporhoda* Th. Fr., Lecidea uliginosa (Schrad.) Ach. f. hyporhoda (Th. Fr.) Hedl., *Saccomorpha hyporhoda* (Th. Fr.) Clauzade & Cl. Roux

L – Subs.: cor, xyl, ter – Alt.: 3–4 – Note: on soil rich in heavy metals in upland areas; probably more widespread in the Alps, but overlooked – **It**: TAA.

***Placynthiella icmalea* (Ach.) Coppins & P. James**

Syn.: *Biatora fuliginea* (Ach.) Fr., Biatora uliginosa (Schrad.) Fr. var. fuliginea (Ach.) Fr., *Lecanora terricola* Ach., *Lecidea fuliginea* Ach., *Lecidea icmalea* Ach., *Lecidea trachylina* Nyl., Lecidea uliginosa (Schrad.) Ach. var. fuliginea (Ach.) Link, *Pannularia perfurfurea* Nyl., *Parmeliella perfurfurea* (Nyl.) Zahlbr., *Placynthiella perfurfurea* (Nyl.) Gyeln., *Saccomorpha icmalea* (Ach.) Clauzade & Cl. Roux

L – Subs.: xyl, bry, deb, cor, ter-sil – Alt.: 2–5 – Note: a widespread, mainly northern holarctic lichen found on disturbed soil, turf, decomposed lignum (common on stumps), much more rarely on acid bark, and then mostly on basal parts of trunks; widespread throughout the Alps. – **Au**: V, T, S, K, St, O, N, B. **Ge**: OB, Schw. **Sw**: BE, GL, GR, LU, SZ, TI, UR, VD, VS. **Fr**: AHP, AMa, Isè, Sav, HSav, Var, Vau. **It**: Frl, Ven, TAA, Lomb, Piem, VA, Lig. **Sl**: SlA.

***Placynthiella oligotropha* (J.R. Laundon) Coppins & P. James**

Syn.: *Lecidea oligotropha* J.R. Laundon, *Saccomorpha oligotropha* (J.R. Laundon) Clauzade & Cl. Roux

L – Subs.: bry, deb, ter-sil, ter-cal, sil – Alt.: 3–5 – Note: a cool-temperate to boreal-montane, probably circumpolar lichen found on soil and turf, more rarely on weathering siliceous rocks, mostly in clearings of woodlands in upland areas; widespread throughout the Alps. – **Au**: V, T, S, K, St, O, N. **Ge**: OB, Schw. **Sw**: GR, LU, SZ, TI, UR, UW, VS. **Fr**: AMa, HSav. **It**: Frl, Ven, TAA, Lomb, Piem. **Sl**: SlA.

***Placynthiella uliginosa* (Schrad.) Coppins & P. James**

Syn.: *Biatora humosa* (Hoffm.) Arnold, *Biatora uliginosa* (Schrad.) Fr., Biatora uliginosa (Schrad.) Fr. var. humosa (Hoffm.) Fr., *Lecidea humosa* (Hoffm.) Leight., *Lecidea uliginosa* (Schrad.) Ach., Lecidea uliginosa (Schrad.) Ach. var. humosa (Hoffm.) Ach., *Lichen uliginosus* Schrad., *Saccomorpha arenicola* Elenkin, *Saccomorpha uliginosa* (Schrad.) Hafellner

L – Subs.: bry, deb, ter-sil – Alt.: 2–5 – Note: a cool-temperate to boreal-montane, probably circumpolar lichen, mostly found on acid soil, more rarely on strongly decomposed lignum; widespread throughout the Alps. – **Au**: V, T, S, K, St, O, N, B. **Ge**: OB, Schw. **Sw**: BE, GR, LU, SZ, TI, UR, UW, VD, VS. **Fr**: AHP, HAl, AMa, Sav, HSav, Var, Vau. **It**: Frl, Ven, TAA, Lomb, Piem, VA. **Sl**: SlA, Tg.

***Placynthium anemoideum* (Servít) Gyeln.**

Syn.: Placynthium subradiatum (Nyl.) Arnold f. anemoideum Servít

L # – Subs.: cal-aqu – Alt.: 2–3 – Note: a species with a blackish crustose thallus composed of subsquamulose, roundish areoles (not effigurate at the margin), with sessile, black apothecia and one-septate ascospores, based on a type from Croatia; on calcareous rocks, ecology poorly known but in the protologue contact to running water is not indicated; the only record from the Alps (France) needs critical re-evaluation. – **Fr**: AHP.

***Placynthium asperellum* (Ach.) Trevis.**

Syn.: *Catillaria subalpina* Th. Fr., *Collema asperellum* Ach., *Placynthium aspratile* (Ach.) Henssen, *Placynthium vrangianum* Gyeln., *Pterygium asperellum* (Ach.) Nyl., *Toninia asperella* (Ach.) A. Massal.

L – Subs.: sil, int – Alt.: 4–5 – Note: on moist calciferous and base-rich siliceous rocks in upland areas. – **Au**: V, T, S, K, St. **Ge**: Schw. **Sw**: GR, SZ, VS. **Fr**: AHP, AMa. **It**: Lomb.

***Placynthium baumgartneri* (Zahlbr.) Gyeln.**

Syn.: *Placynthium coerulescens* (Harm.) Gyeln., *Pterygium baumgartneri* Zahlbr., *Pterygium coerulescens* Harm.

L – Subs.: cal, cal-aqu – Alt.: 2–5 – Note: a species with an effigurate thallus lacking a prothallus, the areoles in the centre often falling off and the marginal lobes in dense radial arrangement, flat, brownish with a greyish pruina, sessile black apothecia, and 3-septate ascospores; on steep faces of calcareous rocks; known from Southern and Central Europe, with several records from Austria and France (paratype of *P. coerulescens*), perhaps overlooked elsewhere, or misidentified as *P. subradiatum*. – **Au**: T, S, St, O. **Fr**: AHP, AMa, Var.

***Placynthium caesium* (Fr.) Jatta**

Syn.: *Bacidia caesitia* (Nyl.) Jatta, *Collolechia caesia* (Fr.) A. Massal., *Lecidea caesitia* Nyl., Lecidea contigua (Hoffm.) Fr. var. caesia Fr., Lecidea triptophylla Ach. var. caesia Schaer., Pannaria nigra (Huds.) Nyl. var. caesia (Fr.) Malbr., *Placynthium caesitium* (Nyl.) Hue, Placynthium garovaglii (A. Massal.) Malme var. subtile G. Czeika, *Scoliciosporum caesitium* (Nyl.) Jatta

L – Subs.: cal – Alt.: 2–4 – Note: a species with a partly unusual set of characters (but generic arrangement recently confirmed by molecular data), with a crustose thallus lacking a prothallus, black biatorine apothecia, and 3–6-septate, fusiform ascospores; a mainly southern species in Europe, found on steeply inclined surfaces of calcareous rocks with some water seepage after rain. In Northern Europe it has been frequently confused with *P. garovaglii*, which is a completely different species, although material with poorly developed marginal lobes may be difficult to identify. – **Au**: T, O, N. **Sw**: GR, UW, VS. **Fr**: AMa, Drô, HSav. **It**: Ven, TAA, Lomb, Piem.

***Placynthium dolichoterum* (Nyl.) Trevis.**

Syn.: *Lecothecium pluriseptatum* Arnold, *Pannaria dolichotera* Nyl., *Parmeliella melantera* (Stirt.) A.L. Sm., *Placynthium pluriseptatum* (Arnold) Arnold

L # – Subs.: cal, sil – Alt.: 4–5 – Note: on basic siliceous or slightly calciferous rocks in humid-sheltered situations near or above treeline. A poorly known species of the *P. nigrum*-complex, which badly needs revision. – **Au**: V, T, K, St, O, N. **Ge**: OB. **Sw**: SZ, VS. **Fr**: AHP, AMa, Sav, HSav. **It**: Frl, TAA. **Sl**: SlA, Tg.

***Placynthium filiforme* (Garov.) M. Choisy**

Syn.: *Leptogium cornicularioides* Bagl., *Parmelia filiformis* Garov., *Polychidium centrifugum* (Nyl.) Jatta, *Pterygium centrifugum* Nyl., *Pterygium filiforme* (Garov.) A.L. Sm., *Wilmsia centrifuga* (Nyl.) Körb.

L – Subs.: cal – Alt.: 1–5 – Note: a Mediterranean (-montane) to mild-temperate lichen found on steeply inclined seepage tracks of calcareous rocks, with a rather wide altitudinal range. – **Au**: V, T, S, K, St, O, N. **Ge**: OB, Schw. **Sw**: BE, SZ, UW. **Fr**: AHP, AMa, HSav, Var, Vau. **It**: Frl, Ven, TAA, Lomb. **Sl**: SlA.

***Placynthium flabellosum* (Tuck.) Zahlbr.**

Syn.: *Anziella adglutinata* (Anzi) Gyeln., *Lecothecium adglutinatum* Anzi, *Pannaria flabellosa* Tuck., *Placynthium adglutinatum* (Anzi) Trevis.

L – Subs.: sil – Alt.: 3–4 – Note: a temperate to boreal-montane, perhaps circumpolar lichen found on moist siliceous rocks (inundation zones along streams, seepage tracks), often near mountain rivulets. – **Au**: T, S, St. **Fr**: HAl, AMa, HSav, Var. **It**: TAA, Lomb.

***Placynthium garovaglii* (A. Massal.) Malme**

Syn.: *Placynthium caesium auct.*, *Racoblenna garovaglii* A. Massal.

L – Subs.: cal – Alt.: 1–5 – Note: on steeply inclined, sunny surfaces of calcareous rocks with some water seepage. The species name is often spelled *garovaglioi*, but the latinised name of Santo Garovaglio (who wrote most of his works in Latin) was *Garovaglius*, whose genitive is *garovaglii*. – **Au**: V, T, S, K, St, O, N. **Ge**: OB, Schw. **Sw**: BE, GR, LU, SZ, UW, VD, VS. **Fr**: AHP. **It**: Frl, Ven, TAA, Lomb, Piem.

***Placynthium hungaricum* Gyeln.**

L – Subs.: cal – Alt.: 1–5 – Note: a Mediterranean to mild-temperate species found on steeply inclined, sun-exposed seepage tracks of calcareous rocks, usually below the subalpine belt. – **Au**: V, T, St, O, N. **Sw**: SZ, GR. **Fr**: AHP, AMa, Drô, Sav, HSav, Var, Vau. **It**: Frl, Lomb.

***Placynthium lismorense* (Cromb.) Vain.**

Syn.: *Pterygium lismorense* Cromb.

L – Subs.: cal-aqu – Alt.: 2–4 – Note: on more or less calcareous rocks along seepage tracks at relatively low elevations. – **Sw**: SZ. **Fr**: AMa, Var.

***Placynthium nigrum* (Huds.) Gray**

Syn.: *Collema nigrum* (Huds.) Hoffm., *Lecothecium nigrum* (Huds.) A. Massal., *Lichen niger* Huds., *Pannaria nigra* (Huds.) Nyl., *Pannaria psotina* (Nyl.) Leight., *Pannularia nigra* (Huds.) Nyl., *Placynthium corallinoides* Jatta, *Placynthium siliceum* Gyeln.

L – Subs.: cal, int, sil – Alt.: 1–5 – Note: a probably holarctic, subtropical to subarctic species found on more or less calciferous rocks, often near the ground in sheltered situations, from the Mediterranean belt (only in shaded-humid situations) to the mountains, also common in small urban settlements (*e.g.* on north-facing walls); widespread and common throughout the Alps. – **Au**: V, T, S, K, St, O, N, B. **Ge**: OB, Schw. **Sw**:  BE, GR, LU, SZ, TI, VD, VS. **Fr**: AHP, HAl, AMa, Drô, Isè, Sav, HSav, Var, Vau. **It**: Frl, Ven, TAA, Lomb, Piem, VA, Lig. **Sl**: SlA, Tg.

***Placynthium pannariellum* (Nyl.) H. Magn.**

Syn.: *Pterygium pannariellum* Nyl.

L – Subs.: sil – Alt.: 2–4 – Note: a species somehow resembling *P. flabellosum*, from which it might not have been always distinguished, but with a squamulose, olive-brown thallus on a bluish-black prothallus, narrow, elongated marginal lobes which are only loosely attached to the substrate, a convex upper surface with longitudinal fine grooves, the central squamules with isidioid to ligulate outgrowths of variable length; rarely fertile; on temporarily inundated siliceous rocks along streams and lake shores; widespread in Europe but much more common in the North. – **Au**: T, K, St. **Fr**: AHP, AMa.

***Placynthium posterulum* (Nyl.) Henssen**

Syn.: *Pterygium posterulum* Nyl.

L – Subs.: cal – Alt.: 3–5 – Note: a species recalling a poorly developed *P. subradiatum*, with which it might have been confused, the olive-brown to blackish brown thalli forming rings with disintegrating centres, but radial growth often less prominent, marginal lobes more or less round in transverse section and lower surface lacking any blue to violet pigment, loosely attached to the substrate by bundles of rhizohyphae; on sunny, steep faces of calcareous rocks; widespread in Central Europe, with a few scattered records from Eastern Alps. – **Au**: V, T, St. **Ge**: OB.

***Placynthium rosulans* (Th. Fr.) Zahlbr.**

Syn.: *Lecothecium rosulans* Th. Fr., Placynthium pannariellum (Nyl.) H. Magn. var. rosulans (Th. Fr.) Degel.

L – Subs.: cal – Alt.: 4–5 – Note: a northern species found on moist siliceous rocks, *e.g.* in inundation zones along brooks, near or above treeline; known from a few localities in the Alps, but perhaps more widespread. The relationship with *P. pannariellum* still needs further study. – **Au**: T. **Ge**: OB. **It**: Piem.

***Placynthium subradiatum* (Nyl.) Arnold**

Syn.: *Lecothecium controversum* Anzi, *Lecothecium radiosum* Anzi, *Lecothecium subradiatum* (Nyl.) Dalla Torre & Sarnth., *Pannaria subradiata* Nyl., *Placynthium radiosum* (Anzi) Jatta, *Pterygium subradiatum* (Nyl.) Nyl., *Wilmsia radiosa* (Anzi) Körb.

L – Subs.: cal – Alt.: 1–5 – Note: on vertical, sun-exposed seepage tracks of calcareous rocks, with a wide altitudinal range; widespread throughout the Alps. – **Au**: V, T, S, K, St, O, N. **Ge**: OB, Schw. **Sw**: BE, GR, SZ, VD, VS. **Fr**: AHP, AMa, Sav, Var, Vau. **It**: Frl, Ven, TAA, Lomb, Lig. **Sl**: SlA.

***Placynthium tantaleum* (Hepp) Hue**

Syn.: Biatora corallinoides (Schaer.) Hepp var. tantalea Hepp, *Placynthium diblastum* Gyeln., Placynthium nigrum (Huds.) Gray var. tantaleum (Hepp) Arnold, *Racoblenna tantalea* (Hepp) Trevis.

L – Subs.: cal, sil, sil-aqu, cal-aqu – Alt.: 3–5 – Note: on basic siliceous rocks along mountain streams. Often considered as a synonym of *P. nigrum*, this species differs in the smooth areolae, the constantly 2-celled ascospores, and the ecology (hydro – and orophilous). – **Au**: T, K, St, O, N. **Ge**: Schw. **Sw**: BE, GR, VD, VS. **Fr**: AHP, HAl, AMa, Sav. **It**: Piem.

***Placynthium tremniacum* (A. Massal.) Jatta**

Syn.: *Racoblenna tremniaca* A. Massal.

L # – Subs.: cal – Alt.: 2–4 – Note: the 1-septate spores and the pruinose thallus with somewhat stouter, more or less flat, minute squamules, different marginal lobes, and the less developed prothallus distinguish this species from *P. nigrum*. The taxonomic value of these characters needs however further study. – **Au**: T, K, St, N. **Sw**: VS. **Fr**: AHP, AMa, Drô, Var. **It**: Ven, Lig.

***Platismatia glauca* (L.) W.L. Culb. & C.F. Culb.**

Syn.: *Cetraria fallax* (Weber) Anders, *Cetraria glauca* (L.) Ach., *Lichen glaucus* L., *Parmelia glauca* (L.) Hepp, *Platysma fallax* (Weber) Hoffm., *Platysma glaucum* (L.) Frege

L – Subs.: cor, xyl, sil – Alt.: 2–4 – Note: a cool-temperate to circumboreal species, abundant in the montane and subalpine belts, on bark of beech and of conifers, sometimes even on lignum. – **Au**: V, T, S, K, St, O, N, B. **Ge**: OB. **Sw**: AP, BE, FR, GL, GR, LU, SG, SZ, TI, UR, UW, VD, VS. **Fr**: AHP, HAl, AMa, Isè, Sav, HSav, Var, Vau. **It**: Frl, Ven, TAA, Lomb, Piem, VA, Lig. **Sl**: SlA, Tg. **Li**.

***Pleopsidium chlorophanum* (Wahlenb.) Zopf**

Syn.: *Acarospora chlorophana* (Wahlenb.) A. Massal., *Gussonea chlorophana* (Wahlenb.) Tornab., *Parmelia chlorophana* Wahlenb., Pleopsidium flavum (Bellardi) Körb. var. chlorophanum (Wahlenb.) Körb.

L – Subs.: sil, met – Alt.: 2–6 – Note: an arctic-alpine, bipolar lichen found on vertical or underhanging surfaces of often metal-rich siliceous rocks in exposed situations with optimum above treeline. – **Au**: V, T, S, K, St, N. **Sw**: BE, GR, TI, UR, VS. **Fr**: HAl, AMa, Isè, Sav, HSav. **It**: Frl, TAA, Lomb, Piem, VA. **Sl**: SlA.

***Pleopsidium oxytonum* (Ach.) Rabenh.**

Syn.: *Acarospora flava* (Bellardi) Trevis. (1853!), *Acarospora oxytona* (Ach.) A. Massal., *Gussonea flava* (Bellardi) Anzi, *Gussonea oxytona* (Ach.) A. Massal., *Lecanora oxytona* Ach., *Lichen flavus* Bellardi (1792) *non* Schreb. (1771), *Pleopsidium flavum* (Bellardi) Körb.

L – Subs.: sil, met – Alt.: 2–6 – Note: somehow more common than *P. chlorophanum* in the Mediterranean mountains, and in areas with a continental climate, as in the Central Alpine chains. – **Au**: V, T, S, K, St, N. **Sw**: BE, GR, UR, VS. **Fr**: AHP, HAl, AMa, Isè, HSav. **It**: TAA, Lomb, Piem, VA, Lig.

***Pleurosticta acetabulum* (Neck.) Elix & Lumbsch**

Syn.: *Imbricaria acetabulum* (Neck.) DC., *Lichen acetabulum* Neck., *Melanelia acetabulum* (Neck.) Essl., *Parmelia acetabulum* (Neck.) Duby, *Parmelia corrugata* Ach. *nom.illeg.*, *Pleurosticta lichenicola* Petr.

L – Subs.: cor – Alt.: 1–4 – Note: an epiphytic species most frequent in somehow continental areas, below the subalpine belt. – **Au**: V, T, S, K, St, O, N, B. **Ge**: OB. **Sw**: BE, GL, LU, SG, SZ, UW, VD, VS. **Fr**: AHP, HAl, AMa, Drô, Isè, Sav, HSav, Var, Vau. **It**: Frl, Ven, TAA, Lomb, Piem. **Sl**: SlA, Tg. **Li**.

***Pleurosticta koflerae* (Clauzade & Poelt) Elix & Lumbsch**

Syn.: *Melanelia koflerae* (Clauzade & Poelt) Essl., *Parmelia koflerae* Clauzade & Poelt

L – Subs.: sil – Alt.: 4 – Note: a species with thalli recalling those of a sterile *P. acetabulum*, but with a different secondary chemistry (major substance: salazinic acid), older parts of thalli sometimes with isidioid warts; rather loosely attached to siliceous rocks, often with a low content in calcium, and there also overgrowing mosses and soil layers; widespread in Eurasia but rare, with two records from the Western Alps (France). – **Fr**: HAl.

***Poeltinula cacuminum* Cl. Roux**

Syn.: Encephalographa cerebrina (DC.) A. Massal. subsp. cacuminum Asta, Clauzade & Cl. Roux [invalidly published, ICN Art. 40.1 + 8], *Poeltinula cacuminum* (Asta, Clauzade & Cl. Roux) Clauzade & Cl. Roux *comb. inval*.

L – Subs.: cal – Alt.: 4–5 – Note: on hard calciferous and dolomitic rocks in exposed, but not sunny situations, with optimum above treeline; much overlooked, probably more widespread in the Alps. – **Au**: ?T, K, St. **Sw**: VS. **Fr**: AMa, Sav, HSav. **It**: Frl.

**Poeltinula cerebrina (DC.) Hafellner subsp. cerebrina**

Syn.: *Buellia cerebrina* (DC.) Th. Fr., *Encephalographa cerebrina* (DC.) A. Massal., *Lecidea cerebrina* (DC.) Schaer., *Lithographa cerebrina* (DC.) Leight., *Melanospora cerebrina* (DC.) Mudd, *Opegrapha cerebrina* DC., *Patellaria cerebrina* (DC.) Duby

L – Subs.: cal – Alt.: 3–5 – Note: on steeply inclined faces of compact calciferous rocks, especially dolomite, mostly in upland areas. – **Au**: T, S, St, O, N. **Ge**: OB, Schw. **Sw**: BE, GL, LU, UR, UW, VD. **Fr**: HAl, Isè. **It**: Ven, TAA, Lomb, Piem. **Sl**: SlA.

**Poeltinula cerebrina (DC.) Hafellner subsp. parvocalcicola Cl. Roux**

Syn.: Encephalographa cerebrina (DC.) A. Massal. subsp. parvocalcicola Asta & Cl. Roux [invalidly published, ICN Art. 40.1 + 8], Poeltinula cerebrina (DC.) Hafellner subsp. parvocalcicola (Asta & Cl. Roux) Clauzade & Cl. Roux *comb. inval*.

L – Subs.: cal, int – Alt.: 4–5 – Note: a taxon differing from the nominal subspecies in the more exposed apothecial discs and the greenish epihymenium; on calcareous schists, ecologically similar to *Stenhammarella turgida*; only known from the Alps, rarely recorded, but probably not consistently distinguished. – **Au**: ?T. **Fr**: Sav.

***Polyblastia abscondita* (Nyl.) Arnold**

Syn.: *Verrucaria abscondita* Nyl.

L # – Subs.: cal – Alt.: 3–5 – Note: on calcareous rocks in rather sheltered situations near and above treeline; closely related to *P. albida* and not always distinguished from the latter. – **Au**: V, T, S, K, O. **Ge**: Ge. **It**: TAA.

***Polyblastia absconditoides* (Servít) ined. (provisionally placed here, ICN Art. 36.1b)**

Syn.: *Amphoroblastia absconditoides* Servít

L # – Subs.: cal – Alt.: 3 – Note: a species of the *P. sepulta*-group with an endolithic thallus forming brownish patches up to 2 cm in diam., immersed ascomata (to about 0.3 mm in diam.), ovoid to oblong ascospores with 3, rarely 5 transversal septa and usually 1 incomplete longitudinal septum (to *c.* 30 µm long); on calcareous rocks, ecology otherwise poorly known; only known from the type locality in the Eastern Alps (Slovenia). – **Sl**: SlA.

***Polyblastia abstrahenda* Arnold**

Syn.: *Polyblastia intercedens auct*.

L # – Subs.: cal – Alt.: 4 – Note: a species close to or perhaps identical with *P. fuscoargillacea*, with a thin, whitish, rimose thallus and small ascomata with a basally pale wall and a descending involucrellum, broadly ellipsoid ascospores with 5–7 transversal septa and 2–3 longitudinal septa; the type is on phyllitic rock, but the species was also found on calcareous schists; rather common in the Alps at high elevations, but often confused with other species. – **Sw**: GR.

***Polyblastia albida* Arnold**

Syn.: *Amphoroblastia albida* (Arnold) Servít, *Polyblastia alpina* Metzler

L – Subs.: cal – Alt.: 2–5 – Note: on hard calciferous rocks and dolomite in sheltered situations, also within forests. – **Au**: V, T, S, K, St, O, N. **Ge**: OB. **Sw**: BE, GR, LU, SZ, TI, VS. **Fr**: AMa, Drô, Sav, HSav, Var. **It**: Frl, Ven, TAA, Piem. **Sl**: SlA.

***Polyblastia amethystina* Servít**

L – Subs.: cal – Alt.: 4 – Note: a calcicolous species resembling *P. forana*, but the granulose thallus whitish to brownish-grey with a rose tinge, ascospores larger (25–33 × 12–14 µm), mostly clavate and 3-septate (occasionally with one longitudinal septum), and ascomata with a basally dark exciple and open involucrellum; widespread in Central Europe from the lowlands to the subalpine belt, but rarely collected, with a single record from the Eastern Alps (Austria). – **Au**: St.

***Polyblastia amota* Arnold**

L # – Subs.: cal – Alt.: 3–5 – Note: on calciferous rocks in sheltered situations, mostly in upland areas; closely related to *P. albida*, from which it differs in the slightly larger spores (see Roux et al. 2014: 899); not always distinguished from *P. albida*. – **Au**: V, T, St. **Fr**: AMa, Sav, HSav, Vau. **It**: Ven, TAA.

***Polyblastia antonii* Zahlbr.**

L # – Subs.: cal – Alt.: 3 – Note: a species described as an endolithic calcicolous lichen, but the minute ascomata, said to be similar to those of *Halospora deminuta* and containing 8-spored asci with muriform brown ascospores, could be those of a lichenicolous fungus (*Merismatium*?); only known from the *locus classicus* in the Eastern Alps (Austria). – **Au**: N.

***Polyblastia ardesiaca* (Bagl. & Carestia) Zschacke**

Syn.: *Polyblastia sprucei* (Anzi) Arnold, *Sagedia sprucei* Anzi, *Thelidium ardesiacum* Bagl. & Carestia

L – Subs.: cal, cal-aqu, sil-aqu, int-aqu – Alt.: 3–5 – Note: a calcicolous species found on periodically submerged rocks in mountain creeks, usually near or above treeline. – **Au**: T, S, K, N. **Sw**: BE, TI. **Fr**: HAl, AMa, Sav. **It**: Frl, Ven, TAA, Lomb, Piem.

***Polyblastia aurantia* Breuss**

L # – Subs.: cal – Alt.: 3 – Note: a species resembling *P. sepulta* in the endolithic thallus and the immersed perithecia lacking an involucrellum, but the outer part of the ascomatal wall with an orange-red pigment, and ascospores with more septa; on calcareous rocks; only known from the locus classicus at low elevation in the Eastern Alps (Austria). – **Au**: N.

***Polyblastia bormiensis* Servít**

L # – Subs.: sil – Alt.: ?3–5 – Note: a silicicolous species resembling *P. fuscoargillacea*, but with a different ecology and with a thin, epilithic, ochraceous, rimose to areolate thallus forming large patches, the areolae superficially granulose to minutely verrucose, hemispherically protruding perithecia (to 0.25 mm in diam.) with an adpressed involucrellum reaching down about 1/3 of the perithecium, and subglobose to broadly obovoid ascospores (to *c.* 25 µm long) with up to 6 transversal septa and up to 2 incomplete longitudinal septa; only known from the type locality in the Eastern Alps (Italy). – **It**: Lomb.

***Polyblastia bryophila* Lönnr.**

L – Subs.: deb, cal – Alt.: 5 – Note: a species resembling *P. sendtneri*, but with a subsquamulose thallus on a black prothallus, only slightly immersed ascomata developing mainly along the edges of the areolae, larger and more muriform ascospores with 5–7 transversal septa and 3–5 longitudinal septa; overgrowing mosses and plant debris on calcareous soil; overall distribution Holarctic, most common in Northern Europe; in the Alps rare, but perhaps not recognised or overlooked in some countries. – **Au**: N. **Sw**: SZ.

***Polyblastia buerensis* Zschacke**

L # – Subs.: cal – Alt.: 3 – Note: a species with a thin thallus and ascomata immersed in pits in the rock, 8-spored asci, and narrowly ellipsoid, submuriform ascospores resembling those of *P. dermatodes* but less regularly divided; on clayey limestone; rare in Central Europe at low elevations; in the Alps known from a few scattered records. – **Au**: St, O. **Fr**: AHP.

***Polyblastia cinerea* (A. Massal.) Jatta**

Syn.: *Amphoridium cinereum* A. Massal., *Verrucaria dictyospora* Stizenb.

L # – Subs.: cal – Alt.: 2–5 – Note: on sheltered calcareous rocks near or above treeline; a critical taxon, which deserves further study. – **Au**: ?T, ?S, O, N. **Sw**: GR. **It**: Ven, Lomb.

***Polyblastia clandestina* (Arnold) Jatta**

Syn.: *Sporodictyon clandestinum* Arnold

L – Subs.: cal – Alt.: 3–5 – Note: on calcareous rocks in sheltered situations; apparently restricted to the Alps, where it is probably more widespread. – **Au**: T, O. **Fr**: HSav. **It**: Ven, TAA. **Sl**: SlA.

**Polyblastia cupularis A. Massal. var. cupularis**

Syn.: *Polyblastia flavicans* Müll. Arg., *Polyblastia intercedens* (Nyl.) Lönnr. *non* *sensu* Th. Fr., *Polyblastia lutosa* Zschacke, *Polyblastia pallescens* Anzi

L – Subs.: cal, int – Alt.: 3–5 – Note: a circumboreal to arctic-alpine species found on hard rocks, including dolomite and calciferous schist, in rather sheltered and humid situations. – **Au**: V, T, S, K, St, O, N. **Ge**: OB. **Sw**: BE, GR, LU, SZ, TI, UR, UW, VD, VS. **Fr**: AHP, HAl, AMa, Drô, Isè, Sav, HSav, Vau. **It**: Frl, Ven, TAA, Lomb, Piem. **Sl**: SlA.

**Polyblastia cupularis A. Massal. var. crepaturae (Zschacke) Zschacke**

L # – Subs.: cal, ?int – Alt.: 4–5 – Note: a critical taxon distinguished from the typical variety by the presence of gloeohyphae in and below the algal layer; on calcareous rocks, ecology otherwise poorly known, with scattered records from Eastern and Central Europe; rare in the Alps but probably not always distinguished. – **Au**: ?V. **Fr**: Sav.

***Polyblastia deplanata* Arnold**

L # – Subs.: cal – Alt.: 4–5 – Note: a calcicolous species, closely related to *P. ventosa*, and rarely collected. – **Au**: ?V, T, K. **Sw**: GR. **Fr**: Sav. **It**: TAA.

***Polyblastia dermatodes* A. Massal.**

Syn.: *Amphoroblastia dermatodes* (A. Massal.) Servít, *Amphoroblastia tyrolensis* (Arnold) Servít, *Polyblastia schraderi* (Gray) A.L. Sm.; incl. *Polyblastia bavarica* Zschacke

L – Subs.: cal – Alt.: 2–5 – Note: on shaded, inclined surfaces of calciferous rocks (limestone, dolomite), with optimum near and above treeline, sometimes with *Eiglera homalomorpha*. – **Au**: V, T, S, K, St, O, N. **Ge**: OB, Schw. **Sw**: BE, GR, UW, VS. **Fr**: Isè, Sav. **It**: Ven, TAA. **Sl**: Tg.

***Polyblastia dominans* (Arnold) Zahlbr.**

Syn.: *Thelidium dominans* Arnold

L – Subs.: cal – Alt.: 3–5 – Note: a species resembling *P. sepulta* (with which it is sometimes merged), with a thin to entirely endolithic thallus and ascomata immersed in pits, lacking an involucrellum, but the narrow ellipsoid ascospores larger, with 3 transversal septa and occasionally an incomplete longitudinal septum; on calcareous rocks; widespread in Europe and most frequent in the Central European mountains; from the Alps there are only a few scattered records. – **Au**: T, St. **Sl**: Tg.

***Polyblastia epigaea* A. Massal.**

L # – Subs.: deb, ter-cal – Alt.: 4–5 – Note: a terricolous taxon in need of critical re-evaluation because the ascomata are rather small for a *Polyblastia* and the muriform ascospores are given as brown in the protologue and hyaline in consecutive treatments. If the statements in the protologue (“*thallus verrucoso-squamulosus sordide fuscus*, … *sporidia*…*fusca*”) are correct, the species could belong to *Agonimia*, or the description could refer to a lichenicolous fungus; based on a type from Switzerland, other records from the Alps are dubious. – **Au**: ?S. **Ge**: ?OB, ?Schw. **Sw**: Sw.

***Polyblastia epomphala* (Nyl.) Zschacke**

Syn.: *Thelidium epomphalum* (Nyl.) Zahlbr., *Verrucaria epomphala* Nyl.

L – Subs.: sil – Alt.: 4 – Note: a species with a thin, whitish thallus and ascomata immersed in thalline warts, with an apical involucrellum and a pale ascomatal wall, ascospores submuriform, resembling those of *P. sepulta*; based on a type from Romania on temporarily submerged calcareous stones; the only record from the Alps (France), however, is on siliceous rocks. – **Fr**: Isè.

***Polyblastia evanescens* Arnold**

L – Subs.: bry, cal, bry-cal – Alt.: 3–5 – Note: on bryophytes (*Racomitrium*, *Distichum*, *Encalypta*), with optimum above treeline; very rarely collected, but perhaps more widespread in the Alps. – **Au**: T, St, O. **Ge**: Ge. **Sw**: BE. **Fr**: AHP, HAl, AMa, HSav, Vau. **It**: TAA.

***Polyblastia forana* (Anzi) Arnold**

Syn.: *Thelotrema foranum* Anzi, *Verrucaria forana* (Anzi) Nyl., *Verrucaria pallidelutea* Garov.

L # – Subs.: cal, sil – Alt.: 3–5 – Note: this rarely collected lichen of hard limestone and dolomite is worthy of further study. – **Ge**: Schw. **Sw**: BE, GR, UR, VD. **It**: TAA, Lomb.

***Polyblastia fuscoargillacea* Anzi**

Syn.: incl. Polyblastia fuscoargillacea Anzi var. cinerea Müll. Arg.

L – Subs.: cal, sil, int – Alt.: 3–6 – Note: an early coloniser of calciferous rocks, including small pebbles on the ground, with optimum near or above treeline; probably widespread throughout the Alps. – **Au**: V, T, S, K, St, N. **Ge**: OB. **Sw**: BE, GR, UR, VD, VS. **Fr**: AHP, HAl, AMa, Sav, HSav, Vau. **It**: TAA, Lomb, Piem.

***Polyblastia gneissiaca* Müll. Arg.**

L # – Subs.: sil – Alt.: 3–4 – Note: a silicicolous species with a thin, whitish thallus, semi-immersed ascomata with a pigmented wall and a descending involucrellum, and hyaline, ovoid ascospores with 6–7 transversal septa and 3–4 longitudinal septa; only known from the type locality in the Western Alps (Switzerland). – **Sw**: VS.

***Polyblastia helvetica* Th. Fr.**

Syn.: *Amphoroblastia helvetica* (Th. Fr.) Servít

L – Subs.: ter-cal, bry-cal – Alt.: 3–5 – Note: on more or less calciferous soil, often amongst bryophytes, with optimum near treeline. – **Au**: V, T. **Sw**: BE, GR, VD. **It**: Frl, Ven, Lomb.

***Polyblastia intermedia* Th. Fr.**

Syn.: ?*Polyblastia kernstockii* Zschacke

L – Subs.: cal – Alt.: 4–5 – Note: an arctic-alpine species of calciferous rocks with optimum above treeline; the Italian record refers to *P. kernstockii*. – **Au**: ?V. **It**: TAA.

***Polyblastia latebrosa* (Bagl. & Carestia) Jatta**

Syn.: *Microglaena latebrosa* (Bagl. & Carestia) Jatta, *Weitenwebera latebrosa* Bagl. & Carestia

L # – Subs.: sil – Alt.: 4–5 – Note: only known from the type collection, on granite, this species is likely to be closely related to, or a synonym of *Protothelenella sphinctrinoides*, but was forgotten because of the unavailability of the type, which is most probably in MOD (see Nimis 1993: 559). – **It**: Piem.

***Polyblastia leptospora* Zschacke**

L – Subs.: cal – Alt.: 3 – Note: a species with a thin, greenish-white thallus with gloeohyphae, ascomata immersed in pits, with a pigmented wall lacking an involucrellum, and narrowly ellipsoid ascospores with 5–7 transversal septa and 1 incomplete longitudinal septum; on calcareous rocks in Eastern and Central Europe, with a few records from the Eastern Alps (Austria). – **Au**: V, S.

***Polyblastia likana* Servít**

L # – Subs.: cal – Alt.: 3 – Note: a species related to *P. ardesiaca*, with a thin whitish thallus and semi-immersed perithecia with a pigmented wall and an apical involucrellum, but the ovoid ascospores smaller, usually with 3 transversal septa and occasionally a single, incomplete longitudinal septum; on limestone in Central Europe and in the mountains of the Mediterranean region; apparently rare in the Alps, being only known from Austria. – **Au**: N.

***Polyblastia maculata* Zschacke**

L – Subs.: cal – Alt.: 5 – Note: a species resembling *P. clandestina* in the endolithic thallus and the ascomata immersed in pits, but with larger, narrow ascospores with 5–7 transversal septa and a single, incomplete longitudinal septum; on limestone in Eastern and Central Europe, with a single record from the Eastern Alps (Austria). – **Au**: V.

***Polyblastia microcarpa* (Arnold) Lettau**

Syn.: Polyblastia cupularis A. Massal. f. microcarpa Arnold, Polyblastia cupularis A. Massal. var. microcarpa (Arnold) ined.

L # – Subs.: cal, int – Alt.: 3–5 – Note: this species differs from *P. cupularis* only in the smaller perithecia, but no valid name is available at the varietal rank, which would perhaps be more appropriate; on base-rich or calcareous rocks in sheltered situations, with optimum near or above treeline. – **Au**: V, T, S, K, St, O, N. **Ge**: OB. **Sw**: BE, GR. **Fr**: AHP, Sav. **It**: Ven, TAA.

***Polyblastia moravica* Zschacke**

L – Subs.: sil, sil-aqu – Alt.: 4–5 – Note: a species with a thin thallus, semi-immersed ascomata with a pigmented wall and a thick involucrellum in the upper half, and ellipsoid ascospores with 3 transversal septa and a single, incomplete longitudinal septum; on long-time moist schists in Central Europe, with a few records from the Eastern Alps (Austria). – **Au**: T, St.

***Polyblastia murorum* B. de Lesd.**

Syn.: incl. Polyblastia murorum B. de Lesd. var. denudata B. de Lesd.

L # – Subs.: cal – Alt.: 2 – Note: a species with a whitish-grey, rimose to areolate thallus with very small areolae, entirely immersed perithecia leaving pits after they have fallen out, and broadly ellipsoid, muriform ascospores; on calcareous walls at low elevations in the northern Mediterranean region, with a single record from a wall near Nice (France). – **Fr**: AMa.

***Polyblastia nidulans* (Stenh.) Arnold**

Syn.: *Verrucaria nidulans* Stenh.

L – Subs.: cal – Alt.: 3–6 – Note: on compact limestone and dolomite in sheltered situations, with optimum above treeline. – **Au**: ?V, T, S, St. **Fr**: AHP, AMa, Drô, Isè, Sav, HSav, Vau.

***Polyblastia pachydermis* Servít**

L # – Subs.: cal – Alt.: 3 – Note: a species with a whitish endolithic thallus, globose perithecia immersed in pits, and narrowly ovoid, muriform ascospores with 4–7 transversal septa and 3–5 longitudinal septa; on calcareous rocks in Central Europe; so far only known from the Eastern Alps (Slovenia). – **Sl**: SlA.

***Polyblastia peminosa* (Nyl.) Zahlbr.**

Syn.: *Verrucaria peminosa* Nyl.

L – Subs.: sil – Alt.: 4 – Note: a species with a reddish-grey, rimose to areolate thallus, immersed perithecia with a descending involucrellum, and ascospores with highly variable septation (partly simple, partly with 2–5 transversal septa, and partly with an incomplete longitudinal septum); on periodically inundated siliceous rocks in the European mountains, with a single record from the Eastern Alps (Austria). – **Au**: T.

***Polyblastia philaea* Zschacke**

Syn.: *Amphoroblastia philaea* (Zschacke) Servít

L # – Subs.: ter – Alt.: 4–5 – Note: a very poorly understood species found on soil, both on bare ground and amongst bryophytes. – **It**: TAA.

***Polyblastia plicata* (A. Massal.) Lönnr.**

Syn.: *Verrucaria plicata* A. Massal.; incl. *Polyblastia singularis* (Kremp.) Arnold

L – Subs.: cal – Alt.: 3–5 – Note: on compact limestone and dolomite in shaded and humid situations, mostly in upland areas. – **Au**: V, T, S, K, St, O. **Sw**: GR, SZ, UR. **Fr**: AHP, HAl, Sav. **It**: Ven, TAA, Piem.

***Polyblastia quartzina* Lynge**

L – Subs.: sil – Alt.: 2–4 – Note: a species with an epilithic, rimose, brownish-green thallus, rather small, semi-immersed ascomata with a diverging involucrellum in the lower part, and small, broadly ellipsoid ascospores with 1–3 transversal septa and a single longitudinal septum, based on a type from Novaya Zemlya on siliceous rocks (quartzite); widespread in Eurasia but apparently rare; in the so far only known from the Western Alps (France). – **Fr**: AMa.

***Polyblastia quinqueseptata* (Arnold) Zschacke**

Syn.: *Thelotrema quinqueseptatum* Arnold

L # – Subs.: cal, ter-cal, deb – Alt.: 4–5 – Note: on shaded surfaces of calcareous rocks in the mountains; closely related to *P. sepulta*. – **Au**: St. **It**: Ven, TAA, Piem.

***Polyblastia rivalis* (Arnold) Zschacke**

Syn.: *Thelidium rivale* Arnold

L – Subs.: cal-aqu, int-aqu, sil-aqu – Alt.: 3–4 – Note: a species with a whitish-grey, partly endolithic thallus, ascomata semi-immersed in pits protruding with the upper third, a dark exciple and a descending involucrellum, and submuriform, ellipsoid, relatively large ascospores (to 75 µm long) with 3–6 transversal septa and (partly) a single, incomplete longitudinal septum; on periodically inundated limestone or calcareous schists; only known from the Eastern Alps (Austria) but likely to occur elsewhere in the Alps. – **Au**: T, S.

***Polyblastia rouxiana* Vězda & Vivant**

L – Subs.: ter – Alt.: 2 – Note: on bare soil in clearings of garrigue and maquis vegetation over calcareous substrata, mainly in the Mediterranean belt, with a few records from the Western and Southern Alps. – **Fr**: AMa, Var. **It**: Ven.

***Polyblastia sendtneri* Kremp.**

Syn.: *Thelotrema sendtneri* (Kremp.) Anzi

L – Subs.: deb, ter-cal, bry-cal – Alt.: 4–6 – Note: a circumpolar, arctic-alpine species found on organic soil, mosses and plant debris with optimum above treeline; widespread throughout the Alps. – **Au**: V, T, S, K, St, O, N. **Ge**: OB, Schw. **Sw**: AP, BE, GR, LU, SG, SZ, TI, UW, VD, VS. **Fr**: AHP, HAl, AMa, Sav, Vau. **It**: Frl, Ven, TAA, Lomb, Piem, VA. **Sl**: SlA. **Li**.

***Polyblastia sepulta* A. Massal.**

Syn.: *Amphoroblastia calcivora* (Nyl.) Servít, *Amphoroblastia pertusula* (Nyl.) Servít, *Amphoroblastia sepulta* (A. Massal.) Servít, *Polyblastia calcivora* (Nyl.) Croz., *Polyblastia pertusula* (Nyl.) Zschacke, *Thelidium calcivorum* (Nyl.) Hulting, *Thelidium epipolaeum* Arnold *non* A. Massal., *Verrucaria calcivora* Nyl., *Verrucaria pertusula* Nyl., *Verrucaria sepulta* (A. Massal.) Wedd.; incl. *Amphoroblastia bavarica* (Dalla Torre & Sarnth.) Servìt, *Polyblastia bavarica* (Dalla Torre & Sarnh.) Zschacke, *Thelidium bavaricum* Dalla Torre & Sarnth.

L – Subs.: cal – Alt.: 2–5 – Note: a species resembling *P. dominans*, but with smaller, narrowly ellipsoid ascospores with usually 5 transversal septa and occasionally a single incomplete longitudinal septum; on hard calciferous rocks in shaded and humid situations, often on pebbles, most frequent above treeline; widespread throughout the Alps. The whole complex – see synonyms – is in need of revision. – **Au**: T, S, K, St, O, N. **Ge**: OB. **Sw**: BE, GR, LU, SZ, TI, UR, UW, VD. **Fr**: AMa, Drô, Isè, Sav, HSav, Var, Vau. **It**: Frl, Ven, TAA, VA. **Sl**: SlA, Tg.

***Polyblastia subglacialis* Riedl**

L # – Subs.: int – Alt.: 5 – Note: a species with an indistinct thallus, hemispherical, protruding perithecia with a basally hyaline wall and a thick involucrellum reaching the substrate, and ellipsoid, muriform ascospores up to *c.* 50 µm long; on siliceous boulders of moraines in the glacier foreland of the high-alpine to subnival belt; only known from the type locality in the Eastern Alps (Austria). – **Au**: K.

***Polyblastia subocellata* Th. Fr.**

L # – Subs.: bry, ter-sil – Alt.: 5 – Note: a species of unclear relationship, perhaps belonging to *Sporodictyon*, with a whitish granulose thallus, and ellipsoid, muriform (to *c.* 60 µm long) ascospores which are first hyaline, then brownish; on cushions of mosses over siliceous substrata; rare, from the boreal-alpine to the temperate-alpine zone, with a few records from the Eastern Alps (Austria). – **Au**: S.

***Polyblastia tatrana* Servít**

L – Subs.: ter-cal, cal, deb – Alt.: 4–5 – Note: a species resembling *P. sendtneri*, but with a basally closed involucrellum; on soil and plant debris over calcareous rocks in the European mountains; distribution insufficiently documented because the species was often not distinguished, with a few records from the Eastern Alps (Austria), but probably still overlooked elsewhere. – **Au**: V, S, K, St, O.

***Polyblastia triglavensis* Servít**

L # – Subs.: cal – Alt.: 3 – Note: a calcicolous species with a whitish endolithic thallus, globose perithecia almost entirely immersed in pits, the ostiolar region surrounded by a weakly developed thalline annulus, and ellipsoid ascospores not exceeding 30 µm in length, with usually 3 transversal septa and a single, incomplete longitudinal septum; in the study area so far only known from the Eastern Alps (Slovenia). – **Sl**: SlA.

***Polyblastia ventosa* Arnold *nom.illeg. non* A. Massal.**

L – Subs.: cal – Alt.: 4–5 – Note: on limestone and dolomite in rather exposed situations, with optimum above treeline. – **Au**: ?V, T, S, K, St, O. **Fr**: AHP, HAl, AMa, Isè, Sav, HSav. **It**: Frl, Ven, TAA, Lomb, Piem.

***Polyblastia verrucosa* (Ach.) Lönnr.**

Syn.: *Pyrenula verrucosa* Ach.

L – Subs.: cal – Alt.: 3–6 – Note: on steeply inclined, sheltered surfaces of calcareous rocks, with optimum above treeline; widespread throughout the Alps. – **Au**: V, T, S, K, St, O. **Ge**: Schw. **Sw**: BE, GR, LU, SG, SZ, UW, VD, VS. **Fr**: AHP, Sav, HSav. **It**: Frl, Ven, TAA, Piem, VA.

***Polyblastidium subneglectum* (Elix) Kalb**

Syn.: *Heterodermia japonicaauct. eur.*, *Heterodermia obscurata auct. eur. p. max. p.*, *Heterodermia subneglecta* Elix

L – Subs.: cor – Alt.: 2–3 – Note: according to Roux et al. (2014) most samples of *Heterodermia obscurata* from Europe do actually belong to this species, which differs in chemical characters and in having a white, yellow-orange-spotted lower surface (whereas the lower surface of *H. obscurata* is yellow-orange throughout). – **It**: Frl.

***Polycauliona candelaria* (L.) Frödén, Arup & Søchting**

Syn.: *Lecanora candelaria* (L.) Ach., *Lichen candelarius* L., *Massjukiella candelaria* (L.) S.Y. Kondr., Fedorenko, S. Stenroos, Kärnefelt, Elix, J.-S. Hur & A. Thell, *Physcia controversa* A. Massal., *Physcia lychnea* (Ach.) Nyl., *Xanthoria candelaria* (L.) Th. Fr., *Xanthoria controversa* (A. Massal.) Rabenh., *Xanthoria lychnea* (Ach.) Th. Fr.

L – Subs.: cor, xyl, sil – Alt.: 2–5 – Note: both on bark and on rock, sometimes on nutrient-enriched lignum, with optimum in upland areas with a subcontinental climate (*e.g.* continental valleys of the Alps). – **Au**: V, T, S, K, St, O, N. **Ge**: OB, Schw. **Sw**: BE, FR, GL, GR, LU, SG, SZ, TI, UR, UW, VD, VS. **Fr**: AHP, HAl, AMa, Drô, Isè, Sav, HSav, Var, Vau. **It**: Frl, Ven, TAA, Lomb, Piem, VA, Lig. **Sl**: SlA.

***Polycauliona phlogina* (Ach.) Arup, Frödén & Søchting**

Syn.: Caloplaca citrina (Hoffm.) Th. Fr. f. phlogina (Ach.) D. Hawksw., Caloplaca citrina (Hoffm.) Th. Fr. var. phlogina (Ach.) H. Olivier, *Caloplaca phlogina* (Ach.) Flagey, Parmelia citrina (Hoffm.) Ach. var. phlogina Ach., *Placodium phloginum* (Ach.) A.L. Sm.

L – Subs.: cor, xyl – Alt.: 1–3 – Note: for a long time regarded as the mainly corticolous ecotype of *Caloplaca citrina*, but evidently different, and according to recent reconstructions of the Teloschistales phylogeny, a species of the “*Xanthoria polycarpa*-*candelaria*-group”; most epiphytic records of supposed *Caloplaca citrina* might belong here; rare, in Central Europe most records are historical. – **Sw**: FR, SZ, VD, VS. **Fr**: AHP, AMa, Sav, Vau. **It**: Frl, Ven, TAA, Lomb, Piem.

***Polycauliona polycarpa* (Hoffm.) Frödén, Arup & Søchting**

Syn.: *Lobaria polycarpa* Hoffm., *Massjukiella polycarpa* (Hoffm.) S.Y. Kondr., Fedorenko, S. Stenroos, Kärnefelt, Elix, J.-S. Hur & A. Thell, *Xanthoria polycarpa* (Hoffm.) Th. Fr. *ex* Rieber

L – Subs.: cor, xyl – Alt.: 2–4 – Note: a mainly boreal-montane, circumpolar species found on isolated trees and sun-exposed branches and small twigs, on wooden poles and fences; widespread throughout the Alps. – **Au**: V, T, S, K, St, O, N. **Ge**: OB, Schw. **Sw**: BE, GL, GR, LU, SG, SZ, TI, UR, VD, VS. **Fr**: HAl, AMa, Drô, Isè, Sav, HSav. **It**: Frl, Ven, TAA, Lomb, Piem, VA, Lig. **Sl**: SlA, Tg. **Li**.

***Polycauliona ucrainica* (S.Y. Kondr.) Frödén, Arup & Søchting**

Syn.: *Massjukiella ucrainica* (S.Y. Kondr.) S.Y. Kondr., Fedorenko, S. Stenroos, Kärnefelt, Elix, Hur & A. Thell, *Xanthoria ucrainica* S.Y. Kondr.

L # – Subs.: cor – Alt.: 3–4 – Note: a species resembling *P. candelaria* (and perhaps just an extreme morphotype of the latter), but lobes distinctly foliose, becoming wider towards the margin, divided into microlobuli forming blastidia (40–60 µm in diam.), and pycnidia containing ellipsoid rather than bacilliform pycnoconidia; usually on bark of deciduous trees; widespread in Europe and Asia but rather rare, perhaps not always distinguished from other *Polycauliona* species; for the Alps there are only a few scattered records from Austria and Switzerland. – **Au**: K. **Sw**: GR.

***Polychidium muscicola* (Sw.) Gray**

Syn.: *Collema muscicola* (Sw.) Ach., *Homodium muscicola* (Sw.) Nyl., *Leptogium muscicola* (Sw.) Fr., *Lichen muscicola* Sw., *Polychidium kalkuense* Räsänen

L – Subs.: bry, sil, bry-sil – Alt.: 1–4 – Note: a widespread mild-temperate to southern boreal lichen found on soil and amongst bryophytes over siliceous substrata, more rarely on the basal parts of ancient trees, with a rather wide altitudinal range. – **Au**: V, T, S, St, N. **Sw**: GR, SZ, TI, VD, VS. **Fr**: AMa, HSav, Var, Vau. **It**: Frl, TAA, Lomb, Piem, Lig. **Sl**: SlA.

***Polysporina cyclocarpa* (Anzi) Vězda**

Syn.: *Acarospora cyclocarpa* (Anzi) Jatta, *Biatorella cyclocarpa* (Anzi) Lindau, *Lithographa cyclocarpa* Anzi, *Sarcogyne cyclocarpa* (Anzi) J. Steiner

L – Subs.: cal, int – Alt.: 3–5 – Note: on dolomite and calcareous schists, but especially on pure limestone, with optimum above treeline; closely related to *P. urceolata*. – **Au**: V, T, S, K, St, O, N. **Ge**: OB, Schw. **Sw**: BE, GR, SZ, VS. **Fr**: AHP, HAl, AMa, Sav, HSav. **It**: Ven, TAA, Lomb, Piem, Lig.

***Polysporina ferruginea* (Lettau) M. Steiner *ex* Kantvilas**

Syn.: Polysporina simplex (Taylor) Vězda f. ferruginea (Lettau) Clauzade & Cl. Roux, Sarcogyne simplex (Taylor) Nyl. f. ferruginea Lettau

L # – Subs.: sil, met – Alt.: 4–5 – Note: on basic or slightly calciferous siliceous rocks, probably lichenicolous on an as yet unidentified host, later forming an autonomous thallus. Perhaps a synonym of *P. subfuscescens*, but a well-distinct species according to Roux. – **Au**: V, T, S, K, St. **Fr**: AHP, HAl, AMa. **It**: Frl.

***Polysporina limborinella* (Müll. Arg.) Hafellner**

Syn.: *Biatorella limborinella* (Müll. Arg.) H. Olivier, *Lecidea limborinella* Müll. Arg.

L # – Subs.: sil – Alt.: 6 – Note: a species resembling in habitus to *P. urceolata*, with a strongly reduced (endolithic) thallus and rough. brown-black apothecia (to 0.3 mm in diam) provided with a thick margin with some radial cracks which widely obtects the punctiform, urceolate disc, and asci containing very numerous, minute ascospores (*c.* 2 μm long); on\ly known from the type locality in the Western Alps (Switzerland), on siliceous schists. – **Sw**: VS.

***Polysporina pusilla* (Anzi) Nimis**

Syn.: *Biatorella pusilla* (Anzi) Zahlbr. *ex* Beck, *Sarcogyne pusilla* Anzi

L – Subs.: cal-par, int-par – Alt.: 3–6 – Note: a widespread lichenicolous lichen, most frequent on calcareous rocks in sunny habitats, growing in the apothecia of *Protoblastenia*-species, mainly *P. incrustans*. – **Au**: V, T, S, K, St, O, N. **Ge**: OB. **Sw**: BE, GR, VS. **Fr**: AHP, AMa. **It**: Ven, TAA, Lomb, Piem.

***Polysporina simplex* (Taylor) Vězda**

Syn.: *Acarospora simplex* (Taylor) Jatta., *Biatorella simplex* (Taylor) Branth & Rostr., *Lecidea privigna* Ach., *Lecidea simplex* Taylor, *Lichen simplex* Davies *nom.illeg.*, *Sarcogyne privigna* (Ach.) A. Massal. *nonauct.*, *Sarcogyne simplex* (Taylor) Nyl., ?Sarcogyne simplex (Taylor) Nyl. var. minor B. de Lesd.; incl. *Biatorella hymenogonia* Zahlbr., Polysporina simplex (Taylor) Vězda var. hymenogonia (Zahlbr.) N.S. Golubk.

L – Subs.: sil – Alt.: 1–6 – Note: a holarctic early coloniser of small cracks of siliceous, sometimes base-rich or slightly calciferous rocks. – **Au**: V, T, S, K, St, O, N, B. **Sw**: BE, GR, LU, SZ, TI, UR, VD, VS. **Fr**: AHP, HAl, AMa, Sav, HSav, Var. **It**: Frl, Ven, TAA, Lomb, Piem, VA, Lig. **Sl**: SlA.

***Polysporina subfuscescens* (Nyl.) K. Knudsen & Kocourk.**

Syn.: *Acarospora silesiaca* (H. Magn.) H. Magn., *Acarospora subfuscescens* (Nyl.) H. Magn., *Biatorella subfuscescens* (Nyl.) H. Olivier, *Lecanora subfuscescens* Nyl., *Polysporina dubia* (H. Magn.) Vězda, *Polysporina lapponica auct.*, *Sarcogyne dubia* H. Magn.

L # – Subs.: sil-par, met-par – Alt.: 2–4 – Note: a widespread, perhaps non-lichenised species, often growing on *Acarospora*. The species is heterogeneous and in need of revision. – **Au**: ?V, T, K. **Ge**: Ge. **Sw**: VS. **Fr**: AMa, Var. **It**: TAA, VA, Lig.

***Polysporina urceolata* (Anzi) Brodo**

Syn.: *Acarospora urceolata* (Anzi) Jatta, *Biatorella urceolata* (Anzi) J. Steiner, *Sarcogyne urceolata* Anzi, Sarcogyne urceolata Anzi var. herpes Norman

L – Subs.: cal – Alt.: 3–5 – Note: a calcicolous species with an endolithic thallus, black apothecia immersed in pits, with prominent, rough margins, differing from the similar *P. pusilla* in having a lower hymenium (only *c.* 60 µm high); widespread in the Holarctic region, in Europe from the Arctic to the Mediterranean-Alpine zones, with several scattered records throughout the Alps, mainly in the alpine belt, but probably more widespread. – **Au**: ?V, T, S, K, St, N. **Ge**: OB. **Sw**: GR, LU, SZ, VS. **Fr**: AHP, HAl, AMa, Sav. **It**: Lomb.

***Porina ahlesiana* (Körb.) Zahlbr.**

Syn.: *Porina globosa* (Taylor) A.L. Sm., *Porina insiliens* (Larbal.) A.L. Sm., *Porina septemseptata* (Hepp *ex* Zwackh) Swinscow, *Segestrella ahlesiana* Körb.

L – Subs.: sil – Alt.: 2–5 – Note: a rare species of shaded-humid siliceous rocks, with a somehow western distribution in Europe and a wide altitudinal range; from the Alps there are only a few scattered records. – **Au**: N. **Fr**: Var. **It**: Piem.

***Porina alpina* (Bagl. & Carestia) Lettau**

Syn.: *Sagedia alpina* (Bagl. & Carestia) Jatta, *Segestrella alpina* Bagl. & Carestia, *Verrucaria alpina* (Bagl. & Car.) Stizenb.

L # – Subs.: sil – Alt.: 4–5 – Note: a long-forgotten species with a subgelatinous-subleprose thallus, small, brownish-black perithecia, the wall black only in the upper half, subcylindrical, 6–8-spored asci, sparse, filiform paraphyses, and fusiform, acute, 3–7-septate, hyaline ascospores measuring 29–37 × 7–9 µm; only known from the type collection, on soil in the alpine belt; the type material (probably in MOD) is worthy of further study. – **It**: Piem.

***Porina arnoldii* Poelt & Vězda *ex* Hafellner & Türk**

L – Subs.: cor – Alt.: 4 – Note: a species with a red-brown involucrellum and narrowly fusiform, 3-septate ascospores; corticolous on branches of *Rhododendron*, apparently rare in the Alps, being only known from Austria, but perhaps overlooked elsewhere. – **Au**: T, K, St.

***Porina calciseda* (Bagl. & Carestia) Lettau**

Syn.: *Sagedia calciseda* Bagl. & Carestia, *Verrucaria calciseda* (Bagl. & Carestia) Stizenb. *non* DC.

L # – Subs.: cal – Alt.: 3 – Note: a long-forgotten species with a rather thick, chalky, dirty white, continuous to rimose thallus, immersed, black perithecia with a flattened ostiolar region, sparse filiform paraphyses, lanceolate 8-spored asci, and elongate-fusiform, straight to slightly curved, 3–5-septate, hyaline ascospores measuring 29–36 × c. 4 µm; only known from the type collection, on limestone, which would be worthy of further study. – **It**: Piem.

***Porina constricta* (Anzi) Lettau**

Syn.: *Sagedia constricta* Anzi

L # – Subs.: cor – Alt.: 3–4 – Note: this taxon, which is known only from the type collection on *Betula*, could be related to *Arthopyrenia grisea* (see Nimis 1993: 565); it has an effuse, thin, episubstratic, continuous, olive-brown thallus, and minute, numerous, black, hemispherical and often confluent perithecia, 6 – to 8-spored, ventricose asci, and fusiform, 3-septate, hyaline ascospores (28–30 × c. 7 μm). – **It**: Lomb.

***Porina hoehneliana* (Jaap) R. Sant.**

Syn.: *Calonectria hoehneliana* Jaap

L – Subs.: fol – Alt.: 2–3 – Note: a humid subtropical to Mediterranean-Atlantic lichen found on smooth bark and leaves of evergreen plants (*e.g.* on leaves of *Buxus* and cladodes of *Ruscus*) in warm-humid woodlands near the coast, with a few records from the base of the Western Alps (France). – **Fr**: AMa, Isè, HSav, Var.

***Porina lectissima* (Fr.) Zahlbr.**

Syn.: ?*Sagedia caliginosa* Anzi, *Sagedia umbonata* (Garov.) Jatta, *Segestrella lectissima* (Fr.) Anzi, *Segestria erysibodes* (Taylor) Trevis., *Segestria lectissima* Fr., *Segestria thelostoma* (Flot.) A. Massal., *Verrucaria erysiboda* Taylor, *Verrucaria irrigua* Taylor, *Verrucaria rubiginosa* Taylor, *Verrucaria umbonata* Garov.

L – Subs.: sil, sil-aqu – Alt.: 1–4 – Note: a mild-temperate to Mediterranean-Atlantic species found on steeply inclined surfaces of siliceous rocks in shaded-moist situations, often in forests, or on periodically submerged rocks near creeks and lakes. – **Au**: ?V, T, S, K, St. **Sw**: BE, TI, UR. **Fr**: AMa, Var. **It**: Frl, TAA, Lomb, Piem, Lig. **Sl**: SlA.

***Porina leptalea* (Durieu & Mont.) A.L. Sm.**

Syn.: *Bacidia micrococcoides* Erichsen, *Biatora leptalea* Durieu & Mont., *Segestria leptalea* (Durieu & Mont.) R.C. Harris

L – Subs.: cor – Alt.: 2–3 – Note: a humid subtropical to Mediterranean-Atlantic lichen found on smooth bark of broad-leaved trees in moist forests, sometimes foliicolous on evergreen trees and shrubs (*e.g.* on *Buxus*). – **Au**: V, T, S, St, O, N. **Sw**: GL, GR, SZ, UW. **Fr**: AMa, HSav, Var. **Sl**: SlA.

***Porina leptosperma* Müll. Arg.**

Syn.: *Phylloporina leptosperma* (Müll. Arg.) Müll. Arg.

L – Subs.: fol – Alt.: 2–3 – Note: an obligately foliicolous lichen with tropical-subtropical affinities; in the study area so far known only from the base of the Western Alps (France). – **Fr**: AMa, Isè.

***Porina mammillosa* (Th. Fr.) Vain.**

Syn.: *Sagedia declivum* Bagl. & Carestia, *Sagedia trechalea* (Nyl.) Arnold, *Segestria mammillosa* Th. Fr.

L – Subs.: sil, bry, deb – Alt.: 4–5 – Note: an arctic-alpine, probably circumpolar species found on bryophytes and plant debris over siliceous substrata. – **Au**: V, T, S, K, St. **It**: Frl, Piem.

***Porina rosei* Sérus.**

Syn.: *Zamenhofia rosei* (Sérus.) P. James

L – Subs.: cor – Alt.: 2 – Note: a species with orange-brown, fragile isidia, perithecia partly immersed in the thallus, and ellipsoid, 3-septate ascospores which are up to *c.* 30 µm long; on bark of deciduous trees in humid forests; widespread and most common in Western Europe and Macaronesia, with a few records from the Western Alps (France). – **Fr**: Drô, Isè, Var.

***Porina rubentior* (Stirt.) Müll. Arg.**

Syn.: *Segestria rubentior* (Stirt.) R.C. Harris, *Verrucaria rubentior* Stirt.

L – Subs.: fol – Alt.: 2–3 – Note: a foliicolous species with very small thalli, minute, dark brownish-red perithecia (the colour of the involucrellum), and fusiform to oblong, 3-septate ascospores which are up to *c.* 20 µm long; widespread in the tropics, with only a few extratropical records including Western Europe, and there usually on *Buxus sempervirens*, with a single record from the Western Alps (France). – **Fr**: Isè.

***Porina sudetica* (Körb.) Lettau**

Syn.: *Sagedia sudetica* (Körb.) Körb., *Verrucaria sudetica* Körb.

L – Subs.: deb, bry – Alt.: 4–5 – Note: a species with an effuse, granulose thallus, red-brown perithecia, and fusiform, up to 5-septate ascospores which are to *c.* 40 µm long; encrusting saxicolous bryophytes (*Andreaea*) and plant debris in the subalpine and alpine belts; rare in the Central European mountains, with a few records from the Eastern Alps (Austria). – **Au**: K, St, O, N.

***Porocyphus coccodes* Flot. *ex* Körb.**

Syn.: *Collema coccodes* Flot. ad int., *Collema furfurellum* Nyl., *Porocyphus areolatus* Flot. *ex* Körb., *Porocyphus cataractarum* Körb., *Porocyphus furfurellus* (Nyl.) Forssell, *Porocyphus vivariensis* Couderc, *Psorotichia pyrenopsoides* (Nyl.) Forssell

L – Subs.: cal, sil, int – Alt.: 2–5 – Note: a temperate to southern boreal-montane, probably holarctic lichen found in seepage tracks on steeply inclined surfaces of basic siliceous rocks, *e.g.* with *Peltula*, more rarely along creeks and rivers. – **Au**: T, S, St. **Sw**: GR. **Fr**: AMa, Sav, HSav, Var.

***Porocyphus rehmicus* (A. Massal.) Zahlbr.**

Syn.: *Porocyphus byssoides* Hepp, *Porocyphus globulosus* Couderc, *Porocyphus riparius* (Arnold) Körb., *Porocyphus rufescens* (Hy) Zahlbr., *Psorotichia geophila* Hy, P*sorotichia rehmica* A. Massal., *Psorotichia riparia* Arnold

L – Subs.: cal – Alt.: 2–3 – Note: on seepage tracks of base-rich or slightly calciferous rocks, more rarely along creeks and rivers, often found on sandstone walls. – **Au**: T, S, N. **Ge**: Ge. **Sw**: GR, ?SZ, VS. **Fr**: AMa, Sav, Var, Vau. **It**: Ven, Lomb, Piem.

***Porpidia albocaerulescens* (Wulfen) Hertel & Knoph**

Syn.: *Haplocarpon albocaerulescens* (Wulfen) M. Choisy, *Huilia albocaerulescens* (Wulfen) Hertel, *Lecidea albocaerulescens* (Wulfen) Ach., *Lecidea alboflavescens* Vain., *Lecidea nitescens* Leight., *Lichen albocaerulescens* Wulfen

L – Subs.: sil – Alt.: 1–3 – Note: on siliceous boulders in sheltered, humid situations, such as in deciduous forests. – **Au**: V, T, S, K, St, N. **Ge**: OB. **Sw**: BE, TI. **It**: Ven, TAA, Lomb, Piem, VA. **Sl**: SlA.

***Porpidia cinereoatra* (Ach.) Hertel & Knoph**

Syn.: *Haplocarpon cinereoatrum* (Ach.) M. Choisy, *Haplocarpon musivum* (Körb.) Vězda, *Huilia cinereoatra* (Ach.) Hertel, Huilia macrocarpa (DC.) Hertel var. convexa (Fr.) Hertel, *Huilia musiva* (Körb.) Vězda, *Lecidea cinereoatra* Ach., *Lecidea convexa* (Fr.) Flagey, Lecidea macrocarpa (DC.) Steud. var. convexa (Fr.) H. Magn., *Lecidea meiospora* (Nyl.) Nyl., *Lecidea musiva* Körb., *Porpidia musiva* (Körb.) Hertel & Knoph

L – Subs.: sil – Alt.: 1–5 – Note: on siliceous rocks wetted by rain, especially low boulders and large pebbles in rainy-humid areas, with a wide altitudinal range; widespread throughout the Alps. – **Au**: V, T, S, K, St, N, B. **Sw**: BE, LU, SZ, UR, VS. **Fr**: AHP, HAl, AMa, Isè, Sav, HSav, Var. **It**: Frl, TAA, Lomb, Piem, VA, Lig. **Sl**: SlA.

***Porpidia contraponenda* (Arnold) Knoph & Hertel**

Syn.: *Lecidea contraponenda* Arnold

L – Subs.: sil – Alt.: 3–4 – Note: on siliceous rocks near the ground, *e.g.* on large pebbles, in humid-moist situations, in open forests on track sides and in shrublands, most frequent in upland areas but generally rare. – **Au**: V, T, K. **Ge**: Schw. **Sw**: BE. **It**: TAA.

***Porpidia crustulata* (Ach.) Hertel & Knoph**

Syn.: *Biatora crustulata* (Ach.) Hepp, *Haplocarpon crustulatum* (Ach.) M. Choisy, *Huilia crustulata* (Ach.) Hertel, *Lecidea chrysoteichiza* Nyl., *Lecidea crustulata* (Ach.) Spreng., *Lecidea martinatiana* A. Massal., Lecidea parasema (Ach.) Ach. var. crustulata Ach., *Lecidea scutellata* Walt. Watson, *Lecidea umensis* H. Magn.

L – Subs.: sil, xyl – Alt.: 1–5 – Note: a widespread, holarctic early coloniser of siliceous pebbles and small stones on the ground, with a wide altitudinal range; common throughout the Alps. – **Au**: V, T, S, K, St, O, N, B. **Ge**: OB, Schw. **Sw**: BE, GR, LU, SZ, TI, UR, VD, VS. **Fr**: AHP, HAl, AMa, Isè, Sav, HSav, Var, Vau. **It**: Frl, Ven, TAA, Lomb, Piem, VA, Lig. **Sl**: SlA, Tg.

***Porpidia flavicunda* (Ach.) Gowan**

Syn.: *Biatora flavocoerulescens* (Hornem.) Hepp, *Haplocarpon flavocaerulescens* (Hornem.) V. Wirth *ex* Hertel, *Huilia flavicunda* (Ach.) Mas. Inoue, *Huilia flavocaerulescens* (Hornem.) Hertel, Lecidea contigua (Hoffm.) Fr. var. flavicunda (Ach.) Nyl., *Lecidea flavicunda* Ach., *Lecidea flavocaerulescens* Hornem., *Porpidia flavocaerulescens* (Hornem.) Hertel & A.J. Schwab

L – Subs.: sil – Alt.: 3–5 – Note: a variable species which can produce both apothecia and soredia, found on siliceous boulders in humid and wind-protected situations, *e.g.* in deep gorges or along mountain creeks in woodlands, reaching beyond treeline in the Alps. – **Au**: V, T, S, K, St. **Sw**: BE, GR. **It**: Ven, TAA, Lomb, Lig.

***Porpidia flavocruenta* Fryday & Buschbom**

L – Subs.: sil – Alt.: 3–4 – Note: a species resembling *P. flavicunda*, but with a usually yellow-orange thallus lacking lichen substances and larger apothecia recalling those of *P. macrocarpa* (but often pruinose), the exciple composed of thick radiating hyphae with the orange-brown inner part reacting K+ red; on siliceous boulders and low outcrops; widespread in Europe and also known from Alaska, with a few records from the Eastern Alps (Austria), mostly near treeline. – **Au**: S, St.

***Porpidia grisea* Gowan**

L – Subs.: sil – Alt.: 3 – Note: a silicicolous species resembling *P. tuberculosa* in the amyloid medulla and the presence of confluentic acid as a major substance, but esorediate and richly fertile, the apothecia usually with pruinose discs and the ascospores only up to 15 µm long; widespread in the Holarctic region from the Arctic to temperate-alpine zones but rare, with a single record from the Eastern Alps (Austria). – **Au**: S.

***Porpidia hydrophila* (Fr.) Hertel & A.J. Schwab**

Syn.: *Haplocarpon hydrophilum* (Fr.) V. Wirth, *Huilia hydrophila* (Fr.) Hertel, *Lecidea hydrophila* Fr.

L – Subs.: sil – Alt.: 2–3 – Note: a species of the *P. albocaerulescens*-group with a bright aeruginose epihymenium and an exciple with a carbonaceous outer layer and a much paler, brown inner layer with easily visible hyphae; on damp siliceous rocks, often along streams; rare throughout Europe, most frequent in Western Europe at low elevations, with a few records from the Eastern Alps (Austria). – **Au**: T, N.

***Porpidia macrocarpa* (DC.) Hertel & A.J.Schwab**

Syn.: *Haplocarpon macrocarpum* (DC.) M. Choisy, *Haplocarpon nigrocruentum* (Anzi) Hertel, *Huilia macrocarpa* (DC.) Hertel, *Huilia nigrocruenta* (Anzi) Hertel, *Lecidea baderi* Müll. Arg., *Lecidea contigua auct. non* (Hoffm.) Fr., *Lecidea contortula* Stirt., *Lecidea macrocarpa* (DC.) Steud., *Lecidea nigrocruenta* Anzi, *Lecidea phylliscina* Nyl., *Lecidea platycarpa* Ach., *Lecidea steriza* (Ach.) Vain., *Lecidea tenebrans* Nyl., *Lecidea vinorubens* Werner, *Patellaria macrocarpa* DC., L – *Porpidia nigrocruenta* (Anzi) Diederich & Sérus.

L – Subs.: sil, int, cal – Alt.: 1–6 – Note: on siliceous rocks near the ground, sometimes on metal-rich substrata in humid-sheltered situations, with a wide altitudinal range; widespread throughout the Alps. – **Au**: V, T, S, K, St, O, N. **Ge**: OB. **Sw**: BE, GL, GR, LU, SZ, TI, UR, VD, VS. **Fr**: AHP, HAl, AMa, Isè, Sav, HSav, Var, Vau. **It**: Frl, Ven, TAA, Lomb, Piem, VA, Lig. **Sl**: SlA, Tg.

***Porpidia melinodes* (Körb.) Gowan & Ahti**

Syn.: *Aspicilia melinodes* Körb.

L – Subs.: sil – Alt.: 5 – Note: on horizontal to moderately inclined surfaces of siliceous rocks lying on or near the ground in scree fields, mostly in upland areas; for a long time treated as a sorediate morph of *P. flavicunda*; some records are likely hidden under those of that species. – **Au**: ?V, T. **It**: TAA.

***Porpidia ochrolemma* (Vain.) Brodo & R. Sant.**

Syn.: *Aspicilia ochrolemma* (Vain.) Hue, *Haplocarpon melinodes auct. non* (Körb.) V. Wirth, *Huilia melinodes auct. non* (Körb.) Hertel, *Hymenelia ochrolemma* (Vain.) Gowan & Ahti, *Lecanora ochrolemma* (Vain.) Vain., *Lecidea melinodes auct. non* (Körb.) H. Magn. *ex* Lynge, *Pertusaria ochrolemma* Vain., *Porpidia melinodes auct. non* (Körb.) Gowan & Ahti, *Porpidia pseudomelinodes* A.J. Schwab

L – Subs.: sil – Alt.: 3–5 – Note: on siliceous rocks near watercourses or in humid, but well-illuminated situations near or above treeline. – **Au**: V, T, S, K, St. **Sw**: SZ, TI. **Fr**: HSav. **It**: TAA, Lomb, Piem.

***Porpidia platycarpoides* (Bagl.) Hertel**

Syn.: *Huilia percontigua* (Nyl.) Mas. Inoue, *Huilia platycarpoides* (Bagl.) Hertel, *Lecidea corollidea* Stirt., *Lecidea percontigua* Nyl., *Lecidea platycarpoides* Bagl., *Lecidea reagens* Zschacke, Porpidia cinereoatra (Ach.) Hertel & Knoph var. percontigua (Nyl.) Boissière & Cl. Roux, Porpidia cinereoatra (Ach.) Hertel & Knoph var. platycarpoides (Bagl.) Boissière & Cl. Roux, Porpidia macrocarpa (DC.) Hertel & A.J. Schwab var. percontigua (Nyl.) Boissière & Cl. Roux, Porpidia macrocarpa (DC.) Hertel & A.J. Schwab var. platycarpoides (Bagl.) Boissière & Cl. Roux

L – Subs.: sil – Alt.: 2 – Note: a Mediterranean-Atlantic lichen found on siliceous rocks in rather sheltered situations, with a single record from the base of the Western Alps. – **Fr**: Vau.

***Porpidia rugosa* (Taylor) Coppins & Fryday**

Syn.: *Endocarpon rugosum* Taylor, *Haplocarpon glaucophaeum* (Körb.) V. Wirth, *Huilia glaucophaea* (Körb.) Hertel, Lecidea albocaerulescens (Wulfen) Ach. var. alpina Schaer., Lecidea albocaerulescens (Wulfen) Ach. var. soralifera Vain., *Lecidea albuginosa* Nyl., *Lecidea glaucophaea* Körb., *Lecidea phaeenterodes* Nyl., *Lecidea soredizodes* (Nyl.) Vain. *non* (Lamy *ex* Nyl.) Sandst., *Porpidia glaucophaea* (Körb.) Hertel & Knoph

L – Subs.: sil, sil-aqu – Alt.: 2–5 – Note: a mainly mild-temperate species found on siliceous, often metamorphic rocks in sheltered situations, such as in forests and deep gorges, along rivers and creeks, or on pebbles on moist ground. – **Au**: ?V, T, S, K, St. **Sw**: BE, GR, SZ, TI, VD, VS. **Fr**: AMa, Isè, Sav, HSav. **It**: TAA, Lomb. **Sl**: SlA.

***Porpidia soredizodes* (Lamy *ex* Nyl.) J.R. Laundon**

Syn.: *Haplocarpon soredizodes* (Lamy *ex* Nyl.) V. Wirth, *Huilia soredizodes* (Lamy *ex* Nyl.) Hertel, Lecidea macrocarpa (DC.) Steud. var. soredizodes Lamy *ex* Nyl., *Lecidea soredizodes* (Lamy *ex* Nyl.) Sandst. *non* (Nyl.) Vain.

L – Subs.: sil – Alt.: 2–4 – Note: a cool-temperate to boreal-montane lichen found on siliceous rocks in forests, gorges, and on N-exposed faces of large siliceous boulders, mostly in upland areas but not reaching beyond treeline. – **Au**: V, T, S, K, St. **Ge**: Ge. **Sw**: LU, SZ. **Fr**: Isè, HSav. **It**: TAA.

**Porpidia speirea (Ach.) Kremp. var. speirea**

Syn.: *Huilia speirea* (Ach.) Kremp., *Lecidea speirea* (Ach.) Ach., *Lichen speireus* Ach.

L – Subs.: sil, int – Alt.: 2–6 – Note: on inclined faces of schist and weakly calciferous rocks in cold-humid situations, mostly in upland areas; widespread throughout the Alps. – **Au**: V, T, S, K, St, N. **Ge**: OB. **Sw**: BE, GL, GR, LU, SG, SZ, TI, UR, UW, VS. **Fr**: AHP, HAl, Isè, Sav, HSav. **It**: Frl, Ven, TAA, Lomb, Piem, VA, Lig. **Sl**: SlA.

**Porpidia speirea (Ach.) Kremp. var. alpina (Hepp *ex* Arnold) Clauzade & Cl. Roux *ex* Hafellner & Türk**

Syn.: Lecidea contigua (Hoffm.) Fr. f. alpina Hepp *ex* Arnold, Lecidea speirea (Ach.) Ach. var alpina (Hepp *ex* Arnold) Hertel

L – Subs.: sil, int – Alt.: 4–5 – Note: a well-distinct taxon, not always distinguished from the other varieties, hence distribution poorly known. – **Au**: V, T, S, K, St. **Fr**: AHP, HAl, AMa, Sav, HSav.

**Porpidia speirea (Ach.) Kremp. var. prochsthallina (A. Massal.) Clauzade & Cl. Roux *ex* Hafellner & Türk**

Syn.: Lecidea elata Schaer. var. prochsthallina A. Massal., Lecidea speirea (Ach.) Ach. var prochsthallina (A. Massal.) Hertel

L – Subs.: sil, int – Alt.: 4–5 – Note: a taxon differing from var. speirea in having a pale yellow thallus; on calcareous schists, widespread in the Holarctic region but rare throughout the Alps. – **Au**: T, S. **Fr**: Sav.

***Porpidia superba* (Körb.) Hertel & Knoph**

Syn.: *Huilia superba* (Körb.) Hertel, *Lecidea incrassata* H. Magn., Lecidea macrocarpa (DC.) Steud. var. superba (Körb.) Th. Fr., *Lecidea superba* Körb.; incl. Porpidia superba (Körb.) Hertel & Knoph f. sorediata Fryday

L – Subs.: sil, int – Alt.: 3–6 – Note: a mainly arctic-alpine lichen found on weakly calciferous or basic siliceous rocks in sheltered situations. – **Au**: V, T, S, K, St, O, N. **Ge**: Ge. **Sw**: VS. SZ. **Fr**: HAl, AMa, Sav, HSav. **It**: TAA, Piem.

***Porpidia trullisata* (Kremp.) Körb.**

Syn.: *Diplotomma trullisatum* Kremp., *Lecidea euspeirea* Nyl., Lecidea speirea (Ach.) Ach. var. trullisata (Kremp.) Arnold, *Lecidea trullisata* (Kremp.) Anzi

L – Subs.: sil, int – Alt.: 4–6 – Note: on north-facing, steeply inclined surfaces of weakly calciferous rocks with optimum above treeline. – **Au**: V, T, S, K, O. **Ge**: Schw. **Sw**: BE, LU, TI, VD. **Fr**: AHP, HAl, Sav, HSav. **It**: TAA, Piem, VA.

***Porpidia tuberculosa* (Sm.) Hertel & Knoph**

Syn.: *Huilia tuberculosa* (Sm.) P. James, Lecidea confluens (Weber) Ach. var. tumida (A. Massal.) A. Massal., ?*Lecidea cyclosora* Lettau, *Lecidea sorediza* Nyl., *Lecidea tumida* A. Massal., *Spiloma tuberculosum* Sm.

L – Subs.: sil, int, cor – Alt.: 2–6 – Note: on steeply inclined surfaces of siliceous rocks, or in woodlands, mostly near the ground. – **Au**: V, T, S, K, St, N. **Sw**: BE, VS. **Fr**: AMa, Var. **It**: Frl, Ven, TAA, Lomb, Piem, VA. **Sl**: SlA.

***Porpidia zeoroides* (Anzi) Knoph & Hertel**

Syn.: Huilia macrocarpa (DC.) Hertel var. trullisata (Arnold) Hertel, Lecidea macrocarpa (DC.) Steud. var. trullisata (Arnold) Mig., Lecidea platycarpa Ach. var. trullisata (Arnold) Arnold, *Lecidea zeoroides* Anzi

L – Subs.: sil, int – Alt.: 3–6 – Note: on steeply inclined, often north-exposed faces of weakly calciferous rocks in upland areas. – **Au**: V, T, S, K, St. **Ge**: OB, Schw. **Sw**: BE, GR, LU, SZ, UR, UW, VS. **Fr**: AHP, HAl, AMa, Sav, HSav. **It**: Frl, TAA, Lomb, Piem, VA.

***Porpidinia tumidula* (Sm.) Timdal**

Syn.: *Biatorina mammillaris* Jatta *nom.illeg.*, *Biatorina tumidula* (Sm.) A.L. Sm., *Lecidea mammillaris* Dufour *nom.illeg.*, *Lecidea mezenteriformis* (Tourr. *ex* Vill.) Vain., *Lichen mammillari*s Gouan *nom.illeg.*, *Lichen tumidulus* Sm., *Thalloidima mammillare* A. Massal. *nom.illeg.*, *Thalloidima mezenteriforme* (Tourr. *ex* Vill.) Arnold, *Thalloidima submammillare* Flagey, *Thalloidima tumidulum* (Sm.) Szatala, *Toninia hercegovinica* Zahlbr., *Toninia mezenteriformis* (Tourr. *ex* Vill.) Schuler, *Toninia submammillaris* (Flagey) Zahlbr., *Toninia tumidula* (Sm.) Zahlbr.

L – Subs.: cal, ter-cal – Alt.: 1–4 – Note: on weathered calciferous rocks, most often in fine crevices and on steeply inclined surfaces, with optimum in the submediterranean belt. – **Au**: T, St, O, N. **Sw**: VD, VS. **Fr**: AHP, HAl, AMa, Drô, Isè, HSav, Var, Vau. **It**: Frl, Ven, TAA, Piem, Lig.

***Protoblastenia aurata* Poelt & Vězda**

L – Subs.: cal – Alt.: 4–5 – Note: a rare species, peculiar in the genus due to its yellow-orange thallus reacting K+ purple; on calciferous siliceous rocks, especially schists. – **Au**: S. **Ge**: Schw. **Fr**: Sav. **It**: Lig.

***Protoblastenia calva* (Dicks.) Zahlbr.**

Syn.: Biatora rupestris (Scop.) Fr. var. calva (Dicks.) Arnold, Blastenia rupestris (Scop.) Zahlbr. var. calva (Dicks.) Lettau, *Lichen calvus* Dicks., Placodium rupestre (Scop.) Branth & Rostr. var. calvum (Dicks.) A.L. Sm., Protoblastenia rupestris (Scop.) J. Steiner var. calva (Dicks.) J. Steiner

L – Subs.: cal – Alt.: 2–5 – Note: on steeply inclined faces of hard limestones and dolomite, with a wide altitudinal range but most common in the mountains, descending to lower altitudes in humid areas; widespread and common throughout the Alps. – **Au**: V, T, S, K, St, O, N. **Ge**: OB, Schw. **Sw**: BE, LU, SZ, VD. **Fr**: AHP, HAl, AMa, Drô, Isè, Sav, HSav, Var, Vau. **It**: Frl, Ven, TAA, Lomb, Piem, Lig. **Sl**: SlA, Tg.

***Protoblastenia calvella* Kainz & Rambold**

L – Subs.: cal – Alt.: 2–4 – Note: a species resembling *P. calva*, but with a distinctly epilithic thallus and smaller apothecia (less than 1 mm in diam.); on shaded calcareous rocks, so far only known from the Eastern Alps (Austria). – **Au**: T, St, O. **Ge**: Ge.

***Protoblastenia cyclospora* (Hepp *ex* Körb.) Poelt**

Syn.: *Biatora cyclospora* Hepp *ex* Körb., *Biatora rubidula* (Nyl.) Walt. Watson, *Lecidea cyclospora* (Hepp *ex* Körb.) Müll. Arg., *Lecidea rubidula* Nyl., *Protoblastenia globulificans* (Nyl.) Zahlbr.

L – Subs.: cal – Alt.: 2–4 – Note: on steeply inclined surfaces of calcareous rocks in humid-rainy areas below the alpine belt. – **Au**: T, K, St. **Ge**: Ge. **Sw**: UW. **Fr**: AHP, Sav. **It**: Frl, TAA.

***Protoblastenia geitleri* Zahlbr.**

L – Subs.: cal – Alt.: 3 – Note: a species resembling *P. cyclospora* in the subglobose ascospores, but with red-brown apothecia with a red-violet hypothecium; on calcareous rocks (dolomite); so far only known from the Eastern Alps (Austria). – **Au**: ?V, N.

**Protoblastenia incrustans (DC.) J. Steiner var. incrustans**

Syn.: Biatora rupestris (Scop.) Fr. var. incrustans (DC.) A. Massal., Blastenia rupestris (Scop.) Zahlbr. var. incrustans (DC.) Lettau, *Caloplaca incrustans* (DC.) Flagey, *Patellaria incrustans* DC., *Placodium incrustans* (DC.) A.L. Sm., Protoblastenia rupestris (Scop.) J. Steiner var. incrustans (DC.) Zahlbr.

L – Subs.: cal, int – Alt.: 1–6 – Note: a widespread, temperate to circum-Arctic lichen, one of the most common species on calcareous rocks in natural habitats throughout the Alps, with a very wide altitudinal range, reaching the nival belt. – **Au**: V, T, S, K, St, O, N. **Ge**: OB, Schw. **Sw**: BE, GR, LU, SZ, VS. **Fr**: AHP, HAl, AMa, Drô, Isè, Sav, HSav, Var, Vau. **It**: Frl, Ven, TAA, Lomb, Piem, VA, Lig. **Sl**: SlA, Tg.

**Protoblastenia incrustans (DC.) J. Steiner var. coniasis (A. Massal.) Nimis**

Syn.: *Biatora coniasis* A. Massal., *Lecidea coniasis* (A. Massal.) Lettau, *Protoblastenia coniasis* (A. Massal.) Poelt

L – Subs.: cal, int – Alt.: 3–5 – Note: on steeply inclined surfaces of more or less calciferous rocks near and above treeline; certainly more widespread throughout the Alps, but not common; this taxon is well worthy of further study. – **Au**: V, T, S, K, N. **Fr**: AHP, HAl, AMa, Sav. **It**: TAA, Lomb.

***Protoblastenia laeta* (Poelt) Kainz & Rambold**

Syn.: Protoblastenia calva (Dicks.) Zahlbr. var. laeta Poelt

L – Subs.: cal – Alt.: 3 – Note: a species resembling *P. calva*, but with an epilithic, whitish thallus, and yellow to yellow-orange apothecia; on shaded calcareous rocks, usually on steep, north-exposed rock faces; widespread in Europe but rare, with a few records from the Eastern Alps (Austria, Germany). – **Au**: S. **Ge**: Schw.

***Protoblastenia lilacina* Poelt & Vězda**

L – Subs.: cal – Alt.: 3 – Note: a rather poorly understood species of sunny calcareous rocks below the subalpine belt, reported from several localities in Central Europe. It is characterised by the weak reaction of the apothecia to K; probably some herbarium specimens of *P. calva* will turn out to be *P. lilacina*. – **Au**: S, K, O, N. **It**: Ven.

***Protoblastenia pseudoincrustans* Kainz & Rambold ined.**

L – Subs.: cal – Alt.: 4–5 – Note: a species resembling *P. incrustans*, but with a distinctly epilithic, whitish to brownish thallus and somewhat larger apothecia (to 1 mm in diam.); on shaded calcareous rocks, so far only known from the Eastern Alps (Austria). – **Au**: S.

**Protoblastenia rupestris (Scop.) J. Steiner var. rupestris**

Syn.: *Biatora irrubata* (Ach.) Kernst., *Biatora rupestris* (Scop.) Fr., *Lecanora irrubata* (Ach.) Nyl., *Lecanora rupestris* (Scop.) Nyl., *Lecidea rupestris* (Scop.) Ach., *Lichen rupestris* Scop., *Placodium rupestre* (Scop.) Branth. & Rostr., Protoblastenia rupestris (Scop.) J. Steiner var. irrubata (Ach.) Szatala, *Verrucaria rupestris* (Scop.) F.H. Wigg. *non* Schrad.

L – Subs.: cal – Alt.: 1–5 – Note: a common and ecologically wide-ranging species, most frequent on faces of calciferous rocks wetted by rain and near the ground, an early coloniser of several substrata, from mortar-cement to basic siliceous pebbles, often found also in urban areas, most frequent below the subalpine belt. – **Au**: V, T, S, K, St, O, N, B. **Ge**: OB. **Sw**: BE, GR, LU, SZ, UW, VD, VS. **Fr**: AHP, HAl, AMa, Drô, Isè, Sav, HSav, Var, Vau. **It**: Frl, Ven, TAA, Lomb, Piem, Lig. **Sl**: SlA. **Li**.

**Protoblastenia rupestris (Scop.) J. Steiner subsp. rhodothecia Cl. Roux**

Syn.: Protoblastenia rupestris (Scop.) J. Steiner var. rhodothecia Asta, Clauzade & Cl. Roux *nom. inval*.

L – Subs.: cal, int – Alt.: 4–5 – Note: a taxon resembling var. rupestris, but apothecia with a purple-rose hypothecium; on superficially decalcified calcareous rocks (ecology similar to that of *Stenhammarella turgida*) in alpine environments; recorded with certainty only from France, but perhaps not recognised elsewhere. – **Au**: ?V. **Fr**: AHP, HAl, AMa, Sav, HSav.

**Protoblastenia rupestris (Scop.) J. Steiner var. sanguinea (Arnold) Zahlbr.**

Syn.: Biatora rupestris (Scop.) Fr. var. sanguinea Arnold, Protoblastenia calva (Dicks.) Zahlbr. var. sanguinea (Arnold) Cl. Roux

L – Subs.: cal – Alt.: 3–4 – Note: a taxon resembling var. rupestris, but with brighter, distinctly red apothecia and a faintly blood-red hypothecium; on shaded, moist rock faces of calcareous boulders; widespread in the Alps, but much rarer than the typical variety. – **Au**: T, O, N. **Sw**: SZ. **Fr**: AHP, AMa, Var, Vau.

**Protoblastenia siebenhaariana (Körb.) J. Steiner var. siebenhaariana**

Syn.: *Biatora siebenhaariana* Körb., *Blastenia siebenhaariana* (Körb.) Lettau, Protoblastenia rupestris (Scop.) J. Steiner subsp. siebenhaariana (Körb.) A.L. Sm.

L – Subs.: int, cal, sil – Alt.: 3–5 – Note: a mainly arctic-alpine, probably circumpolar lichen found on base-rich or calciferous siliceous rocks and on dolomite in upland areas; not frequent in the Alps, but widespread, and locally common. – **Au**: V, T, S, K, St, O, N. **Ge**: OB, Schw. **Sw**: GR, LU, SZ, TI, VS. **Fr**: Sav. **It**: Frl, Ven, TAA, Lomb, Piem, VA. **Sl**: Tg.

**Protoblastenia siebenhaariana (Körb.) J. Steiner subsp. albida Cl. Roux ined.**

Syn.: Protoblastenia rupestris (Scop.) J. Steiner subsp. albida Asta & Cl. Roux [invalidly published, ICN Art. 40.1 + 8], Protoblastenia rupestris (Scop.) J. Steiner subsp. albida Cl. Roux, Protoblastenia siebenhaariana (Körb.) J. Steiner subsp. albida (Asta & Cl. Roux) Clauzade & Cl. Roux *comb. inval*.

L # – Subs.: sil, cal, int – Alt.: 3–5 – Note: similar to var. siebenhaariana, but with a colourless hypothecium and a different ecology (mostly associated to *Lecanora albula*); the application of the name was often inconsistent, so that the distributional data (outside France) are hardly reliable. – **Au**: ?V, ?T. **Fr**: AHP, HAl, AMa.

**Protoblastenia siebenhaariana (Körb.) J. Steiner var. alpina (Arnold) Clauzade & Cl. Roux**

Syn.: Biatora rupestris (Scop.) Fr. var. alpina Arnold

L # – Subs.: cal – Alt.: 3–4 – Note: doubtfully different from var. siebenhaariana (the protologue specifies characters distinguishing it from *P. rupestris*, but not from *P. siebenhaariana*!), so that the application of the name was inconsistent and the distributional data are hardly reliable. – **Au**: S. **Ge**: OB. **It**: TAA.

***Protoblastenia szaferi* J. Nowak**

L – Subs.: cal – Alt.: 4–5 – Note: this is the only species in the genus with persistently flat, orange apothecia basally immersed in shallow pits of the calcareous rock; on N-exposed, steep rock faces, from the treeline ecotone to the alpine belt; only known from the Tatra mountains and the Alps, and there so far only recorded from the Eastern Alps (Austria, Germany), but likely to have been overlooked elsewhere. – **Au**: St, O. **Ge**: OB, Schw.

***Protoblastenia terricola* (Anzi) Lynge**

Syn.: Biatora rupestris (Scop.) Fr. var. terricola Anzi, *Biatora terricola* (Anzi) Th. Fr., *Blastenia terricola* (Anzi) Lindau, *Lecidea terricola* (Anzi) Th. Fr., Protoblastenia siebenhaariana (Körb.) J. Steiner var. terricola (Anzi) Hafellner & Türk

L # – Subs.: ter-cal – Alt.: 3–5 – Note: on soil over weakly calcareous or dolomitic substrata in upland areas; perhaps this is only a terricolous morph of *P. siebenhaariana*; most frequent in the southern part of its distributional range. – **Au**: V, T, S, K, St, O. **Sw**: GR, UR, VS. **Fr**: HAl, Sav, HSav, Vau. **It**: Frl, Ven, TAA, Lomb, Piem.

***Protomicarea limosa* (Ach.) Hafellner**

Syn.: *Lecidea borealis* (Körb.) Anzi, *Lecidea ementiens* Nyl., *Lecidea limosa* Ach., *Lecidella borealis* Körb.

L – Subs.: ter-sil, bry, deb – Alt.: 4–6 – Note: an arctic-alpine to boreal-montane, circumpolar lichen found on naked soil in sites with a long snow cover, in clearings of alpine grasslands, more rarely on moribund bryophytes and plant debris. – **Au**: V, T, S, K, St. **Ge**: OB. **Sw**: BE, GR, SZ, TI, UR, VS. **Fr**: Isè, Sav, HSav. **It**: Frl, Ven, TAA, Lomb, Piem, VA.

***Protopannaria pezizoides* (Weber) P.M. Jørg. & S. Ekman**

Syn.: *Lecanora brunnea* (Sw.) Ach., *Lecanora pezizoides* (Weber) Borrer, *Lichen brunneus* Sw., *Lichen pezizoides* Weber, *Pannaria brunnea* (Sw.) A. Massal., *Pannaria pezizoides* (Weber) Trevis.

L – Subs.: bry, deb, ter-sil, ter-cal, cor – Alt.: 2–6 – Note: an arctic-alpine to boreal-montane, circumpolar lichen found on mosses, plant debris, and organic soil in open habitats, with optimum near and above treeline. – **Au**: V, T, S, K, St, O, N. **Ge**: OB. **Sw**: AP, BE, FR, GL, GR, LU, SG, SZ, TI, UR, UW, VD, VS. **Fr**: AHP, HAl, AMa, Drô, Isè, Sav, HSav, Vau. **It**: Frl, Ven, TAA, Lomb, Piem, VA, Lig. **Sl**: SlA, Tg.

**Protoparmelia badia (Hoffm.) Hafellner var. badia**

Syn.: *Lecanora badia* (Hoffm.) Ach., *Lecanora grandis* H. Magn., *Lecanora picea* (Dicks.) Nyl. *nonauct.*, *Parmelia badia* (Hoffm.) Hepp, *Protoparmelia picea* (Dicks.) Hafellner *nonauct.*, *Solenopsora badia* (Hoffm.) M. Choisy & Werner, *Verrucaria badia* Hoffm.

L – Subs.: sil, int – Alt.: 2–6 – Note: on siliceous rocks, with a wide altitudinal range, reaching the nival belt. The species, in its present circumscription, is heterogeneous. – **Au**: V, T, S, K, St, O, N. **Ge**: Ge. **Sw**: BE, GR, LU, SZ, TI, UR, UW, VD, VS. **Fr**: AHP, HAl, AMa, Isè, Sav, HSav. **It**: Frl, Ven, TAA, Lomb, Piem, VA, Lig. **Sl**: SlA. **Li**.

**Protoparmelia badia (Hoffm.) Hafellner var. cinereobadia (Harm.) Clauzade & Cl. Roux *ex* Hafellner & Türk**

Syn.: Lecanora badia (Hoffm.) Ach. var. cinereobadia Harm.

L # – Subs.: sil – Alt.: 3–6 – Note: a name used for morphs with an ash-grey thallus, but the application of the name was probably not consistent with the original concept (taxon described for grey morphs with a thick, rough thallus, medulla reacting KC+ orange-red, and apothecia mostly smaller than 1 mm in diam.); data on ecology and distribution are therefore difficult to interpret. – **Au**: V, T, S, K, St, N. **Fr**: AHP, HAl, AMa, Sav, HSav.

***Protoparmelia cupreobadia* (Nyl.) Poelt**

Syn.: Lecanora badia (Hoffm.) Ach. var. cupreobadia (Nyl.) Boistel, *Lecanora cupreobadia* Nyl.

L – Subs.: sil – Alt.: 4–5 – Note: on gneiss and compact porphyric rocks near and especially above treeline, starting the life-cycle on yellow *Rhizocarpon*-species, later becoming autonomous; probably more widespread in the Alps. The species does not belong to *Protoparmelia* *s.str.* – **Au**: V, T, St. **Fr**: AHP, AMa, Isè, HSav. **It**: TAA.

***Protoparmelia hypotremella* Herk, Spier & V. Wirth**

L – Subs.: cor, xyl – Alt.: 2–4 – Note: a species only known in the sterile state, resembling *Xylopsora caradocensis* in the spreading, grey to olive thallus consisting of granules developing into extremely minute squamules which occasionally bear isidioid outgrowths; on bark and wood, both in forests and in rural landscapes; widespread in Europe from the lowlands to subalpine coniferous forests, but easily overlooked; the distributional gaps in Alps may be therefore artificial. – **Au**: T, S, K, O. **Ge**: Schw. **Sw**: BE, GR, LU, SZ, TI, UR, UW, VS.

***Protoparmelia leproloma* (R. Sant.) Rambold & Poelt**

Syn.: *Lecidea leproloma* R. Sant.

L – Subs.: sil, sil-par – Alt.: 4–5 – Note: an arctic-alpine silicicolous species which starts the life-cycle on other crustose lichens, more widespread in Scandinavia than in the Alps, where it mostly occurs above treeline. The species does not belong to *Protoparmelia* *s.str.* – **It**: Piem.

***Protoparmelia loricata* Poelt & Vězda**

L – Subs.: sil-par – Alt.: 4–5 – Note: a species with a pale brown thallus consisting of glossy, convex areoles and concolorous apothecia with a later excluded thalline margin; growing as a parasite on *Lecanora umbrosa*, extremely rare on shaded, steeply inclined to vertical faces of basic siliceous rocks with a low content in calcium (*e.g.* amphibolite); only known from the Eastern Alps (Austria) and the Karakorum. – **Au**: T, St.

***Protoparmelia memnonia* Hafellner & Türk**

Syn.: *Lecanora picea auct. non* (Dicks.) Nyl., *Protoparmelia picea auct. non* (Dicks.) Hafellner

L – Subs.: sil – Alt.: 4–5 – Note: on hard siliceous rocks in exposed situations; probably ranging throughout the siliceous Alps, in rainy-humid areas. – **Au**: ?V, T, S, K, St. **Sw**: UR. **Fr**: AMa. **It**: Piem, VA.

***Protoparmelia montagnei* (Fr.) Poelt & Nimis**

Syn.: *Lecanora montagnei* (Fr.) Schaer., *Lecanora psarophana* Nyl., Lecanora psarophana Nyl. var. aquilina Clauzade & Cl. Roux, *Parmelia montagnei* Fr., *Protoparmelia psarophana* (Nyl.) Sancho & A. Crespo, *Solenopsora montagnei* (Fr.) M. Choisy & Werner

L – Subs.: sil – Alt.: 2 – Note: a Mediterranean-Macaronesian, chemically variable species of siliceous rocks, also reported from the limit of the Southern Pre-Alps (France). – **Fr**: Vau.

***Protoparmelia nephaea* (Sommerf.) R. Sant.**

Syn.: *Lecanora atrocincta* Th. Fr., *Lecanora nephaea* Sommerf.

L – Subs.: met, sil – Alt.: 4–5 – Note: a species with a thallus of scattered subumbilicate areoles developing on a distinct black prothallus, usually sterile, but with dark blue thalloconidia on the lower side of the free margin of the areoles, containing substances of the stictic acid syndrome; on vertical to overhanging rock faces of metal-rich siliceous rocks; overall distribution arctic-alpine to boreal-montane, with a few records from the Eastern Alps, but probably overlooked elsewhere. – **Au**: S, K, St. **Ge**: Ge.

***Protoparmelia ochrococca* (Nyl.) P.M. Jørg., Rambold & Hertel**

Syn.: *Lecidea ochrococca* Nyl.

L – Subs.: cor, xyl – Alt.: 1–2 – Note: a species with an ochraceous to chestnut brown thallus consisting of rounded to subglobose areoles, apothecia with red-brown discs, and minute, oblong to fusiform ascospores (to 10 µm long); a mainly western, epiphytic to lignicolous species, in the Alps only reported from South Tyrol. – **It**: TAA.

***Protoparmelia oleagina* (Harm.) Coppins**

Syn.: ?*Lecanora furva* H. Magn., *Lecanora oleagina* Harm.

L – Subs.: xyl, cor – Alt.: 2–5 – Note: mostly on lignum, more rarely on acid bark in damp deciduous forests. – **Au**: T, S, K, St, O. **Ge**: OB, Schw. **Fr**: AHP, AMa. **It**: TAA, Lomb.

***Protoparmelia olivascens* (Nyl.) Llimona**

Syn.: *Lecanora olivascens* Nyl.

L – Subs.: sil – Alt.: 1–2 – Note: a silicicolous species resembling *P. montagnei*, but with effigurate margins, and medulla reacting K+ yellow, then red; apparently more widespread in SW Europe, with a few records from the Western Alps (France). – **Fr**: AMa, Var.

***Protoparmelia phaeonesos* Poelt**

L – Subs.: sil-par – Alt.: 4–5 – Note: parasitic on *Aspilidea myrinii*, on acid siliceous rocks near and especially above treeline; probably much more widespread in the Alps. The species does not belong to *Protoparmelia* *s.str.* – **Au**: T, S, K, St, N. **It**: Frl, TAA.

***Protoparmelia placentiformis* (J. Steiner) Poelt**

Syn.: *Lecanora placentiformis* J. Steiner

L – Subs.: sil – Alt.: 3–4 – Note: on sunny siliceous rock in dry areas; described from arid parts of Asia, after the record from Macedonia, the record from the Ligurian Alps is the second one from Europe. This characteristic lichen probably does not belong to *Protoparmelia* *s.str.* – **It**: Lig.

***Protoparmeliopsis achariana* (A.L. Sm.) Moberg & R. Sant.**

Syn.: *Lecanora achariana* A.L. Sm., *Lichen cartilagineus* *sensu* Ach., *Placolecanora achariana* (A.L. Sm.) Kopach.

L – Subs.: sil – Alt.: 3–4 – Note: on exposed siliceous rocks, usually at the top of large boulders, sometimes overgrowing bryophytes; in the study area so far known only from the Western Alps (France). – **Fr**: AMa, Isè, HSav.

***Protoparmeliopsis admontensis* (Zahlbr.) Hafellner**

Syn.: *Lecanora admontensis* Zahlbr., *Lecanora luridescens* Zahlbr.

L – Subs.: cal – Alt.: 3–5 – Note: on steeply inclined to underhanging surfaces of calciferous rocks in rather dry upland areas with a rather continental climate. – **Au**: T, K, St, N. **Sw**: LU. **Fr**: AMa. **It**: Ven, TAA, Lig. **Sl**: SlA.

***Protoparmeliopsis bolcana* (Pollini) Lumbsch**

Syn.: *Lecanora bolcana* (Pollini) Poelt, Lecanora muralis (Schreb.) Rabenh. subsp. bolcana (Pollini) Clauzade & Cl. Roux, *Lecidea bolcana* Pollini

L – Subs.: sil – Alt.: 1–4 – Note: ecologically similar to *P. muralis*, but more restricted to natural habitats, on less basic substrata, and with optimum in the Mediterranean belt; apparently more frequent in the Western and Southern Alps. – **Fr**: AHP, HAl, AMa, Vau. **It**: Ven, Lomb, Piem, Lig.

***Protoparmeliopsis garovaglii* (Körb.) Arup, Zhao Xin & Lumbsch**

Syn.: *Lecanora cascadensis* H. Magn., *Lecanora garovaglii* (Körb.) Zahlbr., *Lecanora nevadensis* H. Magn., *Placodium garovaglii* Körb., *Placodium peruvianum* Müll. Arg., *Squamaria garovaglii* (Körb.) Anzi

L – Subs.: sil – Alt.: 2–6 – Note: a circumpolar, arctic-alpine to boreal-montane species found on basic siliceous rocks, especially in warm-dry valleys at low altitudes. – **Au**: T, S, K, St. **Sw**: BE, GR, VS. **Fr**: AHP, HAl, Isè, Sav, HSav. **It**: Ven, TAA, Lomb, Piem, VA.

***Protoparmeliopsis graeca* (J. Steiner) Sipman & Cl. Roux**

Syn.: *Lecanora graeca* J. Steiner

L # – Subs.: cal, int – Alt.: 3–4 – Note: hitherto known only from the eastern Mediterranean, the Maritime Alps of France, and the Central Apennines, this lichen occurring on base-rich or slightly calciferous siliceous rocks is worthy of further study. – **Fr**: AMa.

***Protoparmeliopsis laatokkensis* (Räsänen) Moberg & R. Sant.**

Syn.: *Lecanora degener* Poelt *ex* Clauzade & Rondon, *Lecanora laatokkensis* (Räsänen) Poelt, *Parmularia laatokkensis* Räsänen

L – Subs.: sil – Alt.: 2–5 – Note: on schist, serpentine, amphibolite, mostly on horizontal faces near the ground, at least when young, parasitic on other crustose lichens. – **Au**: ?V. **Fr**: AHP, HAl, AMa, Sav. **It**: TAA, Lig.

***Protoparmeliopsis macrocyclos* (H. Magn.) Moberg & R. Sant.**

Syn.: *Lecanora macrocyclos* (H. Magn.) Degel., Lecanora muralis (Schreb.) Rabenh. subsp. macrocyclos (H. Magn.) Clauzade & Cl. Roux, Lecanora muralis (Schreb.) Rabenh. var. macrocyclos H. Magn.

L # – Subs.: sax – Alt.: 4–5 – Note: an arctic-alpine, silicicolous member of the difficult *P. muralis*-complex, which needs further study. – **It**: Ven.

**Protoparmeliopsis muralis (Schreb.) M. Choisy var. muralis**

Syn.: Lecanora muralis (Schreb.) Rabenh. var. muralis, *Lecanora saxicola* (Pollich) Ach., *Lichen muralis* Schreb.

L – Subs.: sil, int, cal, bry, xyl, cor – Alt.: 1–5 – Note: a widespread, polymorphic, holarctic lichen found on siliceous and calcareous rocks, roofing tiles, brick, also occurring inside large conurbations; widespread throughout the Alps, most common below treeline. – **Au**: V, T, S, K, St, O, N, B. **Ge**: OB, Schw. **Sw**: BE, GR, LU, SZ, TI, UR, UW, VD, VS. **Fr**: AHP, HAl, AMa, Isè, Sav, HSav, Var, Vau. **It**: Frl, Ven, TAA, Lomb, Piem, VA, Lig. **Sl**: SlA. **Li**.

**Protoparmeliopsis muralis (Schreb.) M. Choisy var. diffracta (Ach.) M. Choisy *ex* Werner**

Syn.: *Lecanora diffracta* (Ach.) Ach., Lecanora muralis (Schreb.) Rabenh. var. diffracta (Ach.) Rabenh., *Lichen diffractus* Ach.

L # – Subs.: sil – Alt.: 2–5 – Note: a variety differing from typical *P. muralis* in having a black hypothallus visible between the areolae, not distinguished from *P. bolcana* by some authors, merged with *P. muralis* by others; on siliceous rocks in open situations, apparently widespread in Europe but not common; the distribution gaps in the Alps are probably virtual for the reasons given above. – **Fr**: AHP, HAl, AMa, Isè, HSav, Var.

**Protoparmeliopsis muralis (Schreb.) M. Choisy var. dubyi (Müll. Arg.) Hafellner & Türk**

Syn.: *Lecanora dubyi* Müll. Arg., Lecanora muralis (Schreb.) Rabenh. var. dubyi (Müll. Arg.) Poelt

L – Subs.: sil, int – Alt.: 2–5 – Note: on weakly calciferous or basic siliceous rocks in upland areas, with optimum near and above treeline. – **Au**: V, T, K. **Ge**: OB. **Fr**: HAl, AMa, Sav, HSav. **It**: Frl, TAA, Piem, VA.

**Protoparmeliopsis muralis (Schreb.) M. Choisy var. schneebergensis (Zahlbr.) Hafellner & Türk**

Syn.: Lecanora muralis (Schreb.) Rabenh var. schneebergensis Zahlbr.

L # – Subs.: cal – Alt.: 4–5 – Note: a variety with short, whitish-grey marginal lobes which are pruinose when young, and a verruculose-areolate thallus center; on exposed rock heads and boulders of calcareous rocks; not always distinguished and distribution therefore poorly known. – **Au**: St, N.

**Protoparmeliopsis muralis (Schreb.) M. Choisy var. subcartilaginea (A. Massal. *ex* Poelt) ined. (provisionally placed here, ICN Art. 36.1b)**

Syn.: Lecanora muralis (Schreb.) Rabenh. var. subcartilaginea A. Massal. *ex* Poelt

L # – Subs.: sil – Alt.: 4–5 – Note: on horizontal to weakly inclined surfaces of siliceous rocks in upland areas. – **Fr**: AHP, AMa. **It**: Frl, Ven, Piem.

***Protoparmeliopsis peltata* (Ramond) Arup, Zhao Xin & Lumbsch**

Syn.: *Lecanora peltata* (Ramond) Steud., *Lichen peltatus* Ramond, *Psoroma concinnum* Bagl. & Carestia, *Rhizoplaca peltata* (Ramond) Leuckert & Poelt

L – Subs.: sil – Alt.: 4–5 – Note: a chemically variable species also known from Africa, Asia, and North America, found on exposed siliceous rocks; apparently most common in the Western Alps. – **Fr**: AHP, HAl, AMa, Isè, Sav, HSav. **It**: Piem, VA.

***Protoparmeliopsis versicolor* (Pers.) M. Choisy**

Syn.: *Lecanora albomarginata* (Nyl.) Cromb., Lecanora muralis (Schreb.) Rabenh. subsp. versicolor (Pers.) Cl. Roux, Lecanora muralis (Schreb.) Rabenh. var. albomarginata (Nyl.) Tomin, Lecanora muralis (Schreb.) Rabenh. var. albopulverulenta (Schaer.) Rabenh., Lecanora muralis (Schreb.) Rabenh. var. versicolor (Pers.) Tuck., *Lichen versicolor* Pers.

L – Subs.: cal – Alt.: 2–5 – Note: this lichen has been often treated just as a calcicolous morph of *P. muralis*, and hence it has not been always distinguished in the recent lichenological literature. However, since it is easily recognisable, and above all because, contrary to *P. muralis*, it is parasitised by *Placocarpus schaereri*, we treat it separately, using the rank of species only because a combination into *Protoparmeliopsis* was already available. Its status could be solved only by a thorough molecular analysis of the whole complex in Europe. – **Au**: ?V, ?T. **Fr**: AHP, HAl, AMa, Drô, Isè, Sav, HSav, Var, Vau. **It**: Frl, Ven, TAA, Lomb, Piem, VA.

***Protothelenella corrosa* (Körb.) H. Mayrhofer & Poelt**

Syn.: *Acrorixis corrosa* (Körb.) Trevis., *Limboria corrosa* Körb., *Microglaena corrosa* (Körb.) Arnold, *Microglaena gibbosula* (Nyl.) Blomb. & Forssell, *Microglaena nericiensis* Hellb., *Polyblastia arenaria* (Hampe) Jatta, *Thelenella corrosa* (Körb.) Vain.

L – Subs.: sil – Alt.: 3–5 – Note: an arctic-alpine to boreal-montane, probably circumpolar lichen found on slightly calciferous rocks, especially near creeks and lakes, on boulders and pebbles near the ground; much overlooked and certainly more widespread in the Alps, with optimum above treeline. – **Au**: V, T, S, K, St. **Sw**: GR. **It**: TAA, Piem.

***Protothelenella leucothelia* (Nyl.) H. Mayrhofer & Poelt**

Syn.: *Dactyloblastus leucothelius* (Nyl.) Anzi, *Microglaena leucothelia* (Nyl.) Arnold, *Verrucaria leucothelia* Nyl.

L – Subs.: ter-sil, bry, deb, xyl – Alt.: 4–5 – Note: on soil, moribund bryophytes, plant debris and lichens (*Cladonia*), sometimes on rotting wood; probably ranging throughout the Alps, but overlooked. – **Au**: T, K. **Sw**: GR, SG, UR. **It**: Ven, TAA, Lomb.

***Protothelenella petri* H. Mayrhofer & Poelt**

L – Subs.: bry – Alt.: 3–5 – Note: a species with an inconspicuous thallus, minute perithecia (less than 200 µm in diam.), and ellipsoid muriform ascospores with usually 5 transversal septa and 1–2 longitudinal septa (usually less than 25 µm long); on the upper side of leaflets of *Polytrichum*, easily overlooked when the moss is dry and the leaflets enrolled; overall distribution arctic-alpine to boreal-montane, but apparently rare, with a few scattered records throughout the Alps. – **Au**: T, S. **Ge**: OB. **Sw**: GR, VS.

***Protothelenella sphinctrinoidella* (Nyl.) H. Mayrhofer & Poelt**

Syn.: *Gloeopyrenia reducta* (Th. Fr.) Zschacke, *Microglaena coenosa* (Vain.) Zahlbr., *Microglaena geoctona* Hellb., *Microglaena reducta* (Th. Fr.) Hellb., *Microglaena sphinctrinoidella* (Nyl.) Norman, Microglaena sphinctrinoides (Nyl.) Lönnr. subsp. reducta Th. Fr., *Thelenella coenosa* Vain., *Thelenella reducta* (Th. Fr.) Vain., *Verrucaria sphinctrinoidella* Nyl.

L – Subs.: bry, deb, ter-sil, par – Alt.: 3–5 – Note: on acid to subneutral soil, moribund bryophytes and lichens, more rarely on decaying plants in upland areas, often in rather disturbed habitats, *e.g.* along mountain track sides; certainly more widespread in the Alps, but probably overlooked. – **Au**: V, T, S, K, St, O. **Ge**: OB, Schw. **Sw**: BE, GR, SZ, VS. **Fr**: HSav. **It**: TAA, Piem. **Sl**: SlA.

***Protothelenella sphinctrinoides* (Nyl.) H. Mayrhofer & Poelt**

Syn.: *Chromatochlamys sphinctrinoides* (Nyl.) Trevis., *Gloeopyrenia gelatinosa* (Zahlbr.) Zschacke, *Microglaena gelatinosa* Zahlbr., *Microglaena sphinctrinoides* (Nyl.) Lönnr., *Polyblastia sphinctrinoides* (Nyl.) Jatta, *Thelenella sphinctrinoides* (Nyl.) Vain., *Verrucaria sphinctrinoides* Nyl.

L – Subs.: bry, deb, ter-sil – Alt.: 3–5 – Note: an arctic-alpine to boreal-montane, circumpolar lichen found on moribund bryophytes growing on soil and rock, more rarely directly on soil, in rather disturbed sites (*e.g.* on mountain track sides) with a long snow cover, often in crevices or small depressions of the ground; much undercollected and probably ranging throughout the Alps, with optimum above treeline. – **Au**: V, T, S, K, St. **Sw**: GR, TI, UR, VS. **Fr**: HSav. **It**: Frl, TAA, Piem.

***Protothelenella xylina* H. Mayrhofer & Poelt**

L – Subs.: xyl – Alt.: 4 – Note: a species resembling *P. sphinctrinoidella* but with an inconspicuous endoxylic thallus, and broader ascospores (to *c.* 15 µm wide), often with 2 incomplete longitudinal septa; on wood of coniferous trees (*Pinus cembra*); rare in Central Europe, with a few records from the Alps (Switzerland, Austria). – **Au**: T, St. **Sw**: GR.

***Pseudephebe minuscula* (Nyl. *ex* Arnold) Brodo & D. Hawksw.**

Syn.: *Alectoria minuscula* (Nyl. *ex* Arnold) Degel., Imbricaria lanata (Neck.) Arnold var. minuscula Nyl. *ex* Arnold, *Parmelia minuscula* (Nyl. *ex* Arnold) Nyl.

L – Subs.: sil – Alt.: 4–6 – Note: an arctic-alpine, circumpolar species found on hard siliceous rocks (including pure quartz) in wind-exposed situations near and especially above treeline; often confused with *P. pubescens* in the older literature; widespread throughout the Alps wherever siliceous substrata are present. – **Au**: V, T, S, K, St. **Ge**: Ge. **Sw**: BE, GR, TI, UR, VS. **Fr**: AHP, HAl, AMa, HSav. **It**: TAA, Lomb, Piem.

***Pseudephebe pubescens* (L.) M. Choisy**

Syn.: *Alectoria intricans* (Vain.) Motyka, *Alectoria lanata* (Neck.) Nyl., *Alectoria lanea* (Ehrh. *ex* Hoffm.) Vain., *Alectoria pubescens* (L.) R. Howe, *Bryopogon pubescens* (L.) M. Choisy, *Cornicularia lanata* (Neck.) Ach., *Cornicularia pubescens* (L.) Ach., *Ephebe pubescens* (L.) Fr., *Lichen pubescens* L., *Parmelia pubescens* (L.) Vain.

L – Subs.: sil – Alt.: 3–6 – Note: an arctic-alpine to (more rarely) boreal-montane lichen which is ecologically similar to *P. minuscula*, but with a somehow broader altitudinal range; widespread throughout the Alps wherever siliceous substrata are present. – **Au**: V, T, S, K, St, N. **Ge**: Schw. **Sw**: BE, GR, LU, TI, UR, UW, VD, VS. **Fr**: AHP, HAl, AMa, Isè, Sav, HSav. **It**: Frl, TAA, Lomb, Piem, VA, Lig.

**Pseudevernia furfuracea (L.) Zopf var. furfuracea**

Syn.: *Borrera furfuracea* (L.) Ach., *Evernia furfuracea* (L.) W. Mann, *Lichen furfuraceus* L., *Parmelia furfuracea* (L.) Ach., *Parmelia soralifera* (Bitter) Lynge, *Physcia furfuracea* (L.) DC., *Pseudevernia soralifera* (Bitter) Zopf

L – Subs.: cor, xyl, deb, sil – Alt.: 2–5 – Note: a cool-temperate to boreal-montane lichen found on acid bark and lignum, occasionally also on siliceous rocks, with optimum in the montane and subalpine belts; widespread and common throughout the Alps. – **Au**: V, T, S, K, St, O, N, B. **Ge**: OB, Schw. **Sw**: AP, BE, FR, GL, GR, LU, SG, SZ, TI, UR, UW, VD, VS. **Fr**: AHP, HAl, AMa, Drô, Isè, Sav, HSav, Var, Vau. **It**: Frl, Ven, TAA, Lomb, Piem, VA, Lig. **Sl**: SlA, Tg. **Li**.

**Pseudevernia furfuracea (L.) Zopf var. ceratea (Ach.) D. Hawksw.**

Syn.: Evernia furfuracea (L.) W. Mann var. ceratea (Ach.) Opiz, *Evernia olivetorina* Zopf, *Parmelia ceratea* (Ach.) Sandst., Parmelia furfuracea (L.) Ach. var. ceratea Ach., Parmelia furfuracea (L.) Ach. var. olivetorina (Zopf) Zahlbr., *Parmelia olivetorina* (Zopf) Sandst., Pseudevernia furfuracea (L.) Zopf var. olivetorina (Zopf) Zopf, *Pseudevernia olivetorina* (Zopf) Zopf

L – Subs.: cor, xyl, sil – Alt.: 2–5 – Note: We prefer to maintain as a distinct taxon this chemical strain characterised by the presence of olivetoric acid, because there is evidence of ecological and even physiological differences with respect to the typical variety (e.g. var. ceratea is more frequent in dry-continental areas, see Nimis 2016). – **Au**: V, T, S, K, St, O, N. **Ge**: OB. **Sw**: SZ, TI. **Fr**: AHP, HAl, AMa, Isè, Sav, HSav, Var, Vau. **It**: Frl, Ven, TAA, Lomb, Piem, VA, Lig. **Sl**: SlA.

***Pseudoleptogium diffractum* (Kremp. *ex* Körb.) Müll. Arg.**

Syn.: *Leptogium diffractum* Kremp. *ex* Körb., *Leptogium placodiellum* Nyl.

L – Subs.: cal – Alt.: 1–3 – Note: a mainly temperate to Mediterranean species found on steeply inclined seepage tracks of hard calcareous rocks; apparently most frequent in the Western and Southern Alps. – **Au**: S. **Ge**: Schw. **Fr**: AHP, AMa, Drô, Sav, HSav, Var, Vau. **It**: Frl, TAA, Piem. **Sl**: SlA.

***Pseudosagedia aenea* (Körb.) Hafellner & Kalb**

Syn.: *Porina aenea* (Körb.) Zahlbr., *Porina carpinea* (Pers. *ex* Ach.) Zahlbr., Porina chlorotica (Ach.) Müll. Arg. var. carpinea (Pers. *ex* Ach.) Keissl., *Pyrenula carpinea* (Pers. *ex* Ach.) Trevis., *Sagedia abietina* Körb., *Sagedia aenea* Körb., *Sagedia carpinea* (Pers. *ex* Ach.) A. Massal., *Sagedia chloromelaena* A. Massal., *Sagedia decipiens* A. Massal., *Sagedia erumpens* A. Massal., *Spermatodium aeneum* (Körb.) Trevis., *Spermatodium carpineum* (Pers. *ex* Ach.) Trevis., *Spermatodium chloromelaenum* (A. Massal.) Trevis., *Spermatodium erumpens* (A. Massal.) Trevis., *Trichothelium aeneum* (Körb.) R.C. Harris, *Verrucaria aenea* Wallr. *nom.illeg.*, *Verrucaria carpinea* Pers. *ex* Ach., *Verrucaria erumpens* (A. Massal.) Garov.

L – Subs.: cor – Alt.: 1–4 – Note: a mainly temperate to Mediterranean-Atlantic species found on smooth bark of broad-leaved deciduous and evergreen trees and shrubs, mostly in woodlands and forests. The species might belong to *Trichothelium.* – **Au**: T, S, K, St, O, N, B. **Ge**: OB, Schw. **Sw**: AP, BE, FR, GL, GR, LU, SG, SZ, TI, UR, UW, VD, VS. **Fr**: AHP, HAl, AMa, Drô, Isè, HSav, Var, Vau. **It**: Frl, Ven, TAA, Lomb, Piem. **Sl**: SlA, Tg.

***Pseudosagedia austriaca* (Körb.) Hafellner**

Syn.: *Porina austriaca* (Körb.) Arnold, *Sagedia austriaca* Körb.

L # – Subs.: sil – Alt.: 3–5 – Note: a species of shaded and humid surfaces of siliceous rocks near or above treeline, which needs further study. – **Au**: S, O, N. **Ge**: OB. **It**: TAA.

***Pseudosagedia borreri* (Trevis.) Hafellner & Kalb**

Syn.: *Porina borreri* (Trevis.) D. Hawksw. & P. James, Porina borreri (Trevis.) D. Hawksw. & P. James var. leptospora (Nyl.) D. Hawksw., *Porina leptospora* (Nyl.) A.L. Sm., *Spermatodium borreri* Trevis.

L – Subs.: cor – Alt.: 2–3 – Note: a mild-temperate lichen found on bark of deciduous trees in moist forests; probably more widespread in the Alps, but certainly not common. – **Au**: K. **Fr**: AMa, Isè, HSav, Var. **It**: Frl, Lig. **Sl**: SlA.

***Pseudosagedia byssophila* (Körb. *ex* Hepp) Hafellner & Kalb**

Syn.: *Porina byssophila* (Körb. *ex* Hepp) Zahlbr., *Sagedia byssophila* Körb. *ex* Hepp, *Spermatodium cinereorufescens* Trevis.

L – Subs.: cal – Alt.: 1–5 – Note: a mild-temperate to humid subtropical species of calcareous rocks in damp and shaded habitats, *e.g.* in forests; somehow rarer than the closely related *P. linearis*. The species might belong to *Trichothelium.* – **Au**: T, K, St, N. **Sw**: GR, LU, SZ. **Fr**: AHP, AMa, Isè, HSav, Var, Vau. **It**: TAA. **Sl**: Tg.

***Pseudosagedia chlorotica* (Ach.) Hafellner & Kalb**

Syn.: *Porina chlorotella* (Nyl.) Zahlbr., *Porina chlorotica* (Ach.) Müll. Arg., *Porina tenuifera* (Nyl.) A.L. Sm., *Pyrenula chlorotica* (Ach.) Trevis., *Sagedia athallina* Bagl. & Carestia, *Sagedia chlorotica* (Ach.) A. Massal., *Trichothelium chloroticum* (Ach.) R.C. Harris, *Verrucaria chlorotica* Ach.

L – Subs.: sil, sil-aqu, cor, ter – Alt.: 1–5 – Note: a temperate to humid subtropical, probably holarctic species found on siliceous pebbles in humid-shaded situations, mostly in deciduous forests. The species might belong to *Trichothelium.* – **Au**: ?V, T, S, K, St, O, N, B. **Sw**: BE, UR, VD, VS. **Fr**: AHP, AMa, HSav, Var. **It**: Frl, Ven, TAA, Lomb, Piem, Lig. **Sl**: SlA.

***Pseudosagedia globulans* (Vain.) Hafellner**

Syn.: *Porina globulans* Vain.

L # – Subs.: sil – Alt.: 5 – Note: resembling *P. chlorotica* (a lowland species) in the 3-septate ascospores, but with globose perithecia; the value of this silicicolous species, based on type from Finland, is in need of re-evaluation; there are scattered records throughout Europe, including the Eastern Alps (Austria). – **Au**: St.

***Pseudosagedia guentheri* (Flot.) Hafellner & Kalb**

Syn.: *Porina guentheri* (Flot.) Zahlbr., *Porina koerberi* (Flot.) Lettau, *Trichothelium guentheri* (Flot.) R.C. Harris, *Verrucaria guentheri* Flot.

L – Subs.: sil, sil-aqu – Alt.: 2–5 – Note: a cool-temperate to subarctic lichen found along mountain creeks in the montane and subalpine belts, on periodically inundated siliceous rocks, but also on very shaded, not inundated rocks near the ground; probably more widespread in the Alps. – **Au**: T, K, St, N. **Fr**: AMa. **It**: Lomb, Piem. **Sl**: SlA.

***Pseudosagedia linearis* (Leight.) Hafellner & Kalb**

Syn.: Porina chlorotica (Ach.) Müll. Arg. var. linearis (Leight.) A.L. Sm., Porina chlorotica (Ach.) Müll. Arg. var. persicina (Körb.) A.L. Sm., *Porina linearis* (Leight.) Zahlbr., *Porina persicina* (Körb.) Zahlbr., *Sagedia harrimannii* A. Massal., *Sagedia persicina* Körb., *Spermatodium lineare* (Leight.) Trevis., *Trichothelium lineare* (Leight.) R.C. Harris, Verrucaria chlorotica Ach. subsp. leucotica Nyl., *Verrucaria gibelliana* Garov., *Verrucaria linearis* Leight., *Verrucaria ricasolii* Garov.

L – Subs.: cal – Alt.: 1–5 – Note: a mainly mild-temperate species found on limestone in sheltered situations, mostly near the ground, often with *Acrocordia conoidea*. – **Au**: V, K, O, N. **Ge**: Ge. **Sw**: BE, GR. **Fr**: AHP, AMa, Drô, HSav, Var, Vau. **It**: Frl, Ven, TAA, Lomb, Piem.

***Pseudosagedia lucens* (Taylor) Hafellner**

Syn.: Porina guentheri (Flot.) Zahlbr. var. lucens (Taylor) Swinscow, *Porina lucens* (Taylor) A.L. Sm., *Verrucaria lucens* Taylor

L – Subs.: sil, sil-aqu – Alt.: 2–3 – Note: a species probably closely related and similar in habitus to *P. guentheri*, but ascospores partly (up to 25 %) submuriform with usually 7 transversal septa and 1 longitudinal septum in one of the central cells; on moist siliceous rocks near lakes and streams at low elevations; widespread in SW Europe but rare, being most common on the British Isles; reported also from the base of the Western Alps (France). – **Fr**: AMa.

***Pseudosagedia obsoleta* (Oxner) Hafellner & Kalb**

Syn.: *Phylloporina obsoleta* Oxner, *Porina oxneri* R. Sant.

L – Subs.: fol – Alt.: 2 – Note: an obligately foliicolous lichen confined to warm-moist forests, on needles of *Abies*, leaves of evergreen trees and shrubs (*e.g. Buxus*), and cladodes of *Ruscus*; in the study area so far known only from the Western Alps. – **Fr**: AHP, AMa, Isè, HSav, Var, Vau.

***Pseudosagedia oleriana* (A. Massal.) Hafellner & Kalb**

Syn.: *Porina oleriana* (A. Massal.) Lettau, *Sagedia oleriana* A. Massal.

L – Subs.: cal – Alt.: 1–2 – Note: on shaded surfaces of calcareous rocks, often with *Acrocordia conoidea*, but less confined to deep shade; in the study area so far known from the Western and Southern Alps. – **Fr**: Var, Vau. **It**: Ven, Lomb.

***Pseudosagedia provincialis* (Clauzade & Cl. Roux) ined. (provisionally placed here, ICN Art. 36.1b)**

Syn.: Porina oleriana (A. Massal.) Lettau var. provincialis Clauzade & Cl. Roux, *Porina provincialis* (Clauzade & Cl. Roux) Cl. Roux

L – Subs.: cal – Alt.: 2 – Note: on shaded surfaces of calcareous or dolomitic rocks in lowland areas; in the study area so far known from the Western Alps. – **Fr**: AHP, AMa, Var, Vau.

***Pseudoschismatomma rufescens* (Pers.) Ertz & Tehler**

Syn.: *Opegrapha contexta* Stirt., *Opegrapha herpetica* (Ach.) Ach., Opegrapha herpetica (Ach.) Ach. var. subocellata Ach., *Opegrapha lilacina* A. Massal., *Opegrapha rubecula* A. Massal., *Opegrapha rubella* Pers., *Opegrapha rufescens* Pers., *Opegrapha siderella* (Ach.) Ach., *Opegrapha subocellata* (Ach.) Hepp

L – Subs.: cor – Alt.: 1–3 – Note: a mainly temperate, widespread lichen found on the smooth bark of deciduous trees, especially in woodlands near creeks and rivers in humid valleys; widespread throughout the Alps. – **Au**: V, T, S, K, St, O, N, B. **Ge**: OB. **Sw**: AP, BE, FR, GL, GR, LU, SG, SZ, TI, UR, UW, VD, VS. **Fr**: AMa, Drô, Isè, Sav, HSav, Var, Vau. **It**: Frl, Ven, TAA, Lomb, Piem. **Sl**: SlA, Tg. **Li**.

***Pseudothelomma ocellatum* (Körb.) M. Prieto & Wedin**

Syn.: *Acolium ocellatum* Körb., *Cyphelium caliciforme* (Flot.) Zahlbr., *Cyphelium ocellatum* (Körb.) Trevis., *Thelomma ocellatum* (Körb.) Tibell

L – Subs.: xyl, cor – Alt.: 3–5 – Note: a circumboreal-montane species found on hard rotting wood, *e.g.* on poles and fences, more rarely on *Larix* and *Pinus cembra* in the subalpine belt; certainly more widespread in the Alps, especially in subcontinental areas, but overlooked, being mostly sterile. This is one of the few calicioid fungi that reproduce via lichenised diaspores. – **Au**: V, T, S, K, St, O, N. **Ge**: OB. **Sw**: GR, LU, SZ, VS. **Fr**: HSav. **It**: TAA, Lomb, VA. **Sl**: SlA.

***Psilolechia clavulifera* (Nyl.) Coppins**

Syn.: *Lecidea clavulifera* Nyl., *Micarea clavulifera* (Nyl.) Coppins & P. James

L – Subs.: sil, ter-sil, cor – Alt.: 2–3 – Note: on acid siliceous stones and consolidated soil of dry underhangs, on banks or on the roots of fallen trees; perhaps overlooked and more widespread in the Alps, but certainly rare. – **Au**: T.

***Psilolechia lucida* (Ach.) M. Choisy**

Syn.: *Biatora lucida* (Ach.) Fr., *Lecidea lucida* (Ach.) Ach., *Lichen lucidus* Ach.

L – Subs.: sil, xyl, deb – Alt.: 1–5 – Note: in underhangs of siliceous rocks protected from rain in humid areas, but also on a wide range of substrata (soil, exposed roots, bases of ancient trees), with a correspondingly wide altitudinal range. – **Au**: V, T, S, K, St, O, N. **Ge**: Schw. **Sw**: BE, GR, UR, VD, VS. **Fr**: AMa, Isè, HSav, Var, Vau. **It**: Frl, Ven, TAA, Lomb, Piem, VA. **Sl**: SlA.

***Psora decipiens* (Hedw.) Hoffm.**

Syn.: *Biatora decipien*s (Hedw.) Fr., *Lecanora decipiens* (Hedw.) Ach., *Lecidea decipiens* (Hedw.) Ach., *Lichen decipiens* Hedw.

L – Subs.: ter-cal – Alt.: 1–6 – Note: a widespread holarctic species with a broad altitudinal and latitudinal range, found on bare calciferous soil, especially in dry grasslands; rare only in areas with intensive grazing, high trampling, and intense disturbance. The wide ecological amplitude could be due to the capacity of associating with several different species of *Trebouxia* and *Asterochloris*; widespread throughout the Alps. – **Au**: V, T, S, K, St, O, N. **Ge**: OB, Schw. **Sw**: BE, GR, LU, SZ, TI, UR, UW, VD, VS. **Fr**: AHP, HAl, AMa, Drô, Isè, Sav, HSav, Var, Vau. **It**: Frl, Ven, TAA, Lomb, Piem, VA, Lig. **Sl**: SlA. **Li**.

***Psora globifera* (Ach.) A. Massal.**

Syn.: *Lecidea globifera* Ach.

L – Subs.: cal, int, ter-cal, ter-sil, ter-int – Alt.: 3–5 – Note: on slightly calciferous or base-rich soil and weathered siliceous rocks in upland areas. – **Au**: V, T, S, K, St. **Ge**: OB. **Sw**: BE, GR, SZ, TI, VS. **Fr**: AHP, HAl, AMa, Sav, HSav. **It**: Ven, TAA, Lomb, Piem, VA.

***Psora gresinonis* B. de Lesd.**

Syn.: *Lecidea gresinonis* (B. de Lesd.) Zahlbr.

L – Subs.: ter – Alt.: 1–2 – Note: on soil, in fissures of base-rich or slightly calciferous siliceous rocks, with optimum in dry grasslands; chemically heterogeneous (with and without norstictic acid); in the study area known only from the base of the Western Alps. – **Fr**: Drô, Vau. **It**: Lig.

***Psora rubiformis* (Ach.) Hook.**

Syn.: *Baeomyces rubiformis* Ach., *Lecidea rubiformis* (Ach.) Wahlenb.

L – Subs.: cal, int, ter-cal – Alt.: 4–5 – Note: an arctic-alpine species found on loess and calciferous soil, in fissures of calciferous siliceous rocks (*e.g.* calciferous schists); chemically heterogeneous (with and without gyrophoric acid). – **Au**: T, S, K, N. **Fr**: HAl, AMa. **It**: Lomb, Piem, VA.

***Psora sessitana* (Bagl. & Carestia) Bagl. & Carestia**

Syn.: *Biatora sessitana* (Bagl. & Carestia) Jatta, *Lecidea sessitana* Bagl. & Carestia

L # – Subs.: sil – Alt.: 4–5 – Note: a species with a distinctly squamulose thallus, the squamules thick, densely imbricate, brown above, white beneath, and large, rounded to lobed apothecia with a flat, black, white-pruinose disc, a dark greenish epithecium, a colourless hypothecium. rather thick, conglutinated paraphyses with a thicker apex, and small, elliptical-elongate, hyaline ascospores which are *c.* 2 times as long as wide; known only from the type collection, on granite. It is not certain that the species belongs to *Psora* *s.str.* (see Nimis 1993: 583). – **It**: Piem.

***Psora testacea* Hoffm.**

Syn.: *Biatora testacea* (Hoffm.) W. Mann, *Chrysopsora testacea* (Hoffm.) M. Choisy, *Lecanora testacea* (Hoffm.) Ach., *Lecidea testacea* (Hoffm.) Ach., *Protoblastenia testacea* (Hoffm.) Clauzade & Rondon

L – Subs.: cal, ter-cal – Alt.: 2–5 – Note: optimum in fissures of base-rich or lime-containing metamorphic rocks, most frequent in dry valleys of the Alps, but rarely reaching beyond treeline. – **Au**: V, T, K, St, O, N. **Sw**: BE, GR, VD, VS. **Fr**: AHP, AMa, Isè, HSav, Var, Vau. **It**: Ven, TAA, Lomb, Piem, VA, Lig.

***Psora vallesiaca* (Schaer.) Timdal**

Syn.: *Lecidea albilabraauct.*, *Lecidea deceptoria* Nyl., *Lecidea vallesiaca* Schaer., *Psora albilabra auct. non* (Dufour) Körb., *Psora deceptoria* (Nyl.) Flagey, *Psora subdecipiens* (Nyl.) Flagey, *Squamaria deceptoria* (Nyl.) M. Choisy & Werner

L – Subs.: cal, ter-cal – Alt.: 1–4 – Note: on bare soil and in fissures of rocks, not rare where suitable habitats are present (subcontinental conditions and base-rich, slightly calciferous siliceous substrata). – **Au**: T, St. **Sw**: VS. **Fr**: AHP, AMa, Var, Vau. **It**: Ven, Piem, VA, Lig.

***Psorinia conglomerata* (Ach.) Gotth. Schneid.**

Syn.: *Lecidea conglomerata* Ach., *Lecidea glomerans* Nyl., *Lecidea rugifera* Vain., *Lecidea silenii* Vain., *Psora conglomerata* (Ach.) Flot., *Thalloidima conglomeratum* (Ach.) A. Massal., *Toninia conglomerata* (Ach.) Boistel, *Toninia glomerans* (Nyl.) Boistel

L – Subs.: sil – Alt.: 3–6 – Note: a mainly arctic-alpine, circumpolar species found on steeply inclined to underhanging surfaces of acidic to slightly basic siliceous rocks, often in fissures and cracks; most frequent above treeline. – **Au**: V, T, S, K, St, N. **Ge**: Schw. **Sw**: BE, GR, TI, UR, VS. **Fr**: HAl, AMa, Sav, HSav. **It**: Frl, Ven, TAA, Lomb, Piem, VA.

***Psoroglaena abscondita* (Coppins & Vězda) Hafellner & Türk**

Syn.: *Leucocarpia abscondita* (Coppins & Vězda) Hafellner, *Macentina abscondita* Coppins & Vězda

L – Subs.: cor – Alt.: 2–3 – Note: optimum on the bark of *Sambucus* in shaded-humid situations; reported from scattered localities in the Eastern Alps (Austria, Italy). – **Au**: St, O, B. **It**: Ven.

***Psoroglaena biatorella* (Arnold) Lücking & Sérus.**

Syn.: *Leucocarpia biatorella* (Arnold) Vězda, *Microglaena biatorella* Arnold

L – Subs.: bry, deb, ter-cal – Alt.: 3–5 – Note: an inconspicuous lichen found on thin layers of more or less calciferous, humus-rich ground, or over epilithic mosses, mostly in upland areas; probably overlooked and more widespread, but never common in the Alps. – **Au**: V, T, S, K, St, O, N. **Ge**: OB. **It**: Frl.

***Psoroglaena dictyospora* (Orange) H. Harada**

Syn.: *Leucocarpia dictyospora* (Orange) R. Sant., *Macentina dictyospora* Orange

L – Subs.: deb, xyl – Alt.: 3 – Note: a species with a thin granular thallus consisting of goniocysts, partly immersed perithecioid ascomata, fissitunicate asci, and submuriform ascospores, usually with 5 transversal septa and an incomplete longitudinal septum; on soft wood in humid-shaded situations such as in riverine forests and in gorges; rare throughout temperate Europe and also known from North America, but perhaps with a broader distribution, being very easily overlooked; in the study area only recorded from the Western Alps (Switzerland). – **Sw**: UW.

***Psoroglaena stigonemoides* (Orange) Henssen**

Syn.: *Leucocarpia stigonemoides* (Orange) Hafellner & Kalb, *Macentina stigonemoides* Orange

L – Subs.: cor, fol – Alt.: 2–3 – Note: a humid subtropical to Mediterranean-Atlantic lichen mainly found on *Sambucus nigra* in humid-shaded situations, and on the leaves of *Buxus* in the undergrowth of moist-warm forests; certainly not common in the Alps, but probably also overlooked. – **Au**: S, K, St, O, N. **Fr**: AMa, Isè, Var, Vau. **It**: Frl.

***Psoroma hypnorum* (Vahl) Gray**

Syn.: *Lecanora femsjonensis* Fr., *Lecanora hypnorum* (Vahl) Ach., *Lichen hypnorum* Vahl, *Pannaria hypnorum* (Vahl) Körb., *Pannaria porriginosa* Vain., *Parmelia lepidora* Ach., *Psora deaurata* Hoffm., *Psoroma femsjonense* (Fr.) Trevis.

L – Subs.: ter-sil, ter-cal – Alt.: 3–6 – Note: an arctic-alpine to boreal-montane, circumpolar lichen found on soil, often in and amongst bryophytes, mostly over siliceous substrata in moist habitats; widespread throughout the Alps wherever siliceous substrata are present. – **Au**: V, T, S, K, St, O, N. **Ge**: OB. **Sw**: BE, GR, TI, UR, VS. **Fr**: AHP, HAl, AMa, Isè, Sav, HSav, Vau. **It**: Frl, Ven, TAA, Lomb, Piem, VA.

***Psoroma paleaceum* (Fr.) Timdal & Tønsberg**

Syn.: *Parmelia paleacea* Fr., *Psoroma hirsutulum* Nyl. *ex* Cromb., Psoroma hypnorum (Vahl) Gray var. paleaceum (Fr.) Rostr.

L – Subs.: ter-sil – Alt.: 5 – Note: a species with a granular to minutely squamulose thallus covered in scattered white hairs as the thalline margin of the cupulate apothecia (to 2 mm in diam.) (not to be confused with tomentose hairs in the lower part of the exciple as seen in morphs of *P. hypnorum*), with non-septate subglobose ascospores (15–20 × 10–14 μm); on moist soil, encrusting bryophytes and plant debris; overall distribution bipolar, but rare; from the Alps there is a single, historical record (Germany). – **Ge**: OB.

**Psoroma tenue Henssen var. boreale Henssen**

L – Subs.: ter-cal, ter-sil, ter-int, deb, bry – Alt.: 4–6 – Note: an arctic-alpine, circumpolar lichen weak in competition found on wet, naked soil, near glaciers or late snow-beds; certainly more widespread in the nival belt of the Alps, but easily confused with *P. hypnorum*. – **Au**: V, T, S, K, St, N. **Sw**: UR, VS. **Fr**: HAl, AMa, Sav. **It**: TAA, Lomb.

***Psoronactis dilleniana* (Ach.) Ertz & Tehler**

Syn.: *Lecanactis dilleniana* (Ach.) Körb., *Lichen dillenianus* Ach.

L – Subs.: sil – Alt.: 2–5 – Note: on hard crystalline rocks beneath underhangs and in crevices which are seldom wetted by rain, mostly in upland areas. – **Au**: V, T, S, K, St, N. **Sw**: VS. **Fr**: Isè. **It**: TAA, Piem.

***Psorotichia allobrogensis* Hue**

L # – Subs.: cal-aqu – Alt.: 2–3 – Note: a poorly understood species of steeply inclined surfaces of calcareous rocks with some water seepage after rain, mostly at relatively low elevations. – **Fr**: AHP, AMa, Sav, HSav.

***Psorotichia claudelii* Hue**

L # – Subs.: cal – Alt.: 3 – Note: a species in need of re-evaluation, resembling *P. schaereri*, but the areolae with a minutely granular surface and with a bluish-grey pruina, like the margins of the apothecia (finally exceeding 0.5 mm in diam); on calcareous rocks, ecology and distribution poorly documented, with a few records from the Western Alps (France). – **Fr**: AHP, Sav.

***Psorotichia diffracta* (Nyl.) Forssell**

Syn.: *Collema diffractum* Nyl., *Collemopsis diffracta* (Nyl.) Nyl. *ex* Lamy

L – Subs.: cal – Alt.: 2–3 – Note: on sun-exposed seepage tracks of calcareous rocks; overlooked, and perhaps more widespread in the Alps. – **Au**: K. **Sw**: BE. **Fr**: AHP, AMa, Var, Vau.

***Psorotichia frustulosa* Anzi**

Syn.: *Collemopsis frustulosa* (Anzi) Nyl.

L – Subs.: cal, sil – Alt.: 2–5 – Note: a species characterised by a thin, small-areolate thallus, sessile apothecia with a prominent thalline exciple, a conspicuous, thick, smooth thalline margin, and dark red apothecial discs, the asci are 8-spored; on steeply inclined, sunny surfaces of calcareous or basic siliceous rocks in upland areas; a rarely collected species, also known from Korea and North America. – **Au**: T, S, St. **It**: Lomb.

***Psorotichia fuliginascens* (Nyl.) Forssell**

Syn.: *Collemopsis fuliginascens* Nyl., *Porocyphus fuliginascens* (Nyl.) Couderc, *Pyrenopsis lignyota auct. non* (Wahlenb.) Th. Fr.

L # – Subs.: sil – Alt.: 4 – Note: a species perhaps related to *Thelignya lignyota* but with a black thallus of aggregated squamules which are less than 1 mm wide, and with innate, minute apothecia; the generic placement is in need of re-evaluation; based on a type from Finland where it was found on mica-schist, ecology and overall distribution poorly documented, with a record from the Western Alps (France). – **Fr**: HSav.

***Psorotichia gelatinosa* Anzi**

L # – Subs.: sil – Alt.: 2 – Note: a species with a thin, black, gelatinous thallus and innate apothecia with flesh-red discs, known only from the type material (on granitic outcrops in a deciduous lowland forest); the generic placement is unresolved. – **It**: Lomb.

***Psorotichia leprosa* (Anzi) Forssell**

Syn.: *Pyrenopsis leprosa* Anzi

L # – Subs.: cal – Alt.: 2 – Note: a species with a thin, black, subleprose thallus of small granules, very small, globose to urceolate, sessile apothecia with a brown, punctiform disc and a margin concolour with the thallus, a IKI+ intensely blue hymenium of lax paraphyses, and ovoid to ellipsoid, hyaline, simple ascospores measuring 10–15 × 6–7 µm. Described from material growing on steeply inclined surfaces of calciferous marl near Como (Italy); the type material is worthy of further study. – **It**: Lomb.

***Psorotichia lugubris* (A. Massal.) Arnold**

Syn.: *Stenhammara lugubris* A. Massal.

L # – Subs.: cal – Alt.: 3–4 – Note: perhaps related to *P. murorum*, but with a verruculose to squamulose thallus and inconspicuous apothecia which are first immersed, later prominent, and somewhat wider ascospores; on calcareous rocks; overall distribution poorly known, with a few scattered records from the Alps. – **Au**: V. **Sw**: BE.

***Psorotichia murorum* A. Massal.**

Syn.: *Collemopsis murorum* (A. Massal.) Stizenb.

L – Subs.: cal – Alt.: 1–4 – Note: on sunny surfaces of calcareous rocks, mostly below the montane belt. – **Au**: V, T, S, O, N. **Sw**: GR, SZ, TI, VS. **Fr**: HSav, Var. **It**: Ven, TAA, Lomb, Piem, Lig.

***Psorotichia pictava* (Nyl.) Forssell**

Syn.: *Collemopsis pictava* (Nyl.) Nyl., *Pyrenopsis pictava* Nyl.

L # – Subs.: cal – Alt.: 2 – Note: a species with a thin blackish thallus, reddish brown apothecia enclosed in thalline nodules, 8-spored asci, and subglobose ascospores (8–10 μm in diam.), found on calcareous rocks and walls in dry habitats; in the study area so far only recorded from the Western Alps (France). – **Fr**: AHP.

***Psorotichia pontresinae* B. de Lesd.**

L # – Subs.: sil-aqu – Alt.: 4 – Note: a species with a very thin, crustose, spreading grey (in the dry state) thallus, dispersed, red to purple, urceolate apothecia (0.4–0.6 mm in diam.) with entire, thick margins, a I+ blue hymenial gel, 8-spored asci, and broadly ellipsoid, hyaline, simple ascospores (16–21 × 9–13 μm); the generic placement is in need of re-evaluation; on submerged schistose boulders in streams at high elevation; only known from the type locality in the Western Alps (Switzerland). – **Sw**: GR.

***Psorotichia schaereri* (A. Massal.) Arnold**

Syn.: *Collemopsis caesia* Nyl., *Collemopsis schaereri* (A. Massal.) Cromb., *Pannaria schaereri* A. Massal., *Psorotichia caesia* (Nyl.) Forssell, *Pyrenopsis schaereri* (A. Massal.) Nyl., *Synalissa schaereri* (A. Massal.) Tuck., *Trachyderma schaereri* (A. Massal.) Trevis.

L – Subs.: cal, sil – Alt.: 1–4 – Note: on more or less shaded seepage tracks of limestone, dolomite, calcareous sandstone and schists, rarely on walls, with a wide altitudinal range, but not reaching beyond treeline; widespread throughout the Alps. – **Au**: ?V, T, S, K, St, N. **Ge**: OB. **Sw**: BE, SZ, UW. **Fr**: AHP, AMa, Isè, Sav, HSav, Var, Vau. **It**: Ven, TAA, Lomb, Piem, Lig.

***Psorotichia suffugiens* (Nyl.) Forssell**

Syn.: *Collemopsis suffugiens* Nyl., *Psorotichia diaphorotheca* Harm.

L # – Subs.: cal, int – Alt.: 2–4 – Note: a species whose generic placement is provisional, with an indistinct thallus and minute, blackish, *Lecanora*-like apothecia with thalline margins, polysporous asci (16–32) and *c.* 5 µm long ascospores; on surfaces of calcareous or basic siliceous rocks subject to short periods of water seepage; overall distribution poorly known, with a few scattered records from the Alps. – **Au**: K. **Fr**: AHP, HAl, AMa, Var.

***Psorotichia tiroliensis* Zahlbr.**

Syn.: *Porocyphus arnoldii* (Heufl. *ex* Arnold) Arnold, *Psorotichia arnoldii* Heufl. *ex* Arnold

L # – Subs.: cal, ?int – Alt.: 3–4 – Note: similar to *Porocyphus coccodes*, but with 16-spored asci; the generic placement is in need of re-evaluation, and the ecology is poorly documented as well; known from a few localities in the Alps. – **Au**: T. **It**: TAA.

***Psorotichia vermiculata* (Nyl.) Forssell**

Syn.: *Collemopsis vermiculata* Nyl.

L # – Subs.: cal – Alt.: 2 – Note: similar to *P. schaereri*, but with a more even and distinctly areolate thallus, and innate, red-brown apothecia; on calcareous rocks, perhaps a lowland species in areas with a moderately continental climate; overall distribution poorly known; in the study area it was recorded only from the Eastern Alps (Austria). – **Au**: N.

***Psorula rufonigra* (Tuck.) Gotth. Schneid.**

Syn.: *Biatora rufonigra* Tuck., *Lecidea rufonigra* (Tuck.) Nyl., *Psora rufonigra* (Tuck.) A. Schneid.

L – Subs.: sil – Alt.: 1–4 – Note: a widespread, mainly southern species of dry areas, found on sun-exposed, inclined to vertical seepage tracks of base-rich siliceous rocks, always associated with *Spilonema*; probably more widespread in the Alps, especially in dry-continental valleys. – **Au**: ?V, ?T. **It**: TAA.

***Pterygiopsis affinis* (A. Massal.) Henssen**

Syn.: *Enchylium affine* A. Massal., *Enchylium flageyi* Harm., *Enchylium rubbianum* A. Massal., *Forssellia affinis* (A. Massal.) Zahlbr., *Forssellia flageyi* (Harm.) Zahlbr., *Heppia purpurascens* (Nyl.) Nyl., *Lecanora purpurascens* Nyl.

L – Subs.: cal, sil – Alt.: 1–3 – Note: on periodically wetted surfaces of calcareous or siliceous rocks, especially along sun-exposed seepage tracks. – **Au**: S. **Fr**: AMa, Var, Vau. **It**: Frl, Ven, TAA, Piem.

***Pterygiopsis concordatula* (Nyl.) P.M. Jørg.**

Syn.: *Collemopsis coracodiza* Nyl., *Psorotichia coracodiza* (Nyl.) Forssell, *Pterygiopsis coracodiza* (Nyl.) Henssen, *Pyrenopsis concordatula* Nyl.

L – Subs.: sil-aqu – Alt.: 3–4 – Note: a species with a blackish-brown, furfuraceous, areolate thallus and initially immersed but later protruding apothecia with a distinct thalline margins; on frequently moistened siliceous rocks, *e.g.* along streams; more common in NW Europe, with a record from the Western Alps (France). – **Fr**: Sav.

***Punctelia borreri* (Sm.) Krog**

Syn.: *Imbricaria borreri* (Sm.) Körb., *Lichen borreri* Sm., *Parmelia borreri* (Sm.) Turner, *Parmelia pseudoborreri* Asahina

L – Subs.: cor – Alt.: 1–3 – Note: a mainly mild-temperate lichen found on more or less isolated, mostly deciduous trees. – **Au**: T, ?S. **Sw**: BE, SZ, TI, VD, VS. **Fr**: AHP, AMa, Drô, Isè, Var, Vau. **It**: Frl, Ven, TAA, Lomb, Piem, Lig. **Sl**: SlA.

***Punctelia jeckeri* (Roum.) Kalb**

Syn.: Parmelia borreri (Sm.) Turner var. ulophylla (Ach.) Nyl., Parmelia caperata (L.) Ach. var. ulophylla Ach., Parmelia dubia (Wulfen) Schaer. var. ulophylla (Ach.) Harm., *Parmelia ulophylla* (Ach.) F. Wilson, *Punctelia ulophylla* (Ach.) van Herk & Aptroot, *Sticta jeckeri* Roum.

L – Subs.: cor – Alt.: 1–3 – Note: on bark of isolated deciduous trees, ecologically intermediate between *Xanthorion* and *Parmelion*. European specimens might not be identical with North American material, and deserve further study. – **Au**: V, S, K, St, O, N. **Ge**: OB, Schw. **Sw**: BE, GR, SZ, TI, UR, UW, VS. **Fr**: Drô, Isè, HSav. **It**: Frl, Ven, TAA, Lomb, Lig.

***Punctelia perreticulata* (Räsänen) G. Wilh. & Ladd**

Syn.: Parmelia duboscqii Abbayes var. perreticulata Räsänen in Sbarbaro

L – Subs.: sil, cor – Alt.: 1 – Note: a mainly Mediterranean-Atlantic lichen found on siliceous rocks and bark, with a few records from the base of the Western Alps near Spotorno (Italy). – **It**: Lig.

***Punctelia stictica* (Duby) Krog**

Syn.: Parmelia borreri (Sm.) Turner var. stictica Duby, Parmelia dubia (Wulfen) Schaer. var. stictica (Duby) Schaer., *Parmelia stictica* (Duby) Nyl.

L – Subs.: sil, bry-sil – Alt.: 2–4 – Note: on siliceous rocks in open situations; probably more widespread in the Alps. – **Sw**: GR. **Fr**: Isè, Vau. **It**: TAA, Lomb.

***Punctelia subrudecta* (Nyl.) Krog**

Syn.: Parmelia borreri (Sm.) Turner var. subrudecta (Nyl.) Clauzade & Cl. Roux, *Parmelia dubia* (Wulfen) Schaer., *Parmelia helenae* B. de Lesd., *Parmelia maculatosorediosa* (Gyeln.) Gyeln., *Parmelia subrudecta* Nyl., *Punctelia helenae* (B. de Lesd.) Hale *ex* De Priest & B.W. Hale

L – Subs.: cor – Alt.: 1–3 – Note: a mainly temperate species found on bark of isolated deciduous trees, ecologically intermediate between *Xanthorion* and *Parmelion*, most common below the montane belt; widespread throughout the Alps. – **Au**: V, T, S, K, St, O, N, B. **Ge**: OB. **Sw**: BE, FR, GL, GR, LU, SG, SZ, TI, UR, UW, VD, VS. **Fr**: AHP, AMa, Drô, Isè, Sav, HSav, Var, Vau. **It**: Frl, Ven, TAA, Lomb, Piem, VA, Lig. **Sl**: SlA, Tg. **Li**.

***Puttea caesia* (Fr.) M. Svenss. & T. Sprib.**

Syn.: *Agyrium caesium* Fr., *Biatora symmictella* (Nyl) Arnold, *Lecanora symmictella* (Nyl.) Hafellner *non* Vain., *Lecidea symmictella* Nyl.

L – Subs.: cor, xyl – Alt.: 3–4 – Note: on hard lignum., *e.g.* on horizontal faces of old stumps, sometimes on mosses, mostly in upland areas; from the Alps there are several scattered records. – **Au**: V, N. **Ge**: OB. **Sw**: GR, SZ, VS. **Fr**: HSav. **It**: TAA.

***Puttea exsequens* (Nyl) Printzen & Davydov**

Syn.: *Lecidea exsequens* Nyl., *Lecidea gibberosa* *sensu* Th. Fr. *non* Ach.

L – Subs.: xyl – Alt.: 3 – Note: a species with a thin to endosubstratic, whitish thallus and ochraceous to red-brown apothecia with concolorous, hardly prominent margins, the exciple of strongly gelatinised, narrowly radiating hyphae, asci with an amyloid tube, and ellipsoid, simple ascospores; typically on wood, widespread in Eurasia, but rarely collected, with a single record from the Eastern Alps of Slovenia (locality erroneously assigned to Austria), but likely to be still overlooked elsewhere (perhaps misidentified as a *Biatora*-species). – **Sl**: SlA.

***Puttea margaritella* (Hulting) S. Stenroos & Huhtinen**

Syn.: *Fellhanera margaritella* (Hulting) Hafellner, *Lecidea margaritella* Hulting

L – Subs.: bry – Alt.: 3–4 – Note: a species with an inconspicuous thallus, minute, whitish apothecia with soon excluded margins and exciple composed of gelatinised radiating hyphae, asci with an amyloid tube structure, and ellipsoid, simple ascospores; on the leafy hepatic *Ptilidium pulcherrimum*, rarely spreading to adjacent wood or bark; widespread in Europe from the boreal to the temperate-montane zone; in the Alps so far only recorded from Austria and Switzerland, but likely to occur elsewhere in coniferous forests. – **Au**: T, S, K, St, N. **Sw**: BE, GL, GR, SZ, VD.

***Pycnora praestabilis* (Nyl.) Hafellner**

Syn.: *Hypocenomyce praestabilis* (Nyl.) Timdal, *Hypocenomyce xanthococca auct. medioeur*. *non* (Sommerf.) P. James & Gotth. Schneid., *Lecidea praestabilis* Nyl., *Lecidea xanthococca auct. non* Sommerf.

L – Subs.: cor, xyl – Alt.: 2–4 – Note: on wood, more rarely on the bark of conifers in the mountains. – **Au**: V, T, S, K, St, O, N. **Ge**: OB. **Sw**: GR, SZ, VS. **It**: Frl, Ven, TAA. **Sl**: SlA.

***Pycnora sorophora* (Vain.) Hafellner**

Syn.: *Hypocenomyce sorophora* (Vain.) P. James & Poelt, *Lecidea giselae* Zahlbr., Lecidea xanthococca Sommerf. subsp. sorophora Vain.

L – Subs.: cor, xyl – Alt.: 2–4 – Note: on wood and on the bark of conifers in upland areas. – **Au**: V, T, S, K, St, O, N, B. **Ge**: OB. **Sw**: GR, LU, SZ, TI, UR, UW, VS. **It**: Ven, TAA. **Sl**: SlA, Tg.

***Pycnora xanthococca* (Sommerf.) Hafellner**

Syn.: *Hypocenomyce xanthococca* (Sommerf.) P. James & Gotth. Schneid., *Lecidea xanthococca* Sommerf.

L – Subs.: cor, xyl – Alt.: 3 – Note: a species closely related to *P. praestabilis*, but apothecia usually present, as the pycnidia, which contain subglobose conidia; on wood of snags; widespread throughout the boreal zone, confined to *Pinus* (in the Alps *P. sylvestris*), with only a few records from the montane belt of the temperate zone, which need confirmation. – **Au**: O. **Ge**: OB.

***Pycnothelia papillaria* Dufour**

Syn.: *Biatora epimarta* (Nyl.) Walt. Watson, *Cenomyce papillaria* Ach. *nom.illeg.*, *Cladonia papillaria* Hoffm.*nom.illeg.*, *Lecidea epimarta* Nyl., *Lichen papillarius* Ehrh. *nom.illeg*.

L – Subs.: ter-sil – Alt.: 2–6 – Note: an arctic-alpine to cool-temperate lichen found on clay soil, often in *Calluna*-heaths; widespread throughout the Alps. – **Au**: V, T, S, K, St, N, B. **Ge**: Schw. **Sw**: GR, TI, UR, VD, VS. **Fr**: AMa, Sav, HSav. **It**: Frl, Ven, TAA, Lomb, Piem, VA, Lig. **Sl**: SlA, Tg.

***Pyrenocarpon montinii* (A. Massal.) Trevis.**

Syn.: *Porocyphus montinii* (A. Massal.) Arnold, *Psorotichia acrustacea* Harm., *Psorotichia montinii* (A. Massal.) Forssell, *Thelochroa montinii* A. Massal.

L – Subs.: sil-aqu, cal-aqu – Alt.: 1–3 – Note: a mainly southern species found on steeply inclined, south-exposed seepage tracks of calcareous rocks, sometimes invading the thalli of endolithic lichens, especially *Bagliettoa*-species; certainly more widespread in the Alps. – **Au**: St. **Fr**: AHP, AMa, Drô, HSav, Var, Vau. **It**: Frl, Ven, TAA, Lig.

***Pyrenocarpon thelostomum* (Ach. *ex* Winch & Thornhill) Coppins & Aptroot**

Syn.: *Psorotichia flotowiana* (Hepp) Müll. Arg., *Pyrenocarpon flotowianum* (Hepp) Trevis., *Pyrenopsis flotowiana* (Hepp) Nyl., *Thelochroa flotowiana* (Hepp) Körb., *Thrombium thelostomum* (Ach. *ex* Winch & Thornhill) A.L. Sm., *Verrucaria flotowiana* Hepp, *Verrucaria thelostoma* Ach. *ex* Winch & Thornhill

L # – Subs.: cal-aqu – Alt.: 2–3 – Note: on steeply inclined seepage tracks of more or less calcareous rocks at relatively low elevations; from the Alps there are a few scattered records only. – **Ge**: Schw. **Sw**: SZ. **Fr**: HSav. **It**: TAA.

***Pyrenopsis cleistocarpa* (Müll. Arg.) Forssell**

Syn.: *Psorotichia cleistocarpa* Müll. Arg.

L # – Subs.: sil – Alt.: 3 – Note: a species with an effuse, minutely granulose, black thallus turning brown-black to purplish black in the wet state, the highly convex to verruciform granules (0.05–0.1 mm in diam.) densely packed to dispersed, apothecia arising singly at the top of the thalline granules, difficult to discern from the outside, closed, the hymenium only *c.* 25 μm high, with 8-spored asci, and oblong to ellipsoid, hyaline, simple ascospores (7–9 × 4–5 μm); on periodically submerged rocks (limestone?); ecological requirements similar to those of *Gonohymenia heppii* with which it may occur together; only known from the type locality in the Western Alps (Switzerland). – **Sw**: VS.

***Pyrenopsis conferta* (Bornet) Nyl.**

Syn.: *Synalissa conferta* Bornet

L # – Subs.: sil-aqu – Alt.: 2–4 – Note: a species with a minute, dark brown thallus consisting of small erect branches up to 1 mm tall, which in part bear terminal, relatively large apothecia containing 8-spored asci and subspherical ascospores, in part bear terminal pycnidia with oblong, short pycnospores; on moist rocks, ecology similar to that of *Spilonema paradoxum*; overall distribution poorly documented. – **Fr**: AMa, HSav, Var.

***Pyrenopsis endoxantha* Anzi**

Syn.: *Psorotichia endoxantha* (Anzi) Forssell

L # – Subs.: sil – Alt.: 2 – Note: a species with a spreading thallus consisting of minute, brownish black, mostly scattered, granulose areoles, the inner part yellowish in section, and sessile, first concave and marginate, then flat and immarginate, reddish black apothecia arising between the areoles, with a brownish epi – and hypothecium, 8-spored asci, and simple, hyaline, oblong ascospores measuring 15–20 × 7–8 µm; described from material collected on siliceous nodules within limestone rocks in the surroundings of Como (Italy). The type material would be well worthy of further study. – **It**: Lomb.

***Pyrenopsis fuliginoides* Rehm**

L – Subs.: cal, int – Alt.: 3 – Note: on steeply inclined surfaces of more or less base-rich siliceous rocks, or of calcareous rocks, with optimum in the montane belt; from the Alps there are only a few scattered records. – **Au**: S. **Fr**: Sav, HSav. **It**: TAA.

***Pyrenopsis grumulifera* Nyl.**

Syn.: *Pyrenopsis multispora* Coppins

L – Subs.: cal, int – Alt.: 4 – Note: a species with a crustose, blackish-brown, areolate thallus, immersed apothecia with punctiform discs, and polyspored (up to 64) asci with ellipsoid ascospores; on moist rocks; widespread in NW Europe; apparently rare in the Alps but likely to be undercollected. – **Au**: T, K. **Sw**: SZ.

***Pyrenopsis micrococca* (Bornet & Nyl.) Forssell**

Syn.: *Synalissa micrococca* Bornet & Nyl.

L – Subs.: sil – Alt.: 1 – Note: on basic siliceous rocks in sun-exposed seepage tracks, mostly in the Mediterranean belt, with a record from the Western Alps (France). – **Fr**: AMa.

***Pyrenopsis picina* (Nyl.) Forssell**

Syn.: *Synalissa picina* Nyl.

L # – Subs.: sil – Alt.: 2–4 – Note: a species close to *P. grumulifera* from which it is often not distinguished, but with an effuse, thin, black thallus overgrowing mosses; overall distribution poorly documented, with a few records from the Western Alps (France). – **Fr**: AMa, Var.

***Pyrenopsis pleiobola* Nyl.**

L – Subs.: sil – Alt.: 3–4 – Note: a species with a thallus consisting of scattered, brown, hemispherical areoles, often with immersed apothecia, hymenium practically without paraphyses, and polysporous asci containing subspherical ascospores up to 5 µm in diam.; on moist siliceous rocks; rare in Europe from the boreal to temperate-montane zones, with a single record from the Western Alps (France). – **Fr**: HSav.

***Pyrenopsis sanguinea* Anzi**

Syn.: *Psorotichia sanguinea* (Anzi) Jatta

L # – Subs.: sil – Alt.: 3–4 – Note: a species of sunny surfaces of basic siliceous rocks along seepage tracks, which deserves further study. – **It**: TAA, Lomb.

***Pyrenopsis subareolata* Nyl.**

Syn.: *Cryptothele rhodosticta auct. non* (Taylor) Henssen, *Pyrenopsis fuscatula* Nyl., *Pyrenopsis rhodosticta auct. non* (Taylor) Müll. Arg., *Pyrenopsis rocaltensis* Couderc

L – Subs.: sil – Alt.: 2–4 – Note: on siliceous rocks with prolonged water seepage after rain; apparently more common in the Western and Southern Alps, but perhaps overlooked or confused with other species elsewhere. – **Fr**: AHP, AMa, Var. **It**: TAA, Lomb, VA.

***Pyrenopsis subcooperta* Anzi**

Syn.: *Psorotichia subcooperta* (Anzi) Jatta

L # – Subs.: bry, sil – Alt.: 4–5 – Note: on mossy siliceous rocks with a prolonged water seepage after rain; described from the Italian Alps and also reported from France (outside the Alps); the type material, from Mt. Sobretta, was overgrowing epiphytic mosses on schist, in the alpine belt. – **It**: Lomb.

***Pyrenopsis triptococca* Nyl.**

L – Subs.: int – Alt.: 3–4 – Note: on basic siliceous rocks, especially basalt, in sunny seepage tracks. – **Au**: T. **Fr**: Var.

***Pyrenula chlorospila* Arnold**

Syn.: *Pyrenula nitidella* (Flörke *ex* Schaer.) Müll. Arg. var. chlorospila (Arnold) Degel., *Verrucaria chlorospila* Nyl. *nom.illeg*.

L – Subs.: cor – Alt.: 2 – Note: a mild-temperate to Mediterranean-Atlantic species found on smooth bark (especially of *Fraxinus* and *Salix*, but also of *Corylus* and *Quercus*) in deciduous open forests, often along rivers, with a few records from the Western Alps (France). – **Fr**: AMa, Var, Vau.

***Pyrenula laevigata* (Pers.) Arnold**

Syn.: Pyrenula alba A. Massal. var. laevigata (Pers.) Trevis., *Pyrenula glabrata* (Ach.) A. Massal., *Verrucaria glabrata* Ach., *Verrucaria laevigata* Pers.

L – Subs.: cor – Alt.: 2–3 – Note: a temperate species of smooth bark, most frequent on *Carpinus* and *Fagus* in open, humid woodlands; widespread in the Alps, but not very common. – **Au**: V, T, S, K, St, O, N. **Ge**: OB, Schw. **Sw**: BE, GL, GR, SZ, UW. **Fr**: HSav. **It**: Frl, Ven, Lomb. **Sl**: SlA, Tg.

***Pyrenula nitida* (Weigel) Ach.**

Syn.: *Arthopyrenia nitida* (Weigel) H. Olivier, *Bunodea nitida* (Weigel) Beltr., *Sphaeria nitida* Weigel, *Verrucaria nitida* (Weigel) Schrad.

L – Subs.: cor – Alt.: 2–3 – Note: a temperate species with optimum on basal parts of old trunks of *Fagus* in slightly open forests, but also on *Carpinus* and other deciduous trees (*e.g. Quercus*); widespread and often rather common throughout the Alps. – **Au**: V, T, S, K, St, O, N, B. **Ge**: OB. **Sw**: BE, FR, GL, GR, SG, SZ, UW, VD, VS. **Fr**: Drô, Isè, Sav, HSav, Var. **It**: Frl, Ven, TAA, Lomb, Piem. **Sl**: SlA, Tg.

***Pyrenula nitidella* (Flörke *ex* Schaer.) Müll. Arg.**

Syn.: Bunodea nitida (Weigel) Beltr. var. nitidella (Flörke *ex* Schaer.) Beltr., Pyrenula nitida (Weigel) Ach. var. dermatodes (Borrer) Trevis., *Pyrenula nitida* (Weigel) Ach. var. *nitidella* (Flörke *ex* Schaer.) Schaer., Verrucaria nitida (Weigel) Schrad. var. nitidella Flörke *ex* Schaer.

L – Subs.: cor – Alt.: 1–3 – Note: a mild-temperate to Mediterranean-Atlantic species found on the bark of deciduous trees in open, humid woodlands. – **Au**: V, S, K, St, O, N, B. **Ge**: OB. **Sw**: BE, GR. **Fr**: HSav. **It**: Ven, Lomb, VA, Lig. **Sl**: SlA, Tg.

***Pyrgidium montellicum* (Beltr.) Tibell**

Syn.: *Acolium montellicum* Beltr.

L – Subs.: cor – Alt.: 2 – Note: a mainly tropical species described from the pre-Alpine hills of NE Italy (the only European record), where it is probably extinct. The hitherto known distribution includes also India, Colombia, and Costa Rica. – **It**: Ven.

***Pyrrhospora quernea* (Dicks.) Körb.**

Syn.: *Biatora quernea* (Dicks.) Fr., *Lecidea quernea* (Dicks.) Ach., *Lichen querneus* Dicks., *Protoblastenia quernea* (Dicks.) Clauzade

L – Subs.: cor – Alt.: 1–3 – Note: a mainly Mediterranean-Atlantic species found on bark, sometimes on lignum; certainly very rare in the Alps, at low elevations. – **Au**: St, O, N. **Sw**: ?BE, LU, ?TI. **Fr**: AMa, Var. **It**: Frl, Lomb, Piem. **Sl**: SlA.

***Racodium rupestre* Pers.**

Syn.: *Cystocoleus rupestris* (Pers.) Rabenh., *Rhacodiopsis rupestris* (Pers.) Donk

L – Subs.: sil – Alt.: 2–5 – Note: a widespread, temperate to southern boreal-montane, circumpolar lichen found on shaded, vertical or underhanging surfaces of siliceous rocks protected from rain, with a rather wide altitudinal range; undercollected, and certainly more widespread in the Alps. – **Au**: V, T, S, K, St. **Sw**: BE, SG, VS. **It**: TAA, Lomb, VA. **Sl**: SlA.

***Ramalina baltica* Lettau**

Syn.: *Ramalina obtusata auct*.

L – Subs.: cor – Alt.: 2–3 – Note: a species resembling *R. obtusata*, with which it was often confused, but with larger thalli (exceeding 3 cm in length) and wider branches (more than 2 mm wide) bearing parietal, laminal and apical soralia, the medulla mostly filled with arachnoid hyphae; on trunks and branches of deciduous trees; widespread in Europe, with a strain with divaricatic acid most frequent in Western Europe, and a strain with evernic acid in Central and Eastern Europe, with a few records from the Eastern Alps, but likely to occur also elsewhere. – **Au**: T, St, O, N. **Sl**: SlA.

***Ramalina calicaris* (L.) Fr.**

Syn.: *Lichen calicaris* L., Ramalina calicaris (L.) Fr. var. canaliculata Fr., Ramalina calicaris (L.) Fr. var. evernioides (Anzi *ex* Jatta) Motyka, Ramalina calicaris (L.) Fr. var. laciniata Harm., Ramalina calicaris (L.) Fr. var. subamplicata Nyl., *Ramalina fraxinea* (L.) Ach. var. evernioides Anzi *ex* Jatta, Ramalina fraxinea (L.) Ach. subsp. canaliculata (Fr.) B. de Lesd., Ramalina polymorpha (Lilj.) Ach. var. crispa A. Massal. *ex* Beltr.

L – Subs.: cor – Alt.: 2–3 – Note: a mainly temperate species found on deciduous, more rarely coniferous trees, especially on branches in humid beech forests; widespread throughout the Alps but strongly declining. – **Au**: K, St, N. **Sw**: GR, TI, VS. **Fr**: AHP, AMa, Sav, HSav, Var, Vau. **It**: Ven, TAA, Lomb, Piem, VA. **Sl**: SlA, Tg.

***Ramalina capitata* (Ach.) Nyl.**

Syn.: Ramalina capitata (Ach.) Nyl. var. strepsilis (Ach.) Motyka, Ramalina polymorpha (Lilj.) Ach. var. capitata Ach., Ramalina polymorpha (Lilj.) Ach. subsp. capitata (Ach.) Clauzade & Cl. Roux, *Ramalina strepsilis* (Ach.) Zahlbr.

L – Subs.: sil – Alt.: 2–5 – Note: on the top of exposed siliceous boulders frequently visited by birds. – **Au**: V, T, S, K, St. **Ge**: Schw. **Fr**: AHP, HAl, AMa, Sav, HSav. HAl, AMa, Sav, HSav. **It**: TAA, Lomb, Piem, VA.

***Ramalina dilacerata* (Hoffm.) Hoffm.**

Syn.: *Fistulariella dilacerata* (Hoffm.) Bowler & Riefner, *Fistulariella minuscula* (Nyl.) Bowler & Rundel, Lobaria calicaris (L.) Hoffm. var. dilacerata Hoffm., Ramalina calicaris (L.) Fr. f. minuscula Nyl., *Ramalina minuscula* (Nyl.) Nyl.

L – Subs.: cor – Alt.: 3–4 – Note: a rare cool-temperate to boreal-montane, probably circumpolar lichen found on twigs and branches of acid-barked trees (especially conifers) and more rarely on lignum in very humid situations. – **Au**: T, St, N. **Ge**: ?A. **Sw**: GR. **It**: Ven, TAA.

***Ramalina elegans* (Bagl. & Carestia) Stizenb.**

Syn.: Ramalina calicaris (L.) Fr. subsp. elegans Bagl. & Carestia

L – Subs.: cor – Alt.: 3–4 – Note: on bark of old deciduous trees, more rarely on conifers, in very humid, open montane forests; a lichen which deserves further study. – **Ge**: Schw. **It**: TAA, Piem. **Sl**: SlA.

***Ramalina farinacea* (L.) Ach.**

Syn.: *Lichen farinaceus* L., Ramalina calicaris (L.) Fr. var. farinacea (L.) Rabenh., ?*Ramalina fallax* Motyka, Ramalina farinacea (L.) Ach. var. hypoprotocetrarica (W.L. Culb.) D. Hawksw., Ramalina farinacea (L.) Ach. var. multifida Ach., Ramalina farinacea (L.) Ach. var. pendulina (Ach.) Ach., Ramalina farinacea (L.) Ach. var. phalerata (Ach.) Ach., Ramalina farinacea (L.) Ach. var. reagens B. de Lesd., *Ramalina hypoprotocetrarica* W.L. Culb., *Ramalina reagens* (B. de Lesd.) W.L. Culb., *Ramalina subfarinacea* (Nyl. *ex* Cromb.) Nyl. var. salazinica D. Hawksw.

L – Subs.: cor – Alt.: 1–4 – Note: a widespread, Mediterranean-Atlantic to southern boreal lichen found on bark in humid situations, from the mountains to the Mediterranean belt; the species is chemically and morphologically very polymorphic; widespread and locally common throughout the Alps, with optimum in the montane belt. – **Au**: V, T, S, K, St, O, N, B. **Ge**: OB. **Sw**: AP, BE, FR, GL, GR, LU, SG, SZ, UR, UW, VD, VS. **Fr**: AHP, HAl, AMa, Drô, Isè, Sav, HSav, Var, Vau. **It**: Frl, Ven, TAA, Lomb, Piem, VA, Lig. **Sl**: SlA, Tg. **Li**.

***Ramalina fastigiata* (Pers.) Ach.**

Syn.: *Lichen fastigiatus* Pers., Ramalina calicaris (L.) Fr. var. fastigiata (Pers.) Fr., *Ramalina fenestrata* Motyka, Ramalina fraxinea (L.) Ach. var. fastigiata (Pers.) Fr., *Ramalina populina* (Hoffm.) Vain.

L – Subs.: cor – Alt.: 1–4 – Note: a widespread, mainly temperate lichen found on broad-leaved, more rarely coniferous trees in open stands; widespread throughout the Alps. – **Au**: V, T, S, K, St, N, B. **Ge**: OB. **Sw**: BE, GL, LU, SZ, VS. **Fr**: AHP, HAl, AMa, Isè, HSav, Var, Vau. **It**: Frl, Ven, TAA, Lomb, Piem, Lig. **Sl**: SlA, Tg.

***Ramalina fraxinea* (L.) Ach.**

Syn.: *Lichen fraxineus* L., Ramalina calicaris (L.) Fr. var. fraxinea (L.) Mont., Ramalina fraxinea (L.) Ach. var. ampliata Ach., Ramalina fraxinea (L.) Ach. var. calicariformis (Nyl.) Hue, Ramalina fraxinea (L.) Ach. var. taeniata (Ach.) Rebent., Ramalina polymorpha (Lilj.) Ach. var. angulosa A. Massal., Ramalina polymorpha (Lilj.) Ach. var. calycula A. Massal., Ramalina polymorpha (Lilj.) Ach. var. fastuosa A. Massal.

L – Subs.: cor – Alt.: 2–3 – Note: a mainly mild-temperate lichen found on isolated deciduous trees; widespread throughout the Alps but probably declining. – **Au**: V, T, S, K, St, O, N. **Ge**: OB. **Sw**: BE, FR, GL, GR, LU, SG, SZ, TI, UR, VD, VS. **Fr**: AHP, HAl, AMa, Drô, Isè, Sav, HSav, Var, Vau. **It**: Frl, Ven, TAA, Lomb, Piem, Lig. **Sl**: SlA, Tg.

***Ramalina intermedia* (Delise *ex* Nyl.) Nyl.**

L – Subs.: sil – Alt.: 1–3 – Note: a usually sterile species with tufted, stiff, erect, richly branched thalli (to 3 cm tall) with flattened laciniae (to 2 mm wide) and excavate, marginal, laminal and apical soralia producing granular soredia, containing lichen substances of the sekikaic acid syndrome, based on a type from Newfoundland; often confused with the usually larger *R. subfarinacea* (3 strains with either salacinic or/and norstictic acids) and *R. pollinaria* (with diagnostic evernic acid syndrome); on acidic rocks; very rare in Europe; the records from the Alps are in need of critical re-examination. – **Au**: T, S, St. **It**: Lig.

***Ramalina obtusata* (Arnold) Bitter**

Syn.: Ramalina minuscula (Nyl.) Nyl. var. obtusata Arnold

L – Subs.: cor – Alt.: 2–4 – Note: a cool-temperate to southern boreal species found on old conifers, more rarely on deciduous trees and shrubs in cold-moist, but open montane forests. See also note on *R. baltica*. – **Au**: V, T, S, K, St, O, N. **Ge**: OB, Schw. **Sw**: BE, FR, GR, LU, SG, SZ, UR, UW, VD, VS. **Fr**: AMa, Isè, Sav, Vau. **It**: Frl, Ven, TAA.

***Ramalina panizzei* De Not.**

L – Subs.: cor – Alt.: 3 – Note: on bark in humid montane forests, frequently confused with *R. fastigiata*, but differing, among other characters, in the presence of sekikaic and homosekikaic acids. – **Sw**: BE, GR, SZ, VD. **Fr**: HSav. **It**: Frl, Ven, Lig.

***Ramalina pollinaria* (Westr.) Ach.**

Syn.: *Lichen pollinarius* Westr., Ramalina farinacea (L.) Ach. var. bolcana A. Massal., *Ramalina intermedia auct. non* (Delise *ex* Nyl.) Nyl., Ramalina pollinaria (Westr.) Ach. var. humilis Ach.

L – Subs.: cor, sil, cal, xyl – Alt.: 2–5 – Note: a widespread, cool-temperate to subarctic-subalpine, circumpolar lichen found on ancient isolated trees, and on vertical to underhanging surfaces of base-rich or calciferous rocks; widespread throughout the Alps. – **Au**: V, T, S, K, St, O, N, B. **Ge**: OB. **Sw**: AP, BE, FR, GL, GR, LU, SG, SZ, TI, UR, UW, VD, VS. **Fr**: AHP, HAl, AMa, Isè, HSav, Var, Vau. **It**: Frl, Ven, TAA, Lomb, Piem, VA, Lig. **Sl**: SlA. **Li**.

***Ramalina polymorpha* (Lilj.) Ach.**

Syn.: Lichen calicaris L. var. polymorphus Lilj., *Ramalina grappae* Sambo, Ramalina polymorpha (Lilj.) Ach. var. ligulata (Ach.) Ach.

L – Subs.: sil – Alt.: 3–5 – Note: on the top of isolated siliceous boulders manured by birds, *e.g.* in grasslands and pastures, common only wherever suitable substrata are present. – **Au**: T. **Sw**: BE, GL, GR, TI, UR, VD, VS. **Fr**: HAl, AMa, Isè, Sav. **It**: Frl, Ven, TAA, Lomb, Piem, VA, Lig.

***Ramalina requienii* (De Not.) Jatta**

Syn.: *Ramalina colubariensis* Llimona *nom. nud.*, Ramalina pollinaria (Westr.) Ach. var. cetrarioides Bagl., Ramalina polymorpha (Lilj.) Ach. var. requienii De Not.

L – Subs.: sil – Alt.: 1 – Note: a Mediterranean species found on coastal siliceous rocks subject to humid maritime winds; exceptionally found also far from the coast, and then in sheltered, but light-rich situations, with a record from the the limit of the SW Pre-Alps (France). – **Fr**: Vau.

***Ramalina roesleri* (Schaer.) Hue**

Syn.: *Fistulariella roesleri* (Schaer.) Bowler & Rundel, Ramalina fraxinea (L.) Ach. var. roesleri Schaer., *Ramalina pollinariella* (Nyl.) Nyl.

L – Subs.: cor – Alt.: 1–4 – Note: both in open humid beech forests and in very humid Mediterranean maquis vegetation. – **Au**: V, T, S, K, St, O. **Ge**: OB, Schw. **Sw**: BE, FR, GR, SZ, VD, VS. **It**: Ven, TAA. **Sl**: SlA.

***Ramalina sinensis* Jatta**

Syn.: Ramalina calicaris (L.) Fr. f. fibrillosa Th. Fr., Ramalina calicaris (L.) Fr. var. nervosa Nyl., *Ramalina landroensis* Zopf, *Ramalina nervosa* (Nyl.) Räsänen

L – Subs.: cor – Alt.: 3–4 – Note: on twigs of trees and shrubs in montane forests, this species is abundant in *Picea obovata* stands along rivers in Central Asia, but it is very rare in the Alps. – **Au**: T, S, St. **Ge**: OB, Schw. **Sw**: BE, GR. **Fr**: AMa, Vau. **It**: TAA.

***Ramalina subfarinacea* (Nyl. *ex* Cromb.) Nyl.**

Syn.: *Ramalina angustissima* (Anzi) Vain., Ramalina farinacea (L.) Ach. var. angustissima Anzi, Ramalina farinacea (L.) Ach. var. rubescens Räsänen, Ramalina scopulorum (Ach.) Ach. var. subfarinacea Nyl. *ex* Cromb.

L – Subs.: sil – Alt.: 1–2 – Note: on siliceous and weakly calcareous rocks in humid, but very open situations, mostly at low elevations. – **Fr**: Var, Vau. **It**: Ven, Lig.

***Ramalina thrausta* (Ach.) Nyl.**

Syn.: *Alectoria crinalis* Ach., Alectoria sarmentosa (Ach.) Ach. var. crinalis (Ach.) H. Olivier, *Alectoria thrausta* Ach., Bryopogon ochroleucus (Hoffm.) Link var. crinalis (Ach.) Rabenh., Bryopogon sarmentosum (Ach.) Link var. crinalis (Ach.) Körb., *Ramalina crinalis* (Ach.) Gyeln.

L – Subs.: cor – Alt.: 2–4 – Note: a cool-temperate to southern boreal lichen found on branches and twigs of conifers and deciduous trees in montane forests with frequent fog, occasionally lignicolous and saxicolous; widespread but rare and probably declining throughout the Alps. – **Au**: T, S, K, St. **Ge**: Ge. **Sw**: BE, FR, GR, LU, SG, SZ, UR, UW, VD, VS. **Fr**: AHP, Sav, HSav, Vau. **It**: Frl, Ven, TAA. **Sl**: SlA.

***Ramboldia cinnabarina* (Sommerf.) Kalb, Lumbsch & Elix**

Syn.: *Biatora cinnabarina* (Sommerf.) Fr., *Blastenia cinnabarina* (Sommerf.) Mig., *Lecidea cinnabarina* Sommerf., *Protoblastenia cinnabarina* (Sommerf.) Räsänen, *Pyrrhospora cinnabarina* (Sommerf.) M. Choisy

L – Subs.: cor – Alt.: 2–4 – Note: a mainly subarctic-subalpine, circumpolar species found on the smooth bark of small shrubs, usually near the ground; overlooked, being often sterile. – **Au**: T, S, K, St. **Sw**: BE, SZ, UR, VS. **Fr**: Isè, HSav. **It**: Ven, Piem, Lig.

***Ramboldia elabens* (Fr.) Kantvilas & Elix**

Syn.: *Lecidea elabens* Fr., *Lecidea melancheima* Tuck., *Pyrrhospora elabens* (Fr.) Hafellner

L – Subs.: xyl – Alt.: 2–4 – Note: a subarctic-subalpine to boreal-montane, probably circumpolar species found on hard wood, often with *Calicium tigillare*. – **Au**: T, S, K, St, O, N. **Ge**: OB. **Sw**: BE, GR, LU, VS. **Fr**: HAl. **It**: Frl, Ven, TAA, Lomb, Piem.

***Ramboldia insidiosa* (Th. Fr.) Hafellner**

Syn.: *Lecidea insidiosa* Th. Fr., *Nesolechia erichsenii* Räsänen

L – Subs.: xyl-par, cor-par – Alt.: 3–5 – Note: obligately lichenicolous on *Lecanora varia*, on hard lignum, more rarely on smooth, hard bark. – **Au**: V, T, S, K, St, O. **Ge**: OB. **Sw**: BE, GR. **It**: Ven, TAA, Lomb.

***Ramonia calcicola* Canals & Gómez-Bolea**

L – Subs.: cal – Alt.: 2 – Note: a rare species growing on sheltered, shaded surfaces of compact calcareous rocks at relatively low elevations; in the study area so far known only from the base of the Western Alps. – **Fr**: HAl, AMa, Vau.

***Ramonia chrysophaea* (Pers.) Vězda**

Syn.: Gyalecta cupularis (Hedw.) Schaer. var. chrysophaea (Pers.) Boistel, *Lecidea chrysophaea* (Pers.) Nyl., *Peziza chrysophaea* Pers., *Stictis chrysophaea* (Pers.) Pers.

L – Subs.: cor – Alt.: 1–3 – Note: a Mediterranean-Atlantic species found on the soft bark of old trees, especially *Ulmus*, in humid and shaded situations; inconspicuous and easily overlooked, but certainly not common in the Alps. – **Au**: St. **It**: Frl.

***Ramonia luteola* Vězda**

L – Subs.: cor – Alt.: 3–4 – Note: on trunks of deciduous trees in humid and shaded situations; hitherto known from humid areas of Austria, the Balkan Peninsula, the Carpathians, Finland and Scotland, with a few records from the Eastern Alps (Austria). – **Au**: V, St.

***Ramonia subsphaeroides* (Tav.) Vězda**

Syn.: *Gyalecta subsphaeroides* Tav.

L – Subs.: cor – Alt.: 2 – Note: a Mediterranean-Atlantic epiphytic species, mostly found on *Quercus* in shaded and humid stands, with a few records from the Western Alps (France). – **Fr**: AHP, AMa, Var, Vau.

***Reichlingia leopoldii* Diederich & Scheid.**

L – Subs.: sil, cor – Alt.: 2–3 – Note: originally described as a lichenicolous fungus, this species is now recognised as a lichenised hyphomycete. It grows on underhanging surfaces of siliceous rocks, more rarely on the bark of old oaks in sheltered situations, mostly in the submediterranean belt. – **Au**: S, O. **Sw**: BE, GL, LU, UW. **It**: Lomb.

***Reichlingia zwackhii* (Sandst.) Frisch & G. Thor**

Syn.: *Arthonia zwackhii* Sandst.

L – Subs.: cor – Alt.: 2–3 – Note: a mild-temperate to tropical species found on smooth bark, especially of *Fraxinus* and *Carpinus*, in humid woodlands, with a few records from the Western Alps. – **Fr**: Var, Vau.

***Rhizocarpon alpicola* (Fr.) Rabenh.**

Syn.: *Buellia alpicola* (Fr.) Anzi, *Catocarpus chionophilus* (Th. Fr.) Stein, *Catocarpus oreites* (Vain.) Eitner, *Rhizocarpon chionophilum* Th. Fr., *Rhizocarpon conglomeratum* (Fr.) Räsänen *nom.illeg.*, Rhizocarpon geographicum (L.) DC. var. alpicola (Fr.) A. Massal., Rhizocarpon geographicum (L.) DC. var. geronticum (Ach.) Räsänen, *Rhizocarpon geronticum* (Ach.) H. Magn. *comb. inval.*, *Rhizocarpon oreites* (Vain.) Zahlbr., *Rhizocarpon ridniense* Räsänen

L – Subs.: sil – Alt.: 3–6 – Note: an arctic-alpine, circumpolar lichen found on horizontal to weakly inclined surfaces of hard siliceous rocks with a long snow cover in cold situations. – **Au**: V, T, S, K, St, N. **Ge**: OB. **Sw**: BE, GR, TI, UR, UW, VS. **Fr**: AHP, HAl, AMa, Sav, HSav. **It**: Frl, TAA, Lomb, Piem, VA.

***Rhizocarpon amphibium* (Fr.) Th. Fr.**

Syn.: *Lecidea amphibia* Fr.

L – Subs.: sil – Alt.: 3–5 – Note: a species somewhat resembling *Rh. lavatum*, but thallus brownish to greenish grey, apothecia often in circular arrangement and with thinner margins, ascospores smaller (mostly less than 40 µm long) and with fewer cells in optical view (usually 8–12, with only 1 longitudinal septum); on periodically inundated siliceous rocks *e.g.* along streams; widespread in Europe but much rarer than *Rh. lavatum* and mostly in areas with a suboceanic climate; from the Alps there are only a few isolated records. – **Au**: T. **Fr**: HSav.

***Rhizocarpon atroflavescens* Lynge**

Syn.: Rhizocarpon atroflavescens Lynge subsp. pulverulentum (Schaer.) Runemark, *Rhizocarpon chiastomerum* Lettau, *Rhizocarpon pulverulentum* (Schaer.) Räsänen

L – Subs.: int, sil – Alt.: 3–5 – Note: a cool-temperate to arctic-alpine, perhaps circumpolar species found on steeply inclined surfaces of base-rich, or weakly calciferous siliceous rocks near or above treeline, often starting the life-cycle on *Pertusaria*-species; widespread throughout the Alps. – **Au**: V, T, S, K, St. **Ge**: OB, Schw. **Sw**: BE, GR, TI, UW, VS. **Fr**: AHP, HAl, AMa, Sav. **It**: TAA, Lomb, Piem, Lig.

***Rhizocarpon atrovirellum* (Nyl.) Zahlbr.**

Syn.: *Lecidea atrovirella* Nyl.

L – Subs.: sil – Alt.: 2–3 – Note: a species close to *Rh. viridiatrum*, differing in the smaller, submuriform ascospores (to 20 µm long) with a single transversal septum; on siliceous schists in SW Europe, with a few records from the Western Alps (France). – **Fr**: AHP, HAl.

***Rhizocarpon badioatrum* (Flörke *ex* Spreng.) Th. Fr.**

Syn.: *Buellia badioatra* (Flörke *ex* Spreng.) Mudd var. vulgaris Körb., *Catocarpus badioater* (Flörke *ex* Spreng.) Arnold, *Catocarpus badioater* (Flörke *ex* Spreng.) Arnold var. vulgaris (Körb.) Arnold, *Lecidea badioatra* Flörke *ex* Spreng., *Rhizocarpon badioatrum* (Flörke *ex* Spreng.) Th. Fr. var. vulgare (Körb.) Th. Fr., *Rhizocarpon rivulare* (Flot.) Körb.

L – Subs.: sil – Alt.: 1–5 – Note: a holarctic early coloniser of siliceous pebbles and small boulders near the ground, with a wide altitudinal range; widespread throughout the Alps. – **Au**: V, T, S, K, St, O, N. **Ge**: OB, Schw. **Sw**: BE, GR, SZ, TI, UR, VD, VS. **Fr**: AHP, HAl, AMa, Sav, HSav, Var, Vau. **It**: Frl, Ven, TAA, Lomb, Piem, VA. **Sl**: SlA.

***Rhizocarpon carpaticum* Runemark**

Syn.: Rhizocarpon intermediellum Räsänen subsp. carpaticum (Runemark) Clauzade & Cl. Roux

L – Subs.: sil, met – Alt.: 3–5 – Note: on siliceous rocks in underhangs protected from rain in cold sites with frequent fog, with optimum near or above treeline. – **Au**: V, T, S, K, St, N. **Ge**: Schw. **Fr**: AHP, AMa, Sav, HSav. **It**: Frl, Ven, TAA, Piem.

***Rhizocarpon coeruleoalbum* (Kremp.) Zahlbr.**

L – Syn.: *Buellia coeruleoalba* (Kremp.) Th. Fr., *Rehmia coeruleoalba* Kremp.

Subs.: cal, int – Alt.: 3–5 – Note: on weakly calciferous or base-rich siliceous rocks, especially calcareous schists, with optimum above treeline. – **Au**: V, T, S, K, St, O. **Ge**: OB, Schw. **Sw**: GR, SZ. **Fr**: HAl, Sav, HSav, Vau. **It**: TAA, Piem, VA.

***Rhizocarpon copelandii* (Körb.) Th. Fr.**

Syn.: *Buellia copelandii* Körb., *Catocarpus badioater* (Flörke *ex* Spreng.) Arnold *f. copelandii* (Körb.) Eitner, *Catocarpus copelandii* (Körb.) Arnold, *Rhizocarpon cyclodes* Hellb. *ex* Th. Fr., *Rhizocarpon elevatum* H. Magn., *Rhizocarpon hyperboreum* (Vain.) Vain.

L – Subs.: sil – Alt.: 3–6 – Note: an arctic-alpine lichen of siliceous rocks above treeline; much more frequent in the Arctic zone than in the Alps. – **Au**: V, T, S, K. **Sw**: UR. **It**: TAA.

***Rhizocarpon dinothetes* Hertel & Leuckert**

L – Subs.: sil-par – Alt.: 4–5 – Note: on siliceous rocks near and above treeline, starting the life-cycle on the thalli of *Protoparmelia badia*; in the study area there are records from the Eastern Alps only (Austria, Slovenia), but the species might be more widespread. – **Au**: T, S, K, St, N. **Sl**: SlA.

***Rhizocarpon disporum* (Nägeli *ex* Hepp) Müll. Arg.**

Syn.: *Lecidea dispora* Nägeli *ex* Hepp, *Rhizocarpon confervoides* *sensu* A. Massal., *Rhizocarpon disporum* (Nägeli *ex* Hepp) Müll. Arg. var. montagnei (Körb.) Zahlbr., *Rhizocarpon montagnei* Körb.

L – Subs.: sil – Alt.: 2–4 – Note: a widespread, probably holarctic lichen of dry-continental areas, found on exposed surfaces of basic siliceous rocks; most frequent in dry-continental valleys of the Alps. – **Au**: T, S, K, St. **Sw**: GR, UR, VD, VS. **Fr**: AHP, HAl, AMa, Sav, HSav. **It**: TAA, Lomb, Piem, VA. **Sl**: SlA.

***Rhizocarpon distinctum* Th. Fr.**

Syn.: *Lecidea distincta* (Th. Fr.) Stizenb., *Lecidea illota* Sandst., *Lecidea porphyrostrota* Vain., *Rhizocarpon ambiguum* (Schaer.) Zahlbr., *Rhizocarpon atroalbum* *sensu* Arnold *non* (Nyl.) Zahlbr., *Rhizocarpon hyalescens* Vain., *Rhizocarpon illotum* (Sandst.) Lettau, *Rhizocarpon porphyrostrotum* (Vain.) Vain.

L – Subs.: sil – Alt.: 2–6 – Note: on basic siliceous rocks, often on brick and roofing tiles, with a wide altitudinal range; sometimes parasitic, when young, on *Aspicilia caesiocinerea*. – **Au**: V, T, S, K, St, O, N, B. **Ge**: OB, Schw. **Sw**: SZ. **Fr**: AHP, AMa, Isè, Sav, HSav, Var, Vau. **It**: Frl, TAA, Piem, VA, Lig.

***Rhizocarpon drepanodes* Feuerer**

Syn.: Rhizocarpon lecanorinum Anders subsp. drepanodes (Feuerer) Clauzade & Cl. Roux

L – Subs.: sil, met – Alt.: 4–5 – Note: similar to *Rh. ferax* in the thallus with scattered, convex, roundish to lunulate areoles with laterally attached apothecia, but ascospores larger (to 50 µm long), and with a higher number of cells in optical view (mean *c.* 35); on steep rock faces and under overhangs of siliceous rocks, in upland areas; not easy to distinguish from *Rh. ferax*; widespread in Europe, including the Alps. According to Roux, however, all of the records from the French Alps are either very dubious or refer to specimens of *R. lecanorinum* with a medulla reacting P+ yellow. – **Au**: V, T, S, K, St.

***Rhizocarpon effiguratum* (Anzi) Th. Fr.**

Syn.: *Buellia effigurata* Anzi, *Catocarpus anzianus* Müll. Arg., *Lecidea effigurata* (Anzi) Stizenb., *Rhizocarpon sphaericum* (Schaer.) Mig.; incl. *Rhizocarpon italicum* Räsänen

L – Subs.: sil – Alt.: 3–6 – Note: a weak competitor found under overhanging or vertical surfaces of siliceous rocks protected from rain in upland areas, on the thalli of *Pleopsidium*-species. – **Au**: V, T, S, K, St. **Sw**: BE, GR, TI, UR, VS. **Fr**: AHP, HAl, AMa, Sav, HSav. **It**: Frl, TAA, Lomb, Piem, VA.

***Rhizocarpon epispilum* (Nyl.) Zahlbr.**

Syn.: *Buellia epispila* (Nyl.) B. de Lesd., *Lecidea epispila* Nyl., *Rhizocarpon superstratum* J. Steiner

L – Subs.: sil-par – Alt.: 1–2 – Note: on sheltered surfaces of siliceous rocks wetted by rain, starting the life-cycle on thalli of *Pertusaria* below the montane belt; in the study area so far known only from the limit of the SW Pre-Alps (France). – **Fr**: Vau. **It**: Lig.

***Rhizocarpon eupetraeoides* (Nyl.) Blomb. & Forssell**

Syn.: *Lecidea eupetraeoides* Nyl.

L – Subs.: int – Alt.: 4–5 – Note: on weakly calciferous rocks near and above treeline; probably more widespread in the Alps. – **It**: Piem.

***Rhizocarpon eupetraeum* (Nyl.) Arnold**

Syn.: *Lecidea eupetraea* Nyl., *Lecidea parapetraea* Nyl., *Lecidea petraeiza* Nyl., *Rhizocarpon arcticum* Räsänen, *Rhizocarpon dissentiens* Arnold, *Rhizocarpon parapetraeum* (Nyl.) Zahlbr.; incl. *Rhizocarpon petraeizum* (Nyl.) Arnold

L – Subs.: sil – Alt.: 3–6 – Note: on inclined to vertical faces of acidic siliceous rocks, mostly sandstone, with a wide altitudinal range. – **Au**: ?V, T, K, St, N. **Sw**: GR, VS. **It**: Ven, TAA, Piem, VA.

***Rhizocarpon ferax* H. Magn.**

L – Subs.: sil-par – Alt.: 4–5 – Note: a silicicolous species starting the life-cycle on other crustose lichens; probably more widespread in the Alps. – **Au**: V, S, K. **It**: TAA.

***Rhizocarpon furax* Poelt & V. Wirth**

L – Subs.: sil-par – Alt.: 4–6 – Note: on mineral-rich siliceous rocks near and above treeline, starting the life-cycle on the thalli of *Lecidea lapicida* *s.lat.*; certainly more widespread in the Alps. – **Au**: ?V, T, S, K, St. **It**: Frl, TAA.

***Rhizocarpon furfurosum* H. Magn. & Poelt**

L – Subs.: met, sil – Alt.: 2–4 – Note: on metal-rich siliceous rocks, mostly on steeply inclined to underhanging faces in upland areas; easy to overlook, being mostly sterile, and to be looked for further in the Alps. – **Au**: S, St. **Fr**: AMa. **It**: Piem.

***Rhizocarpon geminatum* Körb.**

Syn.: *Biatorina concreta* (Ach.) Mudd, *Buellia concreta* (Ach.) Zwackh, *Rhizocarpon concretum* (Ach.) Zahlbr., *Rhizocarpon disporum auct. non* (Nägeli *ex* Hepp) Müll. Arg.

L – Subs.: sil – Alt.: 2–5 – Note: a widespread lichen of dry-continental areas found on steeply inclined faces of base-rich or weakly calciferous siliceous rocks, both in natural and man-made substrata (*e.g.* on roofing tiles, walls); a chemically heterogeneous species, probably less thermophilous than the closely related *Rh. disporum*. – **Au**: V, T, S, K, St. **Ge**: OB, Schw. **Sw**: BE, GR, LU, SZ, TI, VS. **Fr**: AHP, HAl, AMa, Sav, HSav, Var. **It**: Frl, Ven, TAA, Lomb, Piem, VA, Lig.

**Rhizocarpon geographicum (L.) DC. subsp. geographicum**

Syn.: *Lecidea geographica* (L.) Rebent., *Lichen geographicus* L., *Patellaria geographica* (L.) Duby, *Rhizocarpon haeyrenii* Räsänen, *Rhizocarpon semilecanorinum* Räsänen

L – Subs.: sil, int, cor, lig – Alt.: 2–6 – Note: a cool-temperate to arctic-alpine, circumpolar, polymorphic lichen of siliceous rocks wetted by rain. Here we also place all records of *Rh. geographicum* *s.lat.*: this taxon badly needs a worldwide revision based on molecular data. – **Au**: V, T, S, K, St, O, N. **Ge**: OB, Schw. **Sw**: BE, GR, LU, SZ, TI, UR, VD, VS. **Fr**: AHP, HAl, AMa, Isè, Sav, HSav, Var, Vau. **It**: Frl, Ven, TAA, Lomb, Piem, VA, Lig. **Sl**: SlA. **Li**.

**Rhizocarpon geographicum (L.) DC. subsp. arcticum (Runemark) Hertel**

Syn.: Rhizocarpon tinei (Tornab.) Runemark subsp. arcticum Runemark

L – Subs.: sil – Alt.: 4–6 – Note: an arctic-alpine lichen found on siliceous, exposed rocks, most common in the nival belt of the Alps. – **Au**: ?V, T, K, St. **It**: VA.

**Rhizocarpon geographicum (L.) DC. subsp. diabasicum (Räsänen) Poelt & Vězda**

Syn.: *Rhizocarpon amphiboliticum* Räsänen, *Rhizocarpon diabasicum* Räsänen, *Rhizocarpon havaasii* Räsänen, Rhizocarpon tinei (Tornab.) Runemark subsp. diabasicum (Räsänen) Runemark

L – Subs.: sil, int – Alt.: 3–6 – Note: on siliceous, sometimes also on superficially decalcified calcareous rocks, where it appears in forms with a whitish thallus, with optimum near and above treeline. – **Au**: ?V, T, S, K. **Sw**: BE, GR, TI, UR, UW, VS. **Fr**: AHP, HAl, AMa, Isè, Sav, HSav. **It**: TAA, Lomb, Piem.

**Rhizocarpon geographicum (L.) DC. subsp. frigidum (Räsänen) Hertel**

Syn.: *Rhizocarpon frigidum* Räsänen, Rhizocarpon tinei (Tornab.) Runemark subsp. frigidum (Räsänen) Runemark

L – Subs.: sil – Alt.: 4–6 – Note: an arctic-alpine lichen found on exposed, steeply inclined to underhanging surfaces of siliceous rocks, mostly above treeline. – **Au**: ?V, T, S, K, St. **Sw**: BE, GR, TI, UR, VS. **Fr**: AHP, AMa, Sav, HSav. **It**: TAA, Lomb, VA.

**Rhizocarpon geographicum (L.) DC. subsp. kittilense (Räsänen) Ahti**

Syn.: *Rhizocarpon kittilense* Räsänen, Rhizocarpon lindsayanum Räsänen subsp. kittilense (Räsänen) Runemark, *Rhizocarpon olivetorum* Räsänen, *Rhizocarpon riparium* Räsänen

L # – Subs.: sil – Alt.: 3–6 – Note: on steeply inclined surfaces of siliceous rocks in rather sheltered and humid situations in upland areas. – **Au**: ?V, T, S, K, St. **Fr**: AHP, AMa. **It**: Piem, VA.

**Rhizocarpon geographicum (L.) DC. subsp. lindsayanum (Räsänen) Ahti**

Syn.: *Rhizocarpon lindsayanum* Räsänen, Rhizocarpon riparium Räsänen subsp. lindsayanum (Räsänen) J.W. Thomson

L # – Subs.: sil – Alt.: 2–5 – Note: this subspecies seems to have a circumboreal distribution. It is found on siliceous, often dust-impregnated rocks, also on walls and boulders in semi-urban environments. – **Au**: ?V, ?T, S, K, St. **Sw**: BE, GR, LU, TI, VS. **Fr**: AHP, HAl, AMa, Sav, HSav.

**Rhizocarpon geographicum (L.) DC. subsp. prospectans (Räsänen) D. Hawksw. & Sowter**

Syn.: *Rhizocarpon prospectans* Räsänen, Rhizocarpon tinei (Tornab.) Runemark subsp. prospectans (Räsänen) Runemark

L # – Subs.: sil – Alt.: 2–5 – Note: on exposed surfaces of base-rich siliceous rocks. A western lichen in Europe, which needs further study. – **Sw**: BE, GR, UR, VS. **Fr**: HAl, AMa, HSav. **It**: Lomb.

**Rhizocarpon geographicum (L.) DC. subsp. tinei (Tornab.) Clauzade & Cl. Roux**

Syn.: *Lecidea tinei* Tornab., *Rhizocarpon tinei* (Tornab.) Runemark

L # – Subs.: sil – Alt.: 1–4 – Note: on siliceous rocks; most common in the Mediterranean mountains, but also found in the Alps in dry-warm areas. – **Fr**: AHP, HAl, AMa, Isè, HSav, Var, Vau. **It**: Ven, TAA, Lomb, Piem, VA, Lig.

***Rhizocarpon grande* (Flörke *ex* Flot.) Arnold**

Syn.: Lecidea petraea (Wulfen) Ach. f. grande Flörke *ex* Flot., *Rhizocarpon endamyleum* Th. Fr.

L – Subs.: sil, cor – Alt.: 2–4 – Note: a silicicolous species resembling *Rh. eupetraeum* in the faintly amyloid medulla, the 8-spored asci, and the eumuriform spores soon becoming green-brown, but medulla K+ yellow or K-, with a different secondary chemistry, and ascospores larger (to *c.* 50 µm long); widespread in Europe, including the Alps, but the known distribution is lacunose, since the species was not always distinguished from *Rh. eupetraeum*. – **Au**: V, T, S, K, St. **Sw**: VS. **Fr**: HSav.

***Rhizocarpon hochstetteri* (Körb.) Vain.**

Syn.: *Buellia chlorospora* (Nyl.) Hellb., *Buellia colludens* (Nyl.) Arnold, *Buellia hochstetteri* (Körb.) Mong., *Catillaria colludens* (Nyl.) Jatta, *Catillaria hochstetteri* Körb., *Catocarpus koerberi* Stein, *Lecidea applanata* (Fr.) Leight., *Lecidea colludens* Nyl., *Rhizocarpon applanatum* (Fr.) Th. Fr., *Rhizocarpon crenulatum* H. Magn., *Rhizocarpon koerberi* (Stein) Klem., *Rhizocarpon massalongii* *sensu* Malme *non* (Körb.) Malme

L – Subs.: sil – Alt.: 2–5 – Note: a widespread, probably northern holarctic lichen found on mineral-rich siliceous rocks, in seepage tracks and near creeks. – **Au**: V, T, S, K, St. **Ge**: Schw. **Sw**: GR, VS. **Fr**: AHP, Sav. **It**: Frl, Ven, TAA, Lomb, Piem, VA.

***Rhizocarpon inarense* (Vain.) Vain.**

Syn.: *Lecidea inarensis* Vain.

L – Subs.: sil – Alt.: 5 – Note: a circumboreal to arctic-alpine species of siliceous rocks, known from a few localities only in the Alps. – **Au**: T. **It**: TAA.

***Rhizocarpon infernulum* (Nyl.) Lynge**

Syn.: *Lecidea infernula* Nyl.

L – Subs.: int – Alt.: 5 – Note: a species resembling *Rh. hochstetteri*, but with a thinner thallus and smaller ascospores (only to 20 µm long); on siliceous rocks or schists containing a low content of calcium; widespread in the Northern Hemisphere, with a single record from the Western Alps (Switzerland). – **Sw**: SZ.

***Rhizocarpon intermediellum* Räsänen**

Syn.: *Rhizocarpon wulffianum* Räsänen

L # – Subs.: sil-par – Alt.: 4–6 – Note: a species morphologically resembling *Rh. norvegicum*, but ascospores submuriform, larger (to 20 µm long), with 1–4 transversal septa and 1 incomplete longitudinal septum; the specific difference from *Rh. furax* is in need of critical re-evaluation; on basic siliceous rocks (*e.g.* amphibolite) and schists with low content of calcium in exposed situations, often on pebbles in wind-swept ridges, parasitic on various crustose lichens (incl. species of *Bellemerea*, *Lecidea*, Buellia sect. Melanaspicilia, *Tremolecia*) in the early stages of development; widespread in Europe, arctic to nemoral-alpine; from the Alps there are several scattered records, but apparently the species was overlooked in some regions. – **Au**: ?V, T, S, K, St. **Fr**: AMa, Sav.

***Rhizocarpon intersitum* Arnold**

Syn.: *Rhizocarpon birgittae* H. Magn., *Rhizocarpon diversisporum* Hav.

L – Subs.: sil – Alt.: 3–5 – Note: a rather poorly known species found on inclined to vertical faces of acidic siliceous rocks, often near waterfalls, mostly in upland areas. – **Au**: T, S, K, St. **Sw**: GR. **It**: TAA.

***Rhizocarpon kakurgon* Poelt**

L – Subs.: sil-par, int-par – Alt.: 4–6 – Note: an arctic to nemoral-alpine (-nival) species related to *Rh. viridiatrum*, but thallus sulphur-yellow and ascospores smaller (less than 20 µm long) and with fewer cells in optical view; on hard calcareous schists, obligately parasitic on *Aspicilia*-species (*e.g. A. candida*, *A. mashiginensis*); most records are from the Alps but the species was also reported from Greenland. – **Au**: V, T. **Ge**: Schw. **Sw**: SG. **Fr**: AHP, HAl.

***Rhizocarpon lavatum* (Fr.) Hazsl.**

Syn.: *Lecidea atroalba auct.* var. lavata Fr., Rhizocarpon obscuratum (Ach.) A. Massal. f. lavatum (Fr.) Th. Fr., *Rhizocarpon orphninum* (Vain.) Vain., *Rhizocarpon perlutum* (Nyl.) Zahlbr. *non auct*.

L – Subs.: sil, int, sil-aqu, int-aqu – Alt.: 3–6 – Note: a cool-temperate to arctic-alpine, circumpolar lichen found on perennially humid siliceous rocks, *e.g.* in mountain rivulets, or on small pebbles on moist ground; related to *Rh. obscuratum*; widespread throughout the Alps. – **Au**: V, T, S, K, St. **Ge**: OB, Schw. **Sw**: BE, GR, LU, SZ, TI, VS. **Fr**: AHP, HAl, AMa, Sav, HSav, Var. **It**: Frl, TAA, Lomb, Piem, VA. **Sl**: SlA.

***Rhizocarpon lecanorinum* Anders**

Syn.: Lecidea geographica (L.) Rebent. var. lecanora (Flörke) Nyl., *Rhizocarpon atrovirens auct.*, Rhizocarpon geographicum (L.) DC. var. lecanorum (Flörke) A. Massal., *Rhizocarpon lecanora* (Flörke) Lynge

L – Subs.: sil – Alt.: 1–5 – Note: a temperate to boreal-montane, circumpolar lichen, most common on stone walls, dust-impregnated siliceous boulders, roofing tiles, but also found in natural habitats, *e.g.* with *Umbilicaria deusta*; widespread throughout the Alps. – **Au**: V, T, S, K, St, B. **Ge**: Schw. **Sw**: BE, GR, LU, TI, VD, VS. **Fr**: AHP, HAl, AMa, Isè, Sav, HSav, Vau. **It**: Frl, Ven, TAA, Lomb, Piem, VA, Lig. **Sl**: SlA.

***Rhizocarpon leptolepis* Anzi**

Syn.: *Rhizocarpon phalerosporum* (Vain.) Vain.

L – Subs.: sil – Alt.: 3–5 – Note: a boreal-montane to arctic-alpine species growing on steeply inclined surfaces of hard siliceous rocks in sheltered situations, mostly in upland areas. – **Au**: V, T, S, K, St. **Sw**: GR. **It**: TAA, Lomb, Piem.

***Rhizocarpon macrosporum* Räsänen**

Syn.: *Rhizocarpon sphaerosporum* Räsänen

L – Subs.: sil – Alt.: 3–5 – Note: a chemically heterogeneous species of dust-impregnated, exposed siliceous rocks, including walls in small settlements, most common in upland areas. – **Au**: V, T, S, K, St, O. **Ge**: OB, Schw. **Sw**: BE, GR, SZ, TI, UR, UW, VS. **Fr**: AHP, HAl, AMa, Sav, HSav. **It**: Frl, TAA, Lomb, Piem, VA.

***Rhizocarpon malenconianum* (Llimona & Werner) Hafellner & H. Mayrhofer**

Syn.: *Leciographa malenconiana* Llimona & Werner

L – Subs.: sil-par – Alt.: 2 – Note: a cryptothalline species with convex apothecia and 3-septate, brown ascospores; obligately parasitic on *Diploschistes diacapsis* over gypsum or calcareous soils in arid sites; known from some scattered localities in the Mediterranean Region and from an outpost in Southern Norway, with a few records from low elevations in the Western Alps (France). – **Fr**: Drô.

***Rhizocarpon mosigiae* Poelt & Obermayer**

L – Subs.: sil-par – Alt.: 4 – Note: a species resembling the Mediterranean *Rh. epispilum*, but thallus grey and ascospores somewhat smaller; obligately parasitic on *Rimularia gibbosa* on siliceous boulders at high elevations; only known from the Eastern Alps (Austria). – **Au**: T.

***Rhizocarpon norvegicum* Räsänen**

L – Subs.: sil, met – Alt.: 3–6 – Note: a pioneer species of schistose, slightly calciferous or basic eruptive rocks in upland areas, which often starts the life-cycle on members of Acarosporaceae. – **Au**: V, T, S, K, St. **Sw**: BE, GR, UR. **It**: Frl.

***Rhizocarpon oederi* (Ach.) Körb.**

Syn.: *Lecidea oederi* Ach., *Lichen oederi* Weber *nom.illeg.*, *Lichen koenigii* Retz.

L – Subs.: met, sil – Alt.: 2–4 – Note: a mainly cool-temperate species with a wide but scattered distribution, found on metal-rich siliceous rocks, mostly at low elevations. – **Au**: T, S, K. **Sw**: GR, VS. **Fr**: HSav. **It**: Frl, TAA, Lomb, Piem, VA.

***Rhizocarpon papillatum* Vězda & Poelt**

L – Subs.: sil – Alt.: 5 – Note: a species with a greenish-yellow thallus, the inner areoles apically papillate-isidioid and with an amyloid medulla, older areoles apically decorticated and blackish; on siliceous rocks in wind-exposed sites; so far only known from the high alpine belt in Switzerland. – **Sw**: GR.

***Rhizocarpon parvum* Runemark**

L – Subs.: sil-par – Alt.: 4–5 – Note: an extremely rare and often misidentified silicicolous species resembling *Rh. pusillum*, but with an amyloid medulla; the type is parasitic on *Tremolecia atrata*; known with certainty only from Norway, the only record from the Alps needs confirmation. – **Au**: ?St.

***Rhizocarpon permodestum* Arnold**

L # – Subs.: sil – Alt.: 5–6 – Note: a species resembling *Rh. postumum*, but thallus inconspicuous, exciple reacting K+ purple-brown, and ascospores somewhat smaller (to *c.* 20 µm long); not consistently distinguished from *Rh. postumum* and specific difference in need of re-evaluation; on schistose outcrops in shaded situations above treeline; only reported from the Eastern Alps (Austria). – **Au**: T.

***Rhizocarpon petraeum* (Wulfen) A. Massal.**

Syn.: *Lecidea petraea* (Wulfen) Ach., *Lichen petraeus* Wulfen, *Patellaria petraea* (Wulfen) DC., *Rhizocarpon concentricum auct. non* (Davies) Beltr., *Rhizocarpon excentricum* (Ach.) Arnold, *Rhizocarpon perlutum auct. non* (Nyl.) Zahlbr., *Rhizocarpon richardii auct. non* (Nyl.) Zahlbr., *Rhizocarpon subconcentricum* (Körb.) Körb., *Siegertia petraea* (Wulfen) V. Wirth; incl. *Rhizocarpon variegatum* J. Steiner

L – Subs.: sil, int – Alt.: 1–5 – Note: a holarctic pioneer species of base-rich siliceous rocks, often found on old roofing tiles and on slightly calciferous sandstone, with a wide altitudinal range; widespread throughout the Alps. – **Au**: V, T, S, K, St, N. **Ge**: OB, Schw. **Sw**: BE, GR, LU, SZ, UR, VD, VS. **Fr**: AHP, HAl, AMa, Isè, Sav, HSav, Var, Vau. **It**: Frl, Ven, TAA, Lomb, Piem, VA, Lig. **Sl**: SlA, Tg.

***Rhizocarpon polycarpum* (Hepp) Th. Fr.**

Syn.: *Buellia umensis* H. Magn., *Lecidea atroalbicans* Nyl., *Lecidea confervoides auct.* var. polycarpa Hepp, *Rhizocarpon confervoides* *sensu* Rabenh., *Rhizocarpon cyanescens* (Hellb.) Zahlbr.

L – Subs.: sil, int, cor – Alt.: 1–5 – Note: a probably holarctic pioneer species found on siliceous pebbles over moist ground, or on steeply inclined faces near the ground, present at low altitudes only in humid areas; widespread throughout the Alps. – **Au**: V, T, S, K, St, O, N. **Ge**: OB, Schw. **Sw**: BE, GR, LU, SZ, UR, VD, VS. **Fr**: AHP, HAl, AMa, Isè, Sav, HSav, Var. **It**: Frl, Ven, TAA, Lomb, Piem, VA. **Sl**: SlA. **Li**.

***Rhizocarpon postumum* (Nyl.) Arnold**

Syn.: *Lecidea postuma* Nyl.

L – Subs.: sil – Alt.: 3–5 – Note: a species recalling *Rh. distinctum* in the small-sized apothecia, but medulla not amyloid and with stictic acid, apothecia less than 0.5 mm in diam. with flat, smooth discs, ascospores small (mostly less than 25 µm long) and submuriform; on siliceous rocks, often close to streams and waterfalls; widespread in Europe, but rather rare or not always distinguished, with scattered records from the Alps. – **Au**: St. **Ge**: Schw. **Fr**: AHP, AMa, Sav, HSav.

***Rhizocarpon pusillum* Runemark**

L – Subs.: sil-par – Alt.: 4–6 – Note: an arctic-alpine, circumpolar lichen related to *Rh. effiguratum*, found on exposed surfaces of hard siliceous rocks, starting the life-cycle on species of *Sporastatia*. – **Au**: V, T, S, K, St. **Sw**: GR, TI, UR, VS. **Fr**: HAl, AMa, HSav. **It**: Frl, TAA, Lomb, Piem.

***Rhizocarpon rapax* V. Wirth & Poelt**

L – Subs.: sil-par – Alt.: 4–5 – Note: on siliceous rocks near and above treeline, starting the life-cycle on the thalli of different crustose lichens; related to *Rh. tinei*, but differing in the parasitic growth; probably overlooked and more frequent in the Alps, especially in rainy areas. – **Au**: S, K. **It**: TAA, Piem.

***Rhizocarpon reductum* Th. Fr.**

Syn.: *Rhizocarpon obscuratum auct. non* (Ach.) A. Massal., *Rhizocarpon massalongii* (Körb.) Malme

L – Subs.: sil – Alt.: 1–6 – Note: a morphologically and chemically variable species of siliceous rocks, often found on pebbles or on surfaces of boulders near the ground; the optimum is in upland areas, but the species also occurs within eu-Mediterranean vegetation, in shaded-humid situations; widespread throughout the Alps. – **Au**: V, T, S, K, St, O, N, B. **Ge**: OB, Schw. **Sw**: BE, GR, LU, SZ, VD, VS. **Fr**: AMa, Isè, Sav, HSav, Var, Vau. **It**: Frl, Ven, TAA, Lomb, Piem, VA, Lig. **Sl**: SlA.

***Rhizocarpon renneri* Poelt**

L – Subs.: sil-par – Alt.: 3–5 – Note: on steeply inclined to underhanging surfaces of siliceous rocks, starting the life-cycle on the thalli of *Dimelaena oreina*; probably more widespread, but never common, in the dry valleys of the Alps. – **Au**: T, K. **Sw**: GR. **Fr**: Sav. **It**: TAA.

***Rhizocarpon ridescens* (Nyl.) Zahlbr.**

Syn.: *Lecidea ridescens* Nyl.

L – Subs.: met, sil – Alt.: 3–6 – Note: on iron-rich siliceous rocks, mostly under overhangs, with optimum near or above treeline; easily overlooked, being always sterile. – **Au**: V, T, S, K, St. **Sw**: GR, TI, VS. **Fr**: AHP, HSav. **It**: Frl, TAA, Lomb, Piem.

***Rhizocarpon saanaense* Räsänen**

Syn.: *Rhizocarpon sublucidum* Räsänen

L – Subs.: sil, int – Alt.: 3–6 – Note: an arctic-alpine, probably circumpolar lichen found on slightly calciferous siliceous rocks with a late snow cover, with optimum above treeline. – **Au**: V, T, S, K, St. **Ge**: OB. B. **Sw**: BE, GR, TI, UR, VD, VS. **Fr**: AHP, HAl, AMa, Sav. **It**: Frl, TAA, Lomb, Piem, VA, Lig.

***Rhizocarpon santessonii* Timdal**

L – Subs.: sil-par – Alt.: 5 – Note: an arctic to nemoral-alpine species with a greyish-yellow thallus (rhizocarpic acid absent!), a non-amyloid medulla, and 1-septate ascospores (to 15 µm long); on siliceous boulders, obligately parasitic on *Tremolecia atrata*, with records from the Eastern Alps only (Austria). – **Au**: K.

***Rhizocarpon schedomyces* Hafellner & Poelt**

L – Subs.: sil-par – Alt.: 4–5 – Note: a species with a mainly endothalline thallus visible around the apothecia as a greyish-brownish discoloration and 1-septate, pigmented ascospores; on weakly calciferous schists or basic siliceous rocks, obligately parasitic on *Pertusaria* (*e.g. P*. *pseudocorallina*), forming minute insular patches; so far only known from the Eastern Alps (Austria). – **Au**: T, K, St.

***Rhizocarpon simillimum* (Anzi) Lettau**

Syn.: *Buellia simillima* Anzi, *Catocarpus simillimus* (Anzi) Arnold, *Rhizocarpon atroalbum* (Nyl.) Zahlbr., *Rhizocarpon sublestum* (Nyl.) Zahlbr.

L – Subs.: sil – Alt.: 2–5 – Note: on steeply inclined surfaces of siliceous, base-rich or slightly calciferous rocks in upland areas. – **Au**: ?V, T, S, K, St. **Sw**: VS. **Fr**: HAl, AMa, HSav. **It**: Frl, TAA, Lomb, Piem, VA.

***Rhizocarpon sorediosum* Runemark**

L – Subs.: met, sil – Alt.: 3–5 – Note: closely related and similar to *Rh. ridescens*, but thallus less vivid yellow-green, with mostly flat areoles; on heavy metals-bearing siliceous rocks in upland areas; probably overlooked, being almost always sterile; in the study area so far reported from the Eastern Alps (Austria, Italy). – **Au**: ?V, ?T, S, St. **It**: Lomb.

***Rhizocarpon subgeminatum* Eitner**

Syn.: *Rhizocarpon phaeolepis* Vain.

L – Subs.: sil, sil-aqu – Alt.: 3–4 – Note: a species with 2-spored asci and large eumuriform ascospores (mostly longer than 50 µm), with more than 25 cells visible in optical view (with at least 3 longitudinal septa); on siliceous rocks, often near brooks and lakes; for the Alps most records are from the Eastern Alps. – **Au**: V, T, S, K, St. **Fr**: AMa. **Sl**: SlA.

***Rhizocarpon sublavatum* Fryday**

L – Subs.: sil-aqu – Alt.: 4 – Note: a species resembling *Rh. reductum* in the small apothecia and the eumuriform ascospores, but thallus with flat areoles, lacking lichen substances; on damp siliceous rocks, often along streams, occasionally together with *R. lavatum* (with larger areoles and apothecia); widespread in Europe, from the boreal to the nemoral-alpine zones, with a single record from the Eastern Alps (Austria), but the species was not recognised in earlier times, and might be more widespread in the Alps. – **Au**: K.

***Rhizocarpon submodestum* (Vain.) Vain.**

Syn.: *Rhizocarpon subreductum* (Vain.) Vain., *Rhizocarpon tetramerum* (Vain.) Vain.

L – Subs.: sil – Alt.: 4 – Note: a species resembling *Rh. postumum*, but with a minutely verruculose thallus and constantly 3-septate, first hyaline, later greenish-brownish ascospores; widespread in Northern Europe but altogether rare, with a single record from the Eastern Alps (Austria). – **Au**: St.

***Rhizocarpon subocellatum* (Müll. Arg.) Zahlbr.**

Syn.: *Buellia subocellata* Müll. Arg.

L # – Subs.: sil – Alt.: 3 – Note: a species with a whitish-grey thallus, innate apothecia with greenish-brown epihymenium, and 1-septate, brown ascospores; the generic placement is in need of re-evaluation; on outcrops of calcareous sandstone; only recorded from the Western Alps (France). – **Fr**: HSav.

***Rhizocarpon subpostumum* (Nyl.) Arnold**

Syn.: *Lecidea subpostuma* Nyl.

L # – Subs.: sil, int, cor – Alt.: 3–5 – Note: a silicicolous species resembling *Rh. postumum* in the small apothecia and the small submuriform ascospores, but thallus paler and usually containing gyrophoric acid, exciple in section with Atra-red, reacting K+ purple; widespread in Europe, from the boreal to temperate-alpine zones, mainly under oceanic conditions; from the Alps there are only a few scattered records, but probably the species was not always distinguished. – **Au**: V, T, S, K, St. **Ge**: Ge. **Fr**: Sav. **It**: Piem.

***Rhizocarpon superficiale* (Schaer.) Vain.**

Syn.: *Lecidea superficialis* Schaer., *Rhizocarpon crystalligenum* Lynge, *Rhizocarpon effiguratum auct. non* (Anzi) Th. Fr., *Rhizocarpon scabridum* Räsänen, *Rhizocarpon splendidum* Malme

L – Subs.: sil – Alt.: 4–6 – Note: a mainly arctic-alpine species found on exposed siliceous rocks with a short snow cover, often very abundant in the nival belt of the Alps. – **Au**: V, T, S, K. **Sw**: GR, TI, UR, VS. **Fr**: HAl, Sav, HSav. **It**: Frl, TAA, Lomb, Piem, VA.

***Rhizocarpon tetrasporum* Runemark**

L # – Subs.: sil – Alt.: 3–5 – Note: on siliceous rocks in the mountains; closely related to *Rh. viridiatrum* and *Rh. oportense*, this taxon is worthy of further study. – **It**: Piem, VA.

***Rhizocarpon trapeliicola* M. Brand**

L # – Subs.: int-par – Alt.: 3–4 – Note: a rarely collected species with a pale brown thallus of dispersed, subspherical areoles developing on bleached squamules of *Trapelia glebulosa* and *T. coarctata*, with, convex to subspherical, black-brown apothecia (0.3–0.4 mm in diam.), a dark brown epihymenium and hypothecium, 8-spored asci, and hyaline, submuriform ascospores (12.5–16 × 5.5–7.5 μm) with 2–4 transverssal septa and *c.* 4–7 cells visible in optical section; on various types of schists; rare in Western and Central Europe; the identification of material from Switzerland is not completely certain. – **Sw**: ?SZ.

***Rhizocarpon umbilicatum* (Ramond) Flagey**

Syn.: *Diplotomma calcareum* (Fr.) Flot., *Diplotomma weissii* (Schaer.) A. Massal., *Lecidea calcarea* (Fr.) Hepp *nom.illeg.*, *Lecidea umbilicata* Ramond, *Rhizocarpon calcareum* (Fr.) Anzi, Rhizocarpon calcareum (Fr.) Anzi var. weissii (Schaer.) Th. Fr., *Rhizocarpon pseudospeireum* (Th. Fr.) Lynge, Rhizocarpon umbilicatum (Ramond) Flagey f. pseudospeireum (Th. Fr.) Szatala, *Siegertia calcarea* (Fr.) Körb., *Siegertia pseudospeirea* (Th. Fr.) V. Wirth. *comb. inval.*, *Siegertia umbilicata* (Ramond) V. Wirth

L – Subs.: cal, int – Alt.: 3–6 – Note: a mainly arctic-alpine, circumpolar species found on steeply inclined, often north-facing surfaces of calcareous, more rarely base-rich or slightly calciferous siliceous rocks; widespread throughout the Alps. – **Au**: V, T, S, K, St, O, N. **Ge**: OB, Schw. **Sw**: AP, BE, GL, GR, LU, SZ, TI, UR, UW, VD, VS. **Fr**: AHP, HAl, AMa, Drô, Isè, Sav, HSav, Var, Vau. **It**: Frl, Ven, TAA, Lomb, Piem, VA. **Sl**: SlA, Tg. **Li**.

***Rhizocarpon viridiatrum* (Wulfen) Körb.**

Syn.: *Buellia viridiatra* (Wulfen) H. Olivier, *Diplotomma viridiatrum* (Wulfen) Jatta, *Lecidea viridiatra* (Wulfen) Ach., *Lichen viridiater* Wulfen, *Rhizocarpon subtile* Runemark

L – Subs.: sil, sil-par – Alt.: 1–5 – Note: on basic siliceous rocks with optimum in dry-warm areas, sometimes on roofing tiles, starting the life-cycle on other crustose lichens, with a wide altitudinal range. – **Au**: T, S, K, St, N. **Sw**: BE, VS. **Fr**: HSav, Var, Vau. **It**: Ven, TAA, Lomb, Piem, VA, Lig. **Sl**: SlA.

***Rhizocarpon vorax* Poelt & Hafellner**

L – Subs.: sil-par – Alt.: 4–5 – Note: a rare boreal-montane to nemoral-alpine species resembling *Rh. schedomyces* in the mainly endothalline thallus, but ascospores muriform (with 3–5 transversal septa and 1–2 incomplete longitudinal septa, 10–15 cells visible in optical view); on weakly calciferous schists or basic siliceous rocks, obligately parasitic on *Pertusaria*-species, with a few records from the Eastern Alps only (Austria). – **Au**: ?V, T, S, K.

***Rhizoplaca chrysoleuca* (Sm.) Zopf**

Syn.: *Lecanora chrysoleuca* (Sm.) Ach., *Lecanora rubina* (“Vill.”) Ach., *Lichen chrysoleucus* Sm., *Omphalodina rubina* (“Vill.”) M. Choisy, *Placodium rubinum* (“Vill.”) Müll. Arg., *Squamaria chrysoleuca* (Sm.) Duby, *Squamaria rubina* (“Vill.”) Hoffm.

L – Subs.: sil – Alt.: 3–6 – Note: a widespread holarctic lichen found on birds perching siliceous rocks and boulders, especially in the mountains; most frequent in areas with a dry-subcontinental climate, *e.g.* in the Central Alps. – **Au**: V, T, S, K, St, N. **Sw**: BE, GR, UR, VS. **Fr**: AHP, HAl, AMa, Isè, Sav, HSav. **It**: TAA, Lomb, Piem, VA, Lig.

***Rhizoplaca melanophthalma* (DC.) Leuckert & Poelt**

Syn.: *Lecanora melanophthalma* (DC.) Ramond, *Lecanora subpeltata* Lynge, *Squamaria melanophthalma* DC.

L – Subs.: sil – Alt.: 3–6 – Note: a widespread holarctic lichen found on birds perching over siliceous rocks, especially in the mountains. – **Au**: V, T, S, K, St. **Sw**: BE, GR, VD, VS. **Fr**: AHP, HAl, AMa, Isè, Sav, HSav. **It**: Ven, TAA, Lomb, Piem, VA, Lig.

***Rhizoplaca subdiscrepans* (Nyl.) R. Sant.**

Syn.: *Lecanora subdiscrepans* (Nyl.) Stizenb., ?Squamaria chrysoleuca (Sm.) Duby var. lecanorea Anzi, Squamaria chrysoleuca (Sm.) Duby var. subdiscrepans Nyl.

L – Subs.: sil, int – Alt.: 3–5 – Note: on the top of calciferous or basic siliceous boulders frequently visited by birds, with optimum above treeline. – **Au**: T, K, St. **Fr**: Sav. **It**: Lomb.

***Ricasolia amplissima* (Scop.) De Not. – chloromorph**

Syn.: *Lichen amplissimus* Scop., *Lobaria amplissima* (Scop.) Forssell, *Lobaria glomulifera* (Lightf.) Hoffm., *Lobaria laciniata* (Huds.) Vain., *Parmelia amplissima* (Scop.) Schaer., *Parmelia glomulifera* (Ligthf.) Ach., *Ricasolia glomulifera* (Ligthf.) Nyl., *Sticta amplissima* (Scop.) Rabenh., *Sticta glomulifera* (Lightf.) Delise

L – Subs.: cor, bry – Alt.: 2–3 – Note: a mild-temperate species found on old, isolated deciduous trees in humid areas with high rainfall. – **Au**: V, T, S, K, St, O, N. **Ge**: OB, Schw. **Sw**: BE, FR, GR, SZ, TI, UR, VD, VS. **Fr**: AMa, Drô, Sav, Var. **It**: Frl, Ven, TAA, Lomb, VA. **Sl**: SlA, Tg.

***Ricasolia amplissima* (Scop.) De Not. – cyanomorph**

Syn.: *Cornicularia umhausense* Auersw., *Dendriscocaulon bolacinum* (Ach.) Nyl., *Dendriscocaulon umhausense* (Auersw.) Degel., *Leptogium bolacinum* (Ach.) Nyl., *Polychidium umhausense* (Auersw.) Henssen

L – Subs.: cor – Alt.: 1–3 – Note: on bark of broad-leaved trees and on epiphytic mosses in warm-humid areas. This is the cyanobacterial morph of *R. amplissima*. Besides the obvious differences in morphology, it has a rather different ecology and distribution. – **Au**: T, S, K, St, N. **Sw**: SZ. **Fr**: AHP, AMa, HSav, Var, Vau. **It**: Ven, TAA, Lig.

***Ricasolia virens* (With.) H.H. Blom & Tønsberg**

Syn.: *Lichen herbaceus* Huds., *Lichen laetevirens* Lightf., *Lichen virens* With., *Lobaria herbacea* (Huds.) DC., *Lobaria laetevirens* (Lightf.) Zahlbr., *Lobaria virens* (With.) J.R. Laundon, *Ricasolia herbacea* De Not. *nom.illeg.*, *Sticta herbacea* Ach. *nom.illeg*.

L – Subs.: cor, sax – Alt.: 1–3 – Note: a mild-temperate to humid subtropical species found on old deciduous trees, more rarely on mossy rocks in old, natural, warm-humid forests; very rare, and strongly declining in the Alps. – **Sw**: BE, GR, UR. **Fr**: Isè. **It**: Ven, Lomb. **Sl**: SlA, Tg.

***Rimularia badioatra* (Kremp.) Hertel & Rambold**

Syn.: *Aspicilia badioatra* Kremp., *Aspicilia corrugatula* (Arnold) Hue, *Lecanora badioatra* (Kremp.) Zahlbr., *Lecanora contracta* (Th. Fr.) Zahlbr., *Lecanora corrugatula* (Arnold) Nyl., *Lecanora umbriformis* (Nyl.) Grummann, *Lecidea badioatra* (Kremp.) Arnold, *Lecidea corrugatula* Arnold, *Lecidea illita* Nyl., *Lecidea umbonatula* Nyl., *Lecidea umbriformis* Nyl., *Mosigia illita* (Nyl.) R. Sant.

L – Subs.: sil, int – Alt.: 3–4 – Note: on steeply inclined surfaces of hard, base-rich or weakly calciferous siliceous rocks, mostly in upland areas, but rarely also occurring above treeline. – **Ge**: Schw. **Sw**: GR, UR, VS. **It**: TAA.

***Rimularia gibbosa* (Ach.) Coppins, Hertel & Rambold**

Syn.: *Aspicilia bockii* (Fr.) Boistel, *Lecanora bockii* (Fr.) Rabenh., *Lecanora grimseleana* A. Massal., *Mosigia gibbosa* (Ach.) A. Massal., *Pyrenula gibbosa* Ach.

L – Subs.: sil – Alt.: 2–5 – Note: on steeply inclined surfaces of mineral-rich to basic siliceous rocks wetted by rain, often in seepage tracks, usually in upland areas. The species often produces both apothecia and soredia. – **Au**: V, T, S, K, St, B. **Sw**: BE, GR, TI, UR, VD, VS. **Fr**: HSav. **It**: TAA, Piem. **Sl**: SlA.

***Rimularia limborina* Nyl.**

Syn.: *Lecidea inconcinna* Nyl., *Lecidea limborina* (Nyl.) Lamy, *Lecidea subgyratula* Nyl., *Lecidea trochodes* (Taylor *ex* Leight.) Cromb.

L – Subs.: sil – Alt.: 4 – Note: a species with a thin, whitish to brown thallus and minute, lecideine apothecia with often umbonate discs, prominent, radially incised margins, and simple, broadly ellipsoid (to 30 µm long) ascospores turning brown with age; on siliceous rocks in irrigated places or other damp situations; widespread in the Holarctic region, in Europe most common in the west and more or less restricted to the montane belt; from the Alps there is only a single record (Switzerland). – **Sw**: GR.

***Rinodina alba* Metzler *ex* Arnold**

Syn.: *Lecanora albidorimulosa* Harm., *Lecanora michaudiana* Harm., *Rinodina albidorimulosa* (Harm.) Zahlbr., *Rinodina michaudiana* (Harm.) Croz., *Rinodina subcanella* Zahlbr.

L – Subs.: sil – Alt.: 1–3 – Note: on hard siliceous rocks near the shoreline, but sometimes also found at some distance from the coast; in the study area only known from the Western Alps (France); an earlier record from Switzerland (VS) has been excluded, as it is most probably wrong. – **Fr**: AMa.

***Rinodina albana* (A. Massal.) A. Massal.**

Syn.: *Berengeria albana* (A. Massal.) Trevis., *Hagenia albana* A. Massal., *Psora horiza* *sensu* Hepp, Rinodina horiza (Mudd) Müll. Arg. var. albana (A. Massal.) Körb., Rinodina sophodes (Ach.) A. Massal. var. albana (A. Massal.) Bagl. & Carestia

L – Subs.: cor – Alt.: 2–3 – Note: a temperate species found on isolated deciduous trees with more or less smooth bark. – **Au**: V, T, S, K, St, O, N. **Ge**: OB. **Sw**: BE, GL, GR, SZ, TI, UR, VD, VS. **Fr**: Isè, HSav. **It**: Ven, TAA, Lomb, Piem, Lig. **Sl**: SlA.

***Rinodina anomala* (Zahlbr.) H. Mayrhofer & Giralt**

Syn.: *Buellia anomala* Zahlbr.

L – Subs.: cor – Alt.: 2 – Note: a mainly western species growing on the branches of broad-leaved trees (*Quercus*, *Ulmus*, etc.), mostly at low elevations, with a single record from the Western Alps (Italy). – **It**: Piem.

***Rinodina archaea* (Ach.) Arnold**

Syn.: *Lecanora archaea* (Ach.) Harm., Parmelia sophodes (Ach.) Ach. var. archaea Ach., *Rinodina lecideoides* (Nyl.) Kernst.

L – Subs.: xyl, cor, sil – Alt.: 2–4 – Note: typically lignicolous but also rarely on rough bark and even more rarely on siliceous rocks. – **Au**: T, S, K, St. **Ge**: OB. **Sw**: VS. **Fr**: HAl, AMa, HSav. **It**: TAA, Lomb.

***Rinodina aspersa* (Borrer) J.R. Laundon**

Syn.: *Buellia aspersa* (Borrer) P. James, *Lecanora aspersa* Borrer, Rinodina atrocinerea (Hook.) Körb. var. fatiscens (Th. Fr.) Clauzade & Cl. Roux, Rinodina exigua (Ach.) Gray f. fatiscens Th. Fr., *Rinodina fatiscens* (Th. Fr.) Vain.

L – Subs.: sil – Alt.: 2–3 – Note: on hard siliceous rocks near the ground in cold-humid habitats, sometimes on walls, mostly below the montane belt; in the study area so far recorded from a single station at the limit of the Southern Pre-Alps (France). – **Fr**: Vau.

***Rinodina atrocinerea* (Hook.) Körb.**

Syn.: *Lecanora plumbella* Nyl., *Lecidea atrocinerea* Hook., Rinodina aspersa (Borrer) J.R. Laundon subsp. atrocinerea (Hook.) Cl. Roux, *Rinodina plumbella* (Nyl.) H. Olivier, *Rinodina tympanelloides* Arnold

L – Subs.: sil – Alt.: 1–3 – Note: on steeply inclined to vertical surfaces of hard siliceous rocks, below the subalpine belt. – **Au**: S, St. **Sw**: SZ, VS. **Fr**: Sav, HSav, Var. **It**: Ven, Lomb, Piem, Lig.

***Rinodina beccariana* Bagl.**

Syn.: Lecanora confragosa (Ach.) Röhl. f. dispersa B. de Lesd. *ex* Harm., Lecanora confragosa (Ach.) Röhl. f. glaucescens Nyl., Lecanora confragosa (Ach.) Röhl. var. fumosa Wedd., Lecanora confragosa (Ach.) Röhl. var. turgida Wedd., Rinodina beccariana Bagl. var. cinerea Bagl., Rinodina beccariana Bagl. var. tympanelloides Bagl., *Rinodina bimarginata* Zahlbr., Rinodina confragosa (Ach.) Körb. f. dispersa (B. de Lesd.) Zahlbr., Rinodina confragosa (Ach.) Körb. var. fumosa (Wedd.) H. Olivier, Rinodina confragosa (Ach.) Körb. var. subglaucescens (Nyl.) H. Olivier, Rinodina confragosa (Ach.) Körb. var. turgida (Wedd.) Boistel, *Rinodina subglaucescens* (Nyl.) Sheard

L – Subs.: sil – Alt.: 1–2 – Note: a mainly Mediterranean-Atlantic lichen of siliceous rocks, with a single record from the base of the Western Alps (Italy). – **It**: Lig.

***Rinodina bischoffii* (Hepp) A. Massal.**

Syn.: *Berengeria bischoffii* (Hepp) Trevis., Lecanora exigua (Ach.) Fr. subsp. subrubescens Vain., *Psora bischoffii* Hepp, Rinodina bischoffii (Hepp) A. Massal. f. guttulata Servít & Nádv., Rinodina bischoffii (Hepp) A. Massal. f. lecideina (Nyl.) Boistel, Rinodina bischoffii (Hepp) A. Massal. f. obscurata J. Steiner, Rinodina bischoffii (Hepp) A. Massal. var. convexula Flagey, Rinodina bischoffii (Hepp) A. Massal. var. protuberans Körb., *Rinodina colletica* *sensu* Lettau *non* (Flörke *ex* Körb.) Arnold, *Rinodina nigrella* Müll. Arg., *Rinodina orcularia* H. Mayrhofer & Poelt, *Rinodina subrubescens* (Vain.) Zahlbr.

L – Subs.: cal, int – Alt.: 1–5 – Note: a widespread early coloniser of calciferous or rarely basic siliceous rocks, also found on walls, roofing tiles etc.; common throughout the Alps. – **Au**: V, T, S, K, St, O, N. **Ge**: OB. **Sw**: BE, GR, LU, SG, SZ, TI, UR, VD, VS. **Fr**: AHP, HAl, AMa, Drô, Isè, Sav, HSav, Var, Vau. **It**: Frl, Ven, TAA, Lomb, Piem, VA, Lig. **Sl**: Tg.

***Rinodina calcarea* (Hepp *ex* Arnold) Arnold**

Syn.: *Lecanora calcarea* (Hepp *ex* Arnold) Harm. var. ampsagana Stizenb., Lecanora confragosa (Ach.) Röhl. var. glebulosa Harm., *Rinodina caesiella* (Flörke *ex* Spreng.) Körb. var. calcarea Hepp *ex* Arnold, *Rinodina calcarea* (Hepp *ex* Arnold) Arnold var. ampsagana (Stizenb.) Zahlbr., *Rinodina calcarea* (Hepp *ex* Arnold) Arnold var. melanocarpa J. Steiner, *Rinodina calcarea* (Hepp *ex* Arnold) Arnold var. nummulitica Flagey, Rinodina confragosa (Ach.) Körb. var. glebulosa (Harm.) Zahlbr.

L – Subs.: cal, int – Alt.: 1–4 – Note: on the top of sun-exposed boulders of dolomite, limestone and calcareous schists, with a rather wide altitudinal range. – **Au**: V, T, S, K, St, N. **Ge**: OB. **Sw**: LU. **Fr**: AHP, HAl, AMa, Isè, Var, Vau. **It**: Ven, TAA, VA, Lig.

***Rinodina cana* (Arnold) Arnold**

Syn.: Rinodina arenaria (Hepp) Arnold var. cana Arnold, *Rinodina lesdainii* Samp.

L – Subs.: sil, int – Alt.: 2–3 – Note: on steeply inclined, weakly calcareous schists and basic siliceous rocks in dry-warm areas. – **Au**: K, St. **Sw**: GR, TI. **It**: TAA, Piem.

***Rinodina candidogrisea* Hafellner, Muggia & Obermayer**

L – Subs.: ter-cal, ter-int, deb, bry – Alt.: 4–5 – Note: a recently-described, ornitocoprophilous, terricolous species growing on mosses and plant debris over calcareous substrata near and above treeline; probably more widespread in the Alps. – **Au**: V, K, St, O. **Ge**: OB. **Sw**: GR. **It**: Frl, TAA, Piem. **Sl**: SlA.

***Rinodina capensis* Hampe**

Syn.: *Rinodina corticicola* (Arnold) Dalla Torre & Sarnth., *Rinodina corticola* (Arnold) Arnold, Rinodina teichophila (Nyl.) Arnold var. corticola Arnold

L – Subs.: cor, xyl – Alt.: 3–4 – Note: a cool-temperate to boreal-montane pioneer species, mostly found on smooth bark, but also on lignum; the species, also known from the Canary Islands, is widespread throughout the Alps. – **Au**: V, T, S, K, St, O, N. **Ge**: OB, Schw. **Sw**: BE, FR, GL, GR, LU, SG, SZ, TI, UR, UW, VS. **Fr**: AHP, AMa, HSav, Var. **It**: Frl, TAA, Lomb, Piem. **Sl**: SlA, Tg.

***Rinodina castanomela* (Nyl.) Arnold**

Syn.: *Lecanora castanomela* Nyl.

L – Subs.: int – Alt.: 4–5 – Note: an arctic-alpine to boreal-montane, perhaps circumpolar lichen found under overhanging cliffs of weakly calcareous or basic siliceous rocks, marl and calciferous schists; perhaps more widespread in the Alps. – **Au**: V, T, S, K, St. **Ge**: OB. **Sw**: BE, GR, TI, VD. **Fr**: HAl, Sav. **It**: TAA.

***Rinodina castanomelodes* H. Mayrhofer & Poelt**

Syn.: Rinodina bischoffii (Hepp) A.Massal. subsp. castanomelodes (H. Mayrhofer & Poelt) Cl. Roux, Rinodina bischoffii (Hepp) A. Massal. var. castanomelodes (H. Mayrhofer & Poelt) Giralt & Llimona

L – Subs.: cal, int – Alt.: 4–6 – Note: on limestone, marl and calcareous schists in upland areas; widespread throughout the Alps. – **Au**: V, T, S, K, St. **Ge**: OB, Schw. **Sw**: BE, GR, SZ, TI. **Fr**: AHP, HAl, AMa, Sav, HSav. **It**: Ven, TAA, Lomb, Piem, VA.

***Rinodina cinnamomea* (Th. Fr.) Räsänen**

Syn.: Rinodina mniaroea (Ach.) Körb. var. chrysopasta (Lettau) Zahlbr., Rinodina mniaroea (Ach.) Körb. var. cinnamomea Th. Fr.

L – Subs.: bry, deb, ter-sil, ter-cal – Alt.: 4–5 – Note: on soil, bryophytes, and plant debris in tundra-like environments over more or less calciferous substrata; widespread throughout the Alps. – **Au**: V, T, S, K, St, O. **Ge**: OB, Schw. **Sw**: GR, SZ. **Fr**: AHP, HAl, AMa, Sav. **It**: Ven, TAA, Lomb, Piem. **Sl**: SlA.

***Rinodina clauzadei* H. Mayrhofer & Cl. Roux**

L – Subs.: cal – Alt.: 4 – Note: a rare calcicolous species, in the study area so far reported only from the Western Alps (France). – **Fr**: AHP, HAl.

***Rinodina colobina* (Ach.) Th. Fr.**

Syn.: *Lecanora colobina* Ach., *Rinodina leprosa* A. Massal.

L – Subs.: cor – Alt.: 1–3 – Note: a mild-temperate to Mediterranean lichen found on dust-impregnated bark of isolated trees, especially *Populus*, *Fraxinus*, *Juglans* and *Ulmus*, often on the basal parts of trunks; certainly declining, and presently extinct in several regions, probably due to the disappearance of unpaved roads during this century. – **Au**: V, K, St, N. **Ge**: Schw. **Sw**: GR, TI, VD, VS. **Fr**: AHP, AMa, Isè, Sav, HSav. **It**: Frl, Ven, TAA, Lomb, Piem, Lig. **Sl**: Tg.

***Rinodina conchophylla* H. Mayrhofer & Poelt**

L – Subs.: int, sil – Alt.: 2–5 – Note: on calcareous schists and basic siliceous rocks in warm-dry areas; only reported from South Tyrol (Italy), but perhaps occurring also in other dry valleys of the Alps. – **It**: TAA.

***Rinodina confragosa* (Ach.) Körb.**

Syn.: *Lecanora caesiella* Flörke *ex* Spreng., *Lecanora confragosa* (Ach.) Röhl., Lecanora confragosa (Ach.) Röhl. var. exterior Nyl., Lecanora confragosa (Ach.) Röhl. var. extrusa Vain., *Parmelia confragosa* Ach., *Rinodina aggregata* Bagl., *Rinodina caesiella* (Flörke *ex* Spreng.) Körb., *Rinodina caesiella* (Flörke *ex* Spreng.) Körb. var. aggregata (Bagl.) Arnold, Rinodina confragosa (Ach.) Körb. var. exterior (Nyl.) H. Olivier, Rinodina confragosa (Ach.) Körb. var. extrusa (Vain.) H. Olivier, *Rinodina crassescens* (Nyl.) Arnold, *Rinodina firma* (Nyl.) Arnold, Rinodina metabolica (Ach.) Anzi var. saxicola Anzi, *Rinodina romeana* Müll. Arg., *Rinodina samothrakiana* Szatala

L – Subs.: sil – Alt.: 2–5 – Note: a cool-temperate to boreal-montane, circumpolar lichen found on vertical or underhanging surfaces of hard siliceous rocks protected from rain, exceptionally reaching beyond treeline in dry-warm areas; the species is chemically variable; widespread throughout the Alps. – **Au**: V, T, S, K, St, N. **Ge**: Ge. **Sw**: BE, GR, UR, VD, VS. **Fr**: AHP, HAl, AMa, Isè, Sav, HSav, Var, Vau. **It**: Ven, TAA, Lomb, Piem, Lig.

***Rinodina conradii* Körb.**

L – Subs.: cor, xyl, bry, ter-sil, deb, bry-sil – Alt.: 2–5 – Note: a widespread, short-lived early coloniser of base-rich soil and terricolous bryophytes in open habitats, sometimes on mosses on basal parts of ancient trees, rarely lignicolous or corticolous. – **Au**: V, T, S, K, St, O, N. **Ge**: Ge. **Sw**: GR, SZ, TI, UR, VS. **Fr**: Sav, HSav. **It**: Frl, Ven, TAA, Lomb, Piem. **Sl**: SlA.

***Rinodina dubyana* (Hepp) J. Steiner**

Syn.: *Buellia dubyana* (Hepp) Rabenh., Lecanora bischoffii (Hepp) Nyl. var. mediterranea Stizenb., Lecanora bischoffii (Hepp) Nyl. var. melanops (Müll. Arg.) Stizenb., *Lecanora mediterranea* (Stizenb.) Harmand, *Lecidea dubyana* Hepp, Rinodina bischoffii (Hepp) A. Massal. var. mediterranea (Stizenb.) Flagey, Rinodina bischoffii (Hepp) A. Massal. var. melanops Müll. Arg., *Rinodina mediterranea* (Stizenb.) Flagey

L – Subs.: cal – Alt.: 1–4 – Note: a mainly temperate species found on steeply inclined to underhanging, sunny surfaces of limestone and dolomite, sometimes also on pebbles on the ground; widespread throughout the Alps. – **Au**: V, T, St, O, N. **Sw**: BE, UR, VD, VS. **Fr**: AHP, AMa, Drô, Isè, Sav, HSav, Var, Vau. **It**: Frl, Ven, TAA, Piem. **Sl**: SlA.

***Rinodina efflorescens* Malme**

Syn.: *Lecanora hueiana* Harm., *Rinodina hueiana* (Harm.) H. Olivier

L – Subs.: cor – Alt.: 2–3 – Note: a rare, mild-temperate, suboceanic species found on twigs and boles of deciduous trees, especially *Quercus* and *Fagus*, in open, moist deciduous woodlands. – **Au**: T, K, St, O, N. **Ge**: OB, Schw. **It**: Ven. **Sl**: Tg.

***Rinodina epimilvina* H. Mayrhofer**

L – Subs.: sil-par – Alt.: 3–5 – Note: on acidic siliceous rocks wetted by rain in upland areas, starting the life-cycle on *Rinodina milvina*; not common, but certainly more widespread in the Alps than the few records would suggest, with optimum above treeline. – **Sw**: BE. **It**: TAA.

***Rinodina excrescens* Vain.**

L – Subs.: cor – Alt.: 3 – Note: a rare species of acid bark, with a primarily Eastern North American-Eastern Asian distribution and scattered outliers elsewhere, including the Eastern Alps (Austria). – **Au**: St, N.

***Rinodina exigua* (Ach.) Gray**

Syn.: *Berengeria exigua* (Ach.) Trevis., *Lichen exiguus* Ach., *Psora exigua* (Ach.) Nägeli, *Rinodina kornhuberi* Zahlbr., *Rinodina metabolica auct. p.p. non* (Ach.) Anzi, *Rinodina ramulicola* Kernst.

L – Subs.: cor, xyl – Alt.: 1–4 – Note: a temperate species found on the smooth bark of isolated trees, more rarely on rather eutrophicated wood; common throughout the Alps. – **Au**: V, T, S, K, St, O, N, B. **Ge**: OB. **Sw**: BE, FR, GL, GR, LU, SZ, TI, UR, UW, VD, VS. **Fr**: AHP, AMa, Drô, Isè, Sav, HSav, Var, Vau. **It**: Frl, Ven, TAA, Lomb, Piem, VA, Lig. **Sl**: SlA.

***Rinodina ficta* (Stizenb.) Zahlbr.**

Syn.: *Lecanora ficta* Stizenb., *Rinodina boleana* Giralt & H. Mayrhofer

L – Subs.: cor – Alt.: 1–2 – Note: on evergreen broad-leaved trees in parklands, waysides, and in open maquis or woodlands; also known from South Africa, New Zealand, North America, the Iberian Peninsula, Greece and Croatia, this species is probably more widespread in S Europe, especially in the Mediterranean belt; for the Alps there is a single record from the base of the Eastern Pre-Alps (Italy). – **It**: Frl.

***Rinodina fimbriata* Körb.**

Syn.: Rinodina confragosa (Ach.) Körb. var. inundata (Blomb. *ex* Th. Fr.) H. Olivier, Rinodina exigua (Ach.) Gray var. inundata Blomb. *ex* Th. Fr.

L – Subs.: sil-aqu – Alt.: 2–3 – Note: a very rarely collected species found on periodically inundated siliceous rocks in mountain creeks and rivers; in the study area known only from the Western Alps (France). – **Fr**: AMa.

***Rinodina freyi* H. Magn.**

Syn.: *Rinodina glauca* Ropin, *Rinodina ramulicola* Kernst. *ex* Arnold *nom.illeg*.

L – Subs.: cor, xyl – Alt.: 2–4 – Note: on shrubs and trees in open situations, also on lignum. The species was often treated together with *R. glauca* as a synonym of *R. septentrionalis*. – **Au**: V, T, S, K, St, O, N. **Ge**: OB. **Sw**: BE, GL, GR, SZ, VS. **It**: Ven, TAA, Lomb, Piem, VA. **Sl**: SlA.

***Rinodina furfurea* H. Mayrhofer & Poelt**

L – Subs.: sil – Alt.: 3–4 – Note: known from a few collections, on exposed siliceous rocks in very dry sites, this interesting species needs further study. – **It**: TAA.

***Rinodina griseosoralifera* Coppins**

L – Subs.: cor – Alt.: 2–4 – Note: a species found on trunks of broad-leaved trees, often near the base, sometimes invading epiphytic mosses; easy to overlook, being mostly sterile, and probably more widespread in the Alps. – **Au**: V, T, S, K, St, O, N. **Ge**: OB, Schw. **Sw**: BE, FR, GL, GR, LU, SG, SZ, UW, VS. **It**: Frl, Lig.

***Rinodina guzzinii* Jatta**

Syn.: Rinodina bischoffii (Hepp) A. Massal. var. ochrata J. Steiner, Rinodina controversa A. Massal. var. terricola Flagey, *Rinodina miocenensis* Flagey

L – Subs.: cal – Alt.: 1–3 – Note: an Irano-Turanian-Mediterranean species found on more or less horizontal, exposed surfaces of weakly calciferous rocks, most frequent in dry-warm areas below treeline, with a few records, mainly from the Southern and Western Alps. – **Sw**: VS. **Fr**: AMa. **It**: Frl, Piem, Lig.

***Rinodina immersa* (Körb.) J. Steiner**

Syn.: Rinodina bischoffii (Hepp) A. Massal. var. exigua Müll. Arg., Rinodina bischoffii (Hepp) A. Massal. var. immersa Körb., Rinodina bischoffii (Hepp) A. Massal. var. intermedia Müll. Arg., Rinodina bischoffii (Hepp) A. Massal. var. ochracea Müll. Arg., Rinodina bischoffii (Hepp) A. Massal. var. perexigua Müll. Arg.

L – Subs.: cal – Alt.: 1–5 – Note: a mainly southern species found on horizontal to steeply inclined, dry surfaces of limestone and (more rarely) dolomite wetted by rain, but also on pebbles, exceptionally reaching the alpine belt; widespread throughout the Alps. – **Au**: V, T, S, K, St, O, N. **Ge**: OB. **Sw**: BE, GR, LU, SZ, VD, VS. **Fr**: AHP, HAl, AMa, Drô, Sav, HSav, Var, Vau. **It**: Frl, Ven, TAA, Lomb, Lig. **Sl**: SlA.

***Rinodina inflata* Kalb**

L # – Subs.: cor – Alt.: 3 – Note: a rare sorediate species, only known from the type locality in the Austrian Alps. – **Au**: S.

***Rinodina intermedia* Bagl.**

Syn.: *Rinodina conimbricensis* Samp., *Rinodina lusitanica* Arnold

L – Subs.: ter-sil, bry-sil – Alt.: 1–2 – Note: a widespread species reported from Asia, Central and South America, Europe and Africa, including the Canary Islands and the Cape Verde Islands, found on soil and mosses over basic siliceous substrata, in open grasslands and garrigue vegetation, with a few records, mainly from the Southern and Western Alps. – **Sw**: TI. **It**: TAA, Lomb, Lig.

***Rinodina isidioides* (Borrer) H. Olivier**

Syn.: *Parmelia isidioides* Borrer

L – Subs.: cor – Alt.: 2–3 – Note: a mild-temperate, probably Mediterranean-Atlantic species found on rough bark, more rarely on epiphytic mosses, in ancient, undisturbed forests, with a few records from the Central Alps (Switzerland). – **Sw**: GR, TI.

***Rinodina lecanorina* (A. Massal.) A. Massal.**

Syn.: *Berengeria lecanorina* (A. Massal.) Trevis., Lecanora controversa (Trevis.) Nyl. var. numida Stizenb., Lecanora sophodes (Ach.) Ach. var. pictavica Wedd., *Lecidea lecanorina* (A. Massal.) Nyl., *Mischoblastia lecanorina* A. Massal., Rinodina controversa A. Massal. var. numida (Stizenb.) Zahlbr., Rinodina lecanorina (A. Massal.) A. Massal. var. pictavica (Wedd.) H. Olivier, *Rinodina ocellata* (Hoffm.) Arnold *non* (Flot.) Branth & Rostr., Rinodina sophodes (Ach.) A. Massal. var. pictavica (Wedd.) Zahlbr., *Verrucaria ocellata* Hoffm.

L – Subs.: cal – Alt.: 1–3 – Note: on the top of isolated boulders of limestone and dolomitic rocks, usually on nutrient-enriched surfaces such as in birds’ perching sites, with a wide altitudinal range but usually absent above treeline; widespread throughout the Alps. – **Au**: T, S, K, St, O, N. **Sw**: GR, SZ, VD, VS. **Fr**: AHP, HAl, AMa, Isè, Sav, HSav, Var, Vau. **It**: Frl, Ven, TAA, Lomb, Piem, VA, Lig.

***Rinodina luridata* (Körb.) H. Mayrhofer, Scheid. & Sheard subsp. luridata**

Syn.: *Buellia luridata* Körb., *Rinodina iodes* H. Mayrhofer & Poelt

L – Subs.: int, cal – Alt.: 3–5 – Note: on sun-exposed calcareous outcrops in upland areas. – **Au**: V, T, S, K, St, N. **Fr**: HSav, Vau.

***Rinodina luridata* (Körb.) H. Mayrhofer, Scheid. & Sheard subsp. immersa (H. Mayrhofer & Cl. Roux) Cl. Roux**

Syn.: Rinodina iodes H. Mayrhofer & Poelt var. immersa H. Mayrhofer & Cl. Roux

L – Subs.: cal – Alt.: 2 – Note: this taxon differs from the nominal subspecies in the endolithic thallus and the ecology (on hard, compact limestones, often with *Bagliettoa marmorea*); for the study area there are a few records from the Western Alps only (France), but the species could have not been distinguished elsewhere. – **Fr**: AMa, Drô, Var, Vau.

***Rinodina luridescens* (Anzi) Arnold**

Syn.: *Buellia coniopta* (Nyl.) Malme, *Buellia luridescens* Anzi, *Buellia sciodes* (Nyl.) Boistel, *Lecanora coniopta* Nyl., *Lecanora sciodes* Nyl., *Lecidea coniopta* (Nyl.) Wedd., *Rinodina coniopta* (Nyl.) Hav., *Rinodina sciodes* (Nyl.) H. Olivier

L – Subs.: sil – Alt.: 1–3 – Note: a Mediterranean-Atlantic lichen described from Tuscany, found on hard siliceous rocks subject to frequent humid winds, often near the coast, also reported from the base of the Western Alps (France). – **Fr**: AMa.

***Rinodina malangica* (Norman) Arnold**

Syn.: Rinodina leprosa A. Massal. var. malangica Norman, *Rinodina rhododendri* Hepp *ex* H. Magn., Rinodina sophodes (Ach.) A. Massal. var. malangica (Norman) Th. Fr.

L – Subs.: cor, deb – Alt.: 3–4 – Note: a species known from the Central European mountains, the Pyrenees, Norway and the Canary Islands, found on shrubs (often on *Rhododendron*) in the subalpine belt, especially on the basal parts of stems, where it can be very abundant, sometimes on lignum; probably widespread throughout the Alps. – **Au**: V, T, S, K, St, O. **Ge**: Schw. **Sw**: BE, GL, GR, LU, SZ, TI, VS. **It**: Frl, TAA, Piem.

***Rinodina mayrhoferi* A. Crespo**

L – Subs.: cor – Alt.: 1–3 – Note: a rare corticolous species; in the study area it was reported only from the Western Alps (France). – **Fr**: HAl.

***Rinodina milvina* (Wahlenb.) Th. Fr.**

Syn.: *Lecanora milvina* (Wahlenb.) Ach., Lecanora sophodes (Ach.) Ach. var. scopulina Nyl., Lecanora sophodes (Ach.) Ach. var. submilvina Nyl., *Lecanora subconfragosa* Nyl., *Lecanora submilvina* (Nyl.) Croz., *Parmelia milvina* Wahlenb., *Rinodina arnoldii auct. non* H. Mayrhofer & Poelt, Rinodina milvina (Wahlenb.) Th. Fr. var. karelica Räsänen, Rinodina sophodes (Ach.) A. Massal. f. saxicola Kernst., Rinodina sophodes (Ach.) A. Massal. var. scopulina (Nyl.) Croz., *Rinodina subconfragosa* (Nyl.) Flagey

L – Subs.: sil, int – Alt.: 3–5 – Note: a cool-temperate to arctic-alpine, circumpolar lichen found on boulders of base-rich to weakly calciferous siliceous rocks, usually on steeply inclined surfaces, often (but not always) parasitic on other crustose lichens, with optimum near or above treeline; widespread throughout the Alps. – **Au**: V, T, S, K, St, N. **Sw**: BE, GR, SZ, TI, UR, VD, VS. **Fr**: AHP, HAl, AMa, Sav, HSav. **It**: Frl, Ven, TAA, Lomb, Piem, VA, Lig.

***Rinodina mniaroea* (Ach.) Körb.**

Syn.: *Lecanora amniocola* Ach., *Lecanora mniaroea* Ach., *Pachysporaria mniaroea* (Ach.) M. Choisy, *Rinodina amniocola* (Ach.) Körb., Rinodina mniaroea (Ach.) Körb. var. normalis Th. Fr., Rinodina turfacea (Wahlenb.) Körb. var. nuda Körb.

L – Subs.: bry, deb, ter-sil, ter-cal – Alt.: 4–6 – Note: an arctic-alpine, circumpolar species found on soil, bryophytes, and plant debris in tundra-like environments; widespread throughout the Alps. – **Au**: V, T, S, K, St, O, N. **Ge**: OB, Schw. **Sw**: BE, FR, GR, LU, TI, UR, UW, VD, VS. **Fr**: AHP, HAl, AMa, Isè, Sav, HSav, Vau. **It**: Frl, Ven, TAA, Lomb, Piem, VA. **Sl**: SlA.

***Rinodina mniaroeiza* (Nyl.) Arnold**

Syn.: *Diploicia muscorum* *sensu* A. Massal. *non Lichen muscorum* Wulfen, *Lecanora mniaroeiza* Nyl., Rinodina mniaroea (Ach.) Körb. var. mniaroeiza (Nyl.) H. Magn.

L – Subs.: bry, deb, ter-cal – Alt.: 4–5 – Note: on mainly calcareous soil, bryophytes, plant debris, in tundra-like environments; perhaps more widespread in the Alps. – **Au**: V, T, S, K, St, O, N. **Ge**: OB, Schw. **Sw**: BE, FR, GR, SZ, UW. **Fr**: AHP, HAl, AMa, Sav, HSav. **It**: TAA, Lomb.

***Rinodina moziana* (Nyl.) Zahlbr.**

Syn.: *Lecidea destituta* Nyl., *Lecanora moziana* Nyl., Rinodina atrocinerea (Hook.) Körb. var. nigrocaerulescens (Wedd.) H. Olivier, Rinodina confragosa (Ach.) Körb. var. nigrocaerulescens (Wedd.) Boistel, *Rinodina destituta* (Nyl.) Zahlbr., *Rinodina vezdae* H. Mayrhofer

L – Subs.: sil, sil-aqu – Alt.: 1–3 – Note: a species known from North America, Central and Southern Europe, the Canary Islands, Eastern Asia, Australia, New Zealand and the island Juan Fernandez found on basic siliceous rocks, such as magmatite; closely related to *R. oxydata*. – **Au**: K. **Fr**: Sav, Var. **It**: Lig.

***Rinodina nivalis* H. Mayrhofer**

L – Subs.: cal – Alt.: 5 – Note: this rare species is only known from the Eastern Alps (Italy), on dolomite. – **It**: TAA.

***Rinodina notabilis* (Lynge) Sheard**

Syn.: *Buellia notabilis* Lynge, *Buellia parvula* (H. Mayrhofer & Poelt) H. Mayrhofer & Scheid., *Rinodina parvula* H. Mayrhofer & Poelt

L – Subs.: cal, int – Alt.: 3–5 – Note: on more or less calciferous rocks in upland areas; the species is known also from Slovakia, Spain, and North America. – **Au**: T, S, K, St. **Sw**: BE, GR, TI. **Fr**: AMa. **It**: TAA.

***Rinodina obnascens* (Nyl.) H. Olivier**

Syn.: *Lecanora obnascens* Nyl.

L – Subs.: sil-par – Alt.: 1–3 – Note: a Mediterranean-Atlantic lichen found on weakly inclined to horizontal surfaces of siliceous rocks wetted by rain, starting the life-cycle especially on *Aspicilia intermutans*, but sometimes on other lichens, *e.g. Rhizocarpon*-species. The records from Austria and Switzerland are highly questionable and need to be checked. – **Au**: ?T. **Sw**: ?GR, ?VS. **Fr**: AHP, Var, Vau. **It**: Lig.

***Rinodina occulta* (Körb.) Sheard**

Syn.: *Buellia occulta* Körb., *Lecanora tegulicola* Nyl., *Rinodina diplocheila* Vain. *ex* H. Magn., *Rinodina tegulicola* (Nyl.) J. Steiner, *Rinodina verrucarioides* H. Magn.

L – Subs.: sil – Alt.: 1–4 – Note: on vertical to underhanging surfaces of hard siliceous rocks, with a few scattered records only from the Alps. – **Au**: K. **Fr**: AMa. **It**: VA, Lig. **Sl**: SlA.

***Rinodina oleae* Bagl.**

Syn.: *Lecanora subexigua* Nyl., *Rinodina agavicola* Erichsen, *Rinodina cinerascens* J. Steiner, *Rinodina demissa auct.*, Rinodina exigua (Ach.) Gray var. glauca H. Magn., *Rinodina gennarii* Bagl., *Rinodina pallida* H. Magn., *Rinodina salina* Degel., *Rinodina subexigua* (Nyl.) H. Olivier

L – Subs.: cal, sil, int, cor, xyl – Alt.: 1–3 – Note: corticolous specimens only occur in the Mediterranean belt, while saxicolous specimens, usually named *R. gennarii*, are more frequent in inland sites, such as in the Alps. – **Au**: ?V, T, St, N, B. **Ge**: OB. **Sw**: GR, LU, SZ, TI, VS. **Fr**: HAl, AMa, Isè, Sav, Var, Vau. **It**: Frl, Ven, TAA, Lomb, Piem, VA, Lig.

***Rinodina olivaceobrunnea* C.W. Dodge & G.E. Baker**

Syn.: Rinodina archaea (Ach.) Arnold f. minuta Anzi *ex* Arnold, *Rinodina archaeoides* H. Magn., *Rinodina soredicola* Degel.

L – Subs.: bry, deb, ter – Alt.: 3–5 – Note: an arctic-alpine, circumpolar species found on soil, bryophytes and plant debris in tundra-like environments over siliceous substrata; widespread throughout the Alps. – **Au**: V, T, S, K, St. **Ge**: OB. **Sw**: GL, GR, SG, TI, UR, VS. **Fr**: AHP, HAl, AMa, HSav. **It**: Ven, TAA, Lomb.

***Rinodina orculata* Poelt & M. Steiner**

Syn.: Rinodina exigua (Ach.) Gray var. corticicola Anzi, *Rinodina trevisanii auct. non* (Hepp) Körb.

L – Subs.: cor, xyl – Alt.: 3–5 – Note: on the bark of conifers and subalpine shrubs, especially common on *Rhododendron*, mostly near treeline; widespread throughout the Alps. – **Au**: V, T, S, K, St, O, N. **Ge**: Schw. **Sw**: BE, GL, GR, LU, SZ, TI, UR, UW, VS. **Fr**: HSav. **It**: Frl, TAA, Lomb, Piem, VA. **Sl**: SlA.

***Rinodina oxydata* (A. Massal.) A. Massal.**

Syn.: *Buellia discolor* Arnold, Buellia discolor Arnold var. candida (Schaer.) Anzi, *Buellia griseonigra* (Nyl.) Zahlbr., Lecanora confragosa (Ach.) Röhl. var. lecideotropa Nyl., *Lecanora contribuens* Nyl., *Lecanora dissentanea* Nyl., *Lecanora griseofusca* Nyl., *Lecanora intuta* Nyl., *Lecidea discolor* Hepp, *Lecidea griseonigra* Nyl., *Mischoblastia oxydata* A. Massal., *Rinodina aequalis* (Nyl.) Zahlbr., *Rinodina biatorina* Körb., *Rinodina candida* (Schaer.) Arnold, *Rinodina contribuens* (Nyl.) Boistel, *Rinodina discolor* (Arnold) Arnold, *Rinodina dissimilis* Anzi, *Rinodina griseofusca* (Nyl.) H. Olivier, *Rinodina griseonigra* (Nyl.) Zahlbr., *Rinodina intuta* (Nyl.) H. Olivier, *Rinodina lecideotropa* (Nyl.) Zahlbr., *Rinodina subarenaria* A.L. Sm.

L – Subs.: sil – Alt.: 2–3 – Note: a temperate to tropical, widespread species known from from southern Africa, Asia, Australia, North and South America, Macaronesia and Europe, found on seepage tracks of (mostly) base-rich, hard, smooth metamorphic rocks, often along mountain creeks. – **Au**: S, K, St, N, B. **Ge**: Schw. **Sw**: BE, GR, SG, TI. **Fr**: AMa, Sav, HSav, Var. **It**: Ven, TAA, Lomb, Piem, VA. **Sl**: SlA.

***Rinodina papillata* H. Magn.**

L – Subs.: cor – Alt.: 3 – Note: this epiphytic species was recently reported as new to Europe from an oak forest of South Tyrol. – **It**: TAA.

***Rinodina parasitica* H. Mayrhofer & Poelt**

Syn.: Rinodina milvina (Wahlenb.) Th. Fr. var. amphibolitica Räsänen

L – Subs.: sil-par, sil, int-par – Alt.: 3–5 – Note: an arctic-alpine species found on siliceous rocks in upland areas, often growing on the thalli of other crustose lichens; easy to overlook and certainly more widespread in the Alps. – **Au**: T, S, K, St. **Ge**: Schw. **Sw**: GR, TI, UR, VS. **It**: Frl.

***Rinodina pityrea* Ropin & H. Mayrhofer**

L – Subs.: cor, cal – Alt.: 2–3 – Note: a temperate species found on asbestos-cement and mortar, often on walls or on the base of rough barked deciduous trees; probably more widespread in the Alps, but easy to overlook, being often sterile. – **Au**: St, N. **Ge**: OB. **It**: Ven.

***Rinodina plana* H. Magn.**

L – Subs.: cor – Alt.: 1–3 – Note: an early coloniser of smooth bark, especially of young twigs. – **Au**: V, T, K, St. **Fr**: HAl, Vau. **Sl**: SlA.

***Rinodina poeltiana* Giralt & Obermayer**

L # – Subs.: cor – Alt.: 3 – Note: a rather poorly known epiphytic species. The type material, from Austria, was growing on *Salix alba*. – **Au**: St. **It**: TAA.

***Rinodina polyspora* Th. Fr.**

Syn.: *Berengeria polyspora* (Th. Fr.) Trevis., *Buellia polysporella* (Nyl.) Arnold, *Lecanora polyspora* (Th. Fr.) Nyl., *Lecidea polysporella* Nyl.

L – Subs.: cor – Alt.: 2–3 – Note: a temperate species found on smooth bark, especially of *Fraxinus*, *Sorbus* and *Carpinus* in open woodlands; rather widespread in the Alps, but perhaps declining. – **Au**: V, T, S, K, St. **Fr**: AHP. **Ge**: Schw. **Sw**: GR, LU, SZ, TI. **It**: Ven, TAA, Lomb, Piem. **Sl**: SlA.

***Rinodina polysporoides* Giralt & H. Mayrhofer**

L – Subs.: cor – Alt.: 1–3 – Note: a temperate species found on smooth bark of trunks and branches of deciduous, more rarely of evergreen broad-leaved trees, especially *Juglans* and *Fraxinus*, but also *Quercus*; certainly more widespread in the Alps, at relatively low altitudes. – **Au**: V, T, S, K, St, O. **Sw**: GL, GR, SZ, TI, UR, VS. **Fr**: Var. **It**: TAA, VA. **Sl**: SlA.

***Rinodina pyrina* (Ach.) Arnold**

Syn.: Berengeria exigua (Ach.) Trevis. var. maculiformis (Hepp) Trevis., *Lecanora pyrina* (Ach.) Röhl., *Lichen pyrinus* Ach., Rinodina exigua (Ach.) Gray var. maculiformis (Hepp) Bagl., Rinodina exigua (Ach.) Gray var. pyrina (Ach.) Th. Fr., *Rinodina maculiformis* (Hepp) Arnold

L – Subs.: cor, xyl – Alt.: 1–4 – Note: a temperate to southern boreal-montane, perhaps circumpolar early coloniser of the smooth bark of deciduous trees, often found on twigs and branches, with a broad ecological amplitude; widespread throughout the Alps. – **Au**: T, S, K, St, O, N, B. **Ge**: OB. **Sw**: GL, GR, LU, SG, TI, VS. **Fr**: AHP, HAl, AMa, Isè, Sav, HSav, Var, Vau. **It**: Frl, Ven, TAA, Lomb, Piem, VA, Lig. **Sl**: SlA.

***Rinodina rinodinoides* (Anzi) H. Mayrhofer & Scheid.**

Syn.: *Buellia rinodinoides* Anzi, *Lecidea rinodinoides* (Anzi) Stizenb., *Rinodina melanocarpa* Müll. Arg., *Rinodina serpentini* H. Mayrhofer & Poelt

L – Subs.: sil – Alt.: 3–5 – Note: an arctic-alpine species described from the Italian Alps and also known from the Balkan Peninsula and the Karakorum Mountains, found on usually south-exposed surfaces of very hard, base-rich siliceous rocks and serpentine; apparently rare in the Alps. – **Au**: S, K. **Sw**: VS. **Fr**: HSav. **It**: TAA, Lomb.

***Rinodina roboris* (Dufour *ex* Nyl.) Arnold**

Syn.: *Lecanora roboris* (Dufour *ex* Nyl.) Kremp., Lecanora sophodes (Ach.) Ach. var. roboris Dufour *ex* Nyl.

L – Subs.: cor – Alt.: 1–2 – Note: a mainly Atlantic species also known from Macaronesia, found on dry bark of ancient, more or less isolated trees, especially oaks, in relatively undisturbed, open, humid woodlands. – **Ge**: OB. **Sw**: GR, VS. **Fr**: Sav, HSav, Var. **It**: Lomb, Piem.

***Rinodina roscida* (Sommerf.) Arnold**

Syn.: Berengeria turfacea (Wahlenb.) Trevis. var. microcarpa (Hepp) Trevis., *Lecanora roscida* Sommerf., Rinodina turfacea (Wahlenb.) Körb. var. microcarpa (Hepp) Körb.

L – Subs.: deb, bry, ter-cal – Alt.: 4–6 – Note: an arctic-alpine, circumpolar species found on soil, bryophytes and plant debris over calcareous substrata in tundra-like habitats; widespread throughout the Alps. – **Au**: V, T, S, K, St, O, N. **Ge**: OB. **Sw**: BE, GR, LU, SZ, TI, UR, UW, VD, VS. **Fr**: AHP, HAl, Isè, Sav, HSav, Vau. **It**: Frl, Ven, TAA, Lomb, Piem, VA. **Sl**: SlA.

***Rinodina sheardii* Tønsberg**

L – Subs.: cor – Alt.: 3 – Note: a rare, sorediate, corticolous species with optimum in the montane belt. – **Au**: T, S. **Sw**: SZ. **Sl**: SlA.

***Rinodina sophodes* (Ach.) A. Massal.**

Syn.: *Berengeria sophodes* (Ach.) Trevis., *Dimelaena sophodes* (Ach.) Norman, *Gasparrinia sophodes* (Ach.) Tornab., *Lecanora sophodes* (Ach.) Ach., *Lichen sophodes* Ach., *Parmelia sophodes* (Ach.) Ach., Rinodina sophodes (Ach.) A. Massal. var. lusitanica H. Magn.

L – Subs.: cor, xyl – Alt.: 1–4 – Note: a widespread temperate early coloniser of smooth bark, most common on twigs and branches, with a wide ecological amplitude and a correspondingly wide altitudinal range; widespread throughout the Alps. – **Au**: V, T, S, K, St, O, N, B. **Ge**: OB, Schw. **Sw**: BE, GL, GR, SZ, TI, UR, VD, VS. **Fr**: AHP, HAl, AMa, Isè, Sav, HSav, Var, Vau. **It**: Frl, Ven, TAA, Lomb, Piem, VA, Lig. **Sl**: SlA, Tg.

***Rinodina straussii* J. Steiner**

L – Subs.: cal – Alt.: 4 – Note: a rare species growing under overhangs of calcareous rocks; widespread in Central Asia and also reported from a few sites in the Alps. – **Au**: K. **Sw**: BE.

***Rinodina subpariata* (Nyl.) Zahlbr.**

Syn.: *Lecanora subpariata* Nyl., *Rinodina degeliana* Coppins

L – Subs.: cor – Alt.: 3 – Note: an apparently rare, but also easily overlooked sorediate lichen with a holarctic distribution, growing on bark in rather shaded and humid situations, mostly in the deciduous forest belts, with a few records from the Eastern Alps (Austria, Italy). – **Au**: T. **It**: TAA.

***Rinodina teichophila* (Nyl.) Arnold**

Syn.: *Lecanora teichophila* Nyl., *Rinodina arenaria auct. non* (Hepp) Arnold, *Rinodina calcarea* (Hepp *ex* Arnold) Arnold var. obscurata Arnold, *Rinodina colletica* (Flörke) Arnold, Rinodina metabolica (Ach.) Anzi var. colletica (Flörke) Körb., *Rinodina suberumpens* (Nyl.) H. Olivier

L – Subs.: sil, int – Alt.: 1–3 – Note: a mainly mild-temperate silicicolous lichen, sometimes found in small urban settlements on walls (especially of calciferous sandstone, or of brick), and on roofing tiles; apparently more frequent in the Western and Southern Alps. – **Au**: K, St. **Sw**: BE, GR, TI. **Fr**: AHP, HAl, AMa, Isè, Var. **It**: Ven, TAA, Piem.

***Rinodina tephraspis* (Tuck.) Herre**

Syn.: *Lecanora badiella* Nyl., *Lecanora tephraspis* Tuck., Psora atrocinerea (Hook.) Hepp var. macrospora f. arenaria Hepp, *Rinodina arenaria* (Hepp) Arnold, *Rinodina badiella* (Nyl.) Th. Fr., *Rinodina caesiella* (Flörke *ex* Spreng.) Körb. var. glebulosa Arnold, *Rinodina glebulosa* (Arnold) Arnold, *Rinodina pannarioides* Körb. *ex* Stein

L – Subs.: sil – Alt.: 3 – Note: a rare species of siliceous rocks, in moist and often shaded situations such as near waterfalls, rapids, gorges and lakeshores, often associated with cyanobacteria (*Stigonema*). – **Au**: K. **It**: TAA.

***Rinodina terrestris* Tomin**

Syn.: *Rinodina mucronatula* H. Magn.

L – Subs.: xyl, ter, bry, deb, cal, int – Alt.: 2–5 – Note: on terricolous mosses and plant debris in open grasslands, over more or less calciferous or otherwise basic to neutral substrata, in sunny, dry, non-eutrophicated situations; perhaps more widespread in the Alps, but overlooked, or confused with other species. – **Au**: T. **Sw**: GR, VD, UR, VS. VS. **Fr**: AHP, HAl, Sav.

***Rinodina trachytica* (A. Massal.) Bagl. & Carestia**

Syn.: Lecanora confragosa (Ach.) Röhl. var. immersoareolata Harm., Mischoblastia lecanorina A. Massal. var. lavanea A. Massal., Mischoblastia lecanorina A. Massal. var. trachytica A. Massal., Rinodina confragosa (Ach.) Körb. var. immersoareolata (Harm.) Zahlbr., *Rinodina iberica* H. Mayrhofer, Rinodina lecanorina (A. Massal.) A. Massal. var. lavanea (A. Massal.) Bagl., *Rinodina subtrachytica* J. Steiner

L – Subs.: sil – Alt.: 1–3 – Note: a Mediterranean-Macaronesian to mild-temperate lichen found on base-rich, mostly volcanic rocks, and on serpentinite; most frequent in the Western and Southern Alps. – **Sw**: TI. **Fr**: AHP, HAl, AMa, Vau. **It**: Ven, TAA, Lomb, Piem, Lig.

***Rinodina trevisanii* (Hepp) Körb.**

Syn.: *Psora trevisanii* Hepp, *Rinodina convexula* H. Magn., *Rinodina lignaria* H. Magn., *Rinodina norrlandica* H. Magn.

L – Subs.: cor, xyl – Alt.: 3–4 – Note: a corticolous species rarely occurring on wood, often confused with *R. archaea*, but not very common. – **Au**: S, St, N. **Ge**: Schw. **Sw**: GR, VS. **It**: TAA. **Sl**: SlA.

***Rinodina tunicata* H. Mayrhofer & Poelt**

L – Subs.: cal – Alt.: 2 – Note: a Mediterranean to mild-temperate lichen found on compact, pure limestone or dolomite at relatively low elevations, also reported from the Western Alps (France). – **Fr**: AMa.

***Rinodina turfacea* (Wahlenb.) Körb.**

Syn.: *Berengeria turfacea* (Wahlenb.) Trevis., *Lichen turfaceus* Wahlenb., *Psora turfacea* (Wahlenb.) Hepp, *Rinodina orbata* (Ach.) Vain., Rinodina turfacea (Wahlenb.) Körb. var. orbata (Ach.) Jatta

L – Subs.: deb, ter-cal, bry – Alt.: 4–6 – Note: an arctic-alpine, circumpolar lichen found on soil rich in humus and plant remains in tundra-like habitats; widespread throughout the Alps. – **Au**: V, T, S, K, St, O, N. **Ge**: OB, Schw. **Sw**: BE, GR, LU, SG, SZ, TI, UR, UW, VD, VS. **Fr**: HAl, Sav, HSav. **It**: TAA, Lomb, Piem, VA. **Sl**: SlA.

***Rinodina venostana* Buschardt & H. Mayrhofer**

Syn.: Rinodina exigua (Ach.) Gray var. saxicola Anzi

L – Subs.: int – Alt.: 2–3 – Note: a species known from the southern part of Central Europe to the Mediterranean area, extending to Macaronesia, found on slightly calcareous schists, with a few records from the Western and the Southern Alps (France, Italy). – **Fr**: Sav. **It**: TAA, Lomb, Piem.

***Rinodina ventricosa* Hinteregger & Giralt**

L – Subs.: cor – Alt.: 4–5 – Note: on *Rhododendron ferrugineum* and rarely on *Rh. hirsutum*, with a rather scattered distribution in the Alps. – **Au**: V, T, S, K, St, O. **Sw**: GR. **It**: TAA.

***Rinodina zwackhiana* (Kremp.) Körb.**

Syn.: *Lecanora zwackhiana* Kremp., *Rinodina murorum* B. de Lesd., *Rinodina transsylvanica* (Nyl.) H. Olivier, *Rinodina violascens* H. Magn.

L – Subs.: cal – Alt.: 1–3 – Note: a mainly mild-temperate lichen found on steeply inclined to slightly underhanging surfaces of calcareous rocks, including walls, sometimes a juvenile parasite on other lichens; probably more widespread, but much overlooked in the Alps. – **Au**: S, K. **Ge**: OB. **Sw**: GR. **Fr**: AHP, HAl, Sav. **It**: Lig.

***Rinodinella controversa* (A. Massal.) H. Mayrhofer & Poelt**

Syn.: *Berengeria controversa* (A. Massal.) Trevis., *Berengeria fusca* (A. Massal.) Trevis., Buellia dubyana (Hepp) Rabenh. var. nigrescens Müll. Arg., *Catolechia fusca* A. Massal., *Lecanora budensis* Nyl., *Rinodina budensis* (Nyl.) Zahlbr., *Rinodina controversa* A. Massal., *Rinodina crustulata* (A. Massal.) Arnold, *Rinodina fusca* (A. Massal.) Bagl., *Rinodina sublobata* (Arnold) H. Olivier

L – Subs.: cal – Alt.: 1–3 – Note: a mainly southern species in Europe, found on the top of exposed calcareous boulders, with optimum below the montane belt; apparently most frequent in the Western and the Southern Alps (France, Italy). – **Au**: N. **Fr**: AHP, HAl, AMa, HSav, Var, Vau. **It**: Frl, Ven, TAA, Lomb, Piem, VA, Lig.

***Rinodinella dubyanoides* (Hepp) H. Mayrhofer & Poelt**

Syn.: *Buellia dubyanoides* (Hepp) Müll. Arg., Buellia dubyanoides (Hepp) Müll. Arg. var. evoluta Zahlbr., *Lecanora aequatula* Nyl., *Lecanora dubyanoides* (Hepp) Stizenb., *Lecidea dubyanoides* Hepp, *Rinodina aequatula* (Nyl.) B. de Lesd., *Rinodina dubyanoides* (Hepp) Arnold, Rinodina dubyanoides (Hepp) Arnold var. evoluta Zahlbr., *Rinodina minuta* B. de Lesd., *Rinodina subgranulata* Müll. Arg.

L – Subs.: cal – Alt.: 1–3 – Note: a mild-temperate to Mediterranean species found on hard, compact calcareous rocks, mostly on steeply inclined faces wetted by rain; apparently most frequent in the Western and the Southern Alps (France, Italy). – **Fr**: AHP, AMa, Drô, Sav, HSav, Var, Vau. **It**: TAA, Lomb.

***Roccella fuciformis* (L.) DC.**

Syn.: *Lichen fuciformis* L., *Roccella teneriffensis* Vain.

L – Subs.: cal – Alt.: 1 – Note: a supralittoral Mediterranean-Macaronesian species found on steeply inclined to underhanging surfaces of a wide variety of rocks (mainly calcareous) exposed to humid maritime winds, mostly in rather shaded situations; much rarer and less heliophytic than *R. phycopsis*, also reported from the base of the Western Alps (France). – **Fr**: Var.

***Roccella phycopsis* (Ach.) Ach.**

Syn.: *Lichen fucoides* Dicks. *nom.illeg.*, *Parmelia phycopsis* Ach., *Roccella fucoides* Vain., *Roccella pusilla* De Not., *Roccella pygmaea* Durieu & Mont.

L – Subs.: sax – Alt.: 1 – Note: on a very wide variety of rocks, incl. brick walls, sometimes on littoral shrubs, in rather sheltered situations, and in habitats subject to frequent, salt-laden maritime winds; in the study area so far known only from the base of the Western Alps. – **Fr**: AMa, Var. **It**: Lig.

***Romjularia lurida* (Ach.) Timdal**

Syn.: *Biatora lurida* (Ach.) Stenh., *Lecidea lurida* Ach., *Lecidea petri* (Tuck.) Zahlbr., *Mycobilimbia lurida* (Ach.) Hafellner & Türk, *Psora lurida* (Ach.) DC., *Psora petri* (Tuck.) Fink

L – Subs.: cal, ter-cal – Alt.: 1–5 – Note: a calcicolous, ecologically and altitudinally wide-ranging species, whose development often starts in fissures of the rock subject to temporary water seepage after rain, with a wide altitudinal range; widespread throughout the Alps. – **Au**: V, T, S, K, St, O, N. **Ge**: OB, Schw. **Sw**: BE, GR, LU, SZ, TI, UW, VD, VS. **Fr**: AHP, HAl, AMa, Drô, Isè, Sav, HSav, Var, Vau. **It**: Frl, Ven, TAA, Lomb, Piem, VA, Lig. **Sl**: SlA.

***Ropalospora lugubris* (Sommerf.) Poelt**

Syn.: *Bacidia lugubris* (Sommerf.) Zahlbr., *Bilimbia lugubris* (Sommerf.) Th. Fr., *Fuscidea lugubris* (Sommerf.) P. James & Purvis, *Lecidea caudata* Nyl., *Lecidea funera* Sommerf., *Lecidea lugubris* Sommerf., *Ropalospora cafra* A. Massal., *Ropalospora caudata* (Nyl.) A. Massal., *Schaereria lugubris* (Sommerf.) Körb., *Toninia caudata* (Nyl.) Arnold, *Toninia lugubris* (Sommerf.) Th. Fr.

L – Subs.: sil – Alt.: 3–5 – Note: an arctic-alpine to boreal-montane, perhaps circumpolar lichen found on steeply inclined surfaces of hard siliceous rocks in cold-humid upland areas. – **Sw**: BE, VS. **It**: Frl, TAA, Lomb, Piem.

***Ropalospora viridis* (Tønsberg) Tønsberg**

Syn.: *Fuscidea viridis* Tønsberg

L – Subs.: cor – Alt.: 2–4 – Note: on smooth bark of deciduous and coniferous trees in cold-humid, open woodlands; perhaps more widespread in the Alps, but certainly not very common. – **Au**: V, T, S, K, St, O. **Ge**: OB. **Sw**: BE, GL, GR, SZ, UW, VS. **It**: Frl. **Sl**: SlA.

***Rostania ceranisca* (Nyl.) Otálora, P.M. Jørg. & Wedin**

Syn.: *Collema arcticum* Lynge, *Collema ceraniscum* Nyl., *Collema subhumosum* Nyl., *Collema tetragonoides* Anzi, *Leptogium tetragonoides* (Anzi) Lettau

L – Subs.: ter-cal, ter-sil, deb – Alt.: 5 – Note: an arctic-alpine, perhaps circumpolar lichen found over frost-disturbed, weakly calcareous soil; to be looked for further in the Alps, where it is perhaps more widespread. – **Au**: T, S, St. **Sw**: GR, VS. **It**: Lomb.

***Rostania multipunctata* (Degel.) Otálora, P.M. Jørg. & Wedin**

Syn.: *Collema multipunctatum* Degel.

L – Subs.: cor – Alt.: 1–2 – Note: a mild-temperate, Mediterranean-Atlantic species found on more or less isolated trees in warm-humid areas, especially on *Olea*, with a few records from the base of the Western Alps (France, Italy). – **Fr**: AMa, Var. **It**: Lig.

***Rostania occultata* (Bagl.) Otálora, P.M. Jørg. & Wedin**

Syn.: *Collema coccophylloides* Hepp *ex* Müll. Arg., *Collema occultatum* Bagl., *Collema quadratum* Zwackh, *Leptogium occultatum* (Bagl.) Zahlbr., *Leptogium quadratum* (Zwackh) Nyl., *Rostania quadrata* (Zwackh) Trevis.

L – Subs.: cor – Alt.: 2–3 – Note: a temperate lichen found on smooth, base-rich, but not very eutrophicated bark of more less isolated broad-leaved trees (*Acer*, *Fraxinus*, *Juglans*, *Populus*) in rather humid sites, especially on basal parts of old trunks; easy to overlook and widespread, but certainly not common in the Alps. – **Au**: V, S, K, St, O, N. **Ge**: OB, Schw. **Fr**: AHP, AMa, Var, Vau. **It**: TAA, Piem.

***Sagiolechia protuberans* (Ach.) A. Massal.**

Syn.: *Bilimbia protuberans* (Ach.) A. Massal., *Gyalecta cimbrica* (A. Massal.) Jatta, *Gyalecta protuberans* (Ach.) Anzi, Gyalecta protuberans (Ach.) Anzi var. mammillata (Hepp) Anzi, *Lecidea protuberans* (Ach.) Schaer., *Sagedia protuberans* Ach., *Sagiolechia cimbrica* A. Massal., *Sagiolechia leioplacoides* (Vain.) Vain., *Verrucaria leioplacoides* Vain.

L – Subs.: cal – Alt.: 2–5 – Note: on dolomite and hard calciferous rocks in rather humid situations; widespread throughout the Alps. – **Au**: V, T, S, K, St, O, N. **Ge**: OB. **Sw**: BE, GL, GR, LU, SG, SZ, UW, VD, VS. **Fr**: AHP, HAl, AMa, Drô, Isè, Sav, HSav, Var, Vau. **It**: Frl, Ven, TAA, Lomb, VA. **Sl**: SlA, Tg.

***Santessoniella arctophila* (Th. Fr.) Henssen**

Syn.: *Pannaria arctophila* Th. Fr., *Parmeliella arctophila* (Th. Fr.) Malme

L – Subs.: ter, bry – Alt.: 4 – Note: a circum-arctic-alpine species with a brownish granular thallus becoming gelatinous when wet, brown biatorine apothecia, asci with an amyloid ring structure, and hyaline, ellipsoid ascospores; the generic placement is still unsettled; on slightly calcareous soil and plant debris in snow-bed communities; more common in Northern Europe and with a few records from the Swiss Alps, but perhaps overlooked elsewhere. – **Sw**: GR, VS.

**Sarcogyne algoviae H. Magn. var. algoviae**

Syn.: *Biatorella algoviae* (H. Magn.) Zahlbr.

L – Subs.: cal – Alt.: 4–6 – Note: on non – or weakly calciferous, sometimes dolomitic rocks in sunny situations, with optimum above treeline. – **Au**: ?V, ?T, S, O. **Ge**: Schw. **Fr**: AHP, HAl, AMa. **It**: Piem.

**Sarcogyne algoviae H. Magn. var. euthallina Asta & Cl. Roux**

L # – Subs.: cal – Alt.: 4 – Note: on rocks with a low content in calcium, in communities with *Acarospora badiofusca*; only known from high elevations in the Western Alps (France). This taxon does not belong to *S. algoviae*, and is probably a *Polysporina* of the *simplex*-complex, perhaps growing parasitically on a crustose lichen with a white thallus (Roux et al. 2014). – **Fr**: HAl.

***Sarcogyne clavus* (DC.) Kremp.**

Syn.: *Biatorella clavus* (DC.) Th. Fr., *Lecanora eucarpa* (Nyl.) Nyl., *Lecidea eucarpa* Nyl., *Lichen clavus* (DC.) Ramond, *Patellaria clavus* DC., *Sarcogyne eucarpa* (Nyl.) Hellb., *Stereopeltis carestiae* De Not., *Stereopeltis macrocarpa* Franzoni & De Not.

L – Subs.: sil – Alt.: 2–4 – Note: on steeply inclined to underhanging surfaces of hard, mineral-rich siliceous rocks, especially granite, mostly in fissures of the rock. – **Au**: V, T, S, K, St. **Sw**: BE, TI, UR, VD, VS. **Fr**: AHP, HAl, AMa, Isè, HSav, Var, Vau. **It**: TAA, Lomb, Piem. **Sl**: SlA.

***Sarcogyne coronata* Jatta**

Syn.: *Biatorella coronata* (Jatta) Zahlbr.

L # – Subs.: cal – Alt.: 4–5 – Note: a calcicolous species with an effuse, epilithic, white to yellowish, continuous to somewhat rimulose thallus, initially urceolate, finally sessile apothecia with black discs covered by a bluish-grey pruina, surrounded by a darker, hardly prominent proper margin and a persistent white thalline rim, in section with a black-brown hypothecium, polyspored asci, and oblong to ellipsoid, hyaline ascospores (3–6 × 1.5–2 μm); only known from the type locality in the Eastern Alps (Italy). – **It**: TAA.

***Sarcogyne cretacea* Poelt**

L – Subs.: cal, int – Alt.: 5–6 – Note: a species forming small chalky-white thalli in which the urceolate apothecia are completely immersed; on exposed cliffs of marly limestone and calcareous schists; so far only known from some scattered localities in the Alps. – **Au**: T. **Ge**: Schw. **Sw**: SZ.

***Sarcogyne distinguenda* Th. Fr.**

L – Subs.: cal, int – Alt.: 3–4 – Note: a species with a thin to rimose, greyish-white thallus, and sessile, black apothecia covered by a bluish-white pruina, with a relatively high hymenium and broadly ellipsoid to subspherical ascospores; on outcrops of limestone and calcareous sandstone, widespread in Europe but relatively rare, also in the Alps. – **Au**: T, O. **Ge**: OB, Schw. **Sw**: BE, SZ.

***Sarcogyne fallax* H. Magn.**

L – Subs.: cal, int – Alt.: 2–5 – Note: a mainly mild-temperate lichen found on steeply inclined to underhanging surfaces of base-rich siliceous rocks, more rarely on calcareous rocks, which is worthy of further study. – **Au**: ?V, S, K, St, N. **Ge**: OB. **Sw**: BE, SZ. **Fr**: AHP, AMa, HSav. **It**: Lig. **Sl**: SlA.

***Sarcogyne hypophaea* (Nyl.) Arnold**

Syn.: *Biatorella hypophaea* (Nyl.) Blomb. & Forssell, *Biatorella privigna auct. non* (Ach.) Sandst., *Lecanora hypophaea* Nyl., *Sarcogyne privigna auct. non* (Ach.) A. Massal.

L – Subs.: sil, cal, int – Alt.: 1–6 – Note: a cool-temperate to arctic-alpine, circumpolar species with optimum on steeply inclined faces and in fissures of the rock, mainly on base-rich siliceous substrata, but frequent also on limestone. – **Au**: V, T, S, K, St, O, N. **Ge**: OB, Schw. **Sw**: GR, LU, SZ, TI, VS. **Fr**: AHP, HAl, AMa, Isè, HSav, Var. **It**: Ven, TAA, Lomb, Piem, VA, Lig.

***Sarcogyne hypophaeoides* Vain. *ex* H. Magn.**

L – Subs.: sil – Alt.: 3 – Note: this species appears to be fairly common in humid habitats of the boreal region of Fennoscandia, but its distribution is incompletely known; also reported from the Eastern Alps (Austria). – **Au**: St.

***Sarcogyne lapponica* (Ach. *ex* Schaer.) K. Knudsen & Kocourk.**

Syn.: *Acarospora lapponica* (Ach. *ex* Schaer.) Th. Fr., *Biatorella lapponica* (Ach. *ex* Schaer.) Almq., *Lecidea lapponica* Ach. *ex* Schaer., *Myriospora lapponica* (Ach. *ex* Schaer.) Hue, *Polysporina lapponica* (Ach. *ex* Schaer.) Degel.

L – Subs.: sil-par, met-par – Alt.: 1–5 – Note: this species, whose type material is lignicolous, has been for a long time confused with the lichenicolous *Polysporina subfuscescens*. The records from the Alps need confirmation. – **Sw**: BE. **It**: ?TAA.

***Sarcogyne latericola* (J. Steiner) J. Steiner**

Syn.: *Biatorella latericola* J. Steiner

L # – Subs.: sil – Alt.: 3 – Note: a species resembling *S. regularis*, but with a grey to brownish-grey thallus and prominent to sessile apothecia with black-brown discs and narrow-ellipsoid ascospores; its taxonomic value is in need of re-evaluation; on old roof tiles, so far only known from the Eastern Alps (Austria). – **Au**: K.

***Sarcogyne nivea* Kremp.**

L – Subs.: cal – Alt.: 2–3 – Note: a species forming small, chalky-white, rimose thalli and black, sessile apothecia with relatively few subspherical ascospores per ascus; on limestone at low elevations; rare in Europe, but perhaps not recognised or overlooked, with a few records from the Western Alps only (France). – **Fr**: AHP.

**Sarcogyne regularis Körb. var. regularis**

Syn.: *Biatorella regularis* (Körb.) Lettau, *Sarcogyne pruinosa auct. non* (Ach.) Mudd

L – Subs.: cal, int – Alt.: 1–6 – Note: a very variable, perhaps heterogeneous, holarctic-subcosmopolitan calcicolous species which needs a revision based on molecular data. It is common both in urban areas (*e.g.* on mortar walls) and in natural situations, mostly in lichen-poor stands, with a very wide altitudinal range; widespread throughout the Alps. – **Au**: V, T, S, K, St, O, N, B. **Ge**: OB, Schw. **Sw**: BE, GR, LU, SZ, TI, VD, VS. **Fr**: AHP, AMa, Drô, Isè, Sav, HSav, Var, Vau. **It**: Frl, Ven, TAA, Lomb, Piem, VA, Lig. **Sl**: SlA, Tg.

**Sarcogyne regularis Körb. var. decipiens (A. Massal.) N.S. Golubk.**

Syn.: Sarcogyne privigna (Ach.) A.Massal. var. decipiens A. Massal., Sarcogyne simplex (Taylor) Nyl. var. decipiens (A. Massal.) Jatta

L # – Subs.: cal – Alt.: 1–5 – Note: a taxon with an endolithic thallus and immersed, black, epruinose apothecia, described from M. Baldo in Italy; on calcareous rocks, with optimum in the montane belt; often not distinguished and therefore distributional data hard to interpret. – **Au**: ?V, ?T, K, St, O. **Fr**: AHP, AMa, Drô, Isè, Sav, HSav, Var, Vau. **It**: Ven.

**Sarcogyne regularis Körb. var. intermedia (Körb.) N.S. Golubk.**

Syn.: *Sarcogyne pruinosa auct.* f. intermedia Körb., *Sarcogyne pruinosa auct.* var. intermedia (Körb.) H. Magn.

L # – Subs.: cal – Alt.: 1–5 – Note: a calcicolous variety with pruinose apothecial discs and non-pruinose, black margins; often not distinguished, and therefore distributional data hard to interpret. – **Au**: K, St. **Fr**: AHP, HAl, AMa, Drô, Isè, Sav, HSav, Var, Vau.

**Sarcogyne regularis Körb. var. macroloma (Flörke *ex* Körb.) N.S. Golubk.**

Syn.: *Sarcogyne pruinosa auct.* f. macroloma Flörke *ex* Körb., *Sarcogyne pruinosa auct.* var. macroloma (Flörke *ex* Körb.) H. Magn.

L # – Subs.: cal – Alt.: 2–5 – Note: a calcicolous variety with pruinose apothecial discs and pruinose, thick margins; often not distinguished, and therefore distributional data hard to interpret. – **Au**: ?V, T, St. **Fr**: HAl, AMa, Drô, Isè, Sav, Vau.

**Sarcogyne regularis Körb. var. minuta (A. Massal.) N.S. Golubk.**

Syn.: *Sarcogyne pruinosa auct.* var. minuta A. Massal.

L # – Subs.: cal – Alt.: 5 – Note: a calcicolous variety with a white leprose thallus in which the gyalectoid apothecia (pruinose or not) are immersed; often not distinguished, and therefore distributional data therefore incomplete and hard to interpret. – **Au**: ?V, ?T, St.

**Sarcogyne regularis Körb. var. platycarpoides (Anzi) N.S. Golubk.**

Syn.: *Biatorella platycarpoides* (Anzi) Th. Fr., *Sarcogyne platycarpoides* Anzi, *Sarcogyne pruinosa auct.* var. platycarpoides (Anzi) H. Magn.

L # – Subs.: int, cal – Alt.: 2–5 – Note: this is one of the several morphs of *S. regularis* *s.lat.* which are worthy of a molecular study. It is an interesting taxon, most common on dolomitic pebbles above treeline, and it might be more widespread in the Alps. – **Au**: T, S, K, St. **Ge**: OB. **Fr**: AHP, HAl, Isè. **It**: Ven, Lomb, Piem.

**Sarcogyne regularis Körb. var. psimmythina (Nyl.) N.S. Golubk.**

Syn.: *Lecanora psimmythina* Nyl.

L # – Subs.: int – Alt.: 4–5 – Note: a variety with a thin white thallus and black, marginate apothecia, based on a type from Finland; on micaschists; not consistently distinguished, and distributional data therefore incomplete and hard to interpret. – **Au**: V, T.

***Sarcosagium campestre* (Fr.) Poetsch & Schied.**

Syn.: *Biatora campestris* Fr., *Biatorella campestris* (Fr.) Th. Fr., *Biatorella sarcosagium* Anzi, *Sarcosagium biatorellum* A. Massal.

L – Subs.: ter-cal, bry, xyl, deb, bry-cal – Alt.: 2–5 – Note: an early coloniser of more or less calciferous soil, moribund bryophytes and plant debris, sometimes also growing on decaying wood, mostly in disturbed habitats, *e.g.* at burned sites, in upland areas. – **Au**: T, S, K, St, O, N. **Ge**: OB. **Sw**: GR. **Fr**: Sav, HSav. **It**: Frl, Ven, Lomb.

***Schadonia alpina* Körb.**

Syn.: *Bombyliospora gemella* Anzi, *Lopadium gemellum* (Anzi) Jatta

L – Subs.: deb, ter-sil – Alt.: 4–6 – Note: a mainly arctic-alpine species found on soil and moribund bryophytes over siliceous substrata, with a few scattered records from the Alps. – **Au**: T, S. **It**: Lomb.

***Schadonia fecunda* (Th. Fr.) Vězda & Poelt**

Syn.: *Biatora socialis* Hepp *ex* Körb. *nom. inval.*, *Diplotomma sociale* (Körb.) Jatta, *Lopadium fecundum* Th. Fr., *Lopadium sociale* Körb.

L – Subs.: deb, bry, ter – Alt.: 4–5 – Note: a mainly arctic-alpine species found on mosses and plant remains over acid siliceous substrata; apparently rather rare in the Alps. – **Au**: T, S, N. **Ge**: Ge. **Sw**: GR, LU, UR. **It**: TAA, Lomb, Piem.

***Schaereria cinereorufa* (Schaer.) Th. Fr.**

Syn.: *Lecidea cinereorufa* Schaer., *Lecidea subfurva* Nyl., *Psora cinereorufa* (Schaer.) Hellb., *Schaereria lugubris* (Fr.) Körb.

L – Subs.: sil – Alt.: 3–6 – Note: an arctic-alpine to boreal-montane, circumpolar species found on inclined to vertical faces of mineral-rich rocks wetted by rain, often near the ground, usually associated with species of *Pertusaria*. – **Au**: V, T, S, K, St, N. **Sw**: BE, VS. **It**: Ven, TAA, Piem. **Sl**: SlA.

***Schaereria corticola* Muhr & Tønsberg**

L – Subs.: cor – Alt.: 3 – Note: a species with a greyish to greyish-brown, indistinctly areolate thallus forming small patches among other lichens, and soralia with external soredia becoming brown, containing gyrophoric acid (thallus resembling that of *Rimularia fuscosora*, which however contains norstictic acid), apothecia fairly common, black with concolorous margins and with a green, partly violet epihymenium, the cylindrical asci containing broadly ellipsoid to subglobose, halonate ascospores; on bark of deciduous trees in humid lowland sites; widespread in the Holarctic region including Macaronesia, in Europe most common in the Northwest; reported from the Eastern Alps only (Austria), but perhaps still overlooked elsewhere. – **Au**: T.

**Schaereria fuscocinerea (Nyl.) Clauzade & Cl. Roux var. fuscocinerea**

Syn.: *Aspicilia cambusiana* Walt. Watson, *Aspicilia complanatoides* (A.L. Sm.) Walt. Watson, *Aspicilia tenebrosa* (Flot.) Körb., *Lecanora cambusiana* (Walt. Watson) Cretz., *Lecanora complanatoides* A.L. Sm., *Lecidea atrocinerea* (Schaer.) Vain., *Lecidea endocyanea* Stirt., *Lecidea epiiodiza* Nyl., *Lecidea fuscocinerea* Nyl., *Lecidea griseoatra auct. non* (Flot.) Schaer., *Lecidea tenebrosa* Flot., *Lecidella tenebrosa* (Flot.) Stein, *Schaereria endocyanea* (Stirt.) Hertel & Gotth. Schneid., *Schaereria tenebrosa* (Flot.) Hertel & Poelt

L – Subs.: sil – Alt.: 3–6 – Note: an arctic-alpine to boreal-montane, circumpolar species found on hard siliceous rocks in exposed situations, with optimum above treeline; widespread throughout the Alps. – **Au**: V, T, S, K, St, N. **Ge**: Schw. **Sw**: BE, GR, TI, UR, VD, VS. **Fr**: AHP, HAl, AMa, Isè, Sav, HSav. **It**: Frl, Ven, TAA, Lomb, Piem, VA, Lig.

**Schaereria fuscocinerea (Nyl.) Clauzade & Cl. Roux var. sorediata (Houmeau & Cl. Roux) Coppins**

Syn.: Schaereria tenebrosa (Flot.) Hertel & Poelt var. sorediata Houmeau & Cl. Roux

L – Subs.: sil – Alt.: 4–5 – Note: a rare sorediate morph, perhaps overlooked being mostly sterile; some of the few records from the Alps need confirmation. – **Au**: ?V, ?T, St.

***Schaereria parasemella* (Nyl.) Lumbsch**

Syn.: *Hafellnera parasemella* (Nyl.) Houmeau & Cl. Roux, *Lecidea parasemella* Nyl.

L – Subs.: deb, bry – Alt.: 4–5 – Note: an arctic to nemoral-alpine species with a thin greyish thallus, blackish-brown, sessile apothecia with a thalline margin and a a cupulate proper exciple, a green epihymenium, cylindrical asci ,and broadly ellipsoid to subglobose, halonate ascospores; on plant debris overgrown by crustose lichens (mainly *Biatora vernalis*) over acid substrata; rare, with very few scattered records from the Alps. – **Au**: T. **Fr**: HAl.

***Schismatomma niveum* D. Hawksw. & P. James**

L – Subs.: cor – Alt.: 2 – Note: a western species, never found fertile, growing on the dry sides of trunks of old deciduous oaks in sheltered situations, which most probably does not belong to *Schismatomma* *s.str.*; in the study area so far known only from the base of the Western Alps (France). – **Fr**: AMa.

***Schismatomma pericleum* (Ach.) Branth & Rostr.**

Syn.: *Lecanactis periclea* (Ach.) M. Choisy, *Lecanora periclea* (Ach.) Ach., *Lichen pericleus* Ach., *Platygrapha dolosa* (Ach.) Anzi, *Platygrapha periclea* (Ach.) Nyl., *Schismatomma abietinum* (Humb.) Almq. *non* (Ach.) A. Massal., *Schismatomma dolosum* (Ach.) Flot. & Körb. *ex* A. Massal., *Schismatomma farinosum* (Stenh.) Almq., Schismatomma pericleum (Ach.) Branth & Rostr. var. farinosum (Stenh.) Lettau, *Verrucaria abietina* Humb.

L – Subs.: cor – Alt.: 2–4 – Note: a temperate, mainly western species with optimum in humid beech forests, mostly on conifers (*Abies*, *Picea*), much more rarely on oaks; widespread throughout the Alps, but generally not common. – **Au**: V, T, S, K, St, O, N. **Ge**: OB. **Sw**: BE, GL, GR, LU, SZ, TI, UR, UW, VD, VS. **Fr**: AMa, Isè, Sav, Var, Vau. **It**: Ven, TAA, Lomb, Piem. **Sl**: SlA.

***Schismatomma ricasolii* (A. Massal.) Egea & Torrente**

Syn.: *Chiodecton graphidioides* Leight., *Chiodecton italicum* (B. de Lesd.) Zahlbr., *Enterographa graphidioides* (Leight.) Almb., *Enterographa italica* B. de Lesd., *Enterographa pseudorufescens* (B. de Lesd.) Redinger, *Enterographa rimata* (Flot *ex* Nyl.) Zwackh, *Lecanactis ricasolii* A. Massal., *Opegrapha pseudorufescens* B. de Lesd., *Opegrapha ricasolii* (A. Massal.) Jatta, *Platygrapha rimata* Flot. *ex* Nyl., *Schismatomma graphidioides* (Leight.) Zahlbr., *Schismatomma rimatum* (Flot *ex* Nyl.) Almq.

L – Subs.: cor – Alt.: 1–3 – Note: a cool-temperate, mainly western species with optimum in humid *Abies*-*Fagus* forests of the Mediterranean mountains; rare in the Alps. – **Sw**: GR. **Fr**: AHP, AMa, Var, Vau. **It**: Frl, Lomb.

***Schismatomma umbrinum* (Coppins & P. James) P.M. Jørg. & Tønsberg**

Syn.: *Lecanactis umbrina* Coppins & P. James

L – Subs.: sil – Alt.: 2–4 – Note: a mostly sterile species with a brownish, smooth to tuberculate thallus delimited by a distinct prothalline margin, the tubercles often with apical, concolorous soralia which later coalesce hiding most of the thalline surface, a trentepohlioid photobiont, and containing schizopeltic acid; usually on acidic rocks under overhangs, often with *Enterographa zonata*; most of the records are from the Eastern Alps, but the species might have been overlooked elsewhere. – **Au**: ?V, T, S, K, St. **Sw**: SZ. **Sl**: SlA.

***Sclerophora pallida* (Pers.) Y.J. Yao & Spooner**

Syn.: *Calicium pallidum* Pers., *Coniocybe curta* H. Magn., *Coniocybe nivea* (Hoffm.) Arnold *non* Tuck. & Mont., Coniocybe nivea (Hoffm.) Arnold var. pallida (Pers.) Arnold, *Coniocybe pallida* (Pers.) Fr., Coniocybe pallida (Pers.) Fr. var. nivea (Hoffm.) Arnold, *Coniocybe subpallida* Nyl., *Sclerophora nivea* (Hoffm.) Tibell, *Trichia nivea* Hoffm.

L – Subs.: cor – Alt.: 2–3 – Note: a temperate species found on old trees, such as *Acer*, *Ulmus* and *Fraxinus* in dry crevices of the bark; widespread throughout the Alps, but certainly declining. – **Au**: T, S, K, St, O, N. **Ge**: OB. **Fr**: AMa, Vau. **It**: Ven, TAA, Lomb.

***Sclerophora peronella* (Ach.) Tibell**

Syn.: *Coniocybe hyalinella* Nyl., *Coniocybe peronella* (Ach.) Tibell, *Lichen peronellus* Ach., *Roesleria hyalinella* (Nyl.) Sacc.; incl. *Roesleria norrlinii* Vain., Sclerophora peronella (Ach.) Tibell var. norrlinii (Vain.) Lettau

L – Subs.: cor, xyl – Alt.: 2–4 – Note: on bark and lignum of mature broad-leaved trees, often forming monospecific stands; in the Alps it is rare and probably declining. – **Au**: B. **Sw**: GR, VD. **Sl**: SlA.

***Scoliciosporum chlorococcum* (Graewe *ex* Stenh.) Vězda**

Syn.: *Bacidia chlorococca* (Graewe *ex* Stenh.) Lettau, *Bacidia interspersula* (Nyl.) Zahlbr., *Biatora hypnophila* (Turner *ex* Ach.) Zahlbr. var. chlorococca Graewe *ex* Stenh., *Bilimbia chlorococca* (Graewe *ex* Stenh.) Th. Fr.

L – Subs.: cor, xyl, sil – Alt.: 1–4 – Note: a widespread holarctic, ecologically wide-ranging species found on bark (especially of *Fagus*), lignum, and more rarely siliceous rocks, tolerant to air pollution; widespread throughout the Alps. – **Au**: V, T, S, K, St, O, N, B. **Ge**: OB. **Sw**: BE, GR, LU, TI, UW, VS. **Fr**: AMa. **It**: Frl, Ven, TAA, Lomb, Piem, VA, Lig. **Sl**: SlA, Tg.

***Scoliciosporum curvatum* Sérus.**

L – Subs.: fol – Alt.: 2–3 – Note: a species with a thin greenish thallus composed of small granules, extremely minute, hemispherical apothecia, 8–16-spored asci, and 1-septate ascospores with acute ends or lunulate; foliicolous in the understory of humid forests; in Central Europe mostly on needles of *Abies* and *Picea*, otherwise on leaves and green twigs of *Buxus* in very humid situations; widespread in Europe, but most common in its western parts. – **Au**: K, St. **Sw**: LU, VS. **Fr**: AMa, Drô, Vau.

***Scoliciosporum gallurae* Vězda & Poelt**

L – Subs.: cor – Alt.: 2–3 – Note: on twigs and branches, more rarely on trunks of coniferous and broad-leaved trees at relatively low elevations; quite common in Southern Europe, being often sterile it has been frequently overlooked, and is probably more widespread in the Alps. – **Sw**: LU, UW. **Fr**: AHP, AMa, Var, Vau.

***Scoliciosporum intrusum* (Th. Fr.) Hafellner**

Syn.: *Carbonea intrusa* (Th. Fr.) Rambold & Triebel, *Catillaria intrusa* (Th. Fr.) Th. Fr., *Conida intrusa* (Th. Fr.) Sacc. & D. Sacc., *Lecidea aphanoides* Nyl., *Lecidea contrusa* Vain. *nom.illeg.*, *Lecidea intrusa* Th. Fr., *Lecidea melaphana* Nyl., *Lecideopsis intrusa* (Th. Fr.) Zopf, *Micarea intrusa* (Th. Fr.) Coppins & H. Kilias

L – Subs.: sil-par – Alt.: 3–5 – Note: a species forming insular, small, granulose thalli on and inbetween other crustose lichens, with black, glossy apothecia, early with convex discs and excluded margins, and only slightly curved, unusually mostly simple or 1-septate ascospores; on siliceous rocks in *Umbilicaria cylindrica*-communities; widespread in Europe from the Arctic to the boreal-montane zone, in the Alps mostly from the treeline ecotone to the lower alpine belt. The species does not belong to *Scoliciosporum* *s.str.* – **Au**: V, T, S, K, St. **Sw**: TI. **Fr**: AHP. **It**: Frl, TAA.

***Scoliciosporum perpusillum* J. Lahm *ex* Körb.**

Syn.: *Bacidia perpusilla* (J. Lahm *ex* Körb.) Th. Fr.

L – Subs.: cor, sil – Alt.: 2–3 – Note: a mild-temperate, probably western species, generally found on acid bark, especially of conifers, but also on needles of *Abies* in damp montane forests; from the Alps there are so far a few scattered records only. – **Au**: V, T, St, N. **Sw**: UR. **It**: Piem.

***Scoliciosporum sarothamni* (Vain.) Vězda**

Syn.: *Bacidia sarothamni* Vain.

L – Subs.: cor, fol, sil – Alt.: 2–3 – Note: a mainly mild-temperate, mostly sterile early coloniser of smooth bark, rarely occurring also on siliceous rocks and on leaves of *Buxus* and *Abies*. – **Au**: K, St, N. **Sw**: BE, GL, GR, LU, TI, UW, VS. **Fr**: AHP, HAl, AMa, Drô, Isè, HSav, Var, Vau. **It**: Frl, Ven. **Sl**: SlA.

***Scoliciosporum schadeanum* (Erichsen) Vězda**

Syn.: *Bacidia schadeana* Erichsen

L – Subs.: cor – Alt.: 2–3 – Note: a species resembling *S. pruinosum* in the whitish, minute apothecia, but epihymenium lacking crystals and ascospores somewhat thicker; on bark of deciduous trees in humid forests along creeks; widespread in Europe but rarely collected, with a few records from the Eastern Alps only (Austria), but easy to overlook and perhaps more widespread in the Alps. – **Au**: K, St.

**Scoliciosporum umbrinum (Ach.) Arnold var. umbrinum**

Syn.: *Bacidia compacta* (Körb.) Jatta, *Bacidia holomelaena* (Flörke) Anzi, *Bacidia turgida* (Körb.) Hellb., *Bacidia umbrina* (Ach.) Bausch, Bacidia umbrina (Ach.) Bausch var. compacta (Körb.) Th. Fr., Bacidia umbrina (Ach.) Bausch var. turgida (Körb.) Th. Fr., *Lecidea holomelaena* Flörke, *Lecidea umbrina* Ach., *Scoliciosporum compactum* Körb., *Scoliciosporum holomelaenum* (Flörke) A. Massal., *Scoliciosporum turgidum* Körb., Scoliciosporum umbrinum (Ach.) Arnold var. compactum (Körb.) Clauzade & Cl. Roux

L – Subs.: sil, int, xyl, cor – Alt.: 1–5 – Note: an ecologically wide-ranging, probably holarctic species, also present in urban environments; sometimes parasitic on other lichens (especially when on siliceous rocks); widespread throughout the Alps. – **Au**: V, T, S, K, St, O, N, B. **Ge**: OB. **Sw**: BE, TI, UR, VS. **Fr**: AHP, AMa, Sav, HSav, Var, Vau. **It**: Frl, Ven, TAA, Lomb, Piem, VA, Lig. **Sl**: SlA. **Li**.

**Scoliciosporum umbrinum (Ach.) Arnold var. corticicolum (Anzi) Bagl. & Carestia (“corticolum” *ex* errore)**

Syn.: *Bacidia corticicola* (Anzi) Dalla Torre & Sarnth., *Bacidia holomelaena* (Flörke) Anzi var. [“c”] *corticicola* Anzi, Bacidia umbrina (Ach.) Bausch var. corticicola (Anzi) Bausch (“*corticola” ex errore*), *Scoliciosporum corticicola* (Anzi) Arnold (“*corticolum*”)

L # – Subs.: cor – Alt.: 2–4 – Note: a name applied to corticolous populations of a *Scoliciosporum* with the hymenial characters of *S. umbrinum*, namely the fasciculate, twisted ascospores; the taxonomic value is uncertain; mostly on branches of various trees including conifers (*Larix*), in the Alps from the lowlands to the subalpine belt. – **Au**: V, T, S, K, St, O. **Ge**: OB. **Fr**: AHP, AMa, Sav, Var. **It**: Lomb.

***Scytinium aquale* (Arnold) Otálora, P.M. Jørg. & Wedin**

Syn.: *Leptogium aquale* (Arnold) P.M. Jørg., Leptogium pusillum Nyl. var. aquale Arnold

L # – Subs.: cal-aqu – Alt.: 4 – Note: a minute species, somewhat resembling *S. biatorinum*, with a blackish-brown, mainly crustose-granular thallus, the granules paraplectenchymatous throughout, apothecia frequent (to 0.5 mm in diam.), sessile, with concave to flat, pale brown discs, and occasionally with a crenulate thalline collar, in section with a proper exciple, with submuriform to muriform, relatively large ascospores (30–45 × 10–14 μm); on calcareous pebbles in a stream, so far only known from the Eastern Alps (Austria). – **Au**: T.

***Scytinium aragonii* (Otálora) Otálora, P.M. Jørg. & Wedin**

Syn.: *Leptogium aragonii* Otálora

L – Subs.: cor-bry – Alt.: 2–4 – Note: a recently-described species, widespread throughout Europe in old forests, from 200 m in northern regions to 1,800 m in the South, on pleurocarpous mosses close to the base of trunks, over mossy walls or calcareous rocks within forests, or on mosses in rock fissures within dry subalpine grasslands. – **Ge**: OB, Schw. **Sw**: SZ. **Fr**: AHP, AMa. **It**: Piem.

***Scytinium biatorinum* (Nyl.) Otálora, P.M. Jørg. & Wedin**

Syn.: *Collema biatorinum* Nyl., *Leptogium biatorinum* (Nyl.) Leight., *Leptogium pusillum* Nyl.

L – Subs.: cal, ter-cal – Alt.: 2 – Note: a temperate ephemeral lichen of disturbed habitats, most frequent on concrete walls, but also found on calciferous soil; perhaps more widespread in the Alps but overlooked, or confused with other species. – **Ge**: OB. **Sw**: SZ. **Fr**: HAl, Sav. **It**: Lomb.

***Scytinium callopismum* (A. Massal.) Otálora, P.M. Jørg. & Wedin**

Syn.: *Collema callopismum* A. Massal., *Leptogium callopismum* (A. Massal.) Harm.

L – Subs.: cal – Alt.: 2–5 – Note: a mainly western European, temperate lichen of more or less calciferous rocks, often on seepage tracks. – **Au**: V, ?T, S, K, St, O. **Ge**: OB, Schw. **Sw**: VS. **Fr**: HSav, Var. **It**: Frl, Ven, TAA, Lomb, Piem, VA. **Sl**: Tg.

***Scytinium cretaceum* (Sm.) ined. comb. ad int.**

Syn.: *Leptogium cretaceum* (Sm.) Nyl., *Lichen cretaceus* Sm., *Polychidium cretaceum* (Sm.) Trevis.

L # – Subs.: cal – Alt.: 2–4 – Note: closely related to *S. biatorinum*, but thallus more granular; the status of this taxon is still poorly understood; over calcareous rocks in rather dry situations; from the Alps there are a few records only, all in need of critical evaluation. – **Au**: N. **Fr**: AMa, Sav, Var, Vau. **It**: Lomb.

***Scytinium fragile* (Taylor) Otálora, P.M. Jørg. & Wedin**

Syn.: *Collema fragile* Taylor, *Leptogium fragile* (Taylor) Nyl.

L – Subs.: cal – Alt.: 1–4 – Note: a southwestern species in Europe, found on steeply inclined seepage tracks of calcareous rocks; much overlooked and perhaps more widespread, but certainly not common in the Alps. – **Au**: ?V, O. **It**: Piem, Lig.

***Scytinium fragrans* (Sm.) Otálora, P.M. Jørg. & Wedin**

Syn.: *Collema capniochroum* A. Massal., *Collema fragrans* (Sm.) Ach., *Collema microphyllum* Ach. *nom.illeg.*, *Collema terrulentum* Nyl., *Collemodium microphyllum* (Gray) Nyl. *ex* Lamy, *Enchylium microphyllum* Gray, *Leptogium fragrans* (Sm.) Leight., *Leptogium microphyllum* (Gray) Leight., *Lichen fragrans* Sm.

L – Subs.: cor – Alt.: 1–3 – Note: a mild-temperate lichen found on bark in open but mature, humid, broad-leaved woodlands; widespread, but presently rare in the Alps. – **Au**: V, T, S, K, St, O, N. **Ge**: OB, Schw. **Sw**: BE, GL, TI. **Fr**: AHP, AMa, Sav, HSav, Var, Vau. **It**: Ven, TAA, Lomb, Piem, Lig. **Sl**: SlA.

***Scytinium gelatinosum* (With.) Otálora, P.M. Jørg. & Wedin**

Syn.: *Collema scotinum* (Ach.) Ach., *Leptogium gelatinosum* (With.) J.R. Laundon, *Leptogium scotinum* (Ach.) Fr., Leptogium scotinum (Ach.) Fr. var. sinuatum (Huds.) Torss., *Leptogium sinuatum* (Huds.) A. Massal., *Lichen gelatinosus* With.

L – Subs.: ter-cal, ter-sil, bry, cal – Alt.: 1–5 – Note: a widespread holarctic lichen, most common on base-rich siliceous substrata, especially in open grasslands, well distinguished from the more calcicolous *S. lichenoides*; widespread throughout the Alps. – **Au**: V, T, S, K, St, O, N. **Ge**: OB, Schw. **Sw**: BE, GR, SZ, TI, UW, VD, VS. **Fr**: AHP, HAl, AMa, Drô, Isè, Sav, Var, Vau. **It**: Frl, Ven, TAA, Lomb, Piem, VA, Lig. **Sl**: SlA, Tg.

***Scytinium imbricatum* (P.M. Jørg.) Otálora, P.M. Jørg. & Wedin**

Syn.: *Leptogium imbricatum* P.M. Jørg.

L – Subs.: cal, ter-sil, bry – Alt.: 2–5 – Note: on more or less calciferous ground in alpine grasslands; perhaps more widespread in the Alps, but formerly filed under pulvinate forms of *S. gelatinosum* and *S. lichenoides*. – **Au**: V, T, S, K, St, O, N. **Sw**: BE, FR, GR, SG, SZ, VD, VS. **It**: Piem.

***Scytinium intermedium* (Arnold) Otálora, P.M. Jørg. & Wedin**

Syn.: *Leptogium intermedium* (Arnold) Arnold, *Leptogium minutissimum auct. non* (Flörke) Fr., Leptogium minutissimum (Flörke) Fr. var. intermedium Arnold

L – Subs.: ter-cal, ter-sil, cal, sil, bry, cor-bry – Alt.: 2–5 – Note: a mainly temperate species found on soil, occasionally on the mossy bases of ancient trunks, more rarely on calcareous rocks; widespread throughout the Alps. – **Au**: V, T, S, K, St, O, N. **Ge**: OB, Schw. **Sw**: GR, LU, SZ, TI, UW, VS. **Fr**: AMa, Sav, Var. **It**: Frl, Ven, TAA, Lomb, Piem.

***Scytinium leptogioides* (Anzi) Otálora, P.M. Jørg. & Wedin**

Syn.: *Collema leptogioides* Anzi, *Leptogium diffractum* *sensu* Arnold *non* Kremp. *ex* Körber, *Leptogium marcii* Harm.

L – Subs.: cal – Alt.: 1–2 – Note: a mainly Mediterranean species found on steeply inclined surfaces of calcareous rocks, often on cyanobacterial colonies, sometimes also on walls, with a few records from the Western Alps. – **Sw**: ?BE. **Fr**: AMa. **It**: Lig.

***Scytinium lichenoides* (L.) Otálora, P.M. Jørg. & Wedin**

Syn.: *Collema atrocaeruleum* (Schaer.) Rabenh., *Collema lacerum* (Retz.) DC., *Leptogium atrocaeruleum* (Schaer.) A. Massal., *Leptogium lacerum* (Retz.) Gray, *Leptogium lichenoides* (L.) Zahlbr., *Leptogium lophaeum* (Ach.) Cromb., *Lichen tremelloides* Weiss, *Tremella lichenoides* L.

L – Subs.: cal, bry, bry-cal, deb, cor – Alt.: 1–5 – Note: a widespread holarctic lichen mostly found on soil and amongst mosses in dry grasslands, more rarely on basal parts of trunks. Many records could refer to *S. pulvinatum*; widespread and common throughout the Alps. – **Au**: V, T, S, K, St, O, N, B. **Ge**: OB, Schw. **Sw**: AP, BE, FR, GL, GR, LU, SG, SZ, TI, UR, UW, VD, VS. **Fr**: AHP, HAl, AMa, Isè, Sav, HSav, Var, Vau. **It**: Frl, Ven, TAA, Lomb, Piem, VA, Lig. **Sl**: SlA, Tg. **Li**.

***Scytinium magnussonii* (Degel. & P.M. Jørg.) Otálora, P.M. Jørg. & Wedin**

Syn.: *Leptogium magnussonii* Degel. & P.M. Jørg.

L – Subs.: sil – Alt.: 2–3 – Note: described from Scandinavia, and also known from Western Europe, this species is found in seepage tracks of granitic rocks, gneiss and weakly calcareous rocks below the subalpine belt, with a few records from the Southern and Central Alps. – **Sw**: VS. **It**: Lomb.

***Scytinium massiliense* (Nyl.) Otálora, P.M. Jørg. & Wedin**

Syn.: *Leptogium massiliense* Nyl.

L – Subs.: cal – Alt.: 1–3(-?4) – Note: a mild-temperate to Mediterranean species found on steeply inclined surfaces of calcareous rocks with periodical seepage of water; very rare in the Alps. – **Au**: ?V. **Fr**: AMa, Var, Vau. **It**: Frl.

***Scytinium palmatum* (Huds.) Gray**

Syn.: *Collema corniculatum* Hoffm., *Collema palmatum* (Huds.) Ach., *Leptogium corniculatum* (Hoffm.) Minks, *Leptogium palmatum* (Huds.) Mont., *Lichen palmatus* Huds.

L – Subs.: ter-sil – Alt.: 1–3 – Note: a mild-temperate lichen found amongst terricolous or epilithic mosses in areas with siliceous substrata, sometimes on soil; apparently most frequent in the Western and Southern Alps. – **Au**: N. **Fr**: AHP, AMa, Isè, Var, Vau. **It**: Ven, TAA. **Sl**: Tg.

***Scytinium parvum* (Degel.) Otálora, P.M. Jørg. & Wedin**

Syn.: *Collema parvum* Degel.

L – Subs.: cal – Alt.: 2–5 – Note: on steeply inclined seepage tracks of calcareous rocks with colonies of cyanobacteria, most frequent in upland areas; certainly overlooked or confused with other species in parts of its range. – **Au**: V, S, K, St, O, N. **Ge**: OB, Schw. **Sw**: BE, UW, VD. **Fr**: AMa, HSav. **It**: Frl, Lomb. **Sl**: SlA.

***Scytinium plicatile* (Ach.) Otálora, P.M. Jørg. & Wedin**

Syn.: *Collema hydrocharum* (Ach.) Ach., *Collema plicatile* (Ach.) Nyl., ?*Collema subplicatile* Nyl., *Collemodium plicatile* (Ach.) Nyl., *Leptogium cataclystum* (Körb.) Harm., Leptogium cataclystum (Körb.) Harm. var. fluctuans (Kremp.) Zahlbr., *Leptogium hydrocharum* (Ach.) Zahlbr., *Leptogium plicatile* (Ach.) Leight., *Lichen plicatilis* Ach.

L – Subs.: cal, bry, ter-cal – Alt.: 1–4 – Note: a mainly mild-temperate to Mediterranean lichen found on steeply inclined, but not fully sun-exposed seepage tracks of more or less calcareous rocks; widespread throughout the Alps. – **Au**: V, T, S, K, St, O, N. **Ge**: OB, Schw. **Sw**: BE, GR, LU, SG, UR, VD, VS. **Fr**: HAl, AMa, Isè, Sav, HSav, Var, Vau. **It**: Frl, Ven, TAA, Lomb, Piem, VA, Lig. **Sl**: SlA, Tg.

***Scytinium pulvinatum* (Hoffm.) Otálora, P.M. Jørg. & Wedin**

Syn.: *Collema pulvinatum* Hoffm., Leptogium lacerum (Retz.) Gray var. pulvinatum (Hoffm.) Zahlbr., Leptogium lichenoides (L.) Zahlbr. var. pulvinatum (Hoffm.) Zahlbr., *Leptogium pulvinatum* (Hoffm.) Cromb.

L – Subs.: cal, sil, bry, ter – Alt.: 2–4 – Note: this species occurs among mosses at the base of trees or occasionally directly over bark, but also on walls, rocks or soil in open habitats over acrocarpous mosses, from the coast to the mountains. Though there is molecular evidence that this should be accepted as a species, it is hard to distinguish from extreme forms of *S. lichenoides*. – **Au**: V, T, S, K, St, N. **Ge**: OB, Schw. **Sw**: BE, GR, SZ, VS. **Fr**: AHP, HAl, AMa, Isè, Sav, HSav, Var, Vau. **It**: Frl, Ven, TAA, Lomb, Piem, VA, Lig.

***Scytinium rivale* (Tuck.) Otálora, P.M. Jørg. & Wedin**

Syn.: *Leptogium rivale* Tuck.

L – Subs.: sil-aqu – Alt.: 4 – Note: a species with a grey to green thallus consisting of flat lobes which are *c.* 1 mm wide, attached by hairs to the substrate and paraplectenchymatic throughout, frequent apothecia, and submuriform ascospores; on acidic rocks or overgrowing mosses in or near streams; based on type from the U.S., it is widespread in the Holarctic region, including Western North America and Japan, and rarely occurs also in the Central European mountains, with a single record from the Eastern Alps (Austria). – **Au**: K.

***Scytinium schraderi* (Bernh.) Otálora, P.M. Jørg. & Wedin**

Syn.: *Collema bacillare* (Wallr.) Schaer., *Collemodium schraderi* (Bernh.) Nyl., *Leptogium schraderi* (Bernh.) Nyl., *Lichen schraderi* Bernh.

L – Subs.: ter-sil, sil, ter-cal, bry-cal, cal – Alt.: 1–4 – Note: on more or less calciferous rocks and soil, often on other lichens (*e.g. Romjularia lurida*), sometimes on terricolous mosses; widespread throughout the Alps. – **Au**: T, S, K, St, N. **Ge**: OB. **Sw**: GR, VS. **Fr**: AHP, AMa, Drô, Isè, HSav, Var, Vau. **It**: Frl, Ven, TAA, Lomb, Piem. **Sl**: Tg.

***Scytinium subaridum* (P.M. Jørg. & Goward) Otálora, P.M. Jørg. & Wedin**

Syn.: *Leptogium subaridum* P.M. Jørg. & Goward

L – Subs.: cor – Alt.: 1–2 – Note: a Mediterranean to mild-temperate lichen also known from North America, found on the base of old trees, more rarely on schists, with a single record from the Western Alps (France). – **Fr**: AMa.

***Scytinium subtile* (Schrad.) Otálora, P.M. Jørg. & Wedin**

Syn.: *Collema minutissimum* Flörke *non auct.*, *Collema subtile* (Schrad.) Hoffm., *Homodium subtile* (Schrad.) Boistel, *Leptogium minutissimum* (Flörke) Fr. *non auct.*, *Leptogium subtile* (Schrad.) Torss., *Lichen subtilis* Schrad.

L – Subs.: ter-sil, ter-cal, xyl, deb, cor – Alt.: 2–4 – Note: a mild-temperate lichen found on the basal parts of old trees with a base-rich bark, especially *Juglans, Populus* and *Salix*, sometimes on wood, more rarely on soil. – **Au**: K, St, O, N. **Ge**: OB. **Sw**: BE, GR, SZ, UR, UW, VD, VS. **Fr**: AHP, HAl, AMa, HSav. **It**: Ven, TAA, Lomb, Piem, VA, Lig.

***Scytinium subtorulosum* (Nyl. *ex* Stizenb.) Otálora, P.M. Jørg. & Wedin**

Syn.: *Collema subtorulosum* Nyl. *ex* Stizenb., *Leptogium subtorulosum* (Nyl. *ex* Stizenb.) Degel.

L # – Subs.: sil-aqu, cal-aqu – Alt.: 3 – Note: a species described from Switzerland, with a radiating brownish thallus and flattened marginal branchlets, found on long-time moist siliceous rocks along creeks and rivers, sometimes even submerged; widespread in Europe (including Macaronesia) but rarely collected; from the Alps there are only a few scattered, historical records. – **Ge**: Ge. **Fr**: HSav.

***Scytinium tenuissimum* (Dicks.) Otálora, P.M. Jørg. & Wedin**

Syn.: *Collema tenuissimum* (Dicks.) Hoffm., *Leptogium humosum* Nyl., *Leptogium spongiosum* (Sm.) Nyl., *Leptogium tenuissimum* (Dicks.) Körb., *Lichen tenuissimus* Dicks., *Polychidium tenuissimum* (Dicks.) Trevis.

L – Subs.: bry, ter-cal, ter-bry, cor – Alt.: 1–5 – Note: on base-rich soil, but also on bark, in the basal parts of old trunks, rarely on base-rich rocks; widespread throughout the Alps. – **Au**: T, S, K, St, O, N. **Ge**: OB, Schw. **Sw**: BE, GL, GR, LU, SZ, TI, UR, UW, VD, VS. **Fr**: AHP, HAl, AMa, HSav, Var, Vau. **It**: Frl, Ven, TAA, Lomb, Piem, Lig.

***Scytinium teretiusculum* (Wallr.) Otálora, P.M. Jørg. & Wedin**

Syn.: *Garovaglina microscopica* (Nyl.) Trevis., *Homodium microscopicum* (Nyl.) Boistel, *Leptogium microscopicum* Nyl., *Leptogium teretiusculum* (Wallr.) Arnold, *Parmelia teretiuscula* Wallr.

L – Subs.: bry, cor, ter-cal – Alt.: 1–3 – Note: a mainly temperate, perhaps holarctic lichen found on basal parts of old trees, sometimes also directly on soil or on weathered rocks. – **Au**: V, T, S, K, St. **Ge**: OB, Schw. **Sw**: GL, SZ, TI. **Fr**: AHP, AMa, Var, Vau. **It**: Lig. **Sl**: SlA.

***Scytinium turgidum* (Ach.) Otálora, P.M. Jørg. & Wedin**

Syn.: *Collema turgidum* Ach., *Collemodium turgidum* (Ach.) Nyl., *Leptogium turgidum* (Ach.) Cromb.

L # – Subs.: cal – Alt.: 2–3 – Note: on surfaces of calcareous rocks with some water seepage after rain; apparently most frequent in the Southern and Western Alps. This species could prove to be just a growth form of *S. schraderi*, one where the fruticose part is poorly developed. – **Fr**: Isè, HSav, Var, Vau. **It**: Ven, TAA, Lomb, Piem.

***Seirophora contortuplicata* (Ach.) Frödén**

Syn.: Caloplaca elegans (Link) Th. Fr. var. caespitosa (Müll. Arg.) Zahlbr., *Parmelia contortuplicata* Ach., *Teloschistes contortuplicatus* (Ach.) Clauzade & Rondon, *Xanthoria contortuplicata* (Ach.) Boistel

L – Subs.: cal, ter-cal – Alt.: 3–5 – Note: on south-facing surfaces protected from rain (*e.g.* under overhangs) of calciferous rocks. – **Au**: V, T, St. **Sw**: GR, VD. **Fr**: AHP, HAl, AMa, Sav, Vau. **It**: Frl, Ven, TAA, Lomb, Piem, VA, Lig. **Sl**: SlA.

***Solenopsora candicans* (Dicks.) J. Steiner**

Syn.: *Caloplaca candicans* (Dicks.) Flagey, *Diphratora candicans* (Dicks.) Jatta, *Lecania candicans* (Dicks.) Stizenb., *Lecanora candicans* (Dicks.) Schaer., *Lichen candicans* Dicks., *Placodium candicans* (Dicks.) Duby, *Placolecania candicans* (Dicks.) Zahlbr., *Ricasolia candicans* (Dicks.) A. Massal.

L – Subs.: cal – Alt.: 1–3 – Note: a Mediterranean to mild-temperate species found on calcareous boulders, most often on horizontal surfaces; apparently most frequent in the Southern and Western Alps. – **Sw**: ?VS. **Fr**: AHP, AMa, Drô, Isè, HSav, Var, Vau. **It**: Frl, Ven, TAA, Lomb, Piem, Lig.

***Solenopsora cesatii* (A. Massal.) Zahlbr.**

Syn.: *Berengeria cesatii* (A. Massal.) Trevis., *Diphratora cesatii* (A. Massal.) Jatta, *Lecania cesatii* (A. Massal.) Bagl., *Placolecania cesatii* (A. Massal.) Zahlbr., *Ricasolia cesatii* A. Massal., *Solenopsora carpathica* Pisut & Vězda

L – Subs.: cal – Alt.: 1–2 – Note: a southern lichen found in fissures of calcareous boulders in rather sheltered situations; this and *S. grisea* may be difficult to distinguish, intermediate forms being frequent; most common in the Southern and Western Alps. – **Au**: K, St. **Fr**: AHP, AMa, Var, Vau. **It**: Ven, Lomb, Piem, Lig.

***Solenopsora grisea* (Bagl.) Kotlov**

Syn.: Ricasolia cesatii var. grisea Bagl., *Solenopsora bagliettoana* Tav. ined.

**L** # – Subs.: cal – Alt.: 1–2 – – Note: on calcareous rocks in open to sheltered situations, closely related to, and perhaps just a form of *S. cesatii*. – **It**: Ven, Lomb, Lig.

***Solenopsora holophaea* (Mont.) Samp.**

Syn.: *Candelariella holophaea* (Mont.) Zahlbr., *Lecania holophaea* (Mont.) A.L. Sm., *Lecania requienii* (A. Massal.) Zahlbr., *Lecanora holophaea* (Mont.) Nyl., *Massalongia requienii* (A. Massal.) Jatta, *Pannaria holophaea* (Mont.) B. de Lesd., *Parmelia holophaea* Mont., *Psoroma holophaeum* (Mont.) Pitard & Harm., *Solenopsora requienii* A. Massal., *Squamaria holophaea* (Mont.) H. Olivier, *Thalloidima holophaeum* (Mont.) Arnold

L – Subs.: sil, ter-sil – Alt.: 1 – Note: a Mediterranean-Atlantic lichen found in sheltered crevices of basic siliceous rocks and on soil, especially along the coast, also reported from the base of the Western Alps (France). Probably not closely related to other species of *Solenopsora*, but the synonym *S. requienii* is the type species of the genus! – **Fr**: AMa.

***Solenopsora liparina* (Nyl.) Zahlbr.**

Syn.: *Lecanora liparina* Nyl., Solenopsora cesatii (A. Massal.) Zahlbr. f. liparina (Nyl.) Clauzade & Cl. Roux

L – Subs.: sil – Alt.: 2 – Note: on inclined surfaces of ultrabasic siliceous rocks (*e.g.* serpentine and basalt), often in fissures, in shaded situations also on vertical faces; in the study area only reported from a few localities at the base of the Western Alps. – **Fr**: AMa. **It**: Lig.

**Solenopsora olivacea (Fr.) H. Kilias subsp. olivacea**

Syn.: *Biatora olivacea* Fr., *Biatorina olivacea* (Fr.) Anzi, *Catillaria olivacea* (Fr.) Zahlbr., *Lecanora olivacea* (Fr.) Nyl., *Placodiella olivacea* (Fr.) Szatala, *Ricasolia olivacea* (Fr.) Bagl., *Toninia olivacea* (Fr.) Clauzade

L – Subs.: cal – Alt.: 1–2 – Note: a Mediterranean-Atlantic species found on large boulders of basic siliceous rocks, usually on surfaces near the ground or in seepage tracks, with a few records from the base of the Western Alps (France, Italy). – **Fr**: AHP. **It**: Lig.

**Solenopsora olivacea (Fr.) H. Kilias subsp. olbiensis (Nyl.) Clauzade & Cl. Roux**

Syn.: ?Catillaria olivacea (Fr.) Zahlbr. var. soredifera Zahlbr., *Lecanora olbiensis* Nyl., Toninia olivacea (Fr.) Clauzade var. olbiensis (Nyl.) Clauzade

L # – Subs.: cal – Alt.: 2 – Note: on basic siliceous rocks, often associated with the typical subspecies, but rarer, and bound to more humid and shaded situations. A varietal rank would perhaps be more appropriate. – **Fr**: AHP, AMa, Vau.

***Solenopsora vulturiensis* A. Massal.**

Syn.: Candelariella holophaea (Mont.) Zahlbr. var. glaucospora (Nyl.) Zahlbr., *Candelariella leucospeirea* (Nyl.) Zahlbr., Lecania holophaea (Mont.) A.L. Sm. var. glaucospora (Nyl.) A.L. Sm., *Lecania leucospeirea* (Nyl.) A.L. Sm., Lecanora holophaea (Mont.) Nyl. var. glaucospora Nyl., *Lecanora leucospeirea* Nyl., *Solenopsora leucospeirea* (Nyl.) Zahlbr., *Thalloidima leucospeireum* (Nyl.) Arnold

L – Subs.: sil – Alt.: 1 – Note: a Mediterranean-Atlantic lichen found on basic siliceous substrata, including brick walls, in open to most often sheltered situations, also reported from the limit of the Southern Pre-Alps (France). – **Fr**: Vau.

**Solorina bispora Nyl. subsp. bispora**

L – Subs.: ter-sil, ter-cal – Alt.: 3–6 – Note: an arctic-alpine, circumpolar lichen found on humid soil rich in humus with a long snow cover, with optimum above treeline; widespread throughout the Alps. – **Au**: V, T, S, K, St, O, N. **Ge**: OB. **Sw**: BE, GR, LU, SZ, TI, UR, VD, VS. **Fr**: AHP, HAl, AMa, Sav, HSav, Vau. **It**: Frl, Ven, TAA, Lomb, Piem, VA. **Sl**: SlA. **Li**.

**Solorina bispora Nyl. subsp. macrospora (Harm.) Burgaz & I. Martínez**

L – Syn.: Solorina bispora Nyl. var. macrospora (Harm.) H. Olivier, *Solorina macrospora* Harm.

Subs.: ter-cal – Alt.: 3–5 – Note: distinguished by the larger spores, this subspecies is probably more widespread in the Alps, as it was not always distinguished. – **Au**: V, T, K, St, O. **Sw**: BE, LU, SZ, UR. **It**: Frl, TAA, Piem.

**Solorina bispora Nyl. var. subspongiosa (Zschacke) Frey**

Syn.: Solorina bispora Nyl. f. subspongiosa Zschacke

L – Subs.: ter-cal, ter-sil – Alt.: 4–6 – Note: the morph with a dominant cyanobacterial photobiont in the *S. bispora*-aggregate (comparable to *S. spongiosa* in the *S. saccata*-aggregate); on basic to subneutral soil at high elevations; not always distinguished, and probably more widespread in the Alps. – **Au**: T, K, St. **Sw**: GR. **Fr**: AHP, HAl, Sav.

***Solorina crocea* (L.) Ach.**

Syn.: *Lichen croceus* L., *Peltidea crocea* (L.) Ach., *Peltigera crocea* (L.) Hoffm.

L – Subs.: ter-sil – Alt.: 4–6 – Note: an arctic-alpine, circumpolar lichen found on acid mineral soil with a long snow cover, mostly above treeline; widespread throughout the Alps wherever siliceous substrata are present. – **Au**: V, T, S, K, St, N. **Ge**: OB, Schw. **Sw**: BE, GR, LU, TI, UR, VS. **Fr**: AHP, HAl, AMa, Isè, Sav, HSav. **It**: Frl, Ven, TAA, Lomb, Piem, VA, Lig.

***Solorina monospora* Gyeln.**

Syn.: Solorina bispora Nyl. var. monospora (Gyeln.) Frey

L – Subs.: ter-cal – Alt.: 4–5 – Note: a taxon of the *S. bispora*-group with 1-spored asci and very large (*c.* 120 µm long), usually 2-septate ascospores; on basic soil over shaded limestone or calcareous schists in alpine environments; scattered in the European and Asian mountains, but not consistently distinguished, and perhaps more widespread in the Alps. – **Au**: ?V, S, St, O, N. **Ge**: OB. **Sw**: GR, SZ. **Fr**: Sav.

***Solorina octospora* (Arnold) Arnold**

Syn.: Solorina saccata (L.) Ach. var. octospora Arnold

L – Subs.: ter-sil, bry-ter – Alt.: 4–6 – Note: an arctic-alpine to boreal-montane, probably circumpolar lichen found on soil rich in humus and on terricolous mosses, often in rock fissures. – **Au**: V, T, S, K, St. **Ge**: OB. **Sw**: BE, GR, LU, VD, VS. **Fr**: AHP, HAl, Isè, Sav, HSav. **It**: Ven, TAA, Lomb, Piem.

***Solorina saccata* (L.) Ach.**

Syn.: *Lichen saccatus* L.

L – Subs.: ter-cal, bry-cal, bry-ter – Alt.: 2–5 – Note: a cool-temperate to arctic-alpine, circumpolar lichen found on calciferous soil rich in humus and terricolous mosses, often in cracks of the rock; widespread and often common throughout the Alps. – **Au**: V, T, S, K, St, O, N. **Ge**: OB. **Sw**: BE, FR, GL, GR, LU, SG, SZ, TI, UR, UW, VD, VS. **Fr**: AHP, HAl, AMa, Isè, Sav, HSav, Var, Vau. **It**: Frl, Ven, TAA, Lomb, Piem, VA, Lig. **Sl**: SlA, Tg. **Li**.

***Solorina spongiosa* (Ach.) Anzi**

Syn.: *Collema spongiosum* Ach., Solorina saccata (L.) Ach. var. limbata (Sommerf.) Torss., Solorina saccata (L.) Ach. var. spongiosa (Ach.) Nyl.

L – Subs.: ter-sil, ter-cal – Alt.: 3–6 – Note: an arctic-alpine, circumpolar lichen found on moist calciferous soil; this is the morph with a dominant cyanobiont in the *S. saccata*-complex. – **Au**: V, T, S, K, St, O, N. **Ge**: OB. **Sw**: BE, GR, TI, UR, VD, VS. **Fr**: AHP, HAl, AMa, Sav, HSav. **It**: Frl, Ven, TAA, Lomb, Piem, VA.

***Sparria endlicheri* (Garov.) Ertz & Tehler**

Syn.: *Arthonia decussata* Flot., *Arthonia endlicheri* (Garov.) Oxner, *Arthonia lobata* (Flörke) A. Massal., *Lecanactis lobata* Flörke, *Opegrapha endlicheri* Garov., *Pachnolepia decussata* (Flot.) Körb., *Pachnolepia endlicheri* (Garov.) A. Massal.

L – Subs.: sil, int – Alt.: 1–3 – Note: on vertical to underhanging faces seldom wetted by rain of compact, basic siliceous rocks, much more rarely on weakly calcareous rocks or, exceptionally, on rain-protected bases of ancient trees; rare and perhaps declining in the Alps. – **Au**: K, St. **Sw**: UR. **It**: Ven, TAA, Lomb, Piem, Lig.

***Sphaerophorus fragilis* (L.) Pers.**

Syn.: *Lichen fragilis* L.

L – Subs.: sil, ter-sil – Alt.: 3–6 – Note: an arctic-alpine to boreal-montane, circumpolar lichen of siliceous rocks and mineral soil in very rainy areas, with optimum near or above treeline. – **Au**: V, T, S, K, St, N. **Sw**: BE, GR, UR, VS. **Fr**: HAl, Sav, HSav. **It**: Frl, TAA, Lomb, Piem, VA.

***Sphaerophorus globosus* (Huds.) Vain.**

Syn.: *Lichen globosus* Huds., *Sphaerophorus coralloides* Pers., *Sphaerophorus globiferus* (L.) DC.

L – Subs.: sil, ter-sil, cor – Alt.: 3–4 – Note: restricted to cold-humid areas, mostly on rocks, very rarely at the base of old boles in natural forests; widespread throughout the Alps but very rare and strongly declining, probably extinct in several regions. – **Au**: V, T, S, K, St, N. **Ge**: OB. **Sw**: BE, GL, GR, LU, SZ, TI, UR, UW, VD, VS. **Fr**: Sav, HSav. **It**: Ven, TAA, Lomb, Piem, VA. **Sl**: SlA, Tg.

***Spilonema paradoxum* Bornet**

Syn.: *Spilonema pannosum* Hy, *Spilonema tenellum* Vain.

L – Subs.: sil – Alt.: 1–4 – Note: a widespread lichen found on sun-exposed, inclined to vertical seepage tracks of basic siliceous rocks; perhaps overlooked but certainly not common in the Alps. – **Au**: ?V, T. **Sw**: ?BE. **Fr**: AMa, HSav, Var, Vau. **It**: TAA.

***Spilonema revertens* Nyl.**

Syn.: *Asirosiphon densatulum* Nyl., *Ephebe kerneri* Zukal

L – Subs.: sil, int – Alt.: 2–5 – Note: a species with thalli forming dense, blackish cushions which are several mm across and to *c.* 5 mm thick, composed of erect filaments, basally with blue-green rhizohyphae, and *Stigonema*-photobionts; on damp siliceous rocks along streams, near waterfalls or along seepage tracks; widespread in the Holarctic region, generally not common, but perhaps undercollected and more widespread in the Alps. – **Au**: T. **Sw**: GR, TI. **Fr**: AHP, AMa, HSav, Var.

***Sporastatia polyspora* (Nyl.) Grummann**

Syn.: *Biatorella cinerea* (Schaer.) Th. Fr., *Gyrothecium polysporum* Nyl., Lecidea morio (Duby) Fr. var. cinerea Schaer., *Lecidea nigrocinerea* Nyl., *Sporastatia cinerea* (Schaer.) Körb., Sporastatia morio (Duby) Körb. var. cinerea (Schaer.) Körb.

L – Subs.: sil – Alt.: 4–6 – Note: an arctic-alpine, circumpolar lichen found on steeply inclined to underhanging, surfaces of hard siliceous rocks, with optimum above treeline; widespread throughout the siliceous Alps. – **Au**: V, T, S, K, St, N. **Ge**: Schw. **Sw**: BE, GR, TI, UR, UW, VD, VS. **Fr**: AHP, HAl, AMa, Isè, Sav, HSav. **It**: Frl, Ven, TAA, Lomb, Piem, VA.

***Sporastatia testudinea* (Ach.) A. Massal.**

Syn.: *Acarospora testudinea* (Ach.) A. Massal., *Biatorella morio auct. non* (Duby) Flagey, *Biatorella testudinea* (Ach.) A. Massal., Lecidea cechumena Ach. var. testudinea Ach., *Lecidea morio auct. non* (Duby) Fr., *Sporastatia morio auct. non* (Duby) Körb., *Sporastatia testudinea* (Ach.) A. Massal. var. *coracina auct. p.p.* Th. Fr. *non* (Hoffm.) Bagl. & Carestia, Sporastatia testudinea (Ach.) A. Massal. var. pallens (Fr.) Stein

L – Subs.: sil – Alt.: 3–6 – Note: an arctic-alpine to boreal-montane, circumpolar lichen found on hard siliceous rocks, including pure quartz in wind-exposed sites near or above treeline; widespread and common throughout the siliceous Alps. – **Au**: V, T, S, K, St. **Ge**: Schw. **Sw**: BE, GR, LU, TI, UR, VD, VS. **Fr**: AHP, HAl, AMa, Isè, Sav, HSav. **It**: Frl, Ven, TAA, Lomb, Piem, VA, Lig.

***Sporodictyon bosniacum* (Zahlbr.) ined. (provisionally placed here, ICN Art. 36.1b)**

Syn.: *Polyblastia bosniaca* Zahlbr., *Polyblastia lojkana* Zschacke

L # – Subs.: cal – Alt.: 4–5 – Note: a species said to resemble *Polyblastia turicensis*, a heterotypic synonym of *S. terrestre*, but ascospores much smaller; on calcareous rocks, ecology otherwise poorly known; reported from the mountains of the Balkan Peninsula and Central Europe; from the Alps there are only a few scattered records at high elevations. – **Au**: T. **Sw**: BE, VD. **Fr**: AHP, HAl, Sav.

***Sporodictyon cruentum* (Körb.) Körb.**

Syn.: *Polyblastia cruenta* (Körb.) P. James & Swinscow, *Polyblastia henscheliana* (Körb.) Lönnr., *Segestrella cruenta* Körb., *Sphaeromphale henscheliana* Körb., *Sporodyction henschelianum* (Körb.) Körb., *Verrucaria subumbrina* Nyl.

L – Subs.: sil-aqu – Alt.: 3–5 – Note: a silicicolous species, periodically submerged in montane to Alpine creeks; rarely collected and perhaps more widespread in the Alps. – **Au**: V, T, S, St. **Sw**: BE, GR, UR, VS. **Fr**: Sav. **It**: TAA, Lomb. **Sl**: SlA.

***Sporodictyon schaererianum* A. Massal.**

Syn.: *Polyblastia schaereriana* (A. Massal.) Müll. Arg., *Polyblastia subpyrenophora* (Leight.) Zschacke, *Verrucaria subpyrenophora* Leight.

L – Subs.: cal, int, sil – Alt.: 3–6 – Note: a mainly circum-arctic to boreal-montane lichen found on hard calciferous rocks, often on dolomite, but also on calcareous sandstone and schist in cold-humid situations, with optimum above treeline. – **Au**: V, S, K. **Ge**: OB, Schw. **Sw**: BE, LU, SZ, VD. **Fr**: AMa, HSav. **It**: TAA, Lomb, Piem, VA.

***Sporodictyon terrestre* (Th. Fr.) Savić & Tibell**

Syn.: *Polyblastia fartilis* (Nyl.) Boistel, *Polyblastia inumbrata* (Nyl) Arnold, *Polyblastia sommerfeltii* Lynge, *Polyblastia subviridicans* (Nyl.) A.L. Sm., *Polyblastia tarvesedis* (Anzi) Bagl. & Carestia, *Polyblastia terrestris* Th. Fr

L – Subs.: cal, sil, ter-cal, ter-sil, deb – Alt.: 3–6 – Note: an arctic-alpine, polymorphic species found on calciferous to neutral or slightly acidic siliceous rocks by streams, often in the splash zone, more rarely on base-rich soil (but spreading from small pebbles), sometimes also amongst bryophytes, usually near or above treeline. – **Au**: V, T, S, K, St, O, N. **Sw**: GR, VS. **Fr**: AMa, HSav. **It**: TAA, Lomb, Piem, VA.

***Squamarina cartilaginea* (With.) P. James *s.lat.***

Syn.: *Lecanora benacensis* (A. Massal.) Jatta, *Lecanora cartilaginea* (With.) Ach., *Lecanora crassa* (Huds.) Ach., Lecanora crassa (Huds.) Ach. var. caespitosa (Vill.) Rabenh., *Lichen cartilagineus* With., *Parmularia crassa* (Huds.) Croz., *Placodium crassum* (Huds.) Link, *Placolecanora crassa* (Huds.) B. de Lesd., *Psoroma benacense* A. Massal., *Psoroma crassum* (Huds.) Gray, Squamarina cartilaginea (With.) P. James f. pseudocrassa Mattick, Squamarina cartilaginea (With.) P. James var. pseudocrassa (Mattick) D. Hawksw., *Squamaria crassa* (Huds.) DC., *Squamarina crassa* (Huds.) Poelt, Squamarina crassa (Huds.) Poelt f. pseudocrassa (Mattick) Poelt

L – Subs.: cal, ter-cal – Alt.: 1–6 – Note: a mainly southern, chemically variable species found on calcareous rocks and soil, mostly in dry grasslands; widespread throughout the Alps. – **Au**: K, St, O, N, B. **Ge**: OB. **Sw**: BE, GR, LU, SZ, TI, UW, VD, VS. **Fr**: AHP, HAl, AMa, Drô, Sav, HSav, Var, Vau. **It**: Frl, Ven, TAA, Lomb, Piem, Lig. **Sl**: SlA, Tg.

***Squamarina concrescens* (Müll. Arg.) Poelt**

Syn.: *Lecanora concrescens* (Müll. Arg.) Zahlbr., *Lecanora sublentigera* Jatta, *Placodium concrescens* Müll. Arg.

L – Subs.: ter-cal, cal – Alt.: 1–4 – Note: an often misunderstood species with a southern distribution centered in dry areas, found on base-rich clay soil in clearings of grasslands and garrigues, but also on weathered or fissured rocks; in the Alps it mainly occurs in xerothermic stations. – **Au**: ?Au. **Sw**: VS. **Fr**: AHP, AMa, Sav, Var, Vau. **It**: Lig.

***Squamarina gypsacea* (Sm.) Poelt**

Syn.: *Lecanora fragilis* Zahlbr., *Lecanora gypsacea* (Sm.) Nyl., *Lecanora smithii* Ach., *Lichen gypsaceus* Sm., *Placodium gypsaceum* (Sm.) Trevis., *Psoroma gypsaceum* (Sm.) A. Massal., *Squamaria gypsacea* (Sm.) Nyl., *Squamaria smithii* (Ach.) DC., Squamarina gypsacea (Sm.) Poelt var. subcetrarioides (Zahlbr.) Pišút

L – Subs.: cal, int, ter-cal – Alt.: 1–6 – Note: in fissures of calcareous boulders, with optima both in the Mediterranean-submediterranean belts and in dry-continental parts of the Alps, often growing on the thalli of *Romjularia lurida*. The var. subcetrarioides, found in upland areas and not uncommon in the Alps, is worthy of further study. – **Au**: V, T, S, K, St, O, N. **Ge**: OB. **Sw**: BE, GR, LU, SZ, UR, VD, VS. **Fr**: AHP, HAl, AMa, Isè, Sav, HSav, Var, Vau. **It**: Frl, Ven, TAA, Lomb, Piem, VA, Lig. **Sl**: SlA. **Li**.

***Squamarina lamarckii* (DC.) Poelt**

Syn.: *Lecanora lagascae* Ach., *Lecanora lamarckii* (DC.) Rabenh., *Parmelia lagascae* (Ach.) Spreng., *Placodium lamarckii* (DC.) Müll. Arg., *Psoroma lagascae* (Ach.) Körb., *Squamaria lagascae* (Ach.) Balb., *Urceolaria lamarckii* DC.

L – Subs.: cal, int – Alt.: 3–5 – Note: on steeply inclined surfaces of calcareous rocks with short periods of water seepage after rain, mostly near and above treeline; widespread throughout the Alps, but only locally common. – **Au**: V, T, S, K, St, O, N. **Ge**: OB. **Sw**: BE, GR, LU, SG, UR, UW, VD, VS. **Fr**: AHP, HAl, AMa, Drô, Isè, Sav, HSav. **It**: Frl, Ven, TAA, Lomb, Piem. **Sl**: SlA.

***Squamarina lentigera* (Weber) Poelt**

Syn.: *Lecanora lentigera* (Weber) Ach., *Lichen lentigerus* Weber, *Placodium lentigerum* (Weber) Gray, *Psoroma lentigerum* (Weber) A. Massal., *Squamaria lentigera* (Weber) DC.

L – Subs.: ter-cal, bry-cal – Alt.: 1–3 – Note: a species of dry-continental areas, only locally common, especially on gypsaceous or clayey soil in dry grasslands, also present in dry-warm valleys of the Alps. – **Au**: T, N, B. **Sw**: GR, SZ, TI, VD, VS. **Fr**: AHP, AMa, Drô, Isè, Sav, HSav, Var, Vau. **It**: Frl, Ven, TAA, Lomb, Piem, VA.

***Squamarina nivalis* Frey & Poelt**

L – Subs.: ter-cal, ter-sil, int – Alt.: 5–6 – Note: on wind-exposed outcrops of calcareous schists above treeline, reaching the nival belt in the Alps, where it is probably more widespread, but certainly not common. – **Au**: V, T, S, K. **Sw**: ?Sw. **It**: TAA, VA.

***Squamarina oleosa* (Zahlbr.) Poelt**

Syn.: *Lecanora oleosa* Zahlbr.

L – Subs.: cal – Alt.: 1–3 – Note: a typically submediterranean lichen found in fissures of calcareous rocks; never common, but perhaps more widespread in the Southern Alps. – **Fr**: AHP, AMa, Drô, Isè, Var, Vau. **It**: VA.

***Squamarina pachylepidea* (Hellb.) Poelt**

Syn.: Lecanora crassa (Huds.) Ach. subsp. pachylepidea (Hellb.) Th. Fr., Placodium saxicola (Pollich) Frege var. pachylepideum Hellb.

L # – Subs.: cal – Alt.: 4–5 – Note: a species forming small (*c.* 1 cm in diam.) thalli which are areolate-squamulose in the centre and with marginal lobes (*c.* 2–3 mm long), the apothecia with ochre discs and persistently prominent margins; based on a type from Sweden where it was found on siliceous rocks; the few, calcicolous records from the Alps need confirmation. – **Au**: S. **Sw**: GR.

***Squamarina periculosa* (Dufour *ex* Schaer.) Poelt**

Syn.: Lecanora crassa (Huds.) Ach. var. periculosa Dufour *ex* Schaer., Placolecanora crassa (Huds.) B. de Lesd. var. tricolor B. de Lesd.

L – Subs.: cal – Alt.: 2–4 – Note: in fissures of steeply inclined surfaces of calciferous rocks, with optimum in upland areas. – **Fr**: AHP, HAl, AMa, Var. **It**: Frl, TAA, Lomb, Piem, VA, Lig.

***Squamarina provincialis* Clauzade & Poelt**

L – Subs.: int – Alt.: 2 – Note: a species peculiar in having a rather large, whitish-pruinose thallus with very narrow, deeply incised, convex lobes, and sessile apothecia with brown, non-pruinose discs; on schists with various contents in calcium; so far only known from the Western Alps (France). – **Fr**: Vau.

***Squamarina stella-petraea* Poelt**

L – Subs.: cal, ter-cal – Alt.: 1–3 – Note: a mainly Mediterranean species with outposts in dry-warm areas of the submediterranean belt, found on calcareous rocks or on thin soil layers in dry grasslands; apparently restricted to the Western Alps (France, Italy), at low elevations. – **Fr**: AHP, AMa, Drô, Var, Vau. **It**: Piem, Lig.

***Staurolemma omphalarioides* (Anzi) P.M. Jørg. & Henssen**

Syn.: *Collema omphalarioides* Anzi, *Lempholemma hispanicum* (Samp.) Zahlbr., *Lempholemma omphalarioides* (Anzi) Zahlbr., *Physma hispanicum* Samp., *Physma omphalarioides* (Anzi) Arnold, *Staurolemma dalmaticum* Körb.

L – Subs.: cor – Alt.: 1–2 – Note: a mild-temperate, Mediterranean-Atlantic epiphytic lichen with several records from the base of the Western Alps (France, Italy). – **Fr**: AHP, AMa, Var, Vau. **It**: Lig.

***Staurothele alpina* Zschacke**

L # – Subs.: cal – Alt.: 5 – Note: a species of the *S. clopima*-group with an areolate thallus surrounded by a paler prothallus, narrowly oblong, often 1-septate hymenial algae, and 2-spored asci; on dolomite in the high alpine belt; only recorded from the Swiss Alps. – **Sw**: GR.

***Staurothele ambrosiana* (A. Massal.) Zschacke**

Syn.: *Dermatocarpon ambrosianum* (A. Massal.) A. Massal., *Paraphysorma ambrosianum* A. Massal., *Staurothele catalepta auct. medioeur*. *p.p. non* (Ach.) Blomb. & Forssell

L # – Subs.: cal, int, sil, cal-aqu, sil-aqu – Alt.: 2–5 – Note: on sheltered surfaces of calcareous rocks in humid situations. Related to *S. frustulenta* and *S. areolata*, and perhaps a synonym of one of these two species. – **Au**: V, T, S, K, St, O, N, B. **Fr**: AHP, HAl, Isè, Sav, HSav. **It**: Ven, VA.

***Staurothele areolata* (Ach.) Lettau**

Syn.: *Pyrenula areolata* Ach., *Staurothele clopima auct. non* (Wahlenb.) Th. Fr., ?*Staurothele turgidella* Vain.

L – Subs.: cal, sil – Alt.: 3–5 – Note: an arctic-alpine to boreal-montane, circumpolar species found on calcareous to basic siliceous rocks wetted by rain in open habitats (*e.g.* on boulders in alpine and subalpine grasslands); very common throughout the Alps. – **Au**: V, T, S, K, St, O, N. **Ge**: Ge. **Sw**: BE, GL, GR, SZ, TI, UR, UW, VS. **Fr**: AHP, HAl, AMa, Isè, Sav, HSav. **It**: Frl, Ven, TAA, Lomb, Piem, VA, Lig. **Sl**: SlA.

***Staurothele bacilligera* (Arnold) Arnold**

Syn.: *Polyblastia bacilligera* Arnold

L # – Subs.: cal – Alt.: 2–5 – Note: on calcareous rocks near or above treeline; a taxon which needs further study. – **Au**: V, T, K, St, O, N. **Ge**: OB. **Sw**: BE, SZ, UR, UW, VS. **Fr**: Sav. **It**: Ven, TAA.

***Staurothele caesia* (Arnold) Arnold**

Syn.: *Polyblastia caesia* Arnold; incl. *Staurothele ebborensis* Walt. Watson, *Staurothele saprophila* Arnold

L – Subs.: cal – Alt.: 2–5 – Note: on limestone and dolomite in exposed situations, mostly in upland areas, usually not reaching the alpine belt. – **Au**: T, S, O. **Sw**: BE, UR, UW, VD. **Fr**: AHP, Isè, Sav, HSav. **It**: Ven, TAA.

***Staurothele clopima* (Wahlenb.) Th. Fr.**

Syn.: ?*Staurothele drummondii* (Tuck.) Tuck., *Staurothele fuscocuprea* (Nyl.) Zschacke, *Staurothele perradiata* Lynge, *Staurothele septentrionalis* Lynge, *Verrucaria clopima* Wahlenb., Verrucaria cuprea Eschw. var. fuscocuprea Nyl.

L – Subs.: sil-aqu – Alt.: 3–5 – Note: on siliceous rocks, amphibious in montane to alpine creeks. – **Au**: ?V, T, S, K, St. **Ge**: OB. **Sw**: BE, GR, TI, UR, VS. **Fr**: AHP, HAl, AMa, HSav. **It**: TAA, Lomb.

***Staurothele clopimoides* (Bagl. & Carestia) J. Steiner**

Syn.: *Sphaeromphale clopimoides* (Bagl. & Carestia) Arnold, Stigmatomma fissum (Taylor) Bagl. & Carestia var. clopimoides Bagl. & Carestia

L – Subs.: sil-aqu – Alt.: 4–5 – Note: a probably circumboreal-montane freshwater species found on siliceous rocks in creeks. – **Au**: V, T, S, K, St. **Sw**: BE, GR, VS. **Fr**: AHP, HAl, AMa, Sav, HSav. **It**: TAA, Lomb, Piem.

***Staurothele fissa* (Taylor) Zwackh**

Syn.: *Polyblastia umbrina* (Schaer.) Rostr., *Pyrenula umbrina* Schaer., *Sphaeromphale fissa* (Taylor) Körb., *Sphaeromphale silesiaca* A. Massal., *Staurothele hazslinszkyi* (Körb.) Blomb. & Forssell, *Staurothele inconversa* (Nyl.) Blomb. & Forssell, *Staurothele lithina* *sensu* Zahlbr., *Staurothele oenipontana* Beschel, *Staurothele silesiaca* (A. Massal.) Zschacke, *Staurothele umbrina* (Schaer.) Tuck., *Staurothele viridis* Zschacke, *Thelotrema fissum* (Taylor) Hepp, *Verrucaria fissa* Taylor

L – Subs.: sil-aqu, sil – Alt.: 2–5 – Note: a probably circumpolar lichen growing amphibious in montane to alpine creeks, or on moist siliceous rocks, with a wide altitudinal range. – **Au**: V, T, K, St. **Sw**: BE, GR, UR, VS. **Fr**: AHP, HAl, AMa, Isè, Sav, HSav, Var. **It**: TAA, Lomb, Piem, VA.

***Staurothele frustulenta* Vain.**

Syn.: *Polyblastia spadicea* (Wallr.) Jatta, *Staurothele catalepta auct. p.p. non* (Ach.) Blomb. & Forssell, ?*Staurothele elegans* (Wallr.) Zwackh

L – Subs.: sil, int, cal – Alt.: 2–5 – Note: on calcareous or basic siliceous rocks in open habitats, mostly below the subalpine belt. – **Au**: V, T, S, K, O. **Ge**: OB. **Sw**: BE, GR, LU, SZ, TI, UR, VS. **Fr**: AHP, HAl, AMa, HSav, Vau. **It**: VA, Lig.

***Staurothele geoica* Zschacke**

L # – Subs.: ter, bry, deb – Alt.: 2–5 – Note: on soil amongst bryophytes and on plant debris on more or less calcareous substrata, apparently with a wide altitudinal range. This taxon deserves further study; in the study area only recorded from a single station in the Eastern Alps (Italy). – **It**: Frl.

***Staurothele guestphalica* (J. Lahm *ex* Körb.) Arnold**

Syn.: *Polyblastia guestphalica* J. Lahm *ex* Körb., *Porphyriospora guestphalica* (J. Lahm *ex* Körb.) Arnold, *Staurothele orbicularis auct. non* (A. Massal.) Th. Fr.; incl. *Staurothele dalmatica* Servít

L # – Subs.: cal, int – Alt.: 2–5 – Note: on calcareous walls and boulders, and on large pebbles in dry grasslands; related to *S. orbicularis*, and in need of further study. – **Au**: T, S, O. **Ge**: Ge. **Sw**: BE, UW. **Fr**: AMa, Isè, Vau. **It**: Lig.

***Staurothele hymenogonia* (Nyl.) Th. Fr.**

Syn.: *Polyblastia hymenogonia* (Nyl.) H. Olivier, *Polyblastia spurcella* (Nyl.) A.L. Sm., *Polyblastia ventosa* A. Massal. *non* Arnold, *Staurothele arenarum* B. de Lesd., ?*Staurothele extabescens* (Nyl.) Zahlbr., *Staurothele mediterranea* B. de Lesd., *Staurothele ventosa* (A. Massal.) P. Syd., *Verrucaria hymenogonia* Nyl.

L – Subs.: cal – Alt.: 1–5 – Note: on soft calciferous rocks, including calcareous sandstone and dolomite, sometimes also on concrete, with a wide altitudinal range. – **Au**: T, K, St. **Ge**: Ge. **Sw**: BE, SZ. **Fr**: AHP, AMa, Isè, Sav, Var, Vau. **It**: Ven, TAA, Piem, VA. **Sl**: SlA.

***Staurothele immersa* (Bagl. *ex* A. Massal.) Dalla Torre & Sarnth.**

Syn.: *Porphyriospora immersa* Bagl. *ex* A. Massal.; incl. *Staurothele isarina* Riehm.

L – Subs.: cal – Alt.: 1–2 – Note: on limestone and dolomite, usually on steeply inclined surfaces; this is one of the few species of the genus with optimum below the montane belt; apparently most frequent in the Western and Southern Alps. – **Fr**: AHP, HAl, AMa, Drô, Isè, HSav, Var, Vau. **It**: TAA.

***Staurothele nantiana* (B. de Lesd.) Zschacke**

Syn.: *Polyblastia nantiana* B. de Lesd.

L # – Subs.: cal, cal-aqu – Alt.: 2–4 – Note: a species recalling *Verrucaria hochstetteri*, with a whitish-grey, epilithic thallus, perithecia immersed in hemispherical thalline warts, spherical hymenial algae, and 8-spored asci; on occasionally submerged limestone rocks; all known records are from SW Europe, including the Western Alps (France). – **Fr**: Sav.

***Staurothele oenipontana* Beschel**

L # – Subs.: cal – Alt.: 3 – Note: a species resembling *S. fissa* in the verruculose-areolate, grey-brown thallus and the ellipsoid hymenial algae, but asci 8-spored, and ascospores constantly only 3-septate and soon dark-brown (22–24 × 9–11 µm); on dolomitic boulders along river banks, only recorded from the Eastern Alps (Austria). – **Au**: T.

***Staurothele orbicularis* (A. Massal.) Th. Fr.**

Syn.: *Porphyriospora orbicularis* A. Massal., *Staurothele nigella* (Kremp.) Dalla Torre & Sarnth.; incl. *Staurothele viperae* Servít

L – Subs.: int – Alt.: 3–5 – Note: related to *S. guestphalica*, but with 2-spored asci; on more or less calciferous rocks in upland areas; apparently more frequent in the Western and Southern Alps. – **Ge**: OB. **Fr**: AHP, AMa, Drô, HSav, Var, Vau. **It**: Ven, TAA.

***Staurothele pulvinata* (Th. Fr.) Heiðmarsson**

Syn.: *Endocarpon pulvinatum* Th. Fr., *Dermatocarpon pulvinatum* (Th. Fr.) Körb., *Polyblastia pulvinata* (Th. Fr.) Jatta

L – Subs.: cal, ter-cal – Alt.: 2–3 – Note: an arctic-alpine, circumpolar, peculiar species with subfruticose thalli consisting of several mm long, narrow, more or less erect squamules recalling those of *Endocarpon*-species with cushion-like thalli (the genus in which the species was traditionally classified); on calciferous soil, sometimes on calcareous rocks; most records from the Alps need re-confirmation. – **Sw**: GR, TI, VS. **It**: TAA, Lomb, Piem, VA.

***Staurothele rufa* (A. Massal.) Zschacke**

Syn.: *Polyblastia rufa* A. Massal., *Polyblastia scabrida* (Anzi) Jatta, *Staurothele scabrida* (Anzi) B. de Lesd., *Thelotrema scabridum* Anzi

L – Subs.: cal, int – Alt.: 2–5 – Note: a species with a thin, brownish thallus, black, hemispherically protruding perithecia of variable size (not immersed in thalline warts), and spherical hymenial algae; on limestone and various types of calciferous schists; widespread in Central Europe, but rarely collected, most records being from the montane belt of the Alps. – **Au**: T, S, N. **Ge**: Ge. **Sw**: GR, UR, VS. **Fr**: AHP, HAl, AMa, Isè. **It**: TAA, Lomb, Lig.

***Staurothele rugulosa* (A. Massal.) Arnold**

Syn.: *Polyblastia amphiboloides* (Nyl.) Trevis., *Polyblastia rugulosa* A. Massal., *Staurothele amphiboloides* (Nyl.) Zahlbr., *Staurothele innata* Walt. Watson, *Thelidium hammoniense* Erichsen, *Verrucaria amphiboloides* Nyl.

L – Subs.: cal – Alt.: 2–5 – Note: on limestone, dolomite, calcareous sandstone, often on walls, roofing tiles etc., usually in upland areas; probably more widespread in the Alps. – **Au**: O. **Ge**: Schw. **Fr**: Sav, HSav, Var. **It**: Ven, TAA, Piem, Lig.

***Staurothele rupifraga* (A. Massal.) Arnold**

Syn.: *Polyblastia calcarea* (Nyl.) Parrique, *Polyblastia rupifraga* A. Massal., Polyblastia umbrina (Schaer.) Rostr. var. calcarea (Nyl.) Boistel

L – Subs.: cal – Alt.: 2–5 – Note: a probably arctic-alpine species found on hard limestones and dolomite, with optimum near and above treeline; widespread throughout the Alps. – **Au**: V, T, S, K, St, O, N. **Ge**: OB. **Sw**: BE, GR. **Fr**: AHP, AMa, Drô, Sav, HSav, Var, Vau. **It**: Ven, TAA, Lomb, VA. **Sl**: Tg.

***Staurothele sapaudica* Cl. Roux**

Syn.: *Staurothele sapaudica* Asta, Clauzade & Cl. Roux [invalidly published, ICN Art. 40.1 + 8]

L – Subs.: cal-aqu – Alt.: 4–5 – Note: a species resembling *S. ventosa*, but thallus brown and continuous, ascomata with poorly developed involucrellum, hymenial algae narrowly oblong, and ascospores persistently pale; together with *S. solvens* on periodically moist calcareous rocks, *e.g.* along streams at high elevations; only known from the Alps, with certainty only from the Western Alps (France). – **Au**: ?V. **Fr**: Sav.

**Staurothele solvens (Anzi) Zschacke var. solvens**

Syn.: *Polyblastia solvens* Anzi, ?*Staurothele meylanii* B. de Lesd., Staurothele meylanii B. de Lesd. f. geographica O. Behr *ex* Servít, Staurothele meylanii B. de Lesd. f. papularis Servít, Staurothele meylanii B. de Lesd. f. superba Servít

L – Subs.: cal, cal-aqu – Alt.: 3–5 – Note: a species with a dark brown exciple, a concolorous, shield-like involucrellum, subspherical hymenial algae, 4-spored asci, and persistently subhyaline ascospores; on limestone and dolomite, often along creeks in the alpine belt; only known from the South European mountains (Alps, Pyrenees). – **Au**: ?V, T, S, St, N. **Ge**: Schw. **Sw**: BE, GR, SZ, VD. **Fr**: AHP, Sav. **It**: TAA, Lomb.

**Staurothele solvens (Anzi) Zschacke var. fusca Cl. Roux**

Syn.: Staurothele solvens (Anzi) Zschacke var. fusca Asta, Clauzade & Cl. Roux [invalidly published, ICN Art. 40.1 + 8]

L – Subs.: cal – Alt.: 3–5 – Note: differing from the type variety in the dark brown thallus and the 8-spored asci; on periodically inundated, compact calcareous rocks at mid – to moderately high elevations; so far only known from the Western Alps (France). – **Fr**: AHP, Sav.

**Staurothele solvens (Anzi) Zschacke var. intermedia Cl. Roux & Vivant**

L – Subs.: cal – Alt.: 3–4 – Note: a taxon with a pale brownish-grey thallus as the type variety, but asci 8-spored; on compact calciferous, schistose rocks at mid – to moderately high elevations in the mountains of SW Europe, including the Western Alps (France). – **Fr**: AHP, HAl, AMa.

***Staurothele succedens* (Rehm *ex* Arnold) Arnold**

Syn.: *Polyblastia succedens* Rehm *ex* Arnold

L – Subs.: sil-aqu, cal-aqu, cal – Alt.: 3–5 – Note: on basic siliceous to calcareous rocks with frequent seepage of water, often near creeks, in gorges etc., mostly in upland areas. – **Au**: V, T, S, St, O, N. **Ge**: Ge. **Sw**: VS. **Fr**: AHP, Sav. **It**: TAA, Piem, Lig.

***Steinia geophana* (Nyl.) Stein**

Syn.: *Biatora geophana* (Nyl.) Th. Fr., *Biatorella geophana* (Nyl.) Rehm, *Lecidea boreella* Nyl., *Lecidea geophana* Nyl., ? *Lecidea insita* Stirt., *Lecidea trichogena* Norman, ?*Nesolechia insita* (Stirt.) Vouaux, *Pleolecis geophana* (Nyl.) Clem., *Sarcogyne geophana* (Nyl.) Boistel, *Steinia luridescens* Körb.

L – Subs.: ter-sil, ter-cal, xyl, par, deb – Alt.: 2–4 – Note: an ephemeral, facultatively lichenised species of moist, slightly calciferous soil, rotten wood, small pebbles, terricolous *Peltigera* – and *Solorina*-species and plant debris, often found in rather disturbed habitats such as on earth banks along white roads and on track sides; perhaps overlooked, but certainly never common in the Alps. – **Au**: T, S, K, St, O. **Ge**: OB, Schw. **Sw**: LU, SZ, VS. **Fr**: AHP, Isè, Vau. **It**: TAA.

***Stenhammarella turgida* (Ach.) Hertel**

Syn.: *Biatora turgida* Ach., *Lecidea turgida* (Ach.) A. Dietr., *Porpidia turgida* (Ach.) Cl. Roux & P. Clerc, *Stenhammara turgida* (Ach.) Körb.

L – Subs.: cal, int – Alt.: 4–5 – Note: a specialist of rocks with a low percentage of calcium carbonate, mostly on steeply inclined, north-exposed and rather humid faces; widespread throughout the Alps. – **Au**: V, T, S, K, St. **Ge**: OB, Schw. **Sw**: BE, GR, LU, SZ, TI, UR, UW, VD, VS. **Fr**: HAl, Isè, Sav, HSav. **It**: Frl, Ven, TAA, Lomb, Piem, Lig.

***Stereocaulon alpinum* Laurer**

Syn.: Stereocaulon paschale (L.) Hoffm. var. alpinum (Laurer) Du Rietz, Stereocaulon tomentosum Th. Fr. var. alpinum (Laurer) Th. Fr.; incl. Stereocaulon alpinum Laurer var. erectum Frey

L – Subs.: ter-sil – Alt.: 4–6 – Note: an arctic-alpine, circumpolar early coloniser of mineral soil, especially gravel and sand in the vicinity of glaciers. – **Au**: V, T, S, K, St, N. **Ge**: Ge. **Sw**: BE, GR, LU, SZ, TI, UR, UW, VS. **Fr**: AHP, HAl, AMa, Isè, Sav, HSav, Var. **It**: Frl, Ven, TAA, Lomb, Piem, VA.

***Stereocaulon botryosum* Ach.**

Syn.: Stereocaulon alpinum Laurer var. botryosum (Ach.) Laurer, Stereocaulon evolutum Graewe var. fastigiatum (Anzi) Th. Fr., *Stereocaulon fastigiatum* Anzi

L – Subs.: sil – Alt.: 4–6 – Note: an arctic-alpine, probably circumpolar lichen found on steeply inclined surfaces of siliceous rocks in humid-shaded situations, with optimum above treeline. – **Au**: V, T, S, K, St. **Sw**: BE, GR, LU, TI, UR, VS. **Fr**: HAl, Isè, Sav, HSav. **It**: Frl, TAA, Lomb, Piem, VA.

***Stereocaulon capitellatum* H. Magn.**

Syn.: *Stereocaulon farinaceum* H. Magn.

L – Subs.: ter, sax – Alt.: 4–5 – Note: a rare arctic-alpine, sorediate species with delicate pseudopodetia, cephalodia containing nostociform cyanobacteria, and granular phyllocladia soon transformed into globose soralia, containing atranorin and usually perlatolic and anziaic acids; on stones and acidic soil; in the Alps only recorded from Switzerland. – **Sw**: BE, GR, VS.

***Stereocaulon condensatum* Hoffm.**

Syn.: *Stereocaulon acaulon* Nyl., Stereocaulon condensatum Hoffm. var. acaulon (Nyl.) H. Olivier

L – Subs.: ter-sil – Alt.: 3–4 – Note: a cool-temperate to boreal-montane, circumpolar lichen found on sandy to gravelly, often disturbed soil in open situations, often associated with *Pycnothelia papillaria*. – **Au**: T, S, K, N. **Sw**: UR, VS. **Fr**: HAl, Sav, HSav. **It**: Ven, Lomb, Piem, VA, Lig.

***Stereocaulon coniophyllum* I.M. Lamb**

L – Subs.: sil – Alt.: 4–5 – Note: an arctic-alpine to boreal-montane, circumpolar lichen found on siliceous rocks near and above treeline. – **Au**: T, S. **It**: TAA.

***Stereocaulon dactylophyllum* Flörke**

Syn.: *Stereocaulon coralloides* Fr., Stereocaulon coralloides Fr. var. dactylophyllum (Flörke) Th. Fr., Stereocaulon dactylophyllum Flörke var. occidentale (H. Magn.) Grummann, *Stereocaulon spissum* Nyl. *ex* Hue

L – Subs.: sil – Alt.: 3–5 – Note: an arctic-alpine to boreal-montane, circumpolar lichen found on siliceous rocks, especially on large boulders. – **Au**: V, T, S, K, St. **Ge**: Ge. **Sw**: BE, GR, TI, UR, VS. **Fr**: Sav, HSav, Var. **It**: Ven, TAA, Lomb, Piem, VA.

***Stereocaulon evolutum* Graewe**

Syn.: *Stereocaulon spissum* Nyl. *ex* Hue var. laxum Frey

L – Subs.: ter-sil – Alt.: 4 – Note: a species of the *S. paschale*-group resembling *S. saxatile*, but pseudopodetia more rigid and completely glabrous, with digitate-squamulose bluish-grey phyllocladia, containing atranorin and lobaric acid, rarely fertile and then with diagnostic short-ellipsoid ascospores (to *c.* 30 µm long); widespread in Europe, but most common in the west, surprisingly with a few records from the Eastern Alps only (Austria). – **Au**: S, K.

**Stereocaulon glareosum (Savicz) H. Magn. var. glareosum**

Syn.: Stereocaulon tomentosum Th. Fr. f. glareosum Savicz

L – Subs.: ter-sil – Alt.: 4–6 – Note: an arctic-alpine to boreal-montane, probably circumpolar lichen found on sandy or gravelly ground, such as on banks of streams and in snow-beds, forming low, compact mats; probably more widespread in the Alps. – **Au**: T, S, K. **Sw**: BE, GR, UR, VS. **Fr**: Sav. **It**: Ven.

**Stereocaulon glareosum (Savicz) H. Magn. var. brachyphylloides I.M. Lamb**

L # – Subs.: ter-sil – Alt.: 3–5 – Note: a terricolous taxon differing from the type variety in the granular, verrucose to lobate-verrucose phyllocladia, with atranorin and lobaric acid as in the type variety, based on a type from arctic Alaska; the few records from the Eastern Alps need verification. – **Au**: T, K.

***Stereocaulon grande* (H. Magn.) H. Magn.**

Syn.: Stereocaulon paschale (L.) Hoffm. var. grande H. Magn.

L – Subs.: ter-sil – Alt.: 3–4 – Note: a species of alluvial soils with characters intermediate between *S. paschale* and *S. alpinum*: podetia crenate-squamulose to digitate-squamulose (larger than in *S. paschale*, more delicate and divided than in *S. alpinum*), secondary chemistry with atranorin and lobaric acid; widespread in Europe from the boreal-montane to the subarctic-subalpine zone, but not common; from the Alps there are only some scattered records, but perhaps the species was not always distinguished. – **Au**: T, St. **Sw**: BE. **Fr**: HSav.

***Stereocaulon incrustatum* Flörke**

Syn.: *Stereocaulon abduanum* Anzi, Stereocaulon incrustatum Flörke var. abduanum (Anzi) Frey

L – Subs.: ter-sil – Alt.: 2–5 – Note: an arctic-alpine to boreal-montane, circumpolar lichen found on mineral, nutrient-poor soil, in *Pinus*-woodlands, in the vicinity of glaciers and by rivers, with optimum near treeline. – **Au**: T, K, St. **Sw**: GR, TI, UR, VS. **Fr**: HSav. **It**: Ven, TAA, Lomb, Piem, Lig.

***Stereocaulon nanodes* Tuck.**

Syn.: Stereocaulon alpinum Laurer var. tyroliense (Nyl.) Arnold, *Stereocaulon carinthiacum* Frey, *Stereocaulon hypopetraeum* Vain. *ex* Räsänen, Stereocaulon nanodes Tuck. f. carinthiacum (Frey) I.M. Lamb *ex* Frey, Stereocaulon nanodes Tuck. f. tyroliense (Nyl.) I.M. Lamb, *Stereocaulon ostrobothniae* H. Magn., *Stereocaulon tyroliense* (Nyl.) Lettau

L – Subs.: sil, met – Alt.: 2–6 – Note: an arctic-alpine to boreal-montane, circumpolar lichen found on mineral-rich rocks, also under overhanging faces, on pebbles and large boulders, often on metal-rich substrata and in rather disturbed habitats. – **Au**: V, T, S, K, St, N. **Ge**: OB. **Sw**: BE, GR, TI, UR, VS. **It**: Frl, Ven, TAA, Lomb, Piem.

***Stereocaulon pileatum* Ach.**

Syn.: *Stereocaulon saxonicum* Bachm.

SL – ubstr.: sil, ter-sil – Alt.: 2–4 – Note: a cool-temperate to arctic-alpine, circumpolar early coloniser of mineral-rich siliceous rocks, including roofing tiles, in upland areas, but mostly below the alpine belt. – **Au**: S, K, St, N. **Sw**: BE, TI. **It**: Ven, Lomb, Piem.

***Stereocaulon plicatile* (Leight.) Fryday & Coppins**

Syn.: *Lecidea plicatilis* Leight., *Rhizocarpon plicatile* (Leight.) A.L. Sm., *Rhizocarpon rubescens* Th. Fr.

L – Subs.: sil – Alt.: 3–5 – Note: on siliceous rocks and pebbles in damp habitats with a late snow cover, either on vertical surfaces of north-facing rocks or on stones and pebbles near snow beds; in the study area only recorded from the Eastern Alps (Austria, Italy). – **Au**: ?V, T, K, St. **It**: TAA.

***Stereocaulon rivulorum* H. Magn.**

L – Subs.: ter-sil – Alt.: 4–6 – Note: an arctic-alpine to boreal-montane lichen found on gravel and sand in snow-beds or on banks of streams near glaciers, sometimes on weakly calciferous schists. – **Au**: T, S, K. **Sw**: BE, GR, SG, TI, UR, VS. **Fr**: HAl. **It**: Frl, Lomb, Piem, VA.

***Stereocaulon symphycheilum* I.M. Lamb**

L – Subs.: sil – Alt.: 4–5 – Note: a circum-arctic-alpine, sorediate, silicicolous species resembling *S. vesuvianum* in the phyllocladia with darker centers and paler margins, but pseudopodetia prostrate-decumbent and dorsiventral, with confluent phyllocladia, containing atranorin and lobaric acid, with a few records from the Eastern Alps only (Austria), but perhaps more widespread, and hidden behind some of the Alpine records of *S. vesuvianum*. – **Au**: T, K, St.

***Stereocaulon tomentosum*Fr.**

Syn.: *Stereocaulon botryocarpum* H. Magn., Stereocaulon tomentosum Fr. var. alpestre Flot., Stereocaulon tomentosum Fr. var. campestre Körb.

L – Subs.: ter-sil – Alt.: 3–5 – Note: a mainly boreal-montane, circumpolar lichen found on mineral soil in open habitats, such as in clearings of *Pinus*-stands; several records from the Alps need re-confirmation; see also note on *S. symphycheilum*. – **Au**: T, S, K, St, N. **Ge**: Ge. **Sw**: VS. **Fr**: HAl, Isè, Sav, HSav. **It**: Ven, TAA, Lomb, Piem, VA. **Sl**: SlA.

***Stereocaulon tornense* (H. Magn.) P. James & Purvis**

Syn.: *Bilimbia tornensis* H. Magn.

L – Subs.: sil, ter-sil – Alt.: 4 – Note: a species with a crustose thallus, and therefore difficult to recognise as a *Stereocaulon*, resembling *S. leucophaeopsis*, but areoles smaller, ascospores shorter (to 30 µm long), and secondary chemistry with atranorin and stictic acid; on acidic pebbles in humid situations; widespread in Europe but rare in the study area, being known only from the Eastern Alps (Austria). – **Au**: T.

**Stereocaulon vesuvianum Pers. var. vesuvianum**

L – Subs.: sil, bry-sil – Alt.: 3–5 – Note: on (mostly) acid volcanic rocks; several records from the Alps need re-confirmation. – **Au**: V, T, K, St. **Sw**: BE, GR, TI, UR, UW. **Fr**: HSav. **It**: TAA, Lomb, Piem.

**Stereocaulon vesuvianum Pers. var. nodulosum (Wallr.) I.M. Lamb**

Syn.: *Stereocaulon denudatum* Flörke, Stereocaulon denudatum Flörke var. depressum H. Magn., Stereocaulon vesuvianum Pers. var. denudatum (Flörke) I.M. Lamb *ex* Poelt *comb. inval.*, Stereocaulon vesuvianum Pers. f. depressum (H. Magn.) I.M. Lamb, Stereocaulon vesuvianum Pers. var. depressum (H. Magn.) I.M. Lamb (non *Stereocaulon depressum* (Frey) I.M. Lamb!)

L # – Subs.: sil – Alt.: 5 – Note: a name used for morphs with glabrous podetia and partly confluent phyllocladia forming plate-like squamules, sorediate or not; reported from both Hemispheres, including Europe, but not always distinguished; probably more widespread in the Alps. – **Au**: T.

***Sticta fuliginosa* (Hoffm.) Ach.**

Syn.: *Lobaria fuliginosa* Hoffm., Sticta sylvatica (Huds.) Ach. var. fuliginosa (Hoffm.) Hepp, *Stictina fuliginosa* (Hoffm.) Nyl.

L – Subs.: cor, bry, sil, bry-sil – Alt.: 2–3 – Note: a western species in Europe, found on bark, more rarely on mossy rocks in semi-natural forests; most records date back to the previous century. Material from the Alps should be compared with the recently described *Sticta fuliginoides* Magain & Sérus., whose occurrence in the Alps is possible. – **Au**: V, T, S, K, St, O, N. **Ge**: OB. **Sw**: BE, GR, LU, SG, SZ, TI, UR, UW, VD, VS. **Fr**: AMa, Sav, HSav. **It**: Frl, Ven, TAA, Lomb, Piem, VA. **Sl**: SlA.

***Sticta limbata* (Sm.) Ach.**

Syn.: *Lichen limbatus* Sm., *Stictina limbata* (Sm.) Nyl.

L – Subs.: cor, sax – Alt.: 1–3 – Note: a humid subtropical to Mediterranean-Atlantic species found on bark, often associated with bryophytes, on mossy rocks and soil in very humid situations; very rare and declining in the Alps. – **Au**: S, K. **Sw**: GR, VS. **Fr**: Isè, Var. **It**: Frl, Ven.

***Sticta sylvatica* (Huds.) Ach.**

Syn.: *Lichen sylvaticus* Huds., Stictina fuliginosa (Hoffm.) Nyl. var. sylvatica (Huds.) Flagey, *Stictina sylvatica* (Huds.) Nyl.

L – Subs.: cor, bry, sil, bry-sil – Alt.: 2–3 – Note: a western species in Europe, found on mossy trunks and on epilithic bryophytes in natural forests; widespread throughout the Alps, but presently very rare and declining, several records being old. – **Au**: V, T, S, K, St, O, N. **Ge**: OB, Schw. **Sw**: BE, GL, GR, LU, SG, SZ, TI, UR, UW, VD, VS. **Fr**: Isè, Sav, HSav, Var. **It**: Frl, Ven, TAA, Lomb, Piem, VA. **Sl**: SlA.

***Strangospora deplanata* (Almq.) Clauzade & Cl. Roux**

Syn.: *Biatorella deplanata* Almq.

L – Subs.: cor – Alt.: 2–3 – Note: on *Fraxinus*, *Salix*, *Populus*, and other trees with base-rich bark in rather shaded situations; from the Alps there are only a few scattered records. – **Fr**: AHP. **It**: TAA. **Sl**: SlA.

***Strangospora moriformis* (Ach.) Stein**

Syn.: *Arthonia moriformis* Ach., ?*Biatorella improvisa* (Nyl.) Almq., *Biatorella moriformis* (Ach.) Th. Fr., *Biatorella nitens* Th. Fr.

L – Subs.: xyl, cor – Alt.: 2–4 – Note: on hard lignum (*e.g.* on poles), and on the bark of conifers, rarely of deciduous trees, mostly on the basal parts of old trunks; widespread throughout the Alps, but generally not very common. – **Au**: V, T, S, K, St, O, N. **Ge**: OB. **Sw**: GR, TI, VS. **Fr**: AMa. **It**: TAA, Lomb, Piem, VA. **Sl**: SlA, Tg.

***Strangospora pinicola* (A. Massal.) Körb.**

Syn.: *Biatorella pinicola* (A. Massal.) Anzi, *Sarcogyne pinicola* A. Massal.

L – Subs.: cor, xyl – Alt.: 2–4 – Note: on hard lignum (*e.g.* on poles) and on acid bark, especially of conifers; perhaps more widespread in the Alps. – **Au**: S, K, St, O, N, B. **Sw**: UW. **Fr**: Var. **It**: Frl, Lomb. **Sl**: SlA.

***Strigula affinis* (A. Massal.) R.C. Harris**

Syn.: *Arthopyrenia affinis* (A. Massal.) Boistel, *Porina affinis* (A. Massal.) Zahlbr., *Sagedia affinis* A. Massal.

L – Subs.: cor, bry – Alt.: 1–3 – Note: a mainly temperate species found on the smooth bark of deciduous trees, *e.g. Fraxinus*, *Juglans, Tilia*. – **Au**: St, O, N. **Sw**: BE, LU, SZ, UR. **Fr**: AMa, Sav, Var, Vau. **It**: Frl, Ven, TAA, Lomb, Piem.

***Strigula alpestris* (Vězda) Hafellner**

Syn.: Arthopyrenia faginea (Schaer.) Swinscow var. alpestris (Vězda) Swinscow, Porina faginea (Schaer.) Arnold var. alpestris Vězda, Strigula stigmatella (Ach.) R.C. Harris var. alpestris (Vězda) Coppins

L # – Subs.: ter-cal, deb, bry – Alt.: 3–5 – Note: a critical taxon, often subsumed under *S. stigmatella*, from which it differs in the slightly larger ascospores; on plant debris and encrusting bryophytes, with a boreal-montane to – alpine distribution, reported from Scotland and the Central European mountains; from the Alps there are only scattered records (Austria), but the species might have been not recognised and/or undercollected elsewhere. – **Au**: V, T, S, K, St.

***Strigula angustata* Cl. Roux & Sérus.**

L – Subs.: fol – Alt.: 2 – Note: a species differing from *S. minor* in the longer macroconidia; foliicolous and on the uppermost twigs of *Buxus* in the understory of broad-leaved forests in humid, shaded situations; known from SW Europe and Macaronesia, with a few records from the Western Alps (France). – **Fr**: AMa, Drô, Var, Vau.

***Strigula brevis* Bricaud & Cl. Roux**

L – Subs.: cor – Alt.: 2 – Note: a species resembling *S. taylorii*, from which it differs only in the shorter macroconidia (to *c.* 12 µm long), which however are not always present; on bark of broad-leaved trees in closed forests; known from the lowlands of Western Europe and the laurel-forest belt in Macaronesia, with a few records from the Western Alps (France). – **Fr**: Drô, Vau.

***Strigula buxi* Chodat**

Syn.: *Strigula elegans auct. eur. non* (Fée) Müll. Arg., *Strigula smaragdula auct. eur. non* Fr.

L – Subs.: fol – Alt.: 2–3 – Note: an Atlantic-Macaronesian, easily overlooked species, also known from the Pyrenees and Western France, found on leaves and on the uppermost twigs of *Buxus* in the understorey of broad-leaved forests in humid, shaded situations, with a few records from the Western Alps (France). – **Fr**: AMa, Drô, Isè, HSav.

***Strigula calcarea* Bricaud & Cl. Roux**

L – Subs.: cal – Alt.: 2 – Note: a recently-described species found on limestone in deciduous woodlands, mostly in rather shaded situations; in the study area hitherto known only from the Western Alps, but perhaps more widespread. – **Fr**: AHP, AMa, Vau.

***Strigula cavicola* Cl. Roux & Bricaud**

L – Subs.: cal – Alt.: 2 – Note: a species differing from the similar *S. calcarea* in the shorter macroconidia (to *c.* 15 µm long), while the ascomata are so far unknown; on limestone in the entry areas of caves and in overhangs in gorges, hence in sites with a very stable microclimate; known from SW Europe, including the Western Alps (France). – **Fr**: Vau.

***Strigula endolithea* Cl. Roux & Bricaud**

L – Subs.: cal – Alt.: 2 – Note: a recently-described species, hitherto known from Southern France at the foot of the Alps, and the Park of the Miramare Castle near Trieste (Italy), on shaded calcareous rocks near the coast. – **Fr**: AMa, Vau.

***Strigula glabra* (A. Massal.) V. Wirth**

Syn.: *Porina glabra* (A. Massal.) Zahlbr., *Porina lactea* (Körb.) Mig., *Pyrenula netrospora* Nägeli, *Sagedia candida* Anzi, *Sagedia garovaglii* A. Massal., *Sagedia glabra* A. Massal., *Sagedia phillyreae* Jatta, *Spermatodium glabrum* (A. Massal.) Trevis.

L – Subs.: cor – Alt.: 1–3 – Note: a mild-temperate lichen found on smooth bark of deciduous trees (*e.g. Carpinus*, *Fagus*, *Fraxinus*), especially in humid deciduous woodlands along rivers and creeks. – **Au**: V, S, O, N. **Sw**: BE, GL, GR, UR. **It**: Ven, Lomb.

***Strigula jamesii* (Swinscow) R.C. Harris**

Syn.: *Geisleria jamesii* Swinscow

L – Subs.: cor, bry – Alt.: 2–3 – Note: a species differing from the similar *S. affinis* in the smaller perithecia (to *c.* 0.2 mm in diam.) and macroconidia, found on bark of broad-leaved trees, more rarely on mosses over calciferous substrata, in humid-shaded situations; widespread in Europe, but most common in the West, and also reported from North America. – **Au**: O. **Sw**: BE, LU, SZ. **Fr**: AMa, Var, Vau.

***Strigula minor* (Vězda) Cl. Roux & Sérus.**

Syn.: *Raciborskiella minor* Vězda

L – Subs.: fol – Alt.: 2 – Note: a mild-temperate to Mediterranean-Atlantic foliicolous species with subtropical affinities, described from Georgia and also known from the Pyrenees, with a few records from the base of the Western Alps (France). – **Fr**: AMa, Isè, Vau.

***Strigula muscicola* F. Berger, Coppins, Cl. Roux & Sérus.**

L – Subs.: bry-cal – Alt.: 4 – Note: a boreal-montane species of the *S. affinis*-group with almost sessile, usually aggregated perithecia, encrusting bryophytes in terricolous lichen communities; there are only a few records from Scandinavia, the British Isles and Central Europe, including the Eastern Alps (Austria). – **Au**: O.

***Strigula phaea* (Ach.) R.C. Harris**

Syn.: *Arthopyrenia virella* (G. Merr.) Zahlbr. nom nud., *Porina cineriseda* Müll. Arg., *Verrucaria cineriseda* Nyl. *nom. nud.*, *Verrucaria phaea* Ach.

L – Subs.: cor – Alt.: 2 – Note: a species with a greenish grey, thin, episubstratic thallus, strongly protruding ascomata (0.2–0.35 mm in diam.) at least partly covered by a thin thalline layer, 8-spored, subcylindrical, fissitunicate asci, 1-septate, fusiform ascospores (*c.* 9–13 × 2.5–4 μm) provided with a thin perispore, protruding macropycnidia (to *c.* 15 mm in diam.) with the cavity lined by narrow long conidiogenous cells (to 25 μm long and *c.* 2.5 μm wide) containing 1-septate, oblong macroconidia (7–9 × 2–3 μm) with terminal perisporal appendages; usually corticolous on deciduous trees in shaded-humid sites; widespread but rare in SW Europe and Macaronesia, most common in the Tropics; very rare at low elevations in the Western Alps; the record from Switzerland needs confirmation. – **Sw**: ?BE. **Fr**: AMa.

***Strigula porinoides* Canals, Boqueras & Gómez-Bolea**

L – Subs.: cal – Alt.: 3 – Note: on calcareous rocks, rarely also on smooth bark, mostly in Mediterranean forests; the species is hitherto known only from the island of Marettimo, from Catalonia in Spain, and from the Carnic Alps (Austria). – **Au**: K.

***Strigula stigmatella* (Ach.) R.C. Harris**

Syn.: *Arthopyrenia faginea* (Schaer.) Swinscow, *Arthopyrenia stigmatella* (Ach.) A. Massal., *Arthopyrenia thuretii* (Hepp) H. Olivier, *Lichen stigmatellus* Ach., *Opegrapha thuretii* Hepp, *Porina cinerea* (Pers.) Zahlbr., *Porina faginea* (Schaer.) Arnold, *Porina illinita* Nyl., *Porina muscorum* A. Massal., *Porina tenebricosa* A. Massal., *Porina thuretii* (Hepp) Lettau, *Sagedia illinita* (Nyl.) Hav., *Sagedia tenebricosa* (A. Massal.) Jatta, *Segestrella illinita* (Nyl.) Körb., *Segestria muscorum* (A. Massal.) Trevis., *Verrucaria stigmatella* (Ach.) Ach.

L – Subs.: cor, bry – Alt.: 1–3 – Note: a temperate species found on bark and on epiphytic mosses on basal parts of trunks of deciduous trees, especially *Fagus*; widespread throughout the Alps. – **Au**: V, T, S, K, St, O, N. **Ge**: OB. **Sw**: BE, FR, GL, GR, SG, SZ, UR, UW, VD, VS. **Fr**: Drô, Isè, HSav. **It**: Frl, Ven, TAA, Lomb, Piem, Lig. **Sl**: SlA, Tg.

***Strigula taylori* (Carroll *ex* Nyl.) R.C. Harris**

Syn.: *Porina meliospila* (Nyl.) Zahlbr., *Porina taylorii* (Carroll *ex* Nyl.) Swinscow, *Verrucaria meliospila* Nyl., *Verrucaria taylorii* Carroll *ex* Nyl.

L – Subs.: cor, cal – Alt.: 2–3 – Note: a species with 1-septate, easily fragmenting ascospores, differing from *S. brevis* only in the longer (to *c.* 18 µm long) macroconidia; on bark of broad-leaved deciduous trees (especially *Quercus, Alnus, Fraxinus, Juglans*) in humid-shaded situations, occasionally also on limestone; widespread in Mediterranean and especially Atlantic Europe, and also known from Macaronesia; probably more widespread in the Alps, but largely overlooked. – **Au**: O. **Fr**: HAl, AMa, Drô, Isè, Var, Vau.

***Strigula ziziphi* (A. Massal.) Cl. Roux & Sérus.**

Syn.: *Porina schizospora* Vain., *Porina ziziphi* (A. Massal.) Zahlbr., *Sagedia ziziphi* A. Massal., *Strigula mediterranea* Etayo

L – Subs.: cor – Alt.: 2 – Note: a mild-temperate to Mediterranean-Atlantic lichen found on bark of deciduous trees in open woodlands (*e.g.* on *Quercus*, *Castanea*); apparently more frequent in the Western and Southern Alps. – **Sw**: TI. **Fr**: AHP, AMa, Drô, Var, Vau. **It**: TAA, Lomb.

***Synalissa ramulosa* (Hoffm. *ex* Bernh.) Fr.**

Syn.: *Collema ramulosum* Hoffm. *ex* Bernh., *Collema synalyssum* (Ach.) Ach., *Lichen symphoreus* Ach., *Synalissa symphorea auct. non* (Ach.) Nyl.

L – Subs.: cal, int – Alt.: 1–5 – Note: a mainly southern species in Europe, found on steeply inclined faces of calcareous rocks with periodical water seepage, often overgrowing the thalli of *Romjularia lurida*, with a wide altitudinal range, but with optimum at relatively low elevations; widespread throughout the Alps. – **Au**: V, T, S, K, St, N. **Ge**: OB. **Sw**: BE, GR, LU, SZ, TI, VD, VS. **Fr**: AHP, HAl, AMa, Drô, Isè, Sav, HSav, Var, Vau. **It**: Frl, Ven, TAA, Lomb, Piem, VA, Lig.

***Synalissa violacea* Geitler**

L – Subs.: cal, cal-aqu – Alt.: 3 – Note: a species with a dwarf-fruticose to coralloid, nearly black thallus becoming gelatinous when moist, the outer envelopes of the photobiont colonies (*Gloeocapsa*-like) violet; only known in the sterile state and generic placement therefore uncertain; on moist limestone rocks in gorges and similar sites with high air humidity; so far only recorded from the Eastern (Austria) and the Western Alps (France). – **Au**: K, St, O, N. **Fr**: AHP, AMa, Drô.

***Teloschistes chrysophthalmos* (L.) Th. Fr.**

Syn.: *Borrera chrysophthalmos* (L.) Ach., *Lichen chrysophthalmos* L., *Niorma chrysophthalmos* (L.) S.Y. Kondr., Kärnefelt, Elix, A. Thell, N.H. Jeong & Hur, *Physcia chrysophtalmos* (L.) De Not., *Tornabenia chrysophthalmos* (L.) A. Massal., *Xanthoanaptychia chrysophthalmos* (L.) S.Y. Kondr. & Kärnefelt, *Xanthoria chrysophthalmos* (L.) Stizenb.

L – Subs.: cor – Alt.: 1–2 – Note: based on a type from the Cape Province and there saxicolous (!), and typical of situations with a dry climate with frequent spells of fog, this species growing on twigs of shrubs and isolated trees in open habitats was much more common in the past, and is presently extinct in many regions; in the study area most of the records are from the Southern and the Western Alps, at low elevations. – **Sw**: GR, TI. **Fr**: AHP, AMa, Drô, Sav, Var, Vau. **It**: Frl, Ven, Lomb, Piem.

**Tephromela atra (Huds.) Hafellner var. atra**

Syn.: *Lecanora atra* (Huds.) Ach., *Lecidea atroides* Walt. Watson, *Lichen ater* Huds.

L – Subs.: sil, cor, xyl, int – Alt.: 1–6 – Note: a widespread, holarctic, polymorphic and ecologically wide-ranging species; in the eu-Mediterranean belt it is restricted to sheltered situations, but elsewhere it occurs in sun-exposed habitats; albeit rarely, it can also occur on bark; widespread and locally common throughout the Alps. – **Au**: V, T, S, K, St, N, B. **Sw**: BE, FR, GR, LU, SG, SZ, TI, UR, UW, VD, VS. **Fr**: AHP, HAl, AMa, Isè, Sav, HSav, Var, Vau. **It**: Frl, Ven, TAA, Lomb, Piem, VA, Lig. **Sl**: SlA.

**Tephromela atra (Huds.) Hafellner var. calcarea (Jatta) Clauzade & Cl. Roux**

Syn.: Lecanora atra (Huds.) Ach. var. calcarea Jatta, *Lecanora cypria* Körb., *Tephromela cypria* (Körb.) Hafellner

L – Subs.: cal – Alt.: 2 – Note: on calciferous rocks wetted by rain, mostly in upland areas; much more frequent in the Mediterranean mountains than in the Alps, where there are only a few records from France. – **Fr**: AHP, Var, Vau.

**Tephromela atra (Huds.) Hafellner var. torulosa (Flot.) Hafellner**

Syn.: Lecanora atra (Huds.) Ach. var. corticola (Hepp) Egeling, Lecanora atra (Huds.) Ach. var. torulosa Flot., Tephromela atra (Huds.) Hafellner var. corticola (Hepp) Hafellner & Hierzer (lapsus “Jerzer”) *nom. inval*.

L # – Subs.: cor – Alt.: 1–3 – Note: a temperate lichen found on the bark of deciduous and evergreen broad-leaved trees. This taxon deserves further study: corticolous forms of typical *T. atra* are not rare, but the most common morph differs in several respects from epiphytic samples of *T. atra* *s.str.*, and there is molecular evidence that two taxa are involved; probably more widespread in the Alps, since not always distinguished from the typical variety. – **Au**: V, T, S, K, St, O, N. **Fr**: AHP, AMa, Isè, Var. **It**: Frl, Ven, TAA, Lomb, Piem, Lig. **Sl**: SlA, Tg.

***Tephromela grumosa* (Pers.) Hafellner & Cl. Roux**

Syn.: Lecanora atra (Huds.) Ach. var. grumosa (Pers.) Ach., *Lecanora grumosa* (Pers.) Du Rietz, *Lichen grumosus* Pers.

L – Subs.: sil – Alt.: 2–5 – Note: a mainly cool-temperate lichen found on steeply inclined surfaces of acidic siliceous rocks; perhaps somehow more widespread in the Alps, being often sterile and hence easy to overlook. – **Au**: ?V, T, S. **Fr**: AMa, Isè, HSav, Vau. **It**: TAA, VA.

***Tephromela pertusarioides* (Degel.) Hafellner & Cl. Roux**

Syn.: *Lecanora massalongiana* Gyeln. *non* Zahlbr., *Lecanora pertusarioides* Degel.

L # – Subs.: sil – Alt.: 3–4 – Note: this may be a sorediate form of *T. atra* with an areolate thallus often devoid of apothecia, and with the same secondary chemistry; on siliceous rocks *e.g.* on cliffs in rather open forests; widespread in Europe but apparently rare; from the Alps there are only a few scattered records. – **Au**: ?V, T. **Sw**: GR, TI.

***Tetramelas chloroleucus* (Körb.) A. Nordin**

Syn.: *Buellia chloroleuca* Körb., Buellia parasema De Not. var. saprophila (Ach.) Körb., *Buellia poeltii* T. Schauer, Buellia punctata (Hoffm.) A. Massal. var. saprophila (Ach.) Anzi, *Buellia zahlbruckneri* *sensu* T. Schauer, *Tetramelas poeltii* (T. Schauer) Kalb

L – Subs.: cor, xyl – Alt.: 3–4 – Note: a mainly boreal-montane species found on lignum, more rarely on bark, especially of conifers, with optimum in the upper montane belt. – **Au**: V, T, S, K, St, O, N. **Ge**: OB. **Sw**: LU, VS. **Fr**: AHP, AMa, Drô, HSav. **It**: Frl, Ven, TAA, Lomb, Piem. **Sl**: SlA.

***Tetramelas concinnus* (Th. Fr.) Giralt**

Syn.: *Buellia concinna* Th. Fr., *Buellia nodulosa* (Lynge) H. Magn., *Buellia subconcinna* (Vain.) Zahlbr., *Buellia subviridescens* (Nyl. *ex* Th. Fr.) Vain., *Lecidea perlutescens* Nyl. *ex* Th. Fr., *Lecidea subconcinna* Vain.

L – Subs.: sil, int – Alt.: 4–5 – Note: a cool-temperate to boreal-montane, circumpolar species found on steeply inclined surfaces of hard siliceous rocks, starting the life-cycle on other crustose lichens; from the Alps there are only a few scattered records. – **Au**: T, St. **Sw**: BE, SG. **Fr**: HAl.

***Tetramelas geophilus* (Flörke *ex* Sommerf.) Norman**

Syn.: *Buellia geophila* (Flörke *ex* Sommerf.) Lynge, Buellia insignis Körb. var. geophila (Flörke *ex* Sommerf.) Th. Fr., *Buellia subnivea* (Nyl.) Jatta, *Buellia trifracta* J. Steiner, *Buellia triphragmia* (Nyl.) Arnold *non auct.*, *Diplotomma geophilum* (Flörke *ex* Sommerf.) D.D. Awasthi & S.R. Singh *nom. inval.*, *Lecidea geophila* Flörke *ex* Sommerf., *Lecidea subnivea* Nyl., *Lecidea triphragmia* Nyl. *non auct*.

L – Subs.: ter-cal, ter-sil, deb – Alt.: 4–5 – Note: a mainly boreal-montane to arctic-alpine, circumpolar lichen overgrowing terricolous and epilithic mosses on more or less calciferous substrata. – **Au**: V, T, S, K, St, O, N. **Ge**: OB, Schw. **Sw**: FR, GR, VD, VS. **Fr**: HAl, Sav, HSav. **It**: TAA, Piem, VA.

***Tetramelas insignis* (Körb.) Kalb**

Syn.: Buellia disciformis (Fr.) Mudd var. insignis (Körb.) Flagey, *Buellia insignis* Körb., Buellia insignis Körb. var. muscorum (Schaer.) Körb., Buellia parasema De Not. var. albocincta Th. Fr., Buellia parasema De Not. var. muscorum (Schaer.) Th. Fr., *Lecidea insignis* Nägeli *ex* Hepp *nom. nud*.

L – Subs.: ter, bry, deb, xyl – Alt.: 3–6 – Note: a species of the high European mountains found on terricolous mosses and plant debris, rarely on rock, lignum and bark, especially on basal parts of trunks. – **Au**: V, T, S, K. **Ge**: OB. **Sw**: BE, GR, SZ, TI, UR, VS. **It**: Frl, Ven, TAA, Lomb, Piem, VA.

***Tetramelas papillatus* (Sommerf.) Kalb**

Syn.: *Buellia papillata* (Sommerf.) Tuck., Buellia parasema De Not. var. papillata (Sommerf.) Th. Fr., *Lecidea papillata* Sommerf.

L – Subs.: ter-cal, bry, deb – Alt.: 4–6 – Note: an arctic-alpine lichen found on terricolous bryophytes, mostly above treeline; it is related to *T. insignis*. – **Au**: V, T, S, K, St, O, N. **Ge**: OB, Schw. **Sw**: GR, VS. **Fr**: Sav. **It**: Frl, Lomb, Piem, Lig.

***Tetramelas pulverulentus* (Anzi) A. Nordin & Tibell**

Syn.: *Abrothallus pulverulentus* Anzi, *Buellia convexa* Th. Fr., *Buellia pulverulenta* (Anzi) Jatta, *Diplotomma pulverulentum* (Anzi) D. Hawksw., *Karschia pulverulenta* (Anzi) Körb., *Leciographa muscigenae* (Anzi) Rehm

L – Subs.: bry-par, deb, sil-par – Alt.: 3–5 – Note: a holarctic endoparasitical lichen (it grows inside the thalli of Physciaceae, but with its own photobiont); certainly more widespread in the Alps, but much overlooked in the past; most frequent above treeline, but descending below the oroboreal belt in dry-continental regions. – **Au**: V, T, S, K, St. **Sw**: VS. **Fr**: HAl. **It**: TAA, Lomb, VA.

***Tetramelas thiopolizus* (Nyl.) Giralt & P. Clerc**

Syn.: *Buellia hypophana* (Nyl.) Zahlbr., *Buellia thiopoliza* (Nyl.) Boistel, *Lecidea hypophana* Nyl., *Lecidea thiopoliza* Nyl.

L – Subs.: ter-sil, deb, bry – Alt.: 3–5 – Note: on *Grimmia* spp. and other mosses overgrowing siliceous rocks with optimum above treeline; probably more widespread in the Alps. – **Au**: T, K. **Sw**: VS. **Fr**: HSav. **It**: TAA, VA.

***Tetramelas triphragmioides* (Anzi) A. Nordin & Tibell**

Syn.: Buellia triphragmia (Nyl.) Arnold var. rugulosa Bagl. & Carestia, *Buellia triphragmioides* Anzi, *Diplotomma triphragmioides* (Anzi) Szatala

L – Subs.: xyl, deb – Alt.: 3–5 – Note: on the smooth bark of *Alnus*, *Populus tremula*, *Salix*, etc., more rarely of conifers; this species is characterised by the yellowish thallus, and the C+ reaction of the medulla. – **Au**: T, N. **Ge**: Ge. **Fr**: AHP, HAl, AMa, Isè, HSav. **It**: TAA, Lomb, Piem, VA.

***Thallinocarpon nigritellum* (Lettau) P.M. Jørg.**

Syn.: *Gonohymenia nigritella* (Lettau) Henssen, *Lichinella nigritella* (Lettau) P.P. Moreno & Egea, *Thyrea nigritella* Lettau

L – Subs.: cal, int, sil – Alt.: 2–3 – Note: a species with a lobed to squamulose thallus forming cushions of *c.* 2 cm in diam., the lobes black or partly grey-pruinose and with mostly globose isidia, usually sterile; on steeply inclined surfaces of calcareous or base-rich siliceous rocks in shaded situations or on surfaces with water seepage after rain; widespread in the Northern Hemisphere, probably undercollected in the Alps. – **Au**: T, St, N. **Ge**: Schw. **Sw**: VS. **Fr**: AHP, AMa, Drô, Var, Vau. **It**: Frl, TAA, Piem, Lig.

**Thamnolia vermicularis (Sw.) Schaer. var. vermicularis**

Syn.: *Cenomyce taurica* (Wulfen) Röhl., *Cenomyce vermicularis* (Sw.) Röhl., *Cladonia taurica* (Wulfen) Hoffm., *Cladonia vermicularis* (Sw.) DC., *Lichen vermicularis* Sw.

L – Subs.: ter-cal, ter-sil – Alt.: 3–6 – Note: an arctic-alpine, circum – and bipolar lichen found on wind-exposed alpine tundras, both on calcareous and siliceous substrata; molecular sequence data suggest that the two chemotypes, which are provisionally still accepted here, do not form well-supported, monophyletic lineages. – **Au**: V, T, S, K, St, O, N. **Ge**: OB, Schw. **Sw**: BE, FR, GR, LU, SG, SZ, TI, UR, UW, VD, VS. **Fr**: AHP, HAl, AMa, Isè, Sav, HSav. **It**: Frl, Ven, TAA, Lomb, Piem, VA, Lig. **Sl**: SlA. **Li**.

**Thamnolia vermicularis (Sw.) Schaer. var. subuliformis (Ehrh.) Schaer.**

Syn.: *Lichen subuliformis* Ehrh., *Thamnolia subuliformis* (Ehrh.) W.L. Culb., *Thamnolia subvermicularis* Asahina

L – Subs.: ter-sil, ter-cal – Alt.: 3–5 – Note: an arctic-alpine, circumpolar lichen found in open, wind-exposed alpine tundras; certainly widespread throughout the Alps, but not always distinguished from the typical variety. – **Au**: V, T, S, K, St, O, N. **Ge**: OB. **Fr**: Sav, HSav. **It**: Frl, Ven, TAA, Lomb, Piem, VA. **Sl**: SlA.

***Thelenella justii* (Servít) H. Mayrhofer & Poelt**

Syn.: *Microglaena justii* Servít

L – Subs.: cor – Alt.: 2 – Note: a mild-temperate, mainly western lichen found on the rough bark of broad-leaved trees and shrubs in sheltered situations, with optimum in the submediterranean belt, with a few records from the base of the Western Alps (France). – **Fr**: AHP, Vau.

***Thelenella modesta* (Nyl.) Nyl.**

Syn.: *Dactyloblastus wallrothianus* (Körb.) A. Massal., *Luykenia modesta* (Nyl.) Trevis., *Microglaena modesta* (Nyl.) A.L. Sm., *Microglaena wallrothiana* Körb., Microglaena wallrothiana Körb. var. septentrionalis Th. Fr., *Phlyctis norvegica* Norman, *Polyblastia modesta* (Nyl.) H. Olivier, *Thelenella wallrothiana* (Körb.) Syd., *Verrucaria modesta* Nyl.

L – Subs.: cor – Alt.: 1–3 – Note: a mainly mild-temperate, western lichen found on bark of broad-leaved trees and shrubs in rather sheltered and humid situations; apparently more frequent in the Southern and Western Alps, but generally not common. – **Au**: S. **Sw**: TI, UR. **Fr**: AMa, HSav, Var, Vau. **It**: Ven, Lomb, Piem, Lig.

**Thelenella muscorum (Fr.) Vain. var. muscorum**

Syn.: *Chromatochlamys muscicola* (Ach. *ex* Nyl.) Trevis., *Chromatochlamys muscorum* (Fr.) H. Mayrhofer & Poelt, *Microglaena lesdainii* (Harm.) Tav., *Microglaena leucothelioides* (Vain.) Zahlbr., *Microglaena macrospora* B. de Lesd., *Microglaena muscorum* (Fr.) Th. Fr., *Polyblastia muscorum* (Fr.) Jatta, *Verrucaria muscicola* Ach. *ex* Nyl., *Verrucaria muscorum* Fr. *nom.illeg.*, *Weitenwebera muscorum* (Fr.) Körb.

L – Subs.: bry, deb, cor – Alt.: 2–5 – Note: a holarctic lichen found on moribund pleurocarpous mosses on rocks and soil, more rarely on the basal parts of old trunks, with optimum in the montane belt, whose generic placement is uncertain; widespread throughout the Alps. – **Au**: T, S, K, St, O. **Ge**: OB. **Sw**: BE, GR, UW, VS. **Fr**: AHP, HAl, AMa, HSav, Var, Vau. **It**: Frl, Ven, TAA, Lomb, Piem. **Sl**: Tg.

**Thelenella muscorum (Fr.) Vain. var. octospora (Nyl.) Coppins & Fryday**

Syn.: Chromatochlamys muscorum (Fr.) H. Mayrhofer & Poelt var. octospora (Nyl.) H. Mayrhofer & Poelt, *Verrucaria muscicola* Ach. *ex* Nyl. var. octospora Nyl.

L – Subs.: bry, deb – Alt.: 2–4 – Note: a mainly western lichen in Europe, found on moribund mosses on rocks and soil, mostly in upland areas; from the Alps there are only a few scattered records. – **Au**: K. **Fr**: HAl, Vau. **It**: Piem.

***Thelenella pertusariella* (Nyl.) Vain.**

Syn.: *Microglaena pertusariella* (Nyl.) Norman, *Phlyctis submuriformis* H. Magn., *Verrucaria pertusariella* Nyl.

L – Subs.: cor – Alt.: 3–4 – Note: on the smooth bark of small shrubs in the mountains (*Daphne*, *Rhododendron*, *Salix*, *Sorbus*); from the Alps there are only a few scattered records. – **Au**: V, T. **Sw**: BE, VS.

***Thelenella vezdae* (H. Mayrhofer & Poelt) Coppins & Fryday**

Syn.: *Chromatochlamys vezdae* H. Mayrhofer & Poelt

L – Subs.: xyl, cor – Alt.: 2–4 – Note: an easily overlooked species characterised by minute perithecia with a hyaline wall and submuriform ascospores, whose generic placement is uncertain; on soft decaying wood, occasionally spreading to lignicolous bryophytes in various forest types, with optimum at low to mid-elevations; so far known from Central Europe only, most records being from the Alps, where it was reported from a few scattered localities. – **Au**: K, St. **Fr**: AHP.

***Thelenidia monosporella* Nyl.**

L – Subs.: ter-cal, cal – Alt.: 5 – Note: a species resembling *Thelenella modesta* but smaller, with a very unusual set of characters: delicate interascal filaments, oblong hymenial algae, and 1-spored asci with large, simple ascospores; on calcareous soil, ecology otherwise poorly known; apparently rare, but easy to overlook; the type is from Switzerland (Canton Zürich), near the northern edge of the Alps. – **Fr**: Sav.

***Thelidium absconditum* (Hepp) Rabenh.**

Syn.: Sagedia nigella (Kremp.) Hepp var. abscondita Hepp, *Thelidium rodellense* Lettau

L – Subs.: cal, int – Alt.: 3–5 – Note: on limestone, dolomite, calciferous schists in upland areas; probably more widespread in the Alps. The relationship with *Th. decipiens* remains to be clarified. – **Au**: ?V, T, S, K, St, O, N. **Ge**: OB. **Sw**: BE, GR, VS. **Fr**: AHP, HAl, AMa, Drô, Isè, Sav, HSav. **It**: TAA. **Sl**: SlA.

***Thelidium abstractum* Lettau**

L # – Subs.: cal – Alt.: 5 – Note: a species of the *Th. pyrenophorum*-group with a thin, yellowish-grey, rimose epilithic thallus and smaller protruding ascomata (0.2–0.4 mm in diam.); on calcareous schists at high elevations; so far recorded from a few localities in the Alps. – **Au**: V. **Sw**: BE.

***Thelidium acrotellum* Arnold**

L # – Subs.: cal, sil – Alt.: 2–5 – Note: on more or less calciferous rocks in upland areas; the species is not easy to distinguish from *Th. minutulum*, but has a colourless excipulum and a thin involucrellum; so far recorded from a few localities in the Alps. – **Au**: V, T, St, O. **Fr**: Sav, HSav. **It**: TAA.

***Thelidium aethioboloides* Zschacke**

L – Subs.: cal-aqu – Alt.: 3–5 – Note: an amphibious, but usually not permanently submerged species of calciferous rocks in shaded situations, with optimum in the upper montane belt; perhaps more widespread in the Alps. – **Au**: O. **Ge**: OB, Schw. **Sw**: GR. **Fr**: Sav. **It**: Ven, TAA.

***Thelidium amylaceum* A. Massal.**

L # – Subs.: cal – Alt.: 2–3 (?5) – Note: a species sometimes synonymised with *Th. decipiens*, characterised by a farinose, whitish thallus with a violet to lilac tinge, and minute, bottle-shaped, entirely immersed ascomata which are only visible by the umbilicate ostioles. The type material urgently needs further study, because this is the type species of the genus. – **Sw**: VS. **It**: Ven.

***Thelidium anisosporum* (Müll. Arg.) Zschacke**

Syn.: *Sagedia anisospora* Müll. Arg., *Verrucaria anisosopora* (Müll. Arg.) Stizenb

L # – Subs.: sil – Alt.: 4–5 – Note: a species with a thin, rusty red thallus, immersed perithecia with a black ostiolar region, and narrowly ellipsoid, 1-septate ascospores which are less than 20 µm long; on gneiss in the subalpine to lower alpine belts; only recorded from the Western Alps (Switzerland). – **Sw**: VS.

***Thelidium antonellianum* Bagl. & Carestia**

Syn.: *Involucrothele antonelliana* (Bagl. & Carestia) Servít

L # – Subs.: sil, int – Alt.: 5–6 – Note: a species found on crystalline, weakly calciferous schists above treeline; the type material, from the Italian Alps, was collected at 4,500 m; in the study area only recorded from the Southern and the Western Alps. – **Sw**: VS. **Fr**: AHP, AMa. **It**: TAA, Piem.

***Thelidium aphanes* J. Lahm**

L # – Subs.: cal – Alt.: 2 – Note: a species of the *Th. incavatum*-group with an endolithic, whitish thallus, entirely immersed perithecia with punctiform ostioles, and 3-septate, less than 40 µm long ascospores; on pebbles of limestone, with scattered records in Central Europe, including the Eastern Alps (Austria). – **Au**: O.

***Thelidium arnoldii* Zschacke**

Syn.: *Thelidium bubulcae* *sensu* Arnold *non* A. Massal.

L # – Subs.: cal – Alt.: 3–5 – Note: a species of the *Th. incavatum*-group with somewhat wider, ellipsoid, 1 – to 3-septate ascospores; on limestone, most common at low elevations; the available distributional data are difficult to interpret, because this taxon was not generally accepted. – **Au**: ?V, T, St, O, N.

***Thelidium auruntii* (A. Massal.) Kremp.**

Syn.: *Involucrothele auruntii* (A. Massal.) Servít, *Verrucaria auruntii* A. Massal.

L – Subs.: cal, int – Alt.: 3–5 – Note: differing from *Th. pyrenophorum* in the well-developed, brown thallus and the smaller spores, this species, also known from Scandinavia, grows on limestone, dolomite and calciferous schists in upland areas. – **Au**: ?V, S, O. **Ge**: OB, Schw. **Fr**: AHP, HAl, AMa, Sav, HSav. **It**: Ven, TAA.

***Thelidium austriacum* Zschacke**

Syn.: *Polyblastia austriaca* (Zschacke) Servít

L – Subs.: cal – Alt.: 2 – Note: a species of the *Th. incavatum*-group with smaller ascospores (less than 25 µm long); on limestone, only recorded from the Eastern Alps (Austria). – **Au**: N.

***Thelidium britzelmayrii* (Servít) ined. (provisionally placed here, ICN Art. 36.1b)**

Syn.: *Involucrothele britzelmayrii* Servít 1953 (“britzelmayeri”)

L # – Subs.: sil – Alt.: ? – Note: a species with an epilithic, rimose to areolate, whitish thallus, semi-immersed perithecia in the centre of areoles, with an involucrellum spreading in the upper third, and 1-septate, oblong to ellipsoid ascospores (to *c.* 30 µm long); on siliceous rocks, only known from the type locality in the Eastern Alps (Germany). – **Ge**: Schw.

***Thelidium bubulcae* (A. Massal.) Arnold**

Syn.: *Lithocia bubulcae* A. Massal.

L # – Subs.: cal – Alt.: 2 (?5) – Note: often considered as a synonym of *Th. zwackhii*, this calcicolous species was accepted by Roux et al. (2014); a record from Austria (V) is considered as very dubious by Hafellner and Türk (2016). – **Au**: ?V. **It**: Ven.

***Thelidium circumspersellum* (Nyl.) Zschacke**

Syn.: *Verrucaria circumspersella* Nyl.

L # – Subs.: cal-aqu – Alt.: 2–3 – Note: a calcicolous, aquatic species; hitherto only known from the type locality in Hungary and a single locality in Upper Austria. – **Au**: O.

***Thelidium dactyloideum* Arnold**

L # – Subs.: cal – Alt.: ?3 – Note: a species with a thin, brownish thallus, minute perithecia protruding only with the ostiolar region, a carbonaceus ascomatal wall, and finger-like, 1 – to 3-septate ascospores turning brown with age; on limestone in the montane belt; only known from the Eastern Alps (Slovenia). – **Sl**: Tg.

***Thelidium decipiens* (Hepp *ex* Nyl.) Kremp.**

Syn.: *Amphoridium uberinum* A. Massal., Thelidium amylaceum *auct. non* A. Massal., *Thelidium cinerascens* (Anzi) Servít, *Thelidium coerulescens* Jatta, *Thelidium decipiens* (Hepp *ex* Nyl.) Kremp. var. scrobiculare (Garov.) Arnold, *Thelidium hymenelioides* Körb., *Thelidium immersum* (Leight.) Mudd, *Thelidium leightonii* M. Choisy, *Thelidium pachysporum* Zschacke, *Thelidium scrobiculare* (Garov.) Arnold, *Thelidium thuringiacum* Zschacke, *Thelidium umbrosum* *sensu* Arnold, *Verrucaria immersa* Leight., Verrucaria pyrenophora Ach. var. decipiens Hepp *ex* Nyl., *Verrucaria scrobicularis* Garov.

L – Subs.: cal, int – Alt.: 2–5 – Note: a cool-temperate to arctic-alpine, circumpolar species of calcareous rocks, including large pebbles, in rather sheltered situations, mostly in upland areas, with optimum above treeline; widespread throughout the Alps. – **Au**: V, T, S, K, St, O, N. **Ge**: OB. **Sw**: BE, FR, GR, LU, SZ, UW, VD, VS. **Fr**: AHP, HAl, AMa, Drô, Isè, Sav, HSav, Var, Vau. **It**: Frl, Ven, TAA, Lomb, Piem, VA. **Sl**: SlA, Tg.

***Thelidium decussatum* (Kremp.) Zschacke**

Syn.: *Acrocordia decussata* Kremp., *Involucrothele decussata* (Kremp.) Servít

L # – Subs.: cal – Alt.: 2–3 – Note: a species of the *Th. pyrenophorum*-group with a thin, greyish thallus surrounded and crossed by black lines, somewhat protruding ascomata (less than 0.5 mm in diam.) with an involucrellum reaching down *c.* half the perithecium, and 1-septate ascospores (less than 30 µm long); on sandstone or calcareous rocks, ecology otherwise poorly known; rare in Central Europe, including the Eastern Alps (Austria). – **Au**: St.

***Thelidium dionantense* (Hue) Zschacke**

Syn.: *Verrucaria dionantensis* Hue

L – Subs.: cal – Alt.: 2–5 – Note: on steeply inclined surfaces of calciferous rocks in upland areas; from the Alps there are only a few scattered records. – **Au**: V, T. **Sw**: VS. **Fr**: AHP, AMa.

***Thelidium exile* Arnold**

L – Subs.: cal – Alt.: 3–5 – Note: on more or less calciferous rocks in upland areas. The species was frequently considered as a synonym of *Th. minutulum*, but according to Roux et al. (2014) it differs in having half-protruding perithecia; from the Alps there are only a few scattered records. – **Au**: St. **Fr**: Sav. **It**: TAA.

***Thelidium fontigenum* A. Massal.**

Syn.: *Involucrothele cataractarum* (Hepp) Servít, *Sagedia cataractarum* Hepp, *Thelidium cataractarum* (Hepp) Lönnr.

L – Subs.: sil-aqu, cal-aqu – Alt.: 2–4 – Note: on limestone, dolomite, calcareous sandstone, near creeks and waterfalls in upland areas, but usually below treeline. – **Au**: T, S, K, O, N. **Ge**: Ge. **Sw**: BE. **Fr**: Sav. **It**: Ven, Piem, VA. **Sl**: SlA, Tg.

***Thelidium fumidum* (Nyl.) Hazsl.**

Syn.: *Verrucaria fumida* Nyl.

L # – Subs.: cal – Alt.: 3 – Note: a calcicolous species with a blackish brown, epilithic thallus, a blackish medullary layer, perithecia immersed in thalline warts, with involucrellum reaching down to the base, and 1-septate ascospores (mostly less than 25 µm long); rare throughout Eastern and Central Europe, with a few scattered records from the Alps. – **Au**: St, N. **Fr**: Vau.

***Thelidium gisleri* (Müll. Arg) Zschacke**

Syn.: *Sagedia gisleri* Müll. Arg., *Verrucaria gisleri* (Müll. Arg.) Stizenb.

L – Subs.: cal – Alt.: 4–5 – Note: a species of the *Th. papulare*-group with a very thin, whitish-grey thallus, semi-immersed ascomata (to 0.3 mm in diam.) with an involucrellum reaching down about half the perithecium, and 3-septate, narrowly ellipsoid ascospores (less than 40 µm long); on calcareous rocks at high elevations; known from a few localities in the Alps. – **Au**: St. **Sw**: UR.

***Thelidium globiferum* Servít**

L # – Subs.: cal – Alt.: 3 – Note: a calcicolous species with a minutely granulose, whitish-greyish thallus, immersed perithecia (to 0.6 mm in diam.), and simple to 1-septate, broadly ellipsoid ascospores; only known from the Eastern Alps (Slovenia). – **Sl**: SlA.

***Thelidium grummannii* Servít**

L # – Subs.: cal – Alt.: 4–5 – Note: a calcicolous species with a mainly endolithic, greyish thallus with brown dots, semi-immersed perithecia (to *c.* 0.3 mm in diam.) with a thin thalline annulus, without involucrellum (?), and 1-septate, oblong ascospores (to *c.* 30 µm long); only known from the type locality in the Western Alps (Switzerland). – **Sw**: BE.

***Thelidium helveticum* (Servít) Hafellner**

Syn.: *Involucrothele helvetica* Servít

L # – Subs.: sil – Alt.: 3 – Note: a species resembling *Th. methorium*, with an epilithic, whitish, spreading, rimose to areolate thallus, hemispherically protruding, often crowded to laterally fusing perithecia (to 0.5 mm in diam.), an involucrellum which is loosely attached to the perithecial wall almost down to the base, and 1-septate, broadly ellipsoid ascospores (mostly to *c.* 25 µm long); on siliceous schists, only known from the type locality in the Western Alps (Switzerland). – **Sw**: BE.

***Thelidium impressulum* Zschacke**

L – Subs.: cal – Alt.: 3–5 – Note: a species of the *Th. pyrenophorum*-group forming small, endolithic, whitish thalli and semi-immersed, small perithecia (to *c.* 0.2 mm in diam.) with involucrellum reaching down to the base, and ellipsoid, 1-septate, halonate ascospores (to 15 µm long); on calcareous rocks (dolomite at type locality) at mid-to high elevations; not rare in the Alps but probably regionally still undercollected. – **Au**: St. **Ge**: OB. **Sw**: GR. **Fr**: AHP, Drô, Sav, Vau.

***Thelidium impressum* (Müll. Arg.) Zschacke**

Syn.: *Sagedia impressa* Müll. Arg.

L – Subs.: cal – Alt.: 2–5 – Note: a species of the *Th. pyrenophorum*-group resembling *Verrucaria dufourii*, but perithecia smaller (to 0.3 mm in diam.) with involucrellum reaching down about half of the perithecium, and with ellipsoid, 1-septate ascospores (to 12 µm long); on inclined surfaces of compact calcareous rocks in upland areas. – **Au**: ?V, K, St, O, N. **Sw**: SZ. **Fr**: AHP, Drô, HSav, Vau.

***Thelidium incavatum* Nyl. *ex* Mudd**

Syn.: *Amphoridium umbrosum* A. Massal., *Amphoroblastia incavata* (Nyl. *ex* Mudd) Servít, *Polyblastia incavata* (Nyl. *ex* Mudd) Croz., *Thelidium umbrosum* (A. Massal.) Arnold, *Verrucaria umbrosa* (A. Massal.) Trevis.

L – Subs.: cal, int – Alt.: 2–5 – Note: on small calcareous pebbles close to the ground, usually in upland areas; probably more widespread, but overlooked. *Th. umbrosum* is perhaps an independent species. – **Au**: V, T, S, K, St, O, N. **Ge**: OB. **Sw**: BE, GR, LU, SZ, UR, UW. **Fr**: AHP, HAl, AMa, Drô, Isè, Sav, HSav, Var, Vau. **It**: Frl, Ven, TAA, Piem, Lig. **Sl**: SlA.

***Thelidium inundatum* Zschacke**

L – Subs.: cal-aqu – Alt.: 3 – Note: an endolithic amphibious lichen found in periodically submerged situations, mostly on calcareous substrata in upland areas, but usually below the alpine belt; from the Alps there are only a few scattered records. – **Au**: O. **Fr**: AHP. **It**: Ven, TAA.

***Thelidium klementii* Servít**

L # – Subs.: int-aqu – Alt.: 3 – Note: a species with a thin, whitish, subareolate thallus, semi-immersed perithecia (to *c.* 0.2 mm in diam.), ellipsoid, 1-septate ascospores with some non-septate intermixed (to *c.* 30 µm long); on temporarily submerged calcareous schists; only known from the type locality in the Eastern Alps (Germany). – **Ge**: Schw.

***Thelidium krempelhuberi* (Servít) ined. (provisionally placed here, ICN Art. 36.1b)**

Syn.: *Involucrothele krempelhuberi* Servít

L # – Subs.: cal – Alt.: 4 – Note: a calcicolous species with a thin, whitish thallus and hemispherically protruding ascomata (to 0.25 mm in diam.), a tightly adpressed involucrellum reaching down about half of the perithecium, ellipsoid, non-septate ascospores with *c.* 20% 1-septate ascospores intermingled (to *c.* 20 µm long); only known from the type locality in the Eastern Alps (Germany). – **Ge**: Schw.

***Thelidium methorium* (Nyl.) Hellb.**

Syn.: *Involucrothele aeneovinosa* (Anzi) Servít, *Involucrothele kutakii* (Servít) Servít, *Polyblastia kutakii* Servít, *Sagedia aeneovinosa* Anzi, *Thelidium aeneovinosum* (Anzi) Arnold, Thelidium aeneovinosum (Anzi) Arnold var. kutakii Servít, *Thelidium diaboli* A. Massal., *Thelidium kutakii* (Servít) Servít, *Verrucaria methoria* Nyl.

L – Subs.: sil, sil-aqu, cal, int – Alt.: 3–5 – Note: an arctic-alpine to boreal-montane, probably circumpolar lichen found on periodically submerged siliceous rocks in alpine to montane creeks; widespread throughout the siliceous Alps. – **Au**: V, T, S, K, St. **Sw**: SZ. BE, GR, UR, VS. **Fr**: HAl, AMa, Sav, HSav. **It**: Frl, TAA, Lomb, Piem, Lig.

***Thelidium microbolum* (Tuck.) Hasse**

Syn.: *Verrucaria microbola* Tuck.

L # – Subs.: sil – Alt.: 4 – Note: a species with a thallus consisting of olivaceous-grey granules and 3-septate ascospores (to 30 µm long), based on a calcicolous type from Canada (Ontario); the identity of the single European record from the Austrian Alps (on temporarily inundated schists in the subalpine belt!) needs confirmation. – **Au**: S.

***Thelidium minimum* (A. Massal. *ex* Nyl.) Arnold**

Syn.: *Involucrothele minima* (A. Massal. *ex* Nyl.) Servít, *Verrucaria minima* A. Massal. *ex* Nyl.

L – Subs.: cal – Alt.: 2–5 – Note: on calcareous pebbles, or on rock surfaces close to the ground in upland areas. – **Au**: ?V, T, S, St, N. **Ge**: Ge. **Sw**: GR, LU, SZ, VD. **Fr**: AHP. **It**: TAA.

***Thelidium minutulum* Körb.**

Syn.: *Arthopyrenia mesotropa* (Nyl.) Arnold, *Involucrothele margacea* (Leight.) Servít, *Thelidium aethioboloides* (Nyl.) Vain. *non* Zschacke, *Thelidium eitneri* Zahlbr., *Thelidium hospitum* Arnold, *Thelidium margaceum* (Leight.) Zschacke, *Thelidium mesotropum* (Nyl.) A.L. Sm., *Thelidium terrestre* Walt. Watson

L – Subs.: cal, int, sil-aqu – Alt.: 2–5 – Note: a widespread, cool-temperate to arctic-alpine, circumpolar, pioneer lichen found on calcareous pebbles close to the ground, on roofing tiles and on brick walls, occasionally also in the splash water zone of creeks; probably more widespread in the Alps. – **Au**: T, S, K, St, O. **Ge**: OB, Schw. **Sw**: GR, SZ. **Fr**: AHP, AMa, Vau. **It**: Ven, TAA. **Sl**: SlA.

***Thelidium montanum* (Hepp) Körb.**

Syn.: *Paraphysothele montana* (Körb.) Zschacke, Thelidium nylanderi (Hepp) Lönnr. var. montanum Hepp in Arnold

L – Subs.: cal – Alt.: 3 – Note: this taxon was described by Körber (Parerga: 351, 1863) as a new species, and not as a new combination of an already existing taxon. His description constitutes the protologue. Körber (l.c.) cited *Th. nylanderi* (Hepp) Lönnr. β *montanum* Hepp as a synonym. This infraspecific taxon was published by Arnold (Flora 41: 554 [misprinted “254”], 1858), who attributed both name and description to Hepp. The two taxa are based on the same type. The species has a greyish, farinose thallus and hemispherical, subsessile perithecia with involucrellum reaching down to the base, partly persisting interascal filaments (*fide* Zschacke), and 1-septate, narrowly ellipsoid ascospores with a somewhat wider upper cell (to *c.* 25 µm long); the generic placement is in need of verification; on calcareous rocks at rather low elevations, known from a few localities in the Eastern Alps and the northern foreland. – **Au**: S, O.

***Thelidium nigricans* Zschacke**

L # – Subs.: sil-aqu, cal-aqu – Alt.: 3 – Note: this species is known only from two widely separated collections from Romania and Switzerland (a record from Bavaria is dubious), but was perhaps overlooked elsewhere. It is very similar to *Th. aethioboloides* and molecular data are needed in order to clarify whether they are two genetically distinct species. – **Ge**: ?Schw. **Sw**: BE.

***Thelidium obscurum* (Garov.) Zschacke**

Syn.: *Involucrothele obscura* (Garov.) Servít, Verrucaria olivacea Fr. var. obscura Garov.

L # – Subs.: cal – Alt.: 2–4 – Note: a species with a blackish thallus, entirely immersed perithecia, and 1-septate ascospores (to *c.* 30 µm long); on sheltered calcareous rocks, with a few records only from the Western and Southern Alps. – **Fr**: HAl, Var. **It**: Lomb.

***Thelidium olivaceum* (Fr.) Körb. [*nom.illeg.***]

Syn.: *Arthopyrenia olivacea* (Fr.) A. Massal., *Arthopyrenia pseudolivacea* (Nyl.) H. Olivier, *Involucrothele olivacea* (Fr.) Servít, *Verrucaria olivacea* Fr. (1831) *non* Pers. (1794) *nec* Hoffm. (1796) *nec* Wallr. (1831), *Verrucaria pseudolivacea* Nyl.

L – Subs.: cal – Alt.: 2–5 – Note: a circumboreal-montane species of calcareous rocks. The combination is based on an illegitimate basionym. – **Au**: T, S, St, O. **Sw**: GR, LU, SZ, VD, VS. **Fr**: Vau. **It**: Ven, TAA, Lomb.

***Thelidium papulare* (Fr.) Arnold**

Syn.: *Arthopyrenia sprucei* (Bab.) H. Olivier, *Polyblastia papularis* (Fr.) Servít, *Sagedia lariana* (A. Massal.) Anzi, *Thelidium jurassicum* Zschacke, *Thelidium larianum* A. Massal., *Thelidium pyrenophorum* *sensu* A. Massal., *Thelidium rubellum* A. Massal., *Thelidium sprucei* (Bab.) Lönnr., *Thelidium subpapulare* Zschacke, *Thelidium umbilicatum* Th. Fr., ?*Thelidium variabile* B. de Lesd., *Verrucaria cryptarum* Garov., *Verrucaria leonina* Anzi, *Verrucaria papularis* Fr., *Verrucaria sprucei* Bab.; incl. Thelidium papulare (Fr.) Arnold f. fuscum Zschacke

L – Subs.: cal, int – Alt.: 3–6 – Note: an arctic-alpine to boreal-montane, circumpolar species of calcareous rocks, with optimum on limestone and dolomite, but also found on calciferous schist and sandstone in upland areas, sometimes growing in temporarily submerged sites along creeks; widespread throughout the Alps. – **Au**: V, T, S, K, St, O, N. **Ge**: OB, Schw. **Sw**: BE, GR, LU, SG, SZ, UR, UW, VD, VS. **Fr**: AHP, HAl, AMa, Isè, Sav, HSav, Vau. **It**: Frl, Ven, TAA, Lomb, Piem, VA. **Sl**: SlA.

***Thelidium paneveggiensis* (Servít) ined. (provisionally placed here, ICN Art. 36.1b)**

Syn.: *Involucrothele paneveggiensis* Servít

L # – Subs.: cal – Alt.: 4 – Note: a species with a thin, epilithic, brownish thallus and subsessile, globose ascomata (to *c.* 0.15 mm in diam.), a tightly adpressed involucrellum reaching down to the base of the perithecium, and ellipsoid, non-septate ascospores with *c.* 5% 1-septate ascospores intermingled (to *c.* 20 µm long); on calciferous siliceous rocks along a stream, only known from the type locality in the Eastern Alps (Italy). – **It**: TAA.

***Thelidium parvulum* Arnold**

L # – Subs.: cal – Alt.: 3 – Note: a species with a thin, greenish thallus, protruding, black, hemispherical perithecia lacking an involucrellum, and 1 – to 3-septate ascospores (to *c.* 25 µm long); on sandstone and limestone at low elevations; widespread in extra-Alpine Central Europe, with a single record from the Eastern Alps (Austria). – **Au**: N.

***Thelidium perexiguum* (Müll. Arg.) Zahlbr.**

Syn.: *Sagedia perexigua* Müll. Arg.

L # – Subs.: cal – Alt.: 2 – Note: a species resembling *Staurothele rupifraga*, with a very thin, lead-grey to bluish grey thallus, minute, entirely immersed ascomata, and 3-septate obovoid ascospores (to 35 µm long); on limestone at low elevations; only known from the Western Alps (France). – **Fr**: HSav.

***Thelidium pertusatii* (Garov.) Jatta**

Syn.: *Verrucaria pertusatii* Garov.

L – Subs.: sil-aqu – Alt.: 4–5 – Note: amphibious on frequently wetted siliceous rocks in alpine rivers and irrigated rocks. Type material on granite, near a creek. – **Au**: S, K. **Fr**: AMa. **It**: TAA, Piem, VA.

***Thelidium pluvium* Orange**

L – Subs.: sil-aqu – Alt.: 2–3 – Note: this species grows in the splash zone of small rivers and creeks on siliceous rocks and pebbles, usually in shaded situations; in the study area it was only reported from the Eastern Alps (Austria). – **Au**: St.

***Thelidium pyrenophorellum* (Servít) Hafellner**

Syn.: *Involucrothele pyrenophorella* Servít

L # – Subs.: cal – Alt.: 3 – Note: a calcicolous species with a very thin, epilithic, whitish-greyish thallus with darker dots, black, hemispherically protruding ascomata (to *c.* 0.25 mm in diam.), an involucrellum reaching down about half of the perithecium, adpressed above and slightly spreading further down, and 1-septate, more or less oblong ascospores, the upper cell somewhat wider and the lower cell slightly attenuated (to *c.* 32 µm long); only known from the type locality the Eastern Alps (Austria). – **Au**: O.

***Thelidium pyrenophorum* (Ach.) A. Massal.**

Syn.: *Involucrothele pyrenophora* (Ach.) Servít, *Paraphysothele viridis* (Deakin) Zschacke, *Sagedia pyrenophora* (Ach.) Hepp, *Thelidium borreri* (Leight.) Mudd, *Thelidium explicatum* (Stirt.) Wheldon & A.Wilson, *Thelidium mortensis* Walt. Watson, *Thelidium nylanderi* (Hepp) Lönnr., *Thelidium viride* (Deakin) Zahlbr., *Verrucaria pyrenophora* Ach.; incl. Thelidium pyrenophorum (Ach.) A. Massal. f. intermedium Asta, Clauzade & Cl. Roux

L – Subs.: cal, int – Alt.: 2–5 – Note: a widespread lichen with optimum on limestone and dolomite, but also found on calciferous sandstone; common throughout the Alps. – **Au**: V, T, S, K, St, O, N. **Ge**: OB. **Sw**: BE, GL, GR, LU, SZ, UR, VD, VS. **Fr**: AHP, HAl, AMa, Sav, HSav, Vau. **It**: Frl, Ven, TAA, Lomb, Piem, VA. **Sl**: SlA, Tg.

***Thelidium rehmii* Zschacke**

L – Subs.: sil, sil-aqu – Alt.: 2–3 – Note: a species with a thin, greenish thallus, black, hemispherically protruding perithecia (to *c.* 0.3 mm in diam.) lacking an involucrellum, and 1-septate, ellipsoid ascospores (to *c.* 30 µm long); on sandstone and siliceous schists in humid situations, at low elevations; rare throughout Central Europe; from the Alps there are only a few scattered records. – **Au**: T, S, St, O. **Sw**: SZ.

***Thelidium rivulicola* (Nyl.) Mig.**

Syn.: *Arthopyrenia rivulicola* (Nyl.) Arnold, *Verrucaria rivulicola* Nyl.

L # – Subs.: cal-aqu – Alt.: 3 – Note: a species with a whitish, subfarinose thallus, hemispherically protruding perithecia (to *c.* 0.25 mm in diam.), and 1-septate, oblong ascospores (to *c.* 30 µm long); on periodically submerged calcareous stones, rare in Western and Central Europe, including the Alps (Germany). – **Ge**: OB.

***Thelidium schibleri* Zschacke**

L # – Subs.: cal – Alt.: 3 – Note: a calcicolous species with a blackish-brown, thin thallus, semi-immersed ascomata (to 0.3 mm in diam.) with involucrellum reaching down about half of the perithecium, and 1-septate, broadly ellipsoid ascospores (to *c.* 45 µm long); only known from the type locality. – **Sw**: GR.

***Thelidium scopolianum* Servít**

L # – Subs.: cal – Alt.: 3 – Note: a species with a thin, grey thallus with a violet tinge, hemispherically protruding ascomata (to *c.* 0.5 mm in diam.), and 3-septate, oblong ascospores (to *c.* 55 µm long); on dolomite in humid forested valleys; only known from the type locality in the Eastern Alps (Slovenia). – **Sl**: SlA.

***Thelidium subabsconditum* Eitner**

Syn.: incl. *Thelidium circumvallatum* Zschacke

L – Subs.: cal – Alt.: 3–5 – Note: a species resembling *Th. absconditum* in the entirely immersed perithecia (to *c.* 0.2 mm in diam.) lacking an involucrellum, but with a very thin, bluish grey thallus, and smaller, 1-septate ascospores (less than 25 µm long); in the Alps it is common on inclined surfaces of compact calciferous rocks in rather shaded, non-eutrophicated situations, but it was not always distinguished, and the distribution appears incomplete. – **Au**: ?V, ?T, St. **Fr**: AHP, AMa, Drô, Isè, Sav, HSav, Var, Vau.

***Thelidium submethorium* (Vain.) Zschacke**

Syn.: *Verrucaria submethoria* Vain.

L – Subs.: sil-aqu – Alt.: 2–5 – Note: a rare species of siliceous substrata in clean creeks and rivers of high mountain ranges, with optimum above treeline; in the Alps there are only a few scattered records – **Au**: T, S. **Fr**: AHP. **It**: TAA.

***Thelidium subrimulatum* (Nyl.) Zschacke**

Syn.: *Verrucaria subrimulata* Nyl.

L – Subs.: cal – Alt.: 3–5 – Note: this species, described from the Pyrenees, has been collected from very few localities in upland areas of Southern Europe, on limestone and calcareous schists; from the Alps there are only a few scattered records. – **Au**: V, ?T, St, O. **Fr**: HAl, AMa, Sav. **It**: Frl.

***Thelidium subsimplex* Zschacke**

L – Subs.: cal, int – Alt.: 4–5 – Note: a calcicolous species of the *Th. pyrenophorum*-group with a rather thick, whitish thallus, finally hemispherically protruding ascomata (to *c.* 0.3 mm in diam.) with involucrellum reaching down about two thirds the perithecium, and most ascospores unicellular, with some 1-septate ones intermingled (to *c.* 20 µm long); known from a few scattered localities in the Alps. – **Au**: ?V, T, S, St, O. **Ge**: OB. **Sw**: UW.

***Thelidium tiroliense* Zschacke**

L – Subs.: cal – Alt.: 3–4 – Note: a calcicolous species of the *Th. pyrenophorum*-group with a thin thallus in various shades of brown, subsessile ascomata with involucrellum reaching down to the base of the perithecium (or even closed), and 1-septate, ellipsoid ascospores (to *c.* 20 µm long); known from a few localities in the Eastern Alps (Austria). – **Au**: T, St.

***Thelidium ungeri* Flot. *ex* Körb.**

Syn.: *Verrucaria ungeri* Flot. *ex* Körb. *nom.illeg*.

L – Subs.: cal, int – Alt.: 3–5 – Note: on inclined surfaces of calciferous rocks in upland areas. Closely related to *Th. pyrenophorum*, from which it differs in the thick, verrucose thallus; widespread throughout the Alps. – **Au**: ?V, T, S, K, St, O, N. **Ge**: OB. **Fr**: AHP, HAl, AMa, Sav, HSav, Vau. **It**: Ven, TAA, Lomb, Piem. **Sl**: SlA.

***Thelidium verrucosum* Zschacke**

L – Subs.: cal – Alt.: 3–5 – Note: a calcicolous species of the *Th. papulare*-group with an endolithic thallus, ascomata immersed in up to 1 mm broad, concolorous warts, a spreading involucrellum, and 3-septate ascospores (to *c.* 35 µm long); rare throughout Central Europe, including the Eastern Alps (Austria). – **Au**: St, N.

***Thelidium zahlbruckneri* Servít**

L # – Subs.: cal – Alt.: 2 – Note: a species with a thin, brown to greyish-brown thallus, hemispherically protruding perithecia (to *c.* 0.15 mm in diam.) lacking an involucrellum, and 1-septate, ellipsoid ascospores (to *c.* 15 µm long); on pebbles of calcareous sandstone; only known from the type locality in the Eastern Alps (Austria). – **Au**: N.

***Thelidium zwackhii* (Hepp) A. Massal.**

Syn.: *Sagedia zwackhii* Hepp, *Thelidium fueistingii* *auct. non* Körb., *Thelidium microcarpum* (Davies *ex* Leight.) A.L. Sm., *Thelidium montinii* Beltr., *Thelidium subgelatinosum* Zschacke, Thelidium velutinum *auct. p.p. non* (Bernh.) Körb., *Thelidium xylospilum* (Nyl.) Zschacke, *Verrucaria microcarpa* Davies *ex* Leight., *Verrucaria xylospila* Nyl.

L – Subs.: cal, ter-cal – Alt.: 2–4 – Note: a mainly temperate, ecologically broad-ranging, pioneer species found both on calcareous and on siliceous rocks, and on thin layers of soil, *e.g.* on walls, pebbles, etc., occasionally also in periodically submerged sites; one of the few species of the genus which occur at low altitudes. – **Au**: V, S, ?St, O, N. **Ge**: Ge. **Sw**: GR, SZ. **Fr**: HAl. **It**: Frl, Ven. **Sl**: SlA.

***Thelignya lignyota* (Wahlenb.) P.M. Jørg. & Henssen**

Syn.: *Arctoheppia scholanderi* Lynge, *Porocyphus dispersus* E. Dahl, *Porocyphus ocellatus* (Th. Fr.) Henssen, *Psorotichia fuliginea* (Ach.) Körb., *Psorotichia lignyota* (Wahlenb.) Forssell, *Psorotichia ocellata* (Th. Fr.) Forssell, *Pyrenopsis lignyota* (Wahlenb.) Th. Fr., *Pyrenopsis ocellata* Th. Fr., *Verrucaria fuliginea* Ach., *Verrucaria lignyota* Wahlenb.

L – Subs.: int – Alt.: 3–4 – Note: a more or less arctic-alpine species found on base – or lime-rich siliceous substrata, periodically submerged in cold creeks, or in seepage tracks; so far only recorded from a few scattered localities in the Alps. – **Au**: T, S, St. **Sw**: VS. **Fr**: AMa.

***Thelocarpella gordensis* Nav.-Ros. & Cl. Roux**

Syn.: *Trimmatothelopsis gordensis* (Nav.-Ros. & Cl. Roux) K. Knudsen & Lendemer

L – Subs.: cal – Alt.: 2–3 – Note: a peculiar, extremely rare species with an endolithic thallus, immersed perithecioid ascomata with persistent interascal filaments, polyspored asci, and simple, oblong ascospores (to 6 µm long). The species has a basal position in the *Trimmatothelopsis*-clade, and perhaps belongs there. It grows on steeply inclined surfaces of calciferous rocks in well-lit situations, being only known from Central Europe and the western Mediterranean region; there are a few disjunct lowland records from the Alps; the Austrian one needs confirmation. – **Au**: ?N. **Fr**: Drô, Vau.

***Thelocarpon citrum* (Wallr.) Rossman**

Syn.: *Sphaeria citrum* Wallr., *Thelocarpon arenicola* Vain., *Thelocarpon herteri* J. Lahm, *Thelocarpon vicinellum* Nyl.

L – Subs.: sil – Alt.: 3–4 – Note: an ephemeral species of disturbed habitats, with optimum near treeline; so far only reported from the Eastern Alps (Austria, Italy). – **Au**: N. **It**: TAA.

***Thelocarpon coccosporum* Lettau**

L – Subs.: deb – Alt.: 3 – Note: a species with pale yellow ascomata, peculiar in lacking paraphyses and in the globose ascospores; on stones and plant debris; rare in Central Europe, including the Eastern Alps (Austria). – **Au**: K.

***Thelocarpon epibolum* Nyl.**

Syn.: *Thelocarpon conoidellum* Nyl., Thelocarpon epibolum Nyl. var. epithallinum (Leight. *ex* Nyl.) G. Salisb., *Thelocarpon epithallinum* Leight. *ex* Nyl.

L – Subs.: xyl, bry, ter-sil, par – Alt.: 3–5 – Note: an ephemeral, facultatively lichenised species found on foliose lichens, rotting wood, decaying bryophytes, peaty soil, mostly in upland areas; overlooked, and certainly more widespread in the Alps. In the *Th. epibolum*-group, two taxa are commonly distinguished: the typical variety, and var. epithallinum, lichenicolous on *Baeomyces rufus* and purported to have longer ascospores. On the other hand, *T. epibolum* itself was described as lichenicolous on *Solorina crocea*, and in our opinion var. epithallinum is not clearly distinguishable, its spores lying in the variation range of those of var. epibolum. Kocourková maintains that the two taxa can can be distinguished by the thickness of the interascal filaments and the host selection, assuming that they are specialised in different photobionts, adding a further undescribed taxon with long spores, specialised in the *Peltigera aphthosa*-group. Since we have found many asci with both shorter and longer spores, we merge the two taxa. – **Au**: T, S, K, St, O, N. **Ge**: OB. **Sw**: SZ. **It**: Frl, Ven, TAA. **Sl**: SlA.

***Thelocarpon impressellum* Nyl.**

Syn.: *Ahlesia impressella* (Nyl.) G. Salisb.

L – Subs.: ter-sil, ter-cal, cal, sil – Alt.: 3–5 – Note: a doubtfully lichenised species found on humus-rich soil, mosses, rotten wood and other lichens in upland areas. The only Italian record, growing on *Squamarina cartilaginea*, is dubious. – **Au**: T, S, K, St, O, N. **Ge**: Ge. **Fr**: HAl, HSav. **It**: ?TAA.

***Thelocarpon intermediellum* Nyl.**

Syn.: *Thelocarpon intermixtulum* Nyl.

L – Subs.: xyl, sil – Alt.: 2–4 – Note: a rarely-collected but apparently widespread ephemeral species of siliceous rocks and, occasionally, rotten wood, mostly in upland areas; from the Alps there are only a few scattered records. – **Au**: K, N. **Fr**: Vau. **It**: TAA.

***Thelocarpon laureri* (Flot.) Nyl.**

Syn.: *Sphaeropsis laureri* Flot., *Thelocarpon epilithellum* Nyl., *Thelocarpon interceptum* Nyl., *Thelocarpon prasinellum* Nyl.

L – Subs.: ter-sil, sil, xyl, bry – Alt.: 2–4 – Note: an ephemeral early coloniser of different substrata, including roofing tiles, rotten wood, and soil; perhaps more widespread in the Alps, but very much overlooked. – **Au**: V, T, S, St, O, N. **Ge**: OB. **Sw**: LU, SZ, VS. **It**: TAA.

***Thelocarpon lichenicola* (Fuckel) Poelt & Hafellner**

Syn.: *Ahlesia lichenicola* Fuckel, *Thelocarpon ahlesii* Fuckel, *Thelocarpon applanatum* H. Magn.

L – Subs.: sil-par, ter-sil-par, xyl – Alt.: 2–4 – Note: on clay soil in disturbed sites, often in *Calluna*-heaths, mostly on *Baeomyces rufus*; doubtfully lichenised, to be searched for further in the Alps. – **Au**: St, N. **It**: TAA.

***Thelocarpon olivaceum* B. de Lesd.**

Syn.: Thelocarpon intermixtulum Nyl. var. olivaceum (B. de Lesd.) H. Magn.

L # – Subs.: sil – Alt.: 3 – Note: a species with hemispherical, variably pruinose ascomata, externally with a thalline sheath, poorly developed to lacking paraphyses, and finally subglobose ascospores; rare throughout Europe, with a single record from the Eastern Alps (Austria). – **Au**: N.

***Thelocarpon saxicola* (Zahlbr.) H. Magn.**

Syn.: Thelocarpon epibolum Nyl. var. saxicola Zahlbr.

L # – Subs.: sil – Alt.: 3 – Note: a species with globose ascomata lacking paraphyses, and ellipsoid ascospores (to 7 µm long); on sandstone and similar rocks in the montane belt; only known from the Eastern Alps (Austria). – **Au**: N.

***Thelocarpon sphaerosporum* H. Magn.**

Syn.: *Ahlesia sphaerospora* (H. Magn.) G. Salisb.

L – Subs.: ter-par, bry – Alt.: 3 – Note: an ephemeral early coloniser of different substrata, including the thalli of other lichens, mostly in upland areas; from the Alps there are only a few scattered records. – **Au**: T, K, O. **Fr**: Sav. **It**: TAA.

***Thelocarpon superellum* Nyl.**

Syn.: *Thelocarpon conoideum* Höhn.

L – Subs.: ter-sil, cor, xyl – Alt.: 3 – Note: a species with relatively conspicuous, conical to globose ascomata, unbranched paraphyses, and oblong, often pseudoseptate ascospores (to 13 µm long); on soil, rotting wood or stones; widespread in the Holarctic region, from the boreal to the temperate zone, but rare; from the Alps there are a few scattered records, but perhaps the species was largely overlooked. – **Au**: T, N. **Sw**: SZ, UW.

***Thelopsis flaveola* Arnold**

L – Subs.: cor – Alt.: 3–5 – Note: a mainly temperate species found on bark of ancient deciduous trees, but also, in the subalpine belt, on bases of old *Rhododendron* shrubs; to be looked for further in the Alps. – **Au**: V, T, K, St. **Ge**: OB. **Sw**: BE, SZ. **It**: Lomb.

***Thelopsis lojkana* (Poetsch *ex* Arnold) Nyl.**

Syn.: *Sagedia lojkana* Poetsch *ex* Arnold, *Thelopsis tholoides* Lettau

L – Subs.: cal, int, cal-aqu – Alt.: 3 – Note: a species with smooth ascomata which are blackish brown in the upper half, immersed at first and later protruding, and 3-septate, halonate ascospores (to 25 µm long), found on vertical to overhanging faces of limestone in the shade of montane forests; rare in the Central European mountains, with a few scattered records from the Alps. – **Au**: S, St, O. **Sw**: BE. **Sl**: SlA.

***Thelopsis melathelia* Nyl.**

Syn.: *Sagedia melathelia* (Nyl.) Jatta, *Sagedia rugosa* Anzi, *Thelopsis rugosa* (Anzi) Jatta, *Thelopsis umbratula* Nyl.

L – Subs.: bry, deb, ter-cal, bry-cal – Alt.: 4–5 – Note: an arctic-alpine, circumpolar species found on moribund bryophytes, humic soil and plant remains over more or less calcareous substrata; widespread throughout the Alps. – **Au**: V, T, S, K, St, O, N. **Ge**: OB, Schw. **Sw**: BE, GR, SG, SZ. **Fr**: HAl. **It**: Frl, Ven, TAA, Lomb, Piem.

***Thelopsis rubella* Nyl.**

Syn.: *Pyrenula bayrhofferi* (Körb.) Hepp, *Sagedia rubella* (Nyl.) Jatta

L – Subs.: cor – Alt.: 1–3 – Note: a mild-temperate species found on old deciduous trees (*e.g.* *Fagus*, *Quercus*), especially near the base of the boles, in areas with high rainfall; widespread, but generally rare in the Alps. – **Au**: St, N. **Ge**: OB. **Sw**: GR, SG, SZ, TI, VS. **Fr**: Var, Vau. **It**: Frl, Lomb.

***Thelotrema lepadinum* (Ach.) Ach.**

Syn.: *Lichen lepadinus* Ach., *Volvaria lepadina* (Ach.) A. Massal.

L – Subs.: cor – Alt.: 1–3 – Note: a mild-temperate lichen found on the bark of *Fagus* and *Abies*, more rarely of other broad-leaved trees in humid montane forests with frequent fog or near rivers; widespread throughout the Alps, but generally not common, and perhaps declining. – **Au**: V, T, S, K, St, O, N. **Ge**: OB. **Sw**: BE, FR, GL, GR, LU, SG, SZ, UR, UW, VD, VS. **Fr**: AMa. **It**: Frl, Ven, TAA, Lomb, Piem. **Sl**: SlA, Tg.

***Thelotrema suecicum* (H. Magn.) P. James**

Syn.: *Ocellularia suecica* H. Magn.

L – Subs.: cor – Alt.: 3 – Note: a rarely collected species found on bark in humid forests; in the study area so far recorded from the Eastern Alps only (Austria, Italy). – **Au**: St, O, N. **It**: Frl.

***Thermutis velutina* (Ach.) Flot.**

Syn.: *Collema pannosum* Hoffm., *Collema velutinum* (Ach.) Ach., Collema velutinum (Ach.) Ach. var. pannosum (Hoffm.) Rabenh., *Gonionema velutinum* (Ach.) Nyl., *Lichen velutinus* Ach.

L – Subs.: sil, int – Alt.: 3–4 – Note: on base-or mineral-rich siliceous rock, in sun-exposed seepage tracks with colonies of cyanobacteria, mostly in upland areas but usually below treeline. – **Au**: ?V, T, S, K, St, N, B. **Ge**: OB. **Sw**: BE, GL, GR, SG, SZ, TI, VS. **Fr**: AMa, HSav. **It**: Frl, Ven, TAA, Lomb, Piem, Lig.

***Tholurna dissimilis* (Norman) Norman**

Syn.: *Podocratera dissimilis* Norman

L – Subs.: cor – Alt.: 4 – Note: a peculiar species with brownish grey, cushion-like, dense, radially arranged podetia arising from a crustose basal thallus, apical ascomata with a black, epruinose mazaedium, and 1-septate ascospores with a helicoid perispore; on the top of not too tall spruce trees often visited by birds; widespread in the Holarctic region, with a single record from the Eastern Alps (Austria). – **Au**: S.

***Thrombium aoristum* (Nyl.) Arnold**

Syn.: *Verrucaria aorista* Nyl.

L # – Subs.: ter-sil – Alt.: 2 – Note: this name is used for a lichen differing from typical *Th. epigaeum* in the hyaline ascomatal wall (except the blackish ostiolar region), and perhaps designates only a morphotype; on acidic soil, mainly in Western Europe, including the limit of the Southern Pre-Alps (France). – **Fr**: Vau.

***Thrombium epigaeum* (Pers.) Wallr.**

Syn.: *Sphaeria epigaea* Pers., *Thrombium aoristoides* I.M. Lamb, *Verrucaria epigaea* (Pers.) Ach.

L – Subs.: ter-sil, ter-cal – Alt.: 2–5 – Note: an ephemeral, probably holarctic coloniser of calciferous, clayey soil in rather disturbed habitats, such as track sides and openings in grasslands; widespread throughout the Alps. – **Au**: V, T, S, K, St, N. **Ge**: OB, Schw. **Sw**: BE, GR, UR, VS. **Fr**: AHP, AMa, Sav, HSav. **It**: Frl, Ven, TAA, Lomb, Piem, Lig. **Sl**: SlA.

***Thyrea confusa* Henssen**

Syn.: Omphalaria pulvinata *auct. non* (Schaer.) Nyl., Thyrea pulvinata *auct. non* (Schaer.) A. Massal.

L – Subs.: cal – Alt.: 1–4 – Note: on steeply inclined, sunny faces of calcareous rocks with short periods of water seepage after rain; widespread throughout the Alps. – **Au**:  V, T, S, K, St, N. **Sw**: TI, UW, VD, VS. **Fr**: AHP, AMa, Isè, Var, Vau. **It**: Frl, Ven, TAA, Lomb, Piem, Lig.

***Thyrea girardii* (Durieu & Mont.) Bagl. & Carestia**

Syn.: *Collema girardii* Durieu & Mont.

L – Subs.: cal – Alt.: 1–2 – Note: a Mediterranean to mild-temperate species found on calcareous rocks; ecology and distribution resemble those of *Th. confusa*; several records from the Alps need confirmation. – **Sw**: ?GR, ?VS. **Fr**: AHP, AMa. **It**: Frl, Ven, Lomb, Piem.

***Thyrea pachyphylla* (Müll. Arg.) Henssen**

Syn.: ?Omphalaria pulvinata (Schaer.) Nyl. var. laxa Müll. Arg., Omphalaria pulvinata (Schaer.) Nyl. var. pachyphylla Müll. Arg.

L # – Subs.: cal – Alt.: 2 – Note: a species resembling *Th. girardii*, but with a polyphyllous thallus, found on humid calcareous rocks at low elevations; only known from the type locality in the Western Alps (Switzerland). – **Sw**: VS.

***Thyrea plectopsora* A. Massal.**

Syn.: *Omphalaria phylliscoides* Nyl., ?*Thyrea nummularioides* (Nyl.) A. Massal., *Thyrea phylliscoides* (Nyl.) Zahlbr.

L – Subs.: cal-aqu – Alt.: 1–2 – Note: on steeply inclined seepage tracks of calcareous rocks; apparently more frequent in the Southern and Western Alps. – **Sw**: ?TI. **Fr**: AHP, AMa, Var, Vau. **It**: Ven, Lig.

***Timdalia intricata* (H. Magn.) Hafellner**

Syn.: *Acarospora intricata* H. Magn.

L – Subs.: sil, met, int – Alt.: 4–5 – Note: on base-rich siliceous rocks in sunny, exposed sites, with optimum above treeline; rarely collected, but perhaps more widespread in the Alps. – **Au**: T, S, St. **It**: TAA, Lomb.

***Toensbergia leucococca* (R. Sant.) Bendiksby & Timdal**

Syn.: *Hypocenomyce leucococca* R. Sant., *Pycnora leucococca* (R. Sant.) R. Sant.

L – Subs.: cor – Alt.: 2–4 – Note: a peculiar, obligately sterile species with a thallus consisting of scattered, whitish, adnate areolae and usually marginal soralia, containing alectorialic acid; on bark of deciduous trees in various forest types; widespread in the Holarctic region from the boreal to the nemoral-montane zone, including the Alps, but still overlooked in some regions. – **Au**: T, S, K, St, O, N. **Ge**: Schw. **Sw**: SZ, UW.

***Toninia albilabra* (Dufour) H. Olivier**

Syn.: *Biatora albilabra* Dufour, *Lecidea albilabra* (Dufour) Dufour, *Psora albilabra* (Dufour) Körb. *non auct.*, *Toninia albomarginata* B. de Lesd.

L – Subs.: ter-cal – Alt.: 1–3 – Note: a mainly Mediterranean species found on more or less calciferous ground and in fissures of rocks and walls, often on cyanobacteria or cyanobacterial lichens when young; common only in dry areas, including the Alpine valleys with a continental climate. – **Au**: T. **Sw**: VS. **Fr**: Var. **It**: TAA, VA, Lig.

***Toninia alutacea* (Anzi) Jatta**

Syn.: *Biatorina alutacea* (Anzi) Jatta, *Thalloidima alutaceum* Anzi, *Thalloidima intermedium* A. Massal. *ex* Arnold, *Toninia intermedia* (A. Massal. *ex* Arnold) H. Olivier, ?*Toninia subcandida* B. de Lesd.

L – Subs.: cal – Alt.: 2–5 – Note: an arctic-alpine, circumpolar species with southern outposts in steppic-continental regions, found in fissures of calciferous rocks; when young it often overgrows cyanobacterial colonies and cyanobacterial lichens; widespread throughout the Alps. – **Au**: V, T, S, K, St, O, N. **Ge**: OB. **Sw**: BE, GR, LU, SZ, TI, VS. **Fr**: AHP, AMa, Isè. **It**: Frl, Ven, TAA, Lomb, Piem, VA. **Sl**: SlA.

***Toninia aromatica* (Sm.) A. Massal.**

Syn.: *Bacidia sardoa* (Körb.) Zahlbr., *Bilimbia acervulata* (Nyl.) Jatta, *Bilimbia aromatica* (Sm.) Jatta, *Bilimbia sanguinaria* (Bagl.) Jatta, *Bilimbia sardoa* Körb., *Lecidea acervulata* Nyl., *Lecidea aromatica* (Sm.) Turner, *Lecidea austerula* Nyl., *Lecidea fusispora* (Hepp *ex* Körb.) Stizenb., *Lecidea geoleuca* Nyl., *Lecidea heterophora* Nyl., *Lecidea hypsophila* Nyl., *Lecidea subaromatica* Nyl., *Lecidea turneri* Leight., *Lichen aromaticus* Sm., *Raphiospora fusispora* Hepp *ex* Körb., *Thalloidima fusisporum* (Hepp *ex* Körb.) Müll. Arg., *Toninia acervulata* (Nyl.) Kremp., *Toninia affinis* Vězda, *Toninia fusispora* (Hepp *ex* Körb.) Th. Fr., *Toninia geoleuca* (Nyl.) Zahlbr., *Toninia heterophora* (Nyl.) Arnold, *Toninia hypsophila* (Nyl.) Zahlbr., *Toninia meridionalis* B. de Lesd., *Toninia pelophila* Poelt & Vězda, *Toninia sanguinaria* Bagl., *Toninia sinensis* Zahlbr., *Toninia squamulosa* Deakin, *Toninia turneri* (Leight.) H. Olivier

L – Subs.: cal, int, ter-cal – Alt.: 1–5 – Note: a holarctic species with a wide latitudinal range, found on horizontal to weakly inclined surfaces of calcareous to basic siliceous substrata, including bricks and roofing tiles in urban areas, often starting the life-cycle on other crustose lichens; the species has a wide altitudinal range, but seems to be most common at relatively low elevations; widespread throughout the Alps. – **Au**: T, S, K, St, O, N. **Ge**: OB. **Sw**: BE, GR, LU, SZ, TI, UW, VD, VS. **Fr**: AHP, HAl, AMa, Isè, Sav, HSav, Var, Vau. **It**: Frl, Ven, TAA, Lomb, Piem.

***Toninia athallina* (Hepp) Timdal**

Syn.: *Biatora athallina* Hepp, *Catillaria acrustacea* Arnold, *Catillaria athallina* (Hepp) Hellb., *Catillaria dvorakii* Servít, *Catinaria acrustacea* (Arnold) Vain., *Catinaria athallina* (Hepp) Lynge, *Kiliasia athallina* (Hepp) Hafellner

L – Subs.: cal – Alt.: 1–6 – Note: a temperate to arctic species of calcareous rocks, mostly on steeply inclined or underhanging faces in open, dry situations, sometimes invading the thalli of endolithic lichens, with a wide altitudinal range; widespread throughout the Alps. – **Au**: V, T, S, K, St, O, N. **Ge**: OB. **Sw**: BE, GR, LU, SZ, VS. **Fr**: AHP, HAl, AMa, Drô, Isè, Sav, HSav, Var, Vau. **It**: TAA, Lomb, Lig. **Sl**: SlA.

***Toninia candida* (Weber) Th. Fr.**

Syn.: *Biatorina candida* (Weber) Jatta, *Lecidea candida* (Weber) Ach., *Lichen candidus* Weber, *Psora candida* (Weber) Hoffm., *Thalloidima candidum* (Weber) A. Massal.

L – Subs.: cal, cal-par, ter-cal – Alt.: 1–5 – Note: a mainly southern, incompletely holarctic species found on steeply inclined surfaces and in fissures of calciferous rocks, chiefly limestone and dolomite, often on cyanobacteria or cyanobacterial lichens when young, with a wide altitudinal range; widespread throughout the Alps. – **Au**: V, T, S, K, St, O, N, B. **Ge**: OB. **Sw**: BE, GR, LU, SZ, TI, VD, VS. **Fr**: AHP, HAl, AMa, Drô, Isè, Sav, HSav, Var, Vau. **It**: Frl, Ven, TAA, Lomb, Piem, VA. **Sl**: SlA, Tg.

***Toninia cinereovirens* (Schaer.) A. Massal.**

Syn.: *Bilimbia cinereovirens* (Schaer.) Jatta, *Bilimbia fallasca* (A. Massal.) Jatta, *Bilimbia nigrescens* (Anzi) Jatta, *Lecidea cinereovirens* Schaer., *Toninia fallasca* A. Massal., *Toninia nigrescens* Anzi, *Toninia olivaceoatra* H. Magn., *Toninia potieri* Maheu & Werner, *Toninia sbarbaronis* B. de Lesd.

L – Subs.: sil, cal, ter-cal – Alt.: 1–4 – Note: a mainly southern, perhaps incompletely holarctic species found on steeply inclined, somehow weathered faces of calciferous and basic siliceous rocks with some seepage of water after rain, often in rock fissures and on colonies of cyanobacteria; widespread throughout the Alps, but only locally common. – **Au**: T, S, K, St, N. **Sw**: BE, GR, VD, VS. **Fr**: AHP, HAl, AMa, Sav, Var, Vau. **It**: Ven, TAA, Lomb, Piem, VA.

***Toninia coelestina* (Anzi) Vězda**

Syn.: Bacidia atrosanguinea (Hepp) Anzi subsp. oribata (Nyl.) A.L. Sm., *Bacidia coelestina* Anzi, *Lecidea oribata* Nyl., Lecidea subincompta Nyl. subsp. oribata (Nyl.) Cromb., *Toninia aggregata* Vězda, *Toninia oribata* (Nyl.) P. James

L – Subs.: ter-sil, int – Alt.: 3–4 – Note: a rare species found on cyanobacterial lichens or cyanobacterial colonies developing on weathered calciferous schists in upland areas; from the Alps there are only a few scattered records. – **Au**: K, St. **Sw**: VD. **Fr**: AHP. **It**: Lomb.

***Toninia diffracta* (A. Massal.) Zahlbr.**

Syn.: *Biatorina diffracta* (A. Massal.) Jatta, *Thalloidima diffractum* (A. Massal.) A. Massal., Thalloidima vesiculare (Hoffm.) A. Massal. var. diffractum A. Massal., Toninia candida (Weber) Th. Fr. subsp. diffracta (A. Massal.) Hild. Baumgärtner

L – Subs.: ter-cal, cal – Alt.: 1–5 – Note: a mainly southern eurasiatic species found in small fissures of steeply inclined faces of calcareous rocks, often on cyanobacteria or cyanobacterial lichens when young, sometimes on soil, with optimum at low altitudes, but exceptionally reaching the alpine belt; widespread throughout the Alps. – **Au**: V, T, S, K, St, O, N. **Ge**: OB. **Sw**: BE, LU, VD, VS. **Fr**: AHP, HAl, AMa, Drô, Isè, Sav, HSav, Var, Vau. **It**: Frl, Ven, TAA, Piem, VA, Lig.

***Toninia lutosa* (Ach.) Timdal**

Syn.: *Biatorina verrucosa* (A. Massal.) Jatta, *Lecidea lutosa* Ach., *Thalloidima verrucosum* A. Massal., *Toninia verrucosa* (A. Massal.) Flagey, *Toninia violacea* B. de Lesd.

L – Subs.: ter-cal, cal – Alt.: 1–3 – Note: a probably incompletely holarctic species of continental areas found on soil and weathered calciferous rocks, often in association with cyanobacteria or cyanobacterial lichens when young, mostly at relatively low elevations, with a few records from the Southern and Western Alps (France, Italy). – **Fr**: AHP. **It**: Ven, TAA, Lomb.

***Toninia nordlandica* Th. Fr.**

Syn.: *Lecidea subrimulosa* Nyl., *Toninia steineri* Poelt & Vězda, *Toninia subrimulosa* (Nyl.) Zahlbr.

L – Subs.: cal, cal-par, sil, sil-par – Alt.: 3–5 – Note: an arctic-alpine species found on steeply inclined to slightly underhanging seepage tracks of calciferous or basic siliceous rocks, almost always on cyanobacterial colonies, or on thalli of *Placynthium*, at least when young, mostly in upland areas. – **Au**: T, K. **Sw**: GR, SG, VD. **It**: TAA, Lomb, Piem, VA.

***Toninia opuntioides* (Vill.) Timdal**

Syn.: *Lichen opuntioides* Vill., ?*Thalloidima bormuelleri* Stein, ?*Toninia bornmuelleri* (Stein) Zahlbr.

L – Subs.: ter-cal, cal – Alt.: 2–5 – Note: an arctic to temperate, circumpolar lichen found often amongst bryophytes, always associated to cyanobacterial colonies or cyanobacterial lichens when young; widespread throughout the Alps. – **Au**: V, T, K, St, N. **Ge**: Ge. **Sw**: GR, SG, TI, VD, VS. **Fr**: AHP, HAl, AMa, Drô, Isè, HSav, Var, Vau. **It**: Frl, Ven, TAA, Lomb, Piem, VA.

***Toninia pennina* (Schaer.) Gyeln.**

Syn.: *Biatora pennina* (Schaer.) Hepp, *Catillaria scotina* (Körb.) Hertel & H. Kilias, *Lecidea aeneiformis* (Anzi) Jatta, *Lecidea pennina* Schaer., *Lecidea scotina* (Körb.) Arnold, *Lecidella scotina* Körb., *Psora aeneiformis* Anzi

L – Subs.: cal – Alt.: 2–4 – Note: a rarely collected lichen of continental-dry areas found on steeply inclined seepage tracks of dolomite, rarely of compact limestone, almost always growing on cyanobacterial colonies when young. – **Au**: T, N. **Sw**: TI, VS. **Fr**: HSav. **It**: Ven, TAA, Lomb.

***Toninia philippea* (Mont.) Timdal**

Syn.: *Catillaria arctica* Lynge, *Catillaria areolata* H. Magn., *Catillaria cirtensis* (Stizenb.) Flagey, *Catillaria holtedahlii* Lynge, *Catillaria ligustica* B. de Lesd., *Catillaria lutosa* A. Massal., *Catillaria philippea* (Mont.) A. Massal., *Catillaria riparia* (Müll. Arg.) Zahlbr., *Catillaria subgrisea* (Nyl.) Flagey, *Kiliasia philippea* (Mont.) Hafellner, *Kiliasia riparia* (Müll. Arg.) Hafellner, *Lecidea capitata* Anzi, *Lecidea cirtensis* Stizenb., *Lecidea lutosa* Mont. *ex* Schaer. *nom.illeg.*, *Lecidea philippea* Mont., *Lecidea subgrisea* Nyl., *Patellaria riparia* Müll. Arg.

L – Subs.: cal, int – Alt.: 1–5 – Note: an incompletely holarctic lichen of dry areas found on limestone, dolomite, calciferous sandstone and schists in open situations, most common in dry grasslands, with a wide altitudinal range. – **Au**: ?V, T, S, K, St, N. **Ge**: OB. **Sw**: GR, LU, SZ, VD. **Fr**: AHP, AMa, Sav, HSav, Vau. **It**: Ven, TAA, Lomb, Lig.

***Toninia physaroides* (Opiz) Zahlbr.**

Syn.: *Bacillina antipolitana* Nyl., *Biatorina lurida* (Bagl. *ex* Arnold) Jatta, *Lecidea physaroides* Opiz, *Thalloidima luridum* Bagl. *ex* Arnold, *Toninia alluviicola* M. Choisy, *Toninia lurida* (Bagl. *ex* Arnold) H. Olivier

L – Subs.: ter-cal, cal, int – Alt.: 1–5 – Note: a mainly temperate species, most common on soil developing from calciferous sandstone, often amongst mosses and associated to cyanobacterial lichens when young, rare in limestone areas. – **Au**: V, K, N. **Sw**: BE, GR, VD, VS. **Fr**: AHP, HAl, AMa, Isè, Sav, Vau. **It**: Ven, Lig.

***Toninia rosulata* (Anzi) H. Olivier**

Syn.: *Biatorina rosulata* (Anzi) Jatta, *Thalloidima rosulatum* Anzi, *Toninia melanocarpizans* Zahlbr.

L – Subs.: ter-cal, cal, bry – Alt.: 3–5 – Note: an arctic-alpine, mainly European species found on soil and in fissures and crevices of calciferous rocks, often on cyanobacteria or cyanobacterial lichens when young, with optimum above treeline; widespread throughout the Alps. – **Au**: V, T, S, K, St, O, N. **Ge**: OB, Schw. **Sw**: BE, FR, GR, SZ, TI, VD, VS. **Fr**: AHP, AMa, Isè, Sav, HSav. **It**: Frl, Ven, TAA, Lomb, Piem, VA. **Sl**: SlA.

***Toninia sedifolia* (Scop.) Timdal**

Syn.: *Biatorina vesicularis* (Hoffm.) Jatta, *Lecidea glebosa* Ach., *Lecidea subtabacina* Nyl., *Lecidea vesicularis* (Hoffm.) Ach., *Lichen sedifolius* Scop., *Patellaria vesicularis* Hoffm., *Psora paradoxa* (Ehrh.) Hoffm., *Psora vesicularis* (Hoffm.) Hoffm., Thalloidima caeruleonigricans *auct. non* (Lightf.) Poetsch, *Thalloidima vesiculosum* M. Choisy, Toninia caeruleonigricans *auct. non* (Lightf.) Th. Fr., *Toninia carolitana* (Arnold) Nimis & Poelt, *Toninia muricola* B. de Lesd., *Toninia subtabacina* (Nyl.) H. Olivier, *Toninia vesicularis* (Hoffm.) Boistel, *Verrucaria grisea* Willd.

L – Subs.: ter-cal, cal – Alt.: 1–6 – Note: a widespread holarctic lichen with a broad altitudinal and latitudinal range found on soil and weathered calciferous, more rarely basic siliceous rocks, often overgrowing mosses and associated with cyanobacteria or cyanobacterial lichens when young; common in dry, open grasslands throughout the Alps. – **Au**: V, T, S, K, St, O, N, B. **Ge**: OB. **Sw**: BE, FR, GR, LU, SZ, TI, UR, UW, VD, VS. **Fr**: AHP, HAl, AMa, Drô, Isè, Sav, HSav, Var, Vau. **It**: Frl, Ven, TAA, Lomb, Piem, VA, Lig. **Sl**: SlA, Tg.

***Toninia squalescens* (Nyl.) Th. Fr.**

Syn.: *Lecidea squalescens* Nyl., *Thalloidima rimulosum* Th. Fr.

L # – Subs.: bry-sil – Alt.: 4–5 – Note: on silicicolous mosses, mostly on *Andreaea* near or above treeline; on the whole, a rather poorly known species which certainly does not belong to *Toninia*. – **Au**: V, T, S. **Sw**: VS. **It**: Lomb.

***Toninia squalida* (Ach.) A. Massal.**

Syn.: *Bacidia acervulans* (Nyl.) B. de Lesd., *Bilimbia caulescens* (Anzi) Jatta, *Bilimbia multiseptata* (Anzi) Jatta, *Bilimbia squalida* (Ach.) Jatta, *Lecidea acervulan*s Nyl., *Lecidea caulescens* (Anzi) Tuck., *Lecidea norvegica* Sommerf., *Lecidea squalida* Ach., *Toninia acervulans* (Nyl.) H. Olivier, *Toninia catalaunica* V. Wirth & Llimona, *Toninia caulescens* Anzi, Toninia cinereovirens (Schaer.) A. Massal. var. verruculosa Th. Fr., *Toninia havaasii* H. Magn., *Toninia multiseptata* Anzi, *Toninia squarrosa* (Ach.) Th. Fr., *Toninia verruculosa* (Th. Fr.) Vain.

L – Subs.: sil, int, cal, bry-sil, bry-cal, ter-sil, ter-cal – Alt.: 3–5 – Note: an incompletely holarctic lichen with a very broad latitudinal range, found on soil, more rarely on weathered base-rich or weakly calciferous siliceous rocks in dry-warm upland areas, often associated to cyanobacteria or cyanobacterial lichen when young. – **Au**: T, S, K, St. **Sw**: BE, GR, SZ, UR, VS. **Fr**: AHP, HAl, Isè, HSav, Var. **It**: Frl, Ven, TAA, Lomb, Piem, VA.

***Toninia subnitida* (Hellb.) Hafellner & Türk**

Syn.: *Catillaria subnitida* Hellb., *Catillaria tristis* (Müll. Arg.) Arnold, *Kiliasia tristis* (Müll. Arg.) Hafellner *non Toninia tristis* (Th. Fr.) Th. Fr., *Lecidea platycarpiza* Nyl., *Patellaria tristis* Müll. Arg.

L – Subs.: cal – Alt.: 3–5 – Note: on calcareous substrata; the generic position is still an open problem. – **Au**: V, T, St, N. **Ge**: OB, Schw. **Sw**: GR, SZ, UR, UW. **Fr**: HAl, Sav. **It**: TAA, Piem.

***Toninia taurica* (Szatala) Oxner**

Syn.: *Thalloidima tauricum* Szatala, *Toninia clemens* H. Baumgärtner, *Toninia schafeevii* Tomin

L – Subs.: cal, ter-cal – Alt.: 1–5 – Note: a mainly southern species with an Eurasiatic distribution found on calciferous soil and in fine crevices of the rocks, often associated with cyanobacterial lichens when young, with a wide altitudinal range. – **Au**: V, T, K, St, O, N. **Sw**: BE, GR, VD, VS. **Fr**: AHP, HAl, AMa, Isè, Sav, Var, Vau. **It**: Frl, Ven, TAA, Lomb, Piem, Lig.

***Toninia toniniana* (A. Massal.) Zahlbr.**

Syn.: *Biatorina toniniana* (A. Massal.) Jatta, *Lecidea caesiocandida* Nyl., *Thalloidima caesiocandidum* (Nyl.) Arnold, Thalloidima mammillare (Gouan) A. Massal. var. toninianum A. Massal., *Thalloidima toninianum* (A. Massal.) A. Massal., *Toninia caesiocandida* (Nyl.) Th. Fr.

L – Subs.: cal, ter-cal – Alt.: 2–4 – Note: a mainly Mediterranean to submediterranean species found on steeply inclined to slightly underhanging seepage tracks of calcareous rocks, always in association with cyanobacterial colonies. – **Au**: T, S, K, St, O, N. **Sw**: GR, VS. **Fr**: AHP, AMa, HSav, Vau. **It**: Frl, Ven, TAA, Lomb, Piem, VA.

**Toninia tristis (Th. Fr.) Th. Fr. subsp. tristis**

Syn.: Psora tabacina DC. var. tristis Th. Fr., Toninia tabacina *auct. non* (DC.) Flagey

L – Subs.: cal, ter-cal – Alt.: 1–5 – Note: in fine crevices of calciferous rocks, with optimum near and above treeline. Here we have placed also all records of *T. tristis* without specification of the subspecies. – **Au**: V, T, S, St, N. **Sw**: GR, TI, VS. **Fr**: HAl, Vau. **It**: Lomb, VA. **Li**.

**Toninia tristis (Th. Fr.) Th. Fr. subsp. asiae-centralis (H. Magn.) Timdal**

Syn.: *Lecidea asiae-centralis* H. Magn.

L – Subs.: cal, ter-cal – Alt.: 2–4 – Note: on calciferous rocks and soil; despite the name, this subspecies is widespread also in Southern and Central Europe, with scattered outposts north to Greenland. – **Au**: T. **Sw**: GR, VS. **Fr**: AHP, HAl, AMa, Sav, Var, Vau. **It**: Frl, TAA, Lomb, Piem.

**Toninia tristis (Th. Fr.) Th. Fr. subsp. pseudotabacina Timdal**

L – Subs.: ter-cal – Alt.: 2–5 – Note: a mainly Mediterranean-Macaronesian taxon found on soil over calcareous substrata. – **Au**: T. **Sw**: TI, UR. **Fr**: AMa, Vau. **It**: Ven, TAA, Lomb, Lig.

**Toninia tristis (Th. Fr.) Th. Fr. subsp. scholanderi (Lynge) Timdal**

Syn.: *Lecidea scholanderi* Lynge

L – Subs.: ter-cal – Alt.: 4 – Note: a variety with squamules as in subsp. tristis, but with a brown epithecium, a hypothecium lacking orange pigments, and simple ascospores; on soil in seasonally dry open situations; widespread in the Northern Hemisphere, with a single record from the Eastern Alps (Austria). – **Au**: T.

**Toninia tristis (Th. Fr.) Th. Fr. subsp. thalloedaemiformis (Szatala) Timdal**

Syn.: *Lecidea thalloedaemiformis* Szatala

L – Subs.: ter-cal – Alt.: 2 – Note: a variety with contiguous squamules which are larger than in subsp. tristis, a bright green epithecium, and simple ascospores; more or less confined to steep faces of calciferous rocks; widespread in the eastern Mediterranean region, the terricolous record from the Western Alps (France) needs confirmation. – **Fr**: AMa.

***Toninia verrucarioides* (Nyl.) Timdal**

Syn.: *Bilimbia carbonacea* (Anzi) Jatta, Lecidea aromatica var. verrucarioides Nyl., *Lecidea subimbricata* Nyl., *Lecidea verrucarioides* (Nyl.) Nyl., *Thalloidima boissieri* Müll. Arg., *Thalloidima carbonacea* Anzi, Toninia aromatica (Sm.) A. Massal. var. cervina (Lönnr.) Th. Fr., *Toninia boissieri* (Müll. Arg.) Arnold, *Toninia carbonacea* Anzi, *Toninia cervina* Lönnr., *Toninia conjungens* Th. Fr., *Toninia kolax* Poelt, *Toninia subimbricata* (Nyl.) H. Olivier

L – Subs.: cal-par, cal – Alt.: 3–5 – Note: an arctic-alpine to cool-temperate lichen found in fissures and fine crevices of calcareous rocks in upland areas, often growing on species of *Placynthium* when young. – **Au**: V, T, K, St, O, N. **Ge**: Ge. **Sw**: LU, TI. **Fr**: Drô, HSav, Vau. **It**: Lomb, Lig.

***Toniniopsis obscura* Frey**

L – Subs.: ter-cal – Alt.: 4–5 – Note: a peculiar, inconspicuous lichen somewhat recalling a *Placynthiella*-species, with a blackish-brown, minutely granulose thallus, small, brownish-black apothecia, and bacilliform, finally 3-septate ascospores (to *c.* 25 µm long); on plant debris and decaying bryophytes over dolomite and calcareous rocks, from the subalpine to the lower alpine belt; known from a few localities in the Alps, but easily overlooked, and perhaps more widespread. – **Au**: T, S, K, O. **Sw**: GR.

***Topelia heterospora* (Zahlbr.) P.M. Jørg. & Vězda**

Syn.: *Clathroporina heterospora* Zahlbr.

L – Subs.: cal – Alt.: 2 – Note: a humid subtropical to Mediterranean-Atlantic lichen found on hard, compact calcareous rocks in sheltered situations, also reported from the base of the SW Pre-Alps (France). – **Fr**: AMa.

***Trapelia coarctata* (Sm.) M. Choisy**

Syn.: *Biatora arridens* (Nyl.) Walt. Watson, *Biatora coarctata* (Sm.) Th. Fr., Biatora coarctata (Sm.) Th. Fr. var. elachista (Ach.) Th. Fr., ?*Biatora comensis* Anzi, *Lecanactis arridens* Nyl., *Lecanora coarctata* (Sm.) Ach., Lecanora coarctata (Sm.) Ach. var. elachista (Ach.) Schaer., *Lecidea arridens* Nyl., *Lecidea coarctata* (Sm.) Nyl., Lecidea coarctata (Sm.) Nyl. var. elachista (Ach.) Th. Fr., *Lichen coarctatus* Sm., *Zeora coarctata* (Sm.) Flot.

L – Subs.: sil, sil-aqu – Alt.: 1–5 – Note: a holarctic early coloniser of siliceous pebbles near the soil surface, sometimes also found on bare clayey soil, with a wide altitudinal and altitudinal range; it is most frequent in upland areas, becoming rare in the eu-Mediterranean belt. The species is genetically heterogeneous; widespread throughout the Alps. – **Au**: V, T, S, K, St, O, N, B. **Ge**: OB, Schw. **Sw**: BE, GR, LU, SZ, TI, UR, VD, VS. **Fr**: HAl, AMa, Isè, Sav, HSav, Var, Vau. **It**: Frl, Ven, TAA, Lomb, Piem, VA. **Sl**: SlA, Tg.

***Trapelia corticola* Coppins & P. James**

L – Subs.: cor, xyl – Alt.: 2–3 – Note: on the spongy, loose bark of deciduous trees, sometimes on moribund epiphytic bryophytes in sheltered, humid woodlands at low elevations; from the Alps there are only a few scattered records. – **Sw**: GL, SZ, UW. **It**: Lomb.

***Trapelia glebulosa* (Sm.) J.R. Laundon**

Syn.: Biatora coarctata (Sm.) Th. Fr. var. glebulosa (Sm.) Arnold, Biatora coarctata (Sm.) Th. Fr. var. ornata (Sommerf.) Th. Fr., Lecanora coarctata (Sm.) Ach. var. involuta (Taylor) Mudd., Lecanora coarctata (Sm.) Ach. var. ornata Sommerf., *Lecanora involuta* Taylor, Lecidea coarctata (Sm.) Nyl. var. glebulosa (Sm.) Mudd, Lecidea coarctata (Sm.) Nyl. var. ornata (Sommerf.) Malbr., *Lecidea glebulosa* (Sm.) Jatta, *Lecidea ornata* (Sommerf.) Hue, *Lichen glebulosus* Sm., *Trapelia involuta* (Taylor) Hertel, *Trapelia ornata* (Sommerf.) Hertel

L – Subs.: sil, xyl – Alt.: 1–5 – Note: a species with a minutely squamulose thallus, not easy to distinguish from some forms of *T. coarctata*; on basic siliceous rocks and various types of schists, roofing tiles, brick walls, mostly close to the ground, often together with *T. coarctata*; widespread in the Holarctic region, but not in the extreme north; widespread throughout the Alps. – **Au**: V, T, S, K, St, O, N, B. **Ge**: OB, Schw. **Sw**: LU, SZ, TI. **Fr**: AMa, HSav, Var, Vau. **It**: Frl, Ven, Lomb, Piem. **Sl**: SlA.

***Trapelia obtegens* (Th. Fr.) Hertel**

Syn.: Biatora coarctata (Sm.) Th. Fr. subsp. obtegens Th. Fr., Biatora coarctata (Sm.) Th. Fr. var. obtegens (Th. Fr.) Th. Fr., Lecidea coarctata (Sm.) Nyl. var. obtegens (Th. Fr.) Th. Fr., *Lecidea obtegens* (Th. Fr.) Vain.

L – Subs.: sil – Alt.: 2–4 – Note: on siliceous pebbles near the ground, sometimes on roofing tiles and over thin soil layers, with scattered records from the Eastern Alps only. – **Au**: S, K, St, N. **It**: Frl. **Sl**: SlA.

***Trapelia placodioides* Coppins & P. James**

L – Subs.: sil – Alt.: 1–5 – Note: on base-rich or slightly calciferous siliceous substrata, sometimes also on walls, in humid areas, with optimum below the montane belt; probably more widespread in the Alps, but never common. – **Au**: V, T, S, St, O, N, B. **Sw**: GR, LU, SZ, VS. **It**: TAA, Piem. **Sl**: SlA.

***Trapeliopsis aeneofusca* (Flörke *ex* Flot.) Coppins & P. James**

Syn.: *Biatora aeneofusca* (Flörke *ex* Flot.) Arnold, *Lecidea aeneofusca* Flörke *ex* Flot., *Lecidea prasinorufa* Nyl.

L – Subs.: ter-sil, bry – Alt.: 3–4 – Note: very similar to *T. gelatinosa*, but apothecia in various shades of brown (instead of blackish green), sterile specimens therefore indistinguishable; on soil, rare throughout Europe and North America, with a few scattered records from the Alps. – **Au**: T, O, N. **Sw**: LU. **Fr**: HAl.

***Trapeliopsis flexuosa* (Fr.) Coppins & P. James**

Syn.: *Biatora flexuosa* Fr., *Lecidea aeruginosa* Borrer, *Lecidea flexuosa* (Fr.) Nyl., Lecidea granulosa (Hoffm.) Ach. subsp. flexuosa (Fr.) Th. Fr., *Lecidea sapinea* (Fr.) Zahlbr. *non* *sensu* Vain., *Lecidea sporodiza* Stirt.

L – Subs.: xyl, cor, ter-sil – Alt.: 2–5 – Note: a temperate to boreal-montane, circumpolar lichen found on lignum (often on wooden fences) and acid bark, especially of *Pinus* and *Castanea*; widespread and often common throughout the Alps. – **Au**: V, T, S, K, St, O, N, B. **Ge**: OB. **Sw**: AP, BE, FR, GR, LU, SG, SZ, TI, UR, UW, VD, VS. **Fr**: AHP, AMa, Sav, HSav, Var, Vau. **It**: Frl, Ven, TAA, Lomb, Piem, Lig. **Sl**: SlA, Tg. **Li**.

***Trapeliopsis gelatinosa* (Flörke) Coppins & P. James**

Syn.: *Biatora gelatinosa* (Flörke) Flot., Biatora viridescens (Schrad.) W. Mann var. gelatinosa (Flörke) Fr., *Lecidea gelatinosa* Flörke, *Micarea gelatinosa* (Flörke) Brodo

L – Subs.: ter-sil, bry – Alt.: 2–5 – Note: a boreal-montane to cool-temperate early coloniser of mineral acid soil, sometimes overgrowing bryophytes and plant debris, mostly in upland areas; widespread throughout the Alps. – **Au**: V, T, S, K, St, O, N, B. **Ge**: OB, Schw. **Sw**: BE, GR, LU, SZ, TI, UR, VD, VS. **Fr**: HAl, HSav. **It**: Frl, TAA, Lomb, Piem, Lig. **Sl**: SlA.

***Trapeliopsis glaucolepidea* (Nyl.) Gotth. Schneid.**

Syn.: *Lecidea glaucolepidea* Nyl., *Lecidea percrenata* Nyl., *Trapelia percrenata* (Nyl.) V. Wirth, *Trapeliopsis percrenata* (Nyl.) Gotth. Schneid.

L – Subs.: deb, ter-sil, xyl – Alt.: 3–4 – Note: a species with a thallus resembling the primary thallus of a *Cladonia*, the grey to greenish squamules with usually lip-shaped soralia, mostly sterile; on plant debris and rotting wood; widespread worldwide, in Central Europe in montane forests, in the Alps still overlooked in some regions. – **Au**: T, S, K, St, N. **Sw**: LU.

***Trapeliopsis granulosa* (Hoffm.) Lumbsch**

Syn.: *Biatora decolorans* *auct.*, *Biatora granulosa* (Hoffm.) Flot., *Lecidea decolorans* *auct.*, *Lecidea granulosa* (Hoffm.) Ach., *Lecidea quadricolor* (Dicks.) Borrer, *Trapelia granulosa* (Hoffm.) V. Wirth, *Verrucaria granulosa* Hoffm.

L – Subs.: xyl, deb, bry, ter-sil, bry-sil – Alt.: 2–5 – Note: an arctic-alpine to cool-temperate, circumpolar lichen mostly found on rotting wood, more rarely on soil rich in humus, bryophytes and peat, mostly in clearings of grasslands and shrublands, with optimum near treeline; widespread throughout the Alps. – **Au**: V, T, S, K, St, O, N, B. **Ge**: OB, Schw. **Sw**: BE, GR, LU, SZ, TI, UR, VD, VS. **Fr**: AHP, HAl, AMa, Isè, Sav, HSav, Var, Vau. **It**: Frl, Ven, TAA, Lomb, Piem, VA, Lig. **Sl**: SlA, Tg.

***Trapeliopsis pseudogranulosa* Coppins & P. James**

L – Subs.: bry, deb, ter-sil – Alt.: 2–4 – Note: this lichens is most frequent in humid *Castanea* woodlands, on mosses on basal parts of trunks, decaying lignum and acid organic soil, especially in areas with siliceous substrata; certainly more widespread in the Alps. – **Au**: T, S, K, St, O, N. **Ge**: OB. **Sw**: BE, GL, SG, SZ, TI, VD, VS. **Fr**: HAl, AMa. **Sl**: Tg.

***Trapeliopsis viridescens* (Schrad.) Coppins & P. James**

Syn.: *Biatora viridescens* (Schrad.) W. Mann, *Lecidea viridescens* (Schrad.) Ach., *Lichen viridescens* Schrad., *Micarea viridescens* (Schrad.) Brodo, *Trapelia viridescens* (Schrad.) V. Wirth

L – Subs.: bry, deb, ter-sil, xyl – Alt.: 2–4 – Note: a mainly boreal-montane lichen found on rotting, soft lignum, sometimes overgrowing mosses, mostly in coniferous forests or in *Castanea*-stands. – **Au**: V, S, K, St, O, N, B. **Ge**: OB. **Sw**: BE, VD. **It**: TAA, Lomb, Piem. **Sl**: SlA.

***Trapeliopsis wallrothii* (Flörke *ex* Spreng.) Hertel & Gotth. Schneid.**

Syn.: *Biatora glebulosa* Fr., *Biatora wallrothii* (Flörke *ex* Spreng.) Körb., *Lecidea wallrothii* Flörke *ex* Spreng., *Trapelia wallrothii* (Flörke *ex* Spreng.) V. Wirth

L – Subs.: ter-sil, bry, bry-sil – Alt.: 1–3 – Note: on base-rich, non – or weakly calciferous soil, sometimes overgrowing mosses, mostly in open situations, with optimum below the montane belt, with several scattered records from the Alps. – **Au**: T, S. **Sw**: LU, UW, VD. **Fr**: HSav, Var. **It**: Lomb, Piem.

***Tremolecia atrata* (Ach.) Hertel**

Syn.: *Aspicilia melanophaea* (Fr.) Körb., *Gyalecta atrata* Ach., *Lecidea atrata* (Ach.) Wahlenb., *Lecidea atroferrata* Branth & Grønlund, *Lecidea circumcisa* H. Magn., Lecidea dicksonii *auct. non* (J.F. Gmel.) Ach., *Lecidea melanophaea* Fr., *Lecidea sincerula* Nyl. *ex* Cromb.

L – Subs.: met, sil – Alt.: 3–6 – Note: a species of cool to cold areas of both Hemispheres, found on hard magmatic and metamorphic rocks often rich in iron, mostly on small boulders in upland areas; widespread and locally common in the Alps. – **Au**: V, T, S, K, St, N. **Ge**: Schw. **Sw**: BE, GR, TI, UR, VD, VS. **Fr**: AHP, HAl, AMa, Isè, Sav, HSav. **It**: Frl, Ven, TAA, Lomb, Piem, VA, Lig.

***Trimmatothele perquisita* (Norman) Norman *ex* Zahlbr.**

Syn.: *Coniothele perquisita* Norman, *Verrucaria perquisita* (Norman) Ertz & Diederich

L – Subs.: cal – Alt.: 3–4 – Note: a calcicolous species with an endolithic to thin, variably coloured thallus, hemispherically protruding ascomata with involucrellum surrounding the perithecium, moderately polyspored asci, and oblong to ellipsoid, simple ascospores (to *c.* 10 µm long). The genus is not generally accepted, and the species was treated in *Verrucaria* by some authors; widespread in Europe, from the boreal to the nemoral-subalpine zone; from the Alps there are a few scattered records only. – **Au**: T, St. **Fr**: Sav.

***Tuckermannopsis chlorophylla* (Willd.) Hale**

Syn.: *Cetraria chlorophylla* (Willd.) Vain., Cetraria scutata *auct. non* (Wulfen) Poetsch, *Cetraria ulophylla* (Ach.) Rebent., *Lichen chlorophyllus* Willd., *Nephromopsis chlorophylla* (Willd.) Divakar, A. Crespo & Lumbsch, *Platysma chlorophyllum* (Willd.) Vain., *Platysma ulophyllum* (Ach.) Nyl.

L – Subs.: cor – Alt.: 2–4 – Note: on isolated conifers (*e.g. Larix* in the subalpine belt), more rarely on old acid-barked deciduous trees in montane forests; widespread throughout the Alps. – **Au**: V, T, S, K, St, O, N, B. **Ge**: OB. **Sw**: BE, FR, GL, GR, LU, SZ, TI, UR, UW, VD, VS. **Fr**: AHP, HAl, AMa, Isè, Sav, HSav, Vau. **It**: Frl, Ven, TAA, Lomb, Piem, VA, Lig. **Sl**: SlA, Tg.

***Umbilicaria aprina* Nyl.**

Syn.: *Gyrophora aprina* (Nyl.) Nyl.

L – Subs.: sil – Alt.: 5–6 – Note: an arctic-alpine species of hard siliceous rocks above treeline; the only record from the Alps is from Mt. Blanc. – **Fr**: HSav.

***Umbilicaria cinerascens* (Arnold) Frey**

Syn.: *Gyrophora cinerascens* Arnold

L – Subs.: sil – Alt.: 4–6 – Note: on steeply inclined, often north-facing surfaces of siliceous rocks, mostly in small colonies, with optimum above treeline; several records from Switzerland need confirmation. – **Au**: V, T, S, K, St. **Sw**: BE, GL, GR, SG, UR, VS. **Fr**: AHP, HAl, AMa, Isè, Sav, HSav. **It**: TAA, Lomb, Piem, VA.

***Umbilicaria cinereorufescens* (Schaer.) Frey**

Syn.: *Gyrophora cinereorufescens* (Schaer.) Schol., *Gyrophora mammulata* Ach., Umbilicaria vellea (L.) Ach. f. cinereorufescens Schaer.

L – Subs.: sil – Alt.: 3–6 – Note: a holarctic species also known from the mountains of Africa found on wind-exposed, vertical or slightly underhanging surfaces of hard siliceous rocks in humid upland areas (frequent fog and high rainfall), but in apparently dry situations. – **Au**: V, T, S, K, St. **Sw**: BE, GR, UR, VD, VS. **Fr**: AHP, HAl, Sav, HSav. **It**: TAA, Lomb, Piem, VA.

***Umbilicaria corsica* Frey (not validly published, ICN Art. 36.1(b))**

Syn.: *Gyrophora corsica* (Frey) Schol. *nom. inval.*, *Omphalodiscus corsicus* (Frey) Llano *nom. inval*.

L – Subs.: sil – Alt.: 4 – Note: a silicicolous species resembling *U. virginis*, with small (to 3 cm in diam.), monophyllous thalli which are whitish grey above and pale and hirsute below, apothecia plane at first finally with plicate discs, ascospores small, non-septate, hyaline; only known from Corsica and the Western Alps (France). – **Fr**: HAl.

**Umbilicaria crustulosa (Ach.) Lamy var. crustulosa**

Syn.: *Gyrophora crustulosa* Ach., *Gyrophora depressa* (Ach.) Röhl., Gyrophora depressa (Ach.) Röhl. var. crustulosa (Ach.) Dalla Torre & Sarnth., *Omphalodiscus crustulosus* (Ach.) Schol., *Umbilicaria spadochroa* *auct.* medioeur. *p.p. non* Ehrh. *ex* Hoffm.

L – Subs.: sil – Alt.: 3–6 – Note: a cool-temperate to arctic-alpine, circumpolar lichen found on exposed, often steeply inclined surfaces of siliceous rocks with some water seepage in upland areas; widespread throughout the siliceous Alps. – **Au**: V, T, S, K, St. **Sw**: BE, GR, TI, UR, VD, VS. **Fr**: AHP, HAl, AMa, Isè, Sav, HSav, Var. **It**: Frl, Ven, TAA, Lomb, Piem, VA.

**Umbilicaria crustulosa (Ach.) Lamy var. badiofusca Frey**

Syn.: *Gyrophora hirsuta* (Sw. *ex* Westr.) Ach. var. meizospora (Harm.) H. Olivier, *Gyrophoropsis meizospora* (Harm.) M. Choisy, *Umbilicaria hirsuta* (Sw. *ex* Westr.) Hoffm. var. meizospora Harm.

L # – Subs.: sil – Alt.: 2–5 – Note: a taxon of the mountains of Southern Europe, worthy of further study. – **Fr**: Isè, Sav. **It**: Lomb, Piem, VA.

***Umbilicaria cylindrica* (L.) Delise *s.lat.***

Syn.: *Gyrophora cylindrica* (L.) Ach., Gyrophora cylindrica (L.) Ach. var. delisei (Nyl.) Syd., Gyrophora cylindrica (L.) Ach. var. nudiuscula (Schaer.) Zahlbr, Gyrophora polymorpha Schrad. var. cylindrica Schaer. f. nudiuscula Schaer., *Gyrophora tornata* Ach., *Lichen cylindricus* L., *Umbilicaria canescens* (Dombr.) Dombr., *Umbilicaria crinita* Hoffm., *Umbilicaria cylindrica* (L.) Delise, Umbilicaria cylindrica (L.) Delise var. corrugatoides Frey, Umbilicaria cylindrica (L.) Delise var. delisei Despr. *ex* Nyl. *nom.illeg.*, Umbilicaria cylindrica (L.) Delise var. fimbriata (Ach.) Nyl., Umbilicaria cylindrica (L.) Delise var. mesenteriformis (Wulfen) Ozenda & Clauzade, Umbilicaria cylindrica (L.) Delise var. nudiuscula (Schaer.) Ozenda & Clauzade, Umbilicaria cylindrica (L.) Delise var. tornata (Ach.) Nyl., *Umbilicaria delisei* Despr. *ex* Nyl. *nom.illeg*.

L – Subs.: sil, int – Alt.: 3–6 – Note: an ecologically wide-ranging, cool-temperate to arctic-alpine, circumpolar lichen found on wind-exposed boulders with a short snow-covering period, often on or near the top, with optimum above treeline. The species is highly polymorphic, with several infraspecific taxa; widespread throughout the siliceous Alps. – **Au**: V, T, S, K, St, O, N. **Ge**: OB, Schw. **Sw**: BE, GR, LU, SZ, TI, UR, UW, VD, VS. **Fr**: AHP, HAl, AMa, Isè, Sav, HSav. **It**: Frl, Ven, TAA, Lomb, Piem, VA, Lig. **Sl**: SlA.

***Umbilicaria decussata* (Vill.) Zahlbr.**

Syn.: *Gyrophora decussata* (Vill.) Zahbr., *Gyrophora discolor* Th. Fr., *Gyrophora ptychophora* (Nyl.) Nyl., *Lichen decussatus* Vill., *Omphalodiscus decussatus* (Vill.) Schol., *Umbilicaria ptychophora* Nyl., *Umbilicaria reticulata* (Schaer.) Carestia *ex* Bagl. & Carestia

L – Subs.: sil – Alt.: 3–6 – Note: an arctic-alpine to boreal-montane, circumpolar lichen found on steeply inclined to slightly underhanging surfaces of wind-exposed siliceous rocks; widespread throughout the siliceous Alps. – **Au**: V, T, S, K, St. **Sw**: BE, GR, UR, VS. **Fr**: AHP, HAl, AMa, Isè, Sav, HSav. **It**: Ven, TAA, Lomb, Piem, VA.

***Umbilicaria dendrophora* (Poelt) Hestmark**

Syn.: Umbilicaria vellea (L.) Ach. var. dendrophora Poelt

L – Subs.: sil – Alt.: 5 – Note: a species resembling *U. vellea*, but with different thalloconidia plus other anatomical differences (algal layer discontinuous and medulla sharply separated from lower cortex), lacking lichen substances; on steeply inclined faces of siliceous cliffs and large boulders; widespread in Europe but rather rare, from the subarctic zone to the alpine belt; in the study area only known from the Eastern Alps (Austria). – **Au**: K.

***Umbilicaria deusta* (L.) Baumg.**

Syn.: Gyrophora aenea Schaer. var. flocculosa (Wulfen) Schaer., *Gyrophora deusta* (L.) Ach., *Gyrophora flocculosa* (Wulfen) Turner & Borrer, Gyrophora polyphylla (L.) Funck var. deusta (L.) Rabenh., *Lichen deustus* L., *Umbilicaria flocculosa* (Wulfen) Hoffm.

L – Subs.: sil – Alt.: 2–5 – Note: an arctic-alpine to boreal-montane, circumpolar lichen found on rocks wetted by rain near the ground, in sites with a long snow cover; one of the most common *Umbilicaria* throughout the Alps. – **Au**: V, T, S, K, St, N. **Ge**: Schw. **Sw**: BE, GR, LU, SZ, TI, UR, VD, VS. **Fr**: AHP, HAl, AMa, Isè, Sav, HSav. **It**: Frl, Ven, TAA, Lomb, Piem, VA, Lig. **Sl**: SlA.

***Umbilicaria freyi* Codogno, Poelt & Puntillo**

Syn.: Umbilicaria grisea Hoffm. f. subpapyria Frey, *Umbilicaria hirsuta* (Sw. *ex* Westr.) Hoffm. var. pyrenaica Frey

L – Subs.: sil – Alt.: 3–4 – Note: on steeply inclined to underhanging surfaces of siliceous rocks, ecologically intermediate between *U. grisea* and *U. deusta*; perhaps more widespread in the Alps. – **Fr**: AMa. **It**: VA.

***Umbilicaria grisea* Hoffm.**

Syn.: *Gyrophora grisea* (Hoffm.) Turner & Borrer, *Gyrophora hirsuta* (Sw. *ex* Westr.) Ach. var. grisea (Hoffm.) Th. Fr., *Gyrophora murina* (Ach.) Ach., *Umbilicaria murina* (Ach.) DC.

L – Subs.: sil – Alt.: 2–4 – Note: on steeply inclined to underhanging surfaces of siliceous rocks only slightly wetted after rain, usually below the alpine belt; several records from the Alps require confirmation. – **Au**: ?T. **Sw**: ?GR, ?TI, ?VS. **Fr**: HAl, Var, Vau. **It**: TAA, Lomb, Piem, VA.

***Umbilicaria hirsuta* (Sw. *ex* Westr.) Hoffm.**

Syn.: *Gyrophora hirsuta* (Sw. *ex* Westr.) Ach., Gyrophora vellea (L.) Ach. var. hirsuta (Sw. *ex* Westr.) Rabenh., *Lichen hirsutus* Sw. *ex* Westr.

L – Subs.: sil – Alt.: 2–6 – Note: an arctic-alpine to boreal-montane, circumpolar lichen found on steeply inclined to slightly underhanging surfaces of siliceous rocks, often in somehow dusty situations; widespread and common throughout the Alps. – **Au**: V, T, S, K, St. **Sw**: BE, GR, TI, UR, VS. **Fr**: HAl, AMa, Isè, Sav, HSav. **It**: Frl, TAA, Lomb, Piem, VA, Lig. **Sl**: SlA.

***Umbilicaria hyperborea* (Ach.) Hoffm.**

Syn.: Gyrophora aenea Schaer. var. hyperborea (Ach.) Schaer., *Gyrophora hyperborea* (Ach.) Ach., *Gyrophora ustulata* (Vain.) Dalla Torre & Sarnth., *Lichen hyperboreus* Ach.

L – Subs.: sil – Alt.: 3–6 – Note: an arctic-alpine, probably circumpolar lichen found on siliceous boulders wetted by rain, usually near the ground, with optimum near treeline. – **Au**: V, T, S, K, St. **Sw**: BE, GR, UR, VS. **Fr**: Isè, HSav. **It**: Ven, TAA, Lomb, Piem, VA.

***Umbilicaria laevis* (Schaer.) Frey**

Syn.: *Agyrophora laevis* (Schaer.) Llano, Gyrophora atropruinosa (Schaer.) L. Mangin var. laevis Schaer., *Gyrophora laevis* (Schaer.) Du Rietz

L – Subs.: sil – Alt.: 3–6 – Note: on inclined, sun-exposed surfaces of siliceous rocks, generally in dry situations, with optimum above treeline. – **Au**: T, K, St. **Ge**: OB, Schw. **Sw**: BE, GR, UR, VD, VS. **Fr**: AHP, HAl, AMa. **It**: TAA, Lomb, Piem, VA.

***Umbilicaria leiocarpa* DC.**

Syn.: *Agyrophora leiocarpa* (DC.) Gyeln., *Agyrophora lyngei* (Schol.) Llano, *Gyrophora leiocarpa* (DC.) Du Rietz, *Umbilicaria atropruinosa* Schaer., *Umbilicaria lyngei* Schol.

L – Subs.: sil – Alt.: 4–6 – Note: a mainly arctic-alpine, probably circumpolar lichen found on vertical, wind – and sun-exposed surfaces of large siliceous boulders wetted by rain with a short snow cover period, with optimum above treeline. – **Au**: V, T, S, K, St. **Sw**: BE, GR, UR, VS. **Fr**: HAl, AMa, Isè, HSav. **It**: TAA, Lomb, Piem, VA.

***Umbilicaria maculata* Krzewicka, M.P. Martín & M.A. García**

L – Subs.: sil – Alt.: 4–5 – Note: a species of the *U. cylindrica*-group with monophyllous thalli tightly adhering to the rock, the grey to grey-brown upper surface with whitish maculae, a whitish to creamy rhizinate lower surface, and sessile, omphalodisc apothecia; on vertical rock faces of boulders and cliffs in shaded, windy situations at high elevations; rare in the Tatra Mountains, with a single record from the Western Alps (France), but perhaps not recognised elsewhere. – **Fr**: AHP.

***Umbilicaria microphylla* (Laurer) A. Massal.**

Syn.: *Agyrophora microphylla* (Laurer) Llano, *Gyrophora microphylla* (Laurer) Arnold, Umbilicaria atropruinosa Schaer. var. microphylla Laurer

L – Subs.: sil – Alt.: 4–6 – Note: on steeply inclined, wind-exposed surfaces of hard siliceous rocks, often forming monospecific stands, with optimum above treeline. – **Au**: ?V, T, S, K, St. **Sw**: BE, GR, VD, VS. **Fr**: AHP, HAl, AMa, HSav. **It**: TAA, Lomb, Piem, VA.

***Umbilicaria nylanderiana* (Zahlbr.) H. Magn.**

Syn.: *Gyrophora corrugata* (Ach.) Lamy *nom.illeg.*, *Gyrophora nylanderiana* Zahlbr., *Umbilicaria corrugata* (Ach.) Nyl.

L – Subs.: sil – Alt.: 3–6 – Note: an arctic-alpine, probably circumpolar lichen found on the top of isolated siliceous boulders, most frequent above treeline. – **Au**: V, T, S, K, St, N. **Sw**: BE, GR, TI, UR, VD, VS. **Fr**: AHP, HAl, AMa, Isè, Sav, HSav. **It**: TAA, Lomb, Piem, VA.

***Umbilicaria pallens* Poelt**

Syn.: Gyrophora cinerascens Arnold var. pallens (Nyl.) Lamy, Umbilicaria atropruinosa Schaer. var. pallens Nyl., Umbilicaria subglabra (Nyl.) Harm. var. pallens (Nyl.) Frey

L – Subs.: sil – Alt.: 4–5 – Note: a silicicolous species resembling *U. subglabra*, but lacking thalloconidia and lower surface therefore pale greyish, regularly bearing apothecia with smooth discs; widespread in the mountains of SW Europe, with several scattered records from the Alps. – **Au**: T. **Sw**: VD, VS. **Fr**: AHP, HAl, AMa, Isè.

***Umbilicaria polyphylla* (L.) Baumg.**

Syn.: Gyrophora aenea Schaer. var. glabra (Ach.) Schaer., *Gyrophora anthracina* (Wulfen) Körb., *Gyrophora glabra* (Ach.) Ach., *Gyrophora polyphylla* (L.) Funck, *Lichen polyphyllus* L., *Umbilicaria anthracina* (Wulfen) Hoffm., *Umbilicaria glabra* Ach.

L – Subs.: sil – Alt.: 2–5 – Note: a variable and ecologically wide-ranging species of rainy areas, with optimum on inclined surfaces of siliceous rocks wetted by rain; widespread throughout the Alps. – **Au**: V, T, S, K, St, N. **Sw**: BE, GR, LU, TI, UR, VD, VS. **Fr**: AHP, HAl, AMa, Isè, Sav, HSav, Vau. **It**: Frl, Ven, TAA, Lomb, Piem, VA. **Sl**: SlA.

***Umbilicaria polyrrhiza* (L.) Fr.**

Syn.: *Actynogyra polyrrhiza* (L.) Schol., *Gyrophora pellita* (Ach.) Ach., *Gyrophora polyrrhiza* (L.) Körb., *Lichen polyrrhizos* L., *Umbilicaria pellita* (Ach.) Ach.

L – Subs.: sil – Alt.: 2–5 – Note: on steeply inclined surfaces of siliceous rocks wetted by rain in humid-rainy areas, with optimum near and above treeline; several records from the Alps require confirmation. – **Au**: T, S, ?N. **Sw**: ?GR, ?VS. **Fr**: Isè, Sav, HSav. **It**: Piem, VA.

***Umbilicaria proboscidea* (L.) Schrad.**

Syn.: Gyrophora polymorpha Schrad. var. proboscidea (L.) Schaer., *Gyrophora proboscidea* (L.) Ach., *Lichen proboscideus* L.

L – Subs.: sil – Alt.: 3–5 – Note: a mainly arctic-alpine, circumpolar lichen found on siliceous rocks, often on small boulders, ecologically similar to *U. cylindrica*, but with a narrower range, with optimum in colder and less continental areas, mostly above treeline. – **Au**: V, T, S, K, St. **Sw**: BE, GR, TI, UR, VD, VS. **Fr**: Isè, HSav. **It**: TAA, Lomb, Piem, VA.

***Umbilicaria ruebeliana* (Du Rietz & Frey) Frey**

Syn.: *Gyrophora ruebeliana* Du Rietz & Frey, *Omphalodiscus ruebelianus* (Du Rietz & Frey) Schol.

L – Subs.: sil – Alt.: 3–6 – Note: on steeply inclined to underhanging, often south – or west-facing surfaces of siliceous rocks above treeline. – **Au**: T, S. **Sw**: BE, GR, TI, VS. **Fr**: AHP, HAl, AMa. **It**: TAA, Lomb, Piem, VA.

***Umbilicaria subglabra* (Nyl.) Harm.**

Syn.: *Agyrophora subglabra* (Nyl.) M. Choisy, *Gyrophora subglabra* Nyl.; incl. Umbilicaria subglabra (Nyl.) Harm. var. schmidtii Frey

L – Subs.: sil – Alt.: 3–5 – Note: on steeply inclined to horizontal, exposed surfaces of siliceous rocks, often at the top of large boulders; widespread throughout the Alps. – **Au**: ?V, T, S, K, St. **Sw**: GR, UR, VD, VS. **Fr**: AHP, HAl, AMa, Isè. **It**: Frl, TAA, Piem, VA.

***Umbilicaria torrefacta* (Lightf.) Schrad.**

Syn.: *Gyrophora erosa* (Weber) Ach., Gyrophora erosa (Weber) Ach. var. torrefacta (Lightf.) Th. Fr., *Gyrophora torrefacta* (Lightf.) Cromb., *Gyrophora torrida* (Ach.) Röhl., *Lichen torrefactus* Lightf., *Umbilicaria erosa* (Weber) Hoffm., *Umbilicaria torrida* (Ach.) Nyl.

L – Subs.: sil – Alt.: 3–5 – Note: an arctic-alpine, circumpolar lichen of siliceous rocks, most frequent above treeline; widespread, but only locally common, in the Alps. – **Au**:  T, S, K, St, N. **Sw**: BE, GR, UR, VD, VS. **Fr**: Isè, Sav, HSav. **It**: Ven, TAA, Lomb, Piem, VA.

***Umbilicaria vellea* (L.) Ach.**

Syn.: *Gyrophora vellea* (L.) Ach., *Gyrophora vellerea* Nyl., *Lichen velleus* L.

L – Subs.: sil – Alt.: 3–6 – Note: an arctic-alpine, probably circumpolar lichen found on steeply inclined, exposed surfaces of siliceous rocks with some water seepage, especially along fissures, with optimum above treeline; widespread throughout the siliceous Alps. – **Au**: V, T, S, K, St. **Sw**: BE, GR, TI, UR, VS. **Fr**: AHP, HAl, AMa, Isè, HSav. **It**: Frl, Ven, TAA, Lomb, Piem, VA, Lig.

***Umbilicaria virginis* Schaer.**

Syn.: *Gyrophora stipitata* (Nyl.) Branth, *Gyrophora virginis* (Schaer.) Frey, *Omphalodiscus virginis* (Schaer.) Schol., *Umbilicaria rugifera* Nyl., *Umbilicaria stipitata* Nyl.

L – Subs.: sil – Alt.: 6 – Note: an arctic-alpine, probably circumpolar lichen found on wind-exposed siliceous cliffs, often in small niches and under overhangs; strictly limited to the nival belt in the Alps. – **Au**: T, S, K. **Sw**: BE, GR, TI, UR, VS. **Fr**: AHP, HAl, AMa, HSav. **It**: TAA, Lomb, Piem, VA.

***Usnea barbata* (L.) F.H. Wigg.**

Syn.: *Lichen barbatus* L., *Usnea alpina* Motyka, *Usnea catenulata* Motyka, *Usnea caucasica* Vain., *Usnea cembricola* Motyka, *Usnea esthonica* Räsänen, *Usnea freyi* Motyka, *Usnea graciosa* Motyka, *Usnea maxima* Motyka, *Usnea pendulina* Motyka, *Usnea plicata* (L.) F.H. Wigg., Usnea plicata (L.) F.H. Wigg. var. pendulina (Motyka) Clauzade & Cl. Roux, Usnea plicata (L.) F.H. Wigg. var. prostrata (Vain. *ex* Räsänen) Clauzade & Cl. Roux, *Usnea rugulosa* Vain., *Usnea scabrata* Nyl., Usnea scabrata Nyl. var. rugulosa (Vain.) Keissl., *Usnea scrobiculata* Motyka, *Usnea tenax* Motyka, *Usnea tortuosa* De Not.

L – Subs.: cor, xyl, sil – Alt.: 2–4 – Note: a boreal-montane species found in oroboreal-montane forests with high rainfall and frequent fog, especially on branches and twigs of *Picea*. This is one of the most frequent and morphologically most variable species of the genus; widespread throughout the Alps. – **Au**: V, T, S, K, St, O, N. **Ge**: OB. **Sw**: AP, BE, FR, GL, GR, LU, SG, SZ, TI, UR, UW, VD, VS. **Fr**: AHP, HAl, AMa, Isè, Sav, HSav, Vau. **It**: Frl, Ven, TAA, Lomb, Piem, VA. **Sl**: SlA, Tg.

***Usnea cavernosa* Tuck.**

Syn.: *Usnea arnoldiana* Zahlbr., *Usnea lacunosa* Willd., *Usnea microcarpa* Arnold

L – Subs.: cor – Alt.: 3–4 – Note: this species seems to be more frequent in Central and Southern Europe; it is restricted to damp montane to subalpine forests, on branches of coniferous and deciduous trees; widespread throughout the Alps. – **Au**: T, S, K, St, N. **Ge**: OB, Schw. **Sw**: BE, GR, LU, SG, SZ, UR, VD, VS. **Fr**: AHP, AMa, Isè, Sav, HSav. **It**: Frl, TAA, Lomb, Piem, Lig. **Sl**: SlA.

***Usnea ceratina* Ach.**

Syn.: Usnea ceratina Ach. f. incurvescens (Arnold) H. Olivier

L – Subs.: cor – Alt.: 2–4 – Note: a cool-temperate to boreal-montane lichen also known from the Southern Hemisphere found on branches of trees in damp forests with frequent fog; widespread throughout the Alps. – **Au**: T, S, St, O, N. **Ge**: OB. **Sw**: BE, GL, GR, LU, SZ, UR, UW, ?VS. **Fr**: Sav, HSav. **It**: Frl, Ven, TAA, Lomb, Piem, VA, Lig. **Sl**: SlA, Tg.

***Usnea cornuta* Körb.**

Syn.: *Usnea constrictula* Stirt., *Usnea inflata* (Duby) Motyka, Usnea inflata (Duby) Motyka var. cornuta (Körb.) Clauzade & Cl. Roux, Usnea *intexta* Stirt., Usnea intexta Stirt. var. constrictula (Stirt.) D. Hawksw. & D. Chapm., *Usnea subpectinata* Stirt.

L – Subs.: cor, sil – Alt.: 2–3 – Note: a chemically and morphologically variable epiphytic species also rarely occurring on siliceous rocks, restricted to damp sites with frequent fog, mostly in the montane belt. – **Sw**: BE, LU, UR, UW. **Fr**: HAl, Var. **It**: Frl, TAA, Lig.

***Usnea dasaea* Stirt.**

Syn.: *Usnea dolosa* Motyka

L – Subs.: cor – Alt.: 1–3 – Note: a mainly southern species in Europe found on twigs in moist woodlands, exceptionally on rocks, at relatively low elevations, with a few records from the Southern Alps. – **It**: Ven, Lomb. **Sl**: SlA.

***Usnea dasopoga* (Ach.) Nyl.**

Syn.: Usnea barbata (L.) F.H. Wigg. var. dasopoga (Ach.) Fr., *Usnea bicolor* (Motyka) Bystrek, *Usnea capillaris* Motyka, *Usnea dasopoga* (Ach.) Nyl., Usnea dasopoga (Ach.) Nyl. subsp. bicolor Motyka, Usnea dasopoga (Ach.) Nyl. subsp. melanopoga Motyka, Usnea dasopoga (Ach.) Nyl. subsp. stramineola Motyka, Usnea dasopoga (Ach.) Nyl. subsp. tuberculata Motyka, *Usnea diplotypus* Vain., *Usnea fibrillosa* Motyka, *Usnea filipendula* Stirt., Usnea filipendula Stirt. var. capillaris (Motyka) Clauzade & Cl. Roux, *Usnea flagellata* Motyka, *Usnea hirtella* (Arnold) Motyka, *Usnea implexa* Motyka *nom.illeg. non* Hoffm., *Usnea melanopoga* (Motyka) Bystrek, *Usnea meylanii* Motyka, *Usnea muricata* Motyka, Usnea plicata (L.) F.H. Wigg. var. dasopoga Ach., *Usnea saxicola* Anders, *Usnea spuria* (Motyka) Bystrek, *Usnea stramineola* (Motyka) Bystrek, *Usnea sublaxa* Vain., *Usnea subscabrata* (Vain.) Motyka, *Usnea tuberculata* (Motyka) Bystrek

L – Subs.: cor, xyl – Alt.: 2–4 – Note: a variable species, most common in humid montane deciduous forests with frequent fog, both on branches and on boles; *U. diplotypus* *s.str.* corresponds to short morphotypes of *U. dasopoga*; widespread throughout the Alps. – **Au**: V, T, S, K, St, O, N. **Ge**: OB. **Sw**: BE, FR, GL, GR, LU, SG, SZ, TI, UR, UW, VD, VS. **Fr**: AHP, HAl, AMa, Isè, Sav, HSav, Vau. **It**: Frl, Ven, TAA, Lomb, Piem, VA, Lig. **Sl**: SlA, Tg.

***Usnea florida* (L.) F.H. Wigg.**

Syn.: *Lichen floridus* L., Usnea barbata (L.) F.H. Wigg. var. florida (L.) Fr., Usnea florida (L.) F.H. Wigg. subsp. arbuscula Motyka, Usnea florida (L.) F.H. Wigg. subsp. fagofila Motyka, Usnea florida (L.) F.H. Wigg. subsp. pseudostrigosa Motyka, *Usnea tominii* Räsänen

L – Subs.: cor, xyl – Alt.: 3–4 – Note: a boreal-montane to cool-temperate lichen found in forests with frequent fog, on twigs and branches, with optimum in the upper montane and subalpine belts; often confused with *U. intermedia*; see also note on *U. subfloridana*; widespread throughout the Alps, but presently extremely rare and declining. – **Au**: V, T, S, K, St, O, N. **Ge**: OB. **Sw**: BE, GL, ?GR, SG, SZ, UR, UW, VD, ?VS. **Fr**: AHP, Isè, Sav, HSav. **It**: Frl, Ven, TAA, Lomb, Piem, VA, Lig. **Sl**: SlA, Tg.

***Usnea glabrata* (Ach.) Vain.**

Syn.: Usnea barbata (L.) F.H. Wigg. var. sorediifera Arnold, Usnea florida (L.) F.H. Wigg. var. sorediifera (Arnold) Hue, *Usnea kujalae* Räsänen, Usnea plicata (L.) F.H. Wigg. var. glabrata Ach., *Usnea sorediifera* (Arnold) Lynge *non auct*.

L – Subs.: cor, xyl – Alt.: 2–4 – Note: on bark, sometimes on lignum, in cold-humid, but open situations, mostly in the upper montane and subalpine belts; declining throughout the Alps. – **Au**: V, T, S, K, St, N. **Ge**: Ge. **Sw**: BE, GR, LU, TI, VD, VS. **Fr**: AHP, HAl, Isè. **It**: Ven, TAA, Lomb, VA. **Sl**: SlA.

***Usnea glabrescens* (Nyl. *ex* Vain.) Vain. var. glabrescens**

Syn.: Usnea barbata (L.) F.H. Wigg. var. glabrescens Nyl. *ex* Vain., *Usnea compacta* Motyka, *Usnea distincta* Motyka, *Usnea extensa* Vain., *Usnea glabrella* (Motyka) Räsänen, *Usnea laricina* Vain. *ex* Räsänen *non auct*.

L – Subs.: cor, xyl – Alt.: 3–4 – Note: on bark and lignum in cold-humid but open situations in montane to subalpine forests; widespread throughout the Alps. – **Au**: V, T, S, K, St, O, N. **Ge**: OB. **Sw**: BE, GL, GR, SZ, UR, VD. **Fr**: AHP, HAl, AMa, Sav, Var. **It**: Ven, TAA, Lomb, Piem, VA. **Sl**: SlA.

***Usnea glabrescens* (Nyl. *ex* Vain.) Vain. var. fulvoreagens Räsänen**

Syn.: *Usnea fulvoreagens* (Räsänen) Räsänen

L – Subs.: cor, xyl – Alt.: 2–4 – Note: on twigs and branches of conifers, more rarely of deciduous trees in cold-humid, open woodlands with frequent fog; chemically heterogeneous. – **Au**: T, S, K, St, O. **Ge**: OB. **Sw**: UW, ?VS. **Fr**: Var. **It**: Frl, Ven, Lomb. **Sl**: A.

***Usnea hirta* (L.) F.H. Wigg.**

Syn.: *Lichen hirtus* L., Usnea barbata (L.) F.H. Wigg. var. hirta (L.) Fr., Usnea florida (L.) F.H. Wigg. var. hirta (L.) Ach., *Usnea foveata* Vain., *Usnea glaucescens* Vain., Usnea hirta (L.) F.H. Wigg. subsp. helvetica Motyka, Usnea hirta (L.) F.H. Wigg. subsp. laricicola Motyka, Usnea hirta (L.) F.H. Wigg. subsp. villosa (Ach.) Motyka, Usnea plicata (L.) F.H. Wigg. var. foveata (Vain.) Clauzade & Cl. Roux, *Usnea pulvinata* Motyka *ex* Räsänen, *Usnea variolosa* Motyka

L – Subs.: cor, xyl – Alt.: 2–4 – Note: most common in climatically continental, but humid areas, on bark (branches and boles) of isolated trees and on lignum (*e.g.* wooden fences and poles); widespread throughout the Alps. – **Au**: V, T, S, K, St, O, N. **Ge**: OB. **Sw**: BE, GR, LU, SZ, TI, UR, UW, VS. **Fr**: AHP, HAl, AMa, Isè, Sav, HSav, Var, Vau. **It**: Frl, Ven, TAA, Lomb, Piem, VA, Lig. **Sl**: SlA.

***Usnea intermedia* (A. Massal.) Jatta**

Syn.: Usnea barbata (L.) F.H. Wigg. var. intermedia A. Massal., *Usnea carpatica* Motyka, *Usnea faginea* Motyka, Usnea florida (L.) F.H. Wigg. subsp. fistulosa Motyka, Usnea florida (L.) F.H. Wigg. subsp. floridula Motyka, Usnea florida (L.) F.H. Wigg. subsp. rubrireagens Vězda, Usnea florida (L.) F.H. Wigg. var. rigida Ach., *Usnea glauca* Motyka, Usnea glauca Motyka var. pseudoflorida (Motyka) Motyka, *Usnea hapalotera* (Harm.) Motyka, *Usnea harmandii* Motyka, *Usnea leiopoga* Motyka, *Usnea montana* Motyka, *Usnea neglecta* Motyka, *Usnea protea* Motyka, *Usnea rigida* Motyka *nom.illeg. non* Vain., Usnea rigida Motyka var. faginea (Motyka) Clauzade & Cl. Roux, Usnea rigida Motyka var. hapalotera (Harm.) Clauzade & Cl. Roux, Usnea rigida Motyka var. neglecta (Motyka) Keissl., Usnea rigida Motyka var. protea (Motyka) Clauzade & Cl. Roux, *Usnea smaragdina* Motyka

L – Subs.: cor, xyl – Alt.: 2–4 – Note: a polymorphic and not clearly understood taxon, most common on conifers in humid montane forests, which is treated here in a very broad sense; widespread throughout the Alps. – **Au**: V, T, S, K, St, O, N. **Ge**: OB. **Sw**: BE, GL, GR, LU, SZ, TI, UR, UW, VD, VS. **Fr**: AHP, HAl, AMa, Isè, Sav, HSav, Var, Vau. **It**: Frl, Ven, TAA, Lomb, Piem, VA. **Sl**: SlA.

***Usnea longissima* Ach.**

Syn.: *Dolichousnea longissima* (Ach.) Articus

L – Subs.: cor – Alt.: 3–4 – Note: a mainly cool-temperate to boreal-montane species found on branches of old (mostly coniferous) trees in closed, semi-natural forests in areas with high rainfall and frequent fog; most records are historical, presently the species is restricted to a few localities and very much declining. – **Au**: T, S, K, St, O, N. **Ge**: OB. **Sw**: BE, FR, GL, GR, LU, SZ, UR, UW, VD, ?VS. **Fr**: Isè. **It**: Frl, Ven, TAA, Piem. **Sl**: SlA, Tg.

***Usnea perplexans* Stirt.**

Syn.: *Usnea arnoldii* Motyka, Usnea fulvoreagens *auct. non* (Räsänen) Räsänen, *Usnea lapponica* Vain., Usnea laricina *auct. non* Vain. *ex* Räsänen

L – Subs.: cor – Alt.: 2–4 – Note: on branches of conifers in montane, cold-humid forests with frequent fog; frequent throughout the Alps. – **Au**: T, S, K, St, N. **Ge**: Ge. **Sw**: BE, GL, GR, LU, SZ, TI, UW, VD, VS. **Fr**: AHP, HAl, AMa, Isè, HSav, Var, Vau. **It**: Ven, TAA, Piem, VA. **Sl**: SlA.

***Usnea rubicunda* Stirt.**

Syn.: *Usnea protensa* Stirt., Usnea rubiginea *auct. non* (Michx.) A. Massal., *Usnea sublurida* Stirt.

L – Subs.: cor – Alt.: 1–4 – Note: a mild-temperate, chiefly Mediterranean-Atlantic species found on ancient specimens of *Quercus cerris*, *Q. suber*, and other acid-barked broad-leaved trees in open, but semi-natural, warm-humid forests below the montane belt; most records from the Alps are historical and/or dubious. – **Au**: S, St. **Sw**: ?GR. **Fr**: Var. **It**: Ven, TAA, Lig.

***Usnea silesiaca* Motyka**

Syn.: *Usnea madeirensis* Motyka

L – Subs.: cor – Alt.: 3 – Note: a species resembling *U. subfloridana* in the rigid, shrubby to rarely (in the Alps) pendant thallus with black base and many annulations, with more or less even, orbicular soralia and a thin medulla, containing salazinic acid; on acid bark, mostly on branches; widespread in the Holarctic region, in Europe most common in the West; from the Alps there are only some scattered records. – **Au**: T. **Sw**: LU, UR, UW.

***Usnea subfloridana* Stirt.**

Syn.: *Usnea comosa* (L.) Vain. *non* Pers., Usnea comosa (L.) Vain. subsp. similis Motyka, *Usnea similis* (Motyka) Räsänen

L # – Subs.: cor, xyl, sil – Alt.: 2–5 – Note: on branches of trees in relatively closed forests (but then in the upper parts of the crowns), and on isolated trees, one of the few species of *Usnea* which, albeit with stunted specimens, is also found at low altitudes and in relatively disturbed habitats. The results of a recent molecular study show that the separation between this species and *U. florida* cannot be maintained. – **Au**: V, T, S, K, St, O, N, B. **Ge**: OB. **Sw**: BE, FR, GL, GR, LU, SZ, TI, UR, UW, VD, VS. **Fr**: AHP, HAl, AMa, Isè, HSav, Var, Vau. **It**: Frl, Ven, TAA, Lomb, Piem, VA. **Sl**: SlA, Tg.

***Usnea subscabrosa* Nyl. *ex* Motyka**

L – Subs.: cor, sil – Alt.: 2–4 – Note: a well-defined, mainly southwestern species in Europe, found both on basic siliceous rocks and on bark in humid situations, mostly in the upper montane and subalpine belts, also reported from the base of the Western Alps (France). – **Fr**: Isè.

***Usnea substerilis* Motyka**

Syn.: *Usnea sorediifera* *sensu* Motyka *non* (Arnold) Lynge, *Usnea stuppea* (Räsänen) Motyka

L – Subs.: cor, xyl – Alt.: 2–4 – Note: a subcontinental species, often confused in the past with the related *U. perplexans*; probably one of the most frequent *Usnea* of the Alps. – **Au**: V, T, S, K, St, N. **Ge**: OB. **Sw**: BE, GL, GR, LU, SZ, TI, VD, VS. **Fr**: AHP, HAl, AMa, Isè, Var, Vau. **It**: Ven, TAA, Lomb, VA. **Sl**: SlA.

***Usnea wasmuthii* Räsänen**

L – Subs.: cor – Alt.: 1–4 – Note: a species resembling *U. subfloridana* in the shrubby thallus with black base, with large, longitudinally streched soralia, containing salazinic and/or barbatic acid; ecologically similar to *U. florida*, but more frequent at lower altitudes in warm-humid areas. – **Au**: K. **Ge**: Schw. **Sw**: LU, SZ, VS. **Fr**: Vau. **It**: Lig.

***Usnocetraria oakesiana* (Tuck.) M.J. Lai & J.C. Wei**

Syn.: *Allocetraria oakesiana* (Tuck.) Randlane & A. Thell, *Cetraria bavarica* Kremp., *Cetraria oakesiana* Tuck., *Cetraria ochrocarpa* (Eggerth) Lettau, *Platysma oakesianum* (Tuck.) Nyl., *Tuckermannopsis oakesiana* (Tuck.) Hale

L – Subs.: cor, xyl – Alt.: 3–4 – Note: a cool-temperate to boreal-montane, incompletely circumpolar species found on basal parts of conifers in humid-cold montane forests, more rarely on lignum (*e.g.* on stumps); widespread throughout the Alps, but generally not common, and probably declining. – **Au**: V, T, S, K, St, O, N. **Ge**: OB, Schw. **Sw**: BE, GR, LU, SZ, UR, UW. **It**: Frl, Ven, TAA. **Sl**: SlA.

***Vahliella leucophaea* (Vahl) P.M. Jørg.**

Syn.: *Fuscopannaria leucophaea* (Vahl) P.M. Jørg., *Lecidea microphylla* (Lilj.) Ach., *Lichen leucophaeus* Vahl, *Massalongia cheilea* Mudd, *Pannaria austriaca* Zahlbr., *Pannaria cheilea* (Mudd) Leight., *Pannaria leucophaea* (Vahl) P.M. Jørg., *Pannaria microphylla* (Lilj.) Delise *ex* Bory, *Pannularia microphylla* (Lilj.) Stizenb., *Parmeliella microphylla* (Lilj.) Müll. Arg., *Parmeliella pseudocraspedia* (Hazsl.) Gyeln.

L – Subs.: sil, int – Alt.: 2–5 – Note: on basic siliceous rocks in sheltered and humid situations, such as in seepage tracks; widespread throughout the Alps. – **Au**: V, T, S, K, St, O, N, B. **Ge**: Schw. **Sw**: BE, GR, SZ, TI, VS. **Fr**: AHP, HAl, AMa, Isè, HSav, Var. **It**: Ven, TAA, Lomb, Piem, VA, Lig. **Sl**: SlA, Tg.

***Vahliella saubinetii* (Mont.) P.M. Jørg.**

Syn.: *Fuscopannaria saubinetii* (Mont.) P.M. Jørg., *Massalongia rabenhorstiana* Gyeln., *Pannaria saubinetii* (Mont.) Nyl., Parmelia rubiginosa (Ach.) Ach. var. pulveraceogranulosa Grognot, *Parmelia saubinetii* Mont., *Parmeliella saubinetii* (Mont.) Zahlbr., Parmeliella saubinetii (Mont.) Zahlbr. f. grisea Gyeln., *Trachyderma saubinetii* (Mont.) Trevis.

L – Subs.: cor – Alt.: 1–2 – Note: a mild-temperate species found on trunks of mature deciduous trees in rather shaded and humid situations; apparently most frequent in the Western Alps. – **Fr**: AHP, AMa, HSav, Var, Vau. **It**: Ven, Piem.

***Varicellaria hemisphaerica* (Flörke) I. Schmitt & Lumbsch**

Syn.: *Pertusaria hemisphaerica* (Flörke) Erichsen, *Pertusaria speciosa* Høeg, *Variolaria hemisphaerica* Flörke

L – Subs.: cor – Alt.: 1–3 – Note: a mild-temperate species found on old deciduous trees, especially oaks, in open, mostly deciduous forests; widespread throughout the Alps. – **Au**: V, T, S, K, St, O, N. **Ge**: OB. **Sw**: BE, LU, SZ, UR, UW, VD. **Fr**: AHP, Isè, HSav, Var, Vau. **It**: Frl, Ven, TAA, Lomb, Piem. **Sl**: SlA, Tg.

***Varicellaria lactea* (L.) I. Schmitt & Lumbsch**

Syn.: *Lichen lacteus* L., *Ochrolechia lactea* (L.) Matzer & Hafellner, *Pertusaria lactea* (L.) Arnold, *Variolaria lactea* (L.) Pers.; incl. Pertusaria lactea (L.) Arnold f. faginea Erichsen

L – Subs.: sil, int, cor – Alt.: 2–5 – Note: optimum on steeply inclined, lime-free, rather shaded surfaces of siliceous rocks in humid areas, rarely corticolous; widespread throughout the Alps. – **Au**: V, T, S, K, St, N, B. **Ge**: Ge. **Sw**: BE, GR, LU, VD, VS. **Fr**: HAl, AMa, Isè, Sav, HSav, Var, Vau. **It**: Frl, Ven, TAA, Lomb, Piem, VA, Lig. **Sl**: SlA.

***Varicellaria rhodocarpa* (Körb.) Th. Fr.**

Syn.: *Pertusaria rhodocarpa* Körb., *Varicellaria kemensis* Räsänen, *Varicellaria microsticta* Nyl.

L – Subs.: deb, cor, xyl, ter-sil – Alt.: 4–5 – Note: a mainly arctic-alpine species found on acid soil and plant remains, more rarely on lignum or on rocks in tundra-like environments. – **Au**: V, T, S, K, St, O, N. **Ge**: OB. **Sw**: BE, GR, TI, UR, UW, VS. **Fr**: HAl, Sav, HSav. **It**: Frl, Ven, TAA, Lomb. **Sl**: SlA.

***Verrucaria aberrans* Garov.**

L # – Subs.: sil – Alt.: 2–3 – Note: a species with a thin, spreading, dark olive to brown, continuous to rimulose thallus, hemispherically protruding perithecia (to 0.25 mm in diam.), an involucrellum adpressed in the apical region, and oblong to ellipsoid ascospores (to *c.* 30 µm long); on siliceous rocks (porphyr, granite) in the shade of deciduous forests; known from a few localities in the Italian Alps. – **It**: Lomb.

***Verrucaria acrotella* Ach.**

Syn.: Verrucaria aethiobola Wahlenb. var. acrotella (Ach.) H. Olivier

L # – Subs.: sil – Alt.: 2–3 – Note: a species found on siliceous rocks, mostly close to the ground, which needs further study. – **Fr**: AMa, Sav, HSav, Vau. **It**: TAA, Lig.

***Verrucaria adelminienii* Zschacke**

L # – Subs.: cal – Alt.: 2–4 – Note: a calcicolous species resembling *V. muralis*, with a thin, greenish-white thallus, slightly protruding ascomata (to *c.* 0.5 mm in diam.) with a closed, black exciple, and ascospores to *c.* 25 µm long; in the Central European mountains it is most common in the montane belt; there are scattered records from the Alps, but several recent ones are uncertain. – **Au**: S, N. **Sw**: LU, SZ. **Fr**: HAl, AMa, Isè, Var, Vau.

***Verrucaria aethiobola* Wahlenb.**

Syn.: *Lithocia aethiobola* (Wahlenb.) Stein, *Lithocia chlorotica* (Hepp) Stein, *Pyrenula aethiobola* (Wahlenb.) Ach., *Verrucaria aquilella* Nyl., *Verrucaria catalepta* *sensu* Schaer. *non* (Ach.) Spreng., *Verrucaria chlorotica* Hepp *non* Ach., *Verrucaria fuscocinerascens* Nyl., Verrucaria hydrela Ach. var. aethiobola (Wahlenb.) A. Massal., ?*Verrucaria hibernica* Zschacke, *Verrucaria laevata* Ach. *non auct. nec* *sensu* Körb., ?*Verrucaria rimosella* Nyl., *Verrucaria viridicana* Erichsen

L – Subs.: sil-aqu, cal-aqu – Alt.: 2–5 – Note: a widespread species, both in Northern Europe and in the Alps, periodically submerged on hard, mostly siliceous rocks along creeks. See also note to *V. csernaensis*. – **Au**: V, T, S, K, St, N. **Sw**: BE, GR, SZ, UR, VS. **Fr**: AHP, HAl, AMa, Isè, Sav, HSav, Var. **It**: Ven, TAA, Lomb, Piem.

***Verrucaria algovica* Servít**

Syn.: *Amphoridium algovicum* (Servít) Servít

L # – Subs.: cal – Alt.: ?5 – Note: a calcicolous species with a mainly endolithic, whitish thallus which is partly emerging as a thin continuous crust with black prothallus lines, entirely immersed perithecia (to 0.7 mm in diam.) without involucrellum, and ellipsoid ascospores (to 30 µm long); only known from the type locality in the Eastern Alps (Germany). – **Ge**: Schw.

***Verrucaria aljazevi* Servít**

L # – Subs.: cal – Alt.: 3 – Note: a calcicolous species with an endolithic, whitish thallus, black, protruding perithecia (to 0.3 mm in diam.), and narrowly ellipsoid ascospores (to *c.* 30 µm long); only known from the type locality in the montane belt of the Eastern Alps (Slovenia). – **Sl**: SlA.

***Verrucaria alpicola* Zschacke**

L – Subs.: sil-aqu, cal-aqu – Alt.: 3–5 – Note: a species of the *V. elaeomelaena*-group with a thin, continuous, epilithic, dark brown to nearly black thallus which is rimose only around the ascomata, in section view the upper cortex with a dark brown to black pigment and partly with a black basal layer, ascomata protruding from the thallus and covered by thin thalline layer, with a brown exciple and a laterally spreading involucrellum reaching the base of the perithecia, and with narrowly ellipsoid ascospores (to *c.* 35 µm long); a typically sub-aquatic species which often occurs in the splash water zone in streams, but also at temporarily inundated sites in springs, both on calcareous and on siliceous rocks, in sunny to moderately shaded sites, mostly in upland areas; probably more widespread in the Alps. – **Au**: K. **Ge**: Schw. **Sw**: BE, GR. **It**: Ven, TAA, VA.

***Verrucaria alpigena* Breuss**

L – Subs.: cal-aqu – Alt.: 3–5 – Note: on calciferous rocks in upland areas; closely related to *V. muralis*, but with larger spores, it has been reported only from the Eastern Alps (Austria, Italy) and the Carpathian Mts. – **Au**: T, St, O, N. **It**: TAA.

***Verrucaria ampezzana* Servít**

L # – Subs.: cal – Alt.: 4–5 – Note: on inclined surfaces of calciferous schists near or above treeline; a species described from South Tyrol and also reported from the French Alps (Roux et al. 2014). According to Breuss (see Nimis 2016) it is closely related to *Parabagliettoa dufourii*, differing in the larger spores, and should be included in *Parabagliettoa*. – **Fr**: HAl. **It**: Ven.

***Verrucaria anceps* Kremp.**

Syn.: *Polyblastia anceps* (Kremp.) Servít

L – Subs.: cal – Alt.: 2–5 – Note: on limestone and dolomite in humid-shaded situations below treeline; reported from several localities in the mountains of Central Europe and the Alps. – **Au**: V, T, S, O, N. **Ge**: OB. **Sw**: GR, TI. **Fr**: AMa, Vau. **It**: Ven, TAA, Piem, VA.

***Verrucaria andesiatica* Servít**

L – Subs.: sil-aqu, int-aqu – Alt.: 3–4 – Note: a species with a very thin, epilithic, olive-brown thallus, ascomata (to 0.5 mm in diam.) in convex to conical warts, with a superficially spreading, carbonised involucrellum, and oblong to ovoid ascospores (to *c.* 30 µm long); on moist siliceous rocks from the lowlands (type!) to treeline; widespread in Europe but very rarely collected. – **Sw**: SZ. **Fr**: AMa.

***Verrucaria anulata* Zschacke**

L # – Subs.: cal – Alt.: 4 – Note: a species resembling in habitus *V. cincta* Hepp, but ascomata larger and ascospores smaller, with a whitish-grey, thin, continuous, wrinkled thallus, black, sessile ascomata (to 1 mm in diam.) with a depressed ostiolar region, surrounded by a thalline ridge, with a well-developed, slightly convex involucrellum, 8-spored asci, and broadly ellipsoid ascospores (16–19 × 11–13 μm); on a calcareous rock at high elevation; only known from the type locality in the Eastern Alps (Switzerland). – **Sw**: GR.

***Verrucaria anziana* Garov.**

L # – Subs.: sil, sil-aqu – Alt.: 3 – Note: this species was often considered as a synonym of *V. latebrosa*. It usually grows on siliceous rocks by streams, rivers and lakes, and is known from both the Alps (Italy) and Scandinavia. – **It**: Lomb.

***Verrucaria apatela* (A. Massal.) Trevis.**

Syn.: *Lithocia apatela* A. Massal.

L # – Subs.: cal – Alt.: 2–5 – Note: on steeply inclined faces of limestone and dolomite; reported from many sites in Central and Southern Europe, closely related to *V. macrostoma*. – **Sw**: GR, VS. **It**: Ven, TAA, Lomb, Piem.

***Verrucaria apomelaena* (A. Massal.) Hepp**

Syn.: *Lithocia apomelaena* A. Massal.

L # – Subs.: cal – Alt.: 2–3 – Note: a rather poorly known species found both on limestone and on calciferous sandstone. – **Au**: V, S. **Fr**: HSav. **It**: Ven.

***Verrucaria aquatilis* Mudd**

Syn.: *Bachmannia maurula* (Müll. Arg.) Zschacke, *Verrucaria maurula* Müll. Arg., *Verrucaria retecta* Zschacke; incl. *Verrucaria vitricola* Nyl.

L – Subs.: sil-aqu – Alt.: 2–4 – Note: distinguished from other freshwater species by the thin blackish thallus and the very small, broadly ellipsoid ascospores, this lichen grows on siliceous or calcareous rocks submerged in cold creeks; probably more widespread in the Alps, but overlooked, like many amphibious lichens. – **Au**: T, S, K, St, N. **Fr**: AHP, HAl, AMa, HSav. **It**: Ven, TAA.

***Verrucaria areolatodiffracta* Zschacke**

L # – Subs.: sil-aqu – Alt.: 4 – Note: a species probably related to *V. latebrosa*, with a cream-coloured to brownish, continuous to partly rimose thallus which around the ascomata is areolate, the areoles of various shapes and even subsquamulose, in section paraplectenchymatic, ascomata (to 0.35 mm in diam.) immersed in the areoles, with an (almost) entire, well-developed involucrellum, 8-spored asci, and obovoid, simple ascospores (26–31 × 13–15 μm); on submerged siliceous rocks in streams; only known from the type locality in the Eastern Alps (Switzerland). – **Sw**: GR.

***Verrucaria arnoldii* J. Steiner**

Syn.: Verrucaria hiascens *auct. non* (Ach.) Spreng., Verrucaria hochstetteri var. arnoldii (J. Steiner) Clauzade & Cl. Roux

L # – Subs.: cal – Alt.: 2–5 – Note: a species of the *V. hochstetteri*-group with an unresolved nomenclature (superfluous new name for a legitimate species), with a white to greyish thallus, ascomata sunken in the rock with the conical ostiolar region breaking through thalline/substratic warts, reported to be distinguishable by the smaller ascospores (24–35 × 12–18 μm); on limestone and dolomite, widespread in Europe including the Alps, but not always distinguished. – **Au**: V, T, S, K, St, O. **Fr**: AHP, AMa, Drô, Isè. **Sl**: Tg.

***Verrucaria asperula* Servít**

L # – Subs.: cal – Alt.: 3–4 – Note: a species described from Germany and also reported from relatively warm sites in the Austrian, French and Italian Alps. – **Au**: St, O. **Fr**: Sav.

***Verrucaria austriaca* Riedl**

Syn.: *Verrucaria irrigua* Zschacke *non* Taylor

L # – Subs.: int-aqu – Alt.: 3 – Note: a species with a thin, epilithic, grey, somewhat rimose thallus, almost completely immersed perithecia (to *c.* 0.25 mm in diam.), and up to *c.* 20 µm long ascospores; on irrigated outcrops of marl, only known from the type locality in the Eastern Alps (Austria). – **Au**: N.

***Verrucaria banatica* Servít**

L – Subs.: cal – Alt.: 3 – Note: a calcicolous species with a thin, rimose, brownish-grey thallus, hemispherically protruding ascomata (to *c.* 0.4 mm in diam.) with an adpressed involucrellum reaching down half of the the perithecium, and ellipsoid to oblong ascospores (to *c.* 25 µm long); in the study area only known from the Eastern Alps (Austria). – **Au**: V, St, O, N.

***Verrucaria bavarica* Servít**

L # – Subs.: cal – Alt.: ?5 – Note: a species resembling *V. dolomitica*, with a very thin, epilithic, whitish thallus, slightly to hemispherically protruding, black perithecia, a thin involucrellum in the upper half, and ellipsoid to oblong ascospores (to *c.* 32 µm long); only known from the type locality in the Eastern Alps (Germany). – **Ge**: Schw.

***Verrucaria beltraminiana* (A. Massal.) Trevis.**

Syn.: *Lithocia beltraminiana* A. Massal.

L # – Subs.: cal – Alt.: 1–5 – Note: on horizontal to weakly inclined surfaces of calcareous rocks, including walls in small settlements. A critical species, closely related to (but perhaps distinct from) *Verruculopsis lecideoides*, which should probably be included in *Verruculopsis*. – **Au**: ?V, ?T, St. **Fr**: HAl, AMa. **It**: Ven, Piem.

***Verrucaria boblensis* Servít**

L # – Subs.: cal – Alt.: 3 – Note: a calcicolous species recalling (in section) *V. muralis*, but with smaller ascospores, characterised by a thin, rimose, dirty-whitish thallus, conically to hemispherically protruding ascomata (to *c.* 0.25 mm in diam.) with a thick, adpressed involucrellum reaching down about a third of the perithecium, and ellipsoid to oblong ascospores (to *c.* 20 µm long); known from a few scattered records in Europe, including the Eastern Alps (Austria). – **Au**: O.

***Verrucaria bryoctona* (Th. Fr.) Orange**

Syn.: *Thelidium bryoctonum* Th. Fr.

L – Subs.: ter-cal – Alt.: 3–4 – Note: a species with a greyish green, granular thallus consisting of goniocysts (to *c.* 40 µm in diam.), subspherical ascomata without involucrellum, partly immersed in the substrate (to *c.* 0.3 mm in diam.), and narrowly ellipsoid ascospores (to *c.* 30 µm long) often with small terminal gelatinous appendages, becoming 1-septate with age; on basic soil and terricolous moribund bryophytes; widespread in Western Europe, with a few records from the Eastern Alps (Austria). – **Au**: V, K, N.

***Verrucaria caerulea* DC.**

Syn.: *Involucrothele bormiensis* (Servít) Servít, *Involucrothele plumbea* (Ach.) Servít, *Thelidium plumbeum* (Ach.) Servít, Thelidium plumbeum (Ach.) Servít f. orbiculare Kremp. *ex* Servít, *Verrucaria bormiensis* Servít, *Verrucaria glaucina* Ach. *non auct.*, *Verrucaria plumbea* Ach., *Verrucaria truncatula* Nyl.

L – Subs.: cal – Alt.: 2–6 – Note: on steeply inclined surfaces of compact calciferous rocks; widespread throughout the Alps. Probably related to *Staurothele*. – **Au**: V, T, S, K, St, O, N, B. **Ge**: Ge. **Sw**: BE, GR, LU, SZ, VD. **Fr**: AHP, AMa, Drô, Isè, Sav, HSav, Var. **It**: Frl, Ven, TAA, Lomb, Piem, Lig. **Sl**: SlA, Tg.

***Verrucaria caesiopsila* Anzi**

Syn.: *Amphoridium caesiopsilum* (Anzi) Arnold *non* *sensu* Arnold, *Verrucaria integrella* (Hue) Nyl.

L # – Subs.: cal, sil – Alt.: 2–4 – Note: a species of more or less calcareous rocks (limestone and dolomite), known from the Southern Alps only. – **Sw**: GR. **It**: Ven, TAA, Lomb, Piem, VA.

***Verrucaria cambrini* Servít**

L # – Subs.: cal – Alt.: 3 – Note: a species with a thin, epilithic, rimulose to subareolate, olive-greenish thallus, ascomata protruding, but laterally obtected by a thin thalline layer, involucrellum slightly spreading with the lower portion, reaching down about a third of the perithecium, ascospores broadly ellipsoid to subglobose (to 20 µm long); on calcareous rocks (the type is on roof tiles!); known from a few scattered localities in Central Europe, including the Eastern Alps (Austria). – **Au**: V.

***Verrucaria cataleptoides* (Nyl.) Nyl.**

Syn.: *Lithocia cataleptoides* (Nyl.) Arnold, Verrucaria margacea (Wahlenb.) Wahlenb. var. cataleptoides Nyl., Verrucaria cataleptoides (Nyl.) Nyl. f. margolae Servít

L – Subs.: cal-aqu – Alt.: 2–3 – Note: on periodically submerged calcareous rocks (records from other rock types need confirmation). This species, frequently considered as a synonym of *V. aethiobola*, clearly differs both in important morphological characters and in the occurrence on calcareous rocks. – **Au**: T. **Fr**: AHP, AMa, Isè. **It**: TAA.

***Verrucaria cataractophila* Servít**

L # – Subs.: cal, cal-aqu – Alt.: ?3 – Note: a species with a thin, greyish-brown thallus forming patches of *c.* 2 cm in diam., hemispherically protruding, black ascomata (to *c.* 0.2 mm in diam.) with an adpressed involucrellum reaching down to the base of the perithecium, and ellipsoid to oblong ascospores (to *c.* 20 µm long); on dolomite near a waterfall, only known from the type locality in the Eastern Alps (Italy). – **It**: TAA.

***Verrucaria cinereorufa* Schaer.**

L # – Subs.: cal – Alt.: 3–4 – Note: on periodically humid surfaces of calcareous or dolomitic rocks, also reported from the Western Pyrenees and from several sites in Western and Central Europe. The records from France include those of *V. elaeodes* (Hue) Zschacke – **Au**: ?V, St, O, N. **Fr**: AMa, Sav, HSav, Vau. **It**: Frl, Lomb, Piem, Lig. **Sl**: SlA.

***Verrucaria clauzadei* B. de Lesd.**

Syn.: Verrucaria cinereorufa Schaer. var. clauzadei (B. de Lesd.) Clauzade & Cl. Roux

L – Subs.: cal – Alt.: 2 (?5) – Note: a species with a thin, pale violet thallus bordered by black lines, half-immersed perithecia (to 0.6 mm in diam.) with the wall black throughout, and ellipsoid ascospores (to *c.* 33 µm long); on calcareous sandstone and similar rock types at low elevations; known from some scattered records in S Europe; the records from high elevations in Austria are very dubious. – **Au**: ?V, ?T. **Fr**: AMa, Vau. **Sl**: SlA.

***Verrucaria collematodes* Garov.**

L # – Subs.: cal, sil – Alt.: 2–3 – Note: mostly on calciferous or base-rich siliceous substrata, including roofing tiles, walls and mortar; a taxon of the *V. nigrescens*-complex, reported from different countries in Central and Southern Europe; apparently most frequent in the Southern and Western Alps, but perhaps not recognised elsewhere. – **Ge**: Ge. **Fr**: AMa, Sav. **It**: Ven, Lomb, Lig.

***Verrucaria concinnasensu* Schaer. [1836] *non* Borrer [1831**]

L – Subs.: cal – Alt.: 3–5 – Note: a calcicolous species with an epilithic, grey-brown, areolate thallus, the areoles with a dark brown margin and basal layer, broadly conical ascomata (or ostiolar region flattened), a conspicuous, adpressed involucrellum reaching down to the base of the perithecium, and ellipsoid ascospores (to *c.* 22 µm long); widespread in the European mountains, mostly at mid-elevations; from the Alps there are a few scattered records. – **Au**: T, O. **Ge**: Schw. **Sw**: BE. **Fr**: HAl, Sav.

***Verrucaria confluens* A. Massal. *nom.illeg. non* (Weber) F.H. Wigg.**

Syn.: Verrucaria muralis Ach. var. confluens (A. Massal.) Körb.

L # – Subs.: sax – Alt.: 2–3 – Note: this species, which has been often considered to be a synonym of *V. muralis*, differs in the thicker thallus and the crowded perithecia with a thick involucrellum. The species has no valid name. – **Au**: St, O, N. **Fr**: Isè, HSav. **It**: Ven, Lomb.

***Verrucaria consociata* Servìt**

L # – Subs.: sil, sil-aqu – Alt.: 3 – Note: a species with a thin, greenish to olivaceous thallus forming patches to 10 mm in diam., minute (less than 0.2 mm in diam.) hemispherically protruding ascomata partly covered by a thin, granulose thalline layer, an entire, thin involucrellum, and ellipsoid, non-halonate ascospores (16–28 × 8–13 μm); on shaded, temporarily wet siliceous rocks, also reported to form isles on other *Verrucaria* species (type!); widespread in Central Europe, with a single record from the Western Alps (France). – **Fr**: AMa.

***Verrucaria constricta* Zschacke**

L # – Subs.: cal – Alt.: 3 – Note: a species with an epilithic, grey-brown, rimose to areolate thallus, entirely immersed ascomata with a conspicuous adpressed involucrellum reaching down to the base of the perithecium, and ellipsoid ascospores (to *c.* 25 µm long); on more or less calcareous rocks; only known from the type locality in the Western Alps (France). – **Fr**: HSav.

***Verrucaria contardonis* Servít**

L # – Subs.: cal – Alt.: 2 – Note: a calcicolous species with a thin, whitish, spreading, continuous thallus (rimulose around the ascomata), hemispherically protruding ascomata (to 0.2 mm in diam.), an involucrellum adpressed to the perithecial wall and reaching down to the base, and oblong to ellipsoid ascospores (to *c.* 25 µm long); known only from the type locality (Italy). – **It**: Frl.

***Verrucaria corticata* Anzi**

L # – Subs.: cal – Alt.: 2–3 – Note: a species with a thin, yellowish white, marginally sublobate thallus forming patches of 2–5 cm in diam, with a well-developed upper cortex of angular cells, numerous, isolated, semi-immersed, black perithecia, oblong to oblong-clavate, 8-spored asci, and simple, hyaline, oblong ascospores measuring *c.* 8.6 × 6.8 µm, purported (in the description) to differ from *Thrombium epigaeum* in the obviously corticate thallus; only reported from the type locality near Bormio (Premadio), where it was collected on a wall of limestone; the type material is well worthy of further study. – **It**: Lomb.

***Verrucaria cretacea* Zschacke**

L # – Subs.: cal – Alt.: 3 – Note: a species with a chalky white thallus, amphora-shaped (to 0.5 mm wide), entirely immersed ascomata (only the ostiolar region visible), 8-spored asci, and ellipsoid, simple ascospores (*c.* 20 × 8–10 μm); on dolomite, only known with certainty from the type locality in the Eastern Alps (Switzerland). – **Sw**: GR, ?SZ.

***Verrucaria crustificans* (Servít) ined. (provisionally placed here, ICN Art. 36.1b)**

Syn.: *Amphoridium crustificans* Servít

L # – Subs.: sil – Alt.: 2 – Note: a silicicolous species recalling a poorly developed *Placocarpus schaereri*, with a thin, epilithic, whitish-pruinose, rimose to areolate thallus, 1–4 perithecia without involucrellum, immersed in the areolae (to *c.* 0.2 mm in diam.), and oblong to ovoid ascospores (to 20 µm long); only known from the type locality at the base of the Western Alps (Italy). – **It**: Lig.

***Verrucaria cryptica* (Arnold) J. Steiner**

Syn.: *Amphoridium crypticum* Arnold

L # – Subs.: cal – Alt.: 2–6 – Note: on compact calcareous rocks and dolomite near and above treeline; a very critical taxon, related to *V. hochstetteri* *s.lat.*, which needs further study. – **Au**: T. **Fr**: AMa, HSav. **It**: Frl, TAA, Lig.

***Verrucaria csernaensis* Zschacke**

L # – Subs.: sil-aqu, cal-aqu – Alt.: 2–5 – Note: a species described from the Carpathians, often considered as a synonym of *V. aethiobola*. According to Thüs (see Nimis 2016) the epithet *aethiobola* was used for two genetically well-separated taxa: *V. aethiobola* *s.str.* and *V. cernaensis*, and it is likely that at least some of the lowland records from the Alps could refer to the latter species. – **Au**: K.

***Verrucaria dalslandensis* Servít**

L – Subs.: sil – Alt.: 3 – Note: a species resembling *V. muralis*, with a thin, epilithic, uncracked to rimulose, brownish to grey thallus with a reddish tinge, hemispherically protruding ascomata (to 0.25 mm in diam.) with adpressed involucrellum reaching down to the base of the perithecium, and oblong ascospores (to *c.* 20 µm long); on schists and similar acidic substrata; a rarely reported species, but apparently with a wide distribution in Europe, including the Eastern Alps (Austria). – **Au**: T.

***Verrucaria davosensis* Zschacke**

L # – Subs.: sax-aqu – Alt.: ?salp – Note: a species resembling in habitus, and probably related to *V. hydrela*, with a greenish-brown thallus, in section with a black basal layer in fertile areas, ascomata (to 0.4 mm in diam.) covered by a thalline layer, the spherical exciple pigmented only in the ostiolar region, otherwise almost hyaline throughout, 8-spored asci, and ellipsoid, simple ascospores (19–22 × 8–9 μm); on submerged rocks in streams; only known from the type locality in the Eastern Alps (Switzerland). – **Sw**: Sw.

***Verrucaria delitescens* Servít**

Syn.: Amphoridium dolomiticum A. Massal. var. obtectum Arnold, *Amphoridium obtectum* (Arnold) Arnold *non Verrucaria obtecta* Müll. Arg.; incl. Verrucaria delitescens Servít f. mulazensis Servít

L # – Subs.: cal – Alt.: 5 – Note: related to, or perhaps identical with *V. caesiopsila* and/or *V. cryptica*, with an endolithic thallus emerging with dots and brownish patches, immersed perithecia (to *c.* 0.4 mm in diam.) with a minute involucrellum around the ostiolar region, and ellipsoid ascospores (to *c.* 30 µm long); on dolomite and probably other calcareous rocks at high elevations; only known from the type locality in the Eastern Alps (Italy). – **It**: TAA.

***Verrucaria dermatoidea* Servít**

Syn.: Verrucaria veronensis A. Massal. f. dermatoidea A. Massal. *ex* Anzi

L # – Subs.: cal – Alt.: 2 – Note: a calcicolous species with a whitish, rimulose to areolate thallus, hemispherically protruding perithecia (to 0.25 mm in diam.) arising in the centre of the areoles, an involucrellum adpressed to the perithecial wall and reaching down about 1/3 of the perithecium, and ellipsoid ascospores (to *c.* 35 µm long); only known from the type locality in the Eastern Alps (Italy). – **It**: Ven.

***Verrucaria despecta* Servít**

L # – Subs.: cal – Alt.: 2 – Note: a calcicolous species with a whitish, epilithic, rimose to areolate thallus forming patches to 3 cm in diam. (the areoles with a minutely verruculose surface), hemispherically protruding ascomata (to 0.2 mm in diam.) mostly arising inbetween or at the edges of the areoles, an involucrellum adpressed to the perithecial wall and reaching down about half of the perithecia, and oblong to ellipsoid ascospores (to *c.* 15 µm long); only known from the type locality at the base of the Western Alps (Italy). – **It**: Lig.

***Verrucaria diaphragmata* Zschacke**

L # – Subs.: sax – Alt.: 4 – Note: a species with a thin, epilithic, grey, continuous thallus, hemispherically protruding, black, ascomata (*c.* 0.2 mm in diam.) with a rough surface, an involucrellum tightly adpressed to the perithecial wall and reaching down to the base, and ellipsoid, simple ascospores (20–22 × 9–10 μm); on small (calcareous?) stones in the shade of closed subalpine forests; only known from the type locality in the Eastern Alps (Switzerland). – **Sw**: GR.

***Verrucaria dilacerata* Zschacke**

L # – Subs.: cal – Alt.: 4 – Note: a species resembling a poorly developed *V. nigrescens*, with an epilithic, black-brown, rimose to areolate thallus, hemispherically protruding, black ascomata (to *c.* 0.25 mm in diam.), an involucrellum tightly adpressed to the perithecial wall and reaching down to the base, 8-spored asci, and ellipsoid, simple ascospores (11–18 × 5–7 μm); only known from the type locality in the Eastern Alps (Switzerland), on dolomite. – **Sw**: GR.

***Verrucaria dinarica* Zahlbr.**

L – Subs.: cal – Alt.: 3–5 – Note: closely related to *V. caerulea*, but with a mainly endolithic, uncracked, grey thallus, almost entirely immersed perithecia (to *c.* 0.3 mm in diam.) with a wall carbonised throughout and without involucrellum, and oblong to ellipsoid ascospores (to *c.* 15 µm long); on limestone, known from scattered records in Southern Europe, including the Alps, but rare. – **Fr**: HSav. **Sl**: SlA.

***Verrucaria discernenda* Zschacke**

Syn.: *Amphoridium caesiopsilum* *sensu* Arnold *non* (Anzi) Arnold *nec Verrucaria caesiopsila* Anzi

L # – Subs.: cal – Alt.: 3–4 – Note: a species resembling in habitus *V. caesiopsila* *sensu* Arnold, but with larger ascospores, with an endolithic thallus indicated by whitish-grey patches, ascomata (to 0.3 mm in diam.) deeply immersed in the rock with only the hardly protruding black ostioles visible, more or less spherical in section, the wall dark brown throughout, 8-spored asci, and broadly ellipsoid, simple ascospores (22–30 × 14–18 μm); on carbonatic rocks (*e.g.* dolomite), with a few records from Central Europe, in the Alps only known from the type locality (Italy). – **It**: TAA.

***Verrucaria dolomitica* (A. Massal.) Kremp.**

Syn.: *Amphoridium dolomiticum* A. Massal.

L – Subs.: cal – Alt.: 2–5 – Note: on calcareous rocks and pebbles, usually near the ground. A species belonging to the poorly understood complex of *V. hochstetteri*, differing from *V. foveolata* in the small apical involucrellum. – **Au**: V, T, S, St, O, N. **Fr**: Drô, Vau. **It**: Ven, Lomb.

***Verrucaria dolosa* Hepp**

Syn.: ?*Verrucaria krempelhuberi* Lindau, Verrucaria mutabilis *auct. p.p. non* Leight.

L – Subs.: cal, sil, int – Alt.: 2–5 – Note: a probably holarctic early coloniser of small pebbles near the ground, both on calcareous and base-rich siliceous rocks, in sheltered situations such as in open woodlands and in moist habitats by watercourses, *e.g.* in the splash zone. The species is related to *V. hydrophila*; widespread throughout the siliceous Alps. – **Au**: ?V, T, S, K, St, O, N, B. **Ge**: OB. **Sw**: BE, GR, LU, SZ, TI, VS. **Fr**: AHP, AMa, Drô, HSav, Var, Vau. **It**: Frl, Ven, TAA, Lomb, Piem, VA, Lig. **Sl**: SlA.

***Verrucaria elaeina* Borrer**

Syn.: *Thelidium elaeinum* (Borrer) Mudd

L – Subs.: cal, sil – Alt.: 3 – Note: a long-forgotten species that seems to be quite common in the British Isles. It grows on shaded limestone, concrete, siliceous rocks and brick, in woodlands or beneath herbaceous vegetation, in natural habitats or on wasteground, in gardens or on damp walls, being characteristic of weakly calcareous rocks in shade; perhaps more widespread in the Alps. – **Ge**: Schw. **Sw**: SZ. **It**: Lomb.

***Verrucaria elaeomelaena* (A. Massal.) Anzi**

Syn.: *Lithocia elaeomelaena* A. Massal., *Verrucaria degenerascens* Nyl. *ex* A.L. Sm., *Verrucaria jurana* Zschacke

L – Subs.: sil-aqu, cal-aqu – Alt.: 2–5 – Note: a cool-temperate to boreal-montane, perhaps circumpolar species, almost perennially submerged in cold montane to alpine creeks, emerging only in very shaded situations; perhaps more widespread in the Alps. In Northern Europe this name was often used for *V. funckii* (Spreng.) Zahlbr. Based on the currently available data from North of the Alps, *V. elaeomelaena* *s.str.* appears to be restricted to limestone, but it cannot be separated by morphology alone from several other unnamed lineages within the aggregate which grow on calcareous and siliceous substrata alike, especially in deep shade. – **Au**: T, S, K, St, O, N. **Ge**: OB. **Sw**: BE, GR, VD, VS. **Fr**: HAl, AMa, Drô, Var, Vau. **It**: Ven, TAA. **Sl**: SlA, Tg.

***Verrucaria elevata* (Nyl.) Zschacke**

Syn.: Lithocia viridula (Schrad.) A. Massal. var. elevata Nyl.

L # – Subs.: sil – Alt.: 2–3 – Note: most frequent on calciferous schists, superficially resembling *V. macrostoma*; reported from a few localities in Central Europe and the Alps. – **Au**: K, St, N.

***Verrucaria endocarpoides* Servít**

L # – Subs.: sil, cal – Alt.: 2–4 – Note: an apparently widespread taxon belonging to a group of species with a thick, brown, areolate thallus, which still needs revision. It has been reported from Italy, Austria, Slovakia and North America. – **Au**: T, O, N. **It**: Frl.

***Verrucaria endolithea* Zschacke**

L # – Subs.: cal – Alt.: 4–5 – Note: a species with a mainly endolithic to thin, continuous thallus indicated by grey patches, numerous minute ascomata (to 0.25 mm in diam.) forming hemispherically protruding black warts, a tightly adpressed involucrellum, 8-spored asci, and ellipsoid, simple ascospores (11–16 × 7–9 μm); on calcareous rocks in the lower alpine belt, only known from the type locality in the Eastern Alps (Switzerland). – **Sw**: GR.

***Verrucaria epixylon* Zschacke**

L – Subs.: xyl – Alt.: 3 – Note: a species with a verruculose-areolate thallus, hemispherically protruding perithecia (to *c.* 0.3 mm in diam.) with an entire involucrellum, and broadly ellipsoid ascospores (to *c.* 12 µm long); on wooden fences in rural environments, only known from the Eastern Alps (Austria). – **Au**: T.

***Verrucaria erubescens* Zschacke**

L # – Subs.: cal-aqu – Alt.: 4 – Note: a species resembling *V. csernaensis*, with a thin thallus which is grey-green when wet, turning reddish and rimulose when dry, wart-like, protruding perithecia (to *c.* 0.5 mm in diam.) and larger ascospores (to *c.* 30 µm long); on temporarily submerged calcareous rocks in streams, only known with certainty from the type locality in Switzerland. – **Au**: ?V. **Sw**: GR.

***Verrucaria euganea* Trevis.**

Syn.: Verrucaria weddellii *auct. non* Servít

L – Subs.: cal – Alt.: 2–3 – Note: an early coloniser of walls (mortar, brick, cement, limestone) in urban settlements; related to *V. macrostoma*, but differing in several important morphological characters; apparently most frequent in the Southern and Western Alps, but probably more widespread. – **Au**: N. **Fr**: AHP, AMa, Drô, Isè, Var, Vau. **It**: Ven, TAA, Piem, Lig.

***Verrucaria eusebii* Servít**

Syn.: *Verrucaria amylacea* Hepp *nom.illeg. non* Ach.

L – Subs.: sil, cal – Alt.: 1–4 – Note: on limestone and dolomite in sheltered situations protected from rain, *e.g.* with *Caloplaca cirrochroa*; perhaps regionally overlooked, and more widespread in the Alps. – **Au**: ?V, T, K, St, O, N. **Fr**: AMa, Drô, Sav, HSav, Vau. **It**: Ven, Piem.

***Verrucaria ferratensis* Servít**

L # – Subs.: ?sil – Alt.: 2 – Note: a species with a thin, blackish brown, rimose to areolate thallus forming patches of *c.* 1 cm. in diam., surrounded by a subdentritic prothallus and with a carbonised basal layer, ascomata immersed in verrucae (to 0.25 mm in diam.), involucrellum adpressed to the perithecial wall in the upper part, somewhat spreading below and fusing with the basal thalline layer, ascospores oblong to ellipsoid (to 25 µm long); on roof tiles, only known from the type locality at the base of the Western Alps (France). – **Fr**: AMa.

***Verrucaria finitima* Breuss & F. Berger**

L – Subs.: cal – Alt.: 3–5 – Note: a recently-described species resembling *V. poeltii*, found above the montane belts in the Alps on hard, exposed limestone rocks with a long snow cover; perhaps more widespread in the Alps. – **Au**: V, K, O, N. **Sw**: SZ. **Fr**: HSav. **It**: Frl.

***Verrucaria fischeri* Müll. Arg.**

Syn.: *Lithocia tristis* A. Massal., *Verrucaria diffracta* Anzi, *Verrucaria tristis* (A. Massal.) Kremp. *non* Hepp

L – Subs.: cal, int – Alt.: 3–6 – Note: on steeply inclined faces of compact limestone and dolomite in open habitats, mostly above treeline. Most records should be checked against the very similar *V. finitima* and *V. poeltii*. The species does not belong to *Verrucaria* and seems to be related to *Staurothele*. – **Au**: V, T, S, K, St, O, N. **Ge**: Ge. **Sw**: BE, GR, LU, VD, VS. **Fr**: AHP, HAl, AMa, Isè, Sav, HSav. **It**: Frl, Ven, TAA, Lomb, Piem, VA, Lig. **Sl**: SlA.

***Verrucaria floerkeana* Dalla Torre & Sarnth.**

L # – Subs.: sil, cal – Alt.: 2–4 – Note: on more or less calciferous rocks, especially on pebbles and small stones in rather sheltered situations. A rather difficult taxon, very similar to *V. dolosa* and often confused with that species. **Au**: T, S, K, St, O, N, B. **Fr**: Isè. **It**: TAA.

***Verrucaria foveolata* (Flörke) A. Massal.**

Syn.: *Amphoridium foveolatum* (Flörke) A. Massal., Verrucaria schraderi Sommerf. var. foveolata Flörke

L – Subs.: cal – Alt.: 2–5 – Note: an ecologically wide-ranging species of compact limestone and dolomite, found both on the top of large boulders and on small pebbles near the ground. It belongs to the poorly understood complex of *V. hochstetteri*. – **Au**: V, T, K, St, O. **Ge**: OB. **Sw**: BE, GR, TI, UW. **Fr**: AHP, Isè, Sav, Var, Vau. **It**: Frl, Ven, TAA, Lomb, Piem, VA, Lig. **Sl**: SlA, Tg.

***Verrucaria fraudulosa* Nyl.**

Syn.: Verrucaria lecideoides (A. Massal.) Trevis. var. fraudulosa (Nyl.) Clauzade & Cl. Roux

L – Subs.: cal – Alt.: 4–5 – Note: in the Alps on weakly to strongly calcareous rocks from the subalpine to the alpine belt; according to Cl. Roux a taxon of the *Verruculopsis lecideoides*-aggregate. – **Au**: S, O. **Fr**: Isè, Sav.

***Verrucaria funckianasensu* Servít**

Syn.: *Lithoicea funckii* „A. Massal.“ (1853: 143, *nom. nud.*!, 1854: 23) *non Verrucaria funckii* (Spreng.) Zahlbr.

L # – Subs.: cal – Alt.: 2 – Note: a calcicolous species with a thin, brownish – to greenish-black, spreading, rimose to areolate thallus, the basal layer brown-black or lacking, ascomata hemispherically protruding (*c.* 0.1 mm in diam.), involucrellum adpressed to the perithecial wall reaching down to the base and fusing with the basal layer, ascospores oblong to ellipsoid (to *c.* 25 µm long); only known from the Eastern Alps (Italy). – **It**: Ven.

***Verrucaria funckii* (Spreng.) Zahlbr.**

Syn.: *Pyrenula funckii* Spreng., Verrucaria elaeomelaena *auct. non* (A. Massal.) Anzi, *Verrucaria silicea* Servít, *Verrucaria silicicola* (Zschacke) Servít

L – Subs.: sil-aqu, sil – Alt.: 2–5 – Note: among freshwater Verrucariaceae, this is one of the few species which are usually found in permanently submerged conditions, more rarely in the splash zone of water courses or on deeply shaded stream banks, always on siliceous substrata. It is a typical element of springs and clear headwaters, where it can dominate the benthic community; probably much more widespread in the Alps. – **Au**: V, T, S, K, St. **Ge**: OB. **Sw**: GR, UR. **Fr**: HAl, AMa, Sav. **It**: TAA. **Sl**: SlA.

***Verrucaria furfuracea* (B. de Lesd.) Breuss**

Syn.: Verrucaria macrostoma DC. f. furfuracea B. de Lesd., Verrucaria macrostoma DC. var. imbricum Garov., Verrucaria tectorum *auct. p.p*.

L – Substrata: cal – Bioclimatic belt: 1–2 – Note: mainly on man-made substrata, including mortar walls, on steeply inclined faces; frequently confused with *V. tectorum*, which is isidiate and not sorediate, and has a thinner thallus (see Roux et al. 2014); certainly more widespread in the Alps, at low elevations. – **Au**: K, St, O, N. **Fr**: AHP, AMa, Var, Vau. **It**: Frl.

***Verrucaria fusca* Pers.**

L # – Subs.: cal, int – Alt.: 3–4 – Note: an often misunderstood taxon, closely related to or even identical with *V. nigrescens*, with a thin, olive-brown, granulose thallus, and up to *c.* 20 µm long ascospores. – **Au**: O. **Ge**: Ge. **Sw**: SZ.

***Verrucaria fuscoatroides* Servít**

L # – Subs.: cal – Alt.: 3–4 – Note: an apparently rather widespread, but poorly understood species described from Germany and also reported from several localities in the Alps, mainly on calcareous rocks. – **Au**: V, K, O, N. **Sw**: SZ. **Fr**: AHP, AMa.

***Verrucaria fusconigrescens* Nyl.**

Syn.: *Lithocia fusconigrescens* (Nyl.) Flagey

L # – Subs.: sil, int – Alt.: 2 – Note: a species with an epilithic, greyish-brown to brown-black, rimulose to subareolate thallus, slightly to hemispherically protruding ascomata, the wall subhyaline in the lower half, involucrellum reaching down to the base of the perithecium, with a carbonised outer layer and brownish inner layer, and oblong ascospores (to *c.* 25 µm long); on siliceous substrata at low elevations; reported from several localities in SW Europe, including the Western Alps (France). – **Fr**: AMa, Isè, Vau.

***Verrucaria fuscovelutina* Servít**

L # – Subs.: cal – Alt.: 3 – Note: a calcicolous species with a thin, spreading, brown, rimose to subareolate thallus, ascomata (to 0.4 mm in diam.) in conical warts, a carbonised involucrellum tightly adpressed to the exciple in the upper half but indistinct further downwards, and ellipsoid ascospores (to *c.* 25 µm long); reported from several localities in Southern and Central Europe, with a single record from the Eastern Alps (Austria). – **Au**: N.

***Verrucaria galactinella* Servít**

Syn.: *Amphoridium galactinum* A. Massal., *Verrucaria galactina* (A. Massal.) Trevis. *non* Ach.

L # – Subs.: cal – Alt.: 2–3 – Note: a calcicolous species with a mainly endolithic, whitish, subfarinose thallus intersected by dark prothallus lines, immersed ascomata (to *c.* 0.3 mm in diam.), an involucrellum forming a small superficial shield but lacking radial cracks, and ellipsoid ascospores (to *c.* 30 µm long); only known from the base of the SE Alps (Italy). – **It**: Ven.

***Verrucaria geomelaena* Anzi**

L # – Subs.: ter-cal – Alt.: 3–4 – Note: a species with a very thin, spreading, subgelatinous thallus, very small, spherical perithecia immersed only with the base, a non-amyloid hymenium with free paraphyses, 6–8-spored asci, and simple, hyaline, oblong ascospores measuring *c.* 18.9 × 6.8 µm; only known from the type collection, on calciferous soil between 1,820 and 2,100 m. The type material is well worthy of further study. – **It**: Lomb.

***Verrucaria geophila* Zahlbr. *nom.illeg. non* Nyl.**

L – Subs.: ter-sil – Alt.: 2–3 – Note: a rare species of slightly calciferous soil in dry Mediterranean grasslands, including those at the base of the Western Alps. The name is illegitimate and would require conservation. – **Au**: St. **Fr**: AMa.

***Verrucaria glacialis* Hepp *non* (Bagl. & Carestia) Stizenb.**

L # – Subs.: cal – Alt.: 4 – Note: a long-forgotten calcicolous species with broadly ellipsoid ascospores (to 32 µm long), only known from the type locality in the Eastern Alps (Austria). – **Au**: K.

***Verrucaria glarensis* Servít**

L # – Subs.: cal – Alt.: 3 – Note: a calcicolous species resembling in habitus *V. tristis*, but with smaller fruiting bodies, with a spreading, epilithic, rimose to areolate, brownish thallus, black and partly protruding, spherical ascomata (0.25–0.5 mm in diam.), an involucrellum tightly adpressed to the wall and reaching down about two third of the perithecium, the wall only weakly pigmented in the lower half, 8-spored asci, and oblong to ellipsoid, simple ascospores (17–20 × 6–7 μm); only known from the type locality in the Western Alps (Switzerland). – **Sw**: GL.

***Verrucaria glaucodes* Nyl.**

L – Subs.: cal – Alt.: 2 – Note: a calcicolous species resembling *V. pinguicula*, but with a thin, rimose to subareolate, whitish-greenish thallus with a bluish tinge, semi-immersed ascomata (to *c.* 0.2 mm in diam.) with hardly pigmented perithecial wall, an adpressed involucrellum reaching down about half the perithecium, and ellipsoid ascospores (to *c.* 15 µm long); most frequent in the western part of continental Europe, including the Western Alps (France). – **Fr**: AHP, AMa, Isè, Var, Vau.

***Verrucaria glauconephela* Nyl.**

L # – Subs.: cal – Alt.: 2–3 – Note: closely related to and perhaps a synonym of *Parabagliettoa cyanea*, with an endolithic thallus indicated by patches of a whitish-greenish colour with a bluish tinge, semi-immersed ascomata (to *c.* 0.15 mm in diam.), an adpressed involucrellum reaching down about half the perithecium, and ellipsoid ascospores (to *c.* 15 µm long); on calcareous rocks at low elevations in continental Europe (based on a type from Hungary), with a few records from the Western Alps (France). – **Fr**: AMa, Var.

***Verrucaria glaucovirens* Grummann**

Syn.: *Verrucaria virens* Nyl. *non* Wallr.

L # – Subs.: cal – Alt.: 3 – Note: a species resembling *V. obfuscans*, with a greyish to greenish-brown thallus with rough areoles, immersed perithecia without involucrellum, and ellipsoid ascospores (to *c.* 20 µm long); on calcareous rocks and walls at low elevations; widespread throughout Europe, but with a few scattered records from the Alps. – **Au**: O. **Sw**: ?VS.

***Verrucaria globulans* Zahlbr.**

L # – Subs.: cal – Alt.: 4 – Note: a calcicolous species with a thin, brownish-grey thallus forming confluent patches limited by dark prothallus lines, hemispherically protruding, black, glossy ascomata (to *c.* 0.8 mm in diam.) with an adpressed involucrellum reaching down one third of the perithecium, and subglobose ascospores (to *c.* 12 µm long); only known from the type locality in the Eastern Alps (Austria). – **Au**: N.

***Verrucaria glowackii* Servít**

L # – Subs.: sil – Alt.: mon – Note: a silicicolous species resembling in habitus *V. papillosa*, with a spreading, epilithic, thin, verrucose to areolate, yellowish to brownish thallus, the black ascomata (to *c.* 0.3 mm in diam.) somewhat protruding with depressed ostioles, an involucrellum reaching down to the base of the perithecium, and ellipsoid to oblong ascospores (23–26 × 10–14 μm); only known from the type locality in the Eastern Alps (Slovenia). – **Sl**: SlA.

***Verrucaria gorzegnoensis* Servít**

L # – Subs.: cal – Alt.: 2 – Note: a species with a thin, continuous to partly rimulose, whitish thallus forming patches to 3 cm in diam., slightly protruding ascomata (to 0.3 mm in diam.), an adpressed involucrellum reaching down to the base of the perithecia (to partly entire), and ellipsoid ascospores (to *c.* 35 µm long); on calcareous schists, only known from the type locality in the Western Alps (Italy). – **It**: Piem.

***Verrucaria gudbrandsdalensis* Zschacke *ex* H. Magn.**

L – Subs.: sil, int – Alt.: 3 – Note: a species with a mainly continuous, partly thicker and subrimose, whitish grey thallus, ascomata (to 0.3 mm in diam.) covered by a conspicuous involucrellum reaching far down at the flanks of the perithecium, ascospores to *c.* 25 µm long; on siliceous slate and similar calcium-poor substrates; widespread in Europe but rarely reported, with a few records from the Eastern Alps (Austria). – **Au**: O, N.

***Verrucaria gypsophila* Zschacke**

L # – Subs.: cal – Alt.: 4 – Note: a species resembling *V. brachyspora*, with a thin, epilithic, continuous thallus of a greyish-brownish colour with a rose tinge, hemispherically protruding ascomata (to *c.* 0.2 mm in diam.) with involucrellum reaching down about two thirds of the perithecium, and obovoid ascospores (to *c.* 20 µm long); based on a type from Northern Germany, on gypsum; in the study area known from a single locality in the Eastern Alps (Austria), a record which however needs confirmation. – **Au**: ?St.

***Verrucaria hegetschweileri* Körb. *ex* Nyl. (*illeg* .) *non* (Naegeli *ex* Hepp) Garov.**

L # – Subs.: cor, ?xyl – Alt.: 2 – Note: a species with a very thin, grey thallus, ascomata to *c.* 0.2 mm in diam., and ellipsoid ascospores (to *c.* 15 µm long); on bark (and wood?) at the base of trunks of broad-leaved trees; so far recorded from a few localities in the Alps. – **Fr**: AMa.

***Verrucaria hemisphaerica* Servít**

L # – Subs.: sil – Alt.: mon – Note: a species resembling in habitus *V. nigresccens*, with a spreading, epilithic, rimose to areolate, brown-black thallus, the black ascomata (to *c.* 0.3 mm in diam.) hemispherically protruding, with an adpressed involucrellum reaching down to the base of the perithecia, and oblong to ellipsoid ascospores (20–24 × 7–10 μm); on porphyric rocks, only known from the type locality in the Eastern Alps (Slovenia). – **Sl**: SlA.

***Verrucaria hilitzeriana* Servít**

L # – Subs.: cal – Alt.: 2 – Note: a calcicolous species with a thin, spreading, brown, rimose to areolate thallus (areoles often with a black rim), black, naked, hemispherically protruding ascomata inbetween the areoles (to 0.2 mm in diam.), an involucrellum adpressed to the perithecial wall reaching down to the base, and oblong to ellipsoid ascospores (to 20 µm long); known from the type locality at the base of the Western Alps (France), and from Eastern Liguria (outside the Alps). – **Fr**: AMa.

**Verrucaria hochstetteri Fr. subsp. hochstetteri var. hochstetteri**

Syn.: Amphoridium hiascens *auct. non* (Ach.) A. Massal., *Amphoridium hochstetteri* (Fr.) A. Massal., Amphoridium hochstetteri (Fr.) A. Massal. f. obtecta Arnold [non *Verrucaria obtecta* Müll. Arg.], Verrucaria hiascens *auct. non* (Ach.) Hepp, Verrucaria hochstetteri Fr. f. papularis Rehm *ex* Servít; incl. Verrucaria hochstetteri Fr. var. crustosa (Arnold) Zahlbr.

L – Subs.: cal, cal-aqu – Alt.: 2–5 – Note: a variable species found on steeply inclined surfaces of compact limestone and dolomite in sheltered situations; widespread throughout the Alps. – **Au**: V, T, S, K, St, O, N. **Ge**: OB. **Sw**: BE, GR, LU, SZ, TI, UR, UW, VD, VS. **Fr**: AHP, HAl, AMa, Drô, Isè, Sav, HSav, Var, Vau. **It**: Frl, Ven, TAA, Lomb, Piem, VA, Lig. **Sl**: SlA.

**Verrucaria hochstetteri Fr. subsp. hochstetteri var. obtecta (Müll. Arg.) Clauzade & Cl. Roux**

Syn.: Verrucaria hochstetteri Fr. var. obtecta (Müll. Arg.) Clauzade & Cl. Roux, *Verrucaria obtecta* Müll. Arg.

L # – Subs.: cal – Alt.: 2–5 – Note: a variety with an endolithic thallus, entirely immersed ascomata detectable only by the dots of the ostioles, lacking both an involucrellum and a protruding rim, and ovoid ascospores (to *c.* 30 µm long); based on type from Egypt, the conspecificity of Central European specimens needs confirmation; the distribution in the Alps is difficult to interpret, since the variety was not always distinguished. – **Au**: ?V, ?T, St. **Fr**: AHP, AMa, Drô, Isè, Sav, HSav, Var, Vau.

**Verrucaria hochstetteri Fr. subsp. rosaeformis Cl. Roux**

Syn.: Verrucaria hochstetteri Fr. var. rosaeformis (Asta, Clauzade & Cl. Roux) Clauzade & Cl. Roux *comb. inval.*, Verrucaria integra (Nyl.) Nyl. var. rosaeformis Asta, Clauzade & Cl. Roux [invalidly published, ICN Art. 40.1. + 8]

L – Subs.: cal – Alt.: 4–5 – Note: a calcicolous taxon peculiar in having a circum-ostiolar involucrellum shaped as a 4-lobed rosette; so far only known with certainty from the Western Alps (France). – **Au**: ?Au. **Fr**: AHP, AMa, Sav, HSav.

***Verrucaria hydrela* Ach.**

Syn.: *Verrucaria denudata* Zschacke, *Verrucaria hydrophila* Orange

L – Subs.: sil-aqu, sil – Alt.: 2–5 – Note: on siliceous pebbles in humid-shaded situations (*e.g.* in open woodlands), sometimes on boulders in creeks, but never submerged for long periods, usually in upland areas but rarely reaching above treeline. Several records need confirmation. For nomenclatural matters, we partly follow Roux et al. (2014: 1246), and partly the suggestion by Thüs (see Nimis 2016) to use the name *V. hydrophila* Orange only for sequenced material with an ITS sequence that fits the one published for the type, which also has a subgelatinous thallus and a widely spreading involucrellum. – **Au**: ?V, T, S, K, St, N. **Sw**: BE, GR, UR, VS. **Fr**: AHP, HAl, AMa, Sav, Var. **It**: Ven, TAA, Lomb, Piem. **Sl**: SlA.

***Verrucaria illinoisensis* Servít**

L – Subs.: cal – Alt.: 3 – Note: a calcicolous species with an epilithic, greyish-white, rimose to areolate thallus, hemispherically protruding, immersed ascomata (*c.* 0.2 mm in diam.), involucrellum spreading and reaching down about half the perithecium, and oblong to ellipsoid ascospores (to *c.* 20 µm long; specimens from NE Europe reported to *c.* 25 µm long); based on a type from North America, and also reported for NE Europe, with a single reord from the Eastern Alps (Austria). – **Au**: O.

***Verrucaria imitatoria* Servít**

L # – Subs.: ?sil – Alt.: 2 – Note: a species resembling *V. rupestris*, with a thin, epilithic, spreading, greyish, rimose to areolate thallus, hemispherically protruding ascomata (to 0.25 mm in diam.), an adpressed involucrellum reaching down almost to the base of the perithecium, and ellipsoid ascospores (to 35 µm long); only known from the type locality at the base of the Western Alps (Italy), on sandstone. – **It**: Lig.

***Verrucaria incertula* (Arnold) Zahlbr.**

Syn.: *Amphoridium incertulum* Arnold

L – Subs.: cal – Alt.: 3 – Note: on very compact calcareous rocks subject to periodical water seepage; related to *V. saprophila*, differing in the smaller perithecia and spores (see Roux et al. 2014: 1247). – **Fr**: AMa. **It**: TAA.

***Verrucaria incompta* Servít**

L # – Subs.: cal – Alt.: 2 – Note: a calcicolous species with a whitish-grey, spreading, partly endolithic thallus densely covered by minute granules (to 20 µm in diam.), semi-immersed ascomata (to 0.2 mm in diam.) laterally covered with a thin thalline layer, an involucrellum adpressed to the perithecial wall and reaching down less than half the perithecia, and oblong ascospores (less than 20 µm long); only known from the type locality at the base of the Western Alps (Italy). – **It**: Lig.

***Verrucaria inordinata* (Servít) ined. (provisionally placed here, ICN Art. 36.1b)**

Syn.: *Involucrothele inordinata* Servít

L # – Subs.: sil – Alt.: 2 – Note: a silicicolous species with a grey, epilithic, rimose to areolate thallus either spreading or forming patches, the areoles with an uneven surface or minutely verrucose, with black granules (to 100 µm in diam.), hemispherically protruding ascomata (to 0.3 mm in diam.), an involucrellum attached to the perithecial wall and reaching down about half of the perithecium, and oblong to ellipsoid, simple ascospores with *c.* 10% one-septate intermixed (to *c.* 20 µm long); only known from the type locality at the base of the Western Alps (Italy). – **It**: Lig.

***Verrucaria inornata* Servít**

L # – Subs.: cal – Alt.: 3 – Note: on rather shaded and moist surfaces of calciferous rocks, this species is similar to *V. memnonia*, differing in the larger spores and the pale excipulum. – **Au**: O. **It**: Lig.

***Verrucaria italica* (B. de Lesd.) Servít**

Syn. *Dermatocarpon italicum* (B. de Lesd.) Zahlbr., *Endopyrenium italicum* B. de Lesd.

L # – Subs.: cal – Alt.: 1 – Note: a calcicolous species characterised by a greenish grey, areolate thallus, perithecia with a dimidiate involucrellum, and ellipsoid to oblong-ellipsoid ascospores measuring *c.* 6 × 15–17 µm; only known from the type collection near Spotorno (Italy). – **It**: Lig.

***Verrucaria jodophila* Servít**

L # – Subs.: cal – Alt.: 4 – Note: a species related to *V. caerulea*, with an epilithic, dark lead grey, rimose to areolate thallus forming patches to 1 cm (!) delimited by black prothallus lines, immersed and hardly protruding ascomata (to 0.15 mm in diam.), an adpressed involucrellum reaching down to the base of the perithecium, and oblong to narrowly ellipsoid ascospores (to 20 µm long); only known from the type locality in the Eastern Alps (Italy), on dolomite. – **It**: TAA.

***Verrucaria lacerata* Servít**

L – Subs.: cal – Alt.: 2–3 – Note: a calcicolouos species with a mainly endolithic, brownish grey thallus with small black patches and black prothallus lines, semi-immersed ascomata (to *c.* 0.4 mm in diam.), a slightly spreading involucrellum reaching down about half of the perithecium, and ellipsoid to oblong ascospores (to *c.* 35 µm long); reported from scattered localities in Central Europe, including the Eastern Alps (Austria). – **Au**: K, O, N. **Sl**: SlA.

***Verrucaria langhensis* Servít**

L # – Subs.: cal, int – Alt.: 2 – Note: a species with a thin, brown, spreading, rimose to areolate thallus (fruiting areolae convex, to 0.8 mm in diam.) with a thick brown-black basal layer, hemispherically protruding ascomata (to 0.3 mm in diam.) covered by a thin thalline layer, involucrellum adpressed to the perithecial wall reaching down to the base and fusing with the basal layer, and oblong to ellipsoid ascospores (exceeding 30 µm in length); on calcareous schists, only known from the type locality in the Western Alps (Italy). – **It**: Piem.

***Verrucaria latebrosa* Körb.**

L # – Subs.: sil-aqu, cal-aqu, xyl – Alt.: 3–5 – Note: a freshwater species periodically submerged on hard siliceous rocks, occasionally also on calcareous substrata. This species was included in *V. aethiobola* by Orange as a member of a “collective species”, and its relation to *V. anziana* remains to be clarified. No material from the type locality has ever been sequenced, which may be necessary to select a sequenced epiptype in order to fix the ambiguity in the use of this name. See also note on *V. anziana*. – **Au**: T, S, K, St. **Sw**: BE, GR, VS. **Fr**: HAl, AMa, Sav, HSav. **It**: Ven, TAA, Lomb, Piem.

***Verrucaria latebrosoides* Servít**

L # – Subs.: sil – Alt.: ?3 – Note: a species with a spreading, olive-brown to blackish-brown, rimose to areolate thallus with a carbonised basal layer, hemispherically protruding ascomata (to *c.* 0.2 mm in diam.), an involucrellum adpressed to the perithecial wall and reaching down to the base, and ellipsoid ascospores (to 25 µm long); on porphyric rocks, only known from the type locality in the Eastern Alps (Italy). – **It**: TAA.

***Verrucaria licentiosa* (Servít) ined. (provisionally placed here, ICN Art. 36.1b)**

Syn.: *Involucrocarpon licentiosum* Servít

L # – Subs.: cal – Alt.: 2–3 – Note: a calcicolous species of unclear relationship, with a bluish grey, epilithic, areolate to verrucose thallus forming patches to *c.* 2 cm in diam., protruding ascomata (to 0.3 mm in diam.) not rarely arising inbetween areoles, an entire involucrellum, and oblong ascospores (to *c.* 20 µm long); only known from the type locality in the Western Alps (Italy). – **It**: Piem.

***Verrucaria lignorum* Servít**

L # – Subs.: xyl, cor – Alt.: 3 – Note: a species with a blackish-brown, rimose to verruculose-areolate thallus forming patches up to 5 cm in diam., a basal layer with dark maculae, immersed ascomata (to *c.* 0.2 mm in diam.), from above hardly discernable from thalline warts, an involucrellum adpressed to the perithecial wall reaching down to the base and fusing with the basal layer, and ellipsoid ascospores (to 25 µm long); on wood, rarely bark; rare throughout Central Europe, including the Eastern Alps (Austria). – **Au**: K.

***Verrucaria lignyodes* Harm. *ex* Crozals**

L # – Subs.: ?sil-aqu – Alt.: 3 – Note: a species of unclear relationship, with an indistinct thallus, ascomata immersed in the rock (to *c.* 0.3 mm in diam.), interascal filaments apparently persistent and with some ramifications, and broadly ovoid ascospores (to 27 µm long); on pebbles in a creek, only known from the type locality in the Western Alps (France). – **Fr**: HSav.

***Verrucaria ligurica* (Servít) ined. (provisionally placed here, ICN Art. 36.1b)**

Syn.: *Involucrothele ligurica* Servít non *Verrucaria ligurica* Zschacke quid est *Hydropunctaria ligurica* (Zschacke) Cl. Roux

L # – Subs.: ?cal – Alt.: 2 – Note: a species with an olive-coloured, epilithic, rimose to areolate thallus forming patches to 3 cm in diam., the areoles with a verruculose surface, protruding ascomata (to 0,4 mm in diam.), an involucrellum adpressed to the perithecial wall and reaching down to the base of the perithecium, and oblong to ellipsoid, simple ascospores intermixed with *c.* 10–20% 1-septate ones (to *c.* 15 µm long); only known from the type locality at the base of the Western Alps (Italy), on a wall. – **It**: Lig.

***Verrucaria limitatoides* Servít**

L # – Subs.: cal – Alt.: 5 – Note: a calcicolous species with a thin, whitish-grey, spreading thallus, hemispherically protruding ascomata (*c.* 0.2 mm in diam.), a loosely attached involucrellum reaching down about half of the perithecium, and oblong to ellipsoid ascospores (to *c.* 25 µm long); only known from the type locality in the Eastern Alps (Austria). – **Au**: T.

***Verrucaria maas-geesterani* Servít**

L # – Subs.: sil-aqu – Alt.: 3 – Note: a species which is similar, and probably closely related to *V. margacea*, with an epilithic, partly rimose, blackish-brown (black in the wet state) thallus, superficially with small black warts, in section with a carbonised basal layer, ascomata (to 0.3 mm in diam.) black and hemispherically protruding, but with a thin thalline layer in the lower part, involucrellum tightly adpressed to the perithecial wall and reaching down to the base, where it fuses with the basal layer, 8-spored asci, and broadly ellipsoid, simple ascospores (20–28 × 13–16 μm); on submerged siliceous rocks in streams, only known from the type locality in the Western Alps (Switzerland). – **Sw**: TI.

***Verrucaria macrostoma* DC.**

Syn.: *Lithocia macrostoma* (DC.) A. Massal., *Verrucaria thrombioides* A. Massal., Verrucaria viridula *auct. non* (Schrad.) Ach.

L – Subs.: cal – Alt.: 1–5 – Note: an early coloniser of walls (mortar, brick, cement, limestone) in urban areas, more rarely found on calcareous rocks in natural environments, with a wide ecological amplitude, from horizontal to steeply inclined faces visited by birds; widespread throughout the Alps. – **Au**: V, ?T, S, St, O, N. **Sw**: BE, LU, SZ, UW, VD, VS. **Fr**: AHP, HAl, AMa, Drô, Sav, Var, Vau. **It**: Frl, Ven, TAA, Lomb, Piem, VA, Lig.

***Verrucaria maculiformis* Kremp. *nom.illeg. non* Hoffm.**

L – Subs.: cal – Alt.: 2–3 – Note: this species seems to be most frequent in Western and Central Europe, on more or less calcareous pebbles or on bricks, especially in rather shaded situations. Most records require confirmation. The name is not legitimate, being a later homonym of *V. maculiformis* Hoffm. (1796). – **Au**: ?V, ?T, O. **Ge**: OB. **Fr**: AMa, HSav. **It**: Ven, Lomb.

***Verrucaria margacea* (Wahlenb.) Wahlenb.**

Syn.: *Lithocia margacea* (Wahlenb.) A. Massal., *Thelotrema margaceum* Wahlenb., *Verrucaria applanata* Hepp *ex* Zwackh, ?*Verrucaria divergens* Nyl., *Verrucaria filarszkyana* Szatala, *Verrucaria leightonii* Hepp *non* A. Massal., *Verrucaria tiroliensis* Zschacke, ?*Verrucaria vallis-fluelae* Zschacke, *Verrucaria zegonensis* Zschacke

L – Subs.: sil-aqu, cal-aqu – Alt.: 2–5 – Note: an amphibious freshwater lichen of siliceous rocks beside streams and lakes; it prefers constantly inundated and even permanently submerged rocks to those merely in the spray zone. The species, widespread in Scandinavia and also known from the Southern Hemisphere, is widespread throughout the Alps. – **Au**: V, T, S, K, St, O, N. **Sw**: BE, GR, TI, UR, VS. **Fr**: AHP, HAl, AMa, Sav, HSav, Var. **It**: Ven, TAA, Lomb, Piem. **Sl**: SlA.

***Verrucaria mastoidea* (A. Massal.) Trevis.**

Syn.: *Amphoridium mastoideum* A. Massal., Verrucaria hochstetteri Fr. var. mastoidea (A. Massal.) Clauzade & Cl. Roux

L # – Subs.: cal – Alt.: 3–4 – Note: on calciferous rocks; this rather poorly understood species differs from *V. hochstetteri* in the presence of a small involucrellum. – **Au**: V, T, K, St, O, N. **It**: Ven, TAA, Piem.

***Verrucaria mauroides* Schaer.**

Syn.: *Lithocia mauroides* (Schaer.) A. Massal., *Thrombium mauroides* (Schaer.) Zschacke

L # – Subs.: sil, ?cal – Alt.: 3–5 – Note: a species of unclear relationship with a thin, blackish thallus, hemispherically protruding ascomata (to *c.* 0.2 mm in diam.), and an involucrellum reaching down to the base of the perithecium (other microscopical characters not documented); so far only recorded from a few localities in the Alps. – **Au**: ?V. **Sw**: BE, VS. **Fr**: HSav.

***Verrucaria memnonia* (Flot. *ex* Körb.) Arnold**

Syn.: Verrucaria maura Wahlenb. var. memnonia Flot. *ex* Körb.

L – Subs.: sil – Alt.: 3–4 – Note: a species with a thin, epilithic, spreading thallus of a black colour with a bluish to greenish-blue tinge, turning gelatinous when wet, hemispherically protruding ascomata (to 0.3 mm in diam.) with involucrellum reaching down to the base of the perithecium, and obovoid ascospores (to *c.* 15 µm long); mostly on hard siliceous rocks in the shade of montane coniferous forests; widespread in the European mountains, with a few scattered records from the Alps. – **Au**: K, O, N. **Sw**: SZ.

**Verrucaria metzleri Servít var. metzleri**

***L*** # – Subs.: cal – Alt.: 2–3 – Note: a calcicolous species with a spreading, olive-brown, subrimose thallus, semi-immersed ascomata (to *c.* 0.4 mm in diam.) covered by a thin thalline layer, an adpressed involucrellum reaching down about half of the perithecium, and ellipsoid ascospores (to 30 µm long); only known from the type locality in the Western Alps (Switzerland), and from Liguria (outside the Alps). – **Sw**: BE.

**Verrucaria metzleri Servít var. carniolica Servít**

L # – Subs.: cal – Alt.: 3 – Note: a calcicolous variety with an adpressed involucrellum and broadly ellipsoid to subglobose ascospores (to *c.* 25 µm long); only known from the type locality in the Eastern Alps (Slovenia). – **Sl**: SlA.

***Verrucaria mimicrans* Servít**

L # – Subs.: cal – Alt.: 2–3 – Note: described from former Yugoslavia, differing from *V. muralis* in the larger spores, the longer periphyses, and the form of the involucrellum; the total distribution covers wide parts of Europe and the species is also known from North America. It is a pioneer species on more or less calcareous substrata, especially on pebbles and on recently exposed rock surfaces; from the Alps there are a few scattered records only. – **Au**: K, O, N. **Fr**: AMa. **It**: Lig. **Sl**: SlA.

***Verrucaria monacensis* Servít**

L # – Subs.: cal – Alt.: 2–3 – Note: according to Breuss (see Nimis 2016), this species was described on the basis of a sample collected on calcareous pebbles in a scree slope near München, and identified by F. Arnold as *Amphoridium dolomiticum* (=*Verrucaria dolomitica*), from which it differs in several important characters; the species also resembles *Verrucaria muralis*, differing in the rimose thallus. Beside the type collection (the original station is probably lost) the species was reported by Sbarbaro (see Nimis 1993: 754) from Piedmont; since Sbarbaro was in close scientific contact with Servít, it is probable that the latter had identified the Italian samples, which constitute the only record from the Alps. – **It**: Piem.

***Verrucaria mortarii* (Arnold) Arnold *ex* Lamy *nom.illeg. non* Leight.**

Syn.: Amphoridium leightonii Arnold f. mortarii Arnold, *Amphoridium mortarii* (Arnold) Flagey

L # – Subs.: cal – Alt.: 2–5 – Note: a species growing on man-made calciferous substrata, including mortar, especially on walls below the montane belt, closely related to *V. foveolata*; apparently most frequent in the Southern and Western Alps. The name is illegitimate. – **Fr**: HAl, AMa, Isè, Sav, Var, Vau. **It**: Piem.

***Verrucaria muelleriana* Servít**

L # – Subs.: cal – Alt.: 3 – Note: a calcicolous species resembling *V. cinereorufa*, with an endolithic to thin-epilithic, white thallus with a reddish tinge, immersed or only slightly protruding ascomata (to 0.5 mm in diam.), an involucrellum spreading around the ostiole, and ascospores to *c.* 40 µm long; only known from the type locality in the Western Alps (France). – **Fr**: HSav.

***Verrucaria muralis* Ach.**

Syn.: ?*Verrucaria argillacea* Fr., *Verrucaria subdendritica* Servít, *Verrucaria submuralis* Nyl.; incl. *Verrucaria rupestris* Schrad. *non* (Scop.) F.H. Wigg.

**L** – Subs.: cal, sil – Alt.: 1–5 – Note: a widespread early coloniser of pebbles, mortar walls, brick and roofing tiles, with optimum in the submediterranean belt. Some records could refer to *V. rupestris*, which until recently was confused with this species, from which it differs in the endolithic thallus and the immersed perithecia; widespread throughout the Alps. – **Au**: V, T, S, K, St, O, N, B. **Ge**: OB, Schw. **Sw**: BE, GR, LU, SZ, VD. **Fr**: AHP, HAl, AMa, Drô, Isè, Sav, HSav, Var, Vau. **It**: Frl, Ven, TAA, Lomb, Piem, VA. **Sl**: SlA, Tg.

***Verrucaria murina* Leight. *non* (Ach.) Arnold**

Syn.: *Amphoridium myriocarpum* (Hepp *ex* Lönnr.) Servít, Verrucaria murina Leight. f. obscurata Servít, Verrucaria murina Leight. var. pusilla Arnold, *Verrucaria myriocarpa* Hepp *ex* Lönnr., *Verrucaria myriocarpa* Hepp *ex* Lönnr. f. geographica Arnold; incl. *Verrucaria brachyspora* Arnold, *Verrucaria pazientii* A. Massal.

L # – Subs.: cal, int – Alt.: 2–5 – Note: on limestone and dolomite in upland areas. The epithet *murina* has been used for widely different species, and the entire complex is presently under revision. – **Au**: V, T, K, St, O, N. **Ge**: OB. **Sw**: BE, LU, UW, VD. **Fr**: HAl, AMa, Drô, Isè, Sav, HSav, Var, Vau. **It**: Ven, TAA, Lomb.

***Verrucaria murorum* (A. Massal.) Lindau**

Syn.: *Lithocia murorum* (A. Massal.) Arnold, *Thrombium murorum* A. Massal.

L # – Subs.: cal – Alt.: 2–4 – Note: a calcicolous species belonging to the *V. macrostoma*-complex, with scattered records from the Alps. – **Au**: V. **Ge**: Ge. **Sw**: SZ, VS. **Fr**: Drô. **It**: Ven, TAA, Lomb.

***Verrucaria nidulifera* Servít**

L # – Subs.: cal – Alt.: 2–3 – Note: according to Breuss (see Nimis 2016) this species resembles *Parabagliattoa dufourii* (which was growing together with the type material), differing in the less developed involucrellum, the more immersed perithecia and the presence of oil hyphae. It was described on the basis of a sample collected by F. Arnold on dolomite (see Nimis 1993: 755), the ecology being similar to that of *Parabagliettoa dufourii*. – **It**: TAA.

***Verrucaria nigrescens* Pers.**

Syn.: *Lithocia controversa* (A. Massal.) A. Massal., ?*Verrucaria acrotelloides* A. Massal. (*fide* Nimis), *Verrucaria controversa* A. Massal., Verrucaria fusca *auct. non* Pers., *Verrucaria fuscoatra* Pers., Verrucaria nigrescens Pers. var. funckii (A. Massal.) Zwackh *non Verrucaria funckii* (Spreng.) Zahlbr., *Verrucaria protothallina* A. Massal., *Verrucaria umbrina* (Ach.) Ach., *Verrucaria velana* (A. Massal.) Zahlbr.; incl. *Verrucaria confusa* Zschacke, *Verrucaria confusionis* Grummann, Verrucaria nigrescens Pers. var. laeviuscula Nyl.

L – Subs.: cal, int – Alt.: 1–6 – Note: a subcosmopolitan species, one of the most common saxicolous lichens throughout the Alps, found both in urban and natural habitats, with a very wide ecological tolerance; several morphs from natural habitats, however, deserve further study. – **Au**: V, T, S, K, St, O, N, B. **Ge**: OB, Schw. **Sw**: BE, GR, LU, SZ, TI, UR, VD, VS. **Fr**: AHP, HAl, AMa, Drô, Isè, Sav, HSav, Var, Vau. **It**: Frl, Ven, TAA, Lomb, Piem, VA, Lig. **Sl**: SlA, Tg.

***Verrucaria nigrofusca* Servít**

L # – Subs.: cal, sil – Alt.: 3–4 – Note: a species described from the Czech Republic and also reported from France (Maritime Alps), and Liguria (outside the Alps) on both calcareous and basic siliceous rocks. It differs from *V. fuscoatroides* in the smaller perithecia and spores. – **Fr**: AMa.

***Verrucaria nigroumbrina* Servít**

Syn.: Lithoicea nigrescens (Pers.) A. Massal. var. umbrina A. Massal.; incl. Verrucaria nigroumbrina Servít f. acrotella (A. Massal.) Servít

L # – Subs.: cal – Alt.: 2 – Note: a calcicolous species with a thin, greyish-brown to brown, spreading, rimose to areolate thallus, the basal layer brown-black or lacking in young areoles, only slightly protruding ascomata covered by a thin thalline layer, an involucrellum adpressed to the perithecial wall, reaching down to the base and fusing with the basal layer, and oblong to ellipsoid ascospores (to *c.* 25 µm long); only known from the Eastern Alps (Italy), and from Liguria (outside the Alps). – **It**: Ven.

***Verrucaria nivalis* Hepp**

L # – Subs.: cal – Alt.: 3 – Note: a calcicolous species with ovoid ascospores (to 36 µm long), only known from the type locality in the Eastern Alps (Austria). – **Au**: K.

***Verrucaria obfuscans* Nyl.**

L # – Subs.: cal – Alt.: 3 – Note: a species apparently belonging to the *V. nigrescens*-group, with a relatively thick, brown, areolate thallus, immersed ascomata (to *c.* 0.3 mm in diam.) with a hardly pigmented perithecial wall, and ellipsoid to oblong ascospores (to *c.* 20 µm long), based on a type from Paris in France; on walls and other anthropogenic substrates at low elevations; rarely recorded because not always distinguished, known from a few localities in the Alps. – **Au**: ?V. **Fr**: Sav.

***Verrucaria ochrostoma* (Borrer *ex* Leight.) Trevis.**

Syn.: *Sagedia ochrostoma* Borrer *ex* Leight.

L # – Subs.: cal – Alt.: 2–3 – Note: closely related to *V. murorum*, this species, characterised by the superficial thallus and immersed perithecia without an involucrellum, seems to prefer concrete walls and nutrient-enriched, dusty surfaces at relatively low elevations; apparently most frequent in the Southern and Western Alps, but perhaps not distinguished elsewhere. – **Au**: S. **Fr**: AHP, AMa, Drô, Var, Vau. **It**: Ven.

***Verrucaria olivacella* Servít**

L # – Subs.: sil – Alt.: 2 – Note: a species related to or even identical with *V. inaspecta*, with a thin, epilithic, continuous to rimose-areolate, dark olivaceus thallus, hemispherically protruding ascomata (to 0.3 mm in diam.), an involucrellum adpressed to the perithecial wall and reaching the base of the perithecia, and ellipsoid ascospores (to *c.* 25 µm long); on schist, only known from the type locality at the base of the Western Alps (Italy). – **It**: Lig.

***Verrucaria olivascens* Servít**

L # – Subs.: cal – Alt.: 3 – Note: a calcicolous species with a thin, spreading, whitish to greyish, minutely granulose to warty-farinose thallus, hemispherically protruding ascomata (to 0.5 mm in diam.) which are externally covered by a very thin, farinose thalline layer, an adpressed involucrellum reaching down to the base of the perithecium, and oblong to obovoid ascospores (to 35 µm long); rarely reported in Central Europe, including the Eastern Alps (Austria). – **Au**: O, N.

***Thelenella pertusariella* (Nyl.) Vain.**

Syn.: *Microglaena pertusariella* (Nyl.) Norman, *Phlyctis submuriformis* H. Magn., *Verrucaria pertusariella* Nyl.

L – Subs.: cor – Alt.: 3–4 – Note: on the smooth bark of small shrubs in the mountains (*Daphne*, *Rhododendron*, *Salix*, *Sorbus*); from the Alps there are only a few scattered records. – **Au**: V, T. **Sw**: BE, VS.

***Thelenella vezdae* (H. Mayrhofer & Poelt) Coppins & Fryday**

Syn.: *Chromatochlamys vezdae* H. Mayrhofer & Poelt

L – Subs.: xyl, cor – Alt.: 2–4 – Note: an easily overlooked species characterised by minute perithecia with a hyaline wall and submuriform ascospores, whose generic placement is uncertain; on soft decaying wood, occasionally spreading to lignicolous bryophytes in various forest types, with optimum at low to mid-elevations; so far known from Central Europe only, most records being from the Alps, where it was reported from a few scattered localities. – **Au**: K, St. **Fr**: AHP.

***Thelenidia monosporella* Nyl.**

L – Subs.: ter-cal, cal – Alt.: 5 – Note: a species resembling *Thelenella modesta* but smaller, with a very unusual set of characters: delicate interascal filaments, oblong hymenial algae, and 1-spored asci with large, simple ascospores; on calcareous soil, ecology otherwise poorly known; apparently rare, but easy to overlook; the type is from Switzerland (Canton Zürich), near the northern edge of the Alps. – **Fr**: Sav.

***Thelidium absconditum* (Hepp) Rabenh.**

Syn.: Sagedia nigella (Kremp.) Hepp var. abscondita Hepp, *Thelidium rodellense* Lettau

L – Subs.: cal, int – Alt.: 3–5 – Note: on limestone, dolomite, calciferous schists in upland areas; probably more widespread in the Alps. The relationship with *Th. decipiens* remains to be clarified. – **Au**: ?V, T, S, K, St, O, N. **Ge**: OB. **Sw**: BE, GR, VS. **Fr**: AHP, HAl, AMa, Drô, Isè, Sav, HSav. **It**: TAA. **Sl**: SlA.

***Thelidium abstractum* Lettau**

L # – Subs.: cal – Alt.: 5 – Note: a species of the *Th. pyrenophorum*-group with a thin, yellowish-grey, rimose epilithic thallus and smaller protruding ascomata (0.2–0.4 mm in diam.); on calcareous schists at high elevations; so far recorded from a few localities in the Alps. – **Au**: V. **Sw**: BE.

***Thelidium acrotellum* Arnold**

L # – Subs.: cal, sil – Alt.: 2–5 – Note: on more or less calciferous rocks in upland areas; the species is not easy to distinguish from *Th. minutulum*, but has a colourless excipulum and a thin involucrellum; so far recorded from a few localities in the Alps. – **Au**: V, T, St, O. **Fr**: Sav, HSav. **It**: TAA.

***Thelidium aethioboloides* Zschacke**

L – Subs.: cal-aqu – Alt.: 3–5 – Note: an amphibious, but usually not permanently submerged species of calciferous rocks in shaded situations, with optimum in the upper montane belt; perhaps more widespread in the Alps. – **Au**: O. **Ge**: OB, Schw. **Sw**: GR. **Fr**: Sav. **It**: Ven, TAA.

***Thelidium amylaceum* A. Massal.**

L # – Subs.: cal – Alt.: 2–3 (?5) – Note: a species sometimes synonymised with *Th. decipiens*, characterised by a farinose, whitish thallus with a violet to lilac tinge, and minute, bottle-shaped, entirely immersed ascomata which are only visible by the umbilicate ostioles. The type material urgently needs further study, because this is the type species of the genus. – **Sw**: VS. **It**: Ven.

***Thelidium anisosporum* (Müll. Arg.) Zschacke**

Syn.: *Sagedia anisospora* Müll. Arg., *Verrucaria anisosopora* (Müll. Arg.) Stizenb

L # – Subs.: sil – Alt.: 4–5 – Note: a species with a thin, rusty red thallus, immersed perithecia with a black ostiolar region, and narrowly ellipsoid, 1-septate ascospores which are less than 20 µm long; on gneiss in the subalpine to lower alpine belts; only recorded from the Western Alps (Switzerland). – **Sw**: VS.

***Thelidium antonellianum* Bagl. & Carestia**

Syn.: *Involucrothele antonelliana* (Bagl. & Carestia) Servít

L # – Subs.: sil, int – Alt.: 5–6 – Note: a species found on crystalline, weakly calciferous schists above treeline; the type material, from the Italian Alps, was collected at 4,500 m; in the study area only recorded from the Southern and the Western Alps. – **Sw**: VS. **Fr**: AHP, AMa. **It**: TAA, Piem.

***Thelidium aphanes* J. Lahm**

L # – Subs.: cal – Alt.: 2 – Note: a species of the *Th. incavatum*-group with an endolithic, whitish thallus, entirely immersed perithecia with punctiform ostioles, and 3-septate, less than 40 µm long ascospores; on pebbles of limestone, with scattered records in Central Europe, including the Eastern Alps (Austria). – **Au**: O.

***Thelidium arnoldii* Zschacke**

Syn.: *Thelidium bubulcae* *sensu* Arnold *non* A. Massal.

L # – Subs.: cal – Alt.: 3–5 – Note: a species of the *Th. incavatum*-group with somewhat wider, ellipsoid, 1 – to 3-septate ascospores; on limestone, most common at low elevations; the available distributional data are difficult to interpret, because this taxon was not generally accepted. – **Au**: ?V, T, St, O, N.

***Thelidium auruntii* (A. Massal.) Kremp.**

Syn.: *Involucrothele auruntii* (A. Massal.) Servít, *Verrucaria auruntii* A. Massal.

L – Subs.: cal, int – Alt.: 3–5 – Note: differing from *Th. pyrenophorum* in the well-developed, brown thallus and the smaller spores, this species, also known from Scandinavia, grows on limestone, dolomite and calciferous schists in upland areas. – **Au**: ?V, S, O. **Ge**: OB, Schw. **Fr**: AHP, HAl, AMa, Sav, HSav. **It**: Ven, TAA.

***Thelidium austriacum* Zschacke**

Syn.: *Polyblastia austriaca* (Zschacke) Servít

L – Subs.: cal – Alt.: 2 – Note: a species of the *Th. incavatum*-group with smaller ascospores (less than 25 µm long); on limestone, only recorded from the Eastern Alps (Austria). – **Au**: N.

***Thelidium britzelmayrii* (Servít) ined. (provisionally placed here, ICN Art. 36.1b)**

Syn.: *Involucrothele britzelmayrii* Servít 1953 (“britzelmayeri”)

L # – Subs.: sil – Alt.: ? – Note: a species with an epilithic, rimose to areolate, whitish thallus, semi-immersed perithecia in the centre of areoles, with an involucrellum spreading in the upper third, and 1-septate, oblong to ellipsoid ascospores (to *c.* 30 µm long); on siliceous rocks, only known from the type locality in the Eastern Alps (Germany). – **Ge**: Schw.

***Thelidium bubulcae* (A. Massal.) Arnold**

Syn.: *Lithocia bubulcae* A. Massal.

L # – Subs.: cal – Alt.: 2 (?5) – Note: often considered as a synonym of *Th. zwackhii*, this calcicolous species was accepted by Roux et al. (2014); a record from Austria (V) is considered as very dubious by Hafellner and Türk (2016). – **Au**: ?V. **It**: Ven.

***Thelidium circumspersellum* (Nyl.) Zschacke**

Syn.: *Verrucaria circumspersella* Nyl.

L # – Subs.: cal-aqu – Alt.: 2–3 – Note: a calcicolous, aquatic species; hitherto only known from the type locality in Hungary and a single locality in Upper Austria. – **Au**: O.

***Thelidium dactyloideum* Arnold**

L # – Subs.: cal – Alt.: ?3 – Note: a species with a thin, brownish thallus, minute perithecia protruding only with the ostiolar region, a carbonaceus ascomatal wall, and finger-like, 1 – to 3-septate ascospores turning brown with age; on limestone in the montane belt; only known from the Eastern Alps (Slovenia). – **Sl**: Tg.

***Thelidium decipiens* (Hepp *ex* Nyl.) Kremp.**

Syn.: *Amphoridium uberinum* A. Massal., Thelidium amylaceum *auct. non* A. Massal., *Thelidium cinerascens* (Anzi) Servít, *Thelidium coerulescens* Jatta, *Thelidium decipiens* (Hepp *ex* Nyl.) Kremp. var. scrobiculare (Garov.) Arnold, *Thelidium hymenelioides* Körb., *Thelidium immersum* (Leight.) Mudd, *Thelidium leightonii* M. Choisy, *Thelidium pachysporum* Zschacke, *Thelidium scrobiculare* (Garov.) Arnold, *Thelidium thuringiacum* Zschacke, *Thelidium umbrosum* *sensu* Arnold, *Verrucaria immersa* Leight., Verrucaria pyrenophora Ach. var. decipiens Hepp *ex* Nyl., *Verrucaria scrobicularis* Garov.

L – Subs.: cal, int – Alt.: 2–5 – Note: a cool-temperate to arctic-alpine, circumpolar species of calcareous rocks, including large pebbles, in rather sheltered situations, mostly in upland areas, with optimum above treeline; widespread throughout the Alps. – **Au**: V, T, S, K, St, O, N. **Ge**: OB. **Sw**: BE, FR, GR, LU, SZ, UW, VD, VS. **Fr**: AHP, HAl, AMa, Drô, Isè, Sav, HSav, Var, Vau. **It**: Frl, Ven, TAA, Lomb, Piem, VA. **Sl**: SlA, Tg.

***Thelidium decussatum* (Kremp.) Zschacke**

Syn.: *Acrocordia decussata* Kremp., *Involucrothele decussata* (Kremp.) Servít

L # – Subs.: cal – Alt.: 2–3 – Note: a species of the *Th. pyrenophorum*-group with a thin, greyish thallus surrounded and crossed by black lines, somewhat protruding ascomata (less than 0.5 mm in diam.) with an involucrellum reaching down *c.* half the perithecium, and 1-septate ascospores (less than 30 µm long); on sandstone or calcareous rocks, ecology otherwise poorly known; rare in Central Europe, including the Eastern Alps (Austria). – **Au**: St.

***Thelidium dionantense* (Hue) Zschacke**

Syn.: *Verrucaria dionantensis* Hue

L – Subs.: cal – Alt.: 2–5 – Note: on steeply inclined surfaces of calciferous rocks in upland areas; from the Alps there are only a few scattered records. – **Au**: V, T. **Sw**: VS. **Fr**: AHP, AMa.

***Thelidium exile* Arnold**

L – Subs.: cal – Alt.: 3–5 – Note: on more or less calciferous rocks in upland areas. The species was frequently considered as a synonym of *Th. minutulum*, but according to Roux et al. (2014) it differs in having half-protruding perithecia; from the Alps there are only a few scattered records. – **Au**: St. **Fr**: Sav. **It**: TAA.

***Thelidium fontigenum* A. Massal.**

Syn.: *Involucrothele cataractarum* (Hepp) Servít, *Sagedia cataractarum* Hepp, *Thelidium cataractarum* (Hepp) Lönnr.

L – Subs.: sil-aqu, cal-aqu – Alt.: 2–4 – Note: on limestone, dolomite, calcareous sandstone, near creeks and waterfalls in upland areas, but usually below treeline. – **Au**: T, S, K, O, N. **Ge**: Ge. **Sw**: BE. **Fr**: Sav. **It**: Ven, Piem, VA. **Sl**: SlA, Tg.

***Thelidium fumidum* (Nyl.) Hazsl.**

Syn.: *Verrucaria fumida* Nyl.

L # – Subs.: cal – Alt.: 3 – Note: a calcicolous species with a blackish brown, epilithic thallus, a blackish medullary layer, perithecia immersed in thalline warts, with involucrellum reaching down to the base, and 1-septate ascospores (mostly less than 25 µm long); rare throughout Eastern and Central Europe, with a few scattered records from the Alps. – **Au**: St, N. **Fr**: Vau.

***Thelidium gisleri* (Müll. Arg) Zschacke**

Syn.: *Sagedia gisleri* Müll. Arg., *Verrucaria gisleri* (Müll. Arg.) Stizenb.

L – Subs.: cal – Alt.: 4–5 – Note: a species of the *Th. papulare*-group with a very thin, whitish-grey thallus, semi-immersed ascomata (to 0.3 mm in diam.) with an involucrellum reaching down about half the perithecium, and 3-septate, narrowly ellipsoid ascospores (less than 40 µm long); on calcareous rocks at high elevations; known from a few localities in the Alps. – **Au**: St. **Sw**: UR.

***Thelidium globiferum* Servít**

L # – Subs.: cal – Alt.: 3 – Note: a calcicolous species with a minutely granulose, whitish-greyish thallus, immersed perithecia (to 0.6 mm in diam.), and simple to 1-septate, broadly ellipsoid ascospores; only known from the Eastern Alps (Slovenia). – **Sl**: SlA.

***Thelidium grummannii* Servít**

L # – Subs.: cal – Alt.: 4–5 – Note: a calcicolous species with a mainly endolithic, greyish thallus with brown dots, semi-immersed perithecia (to *c.* 0.3 mm in diam.) with a thin thalline annulus, without involucrellum (?), and 1-septate, oblong ascospores (to *c.* 30 µm long); only known from the type locality in the Western Alps (Switzerland). – **Sw**: BE.

***Thelidium helveticum* (Servít) Hafellner**

Syn.: *Involucrothele helvetica* Servít

L # – Subs.: sil – Alt.: 3 – Note: a species resembling *Th. methorium*, with an epilithic, whitish, spreading, rimose to areolate thallus, hemispherically protruding, often crowded to laterally fusing perithecia (to 0.5 mm in diam.), an involucrellum which is loosely attached to the perithecial wall almost down to the base, and 1-septate, broadly ellipsoid ascospores (mostly to *c.* 25 µm long); on siliceous schists, only known from the type locality in the Western Alps (Switzerland). – **Sw**: BE.

***Thelidium impressulum* Zschacke**

L – Subs.: cal – Alt.: 3–5 – Note: a species of the *Th. pyrenophorum*-group forming small, endolithic, whitish thalli and semi-immersed, small perithecia (to *c.* 0.2 mm in diam.) with involucrellum reaching down to the base, and ellipsoid, 1-septate, halonate ascospores (to 15 µm long); on calcareous rocks (dolomite at type locality) at mid-to high elevations; not rare in the Alps but probably regionally still undercollected. – **Au**: St. **Ge**: OB. **Sw**: GR. **Fr**: AHP, Drô, Sav, Vau.

***Thelidium impressum* (Müll. Arg.) Zschacke**

Syn.: *Sagedia impressa* Müll. Arg.

L – Subs.: cal – Alt.: 2–5 – Note: a species of the *Th. pyrenophorum*-group resembling *Verrucaria dufourii*, but perithecia smaller (to 0.3 mm in diam.) with involucrellum reaching down about half of the perithecium, and with ellipsoid, 1-septate ascospores (to 12 µm long); on inclined surfaces of compact calcareous rocks in upland areas. – **Au**: ?V, K, St, O, N. **Sw**: SZ. **Fr**: AHP, Drô, HSav, Vau.

***Thelidium incavatum* Nyl. *ex* Mudd**

Syn.: *Amphoridium umbrosum* A. Massal., *Amphoroblastia incavata* (Nyl. *ex* Mudd) Servít, *Polyblastia incavata* (Nyl. *ex* Mudd) Croz., *Thelidium umbrosum* (A. Massal.) Arnold, *Verrucaria umbrosa* (A. Massal.) Trevis.

L – Subs.: cal, int – Alt.: 2–5 – Note: on small calcareous pebbles close to the ground, usually in upland areas; probably more widespread, but overlooked. *Th. umbrosum* is perhaps an independent species. – **Au**: V, T, S, K, St, O, N. **Ge**: OB. **Sw**: BE, GR, LU, SZ, UR, UW. **Fr**: AHP, HAl, AMa, Drô, Isè, Sav, HSav, Var, Vau. **It**: Frl, Ven, TAA, Piem, Lig. **Sl**: SlA.

***Thelidium inundatum* Zschacke**

L – Subs.: cal-aqu – Alt.: 3 – Note: an endolithic amphibious lichen found in periodically submerged situations, mostly on calcareous substrata in upland areas, but usually below the alpine belt; from the Alps there are only a few scattered records. – **Au**: O. **Fr**: AHP. **It**: Ven, TAA.

***Thelidium klementii* Servít**

L # – Subs.: int-aqu – Alt.: 3 – Note: a species with a thin, whitish, subareolate thallus, semi-immersed perithecia (to *c.* 0.2 mm in diam.), ellipsoid, 1-septate ascospores with some non-septate intermixed (to *c.* 30 µm long); on temporarily submerged calcareous schists; only known from the type locality in the Eastern Alps (Germany). – **Ge**: Schw.

***Thelidium krempelhuberi* (Servít) ined. (provisionally placed here, ICN Art. 36.1b)**

Syn.: *Involucrothele krempelhuberi* Servít

L # – Subs.: cal – Alt.: 4 – Note: a calcicolous species with a thin, whitish thallus and hemispherically protruding ascomata (to 0.25 mm in diam.), a tightly adpressed involucrellum reaching down about half of the perithecium, ellipsoid, non-septate ascospores with *c.* 20% 1-septate ascospores intermingled (to *c.* 20 µm long); only known from the type locality in the Eastern Alps (Germany). – **Ge**: Schw.

***Thelidium methorium* (Nyl.) Hellb.**

Syn.: *Involucrothele aeneovinosa* (Anzi) Servít, *Involucrothele kutakii* (Servít) Servít, *Polyblastia kutakii* Servít, *Sagedia aeneovinosa* Anzi, *Thelidium aeneovinosum* (Anzi) Arnold, Thelidium aeneovinosum (Anzi) Arnold var. kutakii Servít, *Thelidium diaboli* A. Massal., *Thelidium kutakii* (Servít) Servít, *Verrucaria methoria* Nyl.

L – Subs.: sil, sil-aqu, cal, int – Alt.: 3–5 – Note: an arctic-alpine to boreal-montane, probably circumpolar lichen found on periodically submerged siliceous rocks in alpine to montane creeks; widespread throughout the siliceous Alps. – **Au**: V, T, S, K, St. **Sw**: SZ. BE, GR, UR, VS. **Fr**: HAl, AMa, Sav, HSav. **It**: Frl, TAA, Lomb, Piem, Lig.

***Thelidium microbolum* (Tuck.) Hasse**

Syn.: *Verrucaria microbola* Tuck.

L # – Subs.: sil – Alt.: 4 – Note: a species with a thallus consisting of olivaceous-grey granules and 3-septate ascospores (to 30 µm long), based on a calcicolous type from Canada (Ontario); the identity of the single European record from the Austrian Alps (on temporarily inundated schists in the subalpine belt!) needs confirmation. – **Au**: S.

***Thelidium minimum* (A. Massal. *ex* Nyl.) Arnold**

Syn.: *Involucrothele minima* (A. Massal. *ex* Nyl.) Servít, *Verrucaria minima* A. Massal. *ex* Nyl.

L – Subs.: cal – Alt.: 2–5 – Note: on calcareous pebbles, or on rock surfaces close to the ground in upland areas. – **Au**: ?V, T, S, St, N. **Ge**: Ge. **Sw**: GR, LU, SZ, VD. **Fr**: AHP. **It**: TAA.

***Thelidium minutulum* Körb.**

Syn.: *Arthopyrenia mesotropa* (Nyl.) Arnold, *Involucrothele margacea* (Leight.) Servít, *Thelidium aethioboloides* (Nyl.) Vain. *non* Zschacke, *Thelidium eitneri* Zahlbr., *Thelidium hospitum* Arnold, *Thelidium margaceum* (Leight.) Zschacke, *Thelidium mesotropum* (Nyl.) A.L. Sm., *Thelidium terrestre* Walt. Watson

L – Subs.: cal, int, sil-aqu – Alt.: 2–5 – Note: a widespread, cool-temperate to arctic-alpine, circumpolar, pioneer lichen found on calcareous pebbles close to the ground, on roofing tiles and on brick walls, occasionally also in the splash water zone of creeks; probably more widespread in the Alps. – **Au**: T, S, K, St, O. **Ge**: OB, Schw. **Sw**: GR, SZ. **Fr**: AHP, AMa, Vau. **It**: Ven, TAA. **Sl**: SlA.

***Thelidium montanum* (Hepp) Körb.**

Syn.: *Paraphysothele montana* (Körb.) Zschacke, Thelidium nylanderi (Hepp) Lönnr. var. montanum Hepp in Arnold

L – Subs.: cal – Alt.: 3 – Note: this taxon was described by Körber (Parerga: 351, 1863) as a new species, and not as a new combination of an already existing taxon. His description constitutes the protologue. Körber (l.c.) cited *Th. nylanderi* (Hepp) Lönnr. β *montanum* Hepp as a synonym. This infraspecific taxon was published by Arnold (Flora 41: 554 [misprinted “254”], 1858), who attributed both name and description to Hepp. The two taxa are based on the same type. The species has a greyish, farinose thallus and hemispherical, subsessile perithecia with involucrellum reaching down to the base, partly persisting interascal filaments (*fide* Zschacke), and 1-septate, narrowly ellipsoid ascospores with a somewhat wider upper cell (to *c.* 25 µm long); the generic placement is in need of verification; on calcareous rocks at rather low elevations, known from a few localities in the Eastern Alps and the northern foreland. – **Au**: S, O.

***Thelidium nigricans* Zschacke**

L # – Subs.: sil-aqu, cal-aqu – Alt.: 3 – Note: this species is known only from two widely separated collections from Romania and Switzerland (a record from Bavaria is dubious), but was perhaps overlooked elsewhere. It is very similar to *Th. aethioboloides* and molecular data are needed in order to clarify whether they are two genetically distinct species. – **Ge**: ?Schw. **Sw**: BE.

***Thelidium obscurum* (Garov.) Zschacke**

Syn.: *Involucrothele obscura* (Garov.) Servít, Verrucaria olivacea Fr. var. obscura Garov.

L # – Subs.: cal – Alt.: 2–4 – Note: a species with a blackish thallus, entirely immersed perithecia, and 1-septate ascospores (to *c.* 30 µm long); on sheltered calcareous rocks, with a few records only from the Western and Southern Alps. – **Fr**: HAl, Var. **It**: Lomb.

***Thelidium olivaceum* (Fr.) Körb. [*nom.illeg.***]

Syn.: *Arthopyrenia olivacea* (Fr.) A. Massal., *Arthopyrenia pseudolivacea* (Nyl.) H. Olivier, *Involucrothele olivacea* (Fr.) Servít, *Verrucaria olivacea* Fr. (1831) *non* Pers. (1794) *nec* Hoffm. (1796) *nec* Wallr. (1831), *Verrucaria pseudolivacea* Nyl.

L – Subs.: cal – Alt.: 2–5 – Note: a circumboreal-montane species of calcareous rocks. The combination is based on an illegitimate basionym. – **Au**: T, S, St, O. **Sw**: GR, LU, SZ, VD, VS. **Fr**: Vau. **It**: Ven, TAA, Lomb.

***Thelidium papulare* (Fr.) Arnold**

Syn.: *Arthopyrenia sprucei* (Bab.) H. Olivier, *Polyblastia papularis* (Fr.) Servít, *Sagedia lariana* (A. Massal.) Anzi, *Thelidium jurassicum* Zschacke, *Thelidium larianum* A. Massal., *Thelidium pyrenophorum* *sensu* A. Massal., *Thelidium rubellum* A. Massal., *Thelidium sprucei* (Bab.) Lönnr., *Thelidium subpapulare* Zschacke, *Thelidium umbilicatum* Th. Fr., ?*Thelidium variabile* B. de Lesd., *Verrucaria cryptarum* Garov., *Verrucaria leonina* Anzi, *Verrucaria papularis* Fr., *Verrucaria sprucei* Bab.; incl. Thelidium papulare (Fr.) Arnold f. fuscum Zschacke

L – Subs.: cal, int – Alt.: 3–6 – Note: an arctic-alpine to boreal-montane, circumpolar species of calcareous rocks, with optimum on limestone and dolomite, but also found on calciferous schist and sandstone in upland areas, sometimes growing in temporarily submerged sites along creeks; widespread throughout the Alps. – **Au**: V, T, S, K, St, O, N. **Ge**: OB, Schw. **Sw**: BE, GR, LU, SG, SZ, UR, UW, VD, VS. **Fr**: AHP, HAl, AMa, Isè, Sav, HSav, Vau. **It**: Frl, Ven, TAA, Lomb, Piem, VA. **Sl**: SlA.

***Thelidium paneveggiensis* (Servít) ined. (provisionally placed here, ICN Art. 36.1b)**

Syn.: *Involucrothele paneveggiensis* Servít

L # – Subs.: cal – Alt.: 4 – Note: a species with a thin, epilithic, brownish thallus and subsessile, globose ascomata (to *c.* 0.15 mm in diam.), a tightly adpressed involucrellum reaching down to the base of the perithecium, and ellipsoid, non-septate ascospores with *c.* 5% 1-septate ascospores intermingled (to *c.* 20 µm long); on calciferous siliceous rocks along a stream, only known from the type locality in the Eastern Alps (Italy). – **It**: TAA.

***Thelidium parvulum* Arnold**

L # – Subs.: cal – Alt.: 3 – Note: a species with a thin, greenish thallus, protruding, black, hemispherical perithecia lacking an involucrellum, and 1 – to 3-septate ascospores (to *c.* 25 µm long); on sandstone and limestone at low elevations; widespread in extra-Alpine Central Europe, with a single record from the Eastern Alps (Austria). – **Au**: N.

***Thelidium perexiguum* (Müll. Arg.) Zahlbr.**

Syn.: *Sagedia perexigua* Müll. Arg.

L # – Subs.: cal – Alt.: 2 – Note: a species resembling *Staurothele rupifraga*, with a very thin, lead-grey to bluish grey thallus, minute, entirely immersed ascomata, and 3-septate obovoid ascospores (to 35 µm long); on limestone at low elevations; only known from the Western Alps (France). – **Fr**: HSav.

***Thelidium pertusatii* (Garov.) Jatta**

Syn.: *Verrucaria pertusatii* Garov.

L – Subs.: sil-aqu – Alt.: 4–5 – Note: amphibious on frequently wetted siliceous rocks in alpine rivers and irrigated rocks. Type material on granite, near a creek. – **Au**: S, K. **Fr**: AMa. **It**: TAA, Piem, VA.

***Thelidium pluvium* Orange**

L – Subs.: sil-aqu – Alt.: 2–3 – Note: this species grows in the splash zone of small rivers and creeks on siliceous rocks and pebbles, usually in shaded situations; in the study area it was only reported from the Eastern Alps (Austria). – **Au**: St.

***Thelidium pyrenophorellum* (Servít) Hafellner**

Syn.: *Involucrothele pyrenophorella* Servít

L # – Subs.: cal – Alt.: 3 – Note: a calcicolous species with a very thin, epilithic, whitish-greyish thallus with darker dots, black, hemispherically protruding ascomata (to *c.* 0.25 mm in diam.), an involucrellum reaching down about half of the perithecium, adpressed above and slightly spreading further down, and 1-septate, more or less oblong ascospores, the upper cell somewhat wider and the lower cell slightly attenuated (to *c.* 32 µm long); only known from the type locality the Eastern Alps (Austria). – **Au**: O.

***Thelidium pyrenophorum* (Ach.) A. Massal.**

Syn.: *Involucrothele pyrenophora* (Ach.) Servít, *Paraphysothele viridis* (Deakin) Zschacke, *Sagedia pyrenophora* (Ach.) Hepp, *Thelidium borreri* (Leight.) Mudd, *Thelidium explicatum* (Stirt.) Wheldon & A.Wilson, *Thelidium mortensis* Walt. Watson, *Thelidium nylanderi* (Hepp) Lönnr., *Thelidium viride* (Deakin) Zahlbr., *Verrucaria pyrenophora* Ach.; incl. Thelidium pyrenophorum (Ach.) A. Massal. f. intermedium Asta, Clauzade & Cl. Roux

L – Subs.: cal, int – Alt.: 2–5 – Note: a widespread lichen with optimum on limestone and dolomite, but also found on calciferous sandstone; common throughout the Alps. – **Au**: V, T, S, K, St, O, N. **Ge**: OB. **Sw**: BE, GL, GR, LU, SZ, UR, VD, VS. **Fr**: AHP, HAl, AMa, Sav, HSav, Vau. **It**: Frl, Ven, TAA, Lomb, Piem, VA. **Sl**: SlA, Tg.

***Thelidium rehmii* Zschacke**

L – Subs.: sil, sil-aqu – Alt.: 2–3 – Note: a species with a thin, greenish thallus, black, hemispherically protruding perithecia (to *c.* 0.3 mm in diam.) lacking an involucrellum, and 1-septate, ellipsoid ascospores (to *c.* 30 µm long); on sandstone and siliceous schists in humid situations, at low elevations; rare throughout Central Europe; from the Alps there are only a few scattered records. – **Au**: T, S, St, O. **Sw**: SZ.

***Thelidium rivulicola* (Nyl.) Mig.**

Syn.: *Arthopyrenia rivulicola* (Nyl.) Arnold, *Verrucaria rivulicola* Nyl.

L # – Subs.: cal-aqu – Alt.: 3 – Note: a species with a whitish, subfarinose thallus, hemispherically protruding perithecia (to *c.* 0.25 mm in diam.), and 1-septate, oblong ascospores (to *c.* 30 µm long); on periodically submerged calcareous stones, rare in Western and Central Europe, including the Alps (Germany). – **Ge**: OB.

***Thelidium schibleri* Zschacke**

L # – Subs.: cal – Alt.: 3 – Note: a calcicolous species with a blackish-brown, thin thallus, semi-immersed ascomata (to 0.3 mm in diam.) with involucrellum reaching down about half of the perithecium, and 1-septate, broadly ellipsoid ascospores (to *c.* 45 µm long); only known from the type locality. – **Sw**: GR.

***Thelidium scopolianum* Servít**

L # – Subs.: cal – Alt.: 3 – Note: a species with a thin, grey thallus with a violet tinge, hemispherically protruding ascomata (to *c.* 0.5 mm in diam.), and 3-septate, oblong ascospores (to *c.* 55 µm long); on dolomite in humid forested valleys; only known from the type locality in the Eastern Alps (Slovenia). – **Sl**: SlA.

***Thelidium subabsconditum* Eitner**

Syn.: incl. *Thelidium circumvallatum* Zschacke

L – Subs.: cal – Alt.: 3–5 – Note: a species resembling *Th. absconditum* in the entirely immersed perithecia (to *c.* 0.2 mm in diam.) lacking an involucrellum, but with a very thin, bluish grey thallus, and smaller, 1-septate ascospores (less than 25 µm long); in the Alps it is common on inclined surfaces of compact calciferous rocks in rather shaded, non-eutrophicated situations, but it was not always distinguished, and the distribution appears incomplete. – **Au**: ?V, ?T, St. **Fr**: AHP, AMa, Drô, Isè, Sav, HSav, Var, Vau.

***Thelidium submethorium* (Vain.) Zschacke**

Syn.: *Verrucaria submethoria* Vain.

L – Subs.: sil-aqu – Alt.: 2–5 – Note: a rare species of siliceous substrata in clean creeks and rivers of high mountain ranges, with optimum above treeline; in the Alps there are only a few scattered records – **Au**: T, S. **Fr**: AHP. **It**: TAA.

***Thelidium subrimulatum* (Nyl.) Zschacke**

Syn.: *Verrucaria subrimulata* Nyl.

L – Subs.: cal – Alt.: 3–5 – Note: this species, described from the Pyrenees, has been collected from very few localities in upland areas of Southern Europe, on limestone and calcareous schists; from the Alps there are only a few scattered records. – **Au**: V, ?T, St, O. **Fr**: HAl, AMa, Sav. **It**: Frl.

***Thelidium subsimplex* Zschacke**

L – Subs.: cal, int – Alt.: 4–5 – Note: a calcicolous species of the *Th. pyrenophorum*-group with a rather thick, whitish thallus, finally hemispherically protruding ascomata (to *c.* 0.3 mm in diam.) with involucrellum reaching down about two thirds the perithecium, and most ascospores unicellular, with some 1-septate ones intermingled (to *c.* 20 µm long); known from a few scattered localities in the Alps. – **Au**: ?V, T, S, St, O. **Ge**: OB. **Sw**: UW.

***Thelidium tiroliense* Zschacke**

L – Subs.: cal – Alt.: 3–4 – Note: a calcicolous species of the *Th. pyrenophorum*-group with a thin thallus in various shades of brown, subsessile ascomata with involucrellum reaching down to the base of the perithecium (or even closed), and 1-septate, ellipsoid ascospores (to *c.* 20 µm long); known from a few localities in the Eastern Alps (Austria). – **Au**: T, St.

***Thelidium ungeri* Flot. *ex* Körb.**

Syn.: *Verrucaria ungeri* Flot. *ex* Körb. *nom.illeg*.

L – Subs.: cal, int – Alt.: 3–5 – Note: on inclined surfaces of calciferous rocks in upland areas. Closely related to *Th. pyrenophorum*, from which it differs in the thick, verrucose thallus; widespread throughout the Alps. – **Au**: ?V, T, S, K, St, O, N. **Ge**: OB. **Fr**: AHP, HAl, AMa, Sav, HSav, Vau. **It**: Ven, TAA, Lomb, Piem. **Sl**: SlA.

***Thelidium verrucosum* Zschacke**

L – Subs.: cal – Alt.: 3–5 – Note: a calcicolous species of the *Th. papulare*-group with an endolithic thallus, ascomata immersed in up to 1 mm broad, concolorous warts, a spreading involucrellum, and 3-septate ascospores (to *c.* 35 µm long); rare throughout Central Europe, including the Eastern Alps (Austria). – **Au**: St, N.

***Thelidium zahlbruckneri* Servít**

L # – Subs.: cal – Alt.: 2 – Note: a species with a thin, brown to greyish-brown thallus, hemispherically protruding perithecia (to *c.* 0.15 mm in diam.) lacking an involucrellum, and 1-septate, ellipsoid ascospores (to *c.* 15 µm long); on pebbles of calcareous sandstone; only known from the type locality in the Eastern Alps (Austria). – **Au**: N.

***Thelidium zwackhii* (Hepp) A. Massal.**

Syn.: *Sagedia zwackhii* Hepp, Thelidium fueistingii *auct. non* Körb., *Thelidium microcarpum* (Davies *ex* Leight.) A.L. Sm., *Thelidium montinii* Beltr., *Thelidium subgelatinosum* Zschacke, Thelidium velutinum *auct. p.p. non* (Bernh.) Körb., *Thelidium xylospilum* (Nyl.) Zschacke, *Verrucaria microcarpa* Davies *ex* Leight., *Verrucaria xylospila* Nyl.

L – Subs.: cal, ter-cal – Alt.: 2–4 – Note: a mainly temperate, ecologically broad-ranging, pioneer species found both on calcareous and on siliceous rocks, and on thin layers of soil, *e.g.* on walls, pebbles, etc., occasionally also in periodically submerged sites; one of the few species of the genus which occur at low altitudes. – **Au**: V, S, ?St, O, N. **Ge**: Ge. **Sw**: GR, SZ. **Fr**: HAl. **It**: Frl, Ven. **Sl**: SlA.

***Thelignya lignyota* (Wahlenb.) P.M. Jørg. & Henssen**

Syn.: *Arctoheppia scholanderi* Lynge, *Porocyphus dispersus* E. Dahl, *Porocyphus ocellatus* (Th. Fr.) Henssen, *Psorotichia fuliginea* (Ach.) Körb., *Psorotichia lignyota* (Wahlenb.) Forssell, *Psorotichia ocellata* (Th. Fr.) Forssell, *Pyrenopsis lignyota* (Wahlenb.) Th. Fr., *Pyrenopsis ocellata* Th. Fr., *Verrucaria fuliginea* Ach., *Verrucaria lignyota* Wahlenb.

L – Subs.: int – Alt.: 3–4 – Note: a more or less arctic-alpine species found on base – or lime-rich siliceous substrata, periodically submerged in cold creeks, or in seepage tracks; so far only recorded from a few scattered localities in the Alps. – **Au**: T, S, St. **Sw**: VS. **Fr**: AMa.

***Thelocarpella gordensis* Nav.-Ros. & Cl. Roux**

Syn.: *Trimmatothelopsis gordensis* (Nav.-Ros. & Cl. Roux) K. Knudsen & Lendemer

L – Subs.: cal – Alt.: 2–3 – Note: a peculiar, extremely rare species with an endolithic thallus, immersed perithecioid ascomata with persistent interascal filaments, polyspored asci, and simple, oblong ascospores (to 6 µm long). The species has a basal position in the *Trimmatothelopsis*-clade, and perhaps belongs there. It grows on steeply inclined surfaces of calciferous rocks in well-lit situations, being only known from Central Europe and the western Mediterranean region; there are a few disjunct lowland records from the Alps; the Austrian one needs confirmation. – **Au**: ?N. **Fr**: Drô, Vau.

***Thelocarpon citrum* (Wallr.) Rossman**

Syn.: *Sphaeria citrum* Wallr., *Thelocarpon arenicola* Vain., *Thelocarpon herteri* J. Lahm, *Thelocarpon vicinellum* Nyl.

L – Subs.: sil – Alt.: 3–4 – Note: an ephemeral species of disturbed habitats, with optimum near treeline; so far only reported from the Eastern Alps (Austria, Italy). – **Au**: N. **It**: TAA.

***Thelocarpon coccosporum* Lettau**

L – Subs.: deb – Alt.: 3 – Note: a species with pale yellow ascomata, peculiar in lacking paraphyses and in the globose ascospores; on stones and plant debris; rare in Central Europe, including the Eastern Alps (Austria). – **Au**: K.

***Thelocarpon epibolum* Nyl.**

Syn.: *Thelocarpon conoidellum* Nyl., Thelocarpon epibolum Nyl. var. epithallinum (Leight. *ex* Nyl.) G. Salisb., *Thelocarpon epithallinum* Leight. *ex* Nyl.

L – Subs.: xyl, bry, ter-sil, par – Alt.: 3–5 – Note: an ephemeral, facultatively lichenised species found on foliose lichens, rotting wood, decaying bryophytes, peaty soil, mostly in upland areas; overlooked, and certainly more widespread in the Alps. In the *Th. epibolum*-group, two taxa are commonly distinguished: the typical variety, and var. epithallinum, lichenicolous on *Baeomyces rufus* and purported to have longer ascospores. On the other hand, *T. epibolum* itself was described as lichenicolous on *Solorina crocea*, and in our opinion var. epithallinum is not clearly distinguishable, its spores lying in the variation range of those of var. epibolum. Kocourková maintains that the two taxa can can be distinguished by the thickness of the interascal filaments and the host selection, assuming that they are specialised in different photobionts, adding a further undescribed taxon with long spores, specialised in the *Peltigera aphthosa*-group. Since we have found many asci with both shorter and longer spores, we merge the two taxa. – **Au**: T, S, K, St, O, N. **Ge**: OB. **Sw**: SZ. **It**: Frl, Ven, TAA. **Sl**: SlA.

***Thelocarpon impressellum* Nyl.**

Syn.: *Ahlesia impressella* (Nyl.) G. Salisb.

L – Subs.: ter-sil, ter-cal, cal, sil – Alt.: 3–5 – Note: a doubtfully lichenised species found on humus-rich soil, mosses, rotten wood and other lichens in upland areas. The only Italian record, growing on *Squamarina cartilaginea*, is dubious. – **Au**: T, S, K, St, O, N. **Ge**: Ge. **Fr**: HAl, HSav. **It**: ?TAA.

***Thelocarpon intermediellum* Nyl.**

Syn.: *Thelocarpon intermixtulum* Nyl.

L – Subs.: xyl, sil – Alt.: 2–4 – Note: a rarely-collected but apparently widespread ephemeral species of siliceous rocks and, occasionally, rotten wood, mostly in upland areas; from the Alps there are only a few scattered records. – **Au**: K, N. **Fr**: Vau. **It**: TAA.

***Thelocarpon laureri* (Flot.) Nyl.**

Syn.: *Sphaeropsis laureri* Flot., *Thelocarpon epilithellum* Nyl., *Thelocarpon interceptum* Nyl., *Thelocarpon prasinellum* Nyl.

L – Subs.: ter-sil, sil, xyl, bry – Alt.: 2–4 – Note: an ephemeral early coloniser of different substrata, including roofing tiles, rotten wood, and soil; perhaps more widespread in the Alps, but very much overlooked. – **Au**: V, T, S, St, O, N. **Ge**: OB. **Sw**: LU, SZ, VS. **It**: TAA.

***Thelocarpon lichenicola* (Fuckel) Poelt & Hafellner**

Syn.: *Ahlesia lichenicola* Fuckel, *Thelocarpon ahlesii* Fuckel, *Thelocarpon applanatum* H. Magn.

L – Subs.: sil-par, ter-sil-par, xyl – Alt.: 2–4 – Note: on clay soil in disturbed sites, often in *Calluna*-heaths, mostly on *Baeomyces rufus*; doubtfully lichenised, to be searched for further in the Alps. – **Au**: St, N. **It**: TAA.

***Thelocarpon olivaceum* B. de Lesd.**

Syn.: Thelocarpon intermixtulum Nyl. var. olivaceum (B. de Lesd.) H. Magn.

L # – Subs.: sil – Alt.: 3 – Note: a species with hemispherical, variably pruinose ascomata, externally with a thalline sheath, poorly developed to lacking paraphyses, and finally subglobose ascospores; rare throughout Europe, with a single record from the Eastern Alps (Austria). – **Au**: N.

***Thelocarpon saxicola* (Zahlbr.) H. Magn.**

Syn.: Thelocarpon epibolum Nyl. var. saxicola Zahlbr.

L # – Subs.: sil – Alt.: 3 – Note: a species with globose ascomata lacking paraphyses, and ellipsoid ascospores (to 7 µm long); on sandstone and similar rocks in the montane belt; only known from the Eastern Alps (Austria). – **Au**: N.

***Thelocarpon sphaerosporum* H. Magn.**

Syn.: *Ahlesia sphaerospora* (H. Magn.) G. Salisb.

L – Subs.: ter-par, bry – Alt.: 3 – Note: an ephemeral early coloniser of different substrata, including the thalli of other lichens, mostly in upland areas; from the Alps there are only a few scattered records. – **Au**: T, K, O. **Fr**: Sav. **It**: TAA.

***Thelocarpon superellum* Nyl.**

Syn.: *Thelocarpon conoideum* Höhn.

L – Subs.: ter-sil, cor, xyl – Alt.: 3 – Note: a species with relatively conspicuous, conical to globose ascomata, unbranched paraphyses, and oblong, often pseudoseptate ascospores (to 13 µm long); on soil, rotting wood or stones; widespread in the Holarctic region, from the boreal to the temperate zone, but rare; from the Alps there are a few scattered records, but perhaps the species was largely overlooked. – **Au**: T, N. **Sw**: SZ, UW.

***Thelopsis flaveola* Arnold**

L – Subs.: cor – Alt.: 3–5 – Note: a mainly temperate species found on bark of ancient deciduous trees, but also, in the subalpine belt, on bases of old *Rhododendron* shrubs; to be looked for further in the Alps. – **Au**: V, T, K, St. **Ge**: OB. **Sw**: BE, SZ. **It**: Lomb.

***Thelopsis lojkana* (Poetsch *ex* Arnold) Nyl.**

Syn.: *Sagedia lojkana* Poetsch *ex* Arnold, *Thelopsis tholoides* Lettau

L – Subs.: cal, int, cal-aqu – Alt.: 3 – Note: a species with smooth ascomata which are blackish brown in the upper half, immersed at first and later protruding, and 3-septate, halonate ascospores (to 25 µm long), found on vertical to overhanging faces of limestone in the shade of montane forests; rare in the Central European mountains, with a few scattered records from the Alps. – **Au**: S, St, O. **Sw**: BE. **Sl**: SlA.

***Thelopsis melathelia* Nyl.**

Syn.: *Sagedia melathelia* (Nyl.) Jatta, *Sagedia rugosa* Anzi, *Thelopsis rugosa* (Anzi) Jatta, *Thelopsis umbratula* Nyl.

L – Subs.: bry, deb, ter-cal, bry-cal – Alt.: 4–5 – Note: an arctic-alpine, circumpolar species found on moribund bryophytes, humic soil and plant remains over more or less calcareous substrata; widespread throughout the Alps. – **Au**: V, T, S, K, St, O, N. **Ge**: OB, Schw. **Sw**: BE, GR, SG, SZ. **Fr**: HAl. **It**: Frl, Ven, TAA, Lomb, Piem.

***Thelopsis rubella* Nyl.**

Syn.: *Pyrenula bayrhofferi* (Körb.) Hepp, *Sagedia rubella* (Nyl.) Jatta

L – Subs.: cor – Alt.: 1–3 – Note: a mild-temperate species found on old deciduous trees (*e.g.* *Fagus*, *Quercus*), especially near the base of the boles, in areas with high rainfall; widespread, but generally rare in the Alps. – **Au**: St, N. **Ge**: OB. **Sw**: GR, SG, SZ, TI, VS. **Fr**: Var, Vau. **It**: Frl, Lomb.

***Thelotrema lepadinum* (Ach.) Ach.**

Syn.: *Lichen lepadinus* Ach., *Volvaria lepadina* (Ach.) A. Massal.

L – Subs.: cor – Alt.: 1–3 – Note: a mild-temperate lichen found on the bark of *Fagus* and *Abies*, more rarely of other broad-leaved trees in humid montane forests with frequent fog or near rivers; widespread throughout the Alps, but generally not common, and perhaps declining. – **Au**: V, T, S, K, St, O, N. **Ge**: OB. **Sw**: BE, FR, GL, GR, LU, SG, SZ, UR, UW, VD, VS. **Fr**: AMa. **It**: Frl, Ven, TAA, Lomb, Piem. **Sl**: SlA, Tg.

***Thelotrema suecicum* (H. Magn.) P. James**

Syn.: *Ocellularia suecica* H. Magn.

L – Subs.: cor – Alt.: 3 – Note: a rarely collected species found on bark in humid forests; in the study area so far recorded from the Eastern Alps only (Austria, Italy). – **Au**: St, O, N. **It**: Frl.

***Thermutis velutina* (Ach.) Flot.**

Syn.: *Collema pannosum* Hoffm., *Collema velutinum* (Ach.) Ach., Collema velutinum (Ach.) Ach. var. pannosum (Hoffm.) Rabenh., *Gonionema velutinum* (Ach.) Nyl., *Lichen velutinus* Ach.

L – Subs.: sil, int – Alt.: 3–4 – Note: on base-or mineral-rich siliceous rock, in sun-exposed seepage tracks with colonies of cyanobacteria, mostly in upland areas but usually below treeline. – **Au**: ?V, T, S, K, St, N, B. **Ge**: OB. **Sw**: BE, GL, GR, SG, SZ, TI, VS. **Fr**: AMa, HSav. **It**: Frl, Ven, TAA, Lomb, Piem, Lig.

***Tholurna dissimilis* (Norman) Norman**

Syn.: *Podocratera dissimilis* Norman

L – Subs.: cor – Alt.: 4 – Note: a peculiar species with brownish grey, cushion-like, dense, radially arranged podetia arising from a crustose basal thallus, apical ascomata with a black, epruinose mazaedium, and 1-septate ascospores with a helicoid perispore; on the top of not too tall spruce trees often visited by birds; widespread in the Holarctic region, with a single record from the Eastern Alps (Austria). – **Au**: S.

***Thrombium aoristum* (Nyl.) Arnold**

Syn.: *Verrucaria aorista* Nyl.

L # – Subs.: ter-sil – Alt.: 2 – Note: this name is used for a lichen differing from typical *Th. epigaeum* in the hyaline ascomatal wall (except the blackish ostiolar region), and perhaps designates only a morphotype; on acidic soil, mainly in Western Europe, including the limit of the Southern Pre-Alps (France). – **Fr**: Vau.

***Thrombium epigaeum* (Pers.) Wallr.**

Syn.: *Sphaeria epigaea* Pers., *Thrombium aoristoides* I.M. Lamb, *Verrucaria epigaea* (Pers.) Ach.

L – Subs.: ter-sil, ter-cal – Alt.: 2–5 – Note: an ephemeral, probably holarctic coloniser of calciferous, clayey soil in rather disturbed habitats, such as track sides and openings in grasslands; widespread throughout the Alps. – **Au**: V, T, S, K, St, N. **Ge**: OB, Schw. **Sw**: BE, GR, UR, VS. **Fr**: AHP, AMa, Sav, HSav. **It**: Frl, Ven, TAA, Lomb, Piem, Lig. **Sl**: SlA.

***Thyrea confusa* Henssen**

Syn.: Omphalaria pulvinata *auct. non* (Schaer.) Nyl., Thyrea pulvinata *auct. non* (Schaer.) A. Massal.

L – Subs.: cal – Alt.: 1–4 – Note: on steeply inclined, sunny faces of calcareous rocks with short periods of water seepage after rain; widespread throughout the Alps. – **Au**:  V, T, S, K, St, N. **Sw**: TI, UW, VD, VS. **Fr**: AHP, AMa, Isè, Var, Vau. **It**: Frl, Ven, TAA, Lomb, Piem, Lig.

***Thyrea girardii* (Durieu & Mont.) Bagl. & Carestia**

Syn.: *Collema girardii* Durieu & Mont.

L – Subs.: cal – Alt.: 1–2 – Note: a Mediterranean to mild-temperate species found on calcareous rocks; ecology and distribution resemble those of *Th. confusa*; several records from the Alps need confirmation. – **Sw**: ?GR, ?VS. **Fr**: AHP, AMa. **It**: Frl, Ven, Lomb, Piem.

***Thyrea pachyphylla* (Müll. Arg.) Henssen**

Syn.: ?Omphalaria pulvinata (Schaer.) Nyl. var. laxa Müll. Arg., Omphalaria pulvinata (Schaer.) Nyl. var. pachyphylla Müll. Arg.

L # – Subs.: cal – Alt.: 2 – Note: a species resembling *Th. girardii*, but with a polyphyllous thallus, found on humid calcareous rocks at low elevations; only known from the type locality in the Western Alps (Switzerland). – **Sw**: VS.

***Thyrea plectopsora* A. Massal.**

Syn.: *Omphalaria phylliscoides* Nyl., ?*Thyrea nummularioides* (Nyl.) A. Massal., *Thyrea phylliscoides* (Nyl.) Zahlbr.

L – Subs.: cal-aqu – Alt.: 1–2 – Note: on steeply inclined seepage tracks of calcareous rocks; apparently more frequent in the Southern and Western Alps. – **Sw**: ?TI. **Fr**: AHP, AMa, Var, Vau. **It**: Ven, Lig.

***Timdalia intricata* (H. Magn.) Hafellner**

Syn.: *Acarospora intricata* H. Magn.

L – Subs.: sil, met, int – Alt.: 4–5 – Note: on base-rich siliceous rocks in sunny, exposed sites, with optimum above treeline; rarely collected, but perhaps more widespread in the Alps. – **Au**: T, S, St. **It**: TAA, Lomb.

***Toensbergia leucococca* (R. Sant.) Bendiksby & Timdal**

Syn.: *Hypocenomyce leucococca* R. Sant., *Pycnora leucococca* (R. Sant.) R. Sant.

L – Subs.: cor – Alt.: 2–4 – Note: a peculiar, obligately sterile species with a thallus consisting of scattered, whitish, adnate areolae and usually marginal soralia, containing alectorialic acid; on bark of deciduous trees in various forest types; widespread in the Holarctic region from the boreal to the nemoral-montane zone, including the Alps, but still overlooked in some regions. – **Au**: T, S, K, St, O, N. **Ge**: Schw. **Sw**: SZ, UW.

***Toninia albilabra* (Dufour) H. Olivier**

Syn.: *Biatora albilabra* Dufour, *Lecidea albilabra* (Dufour) Dufour, *Psora albilabra* (Dufour) Körb. *non auct.*, *Toninia albomarginata* B. de Lesd.

L – Subs.: ter-cal – Alt.: 1–3 – Note: a mainly Mediterranean species found on more or less calciferous ground and in fissures of rocks and walls, often on cyanobacteria or cyanobacterial lichens when young; common only in dry areas, including the Alpine valleys with a continental climate. – **Au**: T. **Sw**: VS. **Fr**: Var. **It**: TAA, VA, Lig.

***Toninia alutacea* (Anzi) Jatta**

Syn.: *Biatorina alutacea* (Anzi) Jatta, *Thalloidima alutaceum* Anzi, *Thalloidima intermedium* A. Massal. *ex* Arnold, *Toninia intermedia* (A. Massal. *ex* Arnold) H. Olivier, ?*Toninia subcandida* B. de Lesd.

L – Subs.: cal – Alt.: 2–5 – Note: an arctic-alpine, circumpolar species with southern outposts in steppic-continental regions, found in fissures of calciferous rocks; when young it often overgrows cyanobacterial colonies and cyanobacterial lichens; widespread throughout the Alps. – **Au**: V, T, S, K, St, O, N. **Ge**: OB. **Sw**: BE, GR, LU, SZ, TI, VS. **Fr**: AHP, AMa, Isè. **It**: Frl, Ven, TAA, Lomb, Piem, VA. **Sl**: SlA.

***Toninia aromatica* (Sm.) A. Massal.**

Syn.: *Bacidia sardoa* (Körb.) Zahlbr., *Bilimbia acervulata* (Nyl.) Jatta, *Bilimbia aromatica* (Sm.) Jatta, *Bilimbia sanguinaria* (Bagl.) Jatta, *Bilimbia sardoa* Körb., *Lecidea acervulata* Nyl., *Lecidea aromatica* (Sm.) Turner, *Lecidea austerula* Nyl., *Lecidea fusispora* (Hepp *ex* Körb.) Stizenb., *Lecidea geoleuca* Nyl., *Lecidea heterophora* Nyl., *Lecidea hypsophila* Nyl., *Lecidea subaromatica* Nyl., *Lecidea turneri* Leight., *Lichen aromaticus* Sm., *Raphiospora fusispora* Hepp *ex* Körb., *Thalloidima fusisporum* (Hepp *ex* Körb.) Müll. Arg., *Toninia acervulata* (Nyl.) Kremp., *Toninia affinis* Vězda, *Toninia fusispora* (Hepp *ex* Körb.) Th. Fr., *Toninia geoleuca* (Nyl.) Zahlbr., *Toninia heterophora* (Nyl.) Arnold, *Toninia hypsophila* (Nyl.) Zahlbr., *Toninia meridionalis* B. de Lesd., *Toninia pelophila* Poelt & Vězda, *Toninia sanguinaria* Bagl., *Toninia sinensis* Zahlbr., *Toninia squamulosa* Deakin, *Toninia turneri* (Leight.) H. Olivier

L – Subs.: cal, int, ter-cal – Alt.: 1–5 – Note: a holarctic species with a wide latitudinal range, found on horizontal to weakly inclined surfaces of calcareous to basic siliceous substrata, including bricks and roofing tiles in urban areas, often starting the life-cycle on other crustose lichens; the species has a wide altitudinal range, but seems to be most common at relatively low elevations; widespread throughout the Alps. – **Au**: T, S, K, St, O, N. **Ge**: OB. **Sw**: BE, GR, LU, SZ, TI, UW, VD, VS. **Fr**: AHP, HAl, AMa, Isè, Sav, HSav, Var, Vau. **It**: Frl, Ven, TAA, Lomb, Piem.

***Toninia athallina* (Hepp) Timdal**

Syn.: *Biatora athallina* Hepp, *Catillaria acrustacea* Arnold, *Catillaria athallina* (Hepp) Hellb., *Catillaria dvorakii* Servít, *Catinaria acrustacea* (Arnold) Vain., *Catinaria athallina* (Hepp) Lynge, *Kiliasia athallina* (Hepp) Hafellner

L – Subs.: cal – Alt.: 1–6 – Note: a temperate to arctic species of calcareous rocks, mostly on steeply inclined or underhanging faces in open, dry situations, sometimes invading the thalli of endolithic lichens, with a wide altitudinal range; widespread throughout the Alps. – **Au**: V, T, S, K, St, O, N. **Ge**: OB. **Sw**: BE, GR, LU, SZ, VS. **Fr**: AHP, HAl, AMa, Drô, Isè, Sav, HSav, Var, Vau. **It**: TAA, Lomb, Lig. **Sl**: SlA.

***Toninia candida* (Weber) Th. Fr.**

Syn.: *Biatorina candida* (Weber) Jatta, *Lecidea candida* (Weber) Ach., *Lichen candidus* Weber, *Psora candida* (Weber) Hoffm., *Thalloidima candidum* (Weber) A. Massal.

L – Subs.: cal, cal-par, ter-cal – Alt.: 1–5 – Note: a mainly southern, incompletely holarctic species found on steeply inclined surfaces and in fissures of calciferous rocks, chiefly limestone and dolomite, often on cyanobacteria or cyanobacterial lichens when young, with a wide altitudinal range; widespread throughout the Alps. – **Au**: V, T, S, K, St, O, N, B. **Ge**: OB. **Sw**: BE, GR, LU, SZ, TI, VD, VS. **Fr**: AHP, HAl, AMa, Drô, Isè, Sav, HSav, Var, Vau. **It**: Frl, Ven, TAA, Lomb, Piem, VA. **Sl**: SlA, Tg.

***Toninia cinereovirens* (Schaer.) A. Massal.**

Syn.: *Bilimbia cinereovirens* (Schaer.) Jatta, *Bilimbia fallasca* (A. Massal.) Jatta, *Bilimbia nigrescens* (Anzi) Jatta, *Lecidea cinereovirens* Schaer., *Toninia fallasca* A. Massal., *Toninia nigrescens* Anzi, *Toninia olivaceoatra* H. Magn., *Toninia potieri* Maheu & Werner, *Toninia sbarbaronis* B. de Lesd.

L – Subs.: sil, cal, ter-cal – Alt.: 1–4 – Note: a mainly southern, perhaps incompletely holarctic species found on steeply inclined, somehow weathered faces of calciferous and basic siliceous rocks with some seepage of water after rain, often in rock fissures and on colonies of cyanobacteria; widespread throughout the Alps, but only locally common. – **Au**: T, S, K, St, N. **Sw**: BE, GR, VD, VS. **Fr**: AHP, HAl, AMa, Sav, Var, Vau. **It**: Ven, TAA, Lomb, Piem, VA.

***Toninia coelestina* (Anzi) Vězda**

Syn.: Bacidia atrosanguinea (Hepp) Anzi subsp. oribata (Nyl.) A.L. Sm., *Bacidia coelestina* Anzi, *Lecidea oribata* Nyl., Lecidea subincompta Nyl. subsp. oribata (Nyl.) Cromb., *Toninia aggregata* Vězda, *Toninia oribata* (Nyl.) P. James

L – Subs.: ter-sil, int – Alt.: 3–4 – Note: a rare species found on cyanobacterial lichens or cyanobacterial colonies developing on weathered calciferous schists in upland areas; from the Alps there are only a few scattered records. – **Au**: K, St. **Sw**: VD. **Fr**: AHP. **It**: Lomb.

***Toninia diffracta* (A. Massal.) Zahlbr.**

Syn.: *Biatorina diffracta* (A. Massal.) Jatta, *Thalloidima diffractum* (A. Massal.) A. Massal., Thalloidima vesiculare (Hoffm.) A. Massal. var. diffractum A. Massal., Toninia candida (Weber) Th. Fr. subsp. diffracta (A. Massal.) Hild. Baumgärtner

L – Subs.: ter-cal, cal – Alt.: 1–5 – Note: a mainly southern eurasiatic species found in small fissures of steeply inclined faces of calcareous rocks, often on cyanobacteria or cyanobacterial lichens when young, sometimes on soil, with optimum at low altitudes, but exceptionally reaching the alpine belt; widespread throughout the Alps. – **Au**: V, T, S, K, St, O, N. **Ge**: OB. **Sw**: BE, LU, VD, VS. **Fr**: AHP, HAl, AMa, Drô, Isè, Sav, HSav, Var, Vau. **It**: Frl, Ven, TAA, Piem, VA, Lig.

***Toninia lutosa* (Ach.) Timdal**

Syn.: *Biatorina verrucosa* (A. Massal.) Jatta, *Lecidea lutosa* Ach., *Thalloidima verrucosum* A. Massal., *Toninia verrucosa* (A. Massal.) Flagey, *Toninia violacea* B. de Lesd.

L – Subs.: ter-cal, cal – Alt.: 1–3 – Note: a probably incompletely holarctic species of continental areas found on soil and weathered calciferous rocks, often in association with cyanobacteria or cyanobacterial lichens when young, mostly at relatively low elevations, with a few records from the Southern and Western Alps (France, Italy). – **Fr**: AHP. **It**: Ven, TAA, Lomb.

***Toninia nordlandica* Th. Fr.**

Syn.: *Lecidea subrimulosa* Nyl., *Toninia steineri* Poelt & Vězda, *Toninia subrimulosa* (Nyl.) Zahlbr.

L – Subs.: cal, cal-par, sil, sil-par – Alt.: 3–5 – Note: an arctic-alpine species found on steeply inclined to slightly underhanging seepage tracks of calciferous or basic siliceous rocks, almost always on cyanobacterial colonies, or on thalli of *Placynthium*, at least when young, mostly in upland areas. – **Au**: T, K. **Sw**: GR, SG, VD. **It**: TAA, Lomb, Piem, VA.

***Toninia opuntioides* (Vill.) Timdal**

Syn.: *Lichen opuntioides* Vill., ?*Thalloidima bormuelleri* Stein, ?*Toninia bornmuelleri* (Stein) Zahlbr.

L – Subs.: ter-cal, cal – Alt.: 2–5 – Note: an arctic to temperate, circumpolar lichen found often amongst bryophytes, always associated to cyanobacterial colonies or cyanobacterial lichens when young; widespread throughout the Alps. – **Au**: V, T, K, St, N. **Ge**: Ge. **Sw**: GR, SG, TI, VD, VS. **Fr**: AHP, HAl, AMa, Drô, Isè, HSav, Var, Vau. **It**: Frl, Ven, TAA, Lomb, Piem, VA.

***Toninia pennina* (Schaer.) Gyeln.**

Syn.: *Biatora pennina* (Schaer.) Hepp, *Catillaria scotina* (Körb.) Hertel & H. Kilias, *Lecidea aeneiformis* (Anzi) Jatta, *Lecidea pennina* Schaer., *Lecidea scotina* (Körb.) Arnold, *Lecidella scotina* Körb., *Psora aeneiformis* Anzi

L – Subs.: cal – Alt.: 2–4 – Note: a rarely collected lichen of continental-dry areas found on steeply inclined seepage tracks of dolomite, rarely of compact limestone, almost always growing on cyanobacterial colonies when young. – **Au**: T, N. **Sw**: TI, VS. **Fr**: HSav. **It**: Ven, TAA, Lomb.

***Toninia philippea* (Mont.) Timdal**

Syn.: *Catillaria arctica* Lynge, *Catillaria areolata* H. Magn., *Catillaria cirtensis* (Stizenb.) Flagey, *Catillaria holtedahlii* Lynge, *Catillaria ligustica* B. de Lesd., *Catillaria lutosa* A. Massal., *Catillaria philippea* (Mont.) A. Massal., *Catillaria riparia* (Müll. Arg.) Zahlbr., *Catillaria subgrisea* (Nyl.) Flagey, *Kiliasia philippea* (Mont.) Hafellner, *Kiliasia riparia* (Müll. Arg.) Hafellner, *Lecidea capitata* Anzi, *Lecidea cirtensis* Stizenb., *Lecidea lutosa* Mont. *ex* Schaer. *nom.illeg.*, *Lecidea philippea* Mont., *Lecidea subgrisea* Nyl., *Patellaria riparia* Müll. Arg.

L – Subs.: cal, int – Alt.: 1–5 – Note: an incompletely holarctic lichen of dry areas found on limestone, dolomite, calciferous sandstone and schists in open situations, most common in dry grasslands, with a wide altitudinal range. – **Au**: ?V, T, S, K, St, N. **Ge**: OB. **Sw**: GR, LU, SZ, VD. **Fr**: AHP, AMa, Sav, HSav, Vau. **It**: Ven, TAA, Lomb, Lig.

***Toninia physaroides* (Opiz) Zahlbr.**

Syn.: *Bacillina antipolitana* Nyl., *Biatorina lurida* (Bagl. *ex* Arnold) Jatta, *Lecidea physaroides* Opiz, *Thalloidima luridum* Bagl. *ex* Arnold, *Toninia alluviicola* M. Choisy, *Toninia lurida* (Bagl. *ex* Arnold) H. Olivier

L – Subs.: ter-cal, cal, int – Alt.: 1–5 – Note: a mainly temperate species, most common on soil developing from calciferous sandstone, often amongst mosses and associated to cyanobacterial lichens when young, rare in limestone areas. – **Au**: V, K, N. **Sw**: BE, GR, VD, VS. **Fr**: AHP, HAl, AMa, Isè, Sav, Vau. **It**: Ven, Lig.

***Toninia rosulata* (Anzi) H. Olivier**

Syn.: *Biatorina rosulata* (Anzi) Jatta, *Thalloidima rosulatum* Anzi, *Toninia melanocarpizans* Zahlbr.

L – Subs.: ter-cal, cal, bry – Alt.: 3–5 – Note: an arctic-alpine, mainly European species found on soil and in fissures and crevices of calciferous rocks, often on cyanobacteria or cyanobacterial lichens when young, with optimum above treeline; widespread throughout the Alps. – **Au**: V, T, S, K, St, O, N. **Ge**: OB, Schw. **Sw**: BE, FR, GR, SZ, TI, VD, VS. **Fr**: AHP, AMa, Isè, Sav, HSav. **It**: Frl, Ven, TAA, Lomb, Piem, VA. **Sl**: SlA.

***Toninia sedifolia* (Scop.) Timdal**

Syn.: *Biatorina vesicularis* (Hoffm.) Jatta, *Lecidea glebosa* Ach., *Lecidea subtabacina* Nyl., *Lecidea vesicularis* (Hoffm.) Ach., *Lichen sedifolius* Scop., *Patellaria vesicularis* Hoffm., *Psora paradoxa* (Ehrh.) Hoffm., *Psora vesicularis* (Hoffm.) Hoffm., Thalloidima caeruleonigricans *auct. non* (Lightf.) Poetsch, *Thalloidima vesiculosum* M. Choisy, Toninia caeruleonigricans *auct. non* (Lightf.) Th. Fr., *Toninia carolitana* (Arnold) Nimis & Poelt, *Toninia muricola* B. de Lesd., *Toninia subtabacina* (Nyl.) H. Olivier, *Toninia vesicularis* (Hoffm.) Boistel, *Verrucaria grisea* Willd.

L – Subs.: ter-cal, cal – Alt.: 1–6 – Note: a widespread holarctic lichen with a broad altitudinal and latitudinal range found on soil and weathered calciferous, more rarely basic siliceous rocks, often overgrowing mosses and associated with cyanobacteria or cyanobacterial lichens when young; common in dry, open grasslands throughout the Alps. – **Au**: V, T, S, K, St, O, N, B. **Ge**: OB. **Sw**: BE, FR, GR, LU, SZ, TI, UR, UW, VD, VS. **Fr**: AHP, HAl, AMa, Drô, Isè, Sav, HSav, Var, Vau. **It**: Frl, Ven, TAA, Lomb, Piem, VA, Lig. **Sl**: SlA, Tg.

***Toninia squalescens* (Nyl.) Th. Fr.**

Syn.: *Lecidea squalescens* Nyl., *Thalloidima rimulosum* Th. Fr.

L # – Subs.: bry-sil – Alt.: 4–5 – Note: on silicicolous mosses, mostly on *Andreaea* near or above treeline; on the whole, a rather poorly known species which certainly does not belong to *Toninia*. – **Au**: V, T, S. **Sw**: VS. **It**: Lomb.

***Toninia squalida* (Ach.) A. Massal.**

Syn.: *Bacidia acervulans* (Nyl.) B. de Lesd., *Bilimbia caulescens* (Anzi) Jatta, *Bilimbia multiseptata* (Anzi) Jatta, *Bilimbia squalida* (Ach.) Jatta, *Lecidea acervulan*s Nyl., *Lecidea caulescens* (Anzi) Tuck., *Lecidea norvegica* Sommerf., *Lecidea squalida* Ach., *Toninia acervulans* (Nyl.) H. Olivier, *Toninia catalaunica* V. Wirth & Llimona, *Toninia caulescens* Anzi, Toninia cinereovirens (Schaer.) A. Massal. var. verruculosa Th. Fr., *Toninia havaasii* H. Magn., *Toninia multiseptata* Anzi, *Toninia squarrosa* (Ach.) Th. Fr., *Toninia verruculosa* (Th. Fr.) Vain.

L – Subs.: sil, int, cal, bry-sil, bry-cal, ter-sil, ter-cal – Alt.: 3–5 – Note: an incompletely holarctic lichen with a very broad latitudinal range, found on soil, more rarely on weathered base-rich or weakly calciferous siliceous rocks in dry-warm upland areas, often associated to cyanobacteria or cyanobacterial lichen when young. – **Au**: T, S, K, St. **Sw**: BE, GR, SZ, UR, VS. **Fr**: AHP, HAl, Isè, HSav, Var. **It**: Frl, Ven, TAA, Lomb, Piem, VA.

***Toninia subnitida* (Hellb.) Hafellner & Türk**

Syn.: *Catillaria subnitida* Hellb., *Catillaria tristis* (Müll. Arg.) Arnold, *Kiliasia tristis* (Müll. Arg.) Hafellner *non Toninia tristis* (Th. Fr.) Th. Fr., *Lecidea platycarpiza* Nyl., *Patellaria tristis* Müll. Arg.

L – Subs.: cal – Alt.: 3–5 – Note: on calcareous substrata; the generic position is still an open problem. – **Au**: V, T, St, N. **Ge**: OB, Schw. **Sw**: GR, SZ, UR, UW. **Fr**: HAl, Sav. **It**: TAA, Piem.

***Toninia taurica* (Szatala) Oxner**

Syn.: *Thalloidima tauricum* Szatala, *Toninia clemens* H. Baumgärtner, *Toninia schafeevii* Tomin

L – Subs.: cal, ter-cal – Alt.: 1–5 – Note: a mainly southern species with an Eurasiatic distribution found on calciferous soil and in fine crevices of the rocks, often associated with cyanobacterial lichens when young, with a wide altitudinal range. – **Au**: V, T, K, St, O, N. **Sw**: BE, GR, VD, VS. **Fr**: AHP, HAl, AMa, Isè, Sav, Var, Vau. **It**: Frl, Ven, TAA, Lomb, Piem, Lig.

***Toninia toniniana* (A. Massal.) Zahlbr.**

Syn.: *Biatorina toniniana* (A. Massal.) Jatta, *Lecidea caesiocandida* Nyl., *Thalloidima caesiocandidum* (Nyl.) Arnold, Thalloidima mammillare (Gouan) A. Massal. var. toninianum A. Massal., *Thalloidima toninianum* (A. Massal.) A. Massal., *Toninia caesiocandida* (Nyl.) Th. Fr.

L – Subs.: cal, ter-cal – Alt.: 2–4 – Note: a mainly Mediterranean to submediterranean species found on steeply inclined to slightly underhanging seepage tracks of calcareous rocks, always in association with cyanobacterial colonies. – **Au**: T, S, K, St, O, N. **Sw**: GR, VS. **Fr**: AHP, AMa, HSav, Vau. **It**: Frl, Ven, TAA, Lomb, Piem, VA.

**Toninia tristis (Th. Fr.) Th. Fr. subsp. tristis**

Syn.: Psora tabacina DC. var. tristis Th. Fr., Toninia tabacina *auct. non* (DC.) Flagey

L – Subs.: cal, ter-cal – Alt.: 1–5 – Note: in fine crevices of calciferous rocks, with optimum near and above treeline. Here we have placed also all records of *T. tristis* without specification of the subspecies. – **Au**: V, T, S, St, N. **Sw**: GR, TI, VS. **Fr**: HAl, Vau. **It**: Lomb, VA. **Li**.

**Toninia tristis (Th. Fr.) Th. Fr. subsp. asiae-centralis (H. Magn.) Timdal**

Syn.: *Lecidea asiae-centralis* H. Magn.

L – Subs.: cal, ter-cal – Alt.: 2–4 – Note: on calciferous rocks and soil; despite the name, this subspecies is widespread also in Southern and Central Europe, with scattered outposts north to Greenland. – **Au**: T. **Sw**: GR, VS. **Fr**: AHP, HAl, AMa, Sav, Var, Vau. **It**: Frl, TAA, Lomb, Piem.

**Toninia tristis (Th. Fr.) Th. Fr. subsp. pseudotabacina Timdal**

L – Subs.: ter-cal – Alt.: 2–5 – Note: a mainly Mediterranean-Macaronesian taxon found on soil over calcareous substrata. – **Au**: T. **Sw**: TI, UR. **Fr**: AMa, Vau. **It**: Ven, TAA, Lomb, Lig.

**Toninia tristis (Th. Fr.) Th. Fr. subsp. scholanderi (Lynge) Timdal**

Syn.: *Lecidea scholanderi* Lynge

L – Subs.: ter-cal – Alt.: 4 – Note: a variety with squamules as in subsp. tristis, but with a brown epithecium, a hypothecium lacking orange pigments, and simple ascospores; on soil in seasonally dry open situations; widespread in the Northern Hemisphere, with a single record from the Eastern Alps (Austria). – **Au**: T.

**Toninia tristis (Th. Fr.) Th. Fr. subsp. thalloedaemiformis (Szatala) Timdal**

Syn.: *Lecidea thalloedaemiformis* Szatala

L – Subs.: ter-cal – Alt.: 2 – Note: a variety with contiguous squamules which are larger than in subsp. tristis, a bright green epithecium, and simple ascospores; more or less confined to steep faces of calciferous rocks; widespread in the eastern Mediterranean region, the terricolous record from the Western Alps (France) needs confirmation. – **Fr**: AMa.

***Toninia verrucarioides* (Nyl.) Timdal**

Syn.: *Bilimbia carbonacea* (Anzi) Jatta, Lecidea aromatica var. verrucarioides Nyl., *Lecidea subimbricata* Nyl., *Lecidea verrucarioides* (Nyl.) Nyl., *Thalloidima boissieri* Müll. Arg., *Thalloidima carbonacea* Anzi, Toninia aromatica (Sm.) A. Massal. var. cervina (Lönnr.) Th. Fr., *Toninia boissieri* (Müll. Arg.) Arnold, *Toninia carbonacea* Anzi, *Toninia cervina* Lönnr., *Toninia conjungens* Th. Fr., *Toninia kolax* Poelt, *Toninia subimbricata* (Nyl.) H. Olivier

L – Subs.: cal-par, cal – Alt.: 3–5 – Note: an arctic-alpine to cool-temperate lichen found in fissures and fine crevices of calcareous rocks in upland areas, often growing on species of *Placynthium* when young. – **Au**: V, T, K, St, O, N. **Ge**: Ge. **Sw**: LU, TI. **Fr**: Drô, HSav, Vau. **It**: Lomb, Lig.

***Toniniopsis obscura* Frey**

L – Subs.: ter-cal – Alt.: 4–5 – Note: a peculiar, inconspicuous lichen somewhat recalling a *Placynthiella*-species, with a blackish-brown, minutely granulose thallus, small, brownish-black apothecia, and bacilliform, finally 3-septate ascospores (to *c.* 25 µm long); on plant debris and decaying bryophytes over dolomite and calcareous rocks, from the subalpine to the lower alpine belt; known from a few localities in the Alps, but easily overlooked, and perhaps more widespread. – **Au**: T, S, K, O. **Sw**: GR.

***Topelia heterospora* (Zahlbr.) P.M. Jørg. & Vězda**

Syn.: *Clathroporina heterospora* Zahlbr.

L – Subs.: cal – Alt.: 2 – Note: a humid subtropical to Mediterranean-Atlantic lichen found on hard, compact calcareous rocks in sheltered situations, also reported from the base of the SW Pre-Alps (France). – **Fr**: AMa.

***Trapelia coarctata* (Sm.) M. Choisy**

Syn.: *Biatora arridens* (Nyl.) Walt. Watson, *Biatora coarctata* (Sm.) Th. Fr., Biatora coarctata (Sm.) Th. Fr. var. elachista (Ach.) Th. Fr., ?*Biatora comensis* Anzi, *Lecanactis arridens* Nyl., *Lecanora coarctata* (Sm.) Ach., Lecanora coarctata (Sm.) Ach. var. elachista (Ach.) Schaer., *Lecidea arridens* Nyl., *Lecidea coarctata* (Sm.) Nyl., Lecidea coarctata (Sm.) Nyl. var. elachista (Ach.) Th. Fr., *Lichen coarctatus* Sm., *Zeora coarctata* (Sm.) Flot.

L – Subs.: sil, sil-aqu – Alt.: 1–5 – Note: a holarctic early coloniser of siliceous pebbles near the soil surface, sometimes also found on bare clayey soil, with a wide altitudinal and altitudinal range; it is most frequent in upland areas, becoming rare in the eu-Mediterranean belt. The species is genetically heterogeneous; widespread throughout the Alps. – **Au**: V, T, S, K, St, O, N, B. **Ge**: OB, Schw. **Sw**: BE, GR, LU, SZ, TI, UR, VD, VS. **Fr**: HAl, AMa, Isè, Sav, HSav, Var, Vau. **It**: Frl, Ven, TAA, Lomb, Piem, VA. **Sl**: SlA, Tg.

***Trapelia corticola* Coppins & P. James**

L – Subs.: cor, xyl – Alt.: 2–3 – Note: on the spongy, loose bark of deciduous trees, sometimes on moribund epiphytic bryophytes in sheltered, humid woodlands at low elevations; from the Alps there are only a few scattered records. – **Sw**: GL, SZ, UW. **It**: Lomb.

***Trapelia glebulosa* (Sm.) J.R. Laundon**

Syn.: Biatora coarctata (Sm.) Th. Fr. var. glebulosa (Sm.) Arnold, Biatora coarctata (Sm.) Th. Fr. var. ornata (Sommerf.) Th. Fr., Lecanora coarctata (Sm.) Ach. var. involuta (Taylor) Mudd., Lecanora coarctata (Sm.) Ach. var. ornata Sommerf., *Lecanora involuta* Taylor, Lecidea coarctata (Sm.) Nyl. var. glebulosa (Sm.) Mudd, Lecidea coarctata (Sm.) Nyl. var. ornata (Sommerf.) Malbr., *Lecidea glebulosa* (Sm.) Jatta, *Lecidea ornata* (Sommerf.) Hue, *Lichen glebulosus* Sm., *Trapelia involuta* (Taylor) Hertel, *Trapelia ornata* (Sommerf.) Hertel

L – Subs.: sil, xyl – Alt.: 1–5 – Note: a species with a minutely squamulose thallus, not easy to distinguish from some forms of *T. coarctata*; on basic siliceous rocks and various types of schists, roofing tiles, brick walls, mostly close to the ground, often together with *T. coarctata*; widespread in the Holarctic region, but not in the extreme north; widespread throughout the Alps. – **Au**: V, T, S, K, St, O, N, B. **Ge**: OB, Schw. **Sw**: LU, SZ, TI. **Fr**: AMa, HSav, Var, Vau. **It**: Frl, Ven, Lomb, Piem. **Sl**: SlA.

***Trapelia obtegens* (Th. Fr.) Hertel**

Syn.: Biatora coarctata (Sm.) Th. Fr. subsp. obtegens Th. Fr., Biatora coarctata (Sm.) Th. Fr. var. obtegens (Th. Fr.) Th. Fr., Lecidea coarctata (Sm.) Nyl. var. obtegens (Th. Fr.) Th. Fr., *Lecidea obtegens* (Th. Fr.) Vain.

L – Subs.: sil – Alt.: 2–4 – Note: on siliceous pebbles near the ground, sometimes on roofing tiles and over thin soil layers, with scattered records from the Eastern Alps only. – **Au**: S, K, St, N. **It**: Frl. **Sl**: SlA.

***Trapelia placodioides* Coppins & P. James**

L – Subs.: sil – Alt.: 1–5 – Note: on base-rich or slightly calciferous siliceous substrata, sometimes also on walls, in humid areas, with optimum below the montane belt; probably more widespread in the Alps, but never common. – **Au**: V, T, S, St, O, N, B. **Sw**: GR, LU, SZ, VS. **It**: TAA, Piem. **Sl**: SlA.

***Trapeliopsis aeneofusca* (Flörke *ex* Flot.) Coppins & P. James**

Syn.: *Biatora aeneofusca* (Flörke *ex* Flot.) Arnold, *Lecidea aeneofusca* Flörke *ex* Flot., *Lecidea prasinorufa* Nyl.

L – Subs.: ter-sil, bry – Alt.: 3–4 – Note: very similar to *T. gelatinosa*, but apothecia in various shades of brown (instead of blackish green), sterile specimens therefore indistinguishable; on soil, rare throughout Europe and North America, with a few scattered records from the Alps. – **Au**: T, O, N. **Sw**: LU. **Fr**: HAl.

***Trapeliopsis flexuosa* (Fr.) Coppins & P. James**

Syn.: *Biatora flexuosa* Fr., *Lecidea aeruginosa* Borrer, *Lecidea flexuosa* (Fr.) Nyl., Lecidea granulosa (Hoffm.) Ach. subsp. flexuosa (Fr.) Th. Fr., *Lecidea sapinea* (Fr.) Zahlbr. *non* *sensu* Vain., *Lecidea sporodiza* Stirt.

L – Subs.: xyl, cor, ter-sil – Alt.: 2–5 – Note: a temperate to boreal-montane, circumpolar lichen found on lignum (often on wooden fences) and acid bark, especially of *Pinus* and *Castanea*; widespread and often common throughout the Alps. – **Au**: V, T, S, K, St, O, N, B. **Ge**: OB. **Sw**: AP, BE, FR, GR, LU, SG, SZ, TI, UR, UW, VD, VS. **Fr**: AHP, AMa, Sav, HSav, Var, Vau. **It**: Frl, Ven, TAA, Lomb, Piem, Lig. **Sl**: SlA, Tg. **Li**.

***Trapeliopsis gelatinosa* (Flörke) Coppins & P. James**

Syn.: *Biatora gelatinosa* (Flörke) Flot., Biatora viridescens (Schrad.) W. Mann var. gelatinosa (Flörke) Fr., *Lecidea gelatinosa* Flörke, *Micarea gelatinosa* (Flörke) Brodo

L – Subs.: ter-sil, bry – Alt.: 2–5 – Note: a boreal-montane to cool-temperate early coloniser of mineral acid soil, sometimes overgrowing bryophytes and plant debris, mostly in upland areas; widespread throughout the Alps. – **Au**: V, T, S, K, St, O, N, B. **Ge**: OB, Schw. **Sw**: BE, GR, LU, SZ, TI, UR, VD, VS. **Fr**: HAl, HSav. **It**: Frl, TAA, Lomb, Piem, Lig. **Sl**: SlA.

***Trapeliopsis glaucolepidea* (Nyl.) Gotth. Schneid.**

Syn.: *Lecidea glaucolepidea* Nyl., *Lecidea percrenata* Nyl., *Trapelia percrenata* (Nyl.) V. Wirth, *Trapeliopsis percrenata* (Nyl.) Gotth. Schneid.

L – Subs.: deb, ter-sil, xyl – Alt.: 3–4 – Note: a species with a thallus resembling the primary thallus of a *Cladonia*, the grey to greenish squamules with usually lip-shaped soralia, mostly sterile; on plant debris and rotting wood; widespread worldwide, in Central Europe in montane forests, in the Alps still overlooked in some regions. – **Au**: T, S, K, St, N. **Sw**: LU.

***Trapeliopsis granulosa* (Hoffm.) Lumbsch**

Syn.: *Biatora decolorans* *auct.*, *Biatora granulosa* (Hoffm.) Flot., *Lecidea decolorans* *auct.*, *Lecidea granulosa* (Hoffm.) Ach., *Lecidea quadricolor* (Dicks.) Borrer, *Trapelia granulosa* (Hoffm.) V. Wirth, *Verrucaria granulosa* Hoffm.

L – Subs.: xyl, deb, bry, ter-sil, bry-sil – Alt.: 2–5 – Note: an arctic-alpine to cool-temperate, circumpolar lichen mostly found on rotting wood, more rarely on soil rich in humus, bryophytes and peat, mostly in clearings of grasslands and shrublands, with optimum near treeline; widespread throughout the Alps. – **Au**: V, T, S, K, St, O, N, B. **Ge**: OB, Schw. **Sw**: BE, GR, LU, SZ, TI, UR, VD, VS. **Fr**: AHP, HAl, AMa, Isè, Sav, HSav, Var, Vau. **It**: Frl, Ven, TAA, Lomb, Piem, VA, Lig. **Sl**: SlA, Tg.

***Trapeliopsis pseudogranulosa* Coppins & P. James**

L – Subs.: bry, deb, ter-sil – Alt.: 2–4 – Note: this lichens is most frequent in humid *Castanea* woodlands, on mosses on basal parts of trunks, decaying lignum and acid organic soil, especially in areas with siliceous substrata; certainly more widespread in the Alps. – **Au**: T, S, K, St, O, N. **Ge**: OB. **Sw**: BE, GL, SG, SZ, TI, VD, VS. **Fr**: HAl, AMa. **Sl**: Tg.

***Trapeliopsis viridescens* (Schrad.) Coppins & P. James**

Syn.: *Biatora viridescens* (Schrad.) W. Mann, *Lecidea viridescens* (Schrad.) Ach., *Lichen viridescens* Schrad., *Micarea viridescens* (Schrad.) Brodo, *Trapelia viridescens* (Schrad.) V. Wirth

L – Subs.: bry, deb, ter-sil, xyl – Alt.: 2–4 – Note: a mainly boreal-montane lichen found on rotting, soft lignum, sometimes overgrowing mosses, mostly in coniferous forests or in *Castanea*-stands. – **Au**: V, S, K, St, O, N, B. **Ge**: OB. **Sw**: BE, VD. **It**: TAA, Lomb, Piem. **Sl**: SlA.

***Trapeliopsis wallrothii* (Flörke *ex* Spreng.) Hertel & Gotth. Schneid.**

Syn.: *Biatora glebulosa* Fr., *Biatora wallrothii* (Flörke *ex* Spreng.) Körb., *Lecidea wallrothii* Flörke *ex* Spreng., *Trapelia wallrothii* (Flörke *ex* Spreng.) V. Wirth

L – Subs.: ter-sil, bry, bry-sil – Alt.: 1–3 – Note: on base-rich, non – or weakly calciferous soil, sometimes overgrowing mosses, mostly in open situations, with optimum below the montane belt, with several scattered records from the Alps. – **Au**: T, S. **Sw**: LU, UW, VD. **Fr**: HSav, Var. **It**: Lomb, Piem.

***Tremolecia atrata* (Ach.) Hertel**

Syn.: *Aspicilia melanophaea* (Fr.) Körb., *Gyalecta atrata* Ach., *Lecidea atrata* (Ach.) Wahlenb., *Lecidea atroferrata* Branth & Grønlund, *Lecidea circumcisa* H. Magn., Lecidea dicksonii *auct. non* (J.F. Gmel.) Ach., *Lecidea melanophaea* Fr., *Lecidea sincerula* Nyl. *ex* Cromb.

L – Subs.: met, sil – Alt.: 3–6 – Note: a species of cool to cold areas of both Hemispheres, found on hard magmatic and metamorphic rocks often rich in iron, mostly on small boulders in upland areas; widespread and locally common in the Alps. – **Au**: V, T, S, K, St, N. **Ge**: Schw. **Sw**: BE, GR, TI, UR, VD, VS. **Fr**: AHP, HAl, AMa, Isè, Sav, HSav. **It**: Frl, Ven, TAA, Lomb, Piem, VA, Lig.

***Trimmatothele perquisita* (Norman) Norman *ex* Zahlbr.**

Syn.: *Coniothele perquisita* Norman, *Verrucaria perquisita* (Norman) Ertz & Diederich

L – Subs.: cal – Alt.: 3–4 – Note: a calcicolous species with an endolithic to thin, variably coloured thallus, hemispherically protruding ascomata with involucrellum surrounding the perithecium, moderately polyspored asci, and oblong to ellipsoid, simple ascospores (to *c.* 10 µm long). The genus is not generally accepted, and the species was treated in *Verrucaria* by some authors; widespread in Europe, from the boreal to the nemoral-subalpine zone; from the Alps there are a few scattered records only. – **Au**: T, St. **Fr**: Sav.

***Tuckermannopsis chlorophylla* (Willd.) Hale**

Syn.: *Cetraria chlorophylla* (Willd.) Vain., Cetraria scutata *auct. non* (Wulfen) Poetsch, *Cetraria ulophylla* (Ach.) Rebent., *Lichen chlorophyllus* Willd., *Nephromopsis chlorophylla* (Willd.) Divakar, A. Crespo & Lumbsch, *Platysma chlorophyllum* (Willd.) Vain., *Platysma ulophyllum* (Ach.) Nyl.

L – Subs.: cor – Alt.: 2–4 – Note: on isolated conifers (*e.g. Larix* in the subalpine belt), more rarely on old acid-barked deciduous trees in montane forests; widespread throughout the Alps. – **Au**: V, T, S, K, St, O, N, B. **Ge**: OB. **Sw**: BE, FR, GL, GR, LU, SZ, TI, UR, UW, VD, VS. **Fr**: AHP, HAl, AMa, Isè, Sav, HSav, Vau. **It**: Frl, Ven, TAA, Lomb, Piem, VA, Lig. **Sl**: SlA, Tg.

***Umbilicaria aprina* Nyl.**

Syn.: *Gyrophora aprina* (Nyl.) Nyl.

L – Subs.: sil – Alt.: 5–6 – Note: an arctic-alpine species of hard siliceous rocks above treeline; the only record from the Alps is from Mt. Blanc. – **Fr**: HSav.

***Umbilicaria cinerascens* (Arnold) Frey**

Syn.: *Gyrophora cinerascens* Arnold

L – Subs.: sil – Alt.: 4–6 – Note: on steeply inclined, often north-facing surfaces of siliceous rocks, mostly in small colonies, with optimum above treeline; several records from Switzerland need confirmation. – **Au**: V, T, S, K, St. **Sw**: BE, GL, GR, SG, UR, VS. **Fr**: AHP, HAl, AMa, Isè, Sav, HSav. **It**: TAA, Lomb, Piem, VA.

***Umbilicaria cinereorufescens* (Schaer.) Frey**

Syn.: *Gyrophora cinereorufescens* (Schaer.) Schol., *Gyrophora mammulata* Ach., Umbilicaria vellea (L.) Ach. f. cinereorufescens Schaer.

L – Subs.: sil – Alt.: 3–6 – Note: a holarctic species also known from the mountains of Africa found on wind-exposed, vertical or slightly underhanging surfaces of hard siliceous rocks in humid upland areas (frequent fog and high rainfall), but in apparently dry situations. – **Au**: V, T, S, K, St. **Sw**: BE, GR, UR, VD, VS. **Fr**: AHP, HAl, Sav, HSav. **It**: TAA, Lomb, Piem, VA.

***Umbilicaria corsica* Frey (not validly published, ICN Art. 36.1(b))**

Syn.: *Gyrophora corsica* (Frey) Schol. *nom. inval.*, *Omphalodiscus corsicus* (Frey) Llano *nom. inval*.

L – Subs.: sil – Alt.: 4 – Note: a silicicolous species resembling *U. virginis*, with small (to 3 cm in diam.), monophyllous thalli which are whitish grey above and pale and hirsute below, apothecia plane at first finally with plicate discs, ascospores small, non-septate, hyaline; only known from Corsica and the Western Alps (France). – **Fr**: HAl.

**Umbilicaria crustulosa (Ach.) Lamy var. crustulosa**

Syn.: *Gyrophora crustulosa* Ach., *Gyrophora depressa* (Ach.) Röhl., Gyrophora depressa (Ach.) Röhl. var. crustulosa (Ach.) Dalla Torre & Sarnth., *Omphalodiscus crustulosus* (Ach.) Schol., *Umbilicaria spadochroa* *auct. medioeur.* *p.p. non* Ehrh. *ex* Hoffm.

L – Subs.: sil – Alt.: 3–6 – Note: a cool-temperate to arctic-alpine, circumpolar lichen found on exposed, often steeply inclined surfaces of siliceous rocks with some water seepage in upland areas; widespread throughout the siliceous Alps. – **Au**: V, T, S, K, St. **Sw**: BE, GR, TI, UR, VD, VS. **Fr**: AHP, HAl, AMa, Isè, Sav, HSav, Var. **It**: Frl, Ven, TAA, Lomb, Piem, VA.

**Umbilicaria crustulosa (Ach.) Lamy var. badiofusca Frey**

Syn.: *Gyrophora hirsuta* (Sw. *ex* Westr.) Ach. var. meizospora (Harm.) H. Olivier, *Gyrophoropsis meizospora* (Harm.) M. Choisy, *Umbilicaria hirsuta* (Sw. *ex* Westr.) Hoffm. var. meizospora Harm.

L # – Subs.: sil – Alt.: 2–5 – Note: a taxon of the mountains of Southern Europe, worthy of further study. – **Fr**: Isè, Sav. **It**: Lomb, Piem, VA.

***Umbilicaria cylindrica* (L.) Delise *s.lat.***

Syn.: *Gyrophora cylindrica* (L.) Ach., Gyrophora cylindrica (L.) Ach. var. delisei (Nyl.) Syd., Gyrophora cylindrica (L.) Ach. var. nudiuscula (Schaer.) Zahlbr, Gyrophora polymorpha Schrad. var. cylindrica Schaer. f. nudiuscula Schaer., *Gyrophora tornata* Ach., *Lichen cylindricus* L., *Umbilicaria canescens* (Dombr.) Dombr., *Umbilicaria crinita* Hoffm., *Umbilicaria cylindrica* (L.) Delise, Umbilicaria cylindrica (L.) Delise var. corrugatoides Frey, Umbilicaria cylindrica (L.) Delise var. delisei Despr. *ex* Nyl. *nom.illeg.*, Umbilicaria cylindrica (L.) Delise var. fimbriata (Ach.) Nyl., Umbilicaria cylindrica (L.) Delise var. mesenteriformis (Wulfen) Ozenda & Clauzade, Umbilicaria cylindrica (L.) Delise var. nudiuscula (Schaer.) Ozenda & Clauzade, Umbilicaria cylindrica (L.) Delise var. tornata (Ach.) Nyl., *Umbilicaria delisei* Despr. *ex* Nyl. *nom.illeg*.

L – Subs.: sil, int – Alt.: 3–6 – Note: an ecologically wide-ranging, cool-temperate to arctic-alpine, circumpolar lichen found on wind-exposed boulders with a short snow-covering period, often on or near the top, with optimum above treeline. The species is highly polymorphic, with several infraspecific taxa; widespread throughout the siliceous Alps. – **Au**: V, T, S, K, St, O, N. **Ge**: OB, Schw. **Sw**: BE, GR, LU, SZ, TI, UR, UW, VD, VS. **Fr**: AHP, HAl, AMa, Isè, Sav, HSav. **It**: Frl, Ven, TAA, Lomb, Piem, VA, Lig. **Sl**: SlA.

***Umbilicaria decussata* (Vill.) Zahlbr.**

Syn.: *Gyrophora decussata* (Vill.) Zahbr., *Gyrophora discolor* Th. Fr., *Gyrophora ptychophora* (Nyl.) Nyl., *Lichen decussatus* Vill., *Omphalodiscus decussatus* (Vill.) Schol., *Umbilicaria ptychophora* Nyl., *Umbilicaria reticulata* (Schaer.) Carestia *ex* Bagl. & Carestia

L – Subs.: sil – Alt.: 3–6 – Note: an arctic-alpine to boreal-montane, circumpolar lichen found on steeply inclined to slightly underhanging surfaces of wind-exposed siliceous rocks; widespread throughout the siliceous Alps. – **Au**: V, T, S, K, St. **Sw**: BE, GR, UR, VS. **Fr**: AHP, HAl, AMa, Isè, Sav, HSav. **It**: Ven, TAA, Lomb, Piem, VA.

***Umbilicaria dendrophora* (Poelt) Hestmark**

Syn.: Umbilicaria vellea (L.) Ach. var. dendrophora Poelt

L – Subs.: sil – Alt.: 5 – Note: a species resembling *U. vellea*, but with different thalloconidia plus other anatomical differences (algal layer discontinuous and medulla sharply separated from lower cortex), lacking lichen substances; on steeply inclined faces of siliceous cliffs and large boulders; widespread in Europe but rather rare, from the subarctic zone to the alpine belt; in the study area only known from the Eastern Alps (Austria). – **Au**: K.

***Umbilicaria deusta* (L.) Baumg.**

Syn.: Gyrophora aenea Schaer. var. flocculosa (Wulfen) Schaer., *Gyrophora deusta* (L.) Ach., *Gyrophora flocculosa* (Wulfen) Turner & Borrer, Gyrophora polyphylla (L.) Funck var. deusta (L.) Rabenh., *Lichen deustus* L., *Umbilicaria flocculosa* (Wulfen) Hoffm.

L – Subs.: sil – Alt.: 2–5 – Note: an arctic-alpine to boreal-montane, circumpolar lichen found on rocks wetted by rain near the ground, in sites with a long snow cover; one of the most common *Umbilicaria* throughout the Alps. – **Au**: V, T, S, K, St, N. **Ge**: Schw. **Sw**: BE, GR, LU, SZ, TI, UR, VD, VS. **Fr**: AHP, HAl, AMa, Isè, Sav, HSav. **It**: Frl, Ven, TAA, Lomb, Piem, VA, Lig. **Sl**: SlA.

***Umbilicaria freyi* Codogno, Poelt & Puntillo**

Syn.: Umbilicaria grisea Hoffm. f. subpapyria Frey, *Umbilicaria hirsuta* (Sw. *ex* Westr.) Hoffm. var. pyrenaica Frey

L – Subs.: sil – Alt.: 3–4 – Note: on steeply inclined to underhanging surfaces of siliceous rocks, ecologically intermediate between *U. grisea* and *U. deusta*; perhaps more widespread in the Alps. – **Fr**: AMa. **It**: VA.

***Umbilicaria grisea* Hoffm.**

Syn.: *Gyrophora grisea* (Hoffm.) Turner & Borrer, *Gyrophora hirsuta* (Sw. *ex* Westr.) Ach. var. grisea (Hoffm.) Th. Fr., *Gyrophora murina* (Ach.) Ach., *Umbilicaria murina* (Ach.) DC.

L – Subs.: sil – Alt.: 2–4 – Note: on steeply inclined to underhanging surfaces of siliceous rocks only slightly wetted after rain, usually below the alpine belt; several records from the Alps require confirmation. – **Au**: ?T. **Sw**: ?GR, ?TI, ?VS. **Fr**: HAl, Var, Vau. **It**: TAA, Lomb, Piem, VA.

***Umbilicaria hirsuta* (Sw. *ex* Westr.) Hoffm.**

Syn.: *Gyrophora hirsuta* (Sw. *ex* Westr.) Ach., Gyrophora vellea (L.) Ach. var. hirsuta (Sw. *ex* Westr.) Rabenh., *Lichen hirsutus* Sw. *ex* Westr.

L – Subs.: sil – Alt.: 2–6 – Note: an arctic-alpine to boreal-montane, circumpolar lichen found on steeply inclined to slightly underhanging surfaces of siliceous rocks, often in somehow dusty situations; widespread and common throughout the Alps. – **Au**: V, T, S, K, St. **Sw**: BE, GR, TI, UR, VS. **Fr**: HAl, AMa, Isè, Sav, HSav. **It**: Frl, TAA, Lomb, Piem, VA, Lig. **Sl**: SlA.

***Umbilicaria hyperborea* (Ach.) Hoffm.**

Syn.: Gyrophora aenea Schaer. var. hyperborea (Ach.) Schaer., *Gyrophora hyperborea* (Ach.) Ach., *Gyrophora ustulata* (Vain.) Dalla Torre & Sarnth., *Lichen hyperboreus* Ach.

L – Subs.: sil – Alt.: 3–6 – Note: an arctic-alpine, probably circumpolar lichen found on siliceous boulders wetted by rain, usually near the ground, with optimum near treeline. – **Au**: V, T, S, K, St. **Sw**: BE, GR, UR, VS. **Fr**: Isè, HSav. **It**: Ven, TAA, Lomb, Piem, VA.

***Umbilicaria laevis* (Schaer.) Frey**

Syn.: *Agyrophora laevis* (Schaer.) Llano, Gyrophora atropruinosa (Schaer.) L. Mangin var. laevis Schaer., *Gyrophora laevis* (Schaer.) Du Rietz

L – Subs.: sil – Alt.: 3–6 – Note: on inclined, sun-exposed surfaces of siliceous rocks, generally in dry situations, with optimum above treeline. – **Au**: T, K, St. **Ge**: OB, Schw. **Sw**: BE, GR, UR, VD, VS. **Fr**: AHP, HAl, AMa. **It**: TAA, Lomb, Piem, VA.

***Umbilicaria leiocarpa* DC.**

Syn.: *Agyrophora leiocarpa* (DC.) Gyeln., *Agyrophora lyngei* (Schol.) Llano, *Gyrophora leiocarpa* (DC.) Du Rietz, *Umbilicaria atropruinosa* Schaer., *Umbilicaria lyngei* Schol.

L – Subs.: sil – Alt.: 4–6 – Note: a mainly arctic-alpine, probably circumpolar lichen found on vertical, wind – and sun-exposed surfaces of large siliceous boulders wetted by rain with a short snow cover period, with optimum above treeline. – **Au**: V, T, S, K, St. **Sw**: BE, GR, UR, VS. **Fr**: HAl, AMa, Isè, HSav. **It**: TAA, Lomb, Piem, VA.

***Umbilicaria maculata* Krzewicka, M.P. Martín & M.A. García**

L – Subs.: sil – Alt.: 4–5 – Note: a species of the *U. cylindrica*-group with monophyllous thalli tightly adhering to the rock, the grey to grey-brown upper surface with whitish maculae, a whitish to creamy rhizinate lower surface, and sessile, omphalodisc apothecia; on vertical rock faces of boulders and cliffs in shaded, windy situations at high elevations; rare in the Tatra Mountains, with a single record from the Western Alps (France), but perhaps not recognised elsewhere. – **Fr**: AHP.

***Umbilicaria microphylla* (Laurer) A. Massal.**

Syn.: *Agyrophora microphylla* (Laurer) Llano, *Gyrophora microphylla* (Laurer) Arnold, Umbilicaria atropruinosa Schaer. var. microphylla Laurer

L – Subs.: sil – Alt.: 4–6 – Note: on steeply inclined, wind-exposed surfaces of hard siliceous rocks, often forming monospecific stands, with optimum above treeline. – **Au**: ?V, T, S, K, St. **Sw**: BE, GR, VD, VS. **Fr**: AHP, HAl, AMa, HSav. **It**: TAA, Lomb, Piem, VA.

***Umbilicaria nylanderiana* (Zahlbr.) H. Magn.**

Syn.: *Gyrophora corrugata* (Ach.) Lamy *nom.illeg.*, *Gyrophora nylanderiana* Zahlbr., *Umbilicaria corrugata* (Ach.) Nyl.

L – Subs.: sil – Alt.: 3–6 – Note: an arctic-alpine, probably circumpolar lichen found on the top of isolated siliceous boulders, most frequent above treeline. – **Au**: V, T, S, K, St, N. **Sw**: BE, GR, TI, UR, VD, VS. **Fr**: AHP, HAl, AMa, Isè, Sav, HSav. **It**: TAA, Lomb, Piem, VA.

***Umbilicaria pallens* Poelt**

Syn.: Gyrophora cinerascens Arnold var. pallens (Nyl.) Lamy, Umbilicaria atropruinosa Schaer. var. pallens Nyl., Umbilicaria subglabra (Nyl.) Harm. var. pallens (Nyl.) Frey

L – Subs.: sil – Alt.: 4–5 – Note: a silicicolous species resembling *U. subglabra*, but lacking thalloconidia and lower surface therefore pale greyish, regularly bearing apothecia with smooth discs; widespread in the mountains of SW Europe, with several scattered records from the Alps. – **Au**: T. **Sw**: VD, VS. **Fr**: AHP, HAl, AMa, Isè.

***Umbilicaria polyphylla* (L.) Baumg.**

Syn.: Gyrophora aenea Schaer. var. glabra (Ach.) Schaer., *Gyrophora anthracina* (Wulfen) Körb., *Gyrophora glabra* (Ach.) Ach., *Gyrophora polyphylla* (L.) Funck, *Lichen polyphyllus* L., *Umbilicaria anthracina* (Wulfen) Hoffm., *Umbilicaria glabra* Ach.

L – Subs.: sil – Alt.: 2–5 – Note: a variable and ecologically wide-ranging species of rainy areas, with optimum on inclined surfaces of siliceous rocks wetted by rain; widespread throughout the Alps. – **Au**: V, T, S, K, St, N. **Sw**: BE, GR, LU, TI, UR, VD, VS. **Fr**: AHP, HAl, AMa, Isè, Sav, HSav, Vau. **It**: Frl, Ven, TAA, Lomb, Piem, VA. **Sl**: SlA.

***Umbilicaria polyrrhiza* (L.) Fr.**

Syn.: *Actynogyra polyrrhiza* (L.) Schol., *Gyrophora pellita* (Ach.) Ach., *Gyrophora polyrrhiza* (L.) Körb., *Lichen polyrrhizos* L., *Umbilicaria pellita* (Ach.) Ach.

L – Subs.: sil – Alt.: 2–5 – Note: on steeply inclined surfaces of siliceous rocks wetted by rain in humid-rainy areas, with optimum near and above treeline; several records from the Alps require confirmation. – **Au**: T, S, ?N. **Sw**: ?GR, ?VS. **Fr**: Isè, Sav, HSav. **It**: Piem, VA.

***Umbilicaria proboscidea* (L.) Schrad.**

Syn.: Gyrophora polymorpha Schrad. var. proboscidea (L.) Schaer., *Gyrophora proboscidea* (L.) Ach., *Lichen proboscideus* L.

L – Subs.: sil – Alt.: 3–5 – Note: a mainly arctic-alpine, circumpolar lichen found on siliceous rocks, often on small boulders, ecologically similar to *U. cylindrica*, but with a narrower range, with optimum in colder and less continental areas, mostly above treeline. – **Au**: V, T, S, K, St. **Sw**: BE, GR, TI, UR, VD, VS. **Fr**: Isè, HSav. **It**: TAA, Lomb, Piem, VA.

***Umbilicaria ruebeliana* (Du Rietz & Frey) Frey**

Syn.: *Gyrophora ruebeliana* Du Rietz & Frey, *Omphalodiscus ruebelianus* (Du Rietz & Frey) Schol.

L – Subs.: sil – Alt.: 3–6 – Note: on steeply inclined to underhanging, often south – or west-facing surfaces of siliceous rocks above treeline. – **Au**: T, S. **Sw**: BE, GR, TI, VS. **Fr**: AHP, HAl, AMa. **It**: TAA, Lomb, Piem, VA.

***Umbilicaria subglabra* (Nyl.) Harm.**

Syn.: *Agyrophora subglabra* (Nyl.) M. Choisy, *Gyrophora subglabra* Nyl.; incl. Umbilicaria subglabra (Nyl.) Harm. var. schmidtii Frey

L – Subs.: sil – Alt.: 3–5 – Note: on steeply inclined to horizontal, exposed surfaces of siliceous rocks, often at the top of large boulders; widespread throughout the Alps. – **Au**: ?V, T, S, K, St. **Sw**: GR, UR, VD, VS. **Fr**: AHP, HAl, AMa, Isè. **It**: Frl, TAA, Piem, VA.

***Umbilicaria torrefacta* (Lightf.) Schrad.**

Syn.: *Gyrophora erosa* (Weber) Ach., Gyrophora erosa (Weber) Ach. var. torrefacta (Lightf.) Th. Fr., *Gyrophora torrefacta* (Lightf.) Cromb., *Gyrophora torrida* (Ach.) Röhl., *Lichen torrefactus* Lightf., *Umbilicaria erosa* (Weber) Hoffm., *Umbilicaria torrida* (Ach.) Nyl.

L – Subs.: sil – Alt.: 3–5 – Note: an arctic-alpine, circumpolar lichen of siliceous rocks, most frequent above treeline; widespread, but only locally common, in the Alps. – **Au**:  T, S, K, St, N. **Sw**: BE, GR, UR, VD, VS. **Fr**: Isè, Sav, HSav. **It**: Ven, TAA, Lomb, Piem, VA.

***Umbilicaria vellea* (L.) Ach.**

Syn.: *Gyrophora vellea* (L.) Ach., *Gyrophora vellerea* Nyl., *Lichen velleus* L.

L – Subs.: sil – Alt.: 3–6 – Note: an arctic-alpine, probably circumpolar lichen found on steeply inclined, exposed surfaces of siliceous rocks with some water seepage, especially along fissures, with optimum above treeline; widespread throughout the siliceous Alps. – **Au**: V, T, S, K, St. **Sw**: BE, GR, TI, UR, VS. **Fr**: AHP, HAl, AMa, Isè, HSav. **It**: Frl, Ven, TAA, Lomb, Piem, VA, Lig.

***Umbilicaria virginis* Schaer.**

Syn.: *Gyrophora stipitata* (Nyl.) Branth, *Gyrophora virginis* (Schaer.) Frey, *Omphalodiscus virginis* (Schaer.) Schol., *Umbilicaria rugifera* Nyl., *Umbilicaria stipitata* Nyl.

L – Subs.: sil – Alt.: 6 – Note: an arctic-alpine, probably circumpolar lichen found on wind-exposed siliceous cliffs, often in small niches and under overhangs; strictly limited to the nival belt in the Alps. – **Au**: T, S, K. **Sw**: BE, GR, TI, UR, VS. **Fr**: AHP, HAl, AMa, HSav. **It**: TAA, Lomb, Piem, VA.

***Usnea barbata* (L.) F.H. Wigg.**

Syn.: *Lichen barbatus* L., *Usnea alpina* Motyka, *Usnea catenulata* Motyka, *Usnea caucasica* Vain., *Usnea cembricola* Motyka, *Usnea esthonica* Räsänen, *Usnea freyi* Motyka, *Usnea graciosa* Motyka, *Usnea maxima* Motyka, *Usnea pendulina* Motyka, *Usnea plicata* (L.) F.H. Wigg., Usnea plicata (L.) F.H. Wigg. var. pendulina (Motyka) Clauzade & Cl. Roux, Usnea plicata (L.) F.H. Wigg. var. prostrata (Vain. *ex* Räsänen) Clauzade & Cl. Roux, *Usnea rugulosa* Vain., *Usnea scabrata* Nyl., Usnea scabrata Nyl. var. rugulosa (Vain.) Keissl., *Usnea scrobiculata* Motyka, *Usnea tenax* Motyka, *Usnea tortuosa* De Not.

L – Subs.: cor, xyl, sil – Alt.: 2–4 – Note: a boreal-montane species found in oroboreal-montane forests with high rainfall and frequent fog, especially on branches and twigs of *Picea*. This is one of the most frequent and morphologically most variable species of the genus; widespread throughout the Alps. – **Au**: V, T, S, K, St, O, N. **Ge**: OB. **Sw**: AP, BE, FR, GL, GR, LU, SG, SZ, TI, UR, UW, VD, VS. **Fr**: AHP, HAl, AMa, Isè, Sav, HSav, Vau. **It**: Frl, Ven, TAA, Lomb, Piem, VA. **Sl**: SlA, Tg.

***Usnea cavernosa* Tuck.**

Syn.: *Usnea arnoldiana* Zahlbr., *Usnea lacunosa* Willd., *Usnea microcarpa* Arnold

L – Subs.: cor – Alt.: 3–4 – Note: this species seems to be more frequent in Central and Southern Europe; it is restricted to damp montane to subalpine forests, on branches of coniferous and deciduous trees; widespread throughout the Alps. – **Au**: T, S, K, St, N. **Ge**: OB, Schw. **Sw**: BE, GR, LU, SG, SZ, UR, VD, VS. **Fr**: AHP, AMa, Isè, Sav, HSav. **It**: Frl, TAA, Lomb, Piem, Lig. **Sl**: SlA.

***Usnea ceratina* Ach.**

Syn.: Usnea ceratina Ach. f. incurvescens (Arnold) H. Olivier

L – Subs.: cor – Alt.: 2–4 – Note: a cool-temperate to boreal-montane lichen also known from the Southern Hemisphere found on branches of trees in damp forests with frequent fog; widespread throughout the Alps. – **Au**: T, S, St, O, N. **Ge**: OB. **Sw**: BE, GL, GR, LU, SZ, UR, UW, ?VS. **Fr**: Sav, HSav. **It**: Frl, Ven, TAA, Lomb, Piem, VA, Lig. **Sl**: SlA, Tg.

***Usnea cornuta* Körb.**

Syn.: *Usnea constrictula* Stirt., *Usnea inflata* (Duby) Motyka, Usnea inflata (Duby) Motyka var. cornuta (Körb.) Clauzade & Cl. Roux, Usnea *intexta* Stirt., Usnea intexta Stirt. var. constrictula (Stirt.) D. Hawksw. & D. Chapm., *Usnea subpectinata* Stirt.

L – Subs.: cor, sil – Alt.: 2–3 – Note: a chemically and morphologically variable epiphytic species also rarely occurring on siliceous rocks, restricted to damp sites with frequent fog, mostly in the montane belt. – **Sw**: BE, LU, UR, UW. **Fr**: HAl, Var. **It**: Frl, TAA, Lig.

***Usnea dasaea* Stirt.**

Syn.: *Usnea dolosa* Motyka

L – Subs.: cor – Alt.: 1–3 – Note: a mainly southern species in Europe found on twigs in moist woodlands, exceptionally on rocks, at relatively low elevations, with a few records from the Southern Alps. – **It**: Ven, Lomb. **Sl**: SlA.

***Usnea dasopoga* (Ach.) Nyl.**

Syn.: Usnea barbata (L.) F.H. Wigg. var. dasopoga (Ach.) Fr., *Usnea bicolor* (Motyka) Bystrek, *Usnea capillaris* Motyka, *Usnea dasopoga* (Ach.) Nyl., Usnea dasopoga (Ach.) Nyl. subsp. bicolor Motyka, Usnea dasopoga (Ach.) Nyl. subsp. melanopoga Motyka, Usnea dasopoga (Ach.) Nyl. subsp. stramineola Motyka, Usnea dasopoga (Ach.) Nyl. subsp. tuberculata Motyka, *Usnea diplotypus* Vain., *Usnea fibrillosa* Motyka, *Usnea filipendula* Stirt., Usnea filipendula Stirt. var. capillaris (Motyka) Clauzade & Cl. Roux, *Usnea flagellata* Motyka, *Usnea hirtella* (Arnold) Motyka, *Usnea implexa* Motyka *nom.illeg. non* Hoffm., *Usnea melanopoga* (Motyka) Bystrek, *Usnea meylanii* Motyka, *Usnea muricata* Motyka, Usnea plicata (L.) F.H. Wigg. var. dasopoga Ach., *Usnea saxicola* Anders, *Usnea spuria* (Motyka) Bystrek, *Usnea stramineola* (Motyka) Bystrek, *Usnea sublaxa* Vain., *Usnea subscabrata* (Vain.) Motyka, *Usnea tuberculata* (Motyka) Bystrek

L – Subs.: cor, xyl – Alt.: 2–4 – Note: a variable species, most common in humid montane deciduous forests with frequent fog, both on branches and on boles; *U. diplotypus* *s.str.* corresponds to short morphotypes of *U. dasopoga*; widespread throughout the Alps. – **Au**: V, T, S, K, St, O, N. **Ge**: OB. **Sw**: BE, FR, GL, GR, LU, SG, SZ, TI, UR, UW, VD, VS. **Fr**: AHP, HAl, AMa, Isè, Sav, HSav, Vau. **It**: Frl, Ven, TAA, Lomb, Piem, VA, Lig. **Sl**: SlA, Tg.

***Usnea florida* (L.) F.H. Wigg.**

Syn.: *Lichen floridus* L., Usnea barbata (L.) F.H. Wigg. var. florida (L.) Fr., Usnea florida (L.) F.H. Wigg. subsp. arbuscula Motyka, Usnea florida (L.) F.H. Wigg. subsp. fagofila Motyka, Usnea florida (L.) F.H. Wigg. subsp. pseudostrigosa Motyka, *Usnea tominii* Räsänen

L – Subs.: cor, xyl – Alt.: 3–4 – Note: a boreal-montane to cool-temperate lichen found in forests with frequent fog, on twigs and branches, with optimum in the upper montane and subalpine belts; often confused with *U. intermedia*; see also note on *U. subfloridana*; widespread throughout the Alps, but presently extremely rare and declining. – **Au**: V, T, S, K, St, O, N. **Ge**: OB. **Sw**: BE, GL, ?GR, SG, SZ, UR, UW, VD, ?VS. **Fr**: AHP, Isè, Sav, HSav. **It**: Frl, Ven, TAA, Lomb, Piem, VA, Lig. **Sl**: SlA, Tg.

***Usnea glabrata* (Ach.) Vain.**

Syn.: Usnea barbata (L.) F.H. Wigg. var. sorediifera Arnold, Usnea florida (L.) F.H. Wigg. var. sorediifera (Arnold) Hue, *Usnea kujalae* Räsänen, Usnea plicata (L.) F.H. Wigg. var. glabrata Ach., *Usnea sorediifera* (Arnold) Lynge *non auct*.

L – Subs.: cor, xyl – Alt.: 2–4 – Note: on bark, sometimes on lignum, in cold-humid, but open situations, mostly in the upper montane and subalpine belts; declining throughout the Alps. – **Au**: V, T, S, K, St, N. **Ge**: Ge. **Sw**: BE, GR, LU, TI, VD, VS. **Fr**: AHP, HAl, Isè. **It**: Ven, TAA, Lomb, VA. **Sl**: SlA.

***Usnea glabrescens* (Nyl. *ex* Vain.) Vain. var. glabrescens**

Syn.: Usnea barbata (L.) F.H. Wigg. var. glabrescens Nyl. *ex* Vain., *Usnea compacta* Motyka, *Usnea distincta* Motyka, *Usnea extensa* Vain., *Usnea glabrella* (Motyka) Räsänen, *Usnea laricina* Vain. *ex* Räsänen *non auct*.

L – Subs.: cor, xyl – Alt.: 3–4 – Note: on bark and lignum in cold-humid but open situations in montane to subalpine forests; widespread throughout the Alps. – **Au**: V, T, S, K, St, O, N. **Ge**: OB. **Sw**: BE, GL, GR, SZ, UR, VD. **Fr**: AHP, HAl, AMa, Sav, Var. **It**: Ven, TAA, Lomb, Piem, VA. **Sl**: SlA.

***Usnea glabrescens* (Nyl. *ex* Vain.) Vain. var. fulvoreagens Räsänen**

Syn.: *Usnea fulvoreagens* (Räsänen) Räsänen

L – Subs.: cor, xyl – Alt.: 2–4 – Note: on twigs and branches of conifers, more rarely of deciduous trees in cold-humid, open woodlands with frequent fog; chemically heterogeneous. – **Au**: T, S, K, St, O. **Ge**: OB. **Sw**: UW, ?VS. **Fr**: Var. **It**: Frl, Ven, Lomb. **Sl**: A.

***Usnea hirta* (L.) F.H. Wigg.**

Syn.: *Lichen hirtus* L., Usnea barbata (L.) F.H. Wigg. var. hirta (L.) Fr., Usnea florida (L.) F.H. Wigg. var. hirta (L.) Ach., *Usnea foveata* Vain., *Usnea glaucescens* Vain., Usnea hirta (L.) F.H. Wigg. subsp. helvetica Motyka, Usnea hirta (L.) F.H. Wigg. subsp. laricicola Motyka, Usnea hirta (L.) F.H. Wigg. subsp. villosa (Ach.) Motyka, Usnea plicata (L.) F.H. Wigg. var. foveata (Vain.) Clauzade & Cl. Roux, *Usnea pulvinata* Motyka *ex* Räsänen, *Usnea variolosa* Motyka

L – Subs.: cor, xyl – Alt.: 2–4 – Note: most common in climatically continental, but humid areas, on bark (branches and boles) of isolated trees and on lignum (*e.g.* wooden fences and poles); widespread throughout the Alps. – **Au**: V, T, S, K, St, O, N. **Ge**: OB. **Sw**: BE, GR, LU, SZ, TI, UR, UW, VS. **Fr**: AHP, HAl, AMa, Isè, Sav, HSav, Var, Vau. **It**: Frl, Ven, TAA, Lomb, Piem, VA, Lig. **Sl**: SlA.

***Usnea intermedia* (A. Massal.) Jatta**

Syn.: Usnea barbata (L.) F.H. Wigg. var. intermedia A. Massal., *Usnea carpatica* Motyka, *Usnea faginea* Motyka, Usnea florida (L.) F.H. Wigg. subsp. fistulosa Motyka, Usnea florida (L.) F.H. Wigg. subsp. floridula Motyka, Usnea florida (L.) F.H. Wigg. subsp. rubrireagens Vězda, Usnea florida (L.) F.H. Wigg. var. rigida Ach., *Usnea glauca* Motyka, Usnea glauca Motyka var. pseudoflorida (Motyka) Motyka, *Usnea hapalotera* (Harm.) Motyka, *Usnea harmandii* Motyka, *Usnea leiopoga* Motyka, *Usnea montana* Motyka, *Usnea neglecta* Motyka, *Usnea protea* Motyka, *Usnea rigida* Motyka *nom.illeg. non* Vain., Usnea rigida Motyka var. faginea (Motyka) Clauzade & Cl. Roux, Usnea rigida Motyka var. hapalotera (Harm.) Clauzade & Cl. Roux, Usnea rigida Motyka var. neglecta (Motyka) Keissl., Usnea rigida Motyka var. protea (Motyka) Clauzade & Cl. Roux, *Usnea smaragdina* Motyka

L – Subs.: cor, xyl – Alt.: 2–4 – Note: a polymorphic and not clearly understood taxon, most common on conifers in humid montane forests, which is treated here in a very broad sense; widespread throughout the Alps. – **Au**: V, T, S, K, St, O, N. **Ge**: OB. **Sw**: BE, GL, GR, LU, SZ, TI, UR, UW, VD, VS. **Fr**: AHP, HAl, AMa, Isè, Sav, HSav, Var, Vau. **It**: Frl, Ven, TAA, Lomb, Piem, VA. **Sl**: SlA.

***Usnea longissima* Ach.**

Syn.: *Dolichousnea longissima* (Ach.) Articus

L – Subs.: cor – Alt.: 3–4 – Note: a mainly cool-temperate to boreal-montane species found on branches of old (mostly coniferous) trees in closed, semi-natural forests in areas with high rainfall and frequent fog; most records are historical, presently the species is restricted to a few localities and very much declining. – **Au**: T, S, K, St, O, N. **Ge**: OB. **Sw**: BE, FR, GL, GR, LU, SZ, UR, UW, VD, ?VS. **Fr**: Isè. **It**: Frl, Ven, TAA, Piem. **Sl**: SlA, Tg.

***Usnea perplexans* Stirt.**

Syn.: *Usnea arnoldii* Motyka, Usnea fulvoreagens *auct. non* (Räsänen) Räsänen, *Usnea lapponica* Vain., Usnea laricina *auct. non* Vain. *ex* Räsänen

L – Subs.: cor – Alt.: 2–4 – Note: on branches of conifers in montane, cold-humid forests with frequent fog; frequent throughout the Alps. – **Au**: T, S, K, St, N. **Ge**: Ge. **Sw**: BE, GL, GR, LU, SZ, TI, UW, VD, VS. **Fr**: AHP, HAl, AMa, Isè, HSav, Var, Vau. **It**: Ven, TAA, Piem, VA. **Sl**: SlA.

***Usnea rubicunda* Stirt.**

Syn.: *Usnea protensa* Stirt., Usnea rubiginea *auct. non* (Michx.) A. Massal., *Usnea sublurida* Stirt.

L – Subs.: cor – Alt.: 1–4 – Note: a mild-temperate, chiefly Mediterranean-Atlantic species found on ancient specimens of *Quercus cerris*, *Q. suber*, and other acid-barked broad-leaved trees in open, but semi-natural, warm-humid forests below the montane belt; most records from the Alps are historical and/or dubious. – **Au**: S, St. **Sw**: ?GR. **Fr**: Var. **It**: Ven, TAA, Lig.

***Usnea silesiaca* Motyka**

Syn.: *Usnea madeirensis* Motyka

L – Subs.: cor – Alt.: 3 – Note: a species resembling *U. subfloridana* in the rigid, shrubby to rarely (in the Alps) pendant thallus with black base and many annulations, with more or less even, orbicular soralia and a thin medulla, containing salazinic acid; on acid bark, mostly on branches; widespread in the Holarctic region, in Europe most common in the West; from the Alps there are only some scattered records. – **Au**: T. **Sw**: LU, UR, UW.

***Usnea subfloridana* Stirt.**

Syn.: *Usnea comosa* (L.) Vain. *non* Pers., Usnea comosa (L.) Vain. subsp. similis Motyka, *Usnea similis* (Motyka) Räsänen

L # – Subs.: cor, xyl, sil – Alt.: 2–5 – Note: on branches of trees in relatively closed forests (but then in the upper parts of the crowns), and on isolated trees, one of the few species of *Usnea* which, albeit with stunted specimens, is also found at low altitudes and in relatively disturbed habitats. The results of a recent molecular study show that the separation between this species and *U. florida* cannot be maintained. – **Au**: V, T, S, K, St, O, N, B. **Ge**: OB. **Sw**: BE, FR, GL, GR, LU, SZ, TI, UR, UW, VD, VS. **Fr**: AHP, HAl, AMa, Isè, HSav, Var, Vau. **It**: Frl, Ven, TAA, Lomb, Piem, VA. **Sl**: SlA, Tg.

***Usnea subscabrosa* Nyl. *ex* Motyka**

L – Subs.: cor, sil – Alt.: 2–4 – Note: a well-defined, mainly southwestern species in Europe, found both on basic siliceous rocks and on bark in humid situations, mostly in the upper montane and subalpine belts, also reported from the base of the Western Alps (France). – **Fr**: Isè.

***Usnea substerilis* Motyka**

Syn.: *Usnea sorediifera* *sensu* Motyka *non* (Arnold) Lynge, *Usnea stuppea* (Räsänen) Motyka

L – Subs.: cor, xyl – Alt.: 2–4 – Note: a subcontinental species, often confused in the past with the related *U. perplexans*; probably one of the most frequent *Usnea* of the Alps. – **Au**: V, T, S, K, St, N. **Ge**: OB. **Sw**: BE, GL, GR, LU, SZ, TI, VD, VS. **Fr**: AHP, HAl, AMa, Isè, Var, Vau. **It**: Ven, TAA, Lomb, VA. **Sl**: SlA.

***Usnea wasmuthii* Räsänen**

L – Subs.: cor – Alt.: 1–4 – Note: a species resembling *U. subfloridana* in the shrubby thallus with black base, with large, longitudinally streched soralia, containing salazinic and/or barbatic acid; ecologically similar to *U. florida*, but more frequent at lower altitudes in warm-humid areas. – **Au**: K. **Ge**: Schw. **Sw**: LU, SZ, VS. **Fr**: Vau. **It**: Lig.

***Usnocetraria oakesiana* (Tuck.) M.J. Lai & J.C. Wei**

Syn.: *Allocetraria oakesiana* (Tuck.) Randlane & A. Thell, *Cetraria bavarica* Kremp., *Cetraria oakesiana* Tuck., *Cetraria ochrocarpa* (Eggerth) Lettau, *Platysma oakesianum* (Tuck.) Nyl., *Tuckermannopsis oakesiana* (Tuck.) Hale

L – Subs.: cor, xyl – Alt.: 3–4 – Note: a cool-temperate to boreal-montane, incompletely circumpolar species found on basal parts of conifers in humid-cold montane forests, more rarely on lignum (*e.g.* on stumps); widespread throughout the Alps, but generally not common, and probably declining. – **Au**: V, T, S, K, St, O, N. **Ge**: OB, Schw. **Sw**: BE, GR, LU, SZ, UR, UW. **It**: Frl, Ven, TAA. **Sl**: SlA.

***Vahliella leucophaea* (Vahl) P.M. Jørg.**

Syn.: *Fuscopannaria leucophaea* (Vahl) P.M. Jørg., *Lecidea microphylla* (Lilj.) Ach., *Lichen leucophaeus* Vahl, *Massalongia cheilea* Mudd, *Pannaria austriaca* Zahlbr., *Pannaria cheilea* (Mudd) Leight., *Pannaria leucophaea* (Vahl) P.M. Jørg., *Pannaria microphylla* (Lilj.) Delise *ex* Bory, *Pannularia microphylla* (Lilj.) Stizenb., *Parmeliella microphylla* (Lilj.) Müll. Arg., *Parmeliella pseudocraspedia* (Hazsl.) Gyeln.

L – Subs.: sil, int – Alt.: 2–5 – Note: on basic siliceous rocks in sheltered and humid situations, such as in seepage tracks; widespread throughout the Alps. – **Au**: V, T, S, K, St, O, N, B. **Ge**: Schw. **Sw**: BE, GR, SZ, TI, VS. **Fr**: AHP, HAl, AMa, Isè, HSav, Var. **It**: Ven, TAA, Lomb, Piem, VA, Lig. **Sl**: SlA, Tg.

***Vahliella saubinetii* (Mont.) P.M. Jørg.**

Syn.: *Fuscopannaria saubinetii* (Mont.) P.M. Jørg., *Massalongia rabenhorstiana* Gyeln., *Pannaria saubinetii* (Mont.) Nyl., Parmelia rubiginosa (Ach.) Ach. var. pulveraceogranulosa Grognot, *Parmelia saubinetii* Mont., *Parmeliella saubinetii* (Mont.) Zahlbr., Parmeliella saubinetii (Mont.) Zahlbr. f. grisea Gyeln., *Trachyderma saubinetii* (Mont.) Trevis.

L – Subs.: cor – Alt.: 1–2 – Note: a mild-temperate species found on trunks of mature deciduous trees in rather shaded and humid situations; apparently most frequent in the Western Alps. – **Fr**: AHP, AMa, HSav, Var, Vau. **It**: Ven, Piem.

***Varicellaria hemisphaerica* (Flörke) I. Schmitt & Lumbsch**

Syn.: *Pertusaria hemisphaerica* (Flörke) Erichsen, *Pertusaria speciosa* Høeg, *Variolaria hemisphaerica* Flörke

L – Subs.: cor – Alt.: 1–3 – Note: a mild-temperate species found on old deciduous trees, especially oaks, in open, mostly deciduous forests; widespread throughout the Alps. – **Au**: V, T, S, K, St, O, N. **Ge**: OB. **Sw**: BE, LU, SZ, UR, UW, VD. **Fr**: AHP, Isè, HSav, Var, Vau. **It**: Frl, Ven, TAA, Lomb, Piem. **Sl**: SlA, Tg.

***Varicellaria lactea* (L.) I. Schmitt & Lumbsch**

Syn.: *Lichen lacteus* L., *Ochrolechia lactea* (L.) Matzer & Hafellner, *Pertusaria lactea* (L.) Arnold, *Variolaria lactea* (L.) Pers.; incl. Pertusaria lactea (L.) Arnold f. faginea Erichsen

L – Subs.: sil, int, cor – Alt.: 2–5 – Note: optimum on steeply inclined, lime-free, rather shaded surfaces of siliceous rocks in humid areas, rarely corticolous; widespread throughout the Alps. – **Au**: V, T, S, K, St, N, B. **Ge**: Ge. **Sw**: BE, GR, LU, VD, VS. **Fr**: HAl, AMa, Isè, Sav, HSav, Var, Vau. **It**: Frl, Ven, TAA, Lomb, Piem, VA, Lig. **Sl**: SlA.

***Varicellaria rhodocarpa* (Körb.) Th. Fr.**

Syn.: *Pertusaria rhodocarpa* Körb., *Varicellaria kemensis* Räsänen, *Varicellaria microsticta* Nyl.

L – Subs.: deb, cor, xyl, ter-sil – Alt.: 4–5 – Note: a mainly arctic-alpine species found on acid soil and plant remains, more rarely on lignum or on rocks in tundra-like environments. – **Au**: V, T, S, K, St, O, N. **Ge**: OB. **Sw**: BE, GR, TI, UR, UW, VS. **Fr**: HAl, Sav, HSav. **It**: Frl, Ven, TAA, Lomb. **Sl**: SlA.

***Verrucaria aberrans* Garov.**

L # – Subs.: sil – Alt.: 2–3 – Note: a species with a thin, spreading, dark olive to brown, continuous to rimulose thallus, hemispherically protruding perithecia (to 0.25 mm in diam.), an involucrellum adpressed in the apical region, and oblong to ellipsoid ascospores (to *c.* 30 µm long); on siliceous rocks (porphyr, granite) in the shade of deciduous forests; known from a few localities in the Italian Alps. – **It**: Lomb.

***Verrucaria acrotella* Ach.**

Syn.: Verrucaria aethiobola Wahlenb. var. acrotella (Ach.) H. Olivier

L # – Subs.: sil – Alt.: 2–3 – Note: a species found on siliceous rocks, mostly close to the ground, which needs further study. – **Fr**: AMa, Sav, HSav, Vau. **It**: TAA, Lig.

***Verrucaria adelminienii* Zschacke**

L # – Subs.: cal – Alt.: 2–4 – Note: a calcicolous species resembling *V. muralis*, with a thin, greenish-white thallus, slightly protruding ascomata (to *c.* 0.5 mm in diam.) with a closed, black exciple, and ascospores to *c.* 25 µm long; in the Central European mountains it is most common in the montane belt; there are scattered records from the Alps, but several recent ones are uncertain. – **Au**: S, N. **Sw**: LU, SZ. **Fr**: HAl, AMa, Isè, Var, Vau.

***Verrucaria aethiobola* Wahlenb.**

Syn.: *Lithocia aethiobola* (Wahlenb.) Stein, *Lithocia chlorotica* (Hepp) Stein, *Pyrenula aethiobola* (Wahlenb.) Ach., *Verrucaria aquilella* Nyl., *Verrucaria catalepta* *sensu* Schaer. *non* (Ach.) Spreng., *Verrucaria chlorotica* Hepp *non* Ach., *Verrucaria fuscocinerascens* Nyl., Verrucaria hydrela Ach. var. aethiobola (Wahlenb.) A. Massal., ?*Verrucaria hibernica* Zschacke, *Verrucaria laevata* Ach. *non auct. nec* *sensu* Körb., ?*Verrucaria rimosella* Nyl., *Verrucaria viridicana* Erichsen

L – Subs.: sil-aqu, cal-aqu – Alt.: 2–5 – Note: a widespread species, both in Northern Europe and in the Alps, periodically submerged on hard, mostly siliceous rocks along creeks. See also note to *V. csernaensis*. – **Au**: V, T, S, K, St, N. **Sw**: BE, GR, SZ, UR, VS. **Fr**: AHP, HAl, AMa, Isè, Sav, HSav, Var. **It**: Ven, TAA, Lomb, Piem.

***Verrucaria algovica* Servít**

Syn.: *Amphoridium algovicum* (Servít) Servít

L # – Subs.: cal – Alt.: ?5 – Note: a calcicolous species with a mainly endolithic, whitish thallus which is partly emerging as a thin continuous crust with black prothallus lines, entirely immersed perithecia (to 0.7 mm in diam.) without involucrellum, and ellipsoid ascospores (to 30 µm long); only known from the type locality in the Eastern Alps (Germany). – **Ge**: Schw.

***Verrucaria aljazevi* Servít**

L # – Subs.: cal – Alt.: 3 – Note: a calcicolous species with an endolithic, whitish thallus, black, protruding perithecia (to 0.3 mm in diam.), and narrowly ellipsoid ascospores (to *c.* 30 µm long); only known from the type locality in the montane belt of the Eastern Alps (Slovenia). – **Sl**: SlA.

***Verrucaria alpicola* Zschacke**

L – Subs.: sil-aqu, cal-aqu – Alt.: 3–5 – Note: a species of the *V. elaeomelaena*-group with a thin, continuous, epilithic, dark brown to nearly black thallus which is rimose only around the ascomata, in section view the upper cortex with a dark brown to black pigment and partly with a black basal layer, ascomata protruding from the thallus and covered by thin thalline layer, with a brown exciple and a laterally spreading involucrellum reaching the base of the perithecia, and with narrowly ellipsoid ascospores (to *c.* 35 µm long); a typically sub-aquatic species which often occurs in the splash water zone in streams, but also at temporarily inundated sites in springs, both on calcareous and on siliceous rocks, in sunny to moderately shaded sites, mostly in upland areas; probably more widespread in the Alps. – **Au**: K. **Ge**: Schw. **Sw**: BE, GR. **It**: Ven, TAA, VA.

***Verrucaria alpigena* Breuss**

L – Subs.: cal-aqu – Alt.: 3–5 – Note: on calciferous rocks in upland areas; closely related to *V. muralis*, but with larger spores, it has been reported only from the Eastern Alps (Austria, Italy) and the Carpathian Mts. – **Au**: T, St, O, N. **It**: TAA.

***Verrucaria ampezzana* Servít**

L # – Subs.: cal – Alt.: 4–5 – Note: on inclined surfaces of calciferous schists near or above treeline; a species described from South Tyrol and also reported from the French Alps (Roux et al. 2014). According to Breuss (see Nimis 2016) it is closely related to *Parabagliettoa dufourii*, differing in the larger spores, and should be included in *Parabagliettoa*. – **Fr**: HAl. **It**: Ven.

***Verrucaria anceps* Kremp.**

Syn.: *Polyblastia anceps* (Kremp.) Servít

L – Subs.: cal – Alt.: 2–5 – Note: on limestone and dolomite in humid-shaded situations below treeline; reported from several localities in the mountains of Central Europe and the Alps. – **Au**: V, T, S, O, N. **Ge**: OB. **Sw**: GR, TI. **Fr**: AMa, Vau. **It**: Ven, TAA, Piem, VA.

***Verrucaria andesiatica* Servít**

L – Subs.: sil-aqu, int-aqu – Alt.: 3–4 – Note: a species with a very thin, epilithic, olive-brown thallus, ascomata (to 0.5 mm in diam.) in convex to conical warts, with a superficially spreading, carbonised involucrellum, and oblong to ovoid ascospores (to *c.* 30 µm long); on moist siliceous rocks from the lowlands (type!) to treeline; widespread in Europe but very rarely collected. – **Sw**: SZ. **Fr**: AMa.

***Verrucaria anulata* Zschacke**

L # – Subs.: cal – Alt.: 4 – Note: a species resembling in habitus *V. cincta* Hepp, but ascomata larger and ascospores smaller, with a whitish-grey, thin, continuous, wrinkled thallus, black, sessile ascomata (to 1 mm in diam.) with a depressed ostiolar region, surrounded by a thalline ridge, with a well-developed, slightly convex involucrellum, 8-spored asci, and broadly ellipsoid ascospores (16–19 × 11–13 μm); on a calcareous rock at high elevation; only known from the type locality in the Eastern Alps (Switzerland). – **Sw**: GR.

***Verrucaria anziana* Garov.**

L # – Subs.: sil, sil-aqu – Alt.: 3 – Note: this species was often considered as a synonym of *V. latebrosa*. It usually grows on siliceous rocks by streams, rivers and lakes, and is known from both the Alps (Italy) and Scandinavia. – **It**: Lomb.

***Verrucaria apatela* (A. Massal.) Trevis.**

Syn.: *Lithocia apatela* A. Massal.

L # – Subs.: cal – Alt.: 2–5 – Note: on steeply inclined faces of limestone and dolomite; reported from many sites in Central and Southern Europe, closely related to *V. macrostoma*. – **Sw**: GR, VS. **It**: Ven, TAA, Lomb, Piem.

***Verrucaria apomelaena* (A. Massal.) Hepp**

Syn.: *Lithocia apomelaena* A. Massal.

L # – Subs.: cal – Alt.: 2–3 – Note: a rather poorly known species found both on limestone and on calciferous sandstone. – **Au**: V, S. **Fr**: HSav. **It**: Ven.

***Verrucaria aquatilis* Mudd**

Syn.: *Bachmannia maurula* (Müll. Arg.) Zschacke, *Verrucaria maurula* Müll. Arg., *Verrucaria retecta* Zschacke; incl. *Verrucaria vitricola* Nyl.

L – Subs.: sil-aqu – Alt.: 2–4 – Note: distinguished from other freshwater species by the thin blackish thallus and the very small, broadly ellipsoid ascospores, this lichen grows on siliceous or calcareous rocks submerged in cold creeks; probably more widespread in the Alps, but overlooked, like many amphibious lichens. – **Au**: T, S, K, St, N. **Fr**: AHP, HAl, AMa, HSav. **It**: Ven, TAA.

***Verrucaria areolatodiffracta* Zschacke**

L # – Subs.: sil-aqu – Alt.: 4 – Note: a species probably related to *V. latebrosa*, with a cream-coloured to brownish, continuous to partly rimose thallus which around the ascomata is areolate, the areoles of various shapes and even subsquamulose, in section paraplectenchymatic, ascomata (to 0.35 mm in diam.) immersed in the areoles, with an (almost) entire, well-developed involucrellum, 8-spored asci, and obovoid, simple ascospores (26–31 × 13–15 μm); on submerged siliceous rocks in streams; only known from the type locality in the Eastern Alps (Switzerland). – **Sw**: GR.

***Verrucaria arnoldii* J. Steiner**

Syn.: Verrucaria hiascens *auct. non* (Ach.) Spreng., Verrucaria hochstetteri var. arnoldii (J. Steiner) Clauzade & Cl. Roux

L # – Subs.: cal – Alt.: 2–5 – Note: a species of the *V. hochstetteri*-group with an unresolved nomenclature (superfluous new name for a legitimate species), with a white to greyish thallus, ascomata sunken in the rock with the conical ostiolar region breaking through thalline/substratic warts, reported to be distinguishable by the smaller ascospores (24–35 × 12–18 μm); on limestone and dolomite, widespread in Europe including the Alps, but not always distinguished. – **Au**: V, T, S, K, St, O. **Fr**: AHP, AMa, Drô, Isè. **Sl**: Tg.

***Verrucaria asperula* Servít**

L # – Subs.: cal – Alt.: 3–4 – Note: a species described from Germany and also reported from relatively warm sites in the Austrian, French and Italian Alps. – **Au**: St, O. **Fr**: Sav.

***Verrucaria austriaca* Riedl**

Syn.: *Verrucaria irrigua* Zschacke *non* Taylor

L # – Subs.: int-aqu – Alt.: 3 – Note: a species with a thin, epilithic, grey, somewhat rimose thallus, almost completely immersed perithecia (to *c.* 0.25 mm in diam.), and up to *c.* 20 µm long ascospores; on irrigated outcrops of marl, only known from the type locality in the Eastern Alps (Austria). – **Au**: N.

***Verrucaria banatica* Servít**

L – Subs.: cal – Alt.: 3 – Note: a calcicolous species with a thin, rimose, brownish-grey thallus, hemispherically protruding ascomata (to *c.* 0.4 mm in diam.) with an adpressed involucrellum reaching down half of the the perithecium, and ellipsoid to oblong ascospores (to *c.* 25 µm long); in the study area only known from the Eastern Alps (Austria). – **Au**: V, St, O, N.

***Verrucaria bavarica* Servít**

L # – Subs.: cal – Alt.: ?5 – Note: a species resembling *V. dolomitica*, with a very thin, epilithic, whitish thallus, slightly to hemispherically protruding, black perithecia, a thin involucrellum in the upper half, and ellipsoid to oblong ascospores (to *c.* 32 µm long); only known from the type locality in the Eastern Alps (Germany). – **Ge**: Schw.

***Verrucaria beltraminiana* (A. Massal.) Trevis.**

Syn.: *Lithocia beltraminiana* A. Massal.

L # – Subs.: cal – Alt.: 1–5 – Note: on horizontal to weakly inclined surfaces of calcareous rocks, including walls in small settlements. A critical species, closely related to (but perhaps distinct from) *Verruculopsis lecideoides*, which should probably be included in *Verruculopsis*. – **Au**: ?V, ?T, St. **Fr**: HAl, AMa. **It**: Ven, Piem.

***Verrucaria boblensis* Servít**

L # – Subs.: cal – Alt.: 3 – Note: a calcicolous species recalling (in section) *V. muralis*, but with smaller ascospores, characterised by a thin, rimose, dirty-whitish thallus, conically to hemispherically protruding ascomata (to *c.* 0.25 mm in diam.) with a thick, adpressed involucrellum reaching down about a third of the perithecium, and ellipsoid to oblong ascospores (to *c.* 20 µm long); known from a few scattered records in Europe, including the Eastern Alps (Austria). – **Au**: O.

***Verrucaria bryoctona* (Th. Fr.) Orange**

Syn.: *Thelidium bryoctonum* Th. Fr.

L – Subs.: ter-cal – Alt.: 3–4 – Note: a species with a greyish green, granular thallus consisting of goniocysts (to *c.* 40 µm in diam.), subspherical ascomata without involucrellum, partly immersed in the substrate (to *c.* 0.3 mm in diam.), and narrowly ellipsoid ascospores (to *c.* 30 µm long) often with small terminal gelatinous appendages, becoming 1-septate with age; on basic soil and terricolous moribund bryophytes; widespread in Western Europe, with a few records from the Eastern Alps (Austria). – **Au**: V, K, N.

***Verrucaria caerulea* DC.**

Syn.: *Involucrothele bormiensis* (Servít) Servít, *Involucrothele plumbea* (Ach.) Servít, *Thelidium plumbeum* (Ach.) Servít, Thelidium plumbeum (Ach.) Servít f. orbiculare Kremp. *ex* Servít, *Verrucaria bormiensis* Servít, *Verrucaria glaucina* Ach. *non auct.*, *Verrucaria plumbea* Ach., *Verrucaria truncatula* Nyl.

L – Subs.: cal – Alt.: 2–6 – Note: on steeply inclined surfaces of compact calciferous rocks; widespread throughout the Alps. Probably related to *Staurothele*. – **Au**: V, T, S, K, St, O, N, B. **Ge**: Ge. **Sw**: BE, GR, LU, SZ, VD. **Fr**: AHP, AMa, Drô, Isè, Sav, HSav, Var. **It**: Frl, Ven, TAA, Lomb, Piem, Lig. **Sl**: SlA, Tg.

***Verrucaria caesiopsila* Anzi**

Syn.: *Amphoridium caesiopsilum* (Anzi) Arnold *non* *sensu* Arnold, *Verrucaria integrella* (Hue) Nyl.

L # – Subs.: cal, sil – Alt.: 2–4 – Note: a species of more or less calcareous rocks (limestone and dolomite), known from the Southern Alps only. – **Sw**: GR. **It**: Ven, TAA, Lomb, Piem, VA.

***Verrucaria cambrini* Servít**

L # – Subs.: cal – Alt.: 3 – Note: a species with a thin, epilithic, rimulose to subareolate, olive-greenish thallus, ascomata protruding, but laterally obtected by a thin thalline layer, involucrellum slightly spreading with the lower portion, reaching down about a third of the perithecium, ascospores broadly ellipsoid to subglobose (to 20 µm long); on calcareous rocks (the type is on roof tiles!); known from a few scattered localities in Central Europe, including the Eastern Alps (Austria). – **Au**: V.

***Verrucaria cataleptoides* (Nyl.) Nyl.**

Syn.: *Lithocia cataleptoides* (Nyl.) Arnold, Verrucaria margacea (Wahlenb.) Wahlenb. var. cataleptoides Nyl., Verrucaria cataleptoides (Nyl.) Nyl. f. margolae Servít

L – Subs.: cal-aqu – Alt.: 2–3 – Note: on periodically submerged calcareous rocks (records from other rock types need confirmation). This species, frequently considered as a synonym of *V. aethiobola*, clearly differs both in important morphological characters and in the occurrence on calcareous rocks. – **Au**: T. **Fr**: AHP, AMa, Isè. **It**: TAA.

***Verrucaria cataractophila* Servít**

L # – Subs.: cal, cal-aqu – Alt.: ?3 – Note: a species with a thin, greyish-brown thallus forming patches of *c.* 2 cm in diam., hemispherically protruding, black ascomata (to *c.* 0.2 mm in diam.) with an adpressed involucrellum reaching down to the base of the perithecium, and ellipsoid to oblong ascospores (to *c.* 20 µm long); on dolomite near a waterfall, only known from the type locality in the Eastern Alps (Italy). – **It**: TAA.

***Verrucaria cinereorufa* Schaer.**

L # – Subs.: cal – Alt.: 3–4 – Note: on periodically humid surfaces of calcareous or dolomitic rocks, also reported from the Western Pyrenees and from several sites in Western and Central Europe. The records from France include those of *V. elaeodes* (Hue) Zschacke – **Au**: ?V, St, O, N. **Fr**: AMa, Sav, HSav, Vau. **It**: Frl, Lomb, Piem, Lig. **Sl**: SlA.

***Verrucaria clauzadei* B. de Lesd.**

Syn.: Verrucaria cinereorufa Schaer. var. clauzadei (B. de Lesd.) Clauzade & Cl. Roux

L – Subs.: cal – Alt.: 2 (?5) – Note: a species with a thin, pale violet thallus bordered by black lines, half-immersed perithecia (to 0.6 mm in diam.) with the wall black throughout, and ellipsoid ascospores (to *c.* 33 µm long); on calcareous sandstone and similar rock types at low elevations; known from some scattered records in S Europe; the records from high elevations in Austria are very dubious. – **Au**: ?V, ?T. **Fr**: AMa, Vau. **Sl**: SlA.

***Verrucaria collematodes* Garov.**

L # – Subs.: cal, sil – Alt.: 2–3 – Note: mostly on calciferous or base-rich siliceous substrata, including roofing tiles, walls and mortar; a taxon of the *V. nigrescens*-complex, reported from different countries in Central and Southern Europe; apparently most frequent in the Southern and Western Alps, but perhaps not recognised elsewhere. – **Ge**: Ge. **Fr**: AMa, Sav. **It**: Ven, Lomb, Lig.

***Verrucaria concinnasensu* Schaer. [1836] *non* Borrer [1831**]

L – Subs.: cal – Alt.: 3–5 – Note: a calcicolous species with an epilithic, grey-brown, areolate thallus, the areoles with a dark brown margin and basal layer, broadly conical ascomata (or ostiolar region flattened), a conspicuous, adpressed involucrellum reaching down to the base of the perithecium, and ellipsoid ascospores (to *c.* 22 µm long); widespread in the European mountains, mostly at mid-elevations; from the Alps there are a few scattered records. – **Au**: T, O. **Ge**: Schw. **Sw**: BE. **Fr**: HAl, Sav.

***Verrucaria confluens* A. Massal. *nom.illeg. non* (Weber) F.H. Wigg.**

Syn.: Verrucaria muralis Ach. var. confluens (A. Massal.) Körb.

L # – Subs.: sax – Alt.: 2–3 – Note: this species, which has been often considered to be a synonym of *V. muralis*, differs in the thicker thallus and the crowded perithecia with a thick involucrellum. The species has no valid name. – **Au**: St, O, N. **Fr**: Isè, HSav. **It**: Ven, Lomb.

***Verrucaria consociata* Servìt**

L # – Subs.: sil, sil-aqu – Alt.: 3 – Note: a species with a thin, greenish to olivaceous thallus forming patches to 10 mm in diam., minute (less than 0.2 mm in diam.) hemispherically protruding ascomata partly covered by a thin, granulose thalline layer, an entire, thin involucrellum, and ellipsoid, non-halonate ascospores (16–28 × 8–13 μm); on shaded, temporarily wet siliceous rocks, also reported to form isles on other *Verrucaria* species (type!); widespread in Central Europe, with a single record from the Western Alps (France). – **Fr**: AMa.

***Verrucaria constricta* Zschacke**

L # – Subs.: cal – Alt.: 3 – Note: a species with an epilithic, grey-brown, rimose to areolate thallus, entirely immersed ascomata with a conspicuous adpressed involucrellum reaching down to the base of the perithecium, and ellipsoid ascospores (to *c.* 25 µm long); on more or less calcareous rocks; only known from the type locality in the Western Alps (France). – **Fr**: HSav.

***Verrucaria contardonis* Servít**

L # – Subs.: cal – Alt.: 2 – Note: a calcicolous species with a thin, whitish, spreading, continuous thallus (rimulose around the ascomata), hemispherically protruding ascomata (to 0.2 mm in diam.), an involucrellum adpressed to the perithecial wall and reaching down to the base, and oblong to ellipsoid ascospores (to *c.* 25 µm long); known only from the type locality (Italy). – **It**: Frl.

***Verrucaria corticata* Anzi**

L # – Subs.: cal – Alt.: 2–3 – Note: a species with a thin, yellowish white, marginally sublobate thallus forming patches of 2–5 cm in diam, with a well-developed upper cortex of angular cells, numerous, isolated, semi-immersed, black perithecia, oblong to oblong-clavate, 8-spored asci, and simple, hyaline, oblong ascospores measuring *c.* 8.6 × 6.8 µm, purported (in the description) to differ from *Thrombium epigaeum* in the obviously corticate thallus; only reported from the type locality near Bormio (Premadio), where it was collected on a wall of limestone; the type material is well worthy of further study. – **It**: Lomb.

***Verrucaria cretacea* Zschacke**

L # – Subs.: cal – Alt.: 3 – Note: a species with a chalky white thallus, amphora-shaped (to 0.5 mm wide), entirely immersed ascomata (only the ostiolar region visible), 8-spored asci, and ellipsoid, simple ascospores (*c.* 20 × 8–10 μm); on dolomite, only known with certainty from the type locality in the Eastern Alps (Switzerland). – **Sw**: GR, ?SZ.

***Verrucaria crustificans* (Servít) ined. (provisionally placed here, ICN Art. 36.1b)**

Syn.: *Amphoridium crustificans* Servít

L # – Subs.: sil – Alt.: 2 – Note: a silicicolous species recalling a poorly developed *Placocarpus schaereri*, with a thin, epilithic, whitish-pruinose, rimose to areolate thallus, 1–4 perithecia without involucrellum, immersed in the areolae (to *c.* 0.2 mm in diam.), and oblong to ovoid ascospores (to 20 µm long); only known from the type locality at the base of the Western Alps (Italy). – **It**: Lig.

***Verrucaria cryptica* (Arnold) J. Steiner**

Syn.: *Amphoridium crypticum* Arnold

L # – Subs.: cal – Alt.: 2–6 – Note: on compact calcareous rocks and dolomite near and above treeline; a very critical taxon, related to *V. hochstetteri* *s.lat.*, which needs further study. – **Au**: T. **Fr**: AMa, HSav. **It**: Frl, TAA, Lig.

***Verrucaria csernaensis* Zschacke**

L # – Subs.: sil-aqu, cal-aqu – Alt.: 2–5 – Note: a species described from the Carpathians, often considered as a synonym of *V. aethiobola*. According to Thüs (see Nimis 2016) the epithet *aethiobola* was used for two genetically well-separated taxa: *V. aethiobola* *s.str.* and *V. cernaensis*, and it is likely that at least some of the lowland records from the Alps could refer to the latter species. – **Au**: K.

***Verrucaria dalslandensis* Servít**

L – Subs.: sil – Alt.: 3 – Note: a species resembling *V. muralis*, with a thin, epilithic, uncracked to rimulose, brownish to grey thallus with a reddish tinge, hemispherically protruding ascomata (to 0.25 mm in diam.) with adpressed involucrellum reaching down to the base of the perithecium, and oblong ascospores (to *c.* 20 µm long); on schists and similar acidic substrata; a rarely reported species, but apparently with a wide distribution in Europe, including the Eastern Alps (Austria). – **Au**: T.

***Verrucaria davosensis* Zschacke**

L # – Subs.: sax-aqu – Alt.: ?salp – Note: a species resembling in habitus, and probably related to *V. hydrela*, with a greenish-brown thallus, in section with a black basal layer in fertile areas, ascomata (to 0.4 mm in diam.) covered by a thalline layer, the spherical exciple pigmented only in the ostiolar region, otherwise almost hyaline throughout, 8-spored asci, and ellipsoid, simple ascospores (19–22 × 8–9 μm); on submerged rocks in streams; only known from the type locality in the Eastern Alps (Switzerland). – **Sw**: Sw.

***Verrucaria delitescens* Servít**

Syn.: Amphoridium dolomiticum A. Massal. var. obtectum Arnold, *Amphoridium obtectum* (Arnold) Arnold *non Verrucaria obtecta* Müll. Arg.; incl. Verrucaria delitescens Servít f. mulazensis Servít

L # – Subs.: cal – Alt.: 5 – Note: related to, or perhaps identical with *V. caesiopsila* and/or *V. cryptica*, with an endolithic thallus emerging with dots and brownish patches, immersed perithecia (to *c.* 0.4 mm in diam.) with a minute involucrellum around the ostiolar region, and ellipsoid ascospores (to *c.* 30 µm long); on dolomite and probably other calcareous rocks at high elevations; only known from the type locality in the Eastern Alps (Italy). – **It**: TAA.

***Verrucaria dermatoidea* Servít**

Syn.: Verrucaria veronensis A. Massal. f. dermatoidea A. Massal. *ex* Anzi

L # – Subs.: cal – Alt.: 2 – Note: a calcicolous species with a whitish, rimulose to areolate thallus, hemispherically protruding perithecia (to 0.25 mm in diam.) arising in the centre of the areoles, an involucrellum adpressed to the perithecial wall and reaching down about 1/3 of the perithecium, and ellipsoid ascospores (to *c.* 35 µm long); only known from the type locality in the Eastern Alps (Italy). – **It**: Ven.

***Verrucaria despecta* Servít**

L # – Subs.: cal – Alt.: 2 – Note: a calcicolous species with a whitish, epilithic, rimose to areolate thallus forming patches to 3 cm in diam. (the areoles with a minutely verruculose surface), hemispherically protruding ascomata (to 0.2 mm in diam.) mostly arising inbetween or at the edges of the areoles, an involucrellum adpressed to the perithecial wall and reaching down about half of the perithecia, and oblong to ellipsoid ascospores (to *c.* 15 µm long); only known from the type locality at the base of the Western Alps (Italy). – **It**: Lig.

***Verrucaria diaphragmata* Zschacke**

L # – Subs.: sax – Alt.: 4 – Note: a species with a thin, epilithic, grey, continuous thallus, hemispherically protruding, black, ascomata (*c.* 0.2 mm in diam.) with a rough surface, an involucrellum tightly adpressed to the perithecial wall and reaching down to the base, and ellipsoid, simple ascospores (20–22 × 9–10 μm); on small (calcareous?) stones in the shade of closed subalpine forests; only known from the type locality in the Eastern Alps (Switzerland). – **Sw**: GR.

***Verrucaria dilacerata* Zschacke**

L # – Subs.: cal – Alt.: 4 – Note: a species resembling a poorly developed *V. nigrescens*, with an epilithic, black-brown, rimose to areolate thallus, hemispherically protruding, black ascomata (to *c.* 0.25 mm in diam.), an involucrellum tightly adpressed to the perithecial wall and reaching down to the base, 8-spored asci, and ellipsoid, simple ascospores (11–18 × 5–7 μm); only known from the type locality in the Eastern Alps (Switzerland), on dolomite. – **Sw**: GR.

***Verrucaria dinarica* Zahlbr.**

L – Subs.: cal – Alt.: 3–5 – Note: closely related to *V. caerulea*, but with a mainly endolithic, uncracked, grey thallus, almost entirely immersed perithecia (to *c.* 0.3 mm in diam.) with a wall carbonised throughout and without involucrellum, and oblong to ellipsoid ascospores (to *c.* 15 µm long); on limestone, known from scattered records in Southern Europe, including the Alps, but rare. – **Fr**: HSav. **Sl**: SlA.

***Verrucaria discernenda* Zschacke**

Syn.: *Amphoridium caesiopsilum* *sensu* Arnold *non* (Anzi) Arnold *nec Verrucaria caesiopsila* Anzi

L # – Subs.: cal – Alt.: 3–4 – Note: a species resembling in habitus *V. caesiopsila* *sensu* Arnold, but with larger ascospores, with an endolithic thallus indicated by whitish-grey patches, ascomata (to 0.3 mm in diam.) deeply immersed in the rock with only the hardly protruding black ostioles visible, more or less spherical in section, the wall dark brown throughout, 8-spored asci, and broadly ellipsoid, simple ascospores (22–30 × 14–18 μm); on carbonatic rocks (*e.g.* dolomite), with a few records from Central Europe, in the Alps only known from the type locality (Italy). – **It**: TAA.

***Verrucaria dolomitica* (A. Massal.) Kremp.**

Syn.: *Amphoridium dolomiticum* A. Massal.

L – Subs.: cal – Alt.: 2–5 – Note: on calcareous rocks and pebbles, usually near the ground. A species belonging to the poorly understood complex of *V. hochstetteri*, differing from *V. foveolata* in the small apical involucrellum. – **Au**: V, T, S, St, O, N. **Fr**: Drô, Vau. **It**: Ven, Lomb.

***Verrucaria dolosa* Hepp**

Syn.: ?*Verrucaria krempelhuberi* Lindau, Verrucaria mutabilis *auct. p.p. non* Leight.

L – Subs.: cal, sil, int – Alt.: 2–5 – Note: a probably holarctic early coloniser of small pebbles near the ground, both on calcareous and base-rich siliceous rocks, in sheltered situations such as in open woodlands and in moist habitats by watercourses, *e.g.* in the splash zone. The species is related to *V. hydrophila*; widespread throughout the siliceous Alps. – **Au**: ?V, T, S, K, St, O, N, B. **Ge**: OB. **Sw**: BE, GR, LU, SZ, TI, VS. **Fr**: AHP, AMa, Drô, HSav, Var, Vau. **It**: Frl, Ven, TAA, Lomb, Piem, VA, Lig. **Sl**: SlA.

***Verrucaria elaeina* Borrer**

Syn.: *Thelidium elaeinum* (Borrer) Mudd

L – Subs.: cal, sil – Alt.: 3 – Note: a long-forgotten species that seems to be quite common in the British Isles. It grows on shaded limestone, concrete, siliceous rocks and brick, in woodlands or beneath herbaceous vegetation, in natural habitats or on wasteground, in gardens or on damp walls, being characteristic of weakly calcareous rocks in shade; perhaps more widespread in the Alps. – **Ge**: Schw. **Sw**: SZ. **It**: Lomb.

***Verrucaria elaeomelaena* (A. Massal.) Anzi**

Syn.: *Lithocia elaeomelaena* A. Massal., *Verrucaria degenerascens* Nyl. *ex* A.L. Sm., *Verrucaria jurana* Zschacke

L – Subs.: sil-aqu, cal-aqu – Alt.: 2–5 – Note: a cool-temperate to boreal-montane, perhaps circumpolar species, almost perennially submerged in cold montane to alpine creeks, emerging only in very shaded situations; perhaps more widespread in the Alps. In Northern Europe this name was often used for *V. funckii* (Spreng.) Zahlbr. Based on the currently available data from North of the Alps, *V. elaeomelaena* *s.str.* appears to be restricted to limestone, but it cannot be separated by morphology alone from several other unnamed lineages within the aggregate which grow on calcareous and siliceous substrata alike, especially in deep shade. – **Au**: T, S, K, St, O, N. **Ge**: OB. **Sw**: BE, GR, VD, VS. **Fr**: HAl, AMa, Drô, Var, Vau. **It**: Ven, TAA. **Sl**: SlA, Tg.

***Verrucaria elevata* (Nyl.) Zschacke**

Syn.: Lithocia viridula (Schrad.) A. Massal. var. elevata Nyl.

L # – Subs.: sil – Alt.: 2–3 – Note: most frequent on calciferous schists, superficially resembling *V. macrostoma*; reported from a few localities in Central Europe and the Alps. – **Au**: K, St, N.

***Verrucaria endocarpoides* Servít**

L # – Subs.: sil, cal – Alt.: 2–4 – Note: an apparently widespread taxon belonging to a group of species with a thick, brown, areolate thallus, which still needs revision. It has been reported from Italy, Austria, Slovakia and North America. – **Au**: T, O, N. **It**: Frl.

***Verrucaria endolithea* Zschacke**

L # – Subs.: cal – Alt.: 4–5 – Note: a species with a mainly endolithic to thin, continuous thallus indicated by grey patches, numerous minute ascomata (to 0.25 mm in diam.) forming hemispherically protruding black warts, a tightly adpressed involucrellum, 8-spored asci, and ellipsoid, simple ascospores (11–16 × 7–9 μm); on calcareous rocks in the lower alpine belt, only known from the type locality in the Eastern Alps (Switzerland). – **Sw**: GR.

***Verrucaria epixylon* Zschacke**

L – Subs.: xyl – Alt.: 3 – Note: a species with a verruculose-areolate thallus, hemispherically protruding perithecia (to *c.* 0.3 mm in diam.) with an entire involucrellum, and broadly ellipsoid ascospores (to *c.* 12 µm long); on wooden fences in rural environments, only known from the Eastern Alps (Austria). – **Au**: T.

***Verrucaria erubescens* Zschacke**

L # – Subs.: cal-aqu – Alt.: 4 – Note: a species resembling *V. csernaensis*, with a thin thallus which is grey-green when wet, turning reddish and rimulose when dry, wart-like, protruding perithecia (to *c.* 0.5 mm in diam.) and larger ascospores (to *c.* 30 µm long); on temporarily submerged calcareous rocks in streams, only known with certainty from the type locality in Switzerland. – **Au**: ?V. **Sw**: GR.

***Verrucaria euganea* Trevis.**

Syn.: Verrucaria weddellii *auct. non* Servít

L – Subs.: cal – Alt.: 2–3 – Note: an early coloniser of walls (mortar, brick, cement, limestone) in urban settlements; related to *V. macrostoma*, but differing in several important morphological characters; apparently most frequent in the Southern and Western Alps, but probably more widespread. – **Au**: N. **Fr**: AHP, AMa, Drô, Isè, Var, Vau. **It**: Ven, TAA, Piem, Lig.

***Verrucaria eusebii* Servít**

Syn.: *Verrucaria amylacea* Hepp *nom.illeg. non* Ach.

L – Subs.: sil, cal – Alt.: 1–4 – Note: on limestone and dolomite in sheltered situations protected from rain, *e.g.* with *Caloplaca cirrochroa*; perhaps regionally overlooked, and more widespread in the Alps. – **Au**: ?V, T, K, St, O, N. **Fr**: AMa, Drô, Sav, HSav, Vau. **It**: Ven, Piem.

***Verrucaria ferratensis* Servít**

L # – Subs.: ?sil – Alt.: 2 – Note: a species with a thin, blackish brown, rimose to areolate thallus forming patches of *c.* 1 cm. in diam., surrounded by a subdentritic prothallus and with a carbonised basal layer, ascomata immersed in verrucae (to 0.25 mm in diam.), involucrellum adpressed to the perithecial wall in the upper part, somewhat spreading below and fusing with the basal thalline layer, ascospores oblong to ellipsoid (to 25 µm long); on roof tiles, only known from the type locality at the base of the Western Alps (France). – **Fr**: AMa.

***Verrucaria finitima* Breuss & F. Berger**

L – Subs.: cal – Alt.: 3–5 – Note: a recently-described species resembling *V. poeltii*, found above the montane belts in the Alps on hard, exposed limestone rocks with a long snow cover; perhaps more widespread in the Alps. – **Au**: V, K, O, N. **Sw**: SZ. **Fr**: HSav. **It**: Frl.

***Verrucaria fischeri* Müll. Arg.**

Syn.: *Lithocia tristis* A. Massal., *Verrucaria diffracta* Anzi, *Verrucaria tristis* (A. Massal.) Kremp. *non* Hepp

L – Subs.: cal, int – Alt.: 3–6 – Note: on steeply inclined faces of compact limestone and dolomite in open habitats, mostly above treeline. Most records should be checked against the very similar *V. finitima* and *V. poeltii*. The species does not belong to *Verrucaria* and seems to be related to *Staurothele*. – **Au**: V, T, S, K, St, O, N. **Ge**: Ge. **Sw**: BE, GR, LU, VD, VS. **Fr**: AHP, HAl, AMa, Isè, Sav, HSav. **It**: Frl, Ven, TAA, Lomb, Piem, VA, Lig. **Sl**: SlA.

***Verrucaria floerkeana* Dalla Torre & Sarnth.**

L # – Subs.: sil, cal – Alt.: 2–4 – Note: on more or less calciferous rocks, especially on pebbles and small stones in rather sheltered situations. A rather difficult taxon, very similar to *V. dolosa* and often confused with that species. **Au**: T, S, K, St, O, N, B. **Fr**: Isè. **It**: TAA.

***Verrucaria foveolata* (Flörke) A. Massal.**

Syn.: *Amphoridium foveolatum* (Flörke) A. Massal., Verrucaria schraderi Sommerf. var. foveolata Flörke

L – Subs.: cal – Alt.: 2–5 – Note: an ecologically wide-ranging species of compact limestone and dolomite, found both on the top of large boulders and on small pebbles near the ground. It belongs to the poorly understood complex of *V. hochstetteri*. – **Au**: V, T, K, St, O. **Ge**: OB. **Sw**: BE, GR, TI, UW. **Fr**: AHP, Isè, Sav, Var, Vau. **It**: Frl, Ven, TAA, Lomb, Piem, VA, Lig. **Sl**: SlA, Tg.

***Verrucaria fraudulosa* Nyl.**

Syn.: Verrucaria lecideoides (A. Massal.) Trevis. var. fraudulosa (Nyl.) Clauzade & Cl. Roux

L – Subs.: cal – Alt.: 4–5 – Note: in the Alps on weakly to strongly calcareous rocks from the subalpine to the alpine belt; according to Cl. Roux a taxon of the *Verruculopsis lecideoides*-aggregate. – **Au**: S, O. **Fr**: Isè, Sav.

***Verrucaria funckianasensu* Servít**

Syn.: *Lithoicea funckii* „A. Massal.“ (1853: 143, *nom. nud.*!, 1854: 23) *non Verrucaria funckii* (Spreng.) Zahlbr.

L # – Subs.: cal – Alt.: 2 – Note: a calcicolous species with a thin, brownish – to greenish-black, spreading, rimose to areolate thallus, the basal layer brown-black or lacking, ascomata hemispherically protruding (*c.* 0.1 mm in diam.), involucrellum adpressed to the perithecial wall reaching down to the base and fusing with the basal layer, ascospores oblong to ellipsoid (to *c.* 25 µm long); only known from the Eastern Alps (Italy). – **It**: Ven.

***Verrucaria funckii* (Spreng.) Zahlbr.**

Syn.: *Pyrenula funckii* Spreng., Verrucaria elaeomelaena *auct. non* (A. Massal.) Anzi, *Verrucaria silicea* Servít, *Verrucaria silicicola* (Zschacke) Servít

L – Subs.: sil-aqu, sil – Alt.: 2–5 – Note: among freshwater Verrucariaceae, this is one of the few species which are usually found in permanently submerged conditions, more rarely in the splash zone of water courses or on deeply shaded stream banks, always on siliceous substrata. It is a typical element of springs and clear headwaters, where it can dominate the benthic community; probably much more widespread in the Alps. – **Au**: V, T, S, K, St. **Ge**: OB. **Sw**: GR, UR. **Fr**: HAl, AMa, Sav. **It**: TAA. **Sl**: SlA.

***Verrucaria furfuracea* (B. de Lesd.) Breuss**

Syn.: Verrucaria macrostoma DC. f. furfuracea B. de Lesd., Verrucaria macrostoma DC. var. imbricum Garov., Verrucaria tectorum *auct. p.p*.

L – Substrata: cal – Bioclimatic belt: 1–2 – Note: mainly on man-made substrata, including mortar walls, on steeply inclined faces; frequently confused with *V. tectorum*, which is isidiate and not sorediate, and has a thinner thallus (see Roux et al. 2014); certainly more widespread in the Alps, at low elevations. – **Au**: K, St, O, N. **Fr**: AHP, AMa, Var, Vau. **It**: Frl.

***Verrucaria fusca* Pers.**

L # – Subs.: cal, int – Alt.: 3–4 – Note: an often misunderstood taxon, closely related to or even identical with *V. nigrescens*, with a thin, olive-brown, granulose thallus, and up to *c.* 20 µm long ascospores. – **Au**: O. **Ge**: Ge. **Sw**: SZ.

***Verrucaria fuscoatroides* Servít**

L # – Subs.: cal – Alt.: 3–4 – Note: an apparently rather widespread, but poorly understood species described from Germany and also reported from several localities in the Alps, mainly on calcareous rocks. – **Au**: V, K, O, N. **Sw**: SZ. **Fr**: AHP, AMa.

***Verrucaria fusconigrescens* Nyl.**

Syn.: *Lithocia fusconigrescens* (Nyl.) Flagey

L # – Subs.: sil, int – Alt.: 2 – Note: a species with an epilithic, greyish-brown to brown-black, rimulose to subareolate thallus, slightly to hemispherically protruding ascomata, the wall subhyaline in the lower half, involucrellum reaching down to the base of the perithecium, with a carbonised outer layer and brownish inner layer, and oblong ascospores (to *c.* 25 µm long); on siliceous substrata at low elevations; reported from several localities in SW Europe, including the Western Alps (France). – **Fr**: AMa, Isè, Vau.

***Verrucaria fuscovelutina* Servít**

L # – Subs.: cal – Alt.: 3 – Note: a calcicolous species with a thin, spreading, brown, rimose to subareolate thallus, ascomata (to 0.4 mm in diam.) in conical warts, a carbonised involucrellum tightly adpressed to the exciple in the upper half but indistinct further downwards, and ellipsoid ascospores (to *c.* 25 µm long); reported from several localities in Southern and Central Europe, with a single record from the Eastern Alps (Austria). – **Au**: N.

***Verrucaria galactinella* Servít**

Syn.: *Amphoridium galactinum* A. Massal., *Verrucaria galactina* (A. Massal.) Trevis. *non* Ach.

L # – Subs.: cal – Alt.: 2–3 – Note: a calcicolous species with a mainly endolithic, whitish, subfarinose thallus intersected by dark prothallus lines, immersed ascomata (to *c.* 0.3 mm in diam.), an involucrellum forming a small superficial shield but lacking radial cracks, and ellipsoid ascospores (to *c.* 30 µm long); only known from the base of the SE Alps (Italy). – **It**: Ven.

***Verrucaria geomelaena* Anzi**

L # – Subs.: ter-cal – Alt.: 3–4 – Note: a species with a very thin, spreading, subgelatinous thallus, very small, spherical perithecia immersed only with the base, a non-amyloid hymenium with free paraphyses, 6–8-spored asci, and simple, hyaline, oblong ascospores measuring *c.* 18.9 × 6.8 µm; only known from the type collection, on calciferous soil between 1,820 and 2,100 m. The type material is well worthy of further study. – **It**: Lomb.

***Verrucaria geophila* Zahlbr. *nom.illeg. non* Nyl.**

L – Subs.: ter-sil – Alt.: 2–3 – Note: a rare species of slightly calciferous soil in dry Mediterranean grasslands, including those at the base of the Western Alps. The name is illegitimate and would require conservation. – **Au**: St. **Fr**: AMa.

***Verrucaria glacialis* Hepp *non* (Bagl. & Carestia) Stizenb.**

L # – Subs.: cal – Alt.: 4 – Note: a long-forgotten calcicolous species with broadly ellipsoid ascospores (to 32 µm long), only known from the type locality in the Eastern Alps (Austria). – **Au**: K.

***Verrucaria glarensis* Servít**

L # – Subs.: cal – Alt.: 3 – Note: a calcicolous species resembling in habitus *V. tristis*, but with smaller fruiting bodies, with a spreading, epilithic, rimose to areolate, brownish thallus, black and partly protruding, spherical ascomata (0.25–0.5 mm in diam.), an involucrellum tightly adpressed to the wall and reaching down about two third of the perithecium, the wall only weakly pigmented in the lower half, 8-spored asci, and oblong to ellipsoid, simple ascospores (17–20 × 6–7 μm); only known from the type locality in the Western Alps (Switzerland). – **Sw**: GL.

***Verrucaria glaucodes* Nyl.**

L – Subs.: cal – Alt.: 2 – Note: a calcicolous species resembling *V. pinguicula*, but with a thin, rimose to subareolate, whitish-greenish thallus with a bluish tinge, semi-immersed ascomata (to *c.* 0.2 mm in diam.) with hardly pigmented perithecial wall, an adpressed involucrellum reaching down about half the perithecium, and ellipsoid ascospores (to *c.* 15 µm long); most frequent in the western part of continental Europe, including the Western Alps (France). – **Fr**: AHP, AMa, Isè, Var, Vau.

***Verrucaria glauconephela* Nyl.**

L # – Subs.: cal – Alt.: 2–3 – Note: closely related to and perhaps a synonym of *Parabagliettoa cyanea*, with an endolithic thallus indicated by patches of a whitish-greenish colour with a bluish tinge, semi-immersed ascomata (to *c.* 0.15 mm in diam.), an adpressed involucrellum reaching down about half the perithecium, and ellipsoid ascospores (to *c.* 15 µm long); on calcareous rocks at low elevations in continental Europe (based on a type from Hungary), with a few records from the Western Alps (France). – **Fr**: AMa, Var.

***Verrucaria glaucovirens* Grummann**

Syn.: *Verrucaria virens* Nyl. *non* Wallr.

L # – Subs.: cal – Alt.: 3 – Note: a species resembling *V. obfuscans*, with a greyish to greenish-brown thallus with rough areoles, immersed perithecia without involucrellum, and ellipsoid ascospores (to *c.* 20 µm long); on calcareous rocks and walls at low elevations; widespread throughout Europe, but with a few scattered records from the Alps. – **Au**: O. **Sw**: ?VS.

***Verrucaria globulans* Zahlbr.**

L # – Subs.: cal – Alt.: 4 – Note: a calcicolous species with a thin, brownish-grey thallus forming confluent patches limited by dark prothallus lines, hemispherically protruding, black, glossy ascomata (to *c.* 0.8 mm in diam.) with an adpressed involucrellum reaching down one third of the perithecium, and subglobose ascospores (to *c.* 12 µm long); only known from the type locality in the Eastern Alps (Austria). – **Au**: N.

***Verrucaria glowackii* Servít**

L # – Subs.: sil – Alt.: mon – Note: a silicicolous species resembling in habitus *V. papillosa*, with a spreading, epilithic, thin, verrucose to areolate, yellowish to brownish thallus, the black ascomata (to *c.* 0.3 mm in diam.) somewhat protruding with depressed ostioles, an involucrellum reaching down to the base of the perithecium, and ellipsoid to oblong ascospores (23–26 × 10–14 μm); only known from the type locality in the Eastern Alps (Slovenia). – **Sl**: SlA.

***Verrucaria gorzegnoensis* Servít**

L # – Subs.: cal – Alt.: 2 – Note: a species with a thin, continuous to partly rimulose, whitish thallus forming patches to 3 cm in diam., slightly protruding ascomata (to 0.3 mm in diam.), an adpressed involucrellum reaching down to the base of the perithecia (to partly entire), and ellipsoid ascospores (to *c.* 35 µm long); on calcareous schists, only known from the type locality in the Western Alps (Italy). – **It**: Piem.

***Verrucaria gudbrandsdalensis* Zschacke *ex* H. Magn.**

L – Subs.: sil, int – Alt.: 3 – Note: a species with a mainly continuous, partly thicker and subrimose, whitish grey thallus, ascomata (to 0.3 mm in diam.) covered by a conspicuous involucrellum reaching far down at the flanks of the perithecium, ascospores to *c.* 25 µm long; on siliceous slate and similar calcium-poor substrates; widespread in Europe but rarely reported, with a few records from the Eastern Alps (Austria). – **Au**: O, N.

***Verrucaria gypsophila* Zschacke**

L # – Subs.: cal – Alt.: 4 – Note: a species resembling *V. brachyspora*, with a thin, epilithic, continuous thallus of a greyish-brownish colour with a rose tinge, hemispherically protruding ascomata (to *c.* 0.2 mm in diam.) with involucrellum reaching down about two thirds of the perithecium, and obovoid ascospores (to *c.* 20 µm long); based on a type from Northern Germany, on gypsum; in the study area known from a single locality in the Eastern Alps (Austria), a record which however needs confirmation. – **Au**: ?St.

***Verrucaria hegetschweileri* Körb. *ex* Nyl. (*illeg* .) *non* (Naegeli *ex* Hepp) Garov.**

L # – Subs.: cor, ?xyl – Alt.: 2 – Note: a species with a very thin, grey thallus, ascomata to *c.* 0.2 mm in diam., and ellipsoid ascospores (to *c.* 15 µm long); on bark (and wood?) at the base of trunks of broad-leaved trees; so far recorded from a few localities in the Alps. – **Fr**: AMa.

***Verrucaria hemisphaerica* Servít**

L # – Subs.: sil – Alt.: mon – Note: a species resembling in habitus *V. nigresccens*, with a spreading, epilithic, rimose to areolate, brown-black thallus, the black ascomata (to *c.* 0.3 mm in diam.) hemispherically protruding, with an adpressed involucrellum reaching down to the base of the perithecia, and oblong to ellipsoid ascospores (20–24 × 7–10 μm); on porphyric rocks, only known from the type locality in the Eastern Alps (Slovenia). – **Sl**: SlA.

***Verrucaria hilitzeriana* Servít**

L # – Subs.: cal – Alt.: 2 – Note: a calcicolous species with a thin, spreading, brown, rimose to areolate thallus (areoles often with a black rim), black, naked, hemispherically protruding ascomata inbetween the areoles (to 0.2 mm in diam.), an involucrellum adpressed to the perithecial wall reaching down to the base, and oblong to ellipsoid ascospores (to 20 µm long); known from the type locality at the base of the Western Alps (France), and from Eastern Liguria (outside the Alps). – **Fr**: AMa.

**Verrucaria hochstetteri Fr. subsp. hochstetteri var. hochstetteri**

Syn.: Amphoridium hiascens *auct. non* (Ach.) A. Massal., *Amphoridium hochstetteri* (Fr.) A. Massal., Amphoridium hochstetteri (Fr.) A. Massal. f. obtecta Arnold [non *Verrucaria obtecta* Müll. Arg.], Verrucaria hiascens *auct. non* (Ach.) Hepp, Verrucaria hochstetteri Fr. f. papularis Rehm *ex* Servít; incl. Verrucaria hochstetteri Fr. var. crustosa (Arnold) Zahlbr.

L – Subs.: cal, cal-aqu – Alt.: 2–5 – Note: a variable species found on steeply inclined surfaces of compact limestone and dolomite in sheltered situations; widespread throughout the Alps. – **Au**: V, T, S, K, St, O, N. **Ge**: OB. **Sw**: BE, GR, LU, SZ, TI, UR, UW, VD, VS. **Fr**: AHP, HAl, AMa, Drô, Isè, Sav, HSav, Var, Vau. **It**: Frl, Ven, TAA, Lomb, Piem, VA, Lig. **Sl**: SlA.

**Verrucaria hochstetteri Fr. subsp. hochstetteri var. obtecta (Müll. Arg.) Clauzade & Cl. Roux**

Syn.: Verrucaria hochstetteri Fr. var. obtecta (Müll. Arg.) Clauzade & Cl. Roux, *Verrucaria obtecta* Müll. Arg.

L # – Subs.: cal – Alt.: 2–5 – Note: a variety with an endolithic thallus, entirely immersed ascomata detectable only by the dots of the ostioles, lacking both an involucrellum and a protruding rim, and ovoid ascospores (to *c.* 30 µm long); based on type from Egypt, the conspecificity of Central European specimens needs confirmation; the distribution in the Alps is difficult to interpret, since the variety was not always distinguished. – **Au**: ?V, ?T, St. **Fr**: AHP, AMa, Drô, Isè, Sav, HSav, Var, Vau.

**Verrucaria hochstetteri Fr. subsp. rosaeformis Cl. Roux**

Syn.: Verrucaria hochstetteri Fr. var. rosaeformis (Asta, Clauzade & Cl. Roux) Clauzade & Cl. Roux *comb. inval.*, Verrucaria integra (Nyl.) Nyl. var. rosaeformis Asta, Clauzade & Cl. Roux [invalidly published, ICN Art. 40.1. + 8]

L – Subs.: cal – Alt.: 4–5 – Note: a calcicolous taxon peculiar in having a circum-ostiolar involucrellum shaped as a 4-lobed rosette; so far only known with certainty from the Western Alps (France). – **Au**: ?Au. **Fr**: AHP, AMa, Sav, HSav.

***Verrucaria hydrela* Ach.**

Syn.: *Verrucaria denudata* Zschacke, *Verrucaria hydrophila* Orange

L – Subs.: sil-aqu, sil – Alt.: 2–5 – Note: on siliceous pebbles in humid-shaded situations (*e.g.* in open woodlands), sometimes on boulders in creeks, but never submerged for long periods, usually in upland areas but rarely reaching above treeline. Several records need confirmation. For nomenclatural matters, we partly follow Roux et al. (2014: 1246), and partly the suggestion by Thüs (see Nimis 2016) to use the name *V. hydrophila* Orange only for sequenced material with an ITS sequence that fits the one published for the type, which also has a subgelatinous thallus and a widely spreading involucrellum. – **Au**: ?V, T, S, K, St, N. **Sw**: BE, GR, UR, VS. **Fr**: AHP, HAl, AMa, Sav, Var. **It**: Ven, TAA, Lomb, Piem. **Sl**: SlA.

***Verrucaria illinoisensis* Servít**

L – Subs.: cal – Alt.: 3 – Note: a calcicolous species with an epilithic, greyish-white, rimose to areolate thallus, hemispherically protruding, immersed ascomata (*c.* 0.2 mm in diam.), involucrellum spreading and reaching down about half the perithecium, and oblong to ellipsoid ascospores (to *c.* 20 µm long; specimens from NE Europe reported to *c.* 25 µm long); based on a type from North America, and also reported for NE Europe, with a single reord from the Eastern Alps (Austria). – **Au**: O.

***Verrucaria imitatoria* Servít**

L # – Subs.: ?sil – Alt.: 2 – Note: a species resembling *V. rupestris*, with a thin, epilithic, spreading, greyish, rimose to areolate thallus, hemispherically protruding ascomata (to 0.25 mm in diam.), an adpressed involucrellum reaching down almost to the base of the perithecium, and ellipsoid ascospores (to 35 µm long); only known from the type locality at the base of the Western Alps (Italy), on sandstone. – **It**: Lig.

***Verrucaria incertula* (Arnold) Zahlbr.**

Syn.: *Amphoridium incertulum* Arnold

L – Subs.: cal – Alt.: 3 – Note: on very compact calcareous rocks subject to periodical water seepage; related to *V. saprophila*, differing in the smaller perithecia and spores (see Roux et al. 2014: 1247). – **Fr**: AMa. **It**: TAA.

***Verrucaria incompta* Servít**

L # – Subs.: cal – Alt.: 2 – Note: a calcicolous species with a whitish-grey, spreading, partly endolithic thallus densely covered by minute granules (to 20 µm in diam.), semi-immersed ascomata (to 0.2 mm in diam.) laterally covered with a thin thalline layer, an involucrellum adpressed to the perithecial wall and reaching down less than half the perithecia, and oblong ascospores (less than 20 µm long); only known from the type locality at the base of the Western Alps (Italy). – **It**: Lig.

***Verrucaria inordinata* (Servít) ined. (provisionally placed here, ICN Art. 36.1b)**

Syn.: *Involucrothele inordinata* Servít

L # – Subs.: sil – Alt.: 2 – Note: a silicicolous species with a grey, epilithic, rimose to areolate thallus either spreading or forming patches, the areoles with an uneven surface or minutely verrucose, with black granules (to 100 µm in diam.), hemispherically protruding ascomata (to 0.3 mm in diam.), an involucrellum attached to the perithecial wall and reaching down about half of the perithecium, and oblong to ellipsoid, simple ascospores with *c.* 10% one-septate intermixed (to *c.* 20 µm long); only known from the type locality at the base of the Western Alps (Italy). – **It**: Lig.

***Verrucaria inornata* Servít**

L # – Subs.: cal – Alt.: 3 – Note: on rather shaded and moist surfaces of calciferous rocks, this species is similar to *V. memnonia*, differing in the larger spores and the pale excipulum. – **Au**: O. **It**: Lig.

***Verrucaria italica* (B. de Lesd.) Servít**

Syn. *Dermatocarpon italicum* (B. de Lesd.) Zahlbr., *Endopyrenium italicum* B. de Lesd.

L # – Subs.: cal – Alt.: 1 – Note: a calcicolous species characterised by a greenish grey, areolate thallus, perithecia with a dimidiate involucrellum, and ellipsoid to oblong-ellipsoid ascospores measuring *c.* 6 × 15–17 µm; only known from the type collection near Spotorno (Italy). – **It**: Lig.

***Verrucaria jodophila* Servít**

L # – Subs.: cal – Alt.: 4 – Note: a species related to *V. caerulea*, with an epilithic, dark lead grey, rimose to areolate thallus forming patches to 1 cm (!) delimited by black prothallus lines, immersed and hardly protruding ascomata (to 0.15 mm in diam.), an adpressed involucrellum reaching down to the base of the perithecium, and oblong to narrowly ellipsoid ascospores (to 20 µm long); only known from the type locality in the Eastern Alps (Italy), on dolomite. – **It**: TAA.

***Verrucaria lacerata* Servít**

L – Subs.: cal – Alt.: 2–3 – Note: a calcicolouos species with a mainly endolithic, brownish grey thallus with small black patches and black prothallus lines, semi-immersed ascomata (to *c.* 0.4 mm in diam.), a slightly spreading involucrellum reaching down about half of the perithecium, and ellipsoid to oblong ascospores (to *c.* 35 µm long); reported from scattered localities in Central Europe, including the Eastern Alps (Austria). – **Au**: K, O, N. **Sl**: SlA.

***Verrucaria langhensis* Servít**

L # – Subs.: cal, int – Alt.: 2 – Note: a species with a thin, brown, spreading, rimose to areolate thallus (fruiting areolae convex, to 0.8 mm in diam.) with a thick brown-black basal layer, hemispherically protruding ascomata (to 0.3 mm in diam.) covered by a thin thalline layer, involucrellum adpressed to the perithecial wall reaching down to the base and fusing with the basal layer, and oblong to ellipsoid ascospores (exceeding 30 µm in length); on calcareous schists, only known from the type locality in the Western Alps (Italy). – **It**: Piem.

***Verrucaria latebrosa* Körb.**

L # – Subs.: sil-aqu, cal-aqu, xyl – Alt.: 3–5 – Note: a freshwater species periodically submerged on hard siliceous rocks, occasionally also on calcareous substrata. This species was included in *V. aethiobola* by Orange as a member of a “collective species”, and its relation to *V. anziana* remains to be clarified. No material from the type locality has ever been sequenced, which may be necessary to select a sequenced epiptype in order to fix the ambiguity in the use of this name. See also note on *V. anziana*. – **Au**: T, S, K, St. **Sw**: BE, GR, VS. **Fr**: HAl, AMa, Sav, HSav. **It**: Ven, TAA, Lomb, Piem.

***Verrucaria latebrosoides* Servít**

L # – Subs.: sil – Alt.: ?3 – Note: a species with a spreading, olive-brown to blackish-brown, rimose to areolate thallus with a carbonised basal layer, hemispherically protruding ascomata (to *c.* 0.2 mm in diam.), an involucrellum adpressed to the perithecial wall and reaching down to the base, and ellipsoid ascospores (to 25 µm long); on porphyric rocks, only known from the type locality in the Eastern Alps (Italy). – **It**: TAA.

***Verrucaria licentiosa* (Servít) ined. (provisionally placed here, ICN Art. 36.1b)**

Syn.: *Involucrocarpon licentiosum* Servít

L # – Subs.: cal – Alt.: 2–3 – Note: a calcicolous species of unclear relationship, with a bluish grey, epilithic, areolate to verrucose thallus forming patches to *c.* 2 cm in diam., protruding ascomata (to 0.3 mm in diam.) not rarely arising inbetween areoles, an entire involucrellum, and oblong ascospores (to *c.* 20 µm long); only known from the type locality in the Western Alps (Italy). – **It**: Piem.

***Verrucaria lignorum* Servít**

L # – Subs.: xyl, cor – Alt.: 3 – Note: a species with a blackish-brown, rimose to verruculose-areolate thallus forming patches up to 5 cm in diam., a basal layer with dark maculae, immersed ascomata (to *c.* 0.2 mm in diam.), from above hardly discernable from thalline warts, an involucrellum adpressed to the perithecial wall reaching down to the base and fusing with the basal layer, and ellipsoid ascospores (to 25 µm long); on wood, rarely bark; rare throughout Central Europe, including the Eastern Alps (Austria). – **Au**: K.

***Verrucaria lignyodes* Harm. *ex* Crozals**

L # – Subs.: ?sil-aqu – Alt.: 3 – Note: a species of unclear relationship, with an indistinct thallus, ascomata immersed in the rock (to *c.* 0.3 mm in diam.), interascal filaments apparently persistent and with some ramifications, and broadly ovoid ascospores (to 27 µm long); on pebbles in a creek, only known from the type locality in the Western Alps (France). – **Fr**: HSav.

***Verrucaria ligurica* (Servít) ined. (provisionally placed here, ICN Art. 36.1b)**

Syn.: *Involucrothele ligurica* Servít non *Verrucaria ligurica* Zschacke quid est *Hydropunctaria ligurica* (Zschacke) Cl. Roux

L # – Subs.: ?cal – Alt.: 2 – Note: a species with an olive-coloured, epilithic, rimose to areolate thallus forming patches to 3 cm in diam., the areoles with a verruculose surface, protruding ascomata (to 0,4 mm in diam.), an involucrellum adpressed to the perithecial wall and reaching down to the base of the perithecium, and oblong to ellipsoid, simple ascospores intermixed with *c.* 10–20% 1-septate ones (to *c.* 15 µm long); only known from the type locality at the base of the Western Alps (Italy), on a wall. – **It**: Lig.

***Verrucaria limitatoides* Servít**

L # – Subs.: cal – Alt.: 5 – Note: a calcicolous species with a thin, whitish-grey, spreading thallus, hemispherically protruding ascomata (*c.* 0.2 mm in diam.), a loosely attached involucrellum reaching down about half of the perithecium, and oblong to ellipsoid ascospores (to *c.* 25 µm long); only known from the type locality in the Eastern Alps (Austria). – **Au**: T.

***Verrucaria maas-geesterani* Servít**

L # – Subs.: sil-aqu – Alt.: 3 – Note: a species which is similar, and probably closely related to *V. margacea*, with an epilithic, partly rimose, blackish-brown (black in the wet state) thallus, superficially with small black warts, in section with a carbonised basal layer, ascomata (to 0.3 mm in diam.) black and hemispherically protruding, but with a thin thalline layer in the lower part, involucrellum tightly adpressed to the perithecial wall and reaching down to the base, where it fuses with the basal layer, 8-spored asci, and broadly ellipsoid, simple ascospores (20–28 × 13–16 μm); on submerged siliceous rocks in streams, only known from the type locality in the Western Alps (Switzerland). – **Sw**: TI.

***Verrucaria macrostoma* DC.**

Syn.: *Lithocia macrostoma* (DC.) A. Massal., *Verrucaria thrombioides* A. Massal., Verrucaria viridula *auct. non* (Schrad.) Ach.

L – Subs.: cal – Alt.: 1–5 – Note: an early coloniser of walls (mortar, brick, cement, limestone) in urban areas, more rarely found on calcareous rocks in natural environments, with a wide ecological amplitude, from horizontal to steeply inclined faces visited by birds; widespread throughout the Alps. – **Au**: V, ?T, S, St, O, N. **Sw**: BE, LU, SZ, UW, VD, VS. **Fr**: AHP, HAl, AMa, Drô, Sav, Var, Vau. **It**: Frl, Ven, TAA, Lomb, Piem, VA, Lig.

***Verrucaria maculiformis* Kremp. *nom.illeg. non* Hoffm.**

L – Subs.: cal – Alt.: 2–3 – Note: this species seems to be most frequent in Western and Central Europe, on more or less calcareous pebbles or on bricks, especially in rather shaded situations. Most records require confirmation. The name is not legitimate, being a later homonym of *V. maculiformis* Hoffm. (1796). – **Au**: ?V, ?T, O. **Ge**: OB. **Fr**: AMa, HSav. **It**: Ven, Lomb.

***Verrucaria margacea* (Wahlenb.) Wahlenb.**

Syn.: *Lithocia margacea* (Wahlenb.) A. Massal., *Thelotrema margaceum* Wahlenb., *Verrucaria applanata* Hepp *ex* Zwackh, ?*Verrucaria divergens* Nyl., *Verrucaria filarszkyana* Szatala, *Verrucaria leightonii* Hepp *non* A. Massal., *Verrucaria tiroliensis* Zschacke, ?*Verrucaria vallis-fluelae* Zschacke, *Verrucaria zegonensis* Zschacke

L – Subs.: sil-aqu, cal-aqu – Alt.: 2–5 – Note: an amphibious freshwater lichen of siliceous rocks beside streams and lakes; it prefers constantly inundated and even permanently submerged rocks to those merely in the spray zone. The species, widespread in Scandinavia and also known from the Southern Hemisphere, is widespread throughout the Alps. – **Au**: V, T, S, K, St, O, N. **Sw**: BE, GR, TI, UR, VS. **Fr**: AHP, HAl, AMa, Sav, HSav, Var. **It**: Ven, TAA, Lomb, Piem. **Sl**: SlA.

***Verrucaria mastoidea* (A. Massal.) Trevis.**

Syn.: *Amphoridium mastoideum* A. Massal., Verrucaria hochstetteri Fr. var. mastoidea (A. Massal.) Clauzade & Cl. Roux

L # – Subs.: cal – Alt.: 3–4 – Note: on calciferous rocks; this rather poorly understood species differs from *V. hochstetteri* in the presence of a small involucrellum. – **Au**: V, T, K, St, O, N. **It**: Ven, TAA, Piem.

***Verrucaria mauroides* Schaer.**

Syn.: *Lithocia mauroides* (Schaer.) A. Massal., *Thrombium mauroides* (Schaer.) Zschacke

L # – Subs.: sil, ?cal – Alt.: 3–5 – Note: a species of unclear relationship with a thin, blackish thallus, hemispherically protruding ascomata (to *c.* 0.2 mm in diam.), and an involucrellum reaching down to the base of the perithecium (other microscopical characters not documented); so far only recorded from a few localities in the Alps. – **Au**: ?V. **Sw**: BE, VS. **Fr**: HSav.

***Verrucaria memnonia* (Flot. *ex* Körb.) Arnold**

Syn.: Verrucaria maura Wahlenb. var. memnonia Flot. *ex* Körb.

L – Subs.: sil – Alt.: 3–4 – Note: a species with a thin, epilithic, spreading thallus of a black colour with a bluish to greenish-blue tinge, turning gelatinous when wet, hemispherically protruding ascomata (to 0.3 mm in diam.) with involucrellum reaching down to the base of the perithecium, and obovoid ascospores (to *c.* 15 µm long); mostly on hard siliceous rocks in the shade of montane coniferous forests; widespread in the European mountains, with a few scattered records from the Alps. – **Au**: K, O, N. **Sw**: SZ.

**Verrucaria metzleri Servít var. metzleri**

***L*** # – Subs.: cal – Alt.: 2–3 – Note: a calcicolous species with a spreading, olive-brown, subrimose thallus, semi-immersed ascomata (to *c.* 0.4 mm in diam.) covered by a thin thalline layer, an adpressed involucrellum reaching down about half of the perithecium, and ellipsoid ascospores (to 30 µm long); only known from the type locality in the Western Alps (Switzerland), and from Liguria (outside the Alps). – **Sw**: BE.

**Verrucaria metzleri Servít var. carniolica Servít**

L # – Subs.: cal – Alt.: 3 – Note: a calcicolous variety with an adpressed involucrellum and broadly ellipsoid to subglobose ascospores (to *c.* 25 µm long); only known from the type locality in the Eastern Alps (Slovenia). – **Sl**: SlA.

***Verrucaria mimicrans* Servít**

L # – Subs.: cal – Alt.: 2–3 – Note: described from former Yugoslavia, differing from *V. muralis* in the larger spores, the longer periphyses, and the form of the involucrellum; the total distribution covers wide parts of Europe and the species is also known from North America. It is a pioneer species on more or less calcareous substrata, especially on pebbles and on recently exposed rock surfaces; from the Alps there are a few scattered records only. – **Au**: K, O, N. **Fr**: AMa. **It**: Lig. **Sl**: SlA.

***Verrucaria monacensis* Servít**

L # – Subs.: cal – Alt.: 2–3 – Note: according to Breuss (see Nimis 2016), this species was described on the basis of a sample collected on calcareous pebbles in a scree slope near München, and identified by F. Arnold as *Amphoridium dolomiticum* (=*Verrucaria dolomitica*), from which it differs in several important characters; the species also resembles *Verrucaria muralis*, differing in the rimose thallus. Beside the type collection (the original station is probably lost) the species was reported by Sbarbaro (see Nimis 1993: 754) from Piedmont; since Sbarbaro was in close scientific contact with Servít, it is probable that the latter had identified the Italian samples, which constitute the only record from the Alps. – **It**: Piem.

***Verrucaria mortarii* (Arnold) Arnold *ex* Lamy *nom.illeg. non* Leight.**

Syn.: Amphoridium leightonii Arnold f. mortarii Arnold, *Amphoridium mortarii* (Arnold) Flagey

L # – Subs.: cal – Alt.: 2–5 – Note: a species growing on man-made calciferous substrata, including mortar, especially on walls below the montane belt, closely related to *V. foveolata*; apparently most frequent in the Southern and Western Alps. The name is illegitimate. – **Fr**: HAl, AMa, Isè, Sav, Var, Vau. **It**: Piem.

***Verrucaria muelleriana* Servít**

L # – Subs.: cal – Alt.: 3 – Note: a calcicolous species resembling *V. cinereorufa*, with an endolithic to thin-epilithic, white thallus with a reddish tinge, immersed or only slightly protruding ascomata (to 0.5 mm in diam.), an involucrellum spreading around the ostiole, and ascospores to *c.* 40 µm long; only known from the type locality in the Western Alps (France). – **Fr**: HSav.

***Verrucaria muralis* Ach.**

Syn.: ?*Verrucaria argillacea* Fr., *Verrucaria subdendritica* Servít, *Verrucaria submuralis* Nyl.; incl. *Verrucaria rupestris* Schrad. *non* (Scop.) F.H. Wigg.

**L** – Subs.: cal, sil – Alt.: 1–5 – Note: a widespread early coloniser of pebbles, mortar walls, brick and roofing tiles, with optimum in the submediterranean belt. Some records could refer to *V. rupestris*, which until recently was confused with this species, from which it differs in the endolithic thallus and the immersed perithecia; widespread throughout the Alps. – **Au**: V, T, S, K, St, O, N, B. **Ge**: OB, Schw. **Sw**: BE, GR, LU, SZ, VD. **Fr**: AHP, HAl, AMa, Drô, Isè, Sav, HSav, Var, Vau. **It**: Frl, Ven, TAA, Lomb, Piem, VA. **Sl**: SlA, Tg.

***Verrucaria murina* Leight. *non* (Ach.) Arnold**

Syn.: *Amphoridium myriocarpum* (Hepp *ex* Lönnr.) Servít, Verrucaria murina Leight. f. obscurata Servít, Verrucaria murina Leight. var. pusilla Arnold, *Verrucaria myriocarpa* Hepp *ex* Lönnr., *Verrucaria myriocarpa* Hepp *ex* Lönnr. f. geographica Arnold; incl. *Verrucaria brachyspora* Arnold, *Verrucaria pazientii* A. Massal.

L # – Subs.: cal, int – Alt.: 2–5 – Note: on limestone and dolomite in upland areas. The epithet *murina* has been used for widely different species, and the entire complex is presently under revision. – **Au**: V, T, K, St, O, N. **Ge**: OB. **Sw**: BE, LU, UW, VD. **Fr**: HAl, AMa, Drô, Isè, Sav, HSav, Var, Vau. **It**: Ven, TAA, Lomb.

***Verrucaria murorum* (A. Massal.) Lindau**

Syn.: *Lithocia murorum* (A. Massal.) Arnold, *Thrombium murorum* A. Massal.

L # – Subs.: cal – Alt.: 2–4 – Note: a calcicolous species belonging to the *V. macrostoma*-complex, with scattered records from the Alps. – **Au**: V. **Ge**: Ge. **Sw**: SZ, VS. **Fr**: Drô. **It**: Ven, TAA, Lomb.

***Verrucaria nidulifera* Servít**

L # – Subs.: cal – Alt.: 2–3 – Note: according to Breuss (see Nimis 2016) this species resembles *Parabagliattoa dufourii* (which was growing together with the type material), differing in the less developed involucrellum, the more immersed perithecia and the presence of oil hyphae. It was described on the basis of a sample collected by F. Arnold on dolomite (see Nimis 1993: 755), the ecology being similar to that of *Parabagliettoa dufourii.* – **It**: TAA.

***Verrucaria nigrescens* Pers.**

Syn.: *Lithocia controversa* (A. Massal.) A. Massal., ?*Verrucaria acrotelloides* A. Massal. (*fide* Nimis), *Verrucaria controversa* A. Massal., Verrucaria fusca *auct. non* Pers., *Verrucaria fuscoatra* Pers., Verrucaria nigrescens Pers. var. funckii (A. Massal.) Zwackh *non Verrucaria funckii* (Spreng.) Zahlbr., *Verrucaria protothallina* A. Massal., *Verrucaria umbrina* (Ach.) Ach., *Verrucaria velana* (A. Massal.) Zahlbr.; incl. *Verrucaria confusa* Zschacke, *Verrucaria confusionis* Grummann, Verrucaria nigrescens Pers. var. laeviuscula Nyl.

L – Subs.: cal, int – Alt.: 1–6 – Note: a subcosmopolitan species, one of the most common saxicolous lichens throughout the Alps, found both in urban and natural habitats, with a very wide ecological tolerance; several morphs from natural habitats, however, deserve further study. – **Au**: V, T, S, K, St, O, N, B. **Ge**: OB, Schw. **Sw**: BE, GR, LU, SZ, TI, UR, VD, VS. **Fr**: AHP, HAl, AMa, Drô, Isè, Sav, HSav, Var, Vau. **It**: Frl, Ven, TAA, Lomb, Piem, VA, Lig. **Sl**: SlA, Tg.

***Verrucaria nigrofusca* Servít**

L # – Subs.: cal, sil – Alt.: 3–4 – Note: a species described from the Czech Republic and also reported from France (Maritime Alps), and Liguria (outside the Alps) on both calcareous and basic siliceous rocks. It differs from *V. fuscoatroides* in the smaller perithecia and spores. – **Fr**: AMa.

***Verrucaria nigroumbrina* Servít**

Syn.: Lithoicea nigrescens (Pers.) A. Massal. var. umbrina A. Massal.; incl. Verrucaria nigroumbrina Servít f. acrotella (A. Massal.) Servít

L # – Subs.: cal – Alt.: 2 – Note: a calcicolous species with a thin, greyish-brown to brown, spreading, rimose to areolate thallus, the basal layer brown-black or lacking in young areoles, only slightly protruding ascomata covered by a thin thalline layer, an involucrellum adpressed to the perithecial wall, reaching down to the base and fusing with the basal layer, and oblong to ellipsoid ascospores (to *c.* 25 µm long); only known from the Eastern Alps (Italy), and from Liguria (outside the Alps). – **It**: Ven.

***Verrucaria nivalis* Hepp**

L # – Subs.: cal – Alt.: 3 – Note: a calcicolous species with ovoid ascospores (to 36 µm long), only known from the type locality in the Eastern Alps (Austria). – **Au**: K.

***Verrucaria obfuscans* Nyl.**

L # – Subs.: cal – Alt.: 3 – Note: a species apparently belonging to the *V. nigrescens*-group, with a relatively thick, brown, areolate thallus, immersed ascomata (to *c.* 0.3 mm in diam.) with a hardly pigmented perithecial wall, and ellipsoid to oblong ascospores (to *c.* 20 µm long), based on a type from Paris in France; on walls and other anthropogenic substrates at low elevations; rarely recorded because not always distinguished, known from a few localities in the Alps. – **Au**: ?V. **Fr**: Sav.

***Verrucaria ochrostoma* (Borrer *ex* Leight.) Trevis.**

Syn.: *Sagedia ochrostoma* Borrer *ex* Leight.

L # – Subs.: cal – Alt.: 2–3 – Note: closely related to *V. murorum*, this species, characterised by the superficial thallus and immersed perithecia without an involucrellum, seems to prefer concrete walls and nutrient-enriched, dusty surfaces at relatively low elevations; apparently most frequent in the Southern and Western Alps, but perhaps not distinguished elsewhere. – **Au**: S. **Fr**: AHP, AMa, Drô, Var, Vau. **It**: Ven.

***Verrucaria olivacella* Servít**

L # – Subs.: sil – Alt.: 2 – Note: a species related to or even identical with *V. inaspecta*, with a thin, epilithic, continuous to rimose-areolate, dark olivaceus thallus, hemispherically protruding ascomata (to 0.3 mm in diam.), an involucrellum adpressed to the perithecial wall and reaching the base of the perithecia, and ellipsoid ascospores (to *c.* 25 µm long); on schist, only known from the type locality at the base of the Western Alps (Italy). – **It**: Lig.

***Verrucaria olivascens* Servít**

L # – Subs.: cal – Alt.: 3 – Note: a calcicolous species with a thin, spreading, whitish to greyish, minutely granulose to warty-farinose thallus, hemispherically protruding ascomata (to 0.5 mm in diam.) which are externally covered by a very thin, farinose thalline layer, an adpressed involucrellum reaching down to the base of the perithecium, and oblong to obovoid ascospores (to 35 µm long); rarely reported in Central Europe, including the Eastern Alps (Austria). – **Au**: O, N.

***Verrucaria onegensis* Vain.**

L # – Subs.: sil, int – Alt.: 2–4 – Note: a species with a relatively thick, areolate, brown thallus consisting of verruculose areoles (to 1 mm in diam.), hemispherically protruding (to 0.7 mm in diam.) ascomata resembling those of *V. viridula*, and ellipsoid to oblong ascospores (to 25 µm long); based on a type from NE Europe on dolomite; rare in temperate Europe; the few records from the Alps are not from calcareous rocks, and therefore need confirmation. – **Au**: S. **Sw**: SZ.

***Verrucaria pachyderma* Arnold**

Syn.: *Verrucaria pissina* Nyl.

L – Subs.: sil-aqu – Alt.: 3–5 – Note: a freshwater species of periodically submerged siliceous rocks in upland areas, also known from the British Isles and Scandinavia, with several scattered records from the Alps. – **Au**: V, T, S, K, St. **Fr**: HAl, AMa, Sav, HSav. **It**: TAA.

***Verrucaria papillosa* Ach.**

L – Subs.: cal, int – Alt.: 2–5 – Note: on more or less calciferous rocks in rather humid situations, closely related to *V. viridula*, but a distinct species according to Roux et al. (2014). – **Au**: T, S, K, O, N. **Fr**: AMa, Sav, HSav. **It**: Frl Ven, TAA, Lomb.

***Verrucaria paradolomitica* Servít**

L # – Subs.: cal – Alt.: 5 – Note: a species resembling *V. dolomitica*, with a very thin, epilithic, greyish to brownish, minutely granulose to flattened-verruculose thallus, black, hemispherically protruding ascomata (to *c.* 0.4 mm in diam.), an involucrellum adpressed in the upper third, and ellipsoid ascospores (to 35 µm long); on dolomite, only known from the type locality in the Eastern Alps (Austria). – **Au**: T.

***Verrucaria pilosoides* Servít**

L # – Subs.: cal – Alt.: 2–3 – Note: a calcicolous species with a thallus forming dark grey patches of *c.* 2 cm in diam., consisting of small warts and granules (to 0.1 mm in diam.), convex ascomata mostly immersed in the thallus (to 0.3 mm in diam.), a spreading involucrellum, and ellipsoid ascospores (to 20 µm long); known from a few low elevation sites in the Eastern Alps (Austria). – **Au**: O, N.

***Verrucaria pinguicula* A. Massal.**

Syn.: *Amphoridium integrum* (Nyl.) B. de Lesd., *Involucrothele pinguicula* (A. Massal.) Servít, *Thelidium persicinum* (Hepp) Servít, *Verrucaria integra* (Nyl.) Nyl. *non auct.*, ?*Verrucaria lilacina* A. Massal., *Verrucaria peloclita* Nyl., *Verrucaria persicina* Hepp

L – Subs.: cal – Alt.: 1–5 – Note: on hard, compact limestones, widespread throughout the Alps. – **Au**: V, T, S, K, St, O, N. **Ge**: Ge. **Sw**: BE, SZ, VD. **Fr**: AHP, HAl, AMa, Drô, Isè, Sav, HSav, Var, Vau. **It**: Frl, Ven, TAA, VA, Lig. **Sl**: SlA.

***Verrucaria poeltii* (Servít) Breuss**

Syn.: *Involucrocarpon poeltii* Servít

L – Subs.: cal – Alt.: 4–5 – Note: on very compact calcareous rocks; probably more widespread in the Alps, likely to have been confused with *V. finitima* and *V. fischeri*. – **Au**: V, T, S, K, St, O. **Ge**: OB. **Fr**: HSav.

***Verrucaria polysticta* Borrer**

Syn.: *Dermatocarpon subfuscellum* (Nyl.) Servít, Verrucaria fuscella (Turner) Winch var. nigricans Nyl., *Verrucaria nigricans* (Nyl.) Zschacke, *Verrucaria subfuscella* Nyl.

L – Subs.: cal – Alt.: 2–4 – Note: a species perhaps belonging to *Placopyrenium*; on calcareous rocks, often growing on the thalli of other crustose lichens, mainly *Aspicilia calcarea* and *V. nigrescens*; widespread in the Alps, but regionally not distinguished. – **Au**: ?V, S, St, O, N. **Ge**: OB. **Sw**: ?GR, ?VS. **Fr**: AHP, AMa, Drô, Sav, HSav, Var, Vau.

***Verrucaria polystictoides* Vain.**

L # – Subs.: cal – Alt.: 4 – Note: a species with a relatively thick, areolate, whitish-grey thallus consisting of mostly smooth, angular areoles (to 2 mm in diam.), ascomata several per areole, immersed (to *c.* 0.2 mm in diam), the black ostiolar regions only slightly protruding, the perithecial wall brownish above, otherwise hyaline, ascospores ellipsoid to oblong (hardly exceeding 20 µm in length); based on a type from Finland (on concrete), rarely recorded in Europe, including the Eastern Alps (Austria). – **Au**: St.

***Verrucaria porphyricola* Servít**

L # – Subs.: sil – Alt.: 5 – Note: a rarely collected species of basic siliceous rocks (see Nimis 1993: 747–748), related to *Verruculopsis minuta*. – **It**: TAA.

**Verrucaria praecellens (Arnold) Servít var. praecellens**

Syn.: *Amphoridium praecellens* Arnold, Verrucaria hochstetteri Fr. f. praecellens (Arnold) Zahlbr.; incl. Verrucaria praecellens (Arnold) Servít f. insculpta (Zschacke) Servít

L # – Subs.: cal – Alt.: 3 – Note: a species resembling *Polyblastia dominans*, with a whitish endolithic thallus, immersed ascomata without involucrellum (to 0.7 mm in diam.), the ostiolar region not exceeding the thalline surface, and ovoid to subglobose, halonate ascospores (to 45 µm long); on dolomite, only known from the type locality in the Eastern Alps (Italy). – **It**: TAA.

**Verrucaria praecellens (Arnold) Servít var. obtecta (Arnold) Servít**

Syn.: Amphoridium hochstetteri Fr. f. obtectum Arnold *non Verrucaria obtecta* Müll. Arg.

L # – Subs.: cal int – Alt.: 4 – Note: a variety with a thin, greyish thallus, weakly protruding to entirely immersed ascomata (to *c.* 0.7 mm in diam.) without involucrellum, and broadly ellipsoid ascospores (to *c.* 45 µm long); on calcareous flintstone, only known from the type locality in the Eastern Alps (Germany). – **Ge**: OB.

***Verrucaria praerupta* Anzi**

L # – Subs.: cal – Alt.: 3–5 – Note: closely related to *V. nigrescens*, but differing in several important morphological characters, this species occurs on calcareous rocks in upland areas; from the Alps there are a few scattered records. – **Au**: O. **Fr**: HSav. **It**: Lomb.

***Verrucaria praetermissa* (Trevis.) Anzi**

Syn.: *Leiophloea praetermissa* Trevis., *Verrucaria laevata* *sensu* Körb. *non* Ach. *nec auct.*, *Verrucaria subturicensis* Zahlbr., *Verrucaria turicensis* Zschacke *non* (G. Winter) Stizenb., *Verrucaria zahlbruckneri* Zschacke

L – Subs.: sil-aqu, cal-aqu – Alt.: 2–4 – Note: a silicicolous, probably circumboreal freshwater species, submerged only for very short periods, mostly found along creeks, on mineral-rich siliceous rocks, more rarely on calcareous substrata; material from the Alps needs revision. – **Au**: V, T, K, St, O. **Sw**: VD, VS. **Fr**: AHP, AMa, Drô, Isè, Var. **It**: TAA, Lomb.

***Verrucaria praeviridula* Nyl.**

L # – Subs.: cal – Alt.: 3 – Note: a species resembling *V. glaucovirens*, with a thin, rimulose, greenish thallus, hemispherically protruding ascomata (to 0.2 mm in diam.), an involucrellum adpressed to the perithecial wall and reaching down about one third of the perithecium (*fide* Servít), and oblong to ellipsoid, minute ascospores (to *c.* 12 µm long); on calcareous rocks in shaded situations; rarely recorded in Europe, including the Western Alps (France). – **Fr**: HAl.

***Verrucaria procopii* Servít**

L – Subs.: cal – Alt.: 3 – Note: one of the few species of the genus with isidia, apparently widespread in Europe, but scattered, with a few records from the Eastern Alps (Austria). – **Au**: S, O, N.

***Verrucaria pseudoacrotella* Servít**

Syn.: Verrucaria nigrescens Pers. var. acrotella sensu Anzi *non Verrucaria acrotella* Ach.

L # – Subs.: cal – Alt.: 2–3 – Note: a calcicolous species with a brown-black, spreading, minutely rimose to areolate thallus, the areolae with a rough to pulverulous surface, a medulla with dark brown patches, hemispherically protruding ascomata covered by a thalline layer (to 0.3 mm in diam.), an involucrellum reaching down about half of the perithecium, and ellipsoid ascospores (to *c.* 30 µm long); known from a few localities in the Eastern Alps (Italy). – **It**: Lomb.

***Verrucaria pseudocoerulea* Servít**

L # – Subs.: cal – Alt.: 3 – Note: a calcicolous species with a mainly epilithic, grey-brown, verruculose to areolate thallus, ascomata immersed in the centre of areoles, with a slightly protruding ostiolar region, flask-shaped in longitudinal section (to 0.4 mm wide), with a thin, entire involucrellum merged with the perithecial wall, and broadly ellipsoid to subglobose ascospores (to *c.* 30 µm long); only known from the type locality in the Eastern Alps (Austria). – **Au**: N.

***Verrucaria pseudomacrostoma* Servít**

L # – Subs.: cal – Alt.: 2 – Note: a species with a thin, epilithic, grey, rimose to areolate thallus, the basal layer blackish, ascomata usually one per areole, slightly protruding (to 0.25 mm in diam.), an adpressed involucrellum reaching down to the base of the perithecium and merging with the basal layer, and ascospores to *c.* 30 µm long; on calcareous sandstone, only known from the type locality in the Western Alps (Italy). – **It**: Piem.

***Verrucaria pseudovirescens* Servít**

Syn.: Verrucaria nigrescens var. virescens Anzi *ex* Garov.

L – Subs.: cal – Alt.: 3 – Note: closely related to *V. macrostoma*, but differing in the thinner thallus, smaller perithecia, and smaller spores; known from a few localities only (Italy, Austria, Germany and Russia), but probably more widespread, having been often subsumed under *V. macrostoma* and *V. nigrescens*. – **Au**: St. **It**: Ven.

***Verrucaria pustulifera* Servít**

L # – Subs.: cal – Alt.: 3 – Note: a calcicolous species with a thin, continuous to irregularly rimulose, smooth to granulose, grey thallus, ascomata (to 0.25 mm wide) immersed into slightly protruding verrucae, for the major part covered by a thin thalline layer, involucrellum slightly spreading towards the lower rim and reaching down about one third of the perithecium, and oblong ascospores (to *c.* 35 µm long); rarely recorded in Central Europe, including the Eastern Alps (Austria). – **Au**: N.

***Verrucaria rechingeri* Servít**

L # – Subs.: cal-aqu – Alt.: 4 – Note: a species resembling *V. aquatilis*, with an epilithic, continuous to rimulose-areolate, brown-black thallus forming patches of about 1 cm in diam., a pigmented lower medulla, hemispherically protruding ascomata (to *c.* 0.2 mm in diam.) partly covered by a thalline layer, an involucrellum adpressed to the perithecial wall and fusing with the basal layer, and subglobose to broadly oblong ascospores (to 10 µm long); on calcareous stones near a lake, only known from the type locality in the Eastern Alps (Austria), and from Liguria (outside the Alps). – **Au**: St.

***Verrucaria rivalis* Zschacke**

L # – Subs.: cal-aqu – Alt.: 3–4 – Note: a species close to *V. submersella*, with a very thin, continuous, grey thallus, semi-immersed ascomata (to *c.* 0.5 mm in diam.), a carbonised involucrellum adpressed to the protruding part of the perithecia and fusing with the perithecial wall, and ellipsoid ascospores (to *c.* 30 µm long); on calcareous stones in montane to subalpine streams, reported from a few scattered localities in the Alps. – **Au**: T, S. **Fr**: AHP.

***Verrucaria ruderum* DC.**

Syn.: *Amphoridium ruderum* (DC.) Servít

L # – Subs.: cal – Alt.: 2–4 – Note: mostly on walls made of mortar and cement; perhaps a synonym of other species, with a few scattered records from the Alps. – **Au**: St. **Fr**: Isè, Vau. **It**: Ven, Lomb, VA.

***Verrucaria ruinicola* Servít**

L # – Subs.: cal – Alt.: 1 – Note: this species is only known from the type collection in the surroundings of Spotorno. – **It**: Lig.

***Verrucaria saprophila* (A. Massal.) Trevis.**

Syn.: *Amphoridium saprophilum* A. Massal., *Thelidium saprophilum* (A. Massal.) Servít

L # – Subs.: cal – Alt.: 3–5 – Note: a rarely reported, southern – to central-European species of rather shaded calcareous rocks, which needs further study. – **Au**: ?V, St, O, N. **Fr**: HSav. **It**: Ven, TAA.

***Verrucaria savonensis* Servít**

L # – Subs.: sil – Alt.: 2 – Note: a silicicolous species with an epilithic, brownish-grey, rimose to areolate thallus forming patches to 3 cm in diam., with a brown to blackish-brown basal layer, almost entirely immersed ascomata (to *c.* 0.2 mm in diam.), an adpressed involucrellum reaching down almost to the base of the perithecium and merging with the basal layer, and oblong to ellipsoid ascospores (to *c.* 25 µm long); only known from the type locality at the base of the Western Alps (Italy). – **It**: Lig.

***Verrucaria sbarbaronis* B. de Lesd.**

L # – Subs.: cal – Alt.: 3–4 – Note: this calcicolous species described from the base of the Western Alps and known also from Greece needs further study. – **Au**: V, O. **It**: Lig. **Sl**: SlA.

***Verrucaria schaereriana* Servít**

L # – Subs.: cal – Alt.: ? – Note: a species with a mainly endolithic to very thin epilithic, continuous, whitish to pale brown thallus, the black immersed perithecia (0.2–0.3 mm in diam.) hardly protruding and with a thin thalline annulus, without an involucrellum, more or less spherical with a conical ostiolar region, the wall black-brown in the uppermost part but pigmentation fading towards the base, 8-spored asci, and ellipsoid to fusiform, simple ascospores (23–36 × 12–15 μm); only known from the type locality in Switzerland. – **Sw**: Sw.

***Verrucaria schindleri* Servít**

Syn.: *Verrucaria hypophaea* (J. Steiner & Zahlbr.) Servít, Verrucaria rupestris Schrad. var hypophaea J. Steiner & Zahlbr.

L # – Subs.: cal – Alt.: 2–4 – Note: a species of the *V. muralis*-group, with an endolithic to thin-epilithic, whitish, rimose thallus, ascomata (to 0.3 mm in diam.) partly protruding from the rock, a plane involucrellum (to 0.4 mm broad), and ellipsoid ascospores (to *c.* 25 µm long); on calcareous stones and boulders in humid situations; rather rare, but perhaps overlooked, throughout Central Europe; with several scattered records from the Alps, from the lowlands to treeline. – **Au**: V, S, St, O, N. **Ge**: Ge. **Fr**: AHP. **It**: Piem, Lig.

***Verrucaria selecta* Servít**

L # – Subs.: cal – Alt.: 4 – Note: a calcicolous species resembling *V. caerulea*, with a thin, spreading, whitish to yellowish thallus which is rimose-areolate only around the perithecia, hemispherically protruding ascomata (to 0.2 mm in diam.), an adpressed involucrellum reaching down to the base of the perithecium, and oblong ascospores (to *c.* 25 µm long); only reported from a few scattered localities in the Eastern Alps (Austria). – **Au**: T, O.

***Verrucaria serlosensis* Servít**

L # – Subs.: cal – Alt.: 5 – Note: a species with an endolithic thallus, black, protruding ascomata (to 0.4 mm in diam.) without an involucrellum, and broadly ellipsoid to subglobose ascospores (to *c.* 30 µm long); on limestone, only known from the type locality in the Eastern Alps (Austria). – **Au**: T.

***Verrucaria serpentinicola* (Servít) Servít**

Syn.: Dermatocarpon subpruinosum (Servít) Servít var. serpentinicola Servít

L – Subs.: sil – Alt.: 3 – Note: a species with a spreading, epilithic, rimose to areolate, brownish-grey thallus with a dark basal layer, ascomata immersed in the centre of areoles (to 0.25 mm in diam.), an involucrellum adpressed to the perithecial wall, reaching down to the base and merging with the basal layer, and ellipsoid ascospores (to *c.* 20 µm long); on ophiolithic rocks at low elevations; widespread but rare in Europe, including the Eastern Alps (Austria). – **Au**: St.

***Verrucaria slavonica* Servít**

L # – Subs.: cal – Alt.: 1–2 – Note: a very poorly known species described from Eastern Europe and reported by Servít also from the surroundings of Spotorno in Liguria. – **It**: Lig.

***Verrucaria slovaca* Servít**

L # – Subs.: cal – Alt.: 3–4 – Note: a species with a spreading, hemiendolithic, whitish thallus, finally hemispherically protruding ascomata (to *c.* 0.35 mm in diam.) often with a thalline annulus and separated by a cleft from the thallus, an adpressed involucrellum reaching down to the base of the perithecium, and oblong to ellipsoid ascospores (to *c.* 30 µm long); on rather shaded surfaces of calcareous rocks in Central Europe, with a few records from the Western Alps (France). – **Fr**: AHP, AMa.

***Verrucaria sphaerospora* Anzi**

Syn.: *Catapyrenium sphaerosporum* (Anzi) Arnold, *Dermatocarpon anzianum* Servít, *Dermatocarpon sphaerosporum* (Anzi) Servít, *Involucrocarpon sphaerosporum* (Anzi) Servít; incl. *Endocarpon pulvinulosum* Harm., *Dermatocarpon pulvinulosum* (Harm.) Lettau

L – Subs.: cal, int, bry, bry-cal – Alt.: 2–5 – Note: a very characteristic species forming a complex of still poorly known entities growing on calciferous sandstone, often on walls, that probably belongs to *Verruculopsis*. – **Au**: V, T, S, K, St, O. **Sw**: GR, VS. **Fr**: AHP, HAl, AMa, Drô, HSav. **It**: Lomb, Piem.

***Verrucaria spotornensis* Servít**

L # – Subs.: sil – Alt.: 2 – Note: a silicicolous species with an epilithic, grey, continuous to areolate thallus, the areoles smooth to minutely verruculose and with minute dark maculae, hemispherically protruding ascomata (to 0.4 mm in diam.), an involucrellum adpressed to the perithecial wall und reaching down to the base of the perithecium, and oblong to ellipsoid ascospores (to *c.* 25 µm long); only known from the type locality at the base of the Western Alps (Italy). – **It**: Lig.

***Verrucaria strasseri* Servít**

L # – Subs.: cal – Alt.: 2 – Note: a species with an endolithic thallus becoming minutely granulose with age, ascomata (to *c.* 0.4 mm in diam.) with a flattened ostiolar region not exceeding the thallus, a poorly developed, adpressed involucrellum, and oblong ascospores (to *c.* 30 µm long); on calcareous conglomerate; only known from the type locality in the Eastern Alps (Italy). – **It**: TAA.

***Verrucaria subcincta* Nyl.**

Syn.: ?*Verrucaria cincta* Hepp *non* Fée

L # – Subs.: cal – Alt.: 3–5 – Note: a calcicolous species of the *V. muralis*-group, with a very thin, whitish thallus, ascomata (to 0.3 mm in diam.), a dimidiate involucrellum, and ellipsoid to oblong ascospores (to *c.* 20 µm long); rare throughout Central Europe, with a few scattered records from the Alps. – **Au**: N. **Fr**: Sav, HSav.

***Verrucaria subdolosa* Servít**

L # – Subs.: cal – Alt.: 2–3 – Note: a species resembling *V. dolosa*, with a spreading, thin, olive-grey, continuous to subrimulose, minutely verruculose to granular thallus, hemispherically protruding to subsessile ascomata (to 0.3 mm in diam.), an involucrellum reaching down to the base of the perithecium and there spreading, and ellipsoid ascospores (exceeding 25 µm in length); on calcareous rocks at low elevations; here and there throughout Central Europe, with scattered records from the Alps. – **Au**: V. **It**: Frl, Piem. **Sl**: SlA.

***Verrucaria subintegra* Servít**

L # – Subs.: cal – Alt.: 3 – Note: this calcicolous species differs from *V. acrotella* in several morphological characters and in the ecology. – **Au**: O. **Fr**: AMa.

***Verrucaria submersella* Servít**

Syn.: *Verrucaria submersa* Schaer. *non* Borrer

L # – Subs.: sil-aqu, cal-aqu – Alt.: 2–5 – Note: closely related to *V. elaeomelaena* and *V. funckii*, but differing in several morphological characters, this freshwater lichen needs further study. – **Au**: T, S, St, O, N. **Ge**: Ge. **Sw**: SG. **Fr**: AMa. **It**: TAA, Lomb.

***Verrucaria subtilis* Müll. Arg.**

Syn.: *Amphoridium subtile* (Müll. Arg.) Servít

L # – Subs.: cal – Alt.: 4 – Note: a species resembling in habitus *V. caesiopsila*, with a mainly endolithic to very thin epilithic, continuous, brown-grey thallus, the black perithecia (0.2–0.3 mm in diam.) semi-immersed in the rock, more or less spherical, the wall black-brown throughout, 8-spored asci, and ellipsoid to obovoid, simple ascospores (20–25 × 10–13 μm); on dolomite, only known from the type locality in the Eastern Alps (Switzerland). – **Sw**: VS.

***Verrucaria subtruncatula* B. de Lesd.**

L # – Subs.: cal – Alt.: 3 – Note: a species resembling *V. caerulea* but with larger ascospores, a thin, whitish, rimulose thallus, innate ascomata with a truncate apex (to 0.5 mm in diam.), a dimidiate involucrellum, and oblong ascospores (even exceeding 30 µm in length); on calcareous stones at low elevations; rare throughout Europe, including the Alps. – **Au**: N. **Fr**: Drô.

***Verrucaria tabacina* (A. Massal.) Trevis.**

Syn.: *Lithocia tabacina* A. Massal.

L # – Subs.: cal – Alt.: 2–3 – Note: a species of calcareous rocks reported from several localities in Central Europe; it has been frequently considered as a synonym of *V. nigrescens* and its distribution is therefore poorly known. – **Au**: N. **Ge**: Ge. **Sw**: GR. **It**: Ven.

***Verrucaria tapetica* Körb.**

L # – Subs.: sil – Alt.: 4 – Note: a species with a spreading, bluish-grey to brownish, rimose to areolate thallus, relatively small, immersed ascomata, and ellipsoid ascospores (to *c.* 25 µm long); on siliceous boulders in sunny places, often together with *Sarcogyne privigna*; here and there in Europe, including the Eastern Alps (Austria). – **Au**: T.

***Verrucaria tectorum* (A. Massal.) Körb.**

Syn.: *Lithocia tectorum* A. Massal., Verrucaria viridula (Schrad.) Ach. f. tectorum (A. Massal.) J.R. Laundon

L – Subs.: cal – Alt.: 1–3 – Note: mainly on man-made substrata, including mortar walls. frequently confused with V. macrostoma f. furfuracea, which is sorediate and not isidiate, and has a thicker thallus (see Roux et al. 2014). – **Au**: K, St. **Sw**: GR. **Fr**: AHP, AMa, Drô, Vau. **It**: Frl, Ven, Lomb, Piem, Lig. **Sl**: SlA.

***Verrucaria terminalis* Servít**

L # – Subs.: cal – Alt.: 2 – Note: a calcicolous species with a thin, brownish-white, rimose to areolate thallus forming patches to 2 cm in diam., hemispherically protruding ascomata (to 0.3 mm in diam.), an entire involucrellum, and ellipsoid ascospores (to *c.* 25 µm long); only known from the type locality at the base of the Western Alps (Italy). – **It**: Lig.

***Verrucaria transfugiens* Zschacke**

L # – Subs.: cal – Alt.: 3–5 – Note: a calcicolous species with a thallus forming spreading, whitish-grey patches, entirely immersed ascomata (to 0.5 mm in diam.) without involucrellum, the perithecial wall dark brown throughout, and ellipsoid ascospores (to *c.* 25 µm long); in Europe here and there, with scattered records from the Alps, but perhaps not always distinguished. – **Au**: S, O, N. **Fr**: AHP.

***Verrucaria transiliens* (Arnold) Lettau**

Syn.: *Amphoridium transiliens* Arnold

L – Subs.: cal – Alt.: 2–4 – Note: on calcareous pebbles (*e.g.* on calciferous sandstone) near the ground, especially in clearings of woodlands and on track sides; probably overlooked, or confused with similar species, and perhaps more widespread in the Alps. – **Au**: V, St, O, N. **It**: Frl, Piem.

***Verrucaria triglavensis* Servít**

L # – Subs.: cal – Alt.: 3–5 – Note: a calcicolous species with a mainly endolithic thallus emerging only as brown dots or patches, conically to hemispherically protruding ascomata (to *c.* 0.3 mm in diam.), a thick, adpressed involucrellum reaching down about a third of the perithecium, and ellipsoid ascospores (to *c.* 30 µm long); only known from the Eastern Alps (Austria and Slovenia). – **Au**: O. **Sl**: SlA.

***Verrucaria truncigena* Breuss**

L # – Subs.: cor – Alt.: 3 – Note: a species resembling *V. ulmi*, but perithecia with an entire involucrellum, and ascospores smaller (only somewhat exceeding 20 µm in length); on bark *Fagus* in a humid mixed forest, so far only known from the type locality in the Eastern Alps (Austria). – **Au**: O.

***Verrucaria tuerkii* Breuss**

L # – Subs.: cor – Alt.: 2 – Note: a species with a thin, greenish-grey, spreading thallus, ascomata at first immersed but later hemispherically protruding with the naked, black apex (to *c.* 0.2 mm in diam.), an adpressed involucrellum reaching down to the base of the perithecium and there somewhat spreading, and oblong to ellipsoid ascospores (to *c.* 30 µm long); on bark *Fagus* in a humid mixed forest, so far only known from type locality in the Eastern Alps (Austria). – **Au**: N.

***Verrucaria tunicata* Müll. Arg.**

L # – Subs.: sil – Alt.: 4 – Note: a species with a thin, spreading, ochraceous, finally rimose-areolate thallus, immersed to subsessile perithecia (to 0.5 mm in diam.) covered by a thalline layer, and broadly ellipsoid to subglobose ascospores (to *c.* 25 µm long or even longer); on metamorphic schists, only known from the type locality in the Western Alps (Switzerland). – **Sw**: VS.

***Verrucaria ulmi* Breuss**

L # – Subs.: cor – Alt.: 2–3 – Note: a species with a thin thallus forming small blackish patches, hemispherically protruding ascomata (to 0.35 mm in diam.) laterally covered by a thin thalline layer, a tightly adpressed involucrellum reaching the base of the perithecium, and ellipsoid ascospores (to *c.* 30 µm long); on bark of deciduous trees (*e.g. Ulmus*), with a few records, all from Central Europe, including the Eastern Alps (Austria). – **Au**: St, N.

***Verrucaria umbrinula* Nyl.**

L # – Subs.: sil – Alt.: 3–4 – Note: a silicicolous species with a dark olive to blackish, granulose to scurfy thallus, black ascomata (to *c.* 0.25 mm in diam.), and oblong ascospores (to *c.* 20 µm long), based on a type from Northern Finland; from the Alps there are some scattered records, but conspecificity with material from Central Europe needs confirmation. – **Au**: T, K. **Sw**: BE, VS.

***Verrucaria valpellinensis* B. de Lesd.**

L # – Subs.: sil – Alt.: 5 – Note: a silicicolous species with a rather thin, brownish black thallus of 0.2–0,3 mm wide, flat areolae forming irregular patches (2–3 cm in diam.), without a visible hypothallus, black subglobose perithecia which are isolated on in groups of 2–3, the apex subumbilicate near the ostiole, the wall black throughout, a I+ red hymenium, 8-spored asci, and ellipsoid to oblong-ellipsoid, simple, hyaline ascospores measuring 15–18 × 8–9 μm; only known from the type locality at 2,400 m in Valle d’Aosta (Italy). – **It**: VA.

***Verrucaria varigottiana* B. de Lesd.**

L # – Subs.: ?cal – Alt.: 1–2 – Note: a species with a thin thallus, sometimes continuous, sometimes of minute, scattered, dot-like units, forming irregular patches (1–1.5 cm in diam.), black, shiny, semi-immersed, irregularly globose perithecia (*c.* 0.1 mm in diam.) with a continuous black wall, indistinct paraphyses, and simple, hyaline oblong to ellipsoid ascospores (16–18 × 9–11 μm); only reported from the type locality (Saracene castle of Varigotti, Liguria). At least an isotype could be located in the Herb. Sbarbaro (GDOR). – **It**: Lig.

***Verrucaria veronensis* A. Massal.**

Syn.: *Amphoridium veronense* (A. Massal.) A. Massal.

L – Subs.: cal – Alt.: 2–3 – Note: a species of calcareous rocks and dolomite, reported from several localities in Southern and Central Europe, with some scattered records from the Alps. – **Au**: V, T, N. **Fr**: HSav, Vau. **It**: Ven, TAA.

***Verrucaria vicinalis* Arnold**

Syn.: *Thelidium vicinale* (Arnold) Servít

L – Subs.: cal – Alt.: 2–4 – Note: a calcicolous species resembling *V. eusebii* (syn. *V. amylacea* Hepp), with a very thin thallus indicated by bluish patches, and medium-sized perithecia with a dimidiate involucrellum; widespread in the mountains of Europe (Alps, Carpathians, Pyrenees), with a few scattered records from the Alps. – **Fr**: Sav. **It**: Frl, TAA.

***Verrucaria vindobonensis* Zschacke**

Syn.: Verrucaria vindobonensis Zschacke var. prominula Servít

L # – Subs.: cal, ?sil – Alt.: 2–3 – Note: a species with a thin, olive-grey, rimose thallus forming patches of *c.* 1 cm in diam., ascomata (to *c.* 0.3 mm in diam.) immersed in the centre of the areoles and only slightly protruding, involucrellum adpressed to the perithecial wall and reaching down about one third of the perithecium, and oblong ascospores (hardly reaching 25 µm in length); on limestone at low elevations (records from siliceous rocks need re-evaluation); apparently widespread but perhaps not always distinguished; in the study area reported only from the Eastern (Austria) and the Western (Liguria) Alps. – **Au**: N. **It**: Lig.

***Verrucaria viridula* (Schrad.) Ach.**

Syn.: *Amphoridium leightonii* (A. Massal.) Arnold, *Amphoridium viridulum* (Schrad.) Servít, *Endocarpon viridulum* Schrad., *Verrucaria griseorubens* Mig., *Verrucaria leightonii* A. Massal. *non* Hepp, *Verrucaria obductilis* (Nyl.) Zschacke, *Verrucaria polygonia* Körb.

L – Subs.: cal, sil – Alt.: 1–3 – Note: an early coloniser of calciferous substrata, most common on small pebbles, also in urban areas (*e.g.* on roofing tiles); easily mistaken for *V. macrostoma*; widespread throughout the Alps. – **Au**: V, T, S, K, St, O, N. **Ge**: Schw. **Sw**: BE, GR, LU, SZ, UR. **Fr**: AHP, HAl, AMa, Drô, Sav, HSav, Vau. **It**: Frl, Ven, TAA, Lomb, Piem, Lig.

***Verrucaria wolferi* Zschacke**

L # – Subs.: cal-aqu – Alt.: 4 – Note: a species with a thin, smooth, glossy, bronze-coloured thallus, protruding ascomata (to 0.35 mm in diam.), a tightly adpressed involucrellum reaching down to the base of the perithecium, and broadly ellipsoid to subglobose ascospores (to *c.* 12 µm long); on calcareous stones along a creek, only known from the type locality in Switzerland. – **Sw**: GR.

***Verrucaria xylophila* Croz.**

L # – Subs.: xyl – Alt.: 3 – Note: a species with a granular, grey-brown thallus, black and somewhat protruding ascomata (to 0.3 mm in diam.), an adpressed involucrellum, and globose to subglobose ascospores (*c.* 10 µm in diam.); on wood near a stream, so far only known from the type locality in the Western Alps (France). – **Fr**: HSav.

***Verrucaria xyloxena* Norman**

Syn.: *Involucrothele velutinoides* (Hellb.) Servít, *Thelidium velutinoides* (Hellb.) Servít, Verrucaria acrotella Ach. f. terrestris Arnold, *Verrucaria floerkei* Trevis., *Verrucaria melaenella* Vain., *Verrucaria terrestris* (Arnold) Vain. *non* (Th. Fr.) Tuck., *Verrucaria velutinoides* Hellb.

L – Subs.: ter-sil, ter-cal – Alt.: 2–4 – Note: on calciferous soil, often associated with acrocarpous mosses, mostly in upland areas but usually below the alpine belt; in the study area hitherto reported from the Eastern Alps only (Austria, Italy), but easy to overlook, and perhaps more widespread. – **Au**: K, St, O, N. **It**: TAA.

***Verrucaria zschackei* Riedl**

Syn.: Verrucaria aethiobola Wahlenb. f. calcarea Arnold, *Verrucaria calcaria* Zschacke *non* *V. calcarea* (L.) Humb. *nec* C. Knight

L # – Subs.: cal-aqu – Alt.: 2–3 – Note: a species close to *V. hydrela*, with a spreading, smooth, glossy thallus which is somewhat rimose around the fruiting bodies, hemispherically protruding ascomata (to *c.* 0.2 mm in diam.) covered by a thalline layer, involucrellum adpressed to the perithecial wall and reaching down to the base of the perithecium, and ellipsoid ascospores (to *c.* 20 µm long); on calcareous rocks along streams, widespread in Central Europe, with scattered records from the Alps. – **Au**: St, O, N. **Fr**: AMa, Drô, Vau. **Sl**: SlA.

***Verrucula arnoldaria* Nav.-Ros. & Cl. Roux**

L – Subs.: cal-par – Alt.: 2 – Note: a rare species with a thick, brownish-grey thallus of convex to subspherical areoles (or conserving the shape of the host thallus) covered by a crystalline pruina, and ellipsoid ascospores (to *c.* 15 µm long); lichenicolous on *Caloplaca arnoldii* on calcareous rocks or schists containing some calcium; widespread in SW Europe, including a record from the Western Alps (France). – **Fr**: Vau.

***Verrucula biatorinaria* (Zehetl.) Nav.-Ros. & Cl. Roux**

Syn.: *Verrucaria biatorinaria* Zehetl.

L – Subs.: cal-par – Alt.: 3–5 – Note: a species with a thick, grey-brown thallus forming patches of up to 3 cm in diam. consisting of verrucose to subsquamulose areoles, and ellipsoid ascospores (to *c.* 15 µm long); a lichenicolous lichen on *Caloplaca biatorina* at high elevations; widespread in the Northern Hemisphere, including the Alps, but altogether rare; apparently somehow more frequent in the Southern and Western Alps. – **Au**: K. **Fr**: AHP, HAl, AMa. **It**: Ven, TAA, Piem.

***Verrucula clauzadaria* Nav.-Ros. & Cl. Roux**

L – Subs.: sax-par – Alt.: 2–3 – Note: a recently-described parasite on *Caloplaca clauzadeana*, perhaps more widespread in the Alps. – **Fr**: Vau.

***Verrucula coccinearia* (Zehetl.) Nav.-Ros. & Cl. Roux**

Syn.: *Verrucaria coccinearia* Zehetl.

L – Subs.: cal-par – Alt.: 3–5 – Note: a rare species with a grey to brown thallus consisting of a few, thin areoles forming small isles (to *c.* 2 mm in diam.) on the thallus of the host lichen, with subglobose to globose ascospores (to *c.* 12 µm in diam.); with its host *Caloplaca coccinea* on limestone, mainly at high elevations; so far known from a few localities in the Alps, but perhaps overlooked. – **Au**: St. **Sw**: VS. **It**: Piem.

***Verrucula elegantaria* (Zehetl.) Nav.-Ros. & Cl. Roux**

Syn.: *Verrucaria elegantaria* Zehetl.

L – Subs.: cal-par – Alt.: 3–5 – Note: parasitic on *Xanthoria elegans*, certainly more widespread in the Alps. – **Au**: T, S, St. **Fr**: AHP. **It**: Frl, Ven, TAA, Piem.

***Verrucula granulosaria* (Clauzade & Zehetl.) Nav.-Ros. & Cl. Roux**

Syn.: *Verrucaria granulosaria* Clauzade & Zehetl.

L – Subs.: cal-par – Alt.: 1–3 – Note: parasitic on *Caloplaca granulosa*, certainly more widespread in the Alps, mainly at low elevations. – **Fr**: AHP, Vau.

***Verrucula helvetica* (B. de Lesd.) Nav.-Ros. & Cl. Roux**

Syn.: *Dermatocarpon helveticum* (B. de Lesd.) Frey, *Endopyrenium helveticum* B. de Lesd., *Verrucaria helveticorum* Zehetl. *non Verrucaria helvetic*a B. de Lesd.

L – Subs.: cal-par – Alt.: 2–4 – Note: parasitic on *Caloplaca cirrochroa*; certainly more widespread in the Alps, but largely overlooked. – **Au**: St. **Ge**: OB. **Fr**: AHP, AMa, Vau.

***Verrucula inconnexaria* Nav.-Ros. & Cl. Roux**

L – Subs.: cal-par – Alt.: 2–3 – Note: parasitic on *Caloplaca inconnexa*, hitherto known only from the Western Alps (France), but perhaps more widespread. – **Fr**: HSav, Var.

***Verrucula lactearia* Nav.-Ros. & Cl. Roux**

L – Subs.: cal-par – Alt.: 2 – Note: a rare species with a thick, brownish-grey thallus forming small isles of about 2–4 mm in diam. on the host lichen, with ellipsoid ascospores (to *c.* 15 µm long); a lichenicolous lichen on *Caloplaca lactea* on calcareous pebbles; widespread in SW Europe, including the Western Alps (France). – **Fr**: Var.

***Verrucula latericola* (Erichsen) Nav.-Ros. & Cl. Roux**

Syn.: *Verrucaria latericola* Erichsen

L – Subs.: cal-par – Alt.: 2–4 – Note: a lichenicolous lichen on members of the *Caloplaca saxicola*-group, especially *C. pusilla*, probably more widespread in the Alps. – **Au**: B. **Fr**: AHP.

***Verrucula microspora* Nav.-Ros. & Cl. Roux**

L – Subs.: cal-par – Alt.: 2–4 – Note: a rare species with a thick, brownish thallus consisting of polygonal areoles and forming small isles (*c.* 2–4 mm in diam.) on the host lichen, with ellipsoid ascospores (to *c.* 10 µm long); a lichenicolous lichen on *Caloplaca dalmatica* on calcareous rocks; widespread in SW Europe, with a few records from the Western Alps (France). – **Fr**: AHP, AMa.

***Verrucula polycarparia* Nav.-Ros. & Cl. Roux**

L – Subs.: cal-par – Alt.: 2 – Note: parasitic on *Caloplaca polycarpa*; in the study area so far only reported from the Western Alps (France), but perhaps more widespread. – **Fr**: AMa.

***Verrucula protearia* (Zehetl.) Nav.-Ros. & Cl. Roux**

Syn.: *Verrucaria protearia* Zehetl.

L – Subs.: cal-par – Alt.: 3–5 – Note: parasitic on *Caloplaca proteus*. – **Au**: T, St. **Ge**: OB. **Sw**: GR. **Fr**: HSav. **It**: TAA.

***Verrucula pusillaria* Nav.-Ros. & Cl. Roux**

L – Subs.: cal-par – Alt.: 2–4 – Note: parasitic on *Caloplaca pusilla*. – **Fr**: AHP, AMa, Var.

***Verruculopsis flavescentaria* Gueidan, Nav.-Ros. & Cl. Roux**

L – Subs.: cal-par – Alt.: 1–2 – Note: parasitic on *Caloplaca flavescens*, this species has a narrower range than its host, as it mostly occurs in rather shaded situations; in the study area so far only reported from the Western Alps (France), but perhaps more widespread. – **Fr**: AHP, AMa, Var, Vau.

**Verruculopsis lecideoides (A. Massal.) Gueidan & Cl. Roux var. lecideoides**

Syn.: *Thrombium lecideoides* A. Massal., *Verrucaria lecideoides* (A. Massal.) Trevis.

L – Subs.: cal – Alt.: 1–5 – Note: on calciferous rocks, mostly limestone and dolomite, but also on base-rich siliceous substrata, in exposed situations (*e.g.* on the top of isolated boulders); apparently more frequent in the Southern and Western Alps. See also note on *Verrucaria beltraminiana*. – **Au**: N. **Sw**: BE, GR, VD, VS. **Fr**: AHP, HAl, AMa, Drô, Isè, Sav, HSav, Var, Vau. **It**: Frl, Ven, TAA, Lomb, Piem, Lig.

**Verruculopsis lecideoides (A. Massal.) Gueidan & Cl. Roux var. minuta (Hepp) ined. (provisionally placed here, ICN Art. 36.1b)**

Syn.: Verrucaria lecideoides (A. Massal.) Trevis. var. minuta Hepp, *Verrucaria minuta* (Hepp) Zschacke

L – Subs.: sil, int, cal – Alt.: 2–3 – Note: a variety with smaller (about half the size as in *V. lecideoides*), brownish to blackish areoles, and somewhat smaller ascospores; not consistently distinguished, but apparently widespread in Europe, including the Alps. – **Au**: T. **Fr**: AHP, AMa, HSav, Var, Vau. **It**: Ven, TAA, Lomb, Lig.

***Verruculopsis poeltiana* (Clauzade & Cl. Roux) Gueidan, Nav.-Ros. & Cl. Roux**

Syn.: *Verrucaria poeltiana* Clauzade & Cl. Roux

L – Subs.: cal-par – Alt.: 1–2 – Note: parasitic on *Caloplaca aurantia*; in the study area so far only reported from the Western Alps (France), but perhaps more widespread, at low elevations. – **Fr**: AMa, Var, Vau.

***Vezdaea aestivalis* (Ohlert) Tscherm.-Woess & Poelt**

Syn.: *Biatora aestivalis* (Ohlert) Lindau, *Catillaria byssacea* Vězda, *Lecidea aestivalis* Ohlert, *Pachyascus byssaceus* (Vězda) Vězda

L – Subs.: cor, bry, par, sil, deb – Alt.: 2–4 – Note: a mild-temperate to Mediterranean-Atlantic, ephemeral species found on epiphytic bryophytes, mosses, plant debris, soil, much more rarely on mossy trunks of deciduous trees with a base-rich bark. Being inconspicuous, and likely to be confused with the much more common *Bilimbia sabuletorum*, this species might be more widespread, but is certainly not common in the Alps. – **Au**: T, S, K, St, N. **Ge**: OB. **Sw**: GL, GR, SG, SZ. **Fr**: AMa, Var, Vau. **It**: Frl.

***Vezdaea dawsoniae* Döbbeler**

L – Subs.: fol – Alt.: 2–3 – Note: a species resembling *V. stipitata*, with a distinct cylindrical stipe, based on a type from Papua New Guinea (conspecificity of European specimens in need of re-evaluation); on leaves and uppermost parts of branches of evergreen shrubs (*e.g. Buxus*) in the understory of broad-leaved forests in very humid, winter-mild sites, with a few records from the Western Alps (France). – **Fr**: AMa, Isè.

***Vezdaea retigera* Poelt & Döbbeler**

L – Subs.: ter-cal, bry – Alt.: 2–5 – Note: a species with a thallus composed of goniocysts provided with short hyphal spines, brown apothecia (to 0.35 mm in diam.) with a rough surface when moist, branched and anastomosing interascal filaments forming loose envelopes around the asci, and hyaline, simple, ellipsoid ascospores (to *c.* 20 µm long); on soil and moribund bryophytes, widespread in the Northern Hemisphere but rarely collected, probably due to its minute size, with a few records from the Alps. – **Au**: T, K, St, N. **Sw**: BE.

***Vezdaea rheocarpa* Poelt & Döbbeler**

L – Subs.: bry, cor-bry – Alt.: 2–3 – Note: a species with a thallus composed of goniocysts provided with long hyphal spines, translucent to grey, flat apothecia, branched interascal filaments (mostly shorter than the asci), and hyaline, simple, ellipsoid ascospores (to *c.* 20 µm long) with a finally verrucose perispore; on bryophytes and plant debris, mostly in deciduous forests; widespread in the Northern Hemisphere but rarely collected, probably due to its minute size, with scattered records from the Alps. – **Au**: St. **Sw**: SG, UW. **Fr**: AHP.

***Vezdaea stipitata* Poelt & Döbbeler**

L – Subs.: bry, par, cor-bry, xyl-bry – Alt.: 2–4 – Note: a species resembling *V. leprosa* but lacking goniocysts, with a thallus forming an indistinct granular layer, slightly brownish, stipitate apothecia (to *c.* 0.2 mm high and wide) recalling those of a minute calicioid lichen, the stipe (to *c.* 80 µm in diam.) composed of subparallel hyphae, scarce and indistinct interascal filaments, and hyaline, obovoid, 1-septate ascospores (to *c.* 15 µm long) with slightly unequal cells. – **Au**: T, S, K, St. **Sw**: UW.

***Violella fucata* (Stirt.) T. Sprib.**

Syn.: *Lecidea fucata* Stirt., *Megalospora fucata* (Stirt.) H. Olivier, *Mycoblastus fucatus* (Stirt.) Zahlbr., *Mycoblastus sterilis* Coppins & P. James

L – Subs.: cor, xyl – Alt.: 2–4 – Note: a cool-temperate lichen found on bark in humid montane woodlands; perhaps overlooked in the Southern and Western Alps, being mostly sterile. – **Au**: V, T, S, K, St, O, N, B. **Ge**: OB, Schw. **Sw**: AP, BE, FR, GL, GR, LU, SG, SZ, TI, UR, UW, VD, VS. **It**: TAA. **Sl**: SlA, Tg.

***Vulpicida juniperinus* (L.) J.-E. Mattsson & M.J. Lai**

Syn.: *Cetraria juniperina* (L.) Ach., Cetraria juniperina (L.) Ach. var. tubulosa Schaer., Cetraria tilesii *auct. non* Ach., *Cetraria tubulosa* (Schaer.) Zopf, *Lichen juniperinus* L., *Tuckermannopsis juniperina* (L.) Hale, *Vulpicida tubulosus* (Schaer.) J.-E. Mattsson & M.J. Lai

L – Subs.: ter-cal, deb, cor – Alt.: 2–6 – Note: this mainly subarctic-subalpine to boreal-montane species is found on calciferous mineral soil in dry Alpine grasslands and on wind-exposed ridges, more rarely on the twigs of shrubs; widespread throughout the Alps. – **Au**: V, T, S, K, St, O, N. **Ge**: OB, Schw. **Sw**: BE, FR, GR, LU, SG, SZ, TI, UR, UW, VD, VS. **Fr**: AHP, HAl, AMa, Isè, Sav, HSav, Vau. **It**: Frl, Ven, TAA, Lomb, Piem, VA. **Sl**: SlA. **Li**.

**Vulpicida pinastri (Scop.) J.-E. Mattsson & M.J. Lai var. pinastri**

Syn.: *Cetraria caperata* *sensu* Vain., Cetraria juniperina (L.) Ach. var. pinastri (Scop.) Ach., *Cetraria pinastri* (Scop.) Gray, *Lichen pinastri* Scop., *Platysma pinastri* (Scop.) Frege, *Tuckermannopsis pinastri* (Scop.) Hale

L – Subs.: cor, xyl, sil – Alt.: 2–5 – Note: a subarctic-subalpine to boreal-montane, circumpolar species found on basal parts of trunks, especially of conifers, and on twigs with a long snow cover, often associated with *Parmeliopsis*, with optimum near treeline; widespread and common throughout the Alps. – **Au**: V, T, S, K, St, O, N, B. **Ge**: OB, Schw. **Sw**: BE, FR, GL, GR, LU, SG, SZ, TI, UR, UW, VD, VS. **Fr**: AHP, HAl, AMa, Isè, Sav, HSav, Var, Vau. **It**: Frl, Ven, TAA, Lomb, Piem, VA, Lig. **Sl**: SlA, Tg. **Li**.

**Vulpicida pinastri (Scop.) J.-E.Mattsson & M.J. Lai var. soralifera (Frey) J.-E. Mattsson**

Syn.: Cetraria caperata Vain. var. soralifera Frey

L – Subs.: cor – Alt.: 3–4 – Note: a variety with laminal, roundish soralia, mostly on bark; widespread in the Holarctic Region, in Europe most common in Scandinavia, with several records from the Swiss Alps only. – **Sw**: BE, GR, UW, VS.

***Waynea adscendens* V.J. Rico**

L – Subs.: cor – Alt.: 2 – Note: a Mediterranean-Atlantic species found on the bark of more or less isolated, old broad-leaved trees in very humid areas, with a few records from the Western Alps (France). – **Fr**: AMa, Drô, Vau.

***Waynea stoechadiana* (Abbassi Maaf & Cl. Roux) Cl. Roux & P. Clerc**

Syn.: *Hypocenomyce stoechadiana* Abbassi Maaf & Cl. Roux

L – Subs.: cor – Alt.: 1–2 – Note: a Mediterranean-Macaronesian species found on ancient specimens of *Olea* and *Quercus ilex* in warm-humid areas, also reported from the base of the Western Alps (France). – **Fr**: AMa.

***Xalocoa ocellata* (Fr.) Kraichak, Lücking & Lumbsch**

Syn.: *Diploschistes ocellatus* (Fr.) Norman, *Lecanora villarsii* Ach., *Parmelia ocellata* Fr., *Urceolaria ocellata* (Fr.) DC., *Urceolaria villarsii* (Ach.) Boistel

L – Subs.: cal, ter-cal – Alt.: 1–3 – Note: a mild-temperate to Mediterranean lichen found on limestone, dolomite and calciferous sandstone, more rarely on soil; apparently more frequent in the Southern and Western Alps. – **Sw**: BE, VD, VS. **Fr**: AHP, HAl, AMa, Drô, Isè, Var, Vau. **It**: Ven, TAA, Lomb, Piem.

***Xanthomendoza fallax* (Hepp) Søchting, Kärnefelt & S.Y. Kondr.**

Syn.: Lecanora candelaria (L.) Ach. var. substellaris Ach., *Physcia ulophylla* (Wallr.) Lamy, *Placodium fallax* Hepp, *Xanthoria fallax* (Hepp) Arnold, *Xanthoria substellaris* (Ach.) Vain., *Xanthoria ulophylla* (Wallr.) Arnold

L – Subs.: sil, cal, int – Alt.: 2–4 – Note: this mainly northern to montane species mainly grows on siliceous or calcareous rocks. Most records of *X. fallax* from the Alps are from bark, and refer to the mainly epiphytic *X. huculica* (see note on that species). There is an open nomenclatural problem with this species, as the purported basionym, *Physcia fallax* Hepp *ex* Arnold, is a later homonym of *Physcia fallax* (Weber) DC. (1805). – **Au**: ?Au. **It**: Frl, Lomb.

***Xanthomendoza fulva* (Hoffm.) Søchting, Kärnefelt & S.Y. Kondr.**

Syn.: *Gallowayella fulva* (Hoffm.) S.Y. Kondr., N.M. Fedorenko, S. Stenroos, Kärnefelt, Elix, Hur & A. Thell, *Lobaria fulva* Hoffm., *Oxneria fulva* (Hoffm.) S.Y. Kondr. & Kärnefelt, Xanthoria candelaria (L.) Th. Fr. f. fulva (Hoffm.) Zahlbr., *Xanthoria fulva* (Hoffm.) Poelt & Petut., *Xanthoria ligustica* M. Steiner *ex* Poelt nom. sol.

L – Subs.: cor – Alt.: 2–4 – Note: most frequent on isolated deciduous trees along roads in continental valleys of the Alps; often confused with *X. huculica* in the past, and with a similar ecology, but perhaps more xerophytic. – **Au**: T, S, K, St, O, N. **Ge**: OB, Schw. **Sw**: BE, GR, LU, SG, SZ, UR, UW, VS. **Fr**: AHP, HAl, Isè, HSav. **It**: Frl, Ven, TAA, Lomb, Piem, Lig.

***Xanthomendoza huculica* (S.Y. Kondr.) Diederich**

Syn.: *Oxneria huculica* S.Y. Kondr., Xanthoria fallax *auct. non* (Hepp) Arnold

L – Subs.: cor – Alt.: 2–4 – Note: a recently-described epiphytic species differing from *X. fallax* in the much larger lobes with numerous rhizines, the narrower spores with shorter septum, and in the ecology (epiphytic versus saxicolous). Most records of epiphytic *X. fallax* belong here. – **Au**: V, T, S, K, St, O, N, B. **Ge**: OB, Schw. **Sw**: BE, FR, GL, GR, LU, SG, SZ, TI, UR, UW, VD, VS. **Fr**: AHP, HAl, AMa, Isè, Vau. **It**: Frl, Ven, TAA, Lomb, Piem, VA, Lig. **Sl**: SlA, Tg. **Li**.

***Xanthomendoza oregana* (Gyeln.) Søchting, Kärnefelt & S.Y. Kondr.**

Syn.: *Xanthoria oregana* Gyeln., *Xanthoria poeltii* S.Y. Kondr. & Kärnefelt

L – Subs.: cor – Alt.: 2–3 – Note: a species resembling *X. fulva*, forming flattened rosettes with lobes (to 1 mm wide) provided with rhizines, finally ascending and richly branched, the crenulate margins giving raise to blastidia and soralia on the lower side, apothecia rare but quite often with pycnidia containing conidia of variable shapes, often misidentified for other xanthorioid species; based on a type from Western North America where the species is fairly common, also widespread in Europe (most records under the synonym *X. poeltii*), with a few records from the Western Alps (France). – **Fr**: HAl, AHP.

***Xanthomendoza ulophyllodes* (Räsänen) Søchting, Kärnefelt & S.Y. Kondr.**

Syn.: *Oxneria ulophyllodes* (Räsänen) S.Y. Kondr. & Kärnefelt, Physcia lychnea (Ach.) Nyl. f. stenophylla Harm., *Xanthoria stenophylla* (Harm.) B. de Lesd., Xanthoria substellaris (Ach.) Vain. var. isidiigera Räsänen, *Xanthoria ulophyllodes* Räsänen

L – Subs.: cor – Alt.: 1–3 – Note: on isolated trees, often near the base of the trunks along roads, formerly often confused with *X. huculica*. – **Au**: V, T, S, K, St, O, N. **Ge**: OB, Schw. **Sw**: GL, GR, TI, VS. **Fr**: AHP, HAl, AMa. **It**: Frl, Ven, TAA, Lomb, Piem, Lig.

***Xanthoparmelia angustiphylla* (Gyeln.) Hale**

Syn.: *Parmelia angustiphylla* (Gyeln.) Gyeln., *Parmelia conspersa* (Ehrh. *ex* Ach.) Ach. var. angustiphylla Gyeln.

L # – Subs.: sil – Alt.: 1–3 – Note: a southern species in Europe, found on siliceous boulders perhaps better treated as a subspecies of *X. conspersa* (see Roux et al. 2014: 1279). – **Fr**: AMa, Var, Vau. **It**: VA, Lig.

***Xanthoparmelia conspersa* (Ehrh. *ex* Ach.) Hale**

Syn.: *Imbricaria conspersa* (Ehrh. *ex* Ach.) DC., *Lichen conspersus* Ehrh. *ex* Ach., *Parmelia conspersa* (Ehrh. *ex* Ach.) Ach., *Parmelia subconspersa* Nyl.

L – Subs.: sil, ter-sil – Alt.: 1–5 – Note: on siliceous rocks wetted by rain, including pebbles near the ground, with a wide altitudinal range; widespread throughout the Alps. – **Au**: V, T, S, K, St, O, N, B. **Ge**: OB, Schw. **Sw**: BE, FR, GR, LU, SZ, TI, UR, VD, VS. **Fr**: AHP, HAl, AMa, Isè, Sav, HSav, Var, Vau. **It**: Frl, Ven, TAA, Lomb, Piem, VA, Lig. **Sl**: SlA.

***Xanthoparmelia cumberlandia* (Gyeln.) Hale**

Syn.: Parmelia subconspersa Nyl. var. cumberlandia Gyeln.

L – Subs.: sil – Alt.: 1 – Note: on siliceous rocks wetted by rain; perhaps more widespread in the dry valleys of the Alps. – **It**: VA, Lig.

***Xanthoparmelia felkaensis* (Gyeln.) Hale**

Syn.: *Parmelia conspersa* (Ehrh. *ex* Ach.) Ach. var. alpigena Suza, *Parmelia conspersa* (Ehrh. *ex* Ach.) Ach. var. felkaensis Gyeln.

L # – Subs.: sil – Alt.: 5 – Note: a non-isidiate silicicolous species of the *X. stenophylla*-group, forming pulvinate thalli with short imbricate lobes, a brown to black lower surface, and crenate black margins becoming lobulated with age; widespread in Europe, but not always distinguished, with records also from the Eastern Alps (Austria). – **Au**: St.

***Xanthoparmelia loxodes* (Nyl.) O. Blanco, A. Crespo, Elix, D. Hawksw. & Lumbsch**

Syn.: *Neofuscelia loxodes* (Nyl.) Essl., *Parmelia glabrizans* Flagey, *Parmelia isidiotyla* Nyl., *Parmelia loxodes* Nyl.

L – Subs.: sil, cor – Alt.: 1–3 – Note: a mainly temperate, common species of basic siliceous rocks, occurring also in urban areas, with a slightly suboceanic distribution in Europe. – **Au**: T, S, St. **Sw**: BE, FR, GR, UR, VS. **Fr**: AMa, Isè, Sav, Var, Vau. **It**: Frl, TAA, Lomb, Lig.

***Xanthoparmelia mougeotii* (Schaer. *ex* D. Dietr.) Hale**

Syn.: *Parmelia mougeotii* Schaer. *ex* D. Dietr.

L – Subs.: sil – Alt.: 2–4 – Note: a mainly boreal-montane, circumpolar species found on hard siliceous rocks, often near the ground, *e.g.* on pebbles; probably more widespread in the Alps, but never common. – **Fr**: AMa, HSav, Var. **It**: Lomb, VA.

***Xanthoparmelia pokornyi* (Körb.) O. Blanco, A. Crespo, Elix, D. Hawksw. & Lumbsch**

Syn.: *Neofuscelia pokornyi* (Körb.) Essl., *Parmelia pokornyi* (Körb.) Szatala, Parmelia pulla Ach. var. pokornyi (Körb.) Türk & Breuss

L – Subs.: sil-ter – Alt.: 2 – Note: a mainly terricolous species of steppe-like habitats, closely related to *X. pulla*, with a few scattered records from the Alps. – **Au**: T. **Fr**: AHP, Vau.

***Xanthoparmelia protomatrae* (Gyeln.) Hale**

Syn.: *Parmelia protomatrae* Gyeln., Xanthoparmelia somloensis (Gyeln.) Hale var. protomatrae (Gyeln.) R. Sant.

L – Subs.: sil – Alt.: 1–5 – Note: on weathered siliceous rocks, mostly near the ground; apparently more frequent in the Southern and Western Alps. – **Au**: T, St, N. **Fr**: AHP, HAl, AMa, Var, Vau. **It**: Ven, TAA, Lomb, Piem, VA, Lig.

***Xanthoparmelia pulla* (Ach.) O. Blanco, A. Crespo, Elix, D. Hawksw. & Lumbsch subsp. pulla var. pulla**

Syn.: *Neofuscelia pulla* (Ach.) Essl., Neofuscelia pulla (Ach.) Essl. var. locarnensis (Zopf *ex* Rosend.) Hafellner, *Parmelia locarnensis* Zopf *ex* Rosend., *Parmelia prolixa* (Ach.) Röhl., *Parmelia pulla* Ach., Parmelia pulla Ach. var. locarnensis (Zopf *ex* Rosend.) Clauzade & Cl. Roux

L – Subs.: sil – Alt.: 1–5 – Note: a mainly temperate to Mediterranean species found on exposed siliceous rocks, including pebbles, exceptionally also on limestone. Some records could refer to other, morphologically similar but chemically different taxa. – **Au**: V, T, S, K, St, N, B. **Ge**: Schw. **Sw**: BE, GR, TI, VD, VS. **Fr**: AHP, HAl, AMa, Isè, Sav, HSav, Var, Vau. **It**: Frl, Ven, TAA, Lomb, Piem, VA, Lig. **Sl**: SlA.

***Xanthoparmelia pulla* (Ach.) O. Blanco, A. Crespo, Elix, D. Hawksw. & Lumbsch subsp. pulla var. delisei (Duby) ined. (provisionally placed here, ICN Art. 36.1b)**

Syn.: *Neofuscelia delisei* (Duby) Essl., *Parmelia delisei* (Duby) Nyl., Parmelia olivacea var. delisei Duby, Parmelia pulla Ach. var. delisei (Duby) H. Magn., *Xanthoparmelia delisei* (Duby) O. Blanco, A. Crespo, Elix, D. Hawksw. & Lumbsch

L – Subs.: sil – Alt.: 1–3 – Note: on base-rich siliceous rocks. Perhaps this may be the primary, sexually reproducing species of *X. loxodes*, chemically different from the typical variety, and probably often confused with it. – **Au**: T, St. **Sw**: TI. **Fr**: Isè, Var, Vau. **It**: TAA, Lomb, Piem VA, Lig.

***Xanthoparmelia pulla* (Ach.) O. Blanco, A. Crespo, Elix, D. Hawksw. & Lumbsch subsp. pulla var. glabrans (Nyl.) ined. (provisionally placed here, ICN Art. 36.1b)**

Syn.: *Neofuscelia glabrans* (Nyl.) Essl., *Parmelia glabrans* Nyl., Parmelia pulla Ach. subsp. glabrans (Nyl.) Clauzade & Cl. Roux, *Xanthoparmelia glabrans* (Nyl.) O. Blanco, A. Crespo, Elix, D. Hawksw. & Lumbsch

L – Subs.: sil – Alt.: 1 – Note: on siliceous rocks. Differing from the typical variety in the presence of collatolic and alectoronic acids. – **Fr**: Vau. **It**: TAA, VA.

***Xanthoparmelia pulla* (Ach.) O. Blanco, A. Crespo, Elix, D. Hawksw. & Lumbsch subsp. pulla var. perrugata (Nyl.) ined. (provisionally placed here, ICN Art. 36.1b)**

Syn.: *Neofuscelia perrugata* (Nyl.) Elix, *Parmelia perrugata* Nyl., *Xanthoparmelia perrugata* (Nyl.) O. Blanco, A. Crespo, Elix, D. Hawksw. & Lumbsch

L – Subs.: sil, cal – Alt.: 2 – Note: a boreal to (mainly) Mediterranean lichen found on exposed siliceous rocks, including pebbles, exceptionally also on limestone; in the past it has been frequently confused with other taxa of the *X. pulla*-group. – **Au**: ?B. **It**: TAA, Lomb, Piem, VA, Lig.

***Xanthoparmelia pulla* (Ach.) O. Blanco, A. Crespo, Elix, D. Hawksw. & Lumbsch subsp. luteonotata (J. Steiner) ined. (provisionally placed here, ICN Art. 36.1b)**

Syn.: *Neofuscelia luteonotata* (J. Steiner) Essl., *Parmelia luteonotata* J. Steiner, *Xanthoparmelia luteonotata* (J. Steiner) O. Blanco, A. Crespo, Elix, D. Hawksw. & Lumbsch

L – Subs.: sil – Alt.: 1–2 – Note: a mainly Mediterranean lichen of sun-exposed siliceous rocks wetted by rain, occurring also in dry-warm sites of the Alps. – **It**: TAA, Lig.

***Xanthoparmelia stenophylla* (Ach.) Ahti & D. Hawksw.**

Syn.: *Parmelia conspersa* (Ehrh. *ex* Ach.) Ach. var. stenophylla Ach., Parmelia molliuscula *auct. non* Ach., *Parmelia somloensis* Gyeln., *Parmelia stenophylla* (Ach.) Heugel, *Parmelia subdiffluens* (Hale) Cogt, Parmelia taractica *auct. non* Kremp., *Xanthoparmelia somloensis* (Gyeln.) Hale, *Xanthoparmelia subdiffluens* Hale, Xanthoparmelia taractica *auct. non* (Kremp.) Hale

L – Subs.: sil, ter – Alt.: 1–6 – Note: on weathered siliceous rocks and mineral soil in open, dry situations, with a very wide altitudinal range; widespread throughout the Alps. – **Au**: V, T, S, K, St, N, B. **Sw**: BE, GR, LU, SZ, TI, UR, VS. **Fr**: AHP, HAl, AMa, Drô, Isè, Sav, HSav, Var, Vau. **It**: Frl, Ven, TAA, Lomb, Piem, VA, Lig. **Sl**: SlA.

***Xanthoparmelia sublaevis* (Cout.) Hale**

Syn.: *Parmelia conspersa* (Ehrh. *ex* Ach.) Ach. var. hypoclista Nyl., *Parmelia hypoclista* (Nyl.) Klem., *Parmelia sublaevis* Cout.

L – Subs.: sil – Alt.: 1–2 – Note: on siliceous rocks wetted by rain; hitherto known from the Mediterranean region and the dry-warm valleys of the Alps. – **Fr**: AMa. **It**: TAA, VA.

***Xanthoparmelia tinctina* (Maheu & A. Gillet) Hale**

Syn.: *Parmelia conspersa* (Ehrh. *ex* Ach.) Ach. subsp. tinctina (Maheu & A. Gillet) Clauzade & Cl. Roux, *Parmelia conspersa* (Ehrh. *ex* Ach.) Ach. var. isidiosa Nyl., *Parmelia tinctina* Maheu & A. Gillet, *Parmelia tokajensis* Gyeln.

L – Subs.: sil – Alt.: 1–2 – Note: a mainly southern species found on siliceous rocks (including serpentine) in sunny situations, common in the Mediterranean belt, but also occurring in dry-warm Alpine valleys. – **Sw**: VS. **Fr**: AMa, HSav, Var, Vau. **It**: TAA, Lomb, Piem, VA, Lig.

***Xanthoparmelia verrucigera* (Nyl.) Hale**

Syn.: *Parmelia conspersa* (Ehrh. *ex* Ach.) Ach. var. isidiophora Trevis., *Parmelia conspersa* (Ehrh. *ex* Ach.) Ach. var. verrucigera (Nyl.) Boistel, Parmelia isidiigera (Müll. Arg.) Vain. f. ligustica Gyeln., *Parmelia lusitana* Nyl., *Parmelia pseudoservitiana* Gyeln., *Parmelia servitiana* Gyeln., *Parmelia tarpatakensis* Gyeln., *Parmelia verrucigera* Nyl., *Xanthoparmelia lusitana* (Nyl.) Krog

L – Subs.: sil – Alt.: 1–2 – Note: on siliceous rocks; related to *X. conspersa*, but chemically different (stictic acid, *lusitana*-unknown, lacking norstictic acid); in the study area so far reported from the Southern Alps only (Italy). – **It**: Frl, Ven, VA, Lig.

***Xanthoparmelia verruculifera* (Nyl.) O. Blanco, A. Crespo, Elix, D. Hawksw. & Lumbsch**

Syn.: *Neofuscelia verruculifera* (Nyl.) Essl., *Parmelia glomellifera* (Nyl.) Nyl., Parmelia loxodes Nyl. var. verruculifera (Nyl.) Clauzade & Cl. Roux, *Parmelia massalongoana* Gyeln., Parmelia olivacea (L.) Ach. var. leucocheila A. Massal., Parmelia prolixa (Ach.) Carroll var. glomellifera Nyl., *Parmelia verruculifera* Nyl.

L – Subs.: sil – Alt.: 1–4 – Note: on base-rich, sometimes slightly calciferous siliceous rocks, mostly on horizontal faces, this species is characterised by the presence of divaricatic, rarely of stenosporic acids. Related to *X. loxodes*, but with a less suboceanic distribution in Europe – **Au**: V, T, S, K, St, N, B. **Sw**: GR, TI, UR, VS. **Fr**: HAl, AMa, Sav, HSav, Var, Vau. **It**: Ven, TAA, Lomb, Piem, VA, Lig.

***Xanthoria calcicola* Oxner**

Syn.: Xanthoria aureola *auct. non* (Ach.) Erichsen, *Xanthoria ectaneoides* (Nyl.) Zahlbr. *non* *sensu* Lindblom & Ekman, *Xanthoria parietina* (L.) Th. Fr. var. aureola *auct. non* (Ach.) Th. Fr., Xanthoria parietina (L.) Th. Fr. subsp. calcicola (Oxner) Clauzade & Cl. Roux

L – Subs.: cal, sil – Alt.: 1–4 – Note: a mainly Mediterranean to mild-temperate lichen found on the top of isolated calcareous and basic siliceous boulders; in strongly eutrophicated situations it can occasionally overgrow bryophytes and plant remains. – **Au**: St. T. **Sw**: LU. **Fr**: AHP, AMa, Drô, Isè, HSav, Var, Vau. **It**: Frl, Ven, TAA, Lomb, Piem, VA, Lig.

**Xanthoria elegans (Link) Th. Fr. subsp. elegans**

Syn.: *Amphiloma elegans* (Link) Körb., *Caloplaca dissidens* (Nyl.) Mérat, *Caloplaca elegans* (Link) Th. Fr., Caloplaca elegans (Link) Th. Fr. var. tenuis (Wahlenb.) Th. Fr., *Caloplaca tegularis* (Ehrh.) Sandst. *non auct.*, *Lichen elegans* Link, *Placodium dissidens* Nyl., *Placodium elegans* (Link) DC., *Rusavskia elegans* (Link) S.Y. Kondr. & Kärnefelt

L – Subs.: cal, sil, int, bry, xyl, deb – Alt.: 2–6 – Note: a northern holarctic species found both on natural rock outcrops and on man-made substrata (especially tiles), mostly in upland areas, descending to lower elevations in continental sites; in strongly eutrophicated situations it can occasionally overgrow bryophytes and plant remains; widespread and locally common throughout the Alps. – **Au**: V, T, S, K, St, O, N, B. **Ge**: OB, Schw. **Sw**: BE, FR, GL, GR, LU, SZ, TI, UR, UW, VD, VS. **Fr**: AHP, HAl, AMa, Isè, Sav, HSav, Var, Vau. **It**: Frl, Ven, TAA, Lomb, Piem, VA, Lig. **Sl**: SlA. **Li**.

**Xanthoria elegans (Link) Th. Fr. subsp. orbicularis (Schaer.) Clauzade & Cl. Roux**

Syn.: Parmelia elegans (Link) Ach. var. orbicularis Schaer. *nom.illeg.*!, Xanthoria elegans (Link) Th. Fr. var. ectaniza sensu Clauzade & Rondon *non* (Nyl.) Clauzade & Rondon

L # – Subs.: cal – Alt.: 3–5 – Note: a name applied to morphs with flat, relatively wide (2–3 mm) but short lobes, not consistently distinguished; on rocks at high altitudes; distribution incompletely documented but evidently rather common and widespread in the Alps; the purported basionym is illegitimate. – **Fr**: AHP, HAl, AMa, Sav, HSav. **It**: Frl, Ven, TAA, Piem.

**Xanthoria elegans (Link) Th. Fr. var. granulifera Giralt, Nimis & Poelt**

Syn.: *Rusavskia granulifera* (Giralt, Nimis & Poelt) S.Y. Kondr. & Kärnefelt

L # – Subs.: cal – Alt.: 4–5 – Note: a northern taxon growing together with subspecies elegans, hitherto only known from the Eastern Alps. – **Au**: St, N.

***Xanthoria hafellneri* S.Y. Kondr. & Kärnefelt**

L # – Subs.: cal – Alt.: 5–6 – Note: a rare species resembling *X. domogledensis*, with individual thalli (to 25 mm in diam.) in dense populations, marginal lobes convex and hollow, lacking rhizines, towards the centre with a wrinkled upper cortex soon breaking up in coalescing schizidioid masses; on mountain tops of calcareous rocks in exposed sites (high-alpine summits), only known from the type locality in the Eastern Alps (Italy). – **It**: TAA.

***Xanthoria nowakii* S.Y. Kondr. & Bielczyk**

L – Subs.: cal – Alt.: 4 – Note: a rare species recalling *Polycauliona candelaria* but of so far unresolved relationships, forming small thalli consisting of narrow, finally ascending, blastidiate lobes; on vertical faces of rocks containing some calcium; reported from the mountains of Europe, including the Swiss Alps, at high elevations, and from South America. – **Sw**: GR.

**Xanthoria parietina (L.) Th. Fr. subsp. parietina**

Syn.: *Lecanora rutilans* Ach., *Lichen parietinus* L., *Parmelia parietina* (L.) Ach., *Parmelia rutilans* (Ach.) Ach., *Physcia parietina* (L.) De Not., *Xanthoria ectanea* (Ach.) Räsänen, Xanthoria parietina (L.) Th. Fr. subsp. ectanea (Ach.) Clauzade & Cl. Roux

L – Subs.: cor, xyl, cal, sil – Alt.: 1–4 – Note: a common species, absent only from heavily polluted areas; mainly epiphytic, but sometimes present on calciferous or basic siliceous rocks; widespread throughout the Alps, with optimum in the submediterranean belt. – **Au**: V, T, S, K, St, O, N, B. **Ge**: OB, Schw. **Sw**: AP, BE, FR, GL, GR, LU, SG, SZ, TI, UR, UW, VD, VS. **Fr**: AHP, HAl, AMa, Drô, Isè, Sav, HSav, Var, Vau. **It**: Frl, Ven, TAA, Lomb, Piem, VA, Lig. **Sl**: SlA, Tg. **Li**.

**Xanthoria parietina (L.) Th. Fr. subsp. ectanea sensu Clauzade & Cl. Roux *non* (Ach.) Clauzade & Cl. Roux**

Syn.: *Xanthoria ectanea* *sensu* Ozenda & Clauzade *non* (Ach.) Räsänen

L # – Subs.: cal, sil – Alt.: 2 – Note: a taxon with an unresolved nomenclature, forming thalli with narrow (to 1 mm wide), convex lobes lacking erect structures, often fertile; on calcareous and basic siliceous rocks at low elevations; overall distribution unknown, in the study area so far reported only from the Western Alps (France). – **Fr**: AHP, AMa, Vau.

***Xanthoria sorediata* (Vain.) Poelt**

Syn.: *Caloplaca sorediata* (Vain.) Du Rietz, Lecanora elegans (Link) Ach. var. sorediata Vain., Physcia elegans (Link) De Not. var. compacta Arnold *ex* Nyl., *Rusavskia sorediata* (Vain.) S.Y. Kondr. & Kärnefelt, Xanthoria elegans (Link) Th. Fr. subsp. compacta (Arnold *ex* Nyl.) Clauzade & Cl. Roux, *Xanthoria scandinavica* B. de Lesd.

L – Subs.: cal, int, sil – Alt.: 3–6 – Note: a mainly arctic-alpine, circumpolar species found on steeply inclined to underhanging surfaces of exposed calcareous or dolomitic boulders, sometimes also of basic siliceous rocks; widespread throughout the Alps. – **Au**: V, T, S, K, St, O. **Ge**: OB, Schw. **Sw**: BE, GR, LU, SZ, TI, VD, VS. **Fr**: AHP, HAl, AMa, Sav. **It**: Frl, Ven, TAA, Lomb, Piem, VA, Lig. **Li**.

***Xylographa pallens* (Nyl.) Malmgren**

Syn.: *Xylographa parallela* (Ach. : Fr.) Fr. var. pallens Nyl.

L – Subs.: xyl – Alt.: 3–4 – Note: a widespread species in the Northern Hemisphere, growing on wood, especially in exposed habitats becoming dry in summer, mainly in montane to subalpine coniferous forests; with a few scattered records from the Alps. – **Au**: K, St. **Ge**: OB. **Sw**: VS. **Fr**: AMa. **It**: TAA, Lomb.

***Xylographa parallela* (Ach. : Fr.) Fr.**

Syn.: *Hysterium abietinum* Pers., *Lichen parallelus* Ach.:Fr., *Xylographa abietina* (Pers.) Zahlbr., *Xylographa incerta* A. Massal., *Xylographa laricicola* Nyl., *Xylographa minutula* Körb., *Xylographa parallela* (Ach.:Fr.) Fr. var. sessitana Bagl., *Xylographa scaphoidea* Stirt.

L – Subs.: xyl – Alt.: 2–4 – Note: a cool-temperate to boreal-montane, circumpolar species found on hard wood, on poles, fences and on flanks of decorticated boles, especially of conifers; widespread throughout the Alps. – **Au**: V, T, S, K, St, O, N. **Ge**: OB. **Sw**: BE, GR, LU, SZ, TI, UR, UW, VD, VS. **Fr**: AHP, AMa, Isè, Sav, HSav, Vau. **It**: Frl, Ven, TAA, Lomb, Piem, VA, Lig. **Sl**: SlA, Tg. **Li**.

***Xylographa soralifera* Holien & Tønsberg**

L – Subs.: xyl – Alt.: 3–5 – Note: a rather rare, recently-described species occurring on lignum in upland areas; perhaps more widespread in the Alps. – **Au**: T, S, K, St. **Sw**: GR, VD. **It**: TAA, Lomb.

***Xylographa trunciseda* (Th. Fr.) Minks *ex* Redinger**

Syn.: *Lecidea trunciseda* Th. Fr.

L – Subs.: xyl – Alt.: 3–4 – Note: on rotting wood, mostly of conifers, certainly more widespread in the Alps. – **Au**: S, K, St. **Ge**: OB. **Sw**: SZ. **Fr**: AHP, AMa, Vau. **It**: TAA.

***Xylographa vitiligo* (Ach.) J.R. Laundon**

Syn.: *Agyrium spilomaticum* Anzi, *Spiloma vitiligo* Ach., *Xylographa corruscans* Norman, *Xylographa spilomatica* (Anzi) Th. Fr.

L – Subs.: xyl – Alt.: 3–4 – Note: a mainly boreal-montane species found on decaying, decorticated but still hard stumps, mostly of conifers, especially near the base, or on fallen trunks, with optimum near treeline; widespread throughout the Alps. – **Au**: V, T, S, K, St, O, N. **Ge**: OB. **Sw**: BE, GR, LU, SZ, VS. **Fr**: AHP, AMa, Vau. **It**: Frl, Ven, TAA, Lomb, Piem, VA, Lig. **Sl**: SlA.

***Xylopsora caradocensis* (Leight. *ex* Nyl.) Bendiksby & Timdal**

Syn.: *Bilimbia caradocensis* (Leight. *ex* Nyl.) A.L. Sm., *Hypocenomyce caradocensis* (Leight. *ex* Nyl.) P. James & Gotth. Schneid., *Lecidea acutula* Nyl., *Lecidea caradocensis* Leight. *ex* Nyl., *Psora acutula* (Nyl.) Walt. Watson, *Toninia caradocensis* (Leight. *ex* Nyl.) J. Lahm

L – Subs.: cor, xyl – Alt.: 2–4 – Note: a cool-temperate to boreal-montane lichen, mostly found on conifers in the upper montane and subalpine belts. – **Au**: V, T, S, K, St, O, N. **Ge**: OB, Schw. **Sw**: GR, LU, SZ, UW, VS. **Fr**: HAl. **It**: Ven, TAA. **Sl**: SlA.

***Xylopsora friesii* (Ach.) Bendiksby & Timdal**

Syn.: *Hypocenomyce friesii* (Ach.) P. James & Gotth. Schneid., *Lecidea friesii* Ach., *Psora friesii* (Ach.) Hellb.

L – Subs.: cor, xyl – Alt.: 3–4 – Note: a mainly boreal-montane lichen found on bark of conifers (especially *Pinus*) and on charred wood; perhaps more widespread in the Alps. – **Au**: T, S, K, St, O, N. **Ge**: OB. **Sw**: VS. **Fr**: HSav. **It**: TAA, Lomb.

***Zahlbrucknerella calcarea* (Herre) Herre**

Syn.: Ephebe lanata (L.) Vain. f. tenuis H. Magn., *Lecanephebe meylanii* Frey, *Zahlbrucknera calcarea* Herre

L – Subs.: cal – Alt.: 2–5 – Note: on limestone and dolomite, more rarely on basic siliceous rocks, in sheltered seepage tracks on steeply inclined surfaces; certainly more widespread, but never common in the Alps. – **Au**: V, T, K, St. **Ge**: Schw. **Sw**: BE, GR, SZ. **Fr**: AMa, Vau. **It**: TAA.

***Zamenhofia hibernica* (P. James & Swinscow) Clauzade & Cl. Roux**

Syn.: *Porina hibernica* P. James & Swinscow

L – Subs.: cor – Alt.: 2 – Note: a Mediterranean-Atlantic lichen found on ancient trunks, *e.g.* of *Quercus ilex*, in shaded-humid situations, especially in humid forests. Some records could refer to *Z. pseudohibernica*. – **Sw**: GL, LU, SG, SZ, UW. **Fr**: AMa, Var, Vau.

***Zamenhofia pseudohibernica* (Tretiach) Cl. Roux & Tretiach**

Syn.: *Porina pseudohibernica* Tretiach

L – Subs.: epiph – Alt.: 2 – Note: a recently-described species found on the shaded bark of epiphytic bryophytes in humid forests; hitherto known from several localities in Southern Europe; some records of *Z. hibernica* from the Alps could refer to this species. – **It**: Frl.

***Zwackhia viridis* (Ach.) Poetsch & Schied.**

Syn.: *Graphis involuta* Wallr., *Opegrapha involuta* (Wallr.) Jatta, Opegrapha rubella Pers. var. viridis Ach., *Opegrapha viridis* (Ach.) Behlen & Desberger, *Sclerographa squalida* Erichsen, *Zwackhia involuta* (Wallr.) Körb.

L – Subs.: cor – Alt.: 1–3 – Note: a mild-temperate species found in woodlands on the bark of broad-leaved trees, more rarely of conifers; widespread throughout the Alps, but generally not very common. – **Au**: V, T, S, K, St, O, N, B. **Ge**: OB. **Sw**: BE, GL, GR, LU, SZ, UR, UW, VS. **Fr**: AMa. **It**: Ven, TAA, Lomb, Lig. **Sl**: SlA, Tg.

### Non – or doubtfully lichenised taxa frequently treated by lichenologists

(excluding lichenicolous fungi)

***Arthonia bueriana* (J. Lahm) Zahlbr.**

Syn.: *Coniangium bueriana* J. Lahm

F # – Subs.: cor – Alt.: 3 – Note: a rare, non-lichenised corticolous species with a brown hypothecium reacting K+ green, whereas the hymenium reacts K+ red, known from a few stations in the montane belt of the Alps and in Central Europe; at low altitudes usually on bark of *Quercus*; extinct in many parts of Europe. – **Au**: O. **It**: Lomb.

***Arthonia cytisi* A. Massal.**

Syn.: *Lecideopsis cytisi* (A. Massal.) Dalla Torre & Sarnth.

F # – Subs.: cor – Alt.: 3–4 – Note: only known from the Italian Alps, on *Laburnum*, *Fraxinus*, *Pinus cembra* and *Ribes*, this non-lichenised species is worthy of further study. – **It**: Ven, Lomb, Piem.

***Arthonia punctiformis* Ach.**

Syn.: Arthonia armoricana Nyl. f. saltelii B. de Lesd., *Arthonia atomaria* A. Massal., *Arthonia celtidis* A. Massal., *Arthonia griseoalba* Anzi, *Arthonia insinuata* Stirt., *Arthonia melantera* Ach., *Arthonia populina* A. Massal., Arthonia punctiformis Ach. var. glaucescens Ach., Arthonia punctiformis Ach. var. olivacea Ach., *Arthonia quadriseptata* (Ohlert) Lettau, *Arthonia quercuus* Hepp, *Arthonia stenospora* Müll. Arg., *Naevia atomaria* (A. Massal.) A. Massal., *Naevia celtidis* (A. Massal.) A. Massal., *Naevia populina* (A. Massal.) A. Massal., *Opegrapha microscopica* Sm.

F – Subs.: cor – Alt.: 1–4 – Note: a temperate to boreal-montane, circumpolar. doubtfully lichenised early coloniser of smooth bark, especially of twigs, rare in dry areas; widespread throughout the Alps. – **Au**: T, S, K, St, O, B. **Ge**: OB, Schw. **Sw**: BE, GR, TI, VD, VS. **Fr**: AHP, AMa, Isè, Sav, HSav, Var, Vau. **It**: Frl, Ven, TAA, Lomb, Piem. **Sl**: SlA, Tg.

***Arthonia tabidula* Anzi**

F # – Subs.: cor – Alt.: 4 – Note: only known from the type collection on *Pinus cembra*, this probably non-lichenised species with 6-spored asci and 3-septate ascospores with unequal cells measuring 9–10 × 2–3 µm, is worthy of further study. – **It**: Lomb.

***Arthopyrenia analepta* (Ach.) A. Massal.**

Syn.: *Arthopyrenia analeptella* (Nyl.) Arnold, *Arthopyrenia fallax* (Nyl.) Arnold, *Arthopyrenia lapponina* Anzi, *Didymella fallax* (Nyl.) Vain., *Leiophloea fallax* (Nyl.) Riedl, *Lichen analeptus* Ach., *Pseudosagedia fallax* (Nyl.) Oxner, *Verrucaria analeptella* Nyl., *Verrucaria fallax* (Nyl.) Nyl.

F – Subs.: cor – Alt.: 2–3 – Note: a most probably non-lichenised, mainly temperate, perhaps holarctic early coloniser of smooth bark found on twigs of deciduous trees, especially *Carpinus* and *Corylus*, but also on *Quercus* and *Sorbus*; widespread throughout the Alps. – **Au**: V, T, S, K, St, O, N, B. **Ge**: OB, Schw. **Sw**: BE, FR, LU, SZ, UW, VD, VS. **Fr**: Isè, Sav, HSav, Var, Vau. **It**: Frl, Ven, TAA, Lomb, Piem. **Sl**: SlA, Tg.

***Arthopyrenia cembricola* (Anzi) Lettau**

Syn.: *Porina cembricola* (Anzi) Lettau, *Sagedia cembricola* Anzi

F # – Subs.: cor – Alt.: 4 – Note: a most likely non-lichenised species characterised by a whitish, thin thallus, 8-spored asci, and 4-celled ascospores measuring 25–29 × 6–7 µm, whose type material, collected on *Pinus cembra*, well deserves further study. – **It**: Lomb.

***Arthopyrenia cerasi* (Schrad.) A. Massal.**

Syn.: *Arthopyrenia crombei* A.L. Sm., *Metasphaeria cerasi* (Schrad.) Vain., *Pseudosagedia cerasi* (Schrad.) Oxner, *Pyrenula cerasi* (Schrad.) Trevis., *Spermatodium cerasi* (Schrad.) Trevis., *Verrucaria cerasi* Schrad.

F – Subs.: cor – Alt.: 1–3 – Note: a temperate early coloniser of smooth bark, found especially in clearings of long-established deciduous woodlands near rivers, on young twigs of *e.g. Fraxinus* and *Corylus*; most probably non-lichenised. – **Au**: V, S, St, N. **Ge**: OB, Schw. **Sw**: TI. **Fr**: Drô, Isè, Sav, HSav, Var. **It**: Frl, Ven, TAA, Lomb, Piem.

***Arthopyrenia cinerescens* A. Massal.**

Syn.: *Arthopyreniella cinerescens* (A.Massal.) Steiner, *Spermatodium cinerescens* (A. Massal.) Trevis.

F # – Subs.: cor – Alt.: 2 – Note: an early coloniser of base-rich bark, most probably non-lichenised. The type material was growing on *Fraxinus*; this is the type species of the monotypic genus *Arthopyreniella*, based on differences in pycnidial apparatus. – **It**: Ven, TAA. **Sl**: SlA.

***Arthopyrenia cinereopruinosa* (Schaer.) A. Massal.**

Syn.: Arthopyrenia fallax (Nyl.) Arnold var. conspurcata J. Steiner, *Arthopyrenia pinicola* (Hepp) A. Massal., *Verrucaria cinereopruinosa* Schaer.

**F** – Subs.: cor – Alt.: 1–4 – Note: a temperate early coloniser of smooth bark, found especially in clearings of long-established deciduous woodlands near rivers, on young twigs of *e.g. Fraxinus* and *Corylus*; most probably non-lichenised; widespread throughout the Alps. – **Au**: V, T, S, K, St, O, N, B. **Ge**: OB, Schw. **Sw**: SZ. **Fr**: AMa, Isè, Sav, Var, Vau. **It**: Frl, Ven, TAA, Lomb, Piem, VA. **Sl**: SlA.

***Arthopyrenia grisea* (Schleich. *ex* Schaer.) Körb.**

Syn.: Arthopyrenia pluriseptata *auct. non* (Nyl.) Arnold, *Sagedia grisea* (Schleich. *ex* Schaer.) Anzi, Verrucaria epidermidis (Ach.) Ach. var. grisea Schleich. *ex* Schaer.

F – Subs.: cor – Alt.: 2–3 – Note: an early coloniser of smooth and acid bark, especially of *Betula*, doubtfully lichenised. – **Au**: V, St, N. **Ge**: OB, Schw. **Sw**: GR. **Fr**: Sav, HSav. **It**: Ven, TAA, Lomb, Piem. **Sl**: Tg.

***Arthopyrenia molinii* Beltr.**

Syn.: *Spermatodium molinii* (Beltr.) Trevis.

F # – Subs.: cor – Alt.: 2 – Note: a probably non-lichenised species with a thin, episubstratic, dark thallus delimited by a black prothallus, numerous, isolated to confluent, semi-immersed perithecia surrounded at the base by a black ring, 8-spored, clavate asci, and linear-elliptical, subclavate, 5–7-septate (constricted at the septa), hyaline ascospores which are 3–4 times as long as wide; only known from the type collection on *Fraxinus ornus* near Bassano del Grappa. – **It**: Ven.

***Arthopyrenia parolinii* Beltr.**

Syn.: *Spermatodium parolinii* (Beltr.) Trevis.

F # – Subs.: cor – Alt.: 2 – Note: a probably non-lichenised species with a thin, first hypo-, then episubstratic, spreading, grey-brown thallus, semi-immersed hemispherical-conical perithecia, clavate-ventricose, 8-spored asci, and ovoid-elongate, 5–7-septate, hyaline ascospores which are 4–5 times as long as wide; only known from the type collection on *Tilia* near Bassano del Grappa. – **It**: Ven.

***Arthopyrenia persoonii* A. Massal.**

F – Subs.: cor – Alt.: 2–3 – Note: a non-lichenised species, confused with *Naetrocymbe punctiformis* in the past, but apparently closely related to *A. grisea*; most common on *Fagus* in the montane belt. – **Au**: K, St, N, B. **Fr**: Isè. **It**: Frl, Ven.

***Arthopyrenia pluriseptata* (Nyl.) Arnold**

Syn.: Arthopyrenia persoonii A. Massal. var. juglandis A. Massal., Arthopyrenia punctiformis (Pers.) A. Massal. var. juglandis (A. Massal.) Jatta, *Mycarthopyrenia juglandis* (A. Massal.) Keissl., *Spermatodium juglandis* (A. Massal.) Trevis., *Verrucaria pluriseptata* Nyl.

F – Subs.: cor – Alt.: 2–3 – Note: doubtfully lichenised; in the study area only known from the Southern Alps (Italy). – **It**: Ven, TAA, Lomb.

***Arthopyrenia pithyophila* Th. Fr. & Blomb.**

F – Subs.: cor – Alt.: 3–4 – Note: a pioneer, non-lichenised species growing on the bark of coniferous and deciduous trees; the sample from Italy was collected by G. Thor in the Adamello Natural Park, on *Sorbus aucuparia*. – **It**: TAA.

***Arthopyrenia salicis* A. Massal.**

Syn.: *Leiophloea salicis* (A. Massal.) Trevis., *Pyrenula salicis* (A. Massal.) Trevis.

F – Subs.: cor – Alt.: 2–3 – Note: a temperate coloniser of the smooth bark of deciduous trees and shrubs, especially *Carpinus* and *Corylus*, most frequent in upland areas. The species was probably mistaken for *A. punctiformis* and related species in the past. – **Au**: K, N. **Sw**: GR, TI, UW, VS. **It**: Ven, TAA, Piem, Lig. **Sl**: SlA.

***Arthopyrenia stenospora* Körb.**

F – Subs.: cor – Alt.: 3 – Note: a doubtfully lichenised species resembling *A. analepta*, with an hypophloeodic, reddish-brown, spreading thallus, minute, depressed, hemispherical ascomata, 8-spored asci, and 1-septate, almost bacilliform ascospores (*c.* 18–22 × 2.5–4 µm); on smooth bark of deciduous trees, widespread throughout Europe but rarely collected, most records being historical. – **Au**: T, O, N. **It**: TAA.

***Arthopyrenia subcerasi* (Vain.) Zahlbr.**

Syn.: *Metasphaeria subcerasi* (Vain.) Vain., *Verrucaria subcerasi* Vain.

F – Subs.: cor – Alt.: 3 – Note: a mainly boreal-montane, non-lichenised species occurring on the bark of *Betula*, known from Scandinavia, the United Kingdom, Galicia, and the Alps. – **It**: Piem.

***Arthopyrenia subconfluens* (Müll. Arg.) Zahlbr.**

Syn.: *Sagedia subconfluens* Müll. Arg.

F # – Subs.: cor – Alt.: 4 – Note: a very poorly known, non-lichenised species. – **Sw**: Sw. **Fr**: HSav.

***Arthopyrenia tuscanensis* Coppins & Ravera**

F – Subs.: cor – Alt.: 2–3 – Note: a recently-described, most probably non-lichenised species, which grows as a pioneer on the smooth bark of twigs, especially of *Castanea*; hitherto known only from Tuscany and Piemonte, but in the past perhaps confused with *A. salicis*. – **It**: Piem.

***Blastodesmia nitida* A. Massal.**

Syn.: *Polyblastia nitida* (A. Massal.) Trevis., *Pyrenula circumfusa* (Nyl.) Trevis., *Verrucaria massalongii* Garov.

F – Subs.: cor – Alt.: 1–2 – Note: a typically submediterranean early coloniser of smooth bark, especially of *Fraxinus ornus*; in the study area so far only known from the Southern Alps, at low elevations. – **It**: Frl, Ven, TAA, Lomb.

***Chaenothecopsis debilis* (Turner & Borrer *ex* Sm.) Tibell**

Syn.: *Calicium debilis* Turner & Borrer *ex* Sm.

F – Subs.: xyl – Alt.: 2–4 – Note: on dry and weathered lignum of deciduous trees (*Populus*, *Fraxinus*, *Ulmus*), more rarely on conifers, in open situations, often in hollows of the trunks in species-poor stands; probably overlooked and more widespread, but never common. – **Sw**: SZ. **Fr**: AHP. **It**: Frl.

***Chaenothecopsis montana* Rikkinen**

F – Subs.: res – Alt.: 3 – Note: a species with an often reddish brown stalk and a greenish capitulum (both K-), stalk and exciple being composed of cylindrical to isodiametric hyphal cells, paraphyses with pigmented caps, and simple, broadly ellipsoid ascospores; on resin of conifers; based on a type from Western North America, widespread in the boreal zone; in the Alps so far only found on resin of *Abies* in Switzerland. – **Sw**: SZ.

***Chaenothecopsis nana* Tibell**

F – Subs.: cor, lig – Alt.: 3–4 – Note: a mainly boreal-montane species occurring on bark and lignum of coniferous trees in humid-shaded situations. – **Sw**: LU, SZ. **Fr**: AHP. **Sl**: SlA, Tg.

***Chaenothecopsis oregana* Rikkinen**

Syn.: *Chaenothecopsis zebrina* Rikkinen & Tuovila

F – Subs.: res – Alt.: 3 – Note: a species resembling *Ch. resinicola*, with a shiny black stalk and a lenticular to subspherical, black capitulum, under the microscope stalk and exciple brownish-red, composed of periclinally arranged, subparallel hyphae, the dark cell walls giving the stalk a striped appearance; hymenium with an amyloid reaction but reaction fading, ascospores simple, ellipsoid to cylindrical, smooth, reddish brown; on resin of conifers; based on a type from Western North America, widespread in the Northern Hemisphere but altogether rare; in the Alps so far only found on resin of *Abies* in Central Switzerland. – **Sw**: SZ.

***Chaenothecopsis pusilla* (Ach.) A.F.W. Schmidt**

Syn.: *Calicium alboatrum* Flörke, Calicium claviculare Ach. var. pusillum Ach., *Calicium culmigenum* de Not. & Bagl., *Calicium floerkei* Zahlbr., *Calicium italicum* *auct.*, *Calicium nigrum* Schaer., *Calicium parasitaster* (Bagl. & Carestia) Zopf, *Calicium pusillum* (Ach.) Flörke, *Calicium subpusillum* Vain., *Chaenothecopsis alboatra* (Flörke) Nádv., *Chaenothecopsis parasitaster* (Bagl. & Carestia) D. Hawksw, *Chaenothecopsis subpusilla* (Vain.) Tibell

F – Subs.: xyl, xyl-par, cor-par – Alt.: 2–4 – Note: on trunks of old conifers in ancient forests, and on lignum, sometimes on old oaks, perhaps a parasite of free-living algal colonies, or a saprophyte. – **Au**: V, T, S, K, St, O, N. **Ge**: OB, Schw. **Sw**: GL, SZ. **Fr**: AHP, HSav, Var, Vau. **It**: Frl, Ven, TAA, Lomb, Piem. **Sl**: SlA.

***Chaenothecopsis pusiola* (Ach.) Vain.**

Syn.: *Calicium lignicola* Nádv., *Calicium pusiolum* Ach., *Chaenothecopsis lignicola* (Nádv.) A.F.W. Schmidt

F – Subs.: xyl, cor, par – Alt.: 2–4 – Note: on lignum of conifers, more rarely of deciduous trees, most often associated with *Chaenotheca brunneola*, *Ch. trichialis* and *Ch. xyloxena*, and probably a parasite or parasymbiont of these species; widespread in the Alps, but not common. – **Au**: V, T, S, K, St, O, N. **Ge**: OB. **Sw**: LU, SZ. **Fr**: AHP, AMa, HSav. **It**: Ven, TAA, Piem. **Sl**: SlA.

***Chaenothecopsis savonica* (Räsänen) Tibell**

Syn.: *Mycocalicium savonicum* Räsänen

F – Subs.: xyl-par, cor-par – Alt.: 3–4 – Note: a species resembling *Ch. pusilla*: stalk and hypothecium often with a greenish tinge, the former with periclinally arranged hyphae, ascospores pale, simple, with rounded ends; lichenicolous on the thallus of *Chaenotheca*-species or on algal colonies, usually on lignum; widespread from the boreal to the temperate zone in the Holarctic region, also recorded from the Southern Hemisphere; with a few scattered records from the Alps, where it is probably more widespread, but certainly not common. – **Ge**: OB, Schw. **Sw**: SZ. **Fr**: AHP.

***Chaenothecopsis tasmanica* Tibell**

F – Subs.: cor-par – Alt.: 3 – Note: a species with rather short (less than 1 mm tall), dark brown to black, shiny stalks, and black, lenticular to hemispherical capitula, the stalk of interwoven hyphae, with a hyaline centre and a reddish – to greenish brown outer layer, ascospores medium brown, ellipsoid, distinctly 1-septate (*c.* 6–7.5 × 2.5 µm); lichenicolous on the thallus of *Chaenotheca* species, or on algal colonies; based on a type from Tasmania and more common in Australasia; in the Alps only recorded from Central Switzerland. – **Sw**: SZ.

***Chaenothecopsis vainioana* (Nádv.) Tibell**

Syn.: *Calicium vainioanum* Nádv.

F – Subs.: cor-par – Alt.: 3 – Note: a species resembling in habitus *Ch. debilis*: ascomata rather short (to 0.6 mm tall), with black stalks and broadly obovate capitula, outer part of the stalk dark reddish brown, of interwoven hyphae, and inner part pale, of periclinally arranged hyphae, epihymenium and exciple reddish brown, hypothecium dark aeruginose, ascospores with a distinct, single septum, rather large (8–10 × 2.5–3.5 µm); on lichens containing trentepohlioid photobionts, or on free *Trentepohlia* colonies, often on deciduous trees; not uncommon in temperate Fennoscandia, rarer in the rest of Europe; in the Alps only recorded from a few localities (sometimes on *Lecanactis abietina*). – **Au**: T. **Sw**: SZ.

***Chaenothecopsis viridialba* (Kremp.) A.F.W. Schmidt**

Syn.: *Calicium cinerascens* (Nyl.) Zahlbr., Calicium parietinum Ach. var. cinerascens Nyl., *Calicium viridialbum* Kremp., *Mycocalicium cinerascens* (Nyl.) Vain.

F – Subs.: cor-par, xyl-par – Alt.: 3–4 – Note: a species with long, slender, pale, pruinose-looking stalks and black, lenticular, later hemispherical capitula, the stalk with a pale central part of periclinally arranged hyphae and an aeruginose outer part with an irregular surface giving a pruinose appearance, hypothecium in lower part with a yellowish red pigment reacting K+ greenish, ascospores simple, ellipsoid, medium brown, with a distinct but minute perisporal ornamentation; on bark and lignum of conifers and deciduous trees, often found together with *Chaenotheca chrysocephala*; widespread in the temperate zone of the Northern Hemisphere, with scattered records from the Alps, where it is not common. – **Au**: T, S, St. **Ge**: OB. **Sw**: SZ. **Fr**: AMa.

***Chaenothecopsis viridireagens* (Nádv.) A.F.W. Schmidt**

Syn.: *Calicium viridireagens* Nádv.

F – Subs.: cor-par, xyl-par – Alt.: 3–4 – Note: a mainly boreal-montane, perhaps circumpolar species found on decorticated stumps, occasionally on acid bark inside montane and subalpine forests on the thalli of *Chaenotheca* – and *Calicium*-species; probably more widespread in the Alps, but never common. – **Au**: V, T, S, K, St, O, N. **Ge**: OB. **Sw**: BE, GR, SZ. **Fr**: HSav. **It**: Frl.

***Cresporhaphis macrospora* (Eitner) M.B. Aguirre**

Syn.: Leptorhaphis quercus (Beltr.) Körb. f. macrospora Eitner

F – Subs.: cor, bry – Alt.: 2 – Note: doubtfully lichenised with coccaceous green algae, thalline patches whitish, shiny to pulverulent, ascomata perithecioid, black, smooth, semi-immersed (to 0.3 mm in diam.) with a beaked central ostiolum, ascomatal wall of thin-walled, mostly elongated cells with brown walls, in section forming a *textura angularis*, hamathecium of interascal thin-walled filaments with some septations and branchings, embedded in a gelatinous matrix, and short periphyses lining the ostiolar region, with virtually unitunicate, 8-spored, clavate to subcylindrical asci (to 155 µm long), ascospores 1 – to 3-septate and somewhat falcate with attenuated ends, large (50–85 × 3–4.5 µm); on bark of deciduous trees (*e.g. Quercus*); widespread in Europe, including the Eastern Alps (Austria), but most records are historical,. – **Au**: K.

***Cresporhaphis muelleri* (Duby) M.B. Aguirre**

Syn.: *Leptorhaphis aggregatus* Eitner, *Sphaeria muelleri* Duby

F – Subs.: cor – Alt.: 2–3 – Note: doubtfully lichenised with coccaceous green algae, thalline patches greyish-whitish, shiny, immersed in the bark, ascomata perithecioid, black, smooth, semi-immersed (to 0.4 mm in diam.), with a beaked central ostiolum, often confluent, ascomatal wall with two layers, the outer layer pseudostromatic, the inner layer of thin-walled, mostly elongated cells with brown walls, forming a *textura angularis*, hamathecium of thin-walled interascal filaments with some septations and branchings, embedded in a gelatinous matrix, and short periphyses lining the ostiolar region, with virtually unitunicate 8-spored clavate to subcylindrical asci (to 85 µm long), ascospores simple and somewhat falcate (25–30 × 2–3.5 µm); on bark of deciduous trees (*e.g. Acer pseudoplatanus*); rare throughout Europe, including a few scattered localities in the Alps, only known from historical collections. – **Au**: O. **Sw**: GR. **Fr**: HSav.

***Cresporhaphis wienkampii* (J. Lahm *ex* Hazsl.) M.B. Aguirre**

Syn.: *Leptorhaphis wienkampii* J. Lahm *ex* Hazsl.

F – Subs.: cor – Alt.: 2–3 – Note: a mainly temperate species found on rough bark of *Salix*, *Robinia*, deciduous oaks, mainly along bark furrows; certainly more widespread. The species is doubtfully lichenised: photobionts were reported from British material only. – **Au**: V, T, S, N.

***Cyrtidula hippocastani* (DC.) R.C. Harris**

Syn.: *Mycoporum hippocastani* (DC.) Coppins, *Verrucaria hippocastani* DC.

F – Subs.: cor – Alt.: 2–3 – Note: on the branches of different broad-leaved trees, with optimum in the submediterranean belt. – **Au**: St. **It**: TAA.

***Cyrtidula quercus* (A. Massal.) Minks**

Syn.: *Arthopyrenia quercus* A. Massal., *Dermatina quercus* (A. Massal.) Zahlbr., *Mycoporum miserrimum* Nyl., *Mycoporum quercus* (A. Massal.) Müll. Arg.

F – Subs.: cor – Alt.: 2–3 – Note: on young twigs mainly of oaks, but also of *Alnus* and other species, mostly in the submediterranean belt. – **Au**: K, St. **It**: Ven, Lomb.

***Dacampia hookeri* (Borrer) A. Massal.**

Syn.: *Biatorina sphaerica* A. Massal., *Pleospora hookeri* (Borrer) Keissl., *Verrucaria hookeri* Borrer

F – Subs.: par, ter-cal – Alt.: 3–5 – Note: a parasite on *Solorina* species, especially *Solorina bispora*, which is occasionally found with early infection stages on humic soil over calcareous substrata, mostly on north-exposed slopes; widespread throughout the Alps. – **Au**: V, T, S, K, St, O, N. **Ge**: OB, Schw. **Sw**: SZ, UR. **Fr**: HAl, Sav, HSav. **It**: Frl, Ven, TAA, Lomb, Piem, VA. **Sl**: SlA. **Li**.

***Epigloea bactrospora* Zukal**

F – Subs.: cor, xyl – Alt.: 2–4 – Note: a species with polyspored asci (> 32) and bacilliform, 1-septate ascospores (7–10 × 1.5 µm) lacking appendages; most common over algal colonies on rotting wood in the montane and subalpine belts, certainly overlooked and more widespread in the Alps. – **Au**: S, St. **It**: TAA, Lomb.

***Epigloea grummannii* Döbbeler**

F – Subs.: bry – Alt.: 3–4 – Note: a species with 32-spored asci and fusiform, 1-septate ascospores (10–14 × 1.5–2 µm) with a gelatinous appendage at both ends; on algal colonies developing on dying mats of *Grimmia* and *Hypnum*, certainly overlooked and more widespread in the Alps. – **It**: TAA.

***Epigloea medioincrassata* (Grummann) Döbbeler**

Syn.: *Voralbergia medioincrassata* Grummann

F – Subs.: bry – Alt.: 3–5 – Note: a species with 8-spored asci and narrowly ellipsoid to fusiform, 3-septate ascospores (24–33 × 3.5–5 µm) with a filiform, gelatinous appendage at both ends; over algal colonies on moribund bryophytes and, more rarely, on lignum, with optimum near treeline, certainly overlooked and more widespread in the Alps. – **Au**: V, St. **It**: TAA.

***Epigloea renitens* (Grummann) Döbbeler**

Syn.: *Vorarlbergia renitens* Grummann

F – Subs.: bry – Alt.: 2–5 – Note: a species with 8-spored asci and 1-septate ascospores tapering at one end (11.5–16 × 4.5–5.5 µm), lacking appendages; parasitic on algal colonies; in the study area so far only reported from the Eastern Alps (Austria). – **Au**: V, S.

***Epigloea soleiformis* Döbbeler**

F – Subs.: cor, xyl, bry – Alt.: 3–5 – Note: a species resembling *E. renitens*, with 8-spored asci and ellipsoid to oblong, 1-septate ascospores (9.5–12.5 × 3.5–4.5 µm) lacking appendages; over algal colonies developing on moribund bryophytes, squamules of *Cladonia*, decaying wood and humus, sometimes lichenicolous on *Placynthiella*, with optimum near treeline; certainly overlooked and more widespread in the Alps. – **Au**: S, St. **It**: TAA.

***Hazslinszkya gibberulosa* (Ach.) Körb.**

Syn.: *Arthonia gibberulosa* Ach., *Melaspilea deformis* (Nyl.) Nyl., *Melaspilea gibberulosa* (Ach.) Zwackh, *Melaspilea megalyna* (Ach.) Arnold

F – Subs.: cor – Alt.: 2–3 – Note: today regarded as non-lichenised, although an early monographer described a thin episubstratic, smooth to rimulose, whitish to greyish thallus with a trentepohlioid photobiont; ascomata adpressed, sessile, roundish to oval, disc and the somewhat prominent margin black, exciple carbonised, basally lacking, epihymenium red-brown, hypothecium brownish, most interascal filaments unbranched, ascospores 1-septate, with strongly unequal cells, for a long time unpigmented, finally brownish, pycnoconidia bacilliform; on bark of both coniferous and broad-leaved trees; widespread in Europe including the Alps, but undercollected in recent times. – **Au**: S, K, St, O, N. **Ge**: OB, Schw. **Sw**: SZ. **Fr**: AMa, Drô, HSav. **It**: Lomb.

***Julella acuminans* (Nyl.) ined. (provisionally placed here, ICN Art. 36.1b)**

Syn.: *Mycoglaena acuminans* (Nyl.) Vain., *Polyblastia acuminans* (Nyl.) Hulting, *Polyblastiopsis acuminans* (Nyl.) Lettau, *Verrucaria acuminans* Nyl.

F # – Subs.: cor – Alt.: 2 – Note: a probably non-lichenised, rarely recorded species resembling *J. subcoerulescens*, but the hyaline muriform ascospores (30–36 × 9–12 µm) with an acuminate lower end; the type is on bark of *Pinus* from extra-Alpine Central Europe; the only record from the Alps (Austria) needs confirmation. – **Au**: K.

***Julella fallaciosa* (Arnold) R.C. Harris**

Syn.: *Polyblastia fallaciosa* Arnold, *Polyblastiopsis betularia* (Nyl.) Zahlbr., *Polyblastiopsis fallaciosa* (Arnold) Zahlbr., *Polyblastiopsis fallacissima* (Stizenb. *ex* Nyl.) Zahlbr., *Sporodictyon fallaciosum* Stizenb. *ex* Arnold *nom.illeg.*

F – Subs.: cor – Alt.: 2–3 – Note: a probably non-lichenised species with black, hemispherical to somewhat elongated perithecioid ascomata, the wall in upper half clypeus-like, bluish-black to violet-brown, fissitunicate, 8-spored asci, distinct interascal filaments, and hyaline irregularly submuriform to muriform ascospores (17–23 × 7–9 µm) with 4–6 transversal septa and 1–2 longitudinal septa; although the ascomata on the white *Betula* bark are quite conspicuous, records are still few, but the species was probably overlooked. – **Au**: S, St, O, N. **It**: Ven.

***Julella lactea* (A. Massal.) M.E. Barr**

Syn.: *Blastodesmia lactea* A. Massal., Julella lactea (A. Massal.) M.E. Barr var. naegelii (Hepp) M.E. Barr, *Polyblastia naegelii* (Hepp) Trevis., *Polyblastiopsis lactea* (A. Massal.) Zahlbr., *Polyblastiopsis naegelii* (Hepp) Zahlbr., *Sporodyction lacteum* (A. Massal.) Trevis., *Verrucaria lactea* (A. Massal.) Garov., *Verrucaria naegelii* (Hepp) Nyl.

F – Subs.: cor – Alt.: 1–2 – Note: a doubtfully lichenised species with an endophloeodic thallus indicated by white to grey patches, black perithecioid ascomata, the blackish-brown wall clypeus-like, 4-spored asci, anastomosing interascal filaments, and hyaline, muriform ascospores (30–36 × 12–15 µm) with 6–8 transversal septa and 2–3 longitudinal septa; on smooth bark, mostly of *Fraxinus*, most common in Southern Europe, on the foothills and in the valleys of the Southern Alps. – **Au**: St. **Fr**: AMa, Vau. **It**: Frl, Ven, TAA, Piem.

***Julella sericea* (A. Massal.) Coppins**

Syn.: *Microglaena sericea* (A. Massal.) Lönnr., *Polyblastia sericea* A. Massal., *Polyblastiopsis sericea* (A. Massal.) Zahlbr., *Sporodyction sericeum* (A. Massal.) Trevis., *Verrucaria sericea* (A. Massal.) Garov.

F – Subs.: cor – Alt.: 2 – a doubtfully lichenised species similar and probably related to *J. fallaciosa* with equally 8-spored asci, but the hyaline ascospores broader (19–22–25 × 11–14 µm), muriform, with 5–6 transversal septa and 2–3 longitudinal septa, resulting in a higher number of cells in optical section view; on bark of deciduous trees (*Quercus*). – **Au**: St. **It**: Frl, Ven.

***Julella subcoerulescens* (Nyl.) Hafellner & Türk**

Syn.: *Mycoglaena subcoerulescens* (Nyl.) Höhn., *Polyblastia subcoerulescens* (Nyl.) Zahlbr., *Polyblastiopsis subcoerulescens* (Nyl.) Zahlbr., *Verrucaria subcoerulescens* Nyl., *Winteria subcoerulescens* (Nyl.) Rehm

F – Subs.: cor – Alt.: 3 – Note: a probably non-lichenised species with an endophloeodic thallus indicated by whitish patches, black, hemispherical perithecioid ascomata, their wall in upper half clypeus-like with a bluish-greenish pigment, 8-spored asci, persistent and anastomosing interascal filaments, and hyaline, muriform, ellipsoid ascospores (15–22 × 8–13 µm) with 3–5 transversal septa and usually one incomplete longitudinal septum; the generic placement is uncertain, perhaps better placed in *Mycoglaena*; on bark of coniferous trees (*e.g. Pinus*, *Larix*), mostly on twigs; rare throughout Europe, including the Eastern Alps (Austria), but probably often overlooked. – **Au**: N.

***Leptorhaphis atomaria* (Ach.) Szatala**

Syn.: *Arthopyrenia atomaria* (Ach.) Müll. Arg., Arthopyrenia punctiformis (Pers.) A. Massal. var. atomaria (Ach.) Anzi, *Didymella atomaria* (Ach.) Szatala, Leptorhaphis tremulae *auct. non* Körb, *Lichen atomarius* Ach., *Microthelia adspersa* Körb., *Microthelia atomaria* (Ach.) Körb. *non auct*.

F – Subs.: cor – Alt.: 2–4 – Note: a species doubtfully associated with trentepohlioid algae, with an endosubstratic thallus forming smooth, whitish-grey patches, numerous circular ascomata (to *c.* 0.2 mm in diam.), the hymenial gel hemiamyloid (bluish in IKI at low concentrations), and 1-septate ascospores with some 3-septate ones intermingled (25–32 × 2–3.5 µm) with not markedly attenuated ends; usually on bark of *Populus*, but also recorded from *Fraxinus*; widespread in temperate Europe, but also recorded from North America. – **Au**: V, T, K, St, N. **Fr**: Isè, Sav, HSav. **It**: Ven, TAA, Lomb, Piem. **Sl**: SlA.

***Leptorhaphis epidermidis* (Ach.) Th. Fr.**

Syn.: *Arthopyrenia epidermidis* (Ach.) A. Massal., *Arthopyrenia oxyspora* (Nyl.) Mudd, *Campylacia epidermidis* (Ach.) Vain., *Campylacia oxyspora* (Nyl.) Anzi, *Leptorhaphis oxyspora* (Nyl.) Körb., *Lichen epidermidis* Ach., *Pyrenula oxyspora* (Nyl.) Hepp, *Sagedia oxyspora* (Nyl.) Tuck., *Spermatodium epidermidis* (Ach.) Trevis., *Spermatodium oxysporum* (Nyl.) Trevis., *Verrucaria epidermidis* (Ach.) Ach., *Verrucaria oxyspora* Nyl.

F – Subs.: cor – Alt.: 2–3 – Note: a probably non-lichenised species with an inconspicuous thallus, ellipsoid ascomata, and 1-septate, narrowly fusiform, somewhat curved ascospores (25–35 × 2–3.5 µm); on *Betula*, mostly on the smooth bark of thick branches; widespread in Europe, including the Alps, but still not recorded in some countries. – **Au**: V, T, S, K, St, O, N, B. **Ge**: OB. **Fr**: Isè, Sav, HSav. **It**: Ven, TAA, Lomb, Piem, Lig.

***Leptorhaphis lucida* Körb.**

Syn.: *Arthopyrenia lucida* (Körb.) Müll. Arg., Leptorhaphis tremulae Körb. var. lucida (Körb.) Keissl.

F – Subs.: cor – Alt.: 2–3 – Note: a species with an endosubstratic thallus visible as smooth whitish-grey patches, ascomata as in *L. atomaria* but larger (to *c.* 0.5 mm in diam.), and also considerably larger ascospores (45–70 × 2.5–4 µm); on trunks of *Populus*, rare in temperate Europe, including the Eastern Alps (Austria), and Eastern North America, most records being historical. – **Au**: K.

***Leptorhaphis maggiana* (A. Massal.) Körb.**

Syn.: *Campylacia maggiana* A. Massal., *Verrucaria maggiana* (A. Massal.) Stizenb.

F – Subs.: cor – Alt.: 2–3 – Note: a probably non-lichenised species with an inconspicuous thallus, numerous scattered ascomata, and 1 – septate, slender ascospores with some 3-septate intermingled (30–45 × 1.5–2.5 µm), with attenuated ends; on the smooth bark of *Carpinus*, *Corylus*, more rarely of young *Quercus* (*e.g.* on twigs); widespread in Europe but rather rare and most records historical; from the Alps there are a few scattered records only. – **Au**: T, K. **Fr**: HSav. **It**: Ven.

***Leptorhaphis parameca* (A. Massal.) Körb.**

Syn.: *Leptorhaphis xylographoides* Norman, *Sagedia parameca* A. Massal., *Spermatodium paramecum* (A. Massal.) Trevis., *Verrucaria parameca* (A. Massal.) Stizenb.

F – Subs.: cor – Alt.: 2–3 – Note: a probably non-lichenised species with an inconspicuous thallus, ellipsoid ascomata resembling those of *L. epidermidis*, but with larger 1-septate ascospores (30–45 × 3–4 µm); on bark of Prunoideae; widespread in Europe and also known from North America, with a few scattered records from the Alps. – **Fr**: AMa, HSav, Var. **It**: Ven, TAA.

***Leptorhaphis tremulae* Körb.**

Syn.: *Arthopyrenia tremulae* (Körb.) Müll. Arg., *Leptorhaphis sphenospora* (Nyl.) Arnold, *Sagedia tremulae* (Körb.) Anzi, *Verrucaria sphenospora* Nyl., *Verrucaria tremulae* (Körb.) Harm.

F – Subs.: cor – Alt.: 2–3 – Note: a species with an endosubstratic, inconspicuous thallus, ascomata of about equal size as in *L. atomaria* but asci 8 – to 16-spored, ascospores smaller (about 15–25 × 3–4.5 µm); on the smooth bark of *Populus*, especially *P. tremula*, more rarely of *Salix*; rare in temperate Europe and Eastern North America; most records are historical and in the Alps it was only reported from a few scattered localities. – **Ge**: Ge. **Fr**: HSav. **It**: Ven, TAA.

***Melaspilea bagliettoana* Zahlbr.**

F – Subs.: cor – Alt.: 3 – Note: a mild-temperate species found on smooth bark of trees and shrubs such as *Fraxinus ornus* and *Nerium*. – **Sw**: ?LU, SZ.

***Melaspilea rhododendri* (Arnold & Rehm) Almq.**

Syn.: *Arthonia rhododendri* Arnold & Rehm

F – Subs.: cor – Alt.: 3–5 – Note: on twigs of shrubs (mainly *Rhododendron hirsutum*), with optimum in the subalpine belt. – **Au**: V, T, S, K, St, O, N. **Ge**: OB. **It**: TAA, Lomb, Piem. **Sl**: SlA.

***Melaspileella proximella* (Nyl.) Ertz & Diederich**

Syn.: *Arthonia proximella* Nyl., *Arthopyrenia furfuracea* A. Massal., *Buellia mughorum* Anzi, *Caldesia proximella* (Nyl.) Trevis., *Melaspilea fugax* Müll. Arg., *Melaspilea proximella* (Nyl.) Nyl.

F – Subs.: cor – Alt.: 2–4 – Note: an inconspicuous, probably overlooked species found on the bark of deciduous and coniferous trees in upland areas. – **Au**: T, K, St, O, N. **Ge**: OB. **It**: Frl, Ven, TAA, Lomb. **Sl**: SlA.

***Microcalicium ahlneri* Tibell**

F – Subs.: xyl – Alt.: 2–4 – Note: on decorticated stumps of conifers heavily attacked by brown rot fungi, more rarely of deciduous trees, often in cavities and cracks, confined to humid upland areas; probably overlooked in the Alps, but certainly never common. – **Au**: V, T, S, K, St. **It**: Frl, TAA.

***Microcalicium arenarium* (Hampe *ex* A. Massal.) Tibell**

Syn.: *Calicium arenarium* (Hampe *ex* A. Massal.) Hampe *ex* Körb., *Coniocybopsis arenaria* (Hampe *ex* A. Massal.) Vain., *Cyphelium arenarium* Hampe *ex* A. Massal.

F – Subs.: sil, sil-par – Alt.: 3 – Note: on silicicolous lichens, soil, rootlets, decorticated stumps and algal colonies under overhangs of siliceous rocks, often together with *Psilolechia lucida*; probably more widespread in the Alps than the few records would suggest. – **Au**: Au. **It**: TAA, Piem.

***Microcalicium disseminatum* (Ach.) Vain.**

Syn.: *Calicium atomarium* (Ach.) Fr., *Calicium disseminatum* Ach., *Calicium subpedicellatum* Schaer., *Calicium viridulum* (Ach.) Fr., *Cyphelium atomarium* Ach., *Cyphelium viridulum* Ach., *Microcalicium subpedicellatum* (Schaer.) Tibell, *Strongylopsis commixta* Vain., *Strongylopsis discreta* Nádv., *Strongylopsis leucopus* Vain., *Strongylopsis stichococci* Vain.

F – Subs.: cor-par, xyl-par – Alt.: 3–4 – Note: on lignum and bark of both deciduous a coniferous trees, parasitic on calicioid lichens, especially *Chaenotheca*-species, with the conidiomata forming much earlier than the ascomata, or saprophytic on bark, lignum and algal colonies; probably more widespread in the Alps. – **Au**: S, K, St, O. **Ge**: OB. **Sw**: GR, SZ. **Fr**: AHP, AMa. **It**: Frl, Ven, TAA, Lomb.

**Mycocalicium subtile (Pers.) Szatala var. subtile**

Syn.: *Calicium pallescens* Nyl., *Calicium parietinum* Ach., *Calicium parvulum* F.Wilson, *Calicium subtile* Pers., *Mycocalicium pallescens* (Nyl.) Vain., *Mycocalicium parietinum* (Ach.) Fink, Mycocalicium subtile (Pers.) Szatala var. parietinum (Ach.) Vain.

F – Subs.: xyl, cor – Alt.: 2–4 – Note: a saprophyte on dry, hard lignum, especially of conifers, in open situations, mostly in the montane and subalpine belts. – **Au**: V, T, S, K, St, O, N, B. **Ge**: OB. **Sw**: LU, SZ. **Fr**: AHP, AMa, HSav, Var. **It**: Frl, Ven, TAA, Lomb, Piem. **Sl**: SlA, Tg.

**Mycocalicium subtile (Pers.) Szatala var. minutellum (Ach.) Szatala**

Syn.: *Calicium minutellum* Ach., *Mycocalicium minutellum* (Ach.) Nádv.

F – Subs.: xyl – Alt.: 2–4 – Note: in the Austrian, Italian and Swiss checklists this taxon is considered as a synonym of *M. subtile*. – **Fr**: AHP, Var, Vau.

***Mycoglaena myricae* (Nyl.) R.C. Harris**

Syn.: *Arthopyrenia aeruginella* (Nyl.) Arnold, *Arthopyrenia myricae* (Nyl.) Zahlbr., *Verrucaria aeruginella* (Nyl.) Nyl., *Verrucaria myricae* Nyl.

F – Subs.: cor – Alt.: 4 – Note: a probably non-lichenised species with an endosubstratic, inconspicuous thallus indicated by greyish to brownish patches, black, flattened perithecioid ascomata (to 0.5 mm in diam.), the ascomatal wall greenish-brown to greenish-black (N+ reddish to violet), persistent, branched and anastomosing interascal filaments, 8-spored fissitunicate asci lacking apical amyloid structures, and hyaline, fusiform, 3-septate ascospores with pointed ends (17–23 × 6–8 µm); usually on smooth bark of deciduous trees and shrubs (*Myrica*, *Betula*) but also on conifers; widespread in the Holarctic region but rarely collected, with a single record from the Eastern Alps (Austria). – **Au**: St.

***Mycomicrothelia confusa* D. Hawksw.**

F – Subs.: cor – Alt.: 2–3 – Note: on the smooth bark of deciduous trees in shaded-humid situations. – **Au**: St. **It**: Piem.

***Mycomicrothelia inaequalis* (Fabre) D. Hawksw.**

Syn.: *Amphisphaeria inaequalis* Fabre, *Astrosphaeriella inaequalis* (Fabre) Scheinpflug, *Kirschsteiniella inaequalis* (Fabre) Petr., *Microthelia inaequalis* (Fabre) E. Müll.

F – Subs.: cor – Alt.: 1–2 – Note: a probably non-lichenised species with singly arising ascomata (to 0.3 mm in diam.) immersed in the outer layers of the wood, with a well developed ascomatal fringe, a dimidiate olivaceous-black involucrellum consisting of host cells and intermingled hyphae, the ascomatal wall brownish, of densely interwoven hyphae, interascal filaments numerous, anastomosing and persistent, 8-spored fissitunicate asci, and 1-septate, olive-brown to red-brown ascospores (18–22 × 8.5–10.5 µm) with the upper cell larger and with a thin perispore; on wood of *Olea*; a rarely collected species in California and Southern Europe, including the base of the Western Alps (France). – **Fr**: Vau.

***Mycomicrothelia macularis* (Hampe *ex* A. Massal.) Keissl.**

Syn.: *Didymosphaeria analeptoides* (Bagl. & Carestia) Rehm, *Melanotheca macularis* (Hampe *ex* A. Massal.) Th. Fr., *Microthelia analeptoides* Bagl. & Carestia, *Microthelia macularis* Hampe *ex* A. Massal., *Tomasellia macularis* (Hampe *ex* A. Massal.) Blomb. & Forssell, *Verrucaria analeptoides* (Bagl. & Carestia) Hue

F – Subs.: cor – Alt.: 3–4 – Note: an apparently non-lichenised species with subepidermal ascomata (less than 0.2 mm in diam.) arising singly or crowded, with only the ostiolar region exposed and a well developed basal fringe, a dimidiate dark brown involucrellum consisting of host cells and intermingled hyphae, the ascomatal wall brown, of densely interwoven hyphae, paler at the base, rather sparse anastomosing and persistent interascal filaments, 8-spored fissitunicate asci, 1-septate, brown ascospores (12–15 × 5–7 µm) with the upper cell somewhat wider and with a thin, verruculose perispore, and pycnidia containing bacillary conidia; on corticated twigs of small shrubs (*Daphne*, *Ribes*), not rare in the mountains of Central Europe, including the Alps, but rarely recorded. – **Au**: St, N. **Ge**: Ge. **Sw**: GL, GR, VS. **Fr**: HAl. **It**: Piem.

***Mycomicrothelia melanospora* (Hepp) D. Hawksw.**

Syn.: *Microthelia koerberi* Trevis., Microthelia atomaria *auct. non* (Ach.) Körb., *Pyrenula melanospora* Hepp, *Verrucaria cinerella* Flot. *ex* Zwackh *nom. inval*.

F – Subs.: cor – Alt.: 2–3 – Note: an apparently non-lichenised species with subepidermal ascomata, a dark brown involucrellum, dark brown 1-septate ascospores (13–16 × 6–8 µm) with more or less equal cells and a thin, coarsely verruculose perispore; on the smooth bark of deciduous trees, especially *Mespilus germanica*; rare throughout Europe; in the Alps only recorded from Austria and Italy. – **Au**: S. **It**: Piem.

***Mycomicrothelia pachnea* (Körb.) D. Hawksw.**

Syn.: *Microthelia pachnea* Körb.

F – Subs.: cor – Alt.: 3 – Note: doubtfully lichenised with trentepohlioid algae, thalline patches whitish, from which the scattered ascomata arise, ascomata (to 0.3 mm in diam.) immersed in the periderm, with only the ostiolar region exposed, involucrellum dimidiate, dark reddish-brown, consisting of a mixture of host cells and hyphae, exciple of intricate, hyaline to brownish hyphae, interascal filaments numerous, anastomosing and persistent, asci 8-spored, fissitunicate, and dark brown, 1-septate ascospores (16–18 × 7–8 µm) with unequal cells and a thin, distinctly verruculose perispore; on bark of *Abies* (records from deciduous trees are doubtful); there are only some scattered records from the Eastern Alps, but the species might have been largely overlooked. – **Au**: O, N. **Ge**: OB.

***Mycomicrothelia wallrothii* (Hepp) D. Hawskw.**

Syn.: *Didymosphaeria wallrothii* (Hepp) Sacc. & Trotter, *Massariopsis wallrothii* (Hepp) Rehm, *Microthelia betulina* J. Lahm, *Microthelia wallrothii* (Hepp) Grummann, *Pyrenula wallrothii* Hepp

F – Subs.: cor – Alt.: 3 – Note: an apparently non-lichenised species with ascomata (to *c.* 0.25 mm in diam.) immersed in the periderm of the host tree, with a well-developed ascomatal fringe, a dimidiate, dark brown involucrellum consisting of host cells and intermingled hyphae (the wall of intricate hyphae), numerous, anastomosing and persistent interascal filaments, 8-spored, fissitunicate asci, dark brown, 1-septate ascospores (16–18 × 7–8 µm) with more or less equal cells and a thin, indistinctly verruculose perispore, and conidiomata inbetween the ascomata containing 1-distoseptate, ellipsoid conidia; on bark of *Betula*, rarely *Populus*; widespread in the Holarctic region, in the study area only recorded from the Eastern Alps (Austria). – **Au**: S.

***Mycoporellum microscopicum* (Müll. Arg.) Zahlbr.**

Syn.: *Dermatina microscopica* (Müll. Arg.) Zahlbr., *Mycoporum microscopicum* (Müll. Arg.) Nyl., *Pyrenula microscopica* Müll. Arg.

F # – Subs.: cor – Alt.: 2 – Note: doubtfully lichenised with trentepohlioid algae, with a thallus forming olivaceous-grey patches, minute ascomata (*c.* 0.1 mm in diam.), the interascal filaments lacking in mature ascomata, fissitunicate, 8-spored asci, and hyaline to pale brownish, 3-(to 5-)septate ascospores (11–13 × 4–5 µm); on the smooth bark of twigs of deciduous trees (*Populus tremula*, *Quercus*, *Juglans*); rare in Central Europe at low elevations, with a single historical record (regarded as “common” at the type locality) from the Western Alps of France (locality erroneously assigned to Switzerland). – **Fr**: HSav.

***Mycoporellum obscurum* (Pers.) A.L. Sm.**

Syn.: *Arthopyrenia obscura* (Pers.) Riedl, *Mycoporum obscurum* (Pers.) Almq., *Opegrapha obscura* Pers.

F # – Subs.: cor – Alt.: 2 – Note: a species of unclear relationship, doubtfully lichenised with trentepohlioid algae, with a thallus indicated by pale patches, ascomata supposed to be organised in laterally fusing groups, and hyaline, 5 – to 8-septate ascospores; ecology and distribution are very poorly known. – **Fr**: AMa.

***Mycoporellum sacromontanum* (Strasser) Redinger**

Syn.: *Arthonia sacromontana* Strasser

F – Subs.: sil – Alt.: 2–3 – Note: a doubtfully lichenised species whose generic placement is in need of evaluation, with perithecioid ascomata arranged in dense groups (stromatic?), narrowly cylindrical, fissitunicate asci, and 1-septate ascospores with an attenuated lower cell; on sandstone and other types of siliceous rocks; a rare species, only known from low elevations in Central and Western Europe, with a single record from the Eastern Alps (Austria). – **Au**: N.

***Mycoporum antecellens* (Nyl.) R.C. Harris**

Syn.: *Arthopyrenia analeptoides* (Nyl.) A.L. Sm., *Arthopyrenia antecellens* (Nyl.) Arnold, *Verrucaria antecellans* Nyl.

F – Subs.: cor – Alt.: 2–3 – Note: an early coloniser of smooth bark, especially on twigs of broad-leaved trees and shrubs (*e.g. Corylus* and *Fagus*), in humid deciduous woodlands. – **Au**: T, S. **Ge**: Ge. **Fr**: Isè, Var. **It**: Frl, Ven, TAA, Lomb, Piem, Lig. **Sl**: SlA.

***Naetrocymbe ariae* (Müll. Arg.) ined. (provisionally placed here, ICN Art. 36.1b)**

Syn.: *Arthopyrenia ariae* (Müll. Arg.) Zahlbr, *Sagedia ariae* Müll. Arg.

F # – Subs.: cor – Alt.: mon – Note: probably related to *N. laburni*, doubtfully lichenised, with a thin thallus indicated by grey to blackish patches, depressed to hemispherical, black ascomata (to 0.2 mm in diam.), finally dissolving interascal filaments, 8-spored asci, and hyaline, 1-septate ascospores (13–18 × 4–5 μm); on smooth bark of young branches of *Sorbus aria*; only known from the type locality in the French Pre-Alps. – **Fr**: HSav.

***Naetrocymbe fraxini* (A. Massal.) R.C. Harris**

Syn.: *Arthopyrenia fraxini* A. Massal., *Pyrenula fraxini* (A. Massal.) Trevis.

F – Subs.: cor – Alt.: 1–3 – Note: a mild-temperate species found on smooth bark of (mostly) deciduous trees. – **Au**: K, St. **Ge**: Ge. **It**: Ven, TAA, Lomb, Piem. **Sl**: SlA.

***Naetrocymbe fumago* (Wallr.) Magnus**

Syn.: *Arthopyrenia fumago* (Wallr.) Körb., ?*Naetrocymbe fuliginea* Körb., *Spermatodium fumago* (Wallr.) Trevis., *Verrucaria fumago* Wallr.

F # – Subs.: cor – Alt.: 2–3 – Note: a probably non-lichenised species, with a thallus developed as a dark spongy subiculum, minute glossy ascomata, interascal filaments which are indistinct in mature ascomata, ventricose to obpyriform, fissitunicate, 8-spored asci, and first 3-septate, then submuriform and pigmented ascospores (24–28 × 10–12 µm); on branches of various deciduous trees (*e.g. Tilia*, *Populus*, *Alnus*) throughout Europe, with a few historical records from the Eastern Alps (Italy). – **It**: TAA.

***Naetrocymbe laburni* (Leight. *ex* Arnold) ined. (provisionally placed here, ICN Art. 36.1b)**

Syn.: *Arthopyrenia laburni* Leight. *ex* Arnold

F # – Subs.: cor – Alt.: 2–3 – Note: a probably non-lichenised species resembling *N. fumago* in the thallus indicated by dark patches, but the 1-septate ascospores virtually narrower due to their length (18–22 × 3–4 µm); on smooth bark, especially of *Laburnum*. – **Au**: T, St, B. **Fr**: AHP, HSav.

***Naetrocymbe punctiformis* (Pers.) R.C. Harris**

Syn.: *Arthopyrenia analepta auct. non Lichen analeptus* Ach., *Arthopyrenia cembrina* (Anzi) Grummann *ex* D. Hawksw., *Arthopyrenia padi* Rabenh., *Arthopyrenia punctiformis* (Pers.) A. Massal., *Arthopyrenia pyrenastrella* (Nyl.) Norman, Arthopyrenia submicans *auct. non* (Nyl.) Arnold, *Didymella pyrenastrella* (Nyl.) Vain., *Leiophloea punctiformis* (Pers.) Trevis., *Pyrenula punctiformis* (Pers.) Trevis., *Verrucaria punctiformis* Pers.

F – Subs.: cor – Alt.: 1–4 – Note: a holarctic, Mediterranean to boreal-montane early coloniser of smooth bark, especially on twigs of a wide variety of trees; widespread throughout the Alps. – **Au**: T, S, K, St, O, N, B. **Ge**: OB. **Sw**: SZ. **Fr**: AHP, AMa, Drô, Isè, Sav, HSav, Var, Vau. **It**: Frl, Ven, TAA, Lomb, Piem, VA, Lig. **Sl**: SlA, Tg.

***Naetrocymbe rhododendri* (Arnold) ined. (provisionally placed here, ICN Art. 36.1b)**

Syn.: Arthopyrenia punctiformis (Pers.) A. Massal. f. rhododendri Arnold, *Arthopyrenia rhododendri* (Arnold) Dalla Torre & Sarnth., Verrucaria punctiformis Pers. f. rhododendri (Arnold) Stizenb.

F – Subs.: cor – Alt.: 3–5 – Note: on smooth bark of shrubs in subalpine heaths; to be looked for throughout the Alps – **Au**: V, T, S, K, St. **Ge**: OB. **It**: TAA, Piem.

***Naetrocymbe rhyponta* (Ach.) R.C. Harris**

Syn.: *Arthopyrenia rhyponta* (Ach.) A. Massal., *Leiophloea rhyponta* (Ach.) Trevis., *Pyrenula rhyponta* (Ach.) Trevis., *Verrucaria rhyponta* Ach.

F – Subs.: cor – Alt.: 2–4 – Note: a probably circumpolar species found on smooth bark, especially on twigs and branches of deciduous trees, most common in upland areas. – **Au**: T, S, K, St. **Ge**: Ge. **Fr**: Sav. **It**: Frl, Ven, TAA, Lomb, Piem.

***Naetrocymbe subconfluens* (Müll. Arg.) ined. (provisionally placed here, ICN Art. 36.1b)**

Syn.: *Arthopyrenia subconfluens* (Müll. Arg.) Zahlbr.

F # – Subs.: cor – Alt.: 4 – Note: probably related to *N. punctiformis*, doubtfully lichenised, with a very thin thallus indicated by greyish patches, conical to hemispherical, black ascomata (to 0.2 mm in diam.) often fusing in groups of 2–5, interascal filaments in mature perithecia, 8-spored asci, and hyaline, 1-septate ascospores (18–21 × 4–7 μm); on thin branches of conifers (*e.g. Pinus cembra*); known from a few localities in the Western Alps. – **Sw**: VS. **Fr**: HSav.

***Odontura rhaphidospora* (Rehm) Clem.**

Syn.: *Beloniella rhaphidospora* (Rehm) E. Müll. & Défago, *Belonium rhaphidosporum* (Rehm) Sacc., *Dermatella rhaphidospora* (Rehm) Sacc., *Leptorhaphis pyrenopezizoides* Rehm *nom. nud.*, *Odontotrema rhaphidosporum* (Rehm) Rehm, *Odontotremella raphidospora* (Rehm) Rehm, *Pyrenopeziza rhaphidospora* Rehm

F – Subs.: xyl, ?cor – Alt.: 4 – Note: a probably non-lichenised species with an endoxylic thallus and circular, dull black, deeply urceolate ascomata (to 0.5–0.8 mm in diam.) with a central, finally dentate pore and radially arranged furrows, a dark brown, 2-layered exciple lacking crystals, interascal filaments with capitate apical cells, functionally unitunicate, moderately polyspored asci, and filiform, fasciculate ascospores breaking up into 1-septate, fusiform part-spores (16–30 × 1.5–2.5 µm); on decorticated wood of conifers (*e.g. Pinus cembra*), from the boreal zone of Scandinavia to upper montane to subalpine forests, with a few records from the Austrian and Swiss Alps. – **Au**: T. **Sw**: TI, VS.

***Opegrapha parasitica* (A. Massal.) H. Olivier**

Syn.: *Leciographa centrifuga* (A. Massal.) Rehm, *Opegrapha centrifuga* A. Massal., *Leciographa parasitica* A. Massal.

F – Subs.: cal, cal-par – Alt.: 1–5 – Note: a parasite on *Aspicilia calcarea* and related species, certainly much more widespread in the Alps; in the past it was often confused with *O. rupestris*. – **Fr**: AMa, Drô, Sav, Var, Vau. **It**: Ven, TAA, Piem.

***Opegrapha rupestris* Pers.**

Syn.: *Opegrapha monspeliensis* Nyl., *Opegrapha persoonii* (Ach. *ex* Gray) Chevall., *Opegrapha saxatilis* DC., *Opegrapha saxicola* Ach. *non auct.*

F – Subs.: cal, cal-par – Alt.: 1–5 – Note: an ecologically wide-ranging, non-lichenised species found both in natural habitats (especially shaded niches of calcareous rocks in woodlands), and in moderately disturbed situations (such as on north-facing walls); it often grows on Verrucariaceae (especially *Bagliettoa* – and *Verrucaria*-species). – **Au**: V, T, S, K, St, O, N. **Ge**: OB. **Fr**: AHP, AMa, Drô, Sav, HSav, Var, Vau. **It**: Frl, Ven, TAA, Lomb, Piem, Lig. **Sl**: SlA.

***Peridiothelia fuliguncta* (Norman) D. Hawksw.**

Syn.: *Didymosphaeria dannenbergii* (Stein *ex* Eitner) Sacc., *Didymosphaeria fuliguncta* (Norman) Vain., *Microthelia fuliguncta* Norman, *Polycoccum dannenbergii* (Stein *ex* Eitner) Vězda, *Tichothecium dannenbergii* Stein *ex* Eitner

F – Subs.: cor – Alt.: 2–3 – Note: a probably non-lichenised species; ascomata scattered, erumpent, hemispherical with an applanate base, the wall pseudoparenchymatic of subglobose hyphal cells, dark reddish-brown at the flanks, subhyaline at the base, interascal filaments numerous, persistent, branched and with anastomoses, asci broadly subcylindrical, 8-spored fissitunicate, ascospores 1-septate, brown (17–22 × 8–10 µm) with slightly unequal cells and a faintly verruculose perispore; on the bark of *Tilia* (records from other trees need critical re-evaluation), sometimes the ascomata break through the thalli of epiphytic *Lecanora* *s.lat.*, *Ochrolechia*, and *Pertusaria* *s.lat.* and then virtually lichenicolous; widespread in Europe, the distribution gaps in the Alps are probably due to undercollecting. – **Au**: V, T, S, K, St, O, N. **Ge**: OB. **Sw**: SZ. **Fr**: AHP, AMa, Isè, Var, Vau.

***Peridiothelia grandiuscula* (Anzi) D. Hawksw.**

Syn.: *Didymosphaeria grandiuscula* (Anzi) Sacc., *Microthelia grandiuscula* Anzi, Microthelia micula Körb. var. megaspora (Nyl.) B. de Lesd.

F – Subs.: cor – Alt.: 3 – Note: a probably non-lichenised species resembling *P. fuliguncta*, but ascospores distinctly larger (25–33 × 10–12 µm); less restricting in phorophyte selection than *P. fuliguncta*; on bark of various deciduous trees, rarely ascomata breaking through the thalli of epiphytic *Lecanora* *s.lat.* and *Pertusaria* *s.lat.* and then virtually lichenicolous; widespread in Eurasia, with a few scattered records from the Alps. – **Au**: T. **Fr**: HSav, Var, Vau. **It**: Lomb, Piem.

***Phaeocalicium compressulum* (Nyl. *ex* Vain.) A.F.W. Schmidt**

Syn.: Mycocalicium praecedens (Nyl.) Szatala var. compressulum Nyl. *ex* Vain.

F – Subs.: cor – Alt.: 3–4 – Note: saprobic on *Alnus viridis*; widespread throughout the Alps and locally very common, with optimum in the subalpine belt. – **Au**: V, T, S, K, St, O, N. **Ge**: OB. **Sw**: SZ. **It**: Frl, Ven, TAA, Lomb, Piem, VA, Lig. **Sl**: SlA. **Li**.

***Phaeocalicium mildeanum* (Hepp) Puntillo**

Syn.: *Calicium mildeanum* Hepp, *Calicium ornicola* J. Steiner, *Mycocalicium mildeanum* (Hepp) Nádv., *Mycocalicium ornicola* (J. Steiner) Nádv., *Phaeocalicium ornicolum* (J. Steiner) Titov, *Stenocybe mildeana* (Hepp) Jatta

F # – Subs.: cor – Alt.: 2 – Note: a non-lichenised species resembling *Ph. praecedens* but with smaller fruiting bodies approaching in size those of *Ph. tremulicola*; although in *Ph. mildeanum* and *Ph. ornicolum* different sizes have been reported for the non-septate ascospores, it is likely that both are heterotypic synonyms; on twigs of *Fraxinus* (mostly *F. ornus*) in the Southern Alps, certainly declining. – **It**: TAA. **Sl**: SlA.

***Phaeocalicium populneum* (Brond. *ex* Duby) A.F.W. Schmidt**

Syn.: *Calicium populneum* Brond. *ex* Duby, *Embolidium populneum* (Brond. *ex* Duby) Vain., *Phacotium populneum* (Brond. *ex* Duby) Trevis.

F – Subs.: cor – Alt.: 2–3 – Note: saprophytic on thin, mostly decaying twigs of *Populus tremula*. – **Au**: T, S, St, O, N. **It**: TAA, Lomb, Piem. **Sl**: SlA.

***Phaeocalicium praecedens* (Nyl.) A.F.W. Schmidt**

Syn.: *Calicium praecedens* Nyl.

F – Subs.: cor – Alt.: 3 – Note: a non-lichenised species characterised by the non-septate ascospores with smooth walls, a stalk of strongly swollen hyphae reacting K+ aeruginose, and a medium-brown hypothecium; on decaying twigs of *Populus*; widespread in Northern Europe, but very rare in the Alps; the only record from the Eastern Alps (Austria) needs confirmation. – **Au**: N.

***Phaeocalicium tremulicola* (Norrl. *ex* Nyl.) Tibell**

Syn.: *Stenocybe tremulicola* Norrl. *ex* Nyl.

F – Subs.: cor – Alt.: 3 – Note: a non-lichenised species characterised by the less than 0.5 mm high, black, shining ascomata on olivaceous to greyish-brown stalks, a 2-layered exciple of an outer layer of large thick-walled cells and a thin inner layer of periclinally arranged hyphae, and 3-septate ascospores with only slightly uneven walls; only on twigs of *Populus tremula*; widespread in Northern Europe and also reported from the Eastern Alps (Austria). – **Au**: K.

***Protothelenella anodonta* (Nyl.) Hafellner**

Syn.: *Mycoglaena lichenoides* (Rehm *ex* Sacc.) Riedl, *Mycowinteria anodonta* (Nyl.) Sherwood & Boise, *Odontotrema anodontum* Nyl., *Protothelenella lichenoides* (Rehm *ex* Sacc.) *comb. inval.*, *Trematosphaeria lichenoides* Rehm (1875, *nom. nud.*), *Winteria lichenoides* (Rehm *ex* Sacc.) Sacc., *Zignoella lichenoides* Rehm *ex* Sacc. (1878)

F – Subs.: xyl – Alt.: 4 – Note: a doubtfully lichenised species with perithecioid ascomata to 0.6 mm in diam. with a greenish-black ascomatal wall and distinct and numerous interascal filaments, fissitunicate asci with an amyloid apical apparatus in the endoascus, initially 8-spored, but when mature often with reduced spore numbers (4, 6), and hyaline, oblong ascospores (14–18 × 6–8 µm) with a tapering lower end, with 3–5 transversal septa and 1 incomplete longitudinal septum; on bleached wood of coniferous trees; known from Fennoscandia and Central Europe, with a few records from the Eastern Alps (Austria, Italy). – **Au**: T. **It**: TAA.

***Protothelenella croceae* (Bagl. & Carestia) Hafellner & H. Mayrhofer**

Syn.: *Pleospora croceae* (Bagl. & Carestia) Vouaux, *Xenosphaeria croceae* Bagl. & Carestia

F – Subs.: ter-sil-par – Alt.: 4–5 – Note: an arctic-alpine lichenicolous fungus found on old, dying thalli of *Solorina crocea* and *Peltigera*-species, with optimum above treeline; certainly more widespread in the Alps, but probably overlooked. – **Au**: T, K, St. **Sw**: GR. **It**: Frl, Piem.

***Protothelenella polytrichi* Döbbeler & H. Mayrhofer**

F – Subs.: bry – Alt.: 5 – Note: an apparently non-lichenised (contact to algals cells not observed so far), muscicolous species with minute, ovoid or pear-shaped perithecia (less than 300 µm in diam.), and narrowly ellipsoid, phragmospored ascospores with usually 3 transversal septa; on leaflets of *Polytrichastrum sexangulare*, easily overlooked when on the upper side of the dry moss with enrolled leaflets; overall distribution arctic-alpine, but apparently rare, with some scattered records from the Alps. – **Au**: T, S, St. **Sw**: TI, VS.

***Protothelenella viridis* (Rehm) Hafellner**

Syn.: *Melanomma viridis* Rehm, *Mycoglaena viridis* (Rehm) Riedl, *Winteria viridis* (Rehm) Sacc.

F # – Subs.: xyl – Alt.: 4 – Note: a probably non-lichenised species, apparently closely related to *P. anodonta*, the perithecioid ascomata (*c.* 0.3 mm in diam.) with a greenish-black ascomatal wall, the interascal filaments distinct and numerous, with a hemiamyloid hymenial gel, ascospores hyaline, ellipsoid-oblong (17–18 × 4–7 µm), with 3–5 transversal septa and 1 incomplete longitudinal septum; lignicolous on decorticated, dead branches of *Rhododendron ferrugineum*; only known from the Eastern Alps (Austria). – **Au**: T.

***Pseudotryblidium neesii* (Flot. *ex* Körb.) Rehm**

Syn.: *Dactylospora neesii* (Flot. *ex* Körb.) Arnold, *Leciographa neesii* Flot. *ex* Körb., *Peziza neesii* Flot. *ex* Körb.

F – Subs.: cor – Alt.: 2–3 – Note: a non-lichenised helotialean discomycete with erumpent, later sessile, black apothecia with a rusty-red tinge, and a rough prominent margin when dry, and ascospores initially non – to 1-septate and hyaline, but finally 3-septate and pale brown (15–18 × 6–8 µm); on bark of *Abies*, sometimes breaking through crustose lichen thalli (*e.g.* of *Loxospora*-species and *Phlyctis argena*) and then virtually lichenicolous; widespread in Europe, the distribution gaps in the Alps are probably due to undercollecting. – **Au**: S, K, St, O, N. **Ge**: OB, Schw. **Sw**: BE, SG. **It**: Frl. **Sl**: SlA.

***Pyrenula coryli* A. Massal.**

Syn.: *Arthopyrenia coryli* (A. Massal.) Müll. Arg., Arthopyrenia glabrata (Ach.) H. Olivier var. coryli (A. Massal.) H. Olivier, Microthelia glabrata (Ach.) Boistel var. coryli (A. Massal.) Boistel, *Mycopyrenula coryli* (A. Massal.) Vain., *Verrucaria coryli* (A. Massal.) Nyl.

F – Subs.: cor – Alt.: 2–3 – Note: a temperate species of smooth bark, most frequent on *Corylus*; the thallus, indicated by ochre-olive patches, is doubtfully lichenised. – **Sw**: BE, VS. **Fr**: AMa, HSav. **It**: Ven, TAA, Lomb, Piem.

***Resinicium bicolor* (Alb. & Schwein.) Parmasto**

Syn.: *Corticium granulare* Burt, *Hydnum bicolor* Alb. & Schwein., *Hydnum echinosporum* Velen., *Hydnum subtile* Fr., *Odontia bicolor* (Alb. & Schwein.) Quél.

F – Subs.: cor – Alt.: 3 – Note: a species with corticioid carpophores. Although endoxylic to epixylic colonies of coccaceous green algae can often be detected below or in the neighbourhood of the carpophores, it is unlikely to be lichenised. The hymenium has two main types of cystidia, one with an apical compartment filled with oil, the second with an apical bundle of crystals. On rotting wood of both coniferous and deciduous trees; widespread in Europe and not rare; there are many records from the Alps in all countries, but algal colonies usually are not observed. We do not specify records from the different parts of the Alps, since these are usually present in the literature dealing with Basidiomycetes. – **Au**: Au. **Ge**: Ge. **Sw**: Sw. **Fr**: Fr. **It**: It. **Sl**: Sl. **Li**.

***Sarea difformis* (Fr.) Fr.**

Syn.: *Biatorella difformis* (Fr.) Vain., *Peziza difformis* Fr., *Tromera difformis* (Fr.) Arnold, *Tromera sarcogynoides* A. Massal. *ex* Arnold *nom.illeg*.

F – Subs.: res – Alt.: 3–4 – Note: a non-lichenised discomycete with minute brown-black apothecia (to 1 mm in diam.) developing on a subiculum, with polyspored asci and spherical ascospores (2–3 µm in diam.), sometimes accompanied or replaced by an anamorphic brown-black pycnidial state with spherical, pale brown conidia; growing on the resinous exudates of coniferous trees in rather shaded and humid situations, sometimes also found on bark at the base of the trunks of old *Abies* and *Picea* in montane forests; widespread in the Holarctic region, both in Europe and in North America; in the Alps not rare, but easier to be overlooked than the more conspicuous *Tromera resinae*. – **Au**: T, K, St, N. **Ge**: Ge. **Sw**: SZ. **Fr**: HSav, Var, Vau. **It**: Ven, TAA, Lomb, Piem. **Sl**: SlA.

***Spheconisca austriaca* Norman**

F # – Subs.: cor – Alt.: 2 – Note: a doubtfully lichenised species, perhaps belonging to Chaetosphaeriales, with a blackish, subiculum-like, granular thallus, minute, globose ascomata containing 8-spored asci, and 1-septate, brown, oblong ascospores (to 18 µm long); on bark and wood of conifers (*Pinus nigra*); only known from the type locality in the Eastern Alps (Austria). – **Au**: T.

***Spheconisca confusa* Norman**

Syn.: *Phaeomeris confusa* (Norman) Clem.

F # – Subs.: res – Alt.: 3 – Note: a non-lichenised species perhaps belonging to Pleosporales, resembling *S. resinae*, the globose fragile ascomata containing 8-spored asci and brown ascospores with 3–7 transversal septa (to 18 µm long); on resin of conifers (*e.g. Abies*); rare in Europe, in the study area only known from the Eastern Alps (Austria). – **Au**: T.

***Spheconisca ebenea* Norman**

F # – Subs.: cor – Alt.: 3 – Note: a poorly known, non-lichenised species, perhaps belonging to Chaetosphaeriales, with a spreading, pure black, relatively thick, subiculum-like thallus, 8-spored asci, and hyaline, narrowly fusiform, 3-septate ascospores (15–16 × 3–4 μm); on the trunks and twigs of *Alnus viridis*; only known from the type locality in the Eastern Alps (Austria). – **Au**: T.

***Spheconisca humilis* Norman**

F # – Subs.: cor – Alt.: 2 – Note: a non-lichenised species perhaps belonging to Chaetosphaeriales, with a spreading, black, subiculum-like thallus, minute ascomata, 8-spored asci, and hyaline, ellipsoid to fusiform, 3-septate ascospores (8–10 μm long); on *Acer pseudoplatanus*, only known from the type locality in the Eastern Alps (Austria). – **Au**: T.

***Spheconisca hypocrita* Norman**

F # – Subs.: cor – Alt.: 3 – Note: a doubtfully lichenised species with a spreading, black, rough, rather thick, subiculum-like thallus, the rather small ascomata containing 8-spored asci and hyaline to brownish, fusiform, 3-septate to submuriform ascospores (12–13 μm long); on branches of *Larix*, only known from the type locality in the Eastern Alps (Austria). – **Au**: T.

***Spheconisca indifferens* Norman**

F # – Subs.: cor – Alt.: 3 – Note: a doubtfully lichenised species with a thin, blackish, subiculum-like thallus, 8-spored asci, and hyaline, narrowly fusiform, simple ascospores (to *c.* 12 μm long); on branches of *Betula*, only known from the type locality in the Eastern Alps (Austria). – **Au**: T.

***Spheconisca margaritula* (Norman *ex* Bachm.) Mig.**

Syn.: [*Spheconisca* subgen.] *Euspheconisca margaritula* Norman *ex* Bachm.

F # – Subs.: cor – Alt.: 3 – Note: a doubtfully lichenised species with a thallus composed of goniocyst-like, flattened particles, subspherical ascomata (*c.* 0.08 mm in diam.), 8-spored asci, and hyaline, 1 – later 3-septate ascospores (9–10 × 3–3.5 μm); on twigs of *Berberis*, only known from the type locality in the Eastern Alps (Austria). – **Au**: T.

***Spheconisca obducens* Norman**

F # – Subs.: cor – Alt.: 2 – Note: a non-lichenised species perhaps belonging to Pleosporales, with a thallus forming blackish patches composed of tightly adpressed hyphae, minute ascomata, 8-spored asci, and hyaline, ellipsoid to fusiform, 1 – to 3-septate ascospores (8–10 μm long); on the trunk of *Ficus*, only known from the type locality in the Eastern Alps (Italy). – **It**: Lomb.

***Spheconisca semnospora* (Norman *ex* Bachm.) Mig.**

Syn.: [*Spheconisca* subgen.] *Euspheconisca semnospora* Norman *ex* Bachm.

F # – Subs.: cor – Alt.: 3 – Note: a doubtfully lichenised species with a divided, blackish, subiculum-like thallus, subspherical ascomata (*c.* 0.1 mm in diam.), 8-spored asci, and subhyaline, ellipsoid, 3-septate ascospores (9–10 × 3–3.5 μm); on bark of *Rhus typhina*, only known from the type locality in the Eastern Alps (Austria). – **Au**: T.

***Spheconisca translucens* Norman**

F # – Subs.: cor – Alt.: 2 – Note: a non-lichenised species with a dark grey, subiculum-like thallus, brown ascomata, 8-spored asci, and brownish, oblong to narrowly fusiform, 3-septate ascospores (10–15 μm long); on a trunk of *Populus*, only known from the type locality in the Eastern Alps (Italy). – **It**: TAA.

***Stenocybe major* Nyl. *ex* Körb.**

Syn.: *Calicium eusporum* Nyl., *Stenocybe euspora* (Nyl.) Anzi

F – Subs.: cor – Alt.: 3–4 – Note: a non-lichenised species characterised by ascomata with unbranched or furcate stalks, and narrowly clavate capitula (together up to 1.5 mm high), with light brown, 4-celled, narrowly ellipsoid ascospores (19–32 × 5–7 µm); on trunks of old trees, especially *Abies*, in humid montane forests, sometimes breaking through thalli of *Loxospora*-species and then virtually lichenicolous; widespread in temperate Europe and North America, but not common. – **Au**: V, T, S, K, St, O, N. **Ge**: OB. **Sw**: SZ. **Fr**: Isè. **It**: TAA, Lomb, Piem. **Sl**: SlA.

***Stenocybe pullatula* (Ach.) Stein**

Syn.: *Calicium byssaceum* Fr., *Calicium pullatulum* Ach., *Stenocybe byssacea* (Fr.) Körb.

F – Subs.: cor – Alt.: 2–3 – Note: saprobic or parasitic on branches of *Alnus*, on decaying branches and trunks, especially along streams and lakes, usually associated with algal colonies, but most probably non-lichenised. – **Au**: V, T, S, K, St, O, N. **Ge**: OB. **Fr**: Isè. **It**: Frl, TAA, Lomb, Piem. **Sl**: SlA. **Li**.

***Stenocybe septata* (Leight.) A. Massal.**

Syn.: *Sphinctrina septata* Leight.

F – Subs.: cor – Alt.: 3 – Note: a non-lichenised species characterised by the large ascospores (45–60 × 15–20 µm) with various numbers of septa (usually 3-septate); on bark of broad-leaved trees (mainly *Ilex*); a rare species in old woodlands, mainly in Western Europe, with a few ancient records from the Eastern Alps (Austria and Italy), which however need confirmation. – **Au**: T. **It**: TAA.

***Stictis candida* (J. Steiner *ex* Keissl.) M.B. Aguirre**

Syn.: *Leptorhaphis candida* J. Steiner *ex* Keissl.

F – Subs.: cor – Alt.: 2 – Note: a most likely non-lichenised fungus with filiform, phragmospored ascospores (more than 150 µm long); on bark of deciduous trees (*Fraxinus ornus*); only known from the type locality in Slovenia. – **Sl**: SlA.

***Thelocarpon strasseri* Zahlbr.**

Syn.: *Ahlesia strasseri* (Zahlbr.) Keissl.

F # – Subs.: xyl – Alt.: 3–4 – Note: a species developing minute apothecia; the distinction from *Th. lichenicola* (type lichenicolous on *Baeomyces rufus* and asci – according to the protologue – 16-spored) may be not justified, and this taxon is not generally accepted as a separate species, a problem which, however, needs further study. – **Au**: K, St, O, N.

***Tomasellia arthonioides* (A. Massal.) A. Massal.**

Syn.: *Arthopyrenia arthonioides* A. Massal., *Melanotheca arthonioides* (A. Massal.) Nyl., *Pyrenula arthonioides* (A. Massal.) Trevis.

F – Subs.: cor – Alt.: 1–3 – Note: a mild-temperate fungus, most frequent on the smooth bark of *Fraxinus ornus* in the submediterranean belt. – **Au**: K, B. **It**: Frl, Ven, TAA, Lomb, Piem. **Sl**: SlA.

***Tromera resinae* (Fr.) Körb.**

Syn.: *Biatorella resinae* (Fr.) Th. Fr., *Sarea resinae* (Fr.) Kuntze, *Sphaeria resinae* Fr., *Tromera myriospora* (Hepp) Anzi

F – Subs.: res – Alt.: 2–4 – Note: a non-lichenised discomycete with medium-sized, orange to reddish apothecia (to 1.5 mm in diam.) recalling those of *Bacidia rubella*, polyspored asci, and spherical ascospores (2–3 µm in diam.), sometimes accompanied or replaced by an anamorphic pale orange pycnidial state with spherical, hyaline conidia; growing on the resin of coniferous trees in the upper montane and subalpine belts; widespread in the Holarctic region, both in Europe and in North America, and also recorded from the Himalayas and the East African highlands; not rare throughout the Alps. – **Au**: T, S, K, St, O, N. **Ge**: OB. **Sw**: SZ. **Fr**: Var. **It**: Frl, TAA, Lomb, Piem. **Sl**: SlA.

### Particularly dubious records and extremely poorly known taxa

***Arthonia aquatica* Riedl**

Subs.: xyl-aqu – Alt.: 3 – Note: a very dubious taxon, compared by the author with *Micarea prasina*! – **Au**: N.

***Arthonia calcarea* (Turner *ex* Sm.) Ertz & Diederich**

Syn.: *Opegrapha calcarea* Turner *ex* Sm., *Opegrapha chevallieri* Leight., Opegrapha confluens *auct. non* (Ach.) Stizenb.

Subs.: cal – Alt.: 1 – Note: this species, belonging to a difficult group of closely related taxa, has a thick, chalky, pure white thallus, and often was not distinguished from *A. trifurcata*; it grows on limestone, brick, roofing tiles, etc. in sheltered situations. According to Roux, this is a strictly littoral species, and all the inland records from the Alps are likely to refer to *A. trifurcata*. – **Au**: ?T, ?S, ?K, ?St, ?O, ?N. **Ge**: ?OB. **Sw**: ?SZ. **It**: ?Frl, ?Ven, ?TAA, ?Lomb, ?Piem, ?Lig. **Sl**: ?SlA.

***Aspicilia epiglypta* (Norrl. *ex* Nyl.) Hue**

Syn.: *Lecanora epiglypta* Norrl. *ex* Nyl.

Subs.: sil – Alt.: 4 – Note: *A. epiglypta* is restricted to coastal areas in Northern Europe and records from elsewhere may be due to confusion with other species, especially with *A. prestensis*. – **Au**: ?T, ?K.

***Aspicilia erythrantha* Poelt (not validly published)**

Subs.: int – Alt.: 6 – Note: a fertile, not validly published species with rather irregular marginal lobes reacting K+ red, whose taxonomic value is in need of evaluation, with a few records from hard schists rich in calcium. – **Au**: T. **It**: TAA.

***Aspicilia lactea* A. Massal.**

Subs.: cal – Alt.: 3 – Note: Cl. Roux has seen the type and has annotated that it belongs to a *Lecania* (see Nimis 2016). – **Au**: ?V.

***Aspicilia leprosescens* (Sandst.) Hav.**

Syn.: *Circinaria leprosescens* (Sandst.) A. Nordin, Savić & Tibell, *Lecanora leprosescens* Sandst.

Subs.: sil – Alt.: 5 – Note: this is a maritime lichen, and the record from Austria is most probably due to a misidentification. – **Au**: ?T.

***Aspicilia oleosa* Poelt (not validly published)**

Subs.: sil, met – Alt.: 4–5 – Note: a fertile taxon with an indistinctly effigurate thallus and conspicuous “oil-cells” in the medulla; on schist rich in iron, distribution still insufficiently known. – **Au**: K.

***Aspicilia perradiata* (Nyl.) Hue**

Syn.: Aspicilia polychroma Anzi var. perradiata (Nyl.) Clauzade & Cl. Roux, *Lecanora perradiata* Nyl.

Subs.: sil – Alt.: 5–6 – Note: a species with a dark grey, effigurate thallus with narrow lobes; poorly known, based on a type from easternmost Siberia (Bering Strait area); the identity of records from the Alps is uncertain (they may refer to *A. polychroma*). – **Au**: ?V, ?T.

***Aspicilia subradiascens* (Nyl.) Hue**

Syn.: *Lecanora subradiascens* Nyl.

Subs.: sil, int – Alt.: 4–5 – Note: a fertile species with a blackish-grey, subeffigurate thallus not reacting with K; on the whole, a poorly known taxon based on a type from easternmost Siberia (Bering Strait area); the identity of the records from the Alps is highly uncertain. – **Au**: ?T, ?St.

***Aspicilia virginea* Hue**

Subs.: sil – Alt.: 5 – Note: the occurrence in Austria of this mainly Arctic species, based on a non-published dissertation, is doubtful. – **Au**: ?K.

***Bacidia ephemera* Poelt & Vězda ined.**

Subs.: deb – Alt.: 2 – Note: a bacidioid species only provisionally named and not further described, found on *Scirpus sylvaticus*, on leaves of the previous year, and therefore apparently with a short life span; only recorded from the Eastern Alps (Austria). – **Au**: St.

***Bacidina apiahica* (Müll. Arg.) Vězda**

Syn.: *Patellaria apiahica* Müll. Arg.

Subs.: fol – Alt.: 2 – Note: a foliicolous pan – to subtropical species, in Southern Europe restricted to very humid and warm forests near the coast. Its occurrence in Austria was due to a misidentification: the specimen proved to be *Bacidina chloroticula* (det. E. Serusiaux). – **Au**: ?St.

***Biatora cuprea* (Sommerf.) Fr.**

Syn.: *Lecidea cuprea* Sommerf., *Pyrrhospora cuprea* (Sommerf.) M. Choisy

Subs.: ter-sil, deb-sil – Alt.: 3–5 – Note: most frequent on soil and plant debris on siliceous substrata, in upland areas. This species is known with certainty only from Northern Europe and Western North America, but it has been reported from several localities in the Alps (see *e.g.* Roux et al. 2014). Its presence in the Alps, however, is very dubious, and most records from this area could refer to *B. subduplex*. – **Sw**: ?GR. **Fr**: ?HAl. **It**: ?TAA, ?Lomb, ?Piem, ?VA.

***Bryobilimbia diapensiae* (Th. Fr.) Fryday, Printzen & S. Ekman**

Syn.: *Biatora diapensiae* (Th. Fr.) Hellb., *Lecidea diapensiae* Th. Fr.

Subs.: bry – Alt.: 2–3 – Note: an arctic species of the *B. hypnorum*-group with an olive hypothecium; the only record from the Alps (Switzerland), from mid-elevation, is probably based on a misidentification. – **Sw**: ?VD.

***Bryocaulon divergens* (Ach.) Kärnefelt**

Syn.: *Alectoria divergens* (Ach.) Nyl., *Cornicularia divergens* Ach.

Subs.: ter-sil – Alt.: 5 – Note: the occurrence in Austria is doubtful (Hafellner and Türk 2016), as well as all records from the Alps (Nimis 1993). – **Au**: ?St. **Sw**: ?VS. **It**: ?Piem.

***Buellia sequax* (Nyl.) Zahlbr.**

Syn.: *Buellia excelsa* (Leight.) A.L. Sm., *Lecidea excelsa* Leight., *Lecidea sequax* Nyl.

Subs.: sil – Alt.: 1–3 – Note: *B. sequax* in the strict sense is only known with certainty from the type locality in France, outside the Alps. The Austrian record is likely to be due to a misidentification – **Au**: ?T.

**Buellia triphragmia*auct. non* (Nyl.) Arnold**

Subs.: cor – Alt.: 3 – Note: a name applied in Central Europe to a lichen in the *B. disciformis*-group, with 3-septate ascospores (perhaps just *B. disciformis* with overaged ascospores); on bark of various trees, occasionally recorded throughout Europe, including the Alps. – **Au**: ?V, ?T, ?S, ?St, ?N.

**Caloplaca biatorina (A. Massal.) J. Steiner subsp. gyalolechioides (Müll. Arg.) Clauzade & Cl. Roux**

Syn.: Amphiloma murorum var. gyalolechioides Müll. Arg.

Subs.: cal – Alt.: 1–3 – Note: a poorly known calcicolous taxon: the name was usually applied to pruinose populations with the characters of *C. biatorina*; not always distinguished, and distribution therefore incompletely documented, with a few scattered records throughout Europe, including the Alps, at low elevations. – **Au**: K. **Fr**: HSav.

***Caloplaca fraudans* (Th. Fr.) H. Olivier**

Syn.: Caloplaca ferruginea (Huds.) Th. Fr. var. fraudans Th. Fr.

Subs.: cal, sil – Alt.: 4–5 – Note: this is a mainly Arctic species; the records from Austria and Italy are most probably wrong. – **Au**: ?V. **It**: ?VA.

***Caloplaca karakorina* Poelt & Hinteregger**

Subs.: cal – Alt.: 5 – Note: the record from Austria is very unprobable. – **Au**: ?V.

***Caloplaca lecidellae* Poelt & Hinteregger**

Subs.: cal-par – Alt.: 5 – Note: the record from Austria is very unprobable. – **Au**: ?T.

***Candelariella oleaginescens* Rondon**

Subs.: cal – Alt.: 4–5 – Note: a mainly coastal species. Earlier reports from Austria are probably due to misidentifications, since their altitudinal distribution does not fit with that of the type. – **Au**: ?T, ?K.

**Cladonia gracilis (L.) Willd. subsp. elongata (Wulfen) Vain.**

Syn.: *Cladonia elongata* (Wulfen) Hoffm., Cladonia gracilis (L.) Willd. var. elongata (Wulfen) Fr., Cladonia gracilis (L.) Willd. subsp. nigripes (Nyl.) Ahti, *Cladonia nigripes* (Nyl.) Trass, *Lichen elongatus* Wulfen

Subs.: ter – Alt.: 4 – Note: a taxon forming large mats of squamulose podetia with melanotic bases, based on a type from Southern Chile, very similar to *C. macroceras* (with shiny, often slightly squamulose podetia); on soil and in rock beds; bipolar, in Europe mainly northern boreal to Arctic; the presence in the Alps is very dubious. – **Sl**: ?SlA.

***Collema thysanoeum* Ach.**

Subs.: cor – Alt.: 2–3 – Note: a rare species with a thallus of crowded, suberect, roundish lobes with granulose margins, perhaps related to *Lathagrium fuscovirens*; based on a type from Switzerland, but material very poorly developed. – **Sw**: Sw.

***Cladonia perlomera* Kristinsson**

Subs.: xyl – Alt.: 2–3 – Note: on rotting wood; the record from Italy is dubious. – **It**: ?Lomb.

***Diploschistes caesioplumbeus* (Nyl.) Vain.**

Syn.: Diploschistes actinostoma (Ach.) Zahlbr. var. caesioplumbeus (Nyl.) J. Steiner, Urceolaria actinostoma (Ach.) Schaer. var. caesioplumbea Nyl.

Subs.: sil – Alt.: 1–2 – Note: a species with a grey, non-pruinose thallus and entirely immersed apothecia with punctiform discs, 4 – to – 8-spored asci, and large muriform ascospores; optimum on coastal siliceous rocks in the supralittoral zone, often together with *Ramalina scopularis*; widespread along the Atlantic and Mediterranean coasts; an earlier record from Austria (V) was evidently based on a misidentification. – **Au**: ?V.

***Fuscidea badensis* V. Wirth & Poelt ined.**

Subs.: sil – Alt.: 3–5 – Note: a herbarium name, the taxon was never validly published. – **Au**: V, T.

***Fuscidea oculata* Oberholl. & V. Wirth**

Subs.: sil – Alt.: 3 – Note: the record from Austria, the only one from the Alps, is dubious. – **Au**: ?T.

***Heterodermia japonica* (M. Satô) Swinscow & Krog**

Syn.: Anaptychia dendritica (Pers.) Vain. var. japonica M. Satô

Subs.: cor – Alt.: 3 – Note: the record from Slovenia most likely refers to *Polyblastidium subneglectum* (see Nimis 2016). – **Sl**: ?Tg.

***Hypotrachyna rockii* (Zahlbr.) Hale**

Syn.: *Parmelia rockii* Zahlbr.

Subs.: cor – Alt.: 3 – Note: the record from Switzerland most probably refers to *H. taylorensis*. – **Sw**: ?UW.

***Lambiella impavida* (Th. Fr.) M. Westb. & Resl**

Syn.: *Lecidea impavida* Th. Fr., *Rimularia impavida* (Th. Fr.) Hertel & Rambold

Subs.: sil – Alt.: 5 – Note: a circumpolar, arctic species colonising as a pioneer loose siliceous pebbles on the ground. Arnold’s record from Tyrol, based on sterile specimens, is most likely due to a misidentification. – **Au**: ?T.

***Lecanora cinerescens* (Harm.) Ozenda & Clauzade**

Syn.: Lecanora atriseda var. cinerescens Harm.

Subs.: sil – Alt.: 5 – Note: the record from Austria is most probably due to a misidentification. – **Au**: ?V.

***Lecidea atomaria* Th. Fr.**

Subs.: sil – Alt.: 5 – Note: the record from Austria is very dubious (Hafellner and Türk 2016). – **Au**: ?T.

***Lecidea atrosanguinea* (Hoffm.) Nyl.**

Syn.: *Verrucaria atrosanguinea* Hoffm.

Subs.: cal – Alt.: 3 – Note: the records from Austria are very dubious, and probably refer to a species of *Lecidella* (Hafellner and Türk 2016). – **Au**: ?S, ?N.

***Lecidella leprothalla* (Zahlbr.) Knoph & Leuckert**

Syn.: *Lecidea leprothalla* Zahlbr.

Subs.: cor – Alt.: 2–4 – Note: the identity of the Swiss samples is not certain. – **Sw**: ?BE, ?GR, ?TI, ?VS.

***Lempholemma dispansum* H. Magn.**

Subs.: cal – Alt.: 3 – Note: the record from Austria is dubious (Hafellner and Türk 2016). – **Au**: ?St.

***Melanohalea olivacea* (L.) O. Blanco, A. Crespo, Divakar, Essl., D. Hawksw. & Lumbsch**

Syn.: *Lichen olivaceus* L., *Melanelia olivacea* (L.) Essl., *Parmelia olivacea* (L.) Ach.

Subs.: cor – Alt.: 3–4 – Note: the name *Parmelia olivacea* was often used for *Melanelixia glabra* and for several other species of this group. All records from the Alps are unreliable. – **Au**:? T, ?O. **It**: ?Ven, ?Lomb, ?VA.

***Micarea melanobola* (Nyl.) Coppins**

Syn.: *Catillaria melanobola* (Nyl.) B. de Lesd., *Lecidea melanobola* Nyl.

Subs.: xyl – Alt.: 4 – Note: this species, which can be easily confused with *M. prasina*, is known with certainty only from Southern Finland, on bark of *Picea*. The record from Switzerland is very dubious. – **Sw**: ?VS.

**Parmelia omphalodes (L.) Ach. subsp. discordans (Nyl.) Skult**

Syn.: *Parmelia discordans* Nyl.

Subs.: sil – Alt.: 5 – Note: a subspecies with frequently convex lobes and with medulla reacting K-, Pd – (salazinic acid lacking, but with an otherwise complex secondary chemistry); on siliceous rocks, most common in the Northern European lowlands, in Central Europe often not distinguished, and therefore rarely recorded. The Austrian record is dubious. – **Au**: ?V.

***Parmotrema robustum* (Degel.) Hale**

Syn.: Parmelia dilatata *auct. non* Vain., *Parmelia robusta* Degel.

Subs.: cor – Alt.: 1–3 – Note: a humid subtropical species found on broad-leaved trees in humid-warm situations; the record from Switzerland is extremely dubious. – **Sw**: ?BE.

**Pertusaria coccodes (Ach.) Nyl. var. petraea Erichsen**

Subs.: sil – Alt.: 5 – Note: a very dubious taxon (see Hafellner and Türk 2016). – **Au**: ?V.

***Pertusaria jurana* Erichsen**

Subs.: cor – Alt.: 3 – Note: the record from the Slovenian Alps is very dubious. – **Sl**: ?SlA.

**Phaeorrhiza sareptana (Tomin) H. Mayrhofer & Poelt var. sareptana**

Syn.: Rinodina nimbosa (Fr.) Th. Fr. f. sareptana Tomin, *Rinodina sareptana* (Tomin) H. Magn.

Subs.: bry-cal, ter-cal – Alt.: 4 – Note: similar to *Ph. nimbosa*, but apothecia with a proper margin, and differing from var. sphaerocarpa in the constantly epruinose thallus; on saline soils in Eastern Europe; the records from Switzerland are likely to belong to var. sphaerocarpa. – **Sw**: ?GR, ?VS.

**Placynthium stenophyllum (Tuck.) Fink var. stenophyllum**

Syn.: *Pannaria stenophylla* Tuck.

Subs.: cal – Alt.: 3 – Note: a species described from North America and also known from Northern Europe, with pale brown, cylindrical lobes, and apothecia (if present) with a secondary thalline margin (!); Central European records, including that from Bavaria, are in urgent need of confirmation, as they perhaps refer to *P. posterulum*. – **Ge**: ?OB.

**Placynthium stenophyllum (Tuck.) Fink var. isidiatum Henssen**

Subs.: cal – Alt.: 4 – Note: a taxon described and known with certainty only from North America; the Central European records are in need of confirmation, and perhaps refer to *P. posterulum* or *P. subradiatum*. – **Au**: ?V.

***Polyblastia gothica* Th. Fr.**

Subs.: deb, ter-cal – Alt.: 4–5 – Note: most probably this is a synonym of the lichenicolous fungus *Merismatium nigritellum* (Nyl.) Vouaux (Hafellner and Türk 2016). – **Au**: V, K, St. **Sw**: SZ.

**Porpidia tuberculosa (Sm.) Hertel & Knoph var. rubescens A.J. Schwab ined.**

Subs.: sil – Alt.: 5 – Note: this undescribed taxon is probably identical with *Bellemerea subsorediza*. – **Au**: T.

***Protoparmelia atriseda* (Fr.) R. Sant. & V. Wirth**

Syn.: *Lecanora atriseda* (Fr.) Nyl., Lecanora nephaea *auct. non* Sommerf., Parmelia badia (Hoffm.) Hepp var. atriseda Fr.

Subs.: sil – Alt.: 3–5 – Note: on hard siliceous rocks in upland areas, with optimum above treeline, starting the life-cycle on yellow *Rhizocarpon*-species, later becoming autonomous. A heterogeneous taxon, which does not belong to *Protoparmelia* *s.str.*; all records from the Alps are dubious. – **Au**: ?V, ?T. **Sw**: ?GR. **It**: ?TAA.

***Protoparmelia nitens* (Nyl.) Sancho & A. Crespo**

Syn.: *Lecanora nitens* (Pers.) Ach.

Subs.: cal – Alt.: 2–3 – Note: an often misunderstood silicicolous species. The Italian records, especially those from upland areas, are dubious. – **It**: ?TAA, ?Piem, ?VA, ?Lig.

***Rhizocarpon jemtlandicum* (Th. Fr. & Almq.) Malme**

Subs.: sil – Alt.: 4 – Note: the record from Austria is very dubious. – **Au**: ?V.

***Rhizocarpon lusitanicum* (Nyl.) Arnold**

Subs.: int-par – Alt.: 1–2 – Note: a S-European species of siliceous rocks which starts the life-cycle on the thalli of *Pertusaria*-species below the montane belt; the high-altitude record from Austria is very dubious. – **Au**: ?V.

***Rinodina aequata* (Ach.) Flagey**

Syn.: Lecidea coniops Ach. var. aequata Ach.

Subs.: cal, sil – Alt.: 2–4 – Note: a taxon of uncertain generic placement, based on two specimens from the Western Alps (France and Switzerland). In a later publication (Acharius, Syn. Meth. Lich. 1814) Acharius himself synonymised it with *Lecidea lapicida*. Because of the missing types, the records are impossible to interpret, as this name has been used for different taxa from different substrates. – **Sw**: UR, VD. **Fr**: HSav.

***Rinodina arnoldii* H. Mayrhofer & Poelt**

Subs.: sil, int – Alt.: 4–5 – Note: most records from the subalpine and alpine belts refer to *R. milvina*. The type specimens are from northern Bavaria (outside the Alps); the records from the Alps are in need of re-evaluation. – **Au**: ?V, ?T, ?K. **It**: ?TAA, ?Lomb.

**Sarcogyne regularis Körb. var. macrocarpa (B. de Lesd.) N.S. Golubk.**

Syn.: *Sarcogyne pruinosa* *auct.* var. macrocarpa B. de Lesd.

Subs.: int – Alt.: 5 – Note: a taxon with an epilithic, grey thallus and large, sessile apothecia (more than 1 mm in diam.) which are pruinose at first, then non-pruinose, with a persistent prominent margin; not consistently distinguished and distributional data therefore incomplete and hard to interpret. The record from Austria is very dubious (Hafellner and Türk 2016). – **Au**: ?V.

***Scytinum tetrasporum* (Th. Fr.) Otálora, P.M. Jørg. & Wedin**

Syn.: *Leptogium tetrasporum* Th. Fr.

Subs.: ter-cal – Alt.: 5 – Note: the record from Austria is very dubious. – **Au**: ?S.

***Siphulastrum alpinum* Jatta**

Subs.: ter – Alt.: 4–5 – Note: known only from the type collection. The envelope purported to contain the type, in NAP, is empty; the type is presently in S (L474) and appears to be almost completely eaten by insects and thus unidentifiable. An annotation by A. Henssen states that it could be a taxon in the Heppiaceae (see also Nimis 2016). – **It**: Piem.

***Staurothele arctica* Lynge**

Subs.: sax – Alt.: 4–5 – Note: a mainly Arctic species; the identification of the records from Austria and Switzerland are very dubious. – **Au**: ?V. **Sw**: ?VS.

***Stereocaulon cumulatum* (Sommerf.) Timdal**

Syn.: *Lecidea conglomerata* Sommerf., *Lecidea cumulata* Sommerf., *Lecidea paracarpa* Nyl., *Lecidea perfidiosa* Nyl., *Toninia cumulata* (Sommerf.) Th. Fr.

Subs.: sil – Alt.: ?3 – Note: this species is known with certainty only from Greenland and Russia; the records from Switzerland are most probably due to misidentifications. – **Sw**: ?BE, ?VS.

***Stereocaulon paschale* (L.) Hoffm.**

Syn.: *Lichen paschalis* L.

Subs.: ter-sil – Alt.: 3–5 – Note: all records from the Alps are uncertain and in need of confirmation, perhaps referring to either *S. grande* or morphs of *S. alpinum*. – **Au**: ?K, ?St, ?N. **Sw**: ?GR, ?VS.

***Stereocaulon spathuliferum* Vain.**

Subs.: sil – Alt.: 5 – Note: the records from Austria are very dubious (Hafellner and Türk 2016). – **Au**: ?V, ?T.

***Sticta canariensis* (Flörke) Bory ex Delise**

Syn.: *Pulmonaria canariensis* Flörke, *Sticta dufourii* Delise

Subs.: cor – Alt.: 3 – Note: a humid subtropical lichen found on bark and epiphytic mosses in very moist forests, sometimes on mossy rocks. The morph with cyanobacteria is the only one occurring in Europe; the Swiss record is most probably based on a misidentification. – **Sw**: ?VS.

***Toninia sculpturata* (H. Magn.) Timdal**

Syn.: *Catillaria sculpturata* H. Magn.

Subs.: cal, int – Alt.: 3 – Note: a species with a thallus composed of orbicular, convex, pale yellowish squamules, apothecia with a colourless hypothecium and a dark brown epihymenium, and 1-septate ascospores; on steep to underhanging surfaces of calciferous rocks in dry places; widespread in the Holarctic region but rare; the only record from the Alps (Austria) needs confirmation. – **Au**: ?T.

***Umbilicaria spadochroa* (Hoffm.) DC.**

Syn.: *Gyrophora cirrhosa* *auct. non* (Hoffm.) Vain., *Gyrophora spadochroa* (Hoffm.) Ach., *Omphalodiscus spadochrous* (Hoffm.) Schol., Umbilicaria cirrhosa *auct. non* Hoffm.

Subs.: sil – Alt.: 4–5 – Note: a mainly western-oceanic species growing on inclined surfaces of siliceous rocks near or above treeline; all records from the Alps need confirmation, due to frequent confusion with *U. crustulosa*. – **Sw**: ?BE, ?GR, ?VS. **It**: ?TAA, ?Lomb, ?Piem, ?VA.

***Usnea articulata* (L.) Hoffm.**

Syn.: *Lichen articulatus* L., Usnea articulata (L.) Hoffm. subsp. mediterranea Motyka

Subs.: cor – Alt.: 1–3 – Note: a Mediterranean-Macaronesian lichen with subtropical affinities found on the branches of ancient trees in humid forests; in our opinion, considering both the sources and the localities, all records from the Alps are very dubious. – **Sw**: ?GR, ?VS. **It**: ?Ven, ?Lomb.

***Usnea flammea* Stirt.**

Syn.: *Usnea dalmatica* Motyka, *Usnea rupestris* Motyka

Subs.: cor, sax, ter – Alt.: 2–3 – Note: a western and southern European, oceanic species, mainly epiphytic, but also found on rocks and soil in damp situations, below the subalpine belt; the Italian record is very dubious. – **It**: ?Ven.

***Usnea irregularis* Motyka**

Subs.: cor – Alt.: 3 – Note: this taxon has been described on the base of African specimens, and is considered to be a synonym of *U. ruwenzoriana*. European specimens most probably correspond to *U. perplexans* Stirt. – **Au**: T.

***Usnea leucosticta* Vain.**

Subs.: cor – Alt.: 3 – Note: this is most probably a special morphotype of *U. barbata* *s.str.* with unusually stipitate and globulose soralia, perhaps triggered by a lichenicolous fungus. – **Au**: T.

***Usnea majuscula* Motyka**

Subs.: cor – Alt.: 4 – Note: this is a *nomen nudum* (Hafellner and Türk 2016). – **Au**: T.

***Usnea rigidula* Motyka**

Subs.: cor – Alt.: 3 – Note: a species of this name with Motyka as author is not present in the literature. *U. rigidula* (Stirt.) D.D. Awasthi is a tropical species. – **Au**: K.

***Usnea subfaginea* Nádv.**

Subs.: cor – Alt.: 3 – Note: the combination at species level does not exist. It was published under the name U. faginea var. subfaginea Nádv, which is a synonym of *U. intermedia*. – **Au**: K.

***Usnea subgracilis* Vain.**

Syn.: *U. hesperina* Motyka, *U. schadenbergiana* *sensu* P. Clerc *non* Göpp. & Stein

Subs.: cor – Alt.: 3–4 – Note: the identification of the specimen from Slovenia (as *U. hesperina* Motyka) is highly doubtful. – **Sl**: ?SlA.

***Verrucaria brevieri* Servít**

L – Subs.: ? – Alt.: 1–2 – Note: on the whole, a very poorly known species. – **It**: Piem, Lig.

***Verrucaria devensis* (G. Salisb.) Orange**

Syn.: *Leucocarpopsis devensis* G. Salisb.

Subs.: sil-aqu – Alt.: 3 – Note: this species, whose type material is a specimen with exceptionally unpigmented perithecia, is known with certainty from the British Isles and Germany (outside the Alps); the record from Austria most probably refers to an albino-form of an unspecified *Verrucaria*-species (Hafellner and Türk 2001). – **Au**: ?St.

***Verrucaria nigricolor* Arnold**

Subs.: cal – Alt.: 2–3 – Note: a species described from Labrador, whose presence in Switzerland is very dubious. – **Sw**: ?BE.

**Xanthoria calcicola Oxner var. ectaniza (Nyl.) Cl. Roux**

L – Subs.: cal – Alt.: 5 – Note: the only record from the Alps is very dubious (see Hafellner and Türk 2016). – **Au**: ?T.
